# Baicalin Ameliorates Depression-like Behaviors via Inhibiting Neuroinflammation and Apoptosis in Mice

**DOI:** 10.3390/ijms251910259

**Published:** 2024-09-24

**Authors:** Yuhang Yi, Guiyu Liu, Ye Li, Changmin Wang, Bin Zhang, Haiyan Lou, Shuyan Yu

**Affiliations:** 1 Department of Physiology, School of Basic Medical Sciences, Cheeloo College of Medicine, Shandong University, Jinan 250012, China; 201917413015@mail.sdu.edu.cn (Y.Y.); 201900412050@mail.sdu.edu.cn (G.L.); 202390000063@sdu.edu.cn (Y.L.); 202421115@mail.sdu.edu.cn (C.W.); 2 Department of Pharmacology, School of Basic Medical Sciences, Cheeloo College of Medicine, Shandong University, Jinan 250012, China; binzhang@sdu.edu.cn (B.Z.); louhaiyan@sdu.edu.cn (H.L.); 3 Shandong Provincial Key Laboratory of Mental Disorders, School of Basic Medical Sciences, Jinan 250012, China

**Keywords:** depression, baicalin, neuroinflammation, apoptosis, oxidative stress

## Abstract

Depression is a common neuropsychiatric disease which brings an increasing burden to all countries globally. Baicalin, a flavonoid extracted from the dried roots of Scutellaria, has been reported to exert anti-inflammatory, antioxidant, and neuroprotective effects in the treatment of depression. However, the potential biological mechanisms underlying its antidepressant effect are still unclear. In the present study, we conducted extensive research on the potential mechanisms of baicalin’s antidepressant effect using the methods of network pharmacology, including overlapped terms-based analysis, protein–protein interaction (PPI) network topology analysis, and enrichment analysis. Moreover, these results were further verified through molecular docking, weighted gene co-expression network analysis (WGCNA), differential gene expression analysis, and subsequent animal experiments. We identified forty-one genes as the targets of baicalin in the treatment of depression, among which *AKT1*, *IL6*, *TP53*, *IL1B*, and *CASP3* have higher centrality in the more core position. Meanwhile, the roles of peripheral genes derived from direct potential targets were also observed. Our study suggested that biological processes, such as inflammatory reaction, apoptosis, and oxidative stress, may be involved in the therapeutic process of baicalin on depression. These mechanisms were validated at the level of structure, gene, protein, and signaling pathway in the present study. Taken together, these findings propose a new perspective on the potential mechanisms underlying baicalin’s antidepressant effect, and also provide a new basis and clarified perspective for its clinical application.

## 1. Introduction

Depression is a chronic recurrent neuropsychiatric disease which seriously impairs the ability of daily working and learning, and reduces the survival quality of patients [1]. In 2019, the global prevalence of depression was 3.6% [2], while this number has become higher in 2020 affected by COVID-19, according to a meta-analysis of community-based studies [3]. Therefore, depression brings enormous pressure to society and is predicted to become the main burden among global diseases by 2030 [4].

Previous studies have shown that neuroinflammation plays a considerable role in the promotion of depression [5], associated with the homeostasis disequilibrium of glutamatergic neurotransmission [6,7], and the dysfunction of the hypothalamus–pituitary–adrenal (HPA) axis [8,9]. Moreover, it has been suggested that patients with depression experience a performance of elevated levels of oxidative stress [10,11]. The changes in synaptic plasticity resulting from the inhibition of neurogenesis [12] and the increased level of apoptosis are also considered to be significant mechanisms of depression [13,14,15]. Nevertheless, the specific mechanisms have not been fully clarified.

In spite of the fact that there have already been various types of antidepressants, both the monoamine oxidase inhibitors (MAOIs) that are no longer widely used, and the selective serotonin reuptake inhibitors (SSRIs) and serotonin-norepinephrine reuptake inhibitors (SNRIs), these have had disadvantages of obvious adverse reactions and poor prognosis [16]. Therefore, the development of new drugs remains a crucial priority.

A large number of studies have shown that many Traditional Chinese Medicines (TCMs) exhibit antidepressant effects, which are characterized by only small toxic and side effects [17,18]. Baicalin, extracted from the dried roots of Scutellaria, a kind of traditional Chinese medicine that was first listed in the ancient Chinese book Shennong Grass Classic, is a flavonoid compound which has been proven to have anti-inflammatory, antioxidant, immunomodulatory and neuroprotective effects [19,20,21]. The excellent antidepressant effect of baicalin has been reported in animal models of depression [22,23,24]. However, because of the multi-target characteristics common to most traditional Chinese medicinal ingredients, it is an extreme challenge to elucidate the antidepressant mechanisms of baicalin merely via conventional pharmacological experimental approaches. Network pharmacology, which defines the mechanisms of disease treatment as the optimal molecular network targeted by monomers or synergistic drugs, adapts to these multi-target characteristics, offering a good solution for studying the integrated mechanisms of traditional Chinese medicinal ingredients [25,26].

The present work aims to explore the mechanisms underlying the antidepressant effects of baicalin by using a variety of bioinformatic methods and animal experiments. Firstly, the protein–protein interaction (PPI) networks of baicalin and depression were constructed by using the public database. The key potential targets of baicalin’s antidepressant effect were obtained by network topology analysis, which were then mapped to pathways by pathway enrichment analysis. In order to increase the reliability of the results, molecular docking and gene expression difference analysis were used to verify the potential targets of baicalin, while WGCNA was used to verify the pathway. Animal experiments were used to validate the behavioral effects of baicalin treatment in mice, as well as its antidepressant effect at RNA and protein levels.

## 2. Results

### 2.1. Structural Information and Druggability of Baicalin

**Figure 1:**
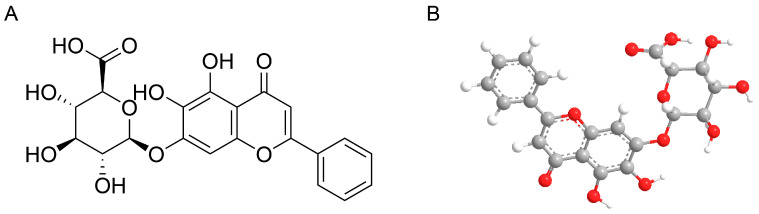
Baicalin structure. (**A**) Displayed formula. (**B**) Ball-and-stick models. Grey balls represent carbon atoms, red balls represent oxygen atoms, and white balls represent hydrogen atoms.

The structure and drug forming parameters of baicalin are as follows (Figure 1A,B; Table 1). The molecular weight and octanol/water partition coefficient (log P) of baicalin met the criteria of Lipinski’s rule of five, while the hydrogen bond donor count and hydrogen bond acceptor count failed. Baicalin satisfied the parameter requirements of Veber’s rule for compounds with oral activity, and the screening indicators for potential drugs in the TCMSP database. Its high oral bioavailability (OB) and drug-likeness (DL) suggested that baicalin possesses favorable pharmacokinetic properties.

### 2.2. Network Construction Revealed the Potential Targets of the Antidepressant Effect of Baicalin and Their Interactions

#### 2.2.1. Target Network for Baicalin

**Figure 2:**
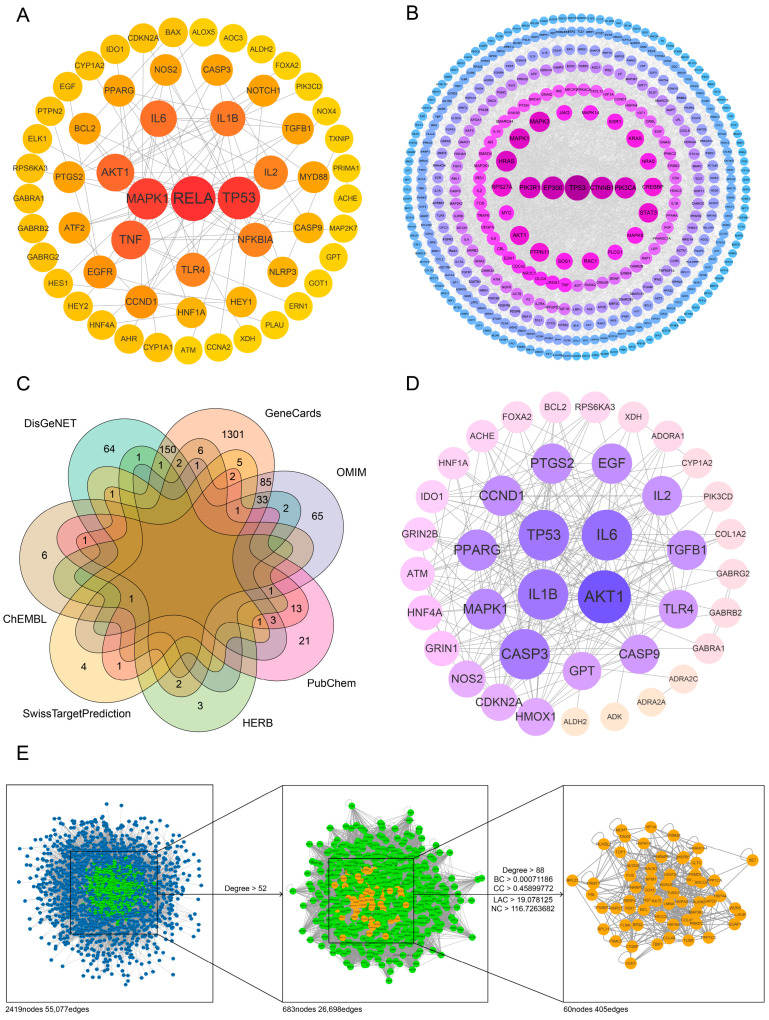
Network construction and topology analysis of antidepressant effect of baicalin. (**A**) PPI network for targets of baicalin. (**B**) PPI network for targets of depression. (**C**) Venn diagram for the common targets of depression and baicalin. (**D**) PPI network for targets of baicalin against depression. (**E**) Topology analysis of network structure-obtained core targets.

Baicalin target genes were retrieved from four databases specializing in Chinese medicine or chemical compounds—HERB (8 genes), PubChem (44 genes), ChEMBL (16 genes), and SwissTargetPrediction (15 genes)—using the keyword ‘baicalin’ (Supplementary Table S1). After de-duplication, these targets were entered into the STRING database using their Ensembl IDs. A network consisting of 58 nodes and 102 edges was generated by selecting ‘*Homo sapiens*’ as the species, setting the minimum required interaction score to 0.9, and excluding disconnected nodes. This network represents the target regulation relationships of baicalin, with each node corresponding to a baicalin target and each edge representing their interactions. The obtained network data were then imported into Cytoscape 3.7.2 for analysis, where *RELA*, *TP53*, and *MAPK1* were identified as having high topological centrality, with degree values of 16, 15, and 14, respectively (Supplementary Table S2). These findings suggest that these genes may play a pivotal role in baicalin’s pharmacological effects. The visualization of the baicalin target network is shown in Figure 2A, and the topological parameters of the top 20 targets by degree are listed in Table 2.

#### 2.2.2. Target Network for Depression

1609, 188 and 259 depression related target genes were gathered from GeneCards, OMIM and DisGeNET databases, respectively (Supplementary Table S3). Similar to the method above, after removing the duplication of these targets and inputting them into the STRING database, an interaction network of depression related proteins with 1311 nodes was obtained (Supplementary Table S4). The topological indexes of depression related target genes was calculated in Cytoscape, and the network of targets with degree > 10 was visualized, as shown in Figure 2B. The topological parameter information for targets with degree ranking in the top 20 is shown in Table 3 below.

#### 2.2.3. Target Network for Antidepressant Effect of Baicalin

A total of 41 overlapping genes were obtained by taking the intersection of baicalin target genes and depression related genes through the Venn package in R (Figure 2C, Supplementary Table S5). These 41 genes were considered to be the potential site of baicalin’s antidepressant effect. The analysis results for the STRING database showed that 40 targets obtained above are correlated at the protein–protein interaction level, forming a network with 233 edges. The topological analysis using Cytoscape 3.7.2 showed that the median degree of each node in the network is 10.5, while the degree values of *AKT1*, *IL6*, *TP53*, *IL1B* and *CASP3* are 28, 24, 23, 23 and 22, respectively, which are twice as high as the median, suggesting that these genes may play a core role in the antidepressant effect of baicalin (Figure 2D, Table 4, Supplementary Table S6).

### 2.3. The Peripheral Genes and Secondary Mechanisms of Baicalin’s Antidepressant Effect Were Explored by Topology Analysis of Network Structure

A total of 41 potential genes which represent the antidepressant targets of baicalin were input as bait nodes into the BisoGenet plugin in Cytoscape 3.7.2 to reconstruct the baicalin antidepressant target network, resulting in a network composed of 2419 nodes and 55,077 edges. According to the experience of previous research, the nodes were preliminarily screened with a threshold of more than twice the median degree value (52) [27], resulting in a protein–protein interaction sub-network composed of 683 nodes and 26,698 edges. Based on this network, the topology parameters of the network were subsequently calculated by using the CytoNCA plugin, and the median values of Degree, BC, CC, NC and LAC were 88, 0.00071186, 0.45899772, 19.078125 and 116.7263682, respectively. Taking these as the threshold, 60 candidate proteins were further screened, and the final protein interaction network was constructed (Figure 2E, Supplementary Table S7). These 60 proteins share the common characteristics of having been obtained by extending 41 potential targets with direct relation outward, probably related to the antidepressant effect of baicalin, and at the relative core site of protein–protein interaction.

### 2.4. Enrichment Analysis Clarified the Physiological Mechanism of Baicalin’s Antidepressant Effect

#### 2.4.1. Enrichment and Analysis of Baicalin’s Antidepressant Targets

To clarify the multiple mechanisms of baicalin on depression at a systematic level, the above 41 potential target genes of baicalin against depression were used for GO and KEGG enrichment analysis through the clusterProfiler package installed on R 4.1.2 software. GO enrichment analysis examined the function of a group of genes at the systematic level from three dimensions, i.e., biological process (BP), cellular component (CC) and molecular function (MF), while KEGG enrichment analysis took the pathways as the benchmark.

**Figure 3:**
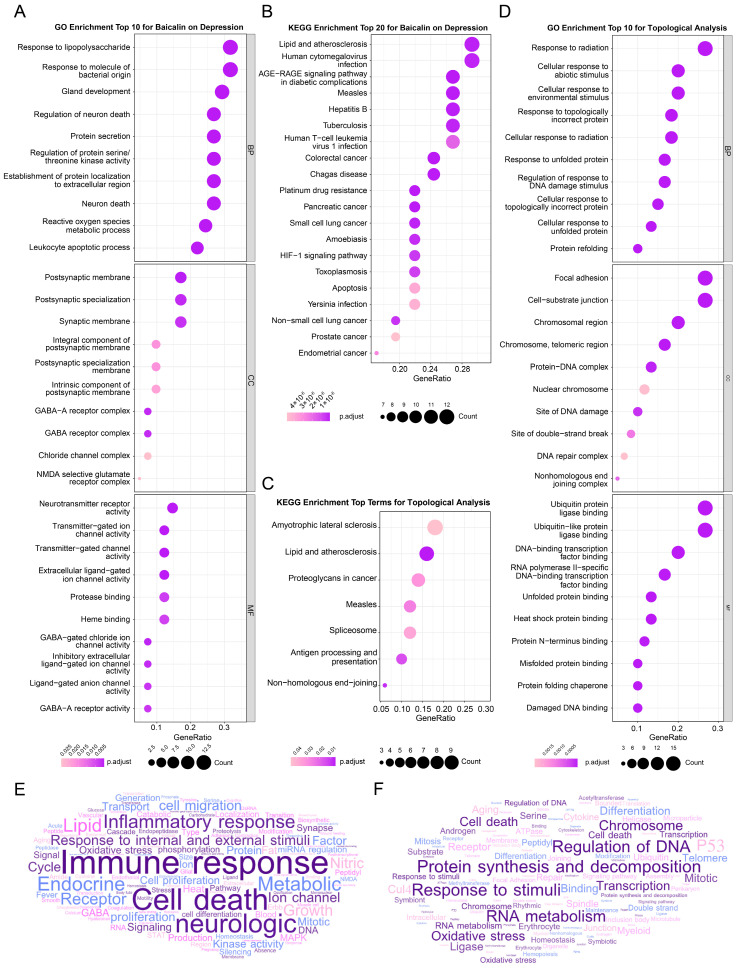
Enrichment analysis results. (**A**) Bubble plot for GO analysis of top 10 terms of Baicalin on depression. (**B**) Bubble plot for KEGG analysis of top 20 terms of Baicalin on depression. (**C**) Bubble plot for KEGG analysis of top 20 terms of topological analysis. (**D**) Bubble plot for GO analysis of top 10 terms of topological analysis. (**E**) Word cloud of baicalin antidepressant targets. (**F**) Word cloud of targets obtained by topological analysis.

GO enrichment analysis revealed significant (Bonferroni-corrected *p* < 0.05) enrichment in 316 BP terms, 13 CC terms, and 27 MF terms (Supplementary Table S8). The word-frequency analysis (Figure 3E), moreover, highlighted terms such as immune response, response to internal and external stimuli, cell death, inflammatory response, neurological processes, ion channels, oxidative stress, and synapses. After ranking the terms by their p.adjust significance and the number of enriched genes, the top 10 terms in BP, CC, and MF were visualized in Figure 3A. Notably, neuron death and reactive oxygen metabolic processes in BP, synaptic membrane, GABA receptor complex, and NMDA-selective glutamate receptor complex in CC, and neurotransmitter receptor activity and ion channel activity in MF were among the most prominent (Figure 3A).

KEGG enrichment analysis identified 81 KEGG pathways, in which PI3K-Akt signaling pathway, AGE-RAGE signaling pathway, pathways of neurodegeneration, HIF-1 signaling pathway, apoptosis, cell aging, MAPK signaling pathway, C-type lectin receptor signaling pathway, TNF signaling pathway, FOXO signaling pathway, P53 signaling pathway, Toll-like receptor signaling pathway, JAK-STAT signaling pathway, IL-17 signaling pathway, and NF-kappa B signaling pathway were particular notable. The gene number, gene proportion and enrichment significance of the first 20 pathways are shown in Figure 3B.

The above results suggest that the antidepressant effect of baicalin may be related to the regulation of immune inflammatory response, cell death and oxidative stress of the nervous system, and may involve a wide range of action pathways.

#### 2.4.2. Enrichment Analysis of Peripheral Genes

Using the same method as above, the GO/KEGG enrichment analysis was executed for 60 genes obtained from network topology analysis. The high frequency of entries related to the regulation of DNA, RNA and protein indicates that baicalin may further play a certain regulatory effect at all levels of transcription and protein translation, so as to achieve the role of treating depression. In addition, cellular responses to internal and external stimuli, oxidative stress and cell death were also significantly enriched (Figure 3C,D,F). These findings support the idea that baicalin may widely affect the various phases of protein expression through the processes of inflammation, cell death and oxidative stress of the nervous system to achieve its antidepressant effect.

### 2.5. The Results of Molecular Docking Suggest the Possibility of the Interaction between Baicalin and the Target Proteins

**Figure 4:**
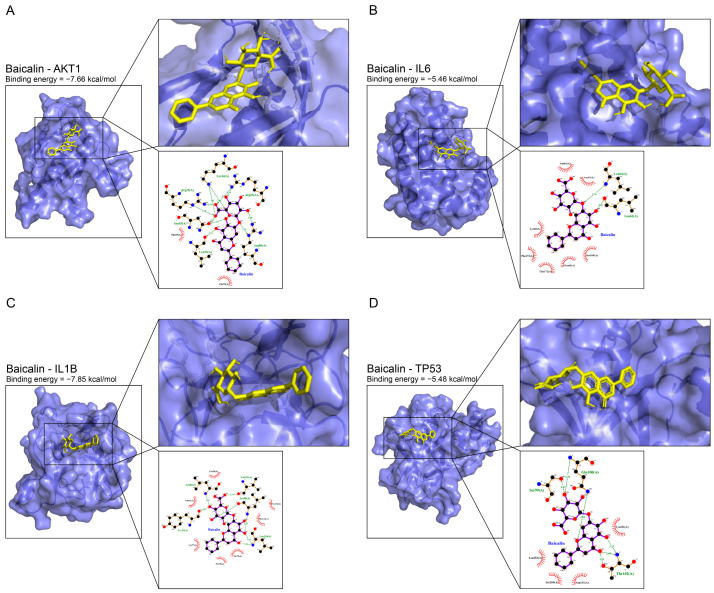
Molecular docking results. (**A**) Prediction of molecule interaction mode between baicalin and AKT1. (**B**) Prediction of molecule interaction mode between baicalin and IL6. (**C**) Prediction of molecule interaction mode between baicalin and IL1B. (**D**) Prediction of molecule interaction mode between baicalin and TP53.

The top-ranked potential targets may play a more central function among the 41 targets in the process involved in the antidepressant effect of baicalin, therefore baicalin was docked with the top 4 proteins, AKT1, IL6, IL1B and TP53, to explore the possibility of interaction between baicalin and key target proteins. The PDBID corresponding to the above 4 structures, 1UNQ, 1ALU, 5R8E and 4KVP, were acquired in the PDB database, respectively, while the structure of baicalin has been discussed in the first part of the Results section. Molecular docking results show the best conformation of baicalin and AKT1 in Figure 4A, where the binding energy is −7.66 kcal/mol. Baicalin formed 11 hydrogen bonds with Lys14, Arg23, Arg25, Leu52, Asn53 and Arg86 of the protein, and hydrophobic interactions with 2 amino acid residues, Phe55 and Gln79. The best for baicalin and IL6 is shown in Figure 4B, with binding energy of −5.46 kcal/mol. Baicalin formed 2 hydrogen bonds with Leu62 and Leu64 and hydrophobic interactions with 7 amino acid residues, Asn61, Asn63, Pro65, Lys66, Ser169, Glu172 and Phe173. The best for baicalin and IL1B is shown in Figure 4C, with a binding energy of −7.85 kcal/mol. Baicalin formed 7 hydrogen bonds with Tyr24, Leu80, Leu82, Val132 and Leu134, and hydrophobic interactions with 7 amino acid residues, Leu26, Lys77, Pro78, Thr79, Gln81, Pro131 and Phe133. The best one for baicalin and TP53 is shown in Figure 4D, with binding energy of −5.48 kcal/mol. Baicalin formed 6 hydrogen bonds with Ser99, Gln100 and Thr102, and hydrophobic interactions with 4 amino acid residues, Lys101, Asn131, Leu252 and Ser269.

The binding energies between baicalin and proteins AKT1, IL6, IL1B and TP53, respectively, were all less than −5 kcal/mol, and all could form hydrogen bonds and hydrophobic interactions, indicating that baicalin has a great affinity and high possibility of interaction with these four proteins, which may be at the core position.

### 2.6. WGCNA Analysis and Expression Difference Analysis Verified the Effect of Baicalin on Depression

#### 2.6.1. Multiple Pathways in the Hippocampus of Patients with Depression Have Changed

**Figure 5:**
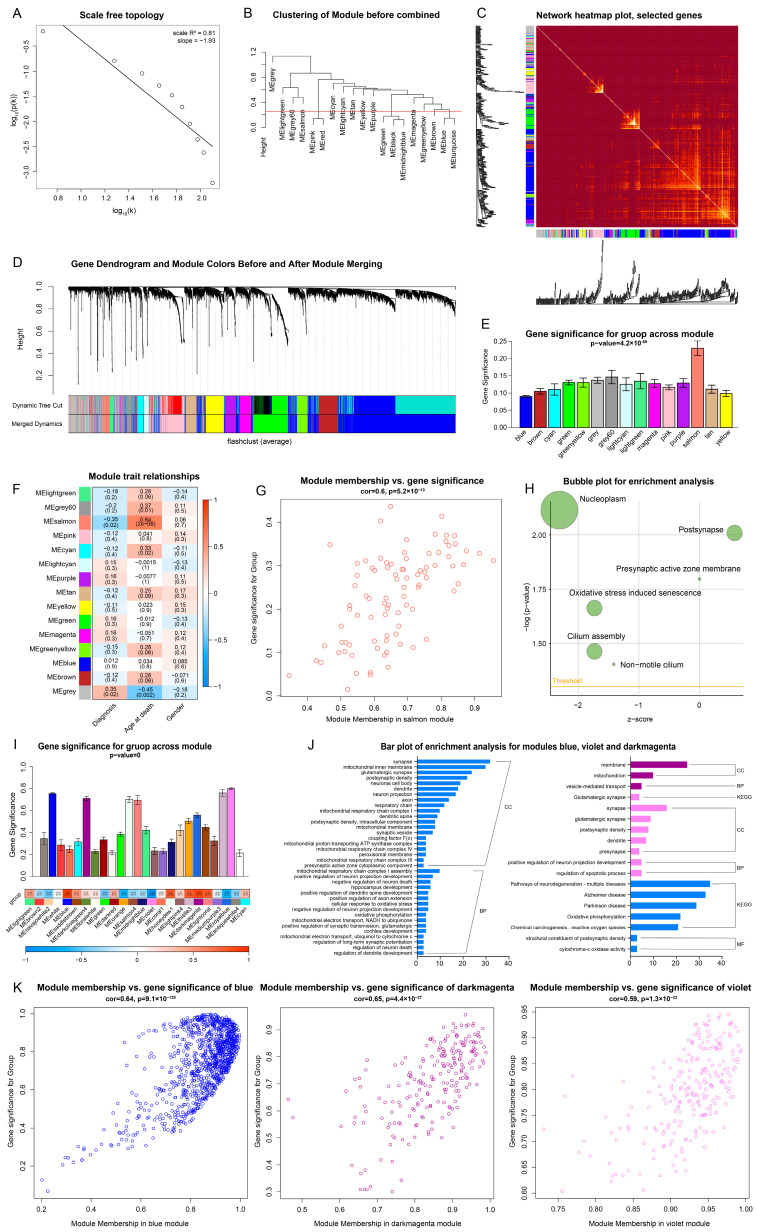
The process and results of WGCNA. (**A**) The inverse proportional fitting curve suggests that the constructed network of GSE42546 has scale-free characteristics. (**B**) Clustering and combination of gene modules. (**C**) Visualization of weighted network by TOM heat map. (**D**) Gene dendrogram and module colors before and after module merging. (**E**) Gene significance of group across modules. (**F**) Module–trait correlations. (**G**) The scatter plot of GS and MM of all genes in the salmon module shows a positive proportional correlation. (**H**) Bubble plot of the enrichment analysis for the genes in the screened salmon module. (**I**) Gene significance of group across the module of GSE160587. (**J**) Bar plot of enrichment analysis for the genes in modules blue, violet and dark magenta. (**K**) The scatter plot for GS and MM of all genes in modules blue, dark magenta and violet shows positive proportional correlation.

For GSE42546, a data set of the full transcriptome sequencing expression profile of the post-mortem hippocampal tissues from patients with depression, a scale-free network was first constructed using WGCNA. After evaluating the scale independence and gene connectivity of the network under each soft threshold, a soft threshold β = 10 was finally selected to ensure adequate connectivity and maintain a scale-free network, in which situation R2 is greater than 0.8 for the first time (Supplementary Figure S1A–C). After verification, it was determined that the network constructed after the operation under the condition of β equals 10 has scale-free characteristics (Figure 5A, Supplementary Figure S1D). Then, the preliminary gene modules were constructed. The average method in the R package flashClust was used for clustering, and 19 gene modules were identified by the Dynamic Tree Cut method (Supplementary Figure S1E). The modules with similarity greater than 0.75 were combined, and 15 gene modules were retained (Figure 5B–D, Supplementary Figure S1F).

The correlation between modules and the phenotypic information of the sample was tested subsequently. A significant correlation (r = −0.35, *p* = 0.02) between the salmon module and depression was found. Consequently, this module was selected for further research (Figure 5E,F, Supplementary Table S9).

The scatter plot of GS and MM of all genes in the salmon module is shown in Figure 5G. Screened with a threshold of *p* < 0.05, 32 genes were selected for GO/KEGG enrichment analysis (Figure 5H). Nucleoplasm, synaptic cell membrane, oxidative stress induced senescence and cilium were observed to be significantly enriched. These findings suggest that the occurrence and development of depression may be related to synaptic changes, oxidative stress and other processes.

#### 2.6.2. Multiple Pathways in the Hippocampus of Mice with the Treatment of Baicalin Have Changed

For data set GSE160587, which reflects the effect of baicalin treatment, WGCNA was performed with the same method as above but with soft threshold β = 7, and finally 24 combined modules were obtained (Supplementary Figure S1H–P). Blue, violet and dark magenta modules were selected for further study, since there was obvious correlation between them and baicalin treatment (*p* < 0.05) (Figure 5I,K). A total of 391, 108 and 58 significant genes in the three modules respectively were reserved after screening, which were then used for enrichment analysis. The results showed that some enriched terms overlapped with those identified in the enrichment analysis of baicalin’s potential targets for depression. In addition, the development of neurons was also significantly enriched. The visualization of the above results is shown in Figure 5J.

The WGCNA analysis of baicalin treatment dataset GSE160587 verified that the antidepressant effect of baicalin may be related to the regulation of synaptic development, in which oxidative stress, cell death and inflammatory reaction may play an important role.

#### 2.6.3. Changes in Gene Expression Level Occur in the Hippocampus of Patients with Depression

**Figure 6:**
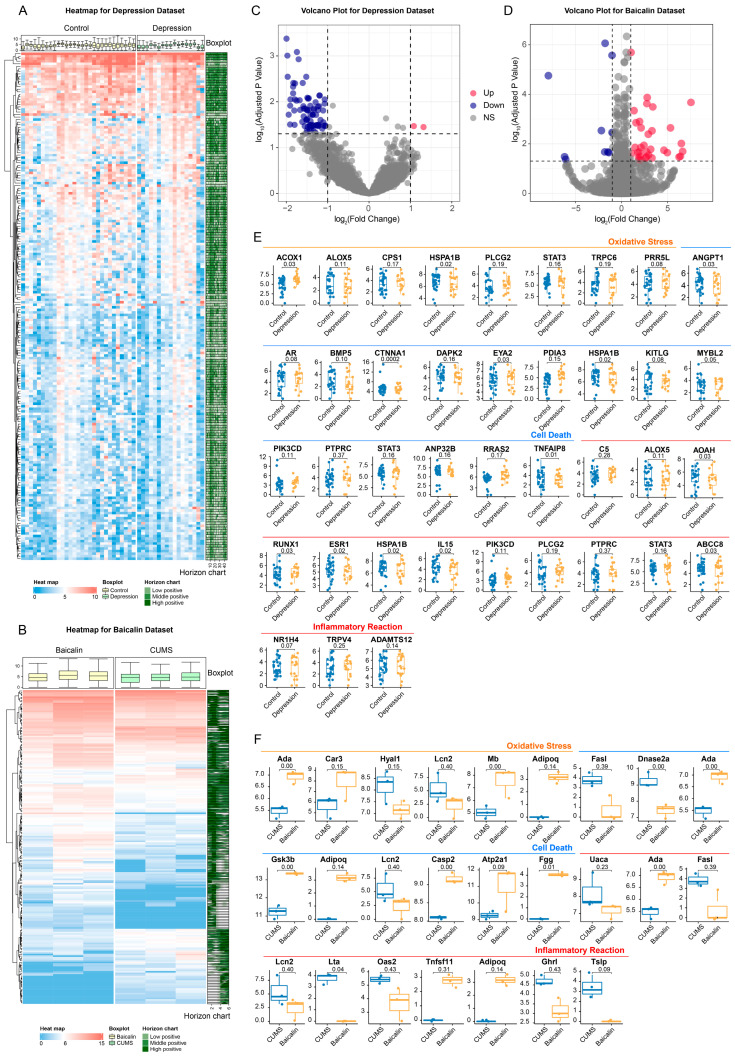
The results of differential gene expression analysis. (**A**) Heatmap for depression dataset GSE42546. (**B**) Heatmap for baicalin dataset GSE160587. (**C**) Volcano plot for depression dataset GSE42546. (**D**) Volcano plot for baicalin dataset GSE160587. (**E**) Box plots of differential analysis for genes in the terms of oxidative stress, cell death, and inflammatory response for depression dataset GSE42546. (**F**) Box plots of differential analysis for genes in terms of oxidative stress, cell death, and inflammatory response for baicalin dataset GSE160587.

The above three aspects continued to be verified at the genetic level. Differential analysis was executed for the dataset GSE42546, and the genes with log_2_(Fold Change) in the top 200 are shown in the heat map (Figure 6A, Supplementary Table S10). Three genes were up-regulated and 95 genes were down-regulated in the hippocampus of patients with depression (Figure 6C). In order to investigate and verify whether oxidative stress, cell death, and inflammatory response are involved in depression, genes related to these terms were screened according to the corresponding relationship between terms and genes in GO/KEGG, and box plots were drawn for visualization. It was found that *HSPA1B* is statistically different in terms for oxidative stress; among the terms for cell death, *ANGPT1*, *CTNNA1*, *EYA2*, *HSPA1B* and *TNFAIP8* genes show statistical differences; among the terms for inflammatory reaction, *AOAH*, *RUNX1*, *ESR1*, *HSPA1B*, *IL15* and *ABCC8* show statistical differences (Figure 6E).

#### 2.6.4. Baicalin Can Play an Antidepressant Role Possibly by Regulating the Gene Expression Level

Similarly, differential analysis of the dataset GSE160587 showed that 41 genes were up-regulated and 15 genes were down-regulated in the hippocampus of baicalin treated mice (Figure 6B,D). In order to verify whether baicalin as antidepressant is related to oxidative stress, cell death and inflammatory response, it was obtained that, among the terms for oxidative stress, *Ada* and *Mb* showed significant differences; among the terms for cell death, *Dnase2a*, *Ada*, *Gsk3b*, *Casp2* and *Fgg* showed significant differences, and *Ada* and Lta showed significant differences in the terms for inflammatory response, suggesting that these the terms may have a certain effect in the process of baicalin’s antidepressant activity (Figure 6F).

### 2.7. Animal Experiments Confirmed the Antidepressant Effect of Baicalin

#### 2.7.1. Baicalin Improved the Behavioral Disorder of Depressed Mice Induced by CUMS

**Figure 7:**
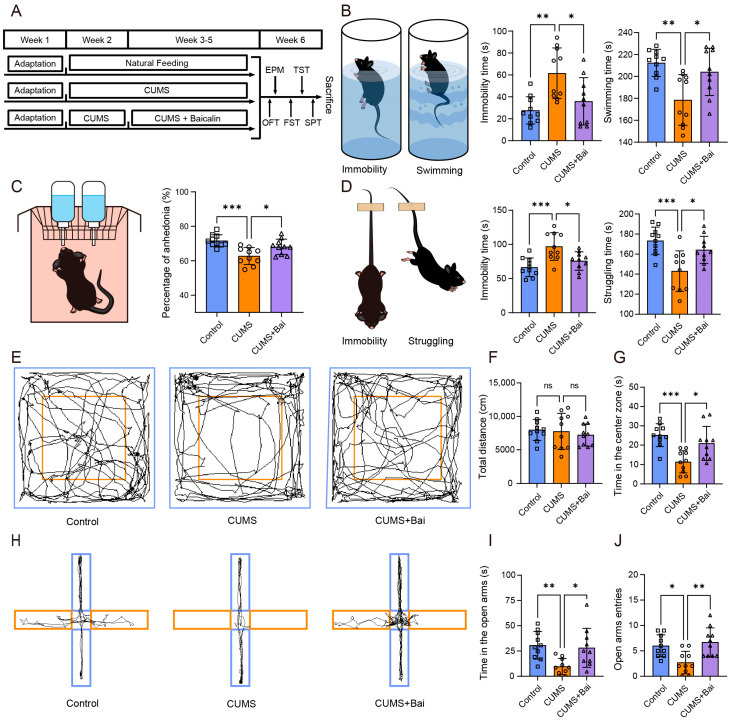
Animal model process and behavioral testing results. (**A**) The modeling process of CUMS-induced depression mice and baicalin administration. (**B**) Pattern diagram and results of FST in Control, CUMS, and CUMS + Bai mice. (**C**) Pattern diagram and results of SPT in Control, CUMS, and CUMS + Bai mice. (**D**) Pattern diagram and results of TST in Control, CUMS, and CUMS + Bai mice. (**E**) Motion traces of mice in OFT. The blue frame represents the observed field, and the orange frame represents the center zone. (**F**) Total distance in the OFT in Control, CUMS, and CUMS + Bai mice. (**G**) Time spent exploring the center zone in the OFT in Control, CUMS, and CUMS + Bai mice. (**H**) Motion traces of mice in EPM. The blue frame represents the closed arm, and the orange frame represents the open arm. (**I**) Time spent in open arms in the EPM in Control, CUMS, and CUMS + Bai mice. (**J**) The number of entering open arms in the EPM in Control, CUMS, and CUMS + Bai mice. Data are presented as the means ± SEMs. * *p* < 0.05, ** *p* < 0.01, *** *p* < 0.001, ns *p* > 0.05.

To investigate the effect of baicalin on depressed mice, the depressed mice model was constructed by using the CUMS process and baicalin was intragastrically administered (Figure 7A). The results of the sucrose preference test showed that, compared with the control mice, the percentage of sucrose consumption for the CUMS group mice was significantly reduced, and this parameter was improved in the CUMS + Bai group. This result indicated that the treatment with baicalin improved symptoms of anhedonia (Figure 7C). The results for forced swimming test and tail suspension test indicated that the CUMS group mice showed an increase in immobility time and a decrease in swimming or struggling time, which was reversed by the use of baicalin, indicating that baicalin improved behavioral despair (Figure 7B,D). In the open field test, it was indicated that exposure to CUMS and treatment with baicalin did not alter the total distance of locomotion in the open field, but altered the time spent exploring the central area (Figure 7E–G). In addition, the results of the elevated plus maze test showed that administration of baicalin reversed the reduction in time and frequency of entering the open arm caused by CUMS exposure (Figure 7H–J). Both OFT and EPM showed an increase in anxiety levels in CUMS mice, and the use of baicalin reversed this to a certain extent. All of these results for behavioral tests in this section indicated that the behavioral disorders in depressed mice caused by chronic stress can be improved by intragastrical administration of baicalin.

#### 2.7.2. Baicalin Exerted Antidepressant Effects by Inhibiting Neuroinflammation

Next, in order to verify the potential mechanism of the antidepressant effect of baicalin obtained from the network pharmacology analysis above, we investigated the effects of baicalin on neuroinflammation and apoptosis.

**Figure 8:**
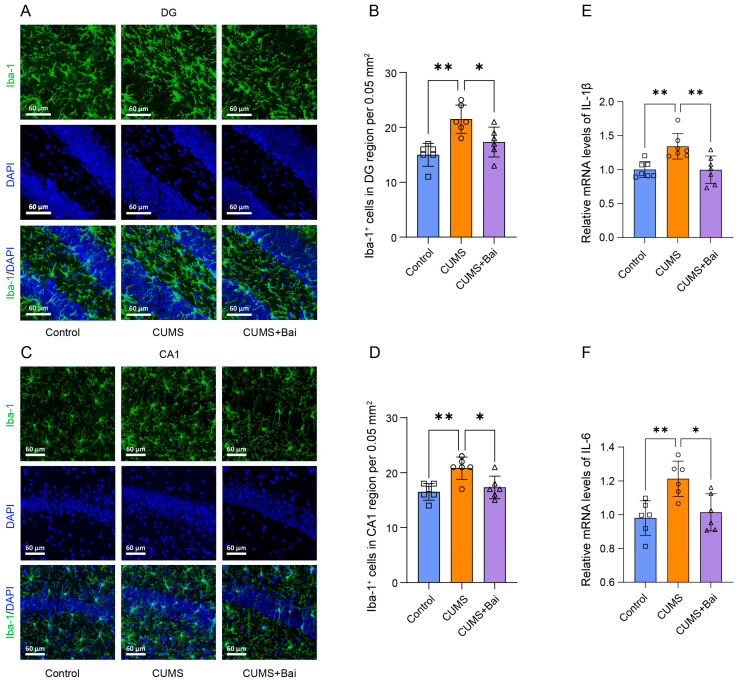
Baicalin exerted antidepressant effects by reducing neuroinflammation. (**A**) Representative diagram of the number of Iba-1-positive microglia in the DG region of the mouse hippocampus by immunofluorescence staining. (**B**) Column chart of the number of Iba-1-positive microglia in the DG region of the mouse hippocampus by immunofluorescence staining. (**C**) Representative diagram of the number of Iba-1-positive microglia in the CA1 region of the mouse hippocampus by immunofluorescence staining. (**D**) Column chart of the number of Iba-1-positive microglia in the CA1 region of the mouse hippocampus by immunofluorescence staining. (**E**) PCR result of IL-1β in Control, CUMS, and CUMS + Bai mice. (**F**) PCR result of IL-6 in Control, CUMS, and CUMS + Bai mice. Data are presented as means ± SEMs. * *p* < 0.05, ** *p* < 0.01.

For neuroinflammation, immunofluorescence was used to investigate the changes in the number and morphology of microglia in the DG and CA1 regions of the mouse hippocampus, and the results showed that the increase in the number of microglia caused by CUMS stimulation was reversed by the administration of baicalin, and the morphology of some microglia cells also returned from an active state to a resting state (Figure 8A–D, Supplementary Figure S2A,B). PCR experiment results indicated that the increase in mRNA of IL-1β and IL-6 in the CUMS group was reversed by the application of baicalin (Figure 8E,F).

#### 2.7.3. Baicalin Exerted Antidepressant Effects by Inhibiting Apoptosis

**Figure 9:**
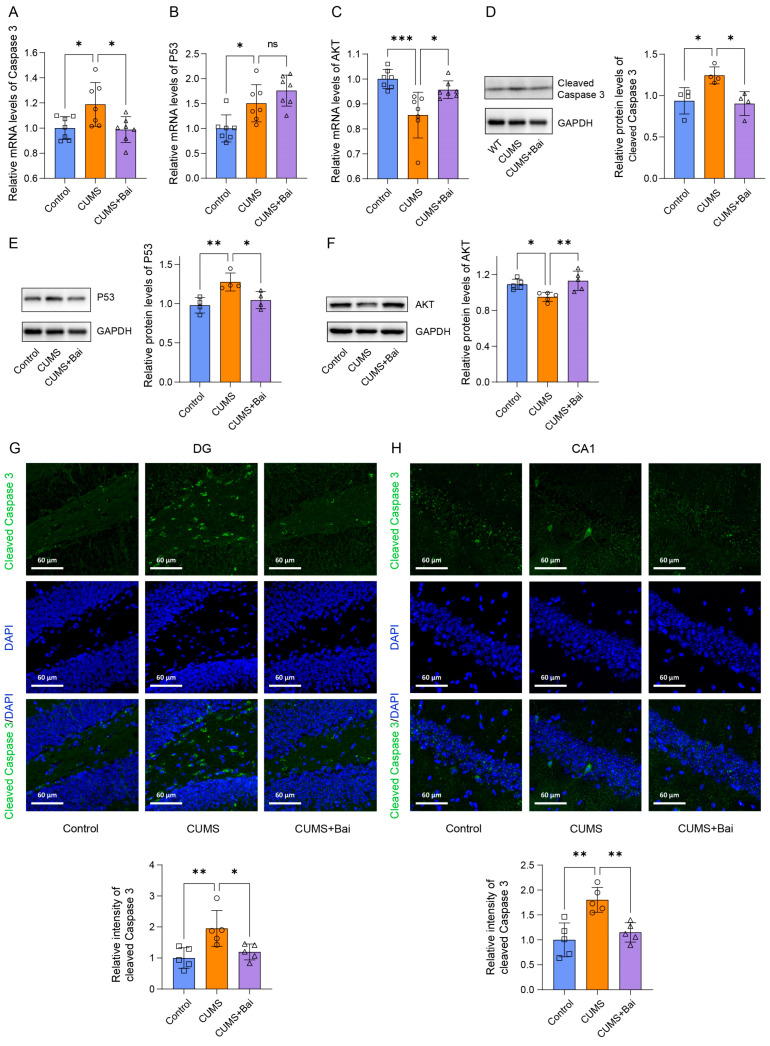
Baicalin exerted antidepressant effects by reducing apoptosis. (**A**) The PCR result of Caspase 3 in Control, CUMS, and CUMS + Bai mice. (**B**) The PCR result of P53 in Control, CUMS, and CUMS + Bai mice. (**C**) The PCR result of AKT in Control, CUMS, and CUMS + Bai mice. (**D**) The protein expression of Cleaved Caspase 3 in Control, CUMS, and CUMS + Bai mice. (**E**) The protein expression of P53 in Control, CUMS, and CUMS + Bai mice. (**F**) The protein expression of AKT in Control, CUMS, and CUMS + Bai mice. (**G**) Representative diagram and column chart of the fluorescence intensity of cleaved Caspase 3-positive cells in the DG region of the mouse hippocampus by immunofluorescence staining. (**H**) Representative diagram and column chart of the fluorescence intensity of cleaved Caspase 3-positive cells in the CA1 region of the mouse hippocampus by immunofluorescence staining. Data are presented as the means ± SEMs. * *p* < 0.05, ** *p* < 0.01, *** *p* < 0.001, ns *p* > 0.05.

As one of the main forms of cell death, the role of apoptosis in the antidepressant effect of baicalin has also been evaluated. PCR showed that, compared with the control group, the apoptogenic factors Caspase-3 and P53 were significantly increased in the CUMS group at mRNA level, while CUMS + Bai group showed a significant decrease in Caspase-3 compared to the CUMS group, which did not show in P53 (Figure 9A,B). Compared with the Control group, the apoptotic inhibitory factor AKT was significantly reduced in the CUMS group, while the CUMS + Bai group showed a significant increase compared to the CUMS group (Figure 9C). The reversal effects for Cleaved Caspase-3, P53 and AKT expression by baicalin were demonstrated at the protein level through Western blot (Figure 9D–F). Through the investigation of Cleaved Caspase-3 immunofluorescence staining in the DG and CA1 regions of the hippocampus of mice, it was found that the immunofluorescence intensity of the CUMS group was significantly increased compared to the Control group, while the CUMS + Bai group was significantly decreased compared to the CUMS group (Figure 9G,H). The above results indicated that the administration of baicalin alleviates apoptosis in the hippocampus of depressed mice.

## 3. Discussion

Depression is a neuropsychiatric disease accompanied by neuronal damage, which has the characteristics of multiple etiology [28]. In recent years, more and more traditional Chinese medicine and its extracts have been studied and proven to have antidepressant effects, including baicalin, a flavonoid compound extracted from scutellaria. Previous studies have revealed that the regulation of pathways, such as Rac/LIMK/cofilin, PI3K/AKT/FoxO1, BDNF/ERK/CREB, etc., may play an important role in the antidepressant process of baicalin. However, as a traditional Chinese medicine compound with multi-target characteristics, studying just one signaling pathway is insufficient to fully elucidate the specific mechanism of its antidepressant effect.

In this study, based on evaluating the druggability and antidepressant targets of baicalin, we identified 41 potential antidepressant targets. Through the topological structure analysis of the interaction network, which is based on centrality, *AKT1*, *IL6*, *TP53*, *IL1B* and *CASP3* were identified as the central targets of baicalin’s antidepressant effect, and the molecular docking was executed as verification for the first four genes. The role of peripheral genes derived from direct potential targets in the antidepressant process of baicalin was also considered and evaluated. We performed GO and KEGG analysis on the potential targets obtained from two analyses, respectively, and concluded that the regulation of inflammatory response, cell death and oxidative stress may be involved in the treatment of depression with baicalin. Subsequently, WGCNA was used to verify whether these changes were reflected in the transcriptome sequencing data of depression and baicalin treatment specimen, and differential gene expression analysis was used to check whether there were significant differences in related genes.

Next, animal experiments validated the behavioral performance of depression mice with baicalin treatment. The sucrose preference test, forced swimming test, and tail suspension test evaluated typical symptoms of depression, such as anhedonia and behavioral despair in mice. In addition, some patients with depression often exhibit comorbid characteristics of anxiety in their behavior or mentality in clinical practice [29,30]. Although these are not typical symptoms of depression, they also have a significant impact on the quality of life of patients with depression. In this study, the open field test and elevated plus maze test were used to evaluate anxious behavior. Combining with the CUMS model and baicalin treatment, the behavioral experimental methods above confirmed that baicalin improves depression-like and anxiety-like behavior in mice. Subsequently, qPCR, western blot, and immunofluorescence methods were used to validate the antidepressant mechanisms of baicalin on inflammatory response and apoptosis—the main form of cell death—at both RNA and protein levels.

As for the central targets of baicalin’s antidepressant effect, AKT1, one of the three isoforms of protein kinase B, is a key component of the PI3K/AKT signaling pathway, which can phosphorylate and inactivate the components of the apoptotic machinery to inhibit apoptosis [31]. In the central nervous system, the inhibition of AKT1 may cause increased neuronal apoptosis and lead to depression. In this study, the reversal of the declined inhibitory function on apoptosis of AKT caused by CUMS stimulation by baicalin indicated that baicalin can reduce neuronal apoptosis through AKT. IL-6 encodes a kind of cytokine, which plays a role in the process of inflammatory response and B-cell maturation by binding to receptors IL6R and IL6ST [32,33], whose dysregulation can precipitate multiple events relevant to depression, such as serotonin depletion and neurodegenerations [34,35]. Cytokine encoded by IL-1β is also an important mediator of inflammatory response and participates in a variety of cell activities, including cell proliferation, differentiation and apoptosis. It is also vital in the occurrence and development of depression, which can be proven by the facts that the level of IL-1β in cerebrospinal fluid (CSF) or serum of patients with major depressive disorder (MMD) increased [36,37], and IL-1β treatment can cause depression-like behavior in rats [38]. TP53 is a transcription factor that regulates cell proliferation and apoptosis through transcriptional regulation of its downstream targets or direct interaction with them. The destruction of its regulatory mechanism may be related to the pathophysiology of MDD [39,40]. In addition, Caspase3 encoded by CASP3 is a member of the cysteine-aspartic acid protease (caspase) family and plays a central role in the executive stage of apoptosis.

The suppression of oxidative stress, apoptosis and inflammatory response in the depression treatment process of baicalin is also proved in the present work. Oxidative stress and inflammatory response play an important role in neuro-progression, which is defined as a potentially progressive stage-related process of neurogenesis that includes cell death and reduced neural plasticity [41], which is one of the pathogenesis for the occurrence and development of depression. Although reactive oxygen species easily oxidize RNA, DNA, proteins and lipids, resulting in the loss of their functions and then damage to neurons, they also have positive effects, such as promoting signal transduction and activating cell survival pathways. However, oxidative stress characterized by high levels of reactive oxygen species can stimulate the inflammatory cascade and is strictly regulated by inflammatory signals. Both of these stimulate the process of neuronal cell death by activating stress kinases, such as p38 and JNK, promoting the occurrence and development of depression [42,43,44]. According to the results of this present study, the potential targets of baicalin antidepressant effect are significantly enriched in this signaling, and the results of WGCNA, gene expression difference analysis and animal experiments also verify the results to a certain extent.

Bioinformatics methods were used in this study to research and explain the antidepressant effect of baicalin from the network level. Based on the principles of network theory and system biology, network pharmacology is well adapted to the multi-target characteristics of traditional Chinese medicine components and provides a good idea for this study of their disease treatment mechanisms. Ultimately, it is necessary to ensure the high reliability of the results of network pharmacology analysis, which acts as a link in the evidence chain of evidence-based medicine. Therefore, the coordinated use of various Bioinformatics approaches and basic animal experiments make these findings robust. As a significant pathway of structural molecular biology and computer aided drug design in new medicines, molecular docking can evaluate the affinity between drugs and targets, and provide a three-dimensional structural hypothesis for the interaction between them [45]. To identify genes differentially expressed across different sample groups, differential analysis of high-throughput sequencing data links changes at the biological and gene levels [46]. WGCNA, mainly evaluating at the multi-gene or pathway level, explores the correlation between different gene modules and certain conditions by constructing a scale-free network [47]. Animal experiments evaluate behavioral performance in animal models and examine specific mechanisms at the RNA and protein levels. Therefore, the network pharmacological analysis based on a public database may provide a new idea for revealing the antidepressant mechanism of baicalin. Molecular docking, difference analysis, WGCNA and animal experiments combined with network pharmacology increase the reliability of the results at the levels of molecule, gene, protein and pathway. Nevertheless, whether the changes obtained in this study can be repeatedly verified in cell models and their specific mechanisms still needs to be further investigated.

Therefore, through the application of bioinformatics methods and animal experiments, we conclude that the regulation of neuroinflammation, apoptosis and oxidative stress may play an important role in the antidepressant effect of baicalin. Baicalin may regulate these processes by acting on key genes, such as *AKT1*, *IL6*, *TP53*, *IL1B* and *CASP3*, which are connected with neural damage and then depression. Our research provides a basis for the follow-up study of the specific molecular mechanism of baicalin’s antidepressant effect, and also provides a new application basis and clarifying perspective for its clinical application.

## 4. Materials and Methods

### 4.1. Data Preparation

#### 4.1.1. Structural Information and Druggability Evaluation of Baicalin

The 2D and 3D chemical structure of baicalin was obtained from PubChem (http://pubchem.ncbi.nlm.nih.gov, accessed on 1 April 2022), which is a highly reliable public database providing information, such as that on the molecular structure and biological characteristics of chemical compounds. Subsequently, PubChem and the Traditional Chinese Medicine Systems Pharmacology (TCMSP) (https://www.tcmsp-e.com/#/database, accessed on 1 April 2022) were used to obtain the biochemical parameters of baicalin to evaluate the possibility of baicalin as a latent medicine. The parameters included hydrogen bond donor count, hydrogen bond acceptor count, molecular weight, octanol/water partition coefficient (log P) in Lipinski’s rule of five [48], rotatable bond count, polar surface area in Veber’s Rule [49], Oral bioavailability (OB) and Drug-likeness (DL). Among them, OB represents the percentage of unchanged drugs reaching systemic circulation via orally administered dose, which is usually the key indicator of whether molecules can be used as therapeutic drugs [50]; DL is used to estimate the “drug-like” degree of a prospective compound and is used as the selection standard of “drug-like” compounds in traditional Chinese herbal medicine [51].

#### 4.1.2. Targets Prediction for Baicalin

HERB (http://herb.ac.cn/, accessed on 18 April 2022), ChEMBL (https://www.ebi.ac.uk/chembl/, accessed on 18 April 2022), PubChem and SwissTargetPrediction (http://www.swisstargetprediction.ch/, accessed on 18 April 2022) databases were used to gather the targets of baicalin comprehensively. HERB is a high-throughput experiment- and reference-guided database of TCM with comprehensive information, which integrates multiple TCM databases and contains more than 56,000 herbs and ingredients created to date [52]. ChEMBL is a manually managed database of bioactive molecules with drug-like properties, where computational predicted targets of small molecules can be retrieved. PubChem and the above two databases were used to collect the biological targets of baicalin with the keyword “baicalin”. In addition, SwissTargetPrediction was used to predict the targets of baicalin as a supplement by inputting the structural information of baicalin in “SMILES” format obtained from the PubChem database. The screening threshold for potential targets was probability >0.1 [53].

#### 4.1.3. Identification of Targets Associated with Depression

Relevant targets of depression were collected from GeneCards (https://www.genecards.org/, accessed on 18 April 2022), DisGeNET (https://www.disgenet.org/, accessed on 18 April 2022) and OMIM (https://omim.org/, accessed on 18 April 2022) databases. Firstly, depression related genes were collected in GeneCards, a comprehensive database with the function of providing the relationship between diseases and genes, with the standard of Score > 2. Then, targets were retrieved in the DisGeNET database with “Mental Depression” as the keyword and “Score gda > 0.3” as the threshold. DisGeNET is a human disease database established by database integrating and text mining, which covers the full spectrum of human diseases, as well as normal and abnormal traits. The depression-related targets in the database of Online Mendelian Inheritance in Man (OMIM) were used as a supplement to those from the two websites above.

#### 4.1.4. Acquirement of Potential Targets for Baicalin’s Antidepressant Effect

Converted into Entrez ID, the collected targets were inputted into the Venn Diagram website (http://bioinformatics.psb.ugent.be/webtools/Venn/, accessed on 19 April 2022) and then intersections were taken, obtaining the genes co-existing in baicalin targets and depression targets as potential targets of baicalin’s antidepressant effect; meanwhile, they were inputted into the Venn package installed in R 4.1.2 software to draw the Venn diagram for visualization of the results [54].

#### 4.1.5. Selection and Preprocessing of High-Throughput Datasets

Datasets related to depression or baicalin were collected by searching the Gene Expression Omnibus (GEO) database (https://www.ncbi.nlm.nih.gov/geo/, accessed on 27 April 2022). GSE42546 and GSE160587 were selected based on the experimental purposes. GSE42546 is a whole transcriptome sequencing expression profile of postmortem hippocampus dentate gyrus granule cells from patients with mental illness, including patients with bipolar disorder or depression or schizophrenia, and control subjects. From this source, information on patients with depression and the control group was extracted and included in the present research to verify the changes in gene expression in the two groups. To maintain data consistency, GSE160587 with hippocampal tissue as the site of study was selected, which is also a whole transcriptome sequencing expression profile but, for the antidepressant effect of baicalin in chronic unpredictable mild stress (CUMS), C57BL/6N mice models were used, as a verification of the change in gene expression level caused by the usage of baicalin. These data, downloaded from the GEO official website, were read into the R 4.1.2 software for data cleaning for subsequent analysis.

### 4.2. Network Construction and Topological Analysis

#### 4.2.1. Target Networks for Baicalin and Depression

Using the STRING protein–protein interaction database (https://string-db.org/, accessed on 19 April 2022), the interaction relationship between baicalin targets and that between depression targets were analyzed. Specifically, the Ensembl ID list corresponding to the genes was inputted into the STRING, with the minimum required interaction score set to 0.9 which indicates the high confidence in protein interaction. After hiding the disconnected nodes in the network, the final interaction information was exported. The visualized protein–protein interaction (PPI) network was constructed in Cytoscape 3.7.2 [55], and the degree value of each node, which is defined as the number of links connected to the node, was calculated at the same time as the centrality evaluation standard of the genes represented by the nodes.

#### 4.2.2. Construction of Network for Target Genes of Baicalin on Depression

The method of building the network is similar to that mentioned above, with the difference of taking the list of potential targeted genes of baicalin’s antidepressant effect obtained by intersection. To display protein–protein interaction information more comprehensively, the minimum required interaction score was set as 0.4.

#### 4.2.3. Topology Analysis

Using potential target genes of baicalin’s antidepressant effect as the bait nodes, reconstruction of the network was performed in Cytoscape 3.7.2 using the BisoGenet plugin, where PPI information was extended to peripheral genes [56]. In order to extract the genes in the relative core position to explain the secondary action mechanism, the topological parameters of each node of the network were calculated with the assistance of the cytoCNA plugin. Among these, Degree, Betweenness Centrality (BC), Closeness Centrality (CC), Neighborhood Connectivity (NC) and Local Average Connectivity (LAC) were used to screen genes and construct sub-networks. These parameters indicate the importance of nodes in the network, being prevalent in the centrality measurement of the network [57].

### 4.3. Enrichment Analysis

The R package clusterProfiler was used for GO and KEGG enrichment analysis [58]. GO, gene ontology, provides biologically meaningful annotation of genes and their products in various organisms. The properties of genes and gene products can be consistently described in three key biological fields shared by all organisms: molecular function (MF), biological process (BP) and cellular component (CC), which are defined separately as the biochemical activity of a gene product, the contributed biological objective, and the place in the cell where a gene product is active [59,60]. In addition, the KEGG (Kyoto Encyclopedia of Genes and Genomes) database was also used for analysis to clarify the role of targets in signal transduction.

### 4.4. Molecular Docking

Molecular docking, binding conformation and binding free energy of small molecule drugs and their targets can be used to assess drug-target binding affinity [61]. Autodock 4.2.6 was used for semiflexible molecular docking. As mentioned above, the three-dimensional chemical structure of baicalin was obtained from the PubChem database. The obtained SDF format file was converted into PDB format through openbabel 2.4.1 and used as the input file of Autodock. The target protein structures of baicalin’s antidepressant effect were acquired from the Protein Data Bank (PDB) (https://www.rcsb.org/, accessed on 7 May 2022) database. Structures co-crystallized with small molecules and with the highest resolution possible were selected as the structures for docking [62]. After docking, PyMOL (version 2.6.0a0) and LigPlus (version v.2.2.8) were used to visualize the results [63,64,65,66].

### 4.5. Differential Gene Expression Analysis

The gene expression difference analysis of the two datasets selected in the above steps was performed in order to further verify the antidepressant effect of baicalin at the gene level. The preprocessed data were input into DESeq2 package [46]. After fitting the generalized linear model (GLM) and executing empirical Bayesian contraction, the differentially expressed genes were screened under the conditions of |logFC| > 1 and *p* value < 0.05. The obtained genes are used to evaluate the differences in gene expression levels in the entries corresponding to depression or the use of baicalin, which are the significant results acquired from the above enrichment analysis.

### 4.6. Weighted Gene Co-Expression Network Analysis

The weighted gene co-expression network analysis was completed with the WGCNA package [47]. The clean expression matrix was inputted, and Pearson correlation coefficients (PCCs) were calculated to quantify the correlation between each pair of genes and to construct the co-expression similarity matrix. Soft threshold power (β) was then calculated and the appropriate one was selected to make the network relationship scale-free. The Dynamic Tree Cut method was used to identify gene modules. The topological overlap matrix (TOM) was constructed and the dissimilarity measure (1-TOM) was calculated, which is used for unsupervised clustering. According to the standard of the Dynamic Tree Cut method, the minimum number of genes in each module was set to 30 and the gene modules were determined. The feature vector of each module was calculated and clustering was performed again, which can make the modules close to each other merge together.

Subsequently, gene selection was executed based on phenotypic correlation. After the evaluation of the correlation between the obtained modules and phenotype was carried out, in the gene module with significant correlation (*p* value < 0.05), GS and MM were taken as the reference to select the genes in the module as the research object for enrichment analysis, which can provide evidence at the biological action level for baicalin’s antidepressant effect. GS refers to Gene Significance, which can be used to describe the correlation between genes and phenotypic traits. MM refers to Module Membership, which can act as the indication for the correlation between gene and module [67,68].

### 4.7. Animals

Male C57/BL 6J mice, weighing 25–30 g, were obtained from Jinan Peng-yue Experimental Animal Breeding Co., Ltd. (Jinan, China). All experimental procedures have been approved by the Ethics Committee of Shandong University (ECSBMSSDU-2022-2-65) and comply with the international guidelines for animal research formulated by the Council of International Medical Organizations. Before the experiment began, mice were housed under standard conditions for one week, freely obtaining food and water to adapt to the environment. During the experiment, efforts were made to reduce the pain of animals, including ensuring a quiet and comfortable basic living environment, constructing models strictly based on the patterns recorded in previous research, and anesthetizing and euthanizing mice before dissecting brain tissue.

### 4.8. Drugs

Baicalin with a purity of ≥98% was obtained from Chengdu Alfa Biotechnology Co., Ltd. (Chengdu, China). A suspension composed of baicalin and physiological saline with a concentration of 6 mg/mL was intragastrically administered to mice at a dose of 60 mg/(kg/day) [69].

### 4.9. Chronic Unpredicted Mild Stress (CUMS) Model and Intragastrical Administration

The mice were housed in the environment in the animal experimental room for one week. Subsequently, the mice were randomly divided into three groups, with 15 mice in each group. The first group, as the Control group, underwent natural feeding with free access to food and water for 4 weeks. The second group, as the CUMS group, was subjected to random chronic stressors once a day, including tail clipping (2 min), cold swimming (5 min, 4 °C), cage shaking (5 min), physical restraints (2 h), food and water deprivation (24 h), wet bedding (24 h), cage tilting (24 h), and overnight illumination, for a duration of four weeks [70]. The third group is the CUMS + Bai group, which was subjected to CUMS stimulation for four weeks, the same as for the CUMS group, but with intragastrically administered baicalin in the last three weeks.

### 4.10. Behavioral Tests

All behavioral tests of mice were conducted during the dark period (18:00–23:00), using the testing methods described in previous studies [71]. The experiments and analysis were conducted by an experimenter who was unaware of the treatment group.

#### 4.10.1. Sucrose Preference Test

The sucrose preference test (SPT) was used as a classical method to evaluate anhedonia in mice [72]. The mice were first placed separately in cages, then adapted to 2 bottles of 1% sucrose for 24 h, and then to 1 bottle of sucrose and 1 bottle of water for 24 h. After 24-h water deprivation, the mice were exposed to two bottles filled with either sucrose or water for 24 h. The bottle position was reversed after 12 h. The sucrose preference index is equal to sucrose consumption/(sucrose consumption + water consumption).

#### 4.10.2. Forced Swimming Test

The forced swimming test (FST) was used to evaluate behavioral despair in mice [73]. The mice were placed separately in a cylindrical glass container with a diameter of 12 cm and a height of 25 cm filled with water (25 ± 2 °C) for 6 min, with the depth of water being such that the animal’s hind limbs or tail could not touch the bottom. Immobility time was defined as the time when a mouse remained floating motionlessly or made only minimal movements necessary to maintain their heads above the water surface. The first 2 min of the 6-min testing period were the adaptation time, and the last 4 min the testing time, which should be recorded.

#### 4.10.3. Tail Suspension Test

The tail suspension test (TST) was also used to define behavioral despair in mice [74]. The mice were suspended separately at a height of 20 cm from the ground, and the immobility time in the last 4 min of the 6-min test was recorded. Immobility was defined as not attempting to move limbs and maintaining a vertical posture during the suspension process.

#### 4.10.4. Open Field Test

The Open Field Test (OFT) is a method to assess basic activities and anxiety-like behaviors [75]. A mice was placed in the center of a square field measuring 40 cm × 40 cm × 40.5 cm for 5 min, and its movements were recorded via a computerized video tracking system (SMART 2.5, Panlab Harvard Apparatus, Holliston, MA, USA). The open field was cleaned with 75% ethanol after testing each mouse to prevent the influence of factors such as the odor or excreta on the next mouse in the experiment. The total distance of locomotion and the time spent in the central area were recorded and analyzed.

#### 4.10.5. Elevated Plus Maze Test

Elevated plus maze (EPM) was used to evaluate anxiety in mice [75]. The maze was placed 40 cm above the ground, consisting of two open arms and two closed arms that are of an equal size of 30 cm × 5 cm, along with a square central platform. Timing started after placing a mouse on the central platform, and exploration of the maze was allowed for 5 min. The locomotion recording method is as same as for OFT. The maze was also cleaned with 75% ethanol after testing each mouse. The time spent in open arms and the number of open arm entries were recorded.

### 4.11. Reverse Transcription PCR (RT-PCR) and Real-Time Quantitative PCR

Seven mice per group were anesthetized with 1% sodium pentobarbital (8ml/kg, i.p.); the tissue of the hippocampus area was carefully dissected within 24 h after completion of behavioral tests, immediately frozen in liquid nitrogen, and stored at −80 °C for subsequent use. Total RNA was separated from hippocampal tissue using the SPARKeasy Improved Tissue/Cell RNA Kit (AC0202-B, Sparkjade, Jinan, China). After the concentration was measured, RNA was reverse transcribed into cDNA, using SureScript™ First-Strand cDNA Synthesis Kit (QP056, GeneCopoeia™, Rockville, Maryland, USA). Then the target genes were amplified using the specific primer sequences shown in Table 5 below when the BlazeTaq™ SYBR Green qPCR Mix 2.0 (QP031, GeneCopoeia™) was used. Bio-Rad’s IQ5 software (version 4.1.2433.1219) was used to analyze PCR data, and quantitative data were normalized based on GAPDH.

### 4.12. Western Blot Analysis

Hippocampal tissue of 4 mice per group was homogenized with a mixture of protease/phosphatase inhibitors (P1260, Solarbio, Beijing, China) in cold RIPA buffer (R0020, Solarbio), the homogenate was centrifuged at 12,000× *g* for 25 min at 4 °C, and then the supernatant was collected. The BCA Protein Assay Kits (CW0014s, CWBIO, Taizhou, China) were used to determine protein concentration in the supernatant obtained from the previous steps. Heated with the loading buffer at 100 °C for 5 min, the protein samples were added to each lane at a quantity of 20 μg and electrophoretically separated on SDS-PAGE gels. Transferred onto PVDF membranes, the separated protein bands were blocked for 1 h in 5% skim milk, and then incubated overnight with primary antibodies at 4 °C. The primary antibodies used included anti-Cleaved Caspase 3 (1:1000, 9661S, Cell Signaling Technology, Danvers, MA, USA), anti-AKT (1:1000, 9272S, Cell Signaling Technology), anti-P53 (1:5000, 10442-1-AP, Proteintech, Rosemont, IL, USA), and anti-glyceraldehyde-3-phase dehydrogenase (GAPDH, 1:5000, 10494-1-AP, Proteintech). After cleaning by TBST, the membranes were incubated with secondary horseradish peroxidase-conjugated antibodies goat anti-rabbit IgG (1:4000, ZB-2301, ZSGB-BIO, Beijing, China). The Enhanced Chemiluminescence Kit (catalog E422-01, Vazyme, Nanjing, China) was used to detect bands on the membranes and ImageJ software (version 1.44p) was used to quantify the density of detected protein bands.

### 4.13. Immunofluorescence Staining

After being anesthetized, 3 mice per group were sequentially perfused intracardially with heparin sodium saline and 4% paraformaldehyde (PFA). The mice brains were removed and fixed overnight in PFA, and then they were transferred to a 10% to 30% sucrose gradient for 3 days for graded dehydration at 4 °C. The freezing microtome (CM1860, Leica, Wetzlar, Germany) was used to cut brain tissues into slices with a thickness of 30 μm. The slices were washed in PBS and then incubated in blocking solution (1 × PBS + 5% goat serum + 2.5% BSA + 0.2% Triton X-100) for 1 h. Then, the slices were incubated overnight with primary antibodies, which include anti-Iba-1 (1:200, 17198S, Cell Signaling Technology) and anti-Cleaved Caspase 3 (1:400, 9661S, Cell Signaling Technology), in the blocking solution at 4 °C, washed with PBS, and incubated with fluorescent secondary antibodies Alexa Fluor 488 goat anti-rabbit immunoglobulin G (IgG) (SA00013-2, Proteintech) under dark conditions for 1 h at 37 °C. The nucleus was stained with DAPI (C1002, Beyotime, Shanghai, China) for 5 min at 37 °C after being washed with PBS again. After washing the brain slices, glass slide specimens were made. Image acquisition was performed with the use of a high-speed confocal platform (Dragonfly 200, Oxford Instruments, Oxford, UK), with excitation light of 488 nm and z-stack distance of 20 μm. For brain slices stained with Iba-1, the number of microglia was evaluated by counting the number of Iba-1-positive cells in the same square measure, and the activation of microglia was evaluated by the size of cell bodies and the structure of synapses. For brain slices stained with Cleaved Caspase 3, apoptosis was characterized by relative fluorescence intensity under the same square measure.

### 4.14. Statistical Analysis

All data in the animal experiment section were reported as means ± SEMs and statistically analyzed using GraphPad Prism software version 8.0. One-way analysis of variance (ANOVA) was used to analyze comparisons between three groups, followed by Tukey’s post hoc test for multiple comparisons. The statistical significance was set at *p* < 0.05.

### 4.15. Ethics Approval and Informed Consent

The ethical approval of experimental animals is as mentioned above. All other data used were obtained from the online public database and there is no personal privacy information for patients involved in this research.

## Figures and Tables

**Table 1:** The parameters of druggability for baicalin.

Parameter	Value	Reference
Hydrogen bond donor count	6	≤5
Hydrogen bond acceptor count	11	≤10
Molecular weight	446.4	<500
Octanol/water partition coefficient	1.1	≤5
Rotatable bond count	4	≤10
Polar surface area (Å^2^)	183	≤140
Oral bioavailability (%)	29.53	≥20
Drug-likeness	0.77	≥0.1

**Table 2:** Topological parameters of the top 20 target genes of baicalin.

Gene Symbol	ASPL	Betweenness Centrality	Closeness Centrality	Clustering Coefficient	Degree
*RELA*	2.0625	0.36635005	0.48484848	0.24166667	16
*TP53*	2.04166667	0.34916413	0.48979592	0.11428571	15
*MAPK1*	2.20833333	0.21595323	0.45283019	0.16483516	14
*TNF*	2.60416667	0.09610351	0.384	0.26666667	10
*AKT1*	2.5	0.24545128	0.4	0.08333333	9
*IL6*	2.41666667	0.03947357	0.4137931	0.46428571	8
*IL1B*	2.77083333	0.02122383	0.36090226	0.47619048	7
*IL2*	2.375	0.06283983	0.42105263	0.46666667	6
*TLR4*	2.83333333	0.04262707	0.35294118	0.53333333	6
*NFKBIA*	2.39583333	0.02804162	0.4173913	0.46666667	6
*CCND1*	2.52083333	0.01892942	0.39669421	0.3	5
*EGFR*	2.70833333	0.1214539	0.36923077	0.2	5
*NOTCH1*	3.35416667	0.12012411	0.29813665	0.33333333	4
*CASP3*	2.64583333	0.00757979	0.37795276	0.66666667	4
*NOS2*	2.60416667	0.03900709	0.384	0.5	4
*BCL2*	2.64583333	0.0114805	0.37795276	0.5	4
*PTGS2*	2.6875	0.04794622	0.37209302	0.16666667	4
*ATF2*	2.60416667	0.00143322	0.384	0.66666667	4
*MYD88*	2.91666667	0.00088652	0.34285714	0.66666667	4
*TGFB1*	2.72916667	0.00150709	0.36641221	0.66666667	4

**Table 3:** Topological parameters of the top 20 target genes of depression.

Gene Symbol	ASPL	Betweenness Centrality	Closeness Centrality	Clustering Coefficient	Degree
*TP53*	2.64510914	0.09478941	0.37805623	0.06731985	113
*EP300*	2.6523848	0.05607056	0.3770192	0.10060606	100
*CTNNB1*	2.64349232	0.06465836	0.37828746	0.08157895	96
*PIK3R1*	2.74777688	0.01763209	0.36393057	0.16117216	91
*PIK3CA*	2.72190784	0.02173176	0.36738937	0.16434676	91
*HRAS*	2.74454325	0.02687518	0.36435935	0.16317671	89
*MAPK1*	2.75101051	0.01976236	0.36350279	0.14883721	86
*MAPK3*	2.75505255	0.01753599	0.36296948	0.15376197	86
*STAT3*	2.77041229	0.02476242	0.36095711	0.15253165	80
*RPS27A*	2.84236055	0.06752715	0.35182025	0.0691334	79
*AKT1*	2.66370251	0.04034818	0.3754173	0.12720613	78
*PTPN11*	2.87146322	0.01752892	0.3482545	0.19824561	76
*CREBBP*	2.71544058	0.03602726	0.36826436	0.12699	74
*KRAS*	2.80679062	0.01429623	0.3562788	0.20372671	70
*RAC1*	2.89814066	0.02085928	0.34504881	0.15364355	68
*SOS1*	2.91835085	0.00537091	0.34265928	0.26686508	64
*NRAS*	2.94017785	0.00885878	0.34011548	0.2202381	64
*MAPK8*	2.82538399	0.02066826	0.35393419	0.12544803	63
*ESR1*	2.74454325	0.03216523	0.36435935	0.15901639	61
*JAK2*	2.95149555	0.01048452	0.33881128	0.1954023	58

**Table 4:** Topological parameters of the top 20 target genes of Baicalin on depression.

Gene Symbol	ASPL	Betweenness Centrality	Closeness Centrality	Clustering Coefficient	Degree
*AKT1*	1.30769231	0.18065866	0.76470588	0.45238095	28
*IL6*	1.53846154	0.05851599	0.65	0.55797101	24
*IL1B*	1.48717949	0.07172935	0.67241379	0.56126482	23
*TP53*	1.58974359	0.03567395	0.62903226	0.60474308	23
*CASP3*	1.53846154	0.03535266	0.65	0.64069264	22
*MAPK1*	1.56410256	0.06271286	0.63934426	0.58421053	20
*PPARG*	1.66666667	0.02191758	0.6	0.68947368	20
*PTGS2*	1.69230769	0.00928826	0.59090909	0.76608187	19
*CCND1*	1.71794872	0.01594669	0.58208955	0.67251462	19
*EGF*	1.71794872	0.01524371	0.58208955	0.70175439	19
*TGFB1*	1.71794872	0.01121916	0.58208955	0.75163399	18
*IL2*	1.71794872	0.01244353	0.58208955	0.76470588	18
*CASP9*	1.74358974	0.00703711	0.57352941	0.79411765	17
*TLR4*	1.74358974	0.01616254	0.57352941	0.72058824	17
*GPT*	1.76923077	0.0714995	0.56521739	0.675	16
*HMOX1*	1.79487179	0.00105304	0.55714286	0.93333333	15
*NOS2*	1.69230769	0.0320215	0.59090909	0.8021978	14
*CDKN2A*	1.84615385	0.00177688	0.54166667	0.87912088	14
*GRIN1*	1.79487179	0.17178252	0.55714286	0.25454545	11
*HNF4A*	1.92307692	0.01964939	0.52	0.65454545	11

**Table 5:** Specific primer sequences used in PCR.

Target Gene	Specific Primer Sequences
IL-1β	forward primer: 5′-AAGATGAAGGGCTGCTTCCAAACC-3′ reverse primer: 5′-ATACTGCCTGCCTGAAGCTCTTGT-3′
IL-6	forward primer: 5′-TGATCGGTCCCAACAAGGA-3′ reverse primer: 5′-CCACTTGTTGGCTTATGTT-3′
Caspase 3	forward primer: 5′-GGAGCTTGGAACGCGAAGAA-3′ reverse primer: 5′-ACACAAGCCCATTTCAGGGT-3′
AKT	forward primer: 5′-GTTTTGTTTCTCGGATGCGCT-3′ reverse primer: 5′-CATGGTCGCGTCAGTCCTTA-3′
P53	forward primer: 5′-GGCGTAAACGCTTCGAGATG-3′ reverse primer: 5′-CTTCAGGTAGCTGGAGTGAGC-3′
GAPDH	forward primer: 5′-TCTCTGCTCCTCCCTGTTC-3′ reverse primer: 5′-ACACCGACCTTCACCATCT-3′

## Data Availability

The transcriptome data mentioned above is from the NCBI GEO database (https://www.ncbi.nlm.nih.gov/geo/, accessed on 27 April 2022). Other data used to support the findings of this study are available from the corresponding authors upon request.

## Supplementary Materials

The following supporting information can be downloaded at: https://www.mdpi.com/article/10.3390/ijms251910259/s1.

## References

[1] Smith K. (2014). Mental health: A world of depression. Nature.

[2] Dattani S., Ritchie H., Roser M. Mental Health. https://ourworldindata.org/mental-health.

[3] Bueno-Notivol J., Gracia-Garcia P., Olaya B., Lasheras I., Lopez-Anton R., Santabarbara J. (2021). Prevalence of depression during the COVID-19 outbreak: A meta-analysis of community-based studies. Int. J. Clin. Health Psychol..

[4] World Health Organization (2017). Depression and Other Common Mental Disorders: Global Health Estimates.

[5] Lively S., Schlichter L.C. (2018). Microglia Responses to Pro-inflammatory Stimuli (LPS, IFNgamma+TNFalpha) and Reprogramming by Resolving Cytokines (IL-4, IL-10). Front. Cell Neurosci..

[6] Raison C.L., Dantzer R., Kelley K.W., Lawson M.A., Woolwine B.J., Vogt G., Spivey J.R., Saito K., Miller A.H. (2010). CSF concentrations of brain tryptophan and kynurenines during immune stimulation with IFN-alpha: Relationship to CNS immune responses and depression. Mol. Psychiatry.

[7] Schwarcz R., Stone T.W. (2017). The kynurenine pathway and the brain: Challenges, controversies and promises. Neuropharmacology.

[8] Pariante C.M. (2017). Why are depressed patients inflamed? A reflection on 20 years of research on depression, glucocorticoid resistance and inflammation. Eur. Neuropsychopharmacol..

[9] Dantzer R. (2018). Neuroimmune Interactions: From the Brain to the Immune System and Vice Versa. Physiol. Rev..

[10] Pandya C.D., Howell K.R., Pillai A. (2013). Antioxidants as potential therapeutics for neuropsychiatric disorders. Prog. Neuropsychopharmacol. Biol. Psychiatry.

[11] Black C.N., Bot M., Scheffer P.G., Cuijpers P., Penninx B.W. (2015). Is depression associated with increased oxidative stress? A systematic review and meta-analysis. Psychoneuroendocrinology.

[12] Ekdahl C.T., Claasen J.H., Bonde S., Kokaia Z., Lindvall O. (2003). Inflammation is detrimental for neurogenesis in adult brain. Proc. Natl. Acad. Sci. USA.

[13] Kubera M., Obuchowicz E., Goehler L., Brzeszcz J., Maes M. (2011). In animal models, psychosocial stress-induced (neuro)inflammation, apoptosis and reduced neurogenesis are associated to the onset of depression. Prog. Neuropsychopharmacol. Biol. Psychiatry.

[14] Wooff Y., Man S.M., Aggio-Bruce R., Natoli R., Fernando N. (2019). IL-1 Family Members Mediate Cell Death, Inflammation and Angiogenesis in Retinal Degenerative Diseases. Front. Immunol..

[15] Bhatt S., Nagappa A.N., Patil C.R. (2020). Role of oxidative stress in depression. Drug Discov. Today.

[16] Sabella D. (2018). Antidepressant Medications. Am. J. Nurs..

[17] Li C., Huang J., Cheng Y.C., Zhang Y.W. (2020). Traditional Chinese Medicine in Depression Treatment: From Molecules to Systems. Front. Pharmacol..

[18] Zhang Y., Long Y., Yu S., Li D., Yang M., Guan Y., Zhang D., Wan J., Liu S., Shi A. (2021). Natural volatile oils derived from herbal medicines: A promising therapy way for treating depressive disorder. Pharmacol. Res..

[19] Sowndhararajan K., Deepa P., Kim M., Park S.J., Kim S. (2018). Neuroprotective and Cognitive Enhancement Potentials of Baicalin: A Review. Brain Sci..

[20] Huang T., Liu Y., Zhang C. (2019). Pharmacokinetics and Bioavailability Enhancement of Baicalin: A Review. Eur. J. Drug Metab. Pharmacokinet..

[21] Li Y., Song K., Zhang H., Yuan M., An N., Wei Y., Wang L., Sun Y., Xing Y., Gao Y. (2020). Anti-inflammatory and immunomodulatory effects of baicalin in cerebrovascular and neurological disorders. Brain Res. Bull..

[22] Guo L.T., Wang S.Q., Su J., Xu L.X., Ji Z.Y., Zhang R.Y., Zhao Q.W., Ma Z.Q., Deng X.Y., Ma S.P. (2019). Baicalin ameliorates neuroinflammation-induced depressive-like behavior through inhibition of toll-like receptor 4 expression via the PI3K/AKT/FoxO1 pathway. J. Neuroinflamm..

[23] Lu Y., Sun G., Yang F., Guan Z., Zhang Z., Zhao J., Liu Y., Chu L., Pei L. (2019). Baicalin regulates depression behavior in mice exposed to chronic mild stress via the Rac/LIMK/cofilin pathway. Biomed. Pharmacother..

[24] Jia Z., Yang J., Cao Z., Zhao J., Zhang J., Lu Y., Chu L., Zhang S., Chen Y., Pei L. (2021). Baicalin ameliorates chronic unpredictable mild stress-induced depression through the BDNF/ERK/CREB signaling pathway. Behav. Brain Res..

[25] Li S., Zhang B. (2013). Traditional Chinese medicine network pharmacology: Theory, methodology and application. Chin. J. Nat. Med..

[26] Casas A.I., Hassan A.A., Larsen S.J., Gomez-Rangel V., Elbatreek M., Kleikers P.W.M., Guney E., Egea J., Lopez M.G., Baumbach J. (2019). From single drug targets to synergistic network pharmacology in ischemic stroke. Proc. Natl. Acad. Sci. USA.

[27] Li J., Zhao P., Tian Y., Li K., Zhang L., Guan Q., Mei X., Qin Y. (2021). The Anti-Inflammatory Effect of a Combination of Five Compounds from Five Chinese Herbal Medicines Used in the Treatment of COPD. Front. Pharmacol..

[28] Berk M., Williams L.J., Jacka F.N., O’Neil A., Pasco J.A., Moylan S., Allen N.B., Stuart A.L., Hayley A.C., Byrne M.L. (2013). So depression is an inflammatory disease, but where does the inflammation come from?. BMC Med..

[29] Cummings C.M., Caporino N.E., Kendall P.C. (2014). Comorbidity of anxiety and depression in children and adolescents: 20 years after. Psychol. Bull..

[30] Lenze E.J. (2003). Comorbidity of depression and anxiety in the elderly. Curr. Psychiatry Rep..

[31] Brunet A., Bonni A., Zigmond M.J., Lin M.Z., Juo P., Hu L.S., Anderson M.J., Arden K.C., Blenis J., Greenberg M.E. (1999). Akt promotes cell survival by phosphorylating and inhibiting a Forkhead transcription factor. Cell.

[32] Mihara M., Hashizume M., Yoshida H., Suzuki M., Shiina M. (2012). IL-6/IL-6 receptor system and its role in physiological and pathological conditions. Clin. Sci..

[33] Rose-John S. (2012). IL-6 trans-signaling via the soluble IL-6 receptor: Importance for the pro-inflammatory activities of IL-6. Int. J. Biol. Sci..

[34] Campbell I.L., Abraham C.R., Masliah E., Kemper P., Inglis J.D., Oldstone M.B., Mucke L. (1993). Neurologic disease induced in transgenic mice by cerebral overexpression of interleukin 6. Proc. Natl. Acad. Sci. USA.

[35] Roohi E., Jaafari N., Hashemian F. (2021). On inflammatory hypothesis of depression: What is the role of IL-6 in the middle of the chaos?. J. Neuroinflamm..

[36] Levine J., Barak Y., Chengappa K.N., Rapoport A., Rebey M., Barak V. (1999). Cerebrospinal cytokine levels in patients with acute depression. Neuropsychobiology.

[37] Tsai S.J. (2017). Effects of interleukin-1beta polymorphisms on brain function and behavior in healthy and psychiatric disease conditions. Cytokine Growth Factor Rev..

[38] Owen B.M., Eccleston D., Ferrier I.N., Young A.H. (2001). Raised levels of plasma interleukin-1beta in major and postviral depression. Acta Psychiatr. Scand..

[39] Mahmood S., Evinova A., Skerenova M., Ondrejka I., Lehotsky J. (2016). Association of EGF, IGFBP-3 and TP53 Gene Polymorphisms with Major Depressive Disorder in Slovak Population. Cent. Eur. J. Public Health.

[40] Ranjan A., Iwakuma T. (2016). Non-Canonical Cell Death Induced by p53. Int. J. Mol. Sci..

[41] Bakunina N., Pariante C.M., Zunszain P.A. (2015). Immune mechanisms linked to depression via oxidative stress and neuroprogression. Immunology.

[42] Arantes-Goncalves F., Coelho R. (2006). Depression and treatment. Apoptosis, neuroplasticity and antidepressants. Acta Med. Port..

[43] Spencer S.L., Sorger P.K. (2011). Measuring and modeling apoptosis in single cells. Cell.

[44] Saveanu R.V., Nemeroff C.B. (2012). Etiology of depression: Genetic and environmental factors. Psychiatr. Clin. N. Am..

[45] Morris G.M., Lim-Wilby M. (2008). Molecular docking. Methods Mol. Biol..

[46] Love M.I., Huber W., Anders S. (2014). Moderated estimation of fold change and dispersion for RNA-seq data with DESeq2. Genome Biol..

[47] Langfelder P., Horvath S. (2008). WGCNA: An R package for weighted correlation network analysis. BMC Bioinform..

[48] Lipinski C.A., Lombardo F., Dominy B.W., Feeney P.J. (2001). Experimental and computational approaches to estimate solubility and permeability in drug discovery and development settings. Adv. Drug Deliv. Rev..

[49] Veber D.F., Johnson S.R., Cheng H.Y., Smith B.R., Ward K.W., Kopple K.D. (2002). Molecular properties that influence the oral bioavailability of drug candidates. J. Med. Chem..

[50] Shen M., Tian S., Li Y., Li Q., Xu X., Wang J., Hou T. (2012). Drug-likeness analysis of traditional Chinese medicines: 1. property distributions of drug-like compounds, non-drug-like compounds and natural compounds from traditional Chinese medicines. J. Cheminform..

[51] Tao W., Xu X., Wang X., Li B., Wang Y., Li Y., Yang L. (2013). Network pharmacology-based prediction of the active ingredients and potential targets of Chinese herbal Radix Curcumae formula for application to cardiovascular disease. J. Ethnopharmacol..

[52] Fang S., Dong L., Liu L., Guo J., Zhao L., Zhang J., Bu D., Liu X., Huo P., Cao W. (2021). HERB: A high-throughput experiment- and reference-guided database of traditional Chinese medicine. Nucleic Acids Res..

[53] Cao X., Zao X., Xue B., Chen H., Zhang J., Li S., Li X., Zhu S., Guo R., Li X. (2021). The mechanism of TiaoGanYiPi formula for treating chronic hepatitis B by network pharmacology and molecular docking verification. Sci. Rep..

[54] Dusa A. (2021). venn: Draw Venn Diagrams, R Package Version 1.10. https://github.com/dusadrian/venn.

[55] Shannon P., Markiel A., Ozier O., Baliga N.S., Wang J.T., Ramage D., Amin N., Schwikowski B., Ideker T. (2003). Cytoscape: A software environment for integrated models of biomolecular interaction networks. Genome Res..

[56] Martin A., Ochagavia M.E., Rabasa L.C., Miranda J., Fernandez-de-Cossio J., Bringas R. (2010). BisoGenet: A new tool for gene network building, visualization and analysis. BMC Bioinform..

[57] Liu X., Hong Z., Liu J., Lin Y., Rodriguez-Paton A., Zou Q., Zeng X. (2020). Computational methods for identifying the critical nodes in biological networks. Brief Bioinform..

[58] Wu T., Hu E., Xu S., Chen M., Guo P., Dai Z., Feng T., Zhou L., Tang W., Zhan L. (2021). clusterProfiler 4.0: A universal enrichment tool for interpreting omics data. Innovation.

[59] Ashburner M., Ball C.A., Blake J.A., Botstein D., Butler H., Cherry J.M., Davis A.P., Dolinski K., Dwight S.S., Eppig J.T. (2000). Gene ontology: Tool for the unification of biology. The Gene Ontology Consortium. Nat. Genet..

[60] Gene Ontology Consortium (2008). The Gene Ontology project in 2008. Nucleic Acids Res..

[61] Forli S., Huey R., Pique M.E., Sanner M.F., Goodsell D.S., Olson A.J. (2016). Computational protein-ligand docking and virtual drug screening with the AutoDock suite. Nat. Protoc..

[62] Huo M., Ma L., Liu G. (2021). Exploring the mechanism of Yixinyin for myocardial infarction by weighted co-expression network and molecular docking. Sci. Rep..

[63] Laskowski R.A., Swindells M.B. (2011). LigPlot+: Multiple ligand-protein interaction diagrams for drug discovery. J. Chem. Inf. Model..

[64] Schrodinger, LLC (2015). The AxPyMOL Molecular Graphics Plugin for Microsoft PowerPoint, Version 1.8.

[65] Schrodinger, LLC (2015). The JyMOL Molecular Graphics Development Component, Version 1.8.

[66] Schrodinger, LLC (2015). The PyMOL Molecular Graphics System, Version 1.8.

[67] Zhou W., Wu J., Zhang J., Liu X., Guo S., Jia S., Zhang X., Zhu Y., Wang M. (2020). Integrated bioinformatics analysis to decipher molecular mechanism of compound Kushen injection for esophageal cancer by combining WGCNA with network pharmacology. Sci. Rep..

[68] Zhang Y., Luo J., Liu Z., Liu X., Ma Y., Zhang B., Chen Y., Li X., Feng Z., Yang N. (2021). Identification of hub genes in colorectal cancer based on weighted gene co-expression network analysis and clinical data from The Cancer Genome Atlas. Biosci. Rep..

[69] Fu Y.J., Xu B., Huang S.W., Luo X., Deng X.L., Luo S., Liu C., Wang Q., Chen J.Y., Zhou L. (2021). Baicalin prevents LPS-induced activation of TLR4/NF-κB p65 pathway and inflammation in mice via inhibiting the expression of CD14. Acta Pharmacol. Sin..

[70] Ma H., Wang W., Xu S., Wang L., Wang X. (2018). Potassium 2-(1-hydroxypentyl)-benzoate improves depressive-like behaviors in rat model. Acta Pharm. Sin. B.

[71] Li Y., Fan C., Wang C., Wang L., Yi Y., Mao X., Chen X., Lan T., Wang W., Yu S.Y. (2022). Stress-induced reduction of Na(+)/H(+) exchanger isoform 1 promotes maladaptation of neuroplasticity and exacerbates depressive behaviors. Sci. Adv..

[72] Secoli S.R., Teixeira N.A. (1998). Chronic prenatal stress affects development and behavioral depression in rats. Stress.

[73] Porsolt R.D., Le Pichon M., Jalfre M. (1977). Depression: A new animal model sensitive to antidepressant treatments. Nature.

[74] Cryan J.F., Mombereau C., Vassout A. (2005). The tail suspension test as a model for assessing antidepressant activity: Review of pharmacological and genetic studies in mice. Neurosci. Biobehav. Rev..

[75] Zhang M., Liu Y., Zhao M., Tang W., Wang X., Dong Z., Yu S. (2017). Depression and anxiety behaviour in a rat model of chronic migraine. J. Headache Pain.

