# Supplementary material for: Baicalin Ameliorates Depression-like Behaviors via Inhibiting Neuroinflammation and Apoptosis in Mice
Source: Int J Mol Sci. 2024 Sep 24;25(19):10259. doi: 10.3390/ijms251910259 (PMC11476789; doi:10.3390/ijms251910259)
Supplement: Supplementary file 1 [file ijms-25-10259-s001.zip › Supplementary Figure.pdf]

Baicalin Ameliorates Depression-like Behaviors via Inhibiting Neuroinflammation and Apoptosis in Mice

Yuhang Yi [1], Guiyu Liu [1], Ye Li [1], Changmin Wang [1], Bin Zhang [2], Haiyan Lou [2,†] and Shuyan Yu [1,3,*,†]

[1] Department of Physiology, School of Basic Medical Sciences, Cheeloo College of Medicine, Shandong University, Jinan 250012, China; 201917413015@mail.sdu.edu.cn (Y.Y.); 201900412050@mail.sdu.edu.cn (G.L.); 202390000063@sdu.edu.cn (Y.L.); 202421115@mail.sdu.edu.cn (C.W.)
[2] Department of Pharmacology, School of Basic Medical Sciences, Cheeloo College of Medicine, Shandong University, Jinan 250012, China; binzhang@sdu.edu.cn (B.Z.); louhaiyan@sdu.edu.cn (H.L.)
[3] Shandong Provincial Key Laboratory of Mental Disorders, School of Basic Medical Sciences, Jinan 250012, China
* Correspondence: shuyanyu@sdu.edu.cn; Tel.: +86-0531-88383902; Fax: +86-0531-88382502
† These authors contributed equally to this work.

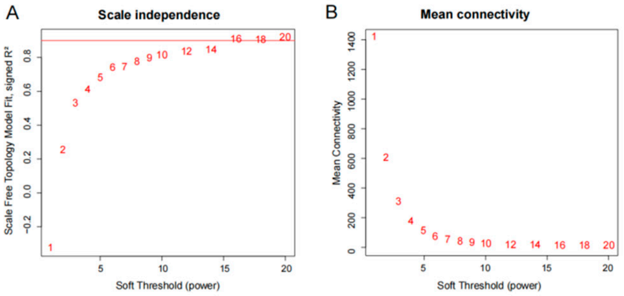

C

Soft Threshold Information

Power	SFT.R.sq	slope	truncated.R.sq	mean.k.	median.k.	max.k.
1	0.32423333	1.407716349	0.878447271	1411.34669	1439.17387	2424.88911
2	0.25098296	-0.65801963	0.785823696	592.712356	568.777102	1455.17
3	0.53002037	-1.13296989	0.866655175	298.387656	262.176606	955.467098
4	0.60909275	-1.40125389	0.905531976	167.259719	135.540217	661.897186
5	0.67941752	-1.53025681	0.945616651	100.869372	76.5233489	475.831378
6	0.73845536	-1.62124215	0.967987705	64.218713	44.7589358	351.720056
7	0.74406648	-1.7393897	0.968464275	42.6605532	27.1458938	265.768377
8	0.77422455	-1.7950691	0.978181099	29.3405736	17.0336918	204.481575
9	0.79460731	-1.80301804	0.984694519	20.7775445	10.8939024	159.738282
10	0.81303496	-1.79665337	0.987624528	15.0881517	7.23618882	126.424545
12	0.83264791	-1.78425789	0.982833943	8.47936412	3.32102739	82.1274254
14	0.84567021	-1.72235463	0.969831416	5.11556324	1.64780529	55.4629254
16	0.90812403	-1.53905161	0.983593134	3.27411484	0.86570795	38.553389
18	0.90308498	-1.58551032	0.970245075	2.20460704	0.47097892	31.7976923
20	0.91731343	-1.59110852	0.980543219	1.55193896	0.2647084	27.4709488

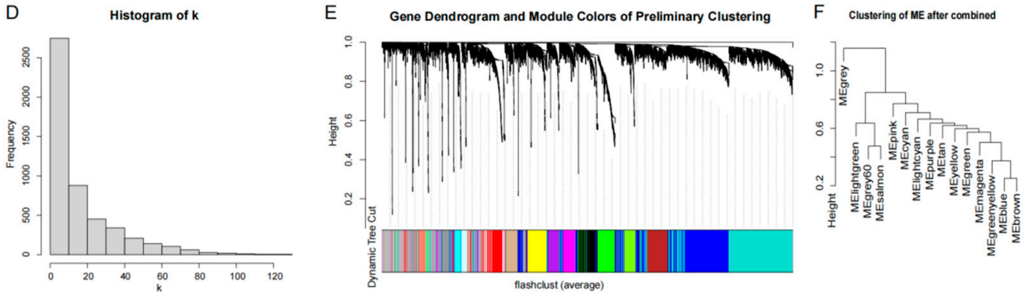

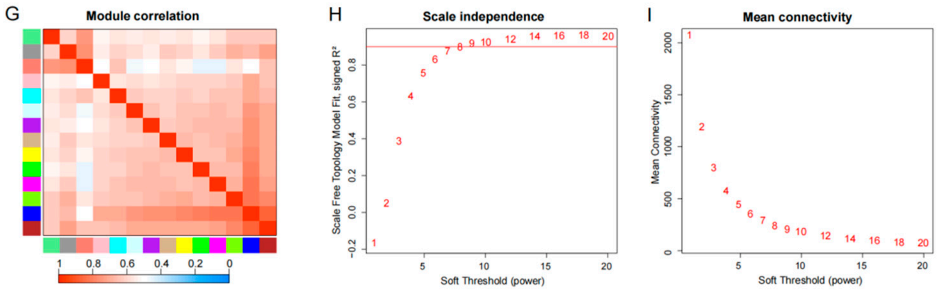

J

Soft Threshold Information

Power	SFT.R.sq	slope	truncated.R.sq	mean.k.	median.k.	max.k.
1	0.176576069	1.152314739	0.449939403	2055.423111	2031.515745	2625.13019
2	0.039026759	-0.259451871	0.314026031	1176.616728	1127.414647	1797.54195
3	0.37604738	-0.71400637	0.553905927	778.2620902	714.9053339	1366.69809
4	0.622398203	-0.953694102	0.731100839	559.1679289	494.9837017	1099.40064
5	0.746568973	-1.091704664	0.823358273	424.0609237	361.320516	916.154929
6	0.820020844	-1.199133994	0.877253372	334.1375118	275.1579775	782.388458
7	0.867189314	-1.270130342	0.908837744	270.9160792	216.8341774	680.336899
8	0.888915311	-1.332289266	0.924797781	224.5910852	175.3485195	599.88851
9	0.908866188	-1.370908746	0.942375886	189.5300025	144.5325273	534.845726
10	0.914975972	-1.407863887	0.946828928	162.2933503	121.8737631	481.189856
12	0.929822128	-1.46550743	0.958403965	123.2076893	89.68128577	397.950375
14	0.946513218	-1.504081762	0.973383371	96.95659406	68.7033569	336.509573
16	0.949626635	-1.538746492	0.97517509	78.41808357	54.34092209	289.44107
18	0.951810142	-1.571265791	0.977647708	64.81081635	43.99943736	252.34853
20	0.944678866	-1.605352963	0.972865741	54.51192538	36.39550615	222.45869

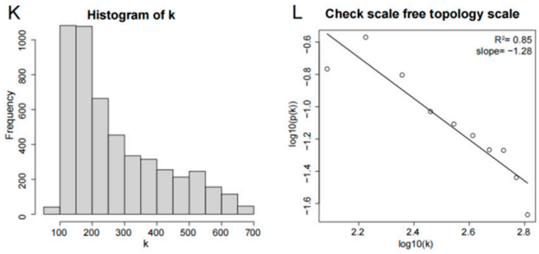

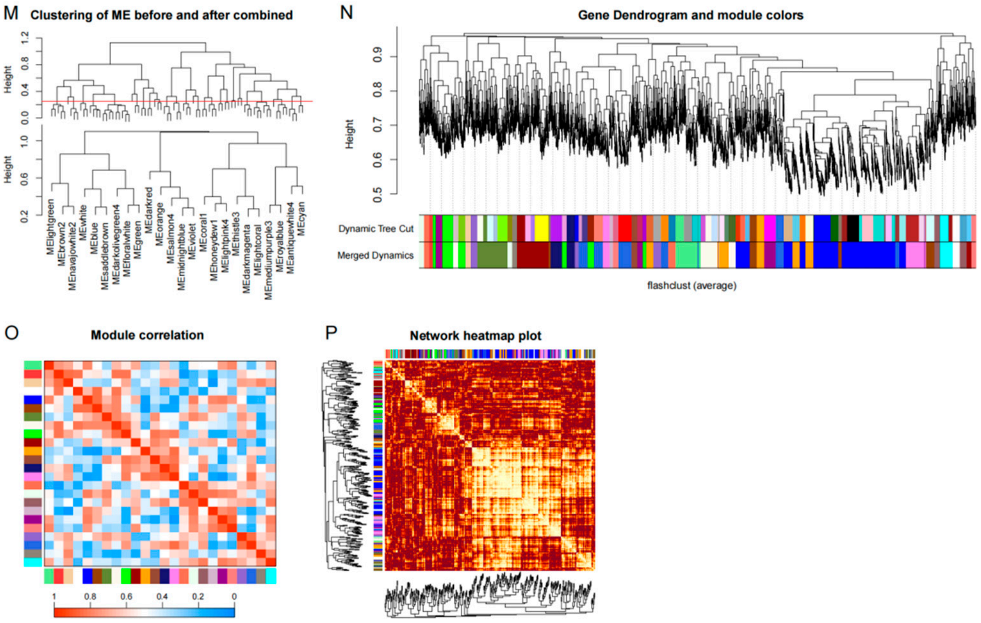

Supplementary Figure S1. Supplementary information of the process and results of WGCNA. (A) Evaluation of the scale independence of the network in different β value for the dataset GSE42546. (B) Evaluation of the mean connectivity of the network in different β value. (C) Information of soft threshold β. (D) Histogram of connectivity k. (E) Gene dendrogram and module colors of preliminary clustering. (F) Clustering of modules after combined. (G) The correlation between modules. (H) Evaluation of the scale independence of the network in different β value for the dataset GSE160587. (I) Evaluation of the mean connectivity of the network in different β value. (J) Information of soft threshold β. (K) Histogram of connectivity k. (L) The inverse proportional fitting curve suggests that the constructed network has the characteristics of scale-free. (M) Clustering and combination of gene modules. (N) Gene dendrogram and module colors before and after module merging. (O) The correlation between modules. (P) Visualization of weighted network by heatmap.

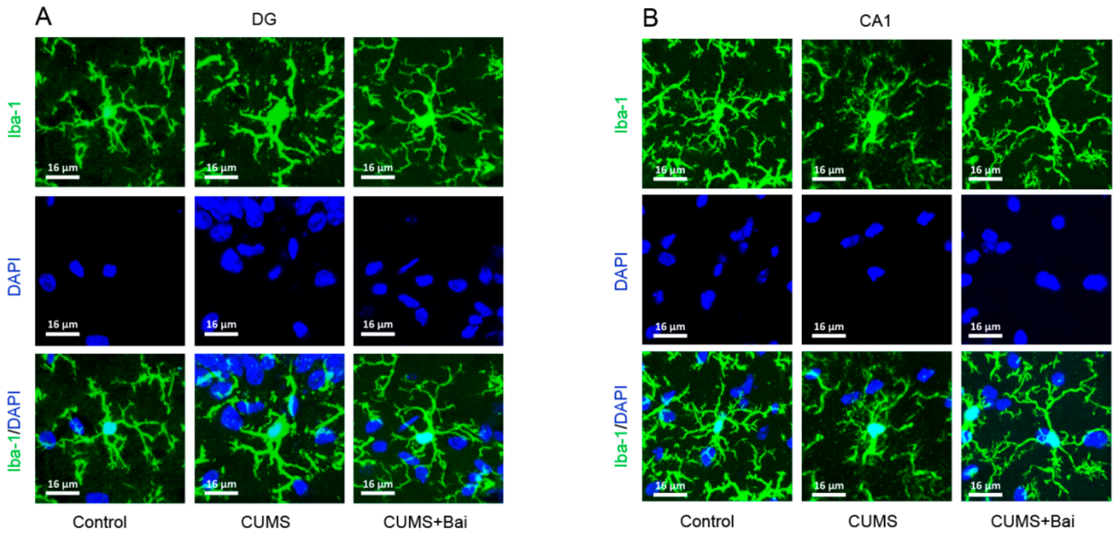

Supplementary Figure S2. Supplementary information of Baicalin exerting antidepressant effects by reducing neuroinflammation. (A) Representative

diagram of the morphology of Iba-1-positive microglia in the DG region of the mouse hippocampus by immunofluorescence staining. (B) Representative diagram of the morphology of Iba-1-positive microglia in the CA1 region of the mouse hippocampus by immunofluorescence staining.
