# Supplementary material for: Baicalin Ameliorates Depression-like Behaviors via Inhibiting Neuroinflammation and Apoptosis in Mice
Source: Int J Mol Sci. 2024 Sep 24;25(19):10259. doi: 10.3390/ijms251910259 (PMC11476789; doi:10.3390/ijms251910259)
Supplement: Supplementary file 1 [file ijms-25-10259-s001.zip › Supplementary Table S1-S10.pdf]

Supplementary Table S1: Targets gathering of baicalin from different database

Targets of baicalin gathering from HERB

Paper id	Target id	Target name	PubMed id	Gene_name_full	Ensembl_Gene_ID	ENTREZ_gene_id	Species
HBREF001714	HBTAR000644	CDKN2A	29440765	cyclin dependent kinase inhibitor 2A(CDKN2A)	ENSG00000147889	1029	Homo sapiens
HBREF001714	HBTAR006174	DEPP1	29440765	DEPP1 autophagy regulator(DEPP1)	ENSG00000165507	11067	Homo sapiens
HBREF001714	HBTAR006379	ZHX2	29440765	zinc fingers and homeoboxes 2(ZHX2)	ENSG00000178764	22882	Homo sapiens
HBREF001714	HBTAR003192	MAPK1	29440765	mitogen-activated protein kinase 1(MAPK1)	ENSG00000100030	5594	Homo sapiens
HBREF001714	HBTAR003207	MAP2K7	29440765	mitogen-activated protein kinase kinase 7(MAP2K7)	ENSG00000076984	5609	Homo sapiens
HBREF001715	HBTAR000517	CASP3	22607709	caspase 3(CASP3)	ENSG00000164305	836	Homo sapiens
HBREF001715	HBTAR000523	CASP9	22607709	caspase 9(CASP9)	ENSG00000132906	842	Homo sapiens
HBREF001715	HBTAR000130	AKT1	22607709	AKT serine/threonine kinase 1(AKT1)	ENSG00000142208	207	Homo sapiens

Targets of baicalin gathering from PubChem

cid	chemicalid	chemicalname	genesymbol	genesymbol_human	Ensembl_id_human	ENTREZ_id_human	geneid	taxname	taxid	pmids
64982	C038044	baicalin	PKM	PKM	ENSG00000067225	5315	5315	Homo sapiens	9606	25388478
64982	C038044	baicalin	PPARG	PPARG	ENSG00000132170	5468	5468	Rattus norvegicus	10116	24312512
64982	C038044	baicalin	PRIMA1	PRIMA1	ENSG00000175785	145270	145270	Rattus norvegicus	10116	27019979
64982	C038044	baicalin	ABCC2	ABCC2	ENSG00000023839	1244	1244	Rattus	10114	15587936
64982	C038044	baicalin	ACHE	ACHE	ENSG00000087085	43	43	Rattus norvegicus	10116	27019979

64982	C038044	baicalin	AHR	AHR	ENSG00000106546	196	196	Mus musculus	10090	17869316
64982	C038044	baicalin	ALOX5	ALOX5	ENSG00000012779	240	240	Rattus norvegicus	10116	20139896
64982	C038044	baicalin	ATF2	ATF2	ENSG00000115966	1386	1386	Rattus norvegicus	10116	24312512
64982	C038044	baicalin	BAX	BAX	ENSG00000087088	581	581	Rattus norvegicus	10116	25423838
64982	C038044	baicalin	BCL2	BCL2	ENSG00000171791	596	596	Rattus norvegicus	10116	25423838
64982	C038044	baicalin	CASP3	CASP3	ENSG00000164305	836	836	Rattus norvegicus	10116	25423838
64982	C038044	baicalin	CCNA2	CCNA2	ENSG00000145386	890	890	Homo sapiens	9606	21457722
64982	C038044	baicalin	CCND1	CCND1	ENSG00000110092	595	595	Homo sapiens	9606	19160421
64982	C038044	baicalin	COL1A2	COL1A2	ENSG00000164692	1278	1278	Rattus norvegicus	10116	19474275
64982	C038044	baicalin	PTGS2	PTGS2	ENSG00000073756	5743	5743	Rattus norvegicus	10116	22019745
64982	C038044	baicalin	CYP1A1	CYP1A1	ENSG00000140465	1543	1543	Homo sapiens	9606	18451504
64982	C038044	baicalin	CYP1A2	CYP1A2	ENSG00000140505	1544	1544	Homo sapiens	9606	26204831
64982	C038044	baicalin	EGF	EGF	ENSG00000138798	1950	1950	Homo sapiens	9606	25625231
64982	C038044	baicalin	EGFR	EGFR	ENSG00000146648	1956	1956	Homo sapiens	9606	25625231
64982	C038044	baicalin	ELK1	ELK1	ENSG00000126767	2002	2002	Homo sapiens	9606	25625231
64982	C038044	baicalin	ERN1	ERN1	ENSG00000178607	2081	2081	Mus musculus	10090	29031535
64982	C038044	baicalin	FOXA2	FOXA2	ENSG00000125798	3170	3170	Homo sapiens	9606	32687838
64982	C038044	baicalin	GOT1	GOT1	ENSG00000120053	2805	2805	Rattus norvegicus	10116	15645217
64982	C038044	baicalin	GPT	GPT	ENSG00000167701	2875	2875	Rattus norvegicus	10116	15645217
64982	C038044	baicalin	HES1	HES1	ENSG00000114315	3280	3280	Homo sapiens	9606	19160421
64982	C038044	baicalin	HEY1	HEY1	ENSG00000164683	23462	23462	Homo sapiens	9606	19160421
64982	C038044	baicalin	HEY2	HEY2	ENSG00000135547	23493	23493	Homo sapiens	9606	19160421
64982	C038044	baicalin	HMOX1	HMOX1	ENSG00000100292	3162	3162	Rattus norvegicus	10116	16824337
64982	C038044	baicalin	HNF1A	HNF1A	ENSG00000135100	6927	6927	Homo sapiens	9606	32687838
64982	C038044	baicalin	HNF4A	HNF4A	ENSG00000101076	3172	3172	Homo sapiens	9606	32687838
64982	C038044	baicalin	IL1B	IL1B	ENSG00000125538	3553	3553	Mus musculus	10090	29031535

64982	C038044	baicalin	IL6	IL6	ENSG00000136244	3569	3569	Rattus norvegicus	10116	22019745
64982	C038044	baicalin	MYD88	MYD88	ENSG00000172936	4615	4615	Rattus norvegicus	10116	22019745
64982	C038044	baicalin	NFKBIA	NFKBIA	ENSG00000100906	4792	4792	Rattus norvegicus	10116	22019745
64982	C038044	baicalin	NLRP3	NLRP3	ENSG00000162711	114548	114548	Mus musculus	10090	29031535
64982	C038044	baicalin	NOS2	NOS2	ENSG00000007171	4843	4843	Rattus norvegicus	10116	22019745
64982	C038044	baicalin	NOTCH1	NOTCH1	ENSG00000148400	4851	4851	Homo sapiens	9606	19160421
64982	C038044	baicalin	RELA	RELA	ENSG00000173039	5970	5970	Rattus norvegicus	10116	22019745
64982	C038044	baicalin	TGFB1	TGFB1	ENSG00000105329	7040	7040	Rattus norvegicus	10116	19474275
64982	C038044	baicalin	TLR4	TLR4	ENSG00000136869	7099	7099	Rattus norvegicus	10116	22019745
64982	C038044	baicalin	TNF	TNF	ENSG00000232810	7124	7124	Rattus norvegicus	10116	22019745
64982	C038044	baicalin	TP53	TP53	ENSG00000141510	7157	7157	Homo sapiens	9606	21457722
64982	C038044	baicalin	TXNIP	TXNIP	ENSG00000265972	10628	10628	Mus musculus	10090	29031535
64982	C038044	baicalin	XDH	XDH	ENSG00000158125	7498	7498	Mus musculus	10090	34044073

Targets of baicalin gathering from ChEMBL

Target Pref. Name	genesymbol	ensembl_id	entrez_id	Organism	Confidence 70%	Confidence 80%	Confidence 90%	Activity Threshold
Adenosine kinase	ADK	ENSG00000156110	132	Homo sapiens	empty	active	active	6

Apoptosis regulator Bcl-2	BCL2	ENSG00000171791	596	Homo sapiens	empty	active	active	5
Indoleamine 2,3-dioxygenase	IDO1	ENSG00000131203	3620	Homo sapiens	active	active	active	6
GABA-A receptor; alpha-1/beta-2/gamma-2	GABRA1<br>GABRB2<br>GABRG2	ENSG00000022355<br>ENSG00000145864<br>ENSG00000113327	2554<br>2561<br>2566	Homo sapiens	active	active	active	5
Urokinase-type plasminogen activator	PLAU	ENSG00000122861	5328	Homo sapiens	empty	active	active	6
PI3-kinase p110-delta subunit	PIK3CD	ENSG00000171608	5293	Homo sapiens	active	active	active	6
Glutamate NMDA receptor; GRIN1/GRIN2B	GRIN1<br>GRIN2B	ENSG00000176884<br>ENSG00000273079	2902<br>2904	Homo sapiens	active	active	active	5
Amine oxidase, copper containing	AOC3	ENSG00000131471	8639	Homo sapiens	active	active	active	6
T-cell protein-tyrosine phosphatase	PTPN2	ENSG00000175354	5771	Homo sapiens	empty	active	active	6
Lysine-specific demethylase 5C	KDM5C	ENSG00000126012	8242	Homo sapiens	empty	empty	active	6

Prostanoid EP2 receptor	PTGER2	ENSG00000125384	5732	Homo sapiens	empty	empty	active	7
Proto-oncogene tyrosine-protein kinase ROS	ROS1	ENSG00000047936	6098	Homo sapiens	empty	empty	active	6.5
Serine-protein kinase ATM	ATM	ENSG00000149311	472	Homo sapiens	empty	empty	active	6.5

Targets of baicalin gathering from SwissTargetPrediction

Target	Common name	ensembl_id	entrez_id	Uniprot ID	ChEMBL ID	Target Class	Probability*	Known actives (3D/2D)
Aldose reductase (by homology)	AKR1B1	ENSG00000085662	231	P15121	CHEMBL1900	Enzyme	0.131434899	14 / 69
Adenosine A1 receptor (by homology)	ADORA1	ENSG00000163485	134	P30542	CHEMBL226	Family A G protein-coupled receptor	0.123138426	42 / 15
TNF-alpha	TNF	ENSG00000232810	7124	P01375	CHEMBL1825	Secreted protein	0.114839423	0 / 3
Interleukin-2	IL2	ENSG00000109471	3558	P60568	CHEMBL5880	Secreted protein	0.114839423	0 / 4
Xanthine dehydrogenase	XDH	ENSG00000158125	7498	P47989	CHEMBL1929	Oxidoreductase	0.106542926	0 / 19

Cyclooxygenase-2	PTGS2	ENSG00000073756	5743	P35354	CHEMBL230	Oxidoreductase	0.106542926	0 / 5
Ribosomal protein S6 kinase alpha 3	RPS6KA3	ENSG00000177189	6197	P51812	CHEMBL2345	Kinase	0.106542926	0 / 19
Epidermal growth factor receptor erbB1	EGFR	ENSG00000146648	1956	P00533	CHEMBL203	Kinase	0.106542926	0 / 21
Acetylcholinesterase	ACHE	ENSG00000087085	43	P22303	CHEMBL220	Hydrolase	0.106542926	0 / 28
Quinone reductase 2	NQO2	ENSG00000124588	4835	P16083	CHEMBL3959	Enzyme	0.106542926	0 / 1
Neuromedin-U receptor 2	NMUR2	ENSG00000132911	56923	Q9GZQ4	CHEMBL1075144	Family A G protein-coupled receptor	0.106542926	0 / 1
Alpha-2a adrenergic receptor	ADRA2A	ENSG00000150594	150	P08913	CHEMBL1867	Family A G protein-coupled receptor	0.106542926	0 / 1
Adrenergic receptor alpha-2	ADRA2C	ENSG00000184160	152	P18825	CHEMBL1916	Family A G protein-coupled receptor	0.106542926	0 / 2
NADPH oxidase 4	NOX4	ENSG00000086991	50507	Q9NPH5	CHEMBL1250375	Enzyme	0.106542926	0 / 7
Aldehyde dehydrogenase	ALDH2	ENSG00000111275	217	P05091	CHEMBL1935	Oxidoreductase	0.106542926	0 / 24

Deduplicated targets of baicalin

ensembl_id
ENSG00000147889
ENSG00000165507

ENSG00000178764
ENSG00000100030
ENSG00000076984
ENSG00000132906
ENSG00000142208
ENSG00000067225
ENSG00000132170
ENSG00000175785
ENSG00000023839
ENSG00000232810
ENSG00000106546
ENSG00000012779
ENSG00000115966
ENSG00000087088
ENSG00000171791
ENSG00000145386
ENSG00000110092
ENSG00000164692
ENSG00000140465
ENSG00000140505
ENSG00000138798
ENSG00000164305
ENSG00000126767
ENSG00000178607
ENSG00000125798
ENSG00000120053

ENSG00000167701
ENSG00000114315
ENSG00000164683
ENSG00000135547
ENSG00000100292
ENSG00000135100
ENSG00000101076
ENSG00000125538
ENSG00000136244
ENSG00000172936
ENSG00000100906
ENSG00000162711
ENSG00000007171
ENSG00000148400
ENSG00000173039
ENSG00000105329
ENSG00000136869
ENSG00000141510
ENSG00000265972
ENSG00000158125
ENSG00000156110
ENSG00000131203
ENSG00000022355
ENSG00000122861
ENSG00000171608
ENSG00000176884

ENSG00000131471
ENSG00000175354
ENSG00000126012
ENSG00000125384
ENSG00000047936
ENSG00000149311
ENSG00000145864
ENSG00000113327
ENSG00000085662
ENSG00000163485
ENSG00000146648
ENSG00000109471
ENSG00000087085
ENSG00000177189
ENSG00000073756
ENSG00000124588
ENSG00000132911
ENSG00000150594
ENSG00000184160
ENSG00000086991
ENSG00000111275

Supplementary Table S2: Network analysis results of baicalin

Network Analysis Results for targets of baicalin

name	AverageShortestPathLength	BetweennessCentrality	ClosenessCentrality	ClusteringCoefficient	Degree
RELA	2.0625	0.36635005	0.48484848	0.24166667	16
TP53	2.04166667	0.34916413	0.48979592	0.11428571	15
MAPK1	2.20833333	0.21595323	0.45283019	0.16483516	14
TNF	2.60416667	0.09610351	0.384	0.26666667	10
AKT1	2.5	0.24545128	0.4	0.08333333	9
IL6	2.41666667	0.03947357	0.4137931	0.46428571	8
IL1B	2.77083333	0.02122383	0.36090226	0.47619048	7
IL2	2.375	0.06283983	0.42105263	0.46666667	6
TLR4	2.83333333	0.04262707	0.35294118	0.53333333	6
NFKBIA	2.39583333	0.02804162	0.4173913	0.46666667	6
CCND1	2.52083333	0.01892942	0.39669421	0.3	5
EGFR	2.70833333	0.1214539	0.36923077	0.2	5
NOTCH1	3.35416667	0.12012411	0.29813665	0.33333333	4
CASP3	2.64583333	0.00757979	0.37795276	0.66666667	4
NOS2	2.60416667	0.03900709	0.384	0.5	4
BCL2	2.64583333	0.0114805	0.37795276	0.5	4
PTGS2	2.6875	0.04794622	0.37209302	0.16666667	4
ATF2	2.60416667	0.00143322	0.384	0.66666667	4
MYD88	2.91666667	8.87E-04	0.34285714	0.66666667	4
TGFB1	2.72916667	0.00150709	0.36641221	0.66666667	4
PPARG	2.52083333	0.0102668	0.39669421	0.5	4
CASP9	2.85416667	0.0052305	0.35036496	0.66666667	3
HNF1A	3.08333333	0.00382683	0.32432432	0	3
HEY1	4.29166667	4.43E-04	0.23300971	0.66666667	3

NLRP3	2.9375	0.04166667	0.34042553	0.33333333	3
AHR	2.875	0.15602837	0.34782609	0	2
CYP1A1	3.72916667	0.11968085	0.26815642	0	2
BAX	3	0	0.33333333	1	2
CDKN2A	2.95833333	0	0.33802817	1	2
IDO1	4.625	0.08156028	0.21621622	0	2
CYP1A2	5.5625	0.04166667	0.17977528	0	2
EGF	3.66666667	0	0.27272727	1	2
PTPN2	3.66666667	0	0.27272727	1	2
ELK1	3.16666667	0	0.31578947	1	2
RPS6KA3	3.16666667	0	0.31578947	1	2
GABRA1	1	0	1	1	2
GABRB2	1	0	1	1	2
GABRG2	1	0	1	1	2
HES1	4.3125	0	0.23188406	1	2
HEY2	4.3125	0	0.23188406	1	2
HNF4A	2.97916667	0.0025857	0.33566434	0	2
ACHE	1	0	1	0	1
PRIMA1	1	0	1	0	1
PIK3CD	3.47916667	0	0.28742515	0	1
FOXA2	3.47916667	0	0.28742515	0	1
ALDH2	1	0	1	0	1
AOC3	1	0	1	0	1
ALOX5	3.66666667	0	0.27272727	0	1
ATM	3.02083333	0	0.33103448	0	1
CCNA2	3.02083333	0	0.33103448	0	1

XDH	6.54166667	0	0.15286624	0	1
PLAU	3.6875	0	0.27118644	0	1
ERN1	3.58333333	0	0.27906977	0	1
GOT1	1	0	1	0	1
GPT	1	0	1	0	1
MAP2K7	3.58333333	0	0.27906977	0	1
TXNIP	3.91666667	0	0.25531915	0	1
NOX4	3.8125	0	0.26229508	0	1

Supplementary Table S3: Targets gathering of depression from different database

Targets of depression gathering from GeneCards

Gene Symbol	ensembl_id	ENTREZ_id
HTR2A	ENSG00000102468	3356
SLC6A4	ENSG00000108576	6532
TPH2	ENSG00000139287	121278
FKBP5	ENSG00000096060	2289
BDNF	ENSG00000176697	627
NOTCH3	ENSG00000074181	4854
COMT	ENSG00000093010	1312
HTR1A	ENSG00000178394	3350
MAOA	ENSG00000189221	4128
CRH	ENSG00000147571	1392
NR3C1	ENSG00000113580	2908
IL6	ENSG00000136244	3569
DRD2	ENSG00000149295	1813
TNF	ENSG00000232810	7124
PRL	ENSG00000172179	5617
APOE	ENSG00000130203	348
TPH1	ENSG00000129167	7166
HTR2C	ENSG00000147246	3358
MTHFR	ENSG00000177000	4524
SLC6A3	ENSG00000142319	6531
CRHR1	ENSG00000120088	1394
SLC6A2	ENSG00000103546	6530
CRP	ENSG00000132693	1401
MAPT	ENSG00000186868	4137
DCTN1	ENSG00000204843	1639
IL1B	ENSG00000125538	3553
GRIA1	ENSG00000155511	2890
POMC	ENSG00000115138	5443
CYP2D6	ENSG00000100197	1565
GNB3	ENSG00000111664	2784
CREB1	ENSG00000118260	1385
GRIN2B	ENSG00000273079	2904
CACNA1C	ENSG00000151067	100874369
OXT	ENSG00000101405	5020
FGFR1	ENSG00000077782	2260
HTR3A	ENSG00000166736	3359
ESR1	ENSG00000091831	2099
DRD4	ENSG00000069696	1815
TH	ENSG00000180176	7054
OXTR	ENSG00000180914	5021
RELN	ENSG00000189056	5649
XBP1	ENSG00000100219	7494

PCLO	ENSG00000186472	27445
NTRK2	ENSG00000148053	4915
GRM1	ENSG00000152822	2911
HTR1B	ENSG00000135312	3351
IL10	ENSG00000136634	3586
WFS1	ENSG00000109501	7466
POLG	ENSG00000140521	5428
INS	ENSG00000254647	3630
LEP	ENSG00000174697	3952
GRIA2	ENSG00000120251	2891
NPY	ENSG00000122585	4852
DRD3	ENSG00000151577	1814
NR3C2	ENSG00000151623	4306
HTR1D	ENSG00000179546	3352
DISC1	ENSG00000162946	27185
PER3	ENSG00000049246	8863
GAD1	ENSG00000128683	2571
GRIN2A	ENSG00000183454	2903
SNCA	ENSG00000145335	6622
OPRM1	ENSG00000112038	4988
GRIA3	ENSG00000125675	2892
CNR1	ENSG00000118432	1268
PRNP	ENSG00000171867	5621
FMR1	ENSG00000102081	2332
GRM7	ENSG00000196277	2917
GBA	ENSG00000177628	2629
NR4A2	ENSG00000153234	4929
CLOCK	ENSG00000134852	9575
MECP2	ENSG00000169057	4204
PICK1	ENSG00000100151	9463
DNMT1	ENSG00000130816	1786
GRID2	ENSG00000152208	2895
ARNTL	ENSG00000133794	406
NPAS2	ENSG00000170485	4862
GSK3B	ENSG00000082701	2932
DRD1	ENSG00000184845	1812
AKT1	ENSG00000142208	207
MIR132	ENSG00000267200	406921
IFNG	ENSG00000111537	3458
CYP2C19	ENSG00000165841	1557
NGF	ENSG00000134259	4803
MAOB	ENSG00000069535	4129
IL2	ENSG00000109471	3558
GDNF	ENSG00000168621	2668

TPO	ENSG00000115705	7173
DCANP1	ENSG00000251380	140947
HCRT	ENSG00000161610	3060
PIK3R1	ENSG00000145675	5295
CBLN1	ENSG00000102924	869
P2RX7	ENSG00000089041	5027
PDLIM5	ENSG00000163110	10611
CHRM2	ENSG00000181072	1129
GHRL	ENSG00000157017	51738
GNRH1	ENSG00000147437	2796
CYP2B6	ENSG00000197408	1555
GRM5	ENSG00000168959	2915
DLG1	ENSG00000075711	1739
CYP1A2	ENSG00000140505	1544
S100B	ENSG00000160307	6285
DTNBP1	ENSG00000047579	84062
SLC6A15	ENSG00000072041	55117
NR1D1	ENSG00000126368	9572
PTCH1	ENSG00000185920	5727
PRKN	ENSG00000185345	5071
BRAF	ENSG00000157764	673
LRRK2	ENSG00000188906	120892
KRAS	ENSG00000133703	3845
DAOA	ENSG00000182346	267012
HTT	ENSG00000197386	3064
PLA2G6	ENSG00000184381	8398
MAP2K1	ENSG00000169032	5604
DVL3	ENSG00000161202	1857
GNAS	ENSG00000087460	2778
GRM2	ENSG00000164082	2912
CREBBP	ENSG00000005339	1387
CYP3A4	ENSG00000160868	1576
RORA	ENSG00000069667	6095
SOD1	ENSG00000142168	6647
CYP2C9	ENSG00000138109	1559
NOS1	ENSG00000089250	4842
CRY2	ENSG00000121671	1408
DLG4	ENSG00000132535	1742
PER2	ENSG00000132326	8864
GAL	ENSG00000069482	51083
MTOR	ENSG00000198793	2475
ATP1A3	ENSG00000105409	478
GABRA1	ENSG00000022355	2554
PSEN1	ENSG00000080815	5663

KDM6A	ENSG00000147050	7403
SST	ENSG00000157005	6750
TOR1A	ENSG00000136827	1861
ACE	ENSG00000159640	1636
SLC18A2	ENSG00000165646	6571
GCH1	ENSG00000131979	2643
HLA-DQB1	ENSG00000179344	3119
COL2A1	ENSG00000139219	1280
ITIH3	ENSG00000162267	3699
COL1A1	ENSG00000108821	1277
GRIN1	ENSG00000176884	2902
GRIK2	ENSG00000164418	2898
SYNJ1	ENSG00000159082	8867
GRM3	ENSG00000198822	2913
CRY1	ENSG00000008405	1407
ALB	ENSG00000163631	213
PRODH	ENSG00000100033	5625
GLI2	ENSG00000074047	2736
C9orf72	ENSG00000147894	203228
FGF8	ENSG00000107831	2253
SHANK3	ENSG00000251322	85358
SHH	ENSG00000164690	6469
NRXN1	ENSG00000179915	9378
TACR1	ENSG00000115353	6869
SIRT1	ENSG00000096717	23411
COL11A2	ENSG00000204248	1302
SLC1A2	ENSG00000110436	6506
AR	ENSG00000169083	367
MAP2K2	ENSG00000126934	5605
COL11A1	ENSG00000060718	1301
ANK3	ENSG00000151150	288
ADCYAP1	ENSG00000141433	116
TP53	ENSG00000141510	7157
PTEN	ENSG00000171862	5728
ZNF804A	ENSG00000170396	91752
CHAT	ENSG00000070748	1103
DAO	ENSG00000110887	1610
DBH	ENSG00000123454	1621
TSPO	ENSG00000100300	706
PVALB	ENSG00000100362	5816
HLA-DRB1	ENSG00000196126	3123
TWIST2	ENSG00000233608	117581
IDUA	ENSG00000127415	3425
DPP4	ENSG00000197635	1803

NTF3	ENSG00000185652	4908
SYN2	ENSG00000157152	6854
KMT2A	ENSG00000118058	4297
FGFR2	ENSG00000066468	2263
MAPK1	ENSG00000100030	5594
IGF1	ENSG00000017427	3479
TBP	ENSG00000112592	6908
SGCE	ENSG00000127990	8910
ALX4	ENSG00000052850	60529
ATP2A2	ENSG00000174437	488
GRN	ENSG00000030582	2896
EP300	ENSG00000100393	2033
TRH	ENSG00000170893	7200
TAC1	ENSG00000006128	6863
WASHC5	ENSG00000164961	9897
CRHBP	ENSG00000145708	1393
GFAP	ENSG00000131095	2670
POU1F1	ENSG00000064835	5449
SNAP25	ENSG00000132639	6616
GABRB3	ENSG00000166206	2562
POLG2	ENSG00000256525	11232
FLNB	ENSG00000136068	2317
GPR50	ENSG00000102195	9248
ADAM10	ENSG00000137845	102
NRG1	ENSG00000157168	3084
SLC1A3	ENSG00000079215	6507
GABRD	ENSG00000187730	2563
GRIA4	ENSG00000152578	2893
VDR	ENSG00000111424	7421
KMT2D	ENSG00000167548	8085
AIP	ENSG00000110711	9049
ATXN2	ENSG00000204842	6311
IL2RB	ENSG00000100385	3560
RRM2B	ENSG00000048392	50484
TSC2	ENSG00000103197	7249
FADS1	ENSG00000149485	3992
CRHR2	ENSG00000106113	1395
PTPN11	ENSG00000179295	5781
PAX6	ENSG00000007372	5080
CISD2	ENSG00000145354	493856
PRKAR1A	ENSG00000108946	5573
OPN4	ENSG00000122375	94233
MEF2C	ENSG00000081189	4208
ABCB1	ENSG00000085563	5243

IMPA1	ENSG00000133731	3612
CNTNAP2	ENSG00000174469	26047
ZIC2	ENSG00000043355	7546
TGIF1	ENSG00000177426	7050
ARSA	ENSG00000100299	410
PDYN	ENSG00000101327	5173
FKBP4	ENSG00000004478	2288
DISP1	ENSG00000154309	84976
TONSL	ENSG00000160949	4796
INPPL1	ENSG00000165458	3636
MT-ND1	ENSG00000198888	4535
DLL1	ENSG00000198719	28514
CDH23	ENSG00000107736	64072
PON1	ENSG00000005421	5444
SORCS2	ENSG00000184985	57537
HP1BP3	ENSG00000127483	50809
SCN1A	ENSG00000144285	6323
MAN2B1	ENSG00000104774	4125
ADRA1A	ENSG00000120907	148
ZBTB20	ENSG00000181722	26137
CXCL8	ENSG00000169429	3576
VCP	ENSG00000165280	7415
CHRNA7	ENSG00000175344	1139
NFIX	ENSG00000008441	4784
MED13L	ENSG00000123066	23389
APP	ENSG00000142192	351
FGFR3	ENSG00000068078	2261
HSD11B1	ENSG00000117594	3290
SIX3	ENSG00000138083	6496
FAM20C	ENSG00000177706	56975
GPC6	ENSG00000183098	10082
TARDBP	ENSG00000120948	23435
XK	ENSG00000047597	7504
SLC25A4	ENSG00000151729	291
CRLF1	ENSG00000006016	9244
GAD2	ENSG00000136750	2572
TFAP2B	ENSG00000008196	7021
ADIPOQ	ENSG00000181092	9370
LRP8	ENSG00000157193	7804
STIL	ENSG00000123473	6491
PDGFB	ENSG00000100311	5155
USH2A	ENSG00000042781	7399
HESX1	ENSG00000163666	8820
GAP43	ENSG00000172020	2596

SMPD1	ENSG00000166311	6609
CDON	ENSG00000064309	50937
PDE4B	ENSG00000184588	5142
GRIK4	ENSG00000149403	2900
ALG9	ENSG00000086848	79796
CTNNB1	ENSG00000168036	1499
SERPINA3	ENSG00000196136	12
TTC9B	ENSG00000174521	148014
PNOC	ENSG00000168081	5368
GABRG2	ENSG00000113327	2566
HTR2B	ENSG00000135914	3357
TIMELESS	ENSG00000111602	8914
GAS1	ENSG00000180447	2619
FOS	ENSG00000170345	2353
DLG3	ENSG00000082458	1741
FOXH1	ENSG00000160973	8928
IFNA1	ENSG00000197919	3439
TP63	ENSG00000073282	8626
PREP	ENSG00000085377	5550
SYNE1	ENSG00000131018	23345
CLCF1	ENSG00000175505	23529
CHD7	ENSG00000171316	55636
MEN1	ENSG00000133895	4221
ELP4	ENSG00000109911	26610
TMEM106B	ENSG00000106460	54664
WDR26	ENSG00000162923	80232
HOMER1	ENSG00000152413	9456
TG	ENSG00000042832	7038
NODAL	ENSG00000156574	4838
SLC2A1	ENSG00000117394	6513
PARK7	ENSG00000116288	11315
BMPER	ENSG00000164619	168667
CD96	ENSG00000153283	10225
IDO1	ENSG00000131203	3620
KLHL7	ENSG00000122550	55975
CCK	ENSG00000187094	885
PDE11A	ENSG00000128655	50940
LMAN2L	ENSG00000114988	81562
MED12	ENSG00000184634	9968
VEGFA	ENSG00000112715	7422
VPS13C	ENSG00000129003	54832
HLA-DQA1	ENSG00000196735	3117
TCF4	ENSG00000196628	6925
BDNF-AS	ENSG00000245573	497258

ADH1C	ENSG00000248144	126
LPL	ENSG00000175445	4023
RGS4	ENSG00000117152	5999
PDGFRB	ENSG00000113721	5159
NEB	ENSG00000183091	4703
SP4	ENSG00000105866	6671
CTLA4	ENSG00000163599	1493
SETD5	ENSG00000168137	55209
GRIK1	ENSG00000171189	2897
TEF	ENSG00000167074	7008
GNAL	ENSG00000141404	2774
TDGF1	ENSG00000241186	6997
PALS1	ENSG00000072415	64398
WAC	ENSG00000095787	51322
PPARG	ENSG00000132170	5468
TRAPPC9	ENSG00000167632	83696
CSNK1E	ENSG00000213923	1454
ERBB4	ENSG00000178568	2066
KMT2B	ENSG00000272333	9757
NTNG2	ENSG00000196358	84628
AVP	ENSG00000101200	551
KCTD17	ENSG00000100379	79734
CHST3	ENSG00000122863	9469
FUS	ENSG00000089280	2521
CCDC22	ENSG00000101997	28952
PRKACA	ENSG00000072062	5566
GABRA2	ENSG00000151834	2555
GPC4	ENSG00000076716	2239
PAH	ENSG00000171759	5053
EIF2B5	ENSG00000145191	8893
TBX1	ENSG00000184058	6899
EHMT1	ENSG00000181090	79813
USP8	ENSG00000138592	9101
LINC02153	ENSG00000253199	286114
PSAP	ENSG00000197746	5660
HTR7	ENSG00000148680	3363
CC2D1A	ENSG00000132024	54862
TACR2	ENSG00000075073	6865
KCNQ3	ENSG00000184156	3786
HP	ENSG00000257017	3240
MAP2	ENSG00000078018	4133
BRCA2	ENSG00000139618	675
PCNT	ENSG00000160299	5116
GABRA3	ENSG00000011677	2556

SMIM30	ENSG00000214194	401397
LINC01108	ENSG00000226673	102216342
FZD2	ENSG00000180340	2535
HSPG2	ENSG00000142798	3339
GABRB1	ENSG00000163288	2560
PEX6	ENSG00000124587	5190
CLN6	ENSG00000128973	54982
CYP27A1	ENSG00000135929	1593
PIGN	ENSG00000197563	23556
SOX5	ENSG00000134532	6660
SYP	ENSG00000102003	6855
STAG2	ENSG00000101972	10735
CHMP2B	ENSG00000083937	25978
SCN9A	ENSG00000169432	6335
PTPN22	ENSG00000134242	26191
GNAI2	ENSG00000114353	2771
TRANK1	ENSG00000168016	9881
NPAS3	ENSG00000151322	64067
SLC25A24	ENSG00000085491	29957
HCRTR1	ENSG00000121764	3061
SNCAIP	ENSG00000064692	9627
NRGN	ENSG00000154146	4900
STUB1	ENSG00000103266	10273
DMD	ENSG00000198947	1756
TREM2	ENSG00000095970	54209
CHRNA4	ENSG00000101204	1137
NPC1	ENSG00000141458	4864
FLNA	ENSG00000196924	2316
SLC6A1	ENSG00000157103	6529
NBN	ENSG00000104320	4683
GABRA5	ENSG00000186297	2558
PTH1R	ENSG00000160801	5745
ADRA2A	ENSG00000150594	150
SMC1A	ENSG00000072501	8243
CASR	ENSG00000036828	846
DNAJC13	ENSG00000138246	23317
PHF21A	ENSG00000135365	51317
IRF6	ENSG00000117595	3664
FBN1	ENSG00000166147	2200
CDH11	ENSG00000140937	1009
GLB1	ENSG00000170266	2720
TBX4	ENSG00000121075	9496
AMY1A	ENSG00000237763	276
HLA-B	ENSG00000234745	3106

IL2RA	ENSG00000134460	3559
ARSB	ENSG00000113273	411
PIGT	ENSG00000124155	51604
SPECC1L	ENSG00000100014	23384
PRMT7	ENSG00000132600	54496
IL18	ENSG00000150782	3606
ASMT	ENSG00000196433	438
EDNRA	ENSG00000151617	1909
ABCC9	ENSG00000069431	10060
ACSL4	ENSG00000068366	2182
VGF	ENSG00000128564	7425
ATP7B	ENSG00000123191	540
CSTB	ENSG00000160213	1476
AMY1C	ENSG00000187733	278
AMY1B	ENSG00000174876	277
PYY	ENSG00000131096	5697
TMEM132D	ENSG00000151952	121256
CSMD1	ENSG00000183117	64478
CDKN1B	ENSG00000111276	1027
DDC	ENSG00000132437	1644
PDCD1	ENSG00000188389	5133
SHANK2	ENSG00000162105	22941
SYN1	ENSG00000008056	6853
SLC17A7	ENSG00000104888	57030
TACR3	ENSG00000169836	6870
DEAF1	ENSG00000177030	10522
GALT	ENSG00000213930	2592
PLOD1	ENSG00000083444	5351
GABRB2	ENSG00000145864	2561
SLC2A3	ENSG00000059804	6515
SIN3A	ENSG00000169375	25942
MARCKS	ENSG00000277443	4082
SLC26A1	ENSG00000145217	10861
PODXL	ENSG00000128567	5420
FAAH	ENSG00000117480	2166
IMPA2	ENSG00000141401	3613
MIR433	ENSG00000207569	574034
GABBR1	ENSG00000204681	2550
KCNH1	ENSG00000143473	3756
EMP1	ENSG00000134531	2012
RAF1	ENSG00000132155	5894
DRD5	ENSG00000169676	1816
NOS2	ENSG00000007171	4843
PMM2	ENSG00000140650	100130283

PINK1	ENSG00000158828	65018
PDE4D	ENSG00000113448	5144
MIR29C	ENSG00000284214	407026
PREPL	ENSG00000138078	9581
CACNA1A	ENSG00000141837	773
IL1A	ENSG00000115008	3552
CCL2	ENSG00000108691	6347
MAF	ENSG00000178573	4094
ESR2	ENSG00000140009	2100
ELN	ENSG00000049540	2006
DNAJC6	ENSG00000116675	9829
INPP1	ENSG00000151689	3628
LBR	ENSG00000143815	3930
AVPR1A	ENSG00000166148	552
GATA1	ENSG00000102145	2623
MIR137	ENSG00000284202	406928
ATRX	ENSG00000085224	546
STX1A	ENSG00000106089	6804
GHR	ENSG00000112964	2690
MYT1L	ENSG00000186487	23040
NF1	ENSG00000196712	4763
IL4	ENSG00000113520	3565
AHDC1	ENSG00000126705	27245
NDUFV2	ENSG00000178127	4729
ERF	ENSG00000105722	2077
NAGA	ENSG00000198951	4668
IL1RN	ENSG00000136689	3557
CLCN1	ENSG00000188037	1180
PRKCG	ENSG00000126583	5582
PMS2	ENSG00000122512	5395
RERE	ENSG00000142599	473
SUFU	ENSG00000107882	51684
CHI3L1	ENSG00000133048	1116
SCN2A	ENSG00000136531	6326
PTGS2	ENSG00000073756	5743
NCAN	ENSG00000130287	1463
AVPR1B	ENSG00000198049	553
RYR1	ENSG00000196218	6261
HDAC6	ENSG00000094631	10013
HMCN1	ENSG00000143341	83872
ZEB2	ENSG00000169554	9839
TFAP2A	ENSG00000137203	7020
CSF1R	ENSG00000182578	1436
SQSTM1	ENSG00000161011	8878

TTR	ENSG00000118271	7276
LGI1	ENSG00000108231	9211
STXBP1	ENSG00000136854	6812
AP2S1	ENSG00000042753	1175
SETD1A	ENSG00000099381	9739
METTL13	ENSG00000010165	51603
PIGA	ENSG00000165195	5277
GADL1	ENSG00000144644	339896
KMT2E	ENSG00000005483	55904
TNFRSF1A	ENSG00000067182	7132
IQSEC2	ENSG00000124313	23096
PLCG1	ENSG00000124181	5335
ANKRD11	ENSG00000167522	29123
TAF1	ENSG00000147133	6872
CACNB2	ENSG00000165995	783
ADH1B	ENSG00000196616	125
TRPV4	ENSG00000111199	59341
TRPV1	ENSG00000196689	7442
PTCHD1	ENSG00000165186	139411
DUSP6	ENSG00000139318	1848
MIR24-1	ENSG00000284459	407012
C19orf12	ENSG00000131943	83636
KAT5	ENSG00000172977	10524
DPYSL2	ENSG00000092964	1808
GJA1	ENSG00000152661	2697
CCBE1	ENSG00000183287	147372
SLC26A2	ENSG00000155850	1836
PKDCC	ENSG00000162878	91461
TRRAP	ENSG00000196367	8295
EDARADD	ENSG00000186197	128178
CHRNB2	ENSG00000160716	1141
CNR2	ENSG00000188822	1269
NOS3	ENSG00000164867	4846
TK2	ENSG00000166548	7084
TLR4	ENSG00000136869	7099
SETBP1	ENSG00000152217	26040
BMP2	ENSG00000125845	650
CTU2	ENSG00000174177	348180
FAT4	ENSG00000196159	79633
CALB1	ENSG00000104327	793
GLUD2	ENSG00000182890	2747
TCF20	ENSG00000100207	6942
ATP13A2	ENSG00000159363	23400
CAT	ENSG00000121691	847

OTX2	ENSG00000165588	5015
AARS2	ENSG00000124608	57505
TGFB1	ENSG00000105329	7040
SMC3	ENSG00000108055	9126
DLK1	ENSG00000185559	8788
GRIP1	ENSG00000155974	23426
ADRB2	ENSG00000169252	154
HRAS	ENSG00000174775	3265
GH1	ENSG00000259384	2688
COQ2	ENSG00000173085	27235
UCHL1	ENSG00000154277	7345
MIR30E	ENSG00000198974	407034
NR1H3	ENSG00000025434	10062
SLC12A2	ENSG00000064651	6558
IL1R1	ENSG00000115594	3554
FMO3	ENSG00000007933	2328
TCF7L2	ENSG00000148737	6934
EDA	ENSG00000158813	1896
PROKR2	ENSG00000101292	128674
SLC7A6OS	ENSG00000103061	84138
FH	ENSG00000091483	2271
HSP90B1	ENSG00000166598	7184
EDN1	ENSG00000078401	1906
MMP1	ENSG00000196611	4312
SCN1A-AS1	ENSG00000236107	101929680
ACHE	ENSG00000087085	43
GNRHR	ENSG00000109163	2798
GLUL	ENSG00000135821	2752
DNMT3B	ENSG00000088305	1789
HTR6	ENSG00000158748	3362
SOX9	ENSG00000125398	6662
MBD5	ENSG00000204406	55777
GLI3	ENSG00000106571	2737
PPARGC1A	ENSG00000109819	10891
ADORA1	ENSG00000163485	134
PIEZO2	ENSG00000154864	63895
MAD1L1	ENSG00000002822	8379
GRHL3	ENSG00000158055	57822
MMP9	ENSG00000100985	4318
CALCA	ENSG00000110680	796
CLIP1	ENSG00000130779	6249
IGFBP3	ENSG00000146674	3486
BCR	ENSG00000186716	613
RAI1	ENSG00000108557	10743

PIGL	ENSG00000108474	9487
PIK3CA	ENSG00000121879	5290
TUBB	ENSG00000196230	203068
HLA-A	ENSG00000206503	3105
DVL1	ENSG00000107404	1855
EDAR	ENSG00000135960	10913
TF	ENSG00000091513	7018
MADD	ENSG00000110514	8567
TREX1	ENSG00000213689	11277
NGFR	ENSG00000064300	4804
ERCC1	ENSG00000012061	2067
SLC1A1	ENSG00000106688	6505
NCAM1	ENSG00000149294	4684
ALDH2	ENSG00000111275	217
SLC18A3	ENSG00000187714	6572
ATRIP	ENSG00000164053	111822955
SLC1A4	ENSG00000115902	6509
PPP1R1B	ENSG00000131771	84152
PIK3C2A	ENSG00000011405	5286
GLA	ENSG00000102393	2717
PCDH19	ENSG00000165194	57526
KDM6B	ENSG00000132510	23135
CDKN1A	ENSG00000124762	1026
ANTXR1	ENSG00000169604	84168
WNT5A	ENSG00000114251	7474
MEG3	ENSG00000214548	55384
KISS1R	ENSG00000116014	84634
COL7A1	ENSG00000114270	1294
TAC3	ENSG00000166863	6866
DGKH	ENSG00000102780	160851
SLC45A1	ENSG00000162426	50651
ASXL3	ENSG00000141431	80816
JAK2	ENSG00000096968	3717
SCN1B	ENSG00000105711	6324
PROK2	ENSG00000163421	60675
IQSEC1	ENSG00000144711	9922
CNTF	ENSG00000242689	1270
INPP5E	ENSG00000148384	56623
CASD1	ENSG00000127995	64921
PANK2	ENSG00000125779	80025
FGF23	ENSG00000118972	8074
NALCN	ENSG00000102452	259232
EGF	ENSG00000138798	1950
PRICKLE2	ENSG00000163637	166336

RUNX2	ENSG00000124813	860
KCNT1	ENSG00000107147	57582
USH1C	ENSG00000006611	10083
SELP	ENSG00000174175	6403
CCND2	ENSG00000118971	894
GPT	ENSG00000167701	2875
ATXN10	ENSG00000130638	25814
ETS1	ENSG00000134954	2113
TBK1	ENSG00000183735	29110
KISS1	ENSG00000170498	3814
POR	ENSG00000127948	5447
RAB39B	ENSG00000155961	116442
BUB1B	ENSG00000156970	701
BRAT1	ENSG00000106009	221927
IL17A	ENSG00000112115	3605
HDAC4	ENSG00000068024	9759
DPYSL5	ENSG00000157851	56896
ROR2	ENSG00000169071	4920
NSUN2	ENSG00000037474	54888
MT-CO1	ENSG00000198804	4512
OPRD1	ENSG00000116329	4985
SPTBN1	ENSG00000115306	6711
GIGYF2	ENSG00000204120	26058
CHD2	ENSG00000173575	1106
NEK1	ENSG00000137601	4750
MSTO1	ENSG00000125459	55154
PIK3CD	ENSG00000171608	5293
RETN	ENSG00000104918	56729
MTNR1B	ENSG00000134640	4544
ITPR1	ENSG00000150995	3708
LIMK1	ENSG00000106683	3984
PTH	ENSG00000152266	5741
APOB	ENSG00000084674	338
TNFAIP3	ENSG00000118503	7128
PROP1	ENSG00000175325	5626
ARC	ENSG00000198576	23237
TENM4	ENSG00000149256	26011
DPH1	ENSG00000108963	1801
SPEN	ENSG00000065526	23013
HTR3B	ENSG00000149305	9177
KCNMA1	ENSG00000156113	3778
MYO7A	ENSG00000137474	4647
MIRLET7D	ENSG00000199133	406886
RPS6KA3	ENSG00000177189	6197

TSHB	ENSG00000134200	7252
SOD2	ENSG00000112096	6648
NSMF	ENSG00000165802	26012
AGER	ENSG00000204305	177
AGTR2	ENSG00000180772	186
BCL2	ENSG00000171791	596
ADA	ENSG00000196839	100
SOST	ENSG00000167941	50964
PIGY	ENSG00000255072	84992
CACNG2	ENSG00000166862	10369
VPS13A	ENSG00000197969	23230
ICAM1	ENSG00000090339	3383
FTO	ENSG00000140718	79068
SMARCA4	ENSG00000127616	6597
ANKK1	ENSG00000170209	255239
SYNGAP1	ENSG00000197283	8831
RNASEH2C	ENSG00000172922	84153
KCNN3	ENSG00000143603	3782
SOS1	ENSG00000115904	6654
SMARCA2	ENSG00000080503	6595
STX16	ENSG00000124222	8675
FOXC1	ENSG00000054598	2296
SERPINE1	ENSG00000106366	5054
HCN1	ENSG00000164588	348980
DEPDC5	ENSG00000100150	9681
ADNP	ENSG00000101126	23394
EPM2A	ENSG00000112425	7957
MYOD1	ENSG00000129152	4654
DYRK1A	ENSG00000157540	1859
NR1H4	ENSG00000012504	9971
PGAP1	ENSG00000197121	80055
HSD17B4	ENSG00000133835	3295
BMPR1A	ENSG00000107779	657
CASP3	ENSG00000164305	836
NDEL1	ENSG00000166579	81565
DYNC2H1	ENSG00000187240	79659
KCNJ2	ENSG00000123700	3759
NPPB	ENSG00000120937	4879
ALG3	ENSG00000214160	10195
ADORA2A	ENSG00000128271	135
ARID2	ENSG00000189079	196528
KAT6B	ENSG00000156650	23522
HMBS	ENSG00000256269	3145
RMRP	ENSG00000269900	6023

PPP2R2B	ENSG00000156475	5521
INSR	ENSG00000171105	3643
TMEM94	ENSG00000177728	9772
MSH2	ENSG00000095002	4436
FGF20	ENSG00000078579	26281
FBXO31	ENSG00000103264	79791
ARID1B	ENSG00000049618	57492
CDKN1C	ENSG00000129757	1028
GRIK5	ENSG00000105737	2901
F2	ENSG00000180210	2147
MT-CO3	ENSG00000198938	4514
FGF2	ENSG00000138685	2247
MIR185	ENSG00000208023	406961
MDH1	ENSG00000014641	4190
SLC18A1	ENSG00000036565	6570
WDR35	ENSG00000118965	57539
GABRA4	ENSG00000109158	2557
RET	ENSG00000165731	5979
SLCO1C1	ENSG00000139155	53919
PURA	ENSG00000185129	5813
RSPRY1	ENSG00000159579	89970
PF4	ENSG00000163737	5196
MUSK	ENSG00000030304	4593
VPS35	ENSG00000069329	55737
ZBTB24	ENSG00000112365	9841
RAPSN	ENSG00000165917	5913
IL6R	ENSG00000160712	3570
NEXMIF	ENSG00000050030	340533
DPP10	ENSG00000175497	57628
CACNA1G	ENSG00000006283	8913
NCS1	ENSG00000107130	23413
PPOX	ENSG00000143224	5498
MILR1	ENSG00000271605	284021
TGFBR2	ENSG00000163513	7048
CACNA1H	ENSG00000196557	8912
RPS20	ENSG00000008988	6224
FOXP2	ENSG00000128573	93986
MLH1	ENSG00000076242	4292
FGF17	ENSG00000158815	8822
SPRY4	ENSG00000187678	81848
HS6ST1	ENSG00000136720	9394
WDR11	ENSG00000120008	55717
EXTL3	ENSG00000012232	2137
GRIN2C	ENSG00000161509	2905

PDZD7	ENSG00000186862	79955
PCDH15	ENSG00000150275	65217
PSAT1	ENSG00000135069	29968
EXT2	ENSG00000151348	2132
CBS	ENSG00000160200	875
XYLT1	ENSG00000103489	64131
ATXN1	ENSG00000124788	6310
ADGRV1	ENSG00000164199	84059
FANCL	ENSG00000115392	55120
HDAC8	ENSG00000147099	55869
GMNN	ENSG00000112312	51053
SLC35C1	ENSG00000181830	55343
CDKN2B	ENSG00000147883	1030
PDE5A	ENSG00000138735	8654
MT-ND5	ENSG00000198786	4540
RSRC1	ENSG00000174891	51319
OPA1	ENSG00000198836	4976
ATXN3	ENSG00000066427	4287
SPTAN1	ENSG00000197694	6709
HEPACAM	ENSG00000165478	220296
CCNF	ENSG00000162063	899
GRIK3	ENSG00000163873	2899
IRAK1	ENSG00000184216	3654
NRAS	ENSG00000213281	4893
MIR133B	ENSG00000199080	442890
MTOR-AS1	ENSG00000225602	100873935
CIT	ENSG00000122966	11113
ADCY2	ENSG00000078295	108
MDGA1	ENSG00000112139	266727
PHGDH	ENSG00000092621	26227
BUD23	ENSG00000071462	114049
ASXL1	ENSG00000171456	171023
VIP	ENSG00000146469	7432
MT-ND4	ENSG00000198886	4538
JPH3	ENSG00000154118	57338
DGUOK	ENSG00000114956	1716
YWHAH	ENSG00000128245	7533
APOD	ENSG00000189058	347
ERVW-1	ENSG00000242950	30816
CYP19A1	ENSG00000137869	1588
CHCHD10	ENSG00000250479	400916
PITX1	ENSG00000069011	5307
DOK7	ENSG00000175920	285489
KIAA0586	ENSG00000100578	9786

MAN1B1	ENSG00000177239	11253
NSD1	ENSG00000165671	64324
PPIEL	ENSG00000243970	728448
HUWE1	ENSG00000086758	10075
FIG4	ENSG00000112367	9896
RNPC3	ENSG00000185946	55599
HNRNPA1	ENSG00000135486	3178
PRKCE	ENSG00000171132	5581
CLRN1	ENSG00000163646	7401
KCNQ2	ENSG00000075043	3785
TMEM216	ENSG00000187049	51259
VCAM1	ENSG00000162692	7412
ACTG1	ENSG00000184009	71
EIF4H	ENSG00000106682	7458
CLIP2	ENSG00000106665	7461
BAZ1B	ENSG00000009954	9031
KIAA1109	ENSG00000138688	84162
CC2D2A	ENSG00000048342	57545
RPS24	ENSG00000138326	6229
EIF4G1	ENSG00000114867	1981
MLXIPL	ENSG00000009950	51085
DPYD	ENSG00000188641	1806
HMGCR	ENSG00000113161	3156
VWF	ENSG00000110799	7450
NKX2-5	ENSG00000183072	1482
PER1	ENSG00000179094	5187
FAN1	ENSG00000198690	22909
APC	ENSG00000134982	324
MIR144	ENSG00000283819	406936
USP9X	ENSG00000124486	8239
MLH3	ENSG00000119684	27030
CEP57	ENSG00000166037	9702
HNF1A	ENSG00000135100	6927
PTS	ENSG00000150787	5805
S100A10	ENSG00000197747	6281
B3GLCT	ENSG00000187676	145173
B3GALT6	ENSG00000176022	126792
BMP4	ENSG00000125378	652
TSC1	ENSG00000165699	7248
PIGG	ENSG00000174227	54872
FOXG1	ENSG00000176165	2290
CNOT3	ENSG00000088038	4849
METTL23	ENSG00000181038	124512
SMARCB1	ENSG00000099956	6598

TRIP11	ENSG00000100815	9321
SLC12A6	ENSG00000140199	9990
REN	ENSG00000143839	5972
MYCN	ENSG00000134323	4613
GTF2I	ENSG00000263001	2969
OBSL1	ENSG00000124006	23363
PRDM16	ENSG00000142611	63976
WHRN	ENSG00000095397	25861
SNCB	ENSG00000074317	6620
CHRNA2	ENSG00000120903	1135
ARL3	ENSG00000138175	403
UBQLN2	ENSG00000188021	29978
SLC5A5	ENSG00000105641	6528
EIF2B2	ENSG00000119718	8892
CYP2E1	ENSG00000130649	1571
RTN4R	ENSG00000040608	65078
AGT	ENSG00000135744	183
COL1A2	ENSG00000164692	1278
PITX2	ENSG00000164093	5308
VRK2	ENSG00000028116	7444
TBX2	ENSG00000121068	6909
ARSG	ENSG00000141337	22901
GLIS3	ENSG00000107249	169792
C4A	ENSG00000244731	720
SLC38A8	ENSG00000166558	146167
PIP4K2A	ENSG00000150867	5305
MIR326	ENSG00000199090	442900
RAD21	ENSG00000164754	5885
CCL5	ENSG00000271503	6352
EDC3	ENSG00000179151	80153
POGZ	ENSG00000143442	23126
ETFDH	ENSG00000171503	2110
GUSB	ENSG00000169919	2990
EEF1A2	ENSG00000101210	1917
SPECC1L-ADORA2A	ENSG00000258555	101730217
TWNK	ENSG00000107815	56652
PRICKLE2-AS1	ENSG00000241111	100652759
STAG3	ENSG00000066923	10734
DICER1	ENSG00000100697	23405
CRKL	ENSG00000099942	1399
ACTA1	ENSG00000143632	58
CST3	ENSG00000101439	1471
FA2H	ENSG00000103089	79152
ARSL	ENSG00000157399	415

TRHR	ENSG00000174417	7201
PPBP	ENSG00000163736	5473
RTL1	ENSG00000254656	388015
ADK	ENSG00000156110	132
SUMF1	ENSG00000144455	285362
ADCYAP1R1	ENSG00000078549	117
KCNA1	ENSG00000111262	3736
PAX3	ENSG00000135903	5077
FAS	ENSG00000026103	355
GRK3	ENSG00000100077	157
HARS1	ENSG00000170445	3035
UBE3B	ENSG00000151148	89910
TRAF6	ENSG00000175104	7189
ARX	ENSG00000004848	170302
GLT8D1	ENSG00000016864	55830
JARID2	ENSG00000008083	3720
SIM1	ENSG00000112246	6492
DHCR7	ENSG00000172893	1717
GSR	ENSG00000104687	2936
MRE11	ENSG00000020922	4361
HNF1B	ENSG00000275410	6928
NCF1	ENSG00000158517	653361
DNAJC30	ENSG00000176410	84277
MAG	ENSG00000105695	4099
FDFT1	ENSG00000079459	2222
DAOA-AS1	ENSG00000232307	282706
LMNB1	ENSG00000113368	4001
MIR140	ENSG00000208017	406932
SNAP29	ENSG00000099940	9342
ATP6V1B2	ENSG00000147416	526
HMOX1	ENSG00000100292	3162
DMPK	ENSG00000104936	1760
PFN1	ENSG00000108518	5216
GRM8	ENSG00000179603	2918
GATA4	ENSG00000136574	2626
SPAST	ENSG00000021574	6683
GPC3	ENSG00000147257	2719
RPS19	ENSG00000105372	6223
CSPP1	ENSG00000104218	79848
KMT2C	ENSG00000055609	58508
GLE1	ENSG00000119392	2733
IGFBP1	ENSG00000146678	3484
GHRH	ENSG00000118702	2691
GNA11	ENSG00000088256	2767

GCG	ENSG00000115263	2641
BGLAP	ENSG00000242252	632
ADM	ENSG00000148926	133
CAMK2G	ENSG00000148660	818
IGF2	ENSG00000167244	3481
CUL7	ENSG00000044090	9820
GPR39	ENSG00000183840	2863
HSPA1A	ENSG00000204389	3303
STAT4	ENSG00000138378	6775
TSHR	ENSG00000165409	7253
RPS23	ENSG00000186468	6228
MT-ATP6	ENSG00000198899	4508
IL5	ENSG00000113525	3567
TRIP12	ENSG00000153827	9320
KDM4B	ENSG00000127663	23030
RREB1	ENSG00000124782	6239
MTR	ENSG00000116984	4548
PLP1	ENSG00000123560	5354
CSGALNACT1	ENSG00000147408	55790
AGTR1	ENSG00000144891	185
NHLRC1	ENSG00000187566	378884
B3GAT3	ENSG00000149541	26229
LTA	ENSG00000226979	4049
TMEM67	ENSG00000164953	91147
FOLH1	ENSG00000086205	2346
DGKI	ENSG00000157680	9162
AKT3	ENSG00000117020	10000
MAPRE2	ENSG00000166974	10982
CAPN3	ENSG00000092529	825
DNA2	ENSG00000138346	1763
GPR101	ENSG00000165370	83550
NDUFS4	ENSG00000164258	4724
FTSJ1	ENSG00000068438	24140
CD40LG	ENSG00000102245	959
LRP2	ENSG00000081479	4036
IL13	ENSG00000169194	3596
ARVCF	ENSG00000099889	421
SHBG	ENSG00000129214	6462
ZMIZ1	ENSG00000108175	57178
CACNG5	ENSG00000075429	27091
ULK4	ENSG00000168038	54986
ADGRA3	ENSG00000152990	166647
NTRK3	ENSG00000140538	4916
RFC2	ENSG00000049541	5982

GTF2IRD1	ENSG00000006704	9569
TBL2	ENSG00000106638	26608
FKBP6	ENSG00000077800	8468
BCL7B	ENSG00000106635	9275
GTF2IRD2	ENSG00000196275	84163
VPS37D	ENSG00000176428	155382
METTL27	ENSG00000165171	155368
TMEM270	ENSG00000175877	135886
CDKL5	ENSG00000008086	6792
PRKCZ	ENSG00000067606	5590
GP1BB	ENSG00000203618	2812
DUOX2	ENSG00000140279	50506
DUOXA2	ENSG00000140274	405753
WASF1	ENSG00000112290	8936
SCN8A	ENSG00000196876	6334
PIGP	ENSG00000185808	51227
IRS2	ENSG00000185950	8660
GPX1	ENSG00000233276	2876
ENPP1	ENSG00000197594	5167
AQP4	ENSG00000171885	361
CHD8	ENSG00000100888	57680
SOX11	ENSG00000176887	6664
MOG	ENSG00000204655	4340
SEMA3E	ENSG00000170381	9723
JAG1	ENSG00000101384	182
IRS1	ENSG00000169047	3667
EDA2R	ENSG00000131080	60401
OPTN	ENSG00000123240	10133
WASHC4	ENSG00000136051	23325
IGF1R	ENSG00000140443	3480
EZR	ENSG00000092820	7430
MIF	ENSG00000240972	4282
ACVRL1	ENSG00000139567	94
RORB	ENSG00000198963	6096
BTBD16	ENSG00000138152	118663
AFG1L	ENSG00000135537	246269
GNPTAB	ENSG00000111670	79158
DLL3	ENSG00000090932	10683
PLCB1	ENSG00000182621	23236
SKI	ENSG00000157933	6497
ESPN	ENSG00000187017	83715
CLCN4	ENSG00000073464	1183
RPL11	ENSG00000142676	6135
EFEMP2	ENSG00000172638	30008

ALAD	ENSG00000148218	210
ACAN	ENSG00000157766	176
IL1RAPL1	ENSG00000169306	11141
NXN	ENSG00000167693	64359
NIPBL	ENSG00000164190	25836
PIGQ	ENSG00000007541	9091
UNC13A	ENSG00000130477	23025
NPC2	ENSG00000119655	10577
ZFPM2	ENSG00000169946	23414
ADRB1	ENSG00000043591	153
DOCK3	ENSG00000088538	1795
CDK5	ENSG00000164885	1020
ATP1A2	ENSG00000018625	477
CLIC2	ENSG00000155962	1193
UFD1	ENSG00000070010	7353
DYNC2I1	ENSG00000126870	55112
HIRA	ENSG00000100084	7290
LHX4	ENSG00000121454	89884
NEFL	ENSG00000277586	4747
RPS27A	ENSG00000143947	6233
SORT1	ENSG00000134243	6272
MT-ND6	ENSG00000198695	4541
SLC17A5	ENSG00000119899	26503
ITGAM	ENSG00000169896	3684
EPCAM	ENSG00000119888	4072
UBE2A	ENSG00000077721	7319
CYP21A2	ENSG00000231852	1589
FRRS1L	ENSG00000260230	23732
LINS1	ENSG00000140471	55180
ST3GAL3	ENSG00000126091	6487
NEFH	ENSG00000100285	4744
JMJD1C	ENSG00000171988	221037
MIR206	ENSG00000207604	406989
M6PR	ENSG00000003056	4074
NLGN1	ENSG00000169760	22871
MRAS	ENSG00000158186	22808
CNKSR2	ENSG00000149970	22866
TWIST1	ENSG00000122691	7291
CFAP410	ENSG00000160226	755
CBL	ENSG00000110395	867
HNMT	ENSG00000150540	3176
MC2R	ENSG00000185231	4158
CP	ENSG00000047457	1356
RPL5	ENSG00000122406	6125

CARS1	ENSG00000110619	833
HTRA2	ENSG00000115317	27429
RIPK4	ENSG00000183421	54101
F3	ENSG00000117525	2152
HBB	ENSG00000244734	3043
PAK3	ENSG00000077264	5063
PSMD9	ENSG00000110801	5715
TMEM107	ENSG00000179029	84314
MST1	ENSG00000173531	4485
COL6A2	ENSG00000142173	1292
COL6A1	ENSG00000142156	1291
LIFR	ENSG00000113594	3977
EIF2AK3	ENSG00000172071	9451
MANBA	ENSG00000109323	4126
NAA10	ENSG00000102030	8260
ZSWIM6	ENSG00000130449	57688
LMBR1	ENSG00000105983	64327
PDE4A	ENSG00000065989	5141
ATXN7	ENSG00000163635	6314
CYCS	ENSG00000172115	54205
DNAJC5	ENSG00000101152	80331
MMP2	ENSG00000087245	4313
P4HTM	ENSG00000178467	54681
RPGRIP1L	ENSG00000103494	23322
RBFOX1	ENSG00000078328	54715
AMMECR1	ENSG00000101935	9949
IFT81	ENSG00000122970	28981
KCNK2	ENSG00000082482	3776
FKRP	ENSG00000181027	79147
SLC6A9	ENSG00000196517	6536
OCRL	ENSG00000122126	4952
PEX13	ENSG00000162928	5194
LIG4	ENSG00000174405	3981
HTR3D	ENSG00000186090	200909
KIF1A	ENSG00000130294	547
JMJD8	ENSG00000161999	339123
LFNG	ENSG00000106003	3955
LCN2	ENSG00000148346	3934
CEP85L	ENSG00000111860	387119
MSH6	ENSG00000116062	2956
AFG3L2	ENSG00000141385	10939
SRPX2	ENSG00000102359	27286
ARMC5	ENSG00000140691	79798
MIR183	ENSG00000207691	406959

ARTN	ENSG00000117407	9048
APOA1	ENSG00000118137	335
EZH2	ENSG00000106462	2146
HCFC1	ENSG00000172534	3054
KCNB1	ENSG00000158445	3745
L1CAM	ENSG00000198910	3897
TNFRSF1B	ENSG00000028137	7133
B3GALNT2	ENSG00000162885	148789
ADAMTS2	ENSG00000087116	9509
MCHR2-AS1	ENSG00000229315	728012
CHEK2	ENSG00000183765	11200
KCNH5	ENSG00000140015	27133
ACTB	ENSG00000075624	60
HYAL1	ENSG00000114378	3373
TRIM8	ENSG00000171206	81603
CANT1	ENSG00000171302	124583
ABCB11	ENSG00000073734	8647
EYA1	ENSG00000104313	2138
DIO2	ENSG00000211448	1734
ABL1	ENSG00000097007	25
RECQL4	ENSG00000160957	9401
HELLS	ENSG00000119969	3070
CD4	ENSG00000010610	920
EFNB1	ENSG00000090776	1947
TTF2	ENSG00000116830	8458
MED25	ENSG00000104973	81857
CAMKMT	ENSG00000143919	79823
SPOP	ENSG00000121067	8405
USH1G	ENSG00000182040	124590
PTK2B	ENSG00000120899	2185
SMARCE1	ENSG00000073584	6605
DPF2	ENSG00000133884	5977
ABCG5	ENSG00000138075	64240
BICC1	ENSG00000122870	80114
SLC20A2	ENSG00000168575	6575
SLC5A7	ENSG00000115665	60482
CPOX	ENSG00000080819	1371
PEPD	ENSG00000124299	5184
HTR4	ENSG00000164270	3360
CTSD	ENSG00000117984	1509
FCGR3B	ENSG00000162747	2215
MIR29A	ENSG00000284032	407021
CYP17A1	ENSG00000148795	1586
IL18R1	ENSG00000115604	8809

RTN4	ENSG00000115310	57142
BRD4	ENSG00000141867	23476
SATB2	ENSG00000119042	23314
DPM1	ENSG00000000419	8813
CRIPT	ENSG00000119878	9419
TBCE	ENSG00000284770	6905
OGG1	ENSG00000114026	4968
SCARF2	ENSG00000244486	91179
VAPB	ENSG00000124164	9217
MIR146A	ENSG00000283733	406938
DUSP19	ENSG00000162999	142679
SEC24C	ENSG00000176986	9632
SMARCD1	ENSG00000066117	6602
HSPA9	ENSG00000113013	3313
SEMA3A	ENSG00000075213	10371
PLA2G4A	ENSG00000116711	5321
RBP4	ENSG00000138207	5950
DYNC2I2	ENSG00000119333	89891
OPRK1	ENSG00000082556	4986
FOXL2	ENSG00000183770	668
CNP	ENSG00000173786	1267
WWOX	ENSG00000186153	51741
CIB2	ENSG00000136425	10518
ANG	ENSG00000214274	283
AMACR	ENSG00000242110	23600
MEFV	ENSG00000103313	4210
TNIK	ENSG00000154310	23043
ASAH1	ENSG00000104763	427
GRM4	ENSG00000124493	2914
KDF1	ENSG00000175707	126695
GNS	ENSG00000135677	2799
B4GALT7	ENSG00000027847	11285
CRYAA	ENSG00000160202	1409
GNAO1	ENSG00000087258	2775
SPR	ENSG00000116096	6697
MIR155	ENSG00000283904	406947
MAPK3	ENSG00000102882	5595
CEP290	ENSG00000198707	80184
STAT3	ENSG00000168610	6774
GDAP2	ENSG00000196505	54834
SPP1	ENSG00000118785	6696
CASK	ENSG00000147044	8573
TMPPE	ENSG00000188167	643853
ITIH4	ENSG00000055955	3700

PEX12	ENSG00000108733	5193
FLT4	ENSG00000037280	2324
KCNJ11	ENSG00000187486	3767
CDH2	ENSG00000170558	1000
PON2	ENSG00000105854	5445
VIM	ENSG00000026025	7431
PAX1	ENSG00000125813	5075
MYH3	ENSG00000109063	4621
SON	ENSG00000159140	6651
SEPTIN9	ENSG00000184640	10801
RIPPLY2	ENSG00000203877	134701
MPO	ENSG00000005381	4353
KCNJ5	ENSG00000120457	3762
LTBP3	ENSG00000168056	4054
TBC1D24	ENSG00000162065	57465
VANGL2	ENSG00000162738	57216
PTPRG	ENSG00000144724	5793
CCR5	ENSG00000160791	1234
RAB3GAP2	ENSG00000118873	25782
FADS2	ENSG00000134824	9415
CTSA	ENSG00000064601	5476
CAMP	ENSG00000164047	820
ERBB2	ENSG00000141736	2064
VIPR2	ENSG00000106018	7434
TSPAN7	ENSG00000156298	7102
PEX5	ENSG00000139197	5830
STK32C	ENSG00000165752	282974
GSN	ENSG00000148180	2934
ABCB4	ENSG00000005471	5244
GPR35	ENSG00000178623	2859
HEXA	ENSG00000213614	3073
SH3PXD2B	ENSG00000174705	285590
ADCY10	ENSG00000143199	55811
HECW2	ENSG00000138411	57520
GATA6	ENSG00000141448	2627
TALDO1	ENSG00000177156	6888
SMAD4	ENSG00000141646	4089
DOCK6	ENSG00000130158	57572
DYNC2LI1	ENSG00000138036	51626
HS2ST1	ENSG00000153936	9653
KAT8	ENSG00000103510	84148
MED27	ENSG00000160563	9442
AGA	ENSG00000038002	175
FHIT	ENSG00000189283	2272

TCTN3	ENSG00000119977	26123
SLC2A2	ENSG00000163581	6514
SELE	ENSG00000007908	6401
KCNQ1	ENSG00000053918	3784
PRKD1	ENSG00000184304	5587
CHRNG	ENSG00000196811	1146
CYP1B1	ENSG00000138061	1545
PAX8	ENSG00000125618	7849
EPO	ENSG00000130427	2056
VAMP2	ENSG00000220205	6844
KCNAB2	ENSG00000069424	8514
MMP23B	ENSG00000189409	8510
PDPN	ENSG00000162493	10630
UBE4B	ENSG00000130939	10277
CASZ1	ENSG00000130940	54897
LUZP1	ENSG00000169641	7798
TRIO	ENSG00000038382	7204
MIR9-1	ENSG00000207933	407046
ATP8B1	ENSG00000081923	5205
BCL2A1	ENSG00000140379	597
CDKN2C	ENSG00000123080	1031
MAMLD1	ENSG00000013619	10046
SLC3A1	ENSG00000138079	6519
RAB23	ENSG00000112210	51715
CNOT1	ENSG00000125107	23019
MTHFD1	ENSG00000100714	4522
EHHADH	ENSG00000113790	1962
ACTL6B	ENSG00000077080	51412
PEX7	ENSG00000112357	5191
HDC	ENSG00000140287	3067
SLITRK1	ENSG00000178235	114798
MIR198	ENSG00000284121	406975
BLK	ENSG00000136573	640
WNT10A	ENSG00000135925	80326
FOXA2	ENSG00000125798	3170
MBP	ENSG00000197971	4155
RETREG1	ENSG00000154153	54463
CUL4B	ENSG00000158290	8450
ADAMTSL2	ENSG00000197859	9719
ARID1A	ENSG00000117713	8289
DAGLA	ENSG00000134780	747
SLC19A1	ENSG00000173638	6573
NLGN4X	ENSG00000146938	57502
UCN	ENSG00000163794	7349

NTNG1	ENSG00000162631	22854
COL18A1	ENSG00000182871	80781
RPS10	ENSG00000124614	6204
COLEC11	ENSG00000118004	78989
EXOC2	ENSG00000112685	55770
NUP88	ENSG00000108559	4927
MEGF8	ENSG00000105429	1954
SMG9	ENSG00000105771	56006
VARS1	ENSG00000204394	7407
ARF1	ENSG00000143761	375
PENK	ENSG00000181195	5179
FCGR2A	ENSG00000143226	2212
FGF14	ENSG00000102466	2259
CEP78	ENSG00000148019	84131
EPHA4	ENSG00000116106	2043
IL1RAPL2	ENSG00000189108	26280
ATM	ENSG00000149311	472
CD14	ENSG00000170458	929
COG4	ENSG00000103051	25839
CD79A	ENSG00000105369	973
IFT52	ENSG00000101052	51098
LIPC	ENSG00000166035	3990
LEPR	ENSG00000116678	3953
IFNAR1	ENSG00000142166	3454
PAM16	ENSG00000217930	51025
ENO2	ENSG00000111674	2026
MESP2	ENSG00000188095	145873
RBFOX3	ENSG00000167281	146713
NPR2	ENSG00000159899	4882
LTBP2	ENSG00000119681	4053
POLR1C	ENSG00000171453	9533
AADAT	ENSG00000109576	51166
KDM5B	ENSG00000117139	10765
ZC3H14	ENSG00000100722	79882
TSNAX	ENSG00000116918	7257
BUB1	ENSG00000169679	699
TLR2	ENSG00000137462	7097
RNF2	ENSG00000121481	6045
UBE2L3	ENSG00000185651	7332
CXCL12	ENSG00000107562	6387
RPL36A-HNRNPH2	ENSG00000257529	100529097
GNAS-AS1	ENSG00000235590	149775
PIK3R2	ENSG00000105647	5296
ALDH4A1	ENSG00000159423	8659

SHROOM4	ENSG00000158352	57477
NEGR1	ENSG00000172260	257194
CDK10	ENSG00000185324	8558
SGO2	ENSG00000163535	151246
AIFM1	ENSG00000156709	9131
ABCB7	ENSG00000131269	22
MN1	ENSG00000169184	4330
SERPINA6	ENSG00000170099	866
PAX7	ENSG00000009709	5081
ADGRG6	ENSG00000112414	57211
PEX1	ENSG00000127980	5189
ICOSLG	ENSG00000160223	23308
ZNF81	ENSG00000197779	347344
KNSTRN	ENSG00000128944	90417
TYR	ENSG00000077498	7299
ADRA2C	ENSG00000184160	152
DCX	ENSG00000077279	1641
NOS1AP	ENSG00000198929	9722
CYP11B1	ENSG00000160882	1584
CHRNA1	ENSG00000138435	1134
CHRND	ENSG00000135902	1144
DCC	ENSG00000187323	1630
FRAS1	ENSG00000138759	80144
INTU	ENSG00000164066	27152
CILK1	ENSG00000112144	22858
PPP2R5D	ENSG00000112640	5528
SOX3	ENSG00000134595	6658
CPLX2	ENSG00000145920	10814
QKI	ENSG00000112531	9444
THBS1	ENSG00000137801	7057
CR2	ENSG00000117322	1380
MTX2	ENSG00000128654	10651
NUCB2	ENSG00000070081	4925
KNG1	ENSG00000113889	3827
SIGMAR1	ENSG00000147955	10280
HSPA1L	ENSG00000204390	3305
TBR1	ENSG00000136535	10716
HSPA1B	ENSG00000204388	3304
PROM1	ENSG00000007062	8842
SP7	ENSG00000170374	121340
FBXW11	ENSG00000072803	23291
TRMT1	ENSG00000104907	55621
ACTL6A	ENSG00000136518	86
IFT80	ENSG00000068885	57560

HLA-C	ENSG00000204525	3107
HSPA4	ENSG00000170606	3308
PLK2	ENSG00000145632	10769
IAPP	ENSG00000121351	3375
SZT2	ENSG00000198198	23334
PRPH	ENSG00000135406	5630
CRBN	ENSG00000113851	51185
PMS1	ENSG00000064933	5378
PGR	ENSG00000082175	5241
CD36	ENSG00000135218	948
NLGN3	ENSG00000196338	54413
CCL3	ENSG00000277632	6348
CHGA	ENSG00000100604	1113
LONP1	ENSG00000196365	9361
FTH1	ENSG00000167996	2495
C4B	ENSG00000224389	721
WDFY3	ENSG00000163625	23001
MED13	ENSG00000108510	9969
TNRC6B	ENSG00000100354	23112
TANC2	ENSG00000170921	26115
ABCC8	ENSG00000006071	6833
GAPDH	ENSG00000111640	2597
SLC39A13	ENSG00000165915	91252
SHOX	ENSG00000185960	6473
TCTN2	ENSG00000168778	79867
MAPK14	ENSG00000112062	1432
VCAN	ENSG00000038427	1462
PEX11B	ENSG00000131779	8799
SNX14	ENSG00000135317	57231
HNF4A	ENSG00000101076	3172
IRF5	ENSG00000128604	3663
MT-CO2	ENSG00000198712	4513
GPD2	ENSG00000115159	2820
PSMD12	ENSG00000197170	5718
AGO2	ENSG00000123908	27161
RLIM	ENSG00000131263	51132
USB1	ENSG00000103005	79650
ARNTL2	ENSG00000029153	56938
LRP1	ENSG00000123384	4035
RPE65	ENSG00000116745	6121
FMR1-AS1	ENSG00000268066	100126270
ADAT3	ENSG00000213638	113179
RPS17	ENSG00000182774	6218
RPL35A	ENSG00000182899	6165

SDHA	ENSG00000073578	6389
IFNB1	ENSG00000171855	3456
TRPS1	ENSG00000104447	7227
LOXL3	ENSG00000115318	84695
OFD1	ENSG00000046651	8481
CCKAR	ENSG00000163394	886
ZNF41	ENSG00000147124	7592
HSD11B2	ENSG00000176387	3291
POLI	ENSG00000101751	11201
TRIP13	ENSG00000071539	9319
MKS1	ENSG00000011143	54903
COL6A3	ENSG00000163359	1293
EED	ENSG00000074266	8726
NMNAT1	ENSG00000173614	64802
BHLHE41	ENSG00000123095	79365
TMEM231	ENSG00000205084	79583
ETFA	ENSG00000140374	2108
ANKH	ENSG00000154122	56172
IFT140	ENSG00000187535	9742
KIF5C	ENSG00000168280	3800
BANK1	ENSG00000153064	55024
AP4E1	ENSG00000081014	23431
PRICKLE1	ENSG00000139174	144165
CCND1	ENSG00000110092	595
SATB1	ENSG00000182568	6304
UBR7	ENSG00000012963	55148
HES7	ENSG00000179111	84667
PPT1	ENSG00000131238	5538
CPN1	ENSG00000120054	1369
PECAM1	ENSG00000261371	5175
CNTNAP1	ENSG00000108797	8506
NCDN	ENSG00000020129	23154
FLNC	ENSG00000128591	2318
HSPA5	ENSG00000044574	3309
BRCA1	ENSG00000012048	672
ABCA7	ENSG00000064687	10347
COL9A1	ENSG00000112280	1297
TFRC	ENSG00000072274	7037
PRKCD	ENSG00000163932	5580
EPG5	ENSG00000152223	57724
IGFBP2	ENSG00000115457	3485
KREMEN1	ENSG00000183762	83999
ETFB	ENSG00000105379	2109
ABCA13	ENSG00000179869	154664

SLC30A8	ENSG00000164756	169026
PLAT	ENSG00000104368	5327
CYLD	ENSG00000083799	1540
DNMT3A	ENSG00000119772	1788
IFNA2	ENSG00000188379	3440
ALDH6A1	ENSG00000119711	4329
ZNF292	ENSG00000188994	23036
PARP1	ENSG00000143799	142
LRRC7	ENSG00000033122	57554
SRD5A3	ENSG00000128039	79644
KCTD1	ENSG00000134504	284252
ARRB2	ENSG00000141480	409
ACOX1	ENSG00000161533	51
SERPINA1	ENSG00000197249	5265
SELL	ENSG00000188404	6402
MUS81	ENSG00000172732	80198
AP1G1	ENSG00000166747	164
KIF11	ENSG00000138160	3832
LMX1B	ENSG00000136944	4010
XPR1	ENSG00000143324	9213
DIS3L2	ENSG00000144535	129563
DHCR24	ENSG00000116133	1718
FREM2	ENSG00000150893	341640
TSR2	ENSG00000158526	90121
GSTM1	ENSG00000134184	2944
COG2	ENSG00000135775	22796
PDSS1	ENSG00000148459	23590
APAF1	ENSG00000120868	317
GM2A	ENSG00000196743	2760
ESCO2	ENSG00000171320	157570
PCSK5	ENSG00000099139	5125
DGKG	ENSG00000058866	1608
MAP3K7	ENSG00000135341	6885
PPARA	ENSG00000186951	5465
BUB3	ENSG00000154473	9184
COL12A1	ENSG00000111799	1303
DMRT1	ENSG00000137090	1761
CDKN3	ENSG00000100526	1033
PPM1B	ENSG00000138032	5495
CDCA7	ENSG00000144354	83879
DCPS	ENSG00000110063	28960
ALKBH8	ENSG00000137760	91801
ADAR	ENSG00000160710	103
LARP7	ENSG00000174720	51574

IARS2	ENSG00000067704	55699
PEX10	ENSG00000157911	5192
RAB3GAP1	ENSG00000115839	22930
PNLIP	ENSG00000175535	5406
RAD54L	ENSG00000085999	8438
NANS	ENSG00000095380	54187
BRDT	ENSG00000137948	676
APOL2	ENSG00000128335	23780
MSH4	ENSG00000057468	4438
TRIM14	ENSG00000106785	9830
APOL4	ENSG00000100336	80832
INSL6	ENSG00000120210	11172
ABCA1	ENSG00000165029	19
RIF1	ENSG00000080345	55183
GATAD2B	ENSG00000143614	57459
SMOC1	ENSG00000198732	64093
PUS7	ENSG00000091127	54517
TRPM3	ENSG00000083067	80036
MYC	ENSG00000136997	4609
RPGRIP1	ENSG00000092200	57096
COASY	ENSG00000068120	80347
DBP	ENSG00000105516	1628
LY86	ENSG00000112799	9450
TBX5	ENSG00000089225	6910
UGP2	ENSG00000169764	7360
ARHGEF6	ENSG00000129675	9459
IYD	ENSG00000009765	389434
ALG13	ENSG00000101901	79868
NTS	ENSG00000133636	4922
GDF1	ENSG00000130283	2657
PLA2G2A	ENSG00000188257	5320
FCGR2B	ENSG00000072694	2213
CTSF	ENSG00000174080	8722
TTC37	ENSG00000198677	9652
CCDC32	ENSG00000128891	90416
BICRA	ENSG00000063169	29998
SERPINA7	ENSG00000123561	6906
LIPN	ENSG00000204020	643418
NR1H2	ENSG00000131408	7376
ABCA12	ENSG00000144452	26154
TBX19	ENSG00000143178	9095
AGL	ENSG00000162688	178
MED12L	ENSG00000144893	116931
CDK13	ENSG00000065883	8621

CASP9	ENSG00000132906	842
HIVEP1	ENSG00000095951	3096
QARS1	ENSG00000172053	5859
CDC42	ENSG00000070831	998
GABRA6	ENSG00000145863	2559
ANK1	ENSG00000029534	286
REV3L	ENSG00000009413	5980
CPLANE1	ENSG00000197603	65250
PLCB3	ENSG00000149782	5331
CLU	ENSG00000120885	1191
GMIP	ENSG00000089639	51291
TMEM161B	ENSG00000164180	153396
FEV	ENSG00000163497	54738
NAGLU	ENSG00000108784	4669
TLR3	ENSG00000164342	7098
FLII	ENSG00000177731	2314
PEX2	ENSG00000164751	5828
PIGU	ENSG00000101464	128869
EIF4E	ENSG00000151247	1977
P4HA2	ENSG00000072682	8974
NDST1	ENSG00000070614	3340
SEMA4D	ENSG00000187764	10507
SEMA4A	ENSG00000196189	64218
ANXA11	ENSG00000122359	311
PON3	ENSG00000105852	5446
TECR	ENSG00000099797	9524
CRADD	ENSG00000169372	8738
AIMP1	ENSG00000164022	9255
MED23	ENSG00000112282	9439
TAF15	ENSG00000270647	8148
TUSC3	ENSG00000104723	7991
PRSS12	ENSG00000164099	8492
FMN2	ENSG00000155816	56776
TBC1D7	ENSG00000145979	51256
UBE4A	ENSG00000110344	9354
MBOAT7	ENSG00000125505	79143
PIGC	ENSG00000135845	5279
UFSP2	ENSG00000109775	55325
CABP4	ENSG00000175544	57010
TTC5	ENSG00000136319	91875
NEMF	ENSG00000165525	9147
JRK	ENSG00000234616	8629
SARS1	ENSG00000031698	6301
C12orf4	ENSG00000047621	57102

NOL4	ENSG00000101746	8715
ACO2	ENSG00000100412	50
XDH	ENSG00000158125	7498
CDC73	ENSG00000134371	79577
PABPN1	ENSG00000100836	8106
NEUROD2	ENSG00000171532	4761
ACP1	ENSG00000143727	52
HMGA1	ENSG00000137309	3159
GNPAT	ENSG00000116906	8443
APBB1	ENSG00000166313	322
USH2A-AS2	ENSG00000233620	102723833
ADAMTS19	ENSG00000145808	171019
KYNU	ENSG00000115919	8942
AMH	ENSG00000104899	268
SCGB1A1	ENSG00000149021	7356
SAT1	ENSG00000130066	6303
IL33	ENSG00000137033	90865
FOXP3	ENSG00000049768	50943
DYNC1H1	ENSG00000197102	1778
PACRG	ENSG00000112530	135138
CUX1	ENSG00000257923	1523
ADA2	ENSG00000093072	51816
POLE	ENSG00000177084	5426
LHX3	ENSG00000107187	8022
ZNF711	ENSG00000147180	7552
PEX19	ENSG00000162735	5824
VEGFC	ENSG00000150630	7424

Targets of depression gathering from OMIM

Gene Symbol	ensembl_id	ENTREZ_id
PRKCZ	ENSG00000067606	5590
PER3	ENSG00000049246	8863
MTOR	ENSG00000198793	2475
MTHFR	ENSG00000177000	4524
PLEKHM2	ENSG00000116786	23207
SDHB	ENSG00000117118	6390
PINK1	ENSG00000158828	65018
KIF17	ENSG00000117245	57576
OPRD1	ENSG00000116329	4985
HPCA	ENSG00000121905	3208

NCDN	ENSG00000020129	23154
PPT1	ENSG00000131238	5538
SLC2A1	ENSG00000117394	6513
MMACHC	ENSG00000132763	25974
PRDX1	ENSG00000117450	5052
LRRC7	ENSG00000033122	57554
COL11A1	ENSG00000060718	1301
CLCC1	ENSG00000121940	23155
RAP1A	ENSG00000116473	5906
KCND3	ENSG00000171385	3752
GDAP2	ENSG00000196505	54834
WARS2	ENSG00000116874	10352
NOTCH2	ENSG00000134250	4853
GJA5	ENSG00000265107	2702
S100A10	ENSG00000197747	6281
MUC1	ENSG00000185499	4582
GBA	ENSG00000177628	2629
MSTO1	ENSG00000125459	55154
AIM2	ENSG00000163568	9447
ACKR1	ENSG00000213088	2532
KCNJ10	ENSG00000177807	3766
ATP1A2	ENSG00000018625	477
CD247	ENSG00000198821	919
FMO3	ENSG00000007933	2328
XPR1	ENSG00000143324	9213
CHI3L1	ENSG00000133048	1116
HSD11B1	ENSG00000117594	3290
USH2A	ENSG00000042781	7399
BPNT1	ENSG00000162813	10380
PSEN2	ENSG00000143801	5664
DISC1	ENSG00000162946	27185
COLEC11	ENSG00000118004	78989
SPAST	ENSG00000021574	6683
PPP3R1	ENSG00000221823	5534
TET3	ENSG00000187605	200424
DCTN1	ENSG00000204843	1639
TACR1	ENSG00000115353	6869
LRRTM1	ENSG00000162951	347730
GGCX	ENSG00000115486	2677
ZEB2	ENSG00000169554	9839
SCN1A	ENSG00000144285	6323
PDE11A	ENSG00000128655	50940
WNT10A	ENSG00000135925	80326
PRSS56	ENSG00000237412	646960

CHRNG	ENSG00000196811	1146
PER2	ENSG00000132326	8864
PDCD1	ENSG00000188389	5133
TRNT1	ENSG00000072756	51095
GRM7	ENSG00000196277	2917
CAV3	ENSG00000182533	859
SYN2	ENSG00000157152	6854
MYL3	ENSG00000160808	4634
TREX1	ENSG00000213689	11277
UQCRC1	ENSG00000010256	7384
BSN	ENSG00000164061	8927
GRM2	ENSG00000164082	2912
TNNC1	ENSG00000114854	7134
PRICKLE2	ENSG00000163637	166336
CPOX	ENSG00000080819	1371
DRD3	ENSG00000151577	1814
GSK3B	ENSG00000082701	2932
CASR	ENSG00000036828	846
ACAD9	ENSG00000177646	28976
CLRN1	ENSG00000163646	7401
TBL1XR1	ENSG00000177565	79718
MASP1	ENSG00000127241	5648
TP63	ENSG00000073282	8626
HTT	ENSG00000197386	3064
WFS1	ENSG00000109501	7466
PHOX2B	ENSG00000109132	8929
GABRA2	ENSG00000151834	2555
CLOCK	ENSG00000134852	9575
ENAM	ENSG00000132464	10117
SNCA	ENSG00000145335	6622
GRID2	ENSG00000152208	2895
ADH1B	ENSG00000196616	125
ADH1C	ENSG00000248144	126
CISD2	ENSG00000145354	493856
EDNRA	ENSG00000151617	1909
TDO2	ENSG00000151790	6999
GRIA2	ENSG00000120251	2891
CASP3	ENSG00000164305	836
SLC25A4	ENSG00000151729	291
SDHA	ENSG00000073578	6389
AMACR	ENSG00000242110	23600
PIK3R1	ENSG00000145675	5295
MCCC2	ENSG00000131844	64087
AP3B1	ENSG00000132842	8546

LMNB1	ENSG00000113368	4001
WNT8A	ENSG00000061492	7478
LRRTM2	ENSG00000146006	26045
PCDHAC2	ENSG00000243232	56134
NR3C1	ENSG00000113580	2908
PPP2R2B	ENSG00000156475	5521
HTR4	ENSG00000164270	3360
ADRB2	ENSG00000169252	154
CSF1R	ENSG00000182578	1436
PDGFRB	ENSG00000113721	5159
GRIA1	ENSG00000155511	2890
DUSP1	ENSG00000120129	1843
DRD1	ENSG00000184845	1812
CPLX2	ENSG00000145920	10814
SNCB	ENSG00000074317	6620
FLT4	ENSG00000037280	2324
NRN1	ENSG00000124785	51299
TBC1D7	ENSG00000145979	51256
DTNBP1	ENSG00000047579	84062
TNF	ENSG00000232810	7124
HSPA1A	ENSG00000204389	3303
HLA-DRB1	ENSG00000196126	3123
HLA-DQA1	ENSG00000196735	3117
HLA-DQB1	ENSG00000179344	3119
TAP1	ENSG00000168394	6890
GRM4	ENSG00000124493	2914
FKBP5	ENSG00000096060	2289
MAPK14	ENSG00000112062	1432
AARS2	ENSG00000124608	57505
EFHC1	ENSG00000096093	114327
RIMS1	ENSG00000079841	22999
HTR1B	ENSG00000135312	3351
HTR1E	ENSG00000168830	3354
CNR1	ENSG00000118432	1268
CEP85L	ENSG00000111860	387119
ENPP1	ENSG00000197594	5167
GRM1	ENSG00000152822	2911
ESR1	ENSG00000091831	2099
OPRM1	ENSG00000112038	4988
TBP	ENSG00000112592	6908
FAM20C	ENSG00000177706	56975
NPY	ENSG00000122585	4852
PPP1R17	ENSG00000106341	10842
CAMK2B	ENSG00000058404	816

ADCY1	ENSG00000164742	107
ABCA13	ENSG00000179869	154664
CHCHD2	ENSG00000106153	51142
SGCE	ENSG00000127990	8910
TRRAP	ENSG00000196367	8295
CYP3A4	ENSG00000160868	1576
LEP	ENSG00000174697	3952
CHRM2	ENSG00000181072	1129
BRAF	ENSG00000157764	673
RP1L1	ENSG00000183638	94137
DPYSL2	ENSG00000092964	1808
FGFR1	ENSG00000077782	2260
SLC20A2	ENSG00000168575	6575
THAP1	ENSG00000131931	55145
TRPA1	ENSG00000104321	8989
RRM2B	ENSG00000048392	50484
RAD21	ENSG00000164754	5885
EXT1	ENSG00000182197	2131
COLEC10	ENSG00000184374	10584
OPLAH	ENSG00000178814	26873
IFNA2	ENSG00000188379	3440
C9orf72	ENSG00000147894	203228
BAG1	ENSG00000107262	573
FXN	ENSG00000165060	2395
VPS13A	ENSG00000197969	23230
GAS1	ENSG00000180447	2619
IARS1	ENSG00000196305	3376
ELP1	ENSG00000070061	8518
STXBP1	ENSG00000136854	6812
TOR1A	ENSG00000136827	1861
NCS1	ENSG00000107130	23413
ABL1	ENSG00000097007	25
TSC1	ENSG00000165699	7248
KCNT1	ENSG00000107147	57582
GRIN1	ENSG00000176884	2902
CACNA1B	ENSG00000148408	774
FZD8	ENSG00000177283	8325
DNAJC12	ENSG00000108176	56521
SIRT1	ENSG00000096717	23411
OPN4	ENSG00000122375	94233
LGI1	ENSG00000108231	9211
HPS1	ENSG00000107521	3257
COX15	ENSG00000014919	1355
TWNK	ENSG00000107815	56652

PDZD7	ENSG00000186862	79955
SORCS3	ENSG00000156395	22986

Targets of depression gathering from DisGeNET

Gene Symbol	ensembl_id	ENTREZ_id
TPH1	ENSG00000129167	7166
S100A10	ENSG00000197747	6281
CRHR1	ENSG00000276191	1394
SLC6A2	ENSG00000103546	6530
NTRK2	ENSG00000148053	4915
HTR2A	ENSG00000102468	3356
NPY	ENSG00000122585	4852
CRH	ENSG00000147571	1392
DRD2	ENSG00000149295	1813
SLC6A4	ENSG00000108576	6532
NR3C1	ENSG00000113580	2908
S100B	ENSG00000160307	6285
BDNF	ENSG00000176697	627
IL1B	ENSG00000125538	3553
IL6	ENSG00000136244	3569
GSK3B	ENSG00000082701	2932
NGF	ENSG00000134259	4803
CHRM2	ENSG00000181072	1129
ADRA2A	ENSG00000150594	150
DRD1	ENSG00000184845	1812
DBH	ENSG00000123454	1621
CRHBP	ENSG00000145708	1393
CRHR2	ENSG00000106113	1395
PPP1R1B	ENSG00000131771	84152
IL6R	ENSG00000160712	3570
HDAC5	ENSG00000108840	10014
PDE4D	ENSG00000113448	5144
TH	ENSG00000180176	7054
KCNK2	ENSG00000082482	3776
HTR2C	ENSG00000147246	3358
OPRK1	ENSG00000082556	4986
DISC1	ENSG00000162946	27185
TPH2	ENSG00000139287	121278
OXTR	ENSG00000180914	5021
FKBP5	ENSG00000096060	2289

HTR1A	ENSG00000178394	3350
DRD4	ENSG00000069696	1815
GRIN2B	ENSG00000273079	2904
HCRT	ENSG00000161610	3060
GAL	ENSG00000069482	51083
GAD1	ENSG00000128683	2571
ARTN	ENSG00000117407	9048
NR3C2	ENSG00000151623	4306
MAOA	ENSG00000189221	4128
CREB1	ENSG00000118260	1385
IDO1	ENSG00000131203	3620
SLC6A3	ENSG00000276996	6531
P2RX7	ENSG00000089041	5027
CNR1	ENSG00000118432	1268
CYP2D6	ENSG00000272000	1565
TAC1	ENSG00000006128	6863
COMT	ENSG00000093010	1312
POMC	ENSG00000115138	5443
AR	ENSG00000169083	367
LEP	ENSG00000174697	3952
ABCB1	ENSG00000085563	5243
MTHFR	ENSG00000177000	4524
ESR1	ENSG00000091831	2099
IGF1	ENSG00000017427	3479
PTGS2	ENSG00000073756	5743
IFNG	ENSG00000111537	3458
VEGFA	ENSG00000112715	7422
TNF	ENSG00000230108	7124
CLOCK	ENSG00000134852	9575
REN	ENSG00000143839	5972
IL18	ENSG00000150782	3606
IL1A	ENSG00000115008	3552
GRIA1	ENSG00000155511	2890
CXCL8	ENSG00000169429	3576
PCLO	ENSG00000186472	27445
BICC1	ENSG00000122870	80114
DRD3	ENSG00000151577	1814
CACNA1C	ENSG00000151067	775
CYP2C19	ENSG00000165841	1557
COX2	ENSG00000198712	4513
SLC18A2	ENSG00000165646	6571
HTR1B	ENSG00000135312	3351
PDE4A	ENSG00000065989	5141
ARNTL	ENSG00000133794	406

GFAP	ENSG00000131095	2670
HP	ENSG00000257017	3240
TNFRSF1A	ENSG00000067182	7132
HTR3A	ENSG00000166736	3359
PDE4B	ENSG00000184588	5142
MAOB	ENSG00000069535	4129
CNR2	ENSG00000188822	1269
PER2	ENSG00000132326	8864
FTO	ENSG00000140718	79068
MAPK3	ENSG00000102882	5595
NOS3	ENSG00000164867	4846
BRCA1	ENSG00000012048	672
NOS2	ENSG00000007171	4843
AKT1	ENSG00000142208	207
HOMER1	ENSG00000152413	9456
VGF	ENSG00000128564	7425
CARTPT	ENSG00000164326	9607
TRH	ENSG00000170893	7200
GNB3	ENSG00000111664	2784
CHRNA4	ENSG00000101204	1137
TNFRSF1B	ENSG00000028137	7133
MCHR1	ENSG00000128285	2847
GRM7	ENSG00000196277	2917
HTR4	ENSG00000164270	3360
HTR7	ENSG00000148680	3363
DTNBP1	ENSG00000047579	84062
DPYSL2	ENSG00000092964	1808
GAP43	ENSG00000172020	2596
PER3	ENSG00000049246	8863
GABRB3	ENSG00000166206	2562
GLO1	ENSG00000124767	2739
PDYN	ENSG00000101327	5173
RORA	ENSG00000069667	6095
GRIN2A	ENSG00000183454	2903
ATXN3	ENSG00000066427	4287
HSD11B1	ENSG00000117594	3290
RELN	ENSG00000189056	5649
SLC1A2	ENSG00000110436	6506
DUSP1	ENSG00000120129	1843
HDAC2	ENSG00000196591	3066
PSEN1	ENSG00000080815	5663
NRG1	ENSG00000157168	3084
OPRM1	ENSG00000112038	4988
APRT	ENSG00000198931	353

APP	ENSG00000142192	351
NCAM1	ENSG00000149294	4684
AGT	ENSG00000135744	183
FGFR1	ENSG00000077782	2260
PDE1B	ENSG00000123360	5153
DAOA	ENSG00000182346	267012
ADCY7	ENSG00000121281	113
SLC18A1	ENSG00000036565	6570
NPS	ENSG00000214285	594857
ADCY5	ENSG00000173175	111
OXT	ENSG00000101405	5020
PMCH	ENSG00000183395	5367
CAMK2A	ENSG00000070808	815
ANKK1	ENSG00000170209	255239
CRY2	ENSG00000121671	1408
NRXN1	ENSG00000179915	9378
GRIA3	ENSG00000125675	2892
CNTF	ENSG00000242689	1270
HTT	ENSG00000197386	3064
UCN	ENSG00000163794	7349
TIMELESS	ENSG00000111602	8914
WFS1	ENSG00000109501	7466
CHAT	ENSG00000070748	1103
TACR1	ENSG00000115353	6869
MTR	ENSG00000116984	4548
MED12	ENSG00000184634	9968
GPX1	ENSG00000233276	2876
STMN1	ENSG00000117632	3925
HDAC9	ENSG00000048052	9734
RAC1	ENSG00000136238	5879
TTR	ENSG00000118271	7276
FOS	ENSG00000170345	2353
GSTM1	ENSG00000134184	2944
SOD1	ENSG00000142168	6647
GH1	ENSG00000259384	2688
CDKN2A	ENSG00000147889	1029
PEX5L	ENSG00000114757	51555
SLCO1C1	ENSG00000139155	53919
CPLX2	ENSG00000145920	10814
RNF123	ENSG00000164068	63891
DAOA-AS1	ENSG00000232307	282706
M6PR	ENSG00000003056	4074
ADCY8	ENSG00000155897	114
ASMT	ENSG00000196433	438

GABRA6	ENSG00000145863	2559
HTR3B	ENSG00000149305	9177
GRIK3	ENSG00000163873	2899
OAS2	ENSG00000111335	4939
AGO1	ENSG00000092847	26523
LTA4H	ENSG00000111144	4048
KCNJ6	ENSG00000157542	3763
CMKLR1	ENSG00000174600	1240
WWC1	ENSG00000113645	23286
NPSR1	ENSG00000187258	387129
PNOC	ENSG00000168081	5368
EGR3	ENSG00000179388	1960
SYN1	ENSG00000008056	6853
DLG4	ENSG00000132535	1742
PYY	ENSG00000131096	5697
CRY1	ENSG00000008405	1407
DGCR8	ENSG00000128191	54487
GYPE	ENSG00000197465	2996
SLC29A3	ENSG00000198246	55315
CPLX1	ENSG00000168993	10815
DEAF1	ENSG00000282712	10522
LGI1	ENSG00000108231	9211
HCN1	ENSG00000164588	348980
ADCYAP1R1	ENSG00000078549	117
PFKFB3	ENSG00000170525	5209
TLE1	ENSG00000196781	7088
ADRB1	ENSG00000043591	153
MC4R	ENSG00000166603	4160
GRK2	ENSG00000173020	156
MC1R	ENSG00000258839	4157
NTS	ENSG00000133636	4922
GLUL	ENSG00000135821	2752
ADCYAP1	ENSG00000141433	116
LDHA	ENSG00000288299	3939
CDH13	ENSG00000140945	1012
ATF3	ENSG00000162772	467
HDAC4	ENSG00000068024	9759
NGFR	ENSG00000064300	4804
CALM2	ENSG00000143933	805
HDAC6	ENSG00000094631	10013
AQP4	ENSG00000171885	361
FOLH1	ENSG00000086205	2346
ERBB3	ENSG00000065361	2065
DPP4	ENSG00000197635	1803

MAPK8	ENSG00000107643	5599
SOD2	ENSG00000112096	6648
HIF1A	ENSG00000100644	3091
OR7D4	ENSG00000174667	125958
PPP3CC	ENSG00000120910	5533
GRID1	ENSG00000182771	2894
ARHGEF10	ENSG00000274726	9639
DGKB	ENSG00000136267	1607
CHRNA6	ENSG00000147434	8973
CHRNA2	ENSG00000120903	1135
CHRNB3	ENSG00000147432	1142
CSMD2	ENSG00000121904	114784
IMPA2	ENSG00000141401	3613
TBX19	ENSG00000143178	9095
NEFM	ENSG00000104722	4741
AANAT	ENSG00000129673	15
FGF20	ENSG00000078579	26281
DKK4	ENSG00000104371	27121
GABRA3	ENSG00000011677	2556
SRD5A1	ENSG00000145545	6715
SGCE	ENSG00000127990	8910
HCRTR1	ENSG00000121764	3061
SERPINA6	ENSG00000277405	866
DUSP4	ENSG00000120875	1846
GRPR	ENSG00000126010	2925
NPAS2	ENSG00000170485	4862
FEV	ENSG00000163497	54738
FGF17	ENSG00000158815	8822
SLC6A1	ENSG00000157103	6529
PRKCI	ENSG00000163558	5584
ARRB2	ENSG00000141480	409
SLC1A1	ENSG00000106688	6505
PENK	ENSG00000181195	5179
ATP1A3	ENSG00000105409	478
BAG1	ENSG00000107262	573
A2M	ENSG00000175899	2
ATP2A2	ENSG00000174437	488
GRM1	ENSG00000152822	2911
DUSP6	ENSG00000139318	1848
ATF4	ENSG00000128272	468
SNAP25	ENSG00000132639	6616
LIF	ENSG00000128342	3976
SFRP1	ENSG00000104332	6422
GRN	ENSG00000030582	2896

SNCA	ENSG00000145335	6622
ALK	ENSG00000171094	238
GSTT1	ENSG00000277656	2952
FGFR2	ENSG00000066468	2263

Deduplicated targets of depression

ensembl_id
ENSG00000129167
ENSG00000197747
ENSG00000276191
ENSG00000103546
ENSG00000148053
ENSG00000102468
ENSG00000122585
ENSG00000147571
ENSG00000149295
ENSG00000108576
ENSG00000113580
ENSG00000160307
ENSG00000176697
ENSG00000125538
ENSG00000136244
ENSG00000082701
ENSG00000134259
ENSG00000181072
ENSG00000150594
ENSG00000184845
ENSG00000123454
ENSG00000145708
ENSG00000106113
ENSG00000131771
ENSG00000160712
ENSG00000108840
ENSG00000113448
ENSG00000180176
ENSG00000082482
ENSG00000147246
ENSG00000082556
ENSG00000162946
ENSG00000139287

ENSG00000180914
ENSG00000096060
ENSG00000178394
ENSG00000069696
ENSG00000273079
ENSG00000161610
ENSG00000069482
ENSG00000128683
ENSG00000117407
ENSG00000151623
ENSG00000189221
ENSG00000118260
ENSG00000131203
ENSG00000276996
ENSG00000089041
ENSG00000118432
ENSG00000272000
ENSG00000006128
ENSG00000093010
ENSG00000115138
ENSG00000169083
ENSG00000174697
ENSG00000085563
ENSG00000177000
ENSG00000091831
ENSG00000017427
ENSG00000073756
ENSG00000111537
ENSG00000112715
ENSG00000230108
ENSG00000134852
ENSG00000143839
ENSG00000150782
ENSG00000115008
ENSG00000155511
ENSG00000169429
ENSG00000186472
ENSG00000122870
ENSG00000151577
ENSG00000151067
ENSG00000165841
ENSG00000198712
ENSG00000165646
ENSG00000135312

ENSG00000065989
ENSG00000133794
ENSG00000131095
ENSG00000257017
ENSG00000067182
ENSG00000166736
ENSG00000184588
ENSG00000069535
ENSG00000188822
ENSG00000132326
ENSG00000140718
ENSG00000102882
ENSG00000164867
ENSG00000012048
ENSG00000007171
ENSG00000142208
ENSG00000152413
ENSG00000128564
ENSG00000164326
ENSG00000170893
ENSG00000111664
ENSG00000101204
ENSG00000028137
ENSG00000128285
ENSG00000196277
ENSG00000164270
ENSG00000148680
ENSG00000047579
ENSG00000092964
ENSG00000172020
ENSG00000049246
ENSG00000166206
ENSG00000124767
ENSG00000101327
ENSG00000069667
ENSG00000183454
ENSG00000066427
ENSG00000117594
ENSG00000189056
ENSG00000110436
ENSG00000120129
ENSG00000196591
ENSG00000080815
ENSG00000157168

ENSG00000112038
ENSG00000198931
ENSG00000142192
ENSG00000149294
ENSG00000135744
ENSG00000077782
ENSG00000123360
ENSG00000182346
ENSG00000121281
ENSG00000036565
ENSG00000214285
ENSG00000173175
ENSG00000101405
ENSG00000183395
ENSG00000070808
ENSG00000170209
ENSG00000121671
ENSG00000179915
ENSG00000125675
ENSG00000242689
ENSG00000197386
ENSG00000163794
ENSG00000111602
ENSG00000109501
ENSG00000070748
ENSG00000115353
ENSG00000116984
ENSG00000184634
ENSG00000233276
ENSG00000117632
ENSG00000048052
ENSG00000136238
ENSG00000118271
ENSG00000170345
ENSG00000134184
ENSG00000142168
ENSG00000259384
ENSG00000147889
ENSG00000114757
ENSG00000139155
ENSG00000145920
ENSG00000164068
ENSG00000232307
ENSG00000003056

ENSG00000155897
ENSG00000196433
ENSG00000145863
ENSG00000149305
ENSG00000163873
ENSG00000111335
ENSG00000092847
ENSG00000111144
ENSG00000157542
ENSG00000174600
ENSG00000113645
ENSG00000187258
ENSG00000168081
ENSG00000179388
ENSG00000008056
ENSG00000132535
ENSG00000131096
ENSG00000008405
ENSG00000128191
ENSG00000197465
ENSG00000198246
ENSG00000168993
ENSG00000282712
ENSG00000108231
ENSG00000164588
ENSG00000078549
ENSG00000170525
ENSG00000196781
ENSG00000043591
ENSG00000166603
ENSG00000173020
ENSG00000258839
ENSG00000133636
ENSG00000135821
ENSG00000141433
ENSG00000288299
ENSG00000140945
ENSG00000162772
ENSG00000068024
ENSG00000064300
ENSG00000143933
ENSG00000094631
ENSG00000171885
ENSG00000086205

ENSG00000065361
ENSG00000197635
ENSG00000107643
ENSG00000112096
ENSG00000100644
ENSG00000174667
ENSG00000120910
ENSG00000182771
ENSG00000274726
ENSG00000136267
ENSG00000147434
ENSG00000120903
ENSG00000147432
ENSG00000121904
ENSG00000141401
ENSG00000143178
ENSG00000104722
ENSG00000129673
ENSG00000078579
ENSG00000104371
ENSG00000011677
ENSG00000145545
ENSG00000127990
ENSG00000121764
ENSG00000277405
ENSG00000120875
ENSG00000126010
ENSG00000170485
ENSG00000163497
ENSG00000158815
ENSG00000157103
ENSG00000163558
ENSG00000141480
ENSG00000106688
ENSG00000181195
ENSG00000105409
ENSG00000107262
ENSG00000175899
ENSG00000174437
ENSG00000152822
ENSG00000139318
ENSG00000128272
ENSG00000132639
ENSG00000128342

ENSG00000104332
ENSG00000030582
ENSG00000145335
ENSG00000171094
ENSG00000277656
ENSG00000066468
ENSG00000074181
ENSG00000232810
ENSG00000172179
ENSG00000130203
ENSG00000142319
ENSG00000120088
ENSG00000132693
ENSG00000186868
ENSG00000204843
ENSG00000100197
ENSG00000100219
ENSG00000136634
ENSG00000140521
ENSG00000254647
ENSG00000120251
ENSG00000179546
ENSG00000171867
ENSG00000102081
ENSG00000177628
ENSG00000153234
ENSG00000169057
ENSG00000100151
ENSG00000130816
ENSG00000152208
ENSG00000267200
ENSG00000109471
ENSG00000168621
ENSG00000115705
ENSG00000251380
ENSG00000145675
ENSG00000102924
ENSG00000163110
ENSG00000157017
ENSG00000147437
ENSG00000197408
ENSG00000168959
ENSG00000075711
ENSG00000140505

ENSG00000072041
ENSG00000126368
ENSG00000185920
ENSG00000185345
ENSG00000157764
ENSG00000188906
ENSG00000133703
ENSG00000184381
ENSG00000169032
ENSG00000161202
ENSG00000087460
ENSG00000164082
ENSG00000005339
ENSG00000160868
ENSG00000138109
ENSG00000089250
ENSG00000198793
ENSG00000022355
ENSG00000147050
ENSG00000157005
ENSG00000136827
ENSG00000159640
ENSG00000131979
ENSG00000179344
ENSG00000139219
ENSG00000162267
ENSG00000108821
ENSG00000176884
ENSG00000164418
ENSG00000159082
ENSG00000198822
ENSG00000163631
ENSG00000100033
ENSG00000074047
ENSG00000147894
ENSG00000107831
ENSG00000251322
ENSG00000164690
ENSG00000096717
ENSG00000204248
ENSG00000126934
ENSG00000060718
ENSG00000151150
ENSG00000141510

ENSG00000171862
ENSG00000170396
ENSG00000110887
ENSG00000100300
ENSG00000100362
ENSG00000196126
ENSG00000233608
ENSG00000127415
ENSG00000185652
ENSG00000157152
ENSG00000118058
ENSG00000100030
ENSG00000112592
ENSG00000052850
ENSG00000100393
ENSG00000164961
ENSG00000064835
ENSG00000256525
ENSG00000136068
ENSG00000102195
ENSG00000137845
ENSG00000079215
ENSG00000187730
ENSG00000152578
ENSG00000111424
ENSG00000167548
ENSG00000110711
ENSG00000204842
ENSG00000100385
ENSG00000048392
ENSG00000103197
ENSG00000149485
ENSG00000179295
ENSG00000007372
ENSG00000145354
ENSG00000108946
ENSG00000122375
ENSG00000081189
ENSG00000133731
ENSG00000174469
ENSG00000043355
ENSG00000177426
ENSG00000100299
ENSG00000004478

ENSG00000154309
ENSG00000160949
ENSG00000165458
ENSG00000198888
ENSG00000198719
ENSG00000107736
ENSG00000005421
ENSG00000184985
ENSG00000127483
ENSG00000144285
ENSG00000104774
ENSG00000120907
ENSG00000181722
ENSG00000165280
ENSG00000175344
ENSG00000008441
ENSG00000123066
ENSG00000068078
ENSG00000138083
ENSG00000177706
ENSG00000183098
ENSG00000120948
ENSG00000047597
ENSG00000151729
ENSG00000006016
ENSG00000136750
ENSG00000008196
ENSG00000181092
ENSG00000157193
ENSG00000123473
ENSG00000100311
ENSG00000042781
ENSG00000163666
ENSG00000166311
ENSG00000064309
ENSG00000149403
ENSG00000086848
ENSG00000168036
ENSG00000196136
ENSG00000174521
ENSG00000113327
ENSG00000135914
ENSG00000180447
ENSG00000082458

ENSG00000160973
ENSG00000197919
ENSG00000073282
ENSG00000085377
ENSG00000131018
ENSG00000175505
ENSG00000171316
ENSG00000133895
ENSG00000109911
ENSG00000106460
ENSG00000162923
ENSG00000042832
ENSG00000156574
ENSG00000117394
ENSG00000116288
ENSG00000164619
ENSG00000153283
ENSG00000122550
ENSG00000187094
ENSG00000128655
ENSG00000114988
ENSG00000129003
ENSG00000196735
ENSG00000196628
ENSG00000245573
ENSG00000248144
ENSG00000175445
ENSG00000117152
ENSG00000113721
ENSG00000183091
ENSG00000105866
ENSG00000163599
ENSG00000168137
ENSG00000171189
ENSG00000167074
ENSG00000141404
ENSG00000241186
ENSG00000072415
ENSG00000095787
ENSG00000132170
ENSG00000167632
ENSG00000213923
ENSG00000178568
ENSG00000272333

ENSG00000196358
ENSG00000101200
ENSG00000100379
ENSG00000122863
ENSG00000089280
ENSG00000101997
ENSG00000072062
ENSG00000151834
ENSG00000076716
ENSG00000171759
ENSG00000145191
ENSG00000184058
ENSG00000181090
ENSG00000138592
ENSG00000253199
ENSG00000197746
ENSG00000132024
ENSG00000075073
ENSG00000184156
ENSG00000078018
ENSG00000139618
ENSG00000160299
ENSG00000214194
ENSG00000226673
ENSG00000180340
ENSG00000142798
ENSG00000163288
ENSG00000124587
ENSG00000128973
ENSG00000135929
ENSG00000197563
ENSG00000134532
ENSG00000102003
ENSG00000101972
ENSG00000083937
ENSG00000169432
ENSG00000134242
ENSG00000114353
ENSG00000168016
ENSG00000151322
ENSG00000085491
ENSG00000064692
ENSG00000154146
ENSG00000103266

ENSG00000198947
ENSG00000095970
ENSG00000141458
ENSG00000196924
ENSG00000104320
ENSG00000186297
ENSG00000160801
ENSG00000072501
ENSG00000036828
ENSG00000138246
ENSG00000135365
ENSG00000117595
ENSG00000166147
ENSG00000140937
ENSG00000170266
ENSG00000121075
ENSG00000237763
ENSG00000234745
ENSG00000134460
ENSG00000113273
ENSG00000124155
ENSG00000100014
ENSG00000132600
ENSG00000151617
ENSG00000069431
ENSG00000068366
ENSG00000123191
ENSG00000160213
ENSG00000187733
ENSG00000174876
ENSG00000151952
ENSG00000183117
ENSG00000111276
ENSG00000132437
ENSG00000188389
ENSG00000162105
ENSG00000104888
ENSG00000169836
ENSG00000177030
ENSG00000213930
ENSG00000083444
ENSG00000145864
ENSG00000059804
ENSG00000169375

ENSG00000277443
ENSG00000145217
ENSG00000128567
ENSG00000117480
ENSG00000207569
ENSG00000204681
ENSG00000143473
ENSG00000134531
ENSG00000132155
ENSG00000169676
ENSG00000140650
ENSG00000158828
ENSG00000284214
ENSG00000138078
ENSG00000141837
ENSG00000108691
ENSG00000178573
ENSG00000140009
ENSG00000049540
ENSG00000116675
ENSG00000151689
ENSG00000143815
ENSG00000166148
ENSG00000102145
ENSG00000284202
ENSG00000085224
ENSG00000106089
ENSG00000112964
ENSG00000186487
ENSG00000196712
ENSG00000113520
ENSG00000126705
ENSG00000178127
ENSG00000105722
ENSG00000198951
ENSG00000136689
ENSG00000188037
ENSG00000126583
ENSG00000122512
ENSG00000142599
ENSG00000107882
ENSG00000133048
ENSG00000136531
ENSG00000130287

ENSG00000198049
ENSG00000196218
ENSG00000143341
ENSG00000169554
ENSG00000137203
ENSG00000182578
ENSG00000161011
ENSG00000136854
ENSG00000042753
ENSG00000099381
ENSG00000010165
ENSG00000165195
ENSG00000144644
ENSG00000005483
ENSG00000124313
ENSG00000124181
ENSG00000167522
ENSG00000147133
ENSG00000165995
ENSG00000196616
ENSG00000111199
ENSG00000196689
ENSG00000165186
ENSG00000284459
ENSG00000131943
ENSG00000172977
ENSG00000152661
ENSG00000183287
ENSG00000155850
ENSG00000162878
ENSG00000196367
ENSG00000186197
ENSG00000160716
ENSG00000166548
ENSG00000136869
ENSG00000152217
ENSG00000125845
ENSG00000174177
ENSG00000196159
ENSG00000104327
ENSG00000182890
ENSG00000100207
ENSG00000159363
ENSG00000121691

ENSG00000165588
ENSG00000124608
ENSG00000105329
ENSG00000108055
ENSG00000185559
ENSG00000155974
ENSG00000169252
ENSG00000174775
ENSG00000173085
ENSG00000154277
ENSG00000198974
ENSG00000025434
ENSG00000064651
ENSG00000115594
ENSG00000007933
ENSG00000148737
ENSG00000158813
ENSG00000101292
ENSG00000103061
ENSG00000091483
ENSG00000166598
ENSG00000078401
ENSG00000196611
ENSG00000236107
ENSG00000087085
ENSG00000109163
ENSG00000088305
ENSG00000158748
ENSG00000125398
ENSG00000204406
ENSG00000106571
ENSG00000109819
ENSG00000163485
ENSG00000154864
ENSG00000002822
ENSG00000158055
ENSG00000100985
ENSG00000110680
ENSG00000130779
ENSG00000146674
ENSG00000186716
ENSG00000108557
ENSG00000108474
ENSG00000121879

ENSG00000196230
ENSG00000206503
ENSG00000107404
ENSG00000135960
ENSG00000091513
ENSG00000110514
ENSG00000213689
ENSG00000012061
ENSG00000111275
ENSG00000187714
ENSG00000164053
ENSG00000115902
ENSG00000011405
ENSG00000102393
ENSG00000165194
ENSG00000132510
ENSG00000124762
ENSG00000169604
ENSG00000114251
ENSG00000214548
ENSG00000116014
ENSG00000114270
ENSG00000166863
ENSG00000102780
ENSG00000162426
ENSG00000141431
ENSG00000096968
ENSG00000105711
ENSG00000163421
ENSG00000144711
ENSG00000148384
ENSG00000127995
ENSG00000125779
ENSG00000118972
ENSG00000102452
ENSG00000138798
ENSG00000163637
ENSG00000124813
ENSG00000107147
ENSG00000006611
ENSG00000174175
ENSG00000118971
ENSG00000167701
ENSG00000130638

ENSG00000134954
ENSG00000183735
ENSG00000170498
ENSG00000127948
ENSG00000155961
ENSG00000156970
ENSG00000106009
ENSG00000112115
ENSG00000157851
ENSG00000169071
ENSG00000037474
ENSG00000198804
ENSG00000116329
ENSG00000115306
ENSG00000204120
ENSG00000173575
ENSG00000137601
ENSG00000125459
ENSG00000171608
ENSG00000104918
ENSG00000134640
ENSG00000150995
ENSG00000106683
ENSG00000152266
ENSG00000084674
ENSG00000118503
ENSG00000175325
ENSG00000198576
ENSG00000149256
ENSG00000108963
ENSG00000065526
ENSG00000156113
ENSG00000137474
ENSG00000199133
ENSG00000177189
ENSG00000134200
ENSG00000165802
ENSG00000204305
ENSG00000180772
ENSG00000171791
ENSG00000196839
ENSG00000167941
ENSG00000255072
ENSG00000166862

ENSG00000197969
ENSG00000090339
ENSG00000127616
ENSG00000197283
ENSG00000172922
ENSG00000143603
ENSG00000115904
ENSG00000080503
ENSG00000124222
ENSG00000054598
ENSG00000106366
ENSG00000100150
ENSG00000101126
ENSG00000112425
ENSG00000129152
ENSG00000157540
ENSG00000012504
ENSG00000197121
ENSG00000133835
ENSG00000107779
ENSG00000164305
ENSG00000166579
ENSG00000187240
ENSG00000123700
ENSG00000120937
ENSG00000214160
ENSG00000128271
ENSG00000189079
ENSG00000156650
ENSG00000256269
ENSG00000269900
ENSG00000156475
ENSG00000171105
ENSG00000177728
ENSG00000095002
ENSG00000103264
ENSG00000049618
ENSG00000129757
ENSG00000105737
ENSG00000180210
ENSG00000198938
ENSG00000138685
ENSG00000208023
ENSG00000014641

ENSG00000118965
ENSG00000109158
ENSG00000165731
ENSG00000185129
ENSG00000159579
ENSG00000163737
ENSG00000030304
ENSG00000069329
ENSG00000112365
ENSG00000165917
ENSG00000050030
ENSG00000175497
ENSG00000006283
ENSG00000107130
ENSG00000143224
ENSG00000271605
ENSG00000163513
ENSG00000196557
ENSG00000008988
ENSG00000128573
ENSG00000076242
ENSG00000187678
ENSG00000136720
ENSG00000120008
ENSG00000012232
ENSG00000161509
ENSG00000186862
ENSG00000150275
ENSG00000135069
ENSG00000151348
ENSG00000160200
ENSG00000103489
ENSG00000124788
ENSG00000164199
ENSG00000115392
ENSG00000147099
ENSG00000112312
ENSG00000181830
ENSG00000147883
ENSG00000138735
ENSG00000198786
ENSG00000174891
ENSG00000198836
ENSG00000197694

ENSG00000165478
ENSG00000162063
ENSG00000184216
ENSG00000213281
ENSG00000199080
ENSG00000225602
ENSG00000122966
ENSG00000078295
ENSG00000112139
ENSG00000092621
ENSG00000071462
ENSG00000171456
ENSG00000146469
ENSG00000198886
ENSG00000154118
ENSG00000114956
ENSG00000128245
ENSG00000189058
ENSG00000242950
ENSG00000137869
ENSG00000250479
ENSG00000069011
ENSG00000175920
ENSG00000100578
ENSG00000177239
ENSG00000165671
ENSG00000243970
ENSG00000086758
ENSG00000112367
ENSG00000185946
ENSG00000135486
ENSG00000171132
ENSG00000163646
ENSG00000075043
ENSG00000187049
ENSG00000162692
ENSG00000184009
ENSG00000106682
ENSG00000106665
ENSG00000009954
ENSG00000138688
ENSG00000048342
ENSG00000138326
ENSG00000114867

ENSG00000009950
ENSG00000188641
ENSG00000113161
ENSG00000110799
ENSG00000183072
ENSG00000179094
ENSG00000198690
ENSG00000134982
ENSG00000283819
ENSG00000124486
ENSG00000119684
ENSG00000166037
ENSG00000135100
ENSG00000150787
ENSG00000187676
ENSG00000176022
ENSG00000125378
ENSG00000165699
ENSG00000174227
ENSG00000176165
ENSG00000088038
ENSG00000181038
ENSG00000099956
ENSG00000100815
ENSG00000140199
ENSG00000134323
ENSG00000263001
ENSG00000124006
ENSG00000142611
ENSG00000095397
ENSG00000074317
ENSG00000138175
ENSG00000188021
ENSG00000105641
ENSG00000119718
ENSG00000130649
ENSG00000040608
ENSG00000164692
ENSG00000164093
ENSG00000028116
ENSG00000121068
ENSG00000141337
ENSG00000107249
ENSG00000244731

ENSG00000166558
ENSG00000150867
ENSG00000199090
ENSG00000164754
ENSG00000271503
ENSG00000179151
ENSG00000143442
ENSG00000171503
ENSG00000169919
ENSG00000101210
ENSG00000258555
ENSG00000107815
ENSG00000241111
ENSG00000066923
ENSG00000100697
ENSG00000099942
ENSG00000143632
ENSG00000101439
ENSG00000103089
ENSG00000157399
ENSG00000174417
ENSG00000163736
ENSG00000254656
ENSG00000156110
ENSG00000144455
ENSG00000111262
ENSG00000135903
ENSG00000026103
ENSG00000100077
ENSG00000170445
ENSG00000151148
ENSG00000175104
ENSG00000004848
ENSG00000016864
ENSG00000008083
ENSG00000112246
ENSG00000172893
ENSG00000104687
ENSG00000020922
ENSG00000275410
ENSG00000158517
ENSG00000176410
ENSG00000105695
ENSG00000079459

ENSG00000113368
ENSG00000208017
ENSG00000099940
ENSG00000147416
ENSG00000100292
ENSG00000104936
ENSG00000108518
ENSG00000179603
ENSG00000136574
ENSG00000021574
ENSG00000147257
ENSG00000105372
ENSG00000104218
ENSG00000055609
ENSG00000119392
ENSG00000146678
ENSG00000118702
ENSG00000088256
ENSG00000115263
ENSG00000242252
ENSG00000148926
ENSG00000148660
ENSG00000167244
ENSG00000044090
ENSG00000183840
ENSG00000204389
ENSG00000138378
ENSG00000165409
ENSG00000186468
ENSG00000198899
ENSG00000113525
ENSG00000153827
ENSG00000127663
ENSG00000124782
ENSG00000123560
ENSG00000147408
ENSG00000144891
ENSG00000187566
ENSG00000149541
ENSG00000226979
ENSG00000164953
ENSG00000157680
ENSG00000117020
ENSG00000166974

ENSG00000092529
ENSG00000138346
ENSG00000165370
ENSG00000164258
ENSG00000068438
ENSG00000102245
ENSG00000081479
ENSG00000169194
ENSG00000099889
ENSG00000129214
ENSG00000108175
ENSG00000075429
ENSG00000168038
ENSG00000152990
ENSG00000140538
ENSG00000049541
ENSG00000006704
ENSG00000106638
ENSG00000077800
ENSG00000106635
ENSG00000196275
ENSG00000176428
ENSG00000165171
ENSG00000175877
ENSG00000008086
ENSG00000067606
ENSG00000203618
ENSG00000140279
ENSG00000140274
ENSG00000112290
ENSG00000196876
ENSG00000185808
ENSG00000185950
ENSG00000197594
ENSG00000100888
ENSG00000176887
ENSG00000204655
ENSG00000170381
ENSG00000101384
ENSG00000169047
ENSG00000131080
ENSG00000123240
ENSG00000136051
ENSG00000140443

ENSG00000092820
ENSG00000240972
ENSG00000139567
ENSG00000198963
ENSG00000138152
ENSG00000135537
ENSG00000111670
ENSG00000090932
ENSG00000182621
ENSG00000157933
ENSG00000187017
ENSG00000073464
ENSG00000142676
ENSG00000172638
ENSG00000148218
ENSG00000157766
ENSG00000169306
ENSG00000167693
ENSG00000164190
ENSG00000007541
ENSG00000130477
ENSG00000119655
ENSG00000169946
ENSG00000088538
ENSG00000164885
ENSG00000018625
ENSG00000155962
ENSG00000070010
ENSG00000126870
ENSG00000100084
ENSG00000121454
ENSG00000277586
ENSG00000143947
ENSG00000134243
ENSG00000198695
ENSG00000119899
ENSG00000169896
ENSG00000119888
ENSG00000077721
ENSG00000231852
ENSG00000260230
ENSG00000140471
ENSG00000126091
ENSG00000100285

ENSG00000171988
ENSG00000207604
ENSG00000169760
ENSG00000158186
ENSG00000149970
ENSG00000122691
ENSG00000160226
ENSG00000110395
ENSG00000150540
ENSG00000185231
ENSG00000047457
ENSG00000122406
ENSG00000110619
ENSG00000115317
ENSG00000183421
ENSG00000117525
ENSG00000244734
ENSG00000077264
ENSG00000110801
ENSG00000179029
ENSG00000173531
ENSG00000142173
ENSG00000142156
ENSG00000113594
ENSG00000172071
ENSG00000109323
ENSG00000102030
ENSG00000130449
ENSG00000105983
ENSG00000163635
ENSG00000172115
ENSG00000101152
ENSG00000087245
ENSG00000178467
ENSG00000103494
ENSG00000078328
ENSG00000101935
ENSG00000122970
ENSG00000181027
ENSG00000196517
ENSG00000122126
ENSG00000162928
ENSG00000174405
ENSG00000186090

ENSG00000130294
ENSG00000161999
ENSG00000106003
ENSG00000148346
ENSG00000111860
ENSG00000116062
ENSG00000141385
ENSG00000102359
ENSG00000140691
ENSG00000207691
ENSG00000118137
ENSG00000106462
ENSG00000172534
ENSG00000158445
ENSG00000198910
ENSG00000162885
ENSG00000087116
ENSG00000229315
ENSG00000183765
ENSG00000140015
ENSG00000075624
ENSG00000114378
ENSG00000171206
ENSG00000171302
ENSG00000073734
ENSG00000104313
ENSG00000211448
ENSG00000097007
ENSG00000160957
ENSG00000119969
ENSG00000010610
ENSG00000090776
ENSG00000116830
ENSG00000104973
ENSG00000143919
ENSG00000121067
ENSG00000182040
ENSG00000120899
ENSG00000073584
ENSG00000133884
ENSG00000138075
ENSG00000168575
ENSG00000115665
ENSG00000080819

ENSG00000124299
ENSG00000117984
ENSG00000162747
ENSG00000284032
ENSG00000148795
ENSG00000115604
ENSG00000115310
ENSG00000141867
ENSG00000119042
ENSG00000000419
ENSG00000119878
ENSG00000284770
ENSG00000114026
ENSG00000244486
ENSG00000124164
ENSG00000283733
ENSG00000162999
ENSG00000176986
ENSG00000066117
ENSG00000113013
ENSG00000075213
ENSG00000116711
ENSG00000138207
ENSG00000119333
ENSG00000183770
ENSG00000173786
ENSG00000186153
ENSG00000136425
ENSG00000214274
ENSG00000242110
ENSG00000103313
ENSG00000154310
ENSG00000104763
ENSG00000124493
ENSG00000175707
ENSG00000135677
ENSG00000027847
ENSG00000160202
ENSG00000087258
ENSG00000116096
ENSG00000283904
ENSG00000198707
ENSG00000168610
ENSG00000196505

ENSG00000118785
ENSG00000147044
ENSG00000188167
ENSG00000055955
ENSG00000108733
ENSG00000037280
ENSG00000187486
ENSG00000170558
ENSG00000105854
ENSG00000026025
ENSG00000125813
ENSG00000109063
ENSG00000159140
ENSG00000184640
ENSG00000203877
ENSG00000005381
ENSG00000120457
ENSG00000168056
ENSG00000162065
ENSG00000162738
ENSG00000144724
ENSG00000160791
ENSG00000118873
ENSG00000134824
ENSG00000064601
ENSG00000164047
ENSG00000141736
ENSG00000106018
ENSG00000156298
ENSG00000139197
ENSG00000165752
ENSG00000148180
ENSG00000005471
ENSG00000178623
ENSG00000213614
ENSG00000174705
ENSG00000143199
ENSG00000138411
ENSG00000141448
ENSG00000177156
ENSG00000141646
ENSG00000130158
ENSG00000138036
ENSG00000153936

ENSG00000103510
ENSG00000160563
ENSG00000038002
ENSG00000189283
ENSG00000119977
ENSG00000163581
ENSG00000007908
ENSG00000053918
ENSG00000184304
ENSG00000196811
ENSG00000138061
ENSG00000125618
ENSG00000130427
ENSG00000220205
ENSG00000069424
ENSG00000189409
ENSG00000162493
ENSG00000130939
ENSG00000130940
ENSG00000169641
ENSG00000038382
ENSG00000207933
ENSG00000081923
ENSG00000140379
ENSG00000123080
ENSG00000013619
ENSG00000138079
ENSG00000112210
ENSG00000125107
ENSG00000100714
ENSG00000113790
ENSG00000077080
ENSG00000112357
ENSG00000140287
ENSG00000178235
ENSG00000284121
ENSG00000136573
ENSG00000135925
ENSG00000125798
ENSG00000197971
ENSG00000154153
ENSG00000158290
ENSG00000197859
ENSG00000117713

ENSG00000134780
ENSG00000173638
ENSG00000146938
ENSG00000162631
ENSG00000182871
ENSG00000124614
ENSG00000118004
ENSG00000112685
ENSG00000108559
ENSG00000105429
ENSG00000105771
ENSG00000204394
ENSG00000143761
ENSG00000143226
ENSG00000102466
ENSG00000148019
ENSG00000116106
ENSG00000189108
ENSG00000149311
ENSG00000170458
ENSG00000103051
ENSG00000105369
ENSG00000101052
ENSG00000166035
ENSG00000116678
ENSG00000142166
ENSG00000217930
ENSG00000111674
ENSG00000188095
ENSG00000167281
ENSG00000159899
ENSG00000119681
ENSG00000171453
ENSG00000109576
ENSG00000117139
ENSG00000100722
ENSG00000116918
ENSG00000169679
ENSG00000137462
ENSG00000121481
ENSG00000185651
ENSG00000107562
ENSG00000257529
ENSG00000235590

ENSG00000105647
ENSG00000159423
ENSG00000158352
ENSG00000172260
ENSG00000185324
ENSG00000163535
ENSG00000156709
ENSG00000131269
ENSG00000169184
ENSG00000170099
ENSG00000009709
ENSG00000112414
ENSG00000127980
ENSG00000160223
ENSG00000197779
ENSG00000128944
ENSG00000077498
ENSG00000184160
ENSG00000077279
ENSG00000198929
ENSG00000160882
ENSG00000138435
ENSG00000135902
ENSG00000187323
ENSG00000138759
ENSG00000164066
ENSG00000112144
ENSG00000112640
ENSG00000134595
ENSG00000112531
ENSG00000137801
ENSG00000117322
ENSG00000128654
ENSG00000070081
ENSG00000113889
ENSG00000147955
ENSG00000204390
ENSG00000136535
ENSG00000204388
ENSG00000007062
ENSG00000170374
ENSG00000072803
ENSG00000104907
ENSG00000136518

ENSG00000068885
ENSG00000204525
ENSG00000170606
ENSG00000145632
ENSG00000121351
ENSG00000198198
ENSG00000135406
ENSG00000113851
ENSG00000064933
ENSG00000082175
ENSG00000135218
ENSG00000196338
ENSG00000277632
ENSG00000100604
ENSG00000196365
ENSG00000167996
ENSG00000224389
ENSG00000163625
ENSG00000108510
ENSG00000100354
ENSG00000170921
ENSG00000006071
ENSG00000111640
ENSG00000165915
ENSG00000185960
ENSG00000168778
ENSG00000112062
ENSG00000038427
ENSG00000131779
ENSG00000135317
ENSG00000101076
ENSG00000128604
ENSG00000115159
ENSG00000197170
ENSG00000123908
ENSG00000131263
ENSG00000103005
ENSG00000029153
ENSG00000123384
ENSG00000116745
ENSG00000268066
ENSG00000213638
ENSG00000182774
ENSG00000182899

ENSG00000073578
ENSG00000171855
ENSG00000104447
ENSG00000115318
ENSG00000046651
ENSG00000163394
ENSG00000147124
ENSG00000176387
ENSG00000101751
ENSG00000071539
ENSG00000011143
ENSG00000163359
ENSG00000074266
ENSG00000173614
ENSG00000123095
ENSG00000205084
ENSG00000140374
ENSG00000154122
ENSG00000187535
ENSG00000168280
ENSG00000153064
ENSG00000081014
ENSG00000139174
ENSG00000110092
ENSG00000182568
ENSG00000012963
ENSG00000179111
ENSG00000131238
ENSG00000120054
ENSG00000261371
ENSG00000108797
ENSG00000020129
ENSG00000128591
ENSG00000044574
ENSG00000064687
ENSG00000112280
ENSG00000072274
ENSG00000163932
ENSG00000152223
ENSG00000115457
ENSG00000183762
ENSG00000105379
ENSG00000179869
ENSG00000164756

ENSG00000104368
ENSG00000083799
ENSG00000119772
ENSG00000188379
ENSG00000119711
ENSG00000188994
ENSG00000143799
ENSG00000033122
ENSG00000128039
ENSG00000134504
ENSG00000161533
ENSG00000197249
ENSG00000188404
ENSG00000172732
ENSG00000166747
ENSG00000138160
ENSG00000136944
ENSG00000143324
ENSG00000144535
ENSG00000116133
ENSG00000150893
ENSG00000158526
ENSG00000135775
ENSG00000148459
ENSG00000120868
ENSG00000196743
ENSG00000171320
ENSG00000099139
ENSG00000058866
ENSG00000135341
ENSG00000186951
ENSG00000154473
ENSG00000111799
ENSG00000137090
ENSG00000100526
ENSG00000138032
ENSG00000144354
ENSG00000110063
ENSG00000137760
ENSG00000160710
ENSG00000174720
ENSG00000067704
ENSG00000157911
ENSG00000115839

ENSG00000175535
ENSG00000085999
ENSG00000095380
ENSG00000137948
ENSG00000128335
ENSG00000057468
ENSG00000106785
ENSG00000100336
ENSG00000120210
ENSG00000165029
ENSG00000080345
ENSG00000143614
ENSG00000198732
ENSG00000091127
ENSG00000083067
ENSG00000136997
ENSG00000092200
ENSG00000068120
ENSG00000105516
ENSG00000112799
ENSG00000089225
ENSG00000169764
ENSG00000129675
ENSG00000009765
ENSG00000101901
ENSG00000130283
ENSG00000188257
ENSG00000072694
ENSG00000174080
ENSG00000198677
ENSG00000128891
ENSG00000063169
ENSG00000123561
ENSG00000204020
ENSG00000131408
ENSG00000144452
ENSG00000162688
ENSG00000144893
ENSG00000065883
ENSG00000132906
ENSG00000095951
ENSG00000172053
ENSG00000070831
ENSG00000029534

ENSG00000009413
ENSG00000197603
ENSG00000149782
ENSG00000120885
ENSG00000089639
ENSG00000164180
ENSG00000108784
ENSG00000164342
ENSG00000177731
ENSG00000164751
ENSG00000101464
ENSG00000151247
ENSG00000072682
ENSG00000070614
ENSG00000187764
ENSG00000196189
ENSG00000122359
ENSG00000105852
ENSG00000099797
ENSG00000169372
ENSG00000164022
ENSG00000112282
ENSG00000270647
ENSG00000104723
ENSG00000164099
ENSG00000155816
ENSG00000145979
ENSG00000110344
ENSG00000125505
ENSG00000135845
ENSG00000109775
ENSG00000175544
ENSG00000136319
ENSG00000165525
ENSG00000234616
ENSG00000031698
ENSG00000047621
ENSG00000101746
ENSG00000100412
ENSG00000158125
ENSG00000134371
ENSG00000100836
ENSG00000171532
ENSG00000143727

ENSG00000137309
ENSG00000116906
ENSG00000166313
ENSG00000233620
ENSG00000145808
ENSG00000115919
ENSG00000104899
ENSG00000149021
ENSG00000130066
ENSG00000137033
ENSG00000049768
ENSG00000197102
ENSG00000112530
ENSG00000257923
ENSG00000093072
ENSG00000177084
ENSG00000107187
ENSG00000147180
ENSG00000162735
ENSG00000150630
ENSG00000116786
ENSG00000117118
ENSG00000117245
ENSG00000121905
ENSG00000132763
ENSG00000117450
ENSG00000121940
ENSG00000116473
ENSG00000171385
ENSG00000116874
ENSG00000134250
ENSG00000265107
ENSG00000185499
ENSG00000163568
ENSG00000213088
ENSG00000177807
ENSG00000198821
ENSG00000162813
ENSG00000143801
ENSG00000221823
ENSG00000187605
ENSG00000162951
ENSG00000115486
ENSG00000237412

ENSG00000072756
ENSG00000182533
ENSG00000160808
ENSG00000010256
ENSG00000164061
ENSG00000114854
ENSG00000177646
ENSG00000177565
ENSG00000127241
ENSG00000109132
ENSG00000132464
ENSG00000151790
ENSG00000131844
ENSG00000132842
ENSG00000061492
ENSG00000146006
ENSG00000243232
ENSG00000124785
ENSG00000168394
ENSG00000096093
ENSG00000079841
ENSG00000168830
ENSG00000106341
ENSG00000058404
ENSG00000164742
ENSG00000106153
ENSG00000183638
ENSG00000131931
ENSG00000104321
ENSG00000182197
ENSG00000184374
ENSG00000178814
ENSG00000165060
ENSG00000196305
ENSG00000070061
ENSG00000148408
ENSG00000177283
ENSG00000108176
ENSG00000107521
ENSG00000014919
ENSG00000156395

Supplementary Table S4: Network analysis results of depression

Network Analysis Results for targets of depression

name	AverageShortest PathLength	Betweenness Centrality	Closeness Centrality	Clustering Coefficient	Degree
TP53	2.64510914	0.09478941	0.37805623	0.06731985	113
EP300	2.6523848	0.05607056	0.3770192	0.10060606	100
CTNNB1	2.64349232	0.06465836	0.37828746	0.08157895	96
PIK3R1	2.74777688	0.01763209	0.36393057	0.16117216	91
PIK3CA	2.72190784	0.02173176	0.36738937	0.16434676	91
HRAS	2.74454325	0.02687518	0.36435935	0.16317671	89
MAPK1	2.75101051	0.01976236	0.36350279	0.14883721	86
MAPK3	2.75505255	0.01753599	0.36296948	0.15376197	86
STAT3	2.77041229	0.02476242	0.36095711	0.15253165	80
RPS27A	2.84236055	0.06752715	0.35182025	0.0691334	79
AKT1	2.66370251	0.04034818	0.3754173	0.12720613	78
PTPN11	2.87146322	0.01752892	0.3482545	0.19824561	76
CREBBP	2.71544058	0.03602726	0.36826436	0.12699	74
KRAS	2.80679062	0.01429623	0.3562788	0.20372671	70
RAC1	2.89814066	0.02085928	0.34504881	0.15364355	68
SOS1	2.91835085	0.00537091	0.34265928	0.26686508	64
NRAS	2.94017785	0.00885878	0.34011548	0.2202381	64
MAPK8	2.82538399	0.02066826	0.35393419	0.12544803	63
ESR1	2.74454325	0.03216523	0.36435935	0.15901639	61
JAK2	2.95149555	0.01048452	0.33881128	0.1954023	58
MAPK14	2.78334681	0.02122381	0.3592797	0.16298701	56
PLCG1	3.02586904	0.01209119	0.33048357	0.1023569	55
MYC	2.83993533	0.02827822	0.35212069	0.15373864	54
CREB1	2.79223929	0.01803185	0.3581355	0.16163265	50
TNF	2.90299111	0.02141706	0.34447229	0.16653061	50
DLG4	3.06871463	0.03489447	0.32586934	0.0952381	49
NR3C1	2.81083266	0.01544652	0.35576647	0.18351064	48
CDC42	2.98383185	0.01020045	0.33513953	0.14545455	45
EDN1	2.87873888	0.01102177	0.34737433	0.24040404	45
CBL	3.03880356	0.00443653	0.32907688	0.24041812	42
VEGFA	2.91915926	0.01353345	0.34256439	0.21829268	41
TRAF6	2.89652385	0.01441227	0.34524142	0.18170732	41
IL6	3.11802749	0.00829684	0.32071558	0.28414634	41
IL2	3.06709782	0.00400162	0.32604112	0.3474359	40
FOS	2.91673403	0.00843844	0.34284922	0.18461538	40
IRS1	3.00485044	0.00357652	0.33279526	0.36842105	39
MAP2K1	3.05173808	0.00537688	0.32768212	0.33333333	39
SMAD4	3.01293452	0.00868524	0.33190233	0.18623482	39
AR	2.85125303	0.01419024	0.35072299	0.13900135	39
IL10	3.21584479	0.00307447	0.31096028	0.2802276	38
SMARCA	3.10105093	0.0092791	0.32247132	0.21906117	38

4					
GSK3B	2.98625707	0.01746441	0.33486735	0.11411411	37
PTEN	2.96362167	0.0128744	0.33742499	0.15615616	37
BRCA1	2.99919159	0.02196946	0.33342318	0.1981982	37
GNAI2	2.96523848	0.02012831	0.337241	0.18730159	36
INS	3.01778496	0.0230941	0.33136887	0.18730159	36
MECP2	3.12611156	0.01966216	0.31988622	0.07777778	36
PRKACA	3.04203719	0.02284317	0.32872708	0.07731092	35
CXCL12	2.98221504	0.00465285	0.33532123	0.31764706	35
HIF1A	2.91592563	0.01076788	0.34294428	0.22352941	35
CCND1	2.98706548	0.00456369	0.33477673	0.27807487	34
CRKL	3.19401778	0.00122759	0.31308529	0.39204545	33
EGF	3.02586904	0.00624584	0.33048357	0.28219697	33
GNAS	3.34518998	0.01524188	0.29893668	0.1344697	33
PRKCZ	2.99191593	0.0050332	0.33423399	0.30113636	33
ERBB2	2.98625707	0.0045635	0.33486735	0.31060606	33
HSPA4	3.0113177	0.0177547	0.33208054	0.10416667	33
IGF1	2.99838319	0.01218833	0.33351308	0.28219697	33
IL1B	3.07113985	0.00397137	0.325612	0.29032258	32
PPARA	3.065481	0.00879349	0.32621308	0.20564516	32
NGF	3.00485044	0.00403036	0.33279526	0.31451613	32
PPARGC1A	2.97089733	0.01356752	0.33659864	0.19758065	32
LEP	3.14874697	0.02012708	0.31758665	0.12903226	32
RAF1	3.01697656	0.00746998	0.33145766	0.25806452	32
CD4	3.18997575	0.00760326	0.31348201	0.17741935	32
HDAC2	3.05820534	0.00878561	0.32698916	0.17137097	32
RUNX2	2.98383185	0.01516063	0.33513953	0.18494624	31
AGT	3.07437348	0.00552028	0.32526952	0.28172043	31
CAMK2B	3.11721908	0.00707013	0.32079876	0.18064516	31
ERBB4	3.12045271	0.00538421	0.32046632	0.27956989	31
BDNF	2.93209378	0.00880372	0.34105321	0.36344086	31
GRIN2B	3.11560226	0.0078957	0.32096523	0.2344086	31
ATM	3.18916734	0.0164228	0.31356147	0.22988506	30
NGFR	2.97170574	0.0203777	0.33650707	0.25287356	30
ACTB	3.12287793	0.00789727	0.32021745	0.18390805	30
F2	3.14551334	0.01104594	0.31791313	0.22528736	30
IL2RA	3.12287793	9.01E-04	0.32021745	0.47126437	30
PPP2R5D	3.043654	0.00905886	0.32855246	0.15632184	30
IGF1R	3.01697656	0.00320145	0.33145766	0.40229885	30
ARRB2	3.09539208	0.0144293	0.32306085	0.13054187	29
GRIA2	3.26596605	0.00520765	0.30618812	0.24630542	29
CAMK2A	3.17784964	0.00555149	0.3146782	0.21182266	29
ABL1	3.06224737	0.00319787	0.32655755	0.21693122	28

CASP3	3.04203719	0.00438009	0.32872708	0.15343915	28
MAP2K2	3.16168149	8.14E-04	0.31628739	0.48677249	28
IL2RB	3.13338723	7.16E-04	0.31914345	0.54761905	28
MTOR	3.14551334	0.00703158	0.31791313	0.20899471	28
IL4	3.23848019	0.00933227	0.30878682	0.29100529	28
APOA1	3.1851253	0.01025801	0.31395939	0.21652422	27
KAT5	3	0.00720913	0.33333333	0.16524217	27
GNAO1	3.08084074	0.00438755	0.32458672	0.28774929	27
YWHAH	3.12449475	0.00452491	0.32005175	0.23361823	27
FGF2	3.20856912	0.00450822	0.31166541	0.32763533	27
CDKN1A	3.00565885	0.00182014	0.33270576	0.31384615	26
APP	3.07033145	0.01060156	0.32569774	0.13538462	26
PRKCD	2.99595796	0.00436774	0.33378305	0.27384615	26
GLI2	3.18270008	0.0166738	0.31419863	0.17538462	26
POMC	3.3807599	0.01327482	0.29579149	0.23384615	26
SNCA	3.17704123	0.01791515	0.31475827	0.10769231	26
PTK2B	3.1851253	0.00478196	0.31395939	0.17538462	26
AGO1	3.19482619	0.00479639	0.31300607	0.20666667	25
TGFB1	3.07679871	0.00757388	0.32501314	0.28666667	25
EZH2	3.08569119	0.00416726	0.3240765	0.25333333	25
VAMP2	3.27405012	0.01716261	0.3054321	0.17666667	25
IRS2	3.24252223	4.09E-04	0.30840189	0.51666667	25
MMP2	3.32497979	0.00530719	0.30075371	0.30797101	24
STX1A	3.55133387	0.01398838	0.28158434	0.1557971	24
CDKN2A	3.09458367	0.00410622	0.32314525	0.25	24
CXCL8	3.12853678	0.00276644	0.31963824	0.35507246	24
LPL	3.2287793	0.00742413	0.30971457	0.23188406	24
MAPT	3.109135	0.00516918	0.32163287	0.14492754	24
SMARCD1	3.28536783	0.00372162	0.30437992	0.40942029	24
DLG1	3.17219078	0.00495456	0.31523955	0.23188406	24
SIRT1	3.13257882	0.00402853	0.31922581	0.22826087	24
MAP3K7	3.24656427	0.01218312	0.30801793	0.13405797	24
LIF	3.34518998	0.00186752	0.29893668	0.44565217	24
CCR5	3.35569927	0.00320204	0.29800048	0.27272727	23
ACTA1	3.1738076	0.00345397	0.31507896	0.23320158	23
ARID1A	3.27890057	0.00204102	0.30498028	0.43873518	23
THBS1	3.32255457	0.01063885	0.30097324	0.09486166	23
PPARG	3.01293452	0.0109479	0.33190233	0.15810277	23
CAMK2G	3.26919968	0.00128955	0.30588526	0.28063241	23
SORT1	3.14389652	0.0142193	0.31807663	0.15810277	23
GLI3	3.13581245	0.01692894	0.31889662	0.20158103	23
LRP1	3.27485853	0.00835059	0.3053567	0.08225108	22
ALB	3.3257882	0.01182121	0.3006806	0.09090909	22

APOE	3.36135812	0.00461231	0.2974988	0.17748918	22
MEF2C	3.12126112	0.00261195	0.32038332	0.22510823	22
SMARCB1	3.35246564	0.00134302	0.29828792	0.43722944	22
SMARCE1	3.43734842	0.00250121	0.29092192	0.45887446	22
ITGAM	3.49312854	0.00572506	0.28627632	0.05627706	22
IFNG	3.22554568	9.48E-04	0.31002506	0.33766234	22
TNFRSF1A	3.17299919	0.00304868	0.31515924	0.2034632	22
TNRC6B	3.21099434	0.00267326	0.31143001	0.24761905	21
PDGFRB	3.15763945	4.37E-04	0.31669227	0.53333333	21
PDGFB	3.13581245	6.01E-04	0.31889662	0.51428571	21
GNA11	3.20291027	0.00400186	0.31221605	0.21428571	21
DVL1	3.14227971	0.00739022	0.31824029	0.27142857	21
CYCS	3.37186742	0.01203503	0.29657157	0.14285714	21
NTRK2	3.10670978	0.00203713	0.32188394	0.42857143	21
NRG1	3.18593371	0.00107668	0.31387973	0.56190476	21
HSPA5	3.27970897	0.006542	0.3049051	0.05789474	20
MMP9	3.34438157	0.00588138	0.29900894	0.41052632	20
EHHADH	3.27243331	0.00802255	0.305583	0.33157895	20
EZR	3.35246564	0.00505817	0.29828792	0.12631579	20
IL1A	3.36701698	0.00171286	0.2969988	0.42631579	20
ADRBK1	3.34033953	0.0038555	0.29937076	0.21052632	20
TLR4	3.13823767	0.00393299	0.31865018	0.2	20
GPC3	3.47210994	0.00946033	0.28800931	0.1	20
FGFR3	3.2497979	3.17E-04	0.30771144	0.46842105	20
IRAK1	3.18189167	0.00331495	0.31427846	0.28421053	20
IL17A	3.33548909	5.57E-04	0.29980611	0.55263158	20
FGFR1	3.23120453	0.00310462	0.30948211	0.33684211	20
SQSTM1	3.23201293	0.00400372	0.3094047	0.26315789	20
CDKN1B	3.15683104	0.00251181	0.31677337	0.26315789	19
CDK5	3.11802749	0.0050172	0.32071558	0.13450292	19
EDNRA	3.25303153	0.00126879	0.30740557	0.33333333	19
ADRB2	3.37025061	0.00794393	0.29671384	0.30409357	19
SIN3A	3.20533549	0.00449114	0.31197982	0.15789474	19
HSPA1A	3.0759903	0.00259209	0.32509855	0.22807018	19
ARNTL	3.41067098	0.00248596	0.29319744	0.38011696	19
CLOCK	3.45109135	0.00418711	0.28976341	0.33918129	19
FGFR2	3.27809216	3.77E-04	0.30505549	0.51461988	19
AGO2	3.26354082	0.00396699	0.30641566	0.24836601	18
TBL1XR1	3.24009701	0.0034415	0.30863273	0.07843137	18
NR1H3	3.35408246	0.00398139	0.29814413	0.36601307	18
ACOX1	3.35650768	0.0098635	0.29792871	0.40522876	18

NOS2	3.12853678	0.0088006	0.31963824	0.32026144	18
NOS3	3.06224737	0.00616682	0.32655755	0.18300654	18
ACTL6A	3.62732417	6.34E-04	0.27568531	0.4248366	18
PSEN1	3.2392886	0.00245415	0.30870976	0.15686275	18
PRKAR1A	3.26434923	0.00206842	0.30633977	0.26797386	18
KMT2D	3.25707357	0.00206442	0.30702408	0.25490196	18
STAT4	3.26192401	0.00614147	0.30656753	0.09150327	18
PIK3R2	3.27647534	2.15E-04	0.30520602	0.47058824	18
ERBB3	3.20452708	2.15E-04	0.31205853	0.64052288	18
CSNK1E	3.24575586	0.00395336	0.30809465	0.2875817	18
WNT5A	3.2611156	0.00429097	0.30664353	0.22222222	18
SUFU	3.32902183	0.0068	0.30038854	0.25490196	18
VCP	3.34357316	0.0095703	0.29908124	0.11764706	18
PARK2	3.31042846	0.00866531	0.3020757	0.23529412	18
HSPG2	3.61681487	0.00571545	0.27648637	0.09803922	18
RAP1A	3.19967664	7.37E-04	0.31253158	0.37908497	18
SNAP25	3.60630558	0.00298474	0.27729209	0.32026144	18
GRIN1	3.39450283	0.00183839	0.29459395	0.39869281	18
EPO	3.09377526	0.00128001	0.32322968	0.32679739	18
CD247	3.40339531	9.96E-04	0.29382423	0.35947712	18
IL18	3.36216653	4.99E-04	0.29742727	0.48366013	18
IL13	3.44462409	0.00404036	0.29030744	0.32679739	18
BLK	3.23039612	0.00337713	0.30955956	0.22058824	17
CAT	3.4130962	0.01092467	0.2929891	0.24264706	17
PAK3	3.27728375	0.0016796	0.30513074	0.34558824	17
IRF5	3.37186742	0.00279346	0.29657157	0.44852941	17
IFNB1	3.2821342	0.00114106	0.3046798	0.41176471	17
HTT	3.07275667	0.00722718	0.32544067	0.14705882	17
BCL2	3.0541633	0.00304849	0.32742192	0.34558824	17
AKT3	3.16248989	6.71E-04	0.31620654	0.24264706	17
NPAS2	3.43411479	0.0018203	0.29119586	0.38235294	17
GRIN2A	3.51333872	5.66E-04	0.28462954	0.36764706	17
RPL11	3.40016168	0.01438098	0.29410366	0.44117647	17
HLA-DRB1	3.44704931	0.0056133	0.29010319	0.28676471	17
FOXP3	3.23524656	0.00143719	0.30909545	0.27941176	17
CDH2	3.15278901	0.00140102	0.31717949	0.3	16
LRP8	3.31689572	0.00384515	0.30148672	0.16666667	16
MMP1	3.43249798	4.60E-04	0.29133302	0.61666667	16
CCL2	3.4672595	2.92E-04	0.28841222	0.58333333	16
HSD17B4	3.37671787	0.00594255	0.29614556	0.5	16
GATA4	3.33468068	0.00442039	0.29987879	0.225	16
NF1	3.19805982	0.00263975	0.31268959	0.3	16
ADIPOQ	3.32093775	0.00275862	0.30111977	0.29166667	16

INSR	3.21907842	2.16E-04	0.31064792	0.59166667	16
LRP2	3.56911884	0.00468222	0.2801812	0.11666667	16
GRIA3	3.68229588	3.60E-04	0.2715697	0.4	16
GRIA1	3.68229588	3.60E-04	0.2715697	0.4	16
STUB1	3.16006467	0.00179324	0.31644922	0.24166667	16
FLNA	3.28375101	0.00465181	0.30452979	0.09166667	16
MSH2	3.44219887	0.00298879	0.29051198	0.325	16
SMC3	3.63217462	0.00269203	0.27531716	0.35	16
HDAC6	3.11398545	0.00926009	0.32113188	0.20833333	16
VCAN	3.81649151	0.00554692	0.26202076	0.19166667	16
EFNB1	3.27566694	0.00137401	0.30528134	0.28333333	16
SHH	3.37267583	0.00548859	0.29650048	0.2	16
CD36	3.30719483	0.0027239	0.30237106	0.23333333	16
CCND2	3.33953112	4.19E-04	0.29944323	0.35238095	15
AGTR2	3.37510105	6.86E-04	0.29628743	0.52380952	15
GHRL	3.63864188	0.00587226	0.27482782	0.17142857	15
TBP	3.12368634	0.00124866	0.32013458	0.3047619	15
IRF6	3.47534357	0.00174592	0.28774134	0.57142857	15
HLA-A	3.55537591	0.00332768	0.28126421	0.35238095	15
PRKCE	3.39369442	4.64E-04	0.29466413	0.37142857	15
PARP1	3.20856912	0.00317779	0.31166541	0.22857143	15
GRIA4	3.68310428	3.60E-04	0.2715101	0.31428571	15
FZD2	3.27809216	0.00434941	0.30505549	0.25714286	15
DVL3	3.21422797	0.0013045	0.3111167	0.34285714	15
NBN	3.40501213	8.25E-04	0.29368471	0.46666667	15
PARK7	3.30638642	0.00590531	0.30244499	0.15238095	15
NTF3	3.3257882	4.47E-05	0.3006806	0.76190476	15
GDNF	3.1843169	4.94E-04	0.3140391	0.55238095	15
MT-CO2	3.48019402	0.01171803	0.2873403	0.36190476	15
MAOA	4.03556993	0.00705021	0.24779647	0.14285714	14
DDC	4.23362975	0.00294298	0.23620393	0.23076923	14
CYP3A4	4.03637833	0.00196959	0.24774685	0.2967033	14
PEX5	3.87873888	0.01375385	0.25781576	0.40659341	14
DMD	3.5982215	0.00480123	0.27791508	0.24175824	14
GAPDH	3.13581245	0.01083868	0.31889662	0.10989011	14
TCF7L2	3.20452708	6.23E-04	0.31205853	0.28571429	14
PRKCG	3.39935327	0.0014541	0.2941736	0.21978022	14
EIF4E	3.46402587	0.00421352	0.28868145	0.18681319	14
MYOD1	3.25060631	0.00164695	0.30763492	0.28571429	14
GRIP1	3.32255457	0.00290555	0.30097324	0.3956044	14
TLE1	3.2279709	0.00107252	0.30979214	0.32967033	14
PER2	3.44058205	9.97E-04	0.2906485	0.51648352	14
TIMELESS	3.87227162	0.00130024	0.25824635	0.27472527	14

RET	3.19725141	8.06E-04	0.31276865	0.49450549	14
SMPD1	3.4567502	0.00531169	0.28928906	0.35164835	14
RNF2	3.34923201	5.74E-04	0.29857591	0.32967033	14
POLE	3.7825384	0.00127133	0.26437273	0.28571429	14
MUS81	3.78577203	0.003516	0.26414691	0.28571429	14
MRE11A	3.39450283	9.38E-04	0.29459395	0.47252747	14
GPC4	4.00485044	0.00101312	0.24969721	0.15384615	14
GPC6	4.00485044	0.00101312	0.24969721	0.15384615	14
NTRK3	3.30638642	1.90E-04	0.30244499	0.62637363	14
RPS23	3.80759903	0.00192798	0.2626327	0.65934066	14
CASK	3.75990299	0.0032992	0.26596431	0.25274725	14
IL6R	3.3589329	3.61E-04	0.2977136	0.50549451	14
TBK1	3.32336297	0.00113097	0.30090002	0.28571429	14
LCN2	3.51253032	5.92E-04	0.28469505	0.72527473	14
BCR	3.35489086	1.71E-04	0.29807229	0.53846154	13
HSPA9	3.36297494	0.00559293	0.29735577	0.11538462	13
TRRAP	3.21988682	0.00105526	0.31056992	0.28205128	13
NOTCH3	3.57073565	0.00326117	0.28005434	0.1025641	13
ADCY1	3.44139046	3.45E-04	0.29058022	0.61538462	13
ADCY7	3.41390461	6.56E-04	0.29291973	0.56410256	13
GNAL	3.55133387	0.00133928	0.28158434	0.43589744	13
CYP2E1	4.10509297	0.00182979	0.24359984	0.29487179	13
DRD2	3.53031528	0.0072547	0.28326082	0.05128205	13
KMT2A	3.46968472	4.23E-04	0.28821062	0.35897436	13
TSC2	3.27324171	3.37E-04	0.30550753	0.41025641	13
SOD1	3.31042846	0.00372835	0.3020757	0.14102564	13
ITPR1	3.28940986	0.00419426	0.3040059	0.03846154	13
NOS1	3.38641876	0.00243051	0.29529721	0.16666667	13
APOB	3.49232013	0.00196792	0.28634259	0.24358974	13
GCG	3.33144705	0.00445567	0.30016986	0.20512821	13
PICK1	3.75101051	0.00225382	0.26659483	0.30769231	13
APC	3.32012935	3.83E-04	0.30119308	0.35897436	13
TFAP2A	3.15521423	0.00208964	0.31693569	0.25641026	13
DCTN1	3.49717057	0.00616933	0.28594545	0.16666667	13
RORA	3.31204527	6.15E-04	0.30192824	0.41025641	13
CRY2	3.52465643	7.40E-04	0.2837156	0.61538462	13
ERCC1	3.80032336	1.40E-04	0.2631355	0.43589744	13
MSH6	3.87388844	4.20E-04	0.25813856	0.41025641	13
SMC1A	3.64995958	0.0022783	0.27397564	0.44871795	13
BRCA2	3.37105901	7.14E-04	0.29664269	0.48717949	13
BRAF	3.42279709	3.81E-04	0.29215872	0.64102564	13
CACNG2	3.7720291	1.36E-04	0.2651093	0.58974359	13
CALM2	3.60711399	0.01178014	0.27722994	0.1025641	13
HDAC4	3.31204527	0.00155609	0.30192824	0.21794872	13

RPS20	3.80921584	0.00156769	0.26252122	0.74358974	13
CCL5	3.52061439	1.72E-04	0.28404133	0.71794872	13
PSMD12	3.37105901	0.00112502	0.29664269	0.35897436	13
TLR2	3.2829426	0.0025531	0.30460478	0.29487179	13
ICAM1	3.49393694	0.00127003	0.28621009	0.21794872	13
PRKCI	3.20937753	0.00139691	0.3115869	0.41025641	13
P4HA2	4.53839935	0.00624746	0.220342	0.16666667	13
PGR	3.19725141	0.00131424	0.31276865	0.37179487	13
DLG3	3.55780113	2.41E-04	0.28107248	0.43589744	13
RPL5	3.47534357	0.00174839	0.28774134	0.69230769	13
MAOB	4.09458367	0.0047502	0.24422507	0.15151515	12
ABCA1	3.3476152	9.91E-04	0.29872012	0.28787879	12
TAF1	3.29587712	3.66E-04	0.30340937	0.42424242	12
SMARCA2	3.92481811	1.19E-04	0.25478888	0.75757576	12
NOTCH2	3.72029103	0.0018699	0.26879618	0.15151515	12
COL1A2	3.96362167	0.00697652	0.25229451	0.21212121	12
ADCY5	3.6628941	2.30E-04	0.27300817	0.65151515	12
CACNA1C	3.66046888	0.00515617	0.27318905	0.27272727	12
ARF1	3.4025869	0.0029211	0.29389404	0.12121212	12
AP3B1	3.4785772	0.00296469	0.28747386	0.27272727	12
FKBP4	3.3476152	0.00154517	0.29872012	0.5	12
CRY1	3.65723525	4.17E-04	0.27343059	0.71212121	12
ATF4	3.34033953	0.0021951	0.29937076	0.15151515	12
LRRK2	3.4349232	0.00191316	0.29112732	0.22727273	12
NCAN	4.28051738	9.24E-04	0.23361662	0.33333333	12
STXBP1	3.64995958	0.00368732	0.27397564	0.48484848	12
TAC1	3.86418755	0.00706948	0.25878661	0.13636364	12
PSMD9	3.39692805	7.03E-04	0.29438363	0.39393939	12
HNF1A	3.20776071	0.00169741	0.31174395	0.46969697	12
DNMT1	3.30072757	0.00368066	0.30296351	0.31818182	12
IGF2	3.31366209	0.00313976	0.30178092	0.12121212	12
HLA-DQA1	3.63298302	0.00334564	0.2752559	0.27272727	12
UQCRC1	4.15763945	8.39E-04	0.2405211	0.57575758	12
RPS19	3.7930477	1.00E-04	0.26364024	0.77272727	12
RPS17	3.8156831	6.26E-06	0.26207627	0.84848485	12
IDO1	4.50848828	0.00107729	0.22180384	0.25454545	11
RELN	3.28132579	3.76E-04	0.30475487	0.34545455	11
MT-ND6	4.42279709	0.00121073	0.22610126	0.65454545	11
FLT4	3.45917542	0.00249294	0.28908624	0.34545455	11
ADAM10	3.27647534	0.00274265	0.30520602	0.27272727	11
ADCY8	3.68067906	7.87E-05	0.271689	0.78181818	11

ADCY2	3.68067906	7.87E-05	0.271689	0.78181818	11
ALDH2	3.99353274	0.00483163	0.25040486	0.16363636	11
HTR2C	3.6200485	9.04E-04	0.27623939	0.69090909	11
PTCH1	3.47130154	0.0029216	0.28807639	0.30909091	11
GATA1	3.46887631	0.00162921	0.28827779	0.2	11
PLCB1	3.54890865	8.63E-04	0.28177677	0.32727273	11
TSC1	3.41875505	3.01E-04	0.29250414	0.4	11
CASP9	3.42118027	4.53E-04	0.29229679	0.32727273	11
PIK3CD	3.34842361	6.96E-05	0.298648	0.65454545	11
FKBP5	3.29991916	0.00158006	0.30303773	0.32727273	11
AP2S1	3.76232821	6.22E-04	0.26579287	0.36363636	11
DISC1	3.48504446	0.00594167	0.28694039	0.01818182	11
GRM5	3.46079224	0.0032228	0.28895118	0.18181818	11
DYNC1H1	3.61115602	0.00459443	0.27691963	0.25454545	11
NR1D1	3.55214228	4.68E-04	0.28152025	0.6	11
PER1	3.46968472	7.63E-04	0.28821062	0.61818182	11
CYP2C19	4.12287793	5.16E-04	0.24254902	0.41818182	11
EED	3.52465643	3.67E-04	0.2837156	0.38181818	11
HCFC1	3.59660469	0.00175689	0.27804001	0.23636364	11
MLH1	3.55860954	0.00287897	0.28100863	0.41818182	11
CHEK2	3.50121261	2.81E-04	0.28561533	0.54545455	11
TARDBP	3.89005659	0.00698974	0.25706567	0.14545455	11
MKS1	4.00404204	0.01043644	0.24974763	0.43636364	11
SST	3.50202102	0.00483375	0.2855494	0.23636364	11
NPY	3.91673403	0.00307175	0.25531476	0.25454545	11
CCL3	3.61358124	1.11E-05	0.27673378	0.89090909	11
DUSP1	3.2829426	1.99E-04	0.30460478	0.52727273	11
MUC1	3.18674212	0.0026439	0.3138001	0.29090909	11
DYNC2H1	3.99110752	0.00572699	0.25055702	0.34545455	11
FGF23	3.45351657	0.00117914	0.28955993	0.63636364	11
GH1	3.38722716	7.19E-04	0.29522673	0.50909091	11
IL1R1	3.42603072	2.71E-04	0.29188296	0.43636364	11
RPL35A	3.81891673	8.90E-07	0.26185436	0.90909091	11
RPS24	3.81649151	4.44E-06	0.26202076	0.90909091	11
AGTR1	3.48989491	0.0011074	0.28654158	0.2	10
KAT8	3.50444624	2.11E-04	0.28535179	0.46666667	10
ARID1B	4.06305578	5.60E-07	0.24612018	0.93333333	10
VDR	3.38561035	3.07E-04	0.29536772	0.62222222	10
DPF2	4.06305578	6.10E-05	0.24612018	0.8	10
ACTL6B	4.00646726	2.41E-05	0.24959645	0.73333333	10
MED12	3.45755861	0.00109325	0.28922142	0.64444444	10
MED13	3.86257074	5.61E-04	0.25889494	0.68888889	10
UBQLN2	3.46483428	0.00501477	0.28861409	0.06666667	10
HDAC5	3.52384802	4.40E-04	0.28378068	0.2	10

OPRM1	3.50202102	0.00188582	0.2855494	0.17777778	10
KMT2C	3.3694422	3.29E-04	0.29678503	0.57777778	10
NUP88	3.63459984	0.00367709	0.27513345	0.08888889	10
CTSD	3.36054972	0.00436511	0.29757036	0.08888889	10
SERPINE1	3.59660469	0.00146951	0.27804001	0.15555556	10
NCF1	3.31447049	3.82E-04	0.30170732	0.37777778	10
CD40LG	3.25222312	4.10E-04	0.30748198	0.42222222	10
ROR2	3.48100243	3.75E-04	0.28727357	0.53333333	10
GFAP	3.46968472	0.00527352	0.28821062	0.04444444	10
CYP1A2	4.04446241	0.00320093	0.24725165	0.31111111	10
POR	3.59337106	0.00471747	0.27829021	0.31111111	10
DNA2	3.41471302	3.00E-04	0.29285038	0.71111111	10
BMP2	3.56911884	0.00193718	0.2801812	0.37777778	10
NKX2-5	3.79223929	2.46E-04	0.26369644	0.42222222	10
ETS1	3.12045271	2.88E-04	0.32046632	0.44444444	10
CCK	3.87469685	0.00242152	0.25808471	0.33333333	10
TWIST1	3.27405012	1.25E-04	0.3054321	0.57777778	10
TNFRSF1B	3.48585287	1.64E-04	0.28687384	0.42222222	10
CD14	3.30962005	0.00335806	0.30214949	0.33333333	10
MEN1	3.35165724	6.61E-04	0.29835986	0.28888889	10
PEX2	4.41147939	0.00170559	0.22668133	0.68888889	10
MT-CO3	4.18189167	4.04E-04	0.23912623	0.71111111	10
MT-CO1	4.18189167	4.04E-04	0.23912623	0.71111111	10
HMOX1	3.38884398	0.00472162	0.29508588	0.2	10
VCAM1	3.44381568	0.00107054	0.29037559	0.13333333	10
PTGS2	3.35489086	0.00640942	0.29807229	0.26666667	10
TH	3.48342765	0.01063064	0.28707357	0.2	10
IGFBP3	3.16814875	0.00121349	0.31564175	0.28888889	10
RAD21	3.76475344	0.00117004	0.26562164	0.55555556	10
ESR2	3.42926435	8.71E-04	0.29160773	0.24444444	10
NCAM1	3.5650768	0.00271713	0.28049887	0.24444444	10
PINK1	3.56426839	0.00265379	0.28056249	0.26666667	10
MT-ND5	4.42522231	1.42E-04	0.22597735	0.8	10
MT-ND4	4.42522231	1.42E-04	0.22597735	0.8	10
MT-ND1	4.42522231	1.42E-04	0.22597735	0.8	10
PEX12	4.8156831	5.40E-04	0.20765486	0.73333333	10
PEX13	4.8156831	5.40E-04	0.20765486	0.73333333	10
RPS10	3.81729992	3.56E-06	0.26196527	0.93333333	10
SOX9	3.36782538	0.00517396	0.29692751	0.19444444	9
GNPAT	3.89814066	0.00181756	0.25653256	0.66666667	9
RAPSN	3.94664511	4.94E-04	0.25337976	0.41666667	9
GJA1	3.32417138	6.17E-04	0.30082685	0.13888889	9

GLTSCR1	4.05012126	8.22E-06	0.24690619	0.80555556	9
TGFBR2	3.37510105	0.0010998	0.29628743	0.27777778	9
ADK	4.59013743	0.00714452	0.2178584	0.19444444	9
ADAMTS 2	4.22069523	8.71E-04	0.23692779	0.33333333	9
CAV3	3.7178658	0.00252286	0.26897152	0.08333333	9
MED13L	3.86337914	5.59E-04	0.25884076	0.69444444	9
MED27	3.86337914	5.59E-04	0.25884076	0.69444444	9
MED23	3.86337914	2.17E-05	0.25884076	0.80555556	9
AVP	3.96281326	0.00258726	0.25234598	0.11111111	9
HSP90B1	3.31204527	5.61E-04	0.30192824	0.22222222	9
APAF1	3.26596605	2.07E-04	0.30618812	0.63888889	9
CRP	3.46645109	0.00879942	0.28847948	0.11111111	9
WBSCR2 2	3.76394503	0.00331291	0.26567869	0.58333333	9
PIK3C2A	3.52708165	0.00127819	0.28352051	0.38888889	9
WNT8A	3.50282943	0.00194677	0.2854835	0.16666667	9
PRNP	3.5222312	0.00260575	0.28391095	0.11111111	9
BHLHE41	3.87954729	3.69E-05	0.25776203	0.75	9
ATXN3	3.46079224	8.46E-04	0.28895118	0.30555556	9
B4GALT7	4.30557801	1.12E-05	0.23225685	0.75	9
B3GAT3	4.30557801	1.55E-04	0.23225685	0.63888889	9
BAG1	3.50444624	2.26E-04	0.28535179	0.27777778	9
CD79A	3.48746968	1.03E-04	0.28674084	0.52777778	9
RIMS1	4.15117219	0.00175799	0.24089581	0.55555556	9
CACNB2	3.79547292	4.26E-04	0.26347178	0.47222222	9
NRXN1	3.97332255	0.00368238	0.25167854	0.16666667	9
EIF4G1	3.56426839	0.00213012	0.28056249	0.25	9
HLA-DQB 1	3.61681487	0.00153652	0.27648637	0.33333333	9
MUSK	3.70250606	0.00253982	0.27008734	0.33333333	9
CYLD	3.43249798	7.09E-04	0.29133302	0.55555556	9
CYP17A1	4.38237672	5.76E-04	0.22818668	0.44444444	9
IFT52	3.99272433	0.00398687	0.25045556	0.52777778	9
STAG2	3.77526273	0.00110606	0.26488223	0.66666667	9
FGF8	3.5004042	1.81E-04	0.28568129	0.72222222	9
GABRA1	1	0.61111111	1	0.36111111	9
GHR	3.45755861	8.24E-06	0.28922142	0.72222222	9
NEFL	3.56103476	0.00193698	0.28081725	0.19444444	9
IL5	3.40986257	3.05E-04	0.29326695	0.52777778	9
RPL36A-HNRNPH 2	3.82053355	0	0.26174355	1	9
PRDX1	3.40824576	6.09E-04	0.29340607	0.07142857	8

DAO	3.93856103	0.00161681	0.25389984	0.75	8
SPTAN1	3.50606306	2.08E-04	0.2852202	0.46428571	8
ADAR	4.11802749	8.39E-04	0.24283471	0.53571429	8
COL1A1	3.73160873	0.00658493	0.26798094	0.28571429	8
HLA-C	4.05335489	1.83E-04	0.24670921	0.67857143	8
VIP	4.32417138	0.00108878	0.23125818	0.25	8
COMT	4.18916734	0.00206445	0.23871092	0.32142857	8
APRT	4.69118836	0.00650152	0.2131656	0.25	8
KMT2B	3.42764753	2.04E-04	0.29174528	0.25	8
INPPL1	3.39773646	0.00287447	0.29431359	0.25	8
SOD2	3.44462409	7.03E-04	0.29030744	0.28571429	8
AP4E1	3.86499596	4.33E-04	0.25873248	0.42857143	8
CLU	3.76313662	4.52E-04	0.26573577	0.39285714	8
BRD4	3.33548909	8.04E-04	0.29980611	0.32142857	8
SPOP	3.46806791	0.00339895	0.28834499	0.28571429	8
XBP1	3.71139854	0.0050757	0.26944021	0.10714286	8
ATRIP	3.55375909	2.32E-04	0.28139217	0.57142857	8
B3GALT6	4.30638642	1.09E-05	0.23221325	0.75	8
CTLA4	3.57073565	0.00173606	0.28005434	0.35714286	8
BMP4	3.62489895	3.18E-04	0.27586976	0.53571429	8
PMS2	3.5982215	6.81E-04	0.27791508	0.57142857	8
CHD8	3.38237672	2.41E-04	0.2956501	0.5	8
GRM1	3.56184317	0.00161517	0.28075352	0.21428571	8
PPP3CC	3.58205335	5.58E-04	0.27916949	0.14285714	8
CARS	4.38399353	0.0112693	0.22810253	0.28571429	8
DCC	3.55699272	1.42E-04	0.28113636	0.25	8
CC2D2A	4.99514956	6.04E-06	0.20019421	0.71428571	8
TMEM67	4.99514956	6.04E-06	0.20019421	0.71428571	8
TMEM216	4.99595796	3.14E-06	0.20016181	0.75	8
PITX2	3.37833468	0.00165581	0.29600383	0.42857143	8
HNF4A	3.23605497	8.13E-05	0.30901824	0.71428571	8
CRH	3.8585287	0.00185001	0.25916614	0.28571429	8
CYP2B6	4.17138238	7.50E-04	0.23972868	0.57142857	8
DNMT3A	3.42118027	3.87E-04	0.29229679	0.42857143	8
DYNC2LI1	4.52061439	7.12E-04	0.22120887	0.5	8
KDM6A	3.60226354	1.09E-04	0.27760323	0.42857143	8
FGF17	3.55133387	5.80E-07	0.28158434	0.92857143	8
NDUFV2	4.25222312	0.00137819	0.2351711	0.60714286	8
PEX1	4.81729992	1.90E-07	0.20758517	0.96428571	8
PEX19	4.81729992	7.80E-07	0.20758517	0.89285714	8
PEX7	4.81729992	5.37E-04	0.20758517	0.82142857	8
PEX10	4.81729992	1.90E-07	0.20758517	0.96428571	8
PEX6	4.81729992	1.90E-07	0.20758517	0.96428571	8

A2M	3.57962813	5.16E-04	0.27935863	0.19047619	7
ASMT	4.4017785	5.09E-04	0.2271809	0.23809524	7
CDKN1C	3.57720291	8.69E-05	0.27954802	0.47619048	7
SDHB	4.30638642	6.04E-04	0.23221325	0.42857143	7
AMACR	3.93936944	0	0.25384773	1	7
ARHGEF6	3.78011318	4.11E-05	0.26454234	0.80952381	7
LIMK1	3.67097817	2.61E-04	0.27240696	0.33333333	7
GSN	3.4244139	2.70E-04	0.29202077	0.38095238	7
ACTG1	3.61681487	2.46E-04	0.27648637	0.33333333	7
ARID2	4.07113985	2.10E-07	0.24563145	0.95238095	7
BAZ1B	3.82215036	7.71E-05	0.26163283	0.71428571	7
HLA-B	4.065481	8.21E-05	0.24597335	0.80952381	7
DICER1	3.956346	6.37E-04	0.25275848	0.42857143	7
MED25	3.86499596	2.13E-05	0.25873248	0.76190476	7
TTR	3.75262732	0.00210719	0.26647997	0.23809524	7
S100B	3.34114794	8.29E-04	0.29929833	0.14285714	7
SETD1A	3.86337914	1.17E-04	0.25884076	0.19047619	7
VIM	3.29830234	7.05E-04	0.30318627	0.57142857	7
GAD1	3.76151981	0.00352474	0.26584999	0.38095238	7
BMPR1A	3.83912692	2.18E-04	0.26047589	0.71428571	7
SPTBN1	3.58852061	0.00745051	0.27866637	0.33333333	7
DNAJC6	3.78415521	0.01450709	0.26425977	0.28571429	7
OCRL	3.62247373	0.00133805	0.27605445	0.33333333	7
PSEN2	3.54244139	2.56E-04	0.28229119	0.23809524	7
AQP4	4.0323363	0.00498526	0.24799519	0	7
CRHR1	3.80113177	0.00234529	0.26307954	0.19047619	7
RFC2	3.56992724	1.93E-04	0.28011775	0.57142857	7
XYLT1	4.30719483	1.09E-05	0.23216967	0.66666667	7
SLC6A4	3.75505255	0.00524703	0.26630786	0	7
STMN1	3.4890865	7.12E-05	0.28660797	0.14285714	7
HDAC9	3.3694422	1.42E-04	0.29678503	0.28571429	7
CPLX1	4.18108327	8.20E-06	0.23917247	0.85714286	7
TMEM231	4.99676637	1.50E-06	0.20012943	0.85714286	7
TCTN2	4.99676637	1.50E-06	0.20012943	0.85714286	7
RPGRIP1L	4.00727567	0.00402438	0.2495461	0.28571429	7
CEP290	4.99595796	3.75E-06	0.20016181	0.80952381	7
IL1RN	3.71544058	2.09E-05	0.26914708	0.57142857	7
USH1C	5.88197251	0.00456452	0.170011	0.23809524	7
DFNB31	5.88197251	0.00456452	0.170011	0.23809524	7
PER3	3.99434115	1.33E-05	0.25035418	0.80952381	7
OPTN	3.76717866	5.63E-04	0.26545064	0.28571429	7
CYP11B1	4.56911884	2.60E-04	0.21886058	0.52380952	7

CYP19A1	3.67825384	0.00522504	0.27186813	0.42857143	7
CYP2C9	4.26677445	4.85E-04	0.23436908	0.66666667	7
RAB3GAP2	4.07356508	0.00114315	0.24548522	0.42857143	7
GRIN2C	3.75101051	1.12E-05	0.26659483	0.80952381	7
PRL	3.5545675	0.00275115	0.28132818	0.28571429	7
FAS	3.34357316	5.85E-04	0.29908124	0.28571429	7
LRRC7	3.64268391	8.95E-05	0.27452286	0.33333333	7
IFNAR1	3.62570736	2.00E-04	0.27580825	0.33333333	7
MT-ATP6	4.4672595	0	0.22385089	1	7
NDUFS4	4.94017785	1.40E-05	0.20242186	0.80952381	7
RSRC1	3.82215036	0	0.26163283	1	7
NR1H2	3.91996766	2.60E-04	0.25510415	0.4	6
TP63	3.32821342	6.89E-05	0.3004615	0.4	6
WASF1	3.55618432	7.79E-06	0.28120027	0.53333333	6
KNG1	3.83023444	6.96E-04	0.26108062	0.26666667	6
ACKR1	3.55295069	1.22E-04	0.2814562	0.4	6
FH	4.52546483	3.18E-04	0.22097178	0.2	6
CHRNA1	3.82053355	1.13E-04	0.26174355	0.66666667	6
PECAM1	3.36863379	6.05E-04	0.29685625	0.2	6
DLL1	3.9458367	0.00137663	0.25343167	0.4	6
OAS2	4.16976556	0	0.23982164	1	6
PDE4D	3.85286985	0.00636668	0.25954679	0.06666667	6
ENPP1	5.57881973	0.00808211	0.17924938	0.2	6
CALCA	4.55699272	3.39E-04	0.21944297	0.2	6
HP	3.92320129	4.38E-04	0.25489388	0.4	6
GLUD2	4.45513339	4.15E-04	0.22446017	0.4	6
GAD2	3.97170574	0.00171696	0.25178099	0.46666667	6
ANK3	4.49070331	0.00689794	0.22268227	0.13333333	6
TFRC	3.96200485	0.00183159	0.25239747	0.2	6
APBB1	3.3912692	1.65E-04	0.29487485	0.4	6
MAF	3.61843169	3.88E-04	0.27636282	0.06666667	6
CNR1	3.78658044	0.00508218	0.26409052	0.2	6
ASXL1	3.60226354	1.41E-04	0.27760323	0.53333333	6
RPS6KA3	3.55537591	3.91E-05	0.28126421	0.6	6
PHF21A	3.84559418	1.92E-04	0.26003784	0.66666667	6
HSPA1B	3.47938561	2.22E-04	0.28740706	0.6	6
FCGR2B	3.64349232	2.18E-04	0.27446195	0.06666667	6
BGLAP	3.87469685	6.07E-04	0.25808471	0.06666667	6
BUB1B	3.97978981	0.00753945	0.25126955	0.33333333	6
CUL7	3.47453517	7.53E-04	0.28780828	0.2	6
KIF11	4.41471302	0.00269937	0.22651529	0.13333333	6
POLG	3.61438965	0.00337164	0.27667189	0.26666667	6
DPYSL2	3.72675829	6.69E-04	0.26832972	0.26666667	6

SLC18A2	3.98383185	0.00120755	0.25101461	0.26666667	6
CASR	3.89894907	6.52E-04	0.25647937	0.13333333	6
CSF1R	3.42279709	8.06E-04	0.29215872	0.26666667	6
CDKN2B	3.68229588	6.52E-06	0.2715697	0.8	6
PDCD1	3.75262732	1.12E-05	0.26647997	0.73333333	6
FCGR3B	3.58852061	1.47E-04	0.27866637	0.4	6
PPP1R1B	3.69684721	9.74E-04	0.27050077	0.13333333	6
LMNB1	3.92320129	6.84E-04	0.25489388	0	6
SYP	3.66774454	0.00105056	0.27264712	0.13333333	6
CHMP2B	3.89490703	3.04E-04	0.25674554	0.33333333	6
GRN	3.65076799	6.73E-04	0.27391497	0.2	6
CYP21A2	4.84478577	1.16E-04	0.20640748	0.53333333	6
HSD11B1	3.94341148	0.00255162	0.25358754	0.2	6
CYP2D6	4.53516572	2.07E-04	0.22049911	0.46666667	6
DNMT3B	3.86742118	3.69E-04	0.25857023	0.6	6
SLC6A3	3.86499596	0.00127704	0.25873248	0.2	6
DUSP4	3.52950687	9.10E-07	0.2833257	0.93333333	6
DUSP6	3.52950687	9.10E-07	0.2833257	0.93333333	6
PRICKLE1	3.71624899	1.24E-04	0.26908854	0.53333333	6
VANGL2	4.05982215	3.90E-05	0.24631621	0.6	6
IFT80	4.92966855	1.05E-05	0.20285339	0.86666667	6
WDR35	4.92966855	1.05E-05	0.20285339	0.86666667	6
IFT140	4.92966855	1.05E-05	0.20285339	0.86666667	6
PLCB3	3.62894099	5.78E-05	0.27556249	0.66666667	6
SYNJ1	3.67097817	0.00132225	0.27240696	0	6
ESCO2	4.59013743	1.75E-04	0.2178584	0.66666667	6
NIPBL	4.6192401	1.74E-06	0.21648582	0.93333333	6
PLAT	3.74050121	0.00338814	0.26734385	0.2	6
FGF20	3.55860954	0	0.28100863	1	6
SPR	4.45594179	0.00371391	0.22441945	0.06666667	6
TAC3	4.66936136	2.42E-04	0.21416205	0.26666667	6
MAG	3.90784155	0.01643471	0.25589574	0.26666667	6
TSPAN7	4.07033145	1.70E-07	0.24568024	0.86666667	6
PLA2G4A	3.56345998	9.43E-05	0.28062613	0.66666667	6
TNFAIP3	3.49474535	1.11E-04	0.28614388	0.8	6
STAG3	4.62732417	2.60E-07	0.21610762	0.93333333	6
PIGA	1.25	0.53571429	0.8	0.66666667	6
AANAT	4.70169766	3.10E-05	0.21268913	0.6	5
ABCB1	3.3047696	0.00167669	0.30259295	0.6	5
ACAN	4.04122878	2.12E-04	0.24744949	0.3	5
ACE	4	2.53E-05	0.25	0.6	5
ACO2	4.31204527	2.58E-04	0.23190851	0.4	5
SDHA	4.32174616	1.91E-04	0.23138795	0.6	5

ADA	4.24818108	0.00312302	0.23539486	0.1	5
DPP4	3.4244139	0.00450667	0.29202077	0.1	5
JAG1	4.19401778	4.44E-04	0.23843485	0.6	5
ADAMTS L2	4.3047696	1.45E-04	0.23230047	0.6	5
COL11A2	4.77041229	2.46E-05	0.20962549	0.8	5
COL2A1	4.30800323	9.59E-04	0.2321261	0.5	5
COL11A1	4.77041229	2.46E-05	0.20962549	0.8	5
ADH1B	4.58528698	9.55E-05	0.21808886	0.6	5
PDE4B	3.87712207	0.00550005	0.25792327	0.1	5
IAPP	3.79143088	0.0023355	0.26375267	0.4	5
DGCR8	3.51010509	2.10E-04	0.28489175	0.7	5
FMR1	3.98544867	1.97E-04	0.25091278	0.7	5
QARS	5.37833468	8.11E-04	0.18593116	0.5	5
IARS	5.37914309	8.08E-04	0.18590322	0.5	5
CP	4.05820534	0.00188888	0.24641434	0.1	5
ALDH4A1	4.65076799	8.01E-04	0.21501825	0.6	5
ALK	3.52708165	2.81E-06	0.28352051	0.6	5
AMH	4.20048504	0.00162321	0.23806774	0.4	5
PON1	3.99676637	4.60E-05	0.25020227	0.6	5
RTN4R	3.59337106	0.00165567	0.27829021	0.4	5
ATRX	3.44058205	4.79E-04	0.2906485	0.2	5
GBA	3.97493937	0.00943246	0.25157616	0.1	5
EIF2AK3	3.71624899	2.45E-04	0.26908854	0.3	5
LIG4	4.11560226	0	0.2429778	1	5
ATXN2	4.10428456	4.16E-04	0.24364782	0.2	5
OXT	4.25949879	9.12E-04	0.23476941	0.1	5
CNTF	3.68310428	4.14E-04	0.2715101	0.1	5
PTH	4.21422797	4.26E-04	0.23729139	0	5
SPP1	3.74130962	0.00145898	0.26728608	0.2	5
EPHA4	3.63379143	3.62E-05	0.27519466	0.4	5
PPP2R2B	3.78577203	0	0.26414691	1	5
BUB1	4.9135004	0.00165045	0.2035209	0.4	5
C4A	4.45917542	0.00645287	0.22425671	0.4	5
CACNA1 A	3.76798707	3.42E-04	0.26539369	0	5
TRPV1	4.58447858	0.00178794	0.21812731	0.2	5
QKI	3.64349232	1.48E-05	0.27446195	0.6	5
SYN1	3.84963622	2.89E-04	0.2597648	0.6	5
SYN2	4.18755053	1.79E-06	0.23880309	0.9	5
GPX1	3.7938561	3.16E-04	0.26358406	0.6	5
CDKN2C	3.55860954	3.67E-05	0.28100863	0.7	5
PTPN22	4.09620049	1.12E-05	0.24412868	0.6	5
SELL	3.90299111	5.62E-04	0.25621375	0.2	5

CDON	3.869038	1.78E-04	0.25846218	0.5	5
CDH23	6.87712207	5.10E-07	0.14540966	0.8	5
MYO7A	6.87712207	5.10E-07	0.14540966	0.8	5
HBB	4.11560226	2.21E-04	0.2429778	0.1	5
VPS37D	3.95311237	1.77E-04	0.25296524	0.4	5
STX16	4.24171382	0.00323101	0.23575376	0.6	5
COL18A1	4.02586904	0.00180882	0.24839357	0.1	5
COL6A1	4.60792239	6.42E-05	0.21701754	0.8	5
DBH	4.30153597	8.01E-05	0.2324751	0.6	5
FDFT1	3.62570736	0.0037739	0.27580825	0.4	5
UCN	4.41956346	8.86E-05	0.22626669	0.6	5
CRHR2	4.17704123	3.40E-04	0.23940391	0.4	5
FBXW11	3.41067098	1.30E-04	0.29319744	0.4	5
TBX5	3.50929669	1.16E-04	0.28495738	0.5	5
TNIK	3.42198868	3.71E-04	0.29222773	0.2	5
GLB1	4.8698464	0.01183185	0.20534529	0.2	5
TPO	4.1301536	8.25E-04	0.24212175	0.3	5
SRD5A3	4.65400162	5.01E-05	0.21486886	0.9	5
SRD5A1	4.65400162	5.01E-05	0.21486886	0.9	5
NR3C2	4.11883589	0.00149098	0.24278705	0.4	5
TPH1	4.59094584	4.22E-04	0.21782004	0.3	5
TPH2	4.59094584	4.22E-04	0.21782004	0.3	5
TDO2	4.94987874	1.15E-04	0.20202515	0.6	5
GRIK2	3.97251415	6.49E-05	0.25172975	0.3	5
SYNGAP1	3.61358124	0	0.27673378	1	5
SLC17A7	3.79062247	0.00324177	0.26380891	0	5
HOMER1	3.88439774	2.38E-04	0.25744017	0.3	5
SHANK2	4	4.05E-04	0.25	0.4	5
FZD8	3.68310428	4.41E-04	0.2715101	0.2	5
ELN	4.25303153	0.00335641	0.2351264	0.1	5
VWF	3.83023444	0.00188892	0.26108062	0	5
GATAD2B	3.38237672	3.74E-05	0.2956501	0.8	5
PAX6	3.45998383	0.00156688	0.28901869	0.2	5
EXT1	4.37025061	6.04E-06	0.22881983	0.4	5
EXT2	4.37025061	6.04E-06	0.22881983	0.4	5
GABRA4	1.44444444	0	0.69230769	1	5
GABRA6	1.44444444	0	0.69230769	1	5
GABRA5	1.44444444	0	0.69230769	1	5
GABRA3	1.44444444	0	0.69230769	1	5
GABRA2	1.44444444	0	0.69230769	1	5
HEXA	5.5432498	0.00832433	0.18039959	0.2	5
GNRH1	4.87873888	2.21E-04	0.204971	0.2	5
TRH	4.3912692	0.00203685	0.22772459	0	5

HS6ST1	4.37025061	6.04E-06	0.22881983	0.4	5
HS2ST1	4.37025061	6.04E-06	0.22881983	0.4	5
PRKD1	3.73322555	3.48E-05	0.26786488	0.6	5
HDAC8	3.94017785	4.01E-04	0.25379565	0.6	5
MBP	3.5974131	0.00308445	0.27797753	0.1	5
TLR3	3.57316087	3.01E-05	0.27986425	0.6	5
LTA	3.74130962	7.90E-07	0.26728608	0.8	5
IL18R1	3.49232013	6.22E-05	0.28634259	0.3	5
INPP1	4.28375101	0.00323685	0.23344027	0.1	5
PEX11B	4.81972514	0	0.20748071	1	5
PIGQ	1.625	0	0.61538462	1	5
PIGL	1.625	0	0.61538462	1	5
PIGC	1.625	0	0.61538462	1	5
PIGY	1.625	0	0.61538462	1	5
PIGP	1.625	0	0.61538462	1	5
SGOL2	4.62813258	0	0.21606987	1	5
KYNU	4.35004042	0.00247066	0.22988292	0.16666667	4
ST3GAL3	4.01455133	1.50E-04	0.24909384	0	4
REN	4.00080841	1.60E-05	0.24994948	0.83333333	4
CUL4B	3.91107518	6.22E-05	0.25568417	0.33333333	4
ADAMTS 19	4.30800323	0	0.2321261	1	4
B3GALTL	4.30800323	0	0.2321261	1	4
FBN1	4.61277284	1.64E-04	0.21678934	0.16666667	4
RETN	3.60064673	7.42E-05	0.27772789	0.33333333	4
MLXIPL	3.67663703	1.04E-04	0.27198769	0.33333333	4
PDE4A	4.15036378	0.00231477	0.24094273	0.16666667	4
ADRBK2	3.91754244	6.07E-05	0.25526207	0.66666667	4
AGER	3.86742118	2.02E-04	0.25857023	0	4
KMT2E	4.03880356	2.26E-05	0.24759808	0.66666667	4
USP8	3.57477769	1.19E-04	0.27973768	0	4
MST1	3.52869846	1.76E-04	0.28339061	0.33333333	4
GPT	3.96038804	0.0013162	0.25250051	0.16666667	4
SHBG	3.6952304	5.15E-04	0.27061912	0.33333333	4
GLUL	4.66531932	0	0.2143476	1	4
SCN2A	4.58043654	0.00336564	0.2183198	0.16666667	4
KCNQ2	5.28860146	0.00130646	0.18908591	0.33333333	4
MPO	4.01697656	3.46E-04	0.24894345	0.16666667	4
ARC	3.57962813	0.00172908	0.27935863	0.16666667	4
TRIP11	3.67097817	2.18E-05	0.27240696	0.5	4
SUMF1	7.83427648	0.00328939	0.12764421	0	4
CYP1B1	4.86499596	4.86E-05	0.20555002	0.16666667	4
ATF3	3.5004042	5.00E-05	0.28568129	0.16666667	4
TTC5	3.38641876	4.99E-05	0.29529721	0.66666667	4

EYA1	3.89409863	3.61E-06	0.25679884	0.66666667	4
FUS	4.29749394	4.39E-04	0.23269375	0.5	4
HSPA1L	3.55780113	1.85E-04	0.28107248	0.16666667	4
POLG2	4.48423605	3.92E-05	0.22300343	0.66666667	4
C4B	5.45594179	1.31E-06	0.18328641	0.66666667	4
RYR1	4.12934519	4.62E-05	0.24216915	0.33333333	4
CACNA1B	4.17623282	3.25E-04	0.23945025	0.16666667	4
GNB3	3.91996766	9.17E-05	0.25510415	0.33333333	4
PVALB	3.99110752	0.0016549	0.25055702	0.33333333	4
CAMKMT	5.23524656	0.00161681	0.19101297	0.5	4
PPM1B	4.2392886	0.00645156	0.23588863	0.5	4
UBE2L3	3.63459984	1.68E-04	0.27513345	0.16666667	4
MTR	1	0.83333333	1	0.16666667	4
TCTN3	4.99919159	0	0.20003234	1	4
NTS	4.01212611	0.0014608	0.24924441	0.16666667	4
PF4	3.78738884	2.87E-05	0.26403415	0.5	4
ITIH4	3.88197251	1.44E-04	0.257601	0.5	4
ERVW-1	4.043654	0.00161681	0.24730108	0.5	4
PCDH15	6.87793048	1.90E-07	0.14539257	0.83333333	4
USH1G	6.87793048	1.90E-07	0.14539257	0.83333333	4
KDM5B	3.75747777	6.19E-05	0.26613597	0.16666667	4
PCNT	4.20533549	2.15E-04	0.23779316	0.16666667	4
LIFR	4.07194826	0.00323489	0.24558269	0.33333333	4
KCNA1	4.44300728	0.00397896	0.22507278	0.16666667	4
COL6A2	4.70412288	4.71E-05	0.21257948	0.83333333	4
COL6A3	4.93775263	1.72E-05	0.20252128	0.83333333	4
CPLX2	4.24413905	0.00161681	0.23561905	0.5	4
CRADD	3.81325788	0	0.26224295	1	4
MADD	3.81325788	0	0.26224295	1	4
IFNA2	3.42522231	9.63E-05	0.29195185	0.33333333	4
HMGCR	3.62813258	3.80E-06	0.27562389	0.66666667	4
CRHBP	4.59983832	3.78E-06	0.21739895	0.83333333	4
TCF4	3.53759095	9.10E-06	0.28267824	0.5	4
CTSA	4.39046079	0.0039242	0.22776653	0.33333333	4
PSAP	3.89571544	4.42E-04	0.25669226	0.33333333	4
EHMT1	3.48423605	2.25E-04	0.28700696	0.16666667	4
PPP3R1	3.88682296	1.63E-04	0.25727953	0.33333333	4
RAB3GAP1	4.41067098	1.71E-04	0.22672287	0.5	4
TYR	4.42522231	3.74E-04	0.22597735	0.66666667	4
GPR98	4.89248181	0.01446192	0.20439524	0	4
LBR	3.96847211	3.54E-04	0.25198615	0	4
GNS	4.73726758	0.01283435	0.21109215	0.16666667	4

SIGMAR1	3.94826192	3.47E-04	0.253276	0	4
WDR34	4.52465643	1.65E-04	0.22101126	0.66666667	4
EDA	4.24090542	4.25E-04	0.2357987	0.16666667	4
TFAP2B	3.83023444	1.66E-04	0.26108062	0.16666667	4
KDM4B	3.46968472	0.00171884	0.28821062	0.16666667	4
FAN1	4.49393694	8.03E-06	0.22252204	0.33333333	4
PMS1	4.43330639	9.63E-06	0.22556528	0.66666667	4
VEGFC	3.64753436	0	0.2741578	1	4
KDM6B	3.50282943	2.41E-05	0.2854835	0.16666667	4
HCRT	4.38884398	0.00195798	0.22785043	0.16666667	4
GMNN	3.94341148	3.30E-04	0.25358754	0.16666667	4
KISS1	4.12934519	0.00283506	0.24216915	0.33333333	4
NDST1	4.37105901	6.04E-06	0.22877751	0	4
PDYN	3.86580437	2.95E-04	0.25867838	0.16666667	4
TGIF1	3.7720291	4.28E-05	0.2651093	0.66666667	4
UBE2A	3.52950687	8.83E-04	0.2833257	0.33333333	4
TAF15	3.59660469	0.00173966	0.27804001	0.33333333	4
IFT81	4.9894907	0	0.20042126	1	4
LEPR	3.51333872	2.54E-05	0.28462954	0.83333333	4
MC4R	4.02586904	1.46E-05	0.24839357	0.66666667	4
VPS35	3.89409863	1.14E-04	0.25679884	0.5	4
WWC1	3.81891673	2.74E-04	0.26185436	0	4
TMEM107	5.00080841	0	0.19996767	1	4
MLH3	4.43330639	1.55E-05	0.22556528	0.66666667	4
NPSR1	4.39369442	0.00161681	0.2275989	0.5	4
PIP4K2A	3.67744543	7.02E-05	0.2719279	0.16666667	4
REV3L	4.35165724	2.13E-05	0.22979751	0.5	4
SNAP29	4.24333064	0	0.23566394	1	4
IARS2	5.3799515	0.00161681	0.18587528	0.33333333	3
ABCB4	3.63136621	3.80E-06	0.27537845	0.33333333	3
ABCC8	3.912692	3.91E-04	0.25557851	0.33333333	3
KCNJ11	4.00646726	0.00161681	0.24959645	0.33333333	3
PDSS1	4.60953921	0.00220759	0.21694142	0	3
ACHE	3.9135004	5.16E-04	0.25552572	0	3
CHAT	4.23443816	9.74E-04	0.23615884	0	3
SLC25A4	4.11479386	6.90E-05	0.24302554	0.33333333	3
ALDH6A1	4.20129345	3.53E-05	0.23802194	0.66666667	3
ACSL4	4.35246564	0.00323232	0.22975483	0	3
PFN1	3.92158448	5.52E-06	0.25499897	0.33333333	3
ADCYAP1	4.33387227	8.63E-04	0.23074053	0.33333333	3
ADCYAP1 R1	4.3039612	1.14E-04	0.2323441	0.33333333	3
ADH1C	4.81891673	0	0.20751552	1	3
PDE1B	4.56345998	0.00129192	0.21913198	0.33333333	3

ADRB1	3.84236055	3.00E-04	0.26025668	0.33333333	3
NCS1	4.16491512	5.78E-05	0.24010093	0.33333333	3
FOXA2	3.60388036	0.00161681	0.27747869	0.33333333	3
TBC1D7	3.65400162	0	0.27367257	1	3
ALAD	1	0	1	1	3
CPOX	1	0	1	1	3
HMBS	1	0	1	1	3
PPOX	1	0	1	1	3
DMRT1	4.20210186	0	0.23797614	1	3
TF	4.043654	2.69E-04	0.24730108	0	3
LIPC	4.04203719	0	0.2474	1	3
TREM2	4.13500404	2.33E-05	0.24183773	0.33333333	3
IQSEC1	3.84559418	2.49E-05	0.26003784	0	3
DBP	4.37833468	0	0.22839734	1	3
PTH1R	3.94502829	2.37E-04	0.25348361	0	3
GLA	5.85529507	0.00633628	0.17078559	0.33333333	3
ARSB	7.7065481	1.61E-04	0.12975978	0.33333333	3
HYAL1	6.71624899	0.00331752	0.14889263	0.33333333	3
IGFBP1	3.8480194	4.04E-05	0.25987395	0	3
ATP2A2	4.30800323	2.38E-04	0.2321261	0	3
DEPDC5	4.14227971	8.08E-04	0.24141296	0.66666667	3
SZT2	4.14227971	8.08E-04	0.24141296	0.66666667	3
FANCL	3.99434115	2.72E-06	0.25035418	0.66666667	3
ATXN7	3.68229588	1.34E-05	0.2715697	0.33333333	3
OXTR	4.34276475	2.25E-04	0.23026806	0	3
CSGALN ACT1	4.7825384	4.80E-06	0.20909398	0.66666667	3
TDGF1	4.043654	0.00162943	0.24730108	0	3
CRBN	4.03637833	4.24E-06	0.24774685	0.33333333	3
UNC13A	4.62894099	1.23E-04	0.21603213	0.66666667	3
MAD1L1	4.97655618	0	0.20094217	1	3
BUB3	4.97655618	0	0.20094217	1	3
MASP1	5.4567502	0	0.18325926	1	3
COLEC11	5.4567502	0	0.18325926	1	3
C9orf72	4.88682296	0.00161681	0.20463193	0.33333333	3
KCNQ1	4.57316087	0.00166993	0.21866714	0	3
SLC3A1	5.23605497	0	0.19098348	1	3
PREPL	5.23605497	0	0.19098348	1	3
VARS	5.3807599	0	0.18584736	1	3
CBLN1	4.79143088	0.00165753	0.20870592	0	3
PYY	4.25303153	0	0.2351264	1	3
CLIP1	3.81729992	1.56E-04	0.26196527	0.33333333	3
HELLS	4.29749394	0.00161681	0.23269375	0.33333333	3
NDEL1	3.99514956	1.16E-04	0.25030352	0	3

CDKL5	3.75181892	2.03E-04	0.26653738	0.33333333	3
HUWE1	3.76313662	6.30E-05	0.26573577	0.33333333	3
GAS1	4.3257882	0	0.23117174	1	3
SLC18A3	4.63459984	0.00171215	0.21576836	0	3
CHGA	4.36216653	5.86E-05	0.22924388	0.33333333	3
ENO2	4.04769604	2.52E-04	0.24705412	0.33333333	3
CHRNA2	1	0	1	1	3
CHRNB2	1	0	1	1	3
CHRNA4	1	0	1	1	3
CHRNA7	1	0	1	1	3
CHRNG	4.14227971	0	0.24141296	1	3
CNOT1	4.3257882	0	0.23117174	1	3
CNOT3	4.3257882	0	0.23117174	1	3
CNTNAP2	4.91754244	0.00161681	0.20335361	0.33333333	3
LGI1	4.03395311	0.00364594	0.24789579	0.33333333	3
COX15	4.4777688	0	0.22332551	1	3
FXN	4.06790622	1.56E-04	0.24582671	0.33333333	3
FADS1	3.63136621	3.80E-06	0.27537845	0.33333333	3
FCGR2A	3.73160873	3.48E-04	0.26798094	0	3
USP9X	3.61115602	0.00161681	0.27691963	0.33333333	3
CTSF	4.42926435	2.60E-07	0.22577113	0.66666667	3
HSD11B2	5.05497171	1.10E-04	0.19782504	0.33333333	3
FMO3	4.97574778	4.40E-07	0.20097482	0.66666667	3
PAH	5.22716249	1.26E-05	0.19130838	0.33333333	3
USH2A	6.87793048	4.77E-06	0.14539257	0	3
DHCR24	3.61843169	0.00350541	0.27636282	0	3
NLGN4X	4.04284559	0	0.24735053	1	3
NLGN1	3.88197251	3.92E-04	0.257601	0.33333333	3
DNAJC5	3.82134196	2.38E-04	0.26168817	0.33333333	3
SEMA3A	3.92239289	2.80E-05	0.25494641	0.33333333	3
DRD1	4.09458367	6.20E-05	0.24422507	0.33333333	3
WDR60	4.93290218	0	0.20272042	1	3
EDARADD	3.89248181	0.00131765	0.2569055	0.33333333	3
PRDM16	3.82780922	2.23E-05	0.26124604	0.33333333	3
FOXH1	3.6200485	3.50E-05	0.27623939	0.66666667	3
NHLRC1	3.74130962	2.36E-05	0.26728608	0.33333333	3
ETFA	7.53354891	0.00323101	0.13273956	0.33333333	3
TALDO1	3.91430881	6.07E-04	0.25547295	0	3
MDH1	4.25949879	4.63E-04	0.23476941	0	3
MAMLD1	3.74777688	1.57E-04	0.26682485	0	3
FOXG1	3.94260307	1.10E-05	0.25363953	0.33333333	3
NSUN2	1	0.66666667	1	0.33333333	3
SFRP1	4.21827001	3.20E-07	0.23706401	0.66666667	3

SOST	4.18674212	4.71E-06	0.2388492	0	3
GABRB3	1.66666667	0.01388889	0.6	0.66666667	3
GABRG2	1.66666667	0.01388889	0.6	0.66666667	3
GHRH	4.39854487	6.43E-06	0.22734791	0.66666667	3
INTU	4.01697656	0	0.24894345	1	3
OFD1	4.01697656	0	0.24894345	1	3
MC1R	3.68795473	3.18E-04	0.271153	0	3
TSHR	3.90784155	7.36E-04	0.25589574	0	3
GNPTAB	3.73403395	0	0.26780688	1	3
HTR2A	4.30800323	1.38E-04	0.2321261	0	3
SKI	3.82295877	0	0.2615775	1	3
HNMT	4.78738884	0.00162287	0.20888213	0	3
SELP	4.25303153	5.13E-05	0.2351264	0.33333333	3
SELE	4.00323363	3.65E-06	0.24979806	0.66666667	3
RTN4	3.94341148	0	0.25358754	1	3
MED12L	4.86014551	0	0.20575516	1	3
MSH4	4.49717057	6.51E-06	0.22236203	0.33333333	3
PEX5L	5.81325788	0	0.17202058	1	3
PIGT	1.625	0.42857143	0.61538462	0.33333333	3
TSHB	4.33791431	0.00326402	0.23052553	0	3
UCHL1	3.40824576	2.25E-04	0.29340607	0.33333333	3
TACR1	4.33063864	4.34E-04	0.23091282	0.33333333	3
ABCB11	4.96443007	0.00161681	0.20143299	0	2
NR1H4	3.9668553	0.00323101	0.25208885	0	2
ABCG5	4.35246564	0	0.22975483	1	2
ACAD9	5.29426031	4.79E-05	0.1888838	0	2
ACVRL1	4.05497171	0	0.24661085	1	2
ADORA1	3.934519	5.65E-04	0.25416067	0	2
CECR1	5.11236863	0	0.19560405	1	2
VIPR2	5.10751819	1.28E-05	0.19578981	0	2
CDH13	4.04769604	3.82E-05	0.24705412	0	2
PDE11A	5.58771221	0	0.17896412	1	2
ADM	4.75828618	0	0.2101597	1	2
ADORA2A	4.34357316	0	0.2302252	1	2
AGL	6.57477769	0.00323101	0.1520964	0	2
UGP2	7.57235247	0.00161681	0.13205936	0	2
TSNAX	4.08164915	1.76E-05	0.24499901	0	2
AIFM1	3.97574778	1.56E-06	0.25152501	0	2
AIMP1	6.37590946	0	0.15684037	1	2
SERPINA7	4.29426031	0	0.23286898	1	2
SERPINA1	4.31689572	0	0.23164794	1	2

SERPINA6	4.27000808	0	0.23419159	1	2
POX2	5.27809216	1.84E-04	0.1894624	0	2
ALG3	1	1	1	0	2
ALKBH8	4.76151981	0.00161681	0.21001698	0	2
ANK1	4.45836702	7.11E-04	0.22429737	0	2
SCN8A	5.48827809	0.00161681	0.18220651	0	2
KCNQ3	5.48827809	0	0.18220651	1	2
AP1G1	4.30153597	6.18E-05	0.2324751	0	2
APOD	4.35246564	0	0.22975483	1	2
CANT1	5.68552951	0	0.17588511	1	2
TRPV4	3.62651576	9.87E-04	0.27574677	0	2
ARNTL2	4.29264349	0	0.23295669	1	2
ARSA	6.84478577	0.00473473	0.14609661	0	2
IDUA	7.71059014	0	0.12969176	1	2
ARTN	3.90784155	0	0.25589574	1	2
ASAH1	4.35489086	0	0.22962688	1	2
ATP13A2	4.18674212	0	0.2388492	1	2
ATP6V1B2	5.14066289	0	0.19452744	1	2
ATXN1	3.82053355	2.23E-06	0.26174355	0	2
RBFOX1	4.09377526	1.51E-04	0.2442733	0	2
AVPR1A	4.79547292	2.99E-05	0.20853001	0	2
AVPR1B	4.63379143	2.96E-05	0.215806	0	2
BANK1	4.06224737	5.64E-06	0.24616915	0	2
BCL2A1	3.61843169	0	0.27636282	1	2
BCL7B	4.28375101	0	0.23344027	1	2
GGCX	4.09377526	7.45E-05	0.2442733	0	2
SP7	3.96847211	0	0.25198615	1	2
MRAS	4.01535974	0	0.24904369	1	2
BSN	5.14955538	0	0.19419152	1	2
TRIP13	4.91673403	0	0.20338704	1	2
C10orf2	4.61277284	0	0.21678934	1	2
COLEC10	5.45755861	0	0.18323211	1	2
KCNMA1	4.65804365	0.00161681	0.2146824	0	2
MARCKS	4.03071948	0	0.24809467	1	2
CAMP	4.13338723	0.00161681	0.24193233	0	2
SARS	5.38156831	0	0.18581944	1	2
KIF17	4.05658852	0	0.24651255	1	2
TBR1	4.51010509	7.00E-05	0.22172432	0	2
GABBR1	4.49878739	0	0.22228212	1	2
GSR	4.37752627	0	0.22843952	1	2
SGCE	4.45109135	0	0.224664	1	2
SPRY4	3.64915117	5.60E-07	0.27403633	0	2

NXN	4.12611156	0.00144852	0.24235893	0	2
LY86	4.10024252	0	0.24388801	1	2
DOCK6	3.83508488	0	0.26075042	1	2
CDC73	3.62328213	0.00272695	0.27599286	0	2
TBX2	4.06305578	1.50E-05	0.24612018	0	2
CHCHD10	4.88763137	0.00161681	0.20459808	0	2
CHST3	4.78334681	4.80E-06	0.20905864	0	2
WFS1	4.70897332	0.00161681	0.21236052	0	2
CLCF1	5.06952304	0.00161681	0.19725722	0	2
CLRN1	6.87954729	1.90E-07	0.1453584	0	2
FAAH	4.75747777	0	0.21019541	1	2
COASY	6.57720291	0	0.15204031	1	2
PANK2	6.57720291	0	0.15204031	1	2
COG4	5.2392886	0.00161681	0.19086561	0	2
SLC2A3	3.71544058	0	0.26914708	1	2
VGF	3.5432498	0	0.28222679	1	2
OBSL1	3.82295877	3.33E-05	0.2615775	0	2
HTRA2	4.15036378	3.76E-05	0.24094273	0	2
DHCR7	4.61600647	0.00161681	0.21663748	0	2
GSTM1	4.56669361	9.30E-05	0.21897681	0	2
TRIO	3.88358933	0	0.25749376	1	2
RRM2B	3.64268391	0.00161681	0.27452286	0	2
DKK4	4.27485853	3.20E-07	0.23392587	0	2
GRIK5	4.06224737	0	0.24616915	1	2
LRRTM2	4.05497171	0	0.24661085	1	2
NLGN3	4.05497171	0	0.24661085	1	2
NOS1AP	4.00565885	0	0.24964682	1	2
DLL3	4.94421989	0	0.20225638	1	2
DOK7	4.66208569	0	0.21449627	1	2
DPYSL5	3.96038804	0	0.25250051	1	2
DRD5	4.34357316	0	0.2302252	1	2
PRICKLE2	4.14066289	0	0.24150722	1	2
EDAR	4.82295877	0	0.2073416	1	2
EDA2R	3.89329022	8.93E-05	0.25685216	0	2
JARID2	4.08407437	0	0.24485352	1	2
F3	3.84721099	6.19E-06	0.25992856	0	2
EIF2B5	3.98383185	0.00161681	0.25101461	0	2
EIF4H	4.3265966	0	0.23112855	1	2
ENSP00000480012	4.06628941	1.06E-05	0.24592445	0	2
EPM2A	3.83993533	0	0.26042105	1	2
WWOX	3.54405821	1.32E-05	0.28216241	0	2

ERF	3.7388844	0	0.26745946	1	2
GRM2	3.73322555	4.67E-04	0.26786488	0	2
ETFB	8.53193209	0	0.11720675	1	2
GM2A	6.53759095	0.0048426	0.15296154	0	2
ETFDH	8.53193209	0	0.11720675	1	2
EXOC2	3.77283751	2.24E-05	0.2650525	0	2
MYCN	3.95472918	0	0.25286182	1	2
SLC12A2	4.33144705	3.20E-04	0.23086973	0	2
PDPN	3.81406629	1.03E-05	0.26218737	0	2
PODXL	4.29991916	0	0.23256251	1	2
HTR1A	4.13419563	3.67E-05	0.24188502	0	2
FTSJ1	1.33333333	0	0.75	1	2
TRMT1	1.33333333	0	0.75	1	2
KREMEN1	4.27485853	3.20E-07	0.23392587	0	2
GABRB2	1.77777778	0	0.5625	1	2
GABRB1	1.77777778	0	0.5625	1	2
ZFPM2	4.22716249	8.62E-06	0.23656531	0	2
GCH1	5.45432498	0	0.18334074	1	2
PTS	5.45432498	0	0.18334074	1	2
GLE1	4.63217462	0.00161681	0.21588133	0	2
RAB23	4.01778496	0	0.24889336	1	2
SIX3	4.39692805	1.85E-05	0.22743151	0	2
GNRHR	4.32417138	5.54E-04	0.23125818	0	2
KISS1R	5.0978173	0	0.19616239	1	2
GUSB	5.72675829	0.00491907	0.17461886	0	2
PDZD7	5.89005659	5.36E-04	0.16977766	0	2
KIF5C	3.83185125	3.54E-05	0.26097046	0	2
POLR1C	3.98383185	0.00161681	0.25101461	0	2
THAP1	4.46887631	4.57E-05	0.2237699	0	2
HIRA	3.63459984	1.56E-04	0.27513345	0	2
HMGA1	3.63459984	1.56E-04	0.27513345	0	2
HNRNPA1	4.50525465	0	0.22196304	1	2
S100A10	4.73807599	0.00161681	0.21105613	0	2
IFNA1	4.03476152	9.70E-07	0.24784612	0	2
IGFBP2	3.98059822	2.09E-06	0.25121852	0	2
IL1RAPL1	3.80598222	2.54E-04	0.26274427	0	2
IMPA1	5.2821342	0	0.18931742	1	2
IMPA2	5.2821342	0	0.18931742	1	2
KCNAB2	5.44058205	0.00161681	0.18380386	0	2
KCNB1	3.91430881	2.48E-04	0.25547295	0	2
SCN1A	6.28617623	1.09E-06	0.15907922	0	2
KCTD1	4.14227971	8.29E-06	0.24141296	0	2

KCTD17	4.46645109	0	0.2238914	1	2
KLHL7	4.46645109	0	0.2238914	1	2
PAX7	4.09296686	1.05E-06	0.24432155	0	2
L1CAM	4.5222312	0	0.22112978	1	2
LFNG	4.49797898	3.20E-07	0.22232207	0	2
POU1F1	5.33548909	0.00161681	0.18742424	0	2
SYNE1	4.56265158	1.73E-05	0.2191708	0	2
PCSK5	3.8156831	1.23E-05	0.26207627	0	2
TG	4.48261924	1.72E-04	0.22308386	0	2
PLP1	4.52869846	0	0.220814	1	2
SAT1	4.78900566	6.06E-06	0.20881161	0	2
OPRK1	4.09943411	2.84E-05	0.24393611	0	2
MTHFD1	1.5	0	0.66666667	1	2
MTHFR	1.5	0	0.66666667	1	2
RECQL4	4.6960388	1.05E-05	0.21294543	0	2
SORCS2	3.84236055	0	0.26025668	1	2
NMNAT1	4.10347615	1.61E-04	0.24369582	0	2
UBE4B	4.31204527	6.46E-05	0.23190851	0	2
PENK	4.19159256	0	0.23857281	1	2
SNCAIP	4.05982215	0	0.24631621	1	2
PGAP1	2.375	0	0.42105263	1	2
PIGU	2.375	0	0.42105263	1	2
POLI	3.83589329	4.37E-04	0.26069547	0	2
STIL	4.33953112	0	0.23043964	1	2
VAPB	4.50525465	4.61E-05	0.22196304	0	2
SATB2	3.94907033	6.75E-04	0.25322416	0	2
SCN1B	5.57801132	3.66E-04	0.17927536	0	2
TOR1A	4.16087308	2.95E-04	0.24033418	0	2
TACR3	4.85933711	0	0.20578939	1	2
TACR2	4.85933711	0	0.20578939	1	2
WNT10A	4.22069523	0	0.23692779	1	2
AADAT	5.34923201	0	0.18694272	0	1
AARS2	6.37914309	0	0.15676087	0	1
ABCA7	4.1843169	0	0.23898764	0	1
ATP8B1	5.96362167	0	0.16768334	0	1
ABCC9	5.00565885	0	0.1997739	0	1
AMMECR1	5.35165724	0	0.18685801	0	1
FADS2	5.35165724	0	0.18685801	0	1
ADNP	4.10024252	0	0.24388801	0	1
ADRA1A	4.20210186	0	0.23797614	0	1
AFG3L2	1	0	1	0	1
LONP1	1	0	1	0	1
AIM2	1	0	1	0	1

MEFV	1	0	1	0	1
AIP	4.2611156	0	0.23468033	0	1
FOLH1	4.32497979	0	0.23121495	0	1
GADL1	4.99272433	0	0.20029145	0	1
DPM1	1.5	0	0.66666667	0	1
ALG9	1.5	0	0.66666667	0	1
CTU2	5.7607114	0	0.17358967	0	1
FOXL2	5.19967664	0	0.19231965	0	1
AMY1A	1	0	1	0	1
AMY1B	1	0	1	0	1
ANKK1	4.52950687	0	0.22077459	0	1
SLC1A2	5.03152789	0	0.19874679	0	1
KCNJ10	5.03152789	0	0.19874679	0	1
MOG	5.03152789	0	0.19874679	0	1
ZMIZ1	3.85044462	0	0.25971027	0	1
EGR3	4.57881973	0	0.21839689	0	1
GPR35	4.09458367	0	0.24422507	0	1
ARSE	8.83346807	0	0.11320582	0	1
ARSG	8.83346807	0	0.11320582	0	1
RIF1	4.18835893	0	0.238757	0	1
ATP7B	5.05739693	0	0.19773018	0	1
B7RP1	4.56992724	0	0.21882186	0	1
BMPER	4.56831043	0	0.21889931	0	1
BRAT1	3.99838319	0	0.25010109	0	1
BRDT	4.43654002	0	0.22540087	0	1
CDKN3	5.912692	0	0.1691277	0	1
RAB39B	5.88601455	0	0.16989425	0	1
CACNA1H	4.7170574	0	0.21199657	0	1
CALB1	4.99029911	0	0.20038879	0	1
PLOD1	6.23443816	0	0.16039938	0	1
P2RX7	5.13257882	0	0.19483383	0	1
CARTPT	4.14793856	0	0.24108361	0	1
GRID2	5.79062247	0	0.17269301	0	1
CBSL	1.75	0	0.57142857	0	1
CCKAR	4.87388844	0	0.20517499	0	1
FBXO31	3.98625707	0	0.25086189	0	1
CDCA7	5.29668553	0	0.18879731	0	1
CDH11	3.64268391	0	0.27452286	0	1
CDK13	1	0	1	0	1
MMP23B	1	0	1	0	1
CHCHD2	5.88682296	0	0.16987091	0	1
CHD7	4.01212611	0	0.24924441	0	1
CHRM2	4.46402587	0	0.22401304	0	1

CISD2	5.70816492	0	0.17518765	0	1
CIT	5.41390461	0	0.18470957	0	1
CRLF1	6.06871463	0	0.16477954	0	1
RAI1	4.45028294	0	0.22470481	0	1
CNKSR2	4.01616815	0	0.24899356	0	1
CNR2	4.78577203	0	0.2089527	0	1
FOXP2	5.91673403	0	0.16901216	0	1
COG2	6.23848019	0	0.16029545	0	1
COL12A1	5.53759095	0	0.18058394	0	1
COL7A1	5.53759095	0	0.18058394	0	1
COL9A1	5.53759095	0	0.18058394	0	1
COQ2	5.6087308	0	0.17829346	0	1
NEUROD2	5.24333064	0	0.19071847	0	1
CRIPT	4.06790622	0	0.24582671	0	1
EPCAM	3.64268391	0	0.27452286	0	1
PROP1	3.64268391	0	0.27452286	0	1
SATB1	3.64268391	0	0.27452286	0	1
SOX3	3.64268391	0	0.27452286	0	1
XDH	5.043654	0	0.19826895	0	1
CYP27A1	5.61519806	0	0.17808811	0	1
DAOA	4.93775263	0	0.20252128	0	1
DCX	4.61034762	0	0.21690338	0	1
DGUOK	4.64187551	0	0.21543016	0	1
DTNBP1	4.48423605	0	0.22300343	0	1
DISP1	4.37186742	0	0.22873521	0	1
GRIK1	4.06790622	0	0.24582671	0	1
NEB	4.5974131	0	0.21751363	0	1
DUOX2	1	0	1	0	1
DUOXA2	1	0	1	0	1
NEK1	4.99029911	0	0.20038879	0	1
EFEMP2	5.25222312	0	0.19039557	0	1
MCCC2	4.2716249	0	0.23410295	0	1
EIF2B2	4.98302344	0	0.20068138	0	1
LOXL3	5.25222312	0	0.19039557	0	1
ELP4	1	0	1	0	1
IKBKAP	1	0	1	0	1
SPEN	3.65157639	0	0.27385433	0	1
SLC1A4	5.04284559	0	0.19830074	0	1
EXTL3	4.71059014	0	0.21228763	0	1
FGF14	6.48746968	0	0.1541433	0	1
FLNB	4.2829426	0	0.23348433	0	1
OTX2	4.60307195	0	0.21724622	0	1
FOXC1	4.37752627	0	0.22843952	0	1

FRAS1	1	0	1	0	1
FREM2	1	0	1	0	1
FTH1	4.96119644	0	0.20156428	0	1
GABRD	1	0	1	0	1
SLC6A1	1	0	1	0	1
GAL	4.91592563	0	0.20342049	0	1
GALT	8.57154406	0	0.11666509	0	1
RBFOX3	4.46887631	0	0.2237699	0	1
GPR39	4.63783347	0	0.21561792	0	1
SON	5.63136621	0	0.1775768	0	1
RGS4	4.08003234	0	0.2450961	0	1
HTR7	4.34438157	0	0.23018236	0	1
GPD2	4.89733226	0	0.2041928	0	1
GP1BB	4.82942603	0	0.20706394	0	1
GPR126	4.5214228	0	0.22116932	0	1
GRM4	4.64915117	0	0.21509303	0	1
GRM7	4.7502021	0	0.21051736	0	1
GTF2I	3.83912692	0	0.26047589	0	1
HCRTR1	5.38803557	0	0.1855964	0	1
PROM1	4.11317704	0	0.24312107	0	1
HDC	5.78658044	0	0.17281364	0	1
NAGA	6.54244139	0	0.15284814	0	1
9-Sep	3.91511722	0	0.25542019	0	1
NAA10	3.91511722	0	0.25542019	0	1
TAP1	4.5545675	0	0.21955982	0	1
SLC2A2	4.2069523	0	0.23770177	0	1
PAM16	4.36216653	0	0.22924388	0	1
HTR1B	5.73726758	0	0.174299	0	1
M6PR	4.31285368	0	0.23186504	0	1
INPP5E	3.96281326	0	0.25234598	0	1
JMJD1C	4.46887631	0	0.2237699	0	1
KCND3	6.43977365	0	0.15528496	0	1
KCNJ2	5.57235247	0	0.17945742	0	1
KCNN3	5.65723525	0	0.17676479	0	1
KIAA0196	1	0	1	0	1
KIAA1033	1	0	1	0	1
LHX4	6.33468068	0	0.15786115	0	1
PNLIP	4.2279709	0	0.23652008	0	1
LRRTM1	4.97251415	0	0.20110551	0	1
LTBP3	4.0759903	0	0.24533915	0	1
MAN2B1	1	0	1	0	1
MANBA	1	0	1	0	1
TUBB	4.1083266	0	0.24340811	0	1
MBD5	3.84155214	0	0.26031145	0	1

MC2R	4.3799515	0	0.22831303	0	1
MCHR1	1	0	1	0	1
PMCH	1	0	1	0	1
MIF	4.2611156	0	0.23468033	0	1
MMACHC	1.75	0	0.57142857	0	1
RAD54L	4.78496362	0	0.208988	0	1
MYH3	1	0	1	0	1
MYL3	1	0	1	0	1
NEFM	4.56022635	0	0.21928736	0	1
NEMF	4.39935327	0	0.22730614	0	1
NFIX	4.98302344	0	0.20068138	0	1
NODAL	5.04284559	0	0.19830074	0	1
NPC1	1	0	1	0	1
NPC2	1	0	1	0	1
NPPB	1	0	1	0	1
NPR2	1	0	1	0	1
NPS	5.39288601	0	0.18542947	0	1
PUS7	1.66666667	0	0.6	0	1
OGG1	4.20776071	0	0.2376561	0	1
OPA1	4.56345998	0	0.21913198	0	1
PAX8	4.01212611	0	0.24924441	0	1
PHGDH	1	0	1	0	1
PSAT1	1	0	1	0	1
PLK2	3.64430073	0	0.27440106	0	1
PROK2	1	0	1	0	1
PROKR2	1	0	1	0	1
RBP4	4.75181892	0	0.21044573	0	1
RPGRIP1	5.00646726	0	0.19974164	0	1
SEC24C	4.75424414	0	0.21033838	0	1
SLC5A7	5.63379143	0	0.17750036	0	1
SLC20A2	1	0	1	0	1
XPR1	1	0	1	0	1
SLC2A1	3.64430073	0	0.27440106	0	1
SLC6A2	4.55052546	0	0.21975484	0	1
WDFY3	4.23120453	0	0.23633932	0	1
TRHR	5.39046079	0	0.1855129	0	1
TRPA1	5.58367017	0	0.17909367	0	1
UBE4A	4.34276475	0	0.23026806	0	1
UFD1L	4.34276475	0	0.23026806	0	1

Supplementary Table S5: Intersection list for targets of baicalin and depression

Intersection List for Targets of Baicalin and Depression

Database_Intersected	Total_gene_count	Genes_included
DisGeNET GeneCards OMIM PubChem SwissTargetPrediction	1	7124
DisGeNET GeneCards PubChem SwissTargetPrediction	1	5743
GeneCards HERB OMIM PubChem	1	836
DisGeNET GeneCards OMIM	33	2932
		7466
		1814
		1268
		2099
		2917
		6281
		8910
		9211
		27185
		4524
		8863
		84062
		6622
		2911
		4852
		1812
		2289
		6869
		2890
		4988
		3064
		2260
		10814
		9575
		3360
		1129
		8864
		2908
		3952
		1808
		3351
		3290
DisGeNET GeneCards PubChem	3	3553
		3569
		4843
DisGeNET GeneCards HERB	1	207
DisGeNET GeneCards SwissTargetPrediction	1	150
ChEMBL DisGeNET GeneCards	2	2904

		3620
ChEMBL GeneCards OMIM	1	2902
GeneCards PubChem SwissTargetPrediction	2	43
		7498
ChEMBL GeneCards PubChem	1	596
DisGeNET GeneCards	150	84152
		1408
		6531
		8822
		153
		7200
		9968
		79068
		6616
		1135
		51083
		438
		5443
		348980
		116
		5020
		7133
		3552
		2752
		7422
		1803
		5972
		54738
		7166
		282706
		5027
		7349
		3458
		4128
		7054
		7276
		4074
		9456
		2944
		1557
		6571
		2876
		3479
		5179

2670
5663
361
1269
4548
3061
4287
1848
5144
1394
2562
9759
3350
6506
4803
2353
2263
5243
10522
5368
5021
5697
2688
2571
1813
5649
2346
117
6648
9048
406
5142
6532
2784
3606
1312
4129
6529
4513
6505
4684
627
367
6530

4862
1270
9378
351
488
80114
2899
6863
1395
1407
3613
3570
1103
10013
1621
53919
672
4922
2559
7132
3356
5173
6853
8914
6285
9095
4986
4915
27445
6570
1815
3359
3240
2556
9177
409
1392
2596
1565
6095
7425
1742
2892
3363

		3084
		4306
		866
		121278
		3358
		1385
		2903
		183
		478
		1393
		6647
		3576
		2896
		4846
		5141
		4804
		1137
		255239
		3776
		3060
		267012
		5595
		26281
DisGeNET OMIM	2	573
		1843
DisGeNET HERB	1	1029
GeneCards OMIM	85	57505
		5521
		5167
		2328
		4001
		154664
		5295
		1576
		1436
		5885
		6513
		477
		5538
		6575
		65018
		2891
		493856
		1146

4985
78989
8295
1116
2619
154
50940
50484
673
25
125
3119
54834
5159
56975
2629
2555
2475
1301
23600
6620
57582
9213
3123
3117
2895
6854
846
1861
55154
2324
80326
6812
203228
57554
23154
3303
1639
11277
5590
6908
126
23411
1371

		166336
		94233
		7401
		23413
		291
		79955
		8626
		2912
		6683
		51256
		6389
		9839
		56652
		3440
		2914
		1432
		387119
		1909
		6323
		5133
		7248
		23230
		7399
GeneCards PubChem	13	3172
		1278
		5468
		7157
		3170
		1950
		7040
		3162
		595
		7099
		2875
		6927
		1544
GeneCards HERB	2	5594
		842
GeneCards SwissTargetPrediction	5	3558
		217
		6197
		152
		134
ChEMBL GeneCards	6	472

		132
		5293
		2566
		2561
		2554
PubChem SwissTargetPrediction	1	1956
DisGeNET	64	5367
		3763
		3925
		5209
		10014
		2952
		3939
		775
		468
		2996
		5533
		125958
		815
		8973
		353
		55315
		10815
		2925
		594857
		2847
		63891
		114784
		3976
		51555
		3091
		1240
		467
		54487
		6715
		2065
		2
		1142
		5584
		4157
		2739
		7088
		9734
		156

		111
		5153
		805
		4160
		15
		27121
		5599
		1012
		1607
		113
		9639
		4741
		387129
		4048
		9607
		114
		3066
		5879
		1960
		6422
		23286
		238
		1846
		26523
		4939
		2894
GeneCards	1301	57724
		6942
		4544
		23019
		71
		1801
		6261
		7249
		2898
		8647
		3596
		5979
		54903
		2812
		100126270
		7290
		4054
		30008

6928
2915
6497
1031
1387
8379
3362
4361
406932
2969
9248
2067
4893
1630
266727
79813
120892
4340
6023
56776
5335
6121
6233
540
58
23334
311
6403
825
2592
5428
7204
9131
51715
79734
10743
5715
2290
79577
89970
2314
9126
1816
1954

55325
706
55112
4761
121340
1608
7345
5630
5473
57459
29998
406975
3786
7458
2767
7504
1778
3667
3554
10273
1859
6865
3782
2901
9897
1000
10611
1760
2033
3955
288
1555
4353
23322
2859
410
10225
7098
55209
1180
51322
4438
126695
57338

1741
4094
415
84992
5378
2597
1292
8085
1356
6303
57010
6855
8558
4099
405753
4784
117581
3176
23030
60529
51025
79823
12
203068
4137
7184
1144
4036
2798
875
6750
9990
8726
340533
2146
10075
116442
339123
2719
23025
1471
2690
3485
89910

177
64921
116931
6709
7424
4330
2213
9509
8913
773
7319
22
64359
8148
8506
6573
64324
8842
4508
51
79644
652
57537
7533
3605
1033
9820
2736
796
1026
6352
5621
55120
56623
51166
1499
28514
2108
29123
1977
10801
6401
3309
1380

2316
100874369
54986
23325
10195
27091
1981
9394
166647
1855
3559
84277
348180
10083
860
135886
22909
8621
7546
6125
57492
7084
8659
2796
9401
80184
80144
9217
3700
2733
4613
5196
56896
3612
54897
145173
146167
6909
91461
9496
3663
6934
257194
2900

79152
64218
80036
79882
9255
10371
55830
1559
196528
5745
6462
100652759
7299
4916
89891
526
6491
6218
182
2905
108
2820
6519
83715
23435
4854
3486
6997
27152
3054
2623
81565
142
27235
7431
1857
25978
57216
6487
23400
28981
5184
4023
23345

4089
54463
6536
50964
27161
2774
1141
7407
375
23126
7018
3930
9049
111822955
93986
5406
7020
6335
278
51603
57102
8893
51574
2626
2990
1302
9971
2239
144165
134701
1294
407021
3313
4750
5063
2200
2056
9469
11141
23308
3481
23036
26123
276

7798
3339
6514
6347
102
3827
10524
54187
5327
1193
11315
9463
7227
406938
5290
1106
1836
6605
175
79583
347
23529
1454
54888
8022
55343
9244
6651
5982
1476
79143
5447
8405
2641
6165
1584
4654
81848
171019
155368
886
2006
643853
3178

80331
1028
1589
1718
56172
65217
2550
9147
54205
85358
7102
4849
7057
3756
4609
79365
4313
91179
54872
4436
23426
894
3425
2222
50943
5279
5625
6239
51604
6229
8289
10577
1463
84168
5605
2893
5320
847
2166
1462
23013
51626
6223
8892

7038
335
1717
2557
6660
9653
7189
55599
79867
2657
4882
552
5894
91252
2495
5793
4908
114798
7957
2317
5331
2936
3699
9881
407046
4668
79796
23476
25782
22796
3456
1277
2137
22941
8510
57502
26610
23314
701
148789
867
3630
7552
3070

643418
10913
89884
10046
64327
24140
9742
657
5573
100130283
1756
1636
4126
4204
3800
4842
5081
4976
338
3628
1947
26058
3105
118663
51227
5781
79659
10651
2956
1644
9439
7050
29968
6654
3784
3832
6249
3745
317
50506
5077
4538
5813
1234

6905
6096
8936
55904
546
4535
64093
64398
2535
56729
83872
5816
2152
3352
4072
998
6792
84131
1280
4621
1789
5241
1545
171023
51259
84138
58508
83879
84667
23590
3305
2934
378884
8567
80347
2185
9031
169026
7412
1191
11200
2717
10891
4900

26280
8106
9419
5125
55384
84628
55621
7592
140947
186
79650
9450
973
6696
4952
1291
653361
51085
221927
3107
6492
5913
5277
64478
1586
6558
51317
9370
4522
1493
5617
54664
221037
51185
59341
3981
8654
84162
728012
3643
25839
5476
7442
23780

5080
6658
407026
5395
157570
80781
57520
6597
22808
2132
5582
6711
5354
2272
2253
22854
2697
57822
10735
6310
574034
5445
210
9459
51291
142679
2560
23237
19
3096
3067
8715
169792
1030
2026
9320
11113
55183
22930
793
400916
80232
9723
5193

5305
63895
5191
2261
283
6324
9632
4512
5580
421
149775
6774
7461
2332
56938
126792
23135
10765
2247
64067
57096
124512
2913
3953
26011
1896
597
57539
4541
3357
55790
9757
9533
57178
7434
3265
57688
1588
8492
959
9091
23384
8912
79848

1795
8867
9722
1020
90865
7253
8514
6595
3484
6609
4035
50937
948
2897
5444
1303
8239
5950
3785
3762
10734
675
55717
3984
7432
26137
56006
4208
833
4729
4329
3684
6662
60482
4485
1293
10347
2799
406959
8878
23363
783
91801
135

348
10630
178
6228
55180
3156
3992
3439
7353
51412
4514
63976
6775
11253
113179
6204
6844
54832
2259
10507
5053
3708
8481
2212
7450
4190
3073
57231
9627
5741
6910
720
9451
324
5566
153396
2066
27030
4838
26154
284021
84059
54681
80816

401397
6348
2182
1267
28960
9572
54982
10369
124590
10939
27133
7021
8928
55148
3586
3664
407034
8660
5830
8243
1628
2521
406928
7804
23112
6473
60401
1734
3565
9354
4925
26608
2113
27286
51319
100
3383
100873935
9702
286
81857
3654
22901
79868

4747
442890
407012
9922
129563
4920
7128
9101
6496
25861
57560
2296
84634
5308
2110
83550
5426
94
213
6804
2775
10000
7494
5307
4010
2668
2863
3159
5660
65078
55811
7201
57572
101730217
57545
2043
8450
5449
55777
5155
9487
339896
2012
11172

100529097
81562
6572
5216
5296
3736
6664
4703
246269
2318
2147
102216342
3035
9442
101929680
7430
50651
259232
91875
8675
9969
7415
128674
5189
10133
4796
4133
4593
8974
5187
4669
60
5587
6301
30816
427
26040
128869
26047
10062
8809
80055
79147
650

51053
168667
10060
7257
3480
220296
6925
1763
5805
27245
406961
8788
83696
8738
3845
10277
5244
64240
3295
2100
2918
5980
6314
80198
176
54496
4053
3145
5999
5071
1540
920
7360
128178
64131
5604
2064
869
6135
55737
6833
23317
51738
9772

79798
6045
6326
8074
2760
84148
90417
4155
91147
8820
632
5727
26191
286114
50
8629
9162
1409
7444
1716
1175
4318
9739
322
54101
6402
285362
90416
7037
5015
6509
151246
9444
6866
8458
699
285590
79791
5626
818
5824
55975
29110
51098

5465
3990
5498
57477
55024
5265
79633
7849
9342
8722
10518
84314
7356
25942
4683
55770
1806
2288
1786
9829
1523
2271
26503
899
3340
10082
285489
10683
10769
79158
6899
147372
347344
4879
497258
5054
3767
26115
4049
3636
1962
6334
26229
9569

4125
6507
7048
1113
4724
5581
6906
389434
1509
2771
5718
9415
8831
10280
64072
4744
54862
9786
23236
355
747
553
160851
7991
7097
2563
23732
155382
5859
8443
2643
57526
4540
10716
5194
4929
23096
60675
22866
6870
5321
1788
7173
84695

5495
25836
55636
1399
57680
25814
1027
103
4082
6888
4297
1401
1139
10982
2077
139411
820
9275
8468
23431
11232
4864
6671
1571
7421
3308
145873
668
52
8260
284252
23040
6224
57142
8438
4292
6697
1641
640
2737
5728
341640
3375
9652

5205
55117
54413
9896
6885
2558
135138
8573
23001
755
84153
406989
7008
3778
1906
90121
80832
51741
9949
23522
23405
411
282974
1593
11285
3720
51816
23291
3934
54209
7403
4763
5550
5351
29978
9719
83999
6598
80153
91752
3814
3291
57030
57465

1739
3373
1610
728448
9524
551
4968
148
9830
9319
9361
121256
81603
8813
55869
4210
157
2627
51684
5446
8942
55699
86
5528
80025
23556
5190
6872
5977
54715
23389
10861
9321
6304
26227
1482
57211
5192
2109
885
4927
5075
6311
268

1297
50809
721
613
6515
5420
9184
64802
28952
114049
2691
5286
8799
2778
26012
4158
7252
442900
6602
9841
29957
5116
6528
4647
54517
146713
84163
7474
676
11201
277
9681
7291
3557
27429
51132
57628
1183
406921
2747
4312
929
2215
200909

133
547
23394
5828
124583
84976
1369
3977
4282
23043
170302
6272
2138
406886
388015
5175
1009
7376
3043
403
22858
1917
148014
3717
6387
83636
1761
8398
406947
3759
7332
22871
3454
23414
4221
3560
1134
102723833
406936
3304
473
2572
3106
185

		6469
		65250
		2720
		9581
		164
		3897
		3567
OMIM	65	51095
		8929
		3752
		9447
		64087
		646960
		10380
		55145
		107
		8325
		26873
		28976
		200424
		919
		8927
		4582
		51142
		10352
		6390
		26045
		23207
		114327
		7478
		347730
		3766
		6999
		4634
		94137
		23155
		2532
		3376
		3208
		2702
		79718
		2395
		8546
		7134

		859
		10117
		2677
		10584
		3354
		816
		1355
		51299
		2131
		3257
		22986
		56521
		6890
		22999
		5648
		4853
		5534
		56134
		774
		8518
		10842
		57576
		7384
		5052
		5906
		5664
		25974
		8989
PubChem	21	1244
		2002
		5970
		145270
		581
		2081
		23462
		3280
		1543
		240
		890
		10628
		196
		23493
		1386
		5315

		4851
		4615
		4792
		114548
		2805
HERB	3	5609
		11067
		22882
SwissTargetPrediction	4	56923
		4835
		231
		50507
ChEMBL	6	8242
		6098
		8639
		5771
		5328
		5732

Supplementary Table S6: Network analysis results of common targets

Network Analysis Results of Common Targets

name	AverageShortest PathLength	Betweenness Centrality	Closeness Centrality	Clustering Coefficient	Degree
ACHE	1.87179487	0.00978086	0.53424658	0.76190476	7
IL1B	1.48717949	0.07172935	0.67241379	0.56126482	23
GRIN1	1.79487179	0.17178252	0.55714286	0.25454545	11
AKT1	1.30769231	0.18065866	0.76470588	0.45238095	28
PTGS2	1.69230769	0.00928826	0.59090909	0.76608187	19
GPT	1.76923077	0.0714995	0.56521739	0.675	16
CASP3	1.53846154	0.03535266	0.65	0.64069264	22
IL6	1.53846154	0.05851599	0.65	0.55797101	24
ADK	2.92307692	0	0.34210526	0	1
ADORA1	1.94871795	0.05372245	0.51315789	0.4	5
ADRA2A	3.69230769	0	0.27083333	0	1
ADRA2C	2.71794872	0.05128205	0.36792453	0	2
MAPK1	1.56410256	0.06271286	0.63934426	0.58421053	20
HMOX1	1.79487179	0.00105304	0.55714286	0.93333333	15
TGFB1	1.71794872	0.01121916	0.58208955	0.75163399	18
IL2	1.71794872	0.01244353	0.58208955	0.76470588	18
CCND1	1.71794872	0.01594669	0.58208955	0.67251462	19
HNF1A	2.05128205	5.85E-04	0.4875	0.85714286	7
EGF	1.71794872	0.01524371	0.58208955	0.70175439	19
TP53	1.58974359	0.03567395	0.62903226	0.60474308	23
ATM	1.97435897	0.00359874	0.50649351	0.82222222	10
PPARG	1.66666667	0.02191758	0.6	0.68947368	20
COL1A2	2.15384615	0	0.46428571	1	4
HNF4A	1.92307692	0.01964939	0.52	0.65454545	11
NOS2	1.69230769	0.0320215	0.59090909	0.8021978	14
CASP9	1.74358974	0.00703711	0.57352941	0.79411765	17
TLR4	1.74358974	0.01616254	0.57352941	0.72058824	17
PIK3CD	2.15384615	3.43E-04	0.46428571	0.66666667	4
BCL2	2.12820513	0	0.46987952	1	6
FOXA2	2.07692308	3.28E-04	0.48148148	0.86666667	6
CDKN2A	1.84615385	0.00177688	0.54166667	0.87912088	14
GRIN2B	1.87179487	0.07386739	0.53424658	0.44444444	9
ALDH2	2.74358974	0	0.36448598	0	1
RPS6KA3	2.05128205	0.00744489	0.4875	0.6	6
CYP1A2	2.43589744	0.00319134	0.41052632	0	4
IDO1	2.1025641	0.00680134	0.47560976	0.75	8
XDH	2.20512821	0.00349762	0.45348837	0.6	5
GABRA1	2.61538462	0	0.38235294	1	4
GABRB2	2.61538462	0	0.38235294	1	4
GABRG2	2.61538462	0	0.38235294	1	4

Supplementary Table S7: Topology analysis of network structure

Results for Topology Analysis of Network Structure - Original Network

bg.annotation.Description	bg.annotation.EntityType	bg.annotation.Name	Degree
tumor protein p53	gene	TP53	948
neurotrophic receptor tyrosine kinase 1	gene	NTRK1	640
cullin 3	gene	CUL3	435
amyloid beta precursor protein	gene	APP	429
estrogen receptor 1	gene	ESR1	417
ubiquitin C	gene	UBC	396
exportin 1	gene	XPO1	366
minichromosome maintenance complex component 2	gene	MCM2	366
epidermal growth factor receptor	gene	EGFR	361
cyclin dependent kinase 2	gene	CDK2	349
heat shock protein 90 alpha family class A member 1	gene	HSP90AA1	344
fibronectin 1	gene	FN1	343
AKT serine/threonine kinase 1	gene	AKT1	340
COP9 signalosome subunit 5	gene	COPS5	333
mitogen-activated protein kinase 1	gene	MAPK1	326
cullin 1	gene	CUL1	317
tyrosine 3-monooxygenase/tryptophan 5-monooxygenase activation protein zeta	gene	YWHAZ	315
E1A binding protein p300	gene	EP300	311
growth factor receptor bound protein 2	gene	GRB2	305
cullin 7	gene	CUL7	295
MDM2 proto-oncogene	gene	MDM2	295
nucleophosmin	gene	NPM1	289
heat shock protein 90 alpha family class B member 1	gene	HSP90AB1	282
valosin containing protein	gene	VCP	277
BRCA1, DNA repair associated	gene	BRCA1	277
v-myc avian myelocytomatosis viral oncogene homolog	gene	MYC	277
histone deacetylase 1	gene	HDAC1	275
ring finger protein 2	gene	RNF2	262
sirtuin 7	gene	SIRT7	261
obscurin like 1	gene	OBSL1	257
ubiquitin conjugating enzyme E2 I	gene	UBE2I	251
TNF receptor associated factor 6	gene	TRAF6	250
F-box protein 6	gene	FBXO6	239
parkin RBR E3 ubiquitin protein ligase	gene	PARK2	239
HECT, UBA and WWE domain containing 1, E3 ubiquitin protein ligase	gene	HUWE1	239
coiled-coil domain containing 8	gene	CCDC8	239
CREB binding protein	gene	CREBBP	230
heterogeneous nuclear ribonucleoprotein U	gene	HNRNPU	229

histone cluster 1 H3 family member f	gene	HIST1H3F	228
histone cluster 1 H3 family member h	gene	HIST1H3H	228
histone cluster 1 H3 family member j	gene	HIST1H3J	228
histone cluster 1 H3 family member b	gene	HIST1H3B	228
histone cluster 1 H3 family member e	gene	HIST1H3E	228
histone cluster 1 H3 family member c	gene	HIST1H3C	228
histone cluster 1 H3 family member g	gene	HIST1H3G	228
histone cluster 1 H3 family member i	gene	HIST1H3I	228
histone cluster 1 H3 family member d	gene	HIST1H3D	228
histone cluster 1 H3 family member a	gene	HIST1H3A	228
heat shock protein family A (Hsp70) member 5	gene	HSPA5	227
SNW domain containing 1	gene	SNW1	226
cell division cycle 5 like	gene	CDC5L	225
tyrosine 3-monooxygenase/tryptophan 5-monooxygenase activation protein theta	gene	YWHAQ	225
heterogeneous nuclear ribonucleoprotein A1	gene	HNRNPA1	223
eukaryotic translation elongation factor 1 alpha 1	gene	EEF1A1	222
catenin beta 1	gene	CTNNB1	222
heat shock protein family A (Hsp70) member 8	gene	HSPA8	219
histone deacetylase 5	gene	HDAC5	219
embryonic ectoderm development	gene	EED	218
cullin 2	gene	CUL2	217
androgen receptor	gene	AR	213
replication protein A1	gene	RPA1	209
von Hippel-Lindau tumor suppressor	gene	VHL	208
SRC proto-oncogene, non-receptor tyrosine kinase	gene	SRC	206
EWS RNA binding protein 1	gene	EWSR1	205
PAN2 poly(A) specific ribonuclease subunit	gene	PAN2	204
tyrosine 3-monooxygenase/tryptophan 5-monooxygenase activation protein epsilon	gene	YWHAE	203
SMAD specific E3 ubiquitin protein ligase 1	gene	SMURF1	196
replication protein A2	gene	RPA2	196
arrestin beta 2	gene	ARRB2	196
SMAD family member 3	gene	SMAD3	193
ribosomal protein S27a	gene	RPS27A	192
RELA proto-oncogene, NF-kB subunit	gene	RELA	192
cullin 5	gene	CUL5	191
inhibitor of nuclear factor kappa B kinase subunit gamma	gene	IKBKG	191
tyrosine 3-monooxygenase/tryptophan 5-monooxygenase activation protein gamma	gene	YWHAG	190
mitogen-activated protein kinase 3	gene	MAPK3	190
heat shock protein family A (Hsp70) member 4	gene	HSPA4	188
histone deacetylase 2	gene	HDAC2	181

caspase 3	gene	CASP3	181
FUS RNA binding protein	gene	FUS	177
RB transcriptional corepressor 1	gene	RB1	176
cyclin dependent kinase inhibitor 2A	gene	CDKN2A	175
ATM serine/threonine kinase	gene	ATM	175
SMAD family member 2	gene	SMAD2	173
poly(ADP-ribose) polymerase 1	gene	PARP1	172
aurora kinase A	gene	AURKA	170
tyrosine 3-monooxygenase/tryptophan 5-monooxygenase activation protein beta	gene	YWHAB	169
cyclin dependent kinase 1	gene	CDK1	168
proliferating cell nuclear antigen	gene	PCNA	166
histone deacetylase 3	gene	HDAC3	165
inhibitor of nuclear factor kappa B kinase subunit epsilon	gene	IKBKE	165
cullin 4B	gene	CUL4B	164
protein kinase, DNA-activated, catalytic polypeptide	gene	PRKDC	163
cyclin dependent kinase inhibitor 1A	gene	CDKN1A	162
promyelocytic leukemia	gene	PML	162
receptor for activated C kinase 1	gene	RACK1	160
Jun proto-oncogene, AP-1 transcription factor subunit	gene	JUN	160
glycogen synthase kinase 3 beta	gene	GSK3B	159
H2A histone family member X	gene	H2AFX	158
Sp1 transcription factor	gene	SP1	155
SWI/SNF related, matrix associated, actin dependent regulator of chromatin, subfamily a, member 4	gene	SMARCA4	155
actin beta	gene	ACTB	155
peroxisome proliferator activated receptor gamma	gene	PPARG	155
tubulin beta class I	gene	TUBB	155
BCL2 associated athanogene 3	gene	BAG3	155
nitric oxide synthase 2	gene	NOS2	154
sirtuin 1	gene	SIRT1	153
filamin A	gene	FLNA	152
ABL proto-oncogene 1, non-receptor tyrosine kinase	gene	ABL1	151
arrestin beta 1	gene	ARRB1	150
protein phosphatase 1 catalytic subunit alpha	gene	PPP1CA	150
X-ray repair cross complementing 6	gene	XRCC6	149
histone cluster 1 H4 family member i	gene	HIST1H4I	148
heat shock protein family B (small) member 1	gene	HSPB1	148
histone cluster 1 H4 family member l	gene	HIST1H4L	148
histone cluster 2 H4 family member a	gene	HIST2H4A	148
histone cluster 1 H4 family member h	gene	HIST1H4H	148
histone cluster 1 H4 family member c	gene	HIST1H4C	148
histone cluster 1 H4 family member e	gene	HIST1H4E	148

histone cluster 1 H4 family member b	gene	HIST1H4B	148
histone cluster 2 H4 family member b	gene	HIST2H4B	148
histone cluster 4 H4	gene	HIST4H4	148
histone cluster 1 H4 family member f	gene	HIST1H4F	148
histone cluster 1 H4 family member d	gene	HIST1H4D	148
histone cluster 1 H4 family member j	gene	HIST1H4J	148
histone cluster 1 H4 family member k	gene	HIST1H4K	148
histone cluster 1 H4 family member a	gene	HIST1H4A	148
tubulin gamma 1	gene	TUBG1	147
nucleolin	gene	NCL	147
cullin 4A	gene	CUL4A	146
clathrin heavy chain	gene	CLTC	146
COP9 signalosome subunit 6	gene	COPS6	146
beta-transducin repeat containing E3 ubiquitin protein ligase	gene	BTRC	145
enhancer of zeste 2 polycomb repressive complex 2 subunit	gene	EZH2	145
nuclear factor kappa B subunit 1	gene	NFKB1	145
SHC adaptor protein 1	gene	SHC1	143
histone deacetylase 6	gene	HDAC6	143
neural precursor cell expressed, developmentally down-regulated 8	gene	NEDD8	142
ribosomal protein S8	gene	RPS8	141
ribosomal protein S3	gene	RPS3	141
STIP1 homology and U-box containing protein 1	gene	STUB1	141
myosin heavy chain 9	gene	MYH9	141
damage specific DNA binding protein 1	gene	DDB1	140
leucine rich repeat kinase 2	gene	LRRK2	140
CRK proto-oncogene, adaptor protein	gene	CRK	140
COMMD3-BMI1 readthrough	gene	COMMD3-BMI1	140
BMI1 proto-oncogene, polycomb ring finger	gene	BMI1	140
protein kinase C alpha	gene	PRKCA	139
lysine acetyltransferase 5	gene	KAT5	139
ribosomal protein L6	gene	RPL6	138
mitogen-activated protein kinase kinase kinase 1	gene	MAP3K1	138
poly(A) binding protein cytoplasmic 1	gene	PABPC1	137
histone cluster 3 H3	gene	HIST3H3	137
nuclear receptor subfamily 3 group C member 1	gene	NR3C1	137
TNF receptor associated factor 2	gene	TRAF2	137
heterogeneous nuclear ribonucleoprotein K	gene	HNRNPK	136
X-ray repair cross complementing 5	gene	XRCC5	136
vimentin	gene	VIM	135
telomeric repeat binding factor 1	gene	TERF1	135
mitogen-activated protein kinase kinase kinase 3	gene	MAP3K3	135

glyceraldehyde-3-phosphate dehydrogenase	gene	GAPDH	134
TNF receptor superfamily member 1A	gene	TNFRSF1A	134
signal transducer and activator of transcription 3	gene	STAT3	134
proteasome subunit alpha 3	gene	PSMA3	134
tripartite motif containing 28	gene	TRIM28	134
F-box and WD repeat domain containing 11	gene	FBXW11	133
caspase 8	gene	CASP8	133
protein phosphatase 2 catalytic subunit alpha	gene	PPP2CA	133
mitogen-activated protein kinase 14	gene	MAPK14	132
ribosomal protein S2	gene	RPS2	132
lysine acetyltransferase 2B	gene	KAT2B	131
phosphatase and tensin homolog	gene	PTEN	129
mitogen-activated protein kinase 8	gene	MAPK8	129
minichromosome maintenance complex component 5	gene	MCM5	129
ubiquitin specific peptidase 7	gene	USP7	128
heat shock protein family A (Hsp70) member 1B	gene	HSPA1B	127
heat shock protein family A (Hsp70) member 1A	gene	HSPA1A	127
stratifin	gene	SFN	127
ribosomal protein lateral stalk subunit P0	gene	RPLP0	127
nuclear receptor corepressor 1	gene	NCOR1	127
cell division cycle 37	gene	CDC37	126
heat shock protein family A (Hsp70) member 9	gene	HSPA9	125
ribosomal protein S3A	gene	RPS3A	125
ribosomal protein L5	gene	RPL5	125
DEAD-box helicase 5	gene	DDX5	124
FYN proto-oncogene, Src family tyrosine kinase	gene	FYN	123
casein kinase 2 beta	gene	CSNK2B	123
ribosomal protein S6	gene	RPS6	123
Raf-1 proto-oncogene, serine/threonine kinase	gene	RAF1	123
ribosomal protein L14	gene	RPL14	121
ribosomal protein S4, X-linked	gene	RPS4X	121
RNA polymerase II subunit A	gene	POLR2A	121
peptidylprolyl cis/trans isomerase, NIMA-interacting 1	gene	PIN1	121
heterogeneous nuclear ribonucleoprotein M	gene	HNRNPM	121
SMAD family member 4	gene	SMAD4	121
BCL2, apoptosis regulator	gene	BCL2	120
ribosomal protein L13	gene	RPL13	120
polo like kinase 1	gene	PLK1	120
sequestosome 1	gene	SQSTM1	119
heat shock protein family D (Hsp60) member 1	gene	HSPD1	119
activating transcription factor 2	gene	ATF2	119
RB binding protein 4, chromatin remodeling factor	gene	RBBP4	119
inhibitor of nuclear factor kappa B kinase subunit beta	gene	IKBKB	119
ribosomal protein S14	gene	RPS14	118

phosphoinositide-3-kinase regulatory subunit 1	gene	PIK3R1	118
heterogeneous nuclear ribonucleoprotein D	gene	HNRNPD	117
IQ motif containing GTPase activating protein 1	gene	IQGAP1	117
ribosomal protein L24	gene	RPL24	117
ribosomal protein L7	gene	RPL7	117
ribosomal protein L4	gene	RPL4	117
huntingtin	gene	HTT	116
small ubiquitin-like modifier 1	gene	SUMO1	115
paxillin	gene	PXN	115
protein phosphatase 2 scaffold subunit Aalpha	gene	PPP2R1A	115
death domain associated protein	gene	DAXX	114
small ubiquitin-like modifier 2	gene	SUMO2	114
lysine demethylase 1A	gene	KDM1A	114
ribosomal protein S16	gene	RPS16	114
RuvB like AAA ATPase 2	gene	RUVBL2	114
neural precursor cell expressed, developmentally down-regulated 4, E3 ubiquitin protein ligase	gene	NEDD4	114
lamin A/C	gene	LMNA	114
tumor necrosis factor	gene	TNF	113
cyclin D1	gene	CCND1	113
proteasome 26S subunit, non-ATPase 2	gene	PSMD2	113
protein kinase cAMP-activated catalytic subunit alpha	gene	PRKACA	113
adrenoceptor beta 2	gene	ADRB2	113
protein phosphatase 1 catalytic subunit gamma	gene	PPP1CC	113
ribosomal protein L23	gene	RPL23	113
erb-b2 receptor tyrosine kinase 2	gene	ERBB2	112
BRCA1 associated RING domain 1	gene	BARD1	112
integrin linked kinase	gene	ILK	111
caveolin 1	gene	CAV1	110
CD81 molecule	gene	CD81	109
ribosomal protein L11	gene	RPL11	109
minichromosome maintenance complex component 7	gene	MCM7	109
conserved helix-loop-helix ubiquitous kinase	gene	CHUK	108
WD repeat domain 5	gene	WDR5	108
ribosomal protein L23a	gene	RPL23A	108
defective in cullin neddylation 1 domain containing 1	gene	DCUN1D1	108
major histocompatibility complex, class I, B	gene	HLA-B	107
ribosomal protein S18	gene	RPS18	107
tubulin alpha 1a	gene	TUBA1A	106
cyclin dependent kinase 9	gene	CDK9	106
ribosomal protein L7a	gene	RPL7A	106
actin gamma 1	gene	ACTG1	106
proteasome 26S subunit, non-ATPase 4	gene	PSMD4	106
Y-box binding protein 1	gene	YBX1	106

telomeric repeat binding factor 2	gene	TERF2	105
PAX interacting protein 1	gene	PAXIP1	105
ribosomal protein S10	gene	RPS10	105
ribosomal protein L18	gene	RPL18	105
proteasome 26S subunit, ATPase 5	gene	PSMC5	105
aurora kinase B	gene	AURKB	105
mutated in colorectal cancers	gene	MCC	105
hypoxia inducible factor 1 alpha subunit	gene	HIF1A	104
protein arginine methyltransferase 5	gene	PRMT5	104
NFKB inhibitor alpha	gene	NFKBIA	104
heterogeneous nuclear ribonucleoprotein A2/B1	gene	HNRNPA2B1	103
BCL2 associated athanogene 6	gene	BAG6	103
chaperonin containing TCP1 subunit 3	gene	CCT3	103
tumor protein p73	gene	TP73	103
survival of motor neuron 1, telomeric	gene	SMN1	103
survival of motor neuron 2, centromeric	gene	SMN2	103
ribosomal protein S7	gene	RPS7	103
F-box and WD repeat domain containing 7	gene	FBXW7	103
ribosomal protein L3	gene	RPL3	103
protein arginine methyltransferase 1	gene	PRMT1	102
tumor protein p53 binding protein 1	gene	TP53BP1	102
ribosomal protein S19	gene	RPS19	102
ribosomal protein L31	gene	RPL31	102
ribosomal protein L8	gene	RPL8	102
ubiquitin C-terminal hydrolase L5	gene	UCHL5	102
SET nuclear proto-oncogene	gene	SET	101
signal transducer and activator of transcription 1	gene	STAT1	101
ribosomal protein L19	gene	RPL19	101
karyopherin subunit beta 1	gene	KPNB1	101
eukaryotic translation elongation factor 2	gene	EEF2	100
splicing factor proline and glutamine rich	gene	SFPQ	100
retinoid X receptor alpha	gene	RXRA	100
ribosomal protein S13	gene	RPS13	100
ras-related C3 botulinum toxin substrate 1 (rho family, small GTP binding protein Rac1)	gene	RAC1	100
ribosomal protein S9	gene	RPS9	99
immunoglobulin superfamily member 8	gene	IGSF8	99
ribosomal protein L15	gene	RPL15	99
APC, WNT signaling pathway regulator	gene	APC	99
nuclear receptor coactivator 3	gene	NCOA3	98
ubiquitin B	gene	UBB	98
complement C1q binding protein	gene	C1QBP	98
protein kinase C delta	gene	PRKCD	98
tubulin alpha 1c	gene	TUBA1C	98

mediator of DNA damage checkpoint 1	gene	MDC1	98
Fos proto-oncogene, AP-1 transcription factor subunit	gene	FOS	97
transforming growth factor beta 1	gene	TGFB1	97
chaperonin containing TCP1 subunit 2	gene	CCT2	97
ring-box 1	gene	RBX1	97
fibrillarin	gene	FBL	96
SIN3 transcription regulator family member A	gene	SIN3A	96
ribosomal protein L27	gene	RPL27	96
LYN proto-oncogene, Src family tyrosine kinase	gene	LYN	96
spectrin alpha, non-erythrocytic 1	gene	SPTAN1	95
S-phase kinase associated protein 1	gene	SKP1	95
retinoic acid receptor alpha	gene	RARA	95
histone deacetylase 4	gene	HDAC4	95
mitogen-activated protein kinase kinase kinase 14	gene	MAP3K14	95
ribosomal protein lateral stalk subunit P2	gene	RPLP2	94
ribosomal protein L12	gene	RPL12	94
OTU deubiquitinase, ubiquitin aldehyde binding 1	gene	OTUB1	94
poly(rC) binding protein 1	gene	PCBP1	94
heterogeneous nuclear ribonucleoprotein R	gene	HNRNPR	94
drebrin 1	gene	DBN1	93
ribosomal protein L10a	gene	RPL10A	93
p21 (RAC1) activated kinase 1	gene	PAK1	92
proteasome subunit alpha 6	gene	PSMA6	92
minichromosome maintenance complex component 3	gene	MCM3	92
intercellular adhesion molecule 1	gene	ICAM1	91
lysine acetyltransferase 2A	gene	KAT2A	91
ribosomal protein S25	gene	RPS25	91
ribosomal protein L21	gene	RPL21	91
KH RNA binding domain containing, signal transduction associated 1	gene	KHDRBS1	91
notch 1	gene	NOTCH1	91
SET domain bifurcated 1	gene	SETDB1	91
nuclear receptor corepressor 2	gene	NCOR2	91
TANK binding kinase 1	gene	TBK1	91
karyopherin subunit alpha 2	gene	KPNA2	91
heterogeneous nuclear ribonucleoprotein H1	gene	HNRNPH1	90
TNF receptor associated factor 1	gene	TRAF1	90
ribosomal protein L18a	gene	RPL18A	90
ATP binding cassette subfamily E member 1	gene	ABCE1	90
LIM domain and actin binding 1	gene	LIMA1	90
DEAD-box helicase 17	gene	DDX17	90
neurofibromin 2	gene	NF2	90
interleukin 1 receptor associated kinase 1	gene	IRAK1	90
SMAD family member 1	gene	SMAD1	90

SWI/SNF related, matrix associated, actin dependent regulator of chromatin, subfamily a, member 5	gene	SMARCA5	89
heat shock protein 90 beta family member 1	gene	HSP90B1	89
topoisomerase (DNA) I	gene	TOP1	89
ribosomal protein S24	gene	RPS24	89
ribosomal protein L37a	gene	RPL37A	89
dynein light chain LC8-type 1	gene	DYNLL1	89
tumor protein p63	gene	TP63	89
mitogen-activated protein kinase kinase kinase 7	gene	MAP3K7	88
synuclein alpha	gene	SNCA	88
carbamoyl-phosphate synthetase 2, aspartate transcarbamylase, and dihydroorotase	gene	CAD	88
tubulin alpha 4a	gene	TUBA4A	87
SWI/SNF related, matrix associated, actin dependent regulator of chromatin subfamily c member 1	gene	SMARCC1	87
histone cluster 2 H2B family member e	gene	HIST2H2BE	87
mechanistic target of rapamycin	gene	MTOR	86
SWI/SNF related, matrix associated, actin dependent regulator of chromatin subfamily c member 2	gene	SMARCC2	86
RB binding protein 7, chromatin remodeling factor	gene	RBBP7	86
p21 (RAC1) activated kinase 2	gene	PAK2	86
proteasome 26S subunit, ATPase 3	gene	PSMC3	86
hepatocyte growth factor-regulated tyrosine kinase substrate	gene	HGS	86
eukaryotic translation initiation factor 6	gene	EIF6	86
heterogeneous nuclear ribonucleoprotein C (C1/C2)	gene	HNRNPC	85
DnaJ heat shock protein family (Hsp40) member A1	gene	DNAJA1	85
DEAD-box helicase 3, X-linked	gene	DDX3X	85
ribosomal protein S27	gene	RPS27	85
cAMP responsive element binding protein 1	gene	CREB1	85
chromodomain helicase DNA binding protein 3	gene	CHD3	85
cofilin 1	gene	CFL1	85
actin, alpha 1, skeletal muscle	gene	ACTA1	85
annexin A2	gene	ANXA2	85
protein phosphatase 1 catalytic subunit beta	gene	PPP1CB	85
40S ribosomal protein S26	gene	LOC101929876	84
TATA-box binding protein	gene	TBP	84
E2F transcription factor 1	gene	E2F1	84
ribosomal protein S26	gene	RPS26	84
cyclin dependent kinase 4	gene	CDK4	84
WW domain containing oxidoreductase	gene	WWOX	84
phospholipase C gamma 1	gene	PLCG1	84
COP9 signalosome subunit 2	gene	COPS2	84
keratin 18	gene	KRT18	84

erb-b2 receptor tyrosine kinase 3	gene	ERBB3	83
C-terminal binding protein 1	gene	CTBP1	83
cadherin 1	gene	CDH1	83
stress induced phosphoprotein 1	gene	STIP1	83
heat shock protein family A (Hsp70) member 1 like	gene	HSPA1L	82
enolase 1	gene	ENO1	82
t-complex 1	gene	TCP1	82
cell division cycle 42	gene	CDC42	82
ribosomal protein L30	gene	RPL30	82
proteasome 26S subunit, non-ATPase 1	gene	PSMD1	82
proteasome 26S subunit, ATPase 2	gene	PSMC2	82
MRE11 homolog, double strand break repair nuclease	gene	MRE11	82
lysine methyltransferase 2A	gene	KMT2A	82
nucleosome assembly protein 1 like 1	gene	NAP1L1	82
SKI proto-oncogene	gene	SKI	81
chaperonin containing TCP1 subunit 6A	gene	CCT6A	81
RAD23 homolog A, nucleotide excision repair protein	gene	RAD23A	81
proteasome subunit alpha 1	gene	PSMA1	81
solute carrier family 25 member 5	gene	SLC25A5	81
chaperonin containing TCP1 subunit 4	gene	CCT4	81
mutS homolog 2	gene	MSH2	81
interferon gamma inducible protein 16	gene	IFI16	81
hepatocyte nuclear factor 4 alpha	gene	HNF4A	80
estrogen receptor 2	gene	ESR2	80
ligand dependent nuclear receptor interacting factor 1	gene	LRIF1	80
mitogen-activated protein kinase kinase 1	gene	MAP2K1	80
protein kinase C zeta	gene	PRKCZ	80
itchy E3 ubiquitin protein ligase	gene	ITCH	80
spleen associated tyrosine kinase	gene	SYK	79
chaperonin containing TCP1 subunit 8	gene	CCT8	79
proteasome subunit alpha 2	gene	PSMA2	79
chaperonin containing TCP1 subunit 7	gene	CCT7	79
heterogeneous nuclear ribonucleoprotein F	gene	HNRNPF	78
ubiquitin protein ligase E3A	gene	UBE3A	78
emerin	gene	EMD	78
TGF-beta activated kinase 1/MAP3K7 binding protein 2	gene	TAB2	78
ataxin 1	gene	ATXN1	78
tripartite motif containing 27	gene	TRIM27	78
adaptor related protein complex 2 mu 1 subunit	gene	AP2M1	78
ATP synthase, H+ transporting, mitochondrial F1 complex, alpha subunit 1, cardiac muscle	gene	ATP5A1	78
proliferation-associated 2G4	gene	PA2G4	78
proteasome 26S subunit, non-ATPase 11	gene	PSMD11	78

tubulin beta 4B class IVb	gene	TUBB4B	78
galectin 3 binding protein	gene	LGALS3BP	78
YY1 transcription factor	gene	YY1	77
topoisomerase (DNA) II alpha	gene	TOP2A	77
ribosomal protein L13a	gene	RPL13A	77
S-phase kinase associated protein 2	gene	SKP2	77
SRSF protein kinase 1	gene	SRPK1	77
RAN, member RAS oncogene family	gene	RAN	77
protein tyrosine phosphatase, non-receptor type 11	gene	PTPN11	77
mutL homolog 1	gene	MLH1	77
nibrin	gene	NBN	77
splicing factor 1	gene	SF1	76
epidermal growth factor receptor pathway substrate 15	gene	EPS15	76
transforming growth factor beta receptor 1	gene	TGFBR1	76
BCL2 like 1	gene	BCL2L1	76
proteasome subunit alpha 7	gene	PSMA7	76
mediator complex subunit 23	gene	MED23	76
serine and arginine rich splicing factor 1	gene	SRSF1	75
ribosomal protein S15	gene	RPS15	75
checkpoint kinase 2	gene	CHEK2	75
ATR serine/threonine kinase	gene	ATR	75
proteasome 26S subunit, ATPase 1	gene	PSMC1	75
X-linked inhibitor of apoptosis	gene	XIAP	75
NCK adaptor protein 1	gene	NCK1	75
nuclear receptor coactivator 1	gene	NCOA1	75
HECT and RLD domain containing E3 ubiquitin protein ligase 2	gene	HERC2	74
OFD1, centriole and centriolar satellite protein	gene	OFD1	74
protein tyrosine kinase 2	gene	PTK2	74
structural maintenance of chromosomes 3	gene	SMC3	74
microtubule associated protein tau	gene	MAPT	74
structural maintenance of chromosomes 1A	gene	SMC1A	73
general transcription factor IIi	gene	GTF2I	73
transducin like enhancer of split 1	gene	TLE1	73
HECT, C2 and WW domain containing E3 ubiquitin protein ligase 2	gene	HECW2	73
chaperonin containing TCP1 subunit 5	gene	CCT5	73
prohibitin	gene	PHB	73
nuclear factor of activated T-cells 1	gene	NFATC1	73
receptor interacting serine/threonine kinase 1	gene	RIPK1	73
protein inhibitor of activated STAT 1	gene	PIAS1	73
heterogeneous nuclear ribonucleoprotein L	gene	HNRNPL	73
tumor susceptibility 101	gene	TSG101	72
TNF receptor superfamily member 1B	gene	TNFRSF1B	72

protection of telomeres 1	gene	POT1	72
eukaryotic translation elongation factor 1 gamma	gene	EEF1G	72
caspase 7	gene	CASP7	72
eukaryotic translation initiation factor 1B	gene	EIF1B	72
LCK proto-oncogene, Src family tyrosine kinase	gene	LCK	72
ubiquilin 1	gene	UBQLN1	72
DEAD-box helicase 1	gene	DDX1	71
ribosomal protein S6 kinase A3	gene	RPS6KA3	71
chromodomain helicase DNA binding protein 4	gene	CHD4	71
actinin alpha 4	gene	ACTN4	71
protein kinase C beta	gene	PRKCB	71
RAD50 double strand break repair protein	gene	RAD50	71
minichromosome maintenance complex component 6	gene	MCM6	71
SERPINE1 mRNA binding protein 1	gene	SERBP1	70
zinc finger and BTB domain containing 16	gene	ZBTB16	70
desmoplakin	gene	DSP	70
CCAAT/enhancer binding protein beta	gene	CEBPB	70
proteasome activator subunit 3	gene	PSME3	70
BCL2 associated athanogene 2	gene	BAG2	70
nuclear receptor subfamily 4 group A member 1	gene	NR4A1	69
vitamin D (1,25- dihydroxyvitamin D3) receptor	gene	VDR	69
SWI/SNF related, matrix associated, actin dependent regulator of chromatin, subfamily b, member 1	gene	SMARCB1	69
Fas associated factor family member 2	gene	FAF2	69
regulator of chromosome condensation 1	gene	RCC1	69
polypyrimidine tract binding protein 1	gene	PTBP1	69
proteasome 26S subunit, ATPase 6	gene	PSMC6	69
ubiquitin specific peptidase 11	gene	USP11	68
DNA methyltransferase 1	gene	DNMT1	68
cyclin A2	gene	CCNA2	68
serine/threonine kinase 11	gene	STK11	68
calnexin	gene	CANX	68
casein kinase 1 alpha 1	gene	CSNK1A1	68
RAS p21 protein activator 1	gene	RASA1	68
checkpoint kinase 1	gene	CHEK1	68
RAN binding protein 2	gene	RANBP2	68
ribosomal protein L26	gene	RPL26	68
sirtuin 6	gene	SIRT6	68
insulin receptor substrate 1	gene	IRS1	68
ribosomal protein S17	gene	RPS17	67
protein tyrosine phosphatase, non-receptor type 1	gene	PTPN1	67
proteasome subunit alpha 5	gene	PSMA5	67
lactate dehydrogenase A	gene	LDHA	67
TSC22 domain family member 1	gene	TSC22D1	66

heat shock transcription factor 1	gene	HSF1	66
growth arrest and DNA damage inducible alpha	gene	GADD45A	66
dynactin subunit 1	gene	DCTN1	66
cyclin dependent kinase 8	gene	CDK8	66
prolyl 4-hydroxylase subunit beta	gene	P4HB	66
proteasome 26S subunit, non-ATPase 3	gene	PSMD3	66
protein inhibitor of activated STAT 4	gene	PIAS4	66
phosphoinositide-3-kinase regulatory subunit 2	gene	PIK3R2	66
TGF-beta activated kinase 1 (MAP3K7) binding protein 1	gene	TAB1	66
thyroid hormone receptor associated protein 3	gene	THRAP3	66
menin 1	gene	MEN1	66
lactate dehydrogenase B	gene	LDHB	66
poly(A) binding protein cytoplasmic 4	gene	PABPC4	66
centrosomal protein 128	gene	CEP128	65
small nuclear ribonucleoprotein polypeptide A'	gene	SNRPA1	65
nuclear receptor coactivator 2	gene	NCOA2	65
mitogen-activated protein kinase kinase kinase 5	gene	MAP3K5	65
ribosomal protein S6 kinase A5	gene	RPS6KA5	65
insulin receptor substrate 4	gene	IRS4	64
tropomyosin 3	gene	TPM3	64
transcription factor 3	gene	TCF3	64
superoxide dismutase 1	gene	SOD1	64
nuclear receptor coactivator 6	gene	NCOA6	64
CCAAT/enhancer binding protein alpha	gene	CEBPA	64
RAN binding protein 9	gene	RANBP9	64
TERF2 interacting protein	gene	TERF2IP	64
metastasis associated 1 family member 2	gene	MTA2	64
HRas proto-oncogene, GTPase	gene	HRAS	63
ubiquitin conjugating enzyme E2 N	gene	UBE2N	63
TATA-box binding protein associated factor 1	gene	TAF1	63
SWI/SNF related, matrix associated, actin dependent regulator of chromatin, subfamily d, member 1	gene	SMARCD1	63
chromobox 3	gene	CBX3	63
acidic nuclear phosphoprotein 32 family member B	gene	ANP32B	63
MET proto-oncogene, receptor tyrosine kinase	gene	MET	63
high mobility group box 1	gene	HMGB1	62
heat shock protein family E (Hsp10) member 1	gene	HSPE1	62
four and a half LIM domains 2	gene	FHL2	62
epithelial cell transforming 2	gene	ECT2	62
dishevelled segment polarity protein 2	gene	DVL2	62
casein kinase 2 alpha 1	gene	CSNK2A1	62
casein kinase 2 alpha 3	gene	CSNK2A3	62
poly(rC) binding protein 2	gene	PCBP2	62

Bruton tyrosine kinase	gene	BTK	62
mitogen-activated protein kinase 9	gene	MAPK9	62
coactivator associated arginine methyltransferase 1	gene	CARM1	62
G protein subunit beta 2	gene	GNB2	61
cyclin dependent kinase inhibitor 1B	gene	CDKN1B	61
cyclin dependent kinase 5	gene	CDK5	61
proteasome 26S subunit, non-ATPase 12	gene	PSMD12	61
proteasome subunit alpha 4	gene	PSMA4	61
MYB binding protein 1a	gene	MYBBP1A	61
myosin IC	gene	MYO1C	61
SUMO1/sentrin/SMT3 specific peptidase 3	gene	SENP3	60
thioredoxin	gene	TXN	60
erythrocyte membrane protein band 4.1	gene	EPB41	60
transcription factor 4	gene	TCF4	60
TATA-box binding protein associated factor 9	gene	TAF9	60
small nuclear ribonucleoprotein D3 polypeptide	gene	SNRPD3	60
autophagy and beclin 1 regulator 1	gene	AMBRA1	60
heat shock protein family H (Hsp110) member 1	gene	HSPH1	60
BCR, RhoGEF and GTPase activating protein	gene	BCR	60
proteasome 26S subunit, ATPase 4	gene	PSMC4	60
baculoviral IAP repeat containing 2	gene	BIRC2	60
proteasome 26S subunit, non-ATPase 14	gene	PSMD14	60
mediator complex subunit 1	gene	MED1	60
RNA binding motif protein 39	gene	RBM39	60
PDZ and LIM domain 7	gene	PDLIM7	60
TNFRSF1A associated via death domain	gene	TRADD	60
ubiquitin protein ligase E3 component n-recognin 5	gene	UBR5	60
tripartite motif containing 24	gene	TRIM24	59
axin 1	gene	AXIN1	59
eukaryotic translation initiation factor 4B	gene	EIF4B	59
BCL2 associated X, apoptosis regulator	gene	BAX	59
ribosomal protein S6 kinase A1	gene	RPS6KA1	59
SSX family member 2 interacting protein	gene	SSX2IP	59
heterogeneous nuclear ribonucleoprotein U like 1	gene	HNRNPUL1	59
RAD51 recombinase	gene	RAD51	59
cell division cycle 27	gene	CDC27	59
prostaglandin E synthase 3	gene	PTGES3	59
splicing factor 3b subunit 2	gene	SF3B2	59
proteasome subunit beta 3	gene	PSMB3	59
apurinic/apyrimidinic endodeoxyribonuclease 1	gene	APEX1	59
annexin A7	gene	ANXA7	59
peptidylprolyl isomerase A	gene	PPIA	59
G3BP stress granule assembly factor 1	gene	G3BP1	59
DDB1 and CUL4 associated factor 1	gene	DCAF1	59

insulin like growth factor 1 receptor	gene	IGF1R	59
DnaJ heat shock protein family (Hsp40) member B1	gene	DNAJB1	58
ribosomal protein L10 like	gene	RPL10L	58
TNF receptor associated factor 3	gene	TRAF3	58
transglutaminase 2	gene	TGM2	58
tripartite motif containing 21	gene	TRIM21	58
casein kinase 1 epsilon	gene	CSNK1E	58
prohibitin 2	gene	PHB2	58
actin like 6A	gene	ACTL6A	58
proteasome subunit beta 4	gene	PSMB4	58
AKT serine/threonine kinase 2	gene	AKT2	58
NFKB inhibitor beta	gene	NFKBIB	58
ubiquitin like with PHD and ring finger domains 1	gene	UHRF1	58
LOC400927-CSNK1E readthrough	gene	LOC400927-CSNK1E	58
transformation/transcription domain associated protein	gene	TRRAP	57
voltage dependent anion channel 1	gene	VDAC1	57
glutamate ionotropic receptor NMDA type subunit 1	gene	GRIN1	57
NOC2 like nucleolar associated transcriptional repressor	gene	NOC2L	57
ERCC excision repair 6, chromatin remodeling factor	gene	ERCC6	57
DEAD-box helicase 24	gene	DDX24	57
serine and arginine rich splicing factor 7	gene	SRSF7	57
lysine acetyltransferase 7	gene	KAT7	57
topoisomerase (DNA) II binding protein 1	gene	TOPBP1	57
BUB1 mitotic checkpoint serine/threonine kinase	gene	BUB1	57
B-cell CLL/lymphoma 6	gene	BCL6	57
eukaryotic translation initiation factor 2 alpha kinase 2	gene	EIF2AK2	57
proteasome 26S subunit, non-ATPase 6	gene	PSMD6	57
high mobility group AT-hook 1	gene	HMGA1	56
ring finger protein 11	gene	RNF11	56
histone cluster 1 H2A family member e	gene	HIST1H2AE	56
mitochondrial ribosomal protein L58	gene	MRPL58	56
ribonucleic acid export 1	gene	RAE1	56
ubiquinol-cytochrome c reductase core protein II	gene	UQCRC2	56
cell cycle and apoptosis regulator 2	gene	CCAR2	56
ETS proto-oncogene 1, transcription factor	gene	ETS1	56
thymidine kinase 1	gene	TK1	56
catenin alpha 1	gene	CTNNA1	56
proteasome 26S subunit, non-ATPase 13	gene	PSMD13	56
plectin	gene	PLEC	56
kinesin family member 11	gene	KIF11	56
histone cluster 1 H2A family member b	gene	HIST1H2AB	56
unc-119 lipid binding chaperone	gene	UNC119	56

squamous cell carcinoma antigen recognized by T-cells 1	gene	SART1	56
ASH2 like histone lysine methyltransferase complex subunit	gene	ASH2L	56
fatty acid synthase	gene	FASN	55
DnaJ heat shock protein family (Hsp40) member C7	gene	DNAJC7	55
cortactin	gene	CTTN	55
regulatory associated protein of MTOR complex 1	gene	RPTOR	55
ATRX, chromatin remodeler	gene	ATRX	55
clusterin	gene	CLU	55
proteasome 26S subunit, non-ATPase 7	gene	PSMD7	55
protein kinase AMP-activated catalytic subunit alpha 1	gene	PRKAA1	55
programmed cell death 6 interacting protein	gene	PDCD6IP	55
Yes associated protein 1	gene	YAP1	55
SEC16 homolog A, endoplasmic reticulum export factor	gene	SEC16A	55
Y-box binding protein 3	gene	YBX3	55
calcyclin binding protein	gene	CACYBP	54
ring finger and WD repeat domain 2	gene	RFWD2	54
WD repeat domain 77	gene	WDR77	54
titin	gene	TTN	54
thyroid hormone receptor, alpha	gene	THRA	54
caspase 9	gene	CASP9	54
replication factor C subunit 1	gene	RFC1	54
RB transcriptional corepressor like 1	gene	RBL1	54
progesterone receptor	gene	PGR	54
Fas cell surface death receptor	gene	FAS	54
euchromatic histone lysine methyltransferase 2	gene	EHMT2	54
NME/NM23 nucleoside diphosphate kinase 2	gene	NME2	54
NME1-NME2 readthrough	gene	NME1-NME2	54
insulin receptor	gene	INSR	54
DnaJ heat shock protein family (Hsp40) member A3	gene	DNAJA3	54
FK506 binding protein 8	gene	FKBP8	53
telomerase reverse transcriptase	gene	TERT	53
HNF1 homeobox A	gene	HNF1A	53
elongin C	gene	ELOC	53
transcriptional adaptor 2A	gene	TADA2A	53
thyroid hormone receptor beta	gene	THRB	53
signal transducer and activator of transcription 5A	gene	STAT5A	53
ribosomal protein S6 kinase B1	gene	RPS6KB1	53
CRK like proto-oncogene, adaptor protein	gene	CRKL	53
prothymosin, alpha	gene	PTMA	53
Aly/REF export factor	gene	ALYREF	53
bromodomain containing 7	gene	BRD7	53

receptor interacting serine/threonine kinase 2	gene	RIPK2	53
SMAD family member 7	gene	SMAD7	53
tubulin beta 2A class IIa	gene	TUBB2A	52
forkhead box O3	gene	FOXO3	52
FK506 binding protein 5	gene	FKBP5	52
tumor protein p53 binding protein 2	gene	TP53BP2	52
ubiquitin like with PHD and ring finger domains 2	gene	UHRF2	52
TRAF2 and NCK interacting kinase	gene	TNIK	52
chromosome segregation 1 like	gene	CSE1L	52
cyclin dependent kinase 6	gene	CDK6	52
protein-L-isoaspartate (D-aspartate) O-methyltransferase	gene	PCMT1	52
aldolase, fructose-bisphosphate A	gene	ALDOA	52
ataxin 3	gene	ATXN3	52
COP9 signalosome subunit 4	gene	COPS4	52
fizzy and cell division cycle 20 related 1	gene	FZR1	52
acidic nuclear phosphoprotein 32 family member A	gene	ANP32A	51
transforming growth factor beta receptor 2	gene	TGFBR2	51
supervillin	gene	SVIL	51
proteasome 26S subunit, non-ATPase 8	gene	PSMD8	51
PPARG coactivator 1 alpha	gene	PPARGC1A	51
ligand of numb-protein X 1	gene	LNX1	51
histone deacetylase 9	gene	HDAC9	51
nucleolar and coiled-body phosphoprotein 1	gene	NOLC1	51
beclin 1	gene	BECN1	51
COP9 signalosome subunit 3	gene	COPS3	51
G protein nucleolar 3	gene	GNL3	50
vav guanine nucleotide exchange factor 1	gene	VAV1	50
runt related transcription factor 1	gene	RUNX1	50
Rho GDP dissociation inhibitor alpha	gene	ARHGDIA	50
actin, alpha 2, smooth muscle, aorta	gene	ACTA2	50
Bloom syndrome RecQ like helicase	gene	BLM	50
baculoviral IAP repeat containing 3	gene	BIRC3	50
albumin	gene	ALB	50
DNA methyltransferase 3 like	gene	DNMT3L	50
myelin basic protein	gene	MBP	50
lamin B1	gene	LMNB1	50
glutamate ionotropic receptor NMDA type subunit 2B	gene	GRIN2B	49
ubiquitin specific peptidase 4	gene	USP4	49
upstream binding transcription factor, RNA polymerase I	gene	UBTF	49
forkhead box O1	gene	FOXO1	49
GRB2 associated binding protein 1	gene	GAB1	49
FtsJ RNA methyltransferase homolog 1 (E. coli)	gene	FTSJ1	49

TNF alpha induced protein 3	gene	TNFAIP3	49
discs large MAGUK scaffold protein 4	gene	DLG4	49
single stranded DNA binding protein 1	gene	SSBP1	49
ninein like	gene	NINL	49
solute carrier family 25 member 3	gene	SLC25A3	49
ATPase Na+/K+ transporting subunit alpha 1	gene	ATP1A1	49
DnaJ heat shock protein family (Hsp40) member A2	gene	DNAJA2	49
translocase of inner mitochondrial membrane 50	gene	TIMM50	49
leucine rich pentatricopeptide repeat containing	gene	LRPPRC	49
nuclear factor, erythroid 2 like 2	gene	NFE2L2	49
MDM4, p53 regulator	gene	MDM4	49
eukaryotic translation initiation factor 3 subunit E	gene	EIF3E	49
B-cell CLL/lymphoma 10	gene	BCL10	48
ubiquitin C-terminal hydrolase L1	gene	UCHL1	48
Fanconi anemia complementation group A	gene	FANCA	48
translocated promoter region, nuclear basket protein	gene	TPR	48
toll like receptor 4	gene	TLR4	48
RALY heterogeneous nuclear ribonucleoprotein	gene	RALY	48
CSK, non-receptor tyrosine kinase	gene	CSK	48
serine/threonine kinase receptor associated protein	gene	STRAP	48
anaplastic lymphoma receptor tyrosine kinase	gene	ALK	48
RPTOR independent companion of MTOR complex 2	gene	RICTOR	48
SET domain containing lysine methyltransferase 7	gene	SETD7	48
interferon regulatory factor 3	gene	IRF3	48
zyxin	gene	ZYX	47
KIAA1549	gene	KIAA1549	47
apoptotic chromatin condensation inducer 1	gene	ACIN1	47
cyclin dependent kinase 7	gene	CDK7	47
ribosomal protein L28	gene	RPL28	47
Parkinsonism associated deglycase	gene	PARK7	47
proteasome subunit beta 7	gene	PSMB7	47
presenilin 1	gene	PSEN1	47
protein kinase C epsilon	gene	PRKCE	47
protein phosphatase, Mg2+/Mn2+ dependent 1B	gene	PPM1B	47
optineurin	gene	OPTN	47
phosphatidylinositol-4,5-bisphosphate 3-kinase catalytic subunit alpha	gene	PIK3CA	47
lysine acetyltransferase 8	gene	KAT8	47
Fas associated via death domain	gene	FADD	47
autophagy related 5	gene	ATG5	47
eukaryotic translation initiation factor 3 subunit I	gene	EIF3I	47
tropomyosin 4	gene	TPM4	46
tropomyosin 1 (alpha)	gene	TPM1	46
erb-b2 receptor tyrosine kinase 4	gene	ERBB4	46

tight junction protein 1	gene	TJP1	46
sterol regulatory element binding transcription factor 1	gene	SREBF1	46
serum response factor	gene	SRF	46
capping actin protein of muscle Z-line beta subunit	gene	CAPZB	46
calcium/calmodulin dependent protein kinase II alpha	gene	CAMK2A	46
EBNA1 binding protein 2	gene	EBNA1BP2	46
POU class 2 homeobox 1	gene	POU2F1	46
WD repeat domain 83	gene	WDR83	46
BCL2 associated athanogene 4	gene	BAG4	46
ribosomal protein L26 like 1	gene	RPL26L1	46
argonaute 2, RISC catalytic component	gene	AGO2	46
TATA-box binding protein associated factor, RNA polymerase I subunit B	gene	TAF1B	46
heat shock protein family A (Hsp70) member 6	gene	HSPA6	45
caspase 1	gene	CASP1	45
calpain 1	gene	CAPN1	45
RB binding protein 5, histone lysine methyltransferase complex subunit	gene	RBBP5	45
adhesion regulating molecule 1	gene	ADRM1	45
protein tyrosine phosphatase, receptor type J	gene	PTPRJ	45
insulin like growth factor 2 mRNA binding protein 1	gene	IGF2BP1	45
B-Raf proto-oncogene, serine/threonine kinase	gene	BRAF	45
aldehyde dehydrogenase 2 family (mitochondrial)	gene	ALDH2	45
adaptor related protein complex 2 beta 1 subunit	gene	AP2B1	45
nucleoporin 153	gene	NUP153	45
kelch like ECH associated protein 1	gene	KEAP1	45
homeodomain interacting protein kinase 2	gene	HIPK2	45
BCL2 family apoptosis regulator	gene	MCL1	45
KH-type splicing regulatory protein	gene	KHSRP	45
metastasis associated 1	gene	MTA1	45
protein inhibitor of activated STAT 2	gene	PIAS2	45
gelsolin	gene	GSN	44
PTEN induced putative kinase 1	gene	PINK1	44
FAU, ubiquitin like and ribosomal protein S30 fusion	gene	FAU	44
small nuclear ribonucleoprotein polypeptides B and B1	gene	SNRPB	44
runt related transcription factor 2	gene	RUNX2	44
par-3 family cell polarity regulator	gene	PARD3	44
collapsin response mediator protein 1	gene	CRMP1	44
nuclear receptor binding SET domain protein 3	gene	NSD3	44
DDB1 and CUL4 associated factor 7	gene	DCAF7	44
amino-terminal enhancer of split	gene	AES	44
ATP citrate lyase	gene	ACLY	44
apoptosis inducing factor mitochondria associated 1	gene	AIFM1	44
microtubule associated protein 1 light chain 3 beta	gene	MAP1LC3B	44

DNA ligase 4	gene	LIG4	44
inosine monophosphate dehydrogenase 2	gene	IMPDH2	44
ubiquitin conjugating enzyme E2 K	gene	UBE2K	43
mutS homolog 6	gene	MSH6	43
AHNAK nucleoprotein	gene	AHNAK	43
tuberous sclerosis 2	gene	TSC2	43
topoisomerase (DNA) II beta	gene	TOP2B	43
inhibitor of Bruton tyrosine kinase	gene	IBTK	43
serpin family H member 1	gene	SERPINH1	43
histone deacetylase 8	gene	HDAC8	43
C-terminal binding protein 2	gene	CTBP2	43
alpha-2-macroglobulin	gene	A2M	43
MYB proto-oncogene, transcription factor	gene	MYB	43
BCL2 associated transcription factor 1	gene	BCLAF1	43
microtubule associated protein 1B	gene	MAP1B	43
Fanconi anemia complementation group C	gene	FANCC	42
eukaryotic translation initiation factor 4A1	gene	EIF4A1	42
eukaryotic translation initiation factor 2 subunit alpha	gene	EIF2S1	42
small nuclear ribonucleoprotein polypeptide A	gene	SNRPA	42
snail family transcriptional repressor 1	gene	SNAI1	42
ring finger protein 20	gene	RNF20	42
small glutamine rich tetratricopeptide repeat containing alpha	gene	SGTA	42
actinin alpha 2	gene	ACTN2	42
RANBP2-type and C3HC4-type zinc finger containing 1	gene	RBCK1	42
TNFAIP3 interacting protein 1	gene	TNIP1	42
programmed cell death 6	gene	PDCD6	42
CASP8 and FADD like apoptosis regulator	gene	CFLAR	41
heat shock protein family A (Hsp70) member 2	gene	HSPA2	41
glial fibrillary acidic protein	gene	GFAP	41
GRB10 interacting GYF protein 2	gene	GIGYF2	41
tropomyosin 2 (beta)	gene	TPM2	41
microtubule affinity regulating kinase 2	gene	MARK2	41
transcription factor AP-4	gene	TFAP4	41
transcription factor CP2	gene	TFCP2	41
anti-silencing function 1A histone chaperone	gene	ASF1A	41
caspase 6	gene	CASP6	41
crystallin alpha B	gene	CRYAB	41
replication factor C subunit 4	gene	RFC4	41
creatine kinase B	gene	CKB	41
UTP14A small subunit processome component	gene	UTP14A	41
BRCA2, DNA repair associated	gene	BRCA2	41
SUB1 homolog, transcriptional regulator	gene	SUB1	41

tyrosine kinase non receptor 2	gene	TNK2	41
peroxisome proliferator activated receptor alpha	gene	PPARA	41
protein phosphatase 2 phosphatase activator	gene	PTPA	41
BCAR1, Cas family scaffolding protein	gene	BCAR1	41
forkhead box K2	gene	FOXK2	41
forkhead box B1	gene	FOXB1	40
hydroxyacyl-CoA dehydrogenase/3-ketoacyl-CoA thiolase/enoyl-CoA hydratase (trifunctional protein), beta subunit	gene	HADHB	40
inhibitor of DNA binding 2, HLH protein	gene	ID2	40
glutathione S-transferase kappa 1	gene	GSTK1	40
lysine acetyltransferase 6A	gene	KAT6A	40
nuclear receptor subfamily 0 group B member 2	gene	NR0B2	40
transcription factor AP-2 alpha	gene	TFAP2A	40
l(3)mbt-like 1 (Drosophila)	gene	L3MBTL1	40
treacle ribosome biogenesis factor 1	gene	TCOF1	40
CD44 molecule (Indian blood group)	gene	CD44	40
BCL2 associated agonist of cell death	gene	BAD	40
scaffold attachment factor B	gene	SAFB	40
DEAD-box helicase 19B	gene	DDX19B	40
RB transcriptional corepressor like 2	gene	RBL2	40
activating transcription factor 3	gene	ATF3	40
insulin like growth factor 2 mRNA binding protein 3	gene	IGF2BP3	40
BH3 interacting domain death agonist	gene	BID	40
centrosomal protein 55	gene	CEP55	40
forkhead box K1	gene	FOXK1	40
G protein-coupled receptor kinase 2	gene	GRK2	40
phospholipase C gamma 2	gene	PLCG2	40
major vault protein	gene	MVP	40
protein phosphatase 2 regulatory subunit Balpha	gene	PPP2R2A	40
COP9 signalosome subunit 7A	gene	COPS7A	40
RB1 inducible coiled-coil 1	gene	RB1CC1	40
centrosomal protein 135	gene	CEP135	40
myosin VI	gene	MYO6	40
inhibitor of growth family member 2	gene	ING2	40
TATA-box binding protein associated factor, RNA polymerase I subunit A	gene	TAF1A	40
nuclear receptor interacting protein 1	gene	NRIP1	39
nuclear receptor binding SET domain protein 1	gene	NSD1	39
general transcription factor IIB	gene	GTF2B	39
SRP receptor beta subunit	gene	SRPRB	39
transferrin receptor	gene	TFRC	39
aminoacyl tRNA synthetase complex interacting multifunctional protein 2	gene	AIMP2	39

dynamin 1	gene	DNM1	39
SOS Ras/Rac guanine nucleotide exchange factor 1	gene	SOS1	39
calumenin	gene	CALU	39
HAUS augmin like complex subunit 1	gene	HAUS1	39
zinc fingers and homeoboxes 1	gene	ZHX1	39
Ras association domain family member 1	gene	RASSF1	39
ATPase sarcoplasmic/endoplasmic reticulum Ca2+ transporting 2	gene	ATP2A2	39
phosphatidylethanolamine binding protein 1	gene	PEBP1	39
proteasome 26S subunit, non-ATPase 10	gene	PSMD10	39
ubiquitin interaction motif containing 1	gene	UIMC1	39
mediator complex subunit 16	gene	MED16	39
TNF receptor associated factor 4	gene	TRAF4	39
dual specificity tyrosine phosphorylation regulated kinase 1B	gene	DYRK1B	39
inhibitor of growth family member 1	gene	ING1	39
phosphatidylinositol binding clathrin assembly protein	gene	PICALM	38
mismatch repair endonuclease PMS2	gene	LOC107984056	38
vasodilator-stimulated phosphoprotein	gene	VASP	38
FK506 binding protein 1A	gene	FKBP1A	38
DAZ associated protein 1	gene	DAZAP1	38
TATA-box binding protein associated factor 10	gene	TAF10	38
SPT6 homolog, histone chaperone	gene	SUPT6H	38
atrophin 1	gene	ATN1	38
CCR4-NOT transcription complex subunit 1	gene	CNOT1	38
BCL2 associated athanogene 1	gene	BAG1	38
ret proto-oncogene	gene	RET	38
RAB5A, member RAS oncogene family	gene	RAB5A	38
pterin-4 alpha-carbinolamine dehydratase 1	gene	PCBD1	38
mitogen-activated protein kinase 7	gene	MAPK7	38
protein kinase N1	gene	PKN1	38
protein phosphatase 4 catalytic subunit	gene	PPP4C	38
PMS1 homolog 2, mismatch repair system component	gene	PMS2	38
synoviolin 1	gene	SYVN1	38
macrophage migration inhibitory factor (glycosylation-inhibiting factor)	gene	MIF	38
eukaryotic translation initiation factor 3 subunit C	gene	EIF3C	38
suppressor of cytokine signaling 1	gene	SOCS1	38
G protein subunit beta 1	gene	GNB1	37
XPC complex subunit, DNA damage recognition and repair factor	gene	XPC	37
ubiquitin fusion degradation 1 like (yeast)	gene	UFD1L	37
Fanconi anemia complementation group D2	gene	FANCD2	37
nuclear receptor subfamily 2 group C member 2	gene	NR2C2	37

enhancer of rudimentary homolog (Drosophila)	gene	ERH	37
enolase 2	gene	ENO2	37
GTP binding protein 4	gene	GTPBP4	37
pescadillo ribosomal biogenesis factor 1	gene	PES1	37
elongin B	gene	ELOB	37
sterol regulatory element binding transcription factor 2	gene	SREBF2	37
protein tyrosine phosphatase, non-receptor type 12	gene	PTPN12	37
N-acetyltransferase 10	gene	NAT10	37
collagen type I alpha 2 chain	gene	COL1A2	37
protein kinase C gamma	gene	PRKCG	37
protein phosphatase 3 catalytic subunit alpha	gene	PPP3CA	37
mesenchyme homeobox 2	gene	MEOX2	37
myocyte enhancer factor 2A	gene	MEF2A	37
karyopherin subunit alpha 4	gene	KPNA4	37
ferritin heavy chain 1	gene	FTH1	36
vitronectin	gene	VTN	36
glycogen synthase kinase 3 alpha	gene	GSK3A	36
WNK lysine deficient protein kinase 1	gene	WNK1	36
mitochondrial ribosomal protein S9	gene	MRPS9	36
tuberous sclerosis 1	gene	TSC1	36
erythropoietin receptor	gene	EPOR	36
signal sequence receptor subunit 4	gene	SSR4	36
S100 calcium binding protein A8	gene	S100A8	36
erbb2 interacting protein	gene	ERBIN	36
Kruppel like factor 5	gene	KLF5	36
basigin (Ok blood group)	gene	BSG	36
cytoskeleton associated protein 4	gene	CKAP4	36
A-kinase anchoring protein 8	gene	AKAP8	36
ERBB receptor feedback inhibitor 1	gene	ERRFI1	36
G3BP stress granule assembly factor 2	gene	G3BP2	36
Mov10 RISC complex RNA helicase	gene	MOV10	36
SLC9A3 regulator 1	gene	SLC9A3R1	36
mediator complex subunit 14	gene	MED14	36
mediator complex subunit 21	gene	MED21	36
insulin receptor substrate 2	gene	IRS2	36
nuclear receptor subfamily 1 group I member 2	gene	NR1I2	35
G protein subunit alpha i2	gene	GNAI2	35
dual specificity tyrosine phosphorylation regulated kinase 2	gene	DYRK2	35
adaptor protein, phosphotyrosine interacting with PH domain and leucine zipper 1	gene	APPL1	35
proline rich coiled-coil 2A	gene	PRRC2A	35
tumor protein, translationally-controlled 1	gene	TPT1	35
transcription factor AP-2 gamma	gene	TFAP2C	35

discs large MAGUK scaffold protein 1	gene	DLG1	35
DCC netrin 1 receptor	gene	DCC	35
death associated protein kinase 1	gene	DAPK1	35
glutamate receptor interacting protein 1	gene	GRIP1	35
mitogen-activated protein kinase kinase 4	gene	MAP2K4	35
signal transducer and activator of transcription 5B	gene	STAT5B	35
tripartite motif containing 32	gene	TRIM32	35
TIR domain containing adaptor protein	gene	TIRAP	35
CYLD lysine 63 deubiquitinase	gene	CYLD	35
casein kinase 1 delta	gene	CSNK1D	35
RB binding protein 8, endonuclease	gene	RBBP8	35
cell division cycle 34	gene	CDC34	35
cadherin 2	gene	CDH2	35
GABA type A receptor-associated protein	gene	GABARAP	35
lysine demethylase 5B	gene	KDM5B	35
BTG3 associated nuclear protein	gene	BANP	35
ubiquitin specific peptidase 39	gene	USP39	35
pyruvate dehydrogenase (lipoamide) beta	gene	PDHB	35
origin recognition complex subunit 4	gene	ORC4	35
mitogen-activated protein kinase kinase 7	gene	MAP2K7	35
ring finger protein 31	gene	RNF31	35
peptidylprolyl isomerase D	gene	PPID	35
denticleless E3 ubiquitin protein ligase homolog	gene	DTL	35
protein phosphatase 1 regulatory subunit 12A	gene	PPP1R12A	35
mitogen-activated protein kinase-activated protein kinase 2	gene	MAPKAPK2	35
ubiquitin specific peptidase 8	gene	USP8	35
mutL homolog 3	gene	MLH3	34
cell division cycle 45	gene	CDC45	34
AT-rich interaction domain 1A	gene	ARID1A	34
heat shock protein family B (small) member 2	gene	HSPB2	34
GLI family zinc finger 1	gene	GLI1	34
serine/threonine kinase 24	gene	STK24	34
XPA, DNA damage recognition and repair factor	gene	XPA	34
glutamate ionotropic receptor NMDA type subunit 2D	gene	GRIN2D	34
fibroblast growth factor receptor 1	gene	FGFR1	34
coronin 1C	gene	CORO1C	34
reticulon 4	gene	RTN4	34
TATA-box binding protein associated factor 6	gene	TAF6	34
secreted phosphoprotein 1	gene	SPP1	34
ribosomal RNA processing 1B	gene	RRP1B	34
coordinator of PRMT5 and differentiation stimulator	gene	COPRS	34
adenosine deaminase, RNA specific	gene	ADAR	34
origin recognition complex subunit 5	gene	ORC5	34

MAGE family member D2	gene	MAGED2	34
mitogen-activated protein kinase 10	gene	MAPK10	34
BCL2 like 11	gene	BCL2L11	34
transcriptional adaptor 3	gene	TADA3	34
mediator complex subunit 24	gene	MED24	34
CXXC finger protein 1	gene	CXXC1	34
JunD proto-oncogene, AP-1 transcription factor subunit	gene	JUND	34
mediator complex subunit 17	gene	MED17	34
ubiquitin specific peptidase 2	gene	USP2	34
integrin subunit beta 3	gene	ITGB3	34
spindle and centriole associated protein 1	gene	SPICE1	33
copper metabolism domain containing 1	gene	COMMD1	33
ubiquitin conjugating enzyme E2 M	gene	UBE2M	33
GATA binding protein 1	gene	GATA1	33
G protein pathway suppressor 1	gene	GPS1	33
transmembrane protein 135	gene	TMEM135	33
forkhead box G1	gene	FOXG1	33
apoptosis antagonizing transcription factor	gene	AATF	33
TIA1 cytotoxic granule associated RNA binding protein like 1	gene	TIAL1	33
ELK1, ETS transcription factor	gene	ELK1	33
serine and arginine rich splicing factor 6	gene	SRSF6	33
caspase 10	gene	CASP10	33
suppression of tumorigenicity 13 (colon carcinoma) (Hsp70 interacting protein)	gene	ST13	33
S100 calcium binding protein A4	gene	S100A4	33
NPL4 homolog, ubiquitin recognition factor	gene	NPLOC4	33
chromosome 14 open reading frame 1	gene	C14orf1	33
reticulocalbin 1	gene	RCN1	33
AT-rich interaction domain 4A	gene	ARID4A	33
cell division cycle 6	gene	CDC6	33
argininosuccinate synthase 1	gene	ASS1	33
ring finger protein 4	gene	RNF4	33
collagen type I alpha 1 chain	gene	COL1A1	33
BCAS3, microtubule associated cell migration factor	gene	BCAS3	33
clathrin light chain A	gene	CLTA	33
bone morphogenetic protein receptor type 1A	gene	BMPR1A	33
ubiquitination factor E4B	gene	UBE4B	33
protein kinase AMP-activated catalytic subunit alpha 2	gene	PRKAA2	33
DnaJ heat shock protein family (Hsp40) member B6	gene	DNAJB6	33
N-ethylmaleimide sensitive factor, vesicle fusing ATPase	gene	NSF	33
toll interacting protein	gene	TOLLIP	33

tubulin beta 6 class V	gene	TUBB6	33
protein kinase C and casein kinase substrate in neurons 3	gene	PACSIN3	33
WD repeat domain 82	gene	WDR82	33
lysyl-tRNA synthetase	gene	KARS	33
eukaryotic translation initiation factor 2 subunit beta	gene	EIF2S2	32
four and a half LIM domains 1	gene	FHL1	32
ring finger and CHY zinc finger domain containing 1	gene	RCHY1	32
mitochondrial ribosomal protein S22	gene	MRPS22	32
WD repeat domain 48	gene	WDR48	32
serine/threonine kinase 3	gene	STK3	32
serine/threonine kinase 4	gene	STK4	32
arginine demethylase and lysine hydroxylase	gene	JMJD6	32
ring finger protein 1	gene	RING1	32
mitogen-activated protein kinase kinase kinase kinase 1	gene	MAP4K1	32
protein tyrosine phosphatase, receptor type C	gene	PTPRC	32
zinc finger MYND-type containing 11	gene	ZMYND11	32
phosphofructokinase, muscle	gene	PFKM	32
COP9 signalosome subunit 8	gene	COPS8	32
protein kinase D1	gene	PRKD1	32
protein arginine methyltransferase 3	gene	PRMT3	32
nerve growth factor receptor	gene	NGFR	32
keratin 8	gene	KRT8	32
KRAS proto-oncogene, GTPase	gene	KRAS	32
methionine adenosyltransferase 2A	gene	MAT2A	32
basic helix-loop-helix family member e40	gene	BHLHE40	32
euchromatic histone lysine methyltransferase 1	gene	EHMT1	32
ring finger protein 8	gene	RNF8	32
F-box and WD repeat domain containing 8	gene	FBXW8	31
twist family bHLH transcription factor 1	gene	TWIST1	31
four and a half LIM domains 3	gene	FHL3	31
thymine DNA glycosylase	gene	TDG	31
RPGRIP1 like	gene	RPGRIP1L	31
caspase 4	gene	CASP4	31
cell division cycle and apoptosis regulator 1	gene	CCAR1	31
RB binding protein 6, ubiquitin ligase	gene	RBBP6	31
chloride nucleotide-sensitive channel 1A	gene	CLNS1A	31
CDC like kinase 2	gene	CLK2	31
CDC like kinase 3	gene	CLK3	31
BCAS2, pre-mRNA processing factor	gene	BCAS2	31
mitogen-activated protein kinase kinase 2	gene	MAP2K2	31
pentatricopeptide repeat domain 3	gene	PTCD3	31
adaptor related protein complex 1 beta 1 subunit	gene	AP1B1	31

N-myristoyltransferase 1	gene	NMT1	31
scaffold attachment factor B2	gene	SAFB2	31
ninein	gene	NIN	31
peroxiredoxin 6	gene	PRDX6	31
myeloid differentiation primary response 88	gene	MYD88	31
mitochondrial ribosomal protein S28	gene	MRPS28	31
interferon regulatory factor 7	gene	IRF7	31
major histocompatibility complex, class I, C	gene	HLA-C	30
Werner syndrome RecQ like helicase	gene	WRN	30
general transcription factor IIH subunit 1	gene	GTF2H1	30
mitogen-activated protein kinase-activated protein kinase 3	gene	MAPKAPK3	30
TTK protein kinase	gene	TTK	30
eukaryotic translation initiation factor 4E binding protein 1	gene	EIF4EBP1	30
superoxide dismutase 2	gene	SOD2	30
SWI/SNF related, matrix associated, actin dependent regulator of chromatin, subfamily d, member 2	gene	SMARCD2	30
ubiquitin protein ligase E3 component n-recognin 4	gene	UBR4	30
cyclin H	gene	CCNH	30
signal transducer and activator of transcription 6	gene	STAT6	30
Rho associated coiled-coil containing protein kinase 1	gene	ROCK1	30
baculoviral IAP repeat containing 5	gene	BIRC5	30
presenilin 2	gene	PSEN2	30
mitogen-activated protein kinase 11	gene	MAPK11	30
TOP1 binding arginine/serine rich protein	gene	TOPORS	30
late endosomal/lysosomal adaptor, MAPK and MTOR activator 5	gene	LAMTOR5	30
GRB2 associated binding protein 2	gene	GAB2	30
Rho GTPase activating protein 32	gene	ARHGAP32	30
neutrophil cytosolic factor 1C pseudogene	gene	NCF1C	30
inhibitor of growth family member 4	gene	ING4	30
MYB proto-oncogene like 2	gene	MYBL2	30
neutrophil cytosolic factor 1	gene	NCF1	30
stathmin 1	gene	STMN1	30
Rho/Rac guanine nucleotide exchange factor 2	gene	ARHGEF2	30
lymphotoxin beta receptor	gene	LTBR	30
lysine methyltransferase 5A	gene	KMT5A	29
G protein subunit alpha i1	gene	GNAI1	29
G protein pathway suppressor 2	gene	GPS2	29
actin, beta like 2	gene	ACTBL2	29
charged multivesicular body protein 4B	gene	CHMP4B	29
ubiquitin conjugating enzyme E2 A	gene	UBE2A	29
dicer 1, ribonuclease III	gene	DICER1	29

calcium/calmodulin dependent protein kinase II gamma	gene	CAMK2G	29
receptor-like tyrosine kinase	gene	RYK	29
ribonucleotide reductase catalytic subunit M1	gene	RRM1	29
coiled-coil domain containing 88A	gene	CCDC88A	29
RE1 silencing transcription factor	gene	REST	29
RAS like proto-oncogene A	gene	RALA	29
cell division cycle 25C	gene	CDC25C	29
adducin 1	gene	ADD1	29
platelet activating factor acetylhydrolase 1b catalytic subunit 3	gene	PAFAH1B3	29
dedicator of cytokinesis 7	gene	DOCK7	29
CCAAT/enhancer binding protein zeta	gene	CEBPZ	29
protein phosphatase 2 regulatory subunit Bbeta	gene	PPP2R2B	29
MNAT1, CDK activating kinase assembly factor	gene	MNAT1	29
eukaryotic translation initiation factor 5B	gene	EIF5B	29
Sec61 translocon alpha 1 subunit	gene	SEC61A1	29
general transcription factor IIIC subunit 3	gene	GTF3C3	29
keratin 10	gene	KRT10	29
drosha ribonuclease III	gene	DROSHA	29
microtubule associated protein 2	gene	MAP2	29
USO1 vesicle transport factor	gene	USO1	29
toll like receptor adaptor molecule 1	gene	TICAM1	28
zinc finger protein 24	gene	ZNF24	28
GATA binding protein 2	gene	GATA2	28
X-ray repair cross complementing 1	gene	XRCC1	28
WEE1 G2 checkpoint kinase	gene	WEE1	28
FK506 binding protein 3	gene	FKBP3	28
tropomodulin 1	gene	TMOD1	28
EPH receptor B2	gene	EPHB2	28
metastasis associated 1 family member 3	gene	MTA3	28
solute carrier family 3 member 2	gene	SLC3A2	28
caspase 2	gene	CASP2	28
mitochondrial ribosomal protein S27	gene	MRPS27	28
calcium/calmodulin dependent protein kinase II delta	gene	CAMK2D	28
BTB domain and CNC homolog 1	gene	BACH1	28
cleavage stimulation factor subunit 2	gene	CSTF2	28
BTB domain containing 2	gene	BTBD2	28
forkhead box Q1	gene	FOXQ1	28
protein tyrosine phosphatase, receptor type F	gene	PTPRF	28
cyclin dependent kinase 11B	gene	CDK11B	28
mitogen-activated protein kinase kinase kinase 8	gene	MAP3K8	28
protein kinase N2	gene	PKN2	28
protein kinase C theta	gene	PRKCQ	28

protein phosphatase, Mg2+/Mn2+ dependent 1A	gene	PPM1A	28
B-cell receptor-associated protein 31	gene	BCAP31	28
DGCR8, microprocessor complex subunit	gene	DGCR8	28
nitric oxide synthase 3	gene	NOS3	28
DLC1 Rho GTPase activating protein	gene	DLC1	28
cytochrome c, somatic	gene	CYCS	28
nuclear transcription factor Y subunit alpha	gene	NFYA	28
nuclear receptor subfamily 1 group I member 3	gene	NR1I3	28
mitogen-activated protein kinase kinase kinase 11	gene	MAP3K11	28
class II major histocompatibility complex transactivator	gene	CIITA	28
aminopeptidase puromycin sensitive	gene	NPEPPS	28
lysosomal associated membrane protein 2	gene	LAMP2	28
thyroid hormone receptor interactor 4	gene	TRIP4	28
NUMB, endocytic adaptor protein	gene	NUMB	28
high mobility group box 2	gene	HMGB2	27
vaccinia related kinase 1	gene	VRK1	27
glutathione S-transferase pi 1	gene	GSTP1	27
lysine demethylase 6A	gene	KDM6A	27
growth factor receptor bound protein 10	gene	GRB10	27
nuclear receptor subfamily 5 group A member 1	gene	NR5A1	27
toll like receptor 2	gene	TLR2	27
integrin subunit beta 3 binding protein	gene	ITGB3BP	27
centrosomal protein 152	gene	CEP152	27
spermidine/spermine N1-acetyltransferase 1	gene	SAT1	27
retinoid X receptor beta	gene	RXRB	27
ERCC excision repair 8, CSA ubiquitin ligase complex subunit	gene	ERCC8	27
minichromosome maintenance 10 replication initiation factor	gene	MCM10	27
prostaglandin-endoperoxide synthase 2	gene	PTGS2	27
protein phosphatase 2 regulatory subunit B'gamma	gene	PPP2R5C	27
transducer of ERBB2, 1	gene	TOB1	27
A-kinase anchoring protein 9	gene	AKAP9	27
nemo like kinase	gene	NLK	27
neurofilament light	gene	NEFL	27
TBC1 domain family member 4	gene	TBC1D4	27
mucin 1, cell surface associated	gene	MUC1	27
endothelial differentiation related factor 1	gene	EDF1	27
ubiquitin specific peptidase 10	gene	USP10	27
microcephalin 1	gene	MCPH1	27
integrin subunit alpha V	gene	ITGAV	26
hyaluronan mediated motility receptor	gene	HMMR	26
BRCA1/BRCA2-containing complex subunit 3	gene	BRCC3	26
sorbin and SH3 domain containing 2	gene	SORBS2	26

glutamate ionotropic receptor NMDA type subunit 2A	gene	GRIN2A	26
ubiquitin conjugating enzyme E2 B	gene	UBE2B	26
tuftelin interacting protein 11	gene	TFIP11	26
fatty acid binding protein 5	gene	FABP5	26
DNA fragmentation factor subunit alpha	gene	DFFA	26
death associated protein kinase 3	gene	DAPK3	26
ubiquitin specific peptidase 22	gene	USP22	26
SIN3 transcription regulator family member B	gene	SIN3B	26
baculoviral IAP repeat containing 6	gene	BIRC6	26
tripartite motif containing 39	gene	TRIM39	26
cysteinyl-tRNA synthetase	gene	CARS	26
calpain small subunit 1	gene	CAPNS1	26
cathepsin D	gene	CTSD	26
SH3 domain containing GRB2 like 1, endophilin A2	gene	SH3GL1	26
ZW10 interacting kinetochore protein	gene	ZWINT	26
dynein axonemal assembly factor 5	gene	DNAAF5	26
cyclin dependent kinase 11A	gene	CDK11A	26
autophagy related 16 like 1	gene	ATG16L1	26
phospholipase D1	gene	PLD1	26
ariadne RBR E3 ubiquitin protein ligase 2	gene	ARIH2	26
RNA polymerase I subunit B	gene	POLR1B	26
telomere maintenance 2	gene	TELO2	26
SUMO1 activating enzyme subunit 1	gene	SAE1	26
PHD finger protein 20	gene	PHF20	26
CREB regulated transcription coactivator 2	gene	CRTC2	26
ubiquitin protein ligase E3 component n-recognin 1	gene	UBR1	26
Kruppel like factor 4	gene	KLF4	26
hydroxysteroid 17-beta dehydrogenase 4	gene	HSD17B4	25
forkhead box S1	gene	FOXS1	25
argonaute 1, RISC catalytic component	gene	AGO1	25
chromodomain helicase DNA binding protein 8	gene	CHD8	25
epidermal growth factor	gene	EGF	25
tec protein tyrosine kinase	gene	TEC	25
scribbled planar cell polarity protein	gene	SCRIB	25
golgi reassembly stacking protein 2	gene	GORASP2	25
discs large MAGUK scaffold protein 3	gene	DLG3	25
transgelin	gene	TAGLN	25
Ras homolog enriched in brain	gene	RHEB	25
PC4 and SFRS1 interacting protein 1	gene	PSIP1	25
reticulocalbin 2	gene	RCN2	25
WD repeat domain 33	gene	WDR33	25
phosphodiesterase 4D	gene	PDE4D	25
tripartite motif containing 15	gene	TRIM15	25
clathrin light chain B	gene	CLTB	25

BLK proto-oncogene, Src family tyrosine kinase	gene	BLK	25
male-specific lethal 2 homolog (Drosophila)	gene	MSL2	25
immunoglobin superfamily member 21	gene	IGSF21	25
mitochondrial ribosomal protein S23	gene	MRPS23	25
phospholipase A2 group IVA	gene	PLA2G4A	25
cAMP responsive element binding protein 3	gene	CREB3	25
neuroblastoma RAS viral oncogene homolog	gene	NRAS	25
nardilysin convertase	gene	NRDC	25
centrosomal protein 350	gene	CEP350	25
ring finger protein 40	gene	RNF40	25
deoxynucleotidyltransferase terminal interacting protein 2	gene	DNTTIP2	25
mediator complex subunit 7	gene	MED7	25
chromodomain Y-like	gene	CDYL	25
zinc finger and BTB domain containing 7A	gene	ZBTB7A	25
ubiquitin like modifier activating enzyme 5	gene	UBA5	25
calcium/calmodulin dependent serine protein kinase	gene	CASK	25
heme oxygenase 1	gene	HMOX1	24
ZFP36 ring finger protein	gene	ZFP36	24
lysine methyltransferase 2C	gene	KMT2C	24
endoplasmic reticulum to nucleus signaling 1	gene	ERN1	24
Sp3 transcription factor	gene	SP3	24
mitogen-activated protein kinase 8 interacting protein 3	gene	MAPK8IP3	24
diablo IAP-binding mitochondrial protein	gene	DIABLO	24
calpastatin	gene	CAST	24
BCL2 antagonist/killer 1	gene	BAK1	24
mitogen-activated protein kinase 12	gene	MAPK12	24
ribosomal protein S6 kinase A2	gene	RPS6KA2	24
SH3 domain containing GRB2 like 3, endophilin A3	gene	SH3GL3	24
RAP1B, member of RAS oncogene family	gene	RAP1B	24
PHD finger protein 1	gene	PHF1	24
Fanconi anemia complementation group I	gene	FANCI	24
6-phosphofructo-2-kinase/fructose-2,6-biphosphatase 2	gene	PFKFB2	24
OS9, endoplasmic reticulum lectin	gene	OS9	24
protein phosphatase 6 catalytic subunit	gene	PPP6C	24
nucleotide binding oligomerization domain containing 1	gene	NOD1	24
caspase recruitment domain family member 11	gene	CARD11	24
NADH:ubiquinone oxidoreductase core subunit S1	gene	NDUFS1	24
HECT and RLD domain containing E3 ubiquitin protein ligase 5	gene	HERC5	24
eukaryotic translation elongation factor 1 epsilon 1	gene	EEF1E1	24
SUMO1/sentrin specific peptidase 1	gene	SENP1	24
thyroid hormone receptor interactor 12	gene	TRIP12	24

formin homology 2 domain containing 1	gene	FHOD1	24
mitogen-activated protein kinase kinase kinase kinase 4	gene	MAP4K4	24
MAP kinase interacting serine/threonine kinase 1	gene	MKNK1	24
insulin like growth factor binding protein 3	gene	IGFBP3	24
forkhead box A3	gene	FOXA3	23
SNRPN upstream reading frame	gene	SNURF	23
MAP kinase interacting serine/threonine kinase 2	gene	MKNK2	23
PR/SET domain 2	gene	PRDM2	23
zinc finger and BTB domain containing 17	gene	ZBTB17	23
ataxin 1 like	gene	ATXN1L	23
E1A binding protein p400	gene	EP400	23
RING1 and YY1 binding protein	gene	RYBP	23
calpain 2	gene	CAPN2	23
hyaluronan binding protein 4	gene	HABP4	23
SH3 domain containing GRB2 like 2, endophilin A1	gene	SH3GL2	23
CDC28 protein kinase regulatory subunit 1B	gene	CKS1B	23
RAN binding protein 1	gene	RANBP1	23
lysine methyltransferase 2E	gene	KMT2E	23
peroxisomal biogenesis factor 5	gene	PEX5	23
CDKN2A interacting protein	gene	CDKN2AIP	23
nuclear assembly factor 1 ribonucleoprotein	gene	NAF1	23
Sec61 translocon beta subunit	gene	SEC61B	23
fem-1 homolog B	gene	FEM1B	23
acetyl-CoA acetyltransferase 2	gene	ACAT2	23
golgi to ER traffic protein 4	gene	GET4	23
lamin B2	gene	LMNB2	23
tRNA methyltransferase O	gene	TRMO	23
geminin, DNA replication inhibitor	gene	GMNN	23
serpin family B member 9	gene	SERPINB9	23
macrophage stimulating 1 receptor	gene	MST1R	23
activating signal cointegrator 1 complex subunit 2	gene	ASCC2	23
brain and reproductive organ-expressed (TNFRSF1A modulator)	gene	BRE	23
pituitary tumor-transforming 1	gene	PTTG1	23
interleukin 1 receptor associated kinase 2	gene	IRAK2	23
protein activator of interferon induced protein kinase EIF2AK2	gene	PRKRA	23
N-myc and STAT interactor	gene	NMI	23
coiled-coil domain containing 33	gene	CCDC33	22
guanine monophosphate synthase	gene	GMPS	22
SPT3 homolog, SAGA and STAGA complex component	gene	SUPT3H	22
gap junction protein alpha 1	gene	GJA1	22

speckle type BTB/POZ protein	gene	SPOP	22
exocyst complex component 4	gene	EXOC4	22
polycomb group ring finger 2	gene	PCGF2	22
ETS proto-oncogene 2, transcription factor	gene	ETS2	22
ERCC excision repair 3, TFIIH core complex helicase subunit	gene	ERCC3	22
early growth response 1	gene	EGR1	22
ribonucleoprotein, PTB binding 1	gene	RAVER1	22
HECT domain E3 ubiquitin protein ligase 1	gene	HECTD1	22
dystroglycan 1	gene	DAG1	22
small nuclear ribonucleoprotein polypeptide N	gene	SNRPN	22
MYC binding protein 2, E3 ubiquitin protein ligase	gene	MYCBP2	22
signal recognition particle 72	gene	SRP72	22
enabled homolog (Drosophila)	gene	ENAH	22
replication factor C subunit 3	gene	RFC3	22
RAD9 checkpoint clamp component A	gene	RAD9A	22
centromere protein E	gene	CENPE	22
activating transcription factor 7	gene	ATF7	22
protein tyrosine phosphatase, receptor type B	gene	PTPRB	22
zinc finger protein 510	gene	ZNF510	22
phosphoglucomutase 1	gene	PGM1	22
exosome component 8	gene	EXOSC8	22
YTH N6-methyladenosine RNA binding protein 1	gene	YTHDF1	22
fermitin family member 2	gene	FERMT2	22
growth arrest and DNA damage inducible gamma	gene	GADD45G	22
apolipoprotein A1	gene	APOA1	22
3-hydroxyacyl-CoA dehydratase 3	gene	HACD3	22
phosphatidylinositol-4,5-bisphosphate 3-kinase catalytic subunit gamma	gene	PIK3CG	22
protein phosphatase 1 regulatory subunit 9B	gene	PPP1R9B	22
nuclear transcription factor Y subunit beta	gene	NFYB	22
ATR interacting protein	gene	ATRIP	22
forkhead box O4	gene	FOXO4	22
mitogen-activated protein kinase kinase kinase 10	gene	MAP3K10	22
glutamate rich WD repeat containing 1	gene	GRWD1	22
SERTA domain containing 1	gene	SERTAD1	22
malate dehydrogenase 1	gene	MDH1	22
zinc finger FYVE-type containing 9	gene	ZFYVE9	22
SET domain containing 2	gene	SETD2	22
interleukin 6 signal transducer	gene	IL6ST	22
DAB2 interacting protein	gene	DAB2IP	22
immunoglobulin heavy constant gamma 1 (G1m marker)	gene	IGHG1	22
granzyme B	gene	GZMB	21

baculoviral IAP repeat containing 7	gene	BIRC7	21
RAN binding protein 3	gene	RANBP3	21
glucosylceramidase beta	gene	GBA	21
TNFAIP3 interacting protein 2	gene	TNIP2	21
mitochondrial ribosomal protein L38	gene	MRPL38	21
FK506 binding protein 2	gene	FKBP2	21
tribbles pseudokinase 3	gene	TRIB3	21
erythrocyte membrane protein band 4.1 like 2	gene	EPB41L2	21
TGFB induced factor homeobox 1	gene	TGIF1	21
eukaryotic translation initiation factor 1A, X-linked	gene	EIF1AX	21
enoyl-CoA hydratase and 3-hydroxyacyl CoA dehydrogenase	gene	EHHADH	21
fibroblast growth factor 2	gene	FGF2	21
thyroid hormone responsive	gene	THRSP	21
caldesmon 1	gene	CALD1	21
cyclin dependent kinase inhibitor 2C	gene	CDKN2C	21
protein tyrosine phosphatase, receptor type G	gene	PTPRG	21
3-phosphoinositide dependent protein kinase 1	gene	PDPK1	21
patched 1	gene	PTCH1	21
ring finger and WD repeat domain 3	gene	RFWD3	21
DNA polymerase alpha 1, catalytic subunit	gene	POLA1	21
DEAF1, transcription factor	gene	DEAF1	21
phosphatidylinositol-4,5-bisphosphate 3-kinase catalytic subunit delta	gene	PIK3CD	21
mitochondrial ribosomal protein S2	gene	MRPS2	21
eukaryotic translation initiation factor 1A, X-chromosomal	gene	LOC107984923	21
general transcription factor IIIC subunit 4	gene	GTF3C4	21
drebrin like	gene	DBNL	21
mitochondrial ribosomal protein S18B	gene	MRPS18B	21
CCR4-NOT transcription complex subunit 9	gene	CNOT9	21
mitogen-activated protein kinase 8 interacting protein 1	gene	MAPK8IP1	21
tripartite motif containing 8	gene	TRIM8	21
serine/threonine kinase 16	gene	STK16	21
LIM domain only 4	gene	LMO4	21
high mobility group nucleosome binding domain 1	gene	HMGN1	20
huntingtin interacting protein 1	gene	HIP1	20
kinase suppressor of ras 1	gene	KSR1	20
mitochondrial ribosomal protein L24	gene	MRPL24	20
cell division cycle 7	gene	CDC7	20
homeobox D13	gene	HOXD13	20
derlin 1	gene	DERL1	20
vaccinia related kinase 2	gene	VRK2	20
golgi associated, gamma adaptin ear containing, ARF	gene	GGA1	20

binding protein 1			
DNA polymerase delta interacting protein 2	gene	POLDIP2	20
mitochondrial ribosomal protein S5	gene	MRPS5	20
thymosin beta 4, X-linked	gene	TMSB4X	20
thrombospondin 1	gene	THBS1	20
eukaryotic translation initiation factor 2B subunit alpha	gene	EIF2B1	20
secreted frizzled related protein 4	gene	SFRP4	20
cyclin D2	gene	CCND2	20
calcium/calmodulin dependent protein kinase II beta	gene	CAMK2B	20
syndecan 2	gene	SDC2	20
target of EGR1, member 1 (nuclear)	gene	TOE1	20
sex hormone binding globulin	gene	SHBG	20
microtubule associated protein 1S	gene	MAP1S	20
BMX non-receptor tyrosine kinase	gene	BMX	20
ralA binding protein 1	gene	RALBP1	20
apoptotic peptidase activating factor 1	gene	APAF1	20
prion protein	gene	PRNP	20
coiled-coil domain containing 180	gene	CCDC180	20
mitogen-activated protein kinase kinase 6	gene	MAP2K6	20
sorbin and SH3 domain containing 3	gene	SORBS3	20
protein phosphatase 2 regulatory subunit B'alpha	gene	PPP2R5A	20
periplakin	gene	PPL	20
N-acetyltransferase 2	gene	NAT2	20
phosphatidylinositol-4,5-bisphosphate 3-kinase catalytic subunit beta	gene	PIK3CB	20
serpin family B member 5	gene	SERPINB5	20
SCO2, cytochrome c oxidase assembly protein	gene	SCO2	20
SET domain containing 1A	gene	SETD1A	20
abnormal spindle microtubule assembly	gene	ASPM	20
TELO2 interacting protein 1	gene	TTI1	20
SHANK associated RH domain interactor	gene	SHARPIN	20
myocilin	gene	MYOC	20
transcription elongation factor A like 1	gene	TCEAL1	20
autophagy related 12	gene	ATG12	20
programmed cell death 5	gene	PDCD5	20
rabaptin, RAB GTPase binding effector protein 1	gene	RABEP1	20
inositol 1,4,5-trisphosphate receptor type 1	gene	ITPR1	20
SAS-6 centriolar assembly protein	gene	SASS6	20
lysozyme	gene	LYZ	20
interleukin 2 receptor subunit beta	gene	IL2RB	20
centrosomal protein 120	gene	CEP120	20
pirin	gene	PIR	20
CDK5 regulatory subunit associated protein 3	gene	CDK5RAP3	20
forkhead box A2	gene	FOXA2	19

forkhead box P1	gene	FOXP1	19
hexokinase 1	gene	HK1	19
basic helix-loop-helix family member e41	gene	BHLHE41	19
AKT interacting protein	gene	AKTIP	19
ring finger protein 115	gene	RNF115	19
myotrophin	gene	MTPN	19
TMED7-TICAM2 readthrough	gene	TMED7-TICAM2	19
ERCC excision repair 2, TFIIH core complex helicase subunit	gene	ERCC2	19
histone cluster 3 H2B family member b	gene	HIST3H2BB	19
eukaryotic translation initiation factor 4 gamma 2	gene	EIF4G2	19
MAF bZIP transcription factor K	gene	MAFK	19
zinc finger E-box binding homeobox 1	gene	ZEB1	19
decorin	gene	DCN	19
SWI/SNF related, matrix associated, actin dependent regulator of chromatin, subfamily d, member 3	gene	SMARCD3	19
caspase recruitment domain family member 8	gene	CARD8	19
ribonucleotide reductase regulatory subunit M2	gene	RRM2	19
regulatory factor X5	gene	RFX5	19
leucine zipper protein 6	gene	LUZP6	19
protein phosphatase 1 regulatory subunit 13 like	gene	PPP1R13L	19
fibroblast growth factor receptor substrate 2	gene	FRS2	19
DLG associated protein 4	gene	DLGAP4	19
ATPase plasma membrane Ca2+ transporting 1	gene	ATP2B1	19
ribulose-5-phosphate-3-epimerase	gene	RPE	19
platelet derived growth factor receptor like	gene	PDGFRL	19
thioredoxin interacting protein	gene	TXNIP	19
amyloid beta precursor like protein 2	gene	APLP2	19
aldolase, fructose-bisphosphate B	gene	ALDOB	19
adrenoceptor alpha 2A	gene	ADRA2A	19
TATA-box binding protein associated factor 9b	gene	TAF9B	19
ribonuclease P/MRP subunit p38	gene	RPP38	19
zinc finger and BTB domain containing 33	gene	ZBTB33	19
neurofibromin 1	gene	NF1	19
BCL2 associated athanogene 5	gene	BAG5	19
v-myc avian myelocytomatosis viral oncogene neuroblastoma derived homolog	gene	MYCN	19
death effector domain containing	gene	DEDD	19
exonuclease 1	gene	EXO1	19
eukaryotic translation initiation factor 4E family member 2	gene	EIF4E2	19
protein interacting with PRKCA 1	gene	PICK1	19
zinc finger and BTB domain containing 8A	gene	ZBTB8A	19

MAX dimerization protein 1	gene	MXD1	19
interleukin 1 beta	gene	IL1B	19
mitogen-activated protein kinase-activated protein kinase 5	gene	MAPKAPK5	19
interferon alpha and beta receptor subunit 1	gene	IFNAR1	19
potassium channel tetramerization domain containing 17	gene	KCTD17	19
Wilms tumor 1	gene	WT1	18
toll like receptor adaptor molecule 2	gene	TICAM2	18
esterase D	gene	ESD	18
cyclin D1 binding protein 1	gene	CCNDBP1	18
nuclear receptor subfamily 2 group F member 1	gene	NR2F1	18
dynein cytoplasmic 1 intermediate chain 1	gene	DYNC1I1	18
dachshund family transcription factor 1	gene	DACH1	18
spermine synthase	gene	SMS	18
protein phosphatase 1 regulatory subunit 13B	gene	PPP1R13B	18
TSC22 domain family member 3	gene	TSC22D3	18
cyclin G1	gene	CCNG1	18
retinoid X receptor gamma	gene	RXRG	18
carnitine palmitoyltransferase 1A	gene	CPT1A	18
zinc finger and SCAN domain containing 1	gene	ZSCAN1	18
RNA binding motif (RNP1, RRM) protein 3	gene	RBM3	18
RAP2A, member of RAS oncogene family	gene	RAP2A	18
egl-9 family hypoxia inducible factor 3	gene	EGLN3	18
RAD17 checkpoint clamp loader component	gene	RAD17	18
caudal type homeobox 2	gene	CDX2	18
protein tyrosine phosphatase, receptor type S	gene	PTPRS	18
protein tyrosine phosphatase, non-receptor type 3	gene	PTPN3	18
kinesin family member 1A	gene	KIF1A	18
NCK associated protein 1	gene	NCKAP1	18
interleukin 24	gene	IL24	18
BCL2 like 2	gene	BCL2L2	18
serpin family A member 1	gene	SERPINA1	18
nuclear receptor subfamily 4 group A member 2	gene	NR4A2	18
neurotrophic receptor tyrosine kinase 3	gene	NTRK3	18
purine nucleoside phosphorylase	gene	PNP	18
nitric oxide synthase 1	gene	NOS1	18
NME/NM23 nucleoside diphosphate kinase 4	gene	NME4	18
nebulin	gene	NEB	18
mediator complex subunit 10	gene	MED10	18
melanogenesis associated transcription factor	gene	MITF	18
necdin, MAGE family member	gene	NDN	18
translocase of outer mitochondrial membrane 20	gene	TOMM20	18
PSMC3 interacting protein	gene	PSMC3IP	18

membrane associated guanylate kinase, WW and PDZ domain containing 1	gene	MAGI1	18
kynureninase	gene	KYNU	17
TATA-box binding protein associated factor 5 like	gene	TAF5L	17
neuropilin 1	gene	NRP1	17
axin 2	gene	AXIN2	17
RAB guanine nucleotide exchange factor 1	gene	RABGEF1	17
nucleic acid binding protein 2	gene	NABP2	17
mannosyl-oligosaccharide glucosidase	gene	MOGS	17
forkhead box C1	gene	FOXC1	17
Sin3A associated protein 25	gene	SAP25	17
transforming growth factor beta 2	gene	TGFB2	17
fibrinogen alpha chain	gene	FGA	17
hes related family bHLH transcription factor with YRPW motif 2	gene	HEY2	17
discs large MAGUK scaffold protein 2	gene	DLG2	17
NAD(P)H quinone dehydrogenase 1	gene	NQO1	17
ADAM metallopeptidase domain 17	gene	ADAM17	17
golgi associated, gamma adaptin ear containing, ARF binding protein 3	gene	GGA3	17
histone cluster 2 H2A family member b	gene	HIST2H2AB	17
bile acid-CoA:amino acid N-acyltransferase	gene	BAAT	17
S100 calcium binding protein B	gene	S100B	17
cathepsin V	gene	CTSV	17
exportin 6	gene	XPO6	17
platelet and endothelial cell adhesion molecule 1	gene	PECAM1	17
hexamethylene bisacetamide inducible 1	gene	HEXIM1	17
nudE neurodevelopment protein 1	gene	NDE1	17
SH3 and multiple ankyrin repeat domains 3	gene	SHANK3	17
negative regulator of ubiquitin like proteins 1	gene	NUB1	17
peptidylprolyl isomerase F	gene	PPIF	17
plasminogen activator, tissue type	gene	PLAT	17
nucleobindin 1	gene	NUCB1	17
inhibitor of growth family member 5	gene	ING5	17
pterin-4 alpha-carbinolamine dehydratase 2	gene	PCBD2	17
ADAM metallopeptidase domain 15	gene	ADAM15	17
REV1, DNA directed polymerase	gene	REV1	17
additional sex combs like 1, transcriptional regulator	gene	ASXL1	17
eukaryotic translation initiation factor 3 subunit J	gene	EIF3J	17
late endosomal/lysosomal adaptor, MAPK and MTOR activator 3	gene	LAMTOR3	17
tumor necrosis factor superfamily member 11	gene	TNFSF11	17
calcium/calmodulin dependent protein kinase I	gene	CAMK1	17
F-box protein 11	gene	FBXO11	17

tubulin alpha 4b	gene	TUBA4B	16
elongation factor for RNA polymerase II	gene	ELL	16
nuclear receptor coactivator 4	gene	NCOA4	16
golgin A3	gene	GOLGA3	16
DNA cross-link repair 1C	gene	DCLRE1C	16
protein phosphatase, Mg2+/Mn2+ dependent 1D	gene	PPM1D	16
vascular endothelial growth factor A	gene	VEGFA	16
transcription factor Dp-1	gene	TFDP1	16
transferrin	gene	TF	16
Fc fragment of IgG receptor IIb	gene	FCGR2B	16
SET and MYND domain containing 2	gene	SMYD2	16
BMP/retinoic acid inducible neural specific 1	gene	BRINP1	16
kallikrein related peptidase 5	gene	KLK5	16
secreted protein acidic and cysteine rich	gene	SPARC	16
SATB homeobox 2	gene	SATB2	16
RAD54-like 2 (S. cerevisiae)	gene	RAD54L2	16
cytoplasmic linker associated protein 2	gene	CLASP2	16
C-C motif chemokine ligand 5	gene	CCL5	16
S100 calcium binding protein A6	gene	S100A6	16
S100 calcium binding protein A1	gene	S100A1	16
nucleus accumbens associated 1	gene	NACC1	16
spectrin repeat containing nuclear envelope protein 2	gene	SYNE2	16
ras-related C3 botulinum toxin substrate 2 (rho family, small GTP binding protein Rac2)	gene	RAC2	16
NLR family pyrin domain containing 1	gene	NLRP1	16
cancer susceptibility 3	gene	CASC3	16
collagen type IV alpha 1 chain	gene	COL4A1	16
SH2B adaptor protein 2	gene	SH2B2	16
methionyl aminopeptidase 2	gene	METAP2	16
phosphoribosyl pyrophosphate synthetase associated protein 1	gene	PRPSAP1	16
adenosine kinase	gene	ADK	16
NIMA related kinase 2	gene	NEK2	16
calcium/calmodulin dependent protein kinase kinase 1	gene	CAMKK1	16
SPT7-like STAGA complex gamma subunit	gene	SUPT7L	16
matrix metallopeptidase 2	gene	MMP2	16
EGF containing fibulin like extracellular matrix protein 2	gene	EFEMP2	16
ring finger protein 14	gene	RNF14	16
myogenin	gene	MYOG	16
myosin light chain kinase	gene	MYLK	16
testis expressed 264	gene	TEX264	16
calmodulin-lysine N-methyltransferase	gene	CAMKMT	16
splA/ryanodine receptor domain and SOCS box	gene	SPSB1	15

containing 1			
adaptor related protein complex 1 mu 1 subunit	gene	AP1M1	15
GATA binding protein 4	gene	GATA4	15
mitogen-activated protein kinase associated protein 1	gene	MAPKAP1	15
NK2 homeobox 1	gene	NKX2-1	15
calcium regulated heat stable protein 1	gene	CARHSP1	15
THAP domain containing 11	gene	THAP11	15
synaptotagmin 1	gene	SYT1	15
syntrophin alpha 1	gene	SNTA1	15
dual specificity phosphatase 1	gene	DUSP1	15
exocyst complex component 7	gene	EXOC7	15
family with sequence similarity 175 member B	gene	FAM175B	15
lysine demethylase 4C	gene	KDM4C	15
vasorin	gene	VASN	15
transcriptional adaptor 1	gene	TADA1	15
Ras protein specific guanine nucleotide releasing factor 1	gene	RASGRF1	15
cyclin dependent kinase inhibitor 1C	gene	CDKN1C	15
fibroblast growth factor receptor substrate 3	gene	FRS3	15
phosphorylase kinase catalytic subunit gamma 2	gene	PHKG2	15
intestinal cell kinase	gene	ICK	15
exocyst complex component 3	gene	EXOC3	15
pygopus family PHD finger 2	gene	PYGO2	15
zinc finger CCHC-type containing 10	gene	ZCCHC10	15
PATJ, crumbs cell polarity complex component	gene	PATJ	15
zinc finger C3HC-type containing 1	gene	ZC3HC1	15
nucleobindin 2	gene	NUCB2	15
AKT1 substrate 1	gene	AKT1S1	15
nuclear receptor subfamily 2 group E member 3	gene	NR2E3	15
msh homeobox 1	gene	MSX1	15
COX17, cytochrome c oxidase copper chaperone	gene	COX17	15
ILK associated serine/threonine phosphatase	gene	ILKAP	15
inositol-tetrakisphosphate 1-kinase	gene	ITPK1	15
integrin subunit alpha 2b	gene	ITGA2B	15
interleukin 1 receptor accessory protein	gene	IL1RAP	15
adaptor related protein complex 3 beta 1 subunit	gene	AP3B1	15
nucleolar protein 3	gene	NOL3	14
ribosomal protein S6 kinase A4	gene	RPS6KA4	14
high mobility group nucleosomal binding domain 2	gene	HMGN2	14
immediate early response 3	gene	IER3	14
cysteine rich with EGF like domains 2	gene	CRELD2	14
protein disulfide isomerase family A member 2	gene	PDIA2	14
polypeptide N-acetylgalactosaminyltransferase 12	gene	GALNT12	14
G protein subunit alpha z	gene	GNAZ	14

G protein subunit alpha o1	gene	GNAO1	14
PPFIA binding protein 1	gene	PPFIBP1	14
Era like 12S mitochondrial rRNA chaperone 1	gene	ERAL1	14
MIS12, kinetochore complex component	gene	MIS12	14
thioredoxin reductase 1	gene	TXNRD1	14
MAF bZIP transcription factor F	gene	MAFF	14
ADP ribosylation factor interacting protein 2	gene	ARFIP2	14
HNF1 homeobox B	gene	HNF1B	14
TATA-box binding protein associated factor 5	gene	TAF5	14
SH3 domain containing ring finger 1	gene	SH3RF1	14
tudor domain containing 7	gene	TDRD7	14
synuclein gamma	gene	SNCG	14
FRAT2, WNT signaling pathway regulator	gene	FRAT2	14
CD19 molecule	gene	CD19	14
snail family transcriptional repressor 2	gene	SNAI2	14
dual specificity phosphatase 3	gene	DUSP3	14
zinc finger SWIM-type containing 8	gene	ZSWIM8	14
BAI1 associated protein 2 like 1	gene	BAIAP2L1	14
PDZ binding kinase	gene	PBK	14
cylicin 2	gene	CYLC2	14
ring finger and FYVE like domain containing E3 ubiquitin protein ligase	gene	RFFL	14
trimethylguanosine synthase 1	gene	TGS1	14
RAB4A, member RAS oncogene family	gene	RAB4A	14
ecdysoneless cell cycle regulator	gene	ECD	14
SPT20 homolog, SAGA complex component	gene	SUPT20H	14
seryl-tRNA synthetase 2, mitochondrial	gene	SARS2	14
Kruppel like factor 6	gene	KLF6	14
paired box 5	gene	PAX5	14
collagen type II alpha 1 chain	gene	COL2A1	14
BCL2 interacting protein 3 like	gene	BNIP3L	14
proteoglycan 2, pro eosinophil major basic protein	gene	PRG2	14
protein phosphatase 3 regulatory subunit B, beta	gene	PPP3R2	14
amylo-alpha-1, 6-glucosidase, 4-alpha-glucanotransferase	gene	AGL	14
pyrophosphatase (inorganic) 1	gene	PPA1	14
AKT serine/threonine kinase 3	gene	AKT3	14
NUAK family kinase 1	gene	NUAK1	14
ribonucleotide reductase regulatory TP53 inducible subunit M2B	gene	RRM2B	14
potassium voltage-gated channel interacting protein 3	gene	KCNIP3	14
transportin 2	gene	TNPO2	14
guanine deaminase	gene	GDA	14
laminin subunit beta 1	gene	LAMB1	14

laminin subunit alpha 4	gene	LAMA4	14
G protein nucleolar 3 like	gene	GNL3L	14
interleukin 16	gene	IL16	14
TATA-box binding protein associated factor, RNA polymerase I subunit C	gene	TAF1C	14
HECT and RLD domain containing E3 ubiquitin protein ligase family member 1	gene	HERC1	13
multiple PDZ domain crumbs cell polarity complex component	gene	MPDZ	13
T-cell leukemia/lymphoma 1A	gene	TCL1A	13
general transcription factor IIH subunit 4	gene	GTF2H4	13
F-box protein 4	gene	FBXO4	13
kelch like family member 3	gene	KLHL3	13
CUE domain containing 2	gene	CUEDC2	13
acyl-CoA synthetase long-chain family member 4	gene	ACSL4	13
zinc finger MIZ-type containing 1	gene	ZMIZ1	13
fibrinogen beta chain	gene	FGB	13
sirtuin 3	gene	SIRT3	13
UFM1 specific ligase 1	gene	UFL1	13
E4F transcription factor 1	gene	E4F1	13
solute carrier family 9 member A1	gene	SLC9A1	13
CD14 molecule	gene	CD14	13
syntaxin 5	gene	STX5	13
suppression of tumorigenicity 14	gene	ST14	13
formin binding protein 1	gene	FNBP1	13
sodium voltage-gated channel beta subunit 2	gene	SCN2B	13
S100 calcium binding protein A2	gene	S100A2	13
related RAS viral (r-ras) oncogene homolog	gene	RRAS	13
PH domain and leucine rich repeat protein phosphatase 1	gene	PHLPP1	13
proline rich coiled-coil 2C	gene	PRRC2C	13
disco interacting protein 2 homolog A	gene	DIP2A	13
phosphorylase kinase regulatory subunit beta	gene	PHKB	13
ADP ribosylation factor like GTPase 3	gene	ARL3	13
phosphodiesterase 3A	gene	PDE3A	13
tetratricopeptide repeat, ankyrin repeat and coiled-coil containing 1	gene	TANC1	13
tubulin alpha 8	gene	TUBA8	13
nuclear receptor subfamily 0 group B member 1	gene	NR0B1	13
zinc finger and BTB domain containing 9	gene	ZBTB9	13
angiotensin II receptor type 1	gene	AGTR1	13
protein phosphatase 3 catalytic subunit gamma	gene	PPP3CC	13
adrenoceptor beta 1	gene	ADRB1	13
LSM8 homolog, U6 small nuclear RNA associated	gene	LSM8	13

cytochrome p450 oxidoreductase	gene	POR	13
alcohol dehydrogenase 5 (class III), chi polypeptide	gene	ADH5	13
homeodomain interacting protein kinase 3	gene	HIPK3	13
acetylcholinesterase (Cartwright blood group)	gene	ACHE	13
SIVA1 apoptosis inducing factor	gene	SIVA1	13
PLAG1 like zinc finger 1	gene	PLAGL1	13
G protein subunit gamma 2	gene	GNG2	13
mitochondrial ribosomal protein L39	gene	MRPL39	13
msh homeobox 2	gene	MSX2	13
UTP15, small subunit processome component	gene	UTP15	13
oxidative stress induced growth inhibitor 1	gene	OSGIN1	13
T-cell leukemia/lymphoma 1B	gene	TCL1B	13
interleukin 1 receptor associated kinase 4	gene	IRAK4	13
coiled-coil domain containing 106	gene	CCDC106	13
internexin neuronal intermediate filament protein alpha	gene	INA	13
torsin 1A interacting protein 2	gene	TOR1AIP2	13
actin like 6B	gene	ACTL6B	13
N-myristoyltransferase 2	gene	NMT2	13
cell division cycle 14B	gene	CDC14B	13
period circadian clock 2	gene	PER2	12
PAXIP1 associated glutamate rich protein 1	gene	PAGR1	12
DiGeorge syndrome critical region gene 14	gene	DGCR14	12
5-hydroxytryptamine receptor 6	gene	HTR6	12
heat shock transcription factor 4	gene	HSF4	12
zinc finger protein 16	gene	ZNF16	12
mitochondrial ribosomal protein S25	gene	MRPS25	12
phosphatidylinositol-4-phosphate 5-kinase type 1 alpha	gene	PIP5K1A	12
tyrosine hydroxylase	gene	TH	12
histone H4 transcription factor	gene	HINFP	12
calcium binding and coiled-coil domain 1	gene	CALCOCO1	12
capping actin protein, gelsolin like	gene	CAPG	12
exosome component 7	gene	EXOSC7	12
phospholipase C beta 1	gene	PLCB1	12
TP53 regulating kinase	gene	TP53RK	12
protein tyrosine phosphatase, receptor type Z1	gene	PTPRZ1	12
ATPase H+ transporting V1 subunit C1	gene	ATP6V1C1	12
ATP synthase, H+ transporting, mitochondrial Fo complex subunit F6	gene	ATP5J	12
regulation of nuclear pre-mRNA domain containing 1A	gene	RPRD1A	12
platelet derived growth factor subunit B	gene	PDGFB	12
platelet derived growth factor subunit A	gene	PDGFA	12
polo like kinase 3	gene	PLK3	12
bromodomain containing 8	gene	BRD8	12

kinase suppressor of ras 2	gene	KSR2	12
rhophilin Rho GTPase binding protein 2	gene	RHPN2	12
THAP domain containing 12	gene	THAP12	12
calmodulin like 5	gene	CALML5	12
protein phosphatase 3 regulatory subunit B, alpha	gene	PPP3R1	12
jumonji domain containing 1C	gene	JMJD1C	12
POU class 1 homeobox 1	gene	POU1F1	12
plexin B1	gene	PLXNB1	12
SLU7 homolog, splicing factor	gene	SLU7	12
ring finger protein 216	gene	RNF216	12
myosin light chain 9	gene	MYL9	12
calcium voltage-gated channel auxiliary subunit gamma 2	gene	CACNG2	12
neuronal differentiation 1	gene	NEUROD1	12
PML-RARA regulated adaptor molecule 1	gene	PRAM1	12
misshapen like kinase 1	gene	MINK1	12
A-kinase anchoring protein 12	gene	AKAP12	12
angiopoietin like 4	gene	ANGPTL4	12
tankyrase 2	gene	TNKS2	12
inositol 1,4,5-trisphosphate receptor type 3	gene	ITPR3	12
MAGE family member C2	gene	MAGEC2	12
ATPase H+ transporting V1 subunit D	gene	ATP6V1D	12
lysyl oxidase	gene	LOX	12
transcription elongation factor A like 4	gene	TCEAL4	12
interleukin 2 receptor subunit gamma	gene	IL2RG	12
interleukin 2	gene	IL2	12
interleukin 1 receptor type 1	gene	IL1R1	12
DIRAS family GTPase 3	gene	DIRAS3	12
transmembrane p24 trafficking protein 9	gene	TMED9	12
ring finger protein 34	gene	RNF34	11
thiosulfate sulfurtransferase like domain containing 2	gene	TSTD2	11
neuregulin 1	gene	NRG1	11
lin-7 homolog A, crumbs cell polarity complex component	gene	LIN7A	11
hemoglobin subunit beta	gene	HBB	11
growth factor independent 1 transcriptional repressor	gene	GFI1	11
modulator of apoptosis 1	gene	MOAP1	11
BRF1, RNA polymerase III transcription initiation factor subunit	gene	BRF1	11
PPARG coactivator 1 beta	gene	PPARGC1B	11
fms related tyrosine kinase 4	gene	FLT4	11
mindbomb E3 ubiquitin protein ligase 2	gene	MIB2	11
zinc finger protein 148	gene	ZNF148	11
sulfide quinone reductase-like (yeast)	gene	SQRDL	11

dihydrofolate reductase pseudogene 1	gene	DHFRP1	11
exostosin glycosyltransferase 2	gene	EXT2	11
phosphatidylinositol transfer protein beta	gene	PITPNB	11
mitochondrial ribosomal protein S14	gene	MRPS14	11
POZ/BTB and AT hook containing zinc finger 1	gene	PATZ1	11
dopamine receptor D4	gene	DRD4	11
ferrochelatase	gene	FECH	11
dihydrofolate reductase	gene	DHFR	11
DDB1 and CUL4 associated factor 13	gene	DCAF13	11
zinc finger and BTB domain containing 2	gene	ZBTB2	11
cilia and flagella associated protein 97	gene	CFAP97	11
CD40 ligand	gene	CD40LG	11
GRIP1 associated protein 1	gene	GRIPAP1	11
F-box protein 21	gene	FBXO21	11
spalt like transcription factor 1	gene	SALL1	11
selenoprotein S	gene	SELENOS	11
ras-related C3 botulinum toxin substrate 3 (rho family, small GTP binding protein Rac3)	gene	RAC3	11
epithelial stromal interaction 1	gene	EPSTI1	11
NADPH oxidase activator 1	gene	NOXA1	11
adenosine deaminase	gene	ADA	11
laminin subunit alpha 1	gene	LAMA1	11
ZFP36 ring finger protein like 1	gene	ZFP36L1	11
bone morphogenetic protein 2	gene	BMP2	11
prostaglandin D2 synthase	gene	PTGDS	11
mannan binding lectin serine peptidase 1	gene	MASP1	11
prospero homeobox 1	gene	PROX1	11
primase (DNA) subunit 1	gene	PRIM1	11
M-phase phosphoprotein 6	gene	MPHOSPH6	11
RNA binding motif protein 12	gene	RBM12	11
adenosine A1 receptor	gene	ADORA1	11
BCL2 like 10	gene	BCL2L10	11
ubiquitin D	gene	UBD	11
potassium channel tetramerization domain containing 13	gene	KCTD13	11
nuclear respiratory factor 1	gene	NRF1	11
notch 4	gene	NOTCH4	11
Cbp/p300 interacting transactivator with Glu/Asp rich carboxy-terminal domain 2	gene	CITED2	11
NADH:ubiquinone oxidoreductase subunit A8	gene	NDUFA8	11
SH3 and multiple ankyrin repeat domains 1	gene	SHANK1	11
F-box protein 45	gene	FBXO45	11
membrane associated guanylate kinase, WW and PDZ domain containing 3	gene	MAGI3	11

matrix metallopeptidase 9	gene	MMP9	11
methyltransferase like 1	gene	METTL1	11
bifunctional apoptosis regulator	gene	BFAR	11
pleckstrin homology domain containing O1	gene	PLEKHO1	11
laminin subunit gamma 1	gene	LAMC1	11
CD274 molecule	gene	CD274	11
N-terminal Xaa-Pro-Lys N-methyltransferase 1	gene	NTMT1	11
adaptor related protein complex 4 mu 1 subunit	gene	AP4M1	11
integrin subunit alpha 2	gene	ITGA2	11
tumor necrosis factor superfamily member 10	gene	TNFSF10	11
heart and neural crest derivatives expressed 1	gene	HAND1	11
lipoprotein lipase	gene	LPL	11
jade family PHD finger 1	gene	JADE1	11
phosphoprotein enriched in astrocytes 15	gene	PEA15	11
nuclear receptor binding factor 2	gene	NRBF2	11
isocitrate dehydrogenase 3 (NAD(+)) beta	gene	IDH3B	11
HECT domain E3 ubiquitin protein ligase 3	gene	HECTD3	11
mitochondrial E3 ubiquitin protein ligase 1	gene	MUL1	10
homeodomain interacting protein kinase 4	gene	HIPK4	10
golgi reassembly stacking protein 1	gene	GORASP1	10
forkhead box N1	gene	FOXN1	10
von Willebrand factor	gene	VWF	10
glutamate metabotropic receptor 1	gene	GRM1	10
LDL receptor related protein 8	gene	LRP8	10
fibrinogen gamma chain	gene	FGG	10
gamma-aminobutyric acid type A receptor alpha1 subunit	gene	GABRA1	10
ETS2 repressor factor	gene	ERF	10
EPH receptor A3	gene	EPHA3	10
endoglin	gene	ENG	10
ELK4, ETS transcription factor	gene	ELK4	10
transforming growth factor beta receptor 3	gene	TGFBR3	10
makorin ring finger protein 1	gene	MKRN1	10
diphthamide biosynthesis 1	gene	DPH1	10
CDGSH iron sulfur domain 2	gene	CISD2	10
SRY-box 4	gene	SOX4	10
Ras homolog enriched in brain like 1	gene	RHEBL1	10
solute carrier family 4 member 1 (Diego blood group)	gene	SLC4A1	10
neural proliferation, differentiation and control 1	gene	NPDC1	10
sulfotransferase family 1E member 1	gene	SULT1E1	10
HECT, C2 and WW domain containing E3 ubiquitin protein ligase 1	gene	HECW1	10
protocadherin alpha 4	gene	PCDHA4	10
sodium channel epithelial 1 gamma subunit	gene	SCNN1G	10

SNF8, ESCRT-II complex subunit	gene	SNF8	10
RAB, member RAS oncogene family-like 6	gene	RABL6	10
proline rich nuclear receptor coactivator 2	gene	PNRC2	10
forkhead box J3	gene	FOXJ3	10
nicotinate phosphoribosyltransferase	gene	NAPRT	10
ATPase sarcoplasmic/endoplasmic reticulum Ca2+ transporting 3	gene	ATP2A3	10
BH3-like motif containing, cell death inducer	gene	BLID	10
OTU deubiquitinase 5	gene	OTUD5	10
activin A receptor like type 1	gene	ACVRL1	10
glucocorticoid modulatory element binding protein 1	gene	GMEB1	10
serpin family E member 1	gene	SERPINE1	10
neuron navigator 1	gene	NAV1	10
BCL2 interacting protein 3	gene	BNIP3	10
serine and arginine rich splicing factor 8	gene	SRSF8	10
biglycan	gene	BGN	10
BRCA1 associated ATM activator 1	gene	BRAT1	10
protein phosphatase 1 regulatory subunit 16A	gene	PPP1R16A	10
RNA polymerase mitochondrial	gene	POLRMT	10
SH2 domain containing 3C	gene	SH2D3C	10
anterior gradient 2, protein disulphide isomerase family member	gene	AGR2	10
ubiquitin specific peptidase 42	gene	USP42	10
zinc finger MIZ-type containing 2	gene	ZMIZ2	10
MAD2L1 binding protein	gene	MAD2L1BP	10
NLR family apoptosis inhibitory protein	gene	NAIP	10
growth arrest and DNA damage inducible beta	gene	GADD45B	10
zinc finger HIT-type containing 3	gene	ZNHIT3	10
integrator complex subunit 5	gene	INTS5	10
latent transforming growth factor beta binding protein 1	gene	LTBP1	10
LDL receptor related protein associated protein 1	gene	LRPAP1	10
TAO kinase 3	gene	TAOK3	10
interleukin 2 receptor subunit alpha	gene	IL2RA	10
zinc finger and BTB domain containing 3	gene	ZBTB3	10
BCL2 interacting protein like	gene	BNIPL	9
acidic residue methyltransferase 1	gene	ARMT1	9
hematopoietic cell-specific Lyn substrate 1	gene	HCLS1	9
ring finger protein 128, E3 ubiquitin protein ligase	gene	RNF128	9
synaptic Ras GTPase activating protein 1	gene	SYNGAP1	9
Kruppel like factor 11	gene	KLF11	9
beta-1,4-galactosyltransferase 1	gene	B4GALT1	9
tripartite motif containing 69	gene	TRIM69	9
glutathione S-transferase mu 4	gene	GSTM4	9
zinc finger and BTB domain containing 20	gene	ZBTB20	9

LSM14A, mRNA processing body assembly factor	gene	LSM14A	9
farnesyltransferase, CAAX box, alpha	gene	FNTA	9
single Ig and TIR domain containing	gene	SIGIRR	9
membrane spanning 4-domains A2	gene	MS4A2	9
FAT atypical cadherin 1	gene	FAT1	9
gamma-aminobutyric acid type A receptor rho1 subunit	gene	GABRR1	9
tetratricopeptide repeat domain 3	gene	TTC3	9
S100 calcium binding protein A14	gene	S100A14	9
trefoil factor 1	gene	TFF1	9
peptidyl arginine deiminase 4	gene	PADI4	9
AT-rich interaction domain 3A	gene	ARID3A	9
ARP3 actin related protein 3 homolog B	gene	ACTR3B	9
telomerase associated protein 1	gene	TEP1	9
CD36 molecule	gene	CD36	9
eukaryotic translation initiation factor 2D	gene	EIF2D	9
SMG5, nonsense mediated mRNA decay factor	gene	SMG5	9
dual specificity phosphatase 9	gene	DUSP9	9
dual specificity phosphatase 4	gene	DUSP4	9
MSANTD3-TMEFF1 readthrough	gene	MSANTD3-TMEFF1	9
G-patch domain containing 8	gene	GPATCH8	9
somatostatin receptor 3	gene	SSTR3	9
dehydrogenase/reductase 4 like 2	gene	DHRS4L2	9
CD93 molecule	gene	CD93	9
nicalin	gene	NCLN	9
mitochondrial inner membrane organizing system 1	gene	MINOS1	9
complement C3d receptor 2	gene	CR2	9
kallikrein related peptidase 9	gene	KLK9	9
citron rho-interacting serine/threonine kinase	gene	CIT	9
caspase 12 (gene/pseudogene)	gene	CASP12	9
protein tyrosine phosphatase, receptor type R	gene	PTPRR	9
TBC1 domain family member 2	gene	TBC1D2	9
cadherin 13	gene	CDH13	9
phosphorylase kinase regulatory subunit alpha 2	gene	PHKA2	9
three prime repair exonuclease 1	gene	TREX1	9
Rho GDP dissociation inhibitor beta	gene	ARHGDIB	9
PDZ domain containing 1	gene	PDZK1	9
Bcl2 modifying factor	gene	BMF	9
phosphodiesterase 3B	gene	PDE3B	9
OTU deubiquitinase with linear linkage specificity	gene	OTULIN	9
catechol-O-methyltransferase	gene	COMT	9
ring finger protein 43	gene	RNF43	9
aprataxin	gene	APTX	9
glutaminyl-peptide cyclotransferase like	gene	QPCTL	9

proline rich nuclear receptor coactivator 1	gene	PNRC1	9
dehydrogenase/reductase 4	gene	DHRS4	9
apolipoprotein H	gene	APOH	9
kallikrein related peptidase 7	gene	KLK7	9
ankyrin 2	gene	ANK2	9
tripartite motif containing 13	gene	TRIM13	9
alpha 2-HS glycoprotein	gene	AHSG	9
lysophosphatidic acid receptor 6	gene	LPAR6	9
chromosome 9 open reading frame 3	gene	C9orf3	9
active BCR-related	gene	ABR	9
phorbol-12-myristate-13-acetate-induced protein 1	gene	PMAIP1	9
protein tyrosine phosphatase, non-receptor type 5	gene	PTPN5	9
zinc finger protein 496	gene	ZNF496	9
STE20 like kinase	gene	SLK	9
ADP ribosylation factor guanine nucleotide exchange factor 2	gene	ARFGEF2	9
nuclear factor, erythroid 2 like 3	gene	NFE2L3	9
piwi like RNA-mediated gene silencing 1	gene	PIWIL1	9
TNF receptor superfamily member 25	gene	TNFRSF25	9
leucyl and cystinyl aminopeptidase	gene	LNPEP	9
anterior gradient 3, protein disulphide isomerase family member	gene	AGR3	9
interleukin 13 receptor subunit alpha 2	gene	IL13RA2	9
transmembrane protein with EGF like and two follistatin like domains 1	gene	TMEFF1	9
F-box protein 31	gene	FBXO31	9
XIAP associated factor 1	gene	XAF1	9
myotubularin related protein 3	gene	MTMR3	8
period circadian clock 3	gene	PER3	8
zinc finger protein 420	gene	ZNF420	8
coiled-coil domain containing 155	gene	CCDC155	8
5-hydroxytryptamine receptor 2A	gene	HTR2A	8
chromosome 22 open reading frame 29	gene	C22orf29	8
zinc finger and SCAN domain containing 20	gene	ZSCAN20	8
G protein subunit alpha 11	gene	GNA11	8
glia maturation factor beta	gene	GMFB	8
glutaredoxin	gene	GLRX	8
glutaminase	gene	GLS	8
PDZ and LIM domain 2	gene	PDLIM2	8
trafficking kinesin protein 2	gene	TRAK2	8
lin-54 DREAM MuvB core complex component	gene	LIN54	8
gamma-aminobutyric acid type A receptor gamma2 subunit	gene	GABRG2	8
F2R like trypsin receptor 1	gene	F2RL1	8

trafficking protein particle complex 11	gene	TRAPPC11	8
ATPase H+ transporting V0 subunit a2	gene	ATP6V0A2	8
synuclein beta	gene	SNCB	8
ubiquitin specific peptidase 24	gene	USP24	8
dual specificity phosphatase 7	gene	DUSP7	8
cullin 9	gene	CUL9	8
tubulin tyrosine ligase like 5	gene	TTLL5	8
prostaglandin reductase 1	gene	PTGR1	8
tyrosyl-DNA phosphodiesterase 1	gene	TDP1	8
C-reactive protein	gene	CRP	8
follistatin like 1	gene	FSTL1	8
glutamate ionotropic receptor NMDA type subunit 3A	gene	GRIN3A	8
protein tyrosine phosphatase, receptor type H	gene	PTPRH	8
protein tyrosine phosphatase, receptor type E	gene	PTPRE	8
acid phosphatase, prostate	gene	ACPP	8
ring finger protein 125	gene	RNF125	8
protocadherin 7	gene	PCDH7	8
orthodenticle homeobox 2	gene	OTX2	8
BCL2 interacting protein 2	gene	BNIP2	8
BCL2 interacting protein 1	gene	BNIP1	8
BCL2 interacting killer	gene	BIK	8
proteinase 3	gene	PRTN3	8
regulator of G-protein signaling 19	gene	RGS19	8
aldehyde dehydrogenase 3 family member B1	gene	ALDH3B1	8
angiotensinogen	gene	AGT	8
poly(ADP-ribose) polymerase family member 4	gene	PARP4	8
splA/ryanodine receptor domain and SOCS box containing 2	gene	SPSB2	8
vesicle amine transport 1	gene	VAT1	8
zinc finger HIT-type containing 1	gene	ZNHIT1	8
homeodomain interacting protein kinase 1	gene	HIPK1	8
mannose receptor C type 2	gene	MRC2	8
centromere protein X	gene	CENPX	8
myeloid cell nuclear differentiation antigen	gene	MNDA	8
suppressor of cytokine signaling 5	gene	SOCS5	8
leukemia inhibitory factor receptor alpha	gene	LIFR	8
secretory carrier membrane protein 1	gene	SCAMP1	8
5-methyltetrahydrofolate-homocysteine methyltransferase	gene	MTR	8
NADH dehydrogenase, subunit 4 (complex I)	gene	ND4	8
protease, serine 50	gene	PRSS50	8
lymphotoxin alpha	gene	LTA	8
interleukin 6 receptor	gene	IL6R	8
protein kinase, membrane associated	gene	PKMYT1	8

tyrosine/threonine 1			
lactate dehydrogenase A like 6A	gene	LDHAL6A	8
mitotic spindle organizing protein 2B	gene	MZT2B	7
MAF bZIP transcription factor A	gene	MAFA	7
forkhead box O6	gene	FOXO6	7
forkhead box N2	gene	FOXN2	7
ADAM metallopeptidase domain 12	gene	ADAM12	7
WNK lysine deficient protein kinase 4	gene	WNK4	7
abhydrolase domain containing 16A	gene	ABHD16A	7
mitochondrial ribosomal protein L41	gene	MRPL41	7
required for meiotic nuclear division 5 homolog A	gene	RMND5A	7
transcription termination factor 1	gene	TTF1	7
cadherin EGF LAG seven-pass G-type receptor 2	gene	CELSR2	7
purinergic receptor P2Y8	gene	P2RY8	7
CAP-Gly domain containing linker protein 3	gene	CLIP3	7
MIF4G domain containing	gene	MIF4GD	7
BRICK1, SCAR/WAVE actin nucleating complex subunit	gene	BRK1	7
anaphase promoting complex subunit 1 pseudogene	gene	LOC285074	7
connective tissue growth factor	gene	CTGF	7
colony stimulating factor 1	gene	CSF1	7
twist family bHLH transcription factor 2	gene	TWIST2	7
lysine demethylase 4D	gene	KDM4D	7
carboxypeptidase M	gene	CPM	7
REV3 like, DNA directed polymerase zeta catalytic subunit	gene	REV3L	7
sphingomyelin phosphodiesterase 3	gene	SMPD3	7
GTPase, IMAP family member 5	gene	GIMAP5	7
aspartate beta-hydroxylase	gene	ASPH	7
solute carrier family 35 member F6	gene	SLC35F6	7
6-phosphofructo-2-kinase/fructose-2,6-biphosphatase 1	gene	PFKFB1	7
ubiquitin conjugating enzyme E2 Q1	gene	UBE2Q1	7
cholinergic receptor nicotinic alpha 9 subunit	gene	CHRNA9	7
MAGE family member A2B	gene	MAGEA2B	7
semaphorin 4C	gene	SEMA4C	7
bisphosphoglycerate mutase	gene	BPGM	7
FXYD domain containing ion transport regulator 6	gene	FXYD6	7
prostaglandin-endoperoxide synthase 1	gene	PTGS1	7
helicase with zinc finger 2	gene	HELZ2	7
adenosine A2a receptor	gene	ADORA2A	7
plexin A1	gene	PLXNA1	7
TP53 regulated inhibitor of apoptosis 1	gene	TRIAP1	7
homeobox B13	gene	HOXB13	7

neurofilament heavy	gene	NEFH	7
Rho guanine nucleotide exchange factor 17	gene	ARHGEF17	7
ATP binding cassette subfamily B member 6 (Langereis blood group)	gene	ABCB6	7
growth regulation by estrogen in breast cancer 1	gene	GREB1	7
dual specificity phosphatase 16	gene	DUSP16	7
ADAM metallopeptidase domain 9	gene	ADAM9	7
integral membrane protein 2B	gene	ITM2B	7
leucine carboxyl methyltransferase 1	gene	LCMT1	7
inositol hexakisphosphate kinase 2	gene	IP6K2	7
MAGE family member A2	gene	MAGEA2	7
latent transforming growth factor beta binding protein 3	gene	LTBP3	7
interleukin 6	gene	IL6	7
homeobox C6	gene	HOXC6	7
homeobox A5	gene	HOXA5	7
integrin subunit beta 6	gene	ITGB6	7
copine 7	gene	CPNE7	6
BCL2 binding component 3	gene	BBC3	6
guanylate cyclase 1 soluble subunit alpha	gene	GUCY1A3	6
BCL2 like 14	gene	BCL2L14	6
ring finger protein 38	gene	RNF38	6
solute carrier family 7 member 5	gene	SLC7A5	6
latent transforming growth factor beta binding protein 4	gene	LTBP4	6
secretogranin II	gene	SCG2	6
nucleic acid binding protein 1	gene	NABP1	6
putative inactive beta-glucuronidase-like protein SMA3	gene	LOC100653061	6
transforming growth factor beta induced	gene	TGFBI	6
transcription factor AP-2 beta	gene	TFAP2B	6
transmembrane BAX inhibitor motif containing 6	gene	TMBIM6	6
dopamine receptor D1	gene	DRD1	6
apoptosis and caspase activation inhibitor	gene	AVEN	6
24-dehydrocholesterol reductase	gene	DHCR24	6
sulfotransferase family 4A member 1	gene	SULT4A1	6
ETHE1, persulfide dioxygenase	gene	ETHE1	6
PH domain and leucine rich repeat protein phosphatase 2	gene	PHLPP2	6
C-C motif chemokine ligand 18	gene	CCL18	6
serine/threonine kinase 11 interacting protein	gene	STK11IP	6
glucuronidase beta pseudogene	gene	SMA4	6
protein tyrosine phosphatase, non-receptor type 7	gene	PTPN7	6
nei like DNA glycosylase 3	gene	NEIL3	6
argininosuccinate lyase	gene	ASL	6
stabilin 2	gene	STAB2	6
tRNA methyltransferase 10C, mitochondrial RNase P	gene	TRMT10C	6

subunit			
oncostatin M	gene	OSM	6
Cdk5 and Abl enzyme substrate 1	gene	CABLES1	6
annexin A3	gene	ANXA3	6
aldehyde dehydrogenase 1 family member A1	gene	ALDH1A1	6
chymotrypsin like elastase family member 2B	gene	CELA2B	6
zyg-11 related cell cycle regulator	gene	ZER1	6
CDP-diacylglycerol--inositol 3-phosphatidyltransferase	gene	CDIPT	6
F-box protein 42	gene	FBXO42	6
Ras and Rab interactor 2	gene	RIN2	6
ArfGAP with coiled-coil, ankyrin repeat and PH domains 1	gene	ACAP1	6
mitochondrial trans-2-enoyl-CoA reductase	gene	MECR	6
phospholipase A2 group IVB	gene	PLA2G4B	6
zinc finger MYM-type containing 5	gene	ZMYM5	6
tumor necrosis factor superfamily member 9	gene	TNFSF9	6
harakiri, BCL2 interacting protein	gene	HRK	6
glucuronidase, beta pseudogene 3	gene	GUSBP3	6
roundabout guidance receptor 4	gene	ROBO4	6
JMJD7-PLA2G4B readthrough	gene	JMJD7-PLA2G4B	6
cell division cycle 14A	gene	CDC14A	6
insulin like growth factor binding protein 7	gene	IGFBP7	6
zinc finger protein 668	gene	ZNF668	6
integrin subunit beta 8	gene	ITGB8	6
one cut homeobox 1	gene	ONECUT1	5
NPR3 like, GATOR1 complex subunit	gene	NPRL3	5
adaptor related protein complex 3 beta 2 subunit	gene	AP3B2	5
poly(ADP-ribose) glycohydrolase	gene	PARG	5
Rho GTPase activating protein 9	gene	ARHGAP9	5
glycerol kinase	gene	GK	5
lin-7 homolog B, crumbs cell polarity complex component	gene	LIN7B	5
ankyrin repeat domain 2	gene	ANKRD2	5
glutamic--pyruvic transaminase	gene	GPT	5
family with sequence similarity 110 member C	gene	FAM110C	5
junction mediating and regulatory protein, p53 cofactor	gene	JMY	5
pannexin 1	gene	PANX1	5
methenyltetrahydrofolate synthetase domain containing	gene	MTHFSD	5
gamma-aminobutyric acid type A receptor beta2 subunit	gene	GABRB2	5
fatty acid binding protein 1	gene	FABP1	5
zinc finger FYVE-type containing 28	gene	ZFYVE28	5

toll like receptor 5	gene	TLR5	5
toll like receptor 1	gene	TLR1	5
serine racemase	gene	SRR	5
MAGE family member B18	gene	MAGEB18	5
phosphatidylinositol-3,4,5-trisphosphate dependent Rac exchange factor 1	gene	PREX1	5
sphingosine-1-phosphate receptor 1	gene	S1PR1	5
dual specificity phosphatase 6	gene	DUSP6	5
heat shock protein family A (Hsp70) member 13	gene	HSPA13	5
SERTA domain containing 4	gene	SERTAD4	5
pituitary tumor-transforming 1 interacting protein	gene	PTTG1IP	5
RAB18, member RAS oncogene family	gene	RAB18	5
cytochrome b5 type A	gene	CYB5A	5
ryanodine receptor 3	gene	RYR3	5
carboxypeptidase D	gene	CPD	5
family with sequence similarity 129 member A	gene	FAM129A	5
baculoviral IAP repeat containing 8	gene	BIRC8	5
tumor protein p53 inducible nuclear protein 1	gene	TP53INP1	5
RAS like family 10 member B	gene	RASL10B	5
pyruvate dehydrogenase kinase 2	gene	PDK2	5
splA/ryanodine receptor domain and SOCS box containing 4	gene	SPSB4	5
secretoglobin family 3A member 1	gene	SCGB3A1	5
biphenyl hydrolase like	gene	BPHL	5
parathyroid hormone like hormone	gene	PTHLH	5
bone morphogenetic protein 3	gene	BMP3	5
biliverdin reductase A	gene	BLVRA	5
prostaglandin D2 receptor	gene	PTGDR	5
protein phosphatase 2 regulatory subunit B'beta	gene	PPP2R5B	5
adrenoceptor alpha 2C	gene	ADRA2C	5
DNA cross-link repair 1A	gene	DCLRE1A	5
mex-3 RNA binding family member D	gene	MEX3D	5
ubiquitin specific peptidase 34	gene	USP34	5
major intrinsic protein of lens fiber	gene	MIP	5
KIT ligand	gene	KITLG	5
shisa family member 5	gene	SHISA5	5
Cdk5 and Abl enzyme substrate 2	gene	CABLES2	5
THAP domain containing 8	gene	THAP8	5
RAB3D, member RAS oncogene family	gene	RAB3D	5
tripartite motif containing 65	gene	TRIM65	5
cell division cycle 20B	gene	CDC20B	5
microtubule associated protein 9	gene	MAP9	5
interleukin 4	gene	IL4	5
interleukin 3	gene	IL3	5

immunoglobulin heavy constant gamma 3 (G3m marker)	gene	IGHG3	5
interferon induced protein 35	gene	IFI35	5
inter-alpha-trypsin inhibitor heavy chain 2	gene	ITIH2	5
PDLIM1 interacting kinase 1 like	gene	PDIK1L	4
N-acetylglucosamine-1-phosphate transferase alpha and beta subunits	gene	GNPTAB	4
histamine receptor H1	gene	HRH1	4
ES cell expressed Ras	gene	ERAS	4
Zic family member 3	gene	ZIC3	4
goosecoid homeobox	gene	GSC	4
tRNA methyltransferase 11 homolog	gene	TRMT11	4
glutathione peroxidase 2	gene	GPX2	4
family with sequence similarity 173 member A	gene	FAM173A	4
CREB/ATF bZIP transcription factor	gene	CREBZF	4
transition protein 1	gene	TNP1	4
dermatopontin	gene	DPT	4
defensin beta 4B	gene	DEFB4B	4
ficolin 1	gene	FCN1	4
aldo-keto reductase family 1 member B10	gene	AKR1B10	4
defensin beta 4A	gene	DEFB4A	4
CKLF like MARVEL transmembrane domain containing 3	gene	CMTM3	4
cytochrome P450 family 1 subfamily A member 2	gene	CYP1A2	4
CD53 molecule	gene	CD53	4
CD37 molecule	gene	CD37	4
leukotriene B4 receptor 2	gene	LTB4R2	4
BARX homeobox 1	gene	BARX1	4
C-C motif chemokine ligand 3	gene	CCL3	4
multimerin 1	gene	MMRN1	4
cystatin E/M	gene	CST6	4
WAP, follistatin/kazal, immunoglobulin, kunitz and netrin domain containing 1	gene	WFIKKN1	4
FIC domain containing	gene	FICD	4
zinc finger protein 302	gene	ZNF302	4
PZP, alpha-2-macroglobulin like	gene	PZP	4
ribonuclease L	gene	RNASEL	4
chymase 1	gene	CMA1	4
membrane associated ring-CH-type finger 6	gene	6-Mar	4
cyclin I	gene	CCNI	4
prostaglandin I2 synthase	gene	PTGIS	4
3-hydroxybutyrate dehydrogenase 1	gene	BDH1	4
YY2 transcription factor	gene	YY2	4
angiogenin	gene	ANG	4

JAZF zinc finger 1	gene	JAZF1	4
proteolipid protein 1	gene	PLP1	4
CXXC finger protein 5	gene	CXXC5	4
natriuretic peptide A	gene	NPPA	4
cyclin D binding myb like transcription factor 1	gene	DMTF1	4
mature T-cell proliferation 1	gene	MTCP1	4
engulfment and cell motility 1	gene	ELMO1	4
aldehyde dehydrogenase 6 family member A1	gene	ALDH6A1	4
heat shock protein family A (Hsp70) member 12A	gene	HSPA12A	4
microfibrillar associated protein 4	gene	MFAP4	4
myosin light chain 3	gene	MYL3	4
neuronal regeneration related protein	gene	NREP	4
regulator of cell cycle	gene	RGCC	4
indoleamine 2,3-dioxygenase 1	gene	IDO1	4
interleukin 18	gene	IL18	4
interleukin 17A	gene	IL17A	4
interferon gamma	gene	IFNG	4
hippocalcin	gene	HPCA	4
elongation factor for RNA polymerase II 3	gene	ELL3	4
SPARC like 1	gene	SPARCL1	3
UDP glycosyltransferase 8	gene	UGT8	3
fibromodulin	gene	FMOD	3
nuclear pore complex interacting protein family, member B13	gene	NPIPB13	3
TAM41 mitochondrial translocator assembly and maintenance homolog	gene	TAMM41	3
TNF alpha induced protein 2	gene	TNFAIP2	3
sorting nexin 14	gene	SNX14	3
EPH receptor B4	gene	EPHB4	3
lymphocyte antigen 96	gene	LY96	3
tripartite motif containing 59	gene	TRIM59	3
fibroblast growth factor 5	gene	FGF5	3
FRAS1 related extracellular matrix protein 2	gene	FREM2	3
nanos C2HC-type zinc finger 1	gene	NANOS1	3
transcription elongation factor A like 5	gene	TCEAL5	3
dual specificity phosphatase 2	gene	DUSP2	3
WAP, follistatin/kazal, immunoglobulin, kunitz and netrin domain containing 2	gene	WFIKKN2	3
calbindin 1	gene	CALB1	3
topoisomerase (DNA) I, mitochondrial	gene	TOP1MT	3
glutamate ionotropic receptor NMDA type subunit 3B	gene	GRIN3B	3
retinol dehydrogenase 13	gene	RDH13	3
sideroflexin 5	gene	SFXN5	3
phosphatidylinositol 4-kinase type 2 beta	gene	PI4K2B	3

transmembrane protein 259	gene	TMEM259	3
acid phosphatase 2, lysosomal	gene	ACP2	3
heparan sulfate 6-O-sulfotransferase 2	gene	HS6ST2	3
butyrophilin subfamily 1 member A1	gene	BTN1A1	3
biotinidase	gene	BTD	3
sphingomyelin phosphodiesterase acid like 3A	gene	SMPDL3A	3
misato 1, mitochondrial distribution and morphology regulator	gene	MSTO1	3
arachidonate 15-lipoxygenase, type B	gene	ALOX15B	3
plexin A2	gene	PLXNA2	3
nicotinamide N-methyltransferase	gene	NNMT	3
MAPK regulated corepressor interacting protein 2	gene	MCRIP2	3
neogenin 1	gene	NEO1	3
zinc finger and BTB domain containing 5	gene	ZBTB5	3
metallothionein 1A	gene	MT1A	3
CD99 molecule like 2	gene	CD99L2	3
zinc finger DHHC-type containing 3	gene	ZDHHC3	3
5-methyltetrahydrofolate-homocysteine methyltransferase reductase	gene	MTRR	3
sphingolipid transporter 1 (putative)	gene	SPNS1	3
MTND2	gene	ND2	3
inducible T-cell costimulator	gene	ICOS	3
keratin 81	gene	KRT81	3
INSM transcriptional repressor 1	gene	INSM1	3
lipase E, hormone sensitive type	gene	LIPE	3
phosphatidylethanolamine binding protein 4	gene	PEBP4	3
ATP/GTP binding protein like 2	gene	AGBL2	3
interferon alpha 13	gene	IFNA13	3
interferon alpha 1	gene	IFNA1	3
interferon alpha inducible protein 27	gene	IFI27	3
inositol-trisphosphate 3-kinase C	gene	ITPKC	3
TM2 domain containing 3	gene	TM2D3	3
UDP-glucuronate decarboxylase 1	gene	UXS1	2
aldehyde dehydrogenase 1 family member A2	gene	ALDH1A2	2
natural killer cell cytotoxicity receptor 3 ligand 1	gene	NCR3LG1	2
sterol O-acyltransferase 2	gene	SOAT2	2
RAS protein activator like 1	gene	RASAL1	2
xanthine dehydrogenase	gene	XDH	2
GATA binding protein 5	gene	GATA5	2
NIMA related kinase 7	gene	NEK7	2
dual specificity phosphatase 26	gene	DUSP26	2
WNK lysine deficient protein kinase 3	gene	WNK3	2
interleukin 1 receptor type 2	gene	IL1R2	2
metaxin 3	gene	MTX3	2

lymphocyte antigen 6 family member G5B	gene	LY6G5B	2
engrailed homeobox 2	gene	EN2	2
dopamine receptor D5	gene	DRD5	2
zinc finger protein 385A	gene	ZNF385A	2
SPARC/osteonectin, cwcv and kazal like domains proteoglycan 1	gene	SPOCK1	2
dual specificity phosphatase 5	gene	DUSP5	2
ATM interactor	gene	ATMIN	2
desmoglein 3	gene	DSG3	2
chromosome 10 open reading frame 90	gene	C10orf90	2
phosphodiesterase 10A	gene	PDE10A	2
prolyl 3-hydroxylase 2	gene	P3H2	2
chromosome 1 open reading frame 27	gene	C1orf27	2
purinergic receptor P2Y1	gene	P2RY1	2
bone morphogenetic protein 1	gene	BMP1	2
biliverdin reductase B	gene	BLVRB	2
autophagy related 4D cysteine peptidase	gene	ATG4D	2
5'-nucleotidase domain containing 3	gene	NT5DC3	2
RNA binding motif protein 47	gene	RBM47	2
mitochondrial translational initiation factor 3	gene	MTIF3	2
KIAA0087 lncRNA	gene	KIAA0087	2
matrix metallopeptidase 17	gene	MMP17	2
ubiquitin specific peptidase 26	gene	USP26	2
peptidyl arginine deiminase 1	gene	PADI1	2
LIM homeobox 1	gene	LHX1	2
marginal zone B and B1 cell specific protein	gene	MZB1	2
phosphodiesterase 5A	gene	PDE5A	2
immunoglobulin heavy constant gamma 4 (G4m marker)	gene	IGHG4	2
hexokinase domain containing 1	gene	HKDC1	1
collagen like tail subunit of asymmetric acetylcholinesterase	gene	COLQ	1
cartilage intermediate layer protein	gene	CILP	1
proline rich membrane anchor 1	gene	PRIMA1	1
neuronal PAS domain protein 3	gene	NPAS3	1
tumor protein p53 regulated apoptosis inducing protein 1	gene	TP53AIP1	1
leucine rich repeat containing 32	gene	LRRC32	1
transmembrane protein 139	gene	TMEM139	1
GUF1 homolog, GTPase	gene	GUF1	1
TCDD inducible poly(ADP-ribose) polymerase	gene	TIPARP	1
KIAA1456	gene	KIAA1456	1
beclin 2	gene	BECN2	1
family with sequence similarity 45, member A	gene	FAM45BP	1

pseudogene			
ubiquitin specific peptidase 29	gene	USP29	1
glutamate rich 2	gene	ERICH2	1
thioesterase superfamily member 4	gene	THEM4	1
calcium/calmodulin dependent protein kinase II inhibitor 1	gene	CAMK2N1	1
zinc finger protein 821	gene	ZNF821	1
family with sequence similarity 45 member A	gene	FAM45A	1
canopy FGF signaling regulator 4	gene	CNPY4	1
apoptosis inducing factor, mitochondria associated 2	gene	AIFM2	1
phospholipase C beta 4	gene	PLCB4	1
zinc finger protein 691	gene	ZNF691	1
chromosome 4 open reading frame 48	gene	C4orf48	1
collagen type V alpha 3 chain	gene	COL5A3	1
NADPH oxidase 4	gene	NOX4	1
thyrotropin releasing hormone degrading enzyme	gene	TRHDE	1
Fc fragment of IgA and IgM receptor	gene	FCAMR	1
beta-1,4-galactosyltransferase 5	gene	B4GALT5	1
solute carrier family 35 member G2	gene	SLC35G2	1
ring finger protein 39	gene	RNF39	1
lumican	gene	LUM	1
solute carrier family 2 member 12	gene	SLC2A12	1
steroid 5 alpha-reductase 3	gene	SRD5A3	1

Results for Topology Analysis of Network Structure - Sub Network

bg.annotation. Name	Degree	Betweenness Centrality	Closeness Centrality	LAC	Neighborhood Connectivity
HUWE1	239	0.00351561	0.49610176	35.83240223	104.3164557
SNW1	226	0.00441138	0.49096447	28.76433121	96.69196429
MLH1	77	0.001178	0.43551873	8.486486486	76.41558442
ATXN1	78	9.74E-04	0.43178571	12.65116279	97.73684211
ATXN3	52	2.59E-04	0.44196673	11.7804878	126.22
ITCH	80	6.21E-04	0.46428571	13.64285714	111.8461539
MET	63	5.94E-04	0.42480675	12.82857143	87.3442623
MAP3K5	65	4.50E-04	0.44019661	12.28888889	113.6825397
MEN1	66	1.39E-04	0.43828167	19.4	156.469697
MAP3K1	138	9.93E-04	0.4781491	24.46728972	122.7794118
MAP3K3	135	8.75E-04	0.46375144	21.8411215	116.518797
CTNNB1	222	0.00613691	0.50145168	23.98648649	99.84684685
CTNNA1	56	5.14E-04	0.45187815	10.94736842	133.2407407

MDM2	295	0.0093704	0.5125053	29.1920904	87.39590444
RXRA	100	0.00189481	0.44083865	16.62	78.26530612
MDC1	98	8.24E-04	0.45838863	17.13432836	107.84375
CTBP1	83	9.33E-04	0.46339594	14.16	108.1728395
LGALS3BP	78	4.54E-04	0.43963636	24.86440678	134.5921053
IKBKE	165	0.00225382	0.46473189	18.14545455	84.77300613
NME1-NME2	54	2.79E-04	0.42257952	11.72222222	96.42307692
LDHB	66	3.77E-04	0.43820225	15.78723404	121.65625
RPS27A	192	0.00378328	0.49610176	31.88888889	114.3789474
LDHA	67	6.84E-04	0.44827586	17.46153846	144.8307692
RPS27	85	1.54E-04	0.45145631	44.59493671	143.060241
LCK	72	5.81E-04	0.44645495	12.4	100.3857143
RPS24	89	1.30E-04	0.44802668	53.05	140.0674157
RPS26	84	8.70E-05	0.45103525	49.64102564	155.5238095
RPS25	91	1.05E-04	0.46330715	53.15909091	159.5384615
LRIF1	80	6.53E-04	0.41920943	11.89583333	87
NCOR2	91	5.65E-04	0.45682978	19.85714286	119.6373626
RPS17	67	3.72E-05	0.44156318	38.95454545	178.8507463
NCOR1	127	0.00102372	0.46127432	28.73626374	113.624
CSNK2B	123	0.0026925	0.47421063	15.26315789	100.9834711
RPS19	102	3.32E-04	0.46392939	53.25806452	144.16
NCL	147	9.38E-04	0.4823459	35.81395349	142.9241379
RPS18	107	6.19E-04	0.46634523	49.64948454	149.8504673
RPS15	75	1.66E-04	0.43828167	48.8030303	137.04
NCK1	75	7.50E-04	0.42052174	9.3	61.38666667
RPS14	118	2.87E-04	0.46806039	51.79464286	155.8559322
RPS16	114	2.50E-04	0.47143693	53.82407407	151.7105263
NBN	77	4.22E-04	0.42788887	17.88235294	107.4545455
CSNK2A1	62	2.49E-04	0.44555003	9.209302326	113.9516129
CSNK1A1	68	8.09E-04	0.45769449	11.89130435	125.8676471
RPS10	105	3.01E-04	0.46661521	50.87628866	157.9428571
RPS13	100	1.84E-04	0.46206765	53.6344086	149.68
CSNK1E	58	6.49E-04	0.456571	11.52777778	111.1785714
RBM39	60	1.93E-04	0.45960844	16.42857143	167.9166667
NAP1L1	82	6.54E-04	0.46419658	23.13846154	157.1375
RPS7	103	1.76E-04	0.45571052	49.96	157.8932039
RPS9	99	1.94E-04	0.46842309	52.72826087	159.5353535
HNRNPM	121	5.27E-04	0.47328244	33.375	139.0578512
RPS8	141	5.97E-04	0.46997085	54.63865546	138.4042553
RPS6KA3	71	0.00362864	0.44212836	12.35714286	113.3188406
HNRNPK	136	0.00115925	0.48427799	32.23478261	156.5895522
RPS6KB1	53	2.74E-04	0.44375115	10.84210526	117.6470588
HNRNPF	78	3.32E-04	0.46491059	24.85074627	173.3461539
HNRNPD	117	0.00104072	0.46960575	27.79545455	132.9043478

RPS6KA1	59	5.16E-04	0.44059767	10.02857143	101.5789474
HNRNPH1	90	3.04E-04	0.46652518	26.53164557	164.2888889
MYO1C	61	2.80E-04	0.45263946	14	127.9016393
HNRNPA2B1	103	4.87E-04	0.47235788	26.06818182	154.9009901
RPS6	123	4.98E-04	0.46815102	55.08333333	140.8780488
HNRNPA1	223	0.00402117	0.49671323	43.03289474	113.2533937
RPS3	141	4.95E-04	0.47852761	52.16923077	146.5177305
HNRNPC	85	8.07E-04	0.46473189	21.21917808	161.6987952
MAPK14	132	0.00399923	0.48554217	16.2238806	92.99230769
BTRC	145	0.00352029	0.48427799	20.16853933	98.31468531
RPS2	132	7.62E-04	0.46869548	51.4122807	136.0454546
COPS4	52	2.04E-04	0.44513991	16.6	143.66
RPS4X	121	2.90E-04	0.47125317	57.0625	152.8181818
HIST1H3F	228	0.00133897	0.47673502	44.42613636	100.4513274
MYH9	141	0.00201442	0.48167331	24.40449438	114.1560284
RPS3A	125	4.60E-04	0.47542273	55.25217391	154.92
CSE1L	52	3.10E-04	0.4580413	13.88095238	170.9230769
HNF4A	80	0.00125189	0.4522162	14.175	95.58974359
BAG2	70	2.49E-04	0.45864947	18.38596491	151.0882353
BAG3	155	0.00405629	0.46634523	15.63333333	79.60784314
NR4A1	69	0.00105415	0.47116134	14.01785714	123.0597015
FAF2	69	4.26E-04	0.43442328	11.26190476	102.238806
HMGA1	56	2.55E-04	0.45700246	14.58695652	150.9285714
CRKL	53	3.93E-04	0.43240343	11.51428571	108.8301887
CRK	140	0.00308128	0.48100259	18.39473684	90.10714286
CACYBP	54	4.61E-04	0.43844062	8.722222222	106.4423077
MYC	277	0.00786604	0.50628141	28.61202186	90.73818182
COMMD3-BMI1	140	0.00144262	0.46634523	21.51724138	97.81884058
HERC2	74	0.00102451	0.44342564	9.441176471	79.77777778
ATF2	119	0.00174155	0.4693323	14.49367089	93.11111111
CREB1	85	9.01E-04	0.45830174	19.92857143	117.5060241
CREBBP	230	0.00541069	0.49569496	35.53146853	96.14473684
HMGB1	62	9.03E-04	0.46357362	9.230769231	117.4677419
SHC1	143	0.00219636	0.47162083	22.57471264	98.02836879
CCDC8	239	0.00342307	0.49076517	29.35928144	96.96202532
CHEK2	75	0.0013652	0.45882353	11.76315789	97.5890411
SQSTM1	119	0.00214347	0.4823459	18.30864198	114.0940171
HLA-B	107	0.00130743	0.4398763	10.66153846	73.38095238
SSX2IP	59	4.00E-04	0.44212836	8.21875	90.66666667
RPL23	113	8.20E-04	0.47189696	47.63	137
PABPC1	137	6.95E-04	0.4749558	37.05263158	144.5185185
TRIM27	78	8.58E-04	0.44969314	10.85	92.68421053
RNF11	56	3.51E-04	0.43201715	11.18918919	94.57407407

HIF1A	104	0.0010368	0.47881188	20.45	131.9903846
RFC1	54	2.41E-04	0.4489417	17.56097561	161.7307692
KAT2B	131	0.00131465	0.46778874	27.25	116.0465116
COPS2	84	4.17E-04	0.44432194	21.66129032	138.9756098
TSC22D1	66	6.16E-04	0.43669857	7.206896552	80.125
HDAC3	165	0.00232581	0.47928642	31.26363636	109.202454
AP2M1	78	6.85E-04	0.44752915	15.76923077	108.8157895
HDAC2	181	0.00175747	0.47579693	34.09375	111.3407821
UHRF1	58	9.29E-05	0.43804348	26.85185185	176.6428571
HDAC1	275	0.0066851	0.49701953	37.93413174	90.48717949
KRT18	84	9.26E-04	0.45778114	18.55555556	140.195122
RELA	192	0.00397214	0.49156333	25.40322581	99.03684211
HTT	116	0.00244414	0.48119403	17.44444444	110.2719298
KAT7	57	2.64E-04	0.43963636	17.97560976	148.4385965
BRD7	53	1.43E-04	0.42066806	20.85365854	149.754717
CHUK	108	0.00105971	0.48379352	23.02352941	130.254717
IQGAP1	117	0.00124761	0.4835033	22.89285714	127.991453
AMBRA1	60	1.73E-04	0.42600423	18	112.35
TRIM24	59	1.20E-04	0.43804348	20.26923077	167.2631579
TBK1	91	0.00102655	0.46742702	19.26865672	127.1460674
HIST1H2AE	56	3.90E-05	0.42555438	28.75925926	182.8148148
CDC37	126	0.0020072	0.47411765	21.10843373	107.8064516
KPNA2	91	7.27E-04	0.47589057	17.1641791	139.7191011
H2AFX	158	0.00171201	0.47758246	31.09565217	121.1410256
KPNB1	101	9.97E-04	0.47421063	17.79452055	139.2222222
RBL1	54	3.56E-04	0.43395549	12	114.3269231
KIF11	56	7.53E-04	0.42766183	13.18918919	130.8703704
PDLIM7	60	4.34E-04	0.4511194	9.078947368	115.4666667
RBBP4	119	0.00101804	0.46428571	32.46067416	139.4033613
RPS6KA5	65	2.84E-04	0.43178571	19.76923077	143.3230769
RBBP7	86	3.09E-04	0.44334433	27.890625	139.2209302
RB1	176	0.00322126	0.48769665	25.17094017	99.90229885
AXIN1	59	4.02E-04	0.4491919	12.13888889	112.4736842
RASA1	68	6.63E-04	0.44794368	13.37209302	100.2424242
MTA2	64	3.41E-04	0.44612546	18.06122449	151.59375
TRRAP	57	5.14E-04	0.43481388	9.09375	108.7017544
AURKB	105	8.42E-04	0.46987952	20.05555556	133.0485437
HNRNPUL1	59	2.47E-04	0.45391402	15.46938776	164.3859649
CHD4	71	2.06E-04	0.43725136	20.76363636	149.4782609
HIST1H4I	148	6.12E-04	0.4749558	28.1	138.0540541
HIST3H3	137	0.00101395	0.45314843	27.08421053	99.35555556
CHD3	85	4.85E-04	0.4511194	20.265625	126.8915663
CHEK1	68	8.62E-04	0.47189696	13.66	146.25
SMC1A	73	2.87E-04	0.44399559	18.27118644	154.4225352

RARA	95	9.65E-04	0.45622642	16.63157895	96.72043011
USP11	68	6.32E-04	0.45069897	9.815789474	99.21212121
RCC1	69	3.08E-04	0.43551873	20.47058824	139.8955224
RANBP2	68	2.65E-04	0.45493885	17.70175439	152.9848485
MRPL58	56	3.00E-04	0.40414508	8.115384615	73.625
WDR5	108	8.88E-04	0.47079439	24.65822785	139.5740741
ICAM1	91	4.49E-04	0.42066806	28.44155844	120.3033708
NCOA3	98	5.53E-04	0.46833236	20.84146341	128.2708333
RAN	77	4.78E-04	0.44538589	13.75	131.5466667
LOC400927-CSNK1E	58	6.49E-04	0.456571	11.52777778	111.1785714
RAF1	123	0.00283023	0.46860465	17.57534247	96.69421488
HGS	86	0.0017354	0.455453	10.63636364	86.6627907
TOPBP1	57	3.03E-04	0.42428496	8.975	119.2
RAD23A	81	4.16E-04	0.45280899	24.06666667	109.3924051
JUN	160	0.0027743	0.47824367	25.32989691	95.63291139
RAD51	59	8.60E-04	0.45019549	11.28571429	112.6491228
HSPD1	119	0.00166415	0.48052464	29.01020408	159.4871795
DNAJB1	58	5.59E-04	0.46455331	13.31578947	141.3965517
SMC3	74	3.13E-04	0.4489417	16.06666667	160.3648649
HSPE1	62	8.08E-04	0.42743504	9.432432432	94.9516129
CFL1	85	0.00148689	0.45734821	17.125	122.4117647
HSP90AB1	282	0.00703629	0.5125053	37.90104167	104.6285714
RAC1	100	0.00215547	0.45213164	13.65	97.95918367
HSP90AA1	344	0.01370627	0.52519548	40.88151659	96.34210526
HSPA9	125	0.00105289	0.49026764	25.27102804	151.144
HSPA8	219	0.00371017	0.50883838	39.27218935	126.9677419
HSPB1	148	0.00286124	0.48622562	26	118.8013699
RIPK2	53	2.40E-04	0.41993748	13.22222222	99.76470588
IRS1	68	3.81E-04	0.4593465	15.68	128.6060606
HSPA5	227	0.00435605	0.50218069	40.39204545	126.6711111
HSPA4	188	0.00385744	0.49896822	28.46616541	120.2553192
CEBPB	70	8.96E-04	0.46004566	19.5	125.0441177
PABPC4	66	1.12E-04	0.44156318	20.68852459	165.4242424
HSPA1B	127	9.19E-04	0.48456914	24.71287129	139.8503937
CEBPA	64	6.49E-04	0.43186283	15.75	108.016129
IRAK1	90	0.001163	0.45204711	17.03448276	102.7045455
HSPA1A	127	9.19E-04	0.48456914	24.71287129	139.8503937
PXN	115	0.00133201	0.45734821	19.19512195	100.300885
HSPA1L	82	5.19E-04	0.46679537	18.47887324	145
INSR	54	2.54E-04	0.43116976	13.875	97.34615385
CDKN2A	175	0.00731951	0.48369674	18.24096386	84.50857143
DNAJA1	85	2.80E-04	0.46824167	21.96202532	163.5647059
CDKN1A	162	0.00300227	0.48878108	22.62745098	103.9875

HSF1	66	3.59E-04	0.43333333	9.936170213	104.84375
MCM7	109	8.69E-04	0.46286371	26.53409091	142.6261682
CDK9	106	9.18E-04	0.46215596	16.27777778	106.7403846
RIPK1	73	5.16E-04	0.43740955	19.30612245	100.3802817
PRMT1	102	9.80E-04	0.46428571	26.03896104	150.2
MCM3	92	4.01E-04	0.44952593	23.94444444	148.423913
CUL5	191	0.00196625	0.46987952	37.2027027	109.3403141
MCM6	71	2.41E-04	0.4353619	24.21428571	156.7101449
CDKN1B	61	3.03E-04	0.44877506	13.36956522	135.3114754
MCM5	129	6.73E-04	0.46760781	29.51327434	136.5581395
MCM2	366	0.01039159	0.51228814	47.04313725	95.69230769
CDK6	52	2.37E-04	0.43294539	11.375	112.94
HRAS	63	0.00100273	0.43310048	7.193548387	76.13114754
RUVBL2	114	7.17E-04	0.4764532	24.0212766	150.0535714
MCC	105	0.00150954	0.44711538	9.052631579	72.8
CDK8	66	4.02E-04	0.44220922	12.26086957	100.0625
CDK2	349	0.00854378	0.51012658	40.04149378	94.84149856
CDK5	61	6.84E-04	0.46162658	12.04651163	125.0169492
MAPT	74	5.16E-04	0.4603084	16.12962963	125.375
CDK4	84	0.00132807	0.45838863	12.96363636	111.2380952
SFN	127	0.00139738	0.47180488	20.42465753	100.24
GNB2	61	4.21E-04	0.455453	9.94	143.0983607
RPL14	121	5.92E-04	0.45769449	53.33653846	141.7603306
CDC42	82	0.00172742	0.46392939	9.133333333	87.6125
MED23	76	6.72E-04	0.43032568	9.275	75.86842105
CDH1	83	0.00104365	0.45196262	10.94117647	94.97530864
CDC27	59	5.47E-04	0.44212836	9.684210526	108.4035088
SMAD7	53	4.10E-04	0.45562465	13.17948718	130.3333333
PTPN11	77	8.47E-04	0.43820225	15.88095238	82.76
SMAD2	173	0.00393168	0.49026764	20.65	91.52631579
SMAD1	90	0.0021368	0.47162083	13.74509804	97.18181818
OFD1	74	6.54E-04	0.44522187	9.7	87.44444444
SMAD4	121	0.00243218	0.46960575	18.77419355	92.23529412
SMAD3	193	0.00549411	0.49907121	25.52586207	94.7486911
RAE1	56	3.43E-04	0.44424031	8.264705882	111.4642857
CDC5L	225	0.00422618	0.4765471	30.62	90.86098655
CDK1	168	0.00302329	0.48691099	24.78703704	109.1506024
RFWD2	54	1.77E-04	0.43186283	13.57142857	119.6346154
PTPN1	67	9.84E-04	0.44703272	11.02631579	94.86153846
LYN	96	0.00244539	0.47356052	13.77777778	98.17021277
SMARCA5	89	2.87E-04	0.44944238	30.13157895	159.1724138
IRS4	64	1.76E-04	0.46022078	15.11320755	155.0967742
CD81	109	9.01E-04	0.4354403	25.56321839	111.6448598
EED	218	0.00285673	0.47042802	34.175	99.22685185

UCHL5	102	0.00100244	0.44869178	20.73417722	101.64
CUL2	217	0.00244799	0.47843292	34.3373494	103.9078341
ATRX	55	9.05E-05	0.41993748	22.9375	150.7636364
TRADD	60	2.59E-04	0.43017257	14.23255814	104.5689655
PHB	73	5.59E-04	0.4658062	16.7	160.0684932
CEP128	65	6.92E-04	0.43654089	6.04	66.26153846
CUL3	435	0.01416689	0.52078398	46.47482014	85.31177829
UBR5	60	6.07E-04	0.45769449	11.46666667	132.8448276
CUL1	317	0.0054367	0.49927731	45.54273504	99.4031746
PGR	54	1.94E-04	0.43263553	12.41666667	112.4423077
FBXW7	103	0.00166562	0.44794368	10.1372549	77.86138614
CUL4A	146	0.00106353	0.46969697	30.51376147	119.9097222
ATR	75	7.94E-04	0.45562465	13.05128205	104.520548
CUL4B	164	0.00111449	0.48004765	34.65384615	119.1341463
FZR1	52	4.86E-04	0.43450135	7.24	93.03846154
IGSF8	99	3.27E-04	0.42354178	28.06097561	113.1313131
RPLP2	94	1.29E-04	0.45899772	52.62921348	152.7065217
RPLP0	127	4.89E-04	0.47346779	54.36521739	151.8031496
LMNA	114	9.65E-04	0.47245018	24.53571429	139.0089286
RPL37A	89	1.31E-04	0.44794368	49.30487805	144.752809
ATP5A1	78	4.43E-04	0.46724638	23.58208955	174.131579
RPL31	102	8.79E-04	0.46206765	51.64948454	153.372549
RPL30	82	1.43E-04	0.45391402	50.64102564	147.097561
RPL27	96	1.82E-04	0.44342564	52.87640449	144.9791667
KAT2A	91	5.68E-04	0.4489417	22.34328358	128.4157303
HIST1H4L	148	6.12E-04	0.4749558	28.1	138.0540541
DYNLL1	89	0.00135403	0.4410799	10.37037037	86.48275862
UBQLN1	72	0.00207475	0.44253294	12.38888889	84.65714286
HIST2H4A	148	6.12E-04	0.4749558	28.1	138.0540541
HIST1H4H	148	6.12E-04	0.4749558	28.1	138.0540541
HIST1H4C	148	6.12E-04	0.4749558	28.1	138.0540541
RPL26	68	9.43E-05	0.43271296	36	154.7205882
HIST1H4E	148	6.12E-04	0.4749558	28.1	138.0540541
RPL24	117	2.99E-04	0.45821489	52.4375	152.1282051
HIST1H4B	148	6.12E-04	0.4749558	28.1	138.0540541
RPL21	91	1.35E-04	0.45425512	54.54216867	147.8901099
RPL23A	108	3.06E-04	0.4623327	53.18556701	145.5
RPL18	105	1.52E-04	0.45960844	57.55102041	151.6380952
RPL19	101	1.10E-04	0.45348837	53.94680851	159.7029703
NCOA1	75	6.84E-04	0.45502446	19.19230769	112.9452055
YWHAZ	315	0.00878523	0.51755137	45.6561086	107.884984
RPL18A	90	2.05E-04	0.45162495	50.27160494	141.5111111
RPL12	94	2.26E-04	0.46312967	51.73333333	155
SF1	76	3.70E-04	0.43551873	16.3	120.6891892

RPL11	109	2.96E-04	0.46670527	54.03	147.1559633
ATM	175	0.01056604	0.47476929	17.82857143	67.46820809
ILK	111	9.15E-04	0.45596832	15.98648649	96.39449541
YWHAB	169	0.00278678	0.48138563	24.69473684	97.73053892
RPL15	99	2.03E-04	0.45340334	56.06741573	147.8484849
YY1	77	4.53E-04	0.46482122	16.80701754	131.1466667
YWHAG	190	0.00292692	0.50031037	30.70247934	114.1755319
RPL13	120	5.33E-04	0.47042802	44.69607843	138.0416667
YWHAE	203	0.00342136	0.49742851	34.42222222	116.7263682
CSNK2A3	62	2.49E-04	0.44555003	9.209302326	113.9516129
TP63	89	9.94E-04	0.45873648	14.32075472	102.1839081
RPL8	102	1.72E-04	0.45786783	54.29347826	149.5392157
RPL7A	106	1.98E-04	0.46136234	55.51546392	160.9716981
GAPDH	134	0.00205104	0.49246436	27.66336634	145.0454546
XRCC5	136	0.00155952	0.46905917	24.37962963	128.9552239
RPL5	125	4.61E-04	0.46896819	55.70642202	141.2845529
XPO1	366	0.0160479	0.51611526	30.79329609	74.9010989
RPL7	117	1.82E-04	0.46589595	55.03669725	151.5652174
RPL6	138	5.83E-04	0.46787926	56.45132743	135.5797101
RPL3	103	2.01E-04	0.46375144	52.69473684	157.7184466
RPL4	117	2.29E-04	0.46661521	53.40366972	145
CBX3	63	2.20E-04	0.41747238	25.18518519	153.9016393
RPA2	196	0.00255475	0.48967193	25.088	96.70408163
RPA1	209	0.00263399	0.48759831	31.3	101.468599
MTOR	86	0.00147533	0.46997085	14.31034483	110.0476191
PHB2	58	3.28E-04	0.45002792	11.1	143.9827586
GTF2I	73	6.15E-04	0.46535797	18.03703704	168.6197183
IKBKB	119	0.00137117	0.48720532	22.58139535	114.2905983
ARRB1	150	0.00193836	0.48486064	21.17857143	114.8666667
HSPH1	60	1.32E-04	0.43255814	12.11538462	140.25
ARRB2	196	0.00357038	0.48282748	28.09459459	101.8877551
PIAS1	73	7.07E-04	0.45838863	15.3	110.6056338
WDR77	54	1.59E-04	0.45382883	14.5	179.2307692
ABCE1	90	0.00107388	0.46464258	12.92592593	104.5113636
VIM	135	0.00203142	0.49016825	18.7826087	122.2406015
VHL	208	0.00366173	0.49226384	28.40397351	102.5631068
GSK3B	159	0.00429012	0.4918633	18.32258065	92.87898089
RNF2	262	0.00531045	0.49387255	29.00555556	89.42307692
VDR	69	5.86E-04	0.44952593	15.78571429	105.4179105
VDAC1	57	5.25E-04	0.43963636	8.342105263	109.5263158
OTUB1	94	9.38E-04	0.47079439	13.68852459	113.5744681
YBX3	55	1.80E-04	0.42910382	13.36363636	160.3454546
VCP	277	0.00817942	0.51196274	37.25280899	102.3854546
AR	213	0.00557338	0.50020687	27.52631579	96.23696682

IKBKG	191	0.00518447	0.49076517	26.8487395	95.65608466
FBXO6	239	0.00731354	0.4764532	25.32692308	86.43933054
HIST1H4F	148	6.12E-04	0.4749558	28.1	138.0540541
IGF1R	59	6.43E-04	0.46419658	17.80487805	148.1052632
FAS	54	8.33E-04	0.42879943	9.142857143	86.96153846
HIST1H4D	148	6.12E-04	0.4749558	28.1	138.0540541
NR3C1	137	0.00261766	0.48215354	20.61538462	99.38518519
HIST1H4J	148	6.12E-04	0.4749558	28.1	138.0540541
HIST1H4K	148	6.12E-04	0.4749558	28.1	138.0540541
HIST1H3H	228	0.00133897	0.47673502	44.42613636	100.4513274
HIST1H3J	228	0.00133897	0.47673502	44.42613636	100.4513274
HIST1H4A	148	6.12E-04	0.4749558	28.1	138.0540541
HIST1H3B	228	0.00133897	0.47673502	44.42613636	100.4513274
HIST1H3E	228	0.00133897	0.47673502	44.42613636	100.4513274
GRIN1	57	0.00210383	0.39933939	4.533333333	48.85454545
HIST1H3C	228	0.00133897	0.47673502	44.42613636	100.4513274
UQCRC2	56	2.38E-04	0.42622951	11.74418605	110.6964286
HIST1H3G	228	0.00133897	0.47673502	44.42613636	100.4513274
HIST1H3I	228	0.00133897	0.47673502	44.42613636	100.4513274
PTGES3	59	2.69E-04	0.43971631	12.29787234	123.9122807
HIST2H2BE	87	4.14E-04	0.44530387	30.37313433	149.7356322
GRB2	305	0.01424428	0.50638743	33.72121212	86.62706271
HIST1H3D	228	0.00133897	0.47673502	44.42613636	100.4513274
ACTL6A	58	2.78E-04	0.44835898	15.02173913	156.0172414
HIST1H3A	228	0.00133897	0.47673502	44.42613636	100.4513274
ACTN4	71	8.60E-04	0.4431006	11.29166667	119.2253521
HIST1H2AB	56	3.90E-05	0.42555438	28.75925926	182.8148148
CCT8	79	2.72E-04	0.47189696	24.33846154	165.8101266
ACTG1	106	8.66E-04	0.45769449	20.66666667	126.1634615
SENP3	60	2.07E-04	0.45434047	13.33333333	149.1
IFI16	81	7.59E-04	0.45596832	7.285714286	87.87654321
UBE3A	78	0.00110544	0.46652518	13.61702128	116.5526316
SUMO1	115	0.00112957	0.46446408	16.69117647	96.52212389
ACTB	155	0.00245843	0.49226384	26.59649123	122.1111111
UNC119	56	4.93E-04	0.40367279	1.913043478	50.96428571
UBE2N	63	0.00108068	0.45348837	11.13513514	115.4754098
NOC2L	57	1.09E-04	0.44505798	21.16666667	186.1403509
UBE2I	251	0.00664791	0.4983512	26.32679739	87.75100402
ACTA1	85	0.00120051	0.46969697	14.52830189	113.7349398
DNAJA3	54	2.85E-04	0.44497608	12.10638298	140.8888889
SART1	56	1.16E-04	0.4491919	19.34042553	180.037037
UBB	98	0.00104965	0.47701716	16.15789474	126.9795918
SERBP1	70	1.58E-04	0.45795455	22.50769231	179.1029412
ASH2L	56	2.46E-04	0.43915728	13.89189189	136.4814815

TXN	60	3.47E-04	0.46242111	12.625	152.65
KHDRBS1	91	8.11E-04	0.4569161	16.81818182	133.2921348
TUBG1	147	0.00137922	0.48311688	30.69747899	126.2965517
TUBA4A	87	8.28E-04	0.46508944	18.68571429	140.5517241
TUBB2A	52	2.13E-04	0.44777778	11.23809524	141.25
PCNA	166	0.00249879	0.48282748	25.76470588	112.3231707
HNRNPL	73	2.45E-04	0.4580413	24.1875	181.056338
PCMT1	52	3.54E-04	0.4522162	9.153846154	165.2
HNRNPU	229	0.00372112	0.49498465	47.14367816	113.0611354
EIF6	86	7.12E-04	0.44802668	16.22033898	108.7093023
MAP3K14	95	3.46E-04	0.44180523	28.50617284	120.1505376
PCBP2	62	3.25E-04	0.4400364	18.68	152.9
PCBP1	94	0.00139417	0.47393179	15.72727273	132.4361702
BAG6	103	0.00216036	0.45665722	9.846153846	79.52475248
USP7	128	0.00180434	0.47235788	17.59302326	108.5634921
TUBA1A	106	0.00146843	0.47476929	18.61728395	130.754717
PARK2	239	0.00400321	0.49066558	28.62048193	93.48101266
PAK2	86	6.45E-04	0.45230079	14.58064516	115.4761905
FOS	97	0.00105718	0.45485327	13.72131148	90.69473684
PAK1	92	6.83E-04	0.46259805	19.34328358	131.0666667
FN1	343	0.01222802	0.50134771	39.18965517	88.70381232
PA2G4	78	6.06E-04	0.45434047	16.08333333	132.8974359
P4HB	66	4.58E-04	0.44736355	18.02083333	156.921875
FLNA	152	0.00313715	0.48661703	27.03846154	125.1533333
CLTC	146	0.00221486	0.48799193	24.64761905	126.7847222
FOXO3	52	3.52E-04	0.46215596	13.875	157
FKBP5	52	2.77E-04	0.44172452	11.45945946	122.6923077
FHL2	62	8.57E-04	0.44994418	13.1	108.5833333
CLU	55	7.37E-04	0.45382883	10.10810811	122.3584906
C1QBP	98	0.00123396	0.46004566	19.52173913	125.2604167
RPL10L	58	6.85E-05	0.42668078	45.60714286	146.5517241
BUB1	57	7.45E-04	0.41682469	5	63.23636364
SF3B2	59	3.90E-04	0.45162495	13.40425532	167.8983051
BTK	62	6.12E-04	0.45204711	10.72972973	105.85
ZBTB16	70	9.72E-04	0.45095114	15.28571429	117.6470588
COPS5	333	0.00805329	0.51294018	45.95633188	97.41087613
FASN	55	1.18E-04	0.44902507	16.42	186.8679245
COPS6	146	0.00213283	0.47900158	28.58585859	108.5416667
BRCA1	277	0.00827234	0.51185436	33.09042553	95.98181818
YWHAQ	225	0.00393018	0.48730351	30.98657718	100.67713
TTN	54	7.68E-04	0.44212836	11	133.9423077
DNAJC7	55	1.85E-04	0.45170932	16.66666667	146.9245283
STIP1	83	6.12E-04	0.4541698	16.3	115.0963855
PTMA	53	1.10E-04	0.42257952	13.875	133.6226415

XRCC6	149	0.00222378	0.48253842	27.30172414	130.9931973
TSG101	72	0.00115765	0.46127432	10.97777778	109.9571429
PTK2	74	0.00111775	0.46616541	16.66666667	114.3611111
HIST2H4B	148	6.12E-04	0.4749558	28.1	138.0540541
FYN	123	0.00288179	0.45864947	15.51515152	75.90082645
CCAR2	56	2.28E-04	0.45374367	11.86666667	159.1607143
FUS	177	0.0015512	0.48148148	37.22068966	128.0571429
BMI1	140	0.00144262	0.46634523	21.51724138	97.81884058
CCT3	103	4.50E-04	0.47291218	24.68604651	143.6796117
PTEN	129	0.00209173	0.46978823	18.65909091	97.14173228
TRAF3	58	4.91E-04	0.42473213	9.828571429	82.875
TRAF2	137	0.00294509	0.46742702	21.28395062	86.67407407
EZH2	145	0.00139102	0.46259805	29.98148148	113.1398601
PTBP1	69	1.70E-04	0.45323336	24.8	169.0597015
TRAF6	250	0.00916431	0.49722394	25.0472973	79.68951613
TRAF1	90	9.65E-04	0.44261395	15.46153846	84.05681818
PSMD13	56	8.25E-05	0.42525501	25.8	113.25
HSP90B1	89	0.00136589	0.46760781	18.59677419	143.137931
EWSR1	205	0.00589464	0.49046653	25.42537313	100.4187192
PSMD12	61	9.76E-05	0.41797753	29.24	108.5932203
TPM3	64	3.33E-04	0.45708885	12.73913043	147.6935484
PSMD11	78	4.38E-04	0.46661521	23.43283582	131.2435897
PSMD4	106	9.60E-04	0.47198907	27.08139535	131.6538462
TP73	103	0.00130657	0.46153846	16.83333333	97.88118812
TP53	948	0.14315688	0.6038961	51.75641026	58.95560254
TP53BP2	52	3.56E-04	0.45170932	10.22222222	127.16
EHMT2	54	2.45E-04	0.43348871	17.18918919	134.5769231
PSMD7	55	1.52E-04	0.42235808	26.18181818	101.9272727
FKBP8	53	2.88E-04	0.42125436	19.62790698	104.3921569
PSMD1	82	3.62E-04	0.4528938	27.39393939	129.4512195
TP53BP1	102	7.28E-04	0.46697567	23.81481481	136.4411765
BCR	60	0.00107214	0.45873648	14.85365854	134.7586207
ETS1	56	3.78E-04	0.45357344	14.08333333	123.8518519
TOP2A	77	2.42E-04	0.46670527	19.34328358	174.1733333
PSMC6	69	1.44E-04	0.43812285	28.76363636	123.4925373
PSMD3	66	2.82E-04	0.43001956	27.01886792	113.6212121
PSMD2	113	0.00113376	0.45778114	25.78313253	108.5225225
ESR2	80	0.00104471	0.4400364	12.57894737	83.67948718
PSMC3	86	7.34E-04	0.46162658	25.31818182	115.4761905
PSMC5	105	9.09E-04	0.47328244	28.04819277	129.5436893
PSMC4	60	8.22E-05	0.43017257	27.51851852	130.4833333
ESR1	417	0.0162846	0.52817824	41.77906977	88.49156627
TOP1	89	9.05E-04	0.46851385	23.94202899	162.045977
PSMC2	82	2.26E-04	0.447612	29.35211268	121.25

FBL	96	7.24E-04	0.46101049	29.22222222	140.96875
PSMC1	75	5.21E-04	0.4459609	26.85964912	113.04
BCL6	57	4.83E-04	0.44935886	13.17647059	121.8909091
TNFRSF1B	72	4.90E-04	0.42940863	13.05882353	95.67142857
ERCC6	57	1.14E-04	0.43411131	19.92	168.1578947
PSMB3	59	1.32E-04	0.44043716	26.11538462	122.9122807
APP	429	0.04197469	0.5313118	26.86413043	64.18735363
TNFRSF1A	134	0.00208911	0.46410749	18.43023256	94.81060606
PSMB4	58	2.31E-04	0.43193998	23.3125	104.6724138
TNF	113	0.01260408	0.42713302	11	39.97297297
PSMA6	92	4.60E-04	0.45170932	26.22666667	115.173913
PSMA5	67	2.17E-04	0.44571429	27.74074074	127.358209
EPS15	76	4.62E-04	0.45053102	14.26666667	129.7972973
PSMA7	76	2.87E-04	0.45425512	27.56716418	133.5405405
ERBB3	83	6.74E-04	0.43497032	15.67924528	94.13580247
PSMA2	79	1.87E-04	0.44293827	28.81428571	121.025974
LOC101929876	84	8.70E-05	0.45103525	49.64102564	155.5238095
PSMA1	81	4.66E-04	0.44711538	26.390625	106.2151899
ERBB2	112	0.00214212	0.4541698	16.07142857	79.08181818
PSMA4	61	1.25E-04	0.42895157	27.68518519	116.7118644
PSMA3	134	0.00125946	0.46357362	29.29	103.3333333
XIAP	75	9.56E-04	0.44059767	12.23809524	85.35616438
APEX1	59	2.59E-04	0.45682978	12.5106383	142.3333333
BIRC2	60	4.41E-04	0.42683142	12.48571429	85.22413793
APC	99	0.00226742	0.45120358	9.962264151	74.73195876
EP300	311	0.00957961	0.51315789	40.94117647	96.65372168
EPB41	60	5.53E-04	0.43915728	7.428571429	97.41666667
TLE1	73	7.47E-04	0.41653747	10.92857143	85.46478873
ENO1	82	0.00109306	0.45987067	17.53968254	140.4146342
TK1	56	5.46E-04	0.44678492	8.483870968	102.5
ANXA7	59	6.08E-04	0.44661987	5.162162162	95.79661017
EIF1B	72	3.43E-04	0.41812208	7.47826087	76.65277778
ANXA2	85	6.24E-04	0.47635934	16.52112676	156.6588235
CTTN	55	2.40E-04	0.44269498	12.76744186	140.5849057
SLC25A5	81	5.63E-04	0.4658062	28.21518987	169.2962963
STUB1	141	0.00195218	0.48779504	24.23529412	110.1294964
EMD	78	5.37E-04	0.45357344	17.33333333	140.1973684
TGM2	58	7.70E-04	0.46065917	12.17948718	132.9107143
TGFBR1	76	0.00113459	0.4410799	11.89130435	85.31081081
EIF2AK2	57	3.95E-04	0.45442586	11.15789474	130.0909091
TGFB1	97	0.00944659	0.42540464	4.666666667	37.43157895
MAPK9	62	6.00E-04	0.45726172	10.38709677	102.5666667
MAP2K1	80	0.00104733	0.45179372	12.22727273	96.6025641
MAPK8	129	0.00304246	0.49016825	18.14102564	98.59055118

MAPK1	326	0.02351273	0.51556503	25.55970149	67.29320988
EIF4B	59	1.52E-04	0.43520518	12.27083333	151.3859649
MAPK3	190	0.0065103	0.49236408	19.08988764	77.64361702
PRKCZ	80	7.51E-04	0.45622642	14.68627451	110.7307692
HNRNPR	94	4.59E-04	0.46330715	26.35365854	151.1276596
PRKDC	163	0.00262417	0.49457967	28.20833333	135.8322981
EGFR	361	0.01999686	0.52112069	32.93617021	80.80501393
UBC	396	0.02028958	0.52656794	38.1622807	83.97969543
PRKCA	139	0.00505572	0.48389033	17.33802817	97.2189781
PRKCD	98	0.00164327	0.47617172	17.71186441	108.5
PRKCB	71	0.00107386	0.44645495	14.45652174	121.6086957
PSMD14	60	1.68E-04	0.42895157	25.52	100.8166667
ALDOA	52	2.85E-04	0.44180523	16.32432432	149.84
PRKACA	113	0.00316598	0.46392939	11.15384615	81.42477876
TERF2	105	0.00111008	0.44075829	12.85714286	79.99029126
TERF1	135	0.00218926	0.45969582	13.40506329	77.88721805
TERT	53	2.13E-04	0.43434525	10.82926829	119.7647059
PRKAA1	55	3.44E-04	0.43733044	9.03030303	112.4363636
AKT1	340	0.02822641	0.51207116	24.58041958	64.83136095
PSME3	70	6.01E-04	0.45120358	17.64	115.0441177
AKT2	58	7.69E-04	0.43669857	13.5625	120.1964286
ALYREF	53	1.20E-04	0.45995815	19.08888889	198.5098039
DNMT1	68	1.74E-04	0.46022078	24.0862069	162.8030303
SMURF1	196	0.00364753	0.48769665	21.83464567	90.95360825
RPL13A	77	2.45E-04	0.42910382	48.95833333	141.6103896
TCP1	82	2.64E-04	0.46778874	24.74242424	163.3658537
WWOX	84	7.68E-04	0.45357344	11.77966102	104.6309524
TRIM28	134	0.00125905	0.48186528	27.63551402	147.1666667
HNF1A	53	0.00216084	0.41610738	8.551724138	82.88235294
TCF3	64	5.55E-04	0.42857143	8.595238095	93.24193548
PPIA	59	4.60E-04	0.4522162	14.90909091	156.6666667
DDX24	57	2.44E-04	0.43147752	16.34146341	130.9636364
TCF4	60	7.92E-04	0.44860853	10.90322581	102
ADRB2	113	0.00203774	0.45069897	13.96875	82.71171171
MED1	60	4.57E-04	0.44604317	12.11428571	111.8275862
ELOC	53	4.32E-04	0.41073552	7.696969697	89.98113208
PPARG	155	0.00630501	0.4749558	18.94594595	79.30718954
TBP	84	5.91E-04	0.44744634	16.38297872	100.3780488
G3BP1	59	1.98E-04	0.46206765	14.84444444	167.5614035
SIN3A	96	7.25E-04	0.45605432	17.62295082	101.7083333
MAP3K7	88	0.00109522	0.45213164	16.2037037	94.60465116
POT1	72	6.94E-04	0.42600423	10.15217391	83
PARP1	172	0.00286412	0.49016825	34.26153846	130.5823529
TAF9	60	4.26E-04	0.43575419	11.85714286	113.7166667

TADA2A	53	2.35E-04	0.43955644	9.162162162	116.1886793
TAF1	63	2.63E-04	0.43372197	17.92	143.3770492
SYK	79	0.00103387	0.44612546	15.39622642	95.38961039
RAD50	71	3.38E-04	0.43772629	16.49090909	143.0985916
POLR2A	121	0.00138947	0.46215596	19.1375	111.2689076
DDX3X	85	2.90E-04	0.46951456	18.52	166.7529412
DDX1	71	2.65E-04	0.45864947	21.4	169.6619718
DDX5	124	8.78E-04	0.48331001	27.96116505	152.0245902
ABL1	151	0.00280367	0.49246436	23.21	113.4161074
GADD45A	66	5.25E-04	0.44819277	11.5106383	123.671875
DCTN1	66	6.49E-04	0.44059767	8.4375	90.140625
RANBP9	64	0.00134155	0.46742702	11.71111111	130.7419355
DDB1	140	0.00152952	0.47664104	22.61458333	127.4492754
SRC	206	0.00818834	0.50532915	24.24137931	88.31372549
PML	162	0.00285288	0.48799193	22.04672897	103.49375
SPTAN1	95	0.00101331	0.45838863	18.83333333	139.1894737
PIAS4	66	4.22E-04	0.44505798	11.59090909	101.8484849
DBN1	93	5.68E-04	0.46401842	19.0625	126.989011
DAXX	114	0.00118936	0.46833236	23.64367816	123.7767857
PDCD6IP	55	9.31E-04	0.43931686	8.1875	110.2830189
SIRT6	68	2.11E-04	0.43512687	8.132075472	104.4264706
CCT2	97	3.82E-04	0.47411765	28.6097561	162.0412371
THRB	53	5.42E-04	0.43528353	11.90322581	100.8627451
THRA	54	1.86E-04	0.42088773	12.48571429	104.5384615
SIRT7	261	0.00468892	0.49116392	31.5952381	90.37547893
PLK1	120	0.00255917	0.47739388	15.76056338	103.6440678
PLEC	56	1.86E-04	0.44497608	13.08333333	156.0535714
CCT4	81	2.27E-04	0.46778874	23.62318841	161.8271605
SP1	155	0.00292009	0.48427799	25.83495146	104.7581699
CCT7	79	3.44E-04	0.47171284	21.21666667	150.4556962
PLCG1	84	0.00103489	0.44711538	14.05128205	85.03571429
SOD1	64	7.00E-04	0.41957314	9.735294118	71.83870968
ANP32B	63	2.98E-04	0.43859967	13.84	149.6190476
SNRPD3	60	1.49E-04	0.44253294	17.53846154	174.9
TUBA1C	98	5.44E-04	0.46616541	24.25675676	137.7959184
LIMA1	90	7.08E-04	0.46760781	21.36923077	142.6111111
KAT5	139	0.00185767	0.4765471	30.17525773	125.9562044
SNRPA1	65	3.06E-04	0.42592919	16.92	132.5076923
SNCA	88	9.43E-04	0.46242111	17.66101695	110.5
PIK3R2	66	4.51E-04	0.44819277	15.73333333	122.2121212
PIN1	121	0.00195206	0.47467609	13.39506173	96.8677686
DDX17	90	4.02E-04	0.47598425	24.7721519	173.9772727
SIRT1	153	0.00205605	0.47551622	26.75700935	113.1456954
MYBBP1A	61	1.65E-04	0.45717527	22.55555556	183.4918033

PIK3R1	118	0.00244307	0.46634523	19.0952381	91.89655172
SUMO2	114	0.00118986	0.46206765	16.67605634	102.4464286
NCOA2	65	2.29E-04	0.43489209	14.88679245	123.7777778
SMN1	103	7.79E-04	0.45812808	19.74666667	115.8415842
SMN2	103	7.79E-04	0.45812808	19.74666667	115.8415842
CARM1	62	2.41E-04	0.44711538	16.78181818	162.9516129
SMARCD1	63	6.49E-04	0.44563214	16.67391304	133.3492064
SMARCC2	86	4.07E-04	0.44952593	18.71641791	133.2093023
SMARCA4	155	0.00188368	0.47514246	33.06481481	121.0784314
EEF2	100	6.49E-04	0.47291218	31.49382716	149.6836735
SMARCC1	87	3.38E-04	0.46733668	22.30882353	144.4
SMARCB1	69	5.15E-04	0.46562681	15.13953488	123.8059702
EEF1G	72	0.0010684	0.45553881	15.88461538	153.6857143
EEF1A1	222	0.00537699	0.49478207	39.59627329	114.1772727
NTRK1	640	0.04345902	0.55496902	44.13450292	71.95297806
OBSL1	257	0.0026635	0.4899696	32.14361702	97.68235294
HECW2	73	9.19E-04	0.42933239	5.4	59.92957746
RPTOR	55	4.60E-04	0.43725136	10.03030303	106.6981132
TAB1	66	7.63E-04	0.44769487	17.51111111	123.421875
HIST4H4	148	6.12E-04	0.4749558	28.1	138.0540541
YBX1	106	7.61E-04	0.48253842	25.4494382	155.8269231
ECT2	62	4.78E-04	0.45752129	6.317073171	106
E2F1	84	5.86E-04	0.46013321	15.66666667	118.75
LRRK2	140	0.00155013	0.45708885	20.94949495	100.1811594
PRMT5	104	6.07E-04	0.46268657	25.77380952	141.4215686
DVL2	62	6.63E-04	0.45272421	9.333333333	104.7833333
YAP1	55	2.83E-04	0.43931686	10.06976744	118.9090909
NPM1	289	0.00483412	0.51261395	56.26222222	124.1358885
RACK1	160	0.00323208	0.47805457	32.51639344	128.2848101
NOTCH1	91	7.61E-04	0.46215596	13.95384615	104.7078652
TERF2IP	64	5.24E-04	0.42162162	10	99.79032258
DSP	70	4.04E-04	0.44043716	14.38	153.2941177
NOS2	154	0.0036062	0.4465374	21.52475248	81.17105263
FBXW11	133	0.00229053	0.47786561	17.06756757	91.70992366
SKP2	77	5.06E-04	0.44629014	15.42592593	115
NME2	54	2.79E-04	0.42257952	11.72222222	96.42307692
TUBB4B	78	3.77E-04	0.46960575	20.50724638	178.0384615
SKI	81	3.56E-04	0.45847554	15.20967742	121.6708861
SKP1	95	9.66E-04	0.46598574	18.41666667	123.7956989
DCUN1D1	108	5.45E-04	0.45519578	26.1547619	118.6388889
SRSF7	57	1.58E-04	0.43497032	16.34693878	168.8070175
CCT6A	81	3.81E-04	0.46437488	23.38461538	150.1975309
SRSF1	75	3.66E-04	0.46796981	22.78333333	172.630137
SFPQ	100	5.93E-04	0.46670527	20.69512195	138.3163265

NFKBIB	58	2.25E-04	0.44027677	13.97777778	123.9285714
NFKB1	145	0.00165219	0.46851385	24.35294118	105.5174825
NFKBIA	104	0.00105292	0.4765471	18.56097561	120.8137255
CCNA2	68	7.45E-04	0.44819277	14.24390244	108.4848485
SET	101	8.46E-04	0.47254251	22.41772152	146.8484849
HDAC5	219	0.00215034	0.48427799	34.29943503	113.9493088
HDAC6	143	0.00178344	0.47626551	23.06306306	122.5248227
NFATC1	73	9.75E-04	0.4511194	11.9245283	113.1917808
NF2	90	7.86E-04	0.43915728	11.68852459	90.47727273
CAV1	110	0.00270517	0.47208122	15.70967742	97.2037037
CASP7	72	6.62E-04	0.42986667	9.56097561	73.95714286
NEDD8	142	0.00105905	0.47061113	32.09565217	118.2428571
CASP9	54	0.0013582	0.42986667	9.64	80.59615385
RPL10A	93	9.74E-05	0.45987067	55.75862069	166.6236559
STK11	68	0.00118489	0.45631251	10.34210526	96.16666667
CASP8	133	0.00515765	0.48119403	14.8245614	76.84732824
RBX1	97	8.97E-04	0.46259805	17.42857143	116.7263158
CASP3	181	0.00914384	0.47938144	12.94736842	63.2122905
NEDD4	114	0.00189054	0.46544755	13.37878788	87.88392857
AURKA	170	0.0054058	0.4886823	23.91919192	97.23214286
THRAP3	66	1.81E-04	0.45365854	15.78947368	162.0757576
TAB2	78	4.50E-04	0.46022078	19.078125	133.9210526
PAN2	204	0.00185495	0.47015361	28.99350649	95.87745098
STAT3	134	0.00277337	0.47356052	20.76470588	97.99242424
SEC16A	55	3.14E-04	0.43725136	10.31818182	132
STAT5A	53	2.87E-04	0.4359113	13	113.3529412
STAT1	101	0.00166953	0.46778874	15.04225352	107.5959596
CANX	68	8.07E-04	0.44116037	12.63829787	122.6176471
PPP2R1A	115	0.00195636	0.48196133	19.02564103	128.1391304
TUBB	155	0.00210727	0.49610176	30.8503937	141.6258065
PPP2CA	133	0.00261554	0.48809043	18.5	111.2255639
PPP1CC	113	0.00184189	0.48789346	19.67567568	121.2566372
PPP1CB	85	6.39E-04	0.45717527	13.9245283	104.9411765
PPP1CA	150	0.00325917	0.49316745	22.93939394	117.5878378
UHRF2	52	1.47E-04	0.43756786	15.09090909	146.46
SETDB1	91	9.82E-04	0.43875885	17.93220339	108.6043956
CAD	88	2.83E-04	0.47198907	22.26829268	149.9302326
PSMD6	57	1.44E-04	0.4398763	25.93877551	118.1929825
MSH2	81	8.58E-04	0.46526842	14.80357143	119.9873418
TRIM21	58	3.54E-04	0.43418926	13.07692308	141.0178571
SRPK1	77	9.00E-04	0.46544755	13.80851064	116.4666667
NCOA6	64	3.20E-04	0.44977679	18.64	127.0322581
TNIK	52	2.51E-04	0.41913677	6.96875	82.56
CUL7	295	0.00373698	0.50134771	35.2629108	96.02389078

MRE11	82	3.37E-04	0.44261395	22.12903226	133.7375
KDM1A	114	0.00244084	0.46321839	22.49315068	115.5446429
HDAC4	95	8.42E-04	0.45196262	19.52173913	115.6129032
BCL2	120	0.00683984	0.47070274	12.76	76.00847458
CCND1	113	0.00339484	0.47254251	17.68253968	103.5045045
BCL2L1	76	0.00200585	0.4489417	8.346153846	64.13513514
PAXIP1	105	0.00171216	0.46162658	15.16666667	98.73333333
DCAF1	59	1.80E-04	0.44530387	16.77083333	162.245614
BAX	59	7.94E-04	0.45061498	9.6	99.50877193
BARD1	112	0.00108316	0.46455331	20.68055556	104.5545455
KMT2A	82	3.08E-04	0.43685637	26.95454545	133.4625
CCT5	73	2.21E-04	0.46455331	22.04918033	167.1780822

Results for Topology Analysis of Network Structure - Final Network

gene_symbol	ensembl_id	ENTREZ_id	gene_name_full
RPL23	ENSG00000125691	9349	ribosomal protein L23(RPL23)
DDB1	ENSG00000167986	1642	damage specific DNA binding protein 1(DDB1)
XRCC6	ENSG00000196419	2547	X-ray repair cross complementing 6(XRCC6)
PRKDC	ENSG00000253729	5591	protein kinase, DNA-activated, catalytic subunit(PRKDC)
HIF1A	ENSG00000100644	3091	hypoxia inducible factor 1 subunit alpha(HIF1A)
SET	ENSG00000119335	6418	SET nuclear proto-oncogene(SET)
DAXX	ENSG00000204209	1616	death domain associated protein(DAXX)
HDAC6	ENSG00000094631	10013	histone deacetylase 6(HDAC6)
FUS	ENSG00000089280	2521	FUS RNA binding protein(FUS)
MAP3K1	ENSG00000095015	4214	mitogen-activated protein kinase kinase kinase 1(MAP3K1)
CHUK	ENSG00000213341	1147	component of inhibitor of nuclear factor kappa B kinase complex(CHUK)
ACTB	ENSG00000075624	60	actin beta(ACTB)
IQGAP1	ENSG00000140575	8826	IQ motif containing GTPase activating protein 1(IQGAP1)
NEDD8	ENSG00000129559	4738	NEDD8 ubiquitin like modifier(NEDD8)
TBK1	ENSG00000183735	29110	TANK binding kinase 1(TBK1)
H2AFX	ENSG00000188486	3014	H2A.X variant histone(H2AX)
CUL4A	ENSG00000139842	8451	cullin 4A(CUL4A)
CUL4B	ENSG00000158290	8450	cullin 4B(CUL4B)
TUBG1	ENSG00000131462	7283	tubulin gamma 1(TUBG1)
PSMD4	ENSG00000159352	5710	proteasome 26S subunit ubiquitin receptor,

			non-ATPase 4(PSMD4)
RBBP4	ENSG00000162521	5928	RB binding protein 4, chromatin remodeling factor(RBBP4)
LMNA	ENSG00000160789	4000	lamin A/C(LMNA)
RPL31	ENSG00000071082	6160	ribosomal protein L31(RPL31)
TP53BP1	ENSG00000067369	7158	tumor protein p53 binding protein 1(TP53BP1)
AURKB	ENSG00000178999	9212	aurora kinase B(AURKB)
KAT5	ENSG00000172977	10524	lysine acetyltransferase 5(KAT5)
PSMC5	ENSG00000087191	5705	proteasome 26S subunit, ATPase 5(PSMC5)
TOP1	ENSG00000198900	7150	DNA topoisomerase I(TOP1)
TUBB	ENSG00000196230	203068	tubulin beta class I(TUBB)
FBL	ENSG00000105202	2091	fibrillarin(FBL)
NCL	ENSG00000115053	4691	nucleolin(NCL)
WDR5	ENSG00000196363	11091	WD repeat domain 5(WDR5)
PPP1CC	ENSG00000186298	5501	protein phosphatase 1 catalytic subunit gamma(PPP1CC)
PPP1CA	ENSG00000172531	5499	protein phosphatase 1 catalytic subunit alpha(PPP1CA)
SMARCA4	ENSG00000127616	6597	SWI/SNF related, matrix associated, actin dependent regulator of chromatin, subfamily a, member 4(SMARCA4)
GAPDH	ENSG00000111640	2597	glyceraldehyde-3-phosphate dehydrogenase(GAPDH)
XRCC5	ENSG00000079246	7520	X-ray repair cross complementing 5(XRCC5)
TRIM28	ENSG00000130726	10155	tripartite motif containing 28(TRIM28)
HSPD1	ENSG00000144381	3329	heat shock protein family D (Hsp60) member 1(HSPD1)
HNRNPK	ENSG00000165119	3190	heterogeneous nuclear ribonucleoprotein K(HNRNPK)
HNRNPD	ENSG00000138668	3184	heterogeneous nuclear ribonucleoprotein D(HNRNPD)
FLNA	ENSG00000196924	2316	filamin A(FLNA)
CLTC	ENSG00000141367	1213	clathrin heavy chain(CLTC)
HSPA9	ENSG00000113013	3313	heat shock protein family A (Hsp70) member 9(HSPA9)
HSPA8	ENSG00000109971	3312	heat shock protein family A (Hsp70) member 8(HSPA8)
HSPB1	ENSG00000106211	3315	heat shock protein family B (small) member 1(HSPB1)
YBX1	ENSG00000065978	4904	Y-box binding protein 1(YBX1)
RPS2	ENSG00000140988	6187	ribosomal protein S2(RPS2)
HSPA5	ENSG00000044574	3309	heat shock protein family A (Hsp70) member 5(HSPA5)
HSPA4	ENSG00000170606	3308	heat shock protein family A (Hsp70) member

			4(HSPA4)
HSPA1B	ENSG00000204388	3304	heat shock protein family A (Hsp70) member 1B(HSPA1B)
HSPA1A	ENSG00000204389	3303	heat shock protein family A (Hsp70) member 1A(HSPA1A)
PARP1	ENSG00000143799	142	poly(ADP-ribose) polymerase 1(PARP1)
C1QBP	ENSG00000108561	708	complement C1q binding protein(C1QBP)
MCM7	ENSG00000166508	4176	minichromosome maintenance complex component 7(MCM7)
NPM1	ENSG00000181163	4869	nucleophosmin 1(NPM1)
PRMT1	ENSG00000126457	3276	protein arginine methyltransferase 1(PRMT1)
RACK1	ENSG00000204628	10399	receptor for activated C kinase 1(RACK1)
RUVBL2	ENSG00000183207	10856	RuvB like AAA ATPase 2(RUVBL2)
DDX5	ENSG00000108654	1655	DEAD-box helicase 5(DDX5)

Supplementary Table S8: Data of enrichment analysis

GO for Common Targets

ONTOLOGY	ID	Description	GeneRatio	BgRatio	pvalue	p.adjust	qvalue	geneID	Count
BP	GO:0032496	response to lipopolysaccharide	13/41	343/18723	2.32E-13	6.99E-10	2.41E-10	TNF/PTGS2/CASP3/IL1B/IL6/NOS2/AKT1/TGFB1/TLR4/CYP1A2/MAPK1/CASP9/RPS6KA3	13
BP	GO:0002237	response to molecule of bacterial origin	13/41	363/18723	4.77E-13	1.44E-09	2.47E-10	TNF/PTGS2/CASP3/IL1B/IL6/NOS2/AKT1/TGFB1/TLR4/CYP1A2/MAPK1/CASP9/RPS6KA3	13
BP	GO:0071887	leukocyte apoptotic process	9/41	106/18723	1.27E-12	3.84E-09	4.41E-10	CASP3/IL6/AKT1/IDO1/CDKN2A/TP53/CASP9/IL2/PIK3CD	9
BP	GO:1901214	regulation of neuron death	11/41	319/18723	5.93E-11	1.79E-07	1.54E-08	TNF/CASP3/AKT1/GRIN2B/BCL2/TP53/HMOX1/TLR4/CASP9/ADORA1/GABRB2	11
BP	GO:0072593	reactive oxygen species metabolic process	10/41	239/18723	7.54E-11	2.27E-07	1.57E-08	TNF/NOS2/AKT1/GRIN1/XDH/BCL2/TP53/TGFB1/TLR4/CYP1A2	10
BP	GO:0048732	gland development	12/41	436/18723	9.38E-11	2.83E-07	1.62E-08	TNF/IL6/AKT1/XDH/BCL2/HNF4A/EGF/TGFB1/HMOX1/CCND1/HNF1A/MAPK1	12
BP	GO:0009306	protein secretion	11/41	359/18723	2.09E-10	6.29E-07	2.19E-08	TNF/IL1B/IL6/NOS2/ADRA2A/ACHE/HNF4A/FOXA2/TGFB1/TLR4/HNF1A	11
BP	GO:0071900	regulation of protein serine/threonine kinase activity	11/41	359/18723	2.09E-10	6.29E-07	2.19E-08	TNF/CASP3/IL1B/AKT1/ADRA2A/CDKN2A/PPARG/EGF/TGFB1/CCND1/TLR4	11
BP	GO:0035592	establishment of protein localization to extracellular region	11/41	360/18723	2.15E-10	6.48E-07	2.19E-08	TNF/IL1B/IL6/NOS2/ADRA2A/ACHE/HNF4A/FOXA2/TGFB1/TLR4/HNF1A	11

BP	GO:0070997	neuron death	11/41	361/18723	2.22E-10	6.68E-07	2.19E-08	TNF/CASP3/AKT1/GRIN2B/BCL2/TP53/HMOX1/TLR4/CASP9/ADORA1/GABRB2	11
BP	GO:0050708	regulation of protein secretion	10/41	268/18723	2.32E-10	6.98E-07	2.19E-08	TNF/IL1B/IL6/NOS2/ADRA2A/ACHE/HNF4A/FOXA2/TGFB1/TLR4	10
BP	GO:0071692	protein localization to extracellular region	11/41	368/18723	2.72E-10	8.19E-07	2.35E-08	TNF/IL1B/IL6/NOS2/ADRA2A/ACHE/HNF4A/FOXA2/TGFB1/TLR4/HNF1A	11
BP	GO:0060965	negative regulation of gene silencing by miRNA	5/41	22/18723	1.00E-09	3.02E-06	8.00E-08	TNF/IL6/PPARG/TP53/TGFB1	5
BP	GO:2000377	regulation of reactive oxygen species metabolic process	8/41	157/18723	1.54E-09	4.65E-06	9.08E-08	TNF/AKT1/GRIN1/XDH/BCL2/TP53/TGFB1/TLR4	8
BP	GO:1901216	positive regulation of neuron death	7/41	97/18723	1.57E-09	4.72E-06	9.08E-08	TNF/CASP3/GRIN2B/TP53/TLR4/CASP9/ADORA1	7
BP	GO:0060149	negative regulation of posttranscriptional gene silencing	5/41	24/18723	1.61E-09	4.86E-06	9.08E-08	TNF/IL6/PPARG/TP53/TGFB1	5
BP	GO:0060967	negative regulation of gene silencing by RNA	5/41	24/18723	1.61E-09	4.86E-06	9.08E-08	TNF/IL6/PPARG/TP53/TGFB1	5
BP	GO:2000116	regulation of cysteine-type endopeptidase	9/41	235/18723	1.64E-09	4.94E-06	9.08E-08	TNF/PTGS2/AKT1/GRIN2B/GRIN1/XDH/PPARG/CASP9/RPS6KA3	9

		activity							
BP	GO:0050673	epithelial cell proliferation	11/41	437/18723	1.66E-09	5.01E-06	9.08E-08	TNF/IL6/AKT1/XDH/PPARG/EGF/TGFB1/HMOX1/CCND1/MAPK1/PIK3CD	11
BP	GO:0045936	negative regulation of phosphate metabolic process	11/41	441/18723	1.83E-09	5.51E-06	9.23E-08	TNF/CASP3/IL1B/AKT1/XDH/CDKN2A/PPARG/TP53/FOXA2/TGFB1/IL2	11
BP	GO:0010563	negative regulation of phosphorus metabolic process	11/41	442/18723	1.87E-09	5.64E-06	9.23E-08	TNF/CASP3/IL1B/AKT1/XDH/CDKN2A/PPARG/TP53/FOXA2/TGFB1/IL2	11
BP	GO:0050995	negative regulation of lipid catabolic process	5/41	25/18723	2.01E-09	6.06E-06	9.23E-08	TNF/IL1B/AKT1/ADRA2A/ADORA1	5
BP	GO:0002790	peptide secretion	9/41	242/18723	2.12E-09	6.39E-06	9.23E-08	TNF/IL1B/IL6/NOS2/ADRA2A/HNF4A/FOXA2/HNF1A/ADORA1	9
BP	GO:0062197	cellular response to chemical stress	10/41	337/18723	2.13E-09	6.43E-06	9.23E-08	PTGS2/CASP3/IL6/AKT1/BCL2/TP53/HMOX1/TLR4/MAPK1/ATM	10
BP	GO:0071216	cellular response to biotic stimulus	9/41	246/18723	2.45E-09	7.38E-06	1.01E-07	TNF/IL1B/IL6/NOS2/AKT1/TP53/TGFB1/TLR4/MAPK1	9
BP	GO:0048661	positive regulation of smooth muscle cell proliferation	7/41	104/18723	2.56E-09	7.72E-06	1.01E-07	TNF/PTGS2/IL6/AKT1/TGFB1/HMOX1/TLR4	7
BP	GO:0033002	muscle cell proliferation	9/41	248/18723	2.63E-09	7.92E-06	1.01E-07	TNF/PTGS2/IL6/AKT1/PPARG/TGFB1/HMOX1/TLR4/MAPK1	9
BP	GO:0001819	positive regulation of cytokine production	11/41	467/18723	3.33E-09	1.00E-05	1.19E-07	TNF/PTGS2/IL1B/IL6/NOS2/ADRA2A/TGFB1/HMOX1/TLR4/IL2/PIK3CD	11

BP	GO:0033674	positive regulation of kinase activity	11/41	467/18723	3.33E-09	1.00E-05	1.19E-07	TNF/IL1B/AKT1/ADRA2A/EGF/TGFB1/CCND1/TLR4/IL2/ADRA2C/ADORA1	11
BP	GO:0031663	lipopolysaccharide-mediated signaling pathway	6/41	60/18723	3.45E-09	1.04E-05	1.19E-07	TNF/IL1B/AKT1/TGFB1/TLR4/MAPK1	6
BP	GO:0060969	negative regulation of gene silencing	5/41	29/18723	4.47E-09	1.35E-05	1.43E-07	TNF/IL6/PPARG/TP53/TGFB1	5
BP	GO:0015833	peptide transport	9/41	264/18723	4.54E-09	1.37E-05	1.43E-07	TNF/IL1B/IL6/NOS2/ADRA2A/HNF4A/FOXA2/HNF1A/ADORA1	9
BP	GO:0048660	regulation of smooth muscle cell proliferation	8/41	180/18723	4.55E-09	1.37E-05	1.43E-07	TNF/PTGS2/IL6/AKT1/PPARG/TGFB1/HMOX1/TLR4	8
BP	GO:0048659	smooth muscle cell proliferation	8/41	184/18723	5.41E-09	1.63E-05	1.63E-07	TNF/PTGS2/IL6/AKT1/PPARG/TGFB1/HMOX1/TLR4	8
BP	GO:0045862	positive regulation of proteolysis	10/41	372/18723	5.51E-09	1.66E-05	1.63E-07	TNF/IL1B/AKT1/ADRA2A/GRIN2B/GRIN1/XDH/PPARG/EGF/CASP9	10
BP	GO:0045860	positive regulation of protein kinase activity	10/41	386/18723	7.83E-09	2.36E-05	2.21E-07	TNF/IL1B/AKT1/ADRA2A/EGF/TGFB1/CCND1/TLR4/ADRA2C/ADORA1	10
BP	GO:0050731	positive regulation of peptidyl-tyrosine phosphorylation	8/41	193/18723	7.87E-09	2.37E-05	2.21E-07	TNF/IL6/ADRA2A/TP53/EGF/TGFB1/IL2/ADORA1	8
BP	GO:0030073	insulin secretion	8/41	195/18723	8.54E-09	2.57E-05	2.33E-07	TNF/IL1B/IL6/NOS2/ADRA2A/HNF4A/FOXA2/HNF1A	8
BP	GO:0097193	intrinsic apoptotic signaling pathway	9/41	288/18723	9.69E-09	2.92E-05	2.58E-07	TNF/PTGS2/CASP3/AKT1/BCL2/TP53/HMOX1/CASP9/ATM	9

BP	GO:0002791	regulation of peptide secretion	8/41	200/18723	1.04E-08	3.14E-05	2.63E-07	TNF/IL1B/IL6/NOS2/ADRA2A/HNF4A/FOXA2/ADORA1	8
BP	GO:0071902	positive regulation of protein serine/threonine kinase activity	8/41	200/18723	1.04E-08	3.14E-05	2.63E-07	TNF/IL1B/AKT1/ADRA2A/EGF/TGFB1/CCND1/TLR4	8
BP	GO:0090087	regulation of peptide transport	8/41	202/18723	1.13E-08	3.39E-05	2.78E-07	TNF/IL1B/IL6/NOS2/ADRA2A/HNF4A/FOXA2/ADORA1	8
BP	GO:0046879	hormone secretion	9/41	295/18723	1.19E-08	3.60E-05	2.88E-07	TNF/IL1B/IL6/NOS2/ADRA2A/HNF4A/FOXA2/HNF1A/ADORA1	9
BP	GO:0001503	ossification	10/41	408/18723	1.33E-08	4.00E-05	3.13E-07	TNF/PTGS2/IL6/AKT1/ACHE/BCL2/COL1A2/PPARG/TGFB1/MAPK1	10
BP	GO:0042886	amide transport	9/41	301/18723	1.42E-08	4.28E-05	3.25E-07	TNF/IL1B/IL6/NOS2/ADRA2A/HNF4A/FOXA2/HNF1A/ADORA1	9
BP	GO:0071222	cellular response to lipopolysaccharide	8/41	209/18723	1.47E-08	4.43E-05	3.25E-07	TNF/IL1B/IL6/NOS2/AKT1/TGFB1/TLR4/MAPK1	8
BP	GO:0006809	nitric oxide biosynthetic process	6/41	76/18723	1.47E-08	4.43E-05	3.25E-07	TNF/PTGS2/IL1B/NOS2/AKT1/TLR4	6
BP	GO:0009914	hormone transport	9/41	306/18723	1.64E-08	4.94E-05	3.54E-07	TNF/IL1B/IL6/NOS2/ADRA2A/HNF4A/FOXA2/HNF1A/ADORA1	9
BP	GO:0060078	regulation of postsynaptic membrane potential	7/41	137/18723	1.76E-08	5.30E-05	3.72E-07	AKT1/GRIN2B/GRIN1/ADORA1/GABRG2/GABRB2/GABRA1	7
BP	GO:00	wound healing	10/41	422/18723	1.83E-08	5.51E-05	3.79E-07	TNF/CASP3/IL6/ADRA2A/HNF4A/FOXA2/TGFB1/HM	10

	42060							OX1/TLR4/ADRA2C	
BP	GO:0018105	peptidyl-serine phosphorylation	9/41	315/18723	2.10E-08	6.34E-05	4.21E-07	TNF/PTGS2/IL6/AKT1/BCL2/TGFB1/MAPK1/RPS6KA3/ATM	9
BP	GO:0046209	nitric oxide metabolic process	6/41	81/18723	2.16E-08	6.52E-05	4.21E-07	TNF/PTGS2/IL1B/NOS2/AKT1/TLR4	6
BP	GO:0071219	cellular response to molecule of bacterial origin	8/41	221/18723	2.27E-08	6.84E-05	4.21E-07	TNF/IL1B/IL6/NOS2/AKT1/TGFB1/TLR4/MAPK1	8
BP	GO:0070661	leukocyte proliferation	9/41	318/18723	2.28E-08	6.88E-05	4.21E-07	CASP3/IL1B/IL6/BCL2/TP53/TLR4/MAPK1/IL2/ATM	9
BP	GO:2001057	reactive nitrogen species metabolic process	6/41	82/18723	2.33E-08	7.03E-05	4.21E-07	TNF/PTGS2/IL1B/NOS2/AKT1/TLR4	6
BP	GO:0062013	positive regulation of small molecule metabolic process	7/41	143/18723	2.37E-08	7.13E-05	4.21E-07	TNF/PTGS2/IL1B/NOS2/AKT1/PPARG/GPT	7
BP	GO:0002683	negative regulation of immune system process	10/41	434/18723	2.38E-08	7.18E-05	4.21E-07	TNF/CASP3/AKT1/PPARG/TGFB1/HMOX1/TLR4/IL2/ADORA1/ATM	10
BP	GO:0009895	negative regulation of catabolic process	9/41	320/18723	2.41E-08	7.26E-05	4.21E-07	TNF/IL1B/NOS2/AKT1/ADRA2A/BCL2/TP53/HMOX1/ADORA1	9
BP	GO:0071496	cellular response to external stimulus	9/41	320/18723	2.41E-08	7.26E-05	4.21E-07	PTGS2/IL1B/AKT1/BCL2/TP53/FOXA2/HMOX1/TLR4/MAPK1	9
BP	GO:0045429	positive regulation of nitric oxide	5/41	40/18723	2.43E-08	7.33E-05	4.21E-07	TNF/PTGS2/IL1B/AKT1/TLR4	5

		biosynthetic process							
BP	GO:1903799	negative regulation of production of miRNAs involved in gene silencing by miRNA	4/41	15/18723	2.65E-08	8.00E-05	4.52E-07	TNF/IL6/TP53/TGFB1	4
BP	GO:0002703	regulation of leukocyte mediated immunity	8/41	226/18723	2.70E-08	8.14E-05	4.52E-07	TNF/IL1B/IL6/NOS2/TGFB1/HMOX1/TLR4/IL2	8
BP	GO:1904407	positive regulation of nitric oxide metabolic process	5/41	41/18723	2.77E-08	8.34E-05	4.56E-07	TNF/PTGS2/IL1B/AKT1/TLR4	5
BP	GO:0051384	response to glucocorticoid	7/41	148/18723	3.00E-08	9.05E-05	4.87E-07	TNF/PTGS2/CASP3/IL6/BCL2/CCND1/CASP9	7
BP	GO:0009411	response to UV	7/41	149/18723	3.15E-08	9.48E-05	4.95E-07	PTGS2/CASP3/AKT1/BCL2/TP53/CCND1/CASP9	7
BP	GO:0045834	positive regulation of lipid metabolic process	7/41	149/18723	3.15E-08	9.48E-05	4.95E-07	TNF/PTGS2/IL1B/AKT1/PPARG/TGFB1/ADORA1	7
BP	GO:0019216	regulation of lipid metabolic process	9/41	331/18723	3.22E-08	9.71E-05	4.99E-07	TNF/PTGS2/IL1B/AKT1/ADRA2A/HNF4A/PPARG/TGFB1/ADORA1	9
BP	GO:0010594	regulation of endothelial cell migration	8/41	232/18723	3.31E-08	9.97E-05	5.02E-07	TNF/PTGS2/AKT1/PPARG/EGF/TGFB1/HMOX1/PIK3CD	8
BP	GO:00	leukocyte	6/41	87/18723	3.34E-08	0.0001005	5.02E-07	CASP3/IL6/AKT1/BCL2/IL2/PIK3CD	6

	01776	homeostasis				2			
BP	GO:0042113	B cell activation	9/41	334/18723	3.48E-08	0.00010497	5.09E-07	CASP3/IL6/BCL2/TP53/TGFB1/TLR4/IL2/ATM/PIK3CD	9
BP	GO:0062012	regulation of small molecule metabolic process	9/41	334/18723	3.48E-08	0.00010497	5.09E-07	TNF/PTGS2/IL1B/NOS2/AKT1/PPARG/TP53/FOXA2/GPT	9
BP	GO:0002699	positive regulation of immune effector process	8/41	235/18723	3.66E-08	0.000110184	5.27E-07	TNF/IL1B/IL6/NOS2/TGFB1/HMOX1/TLR4/IL2	8
BP	GO:0030072	peptide hormone secretion	8/41	236/18723	3.78E-08	0.00011387	5.37E-07	TNF/IL1B/IL6/NOS2/ADRA2A/HNF4A/FOXA2/HNF1A	8
BP	GO:0018209	peptidyl-serine modification	9/41	338/18723	3.86E-08	0.000116267	5.41E-07	TNF/PTGS2/IL6/AKT1/BCL2/TGFB1/MAPK1/RPS6KA3/ATM	9
BP	GO:0023061	signal release	10/41	463/18723	4.38E-08	0.000131963	6.06E-07	TNF/IL1B/IL6/NOS2/ADRA2A/HNF4A/FOXA2/HNF1A/ADRA2C/ADORA1	10
BP	GO:0035094	response to nicotine	5/41	46/18723	5.02E-08	0.000151283	6.72E-07	TNF/CASP3/BCL2/HMOX1/MAPK1	5
BP	GO:0051051	negative regulation of transport	10/41	470/18723	5.04E-08	0.000151915	6.72E-07	TNF/PTGS2/IL1B/AKT1/ADRA2A/BCL2/EGF/HMOX1/ADRA2C/ADORA1	10
BP	GO:0070663	regulation of leukocyte proliferation	8/41	245/18723	5.05E-08	0.000152127	6.72E-07	CASP3/IL1B/IL6/BCL2/TLR4/MAPK1/IL2/ATM	8
BP	GO:0031667	response to nutrient levels	10/41	474/18723	5.46E-08	0.000164474	7.17E-07	PTGS2/AKT1/BCL2/PPARG/TP53/FOXA2/HMOX1/CCND1/GPT/MAPK1	10
BP	GO:0046883	regulation of hormone secretion	8/41	249/18723	5.72E-08	0.000172402	7.42E-07	TNF/IL1B/IL6/NOS2/ADRA2A/HNF4A/FOXA2/ADORA1	8

BP	GO:0002718	regulation of cytokine production involved in immune response	6/41	96/18723	6.03E-08	0.000181887	7.71E-07	TNF/IL1B/IL6/TGFB1/HMOX1/TLR4	6
BP	GO:0002700	regulation of production of molecular mediator of immune response	7/41	164/18723	6.09E-08	0.00018354	7.71E-07	TNF/IL1B/IL6/TGFB1/HMOX1/TLR4/IL2	7
BP	GO:0002673	regulation of acute inflammatory response	5/41	48/18723	6.25E-08	0.000188373	7.82E-07	TNF/PTGS2/IL1B/IL6/ADORA1	5
BP	GO:0050796	regulation of insulin secretion	7/41	165/18723	6.35E-08	0.000191366	7.85E-07	TNF/IL1B/IL6/NOS2/ADRA2A/HNF4A/FOXA2	7
BP	GO:0002367	cytokine production involved in immune response	6/41	98/18723	6.83E-08	0.000205862	8.29E-07	TNF/IL1B/IL6/TGFB1/HMOX1/TLR4	6
BP	GO:0031960	response to corticosteroid	7/41	167/18723	6.90E-08	0.000207867	8.29E-07	TNF/PTGS2/CASP3/IL6/BCL2/CCND1/CASP9	7
BP	GO:0060964	regulation of gene silencing by miRNA	5/41	49/18723	6.95E-08	0.000209443	8.29E-07	TNF/IL6/PPARG/TP53/TGFB1	5
BP	GO:0008630	intrinsic apoptotic signaling pathway in response to DNA damage	6/41	99/18723	7.26E-08	0.000218792	8.56E-07	TNF/BCL2/TP53/HMOX1/CASP9/ATM	6
BP	GO:00	response to metal	9/41	373/18723	8.96E-08	0.0002699	1.04E-06	PTGS2/CASP3/AKT1/BCL2/HMOX1/CCND1/CYP1A	9

	10038	ion				75		2/MAPK1/CASP9	
BP	GO:00 50730	regulation of peptidyl-tyrosine phosphorylation	8/41	264/18723	8.98E-08	0.0002705 96	1.04E-06	TNF/IL6/ADRA2A/TP53/EGF/TGFB1/IL2/ADORA1	8
BP	GO:00 60147	regulation of posttranscriptional gene silencing	5/41	52/18723	9.43E-08	0.0002840 87	1.08E-06	TNF/IL6/PPARG/TP53/TGFB1	5
BP	GO:00 50878	regulation of body fluid levels	9/41	379/18723	1.03E-07	0.0003092 49	1.15E-06	IL6/ADRA2A/XDH/HNF4A/FOXA2/CCND1/TLR4/AD RA2C/ADORA1	9
BP	GO:00 43405	regulation of MAP kinase activity	7/41	177/18723	1.03E-07	0.0003096 1	1.15E-06	TNF/IL1B/ADRA2A/PPARG/EGF/TGFB1/TLR4	7
BP	GO:00 60966	regulation of gene silencing by RNA	5/41	53/18723	1.04E-07	0.0003131 76	1.15E-06	TNF/IL6/PPARG/TP53/TGFB1	5
BP	GO:00 50678	regulation of epithelial cell proliferation	9/41	381/18723	1.07E-07	0.0003234 05	1.17E-06	TNF/AKT1/XDH/PPARG/EGF/TGFB1/HMOX1/CCND 1/PIK3CD	9
BP	GO:00 01936	regulation of endothelial cell proliferation	7/41	179/18723	1.11E-07	0.0003343 31	1.20E-06	TNF/AKT1/XDH/PPARG/EGF/HMOX1/PIK3CD	7
BP	GO:00 42326	negative regulation of phosphorylation	9/41	385/18723	1.17E-07	0.0003534 22	1.24E-06	CASP3/IL1B/AKT1/XDH/CDKN2A/PPARG/FOXA2/T GFB1/IL2	9
BP	GO:00 45786	negative regulation of cell cycle	9/41	385/18723	1.17E-07	0.0003534 22	1.24E-06	TNF/PTGS2/CASP3/BCL2/CDKN2A/TP53/TGFB1/C CND1/ATM	9
BP	GO:00 33138	positive regulation of peptidyl-serine phosphorylation	6/41	108/18723	1.22E-07	0.0003682 74	1.28E-06	TNF/PTGS2/IL6/AKT1/BCL2/TGFB1	6

BP	GO:0043542	endothelial cell migration	8/41	279/18723	1.37E-07	0.000413574	1.42E-06	TNF/PTGS2/AKT1/PPARG/EGF/TGFB1/HMOX1/PIK3CD	8
BP	GO:0043406	positive regulation of MAP kinase activity	6/41	112/18723	1.52E-07	0.000457483	1.56E-06	TNF/IL1B/ADRA2A/EGF/TGFB1/TLR4	6
BP	GO:0046651	lymphocyte proliferation	8/41	288/18723	1.75E-07	0.000527313	1.78E-06	CASP3/IL1B/IL6/BCL2/TP53/TLR4/IL2/ATM	8
BP	GO:0001935	endothelial cell proliferation	7/41	193/18723	1.85E-07	0.000558703	1.85E-06	TNF/AKT1/XDH/PPARG/EGF/HMOX1/PIK3CD	7
BP	GO:0007565	female pregnancy	7/41	193/18723	1.85E-07	0.000558703	1.85E-06	PTGS2/IL1B/AKT1/IDO1/BCL2/MAPK1/ADRA2C	7
BP	GO:0032943	mononuclear cell proliferation	8/41	291/18723	1.89E-07	0.000570763	1.87E-06	CASP3/IL1B/IL6/BCL2/TP53/TLR4/IL2/ATM	8
BP	GO:0010632	regulation of epithelial cell migration	8/41	292/18723	1.94E-07	0.000585913	1.90E-06	TNF/PTGS2/AKT1/PPARG/EGF/TGFB1/HMOX1/PIK3CD	8
BP	GO:0090276	regulation of peptide hormone secretion	7/41	196/18723	2.06E-07	0.000620485	2.00E-06	TNF/IL1B/IL6/NOS2/ADRA2A/HNF4A/FOXA2	7
BP	GO:0050994	regulation of lipid catabolic process	5/41	61/18723	2.13E-07	0.000640968	2.04E-06	TNF/IL1B/AKT1/ADRA2A/ADORA1	5
BP	GO:0050864	regulation of B cell activation	7/41	198/18723	2.21E-07	0.000664796	2.10E-06	CASP3/IL6/BCL2/TGFB1/TLR4/IL2/ATM	7
BP	GO:0010573	vascular endothelial growth factor production	5/41	62/18723	2.31E-07	0.000696076	2.14E-06	TNF/PTGS2/IL1B/IL6/TGFB1	5

BP	GO:0032757	positive regulation of interleukin-8 production	5/41	62/18723	2.31E-07	0.000696076	2.14E-06	TNF/IL1B/IL6/NOS2/TLR4	5
BP	GO:0045428	regulation of nitric oxide biosynthetic process	5/41	62/18723	2.31E-07	0.000696076	2.14E-06	TNF/PTGS2/IL1B/AKT1/TLR4	5
BP	GO:0038127	ERBB signaling pathway	6/41	121/18723	2.40E-07	0.000724383	2.20E-06	AKT1/ADRA2A/EGF/TGFB1/MAPK1/ADORA1	6
BP	GO:1903798	regulation of production of miRNAs involved in gene silencing by miRNA	4/41	25/18723	2.42E-07	0.000729682	2.20E-06	TNF/IL6/TP53/TGFB1	4
BP	GO:0030888	regulation of B cell proliferation	5/41	64/18723	2.71E-07	0.000817531	2.43E-06	CASP3/BCL2/TLR4/IL2/ATM	5
BP	GO:0080164	regulation of nitric oxide metabolic process	5/41	64/18723	2.71E-07	0.000817531	2.43E-06	TNF/PTGS2/IL1B/AKT1/TLR4	5
BP	GO:0001666	response to hypoxia	8/41	307/18723	2.85E-07	0.000858314	2.49E-06	PTGS2/CASP3/NOS2/AKT1/BCL2/TP53/HMOX1/ADORA1	8
BP	GO:0070920	regulation of production of small RNA involved in gene silencing by RNA	4/41	26/18723	2.86E-07	0.000860988	2.49E-06	TNF/IL6/TP53/TGFB1	4
BP	GO:20	positive regulation	4/41	26/18723	2.86E-07	0.0008609	2.49E-06	IDO1/CDKN2A/TP53/PIK3CD	4

	00108	of leukocyte apoptotic process				88			
BP	GO:0038034	signal transduction in absence of ligand	5/41	65/18723	2.93E-07	0.000884239	2.52E-06	TNF/IL1B/AKT1/BCL2/IL2	5
BP	GO:0097192	extrinsic apoptotic signaling pathway in absence of ligand	5/41	65/18723	2.93E-07	0.000884239	2.52E-06	TNF/IL1B/AKT1/BCL2/IL2	5
BP	GO:0052548	regulation of endopeptidase activity	9/41	432/18723	3.10E-07	0.000935704	2.64E-06	TNF/PTGS2/AKT1/GRIN2B/GRIN1/XDH/PPARG/CASP9/RPS6KA3	9
BP	GO:1905953	negative regulation of lipid localization	5/41	66/18723	3.17E-07	0.000955184	2.66E-06	TNF/IL6/AKT1/PPARG/EGF	5
BP	GO:0043281	regulation of cysteine-type endopeptidase activity involved in apoptotic process	7/41	209/18723	3.18E-07	0.000959184	2.66E-06	TNF/PTGS2/AKT1/XDH/PPARG/CASP9/RPS6KA3	7
BP	GO:0031622	positive regulation of fever generation	3/41	7/18723	3.39E-07	0.001021919	2.80E-06	TNF/PTGS2/IL1B	3
BP	GO:0043491	protein kinase B signaling	7/41	211/18723	3.39E-07	0.001023034	2.80E-06	TNF/IL1B/AKT1/XDH/EGF/TGFB1/PIK3CD	7
BP	GO:0043523	regulation of neuron apoptotic process	7/41	212/18723	3.50E-07	0.001056277	2.86E-06	TNF/CASP3/BCL2/TP53/HMOX1/CASP9/GABRB2	7

BP	GO:0050804	modulation of chemical synaptic transmission	9/41	439/18723	3.55E-07	0.001071035	2.87E-06	TNF/PTGS2/IL1B/ADRA2A/GRIN2B/GRIN1/ACHE/MAPK1/ADORA1	9
BP	GO:0002443	leukocyte mediated immunity	9/41	440/18723	3.62E-07	0.001091694	2.87E-06	TNF/IL1B/IL6/NOS2/TGFB1/HMOX1/TLR4/IL2/PIK3CD	9
BP	GO:0051090	regulation of DNA-binding transcription factor activity	9/41	440/18723	3.62E-07	0.001091694	2.87E-06	TNF/IL1B/IL6/AKT1/CDKN2A/PPARG/FOXA2/HMOX1/TLR4	9
BP	GO:0099177	regulation of trans-synaptic signaling	9/41	440/18723	3.62E-07	0.001091694	2.87E-06	TNF/PTGS2/IL1B/ADRA2A/GRIN2B/GRIN1/ACHE/MAPK1/ADORA1	9
BP	GO:0002675	positive regulation of acute inflammatory response	4/41	28/18723	3.90E-07	0.001175455	3.02E-06	TNF/PTGS2/IL1B/IL6	4
BP	GO:0010575	positive regulation of vascular endothelial growth factor production	4/41	28/18723	3.90E-07	0.001175455	3.02E-06	PTGS2/IL1B/IL6/TGFB1	4
BP	GO:0009416	response to light stimulus	8/41	320/18723	3.90E-07	0.001176099	3.02E-06	PTGS2/CASP3/AKT1/GRIN1/BCL2/TP53/CCND1/CASP9	8
BP	GO:0006979	response to oxidative stress	9/41	446/18723	4.06E-07	0.001223081	3.12E-06	PTGS2/CASP3/IL6/AKT1/BCL2/TP53/HMOX1/TLR4/MAPK1	9
BP	GO:0036293	response to decreased oxygen	8/41	322/18723	4.09E-07	0.001232981	3.12E-06	PTGS2/CASP3/NOS2/AKT1/BCL2/TP53/HMOX1/ADORA1	8

		levels							
BP	GO:0010595	positive regulation of endothelial cell migration	6/41	133/18723	4.21E-07	0.001267641	3.19E-06	PTGS2/AKT1/EGF/TGFB1/HMOX1/PIK3CD	6
BP	GO:0097191	extrinsic apoptotic signaling pathway	7/41	219/18723	4.36E-07	0.001315472	3.28E-06	TNF/IL1B/AKT1/BCL2/TGFB1/HMOX1/IL2	7
BP	GO:0002705	positive regulation of leukocyte mediated immunity	6/41	134/18723	4.40E-07	0.001324931	3.28E-06	TNF/IL1B/IL6/NOS2/TGFB1/IL2	6
BP	GO:0044706	multi-multicellular organism process	7/41	220/18723	4.50E-07	0.001356534	3.32E-06	PTGS2/IL1B/AKT1/IDO1/BCL2/MAPK1/ADRA2C	7
BP	GO:0033028	myeloid cell apoptotic process	4/41	29/18723	4.52E-07	0.001361373	3.32E-06	IL6/BCL2/CDKN2A/PIK3CD	4
BP	GO:0032722	positive regulation of chemokine production	5/41	71/18723	4.58E-07	0.00138046	3.35E-06	TNF/IL1B/IL6/HMOX1/TLR4	5
BP	GO:0009314	response to radiation	9/41	456/18723	4.89E-07	0.001472718	3.55E-06	PTGS2/CASP3/AKT1/GRIN1/BCL2/TP53/CCND1/CASP9/ATM	9
BP	GO:0007346	regulation of mitotic cell cycle	9/41	457/18723	4.98E-07	0.001499959	3.58E-06	TNF/IL1B/AKT1/BCL2/TP53/EGF/TGFB1/CCND1/ATM	9
BP	GO:0071214	cellular response to abiotic stimulus	8/41	331/18723	5.04E-07	0.001519118	3.58E-06	PTGS2/CASP3/IL1B/AKT1/TP53/TLR4/CASP9/ATM	8
BP	GO:0104004	cellular response to environmental stimulus	8/41	331/18723	5.04E-07	0.001519118	3.58E-06	PTGS2/CASP3/IL1B/AKT1/TP53/TLR4/CASP9/ATM	8
BP	GO:00	regulation of	7/41	225/18723	5.24E-07	0.0015784	3.70E-06	CASP3/IL1B/IL6/BCL2/TLR4/IL2/ATM	7

	50670	lymphocyte proliferation				07			
BP	GO:0052547	regulation of peptidase activity	9/41	461/18723	5.35E-07	0.001613348	3.73E-06	TNF/PTGS2/AKT1/GRIN2B/GRIN1/XDH/PPARG/CASP9/RPS6KA3	9
BP	GO:0002246	wound healing involved in inflammatory response	3/41	8/18723	5.42E-07	0.001632582	3.73E-06	TGFB1/HMOX1/TLR4	3
BP	GO:0071420	cellular response to histamine	3/41	8/18723	5.42E-07	0.001632582	3.73E-06	GABRG2/GABRB2/GABRA1	3
BP	GO:0009410	response to xenobiotic stimulus	9/41	462/18723	5.45E-07	0.001642832	3.73E-06	PTGS2/CASP3/IL1B/NOS2/GRIN1/BCL2/HNF4A/HMOX1/CYP1A2	9
BP	GO:0045598	regulation of fat cell differentiation	6/41	139/18723	5.46E-07	0.001644226	3.73E-06	TNF/PTGS2/IL6/AKT1/PPARG/TGFB1	6
BP	GO:0032944	regulation of mononuclear cell proliferation	7/41	227/18723	5.56E-07	0.001675322	3.77E-06	CASP3/IL1B/IL6/BCL2/TLR4/IL2/ATM	7
BP	GO:0045444	fat cell differentiation	7/41	229/18723	5.90E-07	0.001777195	3.94E-06	TNF/PTGS2/IL6/AKT1/PPARG/TGFB1/CCND1	7
BP	GO:0032355	response to estradiol	6/41	141/18723	5.93E-07	0.001788441	3.94E-06	PTGS2/CASP3/TGFB1/CCND1/CYP1A2/CASP9	6
BP	GO:0030212	hyaluronan metabolic process	4/41	31/18723	5.96E-07	0.001797832	3.94E-06	IL1B/AKT1/EGF/TGFB1	4
BP	GO:0002697	regulation of immune effector process	8/41	339/18723	6.04E-07	0.001819477	3.94E-06	TNF/IL1B/IL6/NOS2/TGFB1/HMOX1/TLR4/IL2	8

BP	GO:0007568	aging	8/41	339/18723	6.04E-07	0.001819477	3.94E-06	PTGS2/AKT1/BCL2/CDKN2A/TP53/MAPK1/CASP9/ATM	8
BP	GO:0048545	response to steroid hormone	8/41	339/18723	6.04E-07	0.001819477	3.94E-06	TNF/PTGS2/CASP3/IL6/BCL2/TGFB1/CCND1/CASP9	8
BP	GO:0050729	positive regulation of inflammatory response	6/41	142/18723	6.19E-07	0.001864345	4.01E-06	TNF/PTGS2/IL1B/IL6/TLR4/IL2	6
BP	GO:2000379	positive regulation of reactive oxygen species metabolic process	5/41	76/18723	6.45E-07	0.001943183	4.13E-06	GRIN1/XDH/TP53/TGFB1/TLR4	5
BP	GO:0001933	negative regulation of protein phosphorylation	8/41	342/18723	6.45E-07	0.00194453	4.13E-06	CASP3/IL1B/AKT1/XDH/CDKN2A/PPARG/TGFB1/IL2	8
BP	GO:0033135	regulation of peptidyl-serine phosphorylation	6/41	144/18723	6.72E-07	0.002024088	4.28E-06	TNF/PTGS2/IL6/AKT1/BCL2/TGFB1	6
BP	GO:0031016	pancreas development	5/41	77/18723	6.88E-07	0.002074796	4.36E-06	IL6/AKT1/HNF4A/FOXA2/HNF1A	5
BP	GO:0070482	response to oxygen levels	8/41	347/18723	7.20E-07	0.002169322	4.53E-06	PTGS2/CASP3/NOS2/AKT1/BCL2/TP53/HMOX1/ADORA1	8
BP	GO:0043410	positive regulation of MAPK cascade	9/41	480/18723	7.50E-07	0.002259708	4.69E-06	TNF/IL1B/IL6/ADRA2A/XDH/EGF/TGFB1/TLR4/ADRA2C	9
BP	GO:0010165	response to X-ray	4/41	33/18723	7.73E-07	0.002330683	4.75E-06	CASP3/TP53/CCND1/ATM	4
BP	GO:19	negative regulation	4/41	33/18723	7.73E-07	0.0023306	4.75E-06	TNF/IL1B/AKT1/BCL2	4

	01099	of signal transduction in absence of ligand				83			
BP	GO:2001240	negative regulation of extrinsic apoptotic signaling pathway in absence of ligand	4/41	33/18723	7.73E-07	0.002330683	4.75E-06	TNF/IL1B/AKT1/BCL2	4
BP	GO:2001056	positive regulation of cysteine-type endopeptidase activity	6/41	148/18723	7.89E-07	0.00237732	4.82E-06	TNF/GRIN2B/GRIN1/XDH/PPARG/CASP9	6
BP	GO:0031620	regulation of fever generation	3/41	9/18723	8.11E-07	0.002445146	4.92E-06	TNF/PTGS2/IL1B	3
BP	GO:0070665	positive regulation of leukocyte proliferation	6/41	150/18723	8.53E-07	0.002572	5.15E-06	IL1B/IL6/BCL2/TLR4/MAPK1/IL2	6
BP	GO:0060968	regulation of gene silencing	5/41	81/18723	8.87E-07	0.002673353	5.26E-06	TNF/IL6/PPARG/TP53/TGFB1	5
BP	GO:2000106	regulation of leukocyte apoptotic process	5/41	81/18723	8.87E-07	0.002673353	5.26E-06	IDO1/CDKN2A/TP53/IL2/PIK3CD	5
BP	GO:0043535	regulation of blood vessel endothelial cell migration	6/41	151/18723	8.87E-07	0.002674127	5.26E-06	TNF/PTGS2/AKT1/PPARG/TGFB1/HMOX1	6
BP	GO:00	epithelial cell	8/41	357/18723	8.91E-07	0.0026862	5.26E-06	TNF/PTGS2/AKT1/PPARG/EGF/TGFB1/HMOX1/PIK	8

	10631	migration				91		3CD	
BP	GO:0090132	epithelium migration	8/41	360/18723	9.49E-07	0.002860565	5.56E-06	TNF/PTGS2/AKT1/PPARG/EGF/TGFB1/HMOX1/PIK3CD	8
BP	GO:0051402	neuron apoptotic process	7/41	246/18723	9.53E-07	0.00287365	5.56E-06	TNF/CASP3/BCL2/TP53/HMOX1/CASP9/GABRB2	7
BP	GO:0090130	tissue migration	8/41	365/18723	1.05E-06	0.003172522	6.10E-06	TNF/PTGS2/AKT1/PPARG/EGF/TGFB1/HMOX1/PIK3CD	8
BP	GO:0097194	execution phase of apoptosis	5/41	85/18723	1.13E-06	0.003400617	6.51E-06	CASP3/IL6/AKT1/TP53/CASP9	5
BP	GO:0043029	T cell homeostasis	4/41	37/18723	1.24E-06	0.003738008	7.11E-06	CASP3/AKT1/BCL2/IL2	4
BP	GO:0018108	peptidyl-tyrosine phosphorylation	8/41	375/18723	1.29E-06	0.003884478	7.35E-06	TNF/IL6/ADRA2A/TP53/EGF/TGFB1/IL2/ADORA1	8
BP	GO:0042593	glucose homeostasis	7/41	258/18723	1.31E-06	0.003950457	7.43E-06	IL6/AKT1/ADRA2A/HNF4A/PPARG/FOXA2/HNF1A	7
BP	GO:0033500	carbohydrate homeostasis	7/41	259/18723	1.34E-06	0.004053703	7.59E-06	IL6/AKT1/ADRA2A/HNF4A/PPARG/FOXA2/HNF1A	7
BP	GO:0018212	peptidyl-tyrosine modification	8/41	378/18723	1.37E-06	0.004123001	7.67E-06	TNF/IL6/ADRA2A/TP53/EGF/TGFB1/IL2/ADORA1	8
BP	GO:0001660	fever generation	3/41	11/18723	1.59E-06	0.004788357	8.77E-06	TNF/PTGS2/IL1B	3
BP	GO:0031652	positive regulation of heat generation	3/41	11/18723	1.59E-06	0.004788357	8.77E-06	TNF/PTGS2/IL1B	3
BP	GO:0034776	response to histamine	3/41	11/18723	1.59E-06	0.004788357	8.77E-06	GABRG2/GABRB2/GABRA1	3
BP	GO:00	regulation of	8/41	386/18723	1.60E-06	0.0048211	8.78E-06	TNF/PTGS2/IL1B/IL6/PPARG/TLR4/IL2/ADORA1	8

	50727	inflammatory response				15			
BP	GO:0051348	negative regulation of transferase activity	7/41	268/18723	1.69E-06	0.005089183	9.22E-06	CASP3/IL1B/AKT1/CDKN2A/PPARG/TP53/FOXA2	7
BP	GO:0071276	cellular response to cadmium ion	4/41	40/18723	1.71E-06	0.005148001	9.23E-06	AKT1/HMOX1/CYP1A2/MAPK1	4
BP	GO:0150077	regulation of neuroinflammatory response	4/41	40/18723	1.71E-06	0.005148001	9.23E-06	TNF/PTGS2/IL1B/IL6	4
BP	GO:0032755	positive regulation of interleukin-6 production	5/41	93/18723	1.76E-06	0.005319304	9.49E-06	TNF/IL1B/IL6/NOS2/TLR4	5
BP	GO:0051048	negative regulation of secretion	6/41	171/18723	1.83E-06	0.005527157	9.81E-06	IL1B/ADRA2A/EGF/HMOX1/ADRA2C/ADORA1	6
BP	GO:0048872	homeostasis of number of cells	7/41	272/18723	1.86E-06	0.005615866	9.92E-06	CASP3/IL6/AKT1/BCL2/HMOX1/IL2/PIK3CD	7
BP	GO:0010469	regulation of signaling receptor activity	6/41	173/18723	1.96E-06	0.005913651	1.04E-05	TNF/ADRA2A/PPARG/EGF/ADRA2C/ADORA1	6
BP	GO:0002366	leukocyte activation involved in immune response	7/41	275/18723	2.00E-06	0.006040192	1.06E-05	IL6/TP53/TGFB1/HMOX1/TLR4/IL2/PIK3CD	7
BP	GO:0001659	temperature homeostasis	6/41	174/18723	2.03E-06	0.006115016	1.06E-05	TNF/PTGS2/IL1B/ACHE/TLR4/ADORA1	6

BP	GO:1902895	positive regulation of pri-miRNA transcription by RNA polymerase II	4/41	42/18723	2.09E-06	0.006285111	1.09E-05	TNF/PPARG/TP53/TGFB1	4
BP	GO:1903800	positive regulation of production of miRNAs involved in gene silencing by miRNA	3/41	12/18723	2.12E-06	0.00637476	1.10E-05	IL6/TP53/TGFB1	3
BP	GO:0010634	positive regulation of epithelial cell migration	6/41	176/18723	2.17E-06	0.006534563	1.11E-05	PTGS2/AKT1/EGF/TGFB1/HMOX1/PIK3CD	6
BP	GO:0043534	blood vessel endothelial cell migration	6/41	176/18723	2.17E-06	0.006534563	1.11E-05	TNF/PTGS2/AKT1/PPARG/TGFB1/HMOX1	6
BP	GO:2001237	negative regulation of extrinsic apoptotic signaling pathway	5/41	97/18723	2.17E-06	0.006553922	1.11E-05	TNF/IL1B/AKT1/BCL2/HMOX1	5
BP	GO:0002263	cell activation involved in immune response	7/41	279/18723	2.21E-06	0.006647452	1.12E-05	IL6/TP53/TGFB1/HMOX1/TLR4/IL2/PIK3CD	7
BP	GO:0032642	regulation of chemokine production	5/41	98/18723	2.29E-06	0.006895216	1.16E-05	TNF/IL1B/IL6/HMOX1/TLR4	5
BP	GO:20	regulation of	4/41	43/18723	2.30E-06	0.0069187	1.16E-05	TNF/IL1B/AKT1/BCL2	4

	01239	extrinsic apoptotic signaling pathway in absence of ligand				9			
BP	GO:00 06109	regulation of carbohydrate metabolic process	6/41	178/18723	2.31E-06	0.0069773 27	1.16E-05	AKT1/TP53/FOXA2/EGF/TGFB1/GPT	6
BP	GO:00 10950	positive regulation of endopeptidase activity	6/41	179/18723	2.39E-06	0.0072077 2	1.19E-05	TNF/GRIN2B/GRIN1/XDH/PPARG/CASP9	6
BP	GO:00 32602	chemokine production	5/41	99/18723	2.41E-06	0.0072503 45	1.19E-05	TNF/IL1B/IL6/HMOX1/TLR4	5
BP	GO:00 42100	B cell proliferation	5/41	99/18723	2.41E-06	0.0072503 45	1.19E-05	CASP3/BCL2/TLR4/IL2/ATM	5
BP	GO:00 32620	interleukin-17 production	4/41	44/18723	2.52E-06	0.0075986 44	1.23E-05	IL6/TGFB1/TLR4/IL2	4
BP	GO:00 32660	regulation of interleukin-17 production	4/41	44/18723	2.52E-06	0.0075986 44	1.23E-05	IL6/TGFB1/TLR4/IL2	4
BP	GO:01 50076	neuroinflammatory response	4/41	44/18723	2.52E-06	0.0075986 44	1.23E-05	TNF/PTGS2/IL1B/IL6	4
BP	GO:00 97696	receptor signaling pathway via STAT	6/41	181/18723	2.55E-06	0.0076871 52	1.23E-05	TNF/IL6/PPARG/EGF/TGFB1/IL2	6
BP	GO:19 05952	regulation of lipid localization	6/41	181/18723	2.55E-06	0.0076871 52	1.23E-05	TNF/IL1B/IL6/AKT1/PPARG/EGF	6
BP	GO:00	regulation of cell	8/41	414/18723	2.69E-06	0.0081157	1.29E-05	AKT1/BCL2/CDKN2A/HNF4A/TP53/TGFB1/IL2/RPS6	8

	01558	growth				93		KA3	
BP	GO:00 34599	cellular response to oxidative stress	7/41	288/18723	2.72E-06	0.0082026 98	1.30E-05	IL6/AKT1/BCL2/TP53/HMOX1/TLR4/MAPK1	7
BP	GO:00 45741	positive regulation of epidermal growth factor-activated receptor activity	3/41	13/18723	2.75E-06	0.0082745 79	1.31E-05	ADRA2A/EGF/ADORA1	3
BP	GO:20 00273	positive regulation of signaling receptor activity	4/41	45/18723	2.76E-06	0.0083267 99	1.31E-05	ADRA2A/EGF/ADRA2C/ADORA1	4
BP	GO:00 32677	regulation of interleukin-8 production	5/41	102/18723	2.79E-06	0.0084028	1.31E-05	TNF/IL1B/IL6/NOS2/TLR4	5
BP	GO:00 45833	negative regulation of lipid metabolic process	5/41	102/18723	2.79E-06	0.0084028	1.31E-05	TNF/IL1B/AKT1/ADRA2A/ADORA1	5
BP	GO:00 32637	interleukin-8 production	5/41	103/18723	2.93E-06	0.0088173 61	1.37E-05	TNF/IL1B/IL6/NOS2/TLR4	5
BP	GO:00 08217	regulation of blood pressure	6/41	186/18723	2.99E-06	0.0090005 33	1.39E-05	PTGS2/NOS2/COL1A2/PPARG/HMOX1/ADORA1	6
BP	GO:00 31018	endocrine pancreas development	4/41	46/18723	3.02E-06	0.0091054 2	1.40E-05	IL6/AKT1/HNF4A/FOXA2	4
BP	GO:00 50890	cognition	7/41	296/18723	3.26E-06	0.0098301 51	1.50E-05	TNF/PTGS2/CASP3/GRIN2B/GRIN1/MAPK1/ADORA 1	7

BP	GO:1903131	mononuclear cell differentiation	8/41	426/18723	3.33E-06	0.010027114	1.53E-05	IL1B/IL6/BCL2/PPARG/TP53/IL2/ATM/PIK3CD	8
BP	GO:0032103	positive regulation of response to external stimulus	8/41	427/18723	3.38E-06	0.010202364	1.55E-05	TNF/PTGS2/IL1B/IL6/TGFB1/TLR4/MAPK1/IL2	8
BP	GO:0030213	hyaluronan biosynthetic process	3/41	14/18723	3.49E-06	0.010515258	1.58E-05	IL1B/EGF/TGFB1	3
BP	GO:0031650	regulation of heat generation	3/41	14/18723	3.49E-06	0.010515258	1.58E-05	TNF/PTGS2/IL1B	3
BP	GO:0002824	positive regulation of adaptive immune response based on somatic recombination of immune receptors built from immunoglobulin superfamily domains	5/41	107/18723	3.53E-06	0.010639176	1.59E-05	TNF/IL1B/IL6/TGFB1/IL2	5
BP	GO:0032872	regulation of stress-activated MAPK cascade	6/41	192/18723	3.59E-06	0.010812474	1.61E-05	TNF/IL1B/AKT1/XDH/TLR4/MAPK1	6
BP	GO:0042063	gliogenesis	7/41	301/18723	3.64E-06	0.010978112	1.63E-05	TNF/IL1B/IL6/AKT1/TGFB1/TLR4/MAPK1	7
BP	GO:00	epidermal growth	5/41	108/18723	3.70E-06	0.0111377	1.65E-05	AKT1/ADRA2A/EGF/TGFB1/ADORA1	5

	07173	factor receptor signaling pathway				64			
BP	GO:0051222	positive regulation of protein transport	7/41	303/18723	3.80E-06	0.011467619	1.69E-05	TNF/PTGS2/IL1B/ACHE/TGFB1/TLR4/MAPK1	7
BP	GO:0042391	regulation of membrane potential	8/41	434/18723	3.82E-06	0.011503685	1.69E-05	AKT1/GRIN2B/GRIN1/BCL2/ADORA1/GABRG2/GABRB2/GABRA1	8
BP	GO:0006953	acute-phase response	4/41	49/18723	3.90E-06	0.011766346	1.71E-05	TNF/PTGS2/IL1B/IL6	4
BP	GO:0051932	synaptic transmission, GABAergic	4/41	49/18723	3.90E-06	0.011766346	1.71E-05	ADORA1/GABRG2/GABRB2/GABRA1	4
BP	GO:0070302	regulation of stress-activated protein kinase signaling cascade	6/41	195/18723	3.92E-06	0.011823957	1.71E-05	TNF/IL1B/AKT1/XDH/TLR4/MAPK1	6
BP	GO:0010952	positive regulation of peptidase activity	6/41	197/18723	4.16E-06	0.01254012	1.81E-05	TNF/GRIN2B/GRIN1/XDH/PPARG/CASP9	6
BP	GO:0002440	production of molecular mediator of immune response	7/41	308/18723	4.24E-06	0.01277173	1.83E-05	TNF/IL1B/IL6/TGFB1/HMOX1/TLR4/IL2	7
BP	GO:0045986	negative regulation of smooth muscle contraction	3/41	15/18723	4.35E-06	0.013124075	1.87E-05	PTGS2/ADRA2C/ADORA1	3

BP	GO:0090594	inflammatory response to wounding	3/41	15/18723	4.35E-06	0.013124075	1.87E-05	TGFB1/HMOX1/TLR4	3
BP	GO:0002526	acute inflammatory response	5/41	112/18723	4.42E-06	0.013318637	1.88E-05	TNF/PTGS2/IL1B/IL6/ADORA1	5
BP	GO:0002821	positive regulation of adaptive immune response	5/41	112/18723	4.42E-06	0.013318637	1.88E-05	TNF/IL1B/IL6/TGFB1/IL2	5
BP	GO:0002708	positive regulation of lymphocyte mediated immunity	5/41	113/18723	4.62E-06	0.013912909	1.96E-05	TNF/IL1B/IL6/TGFB1/IL2	5
BP	GO:0051054	positive regulation of DNA metabolic process	6/41	201/18723	4.67E-06	0.014078556	1.97E-05	IL6/AKT1/TGFB1/MAPK1/IL2/ATM	6
BP	GO:1904892	regulation of receptor signaling pathway via STAT	5/41	114/18723	4.82E-06	0.014527798	2.02E-05	TNF/IL6/PPARG/EGF/TGFB1	5
BP	GO:0022407	regulation of cell-cell adhesion	8/41	448/18723	4.82E-06	0.014533812	2.02E-05	TNF/CASP3/IL1B/IL6/AKT1/FOXA2/TGFB1/IL2	8
BP	GO:0035196	production of miRNAs involved in gene silencing by miRNA	4/41	52/18723	4.96E-06	0.014963338	2.07E-05	TNF/IL6/TP53/TGFB1	4
BP	GO:0018107	peptidyl-threonine phosphorylation	5/41	116/18723	5.25E-06	0.015821473	2.18E-05	AKT1/BCL2/EGF/TGFB1/MAPK1	5
BP	GO:19	positive regulation	7/41	319/18723	5.34E-06	0.0160811	2.20E-05	TNF/PTGS2/IL1B/ACHE/TGFB1/TLR4/MAPK1	7

	04951	of establishment of protein localization				41			
BP	GO:0051044	positive regulation of membrane protein ectodomain proteolysis	3/41	16/18723	5.35E-06	0.016128133	2.20E-05	TNF/IL1B/ADRA2A	3
BP	GO:1904862	inhibitory synapse assembly	3/41	16/18723	5.35E-06	0.016128133	2.20E-05	GABRG2/GABRB2/GABRA1	3
BP	GO:0002702	positive regulation of production of molecular mediator of immune response	5/41	117/18723	5.47E-06	0.016501303	2.24E-05	IL1B/IL6/TGFB1/TLR4/IL2	5
BP	GO:0031050	dsRNA processing	4/41	54/18723	5.78E-06	0.017424445	2.33E-05	TNF/IL6/TP53/TGFB1	4
BP	GO:0070918	production of small RNA involved in gene silencing by RNA	4/41	54/18723	5.78E-06	0.017424445	2.33E-05	TNF/IL6/TP53/TGFB1	4
BP	GO:1902893	regulation of pri-miRNA transcription by RNA polymerase II	4/41	54/18723	5.78E-06	0.017424445	2.33E-05	TNF/PPARG/TP53/TGFB1	4
BP	GO:0002685	regulation of leukocyte migration	6/41	210/18723	6.01E-06	0.018106193	2.42E-05	TNF/IL6/AKT1/HMOX1/MAPK1/ADORA1	6
BP	GO:00	negative regulation	5/41	120/18723	6.20E-06	0.0186791	2.48E-05	CASP3/IL1B/AKT1/CDKN2A/PPARG	5

	71901	of protein serine/threonine kinase activity				78			
BP	GO:0061614	pri-miRNA transcription by RNA polymerase II	4/41	55/18723	6.22E-06	0.018761387	2.48E-05	TNF/PPARG/TP53/TGFB1	4
BP	GO:0034349	glial cell apoptotic process	3/41	17/18723	6.49E-06	0.019554369	2.57E-05	CASP3/TP53/CASP9	3
BP	GO:0090399	replicative senescence	3/41	17/18723	6.49E-06	0.019554369	2.57E-05	CDKN2A/TP53/ATM	3
BP	GO:0000082	G1/S transition of mitotic cell cycle	6/41	214/18723	6.69E-06	0.020173938	2.64E-05	AKT1/BCL2/CDKN2A/TP53/CCND1/ATM	6
BP	GO:0033619	membrane protein proteolysis	4/41	57/18723	7.19E-06	0.021660856	2.83E-05	TNF/IL1B/ADRA2A/TGFB1	4
BP	GO:0007596	blood coagulation	6/41	217/18723	7.25E-06	0.021847385	2.84E-05	IL6/ADRA2A/HNF4A/FOXA2/TLR4/ADRA2C	6
BP	GO:0001667	ameboidal-type cell migration	8/41	475/18723	7.40E-06	0.02231747	2.89E-05	TNF/PTGS2/AKT1/PPARG/EGF/TGFB1/HMOX1/PIK3CD	8
BP	GO:0018210	peptidyl-threonine modification	5/41	125/18723	7.57E-06	0.022803588	2.94E-05	AKT1/BCL2/EGF/TGFB1/MAPK1	5
BP	GO:0002931	response to ischemia	4/41	58/18723	7.71E-06	0.023228619	2.96E-05	AKT1/BCL2/TP53/CASP9	4
BP	GO:0010574	regulation of vascular endothelial growth factor production	4/41	58/18723	7.71E-06	0.023228619	2.96E-05	PTGS2/IL1B/IL6/TGFB1	4

BP	GO:0043525	positive regulation of neuron apoptotic process	4/41	58/18723	7.71E-06	0.023228619	2.96E-05	TNF/CASP3/TP53/CASP9	4
BP	GO:0030730	sequestering of triglyceride	3/41	18/18723	7.77E-06	0.023429548	2.96E-05	TNF/IL1B/PPARG	3
BP	GO:0031649	heat generation	3/41	18/18723	7.77E-06	0.023429548	2.96E-05	TNF/PTGS2/IL1B	3
BP	GO:0150078	positive regulation of neuroinflammatory response	3/41	18/18723	7.77E-06	0.023429548	2.96E-05	TNF/IL1B/IL6	3
BP	GO:0035270	endocrine system development	5/41	127/18723	8.18E-06	0.024639917	3.08E-05	IL6/AKT1/HNF4A/FOXA2/MAPK1	5
BP	GO:0016049	cell growth	8/41	482/18723	8.24E-06	0.024832409	3.08E-05	AKT1/BCL2/CDKN2A/HNF4A/TP53/TGFB1/IL2/RPS6KA3	8
BP	GO:0000302	response to reactive oxygen species	6/41	222/18723	8.26E-06	0.024885969	3.08E-05	CASP3/IL6/AKT1/BCL2/HMOX1/MAPK1	6
BP	GO:0007599	hemostasis	6/41	222/18723	8.26E-06	0.024885969	3.08E-05	IL6/ADRA2A/HNF4A/FOXA2/TLR4/ADRA2C	6
BP	GO:0050817	coagulation	6/41	222/18723	8.26E-06	0.024885969	3.08E-05	IL6/ADRA2A/HNF4A/FOXA2/TLR4/ADRA2C	6
BP	GO:2001234	negative regulation of apoptotic signaling pathway	6/41	224/18723	8.69E-06	0.02619355	3.23E-05	TNF/PTGS2/IL1B/AKT1/BCL2/HMOX1	6
BP	GO:00	negative regulation	7/41	344/18723	8.74E-06	0.0263274	3.24E-05	TNF/AKT1/BCL2/PPARG/TGFB1/HMOX1/ADORA1	7

	30336	of cell migration				43			
BP	GO:00 42110	T cell activation	8/41	487/18723	8.88E-06	0.0267723 18	3.28E-05	CASP3/IL1B/IL6/AKT1/BCL2/TP53/IL2/PIK3CD	8
BP	GO:00 10001	glial cell differentiation	6/41	225/18723	8.91E-06	0.0268679 37	3.28E-05	TNF/IL6/AKT1/TGFB1/TLR4/MAPK1	6
BP	GO:00 09896	positive regulation of catabolic process	8/41	492/18723	9.57E-06	0.0288390 57	3.51E-05	TNF/IL1B/IL6/AKT1/ADRA2A/EGF/HMOX1/ADORA1	8
BP	GO:00 02260	lymphocyte homeostasis	4/41	62/18723	1.01E-05	0.0303493 14	3.67E-05	CASP3/AKT1/BCL2/IL2	4
BP	GO:00 46824	positive regulation of nucleocytoplasmic transport	4/41	62/18723	1.01E-05	0.0303493 14	3.67E-05	PTGS2/IL1B/TGFB1/MAPK1	4
BP	GO:00 60252	positive regulation of glial cell proliferation	3/41	20/18723	1.08E-05	0.0326329 58	3.93E-05	TNF/IL1B/IL6	3
BP	GO:20 01233	regulation of apoptotic signaling pathway	7/41	356/18723	1.09E-05	0.0329007 19	3.95E-05	TNF/PTGS2/IL1B/AKT1/BCL2/TP53/HMOX1	7
BP	GO:00 45930	negative regulation of mitotic cell cycle	6/41	235/18723	1.14E-05	0.0344202 24	4.11E-05	TNF/BCL2/TP53/TGFB1/CCND1/ATM	6
BP	GO:00 06940	regulation of smooth muscle contraction	4/41	64/18723	1.14E-05	0.0344582 63	4.11E-05	PTGS2/ADRA2A/ADRA2C/ADORA1	4
BP	GO:00	regulation of DNA	7/41	359/18723	1.15E-05	0.0347412	4.11E-05	IL6/AKT1/TP53/TGFB1/MAPK1/IL2/ATM	7

	51052	metabolic process				71			
BP	GO:2000146	negative regulation of cell motility	7/41	359/18723	1.15E-05	0.034741271	4.11E-05	TNF/AKT1/BCL2/PPARG/TGFB1/HMOX1/ADORA1	7
BP	GO:0048762	mesenchymal cell differentiation	6/41	236/18723	1.17E-05	0.035261383	4.16E-05	IL1B/IL6/BCL2/FOXA2/TGFB1/MAPK1	6
BP	GO:0030879	mammary gland development	5/41	137/18723	1.18E-05	0.035629766	4.17E-05	AKT1/XDH/EGF/CCND1/MAPK1	5
BP	GO:0050671	positive regulation of lymphocyte proliferation	5/41	137/18723	1.18E-05	0.035629766	4.17E-05	IL1B/IL6/BCL2/TLR4/IL2	5
BP	GO:0033673	negative regulation of kinase activity	6/41	237/18723	1.20E-05	0.036119123	4.22E-05	CASP3/IL1B/AKT1/CDKN2A/PPARG/FOXA2	6
BP	GO:0051251	positive regulation of lymphocyte activation	7/41	362/18723	1.22E-05	0.036666544	4.27E-05	IL1B/IL6/AKT1/BCL2/TGFB1/TLR4/IL2	7
BP	GO:0032946	positive regulation of mononuclear cell proliferation	5/41	138/18723	1.22E-05	0.036909991	4.28E-05	IL1B/IL6/BCL2/TLR4/IL2	5
BP	GO:0051403	stress-activated MAPK cascade	6/41	239/18723	1.26E-05	0.037885331	4.36E-05	TNF/IL1B/AKT1/XDH/TLR4/MAPK1	6
BP	GO:0006925	inflammatory cell apoptotic process	3/41	21/18723	1.26E-05	0.038013881	4.36E-05	IL6/CDKN2A/PIK3CD	3
BP	GO:0007252	I-kappaB phosphorylation	3/41	21/18723	1.26E-05	0.038013881	4.36E-05	TNF/AKT1/TLR4	3
BP	GO:0014015	positive regulation of gliogenesis	4/41	66/18723	1.29E-05	0.038963365	4.44E-05	TNF/IL1B/IL6/TGFB1	4

BP	GO:0019229	regulation of vasoconstriction	4/41	66/18723	1.29E-05	0.038963365	4.44E-05	PTGS2/AKT1/ADRA2A/ADRA2C	4
BP	GO:0044843	cell cycle G1/S phase transition	6/41	241/18723	1.32E-05	0.039720852	4.51E-05	AKT1/BCL2/CDKN2A/TP53/CCND1/ATM	6
BP	GO:0051271	negative regulation of cellular component movement	7/41	367/18723	1.33E-05	0.040071914	4.54E-05	TNF/AKT1/BCL2/PPARG/TGFB1/HMOX1/ADORA1	7
BP	GO:0030183	B cell differentiation	5/41	141/18723	1.36E-05	0.040967456	4.60E-05	IL6/BCL2/TP53/IL2/ATM	5
BP	GO:0035296	regulation of tube diameter	5/41	141/18723	1.36E-05	0.040967456	4.60E-05	PTGS2/AKT1/ADRA2A/ADRA2C/ADORA1	5
BP	GO:0097746	blood vessel diameter maintenance	5/41	141/18723	1.36E-05	0.040967456	4.60E-05	PTGS2/AKT1/ADRA2A/ADRA2C/ADORA1	5
BP	GO:0050900	leukocyte migration	7/41	369/18723	1.38E-05	0.041505583	4.64E-05	TNF/IL6/AKT1/HMOX1/MAPK1/ADORA1/PIK3CD	7
BP	GO:0035150	regulation of tube size	5/41	142/18723	1.41E-05	0.042394577	4.71E-05	PTGS2/AKT1/ADRA2A/ADRA2C/ADORA1	5
BP	GO:2000045	regulation of G1/S transition of mitotic cell cycle	5/41	142/18723	1.41E-05	0.042394577	4.71E-05	AKT1/BCL2/TP53/CCND1/ATM	5
BP	GO:0046686	response to cadmium ion	4/41	68/18723	1.46E-05	0.043888123	4.84E-05	AKT1/HMOX1/CYP1A2/MAPK1	4
BP	GO:0051926	negative regulation of calcium ion	4/41	68/18723	1.46E-05	0.043888123	4.84E-05	PTGS2/AKT1/ADRA2A/BCL2	4

		transport							
BP	GO:0030098	lymphocyte differentiation	7/41	374/18723	1.50E-05	0.045276968	4.98E-05	IL1B/IL6/BCL2/TP53/IL2/ATM/PIK3CD	7
BP	GO:0031098	stress-activated protein kinase signaling cascade	6/41	247/18723	1.52E-05	0.045663927	5.01E-05	TNF/IL1B/AKT1/XDH/TLR4/MAPK1	6
BP	GO:0050805	negative regulation of synaptic transmission	4/41	69/18723	1.54E-05	0.046515367	5.09E-05	PTGS2/IL1B/ACHE/ADORA1	4
BP	GO:0044282	small molecule catabolic process	7/41	376/18723	1.55E-05	0.046863002	5.11E-05	NOS2/AKT1/IDO1/XDH/TP53/GPT/ALDH2	7
CC	GO:0045211	postsynaptic membrane	7/41	277/19550	1.58E-06	0.000253183	0.000163236	GRIN2B/GRIN1/ADRA2C/ADORA1/GABRG2/GABRB2/GABRA1	7
CC	GO:1902711	GABA-A receptor complex	3/41	19/19550	8.10E-06	0.001296714	0.000245536	GABRG2/GABRB2/GABRA1	3
CC	GO:0099572	postsynaptic specialization	7/41	356/19550	8.26E-06	0.001321432	0.000245536	GRIN2B/GRIN1/MAPK1/ADRA2C/ADORA1/GABRB2/GABRA1	7
CC	GO:1902710	GABA receptor complex	3/41	20/19550	9.52E-06	0.001523323	0.000245536	GABRG2/GABRB2/GABRA1	3
CC	GO:0097060	synaptic membrane	7/41	384/19550	1.35E-05	0.002159961	0.000278521	GRIN2B/GRIN1/ADRA2C/ADORA1/GABRG2/GABRB2/GABRA1	7
CC	GO:0099055	integral component of postsynaptic membrane	4/41	117/19550	0.000103989	0.016638316	0.001532476	ADRA2C/ADORA1/GABRB2/GABRA1	4
CC	GO:0099634	postsynaptic specialization	4/41	117/19550	0.000103989	0.016638316	0.001532476	GRIN2B/ADRA2C/GABRB2/GABRA1	4

		membrane							
CC	GO:0098936	intrinsic component of postsynaptic membrane	4/41	122/19550	0.000122274	0.019563825	0.00157669	ADRA2C/ADORA1/GABRB2/GABRA1	4
CC	GO:0017146	NMDA selective glutamate receptor complex	2/41	9/19550	0.000153049	0.024487837	0.001616404	GRIN2B/GRIN1	2
CC	GO:0034707	chloride channel complex	3/41	50/19550	0.000156692	0.025070753	0.001616404	GABRG2/GABRB2/GABRA1	3
CC	GO:0099699	integral component of synaptic membrane	4/41	150/19550	0.00027036	0.043257624	0.002357713	ADRA2C/ADORA1/GABRB2/GABRA1	4
CC	GO:0034702	ion channel complex	5/41	282/19550	0.000295271	0.047243401	0.002357713	GRIN2B/GRIN1/GABRG2/GABRB2/GABRA1	5
CC	GO:0032589	neuron projection membrane	3/41	62/19550	0.00029712	0.047539191	0.002357713	ADORA1/GABRG2/GABRA1	3
MF	GO:0030594	neurotransmitter receptor activity	6/41	111/18368	1.61E-07	4.67E-05	8.82E-06	GRIN2B/GRIN1/ADORA1/GABRG2/GABRB2/GABRA1	6
MF	GO:0022824	transmitter-gated ion channel activity	5/41	60/18368	2.15E-07	6.23E-05	8.82E-06	GRIN2B/GRIN1/GABRG2/GABRB2/GABRA1	5
MF	GO:0022835	transmitter-gated channel activity	5/41	60/18368	2.15E-07	6.23E-05	8.82E-06	GRIN2B/GRIN1/GABRG2/GABRB2/GABRA1	5
MF	GO:0005230	extracellular ligand-gated ion channel activity	5/41	73/18368	5.78E-07	0.000167731	1.78E-05	GRIN2B/GRIN1/GABRG2/GABRB2/GABRA1	5
MF	GO:00	GABA-gated	3/41	13/18368	2.91E-06	0.0008429	7.16E-05	GABRG2/GABRB2/GABRA1	3

	22851	chloride ion channel activity				75			
MF	GO:0005237	inhibitory extracellular ligand-gated ion channel activity	3/41	15/18368	4.61E-06	0.00133694	9.46E-05	GABRG2/GABRB2/GABRA1	3
MF	GO:0099095	ligand-gated anion channel activity	3/41	18/18368	8.23E-06	0.002386541	0.000144789	GABRG2/GABRB2/GABRA1	3
MF	GO:0004890	GABA-A receptor activity	3/41	19/18368	9.76E-06	0.002829623	0.000150211	GABRG2/GABRB2/GABRA1	3
MF	GO:0002020	protease binding	5/41	135/18368	1.21E-05	0.00349838	0.000151063	TNF/CASP3/BCL2/COL1A2/TP53	5
MF	GO:0020037	heme binding	5/41	139/18368	1.39E-05	0.004030685	0.000151063	PTGS2/NOS2/IDO1/HMOX1/CYP1A2	5
MF	GO:0016917	GABA receptor activity	3/41	22/18368	1.54E-05	0.004476145	0.000151063	GABRG2/GABRB2/GABRA1	3
MF	GO:0015276	ligand-gated ion channel activity	5/41	143/18368	1.59E-05	0.004624218	0.000151063	GRIN2B/GRIN1/GABRG2/GABRB2/GABRA1	5
MF	GO:0022834	ligand-gated channel activity	5/41	143/18368	1.59E-05	0.004624218	0.000151063	GRIN2B/GRIN1/GABRG2/GABRB2/GABRA1	5
MF	GO:0046906	tetrapyrrole binding	5/41	149/18368	1.94E-05	0.005639993	0.000171086	PTGS2/NOS2/IDO1/HMOX1/CYP1A2	5
MF	GO:0005126	cytokine receptor binding	6/41	271/18368	2.84E-05	0.008248377	0.000233529	TNF/CASP3/IL1B/IL6/TGFB1/IL2	6
MF	GO:0001540	amyloid-beta binding	4/41	84/18368	3.62E-05	0.010505904	0.000278855	GRIN2B/GRIN1/ACHE/TLR4	4

MF	GO:0031406	carboxylic acid binding	5/41	176/18368	4.32E-05	0.012540071	0.000313267	NOS2/GRIN2B/GRIN1/HNF4A/PPARG	5
MF	GO:0022849	glutamate-gated calcium ion channel activity	2/41	5/18368	4.84E-05	0.0140377 1	0.000317278	GRIN2B/GRIN1	2
MF	GO:0051721	protein phosphatase 2A binding	3/41	32/18368	4.89E-05	0.014194819	0.000317278	AKT1/BCL2/TP53	3
MF	GO:0004936	alpha-adrenergic receptor activity	2/41	6/18368	7.25E-05	0.021026768	0.000425224	ADRA2A/ADRA2C	2
MF	GO:0051379	epinephrine binding	2/41	6/18368	7.25E-05	0.021026768	0.000425224	ADRA2A/ADRA2C	2
MF	GO:0046982	protein heterodimerization activity	6/41	328/18368	8.22E-05	0.023845901	0.000460315	ADRA2A/BCL2/TP53/TLR4/ADRA2C/ADORA1	6
MF	GO:1904315	transmitter-gated ion channel activity involved in regulation of postsynaptic membrane potential	3/41	44/18368	0.00012829	0.037204075	0.000686953	GABRG2/GABRB2/GABRA1	3
MF	GO:0004972	NMDA glutamate receptor activity	2/41	8/18368	0.000134962	0.039138984	0.000692568	GRIN2B/GRIN1	2
MF	GO:0099529	neurotransmitter receptor activity	3/41	47/18368	0.000156341	0.0453388	0.000711348	GABRG2/GABRB2/GABRA1	3

		involved in regulation of postsynaptic membrane potential							
MF	GO:0019207	kinase regulator activity	5/41	233/18368	0.000162477	0.047118189	0.000711348	CASP3/CDKN2A/EGF/CCND1/IL2	5
MF	GO:0005125	cytokine activity	5/41	235/18368	0.00016908	0.049033138	0.000711348	TNF/IL1B/IL6/TGFB1/IL2	5

KEGG for Common Targets

ID	Description	GeneRatio	BgRatio	pvalue	p.adjust	qvalue	geneID	Count
hsa04933	AGE-RAGE signaling pathway in diabetic complications	11/41	100/8142	1.27E-12	2.94E-10	9.66E-11	7124/836/3553/3569/207/596/1278/7040/595/5594/5293	11
hsa05210	Colorectal cancer	10/41	86/8142	8.68E-12	2.01E-09	3.29E-10	836/207/596/7157/1950/7040/595/5594/842/5293	10
hsa05162	Measles	11/41	139/8142	4.92E-11	1.14E-08	9.36E-10	836/3553/3569/207/596/7157/595/7099/842/3558/5293	11
hsa05142	Chagas disease	10/41	102/8142	4.94E-11	1.14E-08	9.36E-10	7124/3553/3569/4843/207/7040/7099/5594/3558/5293	10
hsa01524	Platinum drug resistance	9/41	73/8142	6.28E-11	1.45E-08	9.52E-10	836/207/596/1029/7157/5594/842/472/5293	9
hsa05212	Pancreatic cancer	9/41	76/8142	9.12E-11	2.11E-08	1.15E-09	207/1029/7157/1950/7040/595/5594/842/5293	9

hsa05161	Hepatitis B	11/41	162/8142	2.60E-10	6.00E-08	2.81E-09	7124/836/3569/207/596/7157/7040/7099/5594/842/5293	11
hsa05417	Lipid and atherosclerosis	12/41	215/8142	3.41E-10	7.87E-08	3.23E-09	7124/836/3553/3569/207/596/5468/7157/7099/5594/842/5293	12
hsa05222	Small cell lung cancer	9/41	92/8142	5.25E-10	1.21E-07	4.37E-09	5743/836/4843/207/596/7157/595/842/5293	9
hsa05163	Human cytomegalovirus infection	12/41	225/8142	5.76E-10	1.33E-07	4.37E-09	7124/5743/836/3553/3569/207/1029/7157/595/5594/842/5293	12
hsa05152	Tuberculosis	11/41	180/8142	8.07E-10	1.86E-07	5.56E-09	7124/836/3553/3569/4843/207/596/7040/7099/5594/842	11
hsa05146	Amoebiasis	9/41	102/8142	1.34E-09	3.09E-07	8.44E-09	7124/836/3553/3569/4843/1278/7040/7099/5293	9
hsa05223	Non-small cell lung cancer	8/41	72/8142	1.90E-09	4.39E-07	1.11E-08	207/1029/7157/1950/595/5594/842/5293	8
hsa04066	HIF-1 signaling pathway	9/41	109/8142	2.43E-09	5.60E-07	1.31E-08	3569/4843/207/596/1950/3162/7099/5594/5293	9
hsa05145	Toxoplasmosis	9/41	112/8142	3.09E-09	7.14E-07	1.56E-08	7124/836/4843/207/596/7040/7099/5594/842	9
hsa05166	Human T-cell leukemia virus 1 infection	11/41	222/8142	7.44E-09	1.72E-06	3.53E-08	7124/3569/207/1029/7157/7040/595/5594/3558/472/5293	11
hsa05213	Endometrial cancer	7/41	58/8142	1.19E-08	2.76E-06	5.32E-08	207/7157/1950/595/5594/842/5293	7
hsa04210	Apoptosis	9/41	136/8142	1.73E-08	3.99E-06	7.28E-08	7124/836/207/596/7157/5594/842/472/5293	9
hsa05135	Yersinia infection	9/41	137/8142	1.84E-08	4.26E-06	7.35E-08	7124/3553/3569/207/7099/5594/3558/6197/5293	9
hsa05215	Prostate cancer	8/41	97/8142	2.10E-08	4.84E-06	7.94E-08	207/596/7157/1950/595/5594/842/5293	8
hsa04625	C-type lectin receptor signaling pathway	8/41	104/8142	3.64E-08	8.41E-06	1.31E-07	7124/5743/3553/3569/207/5594/3558/5293	8
hsa05205	Proteoglycans in cancer	10/41	205/8142	4.67E-08	1.08E-05	1.61E-07	7124/836/207/1278/7157/7040/595/7099/5594/5293	10
hsa05218	Melanoma	7/41	72/8142	5.56E-08	1.28E-05	1.79E-07	207/1029/7157/1950/595/5594/5293	7
hsa04218	Cellular senescence	9/41	156/8142	5.73E-08	1.32E-05	1.79E-07	3569/207/1029/7157/7040/595/5594/472/5293	9
hsa05160	Hepatitis C	9/41	157/8142	6.06E-08	1.40E-05	1.79E-07	7124/836/207/7157/1950/595/5594/842/5293	9

hsa04115	p53 signaling pathway	7/41	73/8142	6.12E-08	1.41E-05	1.79E-07	836/596/1029/7157/595/842/472	7
hsa04668	TNF signaling pathway	8/41	112/8142	6.53E-08	1.51E-05	1.83E-07	7124/5743/836/3553/3569/207/5594/5293	8
hsa05214	Glioma	7/41	75/8142	7.41E-08	1.71E-05	2.00E-07	207/1029/7157/1950/595/5594/5293	7
hsa05133	Pertussis	7/41	76/8142	8.13E-08	1.88E-05	2.05E-07	7124/836/3553/3569/4843/7099/5594	7
hsa05220	Chronic myeloid leukemia	7/41	76/8142	8.13E-08	1.88E-05	2.05E-07	207/1029/7157/7040/595/5594/5293	7
hsa05140	Leishmaniasis	7/41	77/8142	8.91E-08	2.06E-05	2.18E-07	7124/5743/3553/4843/7040/7099/5594	7
hsa04151	PI3K-Akt signaling pathway	12/41	354/8142	9.56E-08	2.21E-05	2.27E-07	3569/207/596/1278/7157/1950/595/7099/5594/842/3558/5293	12
hsa05164	Influenza A	9/41	171/8142	1.27E-07	2.93E-05	2.91E-07	7124/836/3553/3569/207/7099/5594/842/5293	9
hsa04068	FoxO signaling pathway	8/41	131/8142	2.22E-07	5.12E-05	4.94E-07	3569/207/1950/7040/595/5594/472/5293	8
hsa05010	Alzheimer disease	12/41	384/8142	2.33E-07	5.37E-05	5.04E-07	7124/5743/836/3553/3569/4843/207/2904/2902/5594/842/5293	12
hsa05134	Legionellosis	6/41	57/8142	3.35E-07	7.73E-05	7.04E-07	7124/836/3553/3569/7099/842	6
hsa05167	Kaposi sarcoma-associated herpesvirus infection	9/41	194/8142	3.73E-07	8.61E-05	7.63E-07	5743/836/3569/207/7157/595/5594/842/5293	9
hsa04936	Alcoholic liver disease	8/41	142/8142	4.13E-07	9.54E-05	8.24E-07	7124/836/3553/3569/207/595/7099/217	8
hsa05165	Human papillomavirus infection	11/41	331/8142	4.49E-07	0.000103686	8.72E-07	7124/5743/836/207/1278/7157/1950/595/5594/472/5293	11
hsa01522	Endocrine resistance	7/41	98/8142	4.74E-07	0.000109578	8.99E-07	207/596/1029/7157/595/5594/5293	7
hsa05169	Epstein-Barr virus infection	9/41	202/8142	5.25E-07	0.000121234	9.70E-07	7124/836/3569/207/596/7157/595/842/5293	9
hsa05226	Gastric cancer	8/41	149/8142	5.97E-07	0.000137953	1.08E-06	207/596/7157/1950/7040/595/5594/5293	8
hsa04620	Toll-like receptor signaling pathway	7/41	104/8142	7.12E-07	0.000164587	1.26E-06	7124/3553/3569/207/7099/5594/5293	7

hsa05321	Inflammatory bowel disease	6/41	65/8142	7.39E-07	0.000170789	1.27E-06	7124/3553/3569/7040/7099/3558	6
hsa05170	Human immunodeficiency virus 1 infection	9/41	212/8142	7.89E-07	0.000182225	1.33E-06	7124/836/207/596/7099/5594/842/472/5293	9
hsa04932	Non-alcoholic fatty liver disease	8/41	155/8142	8.07E-07	0.000186493	1.33E-06	7124/836/3553/3569/207/5468/7040/5293	8
hsa05033	Nicotine addiction	5/41	40/8142	1.45E-06	0.000336102	2.35E-06	2904/2902/2566/2561/2554	5
hsa05225	Hepatocellular carcinoma	8/41	168/8142	1.49E-06	0.00034368	2.35E-06	207/1029/7157/7040/3162/595/5594/5293	8
hsa05219	Bladder cancer	5/41	41/8142	1.65E-06	0.000381372	2.55E-06	1029/7157/1950/595/5594	5
hsa04071	Sphingolipid signaling pathway	7/41	119/8142	1.78E-06	0.000410684	2.69E-06	7124/207/596/7157/5594/134/5293	7
hsa05206	MicroRNAs in cancer	10/41	310/8142	2.17E-06	0.000501591	3.23E-06	5743/836/596/1029/7157/3162/595/5594/472/5293	10
hsa01521	EGFR tyrosine kinase inhibitor resistance	6/41	79/8142	2.36E-06	0.000545721	3.44E-06	3569/207/596/1950/5594/5293	6
hsa05131	Shigellosis	9/41	247/8142	2.82E-06	0.000651292	4.03E-06	7124/3553/207/596/7157/7099/5594/472/5293	9
hsa04380	Osteoclast differentiation	7/41	128/8142	2.90E-06	0.0006704	4.07E-06	7124/3553/207/5468/7040/5594/5293	7
hsa05132	Salmonella infection	9/41	249/8142	3.01E-06	0.000696117	4.15E-06	7124/836/3553/3569/207/596/7099/5594/5293	9
hsa05144	Malaria	5/41	50/8142	4.52E-06	0.001042996	6.11E-06	7124/3553/3569/7040/7099	5

hsa05418	Fluid shear stress and atherosclerosis	7/41	139/8142	5.03E-06	0.001161953	6.69E-06	7124/3553/207/596/7157/3162/5293	7
hsa05020	Prion disease	9/41	273/8142	6.41E-06	0.001480253	8.37E-06	7124/836/3553/3569/2904/2902/5594/842/5293	9
hsa04657	IL-17 signaling pathway	6/41	94/8142	6.55E-06	0.00151219	8.41E-06	7124/5743/836/3553/3569/5594	6
hsa05207	Chemical carcinogenesis - receptor activation	8/41	212/8142	8.44E-06	0.001949764	1.07E-05	207/596/1950/595/1544/5594/6197/5293	8
hsa04370	VEGF signaling pathway	5/41	59/8142	1.03E-05	0.002383911	1.28E-05	5743/207/5594/842/5293	5
hsa04010	MAPK signaling pathway	9/41	294/8142	1.17E-05	0.002698793	1.41E-05	7124/836/3553/207/7157/1950/7040/5594/6197	9
hsa04064	NF-kappa B signaling pathway	6/41	104/8142	1.18E-05	0.002715675	1.41E-05	7124/5743/3553/596/7099/472	6
hsa04630	JAK-STAT signaling pathway	7/41	162/8142	1.38E-05	0.003187423	1.63E-05	3569/207/596/1950/595/3558/5293	7
hsa05022	Pathways of neurodegeneration - multiple diseases	11/41	476/8142	1.55E-05	0.003571503	1.80E-05	7124/5743/836/3553/3569/4843/2904/2902/596/5594/842	11
hsa04722	Neurotrophin signaling pathway	6/41	119/8142	2.54E-05	0.005874816	2.92E-05	207/596/7157/5594/6197/5293	6
hsa04919	Thyroid hormone signaling pathway	6/41	121/8142	2.80E-05	0.006458624	3.16E-05	207/7157/595/5594/842/5293	6
hsa05143	African trypanosomiasis	4/41	37/8142	3.24E-05	0.007487894	3.56E-05	7124/3553/3569/3620	4
hsa05216	Thyroid cancer	4/41	37/8142	3.24E-05	0.007487894	3.56E-05	5468/7157/595/5594	4

hsa04926	Relaxin signaling pathway	6/41	129/8142	4.02E-05	0.009279662	4.35E-05	4843/207/1278/7040/5594/5293	6
hsa05130	Pathogenic Escherichia coli infection	7/41	197/8142	4.88E-05	0.011277536	5.21E-05	7124/836/3553/3569/7099/5594/842	7
hsa05332	Graft-versus-host disease	4/41	42/8142	5.39E-05	0.012461443	5.68E-05	7124/3553/3569/3558	4
hsa04510	Focal adhesion	7/41	201/8142	5.55E-05	0.012818706	5.76E-05	207/596/1278/1950/595/5594/5293	7
hsa05235	PD-L1 expression and PD-1 checkpoint pathway in cancer	5/41	89/8142	7.66E-05	0.017692301	7.84E-05	207/1950/7099/5594/5293	5
hsa05224	Breast cancer	6/41	147/8142	8.35E-05	0.019287562	8.44E-05	207/7157/1950/595/5594/5293	6
hsa05323	Rheumatoid arthritis	5/41	93/8142	9.45E-05	0.021827607	9.42E-05	7124/3553/3569/7040/7099	5
hsa05171	Coronavirus disease - COVID-19	7/41	232/8142	0.000136983	0.031643182	0.00013483	7124/3553/3569/7099/5594/3558/5293	7
hsa04660	T cell receptor signaling pathway	5/41	104/8142	0.000160548	0.037086659	0.000155998	7124/207/5594/3558/5293	5
hsa04923	Regulation of lipolysis in adipocytes	4/41	56/8142	0.000168235	0.038862321	0.000161398	5743/207/134/5293	4
hsa04659	Th17 cell differentiation	5/41	108/8142	0.000191751	0.044294438	0.000179416	3553/3569/7040/5594/3558	5
hsa04931	Insulin resistance	5/41	108/8142	0.000191751	0.044294438	0.000179416	7124/3569/207/6197/5293	5

GO for Common Topological

ONTOLOGY	ID	Description	Gene Ratio	BgRatio	pvalue	p.adjust	qvalue	geneID	Count
BP	GO:0009314	response to radiation	16/60	456/18723	6.61E-13	1.46E-09	8.53E-10	DDB1/XRCC6/PRKDC/HIF1A/H2AX/CUL4B/TP53BP1/AURKB/KAT5/PPP1CC/PPP1CA/XRCC5/HSPA5/PARP1/NPM1/RUVBL2	16
BP	GO:0035966	response to topologically incorrect protein	11/60	159/18723	2.80E-12	6.19E-09	1.81E-09	DAXX/HDAC6/HSPD1/HSPA9/HSPA8/HSPB1/HSPA5/HSPA4/HSPA1B/HSPA1A/RACK1	11
BP	GO:0071478	cellular response to radiation	11/60	186/18723	1.55E-11	3.43E-08	5.62E-09	DDB1/XRCC6/H2AX/CUL4B/TP53BP1/AURKB/XRCC5/HSPA5/PARP1/NPM1/RUVBL2	11
BP	GO:0006986	response to unfolded protein	10/60	137/18723	1.74E-11	3.86E-08	5.62E-09	DAXX/HSPD1/HSPA9/HSPA8/HSPB1/HSPA5/HSPA4/HSPA1B/HSPA1A/RACK1	10
BP	GO:0042026	protein refolding	6/60	23/18723	8.11E-11	1.79E-07	2.09E-08	HSPD1/HSPA9/HSPA8/HSPA5/HSPA1B/HSPA1A	6
BP	GO:0035967	cellular response to topologically incorrect protein	9/60	116/18723	1.11E-10	2.46E-07	2.40E-08	DAXX/HDAC6/HSPD1/HSPA9/HSPA8/HSPA5/HSPA1B/HSPA1A/RACK1	9
BP	GO:0071214	cellular response to abiotic stimulus	12/60	331/18723	5.00E-10	1.11E-06	8.07E-08	DDB1/XRCC6/MAP3K1/H2AX/CUL4B/TP53BP1/AURKB/XRCC5/HSPA5/PARP1/NPM1/RUVBL2	12

BP	GO:0104004	cellular response to environmental stimulus	12/60	331/18723	5.00E-10	1.11E-06	8.07E-08	DDB1/XRCC6/MAP3K1/H2AX/CUL4B/TP53BP1/AURKB/XRCC5/HSPA5/PARP1/NPM1/RUVBL2	12
BP	GO:0034620	cellular response to unfolded protein	8/60	96/18723	7.30E-10	1.61E-06	1.05E-07	DAXX/HSPD1/HSPA9/HSPA8/HSPA5/HSPA1B/HSPA1A/RACK1	8
BP	GO:2001020	regulation of response to DNA damage stimulus	10/60	219/18723	1.77E-09	3.91E-06	2.28E-07	PRKDC/FUS/H2AX/CUL4A/TP53BP1/TRIM28/HNRNPK/PARP1/NPM1/DDX5	10
BP	GO:0031647	regulation of protein stability	11/60	298/18723	2.37E-09	5.24E-06	2.78E-07	RPL23/PRKDC/HDAC6/LMNA/GAPDH/HSPD1/FLNA/HSPA8/HSPA1B/HSPA1A/NPM1	11
BP	GO:0006458	'de novo' protein folding	6/60	43/18723	4.66E-09	1.03E-05	5.01E-07	HSPD1/HSPA9/HSPA8/HSPA5/HSPA1B/HSPA1A	6
BP	GO:0045646	regulation of erythrocyte differentiation	6/60	47/18723	8.12E-09	1.80E-05	8.06E-07	PRKDC/HIF1A/HSPA9/HSPA1B/HSPA1A/PRMT1	6
BP	GO:0051054	positive regulation of DNA	9/60	201/18723	1.46E-08	3.23E-05	1.34E-06	PRKDC/FUS/H2AX/TP53BP1/AURKB/XRCC5/TRIM28/HNRNPD/PARP1	9

		metabolic process							
BP	GO:0051052	regulation of DNA metabolic process	11/60	359/18723	1.64E-08	3.62E-05	1.41E-06	PRKDC/FUS/H2AX/CUL4A/TP53BP1/AURKB/XRCC5/TRIM28/HNRNPD/PARP1/NPM1	11
BP	GO:0010212	response to ionizing radiation	8/60	148/18723	2.28E-08	5.04E-05	1.72E-06	XRCC6/PRKDC/H2AX/TP53BP1/KAT5/XRCC5/HSPA5/PARP1	8
BP	GO:0043484	regulation of RNA splicing	8/60	148/18723	2.28E-08	5.04E-05	1.72E-06	FUS/NCL/HNRNPK/HSPA8/HSPA1A/C1QBP/NPM1/DDX5	8
BP	GO:0010332	response to gamma radiation	6/60	56/18723	2.40E-08	5.31E-05	1.72E-06	XRCC6/PRKDC/H2AX/XRCC5/HSPA5/PARP1	6
BP	GO:0071103	DNA conformation change	10/60	290/18723	2.59E-08	5.73E-05	1.76E-06	XRCC6/SET/DAXX/H2AX/RBBP4/TOP1/XRCC5/MCM7/NPM1/RUVBL2	10
BP	GO:0048511	rhythmic process	10/60	298/18723	3.35E-08	7.41E-05	2.16E-06	DDB1/PRKDC/CUL4A/TOP1/PPP1CC/PPP1CA/HNRNPD/HSPA5/RACK1/DDX5	10
BP	GO:0006310	DNA recombination	10/60	305/18723	4.17E-08	9.22E-05	2.56E-06	XRCC6/PRKDC/FUS/H2AX/TP53BP1/XRCC5/HSPD1/PARP1/MCM7/RUVBL2	10
BP	GO:0045648	positive regulation of erythrocyte differentiatio	5/60	33/18723	6.32E-08	0.0001397	3.70E-06	PRKDC/HIF1A/HSPA1B/HSPA1A/PRMT1	5

BP	GO:0061077	n chaperone-mediated protein folding	6/60	67/18723	7.19E-08	0.000158952	3.97E-06	HSPA9/HSPA8/HSPB1/HSPA5/HSPA1B/HSPA1A	6
BP	GO:0051085	chaperone cofactor-dependent protein refolding	5/60	34/18723	7.39E-08	0.000163385	3.97E-06	HSPA9/HSPA8/HSPA5/HSPA1B/HSPA1A	5
BP	GO:0006302	double-strand break repair	9/60	251/18723	9.87E-08	0.000218405	5.10E-06	XRCC6/PRKDC/FUS/H2AX/TP53BP1/KAT5/XRCC5/PARP1/MCM7	9
BP	GO:0062197	cellular response to chemical stress	10/60	337/18723	1.06E-07	0.000234542	5.26E-06	XRCC6/HIF1A/HDAC6/CHUK/XRCC5/HSPB1/HSPA1B/HSPA1A/PARP1/RACK1	10
BP	GO:0006338	chromatin remodeling	9/60	255/18723	1.13E-07	0.000249903	5.40E-06	SET/DAXX/ACTB/H2AX/RBBP4/TOP1/SMARCA4/NPM1/RUVBL2	9
BP	GO:0051084	'de novo' posttranslational protein folding	5/60	39/18723	1.51E-07	0.000333955	6.96E-06	HSPA9/HSPA8/HSPA5/HSPA1B/HSPA1A	5
BP	GO:0006282	regulation of DNA repair	7/60	130/18723	1.88E-07	0.000416035	8.37E-06	PRKDC/FUS/H2AX/CUL4A/TP53BP1/TRIM28/PARP1	7
BP	GO:00	histone	11/60	463/18723	2.16E-07	0.000477	9.29E-06	DDB1/SET/HDAC6/CUL4B/LMNA/AURKB/KAT5/FBL/WDR5/PRMT1/	11

	16570	modification				649		RUVBL2	
BP	GO:0097193	intrinsic apoptotic signaling pathway	9/60	288/18723	3.17E-07	0.000700193	1.28E-05	PRKDC/HIF1A/CUL4A/HNRNPK/HSPB1/HSPA1A/PARP1/RACK1/DDX5	9
BP	GO:1903311	regulation of mRNA metabolic process	9/60	288/18723	3.17E-07	0.000700193	1.28E-05	FUS/NCL/HNRNPK/HNRNPD/HSPA8/YBX1/C1QBP/NPM1/DDX5	9
BP	GO:0006457	protein folding	8/60	212/18723	3.65E-07	0.000808414	1.43E-05	HSPD1/HSPA9/HSPA8/HSPB1/HSPA5/HSPA1B/HSPA1A/RUVBL2	8
BP	GO:0034644	cellular response to UV	6/60	90/18723	4.24E-07	0.000936812	1.61E-05	DDB1/CUL4B/AURKB/PARP1/NPM1/RUVBL2	6
BP	GO:1903312	negative regulation of mRNA metabolic process	6/60	92/18723	4.83E-07	0.001067616	1.78E-05	FUS/HNRNPK/HNRNPD/YBX1/C1QBP/NPM1	6
BP	GO:0006325	chromatin organization	10/60	409/18723	6.31E-07	0.001395554	2.26E-05	SET/DAXX/ACTB/H2AX/RBBP4/TOP1/SMARCA4/TRIM28/NPM1/RUVBL2	10
BP	GO:0043161	proteasome-mediated ubiquitin-dependent protein	10/60	412/18723	6.74E-07	0.001491545	2.35E-05	DDB1/CUL4A/CUL4B/PSMD4/KAT5/PSMC5/HSPA5/HSPA1B/HSPA1A/RACK1	10

		catabolic process							
BP	GO:0009416	response to light stimulus	9/60	320/18723	7.65E-07	0.001691291	2.60E-05	DDB1/HIF1A/CUL4B/AURKB/PPP1CC/PPP1CA/PARP1/NPM1/RUVBL2	9
BP	GO:0048024	regulation of mRNA splicing, via spliceosome	6/60	101/18723	8.39E-07	0.001855687	2.78E-05	NCL/HNRNPK/HSPA8/C1QBP/NPM1/DDX5	6
BP	GO:0070841	inclusion body assembly	4/60	24/18723	9.65E-07	0.002134503	3.11E-05	HDAC6/PSMC5/HSPA1B/HSPA1A	4
BP	GO:1903320	regulation of protein modification by small protein conjugation or removal	8/60	242/18723	9.95E-07	0.002199965	3.13E-05	RPL23/HIF1A/DAXX/RPS2/HSPA5/HSPA1B/HSPA1A/NPM1	8
BP	GO:2001022	positive regulation of response to DNA damage stimulus	6/60	105/18723	1.05E-06	0.002333254	3.24E-05	PRKDC/FUS/H2AX/TRIM28/PARP1/DDX5	6
BP	GO:0008380	RNA splicing	10/60	434/18723	1.08E-06	0.002390606	3.24E-05	FUS/NCL/HNRNPK/HSPA8/YBX1/HSPA1A/C1QBP/NPM1/PRMT1/DDX5	10

BP	GO:0042770	signal transduction in response to DNA damage	7/60	172/18723	1.25E-06	0.002770647	3.67E-05	PRKDC/H2AX/CUL4A/TP53BP1/KAT5/NPM1/DDX5	7
BP	GO:2001233	regulation of apoptotic signaling pathway	9/60	356/18723	1.85E-06	0.004085778	5.30E-05	HIF1A/LMNA/PPP1CA/HNRNPK/HSPB1/HSPA1B/HSPA1A/PARP1/RACK1	9
BP	GO:0030522	intracellular receptor signaling pathway	8/60	265/18723	1.96E-06	0.004340273	5.40E-05	DAXX/HDAC6/SMARCA4/HSPA1B/HSPA1A/PARP1/C1QBP/DDX5	8
BP	GO:0007051	spindle organization	7/60	184/18723	1.97E-06	0.004347193	5.40E-05	TUBG1/AURKB/TUBB/FLNA/CLTC/HSPA1B/HSPA1A	7
BP	GO:0070997	neuron death	9/60	361/18723	2.07E-06	0.004581786	5.57E-05	HIF1A/SET/DAXX/TBK1/GAPDH/HSPD1/HSPA5/PARP1/RACK1	9
BP	GO:0006417	regulation of translation	10/60	468/18723	2.13E-06	0.00471034	5.61E-05	PRKDC/NCL/PPP1CA/GAPDH/HNRNPD/HSPB1/YBX1/C1QBP/NPM1/RACK1	10
BP	GO:0007052	mitotic spindle organization	6/60	120/18723	2.31E-06	0.005102867	5.84E-05	TUBG1/AURKB/FLNA/CLTC/HSPA1B/HSPA1A	6
BP	GO:0030218	erythrocyte differentiation	6/60	120/18723	2.31E-06	0.005102867	5.84E-05	PRKDC/HIF1A/HSPA9/HSPA1B/HSPA1A/PRMT1	6
BP	GO:01	non-membra	9/60	367/18723	2.37E-06	0.005244	5.88E-05	PRKDC/AURKB/TUBB/XRCC5/FLNA/HSPA1B/HSPA1A/C1QBP/NP	9

	40694	ne-bounded organelle assembly				883		M1	
BP	GO:00 50821	protein stabilization	7/60	191/18723	2.52E-06	0.005572 206	6.13E-05	RPL23/GAPDH/HSPD1/FLNA/HSPA1B/HSPA1A/NPM1	7
BP	GO:00 71482	cellular response to light stimulus	6/60	123/18723	2.66E-06	0.005892 499	6.35E-05	DDB1/CUL4B/AURKB/PARP1/NPM1/RUVBL2	6
BP	GO:00 10038	response to metal ion	9/60	373/18723	2.71E-06	0.005989 186	6.35E-05	HIF1A/DAXX/FUS/CHUK/IQGAP1/PPP1CA/HNRNPD/HSPA5/PARP 1	9
BP	GO:00 71480	cellular response to gamma radiation	4/60	31/18723	2.81E-06	0.006215 551	6.48E-05	XRCC6/H2AX/XRCC5/HSPA5	4
BP	GO:00 10498	proteasomal protein catabolic process	10/60	490/18723	3.21E-06	0.007094 597	7.26E-05	DDB1/CUL4A/CUL4B/PSMD4/KAT5/PSMC5/HSPA5/HSPA1B/HSPA 1A/RACK1	10
BP	GO:00 71479	cellular response to ionizing radiation	5/60	72/18723	3.38E-06	0.007485 534	7.53E-05	XRCC6/H2AX/TP53BP1/XRCC5/HSPA5	5
BP	GO:00 34101	erythrocyte homeostasis	6/60	129/18723	3.51E-06	0.007771 379	7.68E-05	PRKDC/HIF1A/HSPA9/HSPA1B/HSPA1A/PRMT1	6
BP	GO:00 45739	positive regulation of	5/60	73/18723	3.62E-06	0.008016 298	7.70E-05	PRKDC/FUS/H2AX/TRIM28/PARP1	5

BP	GO:0034599	DNA repair cellular response to oxidative stress	8/60	288/18723	3.64E-06	0.008046796	7.70E-05	HIF1A/HDAC6/CHUK/HSPB1/HSPA1B/HSPA1A/PARP1/RACK1	8
BP	GO:0044403	biological process involved in symbiotic interaction	8/60	290/18723	3.83E-06	0.008468502	7.82E-05	DDB1/SMARCA4/GAPDH/TRIM28/HSPD1/HSPA8/HSPA1B/HSPA1A	8
BP	GO:0000723	telomere maintenance	6/60	131/18723	3.84E-06	0.008496347	7.82E-05	XRCC6/PRKDC/AURKB/XRCC5/HNRNPD/PARP1	6
BP	GO:1903201	regulation of oxidative stress-induced cell death	5/60	74/18723	3.88E-06	0.008576175	7.82E-05	HIF1A/HDAC6/HSPB1/PARP1/RACK1	5
BP	GO:0007569	cell aging	6/60	132/18723	4.01E-06	0.008878943	7.97E-05	PRKDC/H2AX/LMNA/KAT5/YBX1/NPM1	6
BP	GO:0017038	protein import	7/60	206/18723	4.15E-06	0.009188389	8.12E-05	RPL23/LMNA/TRIM28/HSPD1/FLNA/HSPA8/HSPA4	7
BP	GO:0031396	regulation of protein ubiquitination	7/60	210/18723	4.71E-06	0.010429187	8.95E-05	RPL23/DAXX/RPS2/HSPA5/HSPA1B/HSPA1A/NPM1	7
BP	GO:0045637	regulation of myeloid cell differentiatio	7/60	210/18723	4.71E-06	0.010429187	8.95E-05	PRKDC/HIF1A/CUL4A/HSPA9/HSPA1B/HSPA1A/PRMT1	7

		n							
BP	GO:0050684	regulation of mRNA processing	6/60	137/18723	4.98E-06	0.011008324	9.31E-05	NCL/HNRNPK/HSPA8/C1QBP/NPM1/DDX5	6
BP	GO:0072594	establishment of protein localization to organelle	9/60	422/18723	7.37E-06	0.016294673	0.000135707	RPL23/LMNA/TRIM28/HSPD1/FLNA/HSPA8/HSPA5/HSPA4/RUVBL2	9
BP	GO:1902850	microtubule cytoskeleton organization involved in mitosis	6/60	147/18723	7.47E-06	0.016515054	0.000135707	TUBG1/AURKB/FLNA/CLTC/HSPA1B/HSPA1A	6
BP	GO:0071241	cellular response to inorganic substance	7/60	226/18723	7.63E-06	0.016879221	0.000136773	DAXX/FUS/CHUK/IQGAP1/HNRNPD/HSPA5/PARP1	7
BP	GO:0009411	response to UV	6/60	149/18723	8.07E-06	0.017846588	0.000142631	DDB1/CUL4B/AURKB/PARP1/NPM1/RUVBL2	6
BP	GO:0006402	mRNA catabolic process	7/60	232/18723	9.05E-06	0.020026294	0.000157889	FUS/HNRNPD/YBX1/HSPA1B/HSPA1A/NPM1/DDX5	7
BP	GO:1900407	regulation of cellular response to oxidative	5/60	89/18723	9.63E-06	0.021301999	0.000165707	HIF1A/HDAC6/HSPB1/PARP1/RACK1	5

		stress							
BP	GO:0002262	myeloid cell homeostasis	6/60	157/18723	1.09E-05	0.024072713	0.000184796	PRKDC/HIF1A/HSPA9/HSPA1B/HSPA1A/PRMT1	6
BP	GO:0071481	cellular response to X-ray	3/60	14/18723	1.11E-05	0.024566599	0.000184952	XRCC6/TP53BP1/XRCC5	3
BP	GO:0006979	response to oxidative stress	9/60	446/18723	1.15E-05	0.025377075	0.000184952	HIF1A/HDAC6/CHUK/HSPD1/HSPB1/HSPA1B/HSPA1A/PARP1/RACK1	9
BP	GO:0071824	protein-DNA complex subunit organization	7/60	241/18723	1.16E-05	0.025650194	0.000184952	RPL23/SET/DAXX/H2AX/RBBP4/SMARCA4/NPM1	7
BP	GO:0032200	telomere organization	6/60	159/18723	1.17E-05	0.025875934	0.000184952	XRCC6/PRKDC/AURKB/XRCC5/HNRNPD/PARP1	6
BP	GO:0018209	peptidyl-serine modification	8/60	338/18723	1.17E-05	0.025955512	0.000184952	PRKDC/HDAC6/CHUK/TBK1/AURKB/TOP1/PARP1/RACK1	8
BP	GO:0030521	androgen receptor signaling pathway	4/60	44/18723	1.18E-05	0.025995128	0.000184952	DAXX/HDAC6/SMARCA4/DDX5	4
BP	GO:1901796	regulation of signal transduction by p53 class	5/60	93/18723	1.19E-05	0.026412313	0.000185656	RPL23/AURKB/HNRNPK/NPM1/DDX5	5

BP	GO:0051702	mediator biological process involved in interaction with symbiont	5/60	94/18723	1.26E-05	0.027828003	0.000193279	DDB1/SMARCA4/GAPDH/HSPD1/HSPA8	5
BP	GO:0036473	cell death in response to oxidative stress	5/60	95/18723	1.32E-05	0.029302246	0.000201124	HIF1A/HDAC6/HSPB1/PARP1/RACK1	5
BP	GO:0072331	signal transduction by p53 class mediator	6/60	163/18723	1.35E-05	0.02981066	0.000202234	RPL23/AURKB/KAT5/HNRNPK/NPM1/DDX5	6
BP	GO:2001242	regulation of intrinsic apoptotic signaling pathway	6/60	164/18723	1.40E-05	0.030866281	0.000206989	HIF1A/HNRNPK/HSPB1/HSPA1A/PARP1/RACK1	6
BP	GO:1902882	regulation of response to oxidative stress	5/60	98/18723	1.54E-05	0.034093479	0.000224295	HIF1A/HDAC6/HSPB1/PARP1/RACK1	5
BP	GO:0034728	nucleosome organization	6/60	167/18723	1.55E-05	0.034215886	0.000224295	SET/DAXX/H2AX/RBBP4/SMARCA4/NPM1	6

BP	GO:0045639	positive regulation of myeloid cell differentiation	5/60	103/18723	1.96E-05	0.043414482	0.000278711	PRKDC/HIF1A/HSPA1B/HSPA1A/PRMT1	5
BP	GO:0001961	positive regulation of cytokine-mediated signaling pathway	4/60	50/18723	1.97E-05	0.043472455	0.000278711	HIF1A/TBK1/HSPA1B/HSPA1A	4
BP	GO:0090083	regulation of inclusion body assembly	3/60	17/18723	2.06E-05	0.045580362	0.000289049	PSMC5/HSPA1B/HSPA1A	3
BP	GO:1903706	regulation of hemopoiesis	8/60	367/18723	2.12E-05	0.046969063	0.000294653	XRCC6/PRKDC/HIF1A/CUL4A/HSPA9/HSPA1B/HSPA1A/PRMT1	8
BP	GO:0006333	chromatin assembly or disassembly	6/60	177/18723	2.15E-05	0.047564309	0.000295213	SET/DAXX/H2AX/RBBP4/SMARCA4/NPM1	6
CC	GO:0005925	focal adhesion	16/60	418/19550	9.08E-14	2.87E-11	9.63E-12	RPL23/ACTB/IQGAP1/RPL31/PPP1CC/HNRNPK/FLNA/CLTC/HSPA9/HSPA8/HSPB1/RPS2/HSPA5/HSPA1B/HSPA1A/NPM1	16
CC	GO:0030055	cell-substrate junction	16/60	425/19550	1.17E-13	3.71E-11	9.63E-12	RPL23/ACTB/IQGAP1/RPL31/PPP1CC/HNRNPK/FLNA/CLTC/HSPA9/HSPA8/HSPB1/RPS2/HSPA5/HSPA1B/HSPA1A/NPM1	16
CC	GO:0000781	chromosome, telomeric	10/60	162/19550	6.09E-11	1.93E-08	3.34E-09	DDB1/XRCC6/PRKDC/H2AX/TP53BP1/PPP1CC/PPP1CA/XRCC5/PARP1/MCM7	10

		region							
CC	GO:0098687	chromosomal region	12/60	348/19550	5.45E-10	1.72E-07	2.24E-08	DDB1/XRCC6/PRKDC/DAXX/H2AX/TP53BP1/AURKB/PPP1CC/PPP1CA/XRCC5/PARP1/MCM7	12
CC	GO:0032993	protein-DNA complex	8/60	199/19550	1.63E-07	5.14E-05	5.35E-06	XRCC6/PRKDC/H2AX/TOP1/XRCC5/PARP1/MCM7/NPM1	8
CC	GO:0090734	site of DNA damage	6/60	97/19550	5.15E-07	0.000162609	1.41E-05	DDB1/H2AX/LMNA/TP53BP1/XRCC5/PARP1	6
CC	GO:0070419	nonhomologous end joining complex	3/60	9/19550	2.28E-06	0.00071998	5.34E-05	XRCC6/PRKDC/XRCC5	3
CC	GO:0035861	site of double-strand break	5/60	73/19550	2.94E-06	0.000929119	6.04E-05	DDB1/H2AX/LMNA/TP53BP1/PARP1	5
CC	GO:0000228	nuclear chromosome	7/60	227/19550	5.94E-06	0.00187678	9.99E-05	H2AX/TUBG1/RBBP4/KAT5/TOP1/MCM7/RUVBL2	7
CC	GO:1990391	DNA repair complex	4/60	39/19550	6.08E-06	0.001922353	9.99E-05	XRCC6/PRKDC/TP53BP1/XRCC5	4
CC	GO:0036464	cytoplasmic ribonucleoprotein granule	7/60	236/19550	7.66E-06	0.002420364	0.000114341	ACTB/IQGAP1/TOP1/TUBB/NCL/HNRNPK/YBX1	7
CC	GO:0043204	perikaryon	6/60	158/19550	8.85E-06	0.002795378	0.000121052	HDAC6/FUS/TOP1/PPP1CA/FLNA/RACK1	6
CC	GO:0035770	ribonucleoprotein granule	7/60	254/19550	1.24E-05	0.003904143	0.000156062	ACTB/IQGAP1/TOP1/TUBB/NCL/HNRNPK/YBX1	7
CC	GO:00	NuA4	3/60	21/19550	3.51E-05	0.011104	0.0003846	ACTB/KAT5/RUVBL2	3

	35267	histone acetyltransferase complex				431	97		
CC	GO:0043189	H4/H2A histone acetyltransferase complex	3/60	21/19550	3.51E-05	0.011104431	0.000384697	ACTB/KAT5/RUVBL2	3
CC	GO:0000118	histone deacetylase complex	4/60	72/19550	7.06E-05	0.022295857	0.000679433	HDAC6/RBBP4/KAT5/RUVBL2	4
CC	GO:0070603	SWI/SNF superfamily-type complex	4/60	72/19550	7.06E-05	0.022295857	0.000679433	RBBP4/KAT5/SMARCA4/RUVBL2	4
CC	GO:1904949	ATPase complex	4/60	73/19550	7.45E-05	0.023534509	0.000679433	RBBP4/KAT5/SMARCA4/RUVBL2	4
CC	GO:0000123	histone acetyltransferase complex	4/60	75/19550	8.28E-05	0.026161118	0.000681772	ACTB/KAT5/WDR5/RUVBL2	4
CC	GO:0072562	blood microparticle	5/60	146/19550	8.51E-05	0.026895816	0.000681772	ACTB/PSMC5/HSPA8/HSPA1B/HSPA1A	5
CC	GO:0016234	inclusion body	4/60	76/19550	8.72E-05	0.027551469	0.000681772	HDAC6/PSMC5/HSPA1B/HSPA1A	4
CC	GO:00	Cul4B-RING	2/60	5/19550	9.21E-05	0.029096	0.0006872	DDB1/CUL4B	2

	31465	E3 ubiquitin ligase complex				441	76		
CC	GO:19 02562	H4 histone acetyltransfe rase complex	3/60	32/19550	0.000127 94	0.040428 953	0.0008854 18	ACTB/KAT5/RUVBL2	3
CC	GO:00 31248	protein acetyltransfe rase complex	4/60	85/19550	0.000134 799	0.042596 544	0.0008854 18	ACTB/KAT5/WDR5/RUVBL2	4
CC	GO:19 02493	acetyltransfe rase complex	4/60	85/19550	0.000134 799	0.042596 544	0.0008854 18	ACTB/KAT5/WDR5/RUVBL2	4
CC	GO:00 80008	Cul4-RING E3 ubiquitin ligase complex	3/60	34/19550	0.000153 681	0.048563 131	0.0009377 3	DDB1/CUL4A/CUL4B	3
CC	GO:00 34708	methyltransf erase complex	4/60	88/19550	0.000154 184	0.048722 271	0.0009377 3	RBBP4/WDR5/PRMT1/RUVBL2	4
MF	GO:00 31625	ubiquitin protein ligase binding	16/60	297/18368	1.15E-15	3.53E-13	1.94E-13	RPL23/HIF1A/DAXX/HDAC6/NEDD8/CUL4A/CUL4B/TUBB/XRCC5/ TRIM28/HSPD1/HSPA9/HSPA8/HSPA5/HSPA1B/HSPA1A	16
MF	GO:00	ubiquitin-like	16/60	316/18368	3.05E-15	9.35E-13	2.57E-13	RPL23/HIF1A/DAXX/HDAC6/NEDD8/CUL4A/CUL4B/TUBB/XRCC5/	16

	44389	protein ligase binding						TRIM28/HSPD1/HSPA9/HSPA8/HSPA5/HSPA1B/HSPA1A	
MF	GO:0051787	misfolded protein binding	6/60	29/18368	4.21E-10	1.29E-07	2.36E-08	HDAC6/HSPA9/HSPA8/HSPA5/HSPA1B/HSPA1A	6
MF	GO:0044183	protein folding chaperone	6/60	40/18368	3.31E-09	1.02E-06	1.39E-07	HSPA9/HSPA8/HSPB1/HSPA5/HSPA1B/HSPA1A	6
MF	GO:0140297	DNA-binding transcription factor binding	12/60	394/18368	4.44E-09	1.36E-06	1.50E-07	HIF1A/DAXX/FUS/ACTB/TP53BP1/KAT5/PSMC5/SMARCA4/HSPB1/PARP1/NPM1/DDX5	12
MF	GO:0051082	unfolded protein binding	8/60	122/18368	5.76E-09	1.77E-06	1.62E-07	HSPD1/HSPA9/HSPA8/HSPA5/HSPA1B/HSPA1A/NPM1/RUVBL2	8
MF	GO:0031072	heat shock protein binding	8/60	129/18368	8.96E-09	2.75E-06	2.15E-07	HIF1A/DAXX/HDAC6/HSPA9/HSPA8/HSPA5/HSPA1B/HSPA1A	8
MF	GO:0061629	RNA polymerase II-specific DNA-binding transcription factor binding	10/60	299/18368	4.13E-08	1.27E-05	8.70E-07	HIF1A/DAXX/FUS/ACTB/TP53BP1/SMARCA4/HSPB1/PARP1/NPM1/DDX5	10

MF	GO:0047485	protein N-terminus binding	7/60	110/18368	6.77E-08	2.08E-05	1.27E-06	DAXX/PPP1CC/SMARCA4/HSPA1B/HSPA1A/PARP1/NPM1	7
MF	GO:0003684	damaged DNA binding	6/60	68/18368	8.79E-08	2.70E-05	1.39E-06	DDB1/XRCC6/H2AX/CUL4B/TP53BP1/XRCC5	6
MF	GO:0042393	histone binding	9/60	244/18368	9.11E-08	2.80E-05	1.39E-06	SET/DAXX/H2AX/RBBP4/TP53BP1/KAT5/WDR5/SMARCA4/NPM1	9
MF	GO:0042162	telomeric DNA binding	5/60	36/18368	1.09E-07	3.36E-05	1.54E-06	XRCC6/TP53BP1/NCL/XRCC5/HNRNPD	5
MF	GO:0042826	histone deacetylase binding	7/60	127/18368	1.82E-07	5.59E-05	2.36E-06	HIF1A/HDAC6/RBBP4/HNRNPD/HSPA1B/HSPA1A/PARP1	7
MF	GO:0016887	ATP hydrolysis activity	9/60	272/18368	2.29E-07	7.04E-05	2.76E-06	HSPD1/HSPA9/HSPA8/HSPA5/HSPA1B/HSPA1A/MCM7/RUVBL2/DDX5	9
MF	GO:0140545	protein disaggregase activity	3/60	5/18368	3.30E-07	0.000101258	3.70E-06	HSPA8/HSPA1B/HSPA1A	3
MF	GO:0055131	C3HC4-type RING finger domain binding	3/60	6/18368	6.58E-07	0.000202045	6.93E-06	HSPA8/HSPA1B/HSPA1A	3
MF	GO:0140030	modification-dependent protein binding	7/60	160/18368	8.75E-07	0.000268499	8.66E-06	DAXX/HDAC6/PSMD4/TP53BP1/WDR5/SMARCA4/HSPD1	7

MF	GO:0045296	cadherin binding	9/60	332/18368	1.21E-06	0.000372818	1.14E-05	IQGAP1/PPP1CA/HNRNPK/FLNA/HSPA8/RPS2/HSPA5/HSPA1A/RACK1	9
MF	GO:0008094	ATP-dependent activity, acting on DNA	6/60	111/18368	1.63E-06	0.000501009	1.45E-05	XRCC6/RBBP4/SMARCA4/XRCC5/MCM7/RUVBL2	6
MF	GO:0002039	p53 binding	5/60	66/18368	2.41E-06	0.000738914	2.03E-05	HIF1A/DAXX/TP53BP1/SMARCA4/HSPD1	5
MF	GO:0003712	transcription coregulator activity	10/60	489/18368	3.73E-06	0.001144539	2.99E-05	DAXX/FUS/TP53BP1/KAT5/SMARCA4/TRIM28/HSPA1A/C1QBP/NPM1/RUVBL2	10
MF	GO:0030957	Tat protein binding	3/60	10/18368	3.91E-06	0.00120103	2.99E-05	ACTB/SMARCA4/NPM1	3
MF	GO:0016922	nuclear receptor binding	6/60	144/18368	7.39E-06	0.00226881	5.41E-05	HIF1A/DAXX/FUS/SMARCA4/PARP1/DDX5	6
MF	GO:0043021	ribonucleoprotein complex binding	6/60	149/18368	8.99E-06	0.002759454	6.31E-05	PPP1CA/HSPA5/C1QBP/NPM1/RACK1/DDX5	6
MF	GO:0140097	catalytic activity, acting on DNA	7/60	229/18368	9.41E-06	0.002888458	6.34E-05	XRCC6/RBBP4/TOP1/SMARCA4/XRCC5/MCM7/RUVBL2	7
MF	GO:0004386	helicase activity	6/60	156/18368	1.17E-05	0.003587559	7.57E-05	XRCC6/SMARCA4/XRCC5/MCM7/RUVBL2/DDX5	6

MF	GO:00 05080	protein kinase C binding	4/60	55/18368	3.10E-05	0.009509 15	0.0001932 13	FLNA/HSPB1/C1QBP/RACK1	4
MF	GO:00 03714	transcription corepressor activity	6/60	187/18368	3.26E-05	0.010005 551	0.0001960 38	DAXX/SMARCA4/TRIM28/HSPA1A/C1QBP/RUVBL2	6
MF	GO:00 03678	DNA helicase activity	4/60	72/18368	8.96E-05	0.027521 576	0.0005206 35	XRCC6/XRCC5/MCM7/RUVBL2	4
MF	GO:00 50681	androgen receptor binding	3/60	28/18368	0.000102 422	0.031443 433	0.0005664 9	DAXX/SMARCA4/DDX5	3
MF	GO:00 51575	5'-deoxyribos e-5-phosphat e lyase activity	2/60	5/18368	0.000104 27	0.032010 78	0.0005664 9	XRCC6/XRCC5	2
MF	GO:00 01221	transcription coregulator binding	4/60	78/18368	0.000122 495	0.037606 02	0.0006447 11	RPL23/HIF1A/HDAC6/SMARCA4	4
MF	GO:00 19903	protein phosphatase binding	5/60	150/18368	0.000129 236	0.039675 531	0.0006595 79	IQGAP1/TBK1/PPP1CC/PPP1CA/RACK1	5

KEGG for Common Topological

ID	Description	Gene Ratio	BgRatio	pvalue	p.adjust	qvalue	geneID	Count
hsa05417	Lipid and atherosclerosis	8/50	215/8142	4.29E-05	0.006044484	0.003424359	1147/29110/3329/3312/3309/3308/3304/3303	8
hsa03450	Non-homologous end-joining	3/50	13/8142	5.97E-05	0.008416383	0.003424359	2547/5591/7520	3
hsa04612	Antigen processing and presentation	5/50	78/8142	0.000107243	0.015121332	0.004101593	3312/3309/3308/3304/3303	5
hsa05162	Measles	6/50	139/8142	0.000190408	0.026847468	0.004937667	1147/29110/3312/3304/3303/10399	6
hsa05205	Proteoglycans in cancer	7/50	205/8142	0.000230674	0.032525101	0.004937667	3091/60/8826/5501/5499/2316/1655	7
hsa03040	Spliceosome	6/50	147/8142	0.000258208	0.036407364	0.004937667	2521/3190/3312/3304/3303/1655	6
hsa05014	Amyotrophic lateral sclerosis	9/50	364/8142	0.00031782	0.044812684	0.004987656	1616/10013/2521/60/29110/5710/5705/203068/3309	9

Supplementary Table S9: Results of WGCNA

WGCNA AllGeneInfo in Module Salmon for GSE42546

genename	moduleColor	GS.group	p.GS.group	MM.salmon	p.MM.salmon
SPAG16	salmon	-0.436284572	0.002436978	0.648664287	1.09E-06
SLC26A11	salmon	-0.412969041	0.004339922	0.801779031	2.14E-11
NCDN	salmon	0.402480049	0.005554713	-0.618953202	4.54E-06
MOCS2	salmon	-0.372266514	0.010845225	0.781666618	1.44E-10
NOL6	salmon	-0.369891823	0.011401977	0.815692975	5.04E-12
ITPA	salmon	-0.368596003	0.011715984	0.840181033	2.86E-13
CDC40	salmon	-0.359640607	0.014095226	0.69627593	7.74E-08
IFT122	salmon	-0.355709638	0.015262914	0.814126792	5.97E-12
HIAT1	salmon	-0.350015466	0.017099519	0.842078755	2.24E-13
SLCO1C1	salmon	-0.348240973	0.017708786	0.467612107	0.001050311
RASD1	salmon	-0.345864661	0.018553366	0.822917757	2.26E-12
TBRG1	salmon	-0.334440173	0.023103925	0.875263173	1.79E-15
SSFA2	salmon	-0.330883372	0.024698652	0.690446268	1.10E-07
NRN1	salmon	-0.330535896	0.024859264	0.792351131	5.37E-11
TTC8	salmon	-0.32870385	0.025720591	0.914239673	7.10E-19
KDM5C	salmon	-0.328634598	0.025753632	0.763622586	6.74E-10
ISCU	salmon	-0.321130027	0.029550237	0.822395452	2.40E-12
STXBP1	salmon	0.317288095	0.03166698	-0.619063881	4.51E-06
C3orf20	salmon	-0.316364366	0.032194208	0.695458823	8.14E-08
NPEPPS	salmon	-0.312426326	0.034524009	0.955901931	4.82E-25
RMND5A	salmon	0.31139576	0.035156138	-0.574476331	2.98E-05
TXN	salmon	-0.30727757	0.037778085	0.636534475	1.98E-06
SAR1A	salmon	-0.305634433	0.038868081	0.549203087	7.73E-05
C8orf46	salmon	-0.303588844	0.040260949	0.651266758	9.52E-07
PHC3	salmon	-0.302480146	0.041032788	0.866820281	6.90E-15
ICAM5	salmon	-0.300808759	0.042219189	0.781534234	1.46E-10
C6orf62	salmon	-0.29942671	0.043221236	0.834661791	5.68E-13
POFUT1	salmon	-0.29653418	0.045381275	0.755261719	1.32E-09
UBA52	salmon	-0.29545068	0.046212668	0.639309382	1.73E-06
STX3	salmon	-0.294306433	0.047104071	0.594592421	1.32E-05
NHLRC2	salmon	-0.291428484	0.049407912	0.683104135	1.69E-07
ACBD7	salmon	-0.29079633	0.049925987	0.687184126	1.33E-07
ZNF382	salmon	-0.289204607	0.051249959	0.600864043	1.01E-05
MORC4	salmon	-0.288387918	0.051940201	0.737440025	5.05E-09
HAR1A	salmon	-0.284628183	0.055215265	0.750848151	1.86E-09
FAM164A	salmon	-0.279424001	0.060020587	0.730083726	8.53E-09
ATP1A2	salmon	-0.278685816	0.060728507	0.663317401	5.07E-07
TMEM120B	salmon	-0.27785229	0.06153587	0.548865053	7.83E-05
ANP32B	salmon	-0.272152448	0.067289232	0.75595834	1.25E-09
ZNF430	salmon	-0.27157157	0.067898786	0.696891457	7.46E-08
SRPR	salmon	-0.269022718	0.070625463	0.667955538	3.95E-07
CGREF1	salmon	-0.258179703	0.083206746	0.850506807	7.36E-14

TRUB1	salmon	-0.255668109	0.086357	0.733354661	6.77E-09
HIF1AN	salmon	-0.254175604	0.088272608	0.542188151	9.94E-05
SLITRK4	salmon	-0.250486387	0.093149588	0.731590292	7.67E-09
SPIN3	salmon	-0.25014841	0.093606611	0.689058233	1.19E-07
ZMYM3	salmon	-0.241706005	0.105595549	0.838047031	3.74E-13
UQCR10	salmon	-0.229908337	0.124275425	0.575533159	2.86E-05
SH3BGRL2	salmon	0.229486502	0.124986629	-0.688370452	1.24E-07
ZNF197	salmon	-0.225546573	0.131777875	0.635047896	2.13E-06
TSEN34	salmon	-0.224623298	0.133408513	0.584445764	2.00E-05
RCC2	salmon	-0.213486268	0.154281693	0.630367638	2.67E-06
ATG4A	salmon	-0.209589481	0.162123041	0.641886035	1.53E-06
C5orf32	salmon	-0.209445297	0.162418644	0.696066985	7.84E-08
GNAS	salmon	-0.208092344	0.165211589	0.526744643	0.000169511
NAALAD2	salmon	-0.201815677	0.178625915	0.639269596	1.74E-06
THOC3	salmon	-0.200102892	0.18241858	0.71445155	2.46E-08
GALC	salmon	-0.20003024	0.182580719	0.526371762	0.000171656
FAM134A	salmon	-0.192030457	0.201070275	0.671341733	3.28E-07
ARSK	salmon	-0.187285291	0.212640977	0.63261718	2.40E-06
GPATCH4	salmon	-0.185672669	0.216676852	0.549535649	7.64E-05
STK36	salmon	-0.18102831	0.228596476	0.590331883	1.57E-05
FAM131A	salmon	-0.160551756	0.286477485	0.673046607	2.98E-07
ZNF23	salmon	-0.160533542	0.28653288	0.50127235	0.00038731
TMEFF1	salmon	-0.142238743	0.34569776	0.664805774	4.68E-07
HNRNPA2B1	salmon	-0.138606829	0.358278289	0.638036057	1.84E-06
FBXW2	salmon	-0.130457736	0.387500776	0.755960529	1.25E-09
SEC63	salmon	-0.127079525	0.4000146	0.636399283	2.00E-06
VANGL2	salmon	-0.12640105	0.402555838	0.600253255	1.04E-05
POLR3F	salmon	-0.123450166	0.413716577	0.503403148	0.000362333
MMD	salmon	-0.122703532	0.416568239	0.515120275	0.000249157
LUC7L2	salmon	-0.118970184	0.430994127	0.677634083	2.31E-07
IDS	salmon	-0.117344256	0.437363171	0.525453756	0.000177041
CREBL2	salmon	-0.115424228	0.444951138	0.451536675	0.001633729
ELAVL1	salmon	-0.111311146	0.461447405	0.716019081	2.22E-08
CHUK	salmon	-0.109979128	0.466859518	0.609520132	6.93E-06
C12orf65	salmon	-0.101737988	0.501087379	0.512572525	0.000270606
RRP7A	salmon	-0.099870136	0.509019536	0.637116099	1.93E-06
AFG3L1	salmon	-0.099775227	0.509424272	0.494018016	0.000484418
BHLHE41	salmon	-0.093787048	0.535285497	0.630574685	2.64E-06
SPCS3	salmon	-0.093485175	0.536605905	0.533195592	0.000136057
NLGN4Y	salmon	-0.081132968	0.591950381	0.426682844	0.003105884
SENP5	salmon	-0.077069777	0.610692225	0.440717516	0.002173653
BTNL9	salmon	-0.065748183	0.664198383	0.64166017	1.54E-06
DTD1	salmon	-0.053624797	0.723378858	0.588272714	1.71E-05
CYCS	salmon	-0.051260697	0.735120906	0.546462796	8.53E-05

ZDHHC17	salmon	-0.048259097	0.750115532	0.587742105	1.75E-05
BET1	salmon	-0.046829003	0.757292208	0.345457675	0.018701374
DNAJC21	salmon	0.014772526	0.922376947	0.520546273	0.000208518

GO and KEGG for GSE42546

Color	Category	Term	Count	%	PValue	Genes
salmon	GOTERM_CC_DIRECT	GO:0005654~nucleoplasm	12	40	0.007682443	ENSG00000154144, ENSG00000126012, ENSG00000153561, ENSG00000164172, ENSG00000181045, ENSG00000221983, ENSG00000125877, ENSG00000165271, ENSG00000136854, ENSG00000173889, ENSG00000136810, ENSG00000168438
salmon	GOTERM_CC_DIRECT	GO:0098794~postsynapse	3	10	0.009803831	ENSG00000020129, ENSG00000136854, ENSG00000166900
salmon	GOTERM_CC_DIRECT	GO:0048787~presynaptic active zone membrane	2	6.666666667	0.015955537	ENSG00000136854, ENSG00000166900
salmon	REACTOME_PATHWAY	R-HSA-2559580~Oxidative Stress Induced Senescence	3	10	0.021727833	ENSG00000221983, ENSG00000173889, ENSG00000136810
salmon	GOTERM_BP_DIRECT	GO:0060271~cilium assembly	3	10	0.034438191	ENSG00000163913, ENSG00000144451, ENSG00000165533
salmon	GOTERM_CC_DIRECT	GO:0097730~non-motile cilium	2	6.666666667	0.039430049	ENSG00000163913, ENSG00000165533

WGCNA AllGeneInfo in Significant Modules for GSE160587

genename	module Color	GS. group	p.GS. group	MM. blue	p.MM. blue	MM. violet	p.MM. violet	MM. darkmagenta	p.MM. darkmagenta
Gm6741	blue	0.997050743	1.30E-05	0.881215396	0.020326661	-0.836358922	0.03797658	0.820544753	0.045416673
Rps3	blue	0.996366423	1.98E-05	0.869001616	0.024616861	-0.890062136	0.017465128	0.853889918	0.030462644
Sel1l	blue	-0.996284075	2.07E-05	-0.89717347	0.015316335	0.894809976	0.016015451	-0.839557041	0.036547858
Gsk3b	blue	0.996130451	2.24E-05	0.849327161	0.032343146	-0.866818951	0.025424659	0.840813482	0.0359936
Rps13-ps4	blue	0.99479488	4.06E-05	0.901605401	0.014045942	-0.858852716	0.028477826	0.854918659	0.030046014
Cox7a2	blue	0.994138702	5.14E-05	0.855237174	0.029917569	-0.892952864	0.016575303	0.891135017	0.017132266
Rbm33	blue	-0.993658395	6.02E-05	-0.882408873	0.019928504	0.806019962	0.052792818	-0.793927831	0.059323105
Setd3	blue	0.990178388	0.000144222	0.850644004	0.031794962	-0.808065722	0.051722839	0.784791818	0.064488206
Nutf2-ps1	blue	0.989067156	0.000178637	0.845835272	0.033818147	-0.801869688	0.054994567	0.799917215	0.056044712
Snrpd2	blue	0.988935633	0.000182953	0.881327164	0.020289214	-0.829952365	0.040915732	0.865239638	0.026016885
Pcgf1	blue	0.987302948	0.000240799	0.907830567	0.012351307	-0.872059801	0.023505935	0.776010635	0.069637942
Iqsec2	blue	-0.985820473	0.000300163	-0.865298376	0.025994742	0.86177617	0.0273383	-0.785606524	0.064019593
Mief1	blue	-0.985458206	0.000315658	-0.85982508	0.028096363	0.884421004	0.019265775	-0.799130327	0.056470531
Rps14	blue	0.984443207	0.000361138	0.837326576	0.03754158	-0.881024206	0.020390794	0.916524355	0.010161438
Gls	blue	-0.984075155	0.000378382	-0.827945762	0.04185736	0.874386943	0.022676958	-0.811544945	0.049926442
Ssna1	blue	0.984026317	0.0003807	0.849179852	0.032404744	-0.858611052	0.02857301	0.858766649	0.028511708
Arhgef2	blue	0.983849914	0.000389132	0.875577456	0.022258363	-0.825815618	0.042867904	0.82710627	0.042254272
Gm10182	blue	0.983149616	0.000423511	0.911540721	0.011391467	-0.850912422	0.031683766	0.749245101	0.086433543
Myl6	blue	0.982639596	0.000449459	0.886805204	0.018494407	-0.78867654	0.062267807	0.738835953	0.093403426
Zfp605	blue	0.982170291	0.000474014	0.824281872	0.043602479	-0.842410249	0.035294961	0.852250927	0.031132017
Scp2-ps2	blue	0.98211	0.0004770	0.9438	0.00463	-0.897995	0.0150767	0.827665482	0.041989682

		3607	23	79492	5891	445	19		
Smurf2	blue	-0.981066166	0.000534341	-0.844399919	0.034433431	0.913643344	0.010864207	-0.919590386	0.009438607
Kansl2	blue	0.980762407	0.000551568	0.903388298	0.013549853	-0.874640028	0.02258766	0.836604226	0.037866084
Sdc4	blue	0.980456708	0.000569178	0.837957063	0.037259415	-0.80479931	0.053436068	0.753617776	0.083578083
Pcdhac2	blue	-0.979343073	0.000635656	-0.826853898	0.042373936	0.822676962	0.044377365	-0.866243686	0.025639627
Ndufa12	blue	0.979047356	0.000653921	0.906541578	0.01269356	-0.775961105	0.0696675	0.742248519	0.091091773
Raver1	blue	0.978910513	0.00066246	0.815152526	0.048094894	-0.918154184	0.009773975	0.906079031	0.012817477
Gm10232	blue	0.978323275	0.000699728	0.905615987	0.01294211	-0.894961567	0.01597016	0.830120522	0.040837277
Srf	blue	-0.978219435	0.000706423	-0.787168249	0.063125674	0.803123487	0.05432504	-0.835919867	0.038174729
Rpl7a	blue	0.977986109	0.000721583	0.884575637	0.019215288	-0.857716351	0.028926715	0.842857159	0.035100576
Gm43843	blue	-0.977673381	0.000742152	-0.927237456	0.007748965	0.773781185	0.07097406	-0.741802159	0.091392663
Wdr7	blue	-0.977253751	0.000770203	-0.887694936	0.018210421	0.919462218	0.009468304	-0.830470399	0.040674264
Fam120a	blue	-0.97640847	0.000828275	-0.929651894	0.007249213	0.825796247	0.042877145	-0.774483041	0.070552189
B3galt5	blue	0.976246038	0.000839674	0.843984419	0.034612515	-0.932267528	0.006726164	0.889702092	0.017577521
Chmp4b	blue	0.976215314	0.000841839	0.915331268	0.010449705	-0.839253346	0.036682425	0.762620857	0.077835264
Gm25745	blue	0.975516969	0.00089179	0.847312359	0.033190431	-0.795666913	0.058362357	0.782061607	0.066069989
Ctnnd2	blue	-0.973937185	0.001010054	-0.889417318	0.017666663	0.885198094	0.019012702	-0.772255972	0.071894775
Rps3a2	blue	0.973822183	0.001018948	0.902486984	0.013799567	-0.909936715	0.011801823	0.810819972	0.050298335
Rpl17-ps10	blue	0.972768729	0.001102217	0.857781155	0.028901027	-0.942573141	0.004852074	0.910652664	0.011617792
Acot13	blue	0.97269251	0.001108367	0.831925321	0.039999668	-0.815165255	0.048088488	0.816660043	0.047338957
Tmem64	blue	-0.972334536	0.00113748	-0.914502393	0.010652274	0.756812414	0.081519222	-0.754880414	0.082761582
Golph3	blue	-0.97088529	0.00125916	-0.771332082	0.072455115	0.777477745	0.068765008	-0.839117722	0.036742594
Pfdn5	blue	0.97078	0.0012679	0.9564	0.00279	-0.840501	0.0361310	0.739519239	0.092938501

		3778	12	87319	8838	092	34		
Cops5	blue	0.970536125	0.001289391	0.953220568	0.003231289	-0.824374284	0.043558054	0.792648335	0.060034557
Mrgbp	blue	0.970437226	0.001298018	0.946809775	0.004168557	-0.893003398	0.016559946	0.798972471	0.056556132
Mtpn	blue	-0.970232564	0.001315962	-0.935528164	0.006100934	0.928729688	0.007438179	-0.795374254	0.05852353
Zmym1	blue	-0.970186348	0.001320031	-0.830175493	0.040811645	0.931726118	0.006832861	-0.833582168	0.03923788
Ccdc190	blue	-0.96980015	0.001354275	-0.828365079	0.041659762	0.730158659	0.099397363	-0.763186345	0.077480722
Rgs8	blue	-0.96960774	0.001371498	-0.876787669	0.021836657	0.775316738	0.070052559	-0.836047184	0.03811722
Cox4i1	blue	0.969508712	0.001380404	0.843695178	0.034737439	-0.816827131	0.047255514	0.873963982	0.022826571
Gm15427	blue	0.969178661	0.001410293	0.836448453	0.037936234	-0.836724581	0.037811925	0.865232462	0.02601959
Eif4a-ps4	blue	0.968737326	0.001450755	0.814629163	0.048358632	-0.943462322	0.004704402	0.908644674	0.012137477
Ndufa8	blue	0.968432306	0.00147905	0.891101076	0.017142749	-0.742766324	0.090743278	0.726849889	0.101726475
Gm42738	blue	-0.968386508	0.001483322	-0.855134892	0.029958787	0.89284539	0.016607985	-0.759674297	0.079694486
Camk1d	blue	-0.968294432	0.001491929	-0.9065382	0.012694463	0.849500233	0.032270847	-0.701490984	0.120361733
Ubl5	blue	0.968122746	0.001508043	0.946841041	0.004163702	-0.812272804	0.049554356	0.786068662	0.063754469
Atp5g3	blue	0.967067489	0.001608967	0.938107898	0.005627405	-0.831311979	0.040283409	0.799060288	0.056508504
Rps23-ps1	blue	0.967008592	0.001614695	0.828235952	0.041720565	-0.810209698	0.050612381	0.873549387	0.022973679
Scamp4	blue	-0.965787663	0.001735704	-0.831366911	0.040257958	0.825291429	0.043118305	-0.894155796	0.016211607
Pex6	blue	-0.965461759	0.001768735	-0.953171024	0.003238083	0.786451358	0.063535302	-0.6909671	0.128495473
Spred1	blue	-0.965429583	0.001772013	-0.934462812	0.006301939	0.857218537	0.029124409	-0.75397964	0.08334371
Slc16a1	blue	-0.965212573	0.001794198	-0.806333032	0.052628418	0.827211937	0.042204216	-0.752611448	0.084231418
Eef1a1	blue	0.964237388	0.001895577	0.838317189	0.037098695	-0.938386824	0.005577328	0.918803713	0.009621599
Arpc3	blue	0.963399916	0.001984835	0.845783404	0.03384029	-0.70429054	0.118237106	0.771803194	0.072169138
Mid1ip1	blue	0.96339	0.0019851	0.7640	0.07691	-0.731200	0.0986690	0.831073357	0.040394053

		7133	35	90964	5079	309	82		
Psmb7	blue	0.963097051	0.002017614	0.946767842	0.004175073	-0.768668106	0.074081869	0.699980916	0.1215146
Ghitm	blue	-0.962821823	0.002047631	-0.902759181	0.013723922	0.920244891	0.00928766	-0.894447181	0.016124093
Prickle1	blue	-0.962800732	0.00204994	-0.941553114	0.005024229	0.833084741	0.039465867	-0.717546057	0.108403225
Zfhx2	blue	-0.962559942	0.002076396	-0.79997516	0.056013414	0.818553984	0.046397143	-0.767735955	0.074654954
Sgtb	blue	-0.962411268	0.002092814	-0.911024435	0.011522783	0.927919304	0.007606188	-0.887476039	0.018280094
Ryr3	blue	-0.962113627	0.002125875	-0.909798294	0.011837565	0.757487382	0.081087184	-0.66253166	0.15161105
Gm5518	blue	0.962063163	0.002131506	0.788362454	0.062446006	-0.887500665	0.018272249	0.890809115	0.017233052
Glul	blue	0.962055856	0.002132322	0.8690097	0.024613892	-0.9418368	0.004976055	0.806455936	0.052563944
Sap18	blue	0.961951401	0.002144002	0.904326327	0.013292305	-0.82444957	0.043521878	0.711606999	0.112762886
Taf15	blue	0.961674404	0.00217513	0.89508231	0.01593413	-0.813493607	0.048933174	0.779959929	0.06729954
Cers5	blue	0.961626476	0.002180538	0.896458683	0.015526183	-0.819823569	0.045770737	0.704269308	0.118253156
Zbtb16	blue	0.961114413	0.002238734	0.957214968	0.002706678	-0.872326456	0.023410227	0.772384614	0.07181691
Aip	blue	0.960936398	0.002259143	0.875656263	0.022230785	-0.778199739	0.068337262	0.788107686	0.062590721
Rpl27-ps3	blue	0.960639882	0.00229334	0.95593249	0.00287013	-0.890648599	0.017282796	0.781915051	0.066155394
Sqstm1	blue	0.959620473	0.00241284	0.890194771	0.017423811	-0.729571775	0.099808736	0.772863433	0.071527426
Paip2	blue	0.958955202	0.00249244	0.781261626	0.066536784	-0.901687061	0.014023032	0.817680401	0.046830463
Golgb1	blue	-0.958849842	0.002505163	-0.958490947	0.002548742	0.783964737	0.064965536	-0.724246275	0.103576002
Bex2	blue	0.958671428	0.002526781	0.91747205	0.009935251	-0.950767572	0.003576082	0.86407296	0.026458535
Gm5805	blue	0.95858623	0.002537136	0.958827055	0.002507919	-0.781867951	0.066182851	0.71862265	0.107621045
Lman2	blue	-0.958478634	0.002550244	-0.882517021	0.019892611	0.969932488	0.001342492	-0.857049754	0.029191581
Gm6851	blue	0.958177081	0.002587157	0.927710214	0.007649833	-0.803407876	0.054173703	0.731518381	0.098447173
Maml2	blue	0.95817	0.0025875	0.9689	0.00142	-0.896552	0.0154986	0.778500822	0.068159245

		3854	54	65951	9724	088	83		
Gm45774	blue	0.957316166	0.002693981	0.922101615	0.008865888	-0.900045642	0.014486995	0.719069098	0.107297419
Sh2d5	blue	-0.95670143	0.002771562	-0.783072005	0.065482561	0.891138616	0.017131154	-0.795478379	0.058466163
Atp5h	blue	0.956491991	0.002798241	0.853895169	0.03046051	-0.730369364	0.099249855	0.72357921	0.104052237
Tnrc18	blue	-0.954798949	0.003018527	-0.859566981	0.028197379	0.792493912	0.060120686	-0.663187225	0.151059768
Atp5j2	blue	0.954518981	0.003055745	0.944458324	0.004541647	-0.790769853	0.061086121	0.664356404	0.150078698
Tpd52l2	blue	-0.954221338	0.00309556	-0.955393834	0.002940188	0.911643579	0.011365393	-0.819652516	0.045854902
Apoe	blue	0.954187027	0.003100166	0.948085	0.003972791	-0.938310128	0.005591076	0.800713388	0.055615381
Rbfa	blue	0.954038841	0.003120097	0.899554674	0.014627186	-0.825849465	0.042851759	0.842926417	0.035070497
Gm10282	blue	0.953513403	0.003191277	0.930998644	0.006977517	-0.949832054	0.003712102	0.827460141	0.042086748
Cdh11	blue	-0.953334607	0.003215678	-0.917983706	0.00981416	0.828175831	0.041748888	-0.854201645	0.030336111
Gm5905	blue	0.95316774	0.003238533	0.917119674	0.010019064	-0.818591606	0.046378523	0.768263897	0.07433013
Cndp2	blue	0.952848525	0.003282477	0.916790685	0.010097623	-0.924847633	0.008259592	0.880526763	0.02055811
Gm49326	blue	-0.952315042	0.003356569	-0.768332874	0.074287738	0.717175241	0.108673212	-0.826504493	0.042539869
Ctnnd1	blue	-0.951991424	0.003401909	-0.853426001	0.030651413	0.753429073	0.083700419	-0.748468405	0.086945246
Arhgap33	blue	-0.951560988	0.00346268	-0.780000687	0.067275596	0.900331083	0.014405789	-0.954797547	0.003018712
Psma2	blue	0.951294647	0.003500547	0.902246364	0.013866604	-0.934784746	0.006240863	0.783832521	0.065041991
Inpp4a	blue	0.950651887	0.003592767	0.947336111	0.004087197	-0.871051527	0.023869504	0.721811914	0.105318622
Atp5c1	blue	0.950502061	0.003614433	0.937536814	0.00573062	-0.781887753	0.066171307	0.760877771	0.078932723
Psma6	blue	0.949654823	0.003738152	0.97165078	0.001194126	-0.864233468	0.026397565	0.744218724	0.089768967
Nudt2	blue	0.9494623	0.003766551	0.926954491	0.007808597	-0.939699914	0.005344522	0.804867603	0.053399984
Gm13502	blue	-0.949387663	0.003777589	-0.770175824	0.073159169	0.737799722	0.094110477	-0.886767339	0.018506539
Rpl23	blue	0.94898	0.0038379	0.7853	0.06416	-0.732014	0.0981016	0.7227495	0.104645936

		1606	18	58967	182	239	7		
Oaz2	blue	0.948445382	0.003918305	0.886418018	0.018618647	-0.695397712	0.125042942	0.684179127	0.133863803
Pbrm1	blue	-0.947987733	0.00398756	-0.888729244	0.01788294	0.74844673	0.086959546	-0.74404309	0.089886537
Rps23	blue	0.947386062	0.004079516	0.894261944	0.016179701	-0.822041081	0.044686141	0.878304638	0.021313502
Papola	blue	-0.947024314	0.004135299	-0.799429364	0.056308534	0.869780618	0.02433156	-0.737246512	0.094488922
Fnip1	blue	-0.946486928	0.004218852	-0.761166721	0.078750318	0.809362675	0.051049758	-0.767891462	0.074559209
Rps15	blue	0.946169741	0.004268553	0.95355977	0.003184964	-0.856806195	0.029288642	0.785203963	0.064250948
Kars	blue	0.946119924	0.004276385	0.716743357	0.108988036	-0.82775936	0.041945339	0.84404666	0.034585661
Gm10163	blue	0.945955532	0.00430228	0.968432271	0.001479053	-0.904997371	0.013109526	0.738875783	0.093376296
Snrnp25	blue	0.945900888	0.004310905	0.777805184	0.068570866	-0.730702688	0.099016702	0.810432203	0.050497775
Fis1	blue	0.945225287	0.004418234	0.694144572	0.126015303	-0.849448486	0.032292456	0.888580394	0.017929893
Nhp2	blue	0.945189695	0.004423925	0.846056519	0.033723771	-0.934567257	0.006282092	0.772916802	0.071495194
Magi2	blue	-0.945130565	0.004433386	-0.926854008	0.007829826	0.941410236	0.005048578	-0.757513696	0.081070361
Gm13370	blue	0.945017833	0.004451451	0.964023878	0.00191814	-0.911659897	0.011361259	0.73958333	0.092894945
Mlst8	blue	0.944638766	0.004512462	0.821564738	0.044918101	-0.785062626	0.064332266	0.856634065	0.02935733
Psma1	blue	0.94380717	0.004647733	0.771049615	0.072626825	-0.947576197	0.004050346	0.942804183	0.004813488
Macf1	blue	-0.94366091	0.004671727	-0.963759221	0.001946292	0.746557578	0.088209901	-0.691472029	0.128099951
Brk1	blue	0.943651041	0.004673348	0.954957364	0.002997566	-0.74121922	0.091786288	0.7141485	0.110888002
Rpl22	blue	0.943337436	0.004725007	0.931248873	0.006927593	-0.910504206	0.011655838	0.721088834	0.105838707
Idh2	blue	0.9432493	0.004739576	0.887603328	0.018239564	-0.930843393	0.007008579	0.870930695	0.023913253
Mrpl30	blue	0.9430604	0.004770875	0.937689281	0.005702974	-0.828642059	0.041529478	0.811824064	0.049783602
Sap18b	blue	0.942983281	0.004783681	0.922561803	0.008762826	-0.772667498	0.07164582	0.617982927	0.191030345
Nr3c1	blue	-0.9429	0.0047851	-0.9781	0.00071	0.851411	0.0314773	-0.726016658	0.102316772

		74623	2	00133	4155	817	74		
Smc3	blue	-0.94274414	0.004823501	-0.871491493	0.023710532	0.889501561	0.01764027	-0.885317361	0.018974002
Cmip	blue	-0.942475231	0.004868471	-0.974849189	0.00094089	0.895159317	0.015911171	-0.721918239	0.105242242
Htra1	blue	0.942452085	0.004872351	0.917022968	0.010042125	-0.823014786	0.044213727	0.643310157	0.168151062
Ip6k2	blue	0.942415433	0.004878499	0.978012207	0.000719879	-0.88053639	0.020554866	0.721382499	0.105627348
Rpl32	blue	0.942100456	0.004931486	0.968742354	0.001450291	-0.897695294	0.015164006	0.793525206	0.059546561
Shank3	blue	-0.941528447	0.005028429	-0.813615709	0.048871244	0.741590057	0.091535796	-0.735551754	0.095652499
Mrpl16	blue	0.941199894	0.005084529	0.826286016	0.042643778	-0.669157065	0.146079026	0.647365054	0.164601782
Ankrd13c	blue	-0.941081428	0.005104832	-0.890317199	0.017385716	0.812641785	0.049366224	-0.723345032	0.104219652
Rpl21-ps15	blue	0.940658389	0.005177657	0.976171204	0.000844952	-0.899540734	0.014631175	0.741642906	0.091500122
Polr1c	blue	0.940616515	0.005184892	0.891165588	0.017122826	-0.716540763	0.109135855	0.791053415	0.060926847
Gabarapl1	blue	0.940233568	0.005251296	0.865923184	0.025759767	-0.669509732	0.145787021	0.690757442	0.128659858
Adi1	blue	0.939907328	0.005308193	0.939655876	0.005352251	-0.894052766	0.016242606	0.826631324	0.042479602
Nfu1	blue	0.939016639	0.005465058	0.812013761	0.049686633	-0.919365512	0.009490741	0.758631532	0.080357195
Lmbr1	blue	-0.938799672	0.005503608	-0.933670759	0.006453442	0.894950042	0.015973601	-0.685684511	0.132665062
Tmed8	blue	-0.938565502	0.005545363	-0.89360613	0.016377312	0.874804042	0.022529879	-0.717147154	0.108693674
Gm11518	blue	0.938225026	0.00560635	0.976237803	0.000840254	-0.865488505	0.025923132	0.716239471	0.109355853
Fam13b	blue	-0.937994423	0.005647841	-0.862566722	0.027033945	0.800231024	0.055875311	-0.702464281	0.119621209
Per2	blue	-0.937926301	0.005660127	-0.950327154	0.003639806	0.856742783	0.029313938	-0.767046283	0.075080251
Rpsa	blue	0.937655153	0.005709156	0.932445538	0.006691262	-0.938779901	0.005507127	0.849291488	0.032358058
Epb41l2	blue	-0.937183779	0.005794884	-0.690441767	0.12890754	0.864305193	0.026370341	-0.925913975	0.008029789
Sf3a3	blue	0.937115502	0.005807353	0.878436075	0.021268459	-0.921204662	0.009068449	0.748952197	0.086626356
Gm49164	blue	0.93691	0.0058441	0.8956	0.01577	-0.804397	0.0536487	0.683836501	0.134137287

		4311	72	11615	6646	25	3		
AC117239.2	blue	0.936354064	0.005947299	0.925711709	0.008073136	-0.901882057	0.013968399	0.858713536	0.028532627
Rps26	blue	0.935728735	0.006063448	0.985994366	0.000292863	-0.876649593	0.021884576	0.731564702	0.098414875
Gm10132	blue	0.935658708	0.006076523	0.968369703	0.001484891	-0.880790528	0.020469311	0.729184551	0.100080572
Tenm2	blue	-0.934517718	0.006291502	-0.93708878	0.005812237	0.823060971	0.044191378	-0.783554993	0.065202606
Rpl17	blue	0.93394536	0.006400718	0.969266847	0.001402276	-0.915947251	0.010300386	0.770677136	0.072853537
Mertk	blue	0.933633438	0.006460624	0.865430444	0.02594499	-0.895116166	0.015924034	0.717657937	0.108321824
Gm5559	blue	0.932775423	0.006626817	0.761012342	0.078847749	-0.714422299	0.110686844	0.816706048	0.047315976
Fam214a	blue	0.931332313	0.006910984	0.828405047	0.04164095	-0.905366958	0.01300938	0.730499775	0.099158606
Lamtor2	blue	0.930565356	0.007064377	0.790108013	0.061458611	-0.652891594	0.159815821	0.67663008	0.139945068
Clcn4	blue	-0.930152482	0.007147632	-0.895980104	0.015667453	0.650746216	0.161666634	-0.642765606	0.168630132
Gm10335	blue	0.93015223	0.007147683	0.97051378	0.001291338	-0.895445818	0.015825895	0.715271205	0.110064179
Mir7093	blue	0.930122654	0.007153665	0.853843864	0.030481359	-0.760387035	0.079242945	0.721878306	0.105270925
Trappc1	blue	0.929300807	0.007320873	0.80815634	0.051675679	-0.656869493	0.156407974	0.629036907	0.180895638
Bop1	blue	-0.928736318	0.007436812	-0.721911445	0.105247121	0.812504115	0.049436379	-0.789730854	0.061671344
Bnip3l	blue	0.928180849	0.007551764	0.737743533	0.094148884	-0.677531468	0.139212832	0.711678645	0.112709843
Chmp1b	blue	-0.92804875	0.007579228	-0.768188655	0.074376384	0.708943171	0.114742811	-0.808845163	0.051317842
Hif3a	blue	0.927835626	0.00762364	0.829874581	0.040952046	-0.872766931	0.02325254	0.751795603	0.084762766
Gm3531	blue	0.927653747	0.007661641	0.956672307	0.002775264	-0.860605463	0.027791979	0.684878297	0.133306473
Mir6982	blue	-0.927133268	0.007770896	-0.791683218	0.060573777	0.623141959	0.186271912	-0.692218227	0.127516405
Chchd1	blue	0.926679112	0.007866844	0.916963734	0.010056264	-0.950358218	0.003635293	0.768030762	0.07447349
Nedd8	blue	0.926578821	0.00788811	0.94789086	0.004002296	-0.875076149	0.022434174	0.817164265	0.04708736
Rpl29	blue	0.92565	0.0080856	0.9099	0.01179	-0.879890	0.0207729	0.817943718	0.046699653

		3542	23	58549	619	808	66		
Mfsd2a	blue	0.92563 1941	0.0080902 62	0.9678 38515	0.00153 4908	-0.835207 813	0.0384971 11	0.631920045	0.178290019
Srsf2	blue	0.92555 0472	0.0081077 71	0.9220 42432	0.00887 9185	-0.953286 882	0.0032222 06	0.757775636	0.080902992
Cic	blue	0.92539 006	0.0081423 01	0.7458 96979	0.08864 9012	-0.912316 638	0.0111954 87	0.886398705	0.018624855
Gm10240	blue	0.92525 2378	0.0081719 95	0.9669 04291	0.00162 4864	-0.858531 656	0.0286043 15	0.674147323	0.141970437
Mrpl41	blue	0.92512 532	0.0081994 45	0.9512 18346	0.00351 1433	-0.723227 509	0.1043037 14	0.58897711	0.218690659
Zfp961	blue	-0.9245 60475	0.0083220 15	-0.9021 84928	0.01388 3746	0.776334 883	0.0694445 82	-0.617988049	0.191025596
Fcf1	blue	0.92409 7673	0.0084231 02	0.9735 0118	0.00104 3978	-0.872597 543	0.0233131 2	0.682071398	0.135550005
Tuba8	blue	0.92388 1908	0.0084704 33	0.9443 29433	0.00456 2551	-0.758945 204	0.0801575 86	0.742471787	0.090941435
Prkca	blue	-0.9237 60547	0.0084971 12	-0.9676 80841	0.00154 9913	0.909734 472	0.0118540 63	-0.781549861	0.066368424
Rpl13a	blue	0.92142 1812	0.0090192 06	0.9292 46477	0.00733 1993	-0.825076 726	0.0432210 63	0.707928882	0.115500665
Ndufa13	blue	0.92116 0626	0.0090784 52	0.8946 53957	0.01606 2129	-0.751918 97	0.0846823 22	0.817608663	0.04686613
Sall2	blue	-0.9208 07301	0.0091588 97	-0.8311 62256	0.04035 2816	0.890404 559	0.0173585 57	-0.682133316	0.135500341
Ndufb9	blue	0.92072 7379	0.0091771 42	0.9138 1525	0.01082 1635	-0.745967 934	0.0886018	0.788079787	0.062606578
Avl9	blue	-0.9199 32466	0.0093595 66	-0.7924 36657	0.06015 2634	0.710004 241	0.1139523 46	-0.63656818	0.174122506
Ube2m	blue	0.91990 3183	0.0093663 19	0.9811 92971	0.00052 723	-0.757458 315	0.0811057 68	0.683664428	0.134274727
Rpl30	blue	0.91983 6073	0.0093818 06	0.9847 06511	0.00034 9048	-0.872328 822	0.0234093 79	0.676736477	0.139858551
Setd7	blue	-0.9185 85971	0.0096725 5	-0.9126 77572	0.01110 4884	0.855610 879	0.0297672	-0.697937397	0.123082354
Dagla	blue	-0.9184 30048	0.0097091 16	-0.6969 34917	0.12385 4639	0.769163 327	0.0737782 22	-0.771103824	0.072593858
Smad4	blue	-0.9170 58199	0.0100337 21	-0.8531 44607	0.03076 6181	0.858499 872	0.0286168 51	-0.648212763	0.163863806
Fam98b	blue	0.91691 7674	0.0100672 64	0.8540 39068	0.03040 2071	-0.824018 907	0.0437290 08	0.696646847	0.124076949
Atat1	blue	0.91608 5702	0.0102669 68	0.7749 79241	0.07025 4624	-0.606202 614	0.2020802 35	0.749347163	0.086366403
Rpl17-ps9	blue	0.91555	0.0103950	0.9562	0.00282	-0.834124	0.0389901	0.720034683	0.106598946

		622	54	84183	4837	305	06		
Ctsl	blue	0.915331428	0.010449666	0.97768591	0.000741323	-0.828088777	0.041789917	0.65331344	0.159452956
Rsl1d1	blue	0.915277425	0.010462806	0.802428536	0.054695675	-0.696127428	0.124478235	0.598126269	0.209801938
Dusp28	blue	0.914395689	0.010678489	0.880049277	0.020719328	-0.899167832	0.014738102	0.682452582	0.13524438
Imp3	blue	0.91420069	0.010726476	0.92544526	0.008130411	-0.868280889	0.024882228	0.695701017	0.124808088
Gpd1	blue	0.91376536	0.010833981	0.992367114	8.72E-05	-0.802602271	0.054602909	0.625836644	0.183806225
Gm10705	blue	0.91315342	0.01098598	0.911804132	0.01132475	-0.928552865	0.007474682	0.692671625	0.127162401
Ice1	blue	-0.913021162	0.011018966	-0.974678468	0.000953652	0.882245872	0.019982661	-0.665620464	0.149021077
Rpl35a	blue	0.912357064	0.011185321	0.962243701	0.002111396	-0.788014868	0.062643483	0.623782086	0.185684951
Lrp1	blue	-0.91229212	0.011201654	-0.835782961	0.038236614	0.670078727	0.145316427	-0.597447291	0.210456457
Prr18	blue	-0.912187922	0.011227884	-0.653095986	0.159639962	0.86518485	0.026037546	-0.944155228	0.004590878
Psma7	blue	0.911812095	0.011322737	0.632446041	0.17781635	-0.696260046	0.124375725	0.821660201	0.04487157
Gm14648	blue	0.911062929	0.011512966	0.97734619	0.00076398	-0.862999464	0.026868029	0.645894168	0.165885579
Gm6136	blue	0.909759561	0.011847576	0.941576753	0.005020206	-0.888459189	0.01796817	0.778345884	0.068250827
Med14	blue	-0.909330553	0.011958728	-0.894158988	0.016210647	0.81950506	0.045927515	-0.829572714	0.04109312
Ncstn	blue	0.908875728	0.012077118	0.661019847	0.152885627	-0.738189001	0.093844581	0.753484521	0.083664464
Gnptg	blue	0.908799236	0.012097084	0.971444273	0.001211502	-0.892451333	0.016728081	0.677386262	0.139330678
Cpd	blue	-0.907738577	0.012375583	-0.961404626	0.002205658	0.748708151	0.086787152	-0.653523977	0.159271985
Cdc42ep2	blue	-0.907381613	0.01247	-0.804443304	0.053624351	0.6696248	0.1456918	-0.605967009	0.202303822
Dctn6	blue	0.906924256	0.012591479	0.943188758	0.004749596	-0.830672297	0.040580335	0.641971863	0.169329454
Rin2	blue	0.906863863	0.012607563	0.875716487	0.022209721	-0.851979891	0.031243372	0.647769813	0.164249244
Pdpk1	blue	-0.906531679	0.012696206	-0.966142419	0.001700098	0.871145555	0.023835487	-0.632644456	0.177637811
Gapdh	blue	0.90651	0.0126996	0.9565	0.00279	-0.742277	0.0910723	0.654111924	0.158767066

		8887	25	09166	6048	337	62		
Snrpd1	blue	0.906463293	0.012714492	0.921084216	0.00909582	-0.843203152	0.034950432	0.790355219	0.061319359
Nsfl1c	blue	0.906082114	0.012816649	0.729962095	0.09953506	-0.672473926	0.143342588	0.643323301	0.168139506
Gas5	blue	0.905299286	0.013027689	0.961064794	0.002244413	-0.915115836	0.010502173	0.700126473	0.121403265
Erbb4	blue	0.904874444	0.013142918	0.788916606	0.062131762	-0.638110161	0.172749067	0.588205515	0.219447084
Tmem200a	blue	-0.902503368	0.013795008	-0.952285031	0.003360761	0.924838608	0.00826155	-0.754967742	0.082705244
Psmc5	blue	0.901681432	0.014024611	0.834830989	0.038668232	-0.610322982	0.198186395	0.619766905	0.18937929
Gar1	blue	0.901605408	0.01404594	0.926188706	0.007971095	-0.676061183	0.140408056	0.532317391	0.276943122
Zswim6	blue	-0.901522857	0.014069118	-0.823068932	0.044187526	0.793139345	0.059761075	-0.58311499	0.224463796
Egr4	blue	-0.900870256	0.014253	-0.810937559	0.050237929	0.866358665	0.025596592	-0.74740824	0.087645891
Arhgef17	blue	-0.900459994	0.014369187	-0.805005583	0.053327115	0.897604734	0.015190389	-0.691784097	0.127855765
Gm4366	blue	0.900349312	0.014400611	0.873017337	0.023163123	-0.668451596	0.146663896	0.699392404	0.121965199
Ufc1	blue	0.899943123	0.014516214	0.969091301	0.001418257	-0.872994505	0.023171269	0.632169965	0.178064896
Ndufb10	blue	0.899242405	0.014716689	0.976187211	0.000843822	-0.718866255	0.107444405	0.582973666	0.224603719
Wwp1	blue	-0.898855664	0.014827903	-0.94860632	0.003894092	0.81177404	0.049809188	-0.620975991	0.188263657
Fabp3-ps1	blue	0.898731326	0.014863744	0.91159601	0.011377448	-0.777613923	0.068684236	0.693696286	0.12636394
Adam10	blue	-0.898273675	0.014996023	-0.953323722	0.003217166	0.741292728	0.091736611	-0.682828626	0.134943168
Lpgat1	blue	-0.898198575	0.015017784	-0.967594689	0.001558142	0.896882801	0.015401504	-0.756107759	0.081971365
Dstyk	blue	-0.897719013	0.015157099	-0.820250403	0.045561029	0.898266037	0.014998236	-0.742733315	0.090765476
Gm8129	blue	0.897503482	0.015219914	0.953554931	0.003185622	-0.773791358	0.070967937	0.668958336	0.146243682
Sbf1	blue	-0.896912147	0.015392896	-0.875391459	0.022323516	0.723096695	0.104397319	-0.530190729	0.279232799
Rps27a-ps2	blue	0.896865346	0.015406626	0.957716996	0.002643981	-0.712032005	0.112448395	0.547737112	0.260559265
Mea1	blue	0.89577	0.0157274	0.9725	0.00111	-0.700010	0.1214922	0.585439054	0.222167784

		7468	54	51389	9799	118	6		
Ttyh1	blue	0.894916636	0.015983578	0.608351791	0.200045349	-0.74088465	0.092012546	0.857662643	0.028948013
Hist1h1c	blue	-0.894451195	0.016122889	-0.867969062	0.02499746	0.613665517	0.195050451	-0.623316913	0.186111413
Cdkl5	blue	-0.893668592	0.016358441	-0.955344712	0.002946619	0.867344929	0.025228862	-0.771065489	0.072617171
Acox1	blue	-0.893393058	0.016441764	-0.845011265	0.03417073	0.570246699	0.237346733	-0.563886775	0.243818895
Sidt2	blue	-0.892993922	0.016562825	-0.956630733	0.002780554	0.871767353	0.023611114	-0.629790507	0.180213059
Naca	blue	0.891119248	0.017137136	0.972080665	0.001158352	-0.85688779	0.029256109	0.640815984	0.170350004
AC165271.1	blue	-0.891095265	0.017144544	-0.83137151	0.040255828	0.774999425	0.070242532	-0.661337925	0.152617086
Atp6v0e2	blue	0.890776856	0.017243043	0.885262382	0.018991837	-0.685316962	0.132957313	0.577580085	0.229969871
Tmem242	blue	0.890552845	0.017312503	0.800964267	0.055480412	-0.545242282	0.263183883	0.614592337	0.194184553
Chrac1	blue	0.890310451	0.017387814	0.902769127	0.013721161	-0.833762091	0.039155568	0.567430898	0.240203736
Ppia	blue	0.89029735	0.017391889	0.866323547	0.025609732	-0.694188918	0.125980837	0.786844483	0.063310522
Orc6	blue	0.889705739	0.017576381	0.921207455	0.009067815	-0.684577693	0.133545971	0.657004859	0.156292554
Drg1	blue	0.889355308	0.017686102	0.882589129	0.019868696	-0.669615537	0.145699465	0.682835275	0.134937845
Srpk2	blue	-0.888643054	0.017910121	-0.996371262	1.97E-05	0.798677112	0.056716459	-0.661101397	0.152816759
Rps13	blue	0.888369466	0.017996529	0.983444381	0.000408864	-0.70930298	0.114474495	0.579150666	0.228402054
B230334C09Rik	blue	-0.887924155	0.0181376	-0.946260097	0.004254366	0.729272825	0.100018574	-0.696897927	0.123883175
Sorl1	blue	-0.88730172	0.018335669	-0.925370534	0.008146509	0.891934825	0.016886126	-0.808103713	0.051703065
Gm12989	blue	0.887084878	0.018404913	0.951713965	0.003441021	-0.679227695	0.137839395	0.665511287	0.149112299
Zfp580	blue	0.886851023	0.018479731	0.944142328	0.004592979	-0.874864952	0.022508439	0.605359313	0.202880987
Plppr5	blue	-0.886326987	0.018647915	-0.950397139	0.003629643	0.814055176	0.048648651	-0.600224364	0.207784656
Nagpa	blue	-0.884154749	0.019352855	-0.806810542	0.052378126	0.635812227	0.174797491	-0.80528741	0.053178421
Thrsp	blue	0.88343	0.0195887	0.8834	0.01959	-0.817795	0.0467734	0.616852728	0.192079387

		6343	49	19135	4416	098	63		
Psmb1	blue	0.882771747	0.019808192	0.791150073	0.0608726	-0.706439653	0.116617341	0.526550527	0.283168714
Gm7027	blue	0.882529956	0.01988832	0.925463726	0.008126435	-0.673714105	0.142325124	0.709778487	0.114120325
Sdhb	blue	0.881775743	0.020139254	0.747338831	0.08769185	-0.694285085	0.12590611	0.665589713	0.149046769
Gm12033	blue	0.881635938	0.020185934	0.916268236	0.01022299	-0.740130531	0.09252344	0.602435359	0.205667401
Prkar2a	blue	-0.881555307	0.020212879	-0.904845542	0.013150774	0.726148333	0.102223386	-0.514293321	0.296574698
Gm6055	blue	0.881267118	0.020309328	0.986451195	0.000274112	-0.712177305	0.112340965	0.583816945	0.223769316
Gm11361	blue	0.880802211	0.020465381	0.973683176	0.00102975	-0.714364587	0.110729231	0.540462017	0.268241234
Dlx1	blue	0.88079305	0.020468462	0.864084988	0.026453964	-0.859866454	0.028080186	0.603193502	0.204943433
Klhl26	blue	0.88043045	0.02059058	0.746493832	0.088252231	-0.598613882	0.209332401	0.584726916	0.22287032
Hmgcr	blue	-0.880356649	0.020615478	-0.979337569	0.000635993	0.795254174	0.05858972	-0.704232466	0.118281011
Rpl19-ps11	blue	0.880325198	0.020626093	0.986301526	0.000280187	-0.772097044	0.071991024	0.581521725	0.226043291
Lsm14b	blue	0.880159319	0.02068212	0.948662205	0.003885702	-0.716908646	0.1088675	0.591641339	0.216086903
Ctss	blue	0.87980235	0.020802936	0.730814491	0.098938554	-0.861475565	0.027454455	0.667010409	0.147861814
Osbpl8	blue	-0.879660348	0.020851091	-0.925656233	0.008085045	0.690649646	0.128744413	-0.631264237	0.17888132
Sh3glb1	blue	-0.879576275	0.020879626	-0.96184977	0.002155397	0.908310866	0.012224935	-0.688011731	0.130821069
Zcchc24	blue	0.879076867	0.021049513	0.591822841	0.215909979	-0.85072624	0.031760875	0.897408557	0.015247619
Pfdn2	blue	0.878526097	0.021237635	0.962645058	0.002067025	-0.681993027	0.135612879	0.583288841	0.224291716
Gm12254	blue	0.87814754	0.021367399	0.995987027	2.41E-05	-0.82850951	0.041591801	0.6287848	0.181124226
Suclg1	blue	0.878105575	0.021381807	0.960406248	0.002320463	-0.772179471	0.071941098	0.525878589	0.283897529
Rab26os	blue	0.877971639	0.021427824	0.965510568	0.001763768	-0.886096698	0.018722055	0.666833607	0.148009059
Gm44429	blue	0.877619776	0.021548939	0.949843799	0.003710379	-0.678719037	0.138250641	0.619422558	0.18969752
C030006K1	blue	0.87754	0.0215750	0.9852	0.00032	-0.871000	0.0238879	0.659395657	0.154259998

1Rik		4007	63	42209	5082	623	3		
Eif3b	blue	0.876586913	0.021906346	0.915278801	0.010462471	-0.598693152	0.20925611	0.574435603	0.233121652
Atrn	blue	-0.876405722	0.021969333	-0.964209523	0.001898514	0.825747199	0.042900548	-0.639103957	0.171866306
Gm6204	blue	0.8744771	0.022645128	0.861374762	0.027493457	-0.727140068	0.101521256	0.646153034	0.165659335
Gm15920	blue	0.874274889	0.022716549	0.964143564	0.001905476	-0.89521055	0.015895906	0.655343098	0.157711953
Syt13	blue	0.873950842	0.022831226	0.810035041	0.050702426	-0.767624747	0.074723458	0.601095966	0.206948955
Klhl40	blue	0.873329063	0.023052038	0.987401479	0.000237084	-0.859910345	0.02806303	0.650078874	0.162244181
Tceal5	blue	0.872544707	0.023332031	0.968896388	0.001436107	-0.818767794	0.046291372	0.736483501	0.095012001
Rpl19	blue	0.872017999	0.023520955	0.922210956	0.008841347	-0.785270138	0.06421289	0.738650092	0.093530069
Ndufb3	blue	0.871033607	0.02387599	0.947685945	0.004033555	-0.898513656	0.014926589	0.671910399	0.14380594
Rps2	blue	0.870520253	0.024062143	0.96955192	0.001376514	-0.695910209	0.124646219	0.582029387	0.225539535
Usp9x	blue	-0.868382728	0.024844649	-0.963207863	0.00200559	0.703646294	0.118724569	-0.666348971	0.148412995
Gm10076	blue	0.868236464	0.024898629	0.969715949	0.001361798	-0.89534269	0.015856565	0.675194337	0.141114788
Rtcb	blue	0.867886666	0.025027951	0.892870388	0.01660038	-0.728215773	0.100762101	0.790233241	0.061388052
Prelp	blue	-0.867036427	0.025343615	-0.880119009	0.020695746	0.59504349	0.212780299	-0.622106899	0.187222623
Timm8b	blue	0.866395601	0.025582775	0.87678499	0.021837587	-0.595766251	0.212080493	0.636094578	0.174545254
Cdc42bpa	blue	-0.865663491	0.025857308	-0.937677513	0.005705105	0.730656397	0.099049067	-0.565185413	0.242491755
Exosc1	blue	0.865358494	0.02597209	0.766618733	0.075344457	-0.607733264	0.200630121	0.486672832	0.327625091
Mtmr2	blue	0.864497029	0.026297594	0.774579686	0.070494187	-0.826605548	0.042491847	0.631742544	0.17844998
Txn1	blue	0.86423022	0.026398798	0.955012101	0.002990341	-0.768845797	0.073972852	0.505450343	0.306390725
Pank3	blue	-0.864219638	0.026402816	-0.970116675	0.001326177	0.695983774	0.124589317	-0.629430841	0.180538696
Nefm	blue	0.86302212	0.026859355	0.946809094	0.004168663	-0.645525368	0.166208127	0.608976265	0.199455664
Alcam	blue	-0.8629	0.0268815	-0.9480	0.00397	0.735460	0.0957153	-0.718248306	0.107892735

		64157	48	67317	5474	498	33		
Rps3a3	blue	0.862866072	0.02691912	0.979664252	0.000616109	-0.848202987	0.032814621	0.592731321	0.21502529
Rab31	blue	0.862589444	0.027025221	0.9338173	0.00642528	-0.620613264	0.188598062	0.529066532	0.280446078
Pja2	blue	-0.862227311	0.027164417	-0.90998361	0.011789727	0.803045799	0.054366415	-0.78861189	0.062304468
Gm19439	blue	0.861882746	0.027297175	0.84421818	0.034511709	-0.841449452	0.03571457	0.596100132	0.211757532
AC026385.1	blue	0.861594428	0.027408498	0.836840113	0.037759971	-0.533119618	0.276081276	0.51287215	0.298144167
Gm15899	blue	0.861411814	0.027479118	0.955990035	0.002862695	-0.761745071	0.078385797	0.530657723	0.278729391
Ndufb8	blue	0.861285521	0.027528008	0.895601438	0.015779667	-0.689556995	0.129602839	0.555104726	0.252868245
Atp5f1	blue	0.860985537	0.027644303	0.850012033	0.032057492	-0.743212746	0.090443304	0.505837165	0.305958843
Mt2	blue	0.860976981	0.027647623	0.956807595	0.002758086	-0.859655071	0.028162883	0.580077831	0.227478532
Tnfrsf19	blue	-0.86093954	0.027662155	-0.902638139	0.013757535	0.904053386	0.013366999	-0.639609288	0.171418161
Gm5854	blue	0.860824849	0.027706692	0.977162757	0.000776354	-0.833887981	0.039098023	0.707424519	0.115878327
Rere	blue	-0.860166317	0.027963072	-0.873035367	0.023156691	0.819159053	0.046098111	-0.762982903	0.077608189
Rpl35	blue	0.8594902	0.028227463	0.945410547	0.004388674	-0.764796305	0.076475341	0.581232153	0.226330837
Tnr	blue	-0.859316929	0.028295409	-0.981530223	0.000508549	0.706543257	0.116539504	-0.562699067	0.24503517
Cox7c	blue	0.858940338	0.028443352	0.99684628	1.49E-05	-0.758763297	0.080273318	0.604160964	0.204021093
Slc16a2	blue	-0.858647022	0.028558833	-0.873458917	0.02300584	0.553845171	0.254176716	-0.496872187	0.316026112
BC030500	blue	-0.858349611	0.028676153	-0.914345484	0.010690834	0.93829411	0.005593949	-0.755882901	0.082115883
Kcns1	blue	0.858177178	0.028744277	0.968581883	0.001465141	-0.884182381	0.019343809	0.700453744	0.121153102
Gxylt1	blue	-0.857434896	0.029038409	-0.919008215	0.009573864	0.841559596	0.035666349	-0.581546699	0.226018499
Klhdc2	blue	0.857424314	0.029042613	0.942427142	0.004876534	-0.671161946	0.144422335	0.571609009	0.235969353
Naa38	blue	0.856661989	0.029346182	0.981970696	0.000484653	-0.782995123	0.065527174	0.59150828	0.216216643
Tspan7	blue	0.85634	0.0294712	0.9420	0.00493	-0.616429	0.1924731	0.57953033	0.228023701

		9036	37	74412	588	144	64		
1700020I14Rik	blue	-0.856286886	0.029496102	-0.987767119	0.00022355	0.809728279	0.050860757	-0.570607778	0.23698135
Pacs1	blue	-0.856005942	0.029608626	-0.974687865	0.000952947	0.846512636	0.0335296	-0.637034562	0.173706621
Trim37	blue	-0.85571327	0.029726062	-0.954591289	0.003046111	0.903172742	0.013609374	-0.700459793	0.12114848
Hspa2	blue	0.855488708	0.029816318	0.828452782	0.041618488	-0.670303716	0.145130526	0.650548154	0.161837954
Polr2e	blue	0.855433678	0.029838456	0.964659488	0.001851358	-0.686771995	0.131801996	0.553111823	0.254939759
Uqcrh	blue	0.855170193	0.029944558	0.977713095	0.000739524	-0.858975022	0.028429711	0.604918786	0.2032998
Smim19	blue	0.854783093	0.030100762	0.917345306	0.009965357	-0.586180208	0.221437569	0.588641978	0.219019073
Tceal6	blue	0.854282425	0.030303362	0.961799527	0.002161042	-0.88379713	0.019470111	0.725676817	0.102557963
Exoc6b	blue	-0.853843006	0.030481707	-0.93951032	0.005377836	0.890443699	0.017346395	-0.734818347	0.096158003
Flrt2	blue	-0.853501662	0.030620588	-0.974971902	0.00093177	0.765810167	0.075845261	-0.671325858	0.144287247
Dhrs4	blue	0.85279728	0.03090812	0.93950605	0.005378588	-0.7196122	0.106904309	0.552368207	0.255714397
Dock4	blue	-0.85271546	0.030941601	-0.929966426	0.007185305	0.827591835	0.042024483	-0.788812627	0.06219067
Sptbn1	blue	-0.852485915	0.031035625	-0.934301492	0.006332654	0.698368802	0.122750658	-0.554499358	0.253496794
Nop56	blue	0.852302154	0.031110992	0.861139429	0.027584615	-0.611066773	0.197486802	0.602603812	0.205506451
U2af1	blue	0.851373244	0.031493293	0.959795016	0.002392167	-0.870296799	0.024143387	0.727404302	0.101334545
Opa1	blue	-0.851191837	0.03156821	-0.944492348	0.004536137	0.883839254	0.019456281	-0.718265804	0.107880029
Mir3072	blue	-0.85089399	0.031691396	-0.878718998	0.021171656	0.924199527	0.008400804	-0.647753693	0.164263277
Rpl30-ps9	blue	0.850716757	0.031764805	0.904918373	0.01313098	-0.714004685	0.110993727	0.532670478	0.276563668
Gm22771	blue	-0.849872321	0.032115667	-0.913372889	0.010931348	0.743554126	0.090214214	-0.465029625	0.352737484
Dcun1d4	blue	-0.849504814	0.032268934	-0.97485558	0.000940414	0.866957225	0.025373116	-0.615157003	0.19365778
Etnk1	blue	-0.849295956	0.03235619	-0.984441895	0.000361199	0.846091171	0.033709001	-0.653999007	0.158863984
Dab1	blue	-0.8492	0.0323628	-0.9451	0.00442	0.789110	0.0620221	-0.505021412	0.306869886

		80129	07	93808	3267	231	34		
Prpf19	blue	0.848831178	0.032550763	0.949438215	0.003770111	-0.720573734	0.10620989	0.507314006	0.30431206
Ik	blue	0.848737091	0.032590218	0.534193046	0.274929686	-0.729837325	0.099622507	0.826723103	0.042436015
Ndufs4	blue	0.848362581	0.032747492	0.931888421	0.00680079	-0.840994715	0.035913981	0.585912467	0.221701247
Car4	blue	0.847365477	0.03316796	0.927883777	0.007613595	-0.583120018	0.224458818	0.553668119	0.254360856
5730409E04Rik	blue	0.846526154	0.033523854	0.953794343	0.00315312	-0.661283874	0.152662705	0.541639629	0.26699191
Gm8186	blue	0.846230627	0.03364959	0.925366142	0.008147456	-0.774117055	0.070772033	0.77008938	0.073211929
Cenpb	blue	0.84616491	0.03367758	0.953518787	0.003190543	-0.851010595	0.031643142	0.714716723	0.110470711
Kctd10	blue	-0.845947658	0.033770191	-0.901581874	0.014052546	0.889967923	0.017494504	-0.592468326	0.215281247
Gm7334	blue	0.845890784	0.033794456	0.848448251	0.032711484	-0.708258845	0.115253884	0.595394023	0.212440783
Gm7331	blue	0.844771086	0.034273825	0.899059558	0.014769219	-0.902247912	0.013866172	0.783138869	0.065443771
Ilk	blue	0.844749447	0.03428312	0.784379965	0.064725696	-0.807573158	0.051979535	0.621643278	0.187649111
Pou3f3	blue	-0.843976698	0.034615848	-0.793668818	0.059466812	0.865008426	0.02610413	-0.58905303	0.218616287
Rpl36al	blue	0.84395506	0.034625186	0.96631996	0.001682415	-0.74663941	0.088155573	0.490793336	0.322920679
Aff4	blue	-0.843610633	0.034773994	-0.909929468	0.011803693	0.550642722	0.257515393	-0.497084538	0.315786257
Rpl36a-ps2	blue	0.843437882	0.034848744	0.95276991	0.003293345	-0.883175031	0.019674892	0.671815578	0.143883969
Ptpn9	blue	-0.843039141	0.035021567	-0.902234654	0.013869871	0.940658494	0.005177638	-0.727781647	0.101068172
Rpl10-ps6	blue	0.842244562	0.035367155	0.944966334	0.004459716	-0.882531714	0.019887737	0.723637877	0.104010314
Rpl37	blue	0.842067273	0.035444482	0.992767367	7.83E-05	-0.792638772	0.060039889	0.613962559	0.194772762
Klhl7	blue	-0.841604056	0.035646893	-0.844425393	0.034422466	0.765776306	0.075866266	-0.485212621	0.329298185
Gm11808	blue	0.841265199	0.035795306	0.962068513	0.002130909	-0.844130813	0.034549368	0.714628677	0.110535325
Ndufs5	blue	0.840524734	0.036120624	0.929872141	0.007204433	-0.881145309	0.02035016	0.59907125	0.208892376
Psap	blue	0.83988	0.0364013	0.9820	0.00048	-0.810281	0.0505755	0.586186451	0.221431422

		8338	26	50833	0368	167	56		
Ccser2	blue	-0.839878046	0.036405874	-0.969764009	0.001357502	0.679239428	0.137829915	-0.47761051	0.338058531
Rps10-ps2	blue	0.839864756	0.036411747	0.981825828	0.000492449	-0.844266485	0.034490895	0.61481518	0.193976594
Rpl30-ps10	blue	0.838540372	0.036999255	0.801867338	0.054995825	-0.720684889	0.106129743	0.490804782	0.322907645
Bex3	blue	0.837845034	0.037309479	0.935363241	0.006131843	-0.809821308	0.050812717	0.515092536	0.295693455
2310022B05Rik	blue	0.837648569	0.037397352	0.756697388	0.081592951	-0.697244933	0.123615586	0.473092163	0.343304599
Atf6b	blue	0.837519351	0.0374552	0.971792657	0.00118226	-0.777253934	0.068897852	0.67652557	0.140030073
Romo1	blue	0.837482197	0.037471841	0.940032032	0.005286409	-0.646513059	0.165344896	0.523821639	0.286133018
Gm16409	blue	0.837461675	0.037481035	0.926576574	0.007888586	-0.769986397	0.073274807	0.495783997	0.317256296
Snrpf	blue	0.837371218	0.037521568	0.911718475	0.011346425	-0.921499111	0.009001708	0.769423587	0.07361887
Map1lc3b	blue	0.837142248	0.037624262	0.928551573	0.007474949	-0.763799022	0.07709742	0.740301061	0.092407801
Zfp804a	blue	-0.837072596	0.037655527	-0.919513834	0.009456339	0.90248795	0.013799298	-0.766977972	0.075122436
Bloc1s2	blue	0.836506969	0.037909875	0.954110667	0.003110429	-0.701376163	0.120449225	0.502661319	0.309511338
Sacm1l	blue	-0.835298342	0.038456053	-0.920790739	0.009162677	0.9095155	0.011910749	-0.613006694	0.19566693
Calb1	blue	0.834997913	0.038592385	0.836342804	0.037983846	-0.802750712	0.054523706	0.7945874	0.05895788
Gm16477	blue	0.834838026	0.038665033	0.966298062	0.001684591	-0.841872602	0.035529481	0.672046214	0.14369421
Eif3g	blue	0.834464611	0.03883495	0.959348072	0.002445279	-0.684477089	0.133626165	0.447630756	0.37340049
Gabrb1	blue	-0.834457786	0.038838059	-0.86716377	0.025296218	0.583721551	0.223863644	-0.652299456	0.160325758
Uqcc3	blue	0.834151402	0.03897774	0.970585542	0.001285091	-0.72714848	0.10151531	0.596408242	0.211459675
Gm9794	blue	0.833758758	0.039157092	0.975135195	0.000919701	-0.792399305	0.06017348	0.516082156	0.294603631
Rhoq	blue	-0.833650213	0.039206741	-0.895886194	0.015695246	0.536277803	0.272698403	-0.48948052	0.324416827
Nova2	blue	-0.833144928	0.039438249	-0.910256606	0.011719424	0.899901584	0.014528062	-0.610720459	0.19781241
Gas2l1	blue	-0.8331	0.0394531	-0.8768	0.02182	0.875295	0.0223571	-0.836819245	0.037769353

		1251	24	31288	153	654	11		
Tspan31	blue	-0.833104755	0.039456682	-0.947903598	0.004000357	0.844388025	0.034438551	-0.54660595	0.261748009
Snora57	blue	0.832615463	0.03968152	0.845605288	0.033916381	-0.58766895	0.219973719	0.66346418	0.150827126
Dynlrb1	blue	0.832512424	0.039728944	0.967999771	0.001519638	-0.664281903	0.150141131	0.56153726	0.246227224
Gm15501	blue	0.83223444	0.03985702	0.911100632	0.011503356	-0.904519804	0.013239481	0.731074392	0.098756992
Morn2	blue	0.831129934	0.040367806	0.967602644	0.001557381	-0.808290724	0.051605777	0.543259576	0.265276998
Baiap2	blue	-0.830515974	0.040653052	-0.779080002	0.067817397	0.837389157	0.037513528	-0.546245256	0.262127505
Arhgap21	blue	-0.830139543	0.040828406	-0.904675345	0.013197087	0.621720443	0.187578098	-0.432094658	0.392195301
Tmsb10	blue	0.829654494	0.041054879	0.877569465	0.021566284	-0.670791318	0.144727987	0.446279324	0.375022677
Kif21a	blue	-0.828563767	0.041566285	-0.982177001	0.000473658	0.832512325	0.03972899	-0.620859414	0.188371104
Ubb	blue	0.82781483	0.041919149	0.975404647	0.000899958	-0.691777151	0.127861198	0.547965319	0.260319696
Gm5879	blue	0.827416853	0.042107224	0.939323568	0.00541075	-0.797863606	0.057159125	0.610430392	0.198085305
Unc13a	blue	-0.827226174	0.042197474	-0.869701649	0.02436041	0.866691338	0.025472272	-0.554352844	0.25364901
Uqcrb	blue	0.827143487	0.042236639	0.918135489	0.009778378	-0.849109512	0.032434176	0.541660088	0.266970225
Ndufs8	blue	0.826809356	0.042395072	0.932537956	0.006673177	-0.650072109	0.16225004	0.570183179	0.237411033
Ttc1	blue	0.826527121	0.042529114	0.908559135	0.012159859	-0.771016785	0.072646795	0.757317626	0.081195745
Tmem145	blue	0.826122383	0.042721681	0.975577213	0.000887425	-0.765056869	0.076313185	0.629592258	0.18039252
Dr1	blue	-0.825683608	0.0429309	-0.970420917	0.001299444	0.782023568	0.066092151	-0.508857	0.302595057
Mtmr9	blue	-0.825599421	0.042971097	-0.732861657	0.097512459	0.738562649	0.093589678	-0.489106812	0.324843186
Pex3	blue	0.825438664	0.043047903	0.78515717	0.064277865	-0.512976025	0.298029348	0.656659856	0.156586792
Prdx2	blue	0.825255184	0.043135644	0.852735762	0.030933292	-0.576470209	0.231080372	0.694565245	0.125688521
Arxes1	blue	0.824132633	0.043674266	0.876396648	0.02197249	-0.893249282	0.016485323	0.823052656	0.044195401
Camk2n1	blue	0.82408	0.0436975	0.9308	0.00700	-0.822526	0.0444501	0.67482964	0.141412577

		4164	93	8249	075	817	83		
Gm28900	blue	0.824077223	0.043700934	0.968426037	0.001479634	-0.829445687	0.041152553	0.568232964	0.239388556
Gm9762	blue	0.824003619	0.043736369	0.986139774	0.000286827	-0.72331635	0.104240165	0.573896171	0.233664051
Atpif1	blue	0.823514696	0.043972094	0.95744884	0.00267738	-0.709964936	0.113981584	0.593670456	0.214112292
Rps13-ps1	blue	0.823346039	0.044053546	0.990643669	0.000130902	-0.784588404	0.064605453	0.580064017	0.227492281
Rpl36a	blue	0.822692783	0.044369695	0.959721041	0.002400918	-0.878800709	0.021143738	0.620422875	0.188773684
Pgbd5	blue	0.82203825	0.044687518	0.919421434	0.009477763	-0.642830072	0.168573388	0.599903384	0.20809276
Dlg3	blue	-0.820091925	0.045638838	-0.923590019	0.008534669	0.847107346	0.033277224	-0.560775652	0.247009894
Tmed7	blue	-0.820010116	0.045679029	-0.785125131	0.064296298	0.740159514	0.092503782	-0.452118583	0.36803118
Arpc5l	blue	0.819885248	0.045740405	0.938339821	0.005585751	-0.633964415	0.176451975	0.571070172	0.23651377
Lmbrd2	blue	-0.819594235	0.045883596	-0.965414349	0.001773566	0.853431169	0.030649307	-0.663173308	0.151071462
Slc24a3	blue	-0.81934642	0.046005695	-0.914897026	0.010555594	0.851907324	0.031273218	-0.747715125	0.087442816
Zeb2	blue	-0.819189893	0.046082894	-0.951564002	0.003462252	0.766969053	0.075127944	-0.658436184	0.155074346
Luzp2	blue	-0.819154617	0.0461003	-0.908952083	0.012057204	0.779931117	0.067316467	-0.76025352	0.079327443
Mt3	blue	0.818810489	0.046270265	0.991057949	0.000119583	-0.756094553	0.081979849	0.564617354	0.243071931
Gm10136	blue	0.818715309	0.046317326	0.968626053	0.001461046	-0.79785572	0.057163424	0.651874663	0.160692003
Ufsp2	blue	0.818432443	0.046457318	0.837355182	0.037528756	-0.724376045	0.103483469	0.776721491	0.069214354
Cacna2d1	blue	-0.818373878	0.046486327	-0.91257255	0.011131121	0.597422191	0.210480669	-0.584052267	0.223536693
Cox6b1	blue	0.81833895	0.046503632	0.977885797	0.00072815	-0.772535233	0.071725792	0.499445573	0.313123961
Tpt1-ps3	blue	0.817905677	0.046718541	0.922270635	0.008827967	-0.859754939	0.028123798	0.598488045	0.209453532
Gm13577	blue	0.816817199	0.047260472	0.94482157	0.004482989	-0.890947631	0.017190181	0.681576658	0.13594712
Gpam	blue	-0.816555594	0.047391153	-0.838723863	0.036917593	0.491581334	0.322023856	-0.493210293	0.320172839
Snca	blue	0.81648	0.0474277	0.9670	0.00161	-0.722285	0.1049787	0.585026722	0.222574447

		2362	65	412	1522	353	08		
Snapc5	blue	0.815856032	0.047741432	0.926628425	0.007877588	-0.722191847	0.105045803	0.537508709	0.271384265
Hars	blue	0.815536106	0.047902022	0.79010682	0.061459283	-0.484287037	0.330360316	0.600150034	0.207855988
Rps13-ps5	blue	0.814984552	0.048179468	0.932403896	0.006699419	-0.835253761	0.03847627	0.536922605	0.272009697
R3hdm1	blue	-0.814633194	0.048356598	-0.948583846	0.003897469	0.858172057	0.028746302	-0.702195322	0.119825644
Elob	blue	0.81395905	0.048697299	0.933935851	0.00640254	-0.680214431	0.13704313	0.554741242	0.253245574
Slc24a2	blue	-0.813485266	0.048937406	-0.974613892	0.000958502	0.780558851	0.066948096	-0.634721318	0.175773477
Appbp2	blue	-0.813170952	0.049096997	-0.966627515	0.001652	0.694426844	0.125795991	-0.443892597	0.377893545
Srrm1	blue	-0.812170968	0.049606338	-0.883512605	0.019563643	0.557987525	0.249883442	-0.396202706	0.436793214
Reps2	blue	-0.812147593	0.049618273	-0.917542579	0.009918516	0.880552505	0.020549435	-0.753087662	0.083921962
Atp2a2	blue	-0.812105786	0.049639623	-0.898103161	0.015045453	0.845318942	0.034038877	-0.700600048	0.121041341
Hpcal1	blue	0.811974284	0.049706805	0.869437868	0.024456894	-0.734331807	0.096494007	0.763819975	0.077084327
Etfb	blue	0.811516589	0.049940964	0.920595164	0.009207363	-0.673403097	0.142579988	0.561096422	0.246680134
Fus	blue	0.809864883	0.050790222	0.975337907	0.000904828	-0.773183645	0.07133413	0.511501957	0.299660281
Gm10177	blue	0.808725479	0.051379934	0.953161788	0.00323935	-0.775947106	0.069675855	0.645429087	0.166292377
Dctn3	blue	0.808011319	0.051751162	0.880153637	0.02068404	-0.551715032	0.256395577	0.544761377	0.263690978
Gm28437	blue	0.807358464	0.052091605	0.908362919	0.012211277	-0.894380868	0.01614399	0.574458706	0.233098433
Alg10b	blue	-0.807170091	0.052190028	-0.916205653	0.010238058	0.647479851	0.164501763	-0.473368891	0.342982466
Foxo4	blue	0.80710229	0.052225474	0.913651411	0.010862207	-0.811929294	0.0497298	0.750474855	0.08562613
Gdi1	blue	0.806949562	0.05230536	0.750814112	0.085403985	-0.414394592	0.413988628	0.471123197	0.345599766
Tcaf1	blue	-0.806646616	0.052463987	-0.9339066	0.006408147	0.723169572	0.104345167	-0.561713705	0.246046039
mt-Co3	blue	0.80560574	0.053010698	0.90877024	0.012104657	-0.891586865	0.016993	0.570122016	0.237472953
Ddx46	blue	-0.8054	0.0531130	-0.8598	0.02810	0.742214	0.0911144	-0.453803257	0.366022645

		11392	68	0772	3152	91	13		
Rasgrf1	blue	-0.805381345	0.053128902	-0.926583283	0.007887163	0.744865869	0.08933636	-0.43088907	0.393666989
Gm12537	blue	0.805344598	0.053148271	0.954964842	0.002996579	-0.725765949	0.10249468	0.486515432	0.327805288
Arl2	blue	0.804736605	0.053469209	0.750686266	0.085487668	-0.735136013	0.095938905	0.528301549	0.281272825
Dzip1	blue	-0.803984428	0.053867491	-0.91708312	0.010027778	0.782576505	0.065770336	-0.631130294	0.179002187
Atp5l-ps1	blue	0.803515181	0.054116652	0.98595522	0.000294499	-0.740569225	0.092226083	0.548350166	0.259915882
Trappc2l	blue	0.803351343	0.054203771	0.77189068	0.072116088	-0.831640209	0.040131448	0.615644055	0.193203885
Fbxl19	blue	0.802505828	0.054654396	0.921980322	0.00889315	-0.712144873	0.11236494	0.692772945	0.127083351
Tomm5	blue	0.800857534	0.055537814	0.881160169	0.020345176	-0.526951518	0.282734119	0.464471993	0.353393265
Gm8325	blue	0.800591323	0.055681105	0.982610975	0.000450938	-0.696271828	0.12436662	0.483183328	0.331628479
Pqbp1	blue	0.800536924	0.055710407	0.860580703	0.027801613	-0.758990648	0.080128686	0.777166157	0.068949984
Gm6863	blue	0.80048319	0.055739358	0.932296675	0.006720443	-0.820723939	0.045328901	0.529457911	0.280023458
Zyg11b	blue	-0.800183452	0.055900976	-0.983359943	0.000413034	0.779357661	0.067653792	-0.547561639	0.260743533
Rhob	blue	0.800124931	0.055932556	0.889751256	0.017562153	-0.764804467	0.07647026	0.441516325	0.380759372
Akap6	blue	-0.799767952	0.05612537	-0.957596546	0.002658958	0.781654605	0.066307291	-0.660964993	0.152931959
Agtpbp1	blue	-0.799197663	0.056434034	-0.9181861	0.00976646	0.547579119	0.260725175	-0.526003191	0.283762325
Psmb6	blue	0.798925266	0.056581742	0.881911188	0.020094079	-0.761450957	0.078571075	0.457631908	0.361472368
Gm11613	blue	0.798353692	0.056892256	0.881651889	0.020180605	-0.76520345	0.076222031	0.503589354	0.308471663
Tmod2	blue	-0.798126886	0.057015689	-0.914229393	0.010719406	0.809642212	0.050905221	-0.657123344	0.156191557
Acyp2	blue	0.797301651	0.057465836	0.942781591	0.004817255	-0.864448537	0.026315974	0.601748232	0.206324451
Tmed4	blue	0.797209782	0.057516049	0.913698789	0.010850467	-0.637081992	0.173664349	0.468796757	0.34831869
1190002N15Rik	blue	-0.796871102	0.057701337	-0.970270919	0.001312259	0.652545674	0.160113636	-0.522993123	0.287035327
Pigyl	blue	0.79624	0.0580428	0.9284	0.00748	-0.725812	0.1024612	0.570231306	0.237362313

		8168	54	94183	6815	975	99		
Hmgb1-ps2	blue	0.79517<br>0863	0.0586356<br>62	0.8178<br>47439	0.04674<br>7463	-0.880440<br>345	0.0205872<br>43	0.581008886	0.226552641
Prdx5	blue	0.79490<br>2093	0.0587839<br>9	0.9605<br>92188	0.00229<br>8864	-0.620061<br>279	0.1891074<br>18	0.518058814	0.292431369
Mfap3	blue	-0.7942<br>61181	0.0591383<br>88	-0.8832<br>81734	0.01963<br>9695	0.834892<br>962	0.0386400<br>65	-0.540708145	0.267979932
Bloc1s1	blue	0.79354<br>2697	0.0595368<br>45	0.9775<br>1917	0.00075<br>2401	-0.748196<br>73	0.0871245<br>51	0.556906385	0.251001211
Gm3226	blue	0.79327<br>3997	0.0596861<br>76	0.9587<br>72373	0.00251<br>4538	-0.692750<br>883	0.1271005<br>61	0.437818086	0.38523438
Rprd1a	blue	-0.7931<br>02574	0.0597815<br>36	-0.9113<br>53121	0.01143<br>9098	0.733386<br>81	0.0971481<br>11	-0.453536385	0.366340563
R3hcc1	blue	0.79271<br>5395	0.0599971<br>72	0.9650<br>73955	0.00180<br>8441	-0.827741<br>312	0.0419538<br>63	0.568228018	0.239393579
Gm9800	blue	0.79258<br>7485	0.0600684<br>89	0.9635<br>00277	0.00197<br>4032	-0.815803<br>454	0.0477678<br>07	0.580736234	0.226823624
Spcs1	blue	0.79236<br>9807	0.0601899<br>46	0.7364<br>01451	0.09506<br>8326	-0.661352<br>571	0.1526047<br>25	0.483164285	0.331650375
Rpl17-ps5	blue	0.79209<br>5237	0.0603433<br>07	0.9225<br>6277	0.00876<br>261	-0.846488<br>606	0.0335398<br>17	0.591225907	0.216492077
Tub	blue	-0.7919<br>30271	0.0604355<br>36	-0.7505<br>31466	0.08558<br>9043	0.626977<br>865	0.1827660<br>92	-0.381748234	0.455194061
Adipor2	blue	0.79182<br>8792	0.0604923<br>02	0.6420<br>06243	0.16929<br>9139	-0.793754<br>552	0.0594192<br>27	0.634494407	0.175976769
Dapk1	blue	0.79067<br>1297	0.0611415<br>23	0.8592<br>28091	0.02833<br>0276	-0.863640<br>474	0.0266231<br>51	0.792790318	0.059955416
Psmc2	blue	0.79060<br>2304	0.0611803<br>21	0.9175<br>34336	0.00992<br>0471	-0.783547<br>001	0.0652072<br>34	0.48387901	0.330828933
Eno3	blue	0.79044<br>6391	0.0612680<br>38	0.9223<br>01035	0.00882<br>1154	-0.855426<br>604	0.0298413<br>02	0.572611798	0.234957505
Slc2a1	blue	0.79032<br>2952	0.0613375<br>27	0.8226<br>45819	0.04439<br>2464	-0.825197<br>923	0.0431630<br>44	0.478246218	0.337322778
Trmt112-ps<br>2	blue	0.78995<br>8883	0.0615426<br>86	0.8576<br>94041	0.02893<br>5561	-0.746291<br>867	0.0883864<br>06	0.7930597	0.059805396
Ak5	blue	0.78977<br>3237	0.0616474<br>21	0.9127<br>12469	0.01109<br>6143	-0.611959<br>855	0.1966481<br>29	0.594371468	0.213431814
Gm10145	blue	0.78925<br>4426	0.0619405<br>5	0.9284<br>98172	0.00748<br>599	-0.767849<br>253	0.0745851<br>91	0.693107867	0.126822195
Fth1	blue	0.78898<br>0793	0.0620954<br>1	0.9628<br>25849	0.00204<br>719	-0.616167<br>096	0.1927169<br>38	0.422882096	0.403488703
Gm17383	blue	0.78741<br>5257	0.0629848<br>16	0.9557<br>01641	0.00290<br>0053	-0.664082<br>892	0.1503079<br>61	0.406212556	0.424195457
Ctbp1	blue	-0.7871	0.0631375	-0.9468	0.00415	0.859696	0.0281465	-0.638915128	0.172033891

		47464	33	76819	815	737	73		
Nkain4	blue	0.786606142	0.063446757	0.900483488	0.014362522	-0.888471893	0.017964156	0.736375146	0.095086387
Atp6v0a1	blue	-0.786563139	0.063471351	-0.93720399	0.005791195	0.878382434	0.021286836	-0.626077268	0.183586711
Ddit4	blue	0.786538839	0.063485251	0.942332502	0.004892423	-0.766281442	0.075553182	0.446536245	0.374714094
Gm45533	blue	0.786040258	0.06377075	0.717725717	0.108272522	-0.785753606	0.063935159	0.539745272	0.269002727
2900052N01Rik	blue	0.785464226	0.064101329	0.947547869	0.004054685	-0.65011541	0.162212539	0.465700231	0.35194942
Gm29216	blue	0.785405935	0.064134825	0.899055318	0.014770439	-0.785143911	0.064285493	0.452931433	0.367061587
Gm10291	blue	0.785209373	0.064247836	0.982321104	0.000466052	-0.73714759	0.094556665	0.512170461	0.298920223
Rab24	blue	0.784850685	0.064454294	0.850919061	0.031681018	-0.60703325	0.201292773	0.498788965	0.313863514
Rpl38	blue	0.784136217	0.064866438	0.957238586	0.002703712	-0.817043291	0.047147667	0.563114136	0.244609847
mt-Tp	blue	0.783777712	0.065073696	0.9301438	0.007149388	-0.820225883	0.045573064	0.502315027	0.309899621
Ewsr1	blue	0.783418009	0.06528195	0.841464348	0.035708047	-0.843283144	0.034915763	0.76534006	0.076137123
Ptpn11	blue	0.78264755	0.065729039	0.784362706	0.064735657	-0.646564339	0.165300129	0.51183111	0.299295811
Uqcrq	blue	0.782523666	0.065801058	0.969436921	0.001386878	-0.630247082	0.179800035	0.458519779	0.360420019
Asna1	blue	0.782172149	0.066005605	0.786197546	0.06368062	-0.427043947	0.398373342	0.561686996	0.246073462
Clybl	blue	0.782022829	0.066092582	0.889445933	0.017657696	-0.703877758	0.118549333	0.416998727	0.410757434
Cd302	blue	-0.781085646	0.066639671	-0.860898586	0.027678054	0.880529403	0.02055722	-0.550094947	0.258088169
Gm27529	blue	0.780931361	0.066729934	0.966749357	0.001640027	-0.747523673	0.087569481	0.581227488	0.22633547
Gm4735	blue	0.779700332	0.06745213	0.850026098	0.032051637	-0.55824045	0.249622231	0.664581011	0.14989054
Cyfip2	blue	-0.779546525	0.067542612	-0.898913504	0.014811244	0.734558734	0.096337227	-0.567761897	0.239867192
Gm10736	blue	0.779532835	0.067550668	0.888862489	0.01784096	-0.897639019	0.015180398	0.576689737	0.23086055
Nprl2	blue	0.779215803	0.067737357	0.911908632	0.011298335	-0.751784283	0.084770149	0.405369	0.425252434
Hepacam	blue	0.77911	0.0677949	0.8496	0.03221	-0.674590	0.1416076	0.542340122	0.266249829

		8023	83	38457	3159	908	56		
Kcnj3	blue	-0.778592656	0.06810499	-0.900856626	0.014256852	0.644537677	0.167073247	-0.667553021	0.147410306
Rpl31-ps9	blue	0.777697004	0.068634979	0.969685725	0.001364504	-0.727352531	0.101371115	0.466470502	0.351045008
Frzb	blue	0.777621471	0.06867976	0.897769792	0.015142318	-0.614839191	0.193954192	0.513648136	0.297286822
Arrdc2	blue	0.777252892	0.06889847	0.814311073	0.048519254	-0.808354418	0.051572661	0.448772837	0.372031509
Gm13394	blue	0.77602544	0.069629108	0.928835173	0.007416444	-0.732823351	0.097539059	0.455752387	0.363703638
Fuca1	blue	0.775886605	0.06971197	0.583050255	0.224527884	-0.589615044	0.21806606	0.50042347	0.31202373
Gtf2f1	blue	0.775482686	0.069953299	0.733691142	0.096937245	-0.789736544	0.061668132	0.859688586	0.028149763
Flna	blue	-0.775335969	0.070041052	-0.896942918	0.015383871	0.549057302	0.259174531	-0.387939014	0.447283246
Wbp2	blue	0.774416289	0.070592263	0.951363959	0.003490673	-0.587061306	0.220570732	0.483097937	0.331726666
Ost4	blue	0.773691544	0.071028024	0.854307375	0.030293251	-0.52152654	0.288635174	0.607799343	0.200567615
Ppp2r5e	blue	-0.773591726	0.071088136	-0.920590286	0.009208479	0.880807889	0.020463472	-0.646600124	0.165268893
Particl	blue	0.772994282	0.07144841	0.816623794	0.047357069	-0.547142285	0.26118411	0.380952456	0.456214135
Nbdy	blue	0.772858799	0.071530225	0.652399256	0.160239764	-0.777244809	0.06890327	0.533726405	0.275430079
Ppm1e	blue	-0.772829185	0.071548115	-0.889415801	0.017667138	0.847752934	0.033004273	-0.735153926	0.095926557
Scnm1	blue	0.772557444	0.07171236	0.63547358	0.17510022	-0.726915107	0.101680336	0.491017057	0.322665969
Dpm2	blue	0.771397543	0.072415348	0.848684345	0.032612347	-0.517669711	0.292858497	0.520069524	0.290227917
Atox1	blue	0.771313351	0.072466495	0.877212151	0.021689656	-0.567538757	0.24009405	0.351701678	0.494199188
Rin1	blue	-0.770539377	0.072937466	-0.926438938	0.007917817	0.828964912	0.041377857	-0.671782736	0.143910998
Plcb1	blue	-0.77052019	0.072949159	-0.901928086	0.013955518	0.719051105	0.107310453	-0.703549668	0.118797756
Zfp106	blue	-0.770140408	0.073180783	-0.937515178	0.005734548	0.754071657	0.08328416	-0.430779777	0.393800498
Gm14760	blue	0.769910607	0.073321097	0.965044911	0.001811432	-0.730473536	0.099176963	0.468426353	0.348752286
Gm6807	blue	0.76981	0.0733806	0.9602	0.00233	-0.755278	0.0825050	0.520491658	0.289766118

		3102	69	41848	9643	215	87		
Gm2225	blue	0.769786252	0.073397078	0.974170047	0.000992163	-0.708675854	0.114942332	0.447762208	0.373242834
Map4	blue	-0.769716825	0.073439513	-0.866454603	0.02556071	0.871202887	0.023814756	-0.739798078	0.09274907
Ube4a	blue	-0.76913246	0.073797132	-0.721087946	0.105839347	0.706045008	0.116914049	-0.486604375	0.327703458
Rims1	blue	-0.768412407	0.074238873	-0.975928723	0.000862166	0.727120132	0.101535349	-0.501105754	0.311256945
Gpd2	blue	-0.768273926	0.074323965	-0.899073287	0.014765272	0.804444607	0.053623661	-0.515365349	0.295392867
Pclo	blue	-0.767461081	0.074824328	-0.897322536	0.015272746	0.661996594	0.152061634	-0.489304858	0.324617212
Camsap2	blue	-0.766433461	0.075459076	-0.940205976	0.005256096	0.806291296	0.052650321	-0.582627434	0.224946667
Stmn4	blue	0.766410019	0.075473584	0.966494131	0.001665157	-0.757211618	0.081263572	0.464201378	0.353711667
Fam162a	blue	0.765760831	0.075875866	0.881731074	0.020154163	-0.604934322	0.203285024	0.612862646	0.195801827
Ndufs6	blue	0.763948949	0.077003754	0.95849418	0.002548348	-0.686333528	0.132149686	0.566442396	0.241209906
Cox7b	blue	0.762018836	0.078213516	0.952972992	0.003265308	-0.773900766	0.070902101	0.496187924	0.316799457
Srp14	blue	0.761863831	0.07831104	0.965063391	0.001809529	-0.650550495	0.161835929	0.416625331	0.411220222
Gm10051	blue	0.761469259	0.07855954	0.904960952	0.013119415	-0.74932821	0.08637887	0.640365363	0.170748563
Tceal3	blue	0.760464484	0.079193949	0.780520092	0.066970815	-0.807725529	0.051900065	0.453019111	0.366957054
Rpl35a-ps6	blue	0.758989865	0.080129184	0.90362252	0.013485321	-0.746049558	0.088547504	0.41407794	0.414382103
Ube2n-ps1	blue	0.758554272	0.080406394	0.947525282	0.004058147	-0.77406262	0.070804758	0.495727144	0.317320617
Ogt	blue	-0.75851694	0.080430172	-0.86140731	0.027480861	0.477162139	0.338577813	-0.399457058	0.432684284
Gm4617	blue	0.758029752	0.08074077	0.949011949	0.003833393	-0.71979688	0.106770778	0.40323948	0.427924569
2610507B11Rik	blue	-0.757619416	0.081002792	-0.957316134	0.002693986	0.670549387	0.144927653	-0.449439528	0.371233177
Skp1a	blue	0.757138432	0.081310413	0.872061554	0.023505305	-0.472689801	0.343773175	0.434361513	0.389433207
Ptma	blue	0.757076391	0.08135013	0.792767367	0.059968206	-0.80213168	0.054854351	0.805156658	0.053247383
Tsc22d4	blue	0.75699	0.0814040	0.7643	0.07673	-0.816871	0.0472334	0.469852071	0.347084392

		2151	73	75056	7827	248	94		
Smap1	blue	0.756821706	0.081513267	0.82468362	0.043409502	-0.788720385	0.06224295	0.530826017	0.278548059
Anp32a	blue	0.75581305	0.082160799	0.82417185	0.043655396	-0.737695776	0.094181534	0.713886568	0.111080593
Mfge8	blue	0.755520391	0.082349112	0.89025741	0.017404315	-0.682369216	0.135311196	0.568977713	0.238632612
Cfl1	blue	0.755007134	0.082679836	0.951377677	0.003488721	-0.644214638	0.167356607	0.36617571	0.475285706
Cdkl2	blue	-0.754632756	0.082921449	-0.859826081	0.028095972	0.761106957	0.07878803	-0.739911339	0.092672174
Tbc1d22a	blue	0.753963655	0.083354057	0.903773414	0.013443826	-0.552198362	0.255891456	0.510202608	0.301100667
Gm2451	blue	0.753368171	0.08373992	0.918926771	0.009592861	-0.542194863	0.266403645	0.375712522	0.462948988
Gm11703	blue	0.753357892	0.083746587	0.941457942	0.005040442	-0.725798096	0.10247186	0.405076101	0.425619638
Gm13341	blue	0.7533007	0.08378369	0.924958331	0.008235589	-0.735839701	0.095454355	0.473459163	0.342877406
Rpl9-ps6	blue	0.752775991	0.084124436	0.93588929	0.006033521	-0.802305901	0.0547612	0.51015819	0.301149952
Celf3	blue	-0.752094875	0.08456768	-0.818612394	0.046368237	0.675487638	0.140875491	-0.328732159	0.524663955
Asrgl1	blue	-0.752055862	0.0845931	-0.888275008	0.018026409	0.782152181	0.066017233	-0.425309998	0.400501867
Pcbd2	blue	0.751967659	0.084650583	0.912241411	0.011214415	-0.640791528	0.170371624	0.325739212	0.528672629
Gm17241	blue	0.751406189	0.085016914	0.928855995	0.007412158	-0.728227444	0.100753879	0.590077652	0.217613573
Gm10155	blue	0.751368682	0.085041411	0.937755249	0.005691033	-0.782705484	0.065695373	0.498628779	0.314044032
44626	blue	-0.751077215	0.085231884	-0.964590268	0.001858574	0.681221674	0.136232365	-0.450692172	0.369734813
Xpot	blue	-0.749933167	0.085981364	-0.911663683	0.0113603	0.534714204	0.274371244	-0.447152357	0.373974455
Ndufb7	blue	0.748823074	0.086711415	0.831957151	0.039984968	-0.556685256	0.251230071	0.589073909	0.218595836
Ndufa3	blue	0.748585981	0.086867698	0.969002306	0.001426393	-0.719023902	0.107330162	0.470299244	0.346561852
Rb1	blue	-0.748527693	0.086906138	-0.923717234	0.008506644	0.611862056	0.196739898	-0.508578065	0.30290518
Gm8054	blue	0.74837299	0.087008201	0.962842092	0.002045413	-0.69541582	0.125028915	0.483687585	0.331048868
Tmub2	blue	-0.7481	0.0871657	-0.8180	0.04665	0.437843	0.3852041	-0.374703474	0.464249477

		34414	03	2385	988	019	5		
Gm6745	blue	0.746880507	0.0879956	0.953988207	0.003126922	-0.793295157	0.05967441	0.538550819	0.2702736
Hmgcl	blue	0.746812052	0.088041008	0.899314486	0.014696006	-0.807123467	0.052214401	0.699052908	0.122225467
Rpl28	blue	0.746491486	0.088253789	0.944507491	0.004533685	-0.754063216	0.083289622	0.56298467	0.24474248
Eif5a	blue	0.746411001	0.088307248	0.964069616	0.001913296	-0.628067842	0.181774951	0.437987795	0.385028631
Secisbp2l	blue	-0.745299923	0.089046726	-0.932082221	0.006762591	0.685804014	0.132570101	-0.604456042	0.203740115
Polr2j	blue	0.745081174	0.089192639	0.771395264	0.072416732	-0.74505814	0.08920801	0.824074408	0.043702289
Dcaf5	blue	-0.744488317	0.089588635	-0.857364123	0.029066527	0.677658105	0.139110092	-0.322350322	0.533222184
Jcad	blue	-0.743644543	0.090153581	-0.914089451	0.010753897	0.693529819	0.12649351	-0.58737739	0.220260097
Lrrn3	blue	-0.742684657	0.090798201	-0.952128942	0.003382606	0.69191007	0.127757251	-0.43377849	0.390142965
Tpm1	blue	0.742622644	0.090839918	0.935348791	0.006134554	-0.727578761	0.101211353	0.430808815	0.393765025
Gm5160	blue	0.742187079	0.091133163	0.949114532	0.003818117	-0.77905603	0.06783153	0.495171496	0.317949497
Gm14305	blue	-0.741773374	0.091412082	-0.865602661	0.025880182	0.864877817	0.026153476	-0.737774674	0.094127597
Sec61g	blue	0.741576817	0.091544734	0.900445456	0.014373313	-0.81127608	0.050064214	0.633318325	0.177032001
AK157302	blue	0.741495548	0.091599605	0.956013918	0.002859612	-0.688040096	0.130798663	0.404482333	0.42636436
Naaa	blue	0.741061223	0.091893105	0.793929044	0.059322432	-0.858358992	0.028672449	0.780795908	0.066809225
Map1b	blue	-0.740979955	0.091948069	-0.906146245	0.012799434	0.65335991	0.159413004	-0.317718764	0.539457948
Kcnq3	blue	-0.740314258	0.092398854	-0.898102295	0.015045704	0.770725527	0.072824065	-0.682508906	0.135199247
Gm21981	blue	0.740068674	0.092565403	0.956166188	0.002839993	-0.739227891	0.093136615	0.533523216	0.275648074
Man1a2	blue	-0.739390008	0.093026353	-0.844031112	0.034592368	0.591819358	0.215913373	-0.67084661	0.14468237
Thyn1	blue	0.739037664	0.093266069	0.780906363	0.066744564	-0.843187523	0.034957208	0.484814057	0.329755394
Thra	blue	0.73852767	0.093613527	0.86885424	0.024671014	-0.565243197	0.242432769	0.538286908	0.270554707
Tnnc2	blue	0.73818	0.0938440	0.8789	0.02110	-0.826756	0.0424201	0.521811955	0.28832356

		9749	71	12746	5486	565	29		
Gm10156	blue	0.738032314	0.093951565	0.901129533	0.014179806	-0.810277674	0.050577356	0.441422322	0.380872896
Nop10	blue	0.737975929	0.093990078	0.953086454	0.003249696	-0.694724859	0.125564628	0.481209187	0.333901168
Rpl37rt	blue	0.737147029	0.094557049	0.93880203	0.005503188	-0.772780684	0.071577417	0.596602296	0.211272166
Ckm	blue	0.736518605	0.094987907	0.895565477	0.015790343	-0.821758848	0.04482351	0.49348353	0.319862738
Tnnt3	blue	0.736435452	0.095044983	0.899718988	0.014580195	-0.817044235	0.047147196	0.483618448	0.331128315
Atp6v1g1	blue	0.735925876	0.095395091	0.89804525	0.015062258	-0.737803766	0.094107713	0.42310725	0.403211401
B4galt6	blue	-0.73588942	0.095420161	-0.949185384	0.003807583	0.608741891	0.199676897	-0.386339954	0.449322341
Brms1l	blue	-0.735393803	0.095761268	-0.817005268	0.047166629	0.835605986	0.038316681	-0.783545845	0.065207903
Slc39a7	blue	-0.735179876	0.095908669	-0.841012362	0.035906233	0.829395427	0.041176079	-0.482510232	0.332402733
Dynll2	blue	0.734343939	0.096485623	0.903904893	0.01340772	-0.810330152	0.050550324	0.509508307	0.301871391
Osbp	blue	-0.733739296	0.096903899	-0.795824532	0.058275638	0.718251465	0.107890441	-0.341849043	0.507200807
Ccdc124	blue	0.733358531	0.097167715	0.872628917	0.023301893	-0.42754818	0.397755088	0.378753901	0.459036127
Ube2o	blue	-0.733098765	0.09734788	-0.878198946	0.021349756	0.772886788	0.07151332	-0.658279322	0.155207655
Cntn1	blue	-0.732060451	0.098069498	-0.875102807	0.022424808	0.800249356	0.055865423	-0.712891275	0.11181374
Cmtm6	blue	-0.731544388	0.098429038	-0.877069227	0.021739099	0.47220704	0.344335682	-0.379351619	0.458268372
Smim26	blue	0.731379959	0.098543719	0.924586359	0.008316379	-0.771870403	0.072128382	0.617562337	0.191420457
Myh4	blue	0.73080542	0.098944893	0.889942751	0.017502358	-0.809654703	0.050898767	0.487876803	0.326247935
Rab9b	blue	-0.730506828	0.099153672	-0.850415807	0.031889641	0.710322755	0.113715529	-0.7293469	0.099966562
Mylpf	blue	0.729982548	0.099520728	0.889755645	0.017560782	-0.826731887	0.042431845	0.495949619	0.317068949
Nrcam	blue	-0.729411322	0.099921337	-0.952889268	0.003276852	0.625468066	0.184142676	-0.358309621	0.485536499
Tmem136	blue	-0.728384839	0.100643017	-0.543410426	0.265117523	0.616826571	0.192103694	-0.458134715	0.360876283
Prkcsh	blue	0.72834	0.1006716	0.8993	0.01468	-0.520555	0.2896957	0.415894758	0.412126198

		4166	6	36621	9657	966	92		
Lmo7	blue	-0.728018568	0.100901086	-0.926307598	0.007945759	0.670474228	0.144989705	-0.581916431	0.225651583
Sec14l2	blue	0.727932478	0.100961786	0.957268445	0.002699965	-0.616041184	0.192834115	0.398478646	0.433918293
Pdk2	blue	0.727786789	0.101064545	0.776735523	0.069206005	-0.551023746	0.257117271	0.306750208	0.554306624
Gabra1	blue	-0.727621004	0.101181534	-0.946313403	0.004246007	0.692318797	0.127437844	-0.54197269	0.266638976
Gm9385	blue	0.727156166	0.101509877	0.919086378	0.00955565	-0.776527881	0.069329607	0.627632778	0.182170296
Atp2a1	blue	0.726163603	0.102212559	0.889494271	0.017642553	-0.810563576	0.050430165	0.490989792	0.322697006
Rpl37a	blue	0.726126565	0.102238822	0.946503531	0.004216258	-0.749250822	0.086429779	0.490303022	0.323479168
Acta1	blue	0.725817079	0.102458386	0.89723738	0.01529764	-0.807975647	0.051769737	0.483519765	0.331241725
H3f3c	blue	0.725448749	0.102719969	0.935652563	0.006077671	-0.709602286	0.114251508	0.394294851	0.439207924
Gm11353	blue	0.724316162	0.103526164	0.921340121	0.009037716	-0.689541885	0.129614727	0.460389953	0.358206947
Usmg5	blue	0.72385928	0.10385217	0.941425293	0.00504601	-0.627658726	0.182146707	0.452730875	0.367300735
Gm48583	blue	0.723678722	0.103981132	0.908147675	0.012267802	-0.785887531	0.063858322	0.433211072	0.390834151
Kalrn	blue	-0.723550343	0.104072868	-0.950104812	0.003672187	0.612088075	0.196527841	-0.349603332	0.496959697
Gm13340	blue	0.723372351	0.104200115	0.93529819	0.006144055	-0.764277039	0.076798961	0.440027635	0.382558574
Kif5c	blue	-0.722474144	0.104843297	-0.898763927	0.014854342	0.792350477	0.060200737	-0.472390272	0.344122144
Rpl36a-ps1	blue	0.722191313	0.105046187	0.862312046	0.027131817	-0.74531091	0.0890394	0.585042369	0.22255901
Ciao2b	blue	0.721665186	0.105424067	0.824403345	0.043544088	-0.809168341	0.051150352	0.412867586	0.415887252
Pvalb	blue	0.721564558	0.105496409	0.863461321	0.026691484	-0.773741179	0.070998141	0.473058423	0.343343883
Rps27rt	blue	0.721094359	0.105834729	0.94987531	0.003705758	-0.753727901	0.083506725	0.474198682	0.342017176
Unc80	blue	-0.720887018	0.105984068	-0.906836204	0.012614932	0.548993934	0.259240931	-0.334752574	0.516627206
Extl2	blue	0.72064303	0.106159922	0.879032126	0.021064765	-0.636210599	0.174441651	0.284032771	0.585407961
Mcts2	blue	0.72033	0.1063853	0.7888	0.06214	-0.826984	0.0423121	0.445159318	0.376368926

		0532	41	89651	703	24	13		
Rps21	blue	0.720261289	0.106435319	0.920491848	0.009231012	-0.74470871	0.089441333	0.483205621	0.331602847
Gstm5	blue	0.719520137	0.106970902	0.87947541	0.020913884	-0.551513233	0.256606171	0.573494202	0.234068556
Rpl35a-ps4	blue	0.719366564	0.107082027	0.917284432	0.009979833	-0.66313724	0.151101772	0.409017434	0.420687188
Rpl21-ps8	blue	0.718995799	0.107350523	0.850234123	0.031965116	-0.640939678	0.170240669	0.497560194	0.315249239
Gm7536	blue	0.718715048	0.107554032	0.927406693	0.007713407	-0.799435242	0.056305352	0.47932496	0.336075594
Gabarapl2	blue	0.718586108	0.107647553	0.745333913	0.089024063	-0.705722047	0.117157106	0.737277857	0.094467461
Gm24865	blue	0.717950431	0.108109141	0.685509701	0.132804025	-0.694587069	0.125671578	0.804807118	0.053431942
Gm5940	blue	0.717690268	0.108298306	0.900230727	0.014434315	-0.840862387	0.035972107	0.620987536	0.188253017
Myl1	blue	0.716809923	0.108939486	0.867422088	0.0252002	-0.821243444	0.045074874	0.496891312	0.316004506
Ap1s1	blue	0.716681113	0.109033442	0.72256648	0.104777097	-0.34473846	0.503377464	0.500054277	0.312438941
Ndufa7	blue	0.716291931	0.109317533	0.953028457	0.003257672	-0.730741511	0.098989562	0.469701667	0.34726021
Rpp25	blue	0.716281686	0.109325016	0.89908398	0.014762198	-0.615396758	0.193434292	0.371218141	0.468750258
Gtpbp6	blue	0.715950053	0.109567362	0.954806122	0.003017576	-0.67097893	0.144573231	0.457398687	0.361748974
Rps19-ps6	blue	0.715857218	0.109635246	0.937052924	0.005818793	-0.626645431	0.183068827	0.344245011	0.504029797
Fbl	blue	0.714668086	0.110506403	0.931789883	0.006820252	-0.752061028	0.084589733	0.550438673	0.257728697
Gm8451	blue	0.714299709	0.110776891	0.942114003	0.004929201	-0.688218738	0.130657586	0.37342552	0.465898183
Atp5mpl	blue	0.71393906	0.111041985	0.93541432	0.006122262	-0.68804229	0.13079693	0.395231524	0.438021868
Tomm7	blue	0.713894827	0.111074518	0.930723545	0.007032604	-0.569391328	0.238213189	0.302467619	0.560134447
Apex1	blue	0.71270715	0.111949602	0.9149887	0.010533197	-0.586956684	0.22067359	0.502524579	0.309664637
Rpl31-ps12	blue	0.711062746	0.11316618	0.872730436	0.023265586	-0.856150843	0.029550565	0.519807235	0.290514991
Gm13509	blue	0.710930018	0.113264628	0.763331896	0.077389585	-0.323333947	0.531900528	0.374425279	0.464608224
Camk2a	blue	-0.7108	0.1133326	-0.6149	0.19381	0.755713	0.0822250	-0.582690902	0.224883785

		3834	49	92652	1041	147	61		
Ehd1	blue	0.710571866	0.113530466	0.835489086	0.038369613	-0.755392579	0.082431413	0.717741618	0.108260958
Ndufa1	blue	0.71029803	0.113733905	0.888826562	0.017852274	-0.80485846	0.053404815	0.426165811	0.399450825
Cygb	blue	0.710195355	0.113810226	0.856609248	0.02936724	-0.686562058	0.13196842	0.687640891	0.131114159
Cops9	blue	0.710167386	0.113831019	0.858596104	0.028578903	-0.460575275	0.357987908	0.312150386	0.546982055
Cd83	blue	-0.710041584	0.11392457	-0.881154395	0.020347113	0.738551923	0.093596991	-0.669293865	0.145965727
Tnni2	blue	0.709835344	0.114078008	0.826433342	0.042573696	-0.805507088	0.05306265	0.525344552	0.284477278
Gm13456	blue	0.709637705	0.114225133	0.938078512	0.005632694	-0.760220407	0.079348405	0.472027298	0.344545199
Zmym2	blue	-0.708586688	0.115008918	-0.914649792	0.010616114	0.524252452	0.28566426	-0.391445286	0.442822537
Adcy2	blue	-0.708191901	0.115303933	-0.900551829	0.01434314	0.805265917	0.053189753	-0.486093002	0.328289081
Nceh1	blue	-0.707029204	0.116174713	-0.943120478	0.00476091	0.717769886	0.1082404	-0.502069177	0.310175391
Rps27	blue	0.704601253	0.11800232	0.941249768	0.005075994	-0.749768404	0.086089545	0.452293221	0.36782279
Rpl15	blue	0.703726873	0.118663552	0.923862141	0.008474776	-0.507972231	0.303579161	0.351628587	0.494295267
Gfra2	blue	0.703183689	0.119075137	0.822910015	0.044264447	-0.793569268	0.059522087	0.751077877	0.085231451
Trim44	blue	-0.702912752	0.119280666	-0.933449431	0.006496092	0.690259762	0.129050436	-0.395783824	0.43732301
Gm10073	blue	0.702868512	0.11931424	0.943875954	0.00463647	-0.707565467	0.115772732	0.436797946	0.386471955
Asph	blue	-0.702691299	0.119448771	-0.842500348	0.035255731	0.522114082	0.28799384	-0.579536281	0.228017773
Kif3a	blue	-0.702542591	0.119561714	-0.939300243	0.005414868	0.729145874	0.100107742	-0.51357586	0.297366637
Akt3	blue	-0.702538944	0.119564484	-0.881825274	0.020122728	0.664826137	0.149685307	-0.288406596	0.5793847
Rilpl1	blue	0.701674859	0.12022168	0.863936655	0.026510365	-0.737536076	0.09429075	0.684143797	0.133891992
Sumo1	blue	0.701612789	0.120268949	0.946173826	0.004267911	-0.624513464	0.185015259	0.403456073	0.427652535
Ncaph2	blue	-0.701031839	0.120711761	-0.759333832	0.07991059	0.672023177	0.143713159	-0.291859084	0.574641904
Tmem258	blue	0.70058	0.1210516	0.9511	0.00351	-0.687375	0.1313241	0.420658576	0.406230669

		6558	44	91024	5335	39	7		
Hrasls	blue	-0.700343562	0.121237299	-0.873279318	0.023069748	0.794038063	0.059261993	-0.669926517	0.14544225
Gpd1l	blue	-0.699837985	0.121623969	-0.923590396	0.008534585	0.756986432	0.081407736	-0.557736413	0.250142888
Kifap3	blue	-0.698264053	0.122831161	-0.829141795	0.041294898	0.858161444	0.028750497	-0.721589635	0.105478379
Rpl35a-ps3	blue	0.697996718	0.12303672	0.827836662	0.041908843	-0.812867148	0.049251481	0.466793168	0.350666398
Gnl1	blue	0.697335233	0.123545993	0.831894656	0.040013831	-0.403082141	0.428122216	0.4575949	0.361516255
Gm10036	blue	0.697132159	0.123702522	0.884124411	0.019362789	-0.790286018	0.061358326	0.534385971	0.274722907
Ndufb4c	blue	0.696935921	0.123853864	0.885273181	0.018988333	-0.793918463	0.0593283	0.443142245	0.378797673
Eif6	blue	0.696840504	0.12392748	0.833546456	0.039254228	-0.810527171	0.050448897	0.726955138	0.101652021
Tecr	blue	0.69600897	0.124569831	0.812649353	0.049362369	-0.518091398	0.292395612	0.457105252	0.362097099
Tbc1d16	blue	0.695750658	0.124769668	0.78375892	0.065084568	-0.437162091	0.386030039	0.523797813	0.286158951
Nipsnap2	blue	0.695624099	0.124867628	0.806000111	0.052803249	-0.356461981	0.487953975	0.353697065	0.491578439
Gtf2h5	blue	0.695621038	0.124869998	0.904381921	0.013277116	-0.657390726	0.155963742	0.572822665	0.234744952
Rpl15-ps2	blue	0.695501745	0.124962366	0.924024635	0.008439109	-0.670886992	0.144649059	0.511807414	0.299322044
Pygo1	blue	-0.694569324	0.125685354	-0.945161446	0.004428443	0.636805284	0.173911022	-0.401389781	0.430250036
Fam131b	blue	0.693680356	0.126376336	0.721393598	0.105619364	-0.747874044	0.087337738	0.353638649	0.491655103
Arpin	blue	-0.692434362	0.127347598	-0.946375157	0.004236333	0.687380638	0.131320018	-0.441558853	0.380708017
Lrrtm1	blue	-0.692204777	0.127526912	-0.715788858	0.109685244	0.791113469	0.060893141	-0.420088893	0.406934187
Gm5776	blue	0.691178169	0.128330073	0.932271803	0.006725325	-0.587374574	0.220262864	0.398599513	0.433765789
Gm3534	blue	0.691108366	0.128384763	0.921842954	0.008924074	-0.546287245	0.262083316	0.317003966	0.540422155
Gm7600	blue	0.691079996	0.128406993	0.93629336	0.005958526	-0.666865717	0.147982313	0.379007094	0.458710857
Acsl6	blue	-0.690974641	0.128489562	-0.941599963	0.005016258	0.680100459	0.137135	-0.428720958	0.396318374
Ndn	blue	0.69024	0.1290632	0.8063	0.05261	-0.502728	0.3094357	0.525215364	0.284617593

		3427	64	62879	2758	72	85		
Oxct1	blue	-0.689962145	0.129284249	-0.894897186	0.015989388	0.773642138	0.071057774	-0.608931819	0.199497609
Ndufb1-ps	blue	0.689758397	0.129444424	0.91425215	0.010713802	-0.752880755	0.084056353	0.413629306	0.414939795
Gm11273	blue	0.687818203	0.130973987	0.924403705	0.008356191	-0.711803163	0.112617683	0.408582472	0.421230596
Id4	blue	0.68599495	0.132418438	0.811335111	0.05003395	-0.849999996	0.032062502	0.5366491	0.272301743
Uba52-ps	blue	0.68587055	0.132517242	0.802028239	0.054909692	-0.763811408	0.07708968	0.715143959	0.110157414
Ubald2	blue	0.685268468	0.132995892	0.888693409	0.017894239	-0.720204528	0.106476294	0.33080613	0.521891309
Gm10288	blue	0.684502953	0.133605546	0.804565496	0.053559692	-0.709984897	0.113966735	0.66864811	0.146500876
Pdcd5-ps	blue	0.683734678	0.134218609	0.92453622	0.008327298	-0.611000087	0.197549483	0.437177676	0.38601113
Fbxw4	blue	0.68334759	0.134527952	0.825636332	0.042953471	-0.756104896	0.081973204	0.716820384	0.108931857
Fam168b	blue	-0.680310349	0.136965833	-0.8175333	0.046903613	0.623333466	0.18609623	-0.292791467	0.573362844
Sncb	blue	0.6796913	0.137465033	0.763918631	0.077022691	-0.438549589	0.384347804	0.475227961	0.340821183
Gm9616	blue	0.67934328	0.137746018	0.904961793	0.013119186	-0.787833234	0.062746789	0.478130447	0.337456726
Gm17018	blue	0.678020108	0.138816579	0.905663479	0.0129293	-0.668264896	0.146818846	0.307170319	0.553735835
Gm26644	blue	0.677596051	0.139160432	0.926978655	0.007803496	-0.660645496	0.153201936	0.448921803	0.371853078
Gm15421	blue	0.677234255	0.139454088	0.881489062	0.02023503	-0.684840001	0.133336974	0.369944519	0.47039833
Snord14e	blue	0.675792866	0.140626649	0.831886231	0.040017723	-0.572500122	0.235070103	0.606154576	0.202125815
Gm24616	blue	-0.675682291	0.140716776	-0.819798885	0.045782877	0.436655313	0.386645098	-0.252530241	0.629256757
Cds2	blue	-0.675500931	0.14086465	-0.816582037	0.047377936	0.777545334	0.068724913	-0.36241447	0.480178824
Phospho2	blue	-0.675294518	0.141033033	-0.869749136	0.02434306	0.628987681	0.180940263	-0.380939406	0.45623087
Nipsnap1	blue	0.675031303	0.141247877	0.806042201	0.052781132	-0.380565616	0.456710271	0.381847745	0.455066553
Gm4963	blue	0.674939974	0.141322456	0.90790872	0.012330702	-0.65894715	0.15464044	0.317373185	0.539924046
Tjp1	blue	-0.6746	0.1415992	-0.9107	0.01159	0.564241	0.2434558	-0.299417854	0.564294782

		01149	85	36322	638	742	54		
Srsf1	blue	0.674125944	0.141987932	0.779156349	0.067772393	-0.87099997	0.023888166	0.501216486	0.311132565
Rpl28-ps1	blue	0.670993722	0.144561032	0.75176752	0.084781082	-0.69472994	0.125560685	0.676664041	0.13991745
Gm12191	blue	0.670760331	0.144753554	0.843692583	0.034738561	-0.659247806	0.154385367	0.25477927	0.626100272
Eno1b	blue	0.669098223	0.146127771	0.887988518	0.018117178	-0.50724458	0.304389401	0.460201141	0.358430158
Gm5451	blue	0.668402944	0.146704268	0.891218818	0.017106396	-0.6964048	0.124263876	0.363033044	0.479373039
Tmed9	blue	0.668313797	0.146778255	0.892437406	0.016732333	-0.620348425	0.188842377	0.542414763	0.266170803
Vldlr	blue	-0.66700979	0.14786233	-0.898318004	0.014983186	0.560464894	0.247329527	-0.268534034	0.606881015
AC123659.1	blue	0.666865268	0.147982687	0.83150167	0.040195555	-0.656902819	0.156379555	0.660862853	0.153018246
Gm15542	blue	0.666201821	0.148535736	0.755850965	0.082136417	-0.752066353	0.084586263	0.402435625	0.428934679
Cox5b	blue	0.664625573	0.149853222	0.932740097	0.006633704	-0.600373007	0.207642039	0.328050424	0.525576279
Gm13414	blue	0.66369923	0.150629805	0.896715017	0.015450771	-0.66595456	0.148742077	0.350848091	0.495321578
Sertad4	blue	-0.663217427	0.151034391	-0.863611187	0.026634316	0.761771004	0.07836947	-0.438793733	0.38405206
Gm29667	blue	0.662975495	0.151237724	0.913282663	0.010953792	-0.710045147	0.11392192	0.470889931	0.345872041
Naa25	blue	-0.662353662	0.151760881	-0.813264929	0.049049255	0.624254352	0.185252402	-0.523107002	0.286911242
Dhrs1	blue	0.661342702	0.152613055	0.85468449	0.030140612	-0.499410166	0.313163824	0.393050867	0.440784714
Dpysl2	blue	0.659999007	0.15374884	0.903798273	0.013436996	-0.583140983	0.224438064	0.407089359	0.423097741
Pet100	blue	0.659952953	0.153787832	0.918722513	0.009640584	-0.722165303	0.105064854	0.42881609	0.396201911
Llph	blue	0.659129253	0.154485925	0.896107737	0.015629718	-0.604521845	0.203677477	0.466998575	0.350425453
Large1	blue	-0.657713066	0.15568929	-0.765866362	0.075810406	0.796430347	0.057942881	-0.758626676	0.080360287
Gm8942	blue	0.65660613	0.156632634	0.910580036	0.011636397	-0.733154897	0.097308936	0.39434137	0.439148996
Rplp1	blue	0.656280624	0.156910493	0.929593773	0.007261052	-0.647586276	0.164409062	0.357933511	0.486028309
Gpx4-ps2	blue	0.65624	0.1569381	0.9167	0.01010	-0.593739	0.2140456	0.391618008	0.44260317

		829	06	6938	2721	103	18		
Rpsa-ps2	blue	0.655547073	0.157537434	0.910216936	0.011729627	-0.705285849	0.117485736	0.442758421	0.379260446
Tma7	blue	0.655346639	0.157708923	0.842523314	0.035245735	-0.542252135	0.266342995	0.453817904	0.366005199
2410006H16Rik	blue	0.654905591	0.158086554	0.872967368	0.023180953	-0.760407821	0.079229794	0.418488686	0.408912515
1500009C09Rik	blue	0.654535165	0.158404014	0.911942354	0.011289818	-0.585695519	0.221914995	0.386172306	0.449536296
Rpl3	blue	0.650623055	0.161773157	0.806685987	0.052443359	-0.782591985	0.065761337	0.606912556	0.201407116
Usf2-ps1	blue	0.65059224	0.161799815	0.830666555	0.040583005	-0.827822485	0.041915535	0.455687009	0.363781339
Gm5844	blue	0.649917433	0.16238403	0.904738039	0.013180018	-0.661017849	0.152887316	0.309815382	0.55014583
Rpl35a-ps2	blue	0.649721687	0.162553664	0.813211851	0.049076217	-0.575652907	0.231899496	0.203121474	0.699508016
Atp5l	blue	0.649264024	0.162950567	0.921687584	0.008959113	-0.688015395	0.130818174	0.389099797	0.445804897
Prrt3	blue	-0.647904149	0.164132309	-0.776281964	0.069476122	0.834783841	0.038689667	-0.71985505	0.106728735
Gm13461	blue	0.647806624	0.164217197	0.815438962	0.047950834	-0.610962617	0.197584707	0.220847642	0.674114313
Gm4149	blue	0.646943854	0.164968979	0.914484139	0.010656757	-0.653244027	0.159512639	0.358981	0.484658966
Gm41041	blue	0.645915643	0.165866805	0.847192787	0.033241039	-0.583803541	0.223782569	0.477016901	0.338746084
Ndufa4	blue	0.644647234	0.166977193	0.921541436	0.008992134	-0.65627265	0.156917303	0.343358731	0.505202079
Itm2b	blue	-0.642360366	0.168987015	-0.73416325	0.096610534	0.465528953	0.352150638	-0.579506431	0.22804751
Wtap	blue	-0.642233673	0.169098655	-0.754856289	0.082777149	0.56192398	0.245830184	-0.174516243	0.740883162
Gm1821	blue	0.641619359	0.169640415	0.907216874	0.012513691	-0.676271218	0.140237046	0.418968461	0.408319033
Apc	blue	0.641380589	0.169851182	0.723622543	0.104021271	-0.26760141	0.608179422	0.346211353	0.501431815
Pds5b	blue	-0.640480392	0.170646787	-0.865443946	0.025939906	0.508391861	0.303112269	-0.397991263	0.434533426
Arl15	blue	-0.639809901	0.171240386	-0.845320301	0.034038295	0.794408345	0.059056926	-0.589221892	0.218450908
Cst3	blue	0.638667875	0.172253433	0.882554457	0.019880193	-0.476302301	0.339574443	0.29737788	0.567082279
Rasa1	blue	-0.6386	0.1723104	-0.9095	0.01190	0.554907	0.2530726	-0.305132219	0.556506441

		03705	29	53665	0859	772	74		
Pdap1	blue	0.636095816	0.174544148	0.871461496	0.023721355	-0.401227578	0.430454157	0.294704416	0.570741018
Faim2	blue	0.635125568	0.175411549	0.7265336	0.10195037	-0.503899019	0.308125035	0.669224768	0.146022949
Megf9	blue	-0.635046334	0.175482463	-0.87504577	0.022444849	0.720146607	0.106518114	-0.545149398	0.263281796
Hmgn2	blue	0.63405869	0.176367406	0.863331091	0.026741208	-0.75372092	0.083511248	0.562929636	0.244798869
Pfdn1	blue	0.633857062	0.176548296	0.876258086	0.02202072	-0.574734372	0.232821456	0.39489397	0.438449174
Gm13772	blue	0.633587389	0.176790353	0.914058733	0.010761475	-0.601305684	0.206748079	0.305954141	0.555388657
Sema3e	blue	-0.633353294	0.177000588	-0.740753572	0.092101256	0.752782155	0.08412043	-0.754145092	0.083236649
Lyz2	blue	-0.632969562	0.177345432	-0.851986009	0.031240856	0.434979467	0.388681409	-0.384854063	0.451219783
Adarb1	blue	-0.631687639	0.178499472	-0.804579078	0.053552507	0.613085359	0.195593279	-0.198939983	0.705526761
mt-Tt	blue	0.628864343	0.18105209	0.770474495	0.072977009	-0.768575734	0.07413857	0.345847064	0.50191282
Xpo1	blue	-0.628279862	0.181582416	-0.839705086	0.036482344	0.439393303	0.383326104	-0.298566406	0.565457779
Uqcc2	blue	0.628195329	0.181659171	0.877023759	0.021754839	-0.493234298	0.320145591	0.32307821	0.53224406
Noct	blue	-0.627389349	0.182391657	-0.701126961	0.120639209	0.214879615	0.682641422	-0.285552519	0.583313232
Pbx2	blue	0.627060268	0.182691083	0.748935235	0.086637527	-0.472009733	0.344565677	0.58507744	0.222524411
BC002163	blue	0.626936715	0.182803554	0.892805324	0.016620177	-0.628145697	0.181704241	0.280661158	0.590062199
Gm12428	blue	0.626870242	0.182864077	0.907406692	0.012463356	-0.538380374	0.270455138	0.290241468	0.576862784
Gm19196	blue	0.62643274	0.183262624	0.769410717	0.073626746	-0.42712131	0.398278463	0.487907331	0.326213043
Ormdl3	blue	0.62628773	0.183394803	0.893128146	0.016522066	-0.483844478	0.330868605	0.290201217	0.576918076
1700066M21Rik	blue	-0.624492961	0.185034019	-0.894026632	0.016250473	0.602914201	0.205210023	-0.408809613	0.420946795
Pnck	blue	0.62446948	0.185055504	0.839024155	0.036784133	-0.590820201	0.216888062	0.555010499	0.25296604
Pcdhgb1	blue	0.623088073	0.186321357	0.89386134	0.016300275	-0.558008317	0.249861965	0.248155219	0.635407997
Gphn	blue	-0.6225	0.1868543	-0.8883	0.01800	0.486033	0.3283576	-0.352687927	0.492903319

		0757	66	50337	2578	18	14		
Mgea5	blue	-0.622386893	0.186965248	-0.824625882	0.043437212	0.481287012	0.333811467	-0.259616674	0.619324178
Asxl1	blue	-0.622002331	0.18731878	-0.742568501	0.090876346	0.612399955	0.196235379	-0.213362285	0.684813067
Rabep1	blue	-0.621817997	0.187488338	-0.876378568	0.02197878	0.631661923	0.178522654	-0.282883017	0.58699402
Acvr1b	blue	0.618067191	0.190952227	0.860364802	0.02788568	-0.604274826	0.203912653	0.397946721	0.434589657
S1pr1	blue	-0.616306703	0.192587051	-0.791080226	0.060911798	0.765039583	0.076323937	-0.399110802	0.433120864
Diras1	blue	0.61626689	0.192624088	0.867088364	0.025324279	-0.554409684	0.253589953	0.455489959	0.364015563
Atp6v1g2	blue	0.615356846	0.193471488	0.813480889	0.048939626	-0.310841655	0.548754672	0.280833156	0.589824537
Gm4294	blue	0.615303463	0.193521244	0.792350988	0.060200452	-0.700610285	0.121033523	0.561697923	0.246062242
Rasgef1a	blue	0.614471589	0.194297274	0.846858526	0.033382706	-0.390887647	0.443531007	0.284978035	0.584104834
Psmd8	blue	0.613700285	0.19501794	0.897996559	0.015076395	-0.561292707	0.246478434	0.38950983	0.445283063
Gm2574	blue	0.613576408	0.195133787	0.731041366	0.098780055	-0.264418936	0.612615335	0.252986419	0.628616207
H1fx	blue	0.611050335	0.197502252	0.854957204	0.030030457	-0.635447869	0.175123213	0.321819132	0.533936313
B230219D22Rik	blue	-0.610625865	0.197901388	-0.866106002	0.025691205	0.417727479	0.40985472	-0.31579448	0.542054765
Cnnm1	blue	-0.610375311	0.198137142	-0.891574853	0.016996695	0.53508454	0.27397468	-0.313922411	0.544584484
Cep19	blue	0.610288502	0.198218851	0.657887886	0.155540527	-0.224872937	0.668376264	0.419500795	0.407660875
Bmpr1a	blue	-0.609165505	0.199277106	-0.886756411	0.018510042	0.540536865	0.268161761	-0.305368078	0.556185619
Gm10293	blue	0.60885525	0.199569881	0.885803713	0.018816583	-0.456547123	0.362759579	0.302030068	0.560730815
Gm13493	blue	0.60835899	0.200038548	0.899448688	0.014657534	-0.596343294	0.211522448	0.260943033	0.617469422
Ccng1	blue	-0.607086117	0.201242697	-0.844913577	0.034212644	0.476033471	0.339886258	-0.443397986	0.378489436
Slc8a2	blue	0.606989361	0.201334351	0.857513768	0.029007089	-0.640290299	0.170814992	0.39531492	0.43791632
Pex19	blue	0.606212322	0.202071025	0.675180874	0.141125776	-0.558895836	0.24894587	0.729875627	0.099595659
Psmb4	blue	0.60537	0.2028620	0.9014	0.01408	-0.582721	0.2248539	0.260033869	0.618740631

		9255	35	63704	5738	022	46		
Dtd1	blue	0.603188483	0.204948222	0.672437257	0.143372719	-0.268615486	0.60676765	0.464752251	0.353063625
Gnas	blue	0.60071093	0.207317962	0.716819898	0.108932212	-0.212531468	0.686002782	0.26120215	0.617107237
Tmem47	blue	-0.60057448	0.207448797	-0.868591171	0.024767818	0.512717239	0.298315431	-0.217687152	0.678627119
Slc25a33	blue	0.600371551	0.207643435	0.773616472	0.071073231	-0.740140071	0.09251697	0.293479955	0.572418853
1810009A15Rik	blue	0.599911695	0.208084779	0.681259984	0.136201569	-0.378434586	0.459446447	0.537170713	0.271744873
Rpl38-ps2	blue	0.59970806	0.208280339	0.860695419	0.027756993	-0.587499486	0.220140153	0.252029174	0.629960523
Cluh	blue	0.597319354	0.210579878	0.693049409	0.126867761	-0.466844449	0.350606239	0.646685022	0.165194796
Selenow	blue	0.59653715	0.211335108	0.863070531	0.026840827	-0.389070285	0.445842464	0.190345461	0.717930049
Vgf	blue	-0.596198263	0.211662648	-0.690693024	0.128710385	0.238030796	0.649697059	-0.353250166	0.492165032
Zc2hc1a	blue	-0.595773836	0.212073154	-0.779108838	0.067800397	0.262723655	0.614981599	-0.188821912	0.720133233
Banf1	blue	0.595094572	0.212730809	0.893238697	0.016488532	-0.575121565	0.232432641	0.305540167	0.555951569
Gm8399	blue	0.594476093	0.213330329	0.781872298	0.066180317	-0.73332521	0.097190818	0.322875625	0.53251624
2410015M20Rik	blue	0.594179156	0.213618406	0.769778207	0.073401995	-0.415806427	0.412235782	0.491608886	0.321992516
mt-Rnr2	blue	0.594124224	0.213671715	0.825680689	0.042932293	-0.649208272	0.162998945	0.356962332	0.487298948
Rpl9-ps4	blue	0.5931374	0.214630321	0.807703239	0.051911688	-0.605689501	0.202567304	0.308533342	0.551885067
Snta1	blue	0.592421063	0.215327258	0.878371788	0.021290484	-0.514526869	0.296317078	0.180369547	0.732379676
Med30	blue	0.591746475	0.215984412	0.678165294	0.138698937	-0.770973733	0.072672986	0.348287035	0.498693728
Prpf8	blue	-0.591141892	0.216574056	-0.817581969	0.046879405	0.546510369	0.261848552	-0.334503625	0.516958815
Kbtbd7	blue	-0.589826285	0.217859394	-0.841294468	0.035782476	0.530243954	0.279175407	-0.204984514	0.696829815
Rps25-ps1	blue	0.587866264	0.219779998	0.786543123	0.0634828	-0.462606773	0.355589928	0.09446258	0.858727583
Ank3	blue	-0.586825879	0.220802215	-0.877302392	0.021658467	0.603224577	0.204913781	-0.263978876	0.61322935
Ensa	blue	0.58442	0.2231731	0.5637	0.24399	-0.601683	0.2063863	0.751022937	0.085267375

		0254	21	14367	5302	578	18		
AC115954.1	blue	0.583485375	0.224097247	0.771019483	0.072645154	-0.61594251	0.192925964	0.41879379	0.408535068
Dbpht2	blue	-0.582816963	0.22475891	-0.846648183	0.033471999	0.487969112	0.326142435	-0.154358203	0.7703016
Hunk	blue	-0.582413556	0.225158621	-0.88681465	0.018491381	0.624905383	0.184656806	-0.313753652	0.544812689
mt-Rnr1	blue	0.5818472	0.225720269	0.816362783	0.047487576	-0.622332368	0.187015357	0.316846755	0.540634287
Tnks2	blue	-0.58130988	0.22625364	-0.869576396	0.024406201	0.523253209	0.286751961	-0.198910368	0.705569426
Gm12338	blue	0.581275565	0.226287719	0.893353576	0.01645372	-0.569750694	0.237849013	0.310027111	0.549858742
Caly	blue	0.580766523	0.226793514	0.852978693	0.030833945	-0.556311375	0.251617213	0.188894719	0.720027918
2900011O08Rik	blue	0.577485424	0.230064502	0.716178396	0.109400472	-0.363643192	0.47857864	0.488043502	0.326057423
Gm15710	blue	0.576648947	0.230901389	0.883310251	0.019630294	-0.606571992	0.201729902	0.324861237	0.529850232
Slc6a7	blue	0.576264649	0.231286281	0.773523214	0.071129408	-0.334337302	0.517180398	0.186856096	0.722977915
Ntan1	blue	0.575237158	0.232316615	0.781291408	0.066519379	-0.380807673	0.456399805	0.3575002	0.486595105
Gm9803	blue	0.575180011	0.232373973	0.82370087	0.043882264	-0.507007977	0.304653032	0.116165835	0.826535047
Gramd1a	blue	-0.57471466	0.232841257	-0.79982233	0.056095979	0.545769797	0.262628075	-0.217934479	0.678273728
Syp	blue	0.574176759	0.233381857	0.666127956	0.148597364	-0.149830092	0.776936634	0.206158167	0.695143733
Hint1	blue	0.573414878	0.234148414	0.770598777	0.072901271	-0.525992985	0.2837734	0.55420792	0.253799609
Vhl	blue	0.572596823	0.234972601	0.580719562	0.226840199	-0.148992844	0.778164471	0.279107583	0.592210011
9-Sep	blue	-0.572043975	0.235530245	-0.776947241	0.06908008	0.740209594	0.092469819	-0.379039318	0.458669464
Gm5453	blue	0.572015539	0.235558942	0.867527843	0.02516094	-0.639007788	0.171951647	0.266113777	0.610251963
Manbal	blue	0.571895173	0.235680427	0.874040054	0.022799627	-0.536533554	0.272425158	0.331560978	0.520883226
Hif1a	blue	-0.571336432	0.23624469	-0.820883155	0.045250977	0.460039719	0.358621029	-0.418317032	0.40912492
Rps29	blue	0.570250514	0.23734287	0.886810497	0.018492711	-0.560163262	0.247639928	0.255440627	0.6251728
Uqcr10	blue	0.56961	0.2379897	0.8321	0.03988	-0.470264	0.3466024	0.391723548	0.442469146

		1745	96	79355	2422	458	91		
Dbi	blue	0.566717617	0.2409296	0.807680132	0.051923736	-0.690122611	0.129158162	0.248998315	0.634221495
Gm9234	blue	0.566336556	0.241317737	0.862993587	0.026870279	-0.644307974	0.167274715	0.302439114	0.560173294
Nf2	blue	-0.562926278	0.24480231	-0.763573179	0.077238612	0.753444056	0.083690703	-0.33204404	0.520238406
Ipo7	blue	-0.561578961	0.246184398	-0.784281065	0.064782785	0.443725568	0.37809474	-0.421443499	0.405262015
Rps16	blue	0.560699544	0.247088161	0.784859207	0.064449385	-0.572013719	0.235560778	0.415919327	0.41209572
Cyb5r3	blue	0.560499776	0.24729364	0.738828955	0.093408193	-0.244914524	0.639973583	0.295513969	0.569632443
E030024N20Rik	blue	0.56012845	0.247675761	0.859477838	0.028232308	-0.613527398	0.195179627	0.228119082	0.663756843
Fam131a	blue	-0.559724014	0.248092219	-0.737447721	0.094351199	0.270606679	0.603997971	-0.356817055	0.487489108
Calm3	blue	0.557755757	0.250122898	0.848799793	0.032563922	-0.389973245	0.444693528	0.166064153	0.753193571
Gm14303	blue	0.556651237	0.251265288	0.864610335	0.026254671	-0.558146779	0.249718958	0.190681046	0.717444942
Ramp1	blue	0.556350999	0.251576172	0.832552979	0.039710275	-0.427527154	0.397780861	0.324238789	0.530685556
Gm10231	blue	0.554416645	0.253582721	0.857117784	0.029164498	-0.507888365	0.303672504	0.22273997	0.671415454
Tlk1	blue	-0.554137567	0.253872729	-0.848691181	0.032609479	0.592305869	0.215439418	-0.230451158	0.660442633
Gm7079	blue	0.553501708	0.254533975	0.822970221	0.044235298	-0.519041692	0.291353488	0.183894458	0.727267708
Gpr155	blue	-0.55331597	0.254727256	-0.855048613	0.029993577	0.608428366	0.199973001	-0.320590573	0.535589019
Gm14567	blue	0.551516828	0.256602419	0.688563843	0.130385237	-0.743536714	0.090225892	0.294734009	0.570700483
Ppfia3	blue	-0.550572002	0.257589313	-0.787883617	0.062718125	0.657234019	0.156097243	-0.202453895	0.700468205
Car11	blue	0.5483522	0.259913749	0.861541124	0.027429103	-0.588203213	0.219449342	0.266148647	0.610203363
Gm43110	blue	0.548269342	0.26000067	0.837584425	0.037426063	-0.553169996	0.254879198	0.148251579	0.779251807
Zfp148	blue	-0.547257187	0.261063365	-0.843876252	0.034659208	0.461133673	0.357328206	-0.143113744	0.786794979
Sdf2	blue	0.537661296	0.271221531	0.825317358	0.043105903	-0.449844904	0.370748049	0.190770984	0.717314943
Kctd12	blue	-0.5375	0.2713332	-0.5854	0.22216	0.313057	0.5457543	-0.578193554	0.229356973

		56554	34	39817	7031	475	84		
Arrb2	blue	0.534933575	0.27413631	0.703388007	0.118920247	-0.763651807	0.077189442	0.334650607	0.516763023
Cttnbp2	blue	-0.534575731	0.27451958	-0.835167535	0.038515385	0.619175403	0.189926065	-0.261180926	0.617136901
Ranbp1	blue	0.531965747	0.277321223	0.810302346	0.050564646	-0.537869453	0.270999591	0.268005198	0.607617179
Sgta	blue	0.530115042	0.279314421	0.664600259	0.14987442	-0.246136258	0.638251456	0.367780793	0.473202324
Lonp2	blue	-0.529489555	0.279989298	-0.506922352	0.304748459	0.319755458	0.53671328	-0.647222165	0.164726313
Mgll	blue	0.529177942	0.28032575	0.620080598	0.189089582	-0.122289098	0.817480747	0.266814018	0.609276181
Arl8a	blue	0.528366096	0.281203031	0.597680029	0.21023201	-0.124627643	0.814026396	0.245866851	0.638631111
Gm13408	blue	0.528076284	0.281516455	0.821292235	0.045051051	-0.539924142	0.268812611	0.128647712	0.808093007
Dip2c	blue	-0.527527391	0.282110433	-0.849340994	0.032337365	0.601361677	0.20669446	-0.313879187	0.544642931
Tbc1d9	blue	-0.526845354	0.282849155	-0.724456635	0.103426022	0.297884342	0.566389883	-0.369134185	0.471447843
Virma	blue	-0.524219622	0.285699971	-0.82216793	0.044624465	0.463201386	0.354889129	-0.17569074	0.739175434
7-Sep	blue	-0.524192999	0.285728932	-0.789188933	0.061977599	0.476685971	0.339129604	-0.440591794	0.381876397
Alkbh7	blue	0.523287596	0.286714503	0.772155372	0.071955693	-0.447428651	0.373642931	0.388105729	0.447070824
Mfsd6	blue	-0.522989881	0.28703886	-0.832850243	0.03957356	0.614984483	0.19381866	-0.360346667	0.482875457
Pafah1b1	blue	-0.522876895	0.287161994	-0.777700373	0.068632982	0.560334465	0.247463728	-0.196541811	0.708983359
Dock9	blue	-0.522526254	0.287544254	-0.829209749	0.041263048	0.62356298	0.185885773	-0.335994799	0.514973448
Ywhaq	blue	-0.518560486	0.291881019	-0.64344941	0.168028645	0.147400401	0.780500674	-0.258609589	0.620733382
Sptan1	blue	-0.513115944	0.297874712	-0.76600955	0.075721628	0.52194854	0.288174483	-0.174672444	0.740656002
mt-Nd3	blue	0.512973156	0.298032518	0.752280118	0.084447027	-0.545340296	0.263080577	0.185792501	0.724517921
Gm9396	blue	0.509547982	0.301827328	0.841370174	0.035749298	-0.575055542	0.232498923	0.307838473	0.552828374
Ajm1	blue	0.507891784	0.303668699	0.60064303	0.207383063	-0.386268582	0.449413424	0.590237177	0.217457626
Fbll1	blue	0.50511	0.3067704	0.7958	0.05828	-0.475602	0.3403864	0.072320711	0.891708062

		0427	24	107	3246	449	14		
Gm12918	blue	0.504510605	0.307440875	0.827875567	0.041890481	-0.448231799	0.372679818	0.214335678	0.683419751
Mindy3	blue	-0.502900241	0.30924355	-0.825186129	0.043168688	0.583119871	0.224458964	-0.186317094	0.723758271
Psmb5	blue	0.502624554	0.309552552	0.829402472	0.041172781	-0.518641298	0.291792402	0.195810918	0.710037506
Rundc3a	blue	0.500011472	0.312487094	0.812561623	0.049407068	-0.319291725	0.537337862	0.130794523	0.80492698
Ak1	blue	0.499738932	0.312793753	0.591486232	0.216238144	-0.249277628	0.633828532	0.395590251	0.437567908
Lamtor5	blue	0.495184386	0.317934903	0.811166623	0.050120352	-0.493427442	0.319926385	0.291115156	0.575662984
Spp1	blue	-0.494127232	0.319132629	-0.747493012	0.087589773	0.331650921	0.520763147	-0.338260169	0.511961601
Aebp1	blue	-0.49374769	0.319563045	-0.614451636	0.194315904	0.074475259	0.888493653	-0.185404596	0.725079734
Rpl3-ps2	blue	0.492806771	0.320631003	0.818827686	0.046261765	-0.451540079	0.368721784	0.21552568	0.681717206
Rab5c	blue	0.492493321	0.320987065	0.633283904	0.177062925	-0.076625934	0.885286055	0.125831292	0.812249238
Gm15516	blue	0.491661374	0.321932814	0.807347954	0.052097094	-0.588447274	0.219209965	0.223420545	0.670445395
Ap2s1	blue	0.490410889	0.323356273	0.725885187	0.102410049	-0.240937248	0.645587423	0.23435971	0.654896476
Gm28530	blue	0.488594155	0.325428404	0.804471383	0.05360949	-0.54332224	0.265210747	0.335813088	0.515215262
Pdcd4	blue	-0.485453689	0.329021757	-0.791270608	0.060804982	0.518781022	0.291639207	-0.084100553	0.874146588
Rcan1	blue	0.485134679	0.329387577	0.551941229	0.256159602	-0.028454503	0.957329765	0.16877891	0.74923558
Rps19-ps3	blue	0.484876864	0.329683331	0.816882962	0.047227648	-0.53124908	0.278092421	0.273195925	0.60040124
Rmrp	blue	0.484299778	0.330345687	0.808076187	0.051717392	-0.523016261	0.287010114	0.114272058	0.829338001
Ndufc2	blue	0.484128925	0.330541879	0.811782265	0.049804981	-0.548208254	0.26006476	0.150333572	0.776198425
Gprasp1	blue	-0.483743157	0.330985014	-0.738815345	0.093417464	0.194599191	0.711785837	-0.07071866	0.894098846
Rpp25l	blue	0.482592462	0.332308109	0.768643316	0.074097084	-0.449592275	0.371050354	0.394500416	0.43894754
Ubxn2a	blue	-0.481153711	0.333965115	-0.791818646	0.060497979	0.361073869	0.48192658	-0.261970014	0.616034256
Ftl1-ps1	blue	-0.4796	0.3357456	-0.4886	0.32540	0.366886	0.4743623	-0.669324627	0.145940255

		10555	84	13154	6709	841	15		
Pisd-ps1	blue	0.479286956	0.336119504	0.750648764	0.085512222	-0.246655996	0.63751918	0.220669404	0.674368641
Gm8797	blue	0.476756347	0.339048031	0.803026877	0.054376495	-0.487647821	0.326509692	0.29467458	0.570781886
Pigq	blue	-0.473745409	0.342544343	-0.780766304	0.06682656	0.496148727	0.316843777	-0.1017838	0.847851537
Gm10221	blue	0.473434535	0.342906067	0.701034276	0.120709902	-0.667755873	0.147241663	0.188850878	0.720091334
Lage3	blue	0.472280373	0.344250214	0.784540385	0.064633144	-0.47474213	0.341385517	0.304328937	0.557599475
Sgsm2	blue	0.469922675	0.34700187	0.679893453	0.137301931	-0.192248495	0.71517996	0.25704514	0.62292406
Stmn3	blue	0.468790186	0.34832638	0.736509254	0.094994325	-0.184265625	0.726729824	0.125878496	0.812179554
Peg13	blue	-0.466892856	0.350549455	-0.74965544	0.086163751	0.469719085	0.347239847	-0.139782775	0.791691462
Trappc6b	blue	0.465448284	0.352245422	0.780085242	0.067225937	-0.48850739	0.325527487	0.055229025	0.917240694
Klc1	blue	0.46461697	0.353222729	0.591966972	0.215769525	-0.069461018	0.895976042	0.224218781	0.669308023
Rnf126	blue	0.464255048	0.353648512	0.712361305	0.112204988	-0.216402168	0.680463794	0.244013634	0.641244158
Gm8210	blue	0.461198055	0.357252171	0.769850669	0.073357715	-0.589076343	0.218593452	0.24229021	0.643676453
Dlgap1	blue	-0.460163553	0.3584746	-0.762976117	0.077612443	0.549553121	0.258655212	-0.122565134	0.817072899
Slc3a2	blue	-0.458671502	0.360240299	-0.59204168	0.215696737	0.317076865	0.540323799	-0.448471688	0.372392318
Gm12319	blue	0.457716367	0.361372217	0.665614351	0.149026185	-0.632764514	0.177529815	0.135027213	0.798690111
Casd1	blue	-0.457349177	0.361807703	-0.738056995	0.09393471	0.312626588	0.546337458	-0.311724473	0.547558759
Fscn1	blue	0.456436901	0.362890459	0.632035873	0.178185669	-0.098184282	0.853196833	0.178463838	0.735146221
Slc35f3	blue	0.454116866	0.365649174	0.718678252	0.107580716	-0.193034784	0.714044296	0.100368632	0.849952602
Pygb	blue	0.452630162	0.367420847	0.625802099	0.183837747	-0.323648165	0.531478523	0.451112539	0.369232461
Actg1	blue	-0.451793966	0.368418643	-0.585205236	0.222398351	0.077734481	0.883633139	-0.178830345	0.734614006
Mut	blue	-0.451132614	0.369208477	-0.689100903	0.129961891	0.279632429	0.591484187	-0.305920792	0.555433998
Fem1b	blue	-0.4507	0.3696122	-0.7761	0.06954	0.504676	0.3072552	-0.122760815	0.816783793

		94731	3	67169	4565	647	27		
Fau	blue	0.448565097	0.372280392	0.809777652	0.050835258	-0.482908632	0.331944378	0.199174678	0.705188667
Mir703	blue	0.446651664	0.374575495	0.677451584	0.139277659	-0.287611747	0.580478075	0.374435014	0.464595669
Fbxw2	blue	0.442841892	0.37915979	0.654927546	0.158067746	-0.353514348	0.491818245	0.428268704	0.3968722
Slc30a3	blue	0.44224541	0.379879285	0.744766296	0.089402862	-0.349265194	0.497404972	0.274420205	0.598702498
Gm10263	blue	0.440953997	0.381438646	0.803526299	0.054110742	-0.505009249	0.306883477	0.153530538	0.771513675
Ppp1cb	blue	0.438987471	0.383817431	0.545209426	0.263218516	-0.019852874	0.970224601	0.197274468	0.707926984
Neurl1a	blue	0.436805066	0.386463313	0.75170443	0.084822238	-0.302549894	0.560022329	0.241135516	0.645307299
Cog5	blue	-0.434488617	0.389278533	-0.763865682	0.077055768	0.375457302	0.463277814	0.008167173	0.987749513
Gm6063	blue	0.433369784	0.390640777	0.797294947	0.0574695	-0.472160874	0.344389491	0.104585233	0.84369413
Mmd2	blue	0.433134291	0.390927711	0.760492962	0.079175937	-0.487392967	0.326801113	0.306849811	0.554171283
Idh3b	blue	0.430437196	0.394219085	0.771979299	0.07206237	-0.438885401	0.383941038	0.089198197	0.866557549
Arap2	blue	-0.426461369	0.399088062	-0.735519517	0.095674694	0.401772267	0.429768831	-0.34062318	0.508825488
Sec62	blue	-0.423586788	0.40262101	-0.685301608	0.132969527	0.311140042	0.54835038	-0.281505288	0.588896043
Plcg1	blue	-0.423161106	0.40314508	-0.779891516	0.067339737	0.409989199	0.419473978	-0.161903451	0.759266789
R3hdm4	blue	0.422453317	0.404016972	0.69643984	0.124236808	-0.26258654	0.615173083	0.261056846	0.617310331
Tagln3	blue	0.420556464	0.40635674	0.752600599	0.084238474	-0.335336391	0.515849785	0.208483457	0.691805718
Pebp1	blue	0.416400905	0.41149846	0.502185026	0.310045432	0.048304304	0.927599899	0.069512836	0.89589869
Ifitm3	blue	-0.414149904	0.41429267	-0.609930719	0.198555757	0.137716342	0.794731437	-0.287224397	0.581011103
Atp1b3	blue	-0.407829507	0.422171842	-0.541747121	0.266877984	0.068436805	0.897505058	-0.220320691	0.674866279
Atcay	blue	-0.407069643	0.423122415	-0.693409843	0.126586929	0.527390904	0.282258204	-0.034027646	0.948978232
Timm10	blue	0.403715173	0.427327189	0.734925917	0.096083786	-0.352845446	0.492696444	0.233276449	0.656432534
Rab3a	blue	0.39834	0.4340852	0.6506	0.16178	-0.085086	0.8726788	0.095818318	0.856712384

		6362	22	09073	5253	07	91		
Ephx1	blue	-0.39737887	0.435306737	-0.729876576	0.099594994	0.328286501	0.5252603	-0.235940434	0.652656502
Fam173a	blue	0.392865191	0.441020221	0.451100231	0.369247166	-0.096216734	0.85612027	0.393622822	0.440059516
Lhfpl4	blue	0.392603767	0.441351873	0.695207477	0.125190342	-0.234710222	0.654399629	0.229055094	0.662426188
Ctxn1	blue	0.392546898	0.441424031	0.614856437	0.193938103	-0.018139153	0.972794255	-0.01666066	0.975011323
Atp6v1f	blue	0.389921006	0.444759973	0.708799691	0.114849883	-0.187097846	0.722627967	0.128103616	0.8088957
Tmsb15b2	blue	0.389155293	0.445734258	0.730208686	0.099362332	-0.533446336	0.275730573	0.280629141	0.590106442
5031439G07Rik	blue	0.386850688	0.448670739	0.540164072	0.268557679	-0.366714477	0.474586076	0.469974752	0.346941007
Gm13192	blue	0.385583829	0.450287573	0.759160861	0.080020481	-0.465785413	0.351849362	0.12324589	0.816067189
Ppp1r1b	blue	0.384295739	0.451933406	0.733426442	0.097120639	-0.485196997	0.329316103	0.090171197	0.865109788
Sec14l1	blue	-0.379311368	0.45832006	-0.725352486	0.102788382	0.482541701	0.33236652	-0.02727914	0.959091439
Mrpl34	blue	0.377135111	0.461117465	0.512991603	0.298012129	0.019877816	0.970187203	0.166530018	0.752514098
Cnih3	blue	0.375622655	0.463064765	0.586105059	0.221511565	-0.011765057	0.982353229	0.088929829	0.866956908
Ano3	blue	-0.374849558	0.464061129	-0.52945262	0.280029169	0.141998605	0.788433695	-0.279949034	0.591046457
Ptgds	blue	-0.373069193	0.466358212	-0.515348505	0.295411422	-0.042948162	0.935617368	-0.080559026	0.879422865
Mrpl12	blue	0.363940247	0.478192028	0.577731266	0.22981877	-0.176478114	0.738030993	0.320889033	0.535187386
Ndufb6	blue	0.363135149	0.479240073	0.568856028	0.238756061	-0.052271584	0.921664035	0.176410858	0.738128735
Zbtb7a	blue	0.362774923	0.479709229	0.548425305	0.259837069	0.025670783	0.961502284	0.050159489	0.924823866
Hrk	blue	0.346434351	0.501137438	0.64925325	0.162959916	-0.145203114	0.783726056	0.095189688	0.857646728
Prelid1	blue	0.339203631	0.510708786	0.522675388	0.287381648	0.079035761	0.881693213	-0.040554486	0.93920162
Rab28	blue	0.337018487	0.513611796	0.541915245	0.266699835	0.062867923	0.905822354	-0.029751035	0.955386614
Atp1a2	blue	-0.331077769	0.521528476	-0.565870856	0.241792423	-0.029119735	0.956332743	-0.008729296	0.986906388
Lsm12	blue	0.31381	0.5447356	0.4415	0.38069	-0.227292	0.6649328	0.440968391	0.381421253

		064	24	6801	6959	203	52		
Necab2	blue	0.312922987	0.545936354	0.510010966	0.30131333	-0.014048387	0.978928806	0.19025337	0.718063184
Anp32e	blue	-0.311176868	0.548300488	-0.479576411	0.33578512	-0.116643313	0.825828535	0.10349479	0.84531209
Ddx5	blue	-0.306758253	0.554295692	-0.620254566	0.188928994	0.051791461	0.92238227	-0.012904183	0.9806448
Tnk2	blue	0.305981192	0.555351878	0.482951348	0.331895248	-0.117733624	0.82421553	0.28162006	0.588737534
Usp5	blue	0.302060024	0.560689981	0.347782548	0.499358798	-0.043252941	0.935161047	0.33476635	0.516608858
Mrpl55	blue	0.30183251	0.561000138	0.565209912	0.242466745	-0.166568693	0.752457695	0.270897655	0.603593503
Dym	blue	0.293575471	0.572287924	0.37137502	0.468547381	-0.196139077	0.709564173	0.50230802	0.309907479
Slc6a8	blue	-0.292013529	0.574429981	-0.548491315	0.259767838	-0.028973108	0.956552498	-0.035216182	0.947197564
Rasa3	blue	0.286217897	0.582396738	0.37033925	0.469887354	0.118210239	0.823510556	0.122701896	0.816870842
Kcnip4	blue	-0.283963706	0.585503202	-0.419109677	0.408144404	-0.092309899	0.861928443	-0.1439796	0.785522958
Aasdhppt	blue	0.259125394	0.620011522	0.39675133	0.436099639	0.105727123	0.842000236	0.070232157	0.894824977
Mgp	blue	-0.24470677	0.640266538	-0.296109862	0.568816819	-0.17325213	0.742721999	-0.09502001	0.857898943
S100a9	blue	-0.242580355	0.643266816	-0.308647831	0.551729687	-0.199586437	0.704595583	-0.068154885	0.897925965
Rbfox2	blue	0.231830219	0.658484559	0.363851873	0.478307035	0.15714864	0.766217487	0.016468951	0.975298807
Ifitm2	blue	-0.231105889	0.659512841	-0.362726326	0.479772533	-0.191553058	0.7161847	0.047681864	0.928531408
Stip1	blue	0.219498178	0.676040384	0.328449346	0.525042371	0.241567884	0.644696526	-0.049126934	0.926368882
Nras	blue	0.206229252	0.695041638	0.285081683	0.583961994	0.238282055	0.649341546	0.024351619	0.963479791
Pithd1	blue	0.126274401	0.811595135	0.202099745	0.700977694	0.30914754	0.551051645	-0.072939917	0.890784153
Timp3	blue	-0.066811616	0.899931693	-0.225229449	0.66786858	-0.300177329	0.56325796	0.062997532	0.905628711
Glrx3	darkmagenta	-0.955577261	0.002916238	-0.814360485	0.048494287	0.933879445	0.006413355	-0.934433212	0.00630757
Cct7	darkmagenta	0.923570173	0.008539044	0.792067303	0.06035892	-0.855832773	0.029678084	0.955776354	0.002890352
Gm25777	darkmag	0.92280	0.0087096	0.8655	0.02589	-0.822159	0.0446285	0.902116326	0.013902898

	enta	0429	15	59769	6315	531	47		
Fam32a	darkmag	0.91817	0.0097688	0.8048	0.05340	-0.912136	0.0112407	0.941523302	0.005029305
	enta	6067	22	4954	9527	95	26		
Dazap2	darkmag	0.91714	0.0100133	0.8099	0.05074	-0.932136	0.0067519	0.923657184	0.008519867
	enta	355	74	47466	7603	286	53		
Arl5a	darkmag	-0.9160	0.0102765	-0.7554	0.08236	0.858533	0.0286036	-0.953898294	0.003139059
	enta	45916	66	90934	8076	439	12		
Clu	darkmag	0.91487	0.0105621	0.7675	0.07476	-0.895903	0.0156901	0.936867795	0.005852701
	enta	0381	09	65358	0053	361	64		
Rnaseh2c	darkmag	0.90981	0.0118327	0.9037	0.01345	-0.880523	0.0205593	0.876319223	0.021999434
	enta	6871	66	35558	4231	051	61		
Syf2	darkmag	0.90910	0.0120165	0.6929	0.12691	-0.747173	0.0878011	0.908212927	0.012250653
	enta	8104	61	85179	7834	785	78		
Rpl26	darkmag	0.90475	0.0131765	0.7336	0.09697	-0.850966	0.0316613	0.980435455	0.000570413
	enta	0801	45	392	322	665	17		
Pik3ip1	darkmag	0.89423	0.0161892	0.8554	0.02982	-0.880044	0.0207210	0.901279979	0.014137419
	enta	0041	87	70502	3642	093	81		
Sec13	darkmag	0.89072	0.0172593	0.6946	0.12564	-0.932840	0.0066141	0.968623445	0.001461287
	enta	4376	04	15577	9447	267	85		
AA414768	darkmag	0.88243	0.0199211	0.6362	0.17437	-0.909356	0.0119520	0.959390659	0.002440193
	enta	1098	25	80158	955	228	62		
Rpl7a-ps11	darkmag	0.88209	0.0200344	0.7643	0.07674	-0.732921	0.0974706	0.910521001	0.01165153
	enta	0338	02	69716	1157	872	52		
Twf1	darkmag	0.87872	0.0211702	0.6800	0.13716	-0.940678	0.0051742	0.967899467	0.001529127
	enta	3219	13	62655	5479	028	65		
Gm8226	darkmag	0.87697	0.0217720	0.7591	0.08001	-0.732865	0.0975099	0.912155714	0.011235997
	enta	3981	78	69154	5211	292	35		
Aagab	darkmag	0.87646	0.0219499	0.8022	0.05479	-0.727456	0.1012975	0.887672169	0.018217662
	enta	137	79	36506	8294	657	68		
Ranbp3	darkmag	0.87594	0.0221284	0.6284	0.18145	-0.915192	0.0104835	0.94401833	0.004613199
	enta	9173	27	22057	3337	161	7		
Fnta	darkmag	0.87591	0.0221399	0.7129	0.11178	-0.776671	0.0692443	0.911249757	0.011465383
	enta	6227	28	3169	3928	029	85		
Clp1	darkmag	0.87423	0.0227314	0.6558	0.15725	-0.927192	0.0077584	0.970726922	0.001272827
	enta	2642	84	7838	4147	397	46		
G3bp2	darkmag	-0.8691	0.0245604	-0.8449	0.03421	0.758079	0.0807090	-0.84590667	0.033787677
	enta	5518	95	05924	5929	393	98		
Senp2	darkmag	-0.8666	0.0254997	-0.7557	0.08221	0.821652	0.0448755	-0.935357194	0.006132977
	enta	17667	78	27968	5526	045	44		
Gm49376	darkmag	-0.8652	0.0260101	-0.5440	0.26443	0.772367	0.0718274	-0.881670165	0.020174501
	enta	57485	56	52034	9642	261	11		
Sstr4	darkmag	-0.8647	0.0261851	-0.7196	0.10685	0.870675	0.0240057	-0.84026943	0.036233111
	enta	94019	59	77719	6928	567	5		
Sar1b	darkmag	0.86448	0.0263039	0.8885	0.01794	-0.800866	0.0555331	0.84181878	0.035552998

	enta	0181	79	48249	004	144	83		
Rpl27	darkmagenta	0.859264401	0.028316023	0.853101529	0.030783768	-0.909871192	0.011818736	0.87995654	0.020750709
Tex261	darkmagenta	-0.856887848	0.029256086	-0.577249894	0.230300025	0.846875175	0.033375643	-0.955517332	0.002924053
Rab8b	darkmagenta	-0.856158476	0.029547508	-0.654869526	0.158117451	0.946541933	0.004210262	-0.935376245	0.006129403
Astn1	darkmagenta	-0.855743317	0.029713996	-0.736437589	0.095043516	0.900771162	0.014281022	-0.851072925	0.031617363
Napa	darkmagenta	0.855119153	0.029965132	0.652671369	0.160005394	-0.872998955	0.023169682	0.948687013	0.00388198
Rbx1	darkmagenta	0.854335438	0.030281879	0.679664591	0.137486588	-0.69989029	0.121583941	0.922697141	0.008732627
Atp1a1	darkmagenta	0.854264915	0.030310459	0.581320646	0.226242948	-0.838057985	0.037214342	0.975833524	0.000868971
Rps6-ps4	darkmagenta	0.853309057	0.030699084	0.640738979	0.170418085	-0.847395594	0.033155223	0.962444499	0.002089139
Ap4s1	darkmagenta	0.851425163	0.031471867	0.698063715	0.122985191	-0.759341086	0.079905983	0.868462524	0.024815223
Nlk	darkmagenta	-0.851366578	0.031496044	-0.585166865	0.222436197	0.788587315	0.062318406	-0.957184589	0.002710495
Pom121	darkmagenta	-0.850440892	0.031879227	-0.661299968	0.152649121	0.844851266	0.034239392	-0.926931026	0.007813552
6-Sep	darkmagenta	-0.848615533	0.032641227	-0.777269173	0.068888803	0.915533911	0.010400468	-0.907276304	0.012497921
Cwc15	darkmagenta	0.842758082	0.035143626	0.591529974	0.216195488	-0.91553276	0.010400747	0.92814674	0.007558851
Gaa	darkmagenta	-0.842478836	0.035265096	-0.588046688	0.219602919	0.858960045	0.028435601	-0.93149256	0.006879142
Creg2	darkmagenta	-0.84071488	0.036036953	-0.737254589	0.094483392	0.865302509	0.025993185	-0.831636149	0.040133326
Anapc13	darkmagenta	0.836159788	0.038066391	0.585008526	0.2225924	-0.879896013	0.020771204	0.984207363	0.000372142
Fktn	darkmagenta	-0.835437508	0.038392977	-0.8047845	0.053443894	0.795469104	0.058471272	-0.86610788	0.025690501
AC102496.1	darkmagenta	0.832668712	0.039657022	0.591338072	0.216382652	-0.736528225	0.094981305	0.95570235	0.002899961
Sc5d	darkmagenta	-0.831672256	0.040116626	-0.4877237	0.326422943	0.760994818	0.078858812	-0.901196425	0.014160952
Hexa	darkmagenta	-0.828840115	0.041436434	-0.816161505	0.047588331	0.869727063	0.024351124	-0.873892084	0.02285205
Sppl3	darkmagenta	-0.827882205	0.041887348	-0.512078095	0.299022435	0.751790683	0.084765975	-0.959617059	0.002413245
Mrpl18	darkmag	0.82490	0.0433013	0.8509	0.03167	-0.837135	0.0376274	0.862751565	0.026963016

	enta	9081	79	2489	8606	076	8		
Ccdc51	darkmagenta	0.8234595	0.043998743	0.741777422	0.091409351	-0.75072923	0.085459542	0.897170511	0.015317201
Ndufv3	darkmagenta	0.823101187	0.044171921	0.791188307	0.060851147	-0.857529703	0.029000763	0.9171834	0.010003881
Fbxw5	darkmagenta	-0.821162631	0.045114346	-0.765733395	0.075892889	0.69753153	0.12339477	-0.878611833	0.021208298
Cul2	darkmagenta	-0.820729916	0.045325975	-0.60425915	0.203927581	0.889319828	0.017697229	-0.911379803	0.011432318
AI413582	darkmagenta	0.820142059	0.045614217	0.715866863	0.109628192	-0.824207091	0.043638443	0.949924155	0.0036986
Dnajc4	darkmagenta	0.819768992	0.045797583	0.601836779	0.206239732	-0.7048357	0.117825299	0.955337174	0.002947606
Matk	darkmagenta	0.819338457	0.046009621	0.70192344	0.120032456	-0.870420951	0.024098232	0.875488171	0.022289628
Atp5d	darkmagenta	-0.818658271	0.046345539	-0.498535133	0.314149583	0.797425768	0.057398029	-0.952932937	0.003270829
Gm24494	darkmagenta	0.815570786	0.047884602	0.592024029	0.215713933	-0.812669259	0.049352229	0.988934323	0.000182996
Shisa7	darkmagenta	-0.813164684	0.049100182	-0.730150036	0.099403402	0.856554832	0.029388973	-0.921931798	0.008904067
Prelid3a	darkmagenta	0.812959916	0.049204285	0.779775464	0.067407951	-0.801952597	0.054950176	0.902236569	0.013869336
Psmd4	darkmagenta	0.810759526	0.0503294	0.663169916	0.151074313	-0.776930233	0.069090192	0.96455327	0.001862437
Scoc	darkmagenta	-0.806490923	0.052545597	-0.673411288	0.142573273	0.798316908	0.056912267	-0.880191593	0.020671214
Rpl17-ps3	darkmagenta	0.806265848	0.052663678	0.649390259	0.16284105	-0.753702824	0.083522972	0.870014498	0.024246213
Anxa6	darkmagenta	0.805980086	0.052813774	0.634442853	0.17602297	-0.797237512	0.057500891	0.960685564	0.002288055
Sbds	darkmagenta	0.804168941	0.053769664	0.475806253	0.340149888	-0.775555383	0.069909837	0.921648063	0.008968037
Pdxk	darkmagenta	0.802907659	0.054440022	0.685508597	0.132804903	-0.793450066	0.059588306	0.879095786	0.021043065
Trmt112	darkmagenta	0.8028595	0.054465694	0.647537211	0.164451797	-0.597994758	0.209928647	0.871660034	0.023649767
Ptpn23	darkmagenta	-0.800430283	0.055767869	-0.66233401	0.151777426	0.845685565	0.033882077	-0.845886188	0.033796417
Mphosph6	darkmagenta	0.796874448	0.057699505	0.711902208	0.1125444	-0.88654216	0.018578769	0.774633274	0.070462036
BC005624	darkmagenta	0.795011827	0.05872341	0.46098634	0.357502226	-0.792659402	0.060028387	0.903499871	0.013519094
Coq9	darkmag	0.79333	0.0596524	0.7766	0.06925	-0.879947	0.0207536	0.903814621	0.013432505

	enta	4626	67	56536	3011	957	15		
Ehmt2	darkmagenta	0.791985738	0.060404518	0.694274419	0.125914397	-0.563664416	0.244046421	0.821137918	0.04512642
Map2k4	darkmagenta	-0.791099556	0.060900949	-0.586270282	0.22134889	0.878859973	0.0211235	-0.919487561	0.009462428
Plxna1	darkmagenta	-0.79100817	0.060952248	-0.597784987	0.210130822	0.883413171	0.019596381	-0.918587731	0.009672138
Eml2	darkmagenta	0.787036868	0.063200653	0.773009025	0.07143951	-0.600078221	0.207924913	0.673043669	0.142874775
Ahcyl2	darkmagenta	0.786893259	0.063282659	0.515677661	0.2950489	-0.843562407	0.034794854	0.97995192	0.000598859
Rps6	darkmagenta	0.786338891	0.063599675	0.553197805	0.25485025	-0.805520169	0.05305576	0.954124913	0.003108513
Ambra1	darkmagenta	-0.784118806	0.064876497	-0.654311691	0.158595664	0.865474195	0.025928519	-0.822628726	0.044400753
Gm23205	darkmagenta	0.783807139	0.06505672	0.714017933	0.110983986	-0.711191651	0.113070604	0.860469935	0.027844728
Nov	darkmagenta	-0.77860543	0.06809744	-0.665012331	0.149529496	0.84785774	0.03296062	-0.90526538	0.013036867
Otulin	darkmagenta	0.777418671	0.06880006	0.682711571	0.135036899	-0.790793735	0.0610727	0.946106038	0.00427857
Ak6	darkmagenta	0.777260981	0.068893667	0.610923216	0.197621749	-0.832528165	0.039721698	0.982695909	0.000446557
Ahsa1	darkmagenta	0.776550088	0.069316383	0.711090638	0.113145496	-0.535710803	0.273304563	0.765918166	0.075778282
Pex2	darkmagenta	0.774975267	0.070257005	0.417791024	0.409776038	-0.76702198	0.075095258	0.874266143	0.022719641
Emc10	darkmagenta	-0.773699951	0.071022962	-0.429856528	0.39492893	0.787498228	0.062937533	-0.907100187	0.012544683
Mbd3	darkmagenta	0.7730821	0.071395402	0.686092287	0.132341151	-0.666810575	0.148028245	0.888098107	0.018082431
Ddx1	darkmagenta	0.771493642	0.072356986	0.417832756	0.409724368	-0.676311755	0.140204051	0.92490501	0.008247146
Ykt6	darkmagenta	0.769578139	0.073524315	0.783526311	0.065219216	-0.626546886	0.183158607	0.747591584	0.087524542
Ralgapb	darkmagenta	-0.768683496	0.074072424	-0.400510909	0.431356411	0.739979821	0.092625694	-0.851850589	0.031296562
F730043M19Rik	darkmagenta	0.768495565	0.074187796	0.423273387	0.403006826	-0.718563843	0.107663706	0.893844736	0.016305281
Tomm6	darkmagenta	-0.765866687	0.075810205	-0.725808732	0.102464311	0.722187518	0.10504891	-0.876654695	0.021882805
Slc12a5	darkmagenta	-0.765042413	0.076322177	-0.618228455	0.19080276	0.84011094	0.036303024	-0.878793034	0.021146359
Pllp	darkmag	-0.7632	0.0774490	-0.5072	0.30436	0.799012	0.0565343	-0.972477078	0.001125842

	enta	36843	97	64089	7667	669	28		
Rmnd5b	darkmagenta	-0.762618333	0.077836848	-0.38834037	0.446771909	0.706656364	0.116454551	-0.814630575	0.04835792
Marcksl1	darkmagenta	0.760705403	0.079041624	0.626784791	0.182941891	-0.640533706	0.170599624	0.897715736	0.015158053
Rpl11	darkmagenta	0.760510145	0.079165069	0.591185252	0.216531745	-0.771954085	0.072077652	0.92874501	0.00743502
Adap1	darkmagenta	-0.758253324	0.080598169	-0.512318125	0.298756846	0.887616962	0.018235225	-0.951637656	0.003451817
Lrpap1	darkmagenta	0.75176284	0.084784135	0.794141672	0.059204581	-0.561634109	0.246127767	0.669369298	0.14590327
Tmem151a	darkmagenta	-0.750283069	0.085751826	-0.550486188	0.257679021	0.874151246	0.022760273	-0.923432735	0.00856938
Meaf6	darkmagenta	0.746391394	0.088320274	0.586963361	0.220667024	-0.767564951	0.074760304	0.890114163	0.017448916
Cutc	darkmagenta	0.745445566	0.088949636	0.464003394	0.353944677	-0.863931885	0.026512179	0.924119992	0.008418213
Gpx4	darkmagenta	0.741634014	0.091506124	0.667415599	0.147524599	-0.5764196	0.23113106	0.787111257	0.063158194
Trf	darkmagenta	-0.738935529	0.093335607	-0.603879886	0.204288886	0.685497057	0.132814079	-0.909199906	0.011992678
5-Sep	darkmagenta	0.737110215	0.094582266	0.514089791	0.296799275	-0.57395128	0.233608616	0.868334861	0.024862309
Sesn1	darkmagenta	0.73531082	0.095818433	0.645648639	0.166100286	-0.839601744	0.03652807	0.822657495	0.044386803
Bcap31	darkmagenta	-0.735241053	0.095866506	-0.387214186	0.448207166	0.713766726	0.111168758	-0.935427251	0.006119837
Naxe	darkmagenta	0.731818003	0.098238338	0.60116709	0.206880082	-0.481337147	0.333753686	0.783858105	0.065027193
Kctd3	darkmagenta	0.729675982	0.099735638	0.401410962	0.430223384	-0.781487719	0.066404705	0.78937976	0.061869678
Cpt1c	darkmagenta	-0.72645127	0.102008685	-0.359792173	0.483599362	0.709398806	0.114403083	-0.788581178	0.062321887
Haus2	darkmagenta	0.72640482	0.102041593	0.717642918	0.108332749	-0.665252436	0.149328672	0.844387338	0.034438847
Kti12	darkmagenta	0.722017674	0.105170833	0.52052629	0.289728244	-0.664731585	0.149764457	0.937204691	0.005791067
Gm47163	darkmagenta	0.72180822	0.105321276	0.65088223	0.161549027	-0.812721683	0.04932553	0.92794645	0.00760053
Rpl9	darkmagenta	0.721235352	0.105733231	0.559414974	0.248410063	-0.799980369	0.056010601	0.93976941	0.005332336
Ppa1	darkmagenta	0.720223568	0.106462549	0.689655043	0.129525707	-0.464648144	0.353186062	0.652451604	0.160194665
Nfx1	darkmag	0.72013	0.1065235	0.4179	0.40958	-0.834770	0.0386958	0.92099429	0.00911628

	enta	9142	05	48217	1421	209	66		
Dot1l	darkmagenta	-0.717547627	0.108402082	-0.360580085	0.482570823	0.762136819	0.078139322	-0.850451592	0.031874785
Uqcrc1	darkmagenta	0.716525386	0.109147079	0.655391501	0.157670532	-0.497976403	0.314779614	0.640841007	0.170327883
Nadk	darkmagenta	-0.716441511	0.109208305	-0.328968619	0.524347622	0.623912528	0.185565438	-0.846471629	0.033547036
Stx3	darkmagenta	0.71599929	0.109531367	0.348363762	0.498592601	-0.709117519	0.114612762	0.798817132	0.056640427
Mrpl22	darkmagenta	0.712175889	0.112342012	0.350881255	0.495277956	-0.741385933	0.091673639	0.898400978	0.014959171
Polr2m	darkmagenta	0.711900524	0.112545645	0.592110948	0.215629257	-0.745205963	0.089109387	0.813847484	0.04875379
Aldoc	darkmagenta	0.707477992	0.115838261	0.522014229	0.288102796	-0.636548378	0.174140173	0.810033119	0.050703417
Map2	darkmagenta	0.704566265	0.118028748	0.386498745	0.449119721	-0.617867492	0.191137382	0.911834946	0.011316958
Tmem198	darkmagenta	-0.703219802	0.119047754	-0.471603931	0.345038882	0.897498697	0.01522131	-0.875555102	0.022266189
Nicn1	darkmagenta	0.703178361	0.119079177	0.341603498	0.507526113	-0.707131751	0.116097797	0.821222342	0.04508518
Pnmal2	darkmagenta	0.70171196	0.120193431	0.659817886	0.15390221	-0.800750085	0.055595629	0.886046018	0.01873839
Elfn2	darkmagenta	-0.70164017	0.120248097	-0.321948669	0.533762138	0.708311306	0.11521467	-0.781098741	0.066632013
Pfkl	darkmagenta	-0.701251495	0.120544252	-0.423231771	0.403058066	0.7186874	0.10757482	-0.816494325	0.047421784
Gsg1l	darkmagenta	0.698276174	0.122821845	0.442534873	0.379530067	-0.786061094	0.063758807	0.945363836	0.004396118
Mkl1	darkmagenta	0.696786022	0.123969523	0.434037512	0.389827583	-0.703000592	0.119214014	0.964615279	0.001855965
B3galnt1	darkmagenta	0.69458678	0.125671802	0.315765628	0.542093727	-0.588144541	0.219506904	0.827590577	0.042025078
Zfp385a	darkmagenta	-0.693625435	0.126419079	-0.554646984	0.253343459	0.42269523	0.403718899	-0.75725631	0.081234973
Sparcl1	darkmagenta	0.692176052	0.127549356	0.388511731	0.44655365	-0.783780125	0.0650723	0.90745252	0.012451218
Nrep	darkmagenta	0.692113451	0.127598272	0.462784587	0.355380309	-0.781062855	0.066653001	0.89782876	0.015125163
Slc2a3	darkmagenta	0.690782329	0.128640341	0.613066322	0.195611103	-0.426203187	0.399404945	0.7302778	0.099313944
Tmem130	darkmagenta	-0.689981472	0.12926906	-0.384953335	0.451092936	0.812329751	0.049525299	-0.840339666	0.036202149
Fbxo2	darkmag	0.68840	0.1305099	0.6500	0.16231	-0.485226	0.3292822	0.654875751	0.158112117

	enta	5718	95	02198	0596	473	98		
Prmt2	darkmagenta	-0.685904709	0.132490108	-0.432878558	0.391239388	0.768111176	0.074424027	-0.937394243	0.00575653
Homer1	darkmagenta	-0.679065345	0.137970596	-0.397188459	0.435547273	0.806255043	0.05266935	-0.88300168	0.019732138
Ank	darkmagenta	0.677962021	0.138863658	0.523924746	0.286020803	-0.575552753	0.231999953	0.878806994	0.021141591
Cdk11b	darkmagenta	0.677658777	0.139109546	0.518487379	0.291961195	-0.564300047	0.243396243	0.812255174	0.049563353
Snord80	darkmagenta	-0.673815059	0.142242437	-0.307011087	0.553952159	0.701795216	0.120130047	-0.909369021	0.011948741
Dcps	darkmagenta	0.673680878	0.142352344	0.498756472	0.313900128	-0.610922977	0.197621974	0.916899695	0.01007156
Dars	darkmagenta	-0.672594478	0.143243548	-0.563568343	0.244144753	0.420361673	0.406597271	-0.616862159	0.192070624
Msra	darkmagenta	-0.668922091	0.14627372	-0.350054054	0.496366352	0.698891556	0.122349249	-0.881564398	0.02020984
Rpl21	darkmagenta	0.665413706	0.149193851	0.555316789	0.252648206	-0.89244769	0.016729194	0.833587331	0.039235517
Dnajc14	darkmagenta	-0.661490028	0.152488742	-0.544747101	0.263706037	0.665490841	0.149129385	-0.834507631	0.038815356
Setd6	darkmagenta	0.658193404	0.155280692	0.284451263	0.584830941	-0.716442195	0.109207806	0.804525655	0.053580771
Gm15500	darkmagenta	0.654512504	0.158423443	0.486679599	0.327617345	-0.78202336	0.066092272	0.786813226	0.063328381
Plekho1	darkmagenta	0.654018848	0.158846953	0.339367822	0.510490852	-0.766509154	0.075412239	0.918899348	0.009599261
Adgrb2	darkmagenta	-0.64721638	0.164731355	-0.453361513	0.366548936	0.772993956	0.071448607	-0.778842131	0.0679577
Lamp1	darkmagenta	0.646437284	0.165411056	0.221045486	0.673832034	-0.518463401	0.291987494	0.705070489	0.117648136
Ndufs3	darkmagenta	0.642822311	0.168580219	0.663880653	0.150477577	-0.318808186	0.537989339	0.463444239	0.354603049
Dact3	darkmagenta	-0.640625749	0.170518212	-0.301761559	0.561096871	0.755433364	0.082405147	-0.81841806	0.046464442
Tfdp1	darkmagenta	0.635343794	0.175216298	0.48623346	0.328128192	-0.670723542	0.144783911	0.907301338	0.012491281
Polr2f	darkmagenta	0.634160457	0.176276136	0.530550781	0.278844641	-0.578520561	0.229030536	0.861801568	0.027328497
Aatk	darkmagenta	-0.633995341	0.176424231	-0.509079628	0.302347623	0.735806051	0.095477501	-0.741250653	0.091765044
Smim8	darkmagenta	-0.631839434	0.178362656	-0.282985168	0.58685306	0.740621537	0.092190654	-0.757574838	0.081031281
Gm49550	darkmag	-0.6316	0.1785580	-0.2185	0.67733	0.558917	0.2489238	-0.720765099	0.106071925

	enta	2265	6	90584	6454	225	09		
Depp1	darkmag	-0.6315	0.1786514	-0.3731	0.46625	0.552938	0.2551205	-0.877237784	0.021680795
	enta	19054	7	49045	5108	24	02		
Arhgap20	darkmag	0.62846	0.1814132	0.2701	0.60459	-0.743508	0.0902450	0.807338533	0.052102015
	enta	6239	39	79247	2243	095	89		
Prrt1	darkmag	-0.6217	0.1875600	-0.3492	0.49745	0.782496	0.0658170	-0.807923671	0.051796808
	enta	40059	48	28196	37	175	45		
Ubxn1	darkmag	0.62016	0.1890107	0.6287	0.18119	-0.786083	0.0637460	0.841364716	0.03575169
	enta	6024	19	02447	8921	404	2		
Gm45453	darkmag	0.61839	0.1906462	0.2165	0.68023	-0.649477	0.1627652	0.761076977	0.07880695
	enta	7339	84	62355	4774	699	09		
Mafg	darkmag	-0.6172	0.1917194	-0.2128	0.68556	0.547526	0.2607809	-0.666628654	0.148179826
	enta	40224	5	38162	355	026	38		
Rps8	darkmag	0.61671	0.1922081	0.4559	0.36349	-0.783719	0.0651074	0.841607375	0.035645441
	enta	4192	39	27519	5527	443	11		
Kcnc4	darkmag	0.61482	0.1939630	0.5421	0.26644	-0.749296	0.0863995	0.891390667	0.017053404
	enta	97	47	56087	4712	728	78		
Ppp1r1a	darkmag	0.61300	0.1956650	0.4134	0.41514	-0.736480	0.0950137	0.912352527	0.011186462
	enta	8689	63	61065	8998	969	39		
Fam163b	darkmag	0.61278	0.1958736	0.3509	0.49518	-0.737191	0.0945265	0.832203535	0.039871271
	enta	5944	72	50824	6452	559	52		
Tmem35a	darkmag	0.61265	0.1960005	0.5201	0.29011	-0.733849	0.0968273	0.892928104	0.016582829
	enta	055	17	75732	1703	845	64		
Ccdc85b	darkmag	-0.6087	0.1996334	-0.2445	0.64043	0.699617	0.1217926	-0.794303069	0.059115196
	enta	87955	08	88327	3568	633	64		
Shisa9	darkmag	0.60800	0.2003694	0.4954	0.31759	-0.691490	0.1280854	0.899965317	0.014509886
	enta	8938	05	84975	4646	585	25		
Ttc3	darkmag	-0.6027	0.2053964	-0.4183	0.40909	0.709009	0.1146930	-0.797799443	0.057194106
	enta	19003	21	40493	5887	913	2		
Amigo1	darkmag	0.59770	0.2102070	0.4353	0.38817	-0.326704	0.5273787	0.669820638	0.145529802
	enta	5904	63	93079	849	549	23		
Fbxo11	darkmag	0.59542	0.2124099	0.1755	0.73931	-0.425079	0.4007847	0.738912458	0.093351318
	enta	5843	73	95508	3875	763	83		
Ndfip1	darkmag	-0.5895	0.2181477	-0.2342	0.65503	0.675191	0.1411168	-0.889886757	0.017519832
	enta	31577	41	6293	3676	862	08		
Zfp180	darkmag	0.58160	0.2259616	0.6140	0.19471	-0.657703	0.1556976	0.785391009	0.064143403
	enta	3987	31	2447	4905	197	9		
Sox10	darkmag	-0.5789	0.2285979	-0.3678	0.47314	0.738753	0.0934593	-0.818366717	0.046489874
	enta	5417	71	27466	1786	856	56		
Laptm4a	darkmag	-0.5768	0.2307179	-0.5943	0.21345	0.422669	0.4037509	-0.641061054	0.170133412
	enta	32205	38	45829	6686	242	17		
Dctn2	darkmag	0.57537	0.2321740	0.5557	0.25216	-0.288920	0.5786782	0.475540619	0.340458183
	enta	9215	56	81478	6301	392	25		
Jun	darkmag	0.57527	0.2322796	0.5777	0.22979	-0.785900	0.0638508	0.821909214	0.044750299

	enta	4018	21	54116	5936	564	47		
Gm13375	darkmag	-0.5748	0.2327160	-0.2421	0.64383	0.540901	0.2677749	-0.872900516	0.023204818
	enta	39344	18	79238	3144	296	41		
Nudc-ps1	darkmag	0.57295	0.2346144	0.5094	0.30193	-0.674892	0.1413611	0.712355015	0.112209635
	enta	2145	75	53126	2677	607	42		
Them6	darkmag	0.55822	0.2496355	0.3224	0.53314	-0.453716	0.3661265	0.846036925	0.033732124
	enta	7541	6	10076	1869	062	08		
Pkia	darkmag	0.55674	0.2511716	0.4541	0.36561	-0.816420	0.0474585	0.757800062	0.080887393
	enta	167	77	45542	5032	771	67		
Eif1ad	darkmag	0.55634	0.2515782	0.2746	0.59842	-0.599972	0.2080263	0.663208456	0.151041929
	enta	8966	77	18851	6985	509	92		
Pfdn6	darkmag	0.55549	0.2524681	0.5053	0.30647	-0.299226	0.5645556	0.638701981	0.172223142
	enta	0387	34	79146	024	822	56		
Sdcbp	darkmag	0.55514	0.2528285	0.4400	0.38257	-0.719778	0.1067842	0.745442566	0.088951635
	enta	2983	44	12141	7315	211	73		
Ddx42	darkmag	0.55181	0.2562865	0.4052	0.42541	-0.608428	0.1999727	0.732933481	0.097462592
	enta	9488	94	3804	6604	584	95		
Cdv3	darkmag	0.55178	0.2563235	0.3218	0.53384	-0.685953	0.1324515	0.761006198	0.078851627
	enta	4107	07	84863	793	302	13		
Ndufa11	darkmag	0.54818	0.2600861	0.3646	0.47723	-0.527299	0.2823574	0.805217537	0.053215268
	enta	788	36	72537	9377	268	34		
Tceal9	darkmag	0.54571	0.2626819	0.4784	0.33709	-0.784906	0.0644221	0.801890302	0.054983528
	enta	8622	75	45463	2296	569	08		
Gm12184	darkmag	0.54350	0.2650127	0.4343	0.38940	-0.229719	0.6614824	0.603818913	0.204346997
	enta	9553	47	8209	8165	174	82		
Scn3b	darkmag	-0.5389	0.2698536	-0.2979	0.56629	0.613447	0.1952540	-0.902064543	0.013917364
	enta	45292	33	50858	8964	85	4		
Orai3	darkmag	-0.5387	0.2700607	-0.2141	0.68366	0.621963	0.1873547	-0.728629626	0.100470708
	enta	50745	22	67775	0044	21	6		
Mir7013	darkmag	0.53233	0.2769291	0.2852	0.58366	-0.459865	0.3588268	0.845222872	0.034080022
	enta	0378	62	9785	4115	697	4		
Echdc2	darkmag	0.52252	0.2875412	0.1837	0.72751	-0.572134	0.2354388	0.835714884	0.038267404
	enta	9002	57	25253	294	523	76		
Mgat5b	darkmag	0.51426	0.2966031	0.3045	0.55725	-0.465067	0.3526933	0.836778388	0.037787724
	enta	7566	13	80581	6997	206	05		
Plekha2	darkmag	0.50723	0.3044017	0.3690	0.47161	-0.734133	0.0966311	0.741595558	0.091532082
	enta	3452	98	08692	0442	508	02		
Atp5j	darkmag	-0.5021	0.3100710	-0.1817	0.73035	0.506077	0.3056910	-0.799230624	0.056416173
	enta	62197	4	62508	8738	141	28		
Fbxo44	darkmag	-0.5010	0.3113268	-0.2389	0.64838	0.512566	0.2984825	-0.871395435	0.023745197
	enta	43534	41	55332	9135	129	27		
Dok5	darkmag	0.50022	0.3122422	0.1682	0.75002	-0.582493	0.2250791	0.746066763	0.08853606
	enta	914	57	38471	3219	7	89		
Psmd12	darkmag	0.49304	0.3203653	0.3332	0.51867	-0.467825	0.3494566	0.767373385	0.074878402

	enta	0714	52	19062	0887	002	41		
Syngap1	darkmagenta	-0.475734456	0.340233206	-0.404632956	0.426175404	0.477889113	0.337736011	-0.783454054	0.065261068
C1ql2	darkmagenta	0.474851884	0.341257999	0.454314557	0.365413817	-0.763040443	0.077572128	0.734686651	0.096248901
Sertm1	darkmagenta	0.418664863	0.40869455	0.372783204	0.466727535	-0.642542762	0.168826345	0.709522072	0.11431125
Pld3	darkmagenta	-0.412249241	0.416656901	-0.260062341	0.618700812	0.692584192	0.127230633	-0.740005394	0.09260834
Gm8430	darkmagenta	0.398176649	0.434299414	0.314250188	0.54414132	-0.487362563	0.326835886	0.632466763	0.1777977
Cadm3	darkmagenta	-0.380835956	0.456363533	-0.256238762	0.624053958	0.650778702	0.161638541	-0.667749163	0.14724724
Psat1	darkmagenta	-0.368410014	0.472386377	-0.322470177	0.53306109	0.601066095	0.206977572	-0.656380083	0.156825571
Adam9	darkmagenta	0.343026869	0.505641242	0.062978508	0.905657133	-0.350639137	0.495596451	0.764009184	0.076966137
Mpped1	darkmagenta	0.319883997	0.536540194	0.226725521	0.66573907	-0.51210497	0.298992693	0.68199544	0.135610943
Rn7s1	darkmagenta	-0.309743565	0.550243218	-0.194373871	0.712111033	0.5035186	0.308550882	-0.666116465	0.148606952
Rn7s2	darkmagenta	-0.30919302	0.550989947	-0.211680136	0.687222329	0.51995328	0.290355132	-0.655042576	0.157969225
Rnf227	darkmagenta	-0.308738952	0.551606031	-0.271056014	0.603373406	0.612286639	0.196341619	-0.672731209	0.143131253
Tmem68	darkmagenta	-0.300240874	0.563171233	-0.107899373	0.838779037	0.374567363	0.464424989	-0.739753336	0.092779454
Ccdc88c	violet	-0.94570006	0.004342674	-0.885421345	0.018940293	0.977291649	0.000767649	-0.845762392	0.033849262
Micu1	violet	-0.944197787	0.00458395	-0.827155188	0.042231096	0.965523123	0.001762492	-0.877349034	0.021642354
Supt4a	violet	0.94329009	0.004732831	0.881901614	0.020097271	-0.950504616	0.003614063	0.889339627	0.017691019
Rps24	violet	0.938634467	0.00553305	0.875426123	0.022311367	-0.963843543	0.001937301	0.85855405	0.028595484
Gm12966	violet	0.933571181	0.006472614	0.78525307	0.064222705	-0.951460778	0.003476904	0.877716825	0.021515502
Nop53	violet	0.929912919	0.007196158	0.884131584	0.01936044	-0.980989705	0.000538652	0.821779795	0.044813308
Usp31	violet	-0.927799347	0.007631213	-0.780757243	0.066831866	0.944630549	0.004513789	-0.804220071	0.05374257
Camta2	violet	-0.927467785	0.00770059	-0.844807649	0.034258121	0.979814054	0.000607096	-0.888948066	0.017814022
Ero1l	violet	-0.9267	0.0078474	-0.8866	0.01853	0.978815	0.0006684	-0.80549975	0.053066515

		70638	61	65468	9199	361	3		
Rbm14	violet	-0.926333027	0.007940346	-0.91834499	0.009729092	0.93392303	0.006404997	-0.863306747	0.026750508
Gpr37l1	violet	-0.926083194	0.007993612	-0.743233904	0.090429098	0.933279829	0.006528867	-0.82804973	0.041808325
Dusp6	violet	-0.924917451	0.008244449	-0.768443452	0.074219803	0.946656738	0.004192361	-0.907665893	0.01239478
Itpr1	violet	-0.924551826	0.008323899	-0.903537138	0.013508828	0.948939931	0.003844136	-0.839523817	0.036562568
Rps11	violet	0.921354598	0.009034434	0.852125906	0.031183359	-0.985647639	0.000307507	0.828856933	0.041428538
Lrrc58	violet	0.917941191	0.009824195	0.742338073	0.091031458	-0.936859685	0.005854188	0.826785763	0.04240627
Gm10045	violet	0.912877362	0.011054885	0.880566574	0.020544695	-0.949394477	0.00377658	0.863536166	0.026662927
Rpl10-ps1	violet	0.912574975	0.011130602	0.850528659	0.031842802	-0.989764982	0.000156597	0.83004724	0.040871458
Homer2	violet	-0.91097917	0.01153433	-0.951642987	0.003451062	0.936070816	0.005999774	-0.773856684	0.070928624
Prkcb	violet	-0.90913332	0.012009998	-0.813495865	0.048932028	0.971396416	0.001215546	-0.874845588	0.022515254
Rps15a	violet	0.90701287	0.012567898	0.895581118	0.015785699	-0.978510798	0.000687717	0.836680351	0.037831824
Rps27a	violet	0.906578268	0.012683755	0.92876215	0.007431487	-0.932582351	0.006664498	0.837990264	0.037244584
Snx1	violet	0.905805406	0.012891056	0.720588794	0.10619903	-0.938024574	0.005642408	0.8208567	0.045263921
Prpsap2	violet	0.90569451	0.012920934	0.717448458	0.108474257	-0.95246131	0.003336174	0.857948337	0.028834805
Shank2	violet	-0.902266763	0.013860915	-0.804029291	0.053843698	0.979619464	0.000618817	-0.85735163	0.029071492
Sult1a1	violet	0.902020747	0.013929604	0.756832319	0.081506466	-0.949965038	0.003692615	0.787702829	0.062821007
B3gat1	violet	-0.901210348	0.014157029	-0.888991799	0.017800264	0.96675534	0.00163944	-0.798323928	0.056908448
Rps18	violet	0.899169219	0.014737704	0.910258458	0.011718948	-0.937970823	0.005652096	0.856570731	0.029382623
Tspyl2	violet	0.896016886	0.015656574	0.789797195	0.0616339	-0.96088878	0.002264617	0.781679963	0.066292494
Agt	violet	0.89509305	0.015930927	0.742651002	0.09082084	-0.923738993	0.008501855	0.791668523	0.060582005
Fgfr1	violet	-0.893469795	0.016418538	-0.681207144	0.136244047	0.928486935	0.007488314	-0.855485696	0.02981753
Gm10093	violet	0.89265	0.0166667	0.7431	0.09050	-0.949290	0.0037919	0.949929815	0.003697772

		2495	21	20734	5095	763	42		
Rps10	violet	0.891480668	0.017025682	0.937938706	0.005657888	-0.928519502	0.007481579	0.771040615	0.072632299
Ankrd34a	violet	-0.890772618	0.017244356	-0.755325236	0.082474792	0.942688632	0.004832767	-0.769616628	0.073500776
Ccdc28b	violet	0.890371382	0.017368869	0.91018312	0.011738328	-0.966249205	0.001689451	0.782323562	0.065917463
Cnr1	violet	-0.889350284	0.017687677	-0.760486537	0.07918	0.963966971	0.001924176	-0.950049399	0.003680279
Ccdc115	violet	0.888960141	0.017810223	0.953809717	0.003151039	-0.928672835	0.007449906	0.734252929	0.096548529
Egr1	violet	-0.886778936	0.018502823	-0.891375597	0.017058047	0.965348828	0.001780253	-0.818056757	0.04664355
Klf13	violet	0.885108324	0.019041855	0.81566534	0.047837122	-0.99018768	0.00014395	0.802812752	0.054490619
Spsb1	violet	0.882960839	0.019745637	0.76106879	0.078812118	-0.939096381	0.005450923	0.745762411	0.088738581
Rpl10-ps3	violet	0.882799686	0.019798944	0.925592819	0.008098668	-0.95621529	0.002833681	0.737902769	0.094040058
Gm44870	violet	0.881813244	0.020126741	0.905369894	0.013008586	-0.968430445	0.001479224	0.754417026	0.083060819
Ptpru	violet	-0.880624899	0.02052505	-0.808581511	0.051454671	0.962102962	0.002127065	-0.872794326	0.02324275
Pcdh1	violet	-0.879992874	0.020738412	-0.751723004	0.084810121	0.960147774	0.002350653	-0.784433741	0.064694664
Gm9493	violet	0.879605951	0.020869551	0.848764212	0.032578843	-0.946424915	0.004228547	0.836106003	0.038090665
Caprin1	violet	-0.879420941	0.020932396	-0.847802478	0.03298337	0.951609657	0.003455782	-0.72345765	0.104139126
Uhmk1	violet	-0.878757616	0.021158459	-0.852523486	0.031020226	0.960472211	0.002312789	-0.833801998	0.039137322
Gm10095	violet	0.878276188	0.021323258	0.932671865	0.006647015	-0.942990192	0.004782533	0.714177701	0.110866541
Ifngr2	violet	-0.877774905	0.021495502	-0.665263626	0.149319316	0.917764015	0.009866065	-0.816010504	0.047663983
Zdhhc23	violet	-0.877182662	0.021699853	-0.905030607	0.013100505	0.964320945	0.001886783	-0.789738981	0.061666756
Gm2115	violet	-0.876111239	0.022071889	-0.793256282	0.059696028	0.993967679	5.45E-05	-0.886748531	0.018512567
Mcl1	violet	-0.875236197	0.022377973	-0.85396014	0.030434118	0.982874669	0.000437404	-0.762005753	0.078221745
Sbk1	violet	-0.872778638	0.023248356	-0.745934422	0.088624097	0.992160616	9.19E-05	-0.882955379	0.019747442
Raver2	violet	-0.8686	0.0247568	-0.8070	0.05225	0.982924	0.0004348	-0.795354478	0.058534429

		20945	53	51979	1783	968	46		
Rps17	violet	0.86810695	0.024946474	0.941648944	0.00500793	-0.924476769	0.008340254	0.741864824	0.091350394
Rpl36	violet	0.861873293	0.027300822	0.945453304	0.004381866	-0.928840792	0.007415287	0.693871748	0.126227431
Pcdh10	violet	-0.860516552	0.027826579	-0.902815714	0.013708236	0.936511412	0.005918246	-0.826140083	0.042713251
Rplp2	violet	0.859368735	0.028275086	0.932309386	0.006717949	-0.909980553	0.011790515	0.730895122	0.09888221
Rian	violet	-0.858929551	0.028447595	-0.785627365	0.064007626	0.989519831	0.000164175	-0.798558398	0.056780957
Tpd52l1	violet	0.85839413	0.028658577	0.77757933	0.06870475	-0.934816325	0.006234887	0.916863947	0.010080103
Rpl36-ps12	violet	0.858095103	0.02877673	0.747292258	0.087722694	-0.984295905	0.000367991	0.833708119	0.039180251
Eif3h	violet	0.856819161	0.02928347	0.807214315	0.052166913	-0.951209964	0.00351263	0.735766991	0.095504371
Psmb3	violet	0.856095447	0.029572755	0.891548394	0.017004836	-0.91379587	0.01082643	0.774822428	0.070348601
Rps18-ps3	violet	0.855058325	0.02998966	0.910048132	0.011773092	-0.928028458	0.007583451	0.812341661	0.049519223
Epha6	violet	-0.854944794	0.030035465	-0.875018719	0.022454357	0.970192675	0.001319473	-0.817344316	0.046997669
Trappc3	violet	0.854469469	0.030227598	0.918028571	0.009803577	-0.92109587	0.00909317	0.795889849	0.058239719
Shisa6	violet	-0.853542513	0.030603952	-0.666786874	0.14804799	0.890667428	0.017276957	-0.972317432	0.00113888
Chrm1	violet	-0.853026166	0.030814548	-0.737384352	0.094394564	0.988516752	0.00019704	-0.904006903	0.01337974
Usp46	violet	-0.847400642	0.033153089	-0.872117425	0.023485237	0.965066474	0.001809211	-0.8270638	0.042274399
Gm4202	violet	-0.846607987	0.033489076	-0.850025279	0.032051978	0.956002483	0.002861087	-0.74361735	0.090171815
Acadl	violet	0.842670255	0.035181809	0.711583794	0.112780069	-0.985549443	0.000311719	0.901893531	0.013965187
Hpcal4	violet	-0.842023421	0.03546362	-0.563974345	0.243729314	0.876668702	0.021877941	-0.846499086	0.033535361
Foxk1	violet	0.841606495	0.035645826	0.866469235	0.02555524	-0.976019795	0.00085568	0.741714717	0.091451659
Disp2	violet	-0.841366509	0.035750904	-0.670849222	0.144680216	0.899229913	0.014720275	-0.963842523	0.001937409
Rprm	violet	0.840621389	0.036078081	0.90664074	0.01266707	-0.946654396	0.004192726	0.699434729	0.121932768
Med4	violet	0.84031	0.0362135	0.9062	0.01277	-0.946738	0.0041796	0.773775179	0.070977675

		3711	89	55085	0243	604	19		
Neurod6	violet	-0.839005909	0.036792235	-0.815079402	0.048131703	0.97156758	0.001201111	-0.833008534	0.03950085
Zfp869	violet	0.838949597	0.036817247	0.753600372	0.083589362	-0.942139378	0.004924923	0.774929694	0.070284311
Rtn3	violet	-0.838837839	0.036866911	-0.707879772	0.115537414	0.944662447	0.004508639	-0.910956586	0.011540094
Bhlhe40	violet	-0.838555756	0.036992405	-0.716305858	0.109307362	0.983782351	0.000392385	-0.914473953	0.010659258
Btf3	violet	0.836916007	0.03772586	0.718361843	0.107810301	-0.925403007	0.008139512	0.689455243	0.129682904
Hdac3	violet	0.834553208	0.038794604	0.602374209	0.20572584	-0.912493131	0.011151138	0.827787547	0.04193203
Gm10052	violet	0.834162521	0.038972667	0.777577452	0.068705864	-0.959416031	0.002437166	0.882319906	0.019958054
Ehd3	violet	0.834054793	0.039021834	0.697447853	0.123459224	-0.91512533	0.010499858	0.975530828	0.000890785
Thbs4	violet	-0.833566563	0.039245023	-0.868084464	0.024954785	0.943662767	0.004671422	-0.828843992	0.041434614
Rcc2	violet	-0.833165985	0.039428588	-0.766875075	0.075185999	0.97606973	0.000852135	-0.883763071	0.019481295
Sptbn2	violet	-0.832583872	0.039696057	-0.900651626	0.01431486	0.949765035	0.003721942	-0.696015672	0.124564648
Cacna2d3	violet	-0.83161222	0.040144396	-0.812847771	0.049261342	0.940710158	0.005168718	-0.897136534	0.015327145
Ankrd63	violet	-0.829369355	0.041188285	-0.760988805	0.078862608	0.969049446	0.001422081	-0.840455612	0.036151064
Prkar1a	violet	-0.828807571	0.041451716	-0.853567589	0.030593741	0.960702024	0.002286152	-0.703586196	0.118770087
Midn	violet	-0.826528921	0.042528258	-0.814713009	0.048316335	0.979366136	0.000634242	-0.725539676	0.102655366
Gpc5	violet	0.826431251	0.04257469	0.544565715	0.263897404	-0.76931047	0.073688111	0.702554437	0.119552715
Fam114a2	violet	0.826101748	0.042731509	0.816497979	0.047419957	-0.911087045	0.011506819	0.899005631	0.01478473
Snhg8	violet	0.825723211	0.042911997	0.733578665	0.097015155	-0.953405889	0.003205939	0.738937832	0.093334039
Snrnp35	violet	0.825191409	0.043166161	0.710487277	0.113593292	-0.963506985	0.001973311	0.75513959	0.082594429
Commd9	violet	0.823517221	0.043970876	0.834755876	0.038702384	-0.924868239	0.008255121	0.835134746	0.038530264
Nr1d1	violet	-0.823202388	0.044122977	-0.654793061	0.158182965	0.877300636	0.021659073	-0.690072797	0.129197298
Ripor1	violet	-0.8196	0.0458468	-0.6998	0.12160	0.944956	0.0044613	-0.71630747	0.109306184

		68921	27	61612	5887	37	16		
Cwc27	violet	0.818380005	0.046483291	0.57861053	0.228940757	-0.904613756	0.013213866	0.86086523	0.027691007
Krt9	violet	0.817217771	0.047060698	0.706951697	0.116232862	-0.967954682	0.0015239	0.928318566	0.007523184
Pabpn1	violet	0.816926434	0.047205955	0.812584982	0.049395165	-0.933940201	0.006401706	0.823922946	0.043775224
Tmem63c	violet	-0.816063213	0.04763757	-0.537677188	0.271204584	0.836523858	0.037902269	-0.762046456	0.078196144
Pdk4	violet	0.815990225	0.047674147	0.752590143	0.084245275	-0.966977041	0.001617768	0.794558965	0.058973604
Slc9a2	violet	-0.815522518	0.047908849	-0.819453829	0.045952755	0.978735921	0.000673434	-0.800790603	0.055573825
Hmgn3	violet	0.814640162	0.048353083	0.686766299	0.13180651	-0.945350208	0.004398291	0.95492654	0.003001639
Ptpn5	violet	-0.814503307	0.048422155	-0.79262281	0.060048789	0.968753622	0.001449251	-0.855884041	0.029657513
Slc8a1	violet	-0.813154506	0.049105354	-0.844416789	0.034426169	0.9481274	0.003966361	-0.828321873	0.041680102
Ssbp2	violet	0.81291597	0.04922664	0.804182573	0.05376244	-0.972552445	0.001119713	0.705189401	0.117558454
Uhrf1bp1l	violet	-0.811810222	0.049790682	-0.656597636	0.156639882	0.938942501	0.005478216	-0.821153686	0.045118717
Crlf1	violet	0.811613866	0.049891154	0.904594074	0.01321923	-0.918599088	0.009669477	0.757893633	0.080827648
Atf2	violet	-0.80998979	0.050725767	-0.869990195	0.024255076	0.953157343	0.00323996	-0.67078605	0.144732333
Gjb6	violet	0.807513758	0.052010531	0.678177861	0.138688756	-0.900923538	0.014237944	0.654743683	0.158225278
Fam234b	violet	-0.807352447	0.052094747	-0.729188187	0.100078018	0.954497473	0.003058614	-0.877894537	0.021454336
Mxd4	violet	0.807291267	0.052126705	0.774340027	0.070638059	-0.953900546	0.003138755	0.795441274	0.058486603
St6galnac5	violet	-0.806298374	0.052646606	-0.634353793	0.176102795	0.884788061	0.019146037	-0.73856892	0.093585402
Rgl1	violet	-0.805136681	0.053257923	-0.866986273	0.025362295	0.955112929	0.002977053	-0.70581102	0.117090124
Ube2n	violet	-0.804748633	0.053462851	-0.861665303	0.027381112	0.924646921	0.008303199	-0.710242771	0.113774977
Dyrk2	violet	-0.804743518	0.053465555	-0.748893974	0.086664705	0.972980831	0.001085191	-0.838215777	0.037143921
Nos1ap	violet	-0.803734028	0.054000383	-0.790003058	0.061517776	0.913724502	0.010844098	-0.89574044	0.01573843
Btg2	violet	0.80358	0.0540781	0.7835	0.06517	-0.939231	0.0054270	0.867877608	0.025031304

		758	75	99797	6664	122	79		
St8sia1	violet	-0.802419298	0.05470061	-0.830567235	0.0406292	0.977506599	0.000753239	-0.76391198	0.077026845
Shank1	violet	-0.80175841	0.055054172	-0.652898943	0.159809496	0.923262927	0.008606931	-0.804702271	0.05348736
Lmtk2	violet	-0.800854695	0.055539342	-0.776094024	0.069588191	0.946715594	0.004183199	-0.774781986	0.070372847
Nptn	violet	-0.800627528	0.055661607	-0.654734719	0.15823296	0.901108825	0.014185645	-0.929237315	0.007333869
Cadps2	violet	-0.800533686	0.055712151	-0.904893167	0.013137829	0.912887501	0.011052351	-0.735670837	0.095570533
D10Jhu81e	violet	0.799743504	0.056138587	0.887835238	0.018165832	-0.920299035	0.009275226	0.61259913	0.1960487
Wscd2	violet	-0.796415396	0.057951083	-0.707659248	0.115702498	0.976978647	0.000788874	-0.768326835	0.074291449
Ptpn1	violet	-0.796227174	0.05805438	-0.561944862	0.245808751	0.870826045	0.023951174	-0.741921271	0.091312327
Rasgrp1	violet	-0.794405341	0.059058588	-0.897054083	0.015351289	0.929115178	0.007358901	-0.703418522	0.118897121
Sez6	violet	-0.791853467	0.060478497	-0.675399081	0.140947724	0.922300418	0.008821293	-0.931566629	0.006864448
Scn2a	violet	-0.791238587	0.060822942	-0.633767618	0.176628566	0.933780095	0.006432424	-0.822212421	0.044602842
Dnaja4	violet	-0.790927451	0.060997576	-0.558557301	0.249295149	0.927281439	0.007739716	-0.868966352	0.024629814
Kcnk1	violet	0.7908245	0.061055411	0.547143481	0.261182854	-0.886582963	0.018565671	0.862094432	0.027215578
Rab3c	violet	-0.790150432	0.061434706	-0.789009046	0.062079413	0.943977233	0.00461991	-0.868286463	0.02488017
Tprn	violet	0.78988349	0.06158521	0.676419586	0.140116299	-0.920739131	0.009174458	0.728116323	0.100832181
Clip3	violet	-0.789187009	0.061978688	-0.851543114	0.031423217	0.936382329	0.005942075	-0.659710976	0.153992771
6330420H09Rik	violet	-0.789103483	0.062025953	-0.810095701	0.050671144	0.904624283	0.013210997	-0.807123051	0.052214618
Dner	violet	-0.788623412	0.062297934	-0.87114743	0.023834808	0.931637087	0.006850485	-0.765928903	0.075771624
Tmem184b	violet	-0.787872184	0.06272463	-0.513611699	0.297327058	0.759011355	0.080115519	-0.65981615	0.153903681
Mdp1	violet	0.786858089	0.063302749	0.649769295	0.1625124	-0.948754807	0.003871818	0.916967982	0.01005525
Gm7887	violet	0.786753867	0.063362302	0.690928776	0.128525514	-0.98333009	0.000414513	0.807511771	0.052011568
Lix1	violet	-0.7858	0.0638962	-0.7602	0.07936	0.960876	0.0022659	-0.835442158	0.038390871

		21379	7	01292	0507	942	79		
H3f3b	violet	0.785770854	0.063925261	0.786804647	0.063333283	-0.979912356	0.000601217	0.735819972	0.095467925
Dgkz	violet	-0.784303609	0.064769769	-0.759839941	0.079589443	0.964147769	0.001905032	-0.74746631	0.087607448
Hapln4	violet	-0.783539693	0.065211466	-0.664660438	0.149824026	0.944873611	0.004474616	-0.932330748	0.006713758
Inka2	violet	-0.78328789	0.065357359	-0.649479061	0.162764028	0.90655765	0.012689265	-0.679432294	0.137674127
Ncald	violet	0.782355133	0.065899091	0.635363685	0.175198506	-0.891190157	0.017115241	0.945970539	0.004299913
Fibcd1	violet	-0.779415873	0.067619515	-0.675988718	0.140467077	0.956177024	0.0028386	-0.920412962	0.009249089
Gm22357	violet	0.778682198	0.068052107	0.873897973	0.022849962	-0.925601185	0.00809687	0.611634732	0.196953275
Agap2	violet	-0.778244869	0.068310565	-0.581942757	0.225625467	0.93725411	0.005782053	-0.907597268	0.012412918
Kif3c	violet	-0.777987547	0.068462849	-0.804471413	0.053609474	0.924297001	0.008379491	-0.749387292	0.086340012
Bcr	violet	-0.777646964	0.068664645	-0.849343473	0.032336329	0.911958463	0.01128575	-0.572101643	0.235472053
Cntnap2	violet	-0.777561534	0.068715304	-0.740168062	0.092497984	0.899634641	0.014604307	-0.915837197	0.010326988
Crk	violet	-0.775261229	0.070085774	-0.567846381	0.239781322	0.912714929	0.011095527	-0.820996055	0.045195759
Opcml	violet	-0.775141367	0.070157523	-0.785925456	0.063836571	0.935544381	0.006097899	-0.819416004	0.045971395
Dlg2	violet	-0.77436198	0.070624874	-0.57429327	0.233264719	0.89367332	0.016357014	-0.793742705	0.059425801
Git1	violet	-0.773297245	0.071265612	-0.720258193	0.106437554	0.958501276	0.002547483	-0.763633628	0.077200809
H1f0	violet	0.772514515	0.071738322	0.775731156	0.069804801	-0.973647254	0.00103255	0.784286315	0.064779753
Vps41	violet	-0.770413244	0.07301435	-0.860832059	0.027703891	0.929638918	0.007251855	-0.738136091	0.093880702
Mcat	violet	0.769348882	0.073664595	0.674721584	0.14150086	-0.952978425	0.00326456	0.835413876	0.038403685
Nr4a1	violet	-0.769221597	0.073742531	-0.592900904	0.21486031	0.938301131	0.00559269	-0.850343903	0.031919501
Cnksr2	violet	-0.767855631	0.074581265	-0.846181527	0.033670502	0.91022656	0.011727152	-0.768142509	0.074404759
Fbxl16	violet	-0.766345709	0.075513392	-0.520267075	0.290011769	0.847399455	0.033153591	-0.716769974	0.108968622
Junb	violet	-0.7661	0.0756387	-0.4888	0.32510	0.870824	0.0239518	-0.811536754	0.049930637

		43359	08	80558	1417	082	85		
Tmem178b	violet	-0.765320236	0.076149442	-0.674529421	0.141657919	0.909580487	0.011893912	-0.86445114	0.026314987
Ctsf	violet	0.763888004	0.077041823	0.660718341	0.153140364	-0.94849951	0.003910153	0.828040342	0.041812752
Id2	violet	0.761765244	0.078373097	0.795140656	0.058652324	-0.930957166	0.006985809	0.675453368	0.140903442
Rasl10b	violet	-0.76152726	0.078522989	-0.680272338	0.136996463	0.952850288	0.003282234	-0.726526724	0.10195524
Adra1d	violet	-0.759262941	0.07995562	-0.625700783	0.183930213	0.906473124	0.012711862	-0.743854801	0.090012655
Nxf1	violet	0.757292929	0.081211545	0.860676484	0.027764356	-0.911953064	0.011287113	0.58193272	0.225635424
Hmgcs1	violet	-0.755952276	0.082071283	-0.608397509	0.200002153	0.960474661	0.002312504	-0.849260982	0.032370812
Mal2	violet	-0.755788764	0.082176419	-0.719581634	0.106926416	0.9600663	0.002360209	-0.865261255	0.026008735
Cs	violet	-0.75338911	0.083726338	-0.772607315	0.071682204	0.95339033	0.003208063	-0.71804301	0.108041862
Pfdn4	violet	0.752582521	0.084250232	0.817267326	0.047036011	-0.947035555	0.00413356	0.704035896	0.118429675
Slc6a11	violet	-0.752224078	0.084483519	-0.677973414	0.138854424	0.944719792	0.004499387	-0.826782084	0.042408016
Gm42067	violet	0.751426827	0.085003437	0.859040236	0.028404072	-0.930534657	0.007070551	0.66471935	0.149774701
Clip2	violet	-0.751426399	0.085003716	-0.685045544	0.133173305	0.945516292	0.004371845	-0.6718445	0.143860166
Fam53c	violet	-0.751194407	0.085155276	-0.487489443	0.326690783	0.843635497	0.034763242	-0.725034074	0.103014818
Vxn	violet	-0.750242939	0.085778138	-0.655859718	0.157270098	0.940505	0.005204187	-0.899022748	0.014779806
Prox1os	violet	0.750163991	0.085829911	0.726830351	0.1017403	-0.942517511	0.004861387	0.869410987	0.024466736
Tmem121b	violet	-0.747398663	0.087652232	-0.46936468	0.347654251	0.813119401	0.049123195	-0.703895353	0.118536017
Prickle2	violet	-0.739512608	0.092943008	-0.823815756	0.043826874	0.905602284	0.012945808	-0.775818467	0.069752654
Gm12276	violet	-0.738817968	0.093415677	-0.825161688	0.043180386	0.913819434	0.010820599	-0.568848028	0.238764179
Actr2	violet	-0.734767781	0.096192899	-0.744036454	0.08989098	0.887790126	0.018180163	-0.830517976	0.040652121
Calm1	violet	-0.733064965	0.097371333	-0.581997304	0.225571358	0.897577229	0.015198407	-0.674037617	0.14206022
Gm8991	violet	0.73083	0.0989249	0.8494	0.03227	-0.912034	0.0112666	0.590722321	0.21698364

		3916	79	9913	1307	094	61		
Jph4	violet	-0.72938163	0.09994218	-0.45157175	0.368683964	0.843248228	0.034930894	-0.790026464	0.061504579
Ovca2	violet	0.727797626	0.101056899	0.712073338	0.11241783	-0.971608683	0.001197658	0.739329463	0.093067525
Dab2ip	violet	-0.727230928	0.101457036	-0.804483588	0.053603031	0.896176071	0.015609532	-0.541072824	0.267592951
Tmem170b	violet	-0.726717427	0.101820216	-0.818088834	0.046627636	0.93915422	0.005440681	-0.670117846	0.145284097
Rpl8	violet	0.726610484	0.101895925	0.569594831	0.238006935	-0.878455384	0.021261846	0.956686678	0.002773437
Tmed2	violet	0.726480989	0.101987633	0.482657579	0.332233186	-0.867616075	0.025128208	0.780155083	0.067184932
Gde1	violet	-0.725869861	0.102420925	-0.516982592	0.293613339	0.832713661	0.039636348	-0.625487252	0.184125157
Nabp2	violet	0.723999835	0.10375183	0.423758815	0.402409287	-0.730571354	0.099108538	0.728286983	0.100711936
Cbx3	violet	0.723358915	0.104209724	0.465590449	0.352078387	-0.843830981	0.034678759	0.783240108	0.06538506
Dkk3	violet	-0.721840801	0.105297868	-0.563475975	0.244239306	0.881245651	0.020316521	-0.908668543	0.012131235
Zadh2	violet	0.721030886	0.105880436	0.653462785	0.159324574	-0.932918846	0.006598893	0.689754382	0.129447581
Rpl14-ps1	violet	0.719755723	0.10680053	0.857565153	0.028986692	-0.891509388	0.01701684	0.681285459	0.136181092
Smpd3	violet	-0.71906325	0.107301655	-0.587564005	0.220076783	0.944697476	0.004502986	-0.753795745	0.083462779
Ramp3	violet	0.719053035	0.107309056	0.713344238	0.111479818	-0.956466349	0.002801516	0.672078523	0.143667636
Mdga1	violet	-0.71872814	0.107544538	-0.611882106	0.196721083	0.948566059	0.003900142	-0.753644181	0.08356097
Gm7324	violet	0.717384319	0.108520948	0.604399166	0.203794261	-0.821053368	0.04516774	0.516968113	0.293629253
Oxld1	violet	-0.717073548	0.108747306	-0.64356077	0.167930776	0.934028702	0.006384758	-0.677721434	0.139058726
Tbc1d10b	violet	-0.716543685	0.109133723	-0.500207775	0.312266286	0.905009221	0.013106309	-0.858631461	0.028564966
Man1a	violet	-0.714908094	0.110330328	-0.758798779	0.080250738	0.889781457	0.017552716	-0.818213704	0.046565708
Ift20	violet	0.709218639	0.114537366	0.707479882	0.115836845	-0.933629426	0.006461397	0.797545869	0.057332451
Scd2	violet	-0.704353301	0.118189665	-0.690235859	0.129069207	0.920683762	0.009187107	-0.785829506	0.063891607
Crim1	violet	-0.7037	0.1186367	-0.6295	0.18047	0.827734	0.0419570	-0.865801798	0.025805338

		62265	56	00306	5784	549	57		
Edf1	violet	0.703361984	0.118939969	0.587242548	0.220392593	-0.919584102	0.009440062	0.881972579	0.020073619
Mical2	violet	-0.70139279	0.120436554	-0.795118568	0.058664509	0.926470155	0.007911183	-0.664448702	0.150001366
Lefty1	violet	-0.70099179	0.120742314	-0.57673226	0.230817981	0.902112881	0.01390386	-0.868347543	0.02485763
Pnmal1	violet	-0.700522357	0.121100683	-0.532535236	0.276708986	0.784482757	0.064666385	-0.526441094	0.283287361
Lingo1	violet	-0.696628421	0.124091175	-0.457792616	0.361281809	0.864320169	0.026364659	-0.763600397	0.077221589
Arc	violet	-0.69404046	0.126096234	-0.487864472	0.326262028	0.899478219	0.014649075	-0.758485078	0.080450469
Men1	violet	-0.691083635	0.128404142	-0.774095629	0.070784912	0.891720859	0.016951806	-0.537135108	0.271782871
Dsp	violet	0.690565071	0.12881077	0.57218655	0.235386383	-0.890177928	0.017429056	0.894204827	0.016196865
Stau2	violet	-0.689218739	0.129869085	-0.712893774	0.111811897	0.945380156	0.004393517	-0.659088799	0.154520244
Pou3f1	violet	-0.686959725	0.131653253	-0.655402974	0.157660715	0.914369612	0.0106849	-0.859855295	0.028084549
Snora52	violet	-0.682800803	0.134965445	-0.570724291	0.236863495	0.877126455	0.021719295	-0.584649122	0.222947119
Rnps1	violet	0.680292725	0.136980034	0.651667138	0.160871053	-0.914933559	0.010546666	0.80184761	0.05500639
Msl1	violet	-0.679056645	0.137977629	-0.424693136	0.401260027	0.777676327	0.068647237	-0.607306706	0.201033806
Cpne8	violet	-0.671664819	0.144008065	-0.737415587	0.094373188	0.90687788	0.012603829	-0.734391684	0.096452628
Smim15	violet	0.671658673	0.144013125	0.472121649	0.344435213	-0.792501212	0.060116613	0.637780403	0.173042399
Galnt9	violet	-0.667322364	0.147602164	-0.694937219	0.125399876	0.877147049	0.021712171	-0.77262129	0.071673755
Chpf	violet	0.664936519	0.149592928	0.709529258	0.114305897	-0.918598213	0.009669682	0.58854154	0.219117536
Lmo1	violet	-0.658385394	0.155117504	-0.404112002	0.426829057	0.818579406	0.046384561	-0.673870521	0.142197018
Pde6d	violet	0.654261138	0.158639031	0.572069853	0.235504131	-0.903461008	0.013529805	0.87043287	0.024093898
Rnf14	violet	-0.643615543	0.167882648	-0.696127865	0.124477898	0.920124124	0.009315423	-0.62011251	0.18906012
Tmem18	violet	0.643179567	0.168265896	0.4212746	0.405470384	-0.843232262	0.034937814	0.746859321	0.088009652
Tspan13	violet	-0.6421	0.1691677	-0.7530	0.08397	0.907054	0.0125569	-0.597929622	0.209991416

		55283	46	02011	7583	044	48		
Rplp0	violet	0.637451625	0.173335067	0.610040329	0.198452518	-0.858919141	0.02845169	0.855217872	0.029925345
Mapk3	violet	0.636541808	0.174146035	0.5572516	0.250644087	-0.826860755	0.042370682	0.888778221	0.017867504
Pkp4	violet	-0.634488712	0.175981872	-0.759233242	0.079974488	0.886520865	0.018585607	-0.535246893	0.273800897
Casp9	violet	0.634423573	0.176040249	0.413989179	0.414492421	-0.822282925	0.044568587	0.648086735	0.163973431
Lrrn2	violet	-0.634318503	0.17613443	-0.464497746	0.35336297	0.836399258	0.037958401	-0.889670563	0.01758738
Pip4k2b	violet	-0.622939488	0.186457725	-0.476604955	0.339223519	0.820410202	0.045482634	-0.557519518	0.250367065
Srsf7	violet	0.60663884	0.201666527	0.598816343	0.209137571	-0.863648192	0.026620209	0.786726415	0.063377992
Otub2	violet	-0.60393823	0.204233288	-0.295174771	0.570096859	0.75547087	0.082380995	-0.679915815	0.137283894

GO and KEGG for GSE160587

color	category	term	count	PValue	Genes
darkmagenta	CC	mitochondrion	10	0.027352804	ENSMUSG00000057388, ENSMUSG00000004096, ENSMUSG00000003072, ENSMUSG00000024530, ENSMUSG00000030007, ENSMUSG00000025645, ENSMUSG00000024963, ENSMUSG00000036943, ENSMUSG00000022037, ENSMUSG00000024038
darkmagenta	CC	membrane	25	0.011109571	ENSMUSG00000029550, ENSMUSG00000026587, ENSMUSG00000004933, ENSMUSG00000025232, ENSMUSG00000062753, ENSMUSG00000024963, ENSMUSG00000025579, ENSMUSG00000020386, ENSMUSG00000037014, ENSMUSG00000036943,

					ENSMUSG00000006024, ENSMUSG00000030298, ENSMUSG00000034614, ENSMUSG00000053293, ENSMUSG00000014748, ENSMUSG00000053550, ENSMUSG00000003072, ENSMUSG00000028414, ENSMUSG00000033161, ENSMUSG00000025645, ENSMUSG00000022037, ENSMUSG00000024038, ENSMUSG00000022855, ENSMUSG00000020955, ENSMUSG00000032018
darkmagenta	BP	vesicle-mediated transport	5	0.003104083	ENSMUSG00000030298, ENSMUSG00000006024, ENSMUSG00000036093, ENSMUSG00000020955, ENSMUSG00000020386
violet	KEGG	Glutamatergic synapse	4	0.02012458	ENSMUSG00000025813, ENSMUSG00000037541, ENSMUSG00000052889, ENSMUSG00000030102
violet	CC	presynapse	4	0.039779781	ENSMUSG00000044288, ENSMUSG00000032382, ENSMUSG00000067889, ENSMUSG00000030102
violet	CC	dendrite	7	0.043942543	ENSMUSG00000027184, ENSMUSG00000054640, ENSMUSG00000032773, ENSMUSG00000021831, ENSMUSG00000025813, ENSMUSG00000020889, ENSMUSG00000030102
violet	CC	postsynaptic density	8	8.78E-04	ENSMUSG00000054640, ENSMUSG00000032773, ENSMUSG00000025813, ENSMUSG00000032382, ENSMUSG00000037541, ENSMUSG00000053930, ENSMUSG00000008668, ENSMUSG00000030102
violet	CC	glutamatergic synapse	9	0.001646049	ENSMUSG00000027184,

					ENSMUSG00000032773, ENSMUSG00000044288, ENSMUSG00000020612, ENSMUSG00000025813, ENSMUSG00000037541, ENSMUSG00000067889, ENSMUSG00000053930, ENSMUSG00000054814
violet	CC	synapse	16	4.18E-06	ENSMUSG00000057863, ENSMUSG00000027184, ENSMUSG00000054640, ENSMUSG00000032773, ENSMUSG00000061787, ENSMUSG00000008668, ENSMUSG00000024758, ENSMUSG00000044288, ENSMUSG00000020612, ENSMUSG00000025813, ENSMUSG00000020889, ENSMUSG00000025508, ENSMUSG00000037541, ENSMUSG00000053930, ENSMUSG00000054814, ENSMUSG00000030102
violet	BP	regulation of apoptotic process	5	0.013884478	ENSMUSG00000041560, ENSMUSG00000038418, ENSMUSG00000031980, ENSMUSG00000022194, ENSMUSG00000038612
violet	BP	positive regulation of neuron projection development	5	0.007898852	ENSMUSG00000044288, ENSMUSG00000031980, ENSMUSG00000030854, ENSMUSG00000030102, ENSMUSG00000031565
blue	KEGG	Chemical carcinogenesis - reactive oxygen species	21	1.09074E-08	ENSMUSG00000059734, ENSMUSG00000032330, ENSMUSG00000025781, ENSMUSG00000026032, ENSMUSG00000000563, ENSMUSG00000026895, ENSMUSG00000024122, ENSMUSG00000020022, ENSMUSG00000028648, ENSMUSG00000022354,

					ENSMUSG00000021520, ENSMUSG00000040048, ENSMUSG00000025204, ENSMUSG00000021764, ENSMUSG00000063882, ENSMUSG00000031818, ENSMUSG00000018770, ENSMUSG00000017778, ENSMUSG00000036199, ENSMUSG00000036751, ENSMUSG00000034566
blue	KEGG	Oxidative phosphorylation	22	0.003929247	ENSMUSG00000032330, ENSMUSG00000031818, ENSMUSG00000017778, ENSMUSG00000036751
blue	KEGG	Parkinson disease	29	1.61018E-13	ENSMUSG00000028367, ENSMUSG00000020022, ENSMUSG00000028648, ENSMUSG00000040048, ENSMUSG00000063882, ENSMUSG00000031818, ENSMUSG00000018770, ENSMUSG00000019505, ENSMUSG00000020708, ENSMUSG00000034566, ENSMUSG00000059734, ENSMUSG00000057378, ENSMUSG00000032330, ENSMUSG00000025781, ENSMUSG00000026032, ENSMUSG00000000563, ENSMUSG00000026895, ENSMUSG00000026750, ENSMUSG00000021024, ENSMUSG00000022354, ENSMUSG00000021520, ENSMUSG00000025204, ENSMUSG00000021764, ENSMUSG00000025889, ENSMUSG00000015671, ENSMUSG00000017778, ENSMUSG00000036199, ENSMUSG00000036751, ENSMUSG00000030137

blue	KEGG	Alzheimer disease	33	1.89392E-12	ENSMUSG00000057666, ENSMUSG00000054693, ENSMUSG00000040249, ENSMUSG00000020022, ENSMUSG00000028648, ENSMUSG00000040048, ENSMUSG00000063882, ENSMUSG00000031818, ENSMUSG00000018770, ENSMUSG00000020708, ENSMUSG00000034566, ENSMUSG00000059734, ENSMUSG00000057378, ENSMUSG00000032330, ENSMUSG00000025781, ENSMUSG00000026032, ENSMUSG00000000563, ENSMUSG00000026895, ENSMUSG00000002985, ENSMUSG00000026750, ENSMUSG00000029467, ENSMUSG00000021024, ENSMUSG00000022354, ENSMUSG00000021520, ENSMUSG00000025204, ENSMUSG00000021764, ENSMUSG00000025889, ENSMUSG00000015671, ENSMUSG00000022812, ENSMUSG00000017778, ENSMUSG00000036199, ENSMUSG00000036751, ENSMUSG00000030137
blue	KEGG	Pathways of neurodegeneration - multiple diseases	35	2.14605E-11	ENSMUSG00000020022, ENSMUSG00000028648, ENSMUSG00000040048, ENSMUSG00000063882, ENSMUSG00000031818, ENSMUSG00000018770, ENSMUSG00000031516, ENSMUSG00000019505, ENSMUSG00000020708, ENSMUSG00000031812, ENSMUSG00000034566,

					ENSMUSG00000059734, ENSMUSG00000057378, ENSMUSG00000032330, ENSMUSG00000025781, ENSMUSG00000015837, ENSMUSG00000026032, ENSMUSG00000000563, ENSMUSG00000022054, ENSMUSG00000026895, ENSMUSG00000026750, ENSMUSG00000029467, ENSMUSG00000021024, ENSMUSG00000022354, ENSMUSG00000021520, ENSMUSG00000025204, ENSMUSG00000021764, ENSMUSG00000025889, ENSMUSG00000015671, ENSMUSG00000022812, ENSMUSG00000017778, ENSMUSG00000036199, ENSMUSG00000036751, ENSMUSG00000030137, ENSMUSG00000050965
blue	MF	cytochrome-c oxidase activity	3	0.041530207	ENSMUSG00000032330, ENSMUSG00000031818, ENSMUSG00000017778
blue	MF	structural constituent of postsynaptic density	3	0.009087404	ENSMUSG00000022623, ENSMUSG00000040003, ENSMUSG00000000881
blue	CC	presynaptic active zone cytoplasmic component	3	0.029755399	ENSMUSG00000034799, ENSMUSG00000034101, ENSMUSG00000041115
blue	CC	mitochondrial respiratory chain complex III	3	0.01226394	ENSMUSG00000071654, ENSMUSG00000063882, ENSMUSG00000021520
blue	CC	peroxisomal membrane	4	0.046189608	ENSMUSG00000020777, ENSMUSG00000002763, ENSMUSG00000022210, ENSMUSG00000021670
blue	CC	mitochondrial respiratory chain complex IV	4	0.00276459	ENSMUSG00000032330, ENSMUSG00000031818, ENSMUSG00000017778, ENSMUSG00000036751

blue	CC	mitochondrial proton-transporting ATP synthase complex	4	0.002021153	ENSMUSG00000038690, ENSMUSG00000025781, ENSMUSG00000000563, ENSMUSG00000034566
blue	CC	coupling factor F(o)	4	7.47E-04	ENSMUSG00000038690, ENSMUSG00000018770, ENSMUSG00000000563, ENSMUSG00000034566
blue	CC	synaptic vesicle	7	0.047234945	ENSMUSG00000040560, ENSMUSG00000000605, ENSMUSG00000025889, ENSMUSG00000037062, ENSMUSG00000031760, ENSMUSG00000025917, ENSMUSG00000054693
blue	CC	mitochondrial membrane	8	0.010760021	ENSMUSG00000024978, ENSMUSG00000038084, ENSMUSG00000032330, ENSMUSG00000031818, ENSMUSG00000036199, ENSMUSG00000017778, ENSMUSG00000036751, ENSMUSG00000050965
blue	CC	postsynaptic density, intracellular component	8	2.84549E-07	ENSMUSG00000022623, ENSMUSG00000057666, ENSMUSG00000024431, ENSMUSG00000031292, ENSMUSG00000028059, ENSMUSG00000034101, ENSMUSG00000041115, ENSMUSG00000000881
blue	CC	dendritic spine	9	0.010453419	ENSMUSG00000022623, ENSMUSG00000022812, ENSMUSG00000031760, ENSMUSG00000028222, ENSMUSG00000054693, ENSMUSG00000024431, ENSMUSG00000034101, ENSMUSG00000049336, ENSMUSG00000070866
blue	CC	mitochondrial respiratory chain complex I	10	4.02249E-08	ENSMUSG00000021764, ENSMUSG00000059734, ENSMUSG00000026032, ENSMUSG00000036199,

					ENSMUSG00000026895, ENSMUSG00000020022, ENSMUSG00000028648, ENSMUSG00000022354, ENSMUSG00000040048, ENSMUSG00000025204
blue	CC	respiratory chain	12	9.53943E-10	ENSMUSG00000021764, ENSMUSG00000059734, ENSMUSG00000063882, ENSMUSG00000026032, ENSMUSG00000036199, ENSMUSG00000026895, ENSMUSG00000020022, ENSMUSG00000028648, ENSMUSG00000022354, ENSMUSG00000040048, ENSMUSG00000021520, ENSMUSG00000025204
blue	CC	axon	14	0.029004502	ENSMUSG00000034799, ENSMUSG00000031760, ENSMUSG00000029840, ENSMUSG00000028222, ENSMUSG00000054693, ENSMUSG00000022054, ENSMUSG00000028367, ENSMUSG00000025889, ENSMUSG00000022636, ENSMUSG00000022812, ENSMUSG00000022629, ENSMUSG00000031812, ENSMUSG00000050965, ENSMUSG00000070866
blue	CC	neuron projection	17	0.006692987	ENSMUSG00000034799, ENSMUSG00000029212, ENSMUSG00000028222, ENSMUSG00000026473, ENSMUSG00000022054, ENSMUSG00000024431, ENSMUSG00000022240, ENSMUSG00000049336, ENSMUSG00000040003, ENSMUSG00000021477, ENSMUSG00000046447, ENSMUSG00000022623,

					ENSMUSG00000047414,<br>ENSMUSG00000028519,<br>ENSMUSG00000037062,<br>ENSMUSG00000019505,<br>ENSMUSG00000050965
blue	CC	dendrite	18	0.00623173	ENSMUSG00000029212,<br>ENSMUSG00000028222,<br>ENSMUSG00000054693,<br>ENSMUSG00000040249,<br>ENSMUSG00000022240,<br>ENSMUSG00000002985,<br>ENSMUSG00000028367,<br>ENSMUSG00000049336,<br>ENSMUSG00000040003,<br>ENSMUSG00000046447,<br>ENSMUSG00000038084,<br>ENSMUSG00000042671,<br>ENSMUSG00000022636,<br>ENSMUSG00000022812,<br>ENSMUSG00000022629,<br>ENSMUSG00000030744,<br>ENSMUSG00000031812,<br>ENSMUSG00000050965
blue	CC	neuronal cell body	19	0.004806839	ENSMUSG00000028222,<br>ENSMUSG00000054693,<br>ENSMUSG00000040249,<br>ENSMUSG00000049313,<br>ENSMUSG00000028059,<br>ENSMUSG00000002985,<br>ENSMUSG00000028367,<br>ENSMUSG00000000881,<br>ENSMUSG00000046447,<br>ENSMUSG00000025889,<br>ENSMUSG00000022636,<br>ENSMUSG00000028519,<br>ENSMUSG00000022812,<br>ENSMUSG00000037062,<br>ENSMUSG00000032518,<br>ENSMUSG00000019505,<br>ENSMUSG00000031812,<br>ENSMUSG00000050965,<br>ENSMUSG00000070866
blue	CC	postsynaptic density	22	5.91295E-08	ENSMUSG00000024083,<br>ENSMUSG00000058600,

					ENSMUSG00000031760, ENSMUSG00000054693, ENSMUSG00000022054, ENSMUSG00000024431, ENSMUSG00000022240, ENSMUSG00000028059, ENSMUSG00000024122, ENSMUSG00000026113, ENSMUSG00000028649, ENSMUSG00000040003, ENSMUSG00000000881, ENSMUSG00000046447, ENSMUSG00000022623, ENSMUSG00000028519, ENSMUSG00000022812, ENSMUSG00000020315, ENSMUSG00000024608, ENSMUSG00000090862, ENSMUSG00000038467, ENSMUSG00000030744
blue	CC	glutamatergic synapse	24	1.70878E-06	ENSMUSG00000034799, ENSMUSG00000031673, ENSMUSG00000015829, ENSMUSG00000057666, ENSMUSG00000028222, ENSMUSG00000054693, ENSMUSG00000024431, ENSMUSG00000031292, ENSMUSG00000040118, ENSMUSG00000028059, ENSMUSG00000002985, ENSMUSG00000049336, ENSMUSG00000040003, ENSMUSG00000041115, ENSMUSG00000026755, ENSMUSG00000000881, ENSMUSG00000022623, ENSMUSG00000022812, ENSMUSG00000020315, ENSMUSG00000028936, ENSMUSG00000032518, ENSMUSG00000038467, ENSMUSG00000034101, ENSMUSG00000032601

blue	CC	mitochondrial inner membrane	30	7.27996E-12	ENSMUSG00000030541, ENSMUSG00000020022, ENSMUSG00000028648, ENSMUSG00000040048, ENSMUSG00000067847, ENSMUSG00000038084, ENSMUSG00000052738, ENSMUSG00000071654, ENSMUSG00000063882, ENSMUSG00000031818, ENSMUSG00000030744, ENSMUSG00000039016, ENSMUSG00000034566, ENSMUSG00000059734, ENSMUSG00000032330, ENSMUSG00000025781, ENSMUSG00000026032, ENSMUSG00000000563, ENSMUSG00000026895, ENSMUSG00000022354, ENSMUSG00000021520, ENSMUSG00000025204, ENSMUSG00000041028, ENSMUSG00000021764, ENSMUSG00000024978, ENSMUSG00000038690, ENSMUSG00000025889, ENSMUSG00000017778, ENSMUSG00000036199, ENSMUSG00000036751
blue	CC	synapse	32	3.473E-06	ENSMUSG00000031673, ENSMUSG00000024083, ENSMUSG00000029212, ENSMUSG00000056076, ENSMUSG00000028222, ENSMUSG00000026103, ENSMUSG00000031292, ENSMUSG00000040003, ENSMUSG00000046447, ENSMUSG00000048758, ENSMUSG00000022623, ENSMUSG00000047414, ENSMUSG00000025917, ENSMUSG00000017404,

					ENSMUSG00000030744, ENSMUSG00000034101, ENSMUSG00000032601, ENSMUSG00000034799, ENSMUSG00000063457, ENSMUSG00000049336, ENSMUSG00000002985, ENSMUSG00000026113, ENSMUSG00000041841, ENSMUSG00000026755, ENSMUSG00000062328, ENSMUSG00000062647, ENSMUSG00000000881, ENSMUSG00000049517, ENSMUSG00000044533, ENSMUSG00000042671, ENSMUSG00000025889, ENSMUSG00000032518
blue	BP	regulation of dendrite development	3	0.044429201	ENSMUSG00000039145, ENSMUSG00000031292, ENSMUSG00000028655
blue	BP	regulation of neuron death	3	0.044429201	ENSMUSG00000025889, ENSMUSG00000019505, ENSMUSG00000002985
blue	BP	regulation of long-term synaptic potentiation	3	0.025293867	ENSMUSG00000022623, ENSMUSG00000022812, ENSMUSG00000028222
blue	BP	mitochondrial electron transport, ubiquinol to cytochrome c	3	0.010838159	ENSMUSG00000071654, ENSMUSG00000063882, ENSMUSG00000021520
blue	BP	cochlea development	4	0.019422874	ENSMUSG00000038084, ENSMUSG00000028222, ENSMUSG00000054693, ENSMUSG00000004207
blue	BP	positive regulation of synaptic transmission, glutamatergic	4	0.013416653	ENSMUSG00000022623, ENSMUSG00000015829, ENSMUSG00000026473, ENSMUSG00000041115
blue	BP	mitochondrial electron transport, NADH to ubiquinone	4	0.005681129	ENSMUSG00000059734, ENSMUSG00000026895, ENSMUSG00000022354, ENSMUSG00000025204
blue	BP	oxidative phosphorylation	4	0.003929247	ENSMUSG00000032330, ENSMUSG00000031818,

					ENSMUSG00000017778, ENSMUSG00000036751
blue	BP	negative regulation of neuron projection development	5	0.043674228	ENSMUSG00000015829, ENSMUSG00000022812, ENSMUSG00000040249, ENSMUSG00000032290, ENSMUSG00000002985
blue	BP	cellular response to oxidative stress	5	0.035266579	ENSMUSG00000025889, ENSMUSG00000031760, ENSMUSG00000005161, ENSMUSG00000029467, ENSMUSG00000071866
blue	BP	positive regulation of axon extension	5	0.004584669	ENSMUSG00000022812, ENSMUSG00000031292, ENSMUSG00000040249, ENSMUSG00000002985, ENSMUSG00000028649
blue	BP	positive regulation of dendritic spine development	5	0.002503674	ENSMUSG00000022623, ENSMUSG00000024431, ENSMUSG00000031292, ENSMUSG00000002985, ENSMUSG00000042323
blue	BP	hippocampus development	6	0.010535322	ENSMUSG00000028519, ENSMUSG00000022812, ENSMUSG00000031010, ENSMUSG00000028655, ENSMUSG00000026872, ENSMUSG00000041911
blue	BP	negative regulation of neuron death	6	0.006482882	ENSMUSG00000025889, ENSMUSG00000022812, ENSMUSG00000031760, ENSMUSG00000038467, ENSMUSG00000049313, ENSMUSG00000002985
blue	BP	positive regulation of neuron projection development	7	0.047033939	ENSMUSG00000039145, ENSMUSG00000033342, ENSMUSG00000040249, ENSMUSG00000037742, ENSMUSG00000002985, ENSMUSG00000040003, ENSMUSG00000070866
blue	BP	mitochondrial respiratory chain complex I assembly	10	2.50074E-07	ENSMUSG00000021764, ENSMUSG00000059734, ENSMUSG00000026032,

		ENSMUSG00000036199, ENSMUSG00000026895, ENSMUSG00000020022, ENSMUSG00000028648, ENSMUSG00000022354, ENSMUSG00000040048, ENSMUSG00000025204

Supplementary Table S10: Results of differential analysis

DEG for GSE42546

Gene name	log2FoldChange	padj
SLC36A1	-4.770340474	3.4441E-08
PRTFDC1	-4.737252112	3.66535E-07
SLC43A2	4.032344984	1.78073E-07
CTNNA1	-3.72288829	0.000156
PRKG2	-2.71217325	0.004891237
LOC284100	-2.607499219	0.004103828
PTK7	-2.468882551	0.001170111
FAM82A1	-2.435927443	2.67157E-06
GAB1	-2.399267659	0.007298215
C6orf62	-2.336637876	0.000156
NHEDC2	-2.263820898	0.000821688
C7orf72	-2.260824536	0.003900867
NPTX2	-2.224720943	0.00042133
COL6A6	-2.127280616	0.009442617
WDR20	-2.085210129	0.00400547
DFNB31	-2.062338994	0.00042133
SLC26A5	-2.05027312	0.004672726
INO80C	-2.045340376	0.01515922
SHROOM4	-1.995168282	0.00042133
ASAP2	-1.973704925	0.000988854
LPCAT2	-1.967062725	0.002862942
DNAJC5B	-1.942479511	0.019246436
SRPR	-1.90871103	0.03146106
TNFAIP8	-1.906290408	0.012220128
SH3TC2	-1.899912262	0.008890957
C17orf76	-1.854469535	0.1069763
PRLR	-1.835479308	0.006556127
TBXAS1	-1.835188761	0.004707266
CDH23	-1.830325695	0.003900867
EVC2	-1.807612174	0.015623583
HSPB8	-1.803230561	0.053296023
RASD1	-1.800478198	0.032000673
NRN1	-1.792593782	0.008890957
AHNAK	-1.788910317	0.003986288
ADAMTS2	-1.751859072	0.009442617
HIAT1	-1.743836659	0.000821688
FBN2	-1.702546127	0.001189338
NR1H4	-1.679767781	0.067611686
MIR548Q	-1.676806894	0.009270202
BCAS1	-1.666337324	0.01515922
EGF	-1.636967155	0.065891574
KITLG	-1.630288593	0.077513519

SSFA2	-1.61363534	0.027574538
CCDC67	-1.602853219	0.003900867
KLHL22	-1.599434059	0.081099509
CDH6	-1.594911722	0.053296023
GRIK3	-1.587926279	0.012493605
LASS3	-1.580274732	0.020972549
ADAM5P	-1.578701054	0.014181554
MOCS2	-1.555212517	0.034075459
KIF1C	-1.554400758	0.050295954
PIK3CD	-1.552077362	0.109342719
CREB3L2	-1.544867369	0.014181554
SLC26A11	-1.517963561	0.002862942
RUNX1	-1.516883058	0.02503503
ALDH1L1	-1.512100902	0.047793214
GRTP1	-1.507407676	0.088216938
GTF2H2	-1.507156009	0.02503503
LPA	-1.505370785	0.01515922
SLCO1C1	-1.503298807	0.042348547
OTOA	-1.499720078	0.09462456
ASAP3	-1.496687711	0.023712191
SWAP70	-1.490585226	0.008180243
C18orf34	-1.488701826	0.015623583
C1QTNF7	-1.487980967	0.077807982
PSMB2	-1.485902896	0.119402467
C6orf138	-1.481285486	0.035790803
NDE1	-1.473722132	0.008554317
PRKRIR	-1.463954394	0.051992817
SMC1B	-1.463611496	0.119402467
DSCR4	-1.462042252	0.067037845
HSF5	-1.45946237	0.01515922
PKD1L1	-1.456515662	0.008365433
HMGA2	-1.44724092	0.088216938
UNC45B	-1.435848843	0.379974544
CCDC60	-1.432792257	0.039169102
MYO7B	-1.431685234	0.033213826
LOC285441	-1.428200033	0.032000673
POLE2	-1.425150557	0.081099509
LOC285419	-1.420297712	0.012220128
IL15	-1.414437033	0.019246436
NPEPPS	-1.414199536	0.026453394
KCNK12	-1.408757999	0.081099509
PTPRU	-1.406915129	0.126486463
ITPA	-1.401314313	0.033213826
KCNU1	-1.400076294	0.039169102

MCF2L	-1.398119464	0.051015292
SCOC	-1.394524606	0.077513519
ALOX5	-1.387435556	0.107199236
GLUD1	-1.378394339	0.034075459
BMP5	-1.376146044	0.102308035
LATS2	-1.374343661	0.173785205
DCDC2	-1.373507858	0.032000673
HCRTR2	-1.364200679	0.093467929
CEP110	-1.362278845	0.019246436
ICAM5	-1.3601634	0.019442713
C18orf2	-1.35852852	0.078619706
LIMD1	-1.34942249	0.13917175
SCEL	-1.34631895	0.095007874
OR9Q1	-1.344386029	0.033213826
CMAH	-1.344194419	0.060445488
DOK5	-1.343872127	0.1046717
ANKRD49	-1.342257809	0.067611686
ABCC6	-1.341663842	0.077513519
LRRIQ3	-1.340663033	0.043664924
SPATA17	-1.335403478	0.014181554
CPA6	-1.329522927	0.01515922
SLC1A3	-1.322436154	0.108581532
ZNF536	1.316746936	0.035790803
SLCO1B1	-1.31139567	0.13917175
FBLN2	-1.311026861	0.161926803
CES7	-1.307567395	0.059205443
MYBL2	-1.305198839	0.05029331
GDI2	-1.302704511	0.102308035
HSPG2	-1.297280431	0.107199236
SLC2A9	-1.295295256	0.047044932
ADAM28	-1.294248044	0.119402467
NHLRC2	-1.293169207	0.051037588
G2E3	-1.292179685	0.010244115
LOC388630	-1.291484543	0.051015292
MID2	-1.288616558	0.138913603
NCRNA00159	-1.283794599	0.091323641
DNAH12	-1.282595323	0.032853942
HSPA1B	-1.280132856	0.019442713
ANGPT1	-1.278939771	0.032853942
PLA2G4A	-1.278584658	0.09462456
ATP13A5	-1.260233058	0.199476753
MUSK	-1.258928404	0.108581532
C2orf77	-1.258263374	0.137631631
STX3	-1.255343917	0.078482225

ABI3BP	-1.25488903	0.081099509
SCNN1B	-1.254726568	0.081099509
CFTR	-1.253916914	0.10472139
MCF2	-1.24856109	0.087679648
AAA1	-1.240340503	0.032000673
FLVCR2	-1.239386946	0.173785205
PCDH11Y	-1.236614811	0.13917175
EFHC2	-1.233387922	0.012769718
TTC8	-1.232573842	0.015294083
GRK7	-1.23038158	0.178987535
KYNU	-1.229220647	0.034075459
CDH20	-1.224964764	0.033213826
RADIL	-1.224572879	0.192812351
LOC285768	-1.223732787	0.151960279
MGC27382	-1.219529442	0.142359332
AOAH	-1.219526312	0.031871875
COL15A1	-1.210613618	0.10472139
MORC4	-1.210258382	0.091110598
GADL1	-1.209739885	0.098875419
IL1RAPL2	-1.205044245	0.012509053
MAGI2	-1.200446162	0.007298215
STAC	-1.197336952	0.14629282
ST3GAL4	-1.195969806	0.088216938
PDE6A	-1.192643807	0.249188013
IFT122	-1.191940475	0.007298215
C1orf168	-1.190730909	0.148074071
DYTN	-1.190553935	0.160408786
PSMA8	-1.190443735	0.129011995
MCEE	1.184993615	0.160408786
C6	-1.18326284	0.153487158
ANAPC5	-1.182742235	0.058621169
EBF2	-1.178915594	0.108581532
C11orf65	-1.178781167	0.088216938
CHST10	-1.178769196	0.053296023
PRR5L	-1.177513788	0.077513519
ABCC8	-1.173511389	0.02503503
DDR2	-1.165676862	0.158472595
NR5A2	1.164459417	0.136201906
CNKSR3	-1.163082304	0.081099509
QTRT1	-1.159391751	0.093694064
PRPF8	-1.153613579	0.083444705
HEPHL1	-1.151868715	0.19997818
HAR1A	-1.146435271	0.350380986
SLC30A4	-1.145678114	0.09462456

IL1F8	-1.143705016	0.282668639
CPS1	-1.143000166	0.173785205
C6orf225	-1.139807827	0.083444705
B3GAT2	-1.138407603	0.09462456
DDX60	-1.136482682	0.194528221
SH3RF2	-1.136462827	0.151960279
STYK1	1.136360623	0.165077663
AR	-1.135789943	0.081099509
DNAH8	-1.131241949	0.038568833
FLI1	-1.121549875	0.108119529
IMPG1	-1.12131314	0.109999944
VAT1L	-1.121257354	0.093467929
C11orf64	-1.117497267	0.326562586
RXFP1	-1.111142718	0.192812351
SACM1L	-1.109937395	0.163330436
TRPV4	-1.109500331	0.253223242
ESR1	-1.108798258	0.015623583
PALB2	-1.108418739	0.133384725
AGBL1	-1.106070077	0.028210264
PROM1	-1.105066436	0.14877687
SPATA16	-1.104557634	0.052549249
GPR112	-1.104050807	0.124353806
CYYR1	-1.102482712	0.239142712
NOL6	-1.099359296	0.033213826
TMPRSS11F	-1.096286202	0.304416464
LIPG	1.096229104	0.239142712
WNK3	-1.094120427	0.09462456
GALNTL2	-1.091559764	0.192812351
DDX60L	-1.089592971	0.09462456
SEPT14	1.089244927	0.290449952
PSMD9	-1.088121583	0.102127185
SEMA3D	-1.086320771	0.237255522
CREBL2	-1.083757216	0.051992817
PIP4K2B	1.08254829	0.183351509
ACOX1	1.082524014	0.130101002
LIPC	-1.082519639	0.168582537
SLC4A7	1.08223917	0.034075459
HEPACAM	-1.076353517	0.204701387
ASTN2	-1.074796678	0.011023218
ELMO1	-1.074600841	0.243745021
ADAMTS12	-1.074474232	0.136201906
BSDC1	-1.074113926	0.105358725
C5	-1.073133369	0.28400717
RNF220	-1.071891318	0.098875419

PARD3B	-1.071618142	0.008890957
SNX7	-1.070708756	0.101350178
CDC40	-1.067528613	0.099004779
ITFG2	-1.064948847	0.13917175
CD109	-1.060773353	0.151960279
FAM179A	1.060417031	0.241164143
ISCU	-1.060321933	0.363908911
EYA2	-1.059664813	0.034075459
MCTP2	-1.059657568	0.144053328
TRPC6	-1.059533386	0.188056439
CCDC150	-1.058820665	0.185078604
PKHD1L1	-1.058647342	0.247848889
WDFY4	-1.058094123	0.09462456
STAT3	-1.056873189	0.163330436
LRMP	1.056057751	0.158472595
DLG1	-1.054553658	0.19460901
LGR5	-1.054183157	0.219130236
COL4A5	-1.052963959	0.033213826
PIWIL2	-1.050140907	0.160408786
ACSM1	-1.048537869	0.166692362
COL6A3	-1.048526392	0.077513519
LRRC36	-1.045659392	0.231010904
PCYT1A	1.045309254	0.266573784
PDIA3	1.044024151	0.153487158
TAF5L	-1.04083526	0.163330436
CHRM2	-1.040226857	0.195709581
U2AF1	1.039248787	0.198774661
DAPK2	-1.0389139	0.163330436
C2orf89	-1.037304674	0.153487158
FAM124A	1.033893873	0.266504191
DIRC1	-1.032685709	0.205672885
PKD1L3	-1.032400863	0.243621155
SYNPO2	-1.031412596	0.160408786
HPD	-1.030345773	0.164659726
NAT1	-1.030227054	0.242611384
CPXM2	-1.026886273	0.161926803
RAB31	-1.026106863	0.099004779
GRIK1	-1.021554206	0.102127185
PTPRC	-1.019166337	0.368412981
MRP63	1.018878977	0.112099077
RRAS2	1.018715793	0.168582537
COL23A1	-1.018581064	0.083444705
CDH1	-1.018543582	0.173785205
ANP32B	-1.017084066	0.161926803

PLCG2	-1.016654562	0.19456621
WDR76	-1.016458219	0.160408786
TGM3	-1.01571769	0.253223242
C12orf53	1.014719097	0.345480514
ANKRD55	-1.013995823	0.243625064
PTPRA	-1.012736026	0.559945147
C16orf46	-1.012609238	0.329110194
C21orf63	-1.012514278	0.37995426
CYTH1	-1.009337891	0.169372325
SLC16A10	-1.009018623	0.158472595
PEX7	-1.002339902	0.175670719
TAF1	-1.000774013	0.130101002
PARP11	-0.999895221	0.130101002
DCBLD2	-0.998988405	0.299288348
ZFY	-0.995693395	0.509152459
PHC3	-0.995498485	0.067037845
SLC2A5	-0.995256511	0.369044204
PGM5	-0.994294427	0.178987535
SRCAP	-0.992037486	0.265618798
SLC9A7	-0.986318908	0.108581532
CADPS2	-0.982802696	0.140433334
ACSBG1	-0.981325844	0.284003402
TMCC3	-0.980439767	0.171863393
C1orf14	-0.9800869	0.255310391
PLCE1	-0.97804927	0.105358725
VAV1	-0.974847135	0.185078604
TFPI	-0.97431834	0.253223242
LOC253724	-0.973707306	0.244519357
MAP3K3	-0.971935708	0.166692362
ZNF789	-0.97191531	0.259787989
CCDC108	-0.970326867	0.119402467
KIAA0556	-0.967828696	0.087223195
MTUS1	-0.96651817	0.124720822
LACE1	-0.96397685	0.035790803
OTOF	-0.963882158	0.319439283
ADAM3A	-0.960569969	0.43934568
IL12RB2	-0.960227427	0.348531549
CCT3	-0.959173707	0.237706651
PBLD	0.95884979	0.245918521
CCDC109B	-0.958100944	0.298071322
IBTK	-0.957893553	0.053296023
DCHS2	-0.957381109	0.285768529
VAV2	-0.957367014	0.160408786
ATP13A4	-0.957083062	0.09462456

FOXP2	-0.956386952	0.191195696
PLAUR	-0.952590425	0.309412584
S100Z	-0.951842111	0.341682449
MYO3A	-0.951462823	0.149377843
DUSP22	-0.95077194	0.350595048
STK33	-0.950395898	0.195709581
CLNK	-0.950021923	0.193547616
PPP2CA	0.949672817	0.134130715
PIK3R5	-0.949322419	0.243625064
UQCR10	-0.947569954	0.333448726
KIF14	-0.946102298	0.350380986
TGM6	-0.945913952	0.250770867
VWC2L	-0.944514124	0.24529567
DNAH17	-0.943845457	0.243625064
ZNF121	-0.943630223	0.348684521
KIF21A	0.943492851	0.081099509
MECOM	-0.943162588	0.139259294
TSHR	0.941326725	0.101088478
PHF16	-0.940096675	0.223137944
CD226	-0.936737742	0.288361953
RAB37	-0.936203428	0.219130236
TTC29	-0.935862221	0.188977472
LRRC1	-0.93563622	0.264769956
TTYH3	-0.93546629	0.263288622
LOC283922	-0.935160113	0.294287912
LOC100131060	-0.934030751	0.169372325
DTX1	0.932788267	0.307868231
CYP2C18	0.931925256	0.593535977
DENR	0.929392051	0.350380986
KCNAB2	0.927518928	0.299288348
CXorf30	-0.927279978	0.245115264
TRPM6	-0.926878909	0.249168382
ADCY6	-0.926825892	0.166692362
MITF	-0.92537714	0.250950226
EBF1	-0.923906794	0.163330436
GUCY2F	-0.920527562	0.335999983
CDH7	-0.920163474	0.273246191
SEPT10	-0.917757216	0.168128428
SLC4A1AP	-0.914953486	0.307891201
CPM	-0.913155581	0.227977507
FREM2	-0.911581268	0.144053328
DHX30	-0.910551269	0.102308035
UEVLD	-0.910448329	0.151960279
SHROOM3	-0.910409815	0.214533966

GULP1	-0.908502444	0.088216938
CXADR	0.906839517	0.158472595
ISM1	-0.906649855	0.297829108
OGFOD1	-0.906552399	0.349860554
UBE2V1	-0.905244782	0.057184874
GBA3	-0.904562377	0.366602325
RGS3	-0.904004065	0.264769956
PIP4K2A	-0.903722654	0.192812351
C1GALT1	-0.903590263	0.371665587
PRR5-ARHGAP8	-0.902465039	0.195709581
NWD1	-0.900210524	0.234199572
TNFRSF8	-0.899799802	0.326576342
NLRC5	-0.898851198	0.268153249
DZIP1L	-0.897359394	0.34732248
FLJ34690	-0.896923813	0.294287912
AK4	0.89603853	0.151960279
ADAMTSL1	-0.896007528	0.109162385
GPRIN3	-0.895811949	0.284003402
PARP4	-0.895788235	0.356997335
KLHDC4	0.894246888	0.266573784
MAMDC2	-0.892285757	0.375522733
MYO10	-0.890635316	0.138913603
TTC5	-0.888969927	0.267157224
C6orf182	-0.888618342	0.17139106
COX7B2	-0.888425886	0.206450762
LOC727677	0.886791476	0.395372162
NUDCD3	0.885139133	0.272242056
GK5	-0.885119215	0.309412584
POF1B	-0.883835235	0.398464539
DNAJC11	-0.883831654	0.239309174
ANO2	-0.883492841	0.089429822
ALPK3	-0.881684578	0.43934568
FAM3C	-0.88147056	0.383138234
C12orf40	-0.880467383	0.435996866
JAK1	0.880067187	0.214533966
TMEM87B	0.877254641	0.243625064
TMEM120B	-0.87623482	0.163330436
GPC5	-0.876141835	0.012220128
ACTN2	0.874297955	0.356997335
SLC26A8	-0.873673876	0.309412584
CLCN5	-0.872133714	0.237255522
TPRG1	0.871255978	0.276426902
COL8A1	-0.871127064	0.250950226
SH3BP4	-0.870630964	0.371665587

SR140	-0.869705108	0.294287912
CPT1A	-0.869272429	0.272242056
WASL	-0.868475222	0.270142583
BAIAP2L1	-0.868075894	0.356973669
DLG5	-0.866867115	0.081099509
TEP1	-0.866824547	0.395670999
PHF21B	-0.866599651	0.192812351
FAM171B	0.864426444	0.10472139
SLC30A8	-0.862959449	0.264769956
SYTL3	-0.861540805	0.371665587
DNAI1	-0.861350933	0.25269811
WDR88	-0.861062602	0.302586935
GPC3	-0.859467908	0.177362127
TMEM132C	-0.859410464	0.083570874
BRCA1	-0.857881584	0.345480514
FLJ35024	0.857578541	0.304416464
ARHGEF18	-0.855511665	0.251706967
ODZ1	-0.854655425	0.09462456
MGC72080	0.852003489	0.139259294
ATP8B5P	-0.850499112	0.459252469
FLT3	0.849959839	0.229789855
ARGLU1	0.848306028	0.341356245
PEX14	-0.847925534	0.192812351
FANCA	-0.847498764	0.386606394
ELOVL5	0.846999043	0.430899415
IGF1R	0.846869023	0.068658043
MAMLD1	0.846064956	0.168182595
SLC15A5	-0.845723656	0.444343319
DNAH2	-0.84153892	0.225782846
PDE1C	-0.839622647	0.103052118
STK36	-0.839057408	0.264769956
TUBD1	-0.8380962	0.430899415
WIPF3	0.837969685	0.383450242
ADAMTS17	-0.83762343	0.160408786
C4orf22	-0.83758709	0.288361953
DNAH11	-0.83708152	0.340097909
FRMD3	-0.836875534	0.1046717
OGT	0.836595553	0.402374975
GREM2	-0.835935918	0.356973669
KDM5C	-0.835498731	0.2716723
SNCB	0.833929761	0.435996866
WDR47	0.832969676	0.13917175
ACBD7	-0.832615842	0.288555746
BACE2	-0.83155908	0.356973669

FYB	-0.831172053	0.386606394
LRRK1	-0.83116431	0.195801937
ARHGEF7	0.830934835	0.153487158
SEC23B	-0.829516477	0.28400717
OPTN	0.829178542	0.130618419
ZNF804B	-0.829000533	0.1046717
DICER1	-0.8285135	0.244519357
TBC1D22B	0.827648394	0.274094725
ATP10D	-0.826861067	0.384886008
REPS2	-0.826654391	0.316626181
NUB1	-0.825661654	0.381241828
RBM47	-0.825010063	0.302017779
POU2F2	-0.824816876	0.265618798
PPFIBP2	-0.823924322	0.322860116
STAB2	-0.822688056	0.277637893
CELSR1	-0.821774739	0.348531549
ANKFN1	0.821568328	0.037259841
C5orf56	-0.820636112	0.467755425
LOC255130	-0.820530783	0.384886008
PVT1	-0.81988688	0.247494831
EAF2	-0.819132468	0.409846838
USP8	-0.818888572	0.300016693
PXK	-0.816608425	0.300016693
CPPED1	-0.815952311	0.171863393
PTPN21	-0.811210866	0.264769956
VAMP2	0.811014009	0.174317633
IFNAR2	0.810874846	0.23945739
BIN1	0.810382381	0.341356245
SULF1	-0.80972678	0.446113784
DNAJC13	0.808873646	0.356973669
EPC2	-0.807879129	0.276150241
ADORA3	-0.804637081	0.50918467
KLHL24	-0.804015603	0.39574394
CORO1C	0.803264695	0.07686106
CSNK2A2	-0.801409719	0.314160832
NTNG2	0.801207948	0.494444669
OXSR1	-0.801070813	0.181925829
ST3GAL3	-0.801050969	0.137631631
AFAP1L1	-0.80093976	0.384886008
C3orf20	-0.80052323	0.245115264
ANK1	0.799134564	0.429930115
ZNF542	0.797612327	0.173785205
RNF17	-0.797497491	0.330630061
TMC5	-0.796491907	0.375964329

CLPX	0.795956127	0.483953637
FHAD1	-0.795709343	0.403714321
SRBD1	-0.79424154	0.243621155
ETS2	0.794182488	0.243621155
ZNF567	-0.794047809	0.348684521
ZBTB1	0.793943346	0.24529567
SVIL	-0.79345077	0.335999983
CSGALNACT2	0.792040794	0.403714321
SORCS3	-0.791412856	0.050480678
KCNQ2	0.79107299	0.181925829
DPP3	-0.7908453	0.446113784
VRK2	-0.790666938	0.40246609
ZNF382	-0.790470029	0.337784179
C3orf21	-0.790211095	0.239142712
ALG8	-0.79001547	0.348792852
ERG	-0.789647109	0.330630061
ADIPOR2	0.789213658	0.173785205
POLA2	-0.788847064	0.314160832
PHF8	0.788837718	0.428534595
RCAN2	0.787206054	0.433248449
TET3	-0.78718415	0.108581532
KIAA1324	0.787131411	0.087356048
LOC100499467	0.786778709	0.241902031
APOOL	-0.786480639	0.363908911
METAP1	-0.785552413	0.363908911
ARHGAP31	-0.785089516	0.337963208
SRFBP1	0.78419647	0.320082452
CCDC126	0.782139512	0.375522733
CWF19L2	-0.782116981	0.09462456
LAMB4	0.781423105	0.44397465
TUB	-0.780659475	0.338202837
RUFY4	-0.780652463	0.417841248
SLC9A10	-0.780486282	0.326576342
DOCK1	-0.78022341	0.119402467
STX18	-0.780145977	0.331612333
LOC100129827	-0.77985792	0.429930115
TEAD1	-0.778477708	0.299717249
MIR548A2	-0.777262115	0.340097909
ATP6V0A4	-0.776582527	0.41244051
OBSCN	-0.776260043	0.395670999
KIF17	-0.776075629	0.435718037
HGSNAT	0.775857085	0.335999983
VIT	-0.775586557	0.479031065
ZNF430	-0.774873869	0.181098733

STAM2	-0.773995451	0.244519357
HDHD1	-0.773544055	0.278998265
TOP2B	-0.772510312	0.284003402
GRXCR1	-0.771894424	0.42778619
AMOTL1	-0.770975018	0.448455068
UNK	-0.769702593	0.50860492
CXorf59	-0.768899608	0.375522733
GLIS3	-0.768697473	0.144053328
NIPAL3	-0.767840635	0.428534595
LOC286467	-0.767031971	0.331612333
LOC646982	-0.766679207	0.239142712
ZNF675	-0.766442329	0.366602325
SPPL2A	-0.766363313	0.300016693
SDCBP	0.765244966	0.307891201
CGREF1	-0.764546606	0.266504191
SLC39A8	-0.764153833	0.363908911
CNGB3	0.762939076	0.437848419
ALPK2	-0.762906137	0.451189276
SNRK	0.762312376	0.446113784
C18orf19	0.761964962	0.340097909
N6AMT2	-0.760604942	0.356973669
HIP1	-0.760224787	0.22809449
TXNRD3IT1	-0.759955541	0.492601357
ORC3L	-0.759593914	0.295469797
C1orf129	-0.758470137	0.45562834
EGFLAM	-0.757502106	0.495804928
DCLRE1C	0.757108203	0.435996866
RNF217	0.756049112	0.335762021
LRCH3	0.755744419	0.173785205
PART1	-0.755363428	0.417841248
HSF2BP	0.754196658	0.451189276
PLEKHB2	-0.754128501	0.336099207
OSBPL10	-0.754031735	0.246767055
MYO1B	-0.753838908	0.414125861
GRIP1	-0.753726648	0.276150241
ANKRD33B	0.753654526	0.335999983
SLC8A3	-0.753297459	0.315922319
B3GNTL1	-0.752749917	0.335999983
RASGEF1A	0.752209951	0.464089759
C5orf13	-0.752185642	0.276150241
RBMS1	-0.752110588	0.307868231
POLQ	-0.751000469	0.433385889
DNAJC27	-0.750156943	0.417841248
PLD5	-0.750114619	0.363908911

MRPL10	0.749929114	0.517676567
FAM38B	-0.749892883	0.243625064
TXNDC12	0.749548868	0.383450242
RCHY1	-0.749147775	0.341356245
GREB1	-0.749051576	0.347687476
C9orf171	-0.748508805	0.415157407
CD96	-0.74786299	0.525946505
VPS39	0.747820415	0.307868231
C6orf167	-0.746524545	0.423872868
TMEM38A	0.744344736	0.312402895
CMTM7	-0.744157684	0.600792289
PEPD	-0.743081005	0.419826415
DNAH3	-0.742863068	0.266504191
COL4A3	-0.742412511	0.435996866
KATNAL2	-0.742062293	0.285090015
IGF2BP3	-0.741646624	0.595167881
SLCO4C1	0.741582102	0.433385889
LOC100133091	0.741555578	0.459479664
CDH11	0.741016007	0.133384725
LONRF2	0.740940685	0.239142712
DENND2C	0.739229568	0.203821713
DDX24	0.739194823	0.335999983
LOC440704	-0.737144374	0.433385889
SCN10A	0.736622903	0.50918467
SENP1	0.73625709	0.448789806
ITGBL1	-0.73620161	0.264769956
C22orf9	-0.736116322	0.243621155
C16orf89	-0.7350219	0.511604318
NICN1	-0.734494056	0.463321304
SNX6	-0.733088732	0.337615508
GLT25D2	-0.731967555	0.309610645
SYCP1	-0.731684476	0.391867176
ATPBD4	-0.731142289	0.130618419
TULP4	-0.730757652	0.32281299
POLR2B	-0.729944114	0.50860492
C10orf96	-0.729098897	0.53383922
PHF2P1	-0.72821624	0.599110248
SEMA6A	0.728172907	0.464609374
SYT2	-0.727141763	0.600792289
CLMN	-0.726716312	0.337943805
BCAR3	-0.726158507	0.262529337
KIF4A	-0.725277539	0.521388482
DKFZp686O1327	-0.725204027	0.195709581
ATP11B	0.724059382	0.237706651

CISD2	0.723786494	0.349860554
GALNT10	-0.723783494	0.247784615
PCSK6	-0.723394943	0.401882495
MAP3K9	0.722972596	0.372517121
TMEM195	-0.721910708	0.512799421
BFSP2	-0.721370167	0.50860492
SAFB2	0.720550446	0.403714321
EPHA3	-0.720471926	0.319201461
C19orf2	-0.719710964	0.264769956
ZMPSTE24	0.719646712	0.355860775
ELF1	0.719006116	0.391000959
XDH	-0.718759872	0.436186209
SCN9A	-0.718351956	0.409846838
TOX3	-0.718277972	0.468212231
SEMA3E	-0.717872626	0.41244051
IGBP1	0.717711385	0.348750784
PLEKHH2	-0.717158229	0.436186209
CECR2	-0.717106562	0.442091379
SPAG1	-0.716947413	0.433385889
SFI1	-0.716746463	0.514727564
RAB40B	0.715745009	0.499105743
GABPB2	-0.715478765	0.522793419
NCK1	0.714897027	0.335999983
LOC442459	-0.714603065	0.375522733
HPSE2	-0.714355315	0.225782846
C4orf37	-0.712741294	0.108581532
BCO2	-0.71198697	0.410829625
ZNRF3	-0.711968291	0.245918521
MTMR9	0.711938199	0.390204648
ARNTL	-0.710645684	0.460724952
CORIN	-0.710338853	0.320082452
FAM13C	-0.710219515	0.469985602
IL6R	-0.709558137	0.467755425
PCDH11X	-0.709157364	0.46512918
VRK1	-0.708539338	0.562037557
MGC2752	-0.708309013	0.396770891
EIF3K	-0.707422598	0.276150241
SLC9A11	-0.706214135	0.451999404
ACBD3	-0.706167886	0.512799421
TBRG1	-0.7060247	0.14629282
EIF2C4	-0.705970807	0.299288348
FAM160A1	-0.705905011	0.417841248
OPA3	0.705595955	0.356973669
WIPF1	-0.705359976	0.391663758

TMEM231	-0.705181566	0.37995426
KLHL6	-0.705069649	0.530638358
RPH3AL	-0.702534813	0.359476195
POLR3B	-0.702044347	0.329110194
TMCO4	-0.701639779	0.50860492
COX6C	-0.701560835	0.608636352
C6orf186	-0.70118703	0.348684521
GLRA2	-0.700916035	0.435996866
TCF20	-0.700462468	0.328109001
CCM2	-0.699594207	0.395372162
PTGFRN	-0.699402272	0.390726996
FOXRED2	0.697659781	0.536611055
NPRL3	-0.697264893	0.398321169
MED12L	0.696190418	0.034809031
ZNF398	-0.695906309	0.363908911
SEC24D	-0.695570678	0.576588612
FUT8	-0.695403165	0.101350178
WASF2	0.695108064	0.508550311
TNFAIP8L1	0.694635121	0.456195828
ACACB	-0.693597983	0.350595048
CHST15	0.693014995	0.452999692
IFT57	0.692523439	0.350595048
CERK	0.692458403	0.535811253
ECHDC1	-0.69209821	0.419826415
CLIP2	-0.691829935	0.515716969
JHDM1D	0.691588888	0.460724952
OPRM1	-0.69158473	0.372517121
GFRA1	-0.691548225	0.512799421
BRSK1	-0.691256553	0.478109158
ACTR3	0.690907355	0.341356245
ATXN7	-0.690681726	0.375964329
C10orf118	-0.690642515	0.532354044
TRPC7	-0.690255345	0.51817202
PHLPP1	0.690012724	0.130101002
TBL1X	-0.689132672	0.45562834
PTDSS1	0.689113148	0.375005823
C3orf15	-0.689040144	0.251706967
HS3ST4	-0.688643955	0.184252663
ARHGAP18	-0.688618871	0.383138234
NXN	-0.688323497	0.34732254
RYR1	-0.687734188	0.429930115
ATP1A3	0.687519837	0.512799421
CELF5	0.685868734	0.130297582
EAF1	0.6848203	0.559684614

ATP6V1G2	0.68424736	0.584383781
BDH1	-0.683399881	0.239357861
PDCD6IP	-0.683380733	0.294287912
TMOD3	0.68328988	0.508524698
LOC100294362	0.682961868	0.512799421
TBCD	0.68283662	0.364993624
SCTR	-0.682102587	0.446571291
MAPRE2	0.681546451	0.13262537
HOMER1	-0.681519999	0.57763513
WDTC1	0.681159092	0.509959641
PLEKHH1	-0.680754732	0.50860492
ARHGAP39	0.680341522	0.435996866
ZNF831	-0.679663144	0.417841248
RHBDD2	0.679475265	0.508524698
UBR4	-0.678863029	0.435718037
ZNF81	0.678856682	0.42778619
NCDN	0.678344177	0.371665587
TESC	0.67736656	0.403714321
ZNF700	-0.677289923	0.50860492
CDH3	-0.676776418	0.539641564
DCLK3	-0.676527487	0.456195828
RGSL1	-0.675851715	0.588606109
ABCB10	-0.675661824	0.479998631
ATP6V0D1	0.675588242	0.496572435
PRKCH	-0.67470163	0.430899415
PSIP1	-0.67418698	0.403714321
NKIRAS1	-0.673503656	0.566165919
ANKMY2	-0.673115708	0.473999157
UTP20	-0.673097076	0.341356245
PREX2	-0.672794345	0.335999983
ZNF487P	-0.672602145	0.508550311
TTC18	-0.671836469	0.441150705
CHD7	-0.671606044	0.50860492
ZNF257	0.671429641	0.508448388
STRAP	-0.670652807	0.592510579
MGLL	0.670288154	0.571286931
ZMYM3	-0.670155417	0.375964329
SORCS1	-0.670091643	0.098438667
RCC2	-0.669441289	0.560175704
B3GALTL	0.668390577	0.479031065
CDS2	0.667500001	0.401386589
STK4	0.667463039	0.294126559
FAM46D	0.667050343	0.611187411
CCDC55	0.666762084	0.243625064

DNAJB6	-0.665517246	0.491571235
PRDX5	0.665368826	0.559684614
MRPL48	-0.665266588	0.326576342
CCDC122	-0.665218503	0.430899415
ORAI2	0.664114666	0.386606394
PPP1R1A	0.663816406	0.514066724
AGAP3	0.663009845	0.512106504
DEPDC5	-0.662383445	0.403714321
WDR67	0.662247665	0.393487965
DYNLRB1	-0.662092585	0.428439729
DNTTIP1	-0.662084742	0.536160221
TTC7A	-0.661697402	0.384886008
MIR548H3	-0.661167704	0.435996866
PPP2R1B	0.661147076	0.50860492
SNX32	-0.660987627	0.488420675
ETFB	0.660740956	0.473999157
SEC16A	0.660218326	0.50860492
C20orf43	-0.658288201	0.579967189
TRUB1	-0.658240394	0.429014972
ANO6	-0.658183746	0.238415374
PGR	-0.657884645	0.595167881
LRRK2	-0.655900944	0.426360691
KLHL2	0.655850274	0.270142583
MIR548H2	-0.655399114	0.374191036
LMAN2L	0.655226758	0.512799421
IQGAP2	-0.655210564	0.576588612
FGFR1	-0.655039716	0.525107715
TXN	-0.654873624	0.467103167
SLC22A4	-0.654745014	0.50860492
IQCH	-0.654109699	0.246767055
ATP2C2	-0.653083664	0.456195828
44896	-0.652832284	0.521388482
POFUT1	-0.65221744	0.449566814
CXCL13	-0.652187489	0.639115826
C10orf107	-0.651843	0.539641564
L3MBTL4	-0.651705604	0.412894529
TEX261	0.651367539	0.539641564
PDPR	0.651209478	0.492713027
MYH7B	-0.651069575	0.50860492
SLC35F2	-0.650567767	0.511623793
TDRD9	-0.650395876	0.492601357
KIAA1755	-0.650058783	0.412894529
NCRNA00189	-0.649611263	0.570004105
SRD5A1	0.649247253	0.536160221

FAM55C	0.648281257	0.430300133
ZFAT	-0.648158839	0.375679392
FGD5	-0.648084949	0.529869829
RNF149	0.64806843	0.264769956
RAD52	-0.648020321	0.508131286
C8orf46	-0.647857451	0.35864584
PARVB	-0.647683827	0.421348333
44628	-0.647668219	0.340243329
ZNF670	0.647591192	0.340917287
GLI2	-0.647245868	0.472949269
ST3GAL6	0.647224535	0.448007375
CABIN1	-0.646854306	0.461265951
SEPT15	0.646724236	0.577228284
BMPER	-0.645048431	0.403714321
NCOA5	0.644220338	0.371821201
ZNF189	0.644084173	0.3990186
SLC16A7	-0.643653707	0.479998631
EFHC1	-0.643372061	0.435996866
COPS2	0.642824166	0.526064882
GFAP	0.642766611	0.307891201
SAMD8	0.642682698	0.24529567
MPP4	-0.642369026	0.386606394
FZD3	0.64165179	0.312991453
ERO1LB	0.641263601	0.237706651
EDAR	-0.640851579	0.552363401
TRHDE	-0.640633137	0.535811253
LRFN1	0.640346936	0.53795655
C21orf7	-0.640246227	0.581893563
DAB2IP	-0.639840638	0.540080392
STK32A	-0.639771372	0.50860492
THOC3	-0.63969734	0.493687399
WDR72	-0.639385483	0.521388482
OGDH	0.638031897	0.356997335
SLC2A13	0.637959692	0.1046717
RNF2	-0.637851811	0.435718037
C18orf25	0.637592936	0.511604318
TEAD4	0.637479087	0.633786928
RAPGEF1	0.637340848	0.248324119
GABPA	0.63678408	0.479512532
NDST3	-0.636650847	0.287872832
CDK15	-0.635851656	0.532354044
AKAP8L	-0.635453841	0.371968703
GPM6B	0.635402023	0.065891574
CPVL	-0.635353145	0.611040722

ZNF521	-0.634792229	0.43934568
OCA2	-0.634175411	0.403714321
CATSPERB	-0.633683559	0.517676567
GAS2	0.633671103	0.491785308
HUNK	-0.633284658	0.394386563
CBFB	0.633221902	0.450039885
GPR39	-0.633088373	0.379974544
RANGAP1	-0.633029171	0.521388482
WASF3	-0.632959865	0.419826415
AGPAT9	0.632654961	0.565788729
MRPS35	-0.631644114	0.438006865
DHX15	0.631497076	0.50860492
BCL2L2	0.631348153	0.559684614
NCRNA00158	0.629651551	0.45797158
CLTCL1	-0.629502878	0.597235146
C12orf42	-0.628617167	0.284003402
PKD2L2	-0.62859608	0.577557311
IQSEC3	0.628384784	0.565788729
EIF2AK3	0.628270147	0.467755425
LAMA3	-0.628253501	0.340097909
UPF3A	0.627159217	0.327245987
NDUFB6	0.626297097	0.496572435
MAN1C1	-0.626294736	0.334342799
LRSAM1	-0.625967889	0.539641564
WARS2	-0.625745904	0.341844103
PRKX	-0.625611742	0.49851134
CDH4	-0.625499617	0.323342654
BCYRN1	-0.625100147	0.24832443
NSUN6	0.624802161	0.381586412
PLDN	0.624704773	0.511604318
SFRS16	-0.624032561	0.566165919
CWF19L1	-0.623950762	0.449843226
PTGER3	-0.623681476	0.472959174
GALNT1	-0.623318061	0.611187411
QSER1	-0.62321453	0.605954637
PKIB	-0.622033516	0.471994398
MGAT5	-0.621697127	0.160408786
ATF7IP	-0.62169256	0.433385889
HADHB	0.621678349	0.454833232
PDE4A	0.621406701	0.371821201
PGBD1	-0.621352683	0.461068312
MRI1	0.621156931	0.532394853
GP6	-0.61992293	0.594042359
CEP78	-0.619565386	0.637603743

RAMP1	0.618952876	0.633023449
GTF3C1	0.618823577	0.408171733
GGNBP2	0.618040113	0.350595048
HS3ST2	-0.617387535	0.643556325
DLEU2	0.617026233	0.45335253
PIK3R6	-0.616773181	0.539641564
LOC100288974	0.615566016	0.582295892
CHRNA7	0.615297752	0.440304949
KANK1	-0.614712688	0.391867176
C5orf41	0.613205464	0.511604318
TMLHE	-0.612945278	0.350595048
SMAD6	-0.61225813	0.666819087
LOC285577	-0.611914606	0.508524698
MYH14	-0.610405499	0.392319964
SIRT3	0.61031752	0.517676567
ZNF490	-0.609683005	0.508524698
USP13	-0.609640834	0.451189276
LOC100288428	-0.60949081	0.48641565
NOL9	0.609193625	0.481536713
SV2C	0.609192462	0.499796502
NOSIP	-0.607984139	0.569191004
CPLX2	0.607712044	0.517676567
ARID4B	0.60762147	0.081099509
WWP2	-0.60717209	0.195709581
ABCA1	-0.607075977	0.558192867
AQR	-0.606546999	0.530638358
HCFC2	0.606290031	0.442091379
RGS17	0.606192483	0.433248449
TXNDC11	0.605979573	0.511604318
PCYOX1	-0.605862682	0.509959641
LOC554202	-0.60561931	0.643556325
PAQR6	-0.605547777	0.609217743
OTX2OS1	0.605296176	0.608845885
SEC24A	-0.604147296	0.613647961
HRH1	0.60403433	0.529869829
CHD8	0.603061897	0.513718132
RNF19A	0.602820733	0.383450242
ULK3	-0.602532767	0.570066613
FAM133A	0.601781257	0.588606109
GLRA3	-0.600985037	0.438006865
C16orf52	0.600762385	0.349860554
RAD51L1	-0.60072653	0.243625064
PNPLA7	0.600469453	0.50860492
MRPL35	0.600178124	0.717882993

TPX2	-0.600080483	0.511604318
TAF1B	0.599288214	0.508550311
MTR	0.598746695	0.348684521
IQUB	0.598475714	0.582295892
CLOCK	-0.598043413	0.130101002
APBB1IP	-0.597825321	0.435996866
TNPO3	-0.597748304	0.429930115
ZNF674	0.597201486	0.595167881
STK32B	-0.59638501	0.435455241
APOL6	-0.596154804	0.611187411
LEKR1	-0.596102702	0.446113784
C1orf159	-0.595936574	0.536611055
C10orf26	0.595679523	0.539641564
OLFM2	-0.595621966	0.376795804
INTS10	0.595499045	0.195849849
FBXO11	0.59525748	0.09462456
FBRSL1	0.594969988	0.621915243
KREMEN1	-0.594873382	0.50860492
RBBP8	-0.594023394	0.521388482
TDP1	0.593924957	0.479512532
ME1	-0.593158765	0.351331293
CPSF6	0.593086421	0.395372162
CLSTN3	0.593078798	0.429930115
CCDC39	-0.592937362	0.532354044
GRAMD4	-0.592673244	0.666819087
CEP70	0.592388757	0.298071322
VPS37B	0.592206257	0.508524698
LANCL3	-0.591688102	0.61314344
ZBTB4	-0.591525305	0.551092689
LOC728081	-0.591504774	0.50860492
MTFR1	-0.591461593	0.571161321
TIAM1	0.591304992	0.133006244
PRSS3	0.590747156	0.577228284
IL16	-0.590739027	0.588606109
PML	0.590471937	0.592510579
MOCOS	-0.590063433	0.565788729
NOS1	-0.590039537	0.469888905
RASSF8	-0.589737767	0.43063445
KLHL8	0.589610988	0.37995426
MMS19	0.589501548	0.472170959
ADAMTS6	-0.589467721	0.497535759
AACSL	-0.58934088	0.600792289
PTPDC1	-0.588769305	0.620866274
C3orf67	-0.588411591	0.508524698

RABGEF1	0.588230183	0.300016693
C12orf4	-0.588121923	0.41244051
PRDM16	-0.58807539	0.50860492
SLCO1A2	-0.587971679	0.600792289
CROCCP3	0.587875437	0.494509101
LOC283104	-0.587868441	0.569191004
TSEN34	-0.587775416	0.559945147
ZNF667	0.587655491	0.356973669
SERPINE2	-0.587402161	0.446113784
B3GNT1	0.586356598	0.539641564
ZC3H6	-0.586212865	0.600792289
CDK11B	-0.586094973	0.559684614
PXDN	-0.584485885	0.683733977
SEC31B	-0.58442564	0.611187411
ANXA6	0.584028068	0.600058617
PRKY	-0.58401604	0.631813887
CBWD2	-0.583667247	0.520251158
PIGB	0.583523874	0.582295892
NARG2	-0.583115676	0.330630061
CDH17	0.583025964	0.597235146
KCNMA1	-0.581990304	0.338087549
SHF	0.581057227	0.473999157
WDR49	-0.580762179	0.53383922
CRY1	-0.58034545	0.435996866
TNC	0.580285589	0.600792289
CELF1	0.580207021	0.327245987
C6orf142	-0.57964279	0.588606109
PLA2R1	0.578234602	0.613647961
MCM3AP	-0.578225342	0.435996866
CBX7	-0.577682082	0.537899363
BZRAP1	0.577645163	0.651521707
SCRN3	-0.577633806	0.669098339
OPRD1	0.576672542	0.511557077
USP46	-0.576388894	0.582295892
PFKFB1	-0.576367231	0.657154529
TIAM2	-0.575906843	0.539641564
ANKRD44	-0.575737256	0.326576342
ZNF710	-0.575638833	0.50860492
ABCG5	-0.575367404	0.580348396
PNMA2	0.575335586	0.579289252
FMN1	-0.574985125	0.464831051
GRAMD3	-0.574205025	0.539641564
TTC39B	-0.574144694	0.460724952
SGPL1	0.573966905	0.519429582

FBXL16	-0.573948556	0.488741847
GTF2IRD1	-0.573888873	0.555359412
MTM1	-0.573482393	0.539641564
NUP214	-0.573245662	0.521507605
SRXN1	-0.573132507	0.615086613
C21orf29	-0.572860644	0.593535977
WDR43	-0.572316535	0.599110248
DLG3	0.572178249	0.639115826
HDLBP	-0.572147545	0.383450242
PRIM2	-0.572054253	0.435455241
WBSCR17	0.571892013	0.095007874
KIAA1430	-0.571861589	0.643556325
CLDN14	-0.57182164	0.618743664
LSM12	0.571642038	0.595488957
METTL4	-0.571172143	0.610014013
NLRP12	-0.571025403	0.529998171
TRAPPC6B	0.570926182	0.435996866
ERMAP	0.570558646	0.599110248
GREB1L	-0.57050968	0.491571235
STAT4	-0.570225878	0.512799421
PMS1	-0.569988006	0.477930639
KCNQ1	-0.569710237	0.50860492
GRHL2	-0.569704103	0.639115826
POLR2J4	0.569580275	0.479512532
UBA52	-0.569575975	0.529869829
UBXN2A	-0.5691139	0.571286931
FBXO10	0.569036215	0.326576342
C7orf60	-0.568478803	0.081099509
PSTPIP2	-0.568410487	0.509570026
TSEN15	-0.568252576	0.69368675
SRCIN1	0.568237711	0.350595048
CNBD1	-0.567565489	0.460912604
EYA4	-0.566911964	0.473999157
RAI14	-0.566849686	0.57763513
BRD4	-0.566780563	0.593535977
ARHGEF9	0.566741042	0.319201461
LNPEP	-0.565658045	0.555359412
HSD17B14	-0.565619921	0.611187411
CYTSA	-0.563761989	0.307891201
NAA25	0.563660317	0.356973669
C1orf228	-0.563530563	0.537899363
CGNL1	-0.563233903	0.529869829
TRIM4	-0.56289272	0.509873249
LRP11	0.562138159	0.512799421

FAM48A	0.562126463	0.582295892
SEMA3A	-0.561320547	0.585512153
ANKS3	-0.56120017	0.611187411
VPS37A	-0.561129487	0.539641564
PFKP	-0.560841764	0.530638358
WDR66	-0.560722265	0.508550311
SERINC5	-0.560710196	0.457657452
TYW1	-0.56026067	0.368412981
PABPC1	-0.5599953	0.508524698
SAR1A	-0.559939116	0.50860492
CDKN2BAS	-0.559674267	0.568614119
BTBD11	-0.558875182	0.508524698
C5orf53	0.558372546	0.648455514
DUSP7	-0.558339212	0.634107681
MYO3B	-0.558108485	0.318882474
CLDN10	-0.557764474	0.680424131
FOXK1	0.557508022	0.461068312
ROR2	-0.557350241	0.595167881
SAMD13	-0.557119227	0.629131561
DHRS7B	0.557028048	0.582221275
LNX2	-0.556387794	0.558192867
RGS5	0.556300376	0.636876515
RASGEF1C	-0.555310935	0.585512153
FAM107B	0.554490686	0.45562834
CLCN7	0.554271648	0.597642741
ZNF488	0.554228642	0.464609374
C14orf43	-0.553779619	0.633786928
LOC728190	0.553062683	0.539641564
RFTN2	-0.552689305	0.399010034
C1orf26	-0.552534068	0.494380159
CAMK1D	0.550733406	0.25884197
KCTD8	-0.55070481	0.472949269
LARS	0.549817855	0.326576342
TPD52L1	0.549665572	0.551092689
MAN1A1	0.549158812	0.539641564
PION	-0.549123499	0.539641564
DHDDS	-0.548941603	0.633430208
PDZRN3	-0.548849656	0.649720491
RECQL	0.548233496	0.621915243
SLCO6A1	-0.548227109	0.568614119
ARNT2	-0.54820311	0.151960279
DNAJC7	-0.548023848	0.539641564
MYLK	-0.547845397	0.541189861
BDNFOS	-0.546631636	0.48641565

TESK2	-0.545500769	0.525107715
H2AFY2	-0.544736526	0.614354266
FANCM	-0.544391272	0.615556592
C4orf19	0.544279766	0.594042359
SLC41A3	-0.544245152	0.576693809
ZMAT3	-0.543756837	0.622277189
CMTM8	0.543420357	0.559945147
MPRIP	0.543360679	0.203413675
AMFR	-0.542960729	0.536611055
TYW1B	0.542818426	0.35864584
TRAF3IP1	0.542706952	0.597235146
TNPO2	0.542619722	0.666819087
HFM1	-0.54244206	0.511604318
BRD7	0.542412335	0.580348396
TCP11L2	0.542145061	0.580348396
MEF2D	0.542120823	0.640452701
CRTAC1	-0.541943357	0.643556325
PLBD1	-0.541886525	0.660727573
CTNNAL1	-0.541530091	0.53383922
LPAR1	-0.541268571	0.603723228
DACH2	-0.541162311	0.402480694
YARS	-0.540714633	0.580348396
PDGFD	-0.540696243	0.453706129
SUSD5	-0.540508019	0.5681702
XYLT1	0.540475283	0.242957142
C2orf65	0.540174502	0.566759859
TPK1	-0.539746656	0.379974544
EFTUD1	-0.539704372	0.542628575
C3orf59	-0.53937462	0.532354044
C14orf118	0.539233097	0.559684614
USP7	-0.539232593	0.580348396
ULK4	-0.538811624	0.195709581
N4BP1	0.538339422	0.403714321
HIST2H2BE	-0.538127936	0.682043169
SLC39A14	-0.538098476	0.639115826
HMGN5	-0.537765178	0.666819087
TOX2	-0.537516031	0.600792289
ZMYM2	-0.537361029	0.299288348
OS9	0.537128305	0.493941142
WWTR1	-0.53701605	0.635491986
SIKE1	0.536609873	0.535811253
WDR64	-0.536604845	0.655328557
PKHD1	-0.53592458	0.511623793
NEDD9	-0.53584675	0.639115826

PRKD1	-0.53529953	0.512799421
PSMD7	-0.53523553	0.40196466
EFCAB7	0.535104617	0.576312533
ADARB1	0.534872913	0.348684521
OCIAD1	0.53423872	0.639115826
UXS1	0.534213991	0.535811253
DPYSL5	0.533865288	0.375522733
GYS1	-0.533634312	0.641383809
PAQR5	-0.533168618	0.652051317
TMEM189-UBE2V1	-0.532961428	0.249168382
KIAA0391	-0.532870407	0.493287999
PER2	0.532513436	0.652051317
BICD2	0.532450262	0.635924334
SOCS5	0.532264644	0.610014013
ANXA7	0.532013433	0.50860492
PNPT1	-0.531961256	0.294287912
NPSR1	-0.531408446	0.600792289
MRPL19	-0.531334445	0.572735725
AVEN	-0.531215503	0.530638358
RALY	0.530260991	0.612612554
ANKAR	-0.530186174	0.602582378
NGFRAP1	0.530052294	0.638196124
TMCO3	-0.529068462	0.61349846
RSPO2	-0.528776273	0.417415935
ZBTB16	0.528559479	0.175678784
TMEM182	-0.528552219	0.711866262
SETX	-0.527427233	0.403714321
ACOT11	-0.526990842	0.582221275
RPL30	0.526850014	0.717882993
SDK2	-0.526745082	0.422365528
ZNF595	-0.526398651	0.563941874
HMCN1	-0.526091052	0.565007074
LOC400794	-0.52604666	0.64968178
KIAA1704	0.525838655	0.512799421
ST20	-0.525799026	0.521388482
C3orf26	-0.525713687	0.239142712
AHRR	0.525592335	0.599110248
MAST3	0.525344708	0.481734178
MERTK	-0.525304198	0.620285381
OTUD7B	-0.525058635	0.599110248
UGT1A10	0.525050564	0.725386766
USP54	-0.524888997	0.600792289
MTA1	-0.524880376	0.605684484
CLPB	-0.52440697	0.625735535

SRRM2	0.523732286	0.643556325
KPNA4	-0.523701167	0.559684614
TEX10	0.523604134	0.475052728
NDN	0.523588484	0.595167881
RFFL	-0.523378798	0.63260171
ABHD8	-0.522681907	0.642905679
C7orf58	-0.522598281	0.532354044
RALGAPB	-0.522585541	0.45562834
SMEK1	-0.521810386	0.299288348
SYNPO	0.521765539	0.488420675
ATF7	-0.521707523	0.550550361
C2orf56	-0.521234268	0.398464539
MAP3K7	0.520847251	0.559684614
CYSLTR1	-0.520794963	0.735277785
CEP170	0.52034001	0.098438667
HIF1A	0.520314367	0.461833249
ACVR2A	-0.520051229	0.429930115
ZNF623	-0.519822765	0.721895164
SF3B1	0.519395067	0.683203224
RIMBP2	0.519289851	0.375005823
NID1	-0.519111406	0.611187411
RLF	0.518974551	0.508550311
RFTN1	-0.51877971	0.649787771
EPT1	0.518603289	0.525946505
C7orf28B	0.518411267	0.615235429
H6PD	0.51834714	0.630813404
COQ7	-0.518325181	0.57741485
TPTE2P1	0.518277291	0.444862452
INPP5A	-0.518139639	0.612218231
ERCC3	0.517518637	0.639115826
ABAT	-0.517517022	0.5558713
ZBTB43	-0.517235315	0.633023449
GALM	-0.516820693	0.539641564
XPNPEP3	-0.51671228	0.429930115
OSCP1	-0.516472888	0.666819087
EFCAB6	-0.51642293	0.48829274
LOC100129858	0.516360255	0.666819087
PPM1L	-0.516216406	0.511604318
CDS1	-0.516047998	0.689522486
RTTN	-0.515986043	0.454833232
AGPAT3	0.515887199	0.537899363
FAM108B1	-0.515417497	0.535811253
VPS13B	-0.514834424	0.243621155
MTSS1	-0.514746593	0.563078394

ABHD5	-0.514672955	0.5681702
HDGFRP3	-0.514579705	0.550441897
CR1	-0.514220917	0.756242501
PGBD5	-0.514201071	0.582295892
CAMK2B	0.513918005	0.422922438
WSCD2	-0.51368654	0.600792289
LUZP6	0.51316251	0.511604318
MTPN	0.51316251	0.511604318
KIAA0802	-0.513006351	0.685236772
CEP250	-0.512633359	0.582295892
C10orf68	-0.512281321	0.348602118
SH3YL1	-0.512259378	0.582295892
NLRP5	-0.512195316	0.639115826
CLTC	0.512148567	0.443768798
SMAP2	0.511948507	0.662199935
CYP4X1	-0.511860268	0.633786928
WDHD1	0.51098561	0.539641564
CNOT10	-0.51011076	0.517676567
EXT1	0.509923325	0.494509101
RAI1	-0.509858044	0.526064882
LRRC69	-0.509829443	0.670190583
IL1RAP	0.509497824	0.523439722
ANTXR2	-0.50939741	0.639115826
C12orf63	-0.508908107	0.559945147
ATP11A	-0.508768584	0.363908911
ELOVL7	-0.508537219	0.673729527
DUSP11	0.508499645	0.639248356
ST3GAL5	0.508009327	0.517676567
DDHD1	0.507510487	0.429930115
SOX5	-0.507342165	0.459252469
ZNF451	0.506884834	0.472864521
SYNE2	-0.506876738	0.593535977
TSC22D2	0.506059041	0.683203224
IFT52	-0.506034114	0.450591904
KDM6A	-0.505760425	0.425673123
CSGALNACT1	-0.505709726	0.50860492
ANTXR1	-0.505451756	0.638512323
FRS2	0.505213652	0.348684521
NRP1	0.504983402	0.266573784
GPR83	0.504870781	0.666819087
KCNE1	-0.504745472	0.571286931
DTWD2	0.504598486	0.529869829
FRMD6	-0.504596922	0.62338929
44626	0.504481848	0.44512781

MRPL34	-0.504332114	0.529426706
NTN4	0.504076366	0.677194031
WDR27	-0.504005922	0.485004597
FGGY	-0.503648772	0.395372162
TNFRSF10D	-0.503265169	0.680104272
KIAA1377	-0.502934799	0.417841248
NAALADL2	-0.502642771	0.284003402
C1orf9	-0.502593231	0.611187411
ADAMTS20	-0.502519573	0.612612554
C1orf101	-0.50247863	0.536160221
ST8SIA5	-0.502130212	0.666819087
ZNF827	-0.502030231	0.601384816
USP10	-0.501978456	0.639115826
F13A1	-0.501962742	0.69368675
OXNAD1	-0.501903835	0.582221275
TM7SF3	0.50179218	0.53383922
COMMD7	-0.501684423	0.536611055
UGT1A8	0.501341084	0.725443018
NAT8L	0.501194758	0.618969295
LOC100499177	0.501140048	0.576588612
RNLS	-0.500996915	0.453044673
TNFAIP8L3	0.500936217	0.635824017
ATAD2B	0.499832575	0.356997335
ZNF141	0.49951069	0.559684614
CDKN2AIPNL	-0.499041785	0.683430523
RYR3	-0.4988194	0.551866235
BAZ1A	-0.498643655	0.649787771
POU6F2	-0.498432024	0.640448818
ANKRD50	0.4983799	0.655328557
CBR4	0.498340407	0.479998631
KIAA1244	0.498022419	0.241902031
PDE8A	-0.497747923	0.488420675
ADAM9	0.497684308	0.539641564
KIF3B	0.497515736	0.446571291
ZNF431	-0.497374008	0.680104272
PPP1R7	-0.497291143	0.550441897
SPIN3	-0.496904824	0.48641565
FAM154A	-0.496828033	0.725386766
CDK7	-0.496685386	0.595167881
FAM118B	-0.496661628	0.588606109
C7orf63	0.496638028	0.45562834
CLRN1OS	0.496011784	0.664073362
SLC2A3	0.495934583	0.567004735
ARHGAP24	-0.495118648	0.521388482

IDI1	0.495025943	0.655328557
PDE9A	-0.49496793	0.649402504
PIK3C2G	-0.494680807	0.550441897
SLC39A12	-0.494409119	0.637603743
LOC100101938	-0.494082528	0.67075072
SASS6	0.493809224	0.600792289
NSD1	-0.493339917	0.472994938
FBXW7	0.492623511	0.466408568
PELI3	0.492502972	0.699086709
IFLTD1	-0.492387671	0.585512153
FGD1	0.492330109	0.636876515
LOC100130691	-0.492251901	0.539641564
CLASP2	0.492101648	0.104874896
OSBPL3	-0.491923334	0.666819087
PCDHGC3	0.491718478	0.532354044
PTCHD2	0.491461641	0.711560629
KCNN1	-0.491415917	0.588606109
SYCP2L	-0.49126032	0.649720491
VEPH1	-0.491235566	0.580348396
CCDC64	0.491148778	0.409846838
SUFU	-0.490507985	0.539641564
SIN3A	-0.490120315	0.595167881
UBASH3B	-0.490119606	0.600792289
COX10	0.48986783	0.639115826
PKNOX1	0.489863295	0.595167881
GPR137B	0.489068081	0.615235429
CPSF3	-0.488668709	0.705991371
VPS26A	-0.488360329	0.601957315
SNPH	0.488195424	0.467755425
FGFR1OP	0.487827813	0.615216387
COL22A1	-0.486466942	0.680830901
MLL3	0.486209615	0.266300685
NUMBL	0.486097702	0.571161321
ZNF438	-0.485949657	0.467103167
SPAG16	-0.48587609	0.133384725
TTN	-0.485875377	0.633118116
ZNF782	0.485247966	0.576312533
C1orf96	0.484860507	0.535811253
GABRG2	0.483656487	0.513718132
NOP58	0.483476347	0.580348396
FGFR2	-0.483200949	0.655328557
PAPPA2	-0.482811237	0.43934568
RFX4	-0.482608765	0.611187411
C10orf79	-0.482361093	0.50860492

C2orf76	-0.482353006	0.552363401
FAM19A4	0.482278359	0.682567585
EIF2C1	-0.482237688	0.525107715
TOM1L1	-0.482234203	0.655328557
FAM66C	-0.482048957	0.666819087
BRAP	0.481976337	0.633786928
ATP2B1	0.481514331	0.383450242
LOC440970	-0.481471347	0.642905679
PARD3	0.481440722	0.131092975
HELZ	0.481369315	0.253223242
FHL2	0.480764023	0.359476195
TDRD5	-0.480589445	0.666819087
NKD1	-0.480458552	0.640961918
FAM168B	-0.480412114	0.611187411
PTPRB	0.479921504	0.50860492
FRAS1	0.479319631	0.64968178
LOC285692	-0.47928842	0.511604318
RIPK1	-0.478961833	0.539641564
RTN4	0.478891465	0.479031065
SLC17A5	-0.478781129	0.45562834
FAM116A	-0.478591476	0.666819087
BTBD2	-0.478569078	0.718566538
REEP1	0.478466694	0.285090015
KNDC1	-0.478254177	0.50860492
ANKRD36	-0.478073397	0.665635253
PPM1G	-0.477975053	0.635824017
ZNF333	0.477788034	0.540005641
DNHD1	0.477451215	0.665635253
NUP210L	-0.477040185	0.599110248
STX7	-0.477017674	0.668730986
MLL	0.476865811	0.590036196
TJP2	0.47653575	0.666819087
DOCK2	-0.476460428	0.64968178
MYOF	-0.476441082	0.67075072
PPP6C	0.476357656	0.639115826
NPHP4	0.475976213	0.444253645
MPP3	0.475504076	0.706359785
EPM2A	-0.47544136	0.644198752
PUS7	-0.475439629	0.706359785
PIGN	-0.475242465	0.550550361
ZNF83	-0.475010725	0.595443131
PCCB	-0.47485324	0.640961918
C19orf30	0.474842958	0.636876515
EIF5	0.474762551	0.67944934

TP63	0.474598208	0.714658354
NTNG1	-0.473997441	0.611187411
VTI1B	-0.473943464	0.600792289
MGRN1	-0.473729525	0.65338234
SUCLG2	-0.473571156	0.576312533
DDX6	0.473471683	0.623379412
MAP3K4	0.473332877	0.623278824
UBE2F	-0.472953583	0.658015027
MKL1	0.472921594	0.430899415
RAD50	-0.472874237	0.595167881
ATP7A	-0.472410232	0.612612554
C16orf62	-0.47207056	0.517676567
TAF4B	-0.472069764	0.639115826
CCDC41	0.471741929	0.447593796
LMTK2	0.47156046	0.429930115
SNX24	-0.471365738	0.383373558
C17orf51	0.471249064	0.571161321
NOX5	-0.471223077	0.666819087
LMX1A	-0.47121162	0.716952451
TTC23	-0.470913924	0.64968178
SP4	-0.470803756	0.67944934
C10orf72	-0.470146615	0.599110248
ZNF236	-0.469782899	0.560329284
ZFP64	0.469539081	0.600792289
ATP1A2	-0.469043307	0.457081359
ZNF507	0.468989734	0.532354044
NDUFC2	0.468819016	0.677370667
GALNTL6	-0.468558586	0.403714321
ZNF557	-0.46843998	0.639115826
TFCP2L1	-0.46814368	0.64864961
ZC3H3	-0.467881831	0.682043169
KDM4B	-0.467723038	0.521507605
SLC17A7	-0.467510776	0.65079011
ATP2B4	-0.467346102	0.639115826
KIAA1328	-0.467117749	0.326576342
NRIP3	0.467022454	0.639115826
HIF1AN	-0.466763118	0.571161321
FER1L6	-0.466739228	0.679447543
PPFIA4	-0.466269292	0.434111239
SHANK1	0.466261747	0.709335464
LOC729991-MEF2B	0.466151804	0.711866262
CAMK1	0.465825232	0.666819087
APAF1	-0.465678341	0.633430208
IKBKAP	0.465505405	0.675837558

SETDB1	0.465320899	0.665182235
AASDH	0.464906294	0.512799421
ENO2	0.464901404	0.717882993
FADS1	0.464758995	0.665635253
ATP10B	-0.464746812	0.596256045
COL5A2	0.464624592	0.635491986
MAPKAP1	0.464548411	0.274589166
LRRC8B	0.464488923	0.375964329
KLHDC8B	0.464386103	0.707787282
PAX3	-0.464098333	0.642056827
C9orf129	0.4640745	0.675470782
FOXK2	0.463689566	0.55453851
QRICH1	0.463617544	0.530638358
CASC1	-0.463377734	0.633786928
USP37	-0.462984437	0.391663758
TARBP1	0.462667235	0.356997335
MLLT4	0.46182187	0.472959174
UBE2Q2	0.461727688	0.637603743
ZCRB1	0.461492807	0.721760094
CCDC18	-0.461379707	0.649720491
GXYLT2	0.461334211	0.714160838
AJAP1	0.461264236	0.639115826
MAP2K1	0.461249903	0.508524698
FIG4	-0.461238582	0.653338331
MDM4	0.460987965	0.639115826
GABRA4	0.460751592	0.550550361
SNTB1	-0.460677474	0.539641564
SGEF	-0.460617062	0.633023449
ATG4A	-0.460581886	0.617034121
SLC41A2	0.460477938	0.396770891
ZNF428	0.460057694	0.638395789
GOLM1	0.459951735	0.64968178
LOC440839	-0.459690284	0.582221275
DNAH7	-0.459611824	0.243625064
FNBP1	0.459210358	0.592510579
IGF2R	0.458979192	0.530638358
MBTPS2	-0.458842512	0.655328557
INO80D	0.458840694	0.173785205
KCNIP3	-0.458794586	0.638538112
NEDD4	0.458579333	0.609217743
ZNF776	-0.458257427	0.623379412
MLF1	0.457950632	0.460724952
PFAS	0.457948802	0.682043169
MEAF6	-0.457920064	0.716822802

SH3PXD2B	-0.457839071	0.60957321
RPIA	-0.457738249	0.63745903
CHSY1	0.45747486	0.689470334
WDR26	0.457471731	0.618969295
ETS1	0.457284409	0.731329685
ATP1B3	0.45668486	0.512799421
ADNP2	-0.456590478	0.665182235
NELL1	-0.456404842	0.707894128
POLA1	-0.456339098	0.566165919
NLRP1	-0.456163126	0.712588824
LUZP2	-0.45591723	0.535811253
SCUBE2	-0.455910794	0.666819087
SLC26A4	0.455809532	0.707894128
CTAGE5	-0.455136101	0.666819087
GTF2A1L	-0.455048641	0.67944934
STK24	-0.454751827	0.665635253
WWC2	-0.454658586	0.48641565
YWHAQ	0.454543924	0.576588612
ABCA3	0.45453621	0.529869829
EARS2	-0.454516296	0.658496908
PDE2A	0.454342325	0.682043169
MMD2	-0.454076526	0.600792289
PPCS	0.453668706	0.705991371
SYNGR1	0.453589366	0.539641564
RSPH3	0.453529419	0.371665587
ENTPD1	-0.453461729	0.689522486
SEPT11	0.452917657	0.597235146
POP4	0.452268403	0.674527144
LYN	-0.452133876	0.639115826
DNMT3A	0.45192851	0.649787771
SNTG2	-0.451530326	0.542628575
BAT2L2	-0.451479094	0.386606394
ACVR1	0.451408787	0.467755425
KCNA6	-0.451374239	0.64968178
EFR3B	-0.451304731	0.580348396
ZFP3	0.451142819	0.738386885
BMP6	0.451109938	0.717882993
FLJ35220	-0.450793166	0.56249295
WAC	0.450744079	0.427995273
ZNF395	0.450400285	0.595167881
ZFC3H1	0.450352445	0.635824017
HNMT	-0.450106891	0.714160838
CALCRL	0.449731295	0.699086709
CPNE1	-0.449698114	0.649720491

CEP97	-0.449590474	0.643556325
DARS	-0.449532866	0.535811253
ARHGAP15	0.448956787	0.532354044
LOC400655	-0.448917021	0.682567585
CCDC88C	-0.448873806	0.652233223
SOCS7	-0.448855604	0.665635253
C11orf46	0.44819527	0.718505047
ILKAP	0.447696605	0.617034121
OVOL2	0.447586355	0.571286931
GABRA5	0.447072968	0.623379412
PCDHA12	-0.447062614	0.530638358
ADD1	0.446839485	0.559684614
ZNRF2	-0.446619658	0.649402504
PALLD	-0.44659712	0.618969295
FAM134A	-0.446377513	0.689522486
FLJ39534	-0.446327172	0.559684614
SETD5	0.446238068	0.652051317
YWHAG	0.446029542	0.774420007
PCDHA11	-0.445772477	0.532354044
EDEM2	-0.445419027	0.508524698
RPL31	-0.445392112	0.702225266
COTL1	-0.444930817	0.682043169
CCDC159	0.444914713	0.711756165
TTC28AS	0.444730445	0.582221275
SIL1	-0.444538454	0.304557889
RUNX2	0.444295837	0.699664059
AP2A1	-0.444130065	0.572735725
ADAM12	-0.44403839	0.685891847
CDH12	-0.443951721	0.274661092
BTBD3	-0.443735008	0.50860492
FARSB	0.443513803	0.639115826
GSTA4	0.443209035	0.641383809
PCDHA13	-0.443192037	0.536160221
ARFGAP3	0.443063837	0.550441897
WBP5	-0.442593765	0.666819087
HEATR7A	-0.442100047	0.685236772
ADAMTS3	-0.442042763	0.680104272
FSIP2	-0.441835992	0.721895164
PDDC1	-0.441668841	0.643556325
C5orf32	-0.441143718	0.738592999
PCDHA10	-0.440923119	0.536887444
FAM126B	0.440795351	0.462355405
AP1S3	-0.440691892	0.571161321
ABCB7	0.440686208	0.600792289

SPIN4	0.440401362	0.720668319
NUP205	-0.440207167	0.639115826
TSNAX	-0.440064589	0.642590833
C9orf93	-0.439954122	0.430899415
RAB28	-0.439516214	0.571275842
PTS	0.43919912	0.709335464
FAM59A	-0.439149171	0.666819087
PCDHA5	-0.439121511	0.520534391
CCDC152	-0.439104146	0.670190583
RAPGEFL1	0.43907759	0.757591062
GLI3	-0.439038728	0.649787771
PIK3C2A	0.438868403	0.639115826
PLA2G12A	0.438726705	0.716952451
PCDHA8	-0.438662481	0.529869829
FAM188B	-0.438627954	0.681814622
SEL1L3	0.43852792	0.689522486
PCDHA6	-0.438240181	0.529869829
KIF1B	0.437957588	0.195709581
PDLIM5	0.43749636	0.64968178
MYST4	-0.437057911	0.327557043
KCNQ1OT1	-0.43687514	0.615556592
PCDHA7	-0.43674959	0.530638358
OPA1	0.436516637	0.581827656
LPPR1	-0.436357114	0.494509101
SMU1	-0.436147562	0.641607861
INTS6	-0.436127063	0.633786928
CUL2	-0.43611213	0.658496908
AGGF1	-0.436001017	0.555359412
FAM114A1	-0.43577915	0.69368675
GPC4	-0.435736432	0.709335464
SMURF1	0.435722575	0.673729527
CCDC112	0.435633195	0.71710259
MRVI1	-0.435489753	0.665182235
SLC44A5	0.435139383	0.081099509
ACVR1C	0.435108741	0.401270843
ACTL8	-0.434970623	0.758928209
TRPC5	-0.434898887	0.570005438
LOC642597	0.434650967	0.680104272
SPON1	0.434529454	0.17139106
ARRB1	0.434272404	0.600058617
SEPT2	-0.434189712	0.529869829
POM121C	0.434009049	0.633023449
PAAF1	0.433725192	0.559684614
PCDHA9	-0.433723123	0.539641564

SLC9A8	-0.433428038	0.568614119
EFCAB2	-0.433223543	0.50860492
HDAC1	-0.433034415	0.742736721
LRGUK	-0.433000996	0.565959286
TATDN1	-0.432804344	0.633023449
CDC42	-0.432645076	0.675837558
VWA3B	-0.432412527	0.669167794
CECR5	-0.43231717	0.666819087
NPC1	-0.432022627	0.725443018
SLC38A11	0.432019113	0.683733977
UBE3B	-0.431981171	0.658496908
SFRS8	0.431747749	0.680104272
CMC1	0.431242266	0.674527144
KIFAP3	0.431136894	0.536611055
SEC24B	-0.431064985	0.588606109
AGTPBP1	0.430736107	0.299288348
NTRK2	-0.430726694	0.53383922
MKL2	0.430613728	0.298023346
C8orf79	0.430565123	0.513718132
ANAPC1	-0.430490196	0.666819087
FASTKD2	0.429981253	0.550550361
MREG	0.429657603	0.512325043
NEFL	0.429618136	0.725386766
ANKRD45	-0.429505768	0.730682805
FAM102B	0.428990089	0.53383922
TIPRL	0.428976541	0.745863114
PCGF5	0.428699154	0.611187411
AATF	-0.428555901	0.666819087
LOC541471	0.428263263	0.745863114
ZNF717	0.427290477	0.73399391
SRSF4	0.426968219	0.50918467
POLR3C	0.426967179	0.64968178
TSPAN9	-0.426904875	0.767223194
APH1B	0.426604729	0.695734391
CBLB	0.426601954	0.559945147
MIAT	0.426381554	0.666819087
DCTPP1	-0.426306716	0.747668378
DPF3	0.426067401	0.539641564
RPRD1A	0.425514448	0.355860775
NEK6	0.425424704	0.716952451
PRKCQ	-0.425212914	0.707894128
CALD1	0.424750146	0.738057966
FAM126A	0.424328327	0.718803868
POLI	0.423994496	0.686523913

NHEJ1	0.423944816	0.666819087
BMS1	0.423833287	0.766681794
ZNF197	-0.423328257	0.665635253
CRNKL1	-0.423287905	0.664508744
IPW	0.423177766	0.655328557
PET112L	-0.422883031	0.721895164
RBM20	0.422731104	0.637603743
KIAA1632	0.422514265	0.675837558
C14orf132	0.422252604	0.649787771
SESN1	-0.422245655	0.576312533
ABCC5	0.422216095	0.639115826
GFRA2	0.42217708	0.709335464
COMMD10	-0.42212696	0.582221275
NEDD4L	0.42208107	0.597235146
ZNF720	0.422067032	0.612612554
ZNF248	0.422015217	0.666819087
CATSPER2	-0.421959225	0.519811939
TCEAL3	-0.421814621	0.686709623
TSPAN18	-0.421720841	0.649141218
PHYHIPL	-0.421715738	0.639115826
LARP1B	-0.421698792	0.48303106
ZNF585B	0.421680974	0.649787771
HHAT	-0.421241074	0.593535977
PKP4	0.42076236	0.307891201
NT5DC3	0.420462914	0.641704826
FAM35A	0.420427562	0.639115826
PRPF18	0.42008163	0.665635253
ENPP3	-0.420026987	0.769497141
DLGAP3	0.419890809	0.765793784
FLJ33065	-0.419737299	0.665182235
PVR	0.419559823	0.680830901
SLC24A4	0.419440984	0.686523913
NCBP1	0.419429014	0.770855417
FBXO45	0.419361435	0.691196321
PNMA1	0.418901579	0.711866262
ENTHD1	-0.418774804	0.763609493
COPG2	-0.418319889	0.649787771
FN1	-0.41809303	0.738057966
NHEDC1	-0.418002241	0.633023449
TMEM168	-0.417998005	0.614945408
SLC25A46	0.417672369	0.434111239
FGD6	-0.417565781	0.679594133
CNST	-0.417562822	0.682943766
APBB1	-0.417356054	0.71201763

FERMT2	-0.417329037	0.666819087
CHIC1	0.417130671	0.37995426
SAP18	0.417066971	0.711560629
TRPM7	-0.416970634	0.49851134
TFAM	-0.416934044	0.731329685
RASGRP1	0.416640481	0.502988574
YY1AP1	0.416598285	0.539641564
DDAH1	-0.41649109	0.649389686
EFR3A	0.416301627	0.536160221
DGKE	0.416158175	0.539641564
HKR1	0.41598541	0.611187411
CNOT7	-0.415795695	0.737332061
ZRANB2	-0.415318613	0.724765975
DAP3	0.41513229	0.639115826
PEMT	-0.414688241	0.75882855
UBTD2	0.41454185	0.599110248
DZIP1	0.414500175	0.633786928
NHSL1	0.41443523	0.772024202
MAP3K14	0.41412225	0.600792289
SDCCAG8	0.413766982	0.356997335
UFD1L	-0.413598192	0.732134248
PAWR	0.413480289	0.666819087
ATP2A2	0.413425178	0.612612554
C6orf204	-0.413391555	0.521388482
DNAH6	-0.41333269	0.139259294
KBTBD3	0.413213325	0.738057966
USP28	-0.412832291	0.669167794
EPDR1	0.412800917	0.723217017
ERGIC1	0.41279146	0.642905679
PCDHA2	-0.412667064	0.539641564
PCDHA3	-0.412667064	0.539641564
PCDHA4	-0.412667064	0.539641564
ZNF385D	-0.412651513	0.617034121
GUCY2C	-0.412539944	0.701348321
DNAJC10	-0.41251817	0.62606697
STK10	0.412513432	0.716822802
TCEA1	0.412356228	0.738592999
GNG12	0.412328003	0.719090877
KAT2B	-0.411845029	0.599110248
PCDHA1	-0.411772493	0.538436541
NCKAP5	-0.411576491	0.195709581
RABGGTB	0.411520742	0.745565797
CCND3	0.41144069	0.738592999
NR2C1	-0.411293319	0.668730986

C20orf11	-0.41091374	0.716952451
C9orf98	0.41087137	0.716952451
FKTN	0.41080571	0.660727573
BTAF1	0.410717674	0.537094214
FANCI	-0.41070932	0.769497141
HS6ST2	-0.41036099	0.517676567
LINGO1	0.410142952	0.74503496
CABLES1	0.409797645	0.689603128
ANK3	0.40961958	0.023221358
PIK3AP1	-0.409609245	0.715506394
ZNF541	-0.409392688	0.636876515
ITGB3BP	-0.408820146	0.633118116
EDA	-0.408741044	0.643884478
TMED10	-0.408485744	0.717882993
CCDC38	-0.408454961	0.725386766
WDR61	-0.408412692	0.680104272
MEF2C	0.407861844	0.539641564
SLITRK4	-0.407784528	0.720787537
EXOSC7	0.407636465	0.743687136
TBL1Y	-0.407460818	0.737235584
ZNF605	-0.407009428	0.685236772
LAPTM4B	-0.407000794	0.717882993
RAP1A	0.406977124	0.539641564
SDK1	0.406745671	0.417841248
PSMF1	-0.406579576	0.680707446
TTBK2	0.406360019	0.509959641
PROS1	-0.406269309	0.689522486
C2orf60	0.406088358	0.680791773
TBC1D16	-0.40608353	0.636876515
SHCBP1	-0.406054218	0.73228039
MEIS2	0.40592589	0.779167069
MFN1	0.405883454	0.699086709
PRUNE2	-0.405843857	0.521388482
DGKD	-0.405223434	0.701632611
44631	-0.4049101	0.755657119
BFAR	-0.404903294	0.738057966
NECAB1	0.40483082	0.643556325
LOC84856	-0.404559199	0.718566538
STK31	0.404453043	0.757369894
NUP88	-0.404245091	0.742736721
GPATCH2	-0.40423547	0.411612048
CNOT1	-0.404128853	0.665182235
PCDHGA4	0.403951672	0.50860492
KCNH5	-0.403693478	0.537899363

PCDHGB1	0.403587572	0.50860492
RNF146	0.403502781	0.709469789
SNTB2	-0.402936412	0.373751588
ZNF271	-0.402912812	0.633786928
DCX	-0.402816109	0.71710259
LOXHD1	-0.402706861	0.774420007
ANKRD26P1	-0.402526425	0.721760094
UTP6	-0.402524862	0.617034121
ERCC1	0.402358404	0.692609596
NCRNA00259	-0.402348114	0.432538093
VPS8	-0.402258064	0.41244051
ZNF737	-0.40195725	0.756242501
TNNI3K	-0.401644154	0.494509101
EFCAB5	-0.4013866	0.536611055
C7orf44	-0.40127683	0.670433884
GRAMD1C	0.400915369	0.715184974
SLCO5A1	-0.40090045	0.689470334
WWC1	-0.400694585	0.556131395
GTF2E2	-0.400603067	0.720526976
MIR548F1	-0.400373864	0.537899363
CTNNBL1	0.400245311	0.529869829
LARP4	0.400131402	0.666819087
AMMECR1	-0.399923342	0.67944934
ABCA10	0.399876686	0.666819087
ZFHX3	0.399805643	0.660727573
SH3GL2	0.399667175	0.399010034
SH3RF3	0.399521942	0.486027596
CARS2	0.399334074	0.717882993
ATG2B	0.399232245	0.666819087
GIPC1	0.399037016	0.725449643
BBOX1	-0.398812542	0.792375054
MBTPS1	-0.398737072	0.651614595
CDC42BPB	-0.398716119	0.701134748
KCNQ3	0.398700757	0.303137161
PSMD14	0.39861526	0.640455897
APOLD1	-0.398553162	0.769497141
MFHAS1	-0.39825195	0.652051317
MBNL2	0.3981002	0.559945147
HDGFRP2	-0.397876615	0.766875769
ZNF555	0.397726904	0.742736721
OSBP	0.397671703	0.686523913
AKAP7	0.397636622	0.682043169
RABL3	0.397597784	0.615235429
FNBP4	0.397579599	0.539641564

KLHL23	-0.397502295	0.50860492
ALDH9A1	-0.397351233	0.793144888
FAM193B	-0.397171866	0.760192892
PPP4R1	-0.396687546	0.707894128
GPR26	0.396165267	0.71864971
RPL13	-0.395960421	0.723217017
TGFB2	-0.395736678	0.720787537
SCARB2	0.395465628	0.743297264
LOC645166	0.395425541	0.5681702
RCL1	-0.395345282	0.773835293
INPP5F	0.395264026	0.676623755
CLYBL	-0.394853651	0.649720491
TLE4	0.394826196	0.675837558
ATXN10	-0.394644812	0.284744641
LHFPL3	-0.394290746	0.643556325
SNAPC1	0.394255761	0.716822802
BCR	0.394219839	0.71710259
SACS	0.394119033	0.612158837
BEX4	-0.393844727	0.615235429
GABRA1	0.393700471	0.716822802
GALC	-0.393533272	0.631838372
RARS	-0.393484938	0.725475265
ST8SIA1	0.392966858	0.499796502
SEZ6	0.392600309	0.765207588
GNAS	-0.392513722	0.689470334
C3orf70	-0.392095676	0.749861053
RAPGEF2	0.391908947	0.539641564
C18orf45	-0.391839907	0.516812365
MAX	-0.39177108	0.552363401
ALPK1	-0.391739401	0.655328557
LYRM4	-0.391306295	0.327245987
KCNIP1	-0.391253028	0.76484021
ITGA9	0.39123176	0.586353814
CPNE8	0.391207704	0.664508744
NOSTRIN	-0.391070987	0.808096449
TMCO7	-0.391064026	0.71864971
NCRNA00240	-0.391043864	0.782101175
SNAP29	0.390905657	0.726923607
ARHGAP26	0.39075468	0.326522318
ANKRA2	0.390655303	0.769497141
GAD1	0.390645738	0.753200728
CDK6	-0.39061989	0.666819087
SEL1L	0.390373695	0.78103569
RAD18	-0.390298063	0.723539272

IL7	-0.390266374	0.730887774
ZNF25	0.390135827	0.635824017
CHRM5	-0.390020869	0.774420007
IQSEC1	-0.389916601	0.691012836
LIG1	0.389859622	0.769497141
NUFIP2	0.389797942	0.760192892
USP24	-0.389658001	0.675837558
PDE5A	-0.389639437	0.768447793
RRP7A	-0.38961971	0.767223194
DHX29	0.389410198	0.753200728
NFYC	0.389316852	0.680830901
KHDRBS1	0.389302309	0.718505047
PWWP2A	-0.389064595	0.739997641
SEC14L1	0.388743471	0.688565198
CDC73	0.388713363	0.638395789
ANKHD1-EIF4EBP3	0.388207285	0.600792289
CHKA	0.388159613	0.778145609
CRLF3	-0.38793425	0.753200728
FAM107A	-0.387840286	0.702971572
BSN	0.387384875	0.640961918
SYN2	0.387319464	0.467755425
ZNF772	-0.387128539	0.67944934
PSD2	-0.386878879	0.718505047
DDI2	0.386849295	0.715184974
RNF168	-0.386670587	0.725443018
CERKL	-0.386320202	0.666819087
KCNG3	0.386244609	0.665635253
KLF12	0.386020253	0.391867176
LOC285501	-0.385988988	0.757973068
C9	0.385790188	0.785665968
NEK10	0.385768852	0.539641564
BICD1	0.385686601	0.390204648
RBMS3	-0.385655451	0.541189861
ADH5	0.385539863	0.665182235
RHBDD1	-0.385502222	0.623379412
CDC16	0.385464375	0.742948723
SARNP	-0.385256696	0.635824017
IDH3A	-0.385154237	0.634107681
TCF7L2	0.38512699	0.665182235
CCDC132	-0.385050526	0.701381555
NMNAT3	-0.384629113	0.485969231
NIN	0.384323386	0.412894529
PTH2R	0.384230657	0.756950478
TTC33	0.38417306	0.718505047

HMGCLL1	-0.38385655	0.662818688
FAM164A	-0.383718532	0.643556325
C1orf213	-0.383598604	0.787166458
WDR59	-0.383476509	0.666819087
DGKI	0.383470256	0.512799421
ENPP1	-0.38315483	0.805902342
SP100	-0.382493875	0.708925138
TSG1	-0.382434894	0.676623755
XPO1	-0.382341424	0.658905299
RTF1	-0.382271616	0.580348396
MALT1	-0.382217034	0.639115826
SRPK1	0.382188899	0.704225428
ZFP30	-0.382004152	0.714160838
FBXO2	-0.381962214	0.782101175
ARMC4	0.38191209	0.768705629
GPI	-0.38181229	0.533983698
CHUK	-0.381806868	0.686358645
GNG4	-0.381714596	0.711866262
EIF1AX	0.381639745	0.789376554
GAS7	0.381556917	0.585512153
PLCH1	-0.381306235	0.735277785
PDE4B	0.380617848	0.294287912
GTF2I	-0.380580806	0.639115826
ANAPC10	0.380570593	0.685236772
SETDB2	0.380555438	0.675837558
STS	0.38045097	0.726361181
GLB1	-0.380216389	0.738057966
ELL	-0.38014104	0.757973068
LOC284294	0.380081302	0.739874665
SPATA5	-0.379978582	0.542628575
GRM8	-0.379962146	0.639115826
VPS4B	-0.379843333	0.769497141
ZNF251	-0.379716799	0.559945147
PAPD4	-0.379581726	0.537899363
NCRNA00188	0.379446057	0.785327895
NINL	0.379366354	0.588606109
FAM186A	-0.379338953	0.831205091
DNA2	-0.379269698	0.725386766
ARPP21	0.379096378	0.593418611
PRDM5	-0.37880083	0.639569709
FAM131A	-0.378730334	0.738057966
KRCC1	-0.378695578	0.767223194
ARHGEF4	-0.378584592	0.650517077
SMPD4	-0.378575654	0.738386885

C14orf106	0.378382324	0.718505047
SLC25A13	-0.377683965	0.733258229
ABCA13	-0.377664124	0.666819087
PKIA	0.377529853	0.542628575
ZNF182	-0.37730115	0.740399094
LHFPL2	-0.377256024	0.784535666
UNC13B	-0.377177418	0.714160838
RASGRP3	-0.377102175	0.716952451
BBS7	0.377077192	0.759859064
TAF2	0.377051259	0.676623755
APOO	-0.376950725	0.719014023
CCDC12	0.376914435	0.689522486
TAF3	-0.376781769	0.537094214
KDM5A	0.376749617	0.582295892
ARSF	-0.376687264	0.763045207
CRADD	-0.376548364	0.529339243
C10orf11	-0.376508504	0.680830901
PURG	0.376416618	0.773835293
RUNDC2A	0.376309522	0.783398964
EPS8	-0.376301883	0.689470334
SIDT1	0.376251017	0.613647961
NFASC	0.376057539	0.58124695
MED31	-0.375504302	0.600792289
SAE1	-0.375092366	0.738592999
WNK2	-0.374835301	0.337943805
ZNF91	-0.374808742	0.692885129
CPEB1	-0.374776365	0.64968178
UBE2H	0.374183678	0.67944934
ROD1	0.374122879	0.666819087
TNRC6B	-0.374070903	0.435996866
TNKS2	-0.374027554	0.769497141
C9orf123	-0.373944798	0.776331641
44623	-0.373248081	0.726361181
TNPO1	0.372920132	0.639115826
PDE4C	-0.37288425	0.738057966
DCAF8	-0.372745939	0.576693809
CRK	0.372530321	0.738592999
MCPH1	0.372486555	0.450039885
CADM2	0.372472798	0.270486367
STEAP2	0.372422583	0.778145609
HDAC7	-0.372421484	0.738057966
TPM4	-0.37228503	0.757973068
LIFR	-0.372276314	0.725443018
TBC1D23	-0.371689562	0.69368675

DNM1	0.371297244	0.542628575
NDUFS4	-0.371234619	0.582221275
C6orf103	-0.371139089	0.725443018
FAM129A	0.37103754	0.732734607
SLC22A15	-0.370753589	0.576312533
SVEP1	-0.370491581	0.633786928
RTCD1	-0.370366276	0.777370824
PLEKHA2	0.370330918	0.601957315
MTOR	0.37006901	0.479512532
ZFP112	-0.369845277	0.756950478
FER1L4	-0.369612415	0.615235429
HTT	-0.369593132	0.600792289
MND1	-0.369346998	0.756242501
SGCZ	-0.368761999	0.631992903
EBPL	-0.368739491	0.80375854
PRR11	-0.368698328	0.711560629
IFT74	-0.368420423	0.666819087
FIGN	0.368397326	0.363908911
VSTM2L	-0.368279601	0.727281089
CDH19	0.36786163	0.775842274
RAB3GAP2	-0.367712391	0.467103167
ATP10A	-0.367699775	0.641704826
ASXL3	0.367677505	0.683733977
PCDHGA1	0.367563841	0.485965877
TMEM2	0.367496861	0.655728202
ROS1	-0.367445653	0.814605352
C11orf58	0.367362721	0.650517077
COLEC12	0.367361536	0.559164444
C5orf28	-0.367079377	0.751740117
CDC14A	-0.36698561	0.720937195
IQCE	0.36693202	0.720787537
PLXNC1	-0.366830179	0.783398964
PCDHGA9	0.366751768	0.643243379
PCDHGB6	0.366693193	0.643328321
TSC22D1	0.366595874	0.429930115
PCDHGA2	0.366579445	0.501488098
JAK2	0.366555304	0.576588612
B3GALT1	0.366124492	0.705029284
YIPF5	-0.365998507	0.779167069
GPR149	0.365898281	0.804908954
PLS1	-0.36581896	0.759331493
CD59	0.365677657	0.800220112
ECT2	-0.365543185	0.827971242
FAM172A	-0.365354415	0.550441897

RNF128	-0.365353938	0.791776337
KIAA0947	0.365342276	0.722073576
PAK6	0.365152208	0.79741743
TECRL	-0.365121333	0.78103569
DIRC2	0.3650597	0.689522486
SFRS12IP1	0.365047969	0.64968178
TARSL2	0.364971205	0.699086709
GAK	-0.364699487	0.718505047
RMND5A	0.364410585	0.665635253
ZBTB41	-0.364370222	0.778885443
LPCAT4	0.364237747	0.716822802
LRIG1	-0.36405209	0.840200163
TM9SF4	-0.364006091	0.618969295
TPMT	0.363991547	0.751825187
COL27A1	-0.363902022	0.774751614
ZPBP	-0.363810025	0.680830901
UST	0.363751523	0.689522486
C2CD3	0.363393921	0.710024269
ARIH1	0.363390422	0.450039885
STAM	0.363373452	0.715184974
MAPK4	0.363272702	0.711560629
SNX10	0.363163619	0.666819087
ABCB9	-0.363137932	0.725386766
ABR	0.36307173	0.721895164
RNMT	-0.362840952	0.636876515
NRP2	0.36282166	0.773835293
HCK	-0.362588433	0.705029284
FBXO34	0.362503861	0.309412584
PCDHGA10	0.362464474	0.64968178
STARD9	-0.362127501	0.715184974
PGAP2	0.362082508	0.773835293
ELL2	0.361971733	0.673729527
ZNF804A	-0.361722063	0.513718132
WDR25	-0.361657878	0.6769731
PCDHGA11	0.361265378	0.649720491
PCDHGB7	0.361265378	0.649720491
FBXL2	-0.361252275	0.69368675
CAMK2D	0.361143972	0.504113989
PCYT1B	-0.360550331	0.763329655
MIPOL1	-0.360503503	0.565788729
USP14	0.360450995	0.618969295
EHMT1	-0.360388866	0.687821264
PIK3C2B	-0.360307902	0.715184974
PCDHGA7	0.360269325	0.649787771

LTBP2	0.360198873	0.825081413
SLC16A2	-0.359989356	0.71314649
PCDHGB4	0.359696281	0.649932279
RNF11	0.359661878	0.81630481
BCAS2	-0.359589674	0.769497141
MAML2	0.359261553	0.444253645
SPTAN1	-0.359174767	0.600792289
TRAF3	0.359093065	0.666819087
KIAA0141	0.359075671	0.773835293
PCDHGA12	0.359037424	0.655728202
TLK2	-0.35896929	0.615776206
PCDHGA8	0.358825821	0.651521707
LDLRAD3	-0.358486468	0.600792289
INPP5B	0.358356914	0.673729527
BANP	0.35827754	0.793967717
PSMA1	0.35803101	0.655328557
MARK3	0.357994149	0.503144015
PCDH17	0.357925167	0.769497141
LOC729723	0.357793222	0.710024269
NOX4	-0.357788156	0.767223194
MAGEL2	0.357756402	0.767136394
FAM135A	0.35765367	0.50918467
FAM135B	0.357381038	0.270486367
MTMR7	-0.35719478	0.456195828
FAM82B	-0.357171975	0.79998421
ZBTB8A	0.357170771	0.76595106
SMAD9	-0.3569078	0.660727573
COG5	0.356655109	0.37995426
PCDHGB5	0.35648624	0.655328557
SQSTM1	-0.356297126	0.775842274
MTHFD2L	-0.356283374	0.665635253
RBMS2	-0.3562827	0.759815554
FBXL13	-0.356281892	0.649720491
PRKAR1A	0.356273923	0.789376554
PTPN3	-0.356159151	0.797224471
BBS4	0.35609407	0.782101175
CARD11	-0.355936038	0.797224471
LOC401134	-0.355927357	0.767223194
LMF1	-0.355695217	0.709335464
SART3	0.355693474	0.685081528
MIR548G	-0.355626274	0.702166088
ARID3A	-0.355454621	0.863389309
LASS4	-0.355220283	0.767223194
TTC27	-0.354912574	0.643556325

UTRN	-0.354753238	0.666819087
PDE10A	-0.354693732	0.720787537
RNF114	0.354111204	0.730682805
SAMD3	0.354053027	0.639248356
RAPH1	0.353570849	0.574613702
ECM2	-0.353290766	0.819781684
IMPAD1	0.353246537	0.775842274
ZBBX	-0.353089802	0.666819087
FNDC3A	-0.353046652	0.603723228
FOXN3	0.352800299	0.46512918
STX5	0.352570181	0.730202338
DOCK5	-0.352521004	0.716952451
CASP2	0.352434538	0.829255415
KIAA1715	0.35197684	0.681590297
CFH	-0.351789371	0.806012097
ATP8B4	0.351514953	0.384886008
JMY	0.351347034	0.776117017
PPP1R13B	0.351276326	0.536160221
NFATC2	-0.351205181	0.757973068
POLR1A	0.350851451	0.741654603
VGLL4	0.350564897	0.721895164
SLC47A1	0.350291386	0.509959641
MAN2A1	0.350099767	0.666819087
CCNY	-0.35009773	0.636876515
TTC19	0.350090813	0.603706063
TMEM19	0.350086658	0.795222245
ZNF566	-0.349968032	0.716822802
PCDHGA5	0.349731276	0.651614595
PCDHGB2	0.349731276	0.651614595
PICALM	-0.349680027	0.621915243
TEX2	0.349331401	0.511604318
IARS2	0.349215786	0.808096449
KDM2B	-0.349111833	0.760192892
GPHN	0.349089064	0.300016693
ZNF615	0.349079738	0.767223194
ENOX2	0.348998346	0.67075072
ZMYND8	0.348970455	0.435996866
CAMK2A	-0.348849943	0.675837558
RAB3B	0.348620426	0.666819087
PDE7B	-0.34857883	0.697028693
HACL1	0.348569549	0.689522486
USP45	-0.348532526	0.759331493
RNF157	0.348495795	0.539641564
ADRA1B	0.348301583	0.682043169

PCDHGB3	0.348202966	0.655328557
PCDHGA6	0.348201103	0.655328557
GPATCH4	-0.348022095	0.716952451
TCEB1	-0.347920342	0.725386766
TRADD	-0.347539241	0.832737704
NAMPT	0.347257895	0.778145609
PNPLA8	-0.347114152	0.759331493
TRPV1	-0.347021554	0.767975813
SEPT9	-0.347017567	0.773835293
UNC5D	0.347003653	0.512799421
EYA1	-0.346998234	0.778145609
UBP1	-0.346829097	0.682043169
TRAK2	0.346775462	0.688319643
ZBTB38	-0.346747962	0.742736721
NEK4	-0.34671473	0.720787537
BAALC	0.346596632	0.71864971
TRIT1	-0.346228012	0.662580219
ZNF280B	-0.346099796	0.857360627
HINT3	-0.346088523	0.800220112
TGOLN2	0.345846063	0.725386766
C18orf1	0.345380715	0.559684614
NGEF	-0.344951227	0.496572435
C9orf91	0.344860933	0.763609493
RUNDC3B	0.344744958	0.37995426
SLC30A5	-0.344575537	0.753200728
DCAF12	0.344450769	0.789376554
BRE	0.344310076	0.366602325
TMEM150C	-0.344194656	0.759331493
CACNB2	0.343991798	0.333448726
TMEM130	0.343892343	0.681274566
ZNF23	-0.343832458	0.721760094
APP	-0.343767276	0.643556325
RBM9	0.343552694	0.455465505
TMEM38B	-0.343405443	0.797224471
STX2	-0.343386201	0.712349805
MAN1A2	0.343353815	0.375522733
PCDHGA3	0.343334389	0.534128441
PIKFYVE	-0.343210529	0.718577532
EMR3	-0.343167445	0.767862445
RPS6KB1	-0.342845474	0.797801615
UBE2E3	0.342686545	0.677370667
PHACTR3	0.34267666	0.50860492
PSEN1	-0.342554535	0.666819087
SLC38A1	-0.342330962	0.781755389

FBXO36	-0.342298553	0.759878114
ZNF264	0.342123146	0.461189524
TSG101	-0.342095095	0.776331641
LOC91316	0.341993983	0.701134748
PRRG1	0.341993069	0.711866262
MEGF8	0.341966759	0.756160163
MTMR8	0.34194941	0.80178475
ZNF37BP	0.341860905	0.612612554
CLIP1	0.341830569	0.593535977
ARL6IP6	-0.341748274	0.738386885
SRSF11	-0.341500719	0.64968178
SYTL2	-0.341461606	0.765179328
CACNG8	0.341430712	0.805902342
PDE4D	0.341364982	0.270142583
SORCS2	-0.341355039	0.724884029
TLL2	0.341354015	0.832861108
STXBP1	0.341260704	0.715184974
TPRXL	-0.341253831	0.789376554
CLSTN1	0.341087592	0.715184974
SOX6	-0.340713694	0.731329685
ADAM17	-0.340692206	0.479998631
LONRF3	0.340676006	0.769497141
PDE11A	-0.340494336	0.649787771
FSD1L	0.340399929	0.768447793
SKAP2	-0.340314585	0.675837558
EPB41L4A	-0.340141381	0.753200728
ZAK	0.340119235	0.634270995
WDR70	-0.340070633	0.521388482
ARL3	0.339974427	0.802706909
KPNA1	0.339958972	0.704225428
C20orf12	0.339434838	0.676623755
MTAP	-0.339170283	0.797224471
PGM2L1	0.339169655	0.638512323
NFIC	-0.339105096	0.769497141
UBE2K	0.338650694	0.666819087
TUSC3	0.338613728	0.599110248
KIAA1432	0.338505575	0.719991831
PPFIA1	-0.338438098	0.716952451
FAM188A	0.338437538	0.765793784
GIPC2	0.338414833	0.723217017
SLC39A9	0.338359573	0.763779655
ERI3	-0.338329399	0.733258229
SIK3	0.33819908	0.512799421
TBL1XR1	0.33819498	0.63260171

CRISPLD2	-0.338083031	0.81630481
FBLN5	0.338070232	0.831205091
CDKL5	0.337965768	0.665182235
SGK3	-0.337936353	0.705029284
TANC2	0.337713536	0.419826415
FBXL20	-0.337438074	0.639248356
MFSD11	0.337039102	0.698502915
SLC35C2	-0.336890089	0.775842274
ST3GAL2	-0.336842597	0.714160838
RHBDL2	0.336789823	0.823162236
ZC3H11A	0.336757493	0.740399094
C11orf61	0.336430779	0.808007591
ASAM	-0.33642304	0.810905286
GRIA3	0.336419614	0.521388482
STON2	-0.336362312	0.750775328
MED9	-0.336169929	0.820485753
C6orf97	-0.336124972	0.774652175
SETD3	0.336058424	0.730682805
YSK4	-0.336031179	0.817274457
CCNI	0.335967762	0.814605352
EPB41	-0.335709553	0.692100002
NPLOC4	-0.335615805	0.769497141
C3orf63	0.335475711	0.768705629
NHSL2	-0.335409353	0.763609493
SHANK2	0.335365893	0.537899363
ZNF341	-0.335344331	0.716822802
FAM19A5	-0.335113816	0.738592999
VPS33B	-0.334777446	0.846828469
BCKDHB	-0.334726559	0.671538422
ARHGAP29	-0.334356761	0.792375054
ARID1A	-0.334235605	0.760426728
CHP	-0.33415113	0.789376554
EAPP	0.334112007	0.726923607
FNBP1L	-0.334081953	0.775842274
CUX2	-0.334011692	0.773835293
LOC285696	0.333971336	0.599110248
PABPC1L	-0.333760173	0.834683109
GDAP1L1	-0.333693554	0.738057966
PRKAR2B	-0.333673719	0.740399094
NPTN	0.333665072	0.751825187
VANGL2	-0.333161546	0.767223194
ITGA8	0.333135007	0.576312533
AFF1	0.333083915	0.64968178
PPM1A	0.333024893	0.689224736

CDIPT	0.332817787	0.79618738
SNCA	-0.332695314	0.636876515
F8	-0.332587599	0.65079011
CCBE1	-0.332521736	0.725386766
LOXL2	-0.3325114	0.768447793
DBC1	0.33250476	0.550550361
SMAD1	-0.332408372	0.715184974
NCRNA00271	-0.332378477	0.666819087
TMEFF1	-0.332238149	0.588606109
GALNTL1	0.332088421	0.675837558
RAB40C	0.331940175	0.808096449
PDXDC2	0.331399188	0.792375054
AGBL3	-0.331284554	0.670190583
KLHL1	-0.330930518	0.723217017
CCDC82	-0.330852054	0.767136394
SHC4	-0.330646268	0.775842274
SRF	0.330528	0.719683657
GOLPH3	-0.330311888	0.745863114
FSHR	-0.330252737	0.824345194
EFHA2	0.330200776	0.633877767
ABCC9	-0.330171572	0.83838924
LPCAT3	-0.329961018	0.785665968
SKA2	0.329921579	0.767223194
PJA2	-0.329902826	0.64968178
RBKS	-0.329790973	0.715184974
MGA	-0.329607306	0.738057966
ABCE1	0.329440482	0.793924909
ACER2	0.329391129	0.769497141
LMLN	0.329201509	0.748362201
KIAA1731	0.329050085	0.768447793
UQCC	-0.329028004	0.475052728
PRDM2	0.328989408	0.765207588
LOC283194	0.328844772	0.831205091
MDH1	-0.328769278	0.716952451
CELF2	0.328704838	0.485142746
R3HDM2	0.328639716	0.80932859
EFNA5	0.328584758	0.783398964
SMC1A	0.328581522	0.784690569
PPIP5K1	-0.328576523	0.580348396
SGK1	0.328570428	0.640452701
RNF160	0.327999953	0.740094184
GMCL1	0.327665915	0.726020491
RAB12	0.327653557	0.785225039
CCDC13	0.327598128	0.769497141

LOC283050	-0.327419066	0.788631505
GFOD2	0.327385064	0.769497141
SHANK3	0.327380123	0.832737704
HTR1E	-0.327245526	0.537094214
SPPL3	-0.326977401	0.643556325
CHST12	0.326816504	0.692609596
LAMA2	-0.326673028	0.769497141
PPP1R1C	-0.326563596	0.801411909
CYP2J2	0.326365896	0.814605352
AGPAT4	0.326045936	0.639115826
KIAA1549	0.325979413	0.686358645
SERINC1	0.325843129	0.76595106
BTBD7	0.325596144	0.665182235
LOC63930	-0.325484456	0.834841222
ZNF331	0.325430697	0.560329284
CDON	0.325326755	0.651614595
REEP3	-0.324893473	0.766875769
NNT	-0.324870065	0.675837558
FXYD6	-0.324864491	0.776123987
ZNRD1AS	-0.32486104	0.712294283
PTPRQ	-0.324838402	0.741654603
UNC13A	0.324815847	0.674527144
ANKRD20A2	-0.324490934	0.841049381
ANKRD20A3	-0.324490934	0.841049381
PDZD8	0.324344024	0.767136394
LARP4B	0.324163264	0.69368675
XPO5	0.323935115	0.831022546
C4orf32	0.323828382	0.769526203
LRP1	0.323767454	0.789376554
STRADA	0.323707263	0.757591062
LRRIQ1	-0.323689101	0.643556325
NIPA1	-0.32367528	0.80932859
BFSP1	-0.323638392	0.700432775
ASCC3	0.323580785	0.356997335
HSPBAP1	-0.323144463	0.740399094
ZNF618	-0.323063774	0.753200728
USP15	0.322959938	0.559684614
SCML4	0.322858661	0.857360627
NUDT10	0.322801019	0.818529051
CIT	0.322753913	0.759859064
LOC645323	-0.322659889	0.751825187
CHID1	-0.322597761	0.782101175
THSD7A	-0.322503969	0.576312533
HIPK2	-0.322205006	0.711866262

ABCB1		-0.321813153	0.658015027
GPR125		0.321697098	0.773835293
GLB1L3		0.321542251	0.711866262
FAM18A		-0.321321387	0.666819087
DOCK8		-0.321306783	0.792375054
SFMBT2		0.32121406	0.655328557
LOC285965		0.320868647	0.774420007
TBC1D2B		-0.32058561	0.743637226
PLCB4		-0.320524408	0.706359785
TSPAN15		-0.320431563	0.769526203
C20orf152		-0.320428683	0.737332061
SMAD4		-0.32041748	0.80932859
KLHL13		0.320368371	0.775762186
USP49		-0.320195898	0.809823177
PPM1D		0.319967514	0.768447793
	44621	-0.31970544	0.595167881
	44625	0.319643598	0.715184974
GPR1		0.319639039	0.750775328
PPP6R2		-0.319582805	0.64968178
ALG9		0.319529669	0.707894128
WIPF2		0.319452848	0.686523913
TLL1		0.319283499	0.43934568
ST8SIA6		-0.319187894	0.793924909
SMYD4		-0.31912896	0.769497141
KIAA1324L		0.319081422	0.643300339
WASF1		0.318670403	0.785665968
CASC3		0.318491722	0.799342079
MCC		-0.318434248	0.651521707
SETBP1		-0.318360515	0.665635253
LOC643650		-0.318231321	0.83638178
KIF13A		0.318222699	0.537094214
TPD52		-0.318145183	0.680830901
PHEX		-0.317894121	0.799147655
CCPG1		-0.317871792	0.692885129
ZNF850		-0.317826278	0.829255415
CD58		-0.317739402	0.815482297
FHL1		0.317583157	0.805902342
LOC100131564		0.317524841	0.738057966
ZNF805		-0.317470233	0.797488927
NAA35		-0.31737541	0.666819087
CNIH3		0.317288234	0.738386885
GPC6		-0.317181842	0.686523913
NEURL		0.317044601	0.767523836
CCDC115		-0.316985784	0.738386885

DGKH	0.3166827	0.675470782
ZNF532	0.316467574	0.686523913
LOC441204	-0.31643658	0.769497141
NEK5	-0.316428734	0.776646963
ZCWPW2	-0.316383682	0.800220112
HIF3A	-0.316327786	0.79998421
RAP1B	0.316116502	0.814605352
GLCCI1	-0.315991805	0.639115826
ZNF546	0.315860606	0.81630481
HS3ST3A1	-0.315521797	0.817274457
USP25	-0.315450113	0.553499719
GOT1	-0.315396622	0.800220112
ARHGAP21	0.315265341	0.649720491
B4GALNT1	-0.315129134	0.689522486
MYO18B	-0.315056912	0.725386766
RGS7	0.315035591	0.492054732
HIVEP2	0.314898863	0.559684614
CRISPLD1	0.3148157	0.820839714
MGC42105	-0.314762114	0.714160838
WDFY2	-0.314568094	0.393487965
FLJ40330	0.314555819	0.838301757
SMPD3	0.314370949	0.810903267
SLC37A3	-0.314249555	0.763694956
ADAM18	-0.314181743	0.845446387
LAMA1	-0.314162697	0.739874665
KIAA1841	-0.314155065	0.763221246
SEC63	-0.314118759	0.765793784
LRRTM3	-0.313636428	0.738592999
CACNA2D1	0.313493753	0.50860492
ENKUR	-0.313215435	0.769497141
CHRM3	-0.313205067	0.792375054
ELAVL1	-0.313169594	0.715184974
KDM1A	-0.31305375	0.797801615
PRMT8	0.312971713	0.774751614
RASAL2	0.312696894	0.517676567
C20orf94	-0.312554555	0.666819087
IL27RA	0.31244667	0.756415989
C3	0.312334701	0.83851462
PAK2	0.312282014	0.775842274
IREB2	0.312150657	0.721007478
FAM196B	-0.312136453	0.698799032
CASP9	0.312100542	0.78103569
ABL1	-0.311991381	0.707894128
KIAA1958	0.311671674	0.611187411

TOR1AIP2	-0.3116568	0.733258229
HNF4A	-0.31153056	0.802014848
DNAH5	-0.311524635	0.717473164
COL28A1	-0.311408744	0.831205091
FAM45A	-0.311391265	0.775842274
FAM45B	-0.311391265	0.775842274
ANKRD30BL	0.311332288	0.525107715
FAM110B	-0.311267778	0.751263528
CDC14B	0.311189259	0.799488726
KLHL9	-0.311123771	0.686523913
FABP6	-0.31106647	0.777370824
TGFBR3	-0.311057253	0.760152549
CYP7B1	-0.311019046	0.715184974
GOLGA2B	-0.310857519	0.76595106
RHEB	0.310846175	0.788822087
MYO5B	0.310827644	0.640961918
NLRC4	-0.310731419	0.794860797
UBE3A	0.310344575	0.617034121
CCDC146	-0.310194074	0.767223194
ZNF577	-0.310171766	0.784535666
NSMCE2	0.310052391	0.665182235
NCAN	0.310016589	0.738057966
TWSG1	0.309960427	0.805902342
AP4E1	-0.309919985	0.778145609
STAT5B	-0.309915665	0.773835293
DOPEY1	0.309770501	0.666819087
TANC1	0.309756603	0.655728202
ANAPC16	0.309749283	0.753200728
TRAPPC9	-0.309561827	0.64968178
EIF2S1	0.309561141	0.775842274
GPD1L	0.309384253	0.800220112
ADAM10	0.309318009	0.618969295
ATP1B1	0.309291388	0.832737704
ACOXL	-0.3091938	0.725386766
C13orf38	-0.309044299	0.846164113
ITPR2	-0.309007371	0.717882993
DIP2B	0.308940187	0.649720491
GDA	0.30887636	0.753200728
EIF3E	0.308769307	0.769497141
ANKMY1	-0.308644518	0.689522486
NSUN4	0.308558644	0.825660011
FBXO21	-0.30850869	0.844463452
NTM	0.308381055	0.461833249
PKIG	-0.308283057	0.666819087

MYRIP	0.308165509	0.455465505
ZMIZ1	0.308132388	0.829255415
NOVA2	0.307829849	0.805902342
BTNL8	-0.307817267	0.881392042
GON4L	0.307810061	0.738057966
RBPMS	-0.307656433	0.774420007
RC3H1	0.307581811	0.746072701
PLEKHM3	-0.307511085	0.633877767
ZNF423	-0.307479303	0.775842274
RAB7A	-0.307364572	0.705991371
ACSS1	-0.307313318	0.789124805
RNASEH2B	0.307121757	0.723217017
CASK	0.307050265	0.620285381
SORBS1	0.306860259	0.675837558
USP32	0.306829696	0.611187411
TRPC1	0.306736665	0.614945408
PTCD3	0.306657156	0.769497141
FAM13B	-0.306592443	0.676623755
LOC344595	-0.306563073	0.658496908
MSL2	-0.306519862	0.767588604
HBS1L	0.306486823	0.739874665
GDAP2	-0.306454959	0.766875769
ALDH5A1	-0.306426923	0.829255415
KIAA1804	-0.306368119	0.806012097
ADAMTS1	-0.305963274	0.841049381
CLCN3	0.305903914	0.726020491
WNT2B	0.305501557	0.825081413
ANLN	0.305412725	0.855508911
LOH12CR1	-0.305221264	0.80932859
PNRC2	0.305007209	0.797940017
XRN1	0.304939782	0.643556325
NDUFS1	0.304780321	0.699106559
MEMO1	0.30459679	0.529869829
MGAT5B	-0.304337886	0.771763792
SLC9A6	0.303930714	0.797801615
SASH1	-0.303910725	0.785665968
SUSD1	0.303783824	0.649787771
ZCCHC14	-0.303721878	0.733876623
XRCC1	-0.303679295	0.812008725
WDR78	0.303652317	0.666819087
FBXO31	0.30360073	0.773835293
CHRDL1	-0.303423384	0.847207754
NCRNA00119	-0.303238845	0.797801615
TTC13	0.303034116	0.767223194

TEX14	-0.302999684	0.78901896
ZBTB40	-0.302970782	0.824345194
IFNAR1	0.30294178	0.779543911
JDP2	0.302864264	0.846164113
HDHD2	0.302812665	0.831205091
ATXN3	0.302785764	0.722835751
PCID2	0.30273149	0.797801615
TAB2	-0.302593956	0.649720491
ROCK2	0.302566406	0.504113989
CNTN4	-0.302184485	0.679121725
LOC100128252	-0.301911639	0.795054372
KCNH7	0.301803196	0.639115826
TSC1	-0.301761932	0.812008725
PKP2	-0.30167361	0.738057966
RAVER2	0.301629623	0.550550361
CCDC129	-0.301628815	0.801411909
TBCA	-0.30146726	0.67703046
USP6NL	-0.301286549	0.814605352
FAM133B	-0.301284516	0.841049381
LOC728066	-0.301284516	0.841049381
BTNL9	-0.301193016	0.729834562
TMOD2	0.300916379	0.716952451
FAM108C1	0.300838015	0.824345194
ADPGK	0.300830034	0.797801615
PPIG	0.300828048	0.727814325
MYO1H	-0.30080446	0.82421493
ZNF862	-0.300710461	0.78103569
NUDCD1	0.300704952	0.649389686
IPO8	-0.300694539	0.764636593
ZEB2	0.3002704	0.582295892
RUNDC3A	0.300253864	0.691012836
DYSF	-0.299943055	0.783398964
MPP2	0.29986452	0.751825187
KCTD1	-0.299859931	0.620285381
MEG3	0.299762147	0.400648784
EIF2C2	-0.29971762	0.824681514
UACA	-0.299475726	0.79998421
HTR4	0.299369318	0.69368675
GABBR2	0.299367724	0.435996866
SLC22A25	-0.299290904	0.863389309
MYLK3	0.299026233	0.701046279
CD38	-0.29873916	0.804908954
HS6ST3	0.298692977	0.414125861
LOC285045	-0.298677232	0.737235584

SC5DL	0.298368603	0.756242501
ANKRD28	0.298243391	0.588606109
RELN	-0.298178823	0.804908954
WRN	0.298165608	0.666819087
ITPK1	-0.298144976	0.769299053
PPP2R2C	-0.298142029	0.682043169
ADAL	-0.298052312	0.745863114
JRK	0.297740408	0.680707446
C9orf41	0.297721779	0.708925138
SPRED2	-0.297504512	0.757973068
MTX2	0.297474443	0.814605352
DNAL1	0.297195164	0.808096449
KCNJ6	0.297134832	0.611187411
AOX2P	-0.297073062	0.857215568
LOC100130950	-0.296841596	0.737706516
MYO9A	-0.296709921	0.554790624
USP53	-0.296506798	0.829255415
FAM49B	0.296379963	0.767223194
C21orf91	0.296250225	0.845446387
OXCT1	-0.296184398	0.842370734
MYO1F	-0.295764877	0.769497141
TTF2	0.295468935	0.780976732
DST	0.295285381	0.666819087
B3GAT1	0.295246634	0.61346688
EPHA5	0.295193574	0.715184974
ARAP2	-0.295044882	0.769497141
AKD1	0.29492078	0.747668378
GBAS	-0.294799361	0.829255415
SENP7	-0.2947726	0.666819087
CCDC73	-0.294758829	0.831109321
PCGF6	0.294691589	0.848673759
ADCY2	-0.29466554	0.689522486
EPB41L1	0.294642191	0.692206581
LOC100499227	-0.294554131	0.769497141
MDGA2	0.294532047	0.309412584
POMT2	-0.294342331	0.869162179
SLC26A7	0.294295236	0.848673759
ENAH	0.294238212	0.61501659
DRG1	-0.294234072	0.854941787
SPEN	-0.294087118	0.774420007
ARID5B	-0.294023509	0.797687825
ZDHHC7	-0.293832908	0.863389309
IRF2	-0.293718154	0.769497141
ADAMTS19	0.293706194	0.550550361

ALDH1A2	0.293581782	0.62338929
ANKFY1	-0.29352438	0.665182235
KIF13B	0.293357383	0.774420007
GPR64	0.293295532	0.793064726
MKRN2	0.293255453	0.769497141
CSE1L	-0.293093099	0.753200728
TMEM131	0.293084681	0.499796502
N4BP2	-0.293079783	0.782101175
ATL3	-0.292913368	0.842370734
PDIA5	-0.292411697	0.831022546
KIAA1239	0.292252537	0.70650115
FMNL3	0.29211104	0.705991371
TXNRD1	-0.29195566	0.817274457
GPR155	0.2919393	0.716822802
CD163L1	-0.291818746	0.846912594
TBC1D22A	-0.291752399	0.666819087
AASDHPPT	0.291397143	0.810905286
PHKA2	-0.291261276	0.785225039
RAB35	0.291197175	0.874843574
GSDMB	0.291155599	0.805902342
ITGAV	0.290915458	0.773928323
FKBP5	0.290859256	0.671453434
PDZRN4	-0.290747572	0.675837558
ASNSD1	-0.290666217	0.842585832
DGCR2	0.290636883	0.797687825
AARS2	-0.290526339	0.780840098
R3HDML	-0.290456989	0.895292424
SMARCB1	-0.29029346	0.8109058
LONP2	0.290117203	0.658822783
ZC3H12C	-0.290028353	0.806254482
NAALAD2	-0.289961462	0.728447567
DCP1B	0.289917028	0.769497141
LOC339529	-0.289912782	0.769497141
LHFPL4	0.289681123	0.711866262
FAM104B	0.289518207	0.829500812
RSU1	-0.289102586	0.618969295
MCOLN3	0.289060733	0.769497141
SESTD1	0.289044042	0.689522486
KIAA0776	-0.288859504	0.829255415
ACYP2	0.288839805	0.686523913
AHCY	-0.288750826	0.799029936
LRPPRC	-0.288505105	0.723217017
WSB1	0.288340585	0.725590512
KIAA1429	-0.288327322	0.722835751

ITGB5		-0.288085061	0.80932859
PPM1E		0.287847522	0.601606161
LRFN5		0.287568565	0.668730986
TMX4		0.287420084	0.765793784
LOC340515		-0.287388846	0.878636121
MAPK14		-0.287223172	0.80375854
MAGEE1		0.287033356	0.814605352
SGK269		-0.286981672	0.725386766
DVL3		0.286901602	0.82280747
YRDC		-0.286889104	0.834841222
ZNF564		0.286811257	0.795054372
SMC5		0.286781962	0.759815554
C17orf91		0.286681417	0.829349007
TMEM165		0.286549876	0.757591062
ERGIC2		0.286401836	0.811345769
PABPN1		-0.286277995	0.707787282
PHF7		0.286089597	0.802014848
KIAA0247		-0.286081816	0.778145609
VPRBP		0.286048543	0.758928209
AAGAB		0.285957982	0.774420007
IKBKB		-0.285784932	0.738592999
CABP1		0.285774583	0.748362201
PARL		-0.285694103	0.859454229
FAM49A		0.285671168	0.753200728
OPHN1		-0.285553546	0.877668298
COL6A5		0.285551582	0.857360627
NCOA2		0.285476062	0.49851134
INADL		-0.285347417	0.639115826
TSSC1		-0.285267598	0.772024202
CADM1		0.285233771	0.600792289
ARMCX2		-0.285182865	0.721166821
KIF6		-0.285111874	0.774420007
C2orf34		-0.285029844	0.666819087
CASR		-0.284824096	0.886706524
MED13		-0.284768961	0.550441897
FCF1		-0.284627564	0.732208722
RASGRF1		-0.284489846	0.753200728
SLC12A8		0.284437479	0.633023449
APC		-0.284353574	0.600792289
RIMKLA		0.284228478	0.80932859
AK3		-0.28406443	0.829255415
NDFIP2		0.283827263	0.716822802
	44630	-0.283824027	0.814605352
CPNE3		0.28376725	0.823651218

GSTCD	-0.283366393	0.756242501
LRRFIP1	0.283341697	0.701134748
RARB	-0.283189437	0.785225039
HADHA	0.283175109	0.698799032
FGF12	0.283095485	0.448007375
YWHAB	0.283020978	0.753200728
GRB2	0.282999679	0.778145609
CACNA1C	-0.282909545	0.666819087
PGPEP1	0.28287417	0.792375054
SPIRE1	0.282615683	0.583621004
NOTCH2	-0.282526134	0.827952826
LRCH2	-0.282512914	0.705029284
SLC25A17	-0.282295528	0.846828469
DLAT	-0.282277968	0.773835293
C6orf10	-0.282111584	0.867628275
COPS4	-0.282089808	0.842370734
ZNF148	-0.281506645	0.550441897
FAAH2	-0.281383836	0.737684196
FAM120C	0.281368543	0.849968658
PEX13	0.281236938	0.80932859
C8orf83	0.280947506	0.738386885
PRKCB	0.280764665	0.517676567
PAPOLG	0.280749646	0.785665968
CALY	0.280622035	0.774420007
NAV1	0.280499166	0.551092689
PITPNM2	-0.28046217	0.739874665
PI4KA	0.280186905	0.728295853
TTC1	0.280125845	0.846164113
CHIC2	-0.280043773	0.805902342
TRIM26	0.279998911	0.760426728
INTS4	0.279952534	0.769497141
VAC14	0.279308267	0.768447793
CTSB	0.279139536	0.752268055
HDAC3	-0.279132678	0.800220112
SH3BP5	0.278952573	0.711560629
CDC42EP3	0.278728522	0.846164113
NRG3	0.278641918	0.403714321
MIR548I4	-0.278637706	0.846164113
C2orf63	0.278630965	0.767223194
C15orf33	-0.278623102	0.76595106
BBS5	0.278621453	0.726923607
TCEAL4	0.278620348	0.787166458
LOC339290	-0.278524402	0.756242501
SCN3B	0.278466373	0.849859184

ARMC3	-0.278298911	0.85941116
GNA12	0.278281635	0.775842274
RNF24	0.278088979	0.64968178
RMI1	-0.278025042	0.855508911
SH3GL1	-0.27774937	0.86189419
RBM5	0.277532566	0.768447793
ITGAE	-0.277457955	0.841049381
C22orf39	0.277344015	0.747668378
TUBGCP3	0.277177276	0.810062923
ACER3	-0.276875973	0.658905299
AKAP6	0.276778358	0.419826415
TACR1	-0.276769229	0.831022546
PCSK7	0.276756694	0.836364976
C9orf3	0.276366077	0.572735725
FBXL3	0.276278101	0.845446387
PKD1L2	-0.276274729	0.863756803
SHQ1	-0.276217379	0.736849311
CYTSB	-0.276191142	0.641348318
DCK	-0.275815656	0.817274457
DYM	0.275691812	0.600792289
CACNG3	0.275622958	0.683203224
MOSC2	0.27555825	0.840339948
DEPDC6	0.27552385	0.778145609
HEATR5A	0.275516939	0.775842274
ARSG	0.275303624	0.776826259
XPO4	0.275141466	0.769497141
LOC149773	0.275116074	0.846912594
PIR	-0.274836586	0.775842274
TMTC2	-0.274828772	0.723217017
EEFSEC	0.27459953	0.769497141
ZFAND6	-0.274582109	0.834490462
IQCJ	0.274557355	0.810044094
GABRB3	0.27442186	0.536611055
CDKL4	-0.274249248	0.855693115
RIF1	0.274037692	0.842370734
SYT14	0.274000752	0.576693809
ARG2	0.273941926	0.838535791
CAPS2	-0.273916151	0.846912594
WDR44	0.273864814	0.769497141
CNNM1	0.273863129	0.810062923
SLA	-0.273850143	0.877730526
MRE11A	0.273797406	0.817274457
RAB5A	0.273311148	0.840200163
RICTOR	0.273293919	0.736849311

MAP2K5	-0.273179391	0.675837558
UBE2G1	-0.273130516	0.778145609
CDKL2	0.273077286	0.797687825
TAOK3	-0.273035443	0.689522486
SYT16	-0.273032936	0.738386885
UBR2	0.272966908	0.711866262
OSBPL1A	0.272934711	0.721760094
TSPAN5	0.272885905	0.639115826
TTC17	0.27287726	0.64864961
MEIS3	0.272812163	0.817274457
SNX2	0.272719708	0.777370824
ST18	-0.27270496	0.679688626
SEC11A	0.272489875	0.840200163
C17orf57	-0.272486319	0.846164113
COL25A1	0.272314189	0.655728202
LOC375190	-0.271708239	0.810905286
HMGN4	0.271603937	0.805902342
VWA5A	0.271570119	0.848123199
CROCCP2	0.271470705	0.845446387
ZNF611	-0.271341961	0.815328979
KRAS	-0.271288199	0.840200163
MAGI3	0.271247035	0.708558079
C7orf50	0.271165248	0.846912594
GPR133	-0.271095513	0.862500621
THRAP3	-0.271091931	0.814605352
H2AFV	0.270729364	0.76595106
OMA1	-0.270637539	0.857360627
FLJ22536	-0.270618163	0.782794277
ARSK	-0.27020843	0.797224471
STK38	-0.270192752	0.857360627
BCAS3	-0.269783653	0.530964041
LEPR	0.269603356	0.640452701
NR3C2	0.26959314	0.368412981
SPTLC3	-0.269566379	0.807631501
PAPSS2	-0.269312315	0.900677189
QKI	0.269259133	0.659503462
ADAM23	-0.269134099	0.676623755
SMG1	0.269112975	0.769299053
GLS	0.268649972	0.643556325
TACC1	0.268344066	0.719938815
LRP2	0.26821187	0.802552975
CAPRIN1	0.268209207	0.783398964
PAX7	-0.268155682	0.849859184
SNRNP70	-0.268149976	0.743306051

TMX2		-0.268040305	0.86189419
PAPOLA		0.268024622	0.846912594
NUDT15		-0.267943277	0.86127204
LHPP		0.26771117	0.774420007
SCD5		-0.267657697	0.769497141
FRMD5		0.267601413	0.640452701
SOHLH2		-0.267581486	0.862166184
MCART6		0.26738686	0.666819087
ZNF10		0.267047908	0.71710259
NR6A1		-0.266855879	0.715184974
SBNO1		-0.266854524	0.829616541
LOC285954		-0.266569194	0.727814325
ATF1		0.266543057	0.810707417
MTPAP		-0.266523834	0.844051172
	44627	0.266452153	0.846164113
SNX14		-0.266446785	0.689522486
EP400		0.266085777	0.675212725
NEFM		-0.266047264	0.857032817
CCDC14		-0.265994074	0.867741352
ATXN2		-0.265881642	0.767136394
ZCCHC7		-0.265810941	0.655728202
ZNF146		0.265595282	0.78103569
ARID3B		0.265541441	0.824681514
KCNH8		-0.265489224	0.832737704
GRLF1		-0.265451439	0.777370824
ASAP1		-0.265439744	0.747422329
TNRC6A		-0.265200562	0.646780625
CXorf23		-0.265141089	0.773076673
EML1		-0.264998728	0.804369108
PECR		0.264886388	0.829877338
WFDC10B		0.264828433	0.793903047
KIAA0562		-0.264641757	0.846912594
BCAS4		0.264593723	0.82416851
ZNF75A		0.264590944	0.786251809
ANKS6		0.264535883	0.681274566
THADA		-0.264352115	0.649720491
DENND4A		0.26423464	0.50918467
SIRT5		0.264046277	0.738057966
WDR60		0.263853534	0.767223194
PCDH8		0.263852384	0.817274457
ARL13B		-0.263773629	0.783450925
TTC37		-0.263697153	0.723217017
UCHL3		-0.263656034	0.836659846
SLC30A6		-0.263513103	0.738592999

XKR6	0.263236879	0.508524698
FLJ45244	-0.263184067	0.845446387
IFT140	0.263133805	0.814605352
PTGFR	-0.26306571	0.867642707
RFWD2	0.262982575	0.479998631
C3orf33	0.26285282	0.846828469
RAB11FIP3	0.262834214	0.807631501
C1orf55	0.262787827	0.815529336
FNTB	0.262726574	0.767588604
C12orf65	-0.262625648	0.849557876
CLCC1	0.262555691	0.857360627
TACR3	-0.262528174	0.875720412
ZNF713	-0.262487153	0.867037627
B4GALNT3	0.262360042	0.797687825
KPNA3	0.262308379	0.618969295
ZNF407	-0.262204786	0.715184974
CAPZB	0.262183165	0.766531845
KCNIP4	0.262027617	0.430899415
CTNNB1	0.261954625	0.810044094
KDM2A	0.261904592	0.708925138
PUS7L	0.261738553	0.868532694
DERA	-0.261733328	0.846164113
HTR2A	-0.261642346	0.857360627
TNS1	0.261455647	0.814605352
SPATA6	-0.261269423	0.767223194
USP33	0.261191159	0.739521575
LOC220906	-0.261186701	0.811345769
C3orf17	0.261184113	0.829255415
HCN1	0.2610619	0.577557311
CDHR3	0.260972718	0.71864971
MTA3	-0.260879327	0.731042615
NCOR2	-0.260853336	0.812008725
PIP5K1A	0.260844529	0.829255415
DPP8	0.260833798	0.745863114
TNKS	0.260341882	0.759878114
ANKRD16	-0.260187751	0.797801615
ACAD11	-0.260112644	0.831022546
MYBPC1	0.260108908	0.863704345
LYPD6B	-0.260107181	0.695734391
MOBKL1A	0.259508949	0.722835751
LGTN	-0.259365972	0.842370734
RPAIN	-0.259344512	0.838874829
RUFY2	0.259196533	0.778145609
MRPL1	0.259078432	0.82416851

FXN	-0.25903683	0.80845228
PHTF1	0.259033632	0.630253155
SHROOM2	-0.258891696	0.849859184
USP9X	-0.258724268	0.848673759
ALDH2	-0.258358796	0.865028773
ZNF441	0.258345698	0.81098732
PYGB	-0.258301476	0.778885443
ZNF583	0.258202541	0.80932859
FSTL5	-0.258096133	0.797801615
CNGB1	-0.258085251	0.775842274
TBC1D9B	0.258058904	0.806012097
NUP155	0.258044389	0.846164113
AGK	-0.257989068	0.775842274
CD200R1	-0.257889515	0.883755894
SYDE2	-0.257678281	0.779167069
RNF165	-0.25755546	0.757973068
TCERG1L	-0.257414361	0.71710259
COL4A2	-0.257300988	0.84163621
IGSF1	-0.257256657	0.86189419
KIAA0114	-0.256852664	0.849968658
GRIA2	0.256585996	0.636876515
ZNF548	0.256516668	0.846912594
ST6GALNAC3	-0.256481087	0.636876515
GEMIN5	0.256471321	0.837767477
KIF3C	0.25639697	0.797801615
SEC22A	0.256253884	0.666819087
SIN3B	0.256180442	0.849859184
SH3RF1	0.256146378	0.831205091
SNAP25	0.256077992	0.760738523
SLC35E1	-0.256010804	0.831205091
SMARCA4	-0.255959192	0.769299053
C9orf5	0.255915789	0.774420007
EGFR	-0.255906622	0.856786561
RALGPS2	0.255741998	0.50918467
FAM184A	-0.255682915	0.697028693
VHL	0.255550107	0.775145179
BTBD9	0.255403107	0.532354044
COX15	0.255394223	0.857360627
CUL5	0.255379327	0.610014013
UBE2B	0.255067546	0.846912594
ACOT13	-0.254967707	0.849859184
BLM	-0.254909571	0.873237429
BRAF	0.254897995	0.669256124
FAF1	0.254843143	0.559684614

ZMAT4	0.254742213	0.666819087
PCMT1	-0.254733542	0.843755813
NF1	0.25461883	0.559945147
ABCA9	0.254611368	0.867628275
SLC30A7	0.25457162	0.789376554
GATAD2A	-0.254551609	0.878636121
CYB561D1	0.254522191	0.846164113
SULT1A1	0.254255686	0.849859184
RFC3	-0.254217109	0.640163875
AKT2	0.253976678	0.846164113
PHACTR2	0.253803697	0.695113
TMCO1	-0.253553263	0.846164113
CHL1	-0.253473307	0.740399094
TM2D1	0.253356784	0.769526203
STRN3	-0.253355802	0.706359785
SYNRG	0.253303163	0.768150653
ADAMTS18	-0.253224138	0.840200163
ANKRD27	-0.25320125	0.834841222
SYNJ2BP	0.253151377	0.777774763
INTS8	-0.252991351	0.721895164
EIF4G3	0.252791298	0.499105743
AASS	-0.252665805	0.814605352
TMEM67	-0.25252315	0.806254482
ZNF273	-0.252366781	0.719938815
DNM3	0.2522438	0.611187411
ANKRD32	-0.252027241	0.835840588
RB1CC1	0.251799089	0.804762534
TECPR2	-0.251479281	0.812008725
GIGYF2	0.251423242	0.634107681
LRRN1	0.251380398	0.689522486
EPHA7	-0.251352596	0.665182235
C12orf5	-0.251231	0.804369108
MXI1	-0.251069415	0.863389309
PPEF1	-0.250816633	0.867628275
TMEM189	0.250785112	0.666819087
MED13L	-0.250757955	0.70650115
HDAC11	-0.25074098	0.845446387
BDP1	0.250426593	0.708925138
PPHLN1	0.25038678	0.757369894
KIAA0528	0.24958041	0.71710259
AUH	0.249489937	0.726923607
RPRD1B	-0.249394124	0.868532694
INSR	0.24910131	0.756242501
PLIN3	-0.248903829	0.863981934

NDST4	-0.248687097	0.843568343
APLP2	0.248584081	0.87558981
UVRAG	-0.248511947	0.371665587
FNIP1	-0.248469124	0.490679765
KIAA1598	0.248444634	0.71864971
MAPK1	0.248430348	0.67101587
FILIP1L	-0.248203072	0.82416851
STX6	0.248165553	0.776646963
XRRA1	-0.247751803	0.829255415
MARK1	-0.247538328	0.722073576
DDX55	0.247472658	0.846828469
ANKHD1	0.247336169	0.80932859
VTCN1	0.247221676	0.907779136
HEATR1	-0.247208193	0.857360627
CCDC6	-0.247172596	0.775842274
FLJ90757	0.24716647	0.814605352
LRRC3B	-0.247075332	0.71710259
TNRC18	-0.247029088	0.841232599
MAP7	0.246915283	0.576312533
ABCA8	-0.246671319	0.900401187
ORC4L	-0.246539365	0.692609596
DOCK6	-0.246410208	0.846164113
ZNF767	0.246360766	0.725443018
PLEKHA3	-0.246289417	0.846164113
LIPA	-0.246132379	0.85941116
KCMF1	-0.245929146	0.80848083
TMEM178	0.24591814	0.730682805
ETFA	-0.245805068	0.836364976
MFSD4	0.245720791	0.782101175
ARMC8	0.245661527	0.808096449
C16orf74	-0.245554803	0.867628275
SLC30A3	0.245390277	0.83851462
MAP4K4	-0.245337956	0.682043169
FAM151B	0.245271314	0.841049381
PTPRE	0.245260091	0.779357463
C5orf15	-0.245246366	0.857360627
ME2	0.245026602	0.715184974
FARS2	0.244983433	0.514357027
MBNL1	0.244946018	0.726923607
RANBP6	-0.244910752	0.775842274
PILRB	0.244892791	0.902249635
GTF2F1	0.244708439	0.831022546
LYPD6	-0.244620964	0.87558981
DKK3	0.244515435	0.717473164

SCN7A	-0.244205951	0.87884972
ADRA1A	0.244169369	0.530638358
PACS1	-0.24415365	0.792375054
PARP8	-0.24407799	0.747668378
ARHGAP28	0.243723432	0.886822411
SERGEF	0.243639614	0.769497141
ZMYM6	0.243506539	0.778145609
MAP4K5	0.243175719	0.699086709
DHTKD1	0.243169873	0.886822411
EXOC6	-0.243036896	0.620285381
SLC4A10	0.242951294	0.529998171
DCAF5	0.24288366	0.633118116
EPHA4	0.242787453	0.665635253
WDR89	0.242769094	0.773835293
KIF2A	-0.242694249	0.842370734
ZFPM2	0.24239354	0.504113989
DNM2	0.24228886	0.831205091
CNTN3	0.242257594	0.764636593
ARHGAP12	-0.24192209	0.769497141
ABL2	0.24177388	0.711866262
SPG21	-0.241684539	0.867741352
SOX9	-0.241609089	0.867741352
DFNA5	0.241518259	0.882777845
ZBTB20	0.241462569	0.504113989
HEATR5B	-0.241097587	0.784343409
NRXN2	0.241012158	0.895807632
PRKAR1B	-0.241006111	0.810062923
DCTN5	0.240960908	0.856786561
ERN1	-0.240893924	0.878636121
TMEM48	-0.240686355	0.857360627
RNF38	0.240651902	0.825597117
FERMT1	0.240561663	0.829255415
IL1RAPL1	0.240498872	0.640452701
SLC25A23	-0.240468975	0.846912594
SLC16A12	-0.240405682	0.886822411
HIATL1	-0.240385048	0.857360627
OPCML	0.24036585	0.395372162
DNAJC24	0.240266456	0.844542979
TBC1D12	0.240247578	0.716952451
CCDC3	0.24020757	0.774420007
PHACTR1	0.240206789	0.496864742
IGF1	0.24018087	0.870802071
C4orf41	-0.240030437	0.877730526
ATP6V1A	0.240000384	0.633786928

BBS9	0.239955918	0.666819087
DIP2C	0.239896409	0.664508744
NAA50	0.239889228	0.817274457
ABHD10	0.239748879	0.791099658
COLQ	0.239630146	0.814605352
ACTR2	0.239394874	0.85941116
ERCC8	-0.239358215	0.873472463
INVS	-0.239299521	0.716952451
C11orf80	-0.239289808	0.773835293
CCDC57	0.239239378	0.805245427
LOC339751	0.239018999	0.890373571
AP2B1	-0.238968786	0.745863114
FGF14	0.238859529	0.539641564
ANAPC7	-0.238619447	0.805902342
THUMPD1	0.238617921	0.757973068
PLXNA2	0.238567666	0.785379718
PRDM11	0.238281509	0.863569912
STXBP3	0.238221705	0.79442702
ROR1	-0.238154499	0.805902342
MORF4L1	0.238146395	0.890373571
WHSC1	0.23807164	0.639115826
MEGF10	-0.238062806	0.840200163
DUSP16	0.237992583	0.829255415
HSDL2	-0.237970497	0.792375054
NAAA	-0.237935623	0.757973068
POU2F1	0.237734432	0.643556325
ANKS1A	-0.237720454	0.834683109
PRICKLE2	0.237627944	0.738057966
SETD2	-0.237590213	0.666819087
HUWE1	0.237531479	0.807631501
KIAA0355	0.237411163	0.824345194
MTSS1L	-0.237404105	0.87558981
PDE7A	0.237176626	0.768447793
STAG2	-0.236973524	0.769497141
CHCHD6	-0.236774096	0.688565198
LUC7L3	-0.236734255	0.717882993
ARFGEF1	0.236697635	0.811345769
LOC440040	0.236618634	0.705991371
ZNF664	0.236501806	0.797801615
MSH3	-0.236321562	0.699086709
NISCH	0.236162534	0.802552975
LIN52	-0.236149023	0.81630481
UBL3	0.236113677	0.840200163
CYP39A1	0.236021429	0.886822411

EP400NL	-0.235913163	0.857360627
RBAK	-0.235883451	0.863389309
CTCF	0.235794122	0.855508911
CDC27	0.235773449	0.787166458
DPYSL2	0.235760502	0.846912594
MIR548F5	0.235697857	0.682043169
PARK2	-0.235657256	0.571286931
SMARCAL1	-0.235602758	0.846164113
FAM155A	0.235469623	0.436186209
MRPS25	-0.235454794	0.863389309
VSNL1	-0.235417742	0.769497141
RIMKLB	-0.23503352	0.773835293
PPP2R2A	0.23500212	0.792375054
PLCB1	0.234927196	0.48266268
TRPS1	0.234886365	0.838874829
LOC728554	-0.234771076	0.845535656
CLIP4	-0.234719192	0.775842274
C6orf170	0.234472085	0.519811939
NRG2	0.234338212	0.80375854
STIM2	0.234253241	0.785225039
RPAP3	0.234158566	0.86127204
ATPAF1	0.234095904	0.80932859
ERC2	0.234055551	0.430899415
PHF20L1	-0.233887062	0.814605352
CENPC1	0.233635532	0.840200163
CENPE	-0.233537659	0.886822411
SEC23A	-0.233524941	0.820431474
SLC12A2	-0.233413184	0.857360627
BTRC	-0.23340565	0.767223194
NBAS	-0.233271598	0.64968178
FAM115A	0.23326918	0.733258229
UBE4B	-0.233200803	0.774420007
KCNH1	0.233102657	0.550458658
OSMR	0.232911043	0.886822411
LRRC49	0.232699803	0.84163621
SGIP1	0.232686946	0.646780625
TPP2	0.232590891	0.875574401
TMX3	-0.232506998	0.87186901
RCOR3	-0.232361719	0.872613773
LPHN2	-0.232355437	0.840320668
MKKS	0.232278793	0.850751603
DOK6	0.232199039	0.633118116
NFXL1	0.232030071	0.846164113
ABCC1	0.231932432	0.810062923

SYNJ1	0.231906554	0.848075087
INTS4L1	0.231906418	0.897248392
CACNA1B	0.231842299	0.58771878
C21orf131	-0.231735237	0.877730526
SMAD2	-0.23169685	0.78623199
RTN1	0.231676476	0.600792289
ACAD10	-0.231657014	0.838874829
ARL1	-0.231479153	0.806012097
C14orf167	-0.231234576	0.831022546
CATSPER3	-0.231154681	0.846912594
PAMR1	-0.23114324	0.855508911
IRS2	-0.230972683	0.895806501
OLFM3	-0.230503334	0.836364976
IKZF3	-0.230438684	0.890373571
PCNT	-0.230312791	0.897248392
LOC100129620	0.230237689	0.80375854
SLC30A9	0.230235726	0.817274457
C12orf51	-0.230113355	0.666819087
LASS5	0.230068813	0.831205091
LEPREL1	-0.230025263	0.797801615
SMEK2	-0.229982305	0.758928209
PHF3	-0.229836157	0.870802071
SPATA13	-0.229751181	0.739997641
LRRC8D	-0.229610371	0.797687825
SLC8A2	-0.229493875	0.834841222
PDE1A	0.229359628	0.536160221
ZNF365	-0.229235824	0.748510355
TNR	0.229221378	0.680707446
DNMBP	0.229020493	0.877730526
SNX25	0.229015694	0.750530009
LOC285456	0.228926664	0.831022546
PURA	0.228921465	0.882777845
GGT7	-0.228882033	0.786775992
SPEF2	-0.228550549	0.866946966
SEMA5A	0.228442466	0.551092689
BIRC6	0.228388631	0.675837558
ST5	-0.228264804	0.846912594
CAMK4	0.228237583	0.789124805
ACTN4	-0.228155855	0.867741352
KCNN3	-0.228129264	0.867037627
PREP	0.227890837	0.848673759
ARSB	-0.227618602	0.802706909
ZNF69	0.227573791	0.863389309
DSCAML1	0.227463977	0.784690569

PTCHD1	0.227215694	0.839867443
WSB2	-0.227007997	0.854941787
BTD	-0.226925788	0.878636121
KDM5B	0.226923805	0.874611768
SMG6	-0.226805017	0.78901896
CSMD1	0.226761508	0.535811253
RAP2A	0.22675091	0.859803381
TTLL11	-0.22673065	0.65079011
XPR1	0.22643739	0.681274566
CCBL1	-0.226387987	0.886822411
GSPT1	0.22636493	0.857360627
MRPS16	0.226258042	0.836659846
TOP1	0.226184043	0.753200728
MOB2	-0.226131496	0.858013394
PPP1R2	0.226042211	0.870802071
EPB41L5	0.225969348	0.771741272
LIMCH1	0.225842062	0.593535977
MRPS27	-0.225804854	0.85941116
AKAP2	-0.225712495	0.863756803
ZNF512	-0.225493627	0.871574961
ITGA11	0.225430775	0.903025656
ARMC2	0.225415659	0.817274457
AFF2	0.225368563	0.780976732
SCLT1	-0.22524179	0.800220112
CDH8	0.225194518	0.636876515
DHX57	0.225146823	0.777258985
CRTAP	0.224978378	0.8675817
SNX30	0.224958099	0.863569912
ELAVL4	-0.224945371	0.818213475
C3orf77	0.224851155	0.894489843
MTMR3	-0.224706484	0.768447793
HSD17B4	0.224699944	0.829255415
LRRC47	0.224564676	0.824681514
TRPM1	-0.224526032	0.898346316
DGCR5	-0.224462325	0.842370734
KDSR	0.224420073	0.877668298
NT5DC1	-0.224211892	0.848673759
SEH1L	0.224198986	0.850751603
DNAJC3	-0.224156137	0.856354605
LRRC2	-0.223956255	0.870802071
GRIN2A	0.223857787	0.636876515
RERG	-0.223620902	0.834683109
USP48	-0.22350257	0.79998421
CTBP2	0.223471173	0.85941116

HLF	0.223410371	0.845856125
MTF1	-0.223300936	0.874090514
MORC1	0.223287136	0.854941787
LOC647946	-0.223193305	0.85941116
EGLN1	0.223010441	0.845446387
RFWD3	-0.222964394	0.886822411
HSP90AA1	0.222930996	0.797801615
TNS3	-0.222897734	0.877730526
EIF3L	-0.222775438	0.863981934
RYK	-0.222706976	0.877668298
PPIL4	-0.222631395	0.825081413
KCNC2	0.222608775	0.738057966
CAMSAP1	-0.222509266	0.883746314
C2orf88	-0.22234402	0.87558981
EEF2K	0.2223205	0.879983041
PIK3C3	0.22223118	0.831022546
VRK3	0.222160865	0.846164113
PSME4	-0.221842586	0.797224471
PLEKHA7	0.221788539	0.867628275
KRIT1	0.221668392	0.817274457
ZUFSP	0.221629477	0.863756803
CCDC138	0.221565689	0.713977821
SPHKAP	0.221543517	0.655328557
TRIP4	-0.22144684	0.886822411
MINK1	0.221368314	0.896498046
MLXIPL	-0.221239365	0.840567139
CYP20A1	0.221114822	0.846164113
SHPRH	0.221092911	0.711866262
CTPS2	0.221062627	0.867628275
IMPACT	-0.220994006	0.873472463
C10orf119	-0.220812043	0.89720772
TJP1	-0.220662016	0.682043169
LOC100190939	0.220657039	0.829740617
PHKB	-0.220531145	0.730682805
CCDC40	0.220511957	0.87558981
KIAA0564	-0.220051122	0.633786928
HERC2	0.220036678	0.774420007
EZH1	-0.219977675	0.883274428
PKNOX2	0.219937664	0.840200163
KIDINS220	-0.219868858	0.845446387
C1orf175	-0.219835383	0.867628275
KIAA1797	0.219673368	0.643556325
CRYZL1	-0.219662494	0.81630481
KIAA1267	0.219573805	0.689522486

STMN4	0.219569702	0.851906367
ANKRD29	-0.219514737	0.901016707
OSBPL9	-0.219156349	0.720787537
CARS	-0.219081444	0.900401187
RNF115	-0.218976549	0.786251809
RNF13	-0.218919153	0.835840588
FAM65B	0.218903048	0.768447793
OR8U8	-0.21877847	0.849859184
FBLN7	0.21872801	0.890373571
ARID1B	0.218689189	0.568484818
ITIH5	0.218565218	0.897037085
PGCP	-0.218544577	0.738592999
GPR98	-0.218503916	0.773835293
NUP160	0.218359739	0.846164113
ARL6	0.218312672	0.789376554
RNF121	-0.218301621	0.82416851
PRPF38B	0.218170765	0.878636121
ZNF37A	0.218090636	0.71201763
TSGA10	-0.218067784	0.639115826
ELMOD3	0.218052335	0.869162179
ZFP82	-0.217797967	0.870802071
HERC6	-0.217771889	0.847517469
LAMC1	0.217724357	0.882777845
NOL4	0.217457228	0.539641564
ZFHX4	-0.217394238	0.886868769
COPA	-0.217248473	0.80848083
RFX8	0.217150421	0.877668298
TAP2	-0.216967259	0.883412437
ATG12	-0.216825133	0.844051172
GALNT2	0.21675267	0.855508911
SMG7	0.216436917	0.891905291
PIK3R4	-0.21642723	0.891983408
PPPDE1	0.216256556	0.877730526
PALM2	-0.216172244	0.539641564
PHAX	0.216143294	0.87558981
NEK11	-0.216020509	0.867628275
TADA2A	-0.215807993	0.895807632
FSIP1	-0.215509483	0.817274457
VAPA	0.215400188	0.898346316
KLC1	0.215374554	0.807631501
EDIL3	0.215364867	0.559684614
LDOC1L	-0.215336688	0.829469834
PIGX	0.215254305	0.906002419
VWF	0.215215776	0.878833959

MAP3K13	0.215155357	0.86189419
NBEAL1	0.215112613	0.738057966
ARHGEF12	0.21510468	0.824681514
GFOD1	0.215066907	0.719938815
LOC145783	-0.21485558	0.890373571
LBP	0.214659081	0.895586242
FUT10	-0.214538894	0.857360627
VPS13D	-0.214466248	0.797224471
FBXO9	0.214223418	0.895292424
MICAL2	0.214038167	0.738057966
KIAA1671	0.214012827	0.81630481
SMCHD1	0.213916036	0.773835293
WHSC2	-0.213886697	0.775842274
PFDN1	-0.213621322	0.882777845
NINJ2	-0.213564545	0.895057828
LOC642852	0.213530166	0.804908954
LOC92249	0.213431052	0.630162987
DECR1	-0.213414379	0.863389309
FAM169A	0.213403019	0.824681514
SNX1	0.213194636	0.767223194
GRIK5	-0.213149539	0.875720412
ANKDD1A	0.213100299	0.771253308
ADCY5	-0.213028667	0.877730526
C12orf56	0.213011129	0.894936358
VPS36	-0.212851439	0.778145609
RAPGEF5	0.212842231	0.64864961
CNNM4	-0.212720552	0.864083767
CALCR	0.21263633	0.907779136
PPM1B	0.212594811	0.861851422
ALK	0.212563208	0.863981934
RERE	0.212449775	0.653338331
AHI1	0.212426013	0.665635253
UBR3	0.212398857	0.639115826
ZNF662	0.212337927	0.876178359
ACO2	0.212272774	0.863704345
ZNF483	0.212034209	0.840200163
ZNF573	0.2119978	0.863569912
ERBB4	-0.211936143	0.665635253
EVL	0.211861632	0.855508911
PPP1R16B	0.211760604	0.717882993
CCAR1	-0.211707977	0.857360627
LOC100128398	-0.211661777	0.910958188
METT5D1	-0.211558034	0.754319107
CC2D1A	0.211480935	0.853177966

PLCXD3	-0.21131961	0.907326731
LOC254559	0.211251254	0.913540531
SYCE1L	-0.21115413	0.811946189
MYO9B	0.211102008	0.885598524
DLG2	0.21107311	0.527951131
UBE2QL1	-0.21098898	0.842585832
SFXN4	0.210845537	0.874090514
TMEM90B	0.210762199	0.80932859
ZFP14	-0.210563413	0.849557876
FBXW2	-0.210531651	0.809847259
KIAA0226	0.210519171	0.897037085
MAP3K15	0.210445634	0.88787435
SLCO1B3	-0.210437423	0.900401187
RDH11	0.210401017	0.845446387
RPS6KA5	-0.2102693	0.712294283
TTTY15	0.210010417	0.943622135
PHACTR4	-0.209768167	0.810905286
ZNF266	-0.20959776	0.886822411
ROCK1	-0.209576079	0.757591062
SECISBP2L	0.209489397	0.715184974
SAP30BP	0.209446809	0.877668298
DYNLT3	0.209163741	0.886822411
PSPC1	0.209119498	0.805384079
PTK2	0.209110126	0.635924334
ZNF638	0.209060748	0.717882993
KCND2	0.209028635	0.725443018
LUC7L2	-0.209013169	0.806012097
HECTD2	0.208920402	0.863389309
ABCB11	0.208910322	0.886822411
EXOC5	0.208726158	0.85941116
PITPNC1	0.208714342	0.711866262
LARGE	0.208642375	0.697920084
DCTN4	-0.208500473	0.842370734
C17orf63	0.208494342	0.863704345
SLC25A12	0.208477019	0.755744198
ZNF569	0.208296378	0.842370734
ALAD	0.208198873	0.863569912
CSNK1G1	0.20797508	0.649932279
C4orf33	-0.207947837	0.867741352
SYS1-DBNDD2	-0.207819145	0.786251809
EDNRB	-0.20778662	0.915078054
ANKRD54	-0.207743858	0.882777845
DTNA	0.207713452	0.675477811
STIM1	-0.207657927	0.834841222

BAI3	0.207640424	0.433385889
ARFIP1	-0.207486447	0.812529076
MPHOSPH9	-0.207370799	0.895292424
FAM174A	0.207363282	0.913540531
CASC4	0.207334368	0.769299053
IPMK	-0.207286428	0.829255415
DAAM2	-0.207168555	0.886822411
C11orf41	-0.207091165	0.824345194
CCDC109A	0.207057898	0.831022546
POT1	0.207016086	0.755126105
CANX	-0.206985137	0.858013394
LUC7L	0.206972499	0.878269474
C2orf42	0.206864375	0.883755894
PRMT7	0.206741756	0.846828469
MGAT4C	-0.206712141	0.739182737
RBM16	0.206680861	0.831022546
CRYL1	-0.20665021	0.863389309
GRM7	0.206484251	0.675837558
SSH1	-0.206381203	0.843600803
XIRP2	-0.206242416	0.878636121
HOMER2	-0.206210258	0.857360627
VPS41	0.206131323	0.81630481
CDK8	0.206028124	0.829255415
USO1	0.205797175	0.840404726
ERO1L	0.205510323	0.894489843
HP1BP3	-0.205443114	0.812529076
NLK	0.205172068	0.740399094
KSR2	0.205170812	0.665182235
FCHSD2	-0.205038387	0.79998421
FAM171A1	0.204897795	0.76595106
CHD1	-0.204769666	0.889289633
KLHL7	0.204754716	0.818213475
FTSJ1	-0.204393334	0.884936232
DTX3	0.204316998	0.858223421
HECW1	-0.204158686	0.789376554
ABHD2	-0.203891086	0.805902342
LPPR5	0.203456547	0.829255415
POLR1D	0.203383392	0.85941116
PRKRIP1	-0.20337621	0.893653466
RICH2	0.203372688	0.612612554
FAM125B	0.203357162	0.720787537
REEP5	0.203352665	0.81630481
DCUN1D4	-0.203328771	0.797801615
RALGAPA2	-0.203317987	0.721895164

CDK5RAP2	-0.203287708	0.867628275
NRG4	-0.203211397	0.853754386
SFXN5	0.203155019	0.840200163
PLK1S1	-0.20306121	0.829255415
C16orf45	0.203049855	0.767223194
DEAF1	-0.202974389	0.893292822
KDM4C	0.202965247	0.573906746
TPST2	0.202947868	0.907887719
DTNBP1	-0.202940209	0.882777845
BCAT1	0.202909614	0.863389309
TMEM192	0.202816026	0.886822411
PAFAH1B1	0.20277889	0.757973068
PDGFC	0.202736293	0.842585832
CASC2	-0.202426554	0.834841222
CRLS1	0.202359533	0.832737704
HPCAL1	-0.202114988	0.895586242
SGK223	-0.202099399	0.888745066
GPRC5B	-0.202068367	0.810062923
IGFBP5	0.202046412	0.893292822
SCN8A	0.202012904	0.753200728
MAP9	0.201957604	0.88787435
FUT9	-0.201930792	0.763351097
CRMP1	0.20172871	0.782794277
P4HA1	-0.201601328	0.804762534
KIRREL3	0.201535347	0.730682805
MYH15	-0.201449728	0.886822411
RTN4RL1	-0.201307355	0.887559181
ZFYVE28	-0.201254676	0.725386766
STXBP6	0.201138388	0.707894128
GTF2H1	0.201055683	0.890373571
PTPN14	0.201002648	0.844463452
PNKD	0.200950367	0.831205091
TSHZ2	0.200947727	0.846912594
HRNBP3	-0.200861467	0.815529336
TUBA8	-0.200850825	0.915078054
EDARADD	0.200827452	0.927848005
DIAPH2	0.20078861	0.682043169
GPR137C	0.200710782	0.846912594
MTO1	0.200709352	0.895292424
PSMC6	0.200699552	0.900401187
CD2BP2	0.200686511	0.86189419
WDR36	0.200605455	0.880549057
TERF1	-0.20057832	0.894936358
KIAA0922	-0.200454375	0.887670299

GPSM2	-0.200449942	0.87558981
RC3H2	0.200418663	0.867741352
SMOC2	0.200303582	0.907326731
CD2AP	-0.200286874	0.842370734
GDF1	0.200272425	0.857360627
LASS1	0.200272425	0.857360627
PLEKHA1	-0.200267508	0.863569912
COL11A1	0.200253609	0.881392042
PARG	0.200223819	0.855508911
TMC2	0.200190876	0.846912594
GRB14	-0.200080649	0.878636121
CAMK2N1	0.200072964	0.822488875
LY75	-0.200009928	0.890373571
KIAA1009	-0.19998362	0.867628275
CPD	0.199749414	0.805384079
ZMYND11	0.199585564	0.821008578
DCUN1D2	-0.199484218	0.709335464
FANK1	-0.19938964	0.849859184
INO80	-0.199230711	0.814605352
SLCO3A1	-0.199155942	0.716822802
HNRNPD	-0.199113951	0.857360627
MYCBP2	0.199075306	0.655728202
STARD13	-0.199071038	0.867741352
POLH	0.199069852	0.886822411
INPP1	-0.199067158	0.887670299
CACNA2D3	0.199053866	0.582221275
DUSP19	0.198892991	0.846164113
NEK7	-0.19875544	0.868532694
BCCIP	0.19875532	0.900401187
ATP2C1	0.198574701	0.725386766
TOMM20	0.198507272	0.846912594
XIAP	0.198507148	0.857360627
GCOM1	-0.198370658	0.907779136
ZNF304	-0.198327778	0.867628275
CD99L2	-0.198182005	0.876178359
MRAS	-0.19794454	0.861851422
C2orf43	0.197802071	0.840320668
PDS5B	0.197740024	0.666819087
LOC729178	-0.197479553	0.805902342
RASA1	0.197359617	0.840320668
TMEM132B	0.197338809	0.78103569
PLEKHA6	-0.197152038	0.846164113
ANKRD13C	0.197141614	0.71710259
PPP5C	0.197136951	0.902249635

ATP9A	0.197032106	0.831022546
NDUFA5	0.196952124	0.907779136
IMMT	-0.196817991	0.888755423
LOC91948	0.196715591	0.886822411
FILIP1	-0.196584668	0.893292822
KL	-0.19657491	0.900401187
LOC283867	0.19631494	0.882777845
ZBED4	0.196258535	0.894936358
LIN7A	0.196212235	0.87884972
GNG7	-0.196202431	0.874090514
SLC16A1	0.196041245	0.8957775
SMAD3	0.19600989	0.863389309
ZNF204P	0.195855585	0.847207754
FLJ30838	-0.195845197	0.701632611
MIR1256	0.195837742	0.774420007
POC1B	-0.195829781	0.886822411
NPTX1	0.195567015	0.886822411
C19orf28	-0.195510641	0.838874829
RPS6KC1	-0.195421831	0.8052775
GPBP1	-0.195341626	0.87406896
MMP24	-0.195122898	0.829255415
STMN2	0.195049289	0.907004862
TTC39C	0.195041971	0.686523913
RALGPS1	-0.19485289	0.777370824
MYO16	0.194782299	0.78103569
DIXDC1	0.194773565	0.831022546
ZNF761	-0.19466645	0.884455861
PELI2	-0.194643676	0.87558981
RNF169	-0.194608938	0.843568343
CHST11	0.194546979	0.722073576
HMBOX1	-0.194540956	0.847517469
AHDC1	0.194502754	0.862500621
KIAA2026	0.194303652	0.887559181
LASS6	0.194280684	0.597235146
KIAA1407	0.194220036	0.897248392
FBXL5	0.194203957	0.863704345
MAP3K5	0.194018497	0.690789747
TMCC1	0.193995701	0.689522486
DPY19L2P2	0.193990893	0.890373571
KIF1A	0.193972002	0.870690284
TTLL3	0.193959468	0.863756803
EPN2	0.193855456	0.840200163
FAM73A	0.193726243	0.877730526
SCHIP1	0.19365582	0.71710259

KLHL5	0.193534935	0.757973068
GATAD2B	-0.193418619	0.849485136
C14orf2	-0.193251146	0.886822411
A2BP1	0.19324577	0.559945147
NSMAF	-0.193140274	0.846164113
ADRBK2	-0.193060937	0.785225039
KCNK13	-0.193025076	0.877668298
EPRS	-0.192871297	0.886822411
NUP153	-0.192862591	0.863981934
CHST8	0.192760787	0.906758613
LANCL2	0.192712445	0.877730526
WHSC1L1	0.192389839	0.814605352
PIGL	0.19232777	0.874317475
ERP44	-0.192297302	0.785379718
CLSTN2	0.192276059	0.711866262
SLC3A1	-0.192244445	0.942099283
TAG	-0.19220922	0.908422867
LOC643749	0.192197886	0.688489196
KLRAQ1	0.192100543	0.803253091
MRPS28	0.192085756	0.857360627
RNF213	-0.192056748	0.88787435
PPARGC1A	0.191890542	0.882777845
PRKAR2A	-0.191878261	0.886822411
AHCYL2	-0.191870285	0.66041462
NUP93	-0.191852712	0.805902342
LINGO2	0.191773687	0.707894128
MBOAT7	0.191543315	0.863756803
NPAS2	-0.191458304	0.799853188
EP300	0.191384888	0.800552208
SMARCA2	0.191327942	0.817274457
FBXL4	0.191317733	0.888070278
SPCS3	-0.191315384	0.877779157
ELOVL6	0.190998051	0.877730526
NDUFA10	0.190870619	0.834683109
UBAP1	0.190806666	0.886822411
CUL3	0.19076577	0.768447793
COQ4	0.190754411	0.88406385
ESRRG	-0.190745053	0.775842274
TRIM33	-0.190672475	0.738057966
RGS12	0.1905615	0.831980742
MAOB	0.190424903	0.846912594
SRC	-0.190349717	0.895292424
DYNC1LI2	-0.190261561	0.877668298
NMT1	-0.190096561	0.894489843

ABHD6	0.189748429	0.840320668
NR3C1	0.189623559	0.841049381
LOC253039	-0.189495495	0.898346316
SH3BGRL2	0.189492481	0.886121682
C6orf106	0.189442116	0.888226982
ACPL2	0.18935735	0.896498046
LRRC67	-0.189354604	0.895057828
ST6GAL1	0.189290639	0.865704111
WFDC11	0.189289105	0.886822411
DAB1	0.189276251	0.612612554
SV2B	0.189256103	0.8222211
BAT2L1	-0.188988295	0.833183964
FADS3	-0.18872126	0.909677859
FTO	-0.188716075	0.769497141
WBP4	-0.188661118	0.92181255
SNX16	0.18866086	0.920610435
THRB	-0.188635165	0.723217017
NAPB	-0.188584352	0.846164113
FAM83B	0.188444527	0.945851072
HSF2	-0.188337594	0.891425811
FLNB	-0.188310038	0.886457364
DNAJC12	-0.188005611	0.88787435
SYNGAP1	-0.187878324	0.917679156
RABGAP1L	0.187831942	0.539641564
DPY19L3	0.187663246	0.805902342
C1orf27	0.187653033	0.867741352
UHRF2	-0.187458953	0.895292424
HIRA	0.1872194	0.86127204
ZNF143	-0.187137912	0.895807632
ZFYVE9	-0.187025164	0.797801615
XPO7	-0.186987166	0.810062923
CNTLN	-0.186961774	0.774420007
FAM5B	0.186925179	0.831022546
ANK2	0.186856319	0.665635253
MGAT3	0.186679706	0.886822411
ZDHHC14	-0.186623551	0.867628275
AKAP13	-0.186474202	0.867628275
AMN1	-0.186421328	0.867628275
SRP54	-0.18637768	0.894936358
CROT	0.185935958	0.890373571
ZBTB44	0.185761245	0.804908954
CSPP1	0.185728118	0.842585832
BTBD1	0.185641742	0.915078054
POLR3F	-0.185609598	0.849859184

CYB5R4	-0.185537687	0.863704345
ATXN1	0.185406196	0.727281089
WDR35	-0.185402895	0.870105898
CDH22	0.185315062	0.767588604
KIAA0319L	-0.185146883	0.831205091
ZCCHC17	-0.185041204	0.886822411
KCNT2	-0.184982071	0.666819087
PPP2R5E	0.184965122	0.802552975
GLRA1	-0.1849167	0.939144732
CCDC91	-0.184896973	0.712294283
BHLHE41	-0.184817911	0.875039861
SNURF	0.184627884	0.775842274
GLDN	0.18442935	0.886121682
IDE	-0.18438211	0.877668298
EXD2	-0.184218369	0.881392042
MKLN1	0.184157561	0.709335464
PIGT	0.183984692	0.867628275
UBE2W	-0.183765785	0.85941116
VPS53	0.183621084	0.740399094
CYFIP1	-0.183608134	0.898346316
TMEM117	0.183099967	0.750775328
LGR4	0.183082301	0.895807632
PAIP2B	-0.183064527	0.895057828
PRKAG2	-0.182945791	0.872945556
GNPTAB	0.182825434	0.869162179
AGBL4	0.182710493	0.585512153
FLT1	-0.182702297	0.890373571
HDAC4	-0.182676019	0.775842274
KMO	-0.182653142	0.908173235
TTC21B	-0.182556527	0.841049381
CSNK1G3	-0.182252391	0.778145609
ZFP28	0.182238739	0.895057828
CORO2A	0.182091814	0.913540531
MLLT10	0.182029017	0.77243978
CRYM	-0.182023789	0.895807632
RSPO4	-0.182000758	0.893653466
ITPKC	-0.181788503	0.924225123
RBM25	0.181734403	0.83638178
PSMD11	-0.181627695	0.918616956
ARPC5	0.181563694	0.903252434
MYH10	0.181534514	0.846164113
PPP2CB	0.181494886	0.892459242
FER	0.181417833	0.665635253
KIF3A	0.181395393	0.92968159

HS2ST1	-0.181317674	0.775842274
GSK3B	0.181096557	0.831205091
RORA	0.181047205	0.577557311
PIK3CA	0.180795963	0.797801615
ZC3H14	0.180709103	0.797224471
NRG1	0.18068106	0.79998421
ZNF644	0.180658647	0.782101175
PRKCA	0.180571636	0.837843669
IGSF11	-0.180551581	0.841049381
PCDH7	-0.180521836	0.812529076
IDS	-0.180499314	0.848673759
FAM161A	-0.180462085	0.886822411
C1orf43	0.18044229	0.858013394
FLJ13197	-0.180406234	0.852447496
RBM19	-0.180192643	0.913540531
RNFT2	-0.180184602	0.892633725
PBRM1	0.18008079	0.878269474
CPNE4	0.180076573	0.767223194
DOPEY2	-0.179954082	0.875012781
DNM1L	0.179946979	0.863704345
BIVM	-0.179837549	0.894936358
CCT6A	0.179819377	0.944194957
SLC6A17	0.179623486	0.909446504
CAB39	0.179416098	0.816673067
OSBP2	0.179384456	0.786251809
SPRED1	0.179292689	0.882777845
CREM	0.179265875	0.900401187
SHC3	0.179265777	0.886822411
KIF26B	0.179241187	0.870407538
PELI1	0.179163619	0.886822411
KCNN2	-0.179114794	0.901859902
PLS3	0.178837452	0.929062682
TRIM37	0.178643642	0.842988097
TMEM35	0.178605977	0.92181255
MPP7	0.178531433	0.863389309
FAM13A	-0.178442194	0.846912594
POLE	0.178398491	0.889032447
MFSD8	0.178397975	0.909195436
NFIA	-0.178261799	0.839597292
HTR7	-0.178259355	0.915078054
CACNA1D	0.178236698	0.689522486
WDR19	0.178125724	0.89720772
SORBS2	0.178065312	0.79998421
SUMO1	-0.178040636	0.876720394

ICA1	0.177898615	0.814605352
FBXL7	-0.177800369	0.87558981
TAPBP	0.177781744	0.88787435
OSBPL6	0.177748284	0.785225039
SLC37A1	0.177665563	0.903252434
HSD17B6	0.177641306	0.933054351
TTTY14	0.177572958	0.92181255
TRIO	0.177414246	0.562037557
CCDC141	-0.177192243	0.893292822
MYO1E	0.177090149	0.903252434
AP3S1	-0.177029158	0.886822411
CUX1	-0.177010177	0.87558981
TMEM56	-0.176919554	0.897037085
ZNF704	0.17691732	0.781234069
SP140	-0.17690182	0.897037085
PTK2B	0.176800366	0.806171482
RPN2	-0.176718334	0.87558981
PPFIA2	0.176436946	0.666819087
PRKG1	0.176381109	0.725386766
ATG10	-0.176000205	0.811345769
FBN1	0.175986159	0.867628275
C6orf168	-0.175869218	0.903719105
DYNC1I1	0.175823806	0.711560629
PAR5	-0.175682998	0.943622135
ZBED5	0.175679326	0.879983041
OAZ1	0.175665089	0.897230606
FAM193A	0.17564475	0.819781684
IMPA1	0.17545014	0.940837879
FAR2	-0.175403768	0.896528149
JAG1	-0.175314344	0.918396705
ECE2	-0.175247648	0.933054351
RUFY1	0.175138086	0.886822411
CNOT2	0.175076504	0.879885284
KCNB1	-0.174977906	0.783398964
EMID2	0.174855522	0.870407538
SNAPC3	0.174794431	0.897230606
ProSAPiP1	-0.174687306	0.930916738
LSAMP	0.174580937	0.683538577
GNL3L	-0.174489148	0.887559181
PRTG	0.174351206	0.895807632
ARMCX3	0.174239861	0.886822411
RPP14	-0.174155796	0.912288744
FLJ33630	0.174114008	0.919409523
CLIC2	-0.17402445	0.892611212

GAS8	0.173879476	0.903252434
CD46	0.173863872	0.913540531
SYT17	-0.173854837	0.778145609
PAPSS1	-0.173827438	0.907276289
CHURC1	-0.173723835	0.88787435
ARHGAP22	-0.173644512	0.884826682
FMN2	0.173576337	0.712129465
GBE1	0.173443851	0.814605352
CTNNA3	-0.173403316	0.866674601
NARS2	-0.173297416	0.886706524
RAB11FIP4	-0.173253501	0.867741352
GSS	-0.173247631	0.891594117
SAP130	0.1731703	0.886822411
PLAA	0.173054641	0.88787435
SLC12A5	-0.173015278	0.886822411
ZNF33B	-0.173014581	0.846912594
SNX4	-0.17282969	0.895057828
ATG7	-0.172814873	0.846912594
CEP57	0.172789987	0.857360627
DYNC1H1	-0.172588071	0.878269474
AKAP11	0.172267741	0.927848005
IPO7	0.172265851	0.913540531
GDPD1	-0.17221487	0.886822411
FLJ16341	-0.172143706	0.918396705
ZADH2	-0.172107002	0.895807632
RGS20	0.172073884	0.882777845
AP2A2	0.171961568	0.93489955
MGST2	-0.171898496	0.91913785
GNAI1	-0.171847828	0.908422867
NBPF1	0.171713577	0.895292424
SHISA9	0.171673205	0.738057966
SLC25A21	0.171574534	0.895292424
ZNF726	-0.171551144	0.912415041
CTTNBP2NL	0.171471705	0.912046651
ANXA11	0.17131996	0.892459242
ANKRD12	0.171283883	0.844211257
HDAC9	0.171081522	0.740094184
FBXO42	-0.171017039	0.893652964
KIAA1274	-0.170939614	0.879885284
RAB10	0.170794333	0.831205091
DCAF7	-0.170732695	0.894489843
KDM3B	-0.170717892	0.895807632
LLGL1	-0.170606898	0.933054351
GLG1	-0.170393965	0.842585832

HECTD1	-0.170363881	0.867628275
SPATA7	-0.170264296	0.824345194
WLS	0.170246911	0.897037085
ABCA4	-0.170243895	0.940760032
GUSBP1	0.170088353	0.78018008
C5orf25	0.170084746	0.886822411
C1orf115	-0.170073034	0.915718839
FBXO32	0.170006945	0.942452262
LASP1	-0.169951774	0.863389309
MBTD1	0.169889243	0.860254293
SUZ12	-0.169860876	0.900401187
ELF2	-0.169751394	0.886822411
IFNGR2	0.169499028	0.869162179
ARHGAP17	0.16943081	0.806012097
BIN3	0.169399985	0.878636121
SFRS18	-0.169397961	0.85941116
TTLL7	-0.169264275	0.871538419
LRP8	-0.169235965	0.888226982
ANUBL1	0.169169836	0.917611589
MED15	-0.169010732	0.93489955
PPP3CC	-0.168956828	0.842370734
ARHGEF11	0.168716493	0.85941116
ITGA2	0.168505334	0.937721851
LEPROT	0.168504187	0.863756803
NUP62	0.168490848	0.897037085
C2orf72	-0.168429368	0.905262039
DIRAS2	-0.168170667	0.919409523
DNAH14	-0.168156193	0.775842274
ABLIM2	-0.168076693	0.867628275
HACE1	-0.168062937	0.900401187
RHOJ	-0.16802076	0.941701504
NUCKS1	0.168004457	0.898346316
ZNF708	-0.167980853	0.886822411
CWC22	0.167938071	0.913540531
STRBP	0.167830384	0.765207588
CYTH3	0.167784036	0.886822411
TAF15	0.167783322	0.886121682
LOC150622	-0.167767714	0.92929992
ESF1	0.167755708	0.829255415
LONRF1	-0.167683508	0.928920501
ZIM2	-0.16766946	0.890193192
FXR1	0.167651148	0.912415041
ZNF506	-0.167519759	0.877730526
L3MBTL3	-0.167483285	0.912415041

MYEF2	0.167409669	0.877730526
IQCK	0.167247138	0.87558981
UTY	0.167174397	0.944194957
PPP3CA	0.16711241	0.742736721
LYRM2	-0.167098007	0.918396705
OLFM1	0.166818464	0.901859902
OSBPL8	-0.16678128	0.857360627
ATP8A2	0.166701106	0.665635253
AACS	-0.166614545	0.907326731
RNGTT	-0.166610106	0.768447793
ZFAND3	0.16637078	0.721760094
EPS15L1	0.166276629	0.895807632
ELAVL3	0.166250671	0.83851462
LPGAT1	0.16602797	0.879983041
RBCK1	0.166026468	0.896498046
SEMA6D	0.166007787	0.920835926
POM121	0.165965375	0.890373571
MAP4K3	0.165870494	0.753200728
ULK2	0.165815236	0.846164113
ANKIB1	0.165750075	0.665182235
MYO1D	-0.165684169	0.739182737
SPTBN1	0.165667677	0.766875769
AKAP12	0.165649878	0.909446504
MAGED1	0.165571621	0.863704345
ZZEF1	-0.165429082	0.85941116
HNRNPA2B1	-0.165416244	0.92181255
PXDNL	-0.165399125	0.882777845
CNOT4	-0.165359474	0.87406896
UBAP2	0.165329703	0.900401187
ZNF335	-0.165279063	0.882777845
DLEU7	0.16498254	0.849917298
IMMP1L	0.164968527	0.912415041
YME1L1	0.164929624	0.904984772
DYRK1A	0.164860221	0.867535376
CDKAL1	0.164611644	0.689522486
FRY	0.164551324	0.785665968
C20orf132	-0.164505044	0.826213286
CD8B	-0.164501156	0.933054351
TET1	-0.164398755	0.895292424
CDRT4	0.164368452	0.906931745
ATG5	-0.164353477	0.87558981
TMED8	0.16423099	0.901846971
BRWD3	0.163970141	0.842585832
ATP5F1	-0.163927548	0.897248392

PRKDC	-0.163914287	0.846164113
PRDM10	0.163909052	0.886822411
ATP9B	0.163577816	0.794824152
SLC4A4	-0.163544086	0.863389309
C1orf183	-0.163368421	0.867628275
ZNF510	0.163206378	0.87558981
SMAP1	0.163140898	0.859311336
USH2A	-0.163086404	0.87558981
BCAP29	0.162988212	0.87558981
CACNA1I	0.162987408	0.897037085
SGPP2	-0.162875437	0.886822411
C7orf10	-0.162510484	0.87898606
TTC15	-0.162213904	0.900401187
RAB3GAP1	-0.162176475	0.863389309
CEP63	-0.162150484	0.900436837
TMEM135	-0.162116814	0.767223194
SLC24A3	0.162098687	0.769497141
DPP10	-0.161939775	0.79998421
NAA15	0.161888236	0.907326731
DENND1B	0.161862711	0.874090514
ZMYM4	-0.161815563	0.743306051
MANBA	-0.16179862	0.898346316
TAX1BP1	0.161795158	0.893652964
RUSC2	-0.16158393	0.883755894
ZNF208	-0.161202765	0.932396042
EIF2AK2	-0.161175229	0.87884972
ATCAY	0.161105229	0.907326731
DNAH10	-0.161009669	0.92181255
ALS2CR11	-0.160963529	0.888339226
ZNF609	-0.160863941	0.814605352
TSNAX-DISC1	-0.160863527	0.829255415
PEG3	-0.160854829	0.897248392
PCNX	-0.160751385	0.743637226
KIAA0586	-0.160674844	0.886822411
GNPNAT1	0.16064277	0.929229827
TTLL1	-0.160613745	0.932396042
ATXN7L1	-0.160527237	0.763694956
PPP2R2B	0.160505744	0.660727573
ZNF714	0.160501565	0.890373571
DMD	0.160500935	0.666819087
RFX3	0.160375239	0.795054372
APBA2	0.160255715	0.878636121
NCAM2	0.160198056	0.665635253
TSPYL4	0.160194392	0.926608126

OGG1	0.160067109	0.900401187
SHISA6	0.160011444	0.76595106
SPTBN4	0.159887824	0.854941787
DTNB	0.159756361	0.716952451
IL34	0.159707066	0.919543065
ITGA7	-0.159519112	0.895057828
DLGAP1	0.1594853	0.84163621
BCL2L13	-0.159463102	0.911740599
COL4A6	-0.159369955	0.944194957
KTN1	0.15904545	0.87558981
SDC4	-0.159031375	0.894936358
CDK14	0.158968199	0.760192892
LGI1	0.158664738	0.877273789
LOC654342	0.158327763	0.886822411
C9orf25	-0.158321443	0.933654462
KIAA1370	-0.158253699	0.897290608
UBE3C	0.15824225	0.871574961
RNPC3	-0.1582361	0.901859902
LRRC4B	-0.158230011	0.907779136
GOPC	0.158226319	0.924221211
ZSCAN30	0.158113521	0.895807632
SCP2	-0.157924813	0.882777845
KCNK2	0.157691073	0.891633883
ZNF626	0.157689292	0.900401187
PRICKLE1	-0.157632405	0.886822411
TMEM164	-0.15755132	0.907779136
LIN9	-0.157448244	0.927848005
NUP210	0.157349134	0.938277634
WWP1	0.157108039	0.87558981
N4BP2L1	-0.157061949	0.879983041
LCA5	-0.156697885	0.907779136
B4GALT6	0.156680414	0.923649177
SCG3	0.156626986	0.88787435
ATP8B1	0.15643778	0.895057828
RAPGEF4	-0.156348991	0.773835293
GEMIN8	0.156314816	0.908173235
ATP7B	0.15613871	0.869723693
ARMCX1	0.156120373	0.913540531
IPCEF1	-0.156023507	0.92181255
ADK	-0.155826273	0.81630481
MPZL1	-0.155809813	0.924494066
GTF2F2	0.155673267	0.882777845
ABI1	0.155475318	0.81567336
ZNF461	0.155388849	0.920442932

NEO1	-0.155316438	0.739874665
SPOCK2	-0.155257847	0.917611589
SIAH3	-0.155230781	0.919543065
FAF2	-0.155216341	0.927848005
MACROD1	-0.15511986	0.920442932
NUAK1	0.154971871	0.878269474
SLC22A23	-0.154911761	0.907326731
AKAP10	0.154897179	0.867628275
SPATS2	0.154839576	0.85941116
AKT3	0.154837464	0.67944934
SNX9	-0.154815476	0.917611589
CCDC79	-0.154757601	0.933054351
PLCXD2	-0.154756017	0.939144732
NPEPL1	0.154733865	0.906627604
RDX	-0.154536199	0.918396705
AFG3L1	-0.154488542	0.867642707
SRSF2IP	0.154469511	0.87558981
IL4I1	0.15434608	0.903724873
SRGAP3	-0.154303255	0.797801615
PACSIN2	0.154000369	0.895292424
MGMT	-0.15393856	0.885154069
UBE2E1	0.153859101	0.919409523
DENND1A	0.153821374	0.692609596
BAG4	0.15379529	0.933054351
PCDH15	0.153681198	0.642905679
DDX46	0.153464145	0.915078054
TRUB2	0.153417943	0.924221211
LOC100130987	-0.153354603	0.900401187
MICAL3	0.153351157	0.86189419
CNTNAP5	0.153326763	0.710024269
CBARA1	0.153316832	0.815375207
ATP6V0A1	-0.153197865	0.922259611
EIF4E3	0.153058753	0.913007463
RAB6A	0.152932166	0.870407538
LOC100188947	-0.15277233	0.913607302
TTC26	0.152761952	0.929094623
ATM	0.152641101	0.867535376
PRMT3	0.152440364	0.92181255
GIT2	0.152359875	0.907779136
NPTXR	0.152330294	0.890373571
PCDHAC1	-0.152132326	0.895292424
PAQR7	0.152099503	0.944151594
SH3GL3	0.152075093	0.757591062
AMPH	0.152024823	0.834841222

JAKMIP1	0.152018481	0.809823177
ITFG1	-0.151860691	0.846912594
TRIM13	-0.151853563	0.92929992
STX12	-0.15177858	0.92929992
TEX9	-0.151703798	0.859086214
PRH1	-0.151642388	0.679210596
SLC36A4	-0.151524017	0.924221211
RPGRIP1L	-0.151427338	0.907779136
PSMD5	0.151423052	0.925113775
EEF1DP3	0.151417105	0.912046651
AVL9	0.151400346	0.879885284
B3GALNT2	-0.151333986	0.907779136
TSPAN7	0.151287159	0.917611589
IPP	0.151272582	0.899913226
PLCL2	-0.151262936	0.855693115
DIRC3	-0.151199397	0.784690569
SPATA1	-0.151183293	0.920610435
SPATA2	-0.151128878	0.824681514
TSPYL1	0.151022286	0.919262591
PTPN13	-0.151005121	0.866674601
RAB30	-0.151002466	0.925113775
ZNF518A	0.150985699	0.876178359
DTD1	-0.150715566	0.912415041
HORMAD2	0.150602004	0.939400133
WFDC9	-0.150557301	0.939242778
YLPM1	0.150553547	0.918396705
HM13	-0.150496816	0.907779136
KCNAB1	-0.150489757	0.878833959
FBXO28	-0.150432299	0.895292424
DCLK1	0.150416114	0.792375054
KHDRBS2	0.150363409	0.797224471
AMPD3	-0.150080567	0.925113775
COG6	0.149967324	0.861851422
RPAP2	-0.149938347	0.886822411
LATS1	-0.149828868	0.901016707
RAB6B	0.149716024	0.907779136
RNF180	-0.149695271	0.841749492
VAMP4	-0.149628257	0.946648219
LOC348751	-0.149607002	0.900401187
C15orf27	-0.149447952	0.898346316
BRD1	0.149363937	0.912288744
CDH9	-0.149272899	0.863981934
LCOR	0.149198128	0.846164113
SKAP1	0.149156727	0.886822411

PAK7	-0.149095689	0.831022546
TRIP11	0.149069885	0.886822411
LRRC16A	-0.148864996	0.869162179
NFIB	0.148861423	0.846828469
AGAP1	0.148849076	0.769497141
P2RX7	0.148845222	0.942355631
SLC35F3	0.148788996	0.809823177
NFX1	0.147923315	0.846164113
ATG4C	0.147874727	0.927453695
YWHAE	0.147868604	0.886822411
TOM1L2	0.147807789	0.849968658
ZNF28	-0.147791661	0.944194957
PHLDB2	-0.147696333	0.907779136
CLASP1	0.147624961	0.817274457
LOC284578	-0.147506475	0.930741099
ARFGEF2	-0.147406244	0.846164113
PAG1	0.147399826	0.87558981
KIAA0232	-0.147352404	0.845446387
ZER1	-0.147337228	0.92929992
METAP2	-0.147309313	0.897248392
SAMD4A	0.147267735	0.878636121
GOLGA8A	0.147160532	0.940760032
GLCE	0.147135024	0.857360627
UGP2	0.147121415	0.915078054
CHCHD3	0.14711537	0.846912594
PIGS	0.147079501	0.901016707
GOLGA8B	-0.147044855	0.954547944
CYB5B	0.14703995	0.903847563
RNF130	0.146912978	0.831205091
ZNF343	-0.146883993	0.924221211
PRR4	-0.14687271	0.692885129
UBE2G2	0.14668973	0.939400133
KCNK1	0.146689465	0.81630481
MOBKL2B	-0.146538406	0.883788562
GFPT1	0.146430933	0.917611589
PLCL1	0.146334309	0.767223194
SUV420H1	0.146271351	0.881392042
ZNF559	0.146228929	0.917611589
HPS4	0.146167582	0.9379834
KIAA1468	0.146082875	0.867628275
CSTF3	0.146076582	0.934206931
MYO6	-0.145956727	0.882777845
DENND4C	0.145930404	0.900109963
GALNTL4	0.145775352	0.806254482

TMED7-TICAM2	0.145647622	0.921882607
NAA38	-0.145620917	0.942565793
PLEKHG1	-0.14548952	0.900401187
PLA2G4C	-0.145485403	0.894489843
MORN1	0.145099808	0.911680233
TRERF1	0.145027594	0.876326689
ZNF117	-0.144962646	0.942565793
NBEA	0.144940326	0.790847726
KLHL29	-0.144929591	0.769526203
UBQLN1	0.144819133	0.882777845
ZNF587	-0.14474399	0.908422867
AFAP1	-0.144586272	0.936203573
EFHA1	-0.144585455	0.863704345
LNX1	0.144573804	0.888745066
WNK4	0.144399679	0.939400133
C9orf4	0.144367995	0.865028773
GUCY1B3	-0.144132168	0.944194957
VPS24	0.143968123	0.852672836
MSI2	-0.143952906	0.886822411
HDX	-0.143920036	0.854941787
TTC23L	-0.143892867	0.895292424
NFAT5	-0.14387851	0.822277527
GPATCH1	0.143828153	0.953886047
RNF111	-0.143827354	0.895057828
NPAT	-0.14343034	0.933054351
DAAM1	0.143392805	0.883755894
TMEFF2	0.143282158	0.763512391
REPS1	0.143207978	0.843568343
DIAPH3	0.143082614	0.907779136
SNTG1	0.143041206	0.842585832
BCL11A	-0.143019309	0.857360627
NXPH1	-0.142966009	0.908422867
GAB2	0.142908617	0.909446504
ST3GAL1	0.14281806	0.92929992
TBC1D19	0.142585898	0.897037085
DCAF10	0.142458459	0.857360627
SEPT7	-0.142355976	0.929094623
NPAS3	0.142105234	0.814605352
GTDC1	0.142086511	0.69368675
CA10	0.142066494	0.765793784
SRSF8	0.142002945	0.917611589
UNKL	-0.141961172	0.949004166
RANBP9	-0.141936109	0.883274428
XPNPEP1	0.141924955	0.933438912

ANKS1B	0.141919472	0.715184974
HERPUD2	0.141795532	0.937824226
KLHL32	0.141770114	0.85941116
TFCP2	-0.141746563	0.912415041
CNTN1	-0.141610765	0.763779655
L2HGDH	-0.14158366	0.886822411
SPAG9	0.141523565	0.887670299
RASA3	-0.14152234	0.939144732
MCTS1	0.141508842	0.92181255
KCTD13	0.141427141	0.863756803
ARHGEF6	-0.141397336	0.922749914
CUL1	0.141314296	0.896538631
MIPEP	0.141264983	0.846828469
EML4	0.141250957	0.906002419
ERLIN1	-0.141027859	0.933054351
C14orf179	0.14090688	0.895057828
HELQ	0.140885864	0.944194957
ERMP1	-0.140736916	0.94622776
PIGK	0.140721113	0.893652964
MBP	-0.140697784	0.907607042
ATG14	-0.140695584	0.877730526
LOC642924	-0.140567636	0.896548117
AKAP9	-0.140464092	0.890373571
STAG1	-0.140447324	0.84595212
GNG2	-0.140092819	0.895057828
EPS15	-0.140047234	0.893791456
ARNT	-0.139884494	0.907326731
CLCN4	-0.139877116	0.938260836
IPPK	-0.13961539	0.900401187
SLIT1	0.139608704	0.867628275
ZRANB1	0.139559429	0.917679156
TRPC4	0.139422248	0.893652964
TXNDC6	-0.139197812	0.943185221
APBB2	0.139137234	0.849859184
YAF2	0.139092807	0.81630481
C15orf59	0.13908052	0.948364286
CPEB4	0.139075403	0.871505777
GLRB	-0.138958238	0.900401187
STX8	-0.138866099	0.877730526
L3MBTL	-0.138861105	0.867628275
GMDS	0.138845455	0.817274457
EML2	-0.138828435	0.933054351
HERC3	-0.138725562	0.893652964
KIAA0240	-0.138662646	0.942565793

TMEM132D	-0.138661092	0.857360627
TP53BP1	0.138546578	0.877730526
USP47	0.138390522	0.858223421
WDR7	-0.138381439	0.782101175
TYR	-0.138348598	0.949004166
WDR52	-0.138208474	0.928920501
PEX3	0.137902468	0.937112484
MIR3134	0.137833862	0.85941116
APPL1	-0.137789625	0.939406327
SPA17	0.137740678	0.944194957
CSNK1E	-0.137732602	0.927963732
RAI2	0.137694501	0.959708103
EMCN	-0.137525869	0.944194957
TRIM44	0.137502643	0.87558981
ZNF790	-0.13735502	0.92929992
PARM1	0.137304798	0.943182207
MNAT1	0.137092803	0.840320668
C14orf135	0.136976755	0.939144732
RAB1A	-0.136913325	0.907305752
ZYG11B	-0.136856166	0.915078054
SFRS12	0.136802878	0.923750288
HIVEP1	0.136795225	0.944194957
NOVA1	0.136755103	0.883755894
LOC220980	0.136699566	0.909446504
ANO5	0.136445617	0.886822411
NCAM1	0.136364897	0.797687825
TCEAL1	-0.136297422	0.907929552
SLC25A26	0.136244334	0.907779136
EIF2AK4	-0.136233432	0.904542807
STT3B	0.136155488	0.933054351
LTBP1	-0.1359548	0.927848005
SMOC1	-0.135950127	0.944194957
DSCAM	0.135814409	0.78103569
GRIA1	0.135751883	0.706977551
C4orf34	0.135734387	0.863389309
MYOM1	0.135715716	0.918396705
PLAGL1	0.13557657	0.862500621
C1orf151	-0.135489796	0.944194957
PTGIS	0.13534282	0.863756803
ZCCHC2	0.135338621	0.933265174
TRDN	0.135311197	0.93959162
WDFY3	0.135310959	0.831109321
UPF2	-0.135266168	0.924494066
GLMN	0.135160146	0.907779136

RCOR1	-0.135155177	0.93732271
DPY19L2	0.135111449	0.912448092
TMC7	-0.135057264	0.944194957
SLC20A2	-0.135052222	0.907779136
CCDC85A	-0.13501831	0.877668298
CCDC144A	-0.134864336	0.944194957
DCLK2	-0.13472492	0.890373571
KPNA5	-0.134559343	0.944194957
ZNF277	0.134528076	0.904984772
VPS13A	0.134468447	0.861851422
KIAA2022	-0.134464725	0.907083893
CAPN2	-0.134452181	0.947109537
C1orf93	0.134450529	0.944194957
ALS2	-0.134390791	0.902489967
KDM1B	-0.134253136	0.938277634
TUBB	-0.13421914	0.930628999
EXOC2	0.134098454	0.857032817
ABHD3	-0.134091468	0.948510199
NAP1L1	-0.134061262	0.901016707
CORO2B	-0.134036554	0.912288744
FAM120A	-0.133958252	0.943323597
CHD9	0.133866952	0.756950478
LOC642345	-0.133726016	0.913540531
FLVCR1	-0.133713134	0.941413643
ABLIM1	-0.133673346	0.846164113
IGSF21	-0.133636348	0.907779136
LRRC8C	0.133613476	0.948960277
STK3	-0.133584733	0.907305752
VPS45	-0.133554058	0.908173235
CDC37L1	0.133395738	0.9498477
MMD	-0.133380761	0.92929992
PDE4DIP	0.133354521	0.821008578
ASXL1	-0.133325867	0.896498046
THOC2	-0.133108437	0.883788562
SLC6A15	0.132958709	0.944194957
PAM	-0.132777054	0.818213475
CHM	-0.132642193	0.863389309
AIG1	0.132547107	0.842370734
SYT1	0.132542632	0.704398473
HCG18	-0.132356499	0.903252434
TBC1D9	0.13232488	0.935838024
CNKSR2	0.132308404	0.863389309
AP3B2	0.132307844	0.933054351
COG3	0.132287607	0.943185221

KIAA1530	0.132236036	0.949004166
SRGAP2P2	0.132104835	0.944194957
PPP1R12B	0.13171221	0.886822411
LRFN2	-0.131640185	0.915078054
NVL	0.131609985	0.918396705
ZNF283	0.131474417	0.941701504
ZNF238	0.131451959	0.920145374
C9orf68	0.131334575	0.946648219
IRAK3	0.131188243	0.95197154
SLIT2	0.130993291	0.917611589
PIAS1	0.130980301	0.840200163
PIP5K1B	-0.130810421	0.863389309
RAD54B	-0.130773929	0.944194957
FLRT2	0.130721277	0.895807632
C20orf26	-0.130705271	0.886822411
C11orf49	-0.130653168	0.836364976
NRF1	-0.130652946	0.829255415
NSL1	0.130624569	0.917611589
MAGEC3	0.130548636	0.94664618
MBD2	0.130331042	0.92929992
MAP1B	-0.130317241	0.886822411
SLC23A2	0.130148909	0.900401187
VPS33A	-0.130148882	0.939400133
CSRNP3	0.130029182	0.824681514
TRAP1	-0.129984569	0.944194957
MPP5	0.12995092	0.927848005
RABEP1	-0.129806995	0.881392042
JPH4	-0.129645434	0.923540816
PIAS2	0.129495686	0.88787435
LOC646329	0.12919941	0.938277634
C14orf143	-0.129157655	0.936458821
NME5	-0.129082638	0.900677189
C2orf61	0.128976485	0.944194957
TMC1	-0.128955058	0.924335082
SSH2	-0.128950515	0.890373571
NUP98	-0.128948188	0.895057828
PTPN9	-0.128829461	0.943185221
RNF185	-0.128795792	0.953886047
PRELID1	-0.128725876	0.953886047
RFX7	-0.128625483	0.869162179
LRP12	-0.128577421	0.927848005
SH3GLB1	-0.12854564	0.908422867
ZNF565	0.128505417	0.94664618
ZDHHC21	-0.128368155	0.908422867

GLTSCR1	-0.128058977	0.943185221
SRPK2	0.128039277	0.865636376
ZNF235	-0.127609967	0.941753493
DNAJC19	-0.127608729	0.948050406
DDX50	-0.127564645	0.920442932
ABCD3	-0.127503082	0.933054351
RNF144A	-0.127487242	0.906301805
SGK494	0.127482928	0.897037085
ETNK1	0.127432282	0.942565793
DSE	0.127363611	0.918396705
ZCCHC16	-0.127345338	0.92929992
COG7	-0.127258537	0.909446504
AGPS	-0.12724103	0.924225123
CLTA	0.126769478	0.942565793
YWHAZ	0.126571514	0.912288744
DOCK11	0.126568386	0.936264997
MAD1L1	-0.126391306	0.917611589
SSTR2	0.126386931	0.953003648
CYLD	0.126354882	0.940886411
TNIK	0.126253674	0.849859184
CDH13	0.126238128	0.876720394
LRRC20	0.126229988	0.942565793
NRCAM	0.126216161	0.81630481
WWOX	-0.126084448	0.8222211
SCPEP1	-0.126050443	0.877730526
PRKAG1	0.126041401	0.948729935
PIN4	0.126033999	0.953886047
FAM50B	-0.125913747	0.939144732
MTUS2	-0.125809812	0.85941116
HNRNPUL1	-0.125408857	0.935838024
RABGAP1	-0.125357937	0.831022546
LYRM7	0.125289891	0.915325534
RALYL	0.125280336	0.818213475
FAM149B1	0.125164623	0.918616956
CACNA1E	0.125051651	0.783398964
ZNF652	-0.125022809	0.925113775
CCDC66	0.125012303	0.95197154
PPIL6	-0.12495092	0.936574459
MAP7D2	-0.124717618	0.944194957
TANK	-0.124690885	0.936574459
TBCE	0.124613253	0.949004166
KCTD16	0.124575421	0.778145609
DMXL2	0.124548678	0.901312493
MEF2A	0.124452253	0.903634856

HIBADH	-0.124435311	0.907779136
GDPD4	0.124348121	0.953886047
CRIM1	0.124289608	0.944194957
ZNF821	-0.124253568	0.95197154
FCHO2	-0.124209871	0.944194957
DDN	-0.124091856	0.897248392
ADARB2	-0.12393942	0.925328944
MAPK10	0.123937422	0.747668378
RALA	-0.123744981	0.886822411
PKM2	0.123677891	0.944194957
KLHL20	-0.123532327	0.894936358
TBC1D14	-0.123480296	0.940886411
ZNF736	0.123469795	0.944194957
ERBB2IP	-0.123415103	0.929184805
SLC1A2	0.123285386	0.878636121
PHKA1	-0.123275583	0.932038818
BTBD10	0.123274298	0.921252863
HLTF	-0.123254202	0.946648219
AGFG1	-0.12324097	0.874475627
DGKZ	-0.123233155	0.944194957
RIN3	0.123119064	0.947109537
LRCH1	0.122827749	0.893653466
ANXA4	-0.122823914	0.944194957
CHN2	-0.122789846	0.930916738
RGS7BP	-0.12275925	0.842370734
GRB10	-0.122726159	0.932358474
LOC654433	0.122659986	0.954547944
IPO11	-0.122582924	0.917611589
NFYB	-0.122570434	0.946648219
UIMC1	-0.122313983	0.900109963
GALNT13	0.122264404	0.816001721
WDR33	0.122168281	0.855978901
SCN2B	0.122113456	0.944194957
JARID2	-0.122108019	0.938277634
SLC10A7	0.122045852	0.917611589
C11orf73	-0.122037253	0.944194957
GSTM3	-0.121860035	0.944194957
NUP133	0.121835844	0.907779136
MAP6	0.121695654	0.937824226
FOXO1	0.121683805	0.924221211
FLJ10038	-0.121653676	0.946648219
MED27	0.121516732	0.88787435
NR1D2	0.121515761	0.945027722
FAM200B	0.121483074	0.946648219

C12orf64	-0.121438007	0.944194957
RB1	0.121410487	0.891905291
PPIP5K2	0.121313459	0.944194957
JAZF1	0.121291229	0.807631501
C17orf108	0.121218191	0.933054351
SLC25A16	-0.121133601	0.927453695
C15orf40	0.121103921	0.944194957
USP43	-0.121048032	0.949004166
NCKAP1	0.121013678	0.900914431
SNRPN	0.120872492	0.857360627
SCNN1G	-0.120789887	0.959711098
URGCP	0.120737981	0.900401187
FDX1	0.120672221	0.946636505
KIAA1143	-0.120667088	0.95058928
INPP5D	-0.120588515	0.944194957
CLVS1	-0.120553404	0.886822411
MYT1L	0.12043804	0.863389309
RYR2	0.12021821	0.767136394
G3BP1	-0.120144315	0.94664618
ZRANB3	-0.120064032	0.886822411
ZNF142	-0.120036488	0.948050406
PTPRK	0.119867639	0.846912594
EXT2	0.119379811	0.845446387
TRIM2	0.119249988	0.886822411
PTPLAD1	0.119113294	0.901016707
SYNE1	0.119076474	0.675837558
TMEM8B	0.119072733	0.948050406
GABRB2	0.118922777	0.840200163
ADCY8	-0.118772862	0.937112484
LRRC7	0.118736251	0.791601634
ZNF561	-0.118725391	0.955961549
SLC6A11	0.118608587	0.935959302
TG	0.11841191	0.9498477
MTHFR	-0.118344665	0.946648219
DNAJC1	0.118155458	0.90041451
SGCD	-0.118114304	0.888745066
USP42	0.118099445	0.944194957
CDC42BPA	0.117987039	0.846164113
METTL8	0.117956293	0.933054351
TCEAL7	0.117938471	0.954547944
TC2N	-0.117912074	0.953886047
SNED1	0.117895275	0.867628275
SNAP91	0.117863368	0.845446387
TAPT1	0.117815055	0.953850137

EPM2AIP1	0.117775409	0.938277634
KATNAL1	-0.117772255	0.919950264
NRIP1	0.117769556	0.937744165
PUM1	-0.117744533	0.911175775
SLC38A2	-0.11771234	0.954547944
SPOCK1	-0.117670954	0.882777845
FARP1	-0.117520622	0.938277634
ZNF592	-0.117481663	0.930916738
TEX11	-0.117429703	0.932396042
FBXO33	-0.117392435	0.944194957
ZNF544	0.117377112	0.895806501
C2orf46	-0.117368944	0.917611589
SH3PXD2A	-0.117257036	0.92929992
SFMBT1	0.117254778	0.934206931
MID1	-0.11718276	0.913226601
APLF	0.117096762	0.939406327
ZNF429	-0.116982406	0.94664618
PCDP1	-0.116936261	0.895807632
XRCC4	-0.116884798	0.927453695
C19orf12	0.116519437	0.895292424
ATR	-0.116500524	0.91913785
SMARCAD1	0.116416015	0.886822411
ABCB5	-0.116377428	0.955918263
NXPH2	-0.116318118	0.946648219
ALKBH8	0.116152869	0.937140571
UHRF1BP1	0.116133184	0.939925569
STAU1	0.116078323	0.895807632
LMTK3	0.11595995	0.944194957
POLR1B	0.115885543	0.942099283
BPTF	-0.11583056	0.909690458
ALS2CR8	-0.115777333	0.930231228
CHEK1	-0.11576161	0.954547944
NRD1	-0.11565483	0.917679156
RNF14	-0.11555293	0.944194957
ZNF175	-0.115524578	0.954547944
POGZ	0.115504411	0.857360627
PPP1R14C	0.115456205	0.920442932
JUND	-0.115359604	0.940760032
PCDH9	0.11507674	0.791508081
PIK3R1	0.115024539	0.944194957
KLHDC10	-0.115015259	0.92929992
EML5	0.114896104	0.840200163
LOC678655	-0.11479615	0.940760032
ACTN1	-0.114755085	0.948050406

HABP4	0.1146664	0.936458821
LOC646851	-0.114657605	0.925113775
EFCAB4B	0.114612219	0.953886047
SYMPK	-0.114490467	0.944194957
C10orf76	0.11445528	0.913540531
MACROD2	0.114365292	0.769497141
RIN2	0.114300083	0.933054351
COL5A1	0.114067505	0.958508704
ZNF329	-0.114067095	0.947109537
ACOT8	0.114024167	0.91913785
CDK13	-0.114007753	0.911680233
REV3L	-0.113935408	0.856786561
UBE2R2	-0.113805037	0.944194957
ZXDC	0.113796127	0.935915101
SERBP1	-0.113728072	0.927848005
MTHFD1	-0.113716431	0.946648219
SLC38A6	0.113619085	0.917611589
DMXL1	-0.113528872	0.898346316
C20orf194	0.113385543	0.887670299
BASP1	0.113381738	0.932530077
C1orf173	-0.113263289	0.941753493
SKI	-0.113218886	0.954547944
MBOAT1	-0.113127094	0.961050718
BRCA2	-0.113106334	0.95197154
TSNARE1	-0.11305558	0.94818129
MCTP1	0.113031434	0.808096449
LOC651250	0.112963872	0.947109537
GNAL	0.112926837	0.87406896
MEGF11	-0.112849528	0.926140433
CDK12	-0.112832877	0.943185221
CNRIP1	0.112554791	0.930231228
TNRC6C	-0.112450561	0.920669889
ZNF876P	0.111819447	0.932530077
SPTLC2	0.111796563	0.942099283
DLGAP2	-0.111728613	0.93489955
C10orf28	-0.111719077	0.940760032
PTBP2	0.111584364	0.87558981
TBC1D8	-0.111581515	0.915078054
GLYR1	0.111449154	0.94664618
TNFRSF21	0.111410342	0.92968159
SMURF2	-0.111349137	0.930628999
MAPKBP1	0.111339447	0.899799613
RAB8B	0.11132288	0.9483239
PL-5283	-0.11125409	0.954547944

LRRC37B2	-0.111220403	0.954547944
VAV3	-0.111211633	0.940837879
RBL2	-0.111112425	0.943622135
MIB1	-0.11110007	0.907779136
KCNQ5	0.111086889	0.840320668
PDSS2	-0.111005011	0.897037085
ANKRD43	0.1109252	0.94664618
GRM3	0.110894865	0.944194957
TAGLN3	-0.110756829	0.94751091
OR2L13	0.110698649	0.912046651
CCDC147	-0.110574059	0.954547944
MSRA	0.110402286	0.845446387
NBN	-0.110399696	0.929924756
PIBF1	-0.110353183	0.857360627
EPB41L3	-0.110196605	0.9498477
KCTD3	0.110167373	0.948510199
FAM190A	0.110144364	0.751825187
PKN2	0.109966383	0.898126141
GABRA2	-0.109942457	0.895292424
CACNG2	0.109858246	0.929671761
ZNF682	-0.109813905	0.953886047
JMJD1C	0.109801795	0.857360627
ITPKB	-0.109775547	0.959402585
ILDR2	0.109705454	0.961050718
ODF2L	-0.109694633	0.92929992
PRKCI	0.10941552	0.93489955
MTMR12	0.109400829	0.944194957
HIPK3	-0.109377116	0.936572685
GPR176	0.109332385	0.95197154
PPP1R3F	-0.109206226	0.953886047
DSN1	-0.109175344	0.939400133
FSTL4	0.109171803	0.946636505
DCUN1D5	-0.109159321	0.935838024
ODZ2	0.108953486	0.814605352
NRXN1	0.108743534	0.804374539
ADCYAP1R1	-0.108699266	0.953886047
INPP4A	0.108639717	0.890373571
PSMB7	0.108555205	0.944194957
LOC400960	-0.108540176	0.888755423
LOC643542	0.108515087	0.941701504
ZNF608	0.108492151	0.949502088
IP6K1	0.108485995	0.944194957
AUTS2	0.108477675	0.773835293
GPR107	0.108477484	0.946648219

PCCA	-0.108440892	0.876178359
ATRNL1	0.108333853	0.773835293
IFT27	0.108010221	0.962760831
CLINT1	0.10790684	0.946648219
MTDH	0.107896137	0.897248392
RIMS1	0.107735261	0.855508911
UBE2D3	0.107644901	0.930628999
EXPH5	-0.107565461	0.948272923
C10orf18	0.107487088	0.919543065
BCL2	-0.107482276	0.946636505
ASAH1	-0.107472684	0.939144732
TMEM170B	0.107402173	0.957717687
SPOPL	0.107341784	0.933265174
OLA1	0.107204404	0.872668292
MAP3K2	-0.107191273	0.946648219
HERC1	0.107043565	0.863389309
PACRG	-0.107005678	0.882777845
KIAA2018	-0.106984406	0.954547944
FNDC3B	0.106909135	0.900436837
C8orf42	0.106894764	0.940760032
HLCS	-0.10674262	0.857571156
MBD5	0.106562832	0.824681514
NELL2	0.106378633	0.882777845
RHOBTB1	0.106230599	0.953886047
ZFR2	-0.106198691	0.946648219
DHX40	-0.106197491	0.944194957
MTMR1	-0.106187772	0.916932721
FRMD4B	-0.106098736	0.911740599
ASB3	0.105983704	0.863704345
TRIM35	0.105939857	0.949004166
APPL2	-0.105933757	0.95722057
CNTN6	0.105873075	0.948510199
ALDH7A1	-0.105853531	0.948050406
TRAK1	-0.105698458	0.941753493
PBX3	-0.10561284	0.946648219
MIER1	0.105499836	0.939406327
LRP6	-0.105477673	0.943182207
SLC9A9	0.105391769	0.886822411
LRRN2	0.105299622	0.92181255
FUS	0.105155482	0.948050406
FOXP1	0.10513151	0.890373571
MAPRE1	0.10511806	0.946636505
LIMS1	-0.105043079	0.946648219
NCEH1	0.104772055	0.944194957

TMTC1	-0.104756136	0.890373571
TCF7L1	-0.104668077	0.948226251
GSTO2	0.104657965	0.936203573
MON2	0.104640587	0.927848005
HDAC8	-0.104486082	0.876720394
RPS6KA6	-0.104468038	0.944194957
PRKAA2	0.104438535	0.945851072
CYB5D1	-0.104294264	0.949004166
ANKRD17	0.104203964	0.857146986
FANCC	-0.104174007	0.944194957
HSPA9	0.104122437	0.944746961
TTL	-0.103929904	0.953886047
NCAPG2	-0.103911018	0.944194957
MED4	-0.103909775	0.957717687
USP9Y	-0.103894543	0.961050718
GAP43	-0.103860808	0.944194957
RUFY3	-0.103828117	0.896528149
SEC61A2	0.103825769	0.959711098
RIC3	-0.10380425	0.935165946
BAGE	0.103801925	0.961968499
NLGN4Y	0.103655883	0.961470873
ARID4A	0.103631504	0.953003648
CEP135	-0.103556004	0.949004166
STXBP4	-0.103466584	0.886822411
CIR1	-0.103397516	0.959708103
SMARCC2	-0.103293667	0.945222406
SRGAP1	-0.103284663	0.949004166
SEZ6L	-0.10311695	0.886822411
DCAKD	-0.102772178	0.948050406
SH3D19	0.102755645	0.948822684
ORC5L	-0.102572779	0.946648219
PPIL3	0.102563607	0.94692329
ARHGAP6	-0.102362904	0.898346316
JPH2	0.102248074	0.943622135
COPS8	0.102222828	0.947109537
ZHX3	-0.102182002	0.892923978
NAV3	-0.102152531	0.823162236
INTS7	-0.102087535	0.957102852
ARL15	0.102038558	0.842370734
GNB5	-0.101954285	0.951317204
MIR548D2	0.101852828	0.863981934
NKTR	0.101848351	0.942565793
N4BP2L2	0.101747143	0.846164113
BBX	0.101680282	0.944194957

NFKBIZ	-0.101254388	0.961968499
CTNNA2	0.101213543	0.769497141
MAVS	-0.101166824	0.954547944
ATP13A3	0.101111209	0.944194957
GFM2	-0.100842969	0.954547944
ZNF346	-0.100819258	0.944194957
AFF4	0.100802205	0.90805002
KHDRBS3	-0.100786923	0.954547944
UCHL5	-0.100786633	0.961050718
LYPLAL1	-0.100778948	0.944151594
FLJ31306	-0.100716821	0.886822411
S100PBP	-0.100589515	0.947109537
CAMTA1	-0.100362557	0.846164113
UBR1	0.100329686	0.940760032
PCSK5	0.100325237	0.908173235
NTRK3	0.100226018	0.876720394
SOX2OT	-0.100080965	0.936131152
CCBL2	-0.100028155	0.944194957
ZNF529	0.099925155	0.929924756
SLC4A8	0.099828898	0.939144732
PTPRS	0.099779078	0.944194957
ATRN	-0.0997152	0.927963732
UGGT1	-0.09967757	0.939144732
NAV2	0.099546454	0.87558981
NCOR1	0.099420144	0.902249635
IFI6	-0.099363556	0.954547944
MLXIP	0.099273987	0.953886047
MCCC1	-0.099238332	0.9498477
MAN2B2	-0.099043621	0.953643676
ADAMTS16	0.099001729	0.917611589
GDE1	0.098929849	0.948312067
BTN2A1	0.098884182	0.961050718
LPHN3	0.098773717	0.854105076
PIK3R3	0.098643541	0.95197154
SENP5	-0.098567132	0.937744165
RHOBTB3	0.098564736	0.946648219
C3orf23	0.098493591	0.947109537
KALRN	0.098491361	0.840200163
SEC23IP	0.098403374	0.955027939
KIAA0408	0.098366404	0.954547944
ADAM22	0.098344198	0.87558981
FRMD4A	0.0982903	0.878833959
ZNF33A	-0.098262949	0.907326731
C14orf159	-0.098214816	0.944194957

CLVS2	0.098200738	0.961050718
ZNF138	0.098182635	0.95197154
HSPA4	-0.098106758	0.954547944
SPG11	-0.098050304	0.930628999
DOCK4	0.098026136	0.845446387
EPHB1	0.097998554	0.936203573
TASP1	-0.097971252	0.907779136
SCMH1	-0.097950335	0.888385086
MTFMT	-0.09792152	0.939400133
AP4S1	-0.097876119	0.948312067
COX19	0.09767335	0.961050718
PPAPDC1A	0.097559841	0.961050718
SLC12A6	-0.097552787	0.944194957
CREB1	0.097291952	0.944194957
TRPC4AP	0.09728276	0.901859902
MZF1	-0.097129209	0.954547944
RYBP	0.097096967	0.951943121
DIAPH1	0.097071746	0.953886047
LGR6	0.096977104	0.9645372
SLC1A6	-0.096963874	0.951317204
TXNDC16	0.096829955	0.939925569
PPP1R12A	-0.096776998	0.915078054
GTF2H5	-0.096537453	0.959005288
GRK4	-0.09649003	0.948050406
UBR5	0.096448236	0.922974649
ATAD1	0.09643271	0.944194957
SLTM	-0.096368933	0.957622662
FBXO17	-0.096366392	0.944194957
CAP2	-0.096222134	0.933202502
TMEM209	0.096173445	0.962760831
CAST	0.096034573	0.958731287
CREB5	0.096018185	0.952641232
GABPB1	-0.096017367	0.944194957
NEGR1	0.096015898	0.861851422
UNC5C	0.09601303	0.8982287
NCK2	0.096008099	0.946648219
CCDC102B	-0.095991566	0.907779136
EHBP1	0.095909107	0.863389309
RUNX1T1	0.095886689	0.949004166
SORL1	0.09577193	0.938277634
FAM19A2	0.095685882	0.877730526
DTWD1	-0.095668086	0.953886047
TGFBRAP1	-0.095612131	0.961050718
SEZ6L2	-0.095415865	0.956777312

WDR45L	0.095323294	0.963660654
STK17B	-0.095297063	0.921333728
C15orf41	-0.095261635	0.944194957
CACNA1A	0.095251948	0.917679156
LMBRD1	-0.095235327	0.895292424
SEC14L5	-0.095206843	0.941651239
ICA1L	-0.095056121	0.907779136
XRN2	-0.094924191	0.949609566
NEBL	0.094869262	0.807637239
CIAO1	-0.094837787	0.909690458
TCF12	0.094705843	0.88406385
FAM69A	0.094668563	0.911175775
LOC100132891	0.09461962	0.919543065
GOLGA4	-0.09457588	0.924221211
FREM1	0.094271892	0.959402585
OTUD7A	-0.094250376	0.917611589
CPE	-0.094146142	0.908422867
ATF6	-0.09402109	0.944194957
ZNF131	0.094020036	0.955027939
CSMD2	-0.093942106	0.846912594
ASB18	-0.09391218	0.953886047
C6orf94	-0.093894184	0.953886047
KBTBD12	-0.093860972	0.958666561
GALNT11	0.093784453	0.936879315
NUP107	-0.093650476	0.961050718
WDR17	-0.093590571	0.946648219
ACSS3	-0.093412013	0.953886047
ELMOD1	0.093364048	0.906722759
TLN2	0.093285857	0.945027722
FBXO18	-0.093270369	0.954547944
SYNJ2	0.0932682	0.960062638
SLC7A6	-0.093208268	0.954547944
LCLAT1	-0.093201269	0.946648219
TRANK1	-0.093194153	0.953886047
AAK1	0.093178575	0.944194957
SRI	-0.093081936	0.9645372
NKAIN1	-0.093042706	0.949004166
PAPD5	-0.092962942	0.917611589
PTP4A2	-0.092863488	0.959748493
ATG4B	0.092776332	0.961050718
SH3KBP1	-0.09257362	0.92181255
ADCY3	-0.092544037	0.944194957
ECD	0.092402072	0.959708103
RASA2	0.092191598	0.900401187

KIAA1211	0.09213629	0.948050406
MAP2K4	0.092107079	0.947109537
BRCC3	0.091890967	0.958282713
POLK	-0.091824652	0.95197154
ELP4	-0.091805295	0.888745066
PPP3R1	0.091728116	0.897037085
LAMB1	-0.091723631	0.960571432
LRRC6	0.0916721	0.946648219
AK5	-0.091587939	0.883755894
XPO6	0.091385145	0.953886047
IRAK2	-0.091290452	0.9645372
SESN3	0.091263991	0.947109537
EIF3H	0.091188708	0.955750924
ASRGL1	-0.091171305	0.953512426
DSTYK	0.091015247	0.941341525
PID1	-0.090981046	0.935838024
CRTC3	0.090968786	0.945851072
RNF175	0.090854121	0.927963732
GAPVD1	-0.090782059	0.948050406
FGFR1OP2	-0.090751038	0.949004166
METTL10	0.090561807	0.954547944
NRXN3	0.090536264	0.848673759
MIA3	0.090530453	0.9645372
ZNHIT6	-0.090495468	0.961050718
RSPH1	-0.090372772	0.954547944
APBA1	0.090363587	0.92181255
ZCCHC12	0.090310205	0.958731287
NOLC1	0.090274216	0.954547944
RAP1GAP2	-0.090246922	0.933054351
INSIG2	0.090064767	0.953886047
ARL5A	-0.090026579	0.959708103
PHF2	-0.089788953	0.958146178
ACTR10	-0.089751086	0.954547944
AEBP2	-0.089647527	0.944194957
IQCB1	-0.08960424	0.913540531
RNF10	0.089409561	0.946648219
GHR	-0.089308954	0.953886047
CNTNAP4	-0.089300725	0.948364286
IDH1	-0.089234017	0.961328961
MARK4	0.089030513	0.954364434
YES1	0.088997185	0.961050718
CRB1	0.08877926	0.907887719
RPS6KA3	0.08874628	0.954547944
SYNPR	0.088662419	0.887670299

CCDC43	-0.08864691	0.964519792
SYCE1	-0.088592335	0.963660654
MPP6	0.088548219	0.933054351
VEZT	0.088543073	0.944194957
LAS1L	0.088470936	0.948729762
DPY19L4	-0.088460567	0.961050718
HK1	0.088459666	0.948510199
FAM120AOS	0.088383634	0.95982111
FBXO3	-0.088360902	0.960062638
CLEC16A	0.08831685	0.946563681
GTF3C2	-0.088308806	0.945914301
XIST	-0.088296165	0.971260724
KCNJ3	-0.088286539	0.939144732
PLEKHA9	-0.088170727	0.961050718
EIF5B	0.088149261	0.961050718
PHLPP2	0.088107874	0.944194957
ALCAM	0.088008121	0.927848005
SOS1	-0.087668577	0.878636121
EBF4	0.087496697	0.953886047
PTPRM	0.087372086	0.93489955
NFATC3	0.087233052	0.944194957
CSNK2A1	0.087104392	0.936131152
CELF4	0.086987339	0.92968159
APPBP2	-0.086983935	0.940760032
ZEB1	0.086969038	0.944194957
SNX15	-0.086804562	0.961050718
GRID2	-0.086783596	0.937744165
ATAD5	-0.086682185	0.961968499
SUPT16H	0.086642166	0.961470873
HNRPLL	-0.086576681	0.942565793
CCDC93	-0.086544157	0.959402585
SAMD4B	-0.086444627	0.954547944
NCOA6	0.086312376	0.898346316
ABCA5	-0.086180359	0.947109537
IRS1	0.086158491	0.957717687
LOC646762	-0.086151768	0.944194957
ACTR3B	0.085884637	0.953657914
DBNDD2	-0.085835266	0.961050718
WDR3	-0.085708431	0.9645372
FGF13	-0.085529877	0.937721851
XRCC5	0.085196961	0.948050406
LRP1B	0.085189474	0.846164113
STXBP5	-0.085176086	0.900401187
KIAA1486	-0.085096283	0.945222406

CCDC77	-0.085068738	0.964356726
INTS9	0.084991599	0.959708103
WFDC3	-0.08494763	0.959402585
FGD4	0.084886424	0.947109537
PPAP2A	-0.084825761	0.927848005
C1orf21	-0.084739541	0.9528975
SNX13	0.084729094	0.951943121
LOC286002	0.084584967	0.954547944
GRIK2	0.08457594	0.913540531
PRKCZ	-0.084448059	0.961050718
AFTPH	-0.084356439	0.959708103
RILPL1	-0.084307641	0.961050718
USP12	0.084285678	0.944194957
CYCS	-0.084278104	0.962760831
DBT	-0.084168793	0.961865495
ATE1	-0.084086947	0.939925569
USP4	-0.084046168	0.955027939
CNOT6	-0.08404007	0.954547944
RIT2	0.084011829	0.906314882
RAF1	-0.083900653	0.956090229
KCNB2	-0.083895869	0.925113775
PHF14	0.083866783	0.925113775
TPM1	0.083744454	0.960062638
SCARB1	0.083578604	0.961865495
EPC1	0.083421477	0.953886047
PPARGC1B	-0.083378921	0.959708103
PPM1H	0.083363774	0.936574459
PPT1	0.083357346	0.9645372
ZNF337	0.0833294	0.959402585
ACO1	0.083292733	0.961050718
COMMD1	0.083091014	0.936131152
SNX27	-0.083048736	0.954547944
PTPN2	0.082969341	0.946648219
JAM3	0.0828124	0.944194957
PTPN12	-0.082774217	0.961050718
EDC3	0.082636335	0.961050718
GRSF1	0.082543101	0.961050718
PAK1	-0.082470742	0.949004166
FKRP	0.082418437	0.959711098
SIPA1L3	0.082376608	0.944194957
UBE2CBP	0.082365792	0.938277634
GRM5	0.082359102	0.867642707
PPP4R4	-0.082353434	0.938993472
BDNF	-0.082315607	0.944194957

MYST3	-0.08231112	0.948050406
CPAMD8	0.082260307	0.960461524
NLN	-0.082257231	0.95772791
NCOA1	-0.081942688	0.907929552
MACF1	0.081931533	0.894936358
PRNP	0.081869364	0.961050718
MTCH2	-0.081773855	0.954547944
STK38L	-0.081679127	0.96213767
LIPI	0.08164098	0.96685848
SUGT1L1	0.081583051	0.949004166
THAP4	0.081519082	0.95197154
JPH1	0.0811552	0.964356726
NFS1	-0.08101043	0.95197154
C12orf47	0.080963901	0.964519792
BAZ1B	0.08096	0.961050718
CSAD	-0.08094956	0.961050718
TMEM66	0.080836479	0.963660654
FARP2	0.080671052	0.953886047
SLC38A9	0.080634328	0.962144785
LPHN1	0.080505556	0.964519792
EMX2OS	0.080392782	0.9645372
RSBN1	0.080333877	0.954547944
PER3	-0.08028617	0.963660654
GUCY1A3	0.080265174	0.959711098
RHOA	0.080235644	0.961050718
LOC100302640	-0.080212923	0.954547944
GRIA4	-0.080153393	0.904542807
ANKRD20B	-0.079887258	0.960461524
MORC3	-0.07974827	0.95197154
MMP16	-0.079707432	0.882777845
PAX5	0.079659481	0.962760831
ALS2CR12	-0.079647038	0.953886047
C5orf44	0.079613908	0.963660654
AP3B1	0.079359517	0.947109537
C6orf174	0.079340657	0.954547944
NSF	-0.079265912	0.960461524
ZNF24	0.079264164	0.948510199
PPARG	0.079226105	0.954547944
TMED5	0.079189863	0.964519792
MBD4	-0.078979955	0.964356726
NDRG3	-0.078732867	0.938260836
C22orf29	0.078702474	0.9645372
FAM178A	-0.078696063	0.961050718
PREX1	-0.078652022	0.938277634

RASGRF2	0.078580936	0.895025634
RPL32P3	-0.078570883	0.954547944
HPS1	-0.078558947	0.959708103
PDCD4	0.078501302	0.964519792
OLFML2A	-0.078489137	0.968308915
EYS	-0.078458413	0.953886047
TTC7B	0.07844359	0.924225123
ZNF780B	0.078440467	0.958508704
ANKRD10	-0.078285963	0.953886047
CNNM2	0.078225863	0.943622135
MAML3	0.078151995	0.950974333
WDR16	-0.078055926	0.960571432
PRPF40A	-0.078039881	0.942565793
FAT3	0.078013105	0.888745066
UNC5B	-0.077628907	0.967744953
CHD2	-0.077545066	0.944194957
BICC1	0.077403382	0.943622135
NUDT11	-0.077374938	0.958666561
NPHP1	0.07737318	0.953886047
CCDC47	0.077356156	0.964356726
URB1	-0.077342008	0.958666561
SCML2	0.077318581	0.960461524
KIAA0319	0.077316176	0.917611589
PTEN	-0.077183525	0.951317204
CUBN	-0.076927343	0.961050718
ASTN1	0.076922808	0.900401187
NRBP2	0.076870267	0.961050718
AP1G1	0.076852311	0.961050718
ZC3HAV1	0.076760185	0.961050718
COL19A1	0.076745664	0.943323597
TMEM184B	0.076742447	0.961050718
SNRNP27	-0.076616149	0.963660654
CADM4	-0.076584036	0.96685848
ZNF680	0.07654517	0.953886047
PUM2	0.076347859	0.953886047
C14orf145	-0.07634193	0.944194957
TTTY10	0.076330689	0.971260724
DGKB	-0.076243363	0.948050406
MAPK9	0.076212702	0.958666561
MLLT3	0.076146836	0.890373571
ANKRD11	0.076134243	0.944194957
KAL1	-0.076099282	0.961328961
STON1-GTF2A1L	-0.07605339	0.967578176
AP3S2	0.076050364	0.961050718

TRIM24	0.075991661	0.951317204
THEMIS	-0.075974115	0.958666561
C1orf52	0.075885193	0.961050718
LOC595101	0.075847239	0.964519792
CDC42SE2	0.075824945	0.947109537
ADAMTSL3	-0.075677623	0.954547944
CMTM4	0.075542571	0.961050718
DUS2L	-0.075500825	0.961470873
UGGT2	-0.075489042	0.898500725
SERINC3	0.075421111	0.959708103
ZNF169	-0.075420241	0.9645372
NFATC2IP	0.075413723	0.961470873
MXRA7	0.075398773	0.965597964
FRYL	-0.075344763	0.941701504
SATB2	-0.075336549	0.954547944
RAD17	-0.075324044	0.964356726
GPATCH8	0.075312041	0.949004166
SPG20	0.075219051	0.954364434
RAP2B	-0.075211105	0.9645372
C3orf31	0.075205614	0.954547944
SOS2	0.07519849	0.936574459
ARID2	-0.075140358	0.939333048
CCDC88A	0.075070074	0.9497204
BRMS1L	0.074932191	0.961865495
EID1	0.074916765	0.961050718
PRPSAP2	-0.074910745	0.961050718
ATRX	-0.074902502	0.927848005
MIR548W	0.074841798	0.879819037
SAMHD1	0.074838336	0.944194957
ACBD6	-0.074811648	0.924494066
TTC9	-0.074802219	0.961050718
COX11	0.074668967	0.961050718
ZNF721	-0.074641638	0.954547944
MBD1	-0.074570438	0.954547944
CCDC30	0.074261538	0.943090387
CCDC92	0.074244068	0.955027939
C15orf29	0.074239805	0.954547944
EIF2S3	0.074165267	0.964519792
MAST1	0.073999386	0.963660654
NTF3	-0.073982748	0.9645372
HOOK3	-0.073981582	0.92929992
SLC35F1	0.073819027	0.92181255
C4orf3	0.073783066	0.949004166
ZFR	0.073766143	0.946648219

R3HDM1	0.07365819	0.886822411
NMNAT2	0.073649461	0.933054351
ATL2	0.073645183	0.962760831
TAF9B	-0.073612671	0.967744953
SLC44A1	-0.073261674	0.948510199
DRP2	0.073258258	0.961050718
RSRC1	0.073151349	0.948510199
SEMA4F	0.073148103	0.9645372
PDS5A	-0.073110014	0.938277634
MSH4	-0.073085822	0.961050718
ANO4	0.072877079	0.95982111
GOSR1	0.072623545	0.953886047
NDRG4	0.07257734	0.953886047
GNB1L	-0.072453117	0.964356726
ARF3	0.072213784	0.961050718
CENPP	0.072157515	0.949004166
LOC550643	-0.0720815	0.960807874
LOC400657	0.072043649	0.968072508
RBPJ	0.072018463	0.949004166
NHS	-0.071922448	0.964519792
CASD1	0.071867113	0.959402585
ZC3H8	0.071863901	0.961050718
SPAG17	-0.071820343	0.964356726
ABCA12	0.071724555	0.958508704
ABI2	0.071711977	0.93732271
TMEM161B	0.071589479	0.96685848
BET1	0.071567286	0.965947815
IKZF2	0.071371469	0.961328961
PPP1CB	-0.07136602	0.950723929
SUN1	0.071330653	0.954547944
ARPC1A	0.071329909	0.963770361
ZZZ3	0.071174253	0.95197154
TRIM9	-0.071151913	0.944746961
CDK17	0.071059753	0.948050406
SLC27A2	-0.07103435	0.965805199
FAM18B2	0.071015239	0.964519792
RCBTB2	0.070923167	0.965947815
TLK1	-0.070898601	0.900401187
RPP30	0.070895495	0.963660654
ACSS2	-0.070845971	0.961050718
CTTNBP2	-0.070837457	0.939333048
IGF2BP2	0.070797508	0.965947815
FAM81A	0.070734095	0.950183869
ADAMTS9	0.070726335	0.96685848

C11orf30	0.070655319	0.952988899
LRRTM4	0.070519625	0.915078054
ELP2	-0.070464072	0.962760831
MOXD1	-0.070446647	0.967744953
SLC13A3	0.070419407	0.938277634
NDFIP1	-0.070401454	0.956090229
ALG13	0.070396379	0.963660654
SCLY	-0.070375123	0.965447154
SMS	-0.070284243	0.9645372
SLMAP	-0.070251981	0.954364434
ATXN7L3B	-0.070237234	0.967578176
ASH1L	0.070213333	0.953886047
TYW3	0.070134321	0.961050718
DOCK3	0.070132202	0.915078054
SYTL5	-0.070086135	0.965224179
BMPR2	0.070030046	0.93489955
UBE2D2	0.070028824	0.959708103
RAD54L2	-0.069956977	0.963660654
QRSL1	-0.06982668	0.964519792
THOC1	0.069821926	0.946636505
HNRNPC	0.069701131	0.963660654
COL4A3BP	-0.069686415	0.949004166
RAGE	-0.069432311	0.963660654
FBN3	-0.069263282	0.967744953
PDZD2	-0.069251418	0.954547944
INTU	-0.069241894	0.961050718
SLC16A14	0.069161006	0.9645372
CBL	-0.069130855	0.961050718
ROBO2	0.069084485	0.92929992
NOS1AP	0.068985836	0.944194957
PRKD2	0.068925633	0.968743278
LPIN1	0.068924441	0.953886047
ATF2	-0.06888551	0.959708103
CYP46A1	0.06862192	0.944194957
CDC123	0.068589835	0.968072508
ACSL4	0.068507551	0.957887885
PPME1	-0.068457818	0.963660654
FOXJ3	0.068450725	0.9498477
MAGI1	-0.068403494	0.944194957
HSD17B12	0.068399188	0.954547944
AFF3	0.068357082	0.933054351
WDFY1	-0.068351082	0.961050718
DPY19L1	0.068302805	0.967744953
KAZ	0.06827899	0.944194957

CDYL2		-0.068215581	0.959563208
IPO5		0.068187591	0.966767248
DDX26B		0.068160427	0.970373706
ITSN2		-0.068118655	0.943185221
PFKFB4		0.068093847	0.974018045
STXBP5L		0.068086144	0.953886047
LOC441666		0.068022954	0.968072508
WDR37		-0.067927762	0.954547944
RECK		0.067917741	0.944194957
PDXK		0.067840667	0.961050718
ATP2B2		-0.067800994	0.959402585
SCN3A		-0.067783951	0.946648219
ZNF793		-0.067782489	0.964519792
FRMPD4		0.067754202	0.892459242
MGAT4A		0.06752782	0.960964112
ALG14		-0.067484043	0.95982111
GPD2		-0.067407635	0.957124402
TPCN1		0.067357916	0.967744953
MBOAT2		0.067318209	0.949004166
TUG1		0.067314252	0.958282713
ARHGAP10		0.067283361	0.960461524
METT10D		0.067281931	0.960461524
EIF2A		-0.067181437	0.963660654
ACAP2		0.067098094	0.945222406
UBXN7		-0.067069298	0.961328961
TM9SF3		0.067032073	0.967744953
FOXN2		-0.067002582	0.96685848
ATP5G3		0.066991401	0.968743278
CMIP		0.066944746	0.946648219
KIAA1199		0.066735815	0.968743278
C10orf46		-0.06669346	0.963660654
CDK5RAP1		-0.066687091	0.954547944
RIMS2		0.066639823	0.948050406
DCP1A		-0.066473415	0.961050718
ADD3		0.066407714	0.952988899
	44624	-0.06624114	0.947109537
RASSF2		-0.066188597	0.963660654
CHPT1		-0.066152382	0.963660654
COL21A1		0.066031449	0.961050718
PKD2		-0.06597692	0.958508704
ZFYVE16		0.065955244	0.961470873
NLRP11		-0.06592236	0.965947815
PPP1R9A		0.065893912	0.933162474
CNOT6L		0.065874618	0.963660654

CCDC46	-0.065855027	0.930231228
MATN2	0.065854614	0.961050718
WDR41	-0.065685295	0.967744953
MSRB3	0.065657918	0.961050718
THRA	0.06536449	0.960797403
SLC28A3	0.065282144	0.971285439
PACSIN1	-0.06513786	0.954547944
SNTA1	0.065105112	0.963660654
CACNG7	0.065041371	0.965447154
C5orf54	-0.064999628	0.970373706
C1orf204	0.06497519	0.9645372
RCC1	0.064835677	0.970373706
GPR56	0.064672873	0.96685848
PHF20	-0.064609899	0.957722992
PTPRG	0.064518334	0.942565793
FLJ42709	-0.064402506	0.962760831
SSBP3	0.064319956	0.9645372
AHCTF1	0.064281937	0.959402585
BLNK	0.064203562	0.973302936
C6orf89	-0.064136153	0.9645372
ACADSB	0.064079542	0.9645372
RGPD1	0.064054925	0.968743278
GUCY1A2	0.06396614	0.95197154
PDE3B	-0.063852962	0.962760831
KLHDC5	0.063835792	0.964519792
STX17	0.063793216	0.95597696
SPOP	-0.063712212	0.959005288
C14orf23	-0.06361103	0.968743278
C10orf35	-0.063510333	0.963660654
IARS	0.063485973	0.960461524
KIAA0146	-0.063438587	0.933054351
GAN	0.063376404	0.966767248
ANKRD42	-0.06336299	0.962760831
RSBN1L	0.063295362	0.963660654
ZSWIM6	-0.063294335	0.953886047
NUPL2	0.063247405	0.969837079
ZNF419	0.06315187	0.968743278
UBE2O	0.062979163	0.965744961
SS18	0.062873832	0.9645372
C4orf21	-0.062778712	0.965947815
ANKRD26	-0.062676713	0.955750924
ZBTB8OS	-0.062527064	0.961050718
TSTD2	0.06232147	0.963660654
ZC4H2	-0.06219871	0.961050718

C9orf84	0.062101577	0.971285439
GALNT14	0.062084998	0.955944357
MAST2	0.062021981	0.95982111
FAM120B	0.061995995	0.92929992
CTNND2	-0.061979493	0.944151594
UBXN4	0.061911386	0.954547944
LOC729852	0.061854911	0.960461524
RPH3A	-0.061833688	0.96685848
DCAF6	-0.061815804	0.946648219
SAMD12	0.061789907	0.92929992
MIR548H4	-0.061772081	0.958666561
ENDOD1	0.061760232	0.967744953
FYN	0.061756566	0.954547944
PRKACB	0.061639145	0.961050718
ZNF718	-0.061504781	0.953886047
TNFRSF19	-0.061362781	0.972351566
BPNT1	-0.061290703	0.970373706
SBNO2	0.06122968	0.97370401
MIR548F3	0.061150592	0.961050718
PTPRJ	0.061124687	0.953886047
KIF5B	0.060989588	0.971260724
ALMS1	0.060982596	0.954547944
HSDL1	-0.060961896	0.971285439
INPP4B	0.060919059	0.927848005
EDEM3	-0.060897959	0.963660654
DCAF17	-0.060858696	0.963660654
ACACA	0.060849575	0.944194957
NR2C2	0.060758818	0.968491799
SEMA3C	0.060687263	0.960461524
SMARCC1	-0.060686206	0.963660654
LPPR4	-0.060617767	0.961968499
EMR2	0.060608845	0.970373706
PMP2	0.060605023	0.964356726
IFT80	-0.060594017	0.954547944
C20orf117	-0.060572016	0.953886047
FAM179B	0.060567336	0.961050718
IRAK1BP1	-0.060533608	0.965947815
BMP2K	-0.060503111	0.970373706
C21orf34	0.060485546	0.935838024
SUMF1	0.060353066	0.956063888
ENPP6	0.060132697	0.968743278
RAB3C	-0.060100117	0.961050718
NMT2	-0.060098838	0.96685848
OSTM1	-0.060033647	0.971260724

AFG3L2	0.059972025	0.965447154
WDR11	-0.059872894	0.970373706
GPCPD1	0.059867164	0.961050718
FAR1	0.059827929	0.963660654
SPATS2L	0.059789824	0.951317204
LOC283089	0.059701753	0.967744953
SP3	-0.059559001	0.961050718
DPYS	-0.05953479	0.973158666
LEPROTL1	0.059528903	0.971285439
ESCO1	-0.059496929	0.964356726
ZNF562	0.059483953	0.9645372
TMEM57	-0.059322776	0.966072558
VCL	0.059321992	0.967709322
TTC12	0.059234724	0.971285439
TOPBP1	-0.059166015	0.9645372
RPTOR	-0.059127953	0.964356726
FIP1L1	-0.059104584	0.967578176
GRIN2B	0.059018334	0.946648219
ETFDH	0.058945257	0.968743278
MEGF9	0.058941559	0.949004166
RIMS4	-0.058889907	0.9645372
FBXO16	-0.058870299	0.961050718
C5orf36	-0.058745339	0.949609566
ATL1	0.058647432	0.964356726
GSN	-0.058579763	0.968743278
KIF18A	-0.058491325	0.97370401
GARNL3	0.058490878	0.954547944
LYST	-0.058478492	0.959402585
COBLL1	0.058339432	0.9668154
ZMYM5	0.058336555	0.963770361
TMEM144	-0.058276935	0.971285439
NDUFAF2	-0.057840887	0.953886047
FEZ1	0.05779555	0.967578176
GABRA3	0.057747504	0.964356726
COL4A4	0.057618701	0.9645372
SVOP	-0.05761761	0.959708103
DENND5B	-0.057613359	0.946648219
SLK	-0.057604595	0.971285439
RGS6	-0.057538449	0.946648219
CACNB4	0.057484912	0.944194957
RSL1D1	0.057401685	0.9645372
FLCN	-0.057364269	0.967744953
NUP54	0.057349729	0.968743278
RAPGEF6	-0.05719083	0.956090229

KIAA0368	0.057160258	0.959708103
GRAMD1B	0.057022021	0.9528975
ZHX2	0.057016859	0.9645372
DYX1C1	-0.056991369	0.960461524
SPAST	0.056980853	0.9645372
IFT172	0.056921636	0.963660654
CCNYL1	-0.056907729	0.968743278
BEX2	0.056847438	0.965447154
LHCGR	-0.056828966	0.980213384
YTHDC2	-0.056696513	0.9645372
CEP192	-0.056646101	0.959708103
NEB	-0.056606539	0.967744953
ZFAND1	0.056582901	0.968976836
ADD2	0.056514916	0.9645372
NUMB	-0.056466805	0.953886047
PAPPA	-0.056419581	0.972351566
DAPK1	-0.056200635	0.959711098
LRRFIP2	0.056086848	0.961050718
HPCAL4	0.056044521	0.961050718
MTBP	-0.056012811	0.971285439
DKFZp686K1684	-0.055972206	0.978771914
CSMD3	0.055958795	0.920669889
ZCCHC9	-0.055958645	0.971285439
KCNMB4	0.055924286	0.959402585
IPO9	-0.055895753	0.953886047
USPL1	-0.055873408	0.972351566
PTPRN2	0.055763258	0.953886047
FGF1	-0.055635384	0.971285439
NGLY1	0.055612411	0.971260724
IFT81	-0.055484432	0.96685848
MUC16	-0.055432662	0.971285439
NFIX	0.055395051	0.968743278
NKAIN2	-0.055241034	0.950020074
PBX1	0.055150754	0.925113775
JAKMIP2	-0.05502813	0.954547944
CPNE5	0.054954703	0.971285439
ADCK1	-0.054849975	0.967744953
B4GALT5	-0.054814686	0.958146178
EPHA6	-0.054713052	0.922974649
KLHL4	0.054691481	0.967744953
ZNF292	-0.054666938	0.9645372
FBXW4	0.054661072	0.968743278
WDR4	-0.05461535	0.970373706
CFDP1	0.054408477	0.9645372

SARS	-0.054301364	0.971260724
UBA1	-0.054270459	0.97370401
BANK1	0.054167665	0.971260724
ADAM32	-0.054030094	0.961328961
LIMA1	-0.053991806	0.970373706
PGAP1	-0.05388037	0.9645372
SERAC1	-0.053828312	0.968743278
SNX29	-0.053824628	0.959711098
CDKL1	-0.053804846	0.963660654
LUZP1	0.053728778	0.9645372
TBCK	-0.053696676	0.949004166
MCCC2	0.053684566	0.971049515
LOC728723	0.05359984	0.968743278
ADCY10	-0.05359619	0.968743278
SON	-0.053535513	0.963660654
PTPRZ1	-0.053475499	0.963660654
MPDZ	0.053465883	0.9645372
GLT1D1	-0.053430569	0.969382945
RHOT1	-0.053380127	0.965447154
RNF214	-0.05334598	0.970373706
KLHL18	-0.053286204	0.971049515
CYFIP2	0.053285308	0.961050718
SLC24A2	0.053257471	0.950108534
CTDSPL	0.05322474	0.964356726
CEP290	0.05315293	0.9645372
KIAA0513	0.052960768	0.972351566
PFKFB3	-0.052950138	0.965405471
GRM1	0.052787444	0.946648219
FOXO3	0.052648859	0.965447154
CLIC4	0.052603214	0.978861098
RGNEF	-0.052592691	0.960461524
CCDC149	0.052576167	0.9645372
EIF2C3	0.05244335	0.961470873
LHFP	-0.052363159	0.968491799
POLR3A	0.05226768	0.971260724
FAM168A	0.052257662	0.944194957
RFX2	0.052141788	0.968491799
PRPSAP1	0.05197854	0.977867487
CNTNAP2	0.051960495	0.918396705
SHOC2	-0.05194676	0.959708103
SOBP	0.051917762	0.944947365
RBM33	-0.051730369	0.965447154
TCERG1	-0.051684085	0.965597964
SMYD3	0.051508574	0.925113775

NIPAL2	0.05147662	0.963660654
VPS54	0.051431556	0.958731287
PLOD2	-0.051416495	0.971285439
WNT3A	0.051386513	0.978861098
HOOK1	-0.051191141	0.971285439
TUFT1	-0.051108498	0.975625827
PDIA6	-0.051082262	0.971260724
PTPN11	0.050910011	0.971260724
SLIT3	-0.050872628	0.94751091
ADCY1	0.050870822	0.946648219
DNMT1	0.050735972	0.975625827
GLIS1	-0.050678589	0.968914215
C8orf34	0.05067491	0.942565793
TMEM232	0.050651147	0.953464415
RBBP4	-0.050644045	0.9645372
NIPBL	-0.050569292	0.963660654
ARHGAP42	-0.050443655	0.971760161
GPM6A	-0.050389379	0.943185221
TTLL9	-0.050137188	0.972351566
ST7	0.050096275	0.9645372
EFTUD2	0.050091673	0.971260724
COPB1	0.050060292	0.964943991
MTHFD1L	0.050044254	0.9668154
HTR2C	-0.050021944	0.975307212
ANKRD6	-0.050018649	0.971260724
RSF1	-0.050004515	0.961050718
EPB41L2	-0.049998349	0.961050718
NUMA1	-0.049935878	0.97867377
GRIN3A	0.049899804	0.971260724
SLC8A1	0.049894192	0.95197154
GGA2	-0.049669107	0.971459661
SCAMP5	-0.049627668	0.971459661
KIAA0895L	0.049594182	0.963660654
LPP	-0.049572761	0.969777048
EXOC6B	-0.049418004	0.944194957
DOCK9	0.049368707	0.971260724
SYBU	-0.049368285	0.964519792
FAT4	-0.049348865	0.968491799
GABRG3	-0.049338845	0.9645372
PRDM4	0.049276816	0.971285439
EML6	0.049245418	0.961050718
DPP6	0.049139975	0.955944357
RHBDL3	-0.049076933	0.971260724
PRR16	-0.049012925	0.965947815

IMMP2L	-0.048977246	0.961050718
TRRAP	-0.048933819	0.967744953
TBC1D15	-0.048919161	0.965597964
RAB22A	-0.048823324	0.971260724
TBC1D5	-0.048710081	0.92929992
ZDHHC20	0.048677706	0.961470873
ACSL3	0.048471175	0.968743278
ZNF678	-0.048430882	0.971285439
COL24A1	-0.048387915	0.965447154
NFRKB	-0.048225498	0.975409703
MCF2L2	0.048191595	0.953886047
TRPM3	-0.048154367	0.952641232
C2orf55	0.048126608	0.961050718
LSM14A	0.048124015	0.9645372
SGPP1	0.048115704	0.973158666
BMPR1B	0.048029925	0.971049515
TMEM111	-0.048022579	0.979370428
TMEM63B	0.048017396	0.971260724
RGS9	-0.047981472	0.975625827
CADPS	0.047913118	0.939406327
TRAPPC10	-0.047794211	0.971285439
FBXL17	0.047335301	0.954547944
MRRF	-0.047249053	0.97370401
PDE8B	0.047173988	0.961050718
RAB27A	-0.047124309	0.971260724
PALM2-AKAP2	-0.04689912	0.954547944
NLGN4X	-0.046832154	0.963660654
KSR1	-0.046659304	0.967744953
DYNC1I2	0.046584432	0.971049515
EXOC4	0.04646672	0.944194957
ARHGAP20	0.046455921	0.968743278
LOC283480	-0.046392913	0.967578176
EYA3	0.04637755	0.9645372
THSD7B	-0.046150924	0.961050718
MYOCD	-0.046122741	0.978861098
ABTB2	-0.046104768	0.972351566
DLC1	-0.046098278	0.968743278
RBMXL1	-0.046027964	0.975625827
PPP6R3	0.045998951	0.961050718
LY86AS	0.045994717	0.948510199
NAPEPLD	0.045994379	0.972351566
VPS13C	0.045950393	0.965585618
ZCCHC11	0.045822955	0.961050718
ZNF770	0.045778225	0.974703442

ANO3	-0.045754476	0.948510199
FAM196A	-0.045640496	0.971285439
TBC1D4	-0.045424907	0.9645372
DCAF16	0.045422561	0.965224179
TPP1	0.04536145	0.968743278
CPEB2	0.045318237	0.975409703
PPARD	-0.045314439	0.968743278
WDSUB1	0.045242713	0.97431406
PPP3CB	0.045215934	0.971260724
ADA	-0.045210727	0.973158666
MAPKSP1	0.04514991	0.971285439
CKAP5	0.044690088	0.970373706
PTPN18	0.04464111	0.968743278
EPHB2	-0.044612796	0.971260724
ELP3	0.04460046	0.977867487
TMEM116	0.044594108	0.971285439
UPP2	-0.044356156	0.96685848
DNAJC21	0.044249555	0.974018045
SNX3	0.044163322	0.978080098
SLC39A10	0.04405476	0.97370401
DPM1	-0.043896078	0.968743278
PHTF2	-0.04378445	0.965947815
SIPA1L1	0.043767986	0.958666561
TFDP2	0.043741957	0.954547944
CNTNAP1	-0.043716428	0.970373706
PLD1	-0.043691927	0.963660654
ROBO1	0.043573449	0.961328961
MALAT1	0.043527352	0.971260724
ZCCHC18	0.043477598	0.980448152
FAM78B	0.043449331	0.971285439
CTDSPL2	0.04344394	0.961050718
C14orf37	0.043249155	0.967578176
SSR1	-0.043217254	0.97370401
CAMK2G	-0.043195006	0.971285439
DUSP14	0.04318019	0.980448152
ANO10	-0.04314248	0.963660654
GPBP1L1	-0.043111176	0.973158666
KIAA0753	0.043108904	0.97370401
ARPP19	0.043034372	0.971049515
GNAO1	-0.04296771	0.96685848
GNAQ	0.042898558	0.944194957
ZNF100	-0.042802678	0.980448152
PANK2	-0.042725536	0.97764905
COL14A1	0.042686609	0.975583704

GANC	0.042617248	0.977867487
COBL	-0.042597098	0.963063974
LRRC28	0.042533055	0.967744953
UNC13C	0.042509729	0.944194957
LCMT1	0.042498014	0.972351566
RANBP17	-0.042475598	0.975409703
SRRM3	-0.042340462	0.977867487
USP34	0.042204431	0.953886047
FAM190B	-0.042178994	0.963672059
KIAA1109	0.042129211	0.967805048
DPYD	-0.042090147	0.944194957
IRGQ	0.042089932	0.977867487
RAB3IP	0.042020046	0.971049515
ZNF649	0.041834833	0.975625827
PSD3	-0.041797163	0.965021779
LDB2	0.041786919	0.964519792
PREPL	0.041677166	0.971049515
TXNDC15	-0.041570488	0.974801494
C16orf61	0.041502266	0.971285439
LOC202181	0.041474403	0.968743278
RGL1	-0.0414734	0.964356726
SLC4A5	-0.041472111	0.975625827
HECW2	-0.041407426	0.961050718
ARHGEF3	-0.041315288	0.971285439
PLEKHA5	0.041090961	0.953886047
NUBPL	0.041081256	0.967744953
ASXL2	-0.041070196	0.96685848
HSPA12A	0.04095806	0.977328821
CMYA5	0.040833749	0.977867487
CASP8AP2	0.040828026	0.97370401
XYLB	0.040753018	0.978861098
CD47	0.040752511	0.970373706
SNCAIP	0.040739575	0.971285439
SSX2IP	0.040716502	0.971260724
NUDT3	0.040675563	0.968743278
RNU5D	0.040655458	0.967744953
RNU5E	0.040655458	0.967744953
EFHD1	-0.040585232	0.972351566
NETO1	-0.040572842	0.967709322
SSBP2	0.04054941	0.9645372
FASTKD1	-0.040442495	0.971049515
MAST4	0.040441465	0.961470873
FAM184B	0.040439901	0.973158666
ARHGAP32	-0.040423978	0.963660654

HEPH	-0.040273922	0.980857907
LRRC40	-0.040259203	0.975409703
CDH2	-0.040111866	0.963660654
TOMM70A	0.040068465	0.977867487
C9orf102	-0.040003192	0.971260724
XKR4	-0.039996794	0.959711098
BRIP1	-0.039882246	0.969837079
CEP350	-0.039737088	0.971285439
PCLO	-0.039637144	0.954547944
TMEM181	-0.039574628	0.980132631
CREG2	0.03954805	0.971049515
CFLAR	0.039521787	0.972351566
GCC2	0.03951283	0.975625827
MAP2	-0.039500832	0.953886047
SH3BGRL	0.039488125	0.967744953
C1orf216	0.039474762	0.971285439
DIS3L2	0.039462344	0.9645372
SCAPER	0.039396598	0.951317204
GALNT7	0.039395632	0.975996635
BHLHB9	0.039169451	0.97370401
TMEM186	-0.03908905	0.980471633
NLGN1	0.039079968	0.9645372
MYO5A	-0.039063729	0.959708103
CAPN7	0.038972977	0.967744953
ZNF829	-0.038889814	0.980448152
CALN1	0.038865163	0.9645372
SEL1L2	0.038817363	0.968743278
CC2D2A	0.038641225	0.978861098
ZNF43	-0.038561947	0.972351566
EEPD1	0.038343981	0.972351566
EIF4ENIF1	-0.038317301	0.980392548
YPEL1	0.038311564	0.97764905
DNAJB14	0.03826459	0.977735084
UNC80	0.038258232	0.963660654
MLL5	-0.038145009	0.979370428
MDN1	-0.038088361	0.967744953
SGSM1	0.038081146	0.971260724
ST7L	-0.038036736	0.979370428
ISPD	-0.037980972	0.971285439
SEC14L2	-0.037916365	0.977399286
ANKH	-0.037891113	0.965447154
HNRNPA0	0.037880559	0.983965193
SRRM4	-0.037851676	0.972351566
C1orf125	0.03778272	0.979370428

LRRC4C	-0.037700527	0.963412007
TDRD3	-0.03762833	0.967744953
WWC3	-0.037563795	0.977867487
ARHGAP5	-0.03755686	0.971813675
ZFP1	-0.037318319	0.975996635
RANBP10	0.037246979	0.979370428
MOV10L1	0.037230519	0.981453518
UBAC2	-0.037138318	0.971285439
SNRPA1	-0.037130697	0.980448152
IQSEC2	-0.036962501	0.971285439
TMOD1	0.036866602	0.965947815
ABCA11P	0.036847071	0.971260724
MAN2A2	0.036702489	0.972351566
HIVEP3	0.036641235	0.962760831
TRIM71	0.036552493	0.97941065
GRK5	0.036434554	0.977867487
AK7	0.036396382	0.979370428
E2F3	-0.036362299	0.980448152
REV1	0.036193714	0.971285439
BNC2	-0.036143124	0.979370428
ANKRD31	-0.036098289	0.977867487
MTMR2	-0.035995718	0.977867487
MIR548T	0.035785909	0.9645372
CWC27	-0.035725119	0.9645372
TRIM14	0.035683653	0.980448152
SPOCK3	0.035605437	0.962144785
DNAH9	-0.035490971	0.961050718
LOC147727	0.035438942	0.977399286
TACC2	-0.035197936	0.977959918
C4orf29	-0.035126113	0.975409703
DNAJC6	-0.034781661	0.963660654
CCDC113	0.034752205	0.980448152
TMEM146	0.034728844	0.980392548
MAP2K6	-0.034694687	0.977867487
PTPRD	0.034516136	0.962760831
JPH3	0.034489324	0.979994322
MECP2	0.034362719	0.980448152
CREBBP	0.034341156	0.973158666
ZNF254	-0.034339959	0.975233266
SREBF2	-0.034324873	0.975625827
SCAMP1	-0.034077164	0.971285439
TAOK1	-0.033972621	0.968743278
C16orf72	-0.033957246	0.980335777
SKIV2L2	0.033914226	0.97370401

BEX1	-0.033886024	0.977867487
ZFYVE1	0.033796152	0.980448152
LMO3	-0.033769922	0.980857907
NFU1	0.033663503	0.980392548
PPARA	0.033610803	0.975423826
SEC31A	0.033571975	0.977399286
MCAM	-0.033547652	0.983317567
SMC6	-0.033337036	0.980392548
TRAM1	-0.033161446	0.980448152
TTI1	-0.03279217	0.980335777
PLB1	0.032779117	0.980213384
PTAR1	0.032742676	0.980213384
CDH10	-0.032670566	0.978861098
PPP2R3A	0.03240372	0.978080098
ZDHHC15	-0.032333817	0.978550077
RAP1GDS1	0.032284744	0.968072508
RALGAPA1	-0.03228316	0.967744953
ARMCX4	0.032282447	0.971285439
RBM26	-0.032180422	0.96685848
PAIP2	-0.03195227	0.977867487
PLD3	-0.031925235	0.976121924
TXNL1	0.03188549	0.979994322
ACOT7	-0.031870966	0.983965193
PSMA7	-0.031846221	0.981925107
PCNXL2	0.03180703	0.963660654
PARN	0.03175965	0.974018045
RPRD2	0.031690995	0.977735084
AMY2B	-0.031621982	0.980448152
LRBA	0.031602122	0.961968499
CSNK1A1	0.031589982	0.980448152
C2orf3	0.031560383	0.980448152
VKORC1L1	-0.031445354	0.981925107
MED14	0.031418965	0.980448152
ZSCAN18	-0.031367636	0.977867487
PTGR1	-0.031286958	0.978861098
SUPT3H	0.031258713	0.965224179
FAM5C	-0.031074519	0.980335777
RIC8B	-0.031052776	0.967805048
RAB18	0.031045068	0.981925107
ABHD12	0.030976211	0.975583704
CD8A	-0.030899747	0.981925107
KIF20B	-0.030779471	0.983401076
GMPS	0.030700241	0.982631153
SYP	0.030490513	0.980448152

CDK19	0.030476855	0.975625827
PROSC	0.030402263	0.983401076
MPPED2	-0.030257314	0.971285439
LOC100189589	-0.030188575	0.985055894
VWC2	-0.03016689	0.982631153
C5orf42	-0.030110682	0.971285439
OXR1	0.030085479	0.9645372
SLC35A1	0.029998937	0.980448152
FLJ41278	0.029957515	0.975583704
C11orf63	-0.029911009	0.981925107
KCND3	0.029816461	0.971260724
TNFRSF10B	-0.029588227	0.985535626
SGCE	-0.029531542	0.979447487
ITGB8	0.029524664	0.980448152
HIBCH	-0.029469989	0.971260724
FBXW11	-0.029455836	0.980471633
SGCG	0.029266853	0.981476036
WDR69	-0.029249279	0.980213384
WNK1	-0.029243115	0.975625827
SPIN1	0.02923277	0.967744953
SEMA5B	-0.029173435	0.975414195
CNTN5	-0.029150088	0.9645372
ZFP106	0.029141772	0.980213384
DDX10	0.02898869	0.975625827
ERC1	0.028877165	0.965447154
NAA16	-0.028722669	0.975409703
TGS1	-0.028681865	0.981925107
C6orf165	0.028642858	0.983965193
C9orf85	-0.028546647	0.980448152
TTC28	0.028498925	0.964356726
REL	-0.028362549	0.985055894
KIAA1012	0.028338179	0.978771914
UNC5A	-0.028133469	0.980213384
MBIP	-0.028025782	0.979370428
DOCK7	0.028005909	0.967744953
AZIN1	-0.027956447	0.981925107
ADNP	-0.027940834	0.980448152
PCMTD1	-0.027854214	0.972183784
ENOSF1	-0.027688559	0.981925107
PDE3A	-0.02761429	0.980448152
PTPLB	-0.027490583	0.982382884
ZDHHC17	-0.027257823	0.978771914
LARS2	-0.027182891	0.980448152
SLC39A6	-0.027180418	0.985791743

DIP2A	-0.027154012	0.980448152
FAM160B1	0.026913067	0.984426615
STOX2	0.02683533	0.980448152
MAPKAPK5	-0.02666022	0.984989729
LOC57653	0.026589246	0.980448152
TOX	0.026545466	0.986272679
FAM178B	0.026526385	0.984426615
LARP1	0.026520538	0.975625827
RPS23	-0.026404104	0.985535626
RBM39	-0.026393669	0.980448152
ITGA1	-0.026366923	0.983965193
MAP4	-0.026322332	0.979370428
PARVA	-0.026316563	0.983965193
LOC440944	0.02625015	0.981925107
PPP2R5C	-0.026185211	0.980448152
GALK2	0.026174403	0.975625827
C6orf163	0.026156919	0.985055894
TMEM49	-0.026058367	0.980979091
C17orf69	0.02590237	0.980392548
ZNF160	-0.025881066	0.984190441
DPH2	0.025867153	0.984417894
NETO2	-0.025861747	0.985717288
NME7	0.025825274	0.971049515
KIAA0427	0.025776109	0.971285439
GSG1L	-0.025640033	0.974703442
DLGAP4	0.025603211	0.978865643
EXD3	-0.025549906	0.983299624
CACHD1	-0.025508057	0.973158666
ZNF519	-0.025506291	0.979755749
SDCCAG1	0.025468349	0.982432411
OSBPL5	-0.025462957	0.983965193
PCDHAC2	0.025386038	0.985535626
ZC3H13	0.025243415	0.980979091
SLAIN2	-0.025229973	0.983317567
SGOL2	0.024953252	0.986974364
ATP6V1C1	0.02477732	0.983965193
SLAIN1	-0.024751826	0.977867487
ZFX	-0.024686743	0.985055894
KIAA1409	-0.024397003	0.966639693
GPR158	-0.024259523	0.978771914
TRIM25	-0.02422497	0.980593465
FAM154B	-0.024123817	0.983965193
ITPR1	-0.024063494	0.977049648
TATDN3	0.023974685	0.984426615

SCN2A	-0.02375836	0.975583704
PDK3	-0.023643181	0.984426615
ZCCHC6	-0.023642343	0.980448152
GNB1	0.023637519	0.981925107
TCEAL8	0.023229605	0.98631073
MAGED2	0.023182442	0.985055894
ZNF673	0.023125859	0.987941842
SPG7	-0.023041779	0.986974364
GTF2B	-0.022995705	0.986974364
C12orf26	0.022899108	0.980335777
SLC39A11	0.022694673	0.977867487
CDYL	-0.022634607	0.980471633
GNA14	0.022601691	0.973158666
RBM41	0.0225702	0.980593465
LEMD3	0.022559844	0.983965193
USP40	0.022527311	0.983965193
PAN3	0.022519766	0.968743278
KIF16B	-0.022497249	0.974801494
MSH2	-0.022351429	0.985055894
ZNF320	0.022205995	0.988246745
SND1	-0.022205669	0.977867487
CAPZA2	0.022203682	0.985055894
ZNRF1	0.02215567	0.980593465
ITSN1	0.022154808	0.980448152
BRWD1	-0.022152416	0.980593465
RDH13	0.022090289	0.985717288
PHF21A	0.022067823	0.977735084
FANCL	-0.021953492	0.986974364
VTI1A	0.021947079	0.974236902
CACNA2D4	-0.021942474	0.985055894
CDH18	-0.021930496	0.967744953
NDUFS2	-0.021858689	0.983965193
CHN1	-0.02184002	0.968743278
CCDC34	0.021811241	0.983299624
VTA1	0.02167594	0.983401076
ZFP90	0.021652399	0.985535626
MPHOSPH8	0.021611102	0.975625827
PTPRO	-0.021607439	0.975414195
TMEM163	0.021559307	0.983965193
SUPT4H1	0.021447567	0.98741223
CTXN1	0.021400917	0.985535626
DYNC2H1	-0.021360241	0.975583704
CHD6	-0.021288275	0.971756765
ZNF616	-0.021265845	0.987941842

SLC1A1	0.02119937	0.985055894
RPS6KA2	0.02114498	0.980471633
CCDC123	-0.021119464	0.984495123
BACH2	-0.021099537	0.985616838
WDR82	-0.021060739	0.981925107
LIN28B	0.021011771	0.988755735
DISC1	0.020888883	0.981925107
CCT4	0.020822935	0.986702177
ABCC4	0.020775709	0.983317567
SGMS1	-0.020492611	0.974801494
MAPT	-0.020452944	0.980448152
WSCD1	-0.020321175	0.986272679
RAB2A	0.020151934	0.985055894
CLIC5	-0.019994121	0.987941842
BCL11B	-0.019943467	0.98182508
GINS1	0.019937285	0.986974364
ATXN2L	0.019879609	0.989253732
NCOA7	-0.019855043	0.985055894
SDHB	0.019843503	0.98631073
UBN2	0.019656338	0.980448152
ENOX1	0.019653705	0.977867487
BLZF1	-0.019615569	0.989253732
BOD1L	0.019442525	0.989210394
DCC	-0.019441947	0.971049515
ATP8A1	0.019420286	0.980213384
NPFFR2	0.019413473	0.990351862
PDCD2	0.019300222	0.988606376
TEKT5	0.019294744	0.988246745
NTN1	0.019277298	0.98819374
ORC2L	-0.019271643	0.981925107
NEK1	-0.019250348	0.984950434
ATF7IP2	0.01919667	0.985394552
LMBR1	-0.019102059	0.980471633
SLA2	0.019056647	0.985535626
TRIP12	-0.019027764	0.980392548
RPUSD4	-0.018937309	0.989495111
UHRF1BP1L	-0.018794606	0.980857907
SPARCL1	0.018678126	0.989253732
HERC4	-0.018559638	0.980213384
WDR93	0.018487866	0.989253732
PRKCG	-0.018291611	0.983965193
EEA1	-0.018241357	0.983401076
RNF150	0.018234728	0.982036509
FAM189A1	-0.018195606	0.97764905

UBOX5	0.018117915	0.986974364
TMEM108	0.017882901	0.971285439
ZDBF2	0.017823333	0.985791743
ST6GALNAC5	0.017796923	0.975409703
PAR-SN	0.017786892	0.980471633
FAM63B	-0.017782277	0.986974364
ESYT2	-0.017439177	0.988606376
MIR548N	0.017371284	0.984485141
TTC3	0.017289207	0.984426615
CHD1L	0.017282025	0.988246745
DSTN	-0.017220437	0.986272679
NCRNA00183	0.017191098	0.978080098
IFT88	-0.017003933	0.985055894
DACH1	0.016871446	0.986272679
BEND6	0.016863139	0.983965193
SLC35D1	0.016846435	0.986974364
LCORL	0.016765334	0.981925107
IQCA1	0.01672353	0.984950434
KPNA6	-0.016632243	0.988755735
SYT9	0.016586263	0.98631073
NNAT	0.016564687	0.988246745
SLC25A36	-0.016538868	0.990650669
PEBP4	-0.016492998	0.991324521
SYN3	-0.016197154	0.981925107
C20orf196	0.01617539	0.986974364
SCN1A	0.016149065	0.983317567
CTTN	0.016123577	0.991324521
ZNF385B	-0.016000307	0.980448152
ODZ3	-0.015955565	0.985055894
NCALD	-0.015911642	0.986702177
AKIRIN2	-0.015777414	0.989253732
KRR1	0.01575117	0.986702177
SULF2	0.015644901	0.983965193
KCNMB2	-0.01559347	0.983022733
MAPK8IP3	-0.015495761	0.990519622
RGS22	0.015426328	0.990351862
YEATS2	0.015375282	0.986974364
GATS	0.015197856	0.986974364
CRTC1	0.015170639	0.990351862
GNL1	0.015150638	0.990351862
PLXDC1	-0.015037592	0.990351862
TYSND1	-0.014959855	0.990351862
ITCH	0.014898947	0.984426615
ZNF177	-0.014744895	0.984304519

NFKB1	-0.014593106	0.990159548
AP1G2	-0.014592121	0.990351862
SCFD1	-0.014566696	0.986974364
DZIP3	-0.014542997	0.986974364
DDOST	-0.014440148	0.990159548
ARMC9	0.014400959	0.986702177
CHST9	-0.014359667	0.980448152
DGKG	0.01433658	0.980132631
RBL1	0.014183696	0.986702177
ATP6V1H	0.014165718	0.986702177
DPH5	0.014105376	0.992438849
ADCY9	0.014097599	0.985616838
ACCN1	-0.014094105	0.980593465
CAMSAP1L1	0.013980146	0.984909813
PEX5L	-0.013906212	0.981925107
ASCC1	0.013897578	0.986974364
SENP6	0.013669188	0.981925107
LOC147670	0.013655491	0.990351862
UBA6	0.013635544	0.990351862
REPIN1	0.013546274	0.992438849
TSEN2	-0.013543265	0.991324521
TIA1	0.013509227	0.989253732
SUSD4	0.013465303	0.985055894
MFSD6	0.013434453	0.991287652
KCNK10	0.013399971	0.988606376
RNF170	-0.013370165	0.990351862
FLJ39080	0.013311991	0.989253732
GATC	-0.013268892	0.991324521
TPST1	0.013153801	0.985791743
LOC100272228	-0.013088078	0.991324521
SIK2	-0.013018847	0.988246745
TBC1D20	0.01292885	0.990351862
SUZ12P	0.012786809	0.990351862
BAZ2B	-0.012597284	0.985535626
PDE6D	-0.01240541	0.988011983
FBXO15	-0.0123087	0.991673696
HMG20A	0.011956754	0.991324521
GRIK4	0.011872946	0.986974364
CPEB3	-0.011811265	0.983965193
CHSY3	0.011732573	0.983965193
ZNF540	-0.011663164	0.988606376
ARHGEF10L	-0.0116618	0.990351862
PTPRR	-0.011474269	0.986974364
SCFD2	-0.011313772	0.986702177

ANKRD19	0.011289801	0.989253732
CBFA2T2	0.011268219	0.985055894
IQGAP1	0.01122649	0.992359642
FAM149A	0.010820455	0.993124419
BAGE2	0.010772535	0.99427106
BAGE3	0.010772535	0.99427106
BAGE4	0.010772535	0.99427106
BAGE5	0.010772535	0.99427106
POLR2K	0.01076421	0.992656446
ZSWIM5	-0.010701414	0.989253732
NCS1	-0.010656559	0.990351862
PPA2	-0.010388663	0.990351862
RBM27	0.010191041	0.993352778
FMNL2	-0.010187733	0.985055894
LOC158696	0.010166639	0.992438849
RAP1GAP	0.01012377	0.993352778
GOLGB1	-0.010108652	0.992359642
LOC100302652	-0.010030282	0.984174609
STRN	-0.009980997	0.989253732
FAM19A1	-0.009931507	0.987941842
PRMT2	-0.009902722	0.991324521
FHOD3	0.009841295	0.987941842
SBF2	0.009715232	0.982260388
MTF2	-0.009606578	0.992647534
ME3	-0.009577035	0.989253732
EVI5	0.009519406	0.989253732
CCDC7	-0.009511948	0.991673696
SCAI	0.009409234	0.990351862
PIK3CB	-0.009380381	0.990991941
SLC25A43	0.009235789	0.994973961
GBF1	0.00921179	0.992583944
CTSS	-0.009191528	0.994284412
B3GALNT1	-0.009137924	0.992438849
SLC35F5	0.009105869	0.992583944
USP11	0.009098051	0.995363606
DDB1	-0.008980228	0.992438849
STK39	-0.008913542	0.991324521
ESR2	0.008811658	0.99427106
PLXNA4	-0.008727644	0.988755735
TMEM87A	0.00867299	0.991814575
RALBP1	0.008633776	0.992438849
LMO7	0.008590927	0.991814575
GLO1	-0.008580317	0.99427106
EIF2B3	0.008460187	0.991121795

PAK3	-0.008349393	0.991324521
HAT1	0.008345612	0.994893314
ZNF323	-0.008165067	0.992438849
TGFA	-0.008008377	0.99427106
SUGP2	0.007985893	0.994973961
GPRASP2	-0.007904927	0.99427106
PUS10	0.007891575	0.995879532
FAM134B	-0.007807547	0.991324521
FNIP2	0.007706396	0.99427106
SEC62	-0.00762133	0.992647534
LOC283856	0.007384822	0.992647534
SYT7	-0.007378139	0.992438849
LRRC37B	-0.007316574	0.994284412
ANGEL1	0.007211949	0.994284412
INTS2	0.007195887	0.996163663
ZNFX1	0.007171969	0.992438849
PPFIBP1	-0.007116588	0.992369007
TTLL5	-0.007071568	0.992647534
PHIP	-0.006894965	0.992438849
PTPRT	0.0066827	0.992359642
HYDIN	0.006569577	0.983965193
KLHL3	-0.006494045	0.99490897
SARM1	-0.006491781	0.992647534
FLJ43663	0.006419767	0.993352778
C4orf45	-0.006415717	0.995268156
MYH11	-0.00635719	0.996083109
MED24	0.006357007	0.995268156
TCF4	-0.006318939	0.990519622
CEP68	-0.006306716	0.996431833
PBX4	-0.006191595	0.996160351
RASAL1	-0.006138345	0.996141021
HSP90B1	0.006121884	0.996275372
PTPN4	0.006057753	0.992438849
RAB33B	-0.006016314	0.996163663
C22orf30	-0.006006748	0.996163663
ABCA17P	0.005834545	0.996141021
ZNF445	0.005695323	0.995190046
RNF216	0.005685485	0.994693367
UBE2J1	0.00560463	0.996275372
DDX51	0.005507011	0.996275372
RHPN2	0.005475471	0.996275372
MATR3	-0.005363926	0.996431833
PRKCE	-0.005271151	0.992417291
BMPR1A	-0.005233482	0.992963223

NCOA3	0.005170999	0.996431833
SORT1	0.004783075	0.994329994
NOL10	-0.004677967	0.996163663
CEP164	0.004595655	0.99745889
CAB39L	-0.004595538	0.996163663
RBM6	-0.004562439	0.996275372
ECE1	-0.004351242	0.997080923
TMTC3	-0.004285828	0.997080923
PCSK2	-0.004211691	0.994973961
TBC1D1	-0.004134694	0.996163663
MARK2	-0.004071166	0.996163663
AMBRA1	0.003995266	0.995268156
FAM117B	-0.003774902	0.996431833
WIPI2	-0.003764452	0.99822624
EPB41L4B	-0.003656527	0.996977653
C9orf130	0.00365308	0.99745889
NF2	-0.003606212	0.997798095
DOCK10	0.003517575	0.996083109
AGPAT6	0.003412297	0.998539928
C2orf86	-0.003407272	0.992438849
SFXN1	0.003150745	0.99745889
ZNF280D	0.003121176	0.996431833
ASPH	0.003074094	0.99745889
ARHGEF17	0.003015993	0.998539928
DNAJC15	0.002886037	0.998547129
KIF5C	-0.002827997	0.998539928
SLC2A11	0.002636523	0.99822624
CCDC148	0.002628605	0.996431833
GABRB1	0.002592875	0.996163663
PXMP4	-0.002498132	0.998539928
GRID1	0.002484004	0.996431833
ZNF500	0.002421676	0.999272806
TET2	-0.002324873	0.99886568
ODZ4	0.002305435	0.996431833
THSD4	-0.002203394	0.99886568
DNER	0.002179838	0.99745889
NCRNA00182	0.002102955	0.996601423
NT5C2	-0.002093893	0.998539928
FHIT	0.001944651	0.99745889
ABLIM3	0.00188377	0.99886568
PDHX	0.001833326	0.999272806
LOC647107	0.001739065	0.999586057
BRP44L	-0.001688246	0.9993549
PSMD1	0.001259627	0.999367555

STAU2	-0.00118993	0.998571559
UBE2E2	-0.001177073	0.998539928
DENND5A	-0.001157008	0.9993549
TCEAL5	0.000987079	0.999738621
PCM1	0.000899804	0.999272806
RTN3	0.000896821	0.999586057
WAPAL	0.000874389	0.999738621
PLXDC2	-0.000745993	0.999586057
PIGU	-0.000715238	0.999738621
USP31	-0.00064952	0.999738621
WFDC8	-0.000513822	0.999738621
NALCN	0.000511264	0.999586057
C2orf67	0.000496202	0.999738621
CCDC104	-0.000491057	0.999738621
ETV6	0.000458549	0.999738621
ART3	0.000407604	0.999738621
NKAIN3	-0.000252369	0.999738621
OBSL1	-0.000225458	0.999738621
KIAA1310	-0.00020465	0.999738621
POLN	0.000194972	0.999738621
DNAJA1	0.000178424	0.999738621
SLC7A14	-0.000135085	0.999738621
BCL2L1	0.000103289	0.999738621
KIAA1217	9.23571E-05	0.999738621

DEG for GSE160587

	log2FoldChange	padj
Pcdhb19	10.86327829	1.46961E-15
44622	-0.054913715	0.949550088
44623	-0.049343249	0.985673938
44624	-0.092181253	0.91462326
44625	0.049733907	0.956739982
44626	-0.085769855	0.736130784
44627	-0.136612347	0.87564489
44628	-0.437703973	0.874252042
44629	0.011941798	0.994806961
44630	-0.767912111	0.724910334
44631	-0.25150948	0.911631946
A2m	-0.625025428	0.624713015
Alms1-ps2	-7.932831579	1.77461E-05

A3galt2	-0.962283796	0.810908327
A4galt	-0.061080345	0.989840188
A4gnt	0.990689139	0.871869598
AA386476	0.130762555	0.984328758
AA387200	-0.026593539	0.996431931
AA413626	0.52206441	0.905361361
Cacng1	7.530918709	0.00021266
AA414992	0.421312367	0.959659722
AA465934	0.264730371	0.938414123
AA467197	-0.703745031	0.957499962
AA474408	-0.161242791	0.959276014
AA543186	0.137072327	0.985742667
AA914427	0.449517011	0.953505913
AA986860	-0.108904603	0.962648047
Aaas	0.02459899	0.987549838
Aacs	-0.038163672	0.968005385
Aadat	0.159080373	0.985673938
Aaed1	-0.038038659	0.984232465
Lrrc30	6.631461605	0.020243742
Aak1	0.040317189	0.911631946
Aamdc	0.030462876	0.984232465
Aamp	0.027072153	0.972218452
Aanat	0.225731051	0.974962198
Aar2	-0.022295527	0.98459388
Aard	-0.292347977	0.806485043
Aars	0.087499817	0.742849809
Aars2	-0.09235879	0.91462326
Aarsd1	-0.027130383	0.984011244
Aasdh	0.036207653	0.985446046
Aasdhppt	0.169498539	0.936031628
Aass	0.317117214	0.537842333
Aatf	0.118861754	0.897878088
Aatk	-0.16861978	0.603499717
AB041806	-0.034957085	0.99527876
AB124611	0.215992292	0.985742667
Abat	-0.063835679	0.911631946
Abca1	0.067634823	0.949607377
Fgg	6.490962794	0.009690442
Kcna7	6.387473974	0.031985521
Abca17	0.241706961	0.968977192
Abca2	-0.044297851	0.964941885
Abca3	-0.13021742	0.578812716
Abca4	-0.140703841	0.971138085
Abca5	-0.100067487	0.828907376

Abca6	0.06165608	0.989200732
Abca7	-0.006328522	0.99527876
Abca8a	-0.025499789	0.987326705
Tbx21	6.245743797	0.034353121
Abca9	-0.257447841	0.809980757
Abcb10	0.107593076	0.837755777
S100a7a	-6.174798848	0.03331059
Abcb1a	0.108249238	0.911631946
Abcb1b	0.317385396	0.657504489
Abcb4	0.128122784	0.891853883
Abcb6	0.043641669	0.983479691
Abcb7	-0.044232587	0.962648047
Lta	-6.05870069	0.042984015
Abcb9	-0.215849248	0.580757434
Abcc1	0.017520383	0.99100709
Abcc10	0.130223393	0.91462326
Abcc12	0.349583084	0.971661054
Abcc2	-0.515370761	0.962648047
Abcc3	0.430723647	0.873797321
Abcc4	0.133684017	0.884978062
Abcc5	-0.06572989	0.879401677
Abcc6	-0.615883414	0.814814675
Abcc8	-0.026636564	0.972387414
Abcc9	0.230086121	0.759486652
Abcd1	-0.004803913	0.996094752
Abcd2	-0.154560286	0.806077586
Abcd3	0.002617658	0.99765039
Abcd4	-0.080925851	0.941515087
Abce1	-0.055595883	0.911631946
Abcf1	-0.025781397	0.981502501
Abcf2	0.043572539	0.939060335
Abcf3	-0.005319794	0.99527876
Abcg1	-0.126247633	0.732729469
Abcg2	0.152803956	0.7851601
Abcg3	0.462217157	0.946239011
Abcg4	-0.153449713	0.656246996
Abhd1	0.079695542	0.985735386
Abhd10	-0.045233833	0.980339705
Abhd11	-0.177972978	0.656710793
Abhd11os	0.073306656	0.991996237
Abhd12	-0.185980276	0.590745439
Fam81b	-6.0436381	0.079050693
Abhd13	-0.034680005	0.965520236
Abhd14a	-0.006840798	0.99527876

Abhd14b	-0.069304704	0.955993235
Abhd15	0.381965549	0.873753967
Abhd16a	0.026454055	0.970673196
Tslp	-5.968528385	0.087140878
Abhd17a	0.121225468	0.807017299
Abhd17b	0.035648691	0.973100313
Abhd17c	-0.021739827	0.985446046
Abhd18	0.118344106	0.900700653
Abhd2	-0.119450002	0.623042338
Abhd3	0.02124694	0.985742667
Trim75	-5.912603122	0.068660675
Abhd5	-0.027797298	0.982914099
Abhd6	0.153422557	0.882824434
Abhd8	0.018229342	0.98459388
BC050972	-5.901753272	0.063948885
Adh6b	-5.773735608	0.082752362
Sp5	-5.710053157	0.151118156
Abi3bp	-0.285077587	0.969755848
Abl1	-0.828472072	0.539315375
Abl2	-0.071671306	0.837695826
Ablim1	-0.107488406	0.873797321
Ablim2	-0.029164722	0.968005385
Ablim3	0.176744619	0.758595939
Frmd8os	5.708927603	0.117545621
Hsh2d	5.697182629	0.130213261
Abracl	-0.102337803	0.945647427
Abraxas1	0.250750495	0.831697838
Abraxas2	-0.025527858	0.97487264
Abt1	0.19028187	0.521202205
Abtb1	0.089927132	0.898474424
Abtb2	0.116576608	0.929503395
Rnase13	-5.64292587	0.130213261
AC034116.4	0.813328596	0.905559486
AC034116.5	-0.712492134	NA
AC061963.1	-0.144746399	0.964176371
AC078895.1	-0.040563356	0.985742667
AC078895.2	0.366499236	0.991996237
AC079441.1	0.477936123	0.985742667
AC083895.1	-0.102575079	0.993380259
AC084822.2	0.678469857	0.957499962
AC087233.2	-0.362331859	0.976978183
AC087802.1	-0.691615224	0.984305885
Cldn20	5.632954805	0.128640258
AC087898.2	-0.578822914	0.83140737

AC087898.4	-0.753063277	0.962648047
AC087898.5	0.300105859	0.985293518
AC090479.2	-0.150090308	0.972374093
Adipoq	5.596075867	0.136043752
AC091463.1	0.136560391	0.972025261
Tarm1	-5.581879265	0.122206254
AC098880.2	-0.367886758	0.821998633
AC098883.1	0.582818939	0.978548958
AC100751.1	-0.537933047	0.98526072
AC102055.1	0.772428866	0.891972665
AC102342.1	-0.052639953	0.99527876
AC102342.2	0.485021018	0.638244169
AC102496.1	0.15432044	0.724910334
AC103939.1	0.415389993	0.962648047
AC105304.4	-0.99927207	0.574956844
AC107792.1	0.767429072	0.974962198
AC107792.3	0.161310044	0.964011524
AC109204.1	-0.227318354	0.994960308
Hsf3	5.511219297	0.16785722
AC110166.2	-0.461439707	0.91462326
AC110262.3	-0.689370265	0.980731359
AC110534.2	-0.17031772	0.981392364
AC110534.3	0.465719679	0.940059526
AC110534.5	-0.008665433	0.99527876
AC110562.1	0.889101121	0.966213117
Gpr20	5.495764581	0.209384449
Tspo2	5.378150219	0.245096108
AC113006.1	0.48924779	0.852010446
Cnpy1	-5.352744576	0.211336401
AC113595.1	-0.966871468	0.895520088
Spr-ps1	5.323116492	0.240817904
AC114585.1	-0.000399694	0.999242708
AC114990.3	0.340902986	0.928957615
Mylf-ps	5.31581711	0.148468014
AC115954.1	0.105726927	0.790773854
AC117232.1	0.274127064	0.974823941
Smpx	5.311329899	0.002275534
Olfr524	-5.298533524	0.208683335
AC118542.3	-0.064122719	0.993380259
AC119957.1	-0.825433493	0.91462326
Olfr267	5.271752048	0.245096108
Glipr1l3	-5.242170368	0.319241271
AC121151.1	0.316522944	0.886647768
AC121560.1	0.687894553	0.942274311

AC121783.1	0.977229687	0.865486573
Tnfsf11	5.120122055	0.314194702
AC122253.2	-0.057371008	0.98459388
AC122253.3	-0.541834332	0.973100313
AC122306.1	-0.197716518	0.981392364
AC122335.1	0.114606964	0.99527876
AC122399.1	-0.067299481	0.99527876
Dlx4	-5.084865901	0.32696666
AC122413.2	0.371815597	0.97487264
Fam187b	-5.057683041	0.322138936
AC122481.1	0.02674286	0.997258205
AC122481.2	0.016671637	0.99765039
AC122487.1	-0.191457469	0.985742667
AC122487.2	-0.374084555	0.974844452
AC122818.1	-0.026930655	0.995500778
AC122818.3	0.240371053	0.97607383
AC122818.4	-0.167898001	0.987342614
Yy2	4.964509412	0.022882132
AC122901.1	0.08251524	NA
AC123659.1	0.519765268	0.8549794
L3mbtl4	-4.93501998	0.387432606
AC123951.1	0.81875952	0.975631569
AC123956.1	0.244667185	0.93912725
AC124237.1	-0.849146753	0.975831674
mt-Ts2	4.821054301	NA
Dlx6os2	4.865954049	0.467019925
AC124561.1	-0.612504329	0.92886025
AC124681.1	-0.177189289	0.97487264
AC124739.1	0.516182	0.975831674
AC124739.2	-0.331046914	0.962648047
AC125351.1	-0.072745715	0.962648047
Irs4	4.843565888	0.422728382
AC125396.2	-0.070880297	0.992040075
AC126030.1	0.858763019	0.948520404
AC126040.2	0.06086681	NA
AC126055.1	0.395642525	0.984328758
AC126250.1	0.123688901	0.957485565
Nfe2	-4.779528226	0.434762828
AC126280.1	0.213147977	0.988034453
AC126438.1	-0.254752204	0.991438757
AC126455.1	0.246578735	0.994960308
AC126457.1	0.091037588	0.948104382
AC126807.1	0.042878042	0.993290512
Tmem30b	-4.685642123	0.465918106

AC126937.2	0.060189779	0.987342614
Cd209f	4.525638315	0.544661714
AC127341.3	-0.572560693	0.968005385
AC127341.4	0.624920557	0.98526072
AC127341.5	0.536884414	0.895520088
AC120779.1	4.394142218	0.596729594
AC129186.1	-0.075075099	0.98691726
AC129328.1	0.007750185	0.99765039
Apom	-4.669878111	0.503682499
AC130718.1	0.076330269	NA
Cyp2d26	4.654672433	0.504583609
AC130815.1	-0.229643166	0.985673938
AC130815.2	0.325239169	0.684977158
AC131323.2	0.021910659	0.997115148
AC131339.2	-0.282936476	0.927659499
AC131712.1	-0.094384055	0.992385525
AC131796.1	-0.040939898	0.997001768
AC131796.2	-0.936246793	0.630888733
AC131800.1	-0.144125474	0.981392364
Lypd8	4.64615156	0.499392203
Gmfg-ps	4.257126898	0.6883862
AC132353.2	-0.324387313	0.971876411
AC132367.1	0.18662902	0.98526072
AL732309.1	4.604913581	0.018059921
AC132910.1	0.261259464	0.964176371
Xirp2	4.57945587	0.456330081
AC133488.1	-0.031577203	0.989640643
AC133505.1	-0.288486574	0.98043358
AC133517.1	-0.383090541	0.985742667
AC133525.2	-0.01329979	0.997258205
Hist1h3e	3.876825689	0.529257466
Lipm	3.818977327	0.519524068
AC134468.1	-0.738695219	0.74463793
AC134560.2	-0.045957726	0.997375151
AC134560.3	-0.017387191	0.99527876
AC135509.1	0.397572724	0.980325883
Mylk2	3.796357034	0.623438925
AC138768.3	-0.227794412	0.98459388
AC138768.4	0.109110311	NA
AC139131.1	0.21030801	0.980226283
AC139579.1	-0.482381632	0.985293518
AC154542.1	3.685787241	0.74463793
C5ar2	3.640081799	0.6883862
AC140267.1	0.122480034	0.980339705

AC140278.1	0.417763331	0.947033215
Ppp1r3a	3.590819339	0.5141747
AC140300.2	0.83508633	0.819778986
AC140413.1	0.170256394	0.993103295
Mir3101	4.332660753	0.126707005
Epo	3.558017588	NA
Yipf7	3.526965083	0.536206675
AC144797.2	0.329207278	0.985742667
AC144860.1	-0.085293376	0.962648047
AC144860.3	-0.668483475	0.962648047
AC144860.4	-0.150490519	0.985293518
AC167363.1	3.52561571	NA
AC148004.1	-0.088848925	0.985673938
AC148004.2	0.463249147	0.837755777
Abra	4.298991506	0.066208389
AC150314.1	-0.973154472	NA
Plet1os	4.263552488	0.196410522
AC151275.1	-0.824228716	0.97487264
AC151284.1	0.118104505	0.965520236
AC151284.2	-0.356988803	0.6883862
Rnf183	3.449555607	0.795861282
AC151294.1	0.347758943	0.962648047
AC153140.1	-0.035130989	0.99527876
AC154178.2	0.013427577	0.998010523
Lcn2	-4.206392546	0.397244359
AC154200.1	-0.119733752	0.985742667
AC158568.2	3.440021667	0.6883862
AC154232.1	-0.221999236	0.990661552
Cldn22	4.199597205	0.37706439
Trdn	4.004252434	0.066208389
AC154247.1	0.171416466	0.982408617
AC154306.1	-0.559852403	0.974809035
AC154343.2	0.226585703	0.974823941
AC154366.4	-0.199666458	0.975800973
AC154378.1	-0.109900186	0.969755848
Platr22	3.326877437	0.614283919
AC154412.1	-0.920610967	0.972218452
Slc22a18	-3.950248568	0.103906677
Emilin3	3.582248678	0.334735887
AC154458.1	-0.272007808	0.99100709
AC154486.3	-0.75757198	0.836538337
AC154492.2	0.384063096	0.959276014
AC154507.2	0.648586366	0.965520236
CT009627.1	3.228679069	0.629280869

AC154654.1	-0.797917244	0.850260513
AC154667.1	-0.060433396	0.994635674
AC154683.1	-0.025498559	0.993290512
AC154707.1	0.085889055	0.975831674
AC154760.1	0.064714044	0.983977391
AC154763.2	-0.99086925	0.770166577
AC154766.4	-0.012743806	0.995411429
AC154779.2	-0.904043206	NA
AC154779.4	-0.25697911	0.982392834
Nctc1	3.454879412	0.408827768
Trim54	3.451208212	0.095681074
Pcdhga9	3.182894946	NA
AC158605.1	0.12527705	0.984328758
Acpp	-3.442238377	0.386030517
AC158985.1	0.054350623	0.99527876
AC159282.1	0.042419966	0.99527876
AC160336.1	-0.005037757	0.997529192
AC160637.1	0.078370844	0.945182261
AC161166.1	-0.39878366	0.895198797
AC161376.2	-0.255353355	0.938544177
AC161438.1	0.504528282	0.959276014
AC161607.1	0.063496552	0.981392364
Ampd1	3.393464595	0.000324248
Ckmt2	3.105096939	0.618152081
Slc39a5	3.089016533	0.754457658
AC163032.1	0.034412816	0.985446046
AC163399.1	0.828474644	0.738901675
AC163629.2	0.495565749	0.985742667
AC163633.1	0.635398462	0.97469529
AC163634.1	0.797134009	0.783611405
AC163635.1	-0.175064672	0.994960308
AC163694.1	0.359951844	0.722060735
AC164314.1	0.164674263	0.957837415
AC164314.2	0.086035812	0.996094752
AC164424.1	-0.184237623	0.969755848
AC164431.1	0.489444809	0.975831674
Cnksr1	3.08235663	0.655744783
AC164546.2	-0.314869508	0.97487264
AC164647.1	-0.318056089	0.987342614
AC165075.1	0.462611198	0.98187819
AC165079.1	0.16688293	0.813744313
AC165151.1	-0.492504011	0.956072972
AC165153.1	0.204518745	0.993380259
Wt1	3.381366571	0.425190393

AC165271.1	-0.22381017	0.703372057
AC165271.2	0.45429006	0.938544177
AC165278.1	0.018628195	0.991438757
Saa1	3.020634726	0.522054961
AC165953.2	-0.004276312	0.997375151
AC165960.2	-0.679058694	0.962648047
AC165961.3	0.46686971	0.985742667
AC166058.1	-0.115265081	0.985742667
Rps2-ps9	3.001564402	0.869316117
AC166110.4	0.078416368	0.98043358
AC166114.1	0.489439101	0.949550088
Fasl	-3.341116089	0.392811348
AC166341.1	0.211893966	0.986246396
AC166832.3	0.120488106	0.991996237
Xlr4a	2.956340085	0.628806842
AC167363.2	0.693943602	0.953505913
AC168050.2	0.1443523	0.971661054
AC168220.3	0.829447605	0.964941885
AC168306.1	0.98775971	0.812431087
AC169506.3	0.22309598	0.985673938
AC169509.1	0.175374401	0.970348278
AC169675.1	0.7730176	0.713014362
AC170998.1	0.020672042	0.99765039
AC171111.1	0.263356107	0.53452841
AC171205.1	0.443961006	0.948104382
AC173344.1	0.087836999	0.968005385
AC174678.1	0.07854519	0.974852055
Myot	3.304327258	0.0029882
AC183095.1	-0.23415146	0.707520358
Fbxo40	3.298148129	0.037791947
AC183097.2	-0.287723842	0.609927197
AC238676.1	-0.239889001	0.983091673
Acaa1a	-0.143746304	0.624220551
Acaa1b	0.0772407	0.982309415
Acaa2	0.016157107	0.99042585
Acaca	-0.05726131	0.903795193
Acacb	0.318227582	0.663732405
Acad10	-0.000855304	0.999242708
Acad11	-0.074071684	0.962648047
Acad12	-0.044536263	0.98340069
Acad8	0.17027166	0.722843611
Acad9	0.041305999	0.962648047
Fam180a	-3.280613729	0.080124665
Acadm	0.108082068	0.856163928

Acads	0.097573653	0.962648047
Acadsb	-0.165285858	0.544361994
Acadvl	0.118229326	0.848380246
Acan	-0.19743215	0.876359276
Fetub	3.206421075	0.483438211
Acap2	0.003097328	0.995411429
Acap3	-0.144578583	0.590745439
Acat1	-0.0238785	0.968005385
Acat2	-0.024463391	0.974962198
Acat3	0.058152213	0.971661054
Acbd3	0.079925718	0.87847909
Acbd4	0.054841418	0.976374627
Acbd5	0.181583098	0.910328122
Acbd6	0.085327272	0.939052665
Bglap3	3.195194072	0.294902721
Accs	0.322300052	0.6883862
Accsl	-0.047503894	0.996325937
Acd	0.002950028	0.997241386
Ace	-0.154951651	0.962648047
Ace2	0.097149972	0.975831674
Tnnc2	3.160104975	0.016579717
Acer2	0.090017698	0.963500155
Acer3	0.088408761	0.89703631
Ache	0.092222648	0.962648047
Acin1	0.061713247	0.962648047
Ackr1	0.147790436	0.852010446
Ackr2	-0.83103965	0.740833211
Ackr3	-0.003630623	0.997115148
Ackr4	-0.54464652	0.911631946
Acly	-0.021367189	0.974823941
Acmsd	0.629028846	0.984328758
Aco1	-0.034873656	0.973100313
Aco2	0.043463855	0.902496496
Acod1	-0.398351467	0.959778108
Acot1	-0.001638469	0.99900438
Acot10	-0.123508611	0.99527876
Acot11	0.142861856	0.623438925
Tmem182	3.149254659	0.089891481
Cox8b	3.075650911	0.161844023
Acot2	0.067127868	0.956922504
Acot3	0.629311114	0.911276003
Acot4	-0.016265491	0.993103295
Acot5	-0.01234818	0.99527876
Acot6	0.150646223	0.781565911

Acot7	0.010779307	0.991438757
Acot8	-0.136134087	0.869316117
Acot9	-0.120877845	0.872612157
Acox1	-0.02348047	0.963619706
Als2cr12	-2.987557803	0.482876267
Acox3	-0.043375576	0.968005385
Acoxl	0.770333028	0.975831674
Acp1	0.05182787	0.932673884
Acp2	0.013835628	0.985673938
Acp5	-0.036739648	0.99527876
Acp6	-0.074424462	0.938939871
Acp7	-0.374128519	0.90701663
Zfp986	2.739958588	0.875007504
Acr	0.094867347	0.985742667
Acrbp	-0.067698237	0.981392364
Acsbg1	-0.003678881	0.99527876
Acsbg2	-0.121671444	0.99527876
Acsf2	-0.173010416	0.710772024
Acsf3	0.016628469	0.985742667
Acsl1	-0.081603591	0.985293518
Acsl3	0.102786628	0.729048067
Acsl4	-0.097680438	0.758032534
Acsl5	0.030132643	0.965520236
Acsl6	-0.109111996	0.708001635
Acsm3	-0.077651401	0.991996237
Acsm5	-0.70470136	0.968005385
Acss1	-0.071226387	0.911631946
Acss2	-0.048886067	0.936905058
Acss2os	0.217023979	0.984748462
Acss3	0.029585283	0.99527876
Myl1	2.957263379	0.060781486
Acta2	0.097480118	0.968005385
Mylpf	2.933924791	0.028541628
Actc1	2.902709218	0.004094616
Actg-ps1	-0.026325767	0.99527876
Actg1	-0.281826856	0.860521845
Actg2	-0.589995848	0.98302504
Actl10	0.179664233	0.974829384
Actl6a	0.147130575	0.898474424
Actl6b	0.097407408	0.807581821
Actl9	0.508459205	0.971876411
Actn1	-0.107446138	0.65012378
Actn2	0.256188633	0.573101161
Olfr1419	2.722325598	0.836506511

Actn4	-0.037198452	0.958299088
Actr10	-0.013279343	0.984328758
Actr1a	0.024975973	0.968005385
Actr1b	0.029787356	0.952034929
Actr2	-0.112946406	0.69189201
Actr3	0.150841817	0.959276014
Actr3b	-0.016430203	0.985229869
Actr5	0.099360438	0.95043295
Actr6	0.037483888	0.971661054
Tmem233	2.893507545	0.217372573
Actrt3	0.243044341	0.950460219
Acvr1	-0.067738952	0.918235054
Acvr1b	0.075544578	0.852010446
Acvr1c	-0.157047568	0.780528197
Acvr2a	0.017344103	0.985947768
Prss45	2.720231682	0.531625233
Acvrl1	0.029398211	0.991438757
Acy1	-0.04267901	0.985673938
Acy3	0.065172052	0.985742667
Acyp1	0.090807584	0.910328122
Fhad1os2	-2.836659277	0.374915032
Myog	2.706823389	0.659674918
Ywhaq-ps2	2.702473065	0.803910915
Adal	-0.238951298	0.520182905
Cd79a	-2.800556018	0.436138179
Adam11	-0.07281753	0.989640643
Adam12	-0.281602057	0.578812716
Adam15	-0.007434537	0.994960308
Adam17	0.154464002	0.95126064
Adam18	-0.058176706	0.99527876
Adam19	-0.136043166	0.889972515
Adam1a	-0.423415016	0.874252042
Adam1b	-0.403535413	0.777427974
Adam21	-0.130629426	0.981392364
Adam22	-0.049625379	0.882824434
Mb	2.787412426	0.000134553
Adam28	-0.314394075	0.985742667
Adam3	-0.92792968	0.974823941
Adam32	-0.519121787	0.951949091
Boll	2.69307124	0.868415875
Adam4	-0.05931281	0.99527876
Adam5	-0.685126458	0.962648047
Adam7	-0.508322367	0.981746088
Adam8	-0.030570947	0.992152669

Adam9	0.179618155	0.869316117
Gpha2	2.687553681	0.841017002
Adamts1	0.215086686	0.555615011
Adamts10	0.030212647	0.985446046
Adamts12	-0.9663019	0.90358675
Adamts13	0.461568754	0.864150749
Adamts14	0.061040876	0.984232465
Adamts15	-0.108362991	0.962648047
Adamts16	0.094002855	0.985742667
Adamts17	-0.080560607	0.962923888
Adamts18	-0.453801804	0.912042382
Adamts19	0.660784054	0.980731359
Myh2	2.763871597	0.082752362
Tcap	2.74981245	0.00023895
Adamts3	-0.073114063	0.968005385
Adamts4	-0.26775963	0.564375854
Adamts5	-0.617708651	0.821998633
Adamts6	-0.243186131	0.785603619
Adamts7	-0.331288397	0.962648047
Adamts8	0.049975047	0.984748462
Adamts9	0.093975373	0.91241376
Adamtsl1	-0.135572842	0.932673884
Adamtsl2	0.139728881	0.940963923
Adamtsl3	-0.335327042	0.952385926
Adamtsl4	-0.250877999	0.815987743
Adamtsl5	0.149544435	0.962648047
Adap1	-0.042922415	0.932673884
Adap2	-0.030519655	0.991559279
Adap2os	-0.322991466	0.962648047
Adar	-0.088930871	0.90358675
Adarb1	-0.159019789	0.640856315
Adarb2	0.137937878	0.874252042
Adat1	-0.167620348	0.871869598
Adat2	0.127739812	0.906188425
Adat3	-0.430046297	0.91462326
Adck1	0.015066044	0.988034453
Adck2	0.079186839	0.903441375
Adck5	-0.039342113	0.980226283
Adcy1	-0.095898979	0.889972515
Adcy10	-0.064547166	0.99527876
Adcy2	-0.0641908	0.861366392
Adcy3	-0.128539286	0.783229859
Adcy4	-0.343208551	0.855904169
Adcy5	-0.047982384	0.924707867

Adcy6	0.000171035	0.999582233
Tnni2	2.743176673	0.079793019
Adcy8	0.010923814	0.99527876
Adcy9	-0.178859689	0.676637789
Adcyap1	0.468556183	0.627170228
Adcyap1r1	-0.008271732	0.993103295
Add1	-0.034379009	0.941315855
Add2	-0.023693363	0.968005385
Add3	-0.023692276	0.966213117
Adgb	-0.302628972	0.91462326
Adgra1	0.065127728	0.968497942
Adgra2	-0.428209329	0.779964055
Adgra3	0.001444303	0.998010523
Adgrb1	-0.066884094	0.8549794
Adgrb2	-0.027142768	0.962648047
Dhrs7c	2.737693388	0.386030517
Adgrd1	0.58471172	0.871869598
Adgre1	0.108308738	0.96607835
Olfr539	2.728328687	0.021592066
Adgre5	-0.070289696	0.97469529
Adgrf2	0.576259485	0.871869598
Adgrf4	0.134358559	0.96188696
Adgrf5	0.096152256	0.899097089
Adgrg1	-0.125133963	0.938615214
Adgrg2	0.341017552	0.861366392
Adgrg3	0.244200926	0.975631569
Adgrg6	-0.570360043	0.888921078
Adgrl1	-0.152705602	0.803910915
Adgrl2	-0.262538363	0.620124996
Adgrl3	-0.167995281	0.54720111
Adgrl4	-0.14866174	0.789386933
Adgrv1	-0.278705313	0.909243078
Adh1	-0.475255112	0.962648047
Adh5	0.064111375	0.89703631
Ckm	2.686458189	0.034088092
Adh7	-0.422686621	0.98612132
Adhfe1	0.130080087	0.848380246
H19	2.68519193	0.115146901
BC023105	2.680490488	0.492371645
Adipor1	-0.036532698	0.958085726
Tnnt3	2.61740904	0.037510114
Adk	0.102638372	0.745988863
Adm	-0.206728056	0.951468402
Adnp	0.16041049	0.842993113

Adnp2	-0.050945372	0.963666931
Ado	-0.090190459	0.767515266
Adora1	0.022191168	0.975831674
Adora2a	-0.155728152	0.973100313
Adora2b	-0.043863428	0.985742667
Adora3	0.139494457	0.97469529
Adpgk	-0.041960655	0.97487264
Adprh	-0.001554759	0.99765039
Mrln	2.667154905	0.69189201
Adprhl2	0.17385977	0.771684575
Adprm	0.057593106	0.965007958
Adra1a	-0.250619646	0.813552442
Adra1b	0.310862208	0.874252042
Adra1d	-0.167688086	0.571020232
Adra2a	-0.029603111	0.985673938
Cdca5	2.665342028	0.549579107
Adra2c	-0.265987941	0.593692285
Adrb1	0.01199866	0.99527876
Adrb2	-0.047152837	0.99165967
Adrb3	-0.19862455	0.974809035
Adrm1	0.067646376	0.955993235
Adsl	-0.106381978	0.920642026
Adss	-0.098762645	0.745879732
Car3	2.605805921	0.154914835
Aebp1	-0.922554633	0.706406235
Aebp2	-0.070344884	0.949607377
Aen	0.114892661	0.752462699
Aes	0.075981153	0.911631946
Paupar	2.664497685	0.871869598
Lmod2	2.663627429	0.779415976
Afap1	-0.17693323	0.708953765
Afap1l1	0.110577496	0.932673884
Afap1l2	-0.180181147	0.795563654
Afdn	0.093505719	0.946985353
Ptgfr	-2.599596017	0.403453477
Aff2	-0.108055336	0.962648047
Aff3	-0.038759961	0.967455948
Myh4	2.543034699	0.062880313
Afg1l	0.178969486	0.56488584
Afg3l1	-0.018192497	0.98526072
Afg3l2	-0.019408198	0.984328758
Afmid	0.399743914	0.903279667
Aftph	0.149506235	0.891264345
Aga	0.012116048	0.99527876

Agap1	0.470511219	0.892540517
Agap2	-0.041635028	0.915106125
Agap3	-0.005257706	0.99527876
Agbl1	-0.616894062	0.91507125
Agbl2	0.102276492	0.98187819
Agbl3	-0.13247953	0.946929376
Agbl4	0.046634187	0.976978183
Agbl5	0.247224494	0.806077586
Ager	0.314687641	0.932931799
Abca13	-2.488893025	0.509250303
Agfg2	-0.036093059	0.971138085
Aggf1	-0.013123327	0.987342614
Agk	-0.132808612	0.784009698
Agl	0.187043477	0.832980941
Agmat	0.72729507	0.968005385
Agmo	-0.140903227	0.876359276
Ago1	-0.056687521	0.91462326
Fsd2	2.458833188	0.064044233
Myoz1	2.431517378	0.011520526
Ago4	-0.030619595	0.97487264
Agpat1	0.129137255	0.871869598
Agpat2	0.062127247	0.992989855
Agpat3	-0.032579881	0.947745582
Agpat4	0.112906057	0.859228121
Agpat5	-0.004156705	0.99527876
Agps	-0.091461681	0.859685012
Mageb3	2.417954115	0.292282176
Agrp	0.882402464	0.740833211
Fcna	-2.390848892	0.220337275
Mybpc2	2.344128905	0.055853534
Agtr1a	0.353121614	0.962648047
Agtr2	-0.414808289	0.911631946
Scn4a	2.313300328	0.18316876
Ahctf1	-0.080414092	0.874252042
Ahcy	-0.044214499	0.962648047
Ahcyl1	0.033613409	0.956072972
Prickle4	-2.297886679	0.439287299
Ahdc1	0.241313682	0.965520236
Ahi1	0.101189648	0.928672524
Ahnak	-0.162717521	0.891972665
Ahnak2	-0.028986367	0.99527876
Ahr	-0.108269366	0.938939871
Ahrr	0.044069409	0.99100709
Ahsa1	0.111999766	0.620843342

Ahsa2	-0.276339501	0.80931022
AI115009	0.291689995	0.707777756
AI182371	0.152046356	0.985742667
AI197445	-0.457254739	0.925337971
Ppp1r17	2.264728288	0.431061843
AI427809	-0.457653097	0.984962414
AI429214	-0.021750228	0.99527876
AI463170	0.681226504	0.956737477
AI464131	-0.016117431	0.985673938
AI467606	-0.553021397	0.74632237
AI480526	0.029204275	0.98501783
Tfdp2	-2.186045696	0.002930807
AI504432	-0.016833748	0.988481007
AI506816	-0.073032087	0.978063275
AI593442	-0.032114012	0.971661054
AI597479	-0.069034497	0.90358675
AI606181	-0.063143348	0.968005385
AI661453	0.33929931	0.974823941
AI662270	-0.779730996	0.911276003
AI837181	0.068608968	0.91507125
Kcnj13	-2.182903094	0.442068044
AI839979	0.130812543	0.985742667
AI847159	0.450467621	0.97487264
AI849053	0.187919727	0.886647768
AI854703	0.113039477	0.910328122
AI987944	-0.210954527	0.890553353
Aida	-0.027915749	0.974809035
Aif1	-0.023491926	0.993277997
Aif1l	-0.044810281	0.980174892
Aifm1	0.122761525	0.783611405
Aifm2	-0.139428016	0.928672524
Aifm3	0.116502175	0.832619865
Aig1	0.140372153	0.767515266
Aim2	-0.032964483	0.993380259
Aimp1	0.11204277	0.851875536
Aimp2	0.124247528	0.910328122
Atp2a1	2.177931219	0.087805343
D5Ertd605e	2.584369997	0.675634247
Aire	0.118880453	0.98340069
Airn	-0.12577219	0.97932602
Ajap1	-0.03028702	0.976978183
Ajm1	0.073101534	0.87564489
Ajuba	-0.488646113	0.69189201
Ak1	0.096500065	0.852010446

AK157302	0.216437006	0.544661714
Ak2	0.017005948	0.985742667
Ak3	0.073838075	0.890704638
Ak3l2-ps	0.071504764	0.981045362
Gsk3b	2.175260191	1.08093E-43
Ak5	0.096362929	0.655744783
Ak6	0.106008093	0.648278106
Ak7	-0.040858653	0.994002549
Ak8	0.148688043	0.965241989
Ak9	-0.711812361	0.80931022
Akain1	0.115735313	0.815987743
Akap1	-0.095329519	0.889908105
Asgr1	-2.130572718	0.370298654
Akap11	-0.081773053	0.866560751
Akap12	-0.038000739	0.985742667
Klhl41	2.128903408	0.146214787
Akap14	0.431725156	0.962648047
Akap17b	-0.167722364	0.703372057
Akap2	0.011829617	0.997375151
Rpl34-ps2	2.55650872	0.780228245
Akap5	0.199917604	0.975831674
Tceal7	2.120987621	0.002268418
Akap7	-0.000210535	0.999653079
Akap8	0.043887157	0.955908516
Akap8l	0.073384129	0.861366392
Ces1d	2.104823071	0.113604388
Akip1	0.467653621	0.585057974
Akirin1	-0.04129032	0.952748686
Akirin2	0.017511569	0.985742667
Akna	-0.051301487	0.981502501
Aknad1	0.451635824	0.98553345
Akr1a1	-0.017389929	0.980339705
Akr1b10	-0.015220425	0.988208387
Akr1b3	0.085556397	0.908509375
Akr1b8	-0.586926343	0.848460959
Akr1c13	-0.54625675	0.96607835
Akr1c14	-0.130093599	0.971138085
Akr1c18	-0.090632363	0.978063275
Akr1c19	-0.531907037	0.98526072
Akr1e1	-0.056958116	0.953505913
Akr7a5	-0.079342788	0.932673884
Akt1	-0.00841859	0.991438757
Akt1s1	0.006029131	0.99527876
Akt2	0.094932792	0.964941885

Akt2-ps	-0.124389943	0.973100313
Akt3	-0.08979876	0.749519672
Aktip	0.070932692	0.921311891
AL589692.1	0.274273842	0.981144704
AL607142.1	0.08251524	NA
AL731706.1	0.08251524	NA
AL731706.2	0.017684538	0.991438757
Tdh	2.547503278	0.861378744
Nrap	2.096158299	0.013959166
Alad	-0.098836111	0.936031628
Alas1	-0.013342837	0.988034453
Alas2	-0.640806349	0.74032489
Mybpc1	2.073526185	0.045842618
Aldh16a1	-0.085974739	0.968005385
Aldh18a1	0.056945438	0.945647427
Aldh1a1	0.02316937	0.983479691
Cpb1	2.542908037	0.852010446
Aldh1a3	0.029324143	0.99527876
Aldh1a7	-0.852383636	0.942812446
Aldh1b1	-0.072767713	0.956922504
Aldh1l1	0.029854392	0.969755848
Aldh1l2	0.3786259	0.708838948
Aldh2	0.062935349	0.90358675
Aldh3a1	0.349219101	0.966213117
Aldh3a2	-0.052114408	0.91462326
Aldh3b1	-0.149088045	0.92621509
Aldh3b2	0.251425806	0.950195611
Aldh4a1	-0.088449339	0.955993235
Aldh5a1	-0.00109329	0.99765039
Aldh6a1	-0.039356438	0.961373288
Aldh7a1	-0.038359505	0.962648047
Aldh8a1	0.503046576	0.973100313
Aldh9a1	-0.036607356	0.974197921
Aldoa	0.034768482	0.963005032
Aldoart1	-0.875516159	NA
Aldoart2	0.930804399	0.734031302
Aldob	0.035411574	0.991559279
Aldoc	0.111009263	0.532934562
Alg1	-0.03502858	0.973069492
Acta1	2.07168434	0.095681074
Alg11	-0.061096374	0.911631946
Alg12	0.096518914	0.956164302
Fsd1l	-2.044674776	0.31865776
Alg14	-0.034232602	0.98526072

Alg2	-0.001954469	0.996638606
Alg3	0.054548639	0.974962198
Alg5	0.126467415	0.79212401
Alg6	-0.012078894	0.99527876
Alg8	-0.111309874	0.951497773
Alg9	0.00265453	0.997375151
Alk	-0.058917819	0.975831674
Alkal2	-0.10738918	0.97487264
Alkbh1	0.020943239	0.989556168
Alkbh2	0.08239444	0.97487264
Alkbh3	0.111091239	0.893784925
Alkbh3os1	-0.277500514	0.959276014
Alkbh4	0.058683007	0.9908633
Alkbh5	-0.053895857	0.934135315
Alkbh6	0.088542328	0.874252042
Alkbh7	0.196907964	0.847678217
Myh1	2.011122674	0.031985521
Allc	-0.599713971	0.962648047
Alms1	-0.001660705	0.998121731
Alms1-ps1	0.811961413	0.889908105
Myl3	2.461661467	0.684925922
Avpr1a	1.995911721	0.492371645
Alox12b	0.229825818	0.70036487
Alox5	-0.754818516	0.811777777
Pdlim3	1.956565855	0.001264615
Alox8	-0.012414956	0.994960308
Aloxe3	0.090997089	0.962648047
Alpk1	-0.258046282	0.677335597
Prelid2	2.457116244	0.873753967
Alpk3	-0.166496057	0.974197921
Alpl	-0.195639969	0.852010446
Als2	0.074507666	0.836506511
Als2cl	0.07537955	0.965520236
Hspb7	2.45129548	0.699575734
Alx1	-0.952275183	0.974809035
Alx3	-0.693432878	0.962648047
Rs1	2.448230562	0.6883862
Alyref	0.075197435	0.945647427
Alyref2	-0.008152148	0.997115148
Amacr	-0.047970214	0.974823941
Ambra1	-0.139157695	0.573810203
Amd-ps1	0.638121659	0.962648047
Amd-ps3	-0.067960543	0.921473132
Amd-ps4	0.051828606	0.983694404

Hfe2	1.948724382	0.374915032
Amd1	-0.014407625	0.985673938
Amd2	0.01446776	0.985742667
Amdhd2	-0.012984768	0.992385525
Amer1	-0.145138654	0.871869598
Amer2	-0.301391686	0.889972515
Amer3	0.161014653	0.591119873
Amfr	-0.081794845	0.833916587
Amh	0.008497292	0.99765039
Amhr2	-0.664337662	0.882096017
Hrc	1.941125089	0.021359544
Amigo2	-0.00810761	0.99527876
Amigo3	-0.187283877	0.875056988
Ammecr1	-0.260174814	0.895520088
Ammecr1l	-0.107407905	0.828907376
Amn	0.346212491	0.920642026
Amn1	0.040775715	0.976978183
Amot	-0.01877327	0.985673938
Amotl1	-0.055026468	0.968497942
Amotl2	0.096121428	0.936489142
Actn3	1.933093825	0.04370223
Ampd2	0.001051375	0.998010523
Ampd3	0.032196073	0.981144704
Amph	0.014031805	0.984328758
Amt	-0.081710773	0.928489442
Amy1	0.076874759	0.958389182
Amz1	0.09716859	0.962648047
Amz2	0.135601945	0.5542082
Anapc1	-0.108719818	0.6883862
Anapc10	0.211590659	0.573810203
Anapc11	0.177391676	0.603499717
Lyve1	-1.86024739	0.065333411
Anapc15	0.08050514	0.92517502
Anapc16	0.096313362	0.889972515
Anapc2	-0.00553431	0.99527876
Anapc4	0.062088778	0.928672524
Anapc5	-0.018000382	0.980339705
Anapc7	-0.002448715	0.997115148
Ang	0.41470899	0.740833211
Angel1	-0.158420677	0.842256259
Angel2	-0.005059454	0.99527876
Angpt1	-0.089741248	0.965520236
Olfr1393	2.40805784	0.5141747
Angptl1	0.367598661	0.871869598

Angptl2	-0.481233818	0.880133341
Angptl3	-0.485088185	0.972218452
Angptl4	0.681261508	0.539833449
Angptl6	0.019142189	0.992385525
Angptl7	-0.538606622	0.915297582
Ank	0.064143643	0.837755777
Plin4	1.850278026	2.25489E-09
Ank2	-0.133262868	0.590932961
Ank3	-0.454612801	0.787744512
Ankdd1a	0.164563816	0.981045362
Myhas	2.402606761	0.837755777
Ankef1	0.266058837	0.968005385
Ankfn1	0.121449732	0.985673938
Ankfy1	-0.180098627	0.581765233
Art1	1.848649457	0.439287299
Ankib1	-0.102243075	0.744309911
Ankle1	0.65787715	0.962648047
Ankle2	-0.267345812	0.749898795
Ankmy1	-0.154122962	0.985742667
Ankmy2	-0.029579868	0.965520236
Ankra2	0.154819994	0.548208119
Ankrd1	0.158398672	0.99527876
Ankrd10	0.012262406	0.985742667
Ankrd11	-0.092703459	0.729405774
Ankrd12	-0.176688818	0.688142448
Ankrd13a	0.132610844	0.751630609
Ankrd13b	-0.045970188	0.974197921
Casq1	1.842376993	0.036427563
Ankrd13d	-0.005229118	0.99527876
Ccdc125	-1.820195041	0.021359544
Jph2	1.820150918	0.260775968
Ankrd2	0.672324291	0.894953269
Vwa7	1.796189169	0.496022017
Ankrd24	0.002712189	0.997529192
Slc6a20b	-1.78318088	0.357627019
Ankrd27	0.036575898	0.96188696
Ankrd28	-0.08374788	0.894953269
Ankrd29	-0.107833092	0.908509375
Ankrd31	-0.77195469	0.759486652
Ankrd33b	-0.106023569	0.975831674
Dnase2a	-1.779505902	8.68748E-07
Ankrd34b	0.539825389	0.56399886
Ankrd34c	-0.009032463	0.997529192
Ankrd35	0.158420629	0.757721895

Ankrd37	-0.263105554	0.852010446
Ankrd39	-0.049754954	0.975800973
Ankrd40	-0.075869842	0.895520088
Ankrd42	-0.258627651	0.737087313
Ankrd44	-0.082211177	0.969755848
Ankrd45	0.015022105	0.985742667
Ankrd46	0.061810543	0.895520088
Ankrd49	-0.109848456	0.962648047
Ankrd50	-0.124683753	0.819778986
Ankrd52	-0.106313353	0.857344087
Ankrd53	-0.062813283	0.989556168
Mylk4	1.772001275	0.022383499
Ankrd55	0.110296283	0.974823941
Ankrd6	0.100976244	0.872352604
Ankrd61	0.171501457	0.972218452
Capn11	1.754808409	0.332957946
Ankrd66	0.207558239	0.968005385
n-R5s189	2.40065063	0.838354048
Ankrd9	0.329135545	0.792317291
Anks1	0.19527449	0.868415875
Anks1b	-0.069739636	0.896263151
Anks3	-0.102036945	0.833916587
Anks4b	-0.517518318	0.910328122
Anks6	0.117404195	0.927471971
Ankub1	0.092873274	0.980339705
Ankzf1	-0.131366606	0.724910334
Anln	0.023307359	0.985742667
Ano1	0.032133437	0.993872582
Asb10	1.740736909	0.356059662
Ano2	0.249173177	0.858549868
Ano3	-0.056994377	0.942812446
Ano4	-0.10226583	0.89703631
Ano5	-0.168992861	0.962648047
Ano6	-0.152939234	0.959276014
Ano7	0.073391865	0.994960308
Ano8	-0.017438252	0.984748462
Mir8098	2.379832958	0.74463793
Anp32-ps	0.178340047	0.985673938
Anp32a	0.142128738	0.519876661
Anp32b	0.086794646	0.90358675
Anp32b-ps1	-0.22603312	0.988034453
Anp32e	-0.027142626	0.970673196
Anpep	-0.433814374	0.878577494
Antxr1	-0.031593121	0.98340069

Antxr2	0.244164905	0.950460219
Anxa1	-0.787703936	0.655744783
Anxa11	0.071472567	0.89703631
Anxa11os	0.880681402	0.935188133
AC140457.1	2.377811907	0.869316117
Anxa2	-0.343196381	0.74428322
Anxa3	-0.09430934	0.957499962
Anxa4	0.110411768	0.962648047
Anxa5	0.135360025	0.780007405
Anxa6	0.119030254	0.549579107
Rbm33	-1.736360704	1.01475E-51
Anxa8	0.233644854	0.968630572
Anxa9	-0.26683082	0.938615214
Aoah	0.027909637	0.998010523
Aoc1	-0.969179238	0.960176242
Des	1.732397225	0.054884781
Adamdec1	2.348257754	0.873797321
Aox1	0.002110581	0.998010523
Grb7	1.725238478	0.298952496
Kel	2.339447223	0.774896252
Ap1ar	-0.416688652	0.951497773
Ap1b1	-0.010691952	0.987342614
Ap1g1	-0.121105713	0.56651873
Ap1g2	0.177424167	0.868415875
Ap1m1	0.146671831	0.569576306
Ap1m2	0.693581905	0.962648047
Ap1s1	0.110819297	0.674015802
Ap1s2	0.07025347	0.911631946
Ap1s3	0.248607451	0.929997774
Ap2a1	-0.052400253	0.90358675
Ap2a2	0.016921977	0.978063275
Ap2b1	-0.069661748	0.895986783
Ap2m1	0.048770326	0.899930239
Ap2s1	0.164383973	0.78576919
Ap3b1	-0.030074896	0.975831674
Ap3b2	-0.046130894	0.914222839
Ap3d1	0.021294732	0.971661054
Ap3m1	-0.060588622	0.890704638
Ap3m1-ps	-0.227874673	0.91462326
Ap3m2	-0.022862536	0.974197921
Ap3s1	0.103012436	0.950195611
Ap3s1-ps1	0.016934772	0.99527876
Ap3s1-ps2	-0.143100696	0.97487264
Ap3s2	0.089883407	0.821998633

Ap4b1	0.040132757	0.985446046
Ap4e1	-0.111869479	0.89703631
Ap4m1	-0.085344074	0.882096017
Myom2	1.655637182	0.064044233
Acbd7	1.647084449	0.322813856
Ap5m1	0.232400744	0.666064922
Ap5s1	0.04344272	0.991996237
Ap5z1	0.016797771	0.989556168
Apaf1	-0.206668167	0.971661054
Apba1	-0.025065582	0.975831674
Apba2	-0.005471679	0.99527876
Apba3	0.053982524	0.980339705
Apbb1	-0.007664722	0.993489613
Apbb1ip	-0.010633987	0.99527876
Apbb2	-0.028365672	0.985742667
Apc	0.641710371	0.855057773
Apc-ps1	0.771844483	0.952034929
Apc2	-0.140955116	0.744309911
Apcdd1	0.050022366	0.962648047
Apeh	0.037253652	0.975831674
Apela	0.186705497	0.980226283
Apex1	0.227301867	0.539833449
Apex2	0.209560619	0.91462326
Aph1a	-0.098392748	0.860521845
Aph1b	0.001866498	0.99765039
Aph1c	-0.206921186	0.607818467
Api5	-0.05651094	0.897878088
Apip	-0.087193032	0.951355313
Aplf	-0.245478344	0.799853839
Apln	-0.115992606	0.887625873
Ftl1-ps2	2.318701342	0.791353905
Aplp1	-0.043043972	0.914969279
Aplp2	-0.057856029	0.882096017
Apmap	0.083092525	0.87847909
Apoa1	-0.137018809	0.987814724
Apoa2	0.626227138	0.86783605
Apobec1	0.09433778	0.981641687
AC162938.1	-1.642136526	0.41993553
Apobec3	-0.101529258	0.97487264
Apobr	0.469297216	0.740240396
Apoc1	0.383025199	0.911631946
Apoc3	-0.246663019	0.989840188
Apod	-0.020293608	0.99527876
Nme9	-1.634428018	0.121443772

Apoh	0.310879154	0.962648047
Gdf7	2.303652491	0.779231312
Apol6	-0.668475656	0.89703631
Apol8	0.226637702	0.710375942
Apol9b	-0.454338581	0.985742667
Apold1	-0.008249816	0.997115148
Snord14d	2.300221681	0.770793864
Apoo	0.075738488	0.922999286
Apoo-ps	0.137243499	0.86783605
Apool	0.399711806	0.654893207
Apopt1	0.082622443	0.910328122
App	-0.012051832	0.98459388
Styx	-1.629553066	3.66471E-15
Appbp2os	-0.49254413	0.966010627
Appl1	-0.103340606	0.753987708
Appl2	-0.05366311	0.962648047
Aprt	0.055520175	0.974823941
Aprt-ps	0.258150623	0.970461999
Aptx	0.02580014	0.984748462
Aqp1	0.830392396	0.794475043
Pate3	2.29539137	0.911631946
Aqp11	-0.014468976	0.991996237
Aqp4	-0.050345641	0.955401619
Msmp	2.277486443	NA
Aqp6	0.190903374	0.99527876
Aqp9	-0.494065899	0.852275035
Aqr	-0.105345648	0.879401677
Itgb1bp2	1.624321848	0.442302609
Araf	0.074908143	0.868797028
Arap1	-0.171949437	0.80819125
Arap2	-0.092293677	0.896196194
Arap3	-0.149342052	0.84870301
Apobec2	1.607791092	0.397218031
Arcn1	-0.074740173	0.813744313
Arel1	-0.073492344	0.858549868
Arf1	0.019837442	0.97487264
Arf2	-0.077342993	0.962648047
Arf3	0.002334314	0.997115148
Arf4	0.092791602	0.758595939
Arf4os	-0.0619591	0.997529192
Arf5	-0.015695358	0.985673938
Arf6	0.032877357	0.981502501
Arfgap1	-0.009910648	0.989840188
Arfgap2	0.034141497	0.966608583

Arfgap3	0.048843318	0.968005385
Arfgef1	-0.08942451	0.766345155
Arfgef2	-0.077218597	0.810908327
Pnlip	-1.582909237	0.276254053
Arfip1	0.021674052	0.984328758
Arfip2	0.197328289	0.683281689
Arfrp1	0.106005419	0.870983403
Arg1	-0.839463173	0.973100313
Arg2	-0.113979947	0.965520236
Arglu1	0.023585822	0.971661054
Arhgap1	0.066827828	0.91082801
Arhgap10	0.128322855	0.90358675
Arhgap11a	0.193999999	0.925217889
Arhgap12	-0.156585321	0.852010446
Arhgap15	0.000133789	0.999653079
Prg4	-1.575752088	0.355311622
Arhgap18	-0.035945724	0.988034453
Arhgap19	0.340596002	0.924707867
Ada	1.571444736	0.001415569
Arhgap20os	0.995523368	0.956164302
Tcf24	1.559166055	0.135479179
Arhgap22	0.146297637	0.904480183
Arhgap23	-0.071399343	0.898180504
Arhgap24	0.350665749	0.667811993
Arhgap25	-0.457381858	0.771684575
Arhgap26	0.269611205	0.74428322
Arhgap27	0.144965901	0.873753967
Arhgap27os2	0.432069397	0.937550602
Tctex1d1	2.2700878	0.71803276
Arhgap28	-0.869280301	0.789386933
Arhgap29	-0.331295105	0.631374075
Arhgap30	0.079424486	0.974809035
Arhgap31	-0.001354204	0.998010523
Arhgap32	-0.048738507	0.951390592
Ghrl	-1.555139034	0.425165792
Crispld2	1.512122687	0.082752362
CT033749.1	2.264783804	0.707520358
Arhgap39	0.014157415	0.985742667
Arhgap4	-0.094324714	0.971661054
Arhgap40	-0.415471477	0.98459388
Arhgap42	0.050646001	0.974809035
Arhgap44	-0.06944442	0.962648047
Arhgap45	-0.199629004	0.801926713
Arhgap5	-0.333566852	0.534501962

Arhgap6	-0.384170815	0.710772024
Arhgap8	-0.920610967	0.972218452
Arhgap9	0.153099776	0.972218452
Arhgdia	-0.056065592	0.911631946
Arhgdib	-0.03580482	0.985742667
Arhgdig	0.095776575	0.911631946
Arhgef1	0.000128817	0.999582233
Arhgef10	-0.047314393	0.964941885
Arhgef10l	-0.046363189	0.962648047
Oas2	-1.505077644	0.426899054
Arhgef12	-0.103375023	0.722060735
Arhgef15	-0.07461981	0.968005385
Wfikkn2	-1.50445096	0.434676934
Sh2d5	-1.480767683	3.77356E-26
Arhgef18	-0.089774478	0.852010446
Arhgef19	-0.237540208	0.550601446
Mrc1	-1.479043935	0.449949801
Arhgef25	-0.054009614	0.932673884
Arhgef26	0.025566887	0.97607383
Zfp961	-1.475412941	0.021359544
Arhgef3	-0.006596721	0.994960308
Arhgef33	0.466994352	0.74463793
Arhgef37	0.220360354	0.948104382
Arhgef39	-0.322796286	0.957499962
Ttn	1.471231072	0.013959166
Arhgef40	0.013132389	0.993103295
Arhgef5	0.260003427	0.889908105
Arhgef6	-0.128675289	0.806422207
Arhgef7	-0.064872896	0.821998633
Arhgef9	-0.149886606	0.980419928
Arid1a	-0.073054175	0.848460959
Arid1b	-0.029717797	0.974962198
Arid2	-0.010211007	0.99161214
Arid3a	-0.177499784	0.871869598
Rpl32l	1.464535247	0.349775121
AC127347.1	2.239671678	0.749519672
Arid4a	-0.110822276	0.815034071
Arid4b	-0.239101365	0.76034043
Arid5a	-0.07504782	0.970461999
Arid5b	-0.023826407	0.984328758
Arih1	-0.090802811	0.771913242
Arih2	-0.012893542	0.990915306
Arl1	0.012661554	0.985742667
Arl10	0.103403533	0.935188133

Arl11	0.217633777	0.965290366
Hoxd8	2.23806474	0.873797321
Arl13b	-0.151371864	0.89383516
Arl14ep	-0.024671244	0.980896205
Arl15	-0.228908541	0.644513525
Arl16	0.155561992	0.754134958
AC112949.1	1.460800579	0.442302609
Arl2bp	0.068160334	0.87847909
Arl3	0.06503087	0.889908105
Arl4a	0.110737353	0.779231312
Arl4c	-0.062044558	0.939592821
Arl4d	0.224974809	0.886647768
Arl5a	-0.065529802	0.857344087
Arl5b	0.273918465	0.985446046
Arl5c	-0.176907146	0.962094124
Arl6	0.0400849	0.968005385
Arl6ip1	0.05092213	0.895520088
Arl6ip4	-0.133945506	0.893784925
Gxylt1	-1.424009602	0.408134963
Arl6ip6	0.13682131	0.890704638
Arl8a	0.101090535	0.84277074
Arl8b	-0.031845335	0.959276014
Arl9	0.567925766	0.984232465
Armc1	-0.134289853	0.647586234
Armc10	-0.129315964	0.984232465
Armc2	0.008773666	0.99527876
Fsip1	-1.418104571	0.452669584
Armc4	0.088473372	0.985742667
Armc5	0.079092547	0.963619706
Armc6	-0.259142348	0.617743758
Armc7	0.034067621	0.983797678
Armc8	0.04596281	0.916426816
Armc9	-0.011556676	0.991996237
Armcx1	-0.011617448	0.991438757
Armcx2	0.095665376	0.872352604
Armcx3	-0.068726194	0.905767935
Armcx4	-0.032469601	0.962648047
Armcx5	0.05558339	0.957773729
Armcx6	0.107228676	0.932673884
Armh1	0.940105424	0.909547396
Armh2	0.025788074	0.998010523
Armh3	-0.022164691	0.984328758
Impg2	-1.414939611	0.159596426
Rnf17	-1.408503385	0.341690144

Arnt	-0.113705739	0.80931022
Arnt2	-0.079449808	0.854278008
Cdnf	1.397398361	0.501489563
Arntl2	-0.03384087	0.981641687
Lcor	-1.36647504	0.022882132
C1rl	-1.363223915	0.346098168
Arpc2	0.015623848	0.987260085
Eno3	1.356785714	0.000461347
Arpc4	0.03196033	0.979711552
Arpc5	-0.299215632	0.921117568
Arpc5l	0.135839171	0.537584366
Arpin	-0.177163908	0.736130784
Scp2-ps2	1.336649328	5.65716E-12
Arpp21	0.054077878	0.958389182
Arr3	0.190320603	0.965520236
Arrb1	0.636285219	0.91462326
Arrb2	0.124950886	0.806077586
Arrdc1	0.067416175	0.975831674
Slc2a4	1.331835128	0.260708948
Arrdc3	-0.309633626	0.574956844
Arrdc4	-0.059488323	0.971138085
Arsa	-0.080140182	0.923340746
Arsb	0.007393392	0.992385525
Arsg	-0.052524397	0.971661054
Arsi	0.06839082	0.985742667
Arsj	0.27445426	0.779964055
Arsk	-0.18035007	0.795137348
CT010502.1	2.218562796	0.911834483
Art3	0.124628288	0.961373288
Mir3070a	2.21066055	0.770793864
Artn	0.417858529	0.956737477
Arv1	-0.010736161	0.99527876
Arvcf	0.158873211	0.823690933
Arx	0.030277666	0.985446046
Arxes1	0.163139364	0.554782791
Arxes2	0.035256659	0.973100313
As3mt	0.003084606	0.99765039
Asah1	-0.044525327	0.965290366
Asah2	-0.131964024	0.879401677
Asap1	0.053500997	0.962648047
Asap2	-0.215144082	0.52053053
Mlf1	1.310548575	0.259522215
Asb1	0.052374926	0.948104382
Hsd3b2	2.182550531	0.89046185

Asb11	0.267639687	0.889908105
Asb12	-0.191001217	0.99527876
Asb13	0.013850479	0.985742667
Slc15a5	2.180652493	0.813552442
Nup62cl	1.305155868	0.436797552
Asb16	0.210134349	0.870500867
Cacna1s	1.301627381	0.322635312
Asb18	-0.144958108	0.91462326
Asb2	0.608631817	0.650129082
Asb3	0.115249944	0.895198797
Asb4	0.145666824	0.993114821
Zfp740	1.286306424	0.278515147
Asb6	-0.005574729	0.99527876
Asb7	-0.105046556	0.860521845
Asb8	0.032743331	0.968630572
Ascc1	0.088357003	0.89703631
Ascc2	-0.069769742	0.911631946
Ascc3	-0.044753225	0.970892908
Ascl1	0.09945971	0.968630572
Ascl2	-0.203034565	0.98459388
Ascl4	0.224064237	0.965290366
Ascl5	0.431101743	0.980731359
Asf1a	-0.126726351	0.957499962
Asf1b	-0.208997141	0.968005385
Rufy4	2.173519151	0.900330211
Ash1l	-0.141529106	0.52053053
Ash2l	0.015005519	0.993103295
Asic1	0.112453397	0.911631946
Asic2	0.023242296	0.981502501
Samd13	2.168766496	0.873797321
Asic4	0.213655753	0.87847909
Asl	0.092093503	0.920642026
Asna1	0.134444114	0.571020232
Asns	0.026525232	0.984328758
Asnsd1	0.08135906	0.948345831
Aspa	0.042599915	0.981502501
Aspdh	-0.200682619	0.929133542
Aspg	-0.340388161	0.873797321
Gpr160	1.280367649	0.44174097
Asphd1	-0.296901919	0.873753967
Asphd2	-0.012286171	0.985742667
Aspm	-0.673006006	0.907829139
Aspn	-0.083882515	0.993103295
Asprv1	-0.022017073	0.99527876

Aspscr1	-0.021460024	0.984836179
Asrgl1	-0.042664996	0.910197639
Ass1	-0.026208893	0.98459388
Aste1	0.025300904	0.993872582
Astn1	-0.088103141	0.688142448
Astn2	-0.056776063	0.965520236
Asxl1	-0.119625808	0.776289147
Asxl2	-0.239252422	0.903538125
Asxl3	-0.011260962	0.99527876
Atad1	-0.041936625	0.944074069
Lrrc75b	-1.276520175	0.505749671
Atad2b	-0.094354722	0.89703631
Atad3a	0.106915529	0.786324313
Atad3aos	0.181864539	0.971661054
Atad5	0.004498418	0.99765039
Igfbpl1	1.26274157	0.064044233
Atcay	-0.030785737	0.962648047
Atcayos	0.244693598	0.90358675
Ate1	-0.038603328	0.936905058
Atf1	0.000614146	0.999227272
Steap1	2.16510431	0.858106541
Atf2	-0.082610988	0.7851601
Atf3	-0.345379785	0.911631946
Atf4	0.149789453	0.725853134
Atf5	-0.098370425	0.865075531
Atf6	0.021596383	0.97607383
Atf6b	0.093597307	0.749268538
Atf7	-0.519485036	0.909547396
Atf7ip	-0.106938816	0.844084751
Nxf3	2.159475501	0.620124996
Atg10	0.040602261	0.985673938
Atg101	-0.049908431	0.948104382
Atg12	-0.032207098	0.962648047
Atg13	-0.037825833	0.959659722
Atg14	-0.369945666	0.745141078
Atg16l1	0.069624972	0.883333293
Atg16l2	-0.055972699	0.971661054
Atg2a	-0.096679224	0.8616433
Atg2b	-0.198392241	0.579325713
Atg3	0.112526044	0.752941962
Atg4a	0.029942031	0.988034453
Atg4a-ps	-0.013791338	0.997258205
Atg4b	-0.092353073	0.83265523
Atg4c	-0.038370091	0.962648047

Atg4d	0.020241904	0.987150769
Atg5	-0.085819793	0.907829139
Atg7	-0.171035794	0.896263151
Atg9a	-0.075363177	0.947550497
Atg9b	-0.006749832	0.996638606
Atic	-0.04373975	0.965520236
Atl1	-0.090224821	0.770793864
Atl2	0.095404197	0.953505913
Atl3	-0.081584885	0.872352604
Atm	-0.074841137	0.939592821
Atmin	-0.033244894	0.963943597
Atn1	-0.023042685	0.984232465
Atoh7	0.613365256	0.874252042
Atoh8	-0.03854244	0.991559279
Arhgap36	1.261138568	0.387432606
Atp10a	0.372939823	0.510569404
Atp10b	0.36672982	0.861366392
Atp10d	-0.145588636	0.947232837
Ctrl	-1.258957102	0.486807247
Atp11b	-0.171049288	0.771684575
Atp11c	-0.000146747	0.999677699
Atp13a1	-0.082552681	0.845349932
Atp13a2	-0.097793567	0.787744512
Egr2	-1.250474271	0.146214787
Atp13a4	0.000253341	0.999486844
Atp13a5	-0.09005198	0.968005385
Mief1	-1.242813406	9.52104E-23
Atp1a2	-0.041685064	0.956164302
Atp1a3	-0.04201515	0.950195611
Atp1b1	0.023343749	0.968005385
Atp1b2	-0.213842481	0.74428322
Atp1b3	-0.050043044	0.947745582
Atp23	-0.015921307	0.994960308
Otop3	2.157326917	0.590932961
Ncoa4	1.23657529	0.148987519
Atp2a3	0.099670343	0.962648047
Tmod4	1.220930446	0.420373478
Atp2b2	-0.213801426	0.962648047
Atp2b3	-0.101966824	0.774896252
Atp2b4	0.093901869	0.929133542
Uaca	-1.206137608	0.234819833
Atp2c2	0.065774737	0.982934928
Mslnl	2.155887728	0.895520088
Atp5a1	0.018837421	0.972218452

Atp5b	-0.011916316	0.985673938
Gbp6	-1.19630356	0.357105886
Atp5d	-0.076994499	0.833916587
Atp5e	0.105321726	0.820614009
Atp5f1	0.090914832	0.688142448
Atp5g1	0.10296599	0.861366392
Atp5g2	0.085906415	0.911631946
Lao1	1.192958922	0.464952787
Prr22	-1.19223492	0.426109731
Atp5j	-0.186940193	0.798109023
Aoc3	1.179423578	0.329158589
Zfp184	-1.178690401	0.119226239
Atp5l	0.193804465	0.5141747
Atp5l-ps1	0.462223258	0.68906353
Atp5l2-ps	0.813403342	0.668010503
Atp5mpl	0.177310077	0.517425857
Casp2	1.168764116	2.04741E-08
Atp5s	0.039509658	0.971661054
Atp6ap1	0.045915317	0.909243078
Atp6ap1l	0.755627969	0.806581145
Atp6ap2	-0.037249786	0.962648047
AC158774.1	-1.16642288	0.310553098
Atp6v0a2	-0.133678624	0.948104382
Atp6v0a4	0.23862769	0.971661054
Atp6v0b	-0.046875727	0.938414123
Atp6v0c	0.024511353	0.974823941
Atp6v0c-ps2	-0.041888148	0.980132658
Atp6v0d1	0.027104248	0.968497942
Pagr1a	-1.165508155	0.435080924
Atp6v0e	0.076313849	0.918893969
Atp6v0e2	0.083364881	0.665532517
Atp6v1a	-0.006345315	0.991438757
Atp6v1b1	-0.681500043	0.923602336
Atp6v1b2	-0.016782698	0.980419928
Atp6v1c1	0.023081681	0.971138085
Atp6v1c2	0.870657444	0.514712196
Atp6v1d	0.100531561	0.640856315
Atp6v1e1	-0.056113444	0.957485565
Atp6v1e2	-0.928219131	0.974823941
Atp6v1f	0.071156036	0.911631946
Atp6v1g1	0.112008972	0.707777756
Atp6v1g2	0.078848913	0.82814939
Atp6v1h	0.040598296	0.959276014
Brinp2	-1.140269839	0.389153312

Atp7b	-0.037847713	0.990936813
Tgif2	1.13831743	0.38218128
Atp8a2	-0.143356213	0.740628902
Rep15	1.132091297	0.408765725
Atp8b2	-0.254767816	0.770793864
Atp8b3	-0.328052034	0.980419928
Erbb4	2.12682175	0.528197276
Atp9a	-0.064893617	0.874971335
Atp9b	0.158477845	0.73481128
Atpaf1	0.297338045	0.736130784
Atpaf2	-0.023679089	0.985293518
Nrg4	1.130228253	0.145093191
Atr	-0.143372512	0.89383516
Vgll3	1.12856757	0.497690258
Atrip	-0.16891362	0.880133341
Rpl31-ps11	1.122441847	0.395640848
Atrnl1	-0.096566377	0.655006368
Atrx	-0.200750898	0.540997524
Atxn1	-0.050665158	0.962648047
Atxn10	0.06343458	0.837755777
Atxn1l	-0.278489314	0.960201673
Inpp4a	1.112615162	5.39283E-15
Cox6a2	1.108730825	0.024837373
Atxn3	-0.033619305	0.969476616
Atxn7	0.069523676	0.973069492
Atxn7l1	0.011721756	0.991996237
Atxn7l1os2	-0.330891071	0.985742667
Atxn7l2	-0.029103471	0.980419928
Acvr2b	-1.107291388	0.309025051
Atxn7l3b	-0.014274548	0.9813031
AU020206	-0.207427736	0.936031628
Slc28a3	2.126361363	0.889972515
AU021092	-0.130989567	0.959276014
AU022252	-0.088274988	0.825148733
AU022751	0.077489443	0.997115148
Zfp605	1.101960797	2.75386E-10
Sel1l	-1.095917123	1.01475E-51
AU023762	-0.33726035	0.859910936
AU040320	-0.055131336	0.962648047
AU040972	-0.095153994	0.994960308
AU041133	0.218636923	0.928672524
Auh	0.041465423	0.95126064
Aunip	-0.452405845	0.962648047
Aup1	0.065083607	0.91462326

Aurka	0.35982777	0.788592804
Aurkaip1	0.141369028	0.82552579
Aurkb	-0.506024901	0.971138085
Auts2	-0.041226479	0.959276014
AV039307	0.440884794	0.922889447
AV099323	-0.365015782	0.985446046
AV356131	0.328505985	0.90918338
Aven	0.040997157	0.974809035
Prl	2.108303399	0.875723186
Asb14	1.090243953	0.417721745
Avp	0.381388836	0.97487264
Avpi1	0.017941131	0.99100709
Col28a1	2.106611334	0.733820303
Avpr1b	-0.699856529	0.722596123
Avpr2	0.605034863	0.965520236
AW011738	0.580725253	0.610307639
AW046200	-0.732660036	0.815923615
AW047730	-0.091594527	0.956164302
AW112010	-0.577859099	0.813552442
AW121686	0.012279272	0.997115148
AW146154	-0.172319318	0.911631946
AW209491	0.089738995	0.912928483
AW495222	0.093531259	0.984232465
AW549877	-0.041752301	0.945526153
AW551984	0.216114811	0.901661918
AW554918	-0.248527163	0.968005385
AW822252	-0.031362827	0.994960308
Awat2	0.021652619	0.998010523
Axdnd1	0.832698845	0.853090538
Axin1	0.193711558	0.727452809
Axin2	0.074657252	0.97487264
Axl	0.023770939	0.980419928
AY036118	0.386882627	0.695239733
Mypn	1.082082441	0.410106728
Aym1	-0.927746925	0.965520236
AC148013.1	1.078339531	0.509250303
Azin1	0.291200883	0.641233173
Azin2	0.145087948	0.839488666
B2m	0.00032019	0.999391708
B3galnt1	0.162985555	0.537584366
B3galnt2	0.761808807	0.806422207
B3galt1	-0.141288571	0.901479608
B3galt2	0.032206348	0.97487264
B3galt4	-0.075116414	0.97487264

Trim26	-1.074264012	0.431061843
B3galt6	-0.122282046	0.911631946
Phldb2	-1.066241836	0.060781486
B3gat2	-0.072816739	0.915297582
Card14	1.065616173	0.267357838
B3glct	-0.023855365	0.985293518
B3gnt2	-0.515925024	0.939592821
B3gnt3	-0.421806361	0.901479608
B3gnt4	0.704981705	0.963666931
B3gnt5	0.083010126	0.98612132
Pifo	1.061803423	0.314323148
B3gnt7	0.072892276	0.989556168
B3gnt8	-0.21267541	0.852035098
B3gnt9	0.332191632	0.904480183
B3gntl1	-0.128234551	0.91462326
B4galnt1	-0.038988849	0.962648047
B4galnt2	-0.866465737	0.934798735
B4galnt3	0.193330268	0.956922504
B4galnt4	0.089769226	0.737860635
B4galt1	0.338605605	0.66401455
B4galt2	-0.134318862	0.74463793
B4galt3	-0.018425975	0.985742667
B4galt4	0.157901163	0.891972665
B4galt5	0.00802682	0.994806961
B4galt6	-0.076804224	0.779756705
B4galt7	0.055007701	0.959276014
B4gat1	0.006807659	0.994960308
B9d1	0.154758512	0.84870301
B9d1os	0.146745051	0.985742667
B9d2	-0.127663887	0.911631946
Baalc	0.067360123	0.854278008
Dynll2	1.057642036	0.120903042
Babam1	0.050158772	0.91462326
Babam2	0.04753573	0.953505913
Bace1	0.217553284	0.962648047
Bace2	0.315943002	0.894953269
Bach1	-0.040163619	0.980226283
Bach2	0.353094968	0.965520236
Bach2os	-0.304304769	0.975831674
Bad	0.175964453	0.810503622
Hif3a	1.033740745	2.02566E-06
Bag2	-0.068987521	0.965520236
Bag3	-0.014392227	0.994960308
Bag4	0.093655888	0.811219747

Bag5	0.038449937	0.965241989
Bag6	-0.000189202	0.999242708
Bahcc1	-0.305217416	0.663097563
Bahd1	0.045458669	0.964941885
Baiap2	-0.103584362	0.611300007
Baiap2l1	-0.589707537	0.803910915
Baiap2l2	0.421547249	0.912042382
Baiap3	0.02707183	0.993103295
Bak1	0.047332744	0.984748462
Bambi	0.215873034	0.825256691
Bambi-ps1	0.370318432	0.953505913
Banf1	0.073324626	0.898474424
Bank1	-0.289049156	0.890704638
Banp	0.04028775	0.99527876
Bap1	0.042107013	0.936031628
Bard1	0.04178165	0.99527876
Trim63	2.089392047	0.604176395
Barhl2	0.666861707	0.97487264
Barx2	-0.040880157	0.993489613
Basp1	0.073361807	0.911631946
Batf	-0.133862589	0.99527876
Batf2	-0.933566394	0.927659499
Batf3	0.010298458	0.99765039
Bax	0.0388665	0.973100313
Baz1a	-0.611120355	0.688142448
Baz1b	-0.068983992	0.882824434
Baz2a	-0.103301549	0.815987743
Baz2b	-0.107043484	0.873797321
Cd7	2.083068776	0.861397728
BB187690	0.345500633	0.921311891
BB218582	0.202187621	0.935897306
Ccl22	2.078016105	0.585734325
BB557941	-0.055976907	0.99527876
Bbc3	-0.163031521	0.936031628
Bbip1	-0.05488649	0.962648047
Bbof1	-0.08029328	0.962648047
Bbox1	0.437931653	0.660403254
Bbs1	-0.105132927	0.770793864
Bbs10	-0.009305545	0.99527876
Bbs12	-0.073678595	0.97469529
Bbs2	-0.035187686	0.971138085
Bbs4	-0.012673776	0.985742667
Bbs5	-0.043168813	0.968005385
Bbs7	-0.062472127	0.939060335

Bbs9	-0.073234023	0.950460219
Bbx	0.363267406	0.721943953
BC001981	0.282896664	0.993103295
BC002059	0.795556827	0.650504271
BC002163	0.388312651	0.67047476
BC003965	0.013884881	0.988034453
BC004004	0.053625768	0.928957615
BC005537	-0.005230546	0.99527876
BC005561	-0.431611439	0.547986726
BC005624	0.053442382	0.882824434
BC006965	0.319552571	0.899249451
BC016548	-0.515125736	0.984748462
BC016579	0.823182153	0.882096017
BC017158	0.104995387	0.94417444
BC018473	0.317422498	0.953505913
Foxo6os	2.075909024	0.875865995
BC022960	0.219682762	0.740833211
Pbrm1	-1.027242629	2.67154E-06
BC024063	-0.70046839	0.685671097
BC024139	0.170232909	0.968977192
BC024978	-0.038234262	0.980339705
BC025920	-0.032980167	0.985742667
BC028528	0.20607865	0.936031628
BC028777	0.875747903	0.962648047
BC029722	0.076791377	0.925056766
BC030343	0.288203371	0.573101161
BC030499	0.294054722	0.89703631
Nagpa	-1.02311993	0.003487984
BC030867	0.346990996	0.957499962
BC031181	-0.001216865	0.99765039
BC034090	-0.142918731	0.932019556
BC035044	-0.339048778	0.910328122
BC035947	-0.22901874	0.948104382
BC037032	0.04879297	0.989840188
Rpl36-ps12	1.014289033	0.455841215
BC037039	-0.144814229	0.98526072
BC043934	0.594212773	0.910328122
BC046251	0.543513739	0.852010446
BC046401	0.744310725	0.868415875
BC048403	0.207663933	0.975350978
BC048644	0.354161416	0.935131589
BC049352	-0.020196489	0.99765039
BC049715	0.399196918	0.882824434
BC049762	-0.967073174	0.939592821

Cd101	2.072633128	0.56399886
BC051019	-0.642986584	0.908509375
BC051142	-0.10036413	0.982408617
BC051226	-0.009275604	0.996223092
BC052040	-0.14481171	0.905251009
Prcd	1.013039968	0.219354159
BC055308	-0.59768386	0.944753667
BC055324	0.260702144	0.962648047
BC055402	0.204632516	0.97218449
Bpifc	2.064088278	0.816844103
BC064078	0.225787081	0.874422885
BC065397	-0.672978569	0.604176395
BC067074	-0.57472638	0.599763688
BC085271	-0.076885375	0.991438757
BC106175	0.196798199	0.98526072
BC106179	0.918115782	0.711794594
Ano9	2.041234704	0.904480183
Bcam	0.008526882	0.99527876
Bcan	-0.004798922	0.99527876
Bcap29	-0.023222577	0.985293518
Bcap31	-0.081543282	0.864907053
Bcar1	0.02928871	0.972374093
Bcar3	0.139997834	0.89703631
Bcas1	0.081172053	0.985742667
Snord1b	2.035486517	0.890704638
Bcas1os2	-0.433210873	0.962648047
Bcas2	0.138462071	0.819778986
Bcas3	0.049762611	0.958951979
Bcat1	-0.045015178	0.961373288
Bcat2	-0.126691729	0.847556796
Bccip	0.080609297	0.891972665
Bcdin3d	-0.187844499	0.868415875
Bche	-0.459001219	0.740833211
Bckdha	0.002327341	0.997258205
Bckdhb	-0.00443185	0.99527876
Bckdk	-0.218535147	0.563566515
Bcl10	-0.024653255	0.985742667
Bcl11a	-0.098288555	0.744924545
Bcl11b	-0.1235769	0.792517766
Bcl2	-0.109691053	0.89703631
Bcl2a1a	0.308656169	0.975350978
Bcl2a1b	-0.216997858	0.965520236
Bcl2a1d	-0.155686013	0.992654978
Bcl2l1	0.546519603	0.860521845

Bcl2l11	0.06697964	0.97607383
Bcl2l12	0.053281465	0.985742667
Bcl2l13	-0.05853716	0.924640051
Rxfp1	1.009364501	0.496022017
Bcl2l2	0.874304424	0.80818949
Tns4	2.032815493	0.665532517
Bcl6	0.097877048	0.83886846
Bcl6b	-0.088719675	0.976978183
Bcl7a	0.039159645	0.939592821
Bcl7b	0.160306891	0.710772024
Bcl7c	0.042096133	0.980339705
Hyal1	-1.005834692	0.146214787
Fkbp1a	1.003316888	0.224597392
Bclaf1	-0.053448516	0.953505913
Bclaf3	0.332961899	0.736486324
Bco1	0.5481776	0.970348278
Bco2	0.106866166	0.959276014
Ankrd23	0.99562871	0.152369291
Usp43	0.994711219	0.442302609
Bcr	-0.104120121	0.6883862
Bcs1l	-0.116351608	0.854278008
Bdh1	0.124450377	0.973100313
Bdh2	-0.150989014	0.955993235
Bdkrb1	-0.643828957	0.956922504
Bdkrb2	-0.00105799	0.999582233
Bdnf	-0.174250191	0.764783842
Traf1	-0.993885823	0.41351939
BE692007	0.044971559	0.996497797
Bean1	0.036141421	0.989840188
Becn1	-0.103367101	0.962094124
Begain	0.00835603	0.991996237
Bend3	-0.099678862	0.99527876
Bend4	-0.099338392	0.921154826
Bend5	0.215516966	0.876359276
Bend6	0.067508786	0.914969279
Bend7	0.332867023	0.915297582
Best1	-0.01771589	0.995411429
Best3	0.227437512	0.962648047
Bet1	-0.061576036	0.959276014
Bet1l	0.111267533	0.89703631
Cdkn1a	0.991324037	0.098798554
Tpm2	0.982611668	0.144051788
Crk	-0.976973036	0.113604388
Bex4	0.096933708	0.905251009

Bfar	0.110372917	0.920642026
Bfsp1	0.027440381	0.99527876
Bfsp2	0.199451623	0.876612181
Prss54	2.030952619	0.911631946
CAAA01189291.1	2.030658367	0.663393433
Bgn	-0.188198843	0.799441915
Bhlha9	-0.036626716	0.991996237
Bhlhb9	0.0299351	0.976513767
Bhlhe22	0.150668532	0.720841863
Rpl9-ps3	2.015938802	0.874891526
Bhlhe40	-0.138609769	0.521202205
Bhlhe41	-0.042401678	0.980339705
Bhmt2	0.59591603	0.939503568
Bicc1	-0.322146469	0.84870301
Ska3	-0.971611532	0.327922951
Bicd2	-0.060633964	0.882824434
Bicdl1	-0.033852498	0.979037366
Bicra	-0.15447053	0.911276003
Bicral	-0.082028987	0.881407916
Bid	0.208576377	0.630888733
Bin1	-0.016277909	0.976374627
Bin2	-0.10794561	0.951390592
Bin3	0.147745019	0.871869598
Birc2	-0.016031024	0.987152658
Birc3	-0.004727334	0.998010523
Birc5	0.260142044	0.953505913
Birc6	-0.089140208	0.814814675
Birc7	0.555824904	0.935897306
Bivm	-0.028058894	0.981502501
Blcap	0.104734978	0.685061817
Blm	-0.190726673	0.903279667
Blmh	0.110241332	0.8549794
Blnk	0.019086904	0.994960308
Mustn1	0.964123465	0.399286082
Asb5	0.962432881	0.442302609
Bloc1s2-ps	0.064902813	0.985673938
Bloc1s3	-0.0961129	0.929997774
Bloc1s4	0.064610386	0.968005385
Bloc1s5	0.067461769	0.951949091
Bloc1s6	-0.007351177	0.998010523
Blvra	0.157236603	0.779964055
Blvrb	-0.041924683	0.98340069
Blzf1	-0.042296522	0.974962198
Bmf	0.236868426	0.91462326

Bmi1	-0.079859404	0.875723186
Bmp1	0.021695122	0.985742667
B3gnt6	-0.961357852	0.41351939
Bmp2	-0.27331218	0.874971335
Bmp2k	-0.16343116	0.803910915
Bmp3	-0.005715223	0.997529192
Bmp4	-0.0887083	0.98459388
Bmp5	-0.638493743	0.910328122
Bmp6	-0.48878968	0.84870301
Bmp7	-0.641229288	0.740833211
Bmp8b	0.594386049	0.962648047
Bmper	0.045025655	0.985293518
Bmpr1a	-0.100454954	0.828907376
Bmpr1b	-0.11468584	0.89703631
Bmpr2	-0.151565198	0.695239733
Bms1	0.019704357	0.985477412
Bmt2	-0.098195275	0.895326715
Bmx	-0.145180053	0.968005385
Bmyc	0.138372564	0.749229362
Bnc2	-0.290855449	0.989840188
Bnip1	0.176387198	0.572363201
Bnip2	0.123130587	0.786324313
Bnip3	0.025093914	0.975831674
Mfsd13b	0.959458391	0.24802413
Bnip3l-ps	0.296446646	0.813667053
Mir26b	2.000995008	NA
Kcna5	0.956430496	0.066550664
Bod1	0.012534513	0.987453225
Bod1l	-0.056917855	0.950460219
Bok	0.056563453	0.9884736
Bola1	0.208981146	0.862574936
Bola2	0.189933106	0.781552764
Bola3	0.138063612	0.86467336
Neb	0.955731053	0.214136871
Bop1	-0.125869185	0.585734325
Bora	0.13895208	0.949607377
Borcs5	0.11733593	0.803910915
Borcs6	0.07443352	0.954377438
Borcs7	0.009643923	0.993103295
Evi2a	0.94611702	0.039818492
Bpgm	0.058132217	0.904750766
Bphl	-0.083856303	0.883921639
Bpifb1	0.079238448	0.99527876
Bpifb4	0.520631	0.980226283

Slc6a5	1.983194562	0.910328122
Bpnt1	-0.021099828	0.976031422
Meioc	0.945693108	0.468667381
Braf	-0.114394463	0.779231312
Brap	0.02387134	0.97469529
Brat1	-0.011966828	0.993489613
Brca1	0.197809509	0.959276014
Brca2	0.064086801	0.974197921
Raver1	0.937509102	2.58588E-12
Brd1	-0.010720692	0.991606359
Brd2	0.058510218	0.861366392
Brd3	-0.02641008	0.984328758
Brd3os	-0.007303666	0.99527876
Brd4	-0.085216404	0.905585111
Brd7	-0.061292018	0.911631946
Brd8	-0.027398696	0.97487264
Brd9	-0.051389727	0.925678705
Brdt	0.003172726	0.996497797
Brf1	0.01586166	0.985742667
Brf2	0.056147649	0.957849305
Bri3	-0.047282991	0.965520236
Bri3bp	-0.020688719	0.980226283
Bricd5	0.931527801	0.875723186
Brinp1	-0.014404688	0.985673938
Btnl9	1.982468296	0.910328122
Brinp3	0.176914401	0.834315076
Rnu3a	1.979525993	0.918407269
Brip1os	0.010186073	0.99527876
Brix1	-0.005253637	0.99527876
Alkbh8	0.934270085	0.403453477
Brms1	0.048587637	0.968005385
Brms1l	-0.059297263	0.899141332
Brox	0.122288306	0.985673938
Brpf1	-0.013451273	0.988034453
Brpf3	0.270449984	0.745988863
Brsk1	0.0034086	0.99527876
Brsk2	-0.18719609	0.604176395
Brwd1	-0.05779319	0.893225143
Brwd3	-0.20264034	0.717156625
Bscl2	0.028618652	0.969661076
Bsdc1	0.008182233	0.991996237
Bsg	0.066831875	0.908554604
Bsn	-0.123358118	0.605700484
Bspry	0.504593556	0.874971335

Bst1	-0.002451164	0.999242708
Bst2	-0.01594672	0.99527876
Btaf1	-0.133564047	0.710870539
Btbd1	-0.049958941	0.938615214
Btbd10	0.545257607	0.698018236
Btbd11	-0.440803079	0.544721388
Btbd16	0.260776581	0.89703631
Btbd17	-0.122847081	0.953505913
Mir7041	1.974122314	NA
Btbd19	0.005748447	0.99765039
Btbd2	-0.000406989	0.99916609
Btbd3	-0.052193748	0.965520236
Btbd6	-0.325781044	0.889547181
Btbd7	-0.12123341	0.854278008
Btbd8	-0.100547855	0.873797321
Btbd9	-0.017976576	0.976978183
Btc	-0.372156997	0.962812066
Btd	-0.04308879	0.975951183
Cdc25a	-0.933214348	0.003820019
Btf3l4	-0.006436891	0.99527876
Sned1	-0.929200989	0.013771454
Bcl2l15	0.915125854	0.420814913
Btg3	0.100761525	0.904346827
Btk	0.638283355	0.5141747
Btla	-0.633716436	0.844084751
Btn1a1	0.386412487	0.985673938
Btn2a2	0.144414644	0.970673196
Epx	1.969272655	0.842256259
Btnl7-ps	-0.106888053	0.995411429
Nlrp10	1.957491705	0.845764043
Btrc	0.002983579	0.995411429
Bub1	0.181821649	0.993380259
Bub1b	-0.076937777	0.970673196
Bub3	-0.018627265	0.985673938
Bud13	0.030136026	0.985673938
Bud23	-0.00519299	0.99527876
Bud31	0.055875136	0.932292695
Bves	-0.134953479	0.928672524
Bvht	0.266188635	0.939052665
Bysl	-0.073785472	0.89703631
Bzw1	-0.009688513	0.991996237
Bzw2	-0.143409996	0.580668545
C1d	0.086610941	0.864280235
C1galt1	-0.045662985	0.973100313

C1galt1c1	0.035419148	0.970673196
C1qa	-0.002193087	0.99765039
C1qb	0.161636481	0.571020232
C1qbp	0.079813533	0.868415875
C1qc	0.055290675	0.965520236
C1ql1	-0.095549194	0.959276014
C1ql2	0.272314802	0.757607194
C1ql3	0.157802551	0.796563869
C1qtnf1	0.044357666	0.988034453
C1qtnf12	0.07967934	0.927934062
C1qtnf2	-0.500332988	0.889972515
C1qtnf3	-0.410859057	0.955993235
C1qtnf4	-0.0227145	0.984328758
C1qtnf5	-0.192008419	0.887460954
C1qtnf6	-0.280005263	0.861366392
C1qtnf7	-0.980137274	0.745988863
C1qtnf9	0.596303968	0.771450635
C1ra	0.267392349	0.92517502
Dbh	0.909355411	0.421691269
Fmr1os	1.952916765	0.911631946
C1s1	0.140791529	0.97469529
C1s2	-0.100350722	0.988481007
C2	-0.704407391	0.708001635
C2cd2	0.014183798	0.991996237
C2cd2l	-0.022341683	0.97487264
C2cd3	-0.015361131	0.985742667
C2cd4a	-0.028524398	0.99527876
C2cd4b	0.658364049	0.690527118
C2cd4c	0.058817274	0.976978183
C2cd4d	-0.076796662	0.991996237
C2cd5	-0.012730187	0.991541583
C3	-0.688212327	0.85252367
Usp50	0.901808576	0.403453477
C4a	0.097227551	0.968005385
C4b	-0.12724781	0.861366392
C4bp	0.082373221	0.985446046
C5ar1	-0.063333723	0.99527876
Irx3	1.951523873	0.895198797
C7	0.366592251	0.975831674
C77080	0.242648507	0.741466852
C78197	0.525947145	0.882824434
C78859	0.087128959	0.87847909
C79130	0.209826299	0.971661054
C86187	0.362145804	0.985742667

C87436	0.062991177	0.957499962
C8b	0.449451394	0.981641687
C8g	-0.143770481	0.962648047
C9orf72	-0.0639558	0.949607377
CAAA01141682.1	-0.025155264	0.99527876
Slc52a3	-0.896106217	0.380767313
Caap1	-0.018289861	0.994960308
Cab39	-0.079612632	0.809685
Cab39l	-0.024519851	0.985742667
Cabcoco1	0.172583112	0.932956012
Cabin1	-0.054032102	0.949607377
Cables1	0.15743883	0.802538464
Cables2	-0.082230813	0.918475273
Cabp1	-0.12263237	0.86128643
Cabp4	0.238182345	0.97041548
Cabp7	-0.048757718	0.953505913
Cabyr	-0.085626913	0.974823941
Cacfd1	0.009493348	0.994824604
Cachd1	-0.102229632	0.827483016
Armc3	-0.888562877	0.434762828
Cacna1b	-0.075273937	0.854434649
Cacna1c	-0.120822114	0.729344189
Nostrin	0.882436112	0.06045301
Prima1	-0.880938293	0.275969466
Tspan8	1.943484184	0.62543868
Abi2	-0.880670428	0.41351939
Cacna1h	-0.037277484	0.962648047
Cacna1i	-0.105851571	0.868415875
AU022754	0.880568342	0.44636597
Zfp85os	-0.878968639	0.006110401
Cacna2d2	-0.150514261	0.975831674
Col3a1	-0.867953969	0.46711689
Cacna2d4	0.473697949	0.751863834
Cacnb1	-0.078766563	0.860142574
Hist1h2ac	-0.865621776	0.338081307
Cacnb3	-0.018421411	0.981392364
Cacnb4	-0.098937264	0.992654978
Mir1907	1.939951311	NA
Cacng2	-0.065448021	0.903795193
Cacng3	-0.007384551	0.99527876
Cacng4	0.007729341	0.99527876
Cacng5	0.037372126	0.979037366
Cacng6	-0.075953764	0.98459388
Cacng7	-0.045417404	0.946239011

Cacng8	-0.086077606	0.861378744
Cactin	-0.045833238	0.965520236
Cacul1	0.016538787	0.980731359
Cacybp	-0.072971551	0.909109599
Cad	-0.048898544	0.975831674
Cadm1	0.013026328	0.99165967
Cadm2	-0.013540109	0.985742667
Cadm3	-0.10319446	0.890704638
Cadm4	0.139997349	0.604176395
Dlg2	-0.863836073	0.09657124
Xdh	0.862526581	5.97381E-05
Cage1	-0.165834185	0.932197749
Calb1	0.12161107	0.527794911
Cox7a1	0.862426684	0.399005965
Calca	0.037493341	0.99527876
Calcb	-0.249334083	0.98526072
Calcoco1	0.06440203	0.866560751
Calcrl	-0.258642892	0.74463793
Cald1	0.035730554	0.981641687
Calhm2	-0.23722853	0.806422207
Calhm5	0.070733708	0.962648047
Rin3	0.857174933	0.106978188
Calm1	-0.085055206	0.771684575
Calm2	0.08013433	0.793949146
Calm3	0.073269497	0.8549794
Calml4	0.618210414	0.74463793
Caln1	-0.016643827	0.985742667
Calr	-0.044564403	0.953505913
Calr3	-0.11453768	0.974197921
Calr4	-0.250529444	0.962648047
Calu	-0.099488469	0.980419928
Caly	0.085069353	0.83886846
Camk1	-0.053546822	0.961887657
Maff	0.856276982	0.299535449
Camk1g	-0.110622107	0.881371608
Camk2a	-0.071301947	0.849309638
Camk2b	0.153188898	0.891265024
Camk2d	0.138021842	0.868415875
Camk2g	0.217732468	0.736130784
Slc38a11	0.851024917	0.452669584
Camk2n2	0.159987074	0.873762805
Camk4	-0.159836264	0.692234894
Camkk1	0.449827371	0.910196388
Camkk2	-0.044984895	0.936031628

Camkmt	0.056758839	0.974823941
Camkv	-0.032720032	0.962648047
Caml	0.093624573	0.837755777
Camp	0.54004933	0.984328758
Camsap1	-0.040316279	0.971661054
Camsap2	-0.114551822	0.631708623
Camsap3	0.001799847	0.99765039
Camta1	-0.431308133	0.736130784
Noct	-0.844187374	0.460614424
Cand1	-0.061716561	0.835493069
Cand2	-0.318914837	0.655744783
Cant1	0.253760118	0.924707867
Canx	-0.077186995	0.811219747
Cap1	-0.376934941	0.84870301
Cap2	-0.145145984	0.974823941
Capg	0.045966444	0.98459388
Capn1	-0.083818381	0.860521845
Capn10	-0.12339326	0.814814675
Klhl31	1.932619407	0.911631946
Capn12	0.982871478	0.898478018
Capn13	-0.638878235	0.981392364
Capn15	-0.118925675	0.756543929
Capn2	0.082646719	0.865075531
Capn3	0.202177425	0.779346076
Capn5	0.094021439	0.815987743
Capn6	-0.082239551	0.985673938
Capn7	-0.028694345	0.971138085
Capn9	-0.134109265	0.991996237
Capns1	-0.01878526	0.97487264
Mpz	1.920723153	0.909547396
Caprin1	-0.090221756	0.665532517
Caprin2	-0.256858764	0.770793864
Caps2	0.252154743	0.97487264
Capsl	0.212301125	0.874971335
Capza1	-0.150826614	0.703479789
Capza2	0.002298811	0.996094752
Capzb	0.067087637	0.884426973
Car10	0.114060637	0.895198797
Car11	0.139287199	0.740240396
Car12	0.144086661	0.76998058
Car13	-0.564687062	0.911631946
Car14	0.083994422	0.964941885
Car15	-0.378638783	0.742969797
Car2	-0.101102122	0.770166577

Rpl17-ps4	1.920261929	0.707777756
Sema3a	-0.843989133	0.311609026
Car5a	0.813574057	0.936031628
Car5b	-0.040106578	0.99100709
Gemin7	-0.839484565	0.224467533
Car8	0.12279057	0.966213117
Car9	0.456444482	0.89703631
Card10	0.088412861	0.959659722
Lilra5	1.916980248	0.89703631
AC138768.1	1.91229486	0.920642026
Card19	-0.014920618	0.99527876
Card6	0.307714411	0.69189201
Card9	0.043760227	0.987207097
Carf	0.102932938	0.96607835
Carhsp1	0.187243776	0.57472851
Carlr	-0.019802495	0.99527876
Carm1	-0.430415853	0.884426973
Carmil1	0.245151386	0.511540781
Carmil2	-0.041629182	0.965520236
Carmil3	-0.169883522	0.698427195
Klrd1	1.909234687	0.87847909
Carnmt1	-0.036573352	0.98526072
Carns1	0.05761592	0.970461999
Cars	-1.12663E-05	0.99991697
Cars2	-0.142513431	0.759486652
Cartpt	-0.077997209	0.987152658
Casc1	0.35057306	0.910328122
Casc3	0.011409363	0.99100709
Zfp109	-0.837525274	0.442302609
Casd1	-0.070206185	0.90872336
Cask	0.137134061	0.56488584
Caskin1	-0.103151515	0.773876893
Rab26os	0.836405394	0.064044233
Casp1	-0.148171737	0.966538721
Anxa13	1.906976284	0.778424472
Casp16-ps	-0.684465618	0.974809035
Gssos1	1.896389384	0.655744783
Casp3	-0.059852832	0.973100313
Casp4	-0.363089134	0.971661054
Casp6	0.229030902	0.782606509
Casp7	0.254641218	0.91082801
Casp8	-0.03306494	0.993103295
Casp8ap2	-0.233067257	0.724910334
Casp9	0.86687591	0.795563654

Rpl35a-ps7	1.868916041	0.948104382
Casq2	-0.642303159	0.74463793
Cass4	-0.09594652	0.985742667
Cast	-0.205193426	0.810246066
Gpc6	-0.836385201	0.404461151
Castor2	-0.019754805	0.980339705
Casz1	0.184925549	0.954469162
Cat	0.006295538	0.994806961
Catip	0.206641581	0.894953269
Catsper2	-0.171593263	0.935510251
Cyp2j13	1.862646792	0.77176973
Catsperd	-0.247511519	0.957499962
Catspere2	-0.076540581	0.97487264
Catsperg1	0.112310262	0.980339705
Catsperg2	0.200492555	0.993103295
Catsperz	-0.048050276	0.98526072
Cav1	0.136225167	0.968005385
Cav2	-0.051555952	0.969755848
Cav3	0.159602424	0.980896205
Cavin1	0.100612821	0.950417507
Cavin2	0.246495198	0.731132047
Cavin3	-0.000545757	0.999242708
Cavin4	0.448957394	0.878119351
Cbarp	-0.134137088	0.691803511
Tnfaip3	-0.8277998	0.161387122
Cbfa2t2-ps1	-0.18761269	0.975800973
Sh3bgr	0.827149048	0.04370223
Cbfb	0.17887149	0.746275361
Cbl	-0.576240243	0.845024856
Pcdha4	0.81193395	0.398534324
Cbll1	0.157072222	0.944753667
Cbln1	-0.012641328	0.996487647
Cbln2	-0.009945179	0.997115148
Cbln3	0.516212631	0.911631946
Cbln4	-0.116403917	0.921311891
Cbr1	-0.435031768	0.889972515
Cbr2	-0.40858674	0.906200009
Cbr3	0.035120109	0.985673938
Cbr4	0.031045561	0.981502501
Pvalb	0.801633928	0.303490982
Cbwd1	0.126053492	0.895520088
Cbx1	0.285325741	0.742422553
Cbx2	0.051020919	0.985673938
Cbx3	0.590145266	0.585734325

Cbx3-ps2	0.840693548	0.87564489
Cbx3-ps5	0.773131915	0.895198797
Cbx3-ps6	0.373869454	0.863828723
Cbx3-ps7	0.05541828	0.973100313
Cbx3-ps8	-0.124363522	0.99527876
Cbx4	0.117958343	0.822849224
Cbx5	-0.050022631	0.901479608
Cbx6	0.290328864	0.928672524
Cbx7	0.014042803	0.988034453
Cbx8	0.290202115	0.668637933
Cby1	0.146021942	0.535430992
Cby3	-0.76040815	0.91462326
Cc2d1a	0.124185286	0.815987743
Cc2d1b	-0.015129228	0.991996237
Cc2d2a	-0.067613194	0.958951979
Cc2d2b	-0.453014457	0.965520236
Ccar1	-0.102236177	0.813552442
Ccar2	0.031705667	0.962648047
Ccbe1	0.15178095	0.549579107
Ccdc102a	0.243639148	0.935822376
Ccdc103	0.30131738	0.786324313
Ccdc105	-0.724817488	0.889908105
Ccdc106	0.11126078	0.825249193
Ccdc107	0.306213045	0.531625233
Ccdc110	0.764303956	0.851875536
Ccdc112	-0.034429626	0.974962198
Ccdc113	0.195010287	0.961887657
Ccdc114	-0.025397164	0.994960308
Sema3f	0.798932845	0.442302609
Ccdc116	-0.243428659	0.932673884
Ccdc117	-0.125187133	0.911631946
Cmtm3	0.779379185	0.176564196
Ccdc120	-0.230028789	0.690084266
Ccdc121	-0.34688216	0.940059526
Ccdc122	-0.245778131	0.912042382
Ccdc124	0.160819873	0.557507639
Fitm1	1.860265346	0.767515266
Ccdc126	-0.099960065	0.939393695
Ccdc127	-0.051577323	0.962648047
Ccdc13	-0.058530741	0.975800973
Ccdc130	0.135871536	0.871869598
Ccdc134	-0.156331915	0.711494725
Ccdc136	0.099395921	0.852035098
Ccdc137	0.127805039	0.871869598

Ccdc138	0.152594817	0.954377438
Ccdc14	0.302865588	0.753612877
Ccdc24	-0.777380497	0.084047203
Ccdc142	-0.26582579	0.873753967
Ccdc142os	0.207277377	0.968005385
Ccdc146	-0.279212949	0.948104382
Ccdc148	-0.111284489	0.866564418
Ccdc149	0.047399099	0.956164302
Ccdc15	0.359995782	0.932673884
Slco1a5	1.859571754	0.868797028
Ccdc151	-0.125500463	0.958389182
Ccdc152	0.468326818	0.974823941
Ccdc153	0.041067161	0.991438757
AI481877	1.856125728	0.869437456
Ccdc155	0.090852795	0.97607383
Ccdc157	-0.019153891	0.985742667
Ccdc158	-0.369741182	0.922100633
Ccdc159	0.038393121	0.983479691
Ccdc160	-0.014791733	0.99527876
Ccdc162	-0.143921069	0.968005385
Ccdc163	-0.01347408	0.996196093
Ccdc166	0.166080787	0.890692547
Ccdc167	0.109367008	0.911631946
Ccdc169	-0.148071757	0.993290512
Ccdc17	0.318877089	0.89703631
Ccdc170	0.008556114	0.998010523
Ccdc171	0.250903261	0.888622599
Ccdc173	-0.146917267	0.962648047
Ccdc174	-0.034690684	0.97487264
Sh2b2	0.776742095	0.146214787
Ccdc177	0.142576062	0.710708533
Ccdc178	0.032110682	0.998010523
Ccdc18	-0.307325763	0.962648047
Ccdc180	-0.569786493	0.931516666
Ccdc181	-0.037104599	0.974823941
Ccdc183	0.693078728	0.685353017
Ccdc184	-0.159709584	0.875056988
Ccdc185	0.111602698	0.99527876
Ccdc186	-0.132783347	0.707777756
Ccdc187	-0.282473946	0.890704638
Ccdc188	-0.488971048	0.8549794
Armt1	0.769416956	0.086839654
Tekt5	0.76931118	0.237504632
Ccdc191	0.220141929	0.895198797

ccdc198	-0.92792968	0.974823941
Ccdc22	0.19584241	0.873797321
Rbm3-ps	0.768551996	0.32516359
Ccdc25	-0.037085545	0.969755848
Ccdc27	-0.224948132	0.993103295
Ccdc28a	0.209831126	0.560627672
Abi3	-0.767753074	0.2131851
Ccdc3	-0.10476583	0.809571745
Ccdc30	-0.112777335	0.87847909
Ccdc32	-0.051383365	0.959659722
Ccdc33	0.32861511	0.90358675
Ccdc34	-0.105365221	0.909832498
Ccdc36	-0.268002927	0.98340069
Ccdc38	0.483411554	0.837755777
Ccdc39	0.13542519	0.780633205
Ccdc40	0.065309255	0.985742667
Ccdc42	0.420080898	0.881026445
Ccdc43	0.021370036	0.988034453
Ccdc47	0.047905691	0.928957615
Ccdc50	-0.013062262	0.988553438
Cdc45	0.765293768	0.310553098
Ccdc57	-0.175325202	0.803910915
Mlph	0.762630214	0.079050693
Ccdc59	0.174114775	0.619798471
Ccdc6	0.036619176	0.970348278
Ccdc60	-0.499770079	0.783611405
Ccdc61	-0.061603126	0.974809035
Ccdc62	0.094646226	0.965520236
Ccdc63	0.664975892	0.810908327
Ccdc65	0.006872031	0.995411429
Ccdc66	0.074874893	0.969755848
Ccdc68	-0.557809762	0.965520236
Ccdc69	0.20024872	0.968005385
Ccdc71	0.740457239	0.91462326
Ccdc71l	-0.021425105	0.985673938
Ccdc73	-0.052003739	0.97487264
Ccdc74a	0.033108186	0.97469529
Ccdc77	-0.296033963	0.529257466
Ccdc78	-0.320508428	0.941857776
Ccdc7b	-0.586551035	0.957499962
Ccdc8	0.018294502	0.99527876
Ccdc80	0.177905276	0.93685004
Ccdc81	-0.100047132	0.975831674
Ccdc82	0.08195162	0.939592821

Ccdc84	0.048773588	0.974823941
Ccdc85a	0.105332175	0.80818949
Ccdc85b	-0.127798025	0.814814675
Ccdc85c	-0.018114362	0.985673938
Ccdc86	-0.043926011	0.974809035
Ccdc87	-0.131572291	0.962923888
Ccdc88a	-0.061988127	0.962648047
Ccdc88b	0.121027227	0.962648047
Gas2l1	-0.761293907	0.00382139
Ccdc89	0.470295688	0.92874976
Ccdc9	0.130072144	0.795833907
Ccdc90b	-0.119732403	0.893784925
Ccdc91	0.107616345	0.783229859
Ccdc92	0.044827582	0.956164302
Ccdc92b	0.034915432	0.963500155
Slc13a3	-0.750368719	0.434762828
Ccdc96	-0.080917297	0.975800973
Ccdc97	-0.082704125	0.889908105
Ccdc9b	0.191823899	0.858549868
Tnfaip2	-0.745857784	0.440528706
Cchcr1	0.155306605	0.951355313
Atp8b1	-0.744006719	0.46959863
Cck	-0.057987421	0.949450803
Cckbr	-0.014844059	0.99527876
Ccl12	0.921861464	0.928716457
Ccl17	0.219450661	0.962923888
Ccl19	-0.816366308	0.962812066
Ccl19-ps1	0.057692879	0.997258205
Ccl2	-0.619596239	0.981392364
Klk12	1.840244137	0.911631946
Ccl24	0.395603475	0.987549838
Ccl25	-0.095778377	0.929456872
Ccl27a	0.116598064	0.868415875
Ccl27b	-0.065841607	0.99527876
Ccl28	0.271382672	0.985742667
Ccl3	-0.685085276	0.770793864
Ccl4	-0.232903966	0.981045362
Ccl5	0.658602596	0.960201673
Ccl6	0.477177414	0.684925922
Ccl9	0.306790382	0.882096017
Ccm2	0.00412611	0.99527876
Ccm2l	0.167262374	0.922100633
Ccna1	-0.551553679	0.745988863
Ccna2	0.094878599	0.964941885

Ccnb1	0.008939123	0.998010523
Ccnb2	-0.210750351	0.962648047
Slc16a3	0.743769905	0.350732723
Ccnc	-0.139517599	0.742435722
Ccnd1	-0.163058892	0.766366694
Ccnd2	-0.125521851	0.874971335
Ccnd3	0.0828619	0.991438757
Ccndbp1	0.048484867	0.957499962
Ccne1	-0.05576034	0.968005385
Ccne2	-0.080610347	0.965290366
Ccnf	-0.166066766	0.932673884
Ccng1	-0.106736196	0.804540244
Ccng2	-0.080729018	0.868797028
Ccnh	0.078436013	0.931610748
Ccni	-0.005964512	0.99527876
Ccnj	0.106657553	0.929133542
Ccnjl	0.044120858	0.984563037
Ccnk	0.200984787	0.558348658
Ccnl1	0.106949607	0.872352604
Ccnl2	-0.145707121	0.527779763
Ccno	-0.003753384	0.998010523
Ccnq	0.199987689	0.666064922
Ccnt1	0.014753908	0.985742667
Ccnt2	-0.140822752	0.615496563
Ccny	-0.109689435	0.829241745
Ccnyl1	-0.15440355	0.79946291
Ccp110	-0.224930001	0.529257466
Ccpg1	-0.019699535	0.985742667
Ccpg1os	-0.257862444	0.824368034
Ccr1	0.704637952	0.962648047
Ccr10	0.305627136	0.980339705
Ccr2	-0.582244805	0.970673196
Ccr5	-0.350753379	0.509834443
Ccr9	-0.909292146	0.565530155
Ccrl2	-0.861842512	0.6883862
Ccs	0.157587176	0.736486324
Ccsap	0.072848153	0.91462326
Ccser1	0.159474334	0.89703631
Clp1	0.743640711	0.063568908
Cct2	0.071339629	0.837755777
Cct3	0.084301813	0.83886846
Cct4	0.064267346	0.933285475
Cct5	0.093057456	0.688142448
Cct6a	-0.032759025	0.968859834

Cct6b	-0.971323052	0.843261802
Cct7	0.068612488	0.771450635
Cct8	0.022304194	0.97487264
Cct8l1	-0.132469936	0.985742667
Ccz1	0.053420751	0.95057572
RPS10-NUDT3	1.838993095	0.892644796
Cd109	-0.183257266	0.782270407
Cd14	-0.150506494	0.962648047
Cd151	-0.061802081	0.980419928
Cd160	-0.047557912	0.994960308
Cd163	-0.676875507	0.89703631
Cd163l1	0.036042313	0.993872582
Cd164	0.043451496	0.962648047
Cd164l2	0.092227967	0.981392364
Cd180	-0.463659267	0.644111709
Cd1d1	-0.593986877	0.810908327
Cd200	-0.030958175	0.968005385
Cd200r1	-0.609656725	0.968005385
Scn2a	-0.743157107	0.003340235
Cd209a	-0.674048971	0.973100313
Cd209b	0.437052465	0.98340069
Psg29	1.819857189	0.707777756
Cd209g	-0.758637706	0.975800973
Cd22	0.570366483	0.974823941
Cd247	-0.495897925	0.985673938
Cd248	-0.333071263	0.911631946
Cd24a	0.036147705	0.985673938
Cd27	-0.960700333	0.932673884
Cd274	0.142259702	0.962648047
Cd276	-0.061943944	0.962648047
Cd28	-0.623114378	0.974823941
Cd2ap	-0.008009522	0.99527876
Cd2bp2	0.417358327	0.869700647
Cd300a	-0.371128804	0.85252367
Cd300c2	0.19929946	0.892557138
Rps2-ps11	1.811424763	0.796330252
Cd300lb	-0.200837106	0.988034453
Cd300ld	-0.195597528	0.981045362
AC165153.2	1.811394481	0.936031628
Zfp804b	1.810589361	0.873797321
Cd300ld4	-0.360156544	0.949550088
Cd300ld5	-0.161826867	0.975831674
Cd300lf	-0.453462204	0.984232465
Cd300lg	0.109273068	0.993103295

Cd302	-0.155030023	0.795563654
Cd320	0.031793164	0.985742667
Cd33	0.037365442	0.985673938
Cd34	-0.110426221	0.836538337
Cd36	-0.007280924	0.998010523
Cd37	0.123491	0.942511266
Cd38	-0.163762607	0.886647768
Cd3eap	-0.010307933	0.993872582
Cd4	-0.095077075	0.99527876
Six1	1.80295644	0.809102338
Cd44	-0.289255565	0.89703631
Txlnb	0.742128164	0.221279819
Cd47	0.020730593	0.976978183
Cd48	0.250101737	0.962648047
Cd52	0.464254415	0.7851601
Cd53	0.1820758	0.893225143
Tmem260	-0.737036512	0.076524177
Cd59a	-0.318308977	0.810908327
Cd59b	0.104749985	0.985735386
Cd6	0.649760193	0.944391691
Cd63	0.078457167	0.921311891
Cd63-ps	0.085397905	0.974962198
Cd68	-0.183873034	0.852010446
Tulp2	1.799529936	0.728087796
Cd72	0.079436388	0.989840188
Cd74	-0.433761079	0.932673884
Arhgef16	1.797265597	0.911631946
Fas	0.7367241	0.323832374
Cd80	-0.255738487	0.968005385
Cd81	0.105054769	0.738804116
Cd82	0.163007023	0.795137348
Cd83	-0.156647931	0.727452809
Cd84	0.074595166	0.984748462
Cd86	0.061302962	0.985293518
Cd8a	-0.439061612	0.985673938
Cd9	-0.048194823	0.968859834
Insc	-0.736683762	0.414106926
Cd96	-0.447567387	0.975831674
Cd99l2	-0.016994494	0.984512582
Cda	-0.357061777	0.909943341
Cdadc1	0.034429831	0.962648047
Cdan1	0.087125276	0.924361537
Cdc123	0.045370169	0.959659722
Cdc14a	-0.149085787	0.909943341

Cdc14b	-0.45804525	0.80931022
Cdc16	-0.015662272	0.986246396
Cdc20	0.358827134	0.928099251
Cdc23	-0.03070073	0.971661054
Thbd	-0.733622357	0.133588125
Cdc25b	-0.08609586	0.937587431
Cdc25c	-0.703199508	0.910328122
Cdc26	0.070005182	0.938394547
Cdc27	-0.117470731	0.792641681
Cdc34	0.064631733	0.97487264
Cdc34b	0.703891099	0.965520236
Cdc37	0.014931033	0.982119785
Cdc37l1	-0.032674433	0.962648047
Cdc40	-0.201115064	0.5141747
Cdc42	0.089225153	0.708909794
Klhl40	0.733152683	0.020326008
Cdc42bpb	-0.030027748	0.965520236
Coro6	0.728588594	0.316730778
Mirt1	0.724934689	0.501489563
Slc7a11	-0.722157389	0.101376709
Cdc42ep3	0.066345043	0.97607383
Cdc42ep4	-0.014571384	0.993103295
Cdc42ep5	-0.219601902	0.962648047
Cdc42se1	-0.143625311	0.729712198
Cdc42se2	0.03357359	0.965520236
Bdp1	-0.719465601	0.146214787
Cdc5l	-0.03125542	0.968005385
Cdc6	0.24247381	0.965520236
Cdc7	-0.180005326	0.774896252
Cdc73	-0.052525878	0.962648047
Cdca2	-0.731880358	0.911631946
Cdca3	0.523909905	0.771684575
Cdca4	-0.20188029	0.803910915
Baat	1.795071227	0.80931022
Cdca7	0.179343103	0.911631946
Cdca7l	-0.319831995	0.810908327
Cdca8	-0.216753184	0.956072972
Gbp5	-0.714135234	0.216747565
Lrp2	-0.714090272	0.509250303
Ublcp1	0.712547805	0.073934148
Cdh13	0.064256386	0.950195611
Cdh15	0.415480932	0.810908327
Cdh18	-0.058402931	0.974823941
Cdh19	0.254379006	0.642397758

Cdh2	0.048012023	0.90358675
Cdh20	0.111043376	0.911631946
Cdh22	0.076193919	0.973100313
Cdh23	-0.126640906	0.974823941
Cdh24	-0.093436128	0.910328122
Cdh26	-0.004429399	0.999077673
Cdh4	-0.14899062	0.882824434
Cdh5	-0.322890891	0.584293468
Cdh6	-0.35611533	0.657504489
Cdh7	0.133399877	0.962648047
Cdh8	-0.016397885	0.985742667
Cdh9	0.023467396	0.985742667
Cdhr1	0.285294396	0.663393433
Cdhr2	0.716874287	0.889972515
Cdhr3	0.01795419	0.99527876
Cdhr4	-0.228278159	0.910328122
Cdip1	0.496857679	0.837755777
Cdipt	0.394689304	0.858549868
Cdiptos	0.063220047	0.991438757
Cdk1	0.057284909	0.994960308
Cdk10	0.112556776	0.882824434
Cdk11b	0.143467008	0.617743758
Cdk12	-0.180647397	0.699208985
Cdk13	-0.15225972	0.529257466
Cdk14	-0.124097365	0.817435802
Cdk15	0.620698506	0.89703631
Cdk16	-0.003989604	0.99527876
Cdk17	-0.105609173	0.574956844
Cdk18	0.126089433	0.921473132
Cdk19	-0.355918969	0.911631946
AC144408.3	1.79437664	0.8549794
Cdk2	0.008611351	0.997115148
Cdk20	0.321143479	0.69189201
Cdk2ap1	0.065638503	0.957499962
Cdk2ap2	-0.044281137	0.984328758
Cdk3-ps	0.081962383	0.985742667
Cdk4	0.039594428	0.963105252
Cdk5	0.135264907	0.606319546
Cdk5r1	-0.053672513	0.928773525
Cdk5r2	-0.077593903	0.894953269
Cdk5rap1	0.043417826	0.985293518
Cdk5rap2	-0.119711862	0.858549868
Cdk5rap3	0.097090153	0.909547396
Cdk6	0.018942902	0.99527876

Cdk7	-0.011951131	0.994960308
Cdk8	-0.077417499	0.939592821
Cdk9	-0.044344393	0.968630572
Cdkal1	-0.133025549	0.852035098
Cdkl1	0.159004799	0.86783605
Uhrf1bp1l	-0.709964809	0.003660124
Cdkl3	0.115755938	0.871869598
Cdkl4	0.0807079	0.962648047
Chrdl1	-0.708370254	0.037253581
Tcte2	0.70769401	0.121443772
Cdkn1b	-0.012014547	0.993380259
Cdkn1c	-0.246702491	0.930250446
Cdkn2aip	0.06507499	0.965520236
Cdkn2aipnl	0.066833762	0.91462326
Cdkn2b	0.0587397	0.993103295
Cdkn2c	0.265397392	0.864150749
Cdkn2d	0.105253261	0.928489442
Cdkn3	-0.786451272	0.890704638
n-R5s29	1.780620609	0.939904081
Cdo1	0.029491282	0.989840188
Cdon	0.127549636	0.962648047
Cdpf1	-0.077050234	0.966213117
Zfp119b	0.70658714	0.102706007
Cdr2l	0.059070984	0.962648047
Cdrt4	-0.465225687	0.985673938
Cds1	-0.075441798	0.91462326
Cds2	-0.079051121	0.799736155
Cdsn	0.111868524	0.985742667
Cdt1	0.458146093	0.947232837
Cdv3	0.137847025	0.749898795
Cdyl	-0.125654923	0.891972665
Cdyl2	-0.323423037	0.912600198
Ceacam1	0.261480937	0.936031628
Sgcg	1.774042134	0.5141747
Ceacam2	0.368558027	0.911631946
Cebpa	0.153769805	0.798109023
Cebpb	0.253767921	0.912042382
Cebpd	0.199121226	0.724910334
Cebpg	0.13883799	0.791185365
Cebpz	-0.15076995	0.869437456
Cebpzos	0.136488872	0.889972515
Cecr2	-0.035166346	0.993103295
Cela1	0.36700272	0.800386557
Celf1	-0.075935807	0.911834483

Celf2	-0.05469777	0.91221238
Celf3	-0.047126847	0.903795193
Celf4	-0.004254379	0.99527876
Celf5	-0.05872494	0.955908516
Celf6	-0.055179528	0.976978183
Celrr	0.355190945	0.787744512
Celsr1	-0.066022154	0.976956375
Celsr2	-0.113621356	0.527794911
Celsr3	-0.140707776	0.710375942
Cemip	0.033839816	0.98459388
Cemip2	0.044153007	0.976546786
Cend1	0.090588076	0.852010446
Cenpa	0.334138394	0.874252042
Six5	-0.703183143	0.335952215
Cenpc1	-0.140984273	0.795563654
Cenpe	0.295705351	0.903441375
Cenpf	0.414849428	0.934135315
Cenph	0.341588943	0.955908516
Cenpi	-0.376731718	0.959597516
Cenpj	-0.343113636	0.79946291
Cenpk	0.557085115	0.89703631
Cenpl	0.195207932	0.949948601
Cenpm	0.240420419	0.936507368
Cenpn	0.093793593	0.985673938
Cenpo	0.019688052	0.99527876
Cenpp	0.345639712	0.899097089
Cenpq	-0.239578669	0.871869598
Cenps	-0.025204823	0.99527876
Cenpt	0.086162535	0.947745582
Cenpu	-0.200615877	0.957499962
Cenpv	0.153467229	0.758595939
Cenpw	0.327747649	0.742969797
Cenpx	-0.385831147	0.723108545
Cep104	-0.111327381	0.751863834
Cep112	-0.160075854	0.965320168
Rnf170	0.69997639	0.449795449
Cep120	-0.042098926	0.956922504
Cep126	-0.159916283	0.889908105
Cep128	0.219411412	0.872954356
Cep131	0.071052844	0.891265024
Cep135	-0.173374293	0.872352604
Cep152	-0.103173545	0.957485565
Cep162	-0.19638531	0.754457658
Cep164	-0.145624821	0.836720624

Cep170	-0.06225542	0.962648047
Cep170b	-0.220778006	0.921311891
Cep19	0.074261647	0.874252042
Cep192	-0.164456627	0.860142574
Map3k6	0.696092085	0.065698296
Cep290	-0.011377481	0.99527876
Ebf1	0.69577005	0.492371645
Cep295nl	-0.143375224	0.994960308
Ddx4	-0.694383888	0.388363872
Cep41	-0.13213248	0.91907636
Cep44	-0.00740783	0.99527876
Cep55	-0.376699038	0.950195611
Cep57	0.097026102	0.948104382
Cep57l1	0.08260663	0.964011524
Cep63	-0.055597554	0.953505913
Cep68	-0.120943062	0.780007405
Cep70	0.026029619	0.985742667
Cep72	-0.301275579	0.928773525
Cep76	-0.075163537	0.957849305
Cep78	-0.112283663	0.875723186
Cep83	-0.130210028	0.754239817
Cep83os	0.072395388	0.895520088
Cep85	0.186077339	0.580668545
Arrdc2	0.692322803	0.088465115
Cep89	0.026921384	0.985673938
Cep95	-0.093038946	0.936031628
Cep97	-0.075716523	0.936834221
Cept1	-0.065204389	0.944391691
Cercam	0.200184978	0.810767226
Cerk	-0.00410347	0.99527876
Cerkl	-0.034203315	0.991996237
Cers1	-0.40668562	0.812431087
Cers2	0.07001767	0.916370249
Cers3	-0.135785804	0.98302504
Cers4	0.039538936	0.962648047
Qdpr	-0.69157416	0.037109546
Cers6	-0.026056321	0.968005385
Hist1h4d	1.765473712	0.756543929
Ces2g	-0.839321017	0.97487264
Ces5a	-0.387984799	0.939503568
Cetn2	0.059018699	0.946985353
Cetn3	0.083552386	0.950460219
Cetn4	-0.127665046	0.96188696
Cfap100	-0.041867752	0.97487264

Zfp57	-0.690769594	0.135479179
Cfap157	-0.305483092	0.91462326
Cfap161	0.494752355	0.813552442
Cfap20	0.01628019	0.985742667
Cfap206	-0.482431091	0.837755777
Hao2	1.762695573	0.900700653
Cfap298	0.25035083	0.704858662
Cfap299	0.975684002	0.629280869
Cfap300	0.005213022	0.996094752
Cfap36	0.029035811	0.976978183
Cfap43	-0.010404263	0.997115148
Cfap44	-0.132838364	0.92380088
Cfap45	0.133880295	0.962648047
Cfap46	0.302961733	0.869437456
Cfap52	-0.076559716	0.988208387
Cfap53	0.183196076	0.985742667
Cfap54	-0.26213695	0.862838439
Cfap57	-0.276278648	0.957485565
Cfap58	-0.735151139	0.819778986
Cfap61	-0.111817679	0.971891838
Cfap65	0.010879122	0.99527876
Cfap69	-0.13960857	0.869437456
Cfap70	-0.262805078	0.955993235
Cfap73	0.17754767	0.985673938
Cfap74	-0.041762681	0.970461999
Cfap77	0.787402671	0.764783842
Cfap97	-0.041223535	0.965520236
Adprhl1	1.760631995	0.86783605
Cfap97d2	-0.131603966	0.982934928
Cfap99	-0.068718015	0.99527876
Cfb	-0.394597524	0.873753967
Cfdp1	0.026092331	0.975800973
Cfh	-0.434370945	0.710375942
Chst10	-0.681268575	0.136332604
Cfl2	0.054205133	0.950195611
Cflar	-0.094606728	0.921311891
Cfp	-0.077712011	0.962648047
Cftr	-0.718202115	0.87847909
Cgas	-0.370200128	0.750303621
Cggbp1	-0.131042318	0.663732405
Tnfrsf12a	0.67747977	0.265544986
Lrrc26	-0.677346063	0.509250303
Cgref1	0.179672159	0.814191877
Cgrrf1	-0.006021823	0.99527876

Fmo2	0.674837381	0.298968315
Chac1	0.277401772	0.770793864
Chac2	0.145684827	0.864150749
Chad	0.239376646	0.979711552
Chadl	0.073491866	0.965320168
Chaf1a	0.175235723	0.899097089
Chaf1b	0.872993294	0.737087313
Champ1	-0.047437718	0.962648047
Ankrd13c	-0.671369901	5.29215E-05
Chchd10	0.141268126	0.84870301
Chchd2	0.096113939	0.858549868
Chchd2-ps	0.494131373	0.964011524
Chchd3	0.146855191	0.89703631
Chchd4	0.028344821	0.974823941
Chchd5	0.093338596	0.965520236
Chchd6	-0.102340275	0.894430149
Chchd7	0.073062559	0.962648047
Chd1	-0.059936189	0.959644051
Chd1l	0.01815044	0.989840188
Chd2	0.091527119	0.874252042
Chd3	-0.016403247	0.980132658
Chd4	0.018755397	0.978384252
Chd5	-0.005070268	0.99527876
Chd6	-0.14306406	0.716272713
Chd7	0.029992621	0.984328758
Lca5	-0.671002087	0.490781464
Chd9	-0.009380644	0.993103295
Chdh	0.240747972	0.816503564
Chek1	-0.203117595	0.962648047
Chek2	-0.057443904	0.985742667
Cherp	-0.027853803	0.970461999
Chfr	0.070336863	0.921473132
Chga	-0.004001541	0.99527876
Chgb	0.056127623	0.911276003
Chia1	0.167701287	0.962648047
Chic1	-0.07812366	0.932673884
Chic2	0.011627958	0.993380259
Chid1	-0.012647175	0.985742667
Chil1	-0.049801524	0.970461999
Chil3	0.33537937	0.989840188
Chil5	-0.577813125	0.964941885
C3ar1	-0.669663584	0.475240163
Chka	0.049178452	0.965151504
Chkb	0.071160911	0.971661054

Chl1	-0.168154668	0.759365171
Smad7	0.668507688	0.043497858
AC113125.1	1.740529442	0.910524966
Chmp1a	-0.000738397	0.998010523
Mlst8	0.667914099	3.21678E-06
Spsb1	0.667800073	0.009690442
Chmp2b	0.090009069	0.868415875
Chmp3	0.013851791	0.985673938
Riok3	0.664450801	0.000299151
Chmp4c	-0.434694541	0.815987743
Chmp5	0.02937844	0.970461999
Chmp6	-0.002899925	0.997115148
Chmp7	-0.045366664	0.912042382
Chn1	-0.063985267	0.903441375
Chn1os1	-0.460874816	0.911631946
Chn1os3	-0.105210547	0.949607377
Chn2	0.342043288	0.724085902
Chodl	-0.497715187	0.968005385
Prokr2	-0.662005019	0.144051788
Chp1	-0.071794959	0.932019556
Chp2	0.096486152	0.985673938
Chpf	0.104425413	0.782546806
Chpf2	0.044868797	0.962648047
Chpt1	0.211956198	0.728087796
Stat5a	0.660476064	0.480408134
Chrd	-0.033969447	0.974823941
AC026385.1	0.660434708	0.267357838
Chrdl2	0.220686692	0.974823941
Parp14	-0.658633947	0.238827832
Chrm2	-0.224626015	0.89703631
Chrm3	-0.153002048	0.758595939
Chrm4	-0.281976061	0.746275361
Chrm5	0.223182407	0.932673884
Chrna1	0.135335518	0.97487264
Chrna1os	0.056247271	0.991996237
Chrna2	0.733370765	0.790435748
Chrna3	-0.322937727	0.861909929
Chrna4	-0.33823569	0.947745582
Chrna5	-0.273933911	0.850260513
AC118542.1	-0.657221288	0.502326577
Chrnb1	-0.368350462	0.871869598
Chrnb2	0.05822613	0.933285475
Chrnb3	0.455960262	0.947745582
Chrnb4	-0.318670286	0.975831674

Chst1	0.002432223	0.996638606
Sstr4	-0.655738516	0.015062823
Chst11	-0.038497111	0.953505913
Chst12	0.098133321	0.911276003
Chst14	-0.158350784	0.962648047
Chst15	-0.075527485	0.904480183
Chst2	-0.022322502	0.975831674
Chst3	-0.171960936	0.945663104
Chst5	-0.560486592	0.837755777
Chst7	0.335282863	0.89383516
Chst8	0.022797471	0.992040075
Chst9	0.288512556	0.858549868
Chsy1	-0.093239068	0.916426816
Chsy3	0.162972434	0.92517502
Chtf18	-0.502887285	0.816618206
Chtf8	-0.145801175	0.661684145
Chtop	0.095789381	0.981045362
Chuk	-0.067887661	0.935897306
Churc1	0.068443771	0.943094393
Ciao1	0.009525482	0.991996237
Ciao2a	0.023675802	0.98526072
Ciao2b	0.244485892	0.610479949
Ciapin1	-0.013812053	0.986246396
Ciart	0.22948738	0.609167039
Cib1	0.017653366	0.993103295
Cib2	0.091002342	0.924707867
Mir9-3hg	-0.6488142	0.078339326
Cidea	0.171934477	0.84870301
Cideb	0.214438565	0.895772286
Ciita	-0.514018823	0.871869598
Cilp	0.476212121	0.981392364
Cilp2	-0.269953821	0.93912725
Myom1	0.643861672	0.495252404
Cip2a	-0.086713933	0.97487264
Cipc	0.153398796	0.68787219
Cir1	0.065976932	0.949450803
Cdr2	0.643354882	0.115563764
Cisd1	0.117992925	0.736486324
Cisd2	-0.046675166	0.953505913
Cisd3	0.152641417	0.869316117
Cish	0.088452171	0.977133618
Cit	0.050598153	0.974823941
Cited1	0.464142197	0.889972515
Cd93	-0.642047545	0.148467541

Cited4	0.460131119	0.927471971
Ciz1	0.003944625	0.99527876
CK137956	0.089604405	0.98459388
Ckap2	-0.203193009	0.959801186
Ckap2l	-0.626711851	0.76998058
Ckap4	-0.129284302	0.767043922
Ckap5	-0.072201036	0.852010446
Ckb	0.011169657	0.984843578
Cklf	0.39179177	0.7851601
Mir6392	1.739493559	0.821998633
Ckmt1	0.173011705	0.591119873
Tlr3	-0.64091483	0.041568504
Cks1b	0.446193827	0.67239949
Cks2	0.294727324	0.936031628
Clasp1	-0.136453301	0.927471971
Clasp2	-0.081641734	0.852010446
Clasrp	-0.027814137	0.971661054
Clba1	-0.120395875	0.873797321
Clca3a1	-0.076170132	0.991996237
Clca3a2	-0.240960254	0.988383065
Clcc1	-0.000543207	0.999227272
Clcf1	-0.258644596	0.953505913
Clcn1	-0.263449698	0.886647768
Clcn2	-0.071020983	0.911631946
Clcn3	-0.040273342	0.971138085
Clcn4	-0.080474589	0.670918119
Clcn5	0.088855108	0.962648047
Clcn6	-0.379552914	0.891972665
Clcn7	-0.106803228	0.779415976
Yeats4	0.640547863	0.064044233
Cldn1	0.424515082	0.847678217
Cldn10	-0.06589398	0.949607377
Cldn11	0.121472546	0.891972665
Ltbr	-0.63675264	0.070905587
Ripk3	1.71811777	0.909943341
Milr1	1.704809586	0.711294758
Cldn19	-0.846930594	0.84870301
Cldn2	0.462263619	0.92027281
Zfp663	1.693759789	0.908355387
Klk4	1.690615866	0.938615214
Cldn23	-0.216437176	0.97607383
Cldn24	0.161707819	0.965290366
Cldn3	-0.155600029	0.991996237
Cldn34c1	-0.519425374	0.833916587

Trim30e-ps1	1.68785835	0.939052665
Cldn5	-0.17986789	0.816368402
Cldnd1	-0.04090032	0.975800973
Ifi206	1.682630916	0.939052665
Clec11a	-0.362771618	0.621993647
Clec12a	0.624084694	0.963105252
Clec14a	-0.492657252	0.553031868
Clec16a	-0.110880871	0.974823941
Clec18a	0.253380306	0.922100633
Clec1a	-0.292109611	0.87847909
Olfr536	1.682630916	0.939052665
Clec2d	0.265780877	0.872954356
Clec2i	0.034644945	0.998010523
Clec2l	0.111926533	0.871869598
Clec3b	-0.198559572	0.965520236
Psca	1.682630916	0.939052665
Clec4a2	0.127409353	0.985293518
Clec4a3	0.655947076	0.86783605
AC133525.3	1.682111345	0.939052665
Xirp1	1.679324007	0.710772024
Clec4n	-0.849146753	0.975831674
Clec5a	0.077581345	0.984328758
Clec7a	-0.191876697	0.975800973
Clec9a	-0.07479433	0.991996237
Clgn	-0.02241002	0.985673938
Lmod3	1.679217856	0.875995577
Clic1	-0.145129365	0.911631946
Clic3	0.071196356	0.991438757
Clic4	-0.01297928	0.991438757
Clic5	-0.022395271	0.986810631
Clic6	0.761708402	0.661684145
Clint1	-0.080474045	0.872352604
Clip1	-0.14652588	0.813499809
Clip2	-0.100499472	0.732729469
Clip3	-0.065888095	0.818088737
Clip4	-0.185266057	0.813716057
Clk1	0.03925598	0.961373288
Clk2	0.146548728	0.70724751
Clk3	0.001086576	0.998010523
Clk4	0.006348684	0.99527876
Clmn	0.034000332	0.957499962
Clmp	-0.088838552	0.937726496
Cln3	-0.072620996	0.962648047
Cln5	0.041824432	0.976374627

Cln6	0.073469993	0.966213117
Cln8	-0.1121428	0.84277074
Clnk	-0.407161239	0.975831674
Clns1a	0.009112989	0.990550129
Clock	-0.076463238	0.875723186
Gas2l3	-0.631602293	0.369497288
Clpb	-0.033455422	0.971661054
Clpp	0.142489862	0.615496563
Clptm1	-0.032970964	0.967485535
Clptm1l	-0.070561175	0.865075531
Clpx	0.013465437	0.991996237
Clrn1	0.678607732	0.554782791
Clrn2	-0.057737031	0.99527876
Clspn	0.385507597	0.788592804
Clstn1	0.034494553	0.953505913
Clstn2	-0.044723241	0.915297582
Clstn3	-0.088997375	0.904346827
Clta	0.01259512	0.987342614
Cltb	0.090044929	0.758595939
Mthfs	0.63030385	0.121443772
Pbld2	0.628500172	0.442302609
Cluap1	-0.034650823	0.971138085
Cluh	0.237387242	0.56399886
Clvs1	0.072209662	0.962648047
Clvs2	0.119702046	0.773426516
Clybl	0.105942811	0.837755777
Cym	1.676382126	0.939052665
Cmas	0.109709004	0.609167039
Cmbl	0.302582487	0.522639613
Cmc1	-0.096795378	0.935897306
Cmc2	0.1370538	0.959276014
Cmc4	-0.307724049	0.828907376
Cmip	-0.089135852	0.643053315
Cmklr1	0.187293386	0.90358675
Cmpk1	0.00154185	0.997375151
Cmpk2	-0.024580292	0.980419928
Cmss1	-0.017627124	0.993103295
Rsrc1	0.625954446	0.039853793
Cmtm4	-0.030558133	0.962648047
Cmtm5	-0.169429969	0.797651301
Creb5	-0.623517465	0.261420427
Cmtm7	0.307735489	0.924707867
Cmtm8	0.090854984	0.984675558
Cmtr1	0.005492599	0.99527876

Cmtr2	0.13820836	0.942812446
Cmya5	0.270443839	0.67047476
CN725425	-0.995992713	0.953505913
Mrps21	0.622812623	0.031985521
Cnbp	0.021143773	0.97487264
Ssmem1	1.667295954	0.940431936
Slc11a1	0.622713881	0.477528928
Cnep1r1	-0.133050578	0.841017002
Cnga2	0.68097046	0.968977192
Cnga3	-0.101819455	0.99527876
Cnga4	-0.265499995	0.958085726
Cngb1	0.496812418	0.89011487
Cnih1	0.049989	0.928957615
Cnih2	-0.078425857	0.931745318
Cnih3	0.076063649	0.920357668
Cnih4	-0.01337393	0.988034453
Zp3	1.667295954	0.940431936
Tfeb	-0.621580041	0.014874927
Cnksr3	0.283906814	0.723108545
Cnmd	-0.550928835	0.911631946
Cnn1	-0.311615822	0.965290366
Cnn2	-0.450275537	0.664907827
Cnn3	-0.009262572	0.99527876
Cnnm1	-0.085792554	0.833916587
Cnnm2	-0.027449778	0.976978183
Cnnm3	-0.072699737	0.949607377
Cnnm4	0.124870638	0.869316117
Cnot1	-0.097856845	0.767936552
Cnot10	0.017471547	0.985673938
Cnot11	-0.092259268	0.884426973
Cnot2	0.086591676	0.883426561
Cnot3	-0.145636956	0.549579107
Cnot4	-0.117036779	0.89703631
Cnot6	-0.126088205	0.702951568
Cnot6l	0.033853255	0.968005385
Cnot7	-0.162330496	0.704844245
Cnot8	0.024248394	0.975800973
Cnot9	0.023229825	0.980339705
Cnp	0.09666808	0.868415875
Ccdc115	0.620806363	0.001940163
Serpinb7	1.66251774	0.940431936
Cnpy2	0.000724043	0.99900438
Cnpy3	-0.06114149	0.913674735
Cnpy4	-0.066967499	0.955026616

Lyl1	-0.620179958	0.465255523
Cnr2	-0.028930951	0.99527876
Cnrip1	0.030191249	0.974197921
Cnst	-0.126470734	0.67239949
Cntd1	0.303836455	0.896196194
Cntf	0.1549436	0.97487264
Cntfr	0.043121308	0.965290366
Cntln	-0.01062866	0.99527876
Cntn1	-0.121082883	0.619798471
Cntn2	0.052608287	0.972218452
Cntn3	-0.125390873	0.939393695
Cntn4	0.153685137	0.911631946
Cntn5	-0.447210778	0.84277074
Cntn6	0.32430911	0.70602193
Cntnap1	-0.001790996	0.997258205
Cntnap2	-0.130712871	0.598116371
Cntnap3	-0.0400077	0.984232465
Cntnap4	-0.234452811	0.623438925
Cntnap5a	0.079013259	0.957499962
Col6a4	0.620106352	0.323832374
Slc27a3	0.617021844	0.269754422
Asap3	-0.614582912	0.134058191
Cntrob	0.10873052	0.891972665
Coa3	0.050369956	0.961373288
Coa4	0.124905977	0.962648047
Coa5	-0.012248115	0.985446046
Coa6	-0.058081254	0.971138085
Coa7	0.210161827	0.975800973
Coasy	0.0775181	0.951390592
Cobl	-0.145681922	0.911631946
Cobll1	-0.226791192	0.819726438
Coch	-0.593943907	0.84870301
Cog1	-0.01815371	0.98459388
Cog2	0.092914082	0.894953269
Cog3	-0.043938733	0.956164302
Cog4	-0.063270025	0.901129754
Cog5	-0.071203202	0.911631946
Cog6	-0.1006538	0.786324313
Cog7	-0.038484249	0.962648047
Cog8	-0.186779484	0.804540244
Coil	0.016776587	0.985742667
Col10a1	0.730781252	0.97607383
Cfap126	-0.613988389	0.504573153
Col11a2	-0.026281112	0.983977391

Col12a1	0.038903821	0.985673938
Col13a1	-0.211014472	0.975831674
Col14a1	0.433109164	0.911276003
Col15a1	0.202668532	0.864280235
Col16a1	-0.095383689	0.919785445
Col17a1	-0.952564488	0.974809035
Col18a1	0.061051827	0.98606288
Rac2	-0.611905429	0.157002576
Dcc	-0.611726418	0.209137754
Col1a2	-0.690242448	0.852010446
Col20a1	0.163675313	0.957849305
Col22a1	0.455895679	0.760547704
Col23a1	-0.253912569	0.911631946
Snora73b	1.659572297	0.873797321
Col25a1	-0.088097657	0.897878088
Col26a1	-0.115551874	0.968005385
Col27a1	-0.162040519	0.911276003
Senp2	-0.609950297	0.000530216
Col2a1	-0.450705135	0.858549868
Dnase1	-0.6096722	0.456330081
Col4a1	-0.23586999	0.759365171
Col4a2	-0.095907804	0.781552764
Col4a3	0.109200172	0.981502501
Col4a3bp	-0.026333509	0.984328758
Col4a4	-0.180359212	0.968005385
Col4a5	-0.362235291	0.822693227
Col4a6	-0.304738914	0.962648047
Col5a1	0.104171644	0.962648047
Col5a2	-0.024609531	0.985742667
Col5a3	0.323084057	0.76034043
Col6a1	-0.037842681	0.975800973
Col6a2	0.014816085	0.99527876
Col6a3	-0.216529058	0.948104382
AC122453.1	0.60893445	0.159960873
Col6a5	-0.082174077	0.985742667
Col6a6	-0.18838323	0.978984055
Col7a1	0.068010388	0.981502501
Col8a1	0.082505852	0.989378233
Col8a2	-0.308477183	0.922999286
Col9a1	-0.320826055	0.932673884
Col9a2	-0.052180629	0.991559279
Col9a3	0.227750973	0.777427974
Colca2	-0.027758889	0.99527876
Olfr990-ps1	1.652723419	0.923011738

Colec11	-0.0189224	0.99527876
Colec12	-0.178925404	0.968005385
Colgalt1	-0.172179343	0.89703631
Colgalt2	0.069234365	0.962648047
Colq	0.308614556	0.980339705
Commd1	0.142831447	0.818088737
Commd10	0.105513939	0.849526037
Commd2	-0.045461376	0.982908579
Commd3	0.211160064	0.510932065
Commd4	0.119196784	0.872265039
Azi2	-0.60803437	0.028938216
Commd6	0.010988096	0.994960308
Commd7	0.11468973	0.814301191
Commd8	-0.0315054	0.99100709
Lyz2	-0.605603528	0.31865776
Colec10	1.649809783	0.895198797
Comt	0.014908567	0.987066554
Comtd1	0.206504922	0.861366392
Cop1	-0.069078995	0.889908105
Copa	-0.011310256	0.98459388
Copb1	-0.040134156	0.949481068
Copb2	-0.031037099	0.957485565
Cope	0.14266807	0.712146795
Copg1	0.036967596	0.928957615
Copg2	-0.042354024	0.962648047
Coprs	0.136034287	0.861366392
Cops2	0.052464774	0.969755848
Cops3	0.038171899	0.962648047
Cops4	0.033341622	0.965520236
Emilin2	0.60376711	0.333498689
Cops6	0.105550945	0.729344189
Cops7a	0.019128611	0.981694523
Cops7b	-0.04391794	0.969755848
Cops8	0.035359177	0.962648047
Prss53	-0.601641808	0.349136927
Copz1	0.019465557	0.975831674
Copz2	0.075141017	0.971661054
Coq10a	0.077620218	0.90358675
Coq10b	-0.144992174	0.759486652
Coq2	-0.062175765	0.923874063
Coq3	-0.082858596	0.904480183
Coq4	-0.092391618	0.91462326
Coq5	0.007180686	0.99527876
Coq6	0.2128354	0.544361994

Coq7	0.149261847	0.719408219
Coq8a	0.128075548	0.911631946
Coq8b	-0.040503605	0.978063275
Coq9	0.127727311	0.620124996
Corin	-0.723795976	0.950302722
Coro1a	-0.040584763	0.965520236
Coro1b	0.030957989	0.965520236
Coro1c	0.04856082	0.932673884
Coro2a	0.112756631	0.822337024
Coro2b	0.015224011	0.984328758
Rpl5-ps2	0.600204077	0.403784647
Coro7	-0.100293931	0.716433689
Cort	0.046418986	0.985742667
Cotl1	0.084627342	0.889972515
Cox10	-0.087575701	0.902196538
Cox11	0.235630518	0.700354055
Cox14	0.050039007	0.968005385
Cox15	0.056891847	0.936905058
Cox16	0.150651182	0.962648047
Cox17	0.065083601	0.962636653
Cox18	-0.249462935	0.855344864
Cox19	0.0847446	0.883426561
Cox20	0.074487512	0.948104382
Sp100	-0.598376565	0.388066232
Cox4i2	0.505219871	0.84870301
Cox5a	0.043111238	0.962923888
Gpcpd1	-0.590860112	0.084102849
Cox6a1	0.035026376	0.968005385
Pla2g3	0.583633531	0.394955649
Zbtb45	0.583079651	0.139445017
Cox6b2	-0.130822252	0.968005385
Cox6c	0.165646333	0.76998058
AC166110.2	1.645607884	0.950195611
Plpp4	0.582677548	0.186938452
Calb2	0.581836406	0.302984782
Cox7a2l	0.156692419	0.56399886
Castor1	0.580203918	0.255935042
Tnfsf8	1.64515002	0.942812446
Prrx1	-0.579897095	0.104146198
Cox8a	-0.164691612	0.854434649
Clec1b	1.641691424	0.889908105
Cp	-0.254428764	0.923602336
Cpa2	0.593101929	0.6883862
Btnl10	1.633768598	0.704062454

Cpa6	0.406047512	0.980339705
Zfa-ps	1.631982786	0.80931022
Cpb2	-0.113286383	0.99527876
Rfx2	-0.577068705	0.319241271
Cpe	0.056350028	0.861366392
Cpeb1	0.012225609	0.985742667
Cpeb1os1	0.103614606	0.964011524
Cpeb2	-0.322043487	0.529257466
Cpeb3	-0.197107972	0.84870301
Cpeb4	-0.158081226	0.854278008
Cped1	-0.329189274	0.954469162
Rps24-ps3	0.576365052	0.113604388
Cplx1	0.048540631	0.934798735
Cplx2	0.073022385	0.791185365
Cplx3	0.472607978	0.629280869
Cpm	0.151150055	0.736130784
Cpn1	-0.416210444	0.984328758
Cpne1	-0.679904602	0.866560751
Cpne2	0.026353239	0.985742667
Cpne3	-0.147134812	0.777427974
Cpne4	-0.070201007	0.823072541
Cpne5	-0.154578869	0.723108545
Cpne6	-0.020614468	0.975831674
Cpne7	-0.002252784	0.997529192
Cpne8	-0.273787769	0.513797506
Cpne9	-0.071367991	0.911631946
Cpox	-0.037384997	0.962648047
Cpped1	0.074246408	0.924353966
Cpq	0.056884244	0.975800973
Cps1	0.478894561	0.962648047
Cpsf1	-0.026815007	0.97487264
Cpsf2	0.007541559	0.993380259
Cpsf3	0.091077928	0.810908327
Cpsf4	0.143951824	0.710928978
Cpsf4l	0.2329796	0.962648047
Cpsf6	-0.037759235	0.962648047
Cpsf7	-0.044253372	0.936031628
Cpt1a	-0.072157288	0.939592821
Cpt1b	-0.637033135	0.899249451
Cpt1c	-0.064744813	0.882096017
Cpt2	0.208388442	0.911631946
Cptp	0.020566218	0.98526072
Cpxm1	0.138384661	0.948104382
Cpxm2	-0.054596794	0.988034453

Cr1l	-0.045635328	0.965658786
Cr2	-0.741858125	0.926988689
CR974462.1	0.032869087	0.99640101
Crabp1	0.211118221	0.972179317
Atp7a	-0.576016638	0.422728382
Cracr2a	0.25055424	0.891972665
Cracr2b	0.199844796	0.945170183
Cradd	-0.085320496	0.969755848
Cramp1l	-0.006543028	0.99527876
Crat	-0.102985589	0.831837418
Crb1	0.139818286	0.985742667
Crb2	-0.044977929	0.981392364
Crb3	-0.57060951	0.968601153
Crbn	-0.001684419	0.99765039
Crcp	-0.225093597	0.955993235
Creb1	-0.649901475	0.951390592
Creb3	0.0390409	0.959179333
Creb3l1	-0.173652857	0.91462326
Creb3l2	-0.156660859	0.640856315
Creb3l3	0.190167652	0.972218452
Creb3l4	0.163982656	0.98526072
Col1a1	-0.57397061	0.493073764
Crebbp	0.046785354	0.962648047
Crebl2	0.077463037	0.852010446
Crebrf	-0.063547084	0.940743591
Crebzf	0.02267596	0.98526072
Creg1	0.05591588	0.957499962
Creg2	-0.133654396	0.544661714
Creld1	0.014475585	0.988034453
Creld2	-0.08512491	0.939884938
Crem	-0.069393466	0.964941885
Crh	-0.444513538	0.895520088
Crhbp	-0.089555703	0.915297582
Crhr1	-0.071612537	0.973100313
Crhr2	-0.165979733	0.974844452
Crim1	-0.115937004	0.702951568
Crip1	0.343147331	0.786062749
Crip2	0.064445856	0.989200732
Crip3	-0.596778682	0.749626226
Cript	0.110696046	0.780228245
Crispld1	0.020305538	0.994806961
Ear1	1.624039805	0.945117894
Mfsd2a	0.573460426	0.000625701
Crkl	-0.083129579	0.895520088

Smim15	0.572746991	0.27348311
Crlf2	-0.140872413	0.939393695
Crlf3	0.054247367	0.969476616
Setd3	0.570596799	2.90142E-14
Crmp1	0.01319698	0.987624788
Crnde	0.27733353	0.959659722
Crnkl1	0.014236469	0.988553438
Crocc	-0.012685058	0.988581198
Crocc2	-0.367849128	0.953505913
Crot	0.047554744	0.950460219
Crry-ps	0.245365707	0.974809035
Crtac1	0.060512438	0.920642026
Crtam	0.070557031	0.996094752
Crtap	0.186027808	0.891972665
Crtc1	-0.013428157	0.985742667
Crtc2	0.050407761	0.958389182
Crtc3	0.007886207	0.995269201
Cry1	-0.145839588	0.878119351
Cry2	-0.017172223	0.977055579
Prdm12	1.62341027	0.89703631
Cryab	-0.229140322	0.509787206
Ebf2	1.618543994	0.925217889
Cryba4	-0.106836574	0.985742667
Crybb1	0.166092729	0.928333994
Crybb3	-0.426685109	0.861366392
Crybg1	0.418352616	0.873753967
Crybg2	0.220632165	0.968005385
Crybg3	0.402824611	0.789833208
Cryga	-0.433969973	0.965520236
Crygn	-0.545031143	0.875723186
Cryl1	0.029871599	0.984328758
Crym	0.019428565	0.98526072
Cryz	0.095060374	0.949607377
Cryzl1	0.04972279	0.947033215
Cryzl2	0.379030417	0.609927197
Cs	-0.0565864	0.873797321
Csad	0.156541725	0.869316117
Csdc2	0.844048659	0.642397758
Csde1	0.078041377	0.932673884
Cse1l	-0.032668394	0.965520236
Csf1	-0.253817179	0.882096017
Csf1r	-0.008662182	0.994806961
Csf2ra	-0.203138025	0.737279585
Csf2rb	-0.262164099	0.962458041

Csf2rb2	0.366716797	0.812431087
Csf3r	-0.335645142	0.517996718
Tcf7l1	-0.570286623	0.377143991
Csgalnact2	-0.04163845	0.974823941
Csk	0.115386831	0.911631946
Csmd1	-0.164654232	0.736130784
Csmd2	-0.119300188	0.854409578
Csmd3	0.026826249	0.985673938
Csnk1a1	-0.038734867	0.958725703
Csnk1d	0.069273178	0.871869598
Csnk1e	-0.010026566	0.99100709
Csnk1g1	-0.660115818	0.937139141
Csnk1g2	0.062518233	0.904750766
Csnk1g3	-0.024952752	0.975831674
Csnk2a1	0.049795973	0.909604637
Csnk2a2	-0.096916562	0.777427974
Csnk2b	-0.079320603	0.874252042
Cspg4	-0.141837886	0.779231312
Cspg5	0.012659637	0.984328758
Cspp1	-0.070470389	0.948104382
Csrnp1	0.426490703	0.822597041
Csrnp2	0.021842597	0.982408617
Csrnp3	-0.123702635	0.544661714
Csrp1	0.126441111	0.572363201
Csrp2	-0.02227241	0.99527876
Cst3	0.108674679	0.716810682
Cst6	-0.118773944	0.97487264
Prkag3	1.617934783	0.736771155
Cstad	-0.154884542	0.942812446
Cstb	0.125799086	0.852010446
Cstf1	-0.017655739	0.985673938
Cstf2	-0.141247939	0.869437456
Cstf2t	0.045768257	0.904480183
Cntnap5b	-0.569418134	0.392811348
Cstl1	-0.538321564	0.965520236
AC162923.1	1.617094363	NA
Npy4r	1.615689494	0.945663104
CT009627.2	-0.171227786	0.984328758
CT009627.3	-0.250219103	0.99100709
CT010429.1	0.171556136	0.994824604
CT010433.2	0.03608964	0.993489613
CT010445.1	0.107960159	0.91462326
CT010467.1	0.393750507	0.731412373
CT010496.1	-0.084872607	0.980226283

Fam227a	-0.567376584	0.088465115
CT010502.2	0.070141116	0.994960308
CT010524.1	0.452044273	0.902647137
CT025584.1	-0.928219131	0.974823941
CT025592.1	0.071243032	0.992385525
CT025652.1	0.395673949	0.889972515
CT025652.2	0.573622877	0.959276014
CT025659.2	-0.566855807	0.852010446
CT025659.3	0.434149832	0.943152911
CT030190.1	0.311482061	0.974823941
Akap3	1.605279508	0.702951568
Drd4	1.597990657	0.873797321
CT033772.1	0.081362027	NA
Mir7655	1.591114915	NA
AC110573.1	1.588219471	0.872157363
CT486005.1	-0.294707242	0.643053315
CT571242.1	0.807628873	0.947033215
CT573017.1	0.278149448	0.964011524
CT573017.2	-0.610708994	0.89703631
Ctbp1	-0.052452605	0.891972665
Ctbp2	-0.172921762	0.596982103
Ctbs	0.039759306	0.981502501
Ctc1	-0.04466028	0.962648047
Ctcf	-0.021159644	0.984328758
Ctdnep1	0.037018177	0.959659722
Ctdp1	-0.028356449	0.98302504
Ctdsp1	-0.086044717	0.89703631
Ctdsp2	-0.133786111	0.786324313
Lrrc47	-0.566042632	0.307478675
Ctdspl2	-0.062993686	0.952981891
Ctf1	0.409210318	0.758223648
Ctf2	-0.462582751	0.981045362
Ctgf	0.439769092	0.6883862
Cth	-0.403518086	0.544361994
Cthrc1	0.303186408	0.751142243
Ctif	0.051173173	0.916961045
Ctla2a	0.215640427	0.898478018
Ctla2b	0.401707389	0.889908105
Ctla4	-0.652822418	0.984328758
Ctnna1	-0.053248276	0.960201673
Ctnna2	-0.111784116	0.740514972
Ctnna3	-5.22786E-05	0.99991697
Ctnnal1	-0.003626428	0.997115148
Ctnnb1	0.303636442	0.889908105

Ctnnbip1	0.081628482	0.932673884
Ctnnbl1	0.116080137	0.84870301
Mmrn2	-0.562458177	0.496022017
Lcp1	-0.561841205	0.226207189
Ctns	-0.023386557	0.984748462
Ctps	0.065442071	0.921311891
Ctps2	0.009196413	0.994960308
Ctr9	0.022414534	0.97469529
Msx3	1.578084462	0.842486923
Ctsa	0.021411791	0.97469529
Ctsb	-0.04737952	0.89703631
Ctsc	-0.213190481	0.815987743
Ctsd	0.112211288	0.903279667
Ctsf	0.108064497	0.6883862
Ctsh	0.008133648	0.99527876
Ctsk	0.241501112	0.936031628
Polk	-0.560323651	0.315636078
Ctso	-0.048829694	0.956922504
Sap18	0.560133737	4.58976E-07
Ctsw	0.304870672	0.984328758
Ctsz	0.06182183	0.935510251
Cttn	-0.000914254	0.998010523
Cttnbp2	-0.175936	0.732729469
Cttnbp2nl	0.045444909	0.962648047
Ctu1	-0.179158249	0.862285215
Ctu2	0.027875088	0.984512582
Ctxn1	0.057233992	0.91462326
Ctxn2	0.129971122	0.898474424
Ctxn3	-0.154542707	0.980419928
Cubn	-0.768699656	0.873797321
Cuedc1	0.195058283	0.87847909
Cuedc2	0.123837985	0.6883862
Cul1	0.008928378	0.992669879
Npffr1	0.559988169	0.294902721
Cul3	-0.109611467	0.77448401
Cul4a	0.061038703	0.89703631
Cul4b	-0.027997471	0.984328758
Cul5	0.120306692	0.864150749
Cul7	0.02998397	0.975800973
Cul9	-0.031815008	0.971661054
Cuta	0.078966499	0.961887657
Cutal	0.34481793	0.957485565
Zswim6	-0.559216989	0.001899595
Cux1	-0.092635164	0.865075531

Cux2	-0.209914961	0.796563869
Slc4a5	1.57445959	0.887709902
Cwc15	0.110340824	0.629280869
Cwc22	-0.259099635	0.80818949
Cwc25	-0.011766271	0.993103295
Fubp3	0.5582977	0.104879534
Cwf19l1	0.0665754	0.951390592
Cwf19l2	-0.099053133	0.948104382
Cwh43	0.242984241	0.962648047
Cyren	-0.557279981	0.120903042
Cx3cr1	0.238215228	0.813552442
Cxadr	0.761739792	0.928489442
Cxcl1	0.776472781	0.953505913
Nhlrc4	1.570561937	0.813346233
Cxcl12	-0.158497793	0.740833211
Pan3	0.557022668	0.146214787
Cxcl16	-0.719339935	0.816844103
Cxcl2	-0.916523847	0.74463793
Cxcl5	-0.156147611	0.987342614
Krt23	1.565954595	0.911631946
Cxcr4	0.133389846	0.962648047
Mir7032	1.56372021	NA
Cxxc1	0.028502713	0.978259864
Cxxc4	0.174803554	0.950460219
Cxxc5	-0.137013059	0.703372057
Cyb561	-0.040121989	0.974823941
Cyb561a3	-0.084705895	0.959507587
Cyb561d1	0.079217533	0.968005385
Cyb561d2	-0.282862254	0.851055037
Cyb5a	0.035444326	0.98187819
Cyb5b	0.047940624	0.899097089
Cyb5d1	0.008891855	0.99527876
Cyb5d2	-0.193552158	0.732729469
Galnt15	0.556787963	0.468043447
AC113953.1	1.563718924	NA
Cyb5r3	0.096637933	0.839207219
Cyb5r4	-0.023851867	0.984328758
Cyb5rl	0.127377906	0.962648047
Cyba	-0.067506289	0.974823941
Cybb	-0.155834684	0.985446046
Cybc1	0.00377303	0.997115148
Cybrd1	0.082117467	0.968005385
Cyc1	0.12456759	0.829437499
Cycs	-0.005718316	0.99527876

Cyfip1	-0.080702277	0.857611652
Cyfip2	-0.078866344	0.710772024
Cygb	0.175391559	0.544661714
Mboat2	0.55652485	0.387432606
Cyld	0.170665768	0.837755777
Mir26a-2	1.563718908	NA
Cyp11a1	-0.001083658	0.999227272
Mirlet7i	1.563718908	NA
Cyp1a1	-0.565414486	0.968005385
Cyp1b1	-0.676070343	0.839488666
Cyp20a1	-0.040165146	0.979037366
Cyp21a2-ps	-0.928219131	0.974823941
Olfr687	1.562872955	0.886106512
Cyp26a1	-0.514605545	0.976978183
Cyp26b1	-0.096268724	0.968005385
Cyp27a1	-0.041406293	0.992385525
Cyp27b1	-0.257865288	0.966213117
Ankrd7	1.56231465	0.949607377
Cyp2c52-ps	-0.974305911	0.962648047
Cyp2d22	0.074131742	0.897289612
Dntt	1.561430917	0.882096017
Cyp2e1	0.763305293	0.922999286
Pprc1	-0.553218582	0.146214787
Cyp2j12	-0.024419318	0.993489613
Trbj2-7	1.555490846	NA
Cyp2j6	0.100796542	0.911631946
Cyp2j8	-0.223863699	0.984328758
Cyp2j9	0.050449124	0.965520236
Cyp2r1	0.114710144	0.99527876
Cyp2s1	0.033037389	0.988481007
Cyp2t4	-0.188157354	0.962648047
Cyp2u1	0.137983138	0.921473132
Cyp39a1	0.076365468	0.985742667
Cyp3a13	0.321026684	0.89703631
Mpl	1.554761001	0.903795193
Cyp46a1	0.006296511	0.994960308
Cyp4b1	-0.62646889	0.961887657
Cyp4f13	0.007521648	0.99527876
Cyp4f14	-0.16658542	0.891972665
Cyp4f15	0.019623958	0.986246396
Cyp4f16	0.21980692	0.911631946
Cyp4f17	-0.011221057	0.997258205
Cyp4f18	-0.258681905	0.981144704
Cyp4f41-ps	-0.255075345	0.98340069

Cyp4v3	-0.142170731	0.868415875
Fkbp5	0.55256054	0.141036549
Cyp4x1os	0.152687265	0.971138085
Cyp51	-0.067528488	0.922887518
Cyp7b1	0.11554421	0.770793864
Cyr61	-0.094245794	0.972025261
Sox21	-0.552410002	0.09162748
Cys1	-0.690209679	0.698427195
Cysltr1	-0.49281663	0.90358675
Cysltr2	0.416417613	0.965520236
Plek2	1.552350884	0.932673884
Cystm1	0.098701458	0.919415586
Cyth1	0.072894062	0.952390496
Cyth2	-0.017611768	0.981392364
Cyth3	-0.04707877	0.956164302
Cyth4	-0.008686036	0.99527876
Mir6921	1.549173161	0.881407916
Cyyr1	-0.340345995	0.69189201
Arid3b	-0.54865617	0.439287299
D10Wsu102e	-0.019687988	0.984748462
D11Wsu47e	-0.047145367	0.984748462
D16Ertd472e	0.136969912	0.831215716
D17H6S53E	0.292867897	0.544661714
D17Wsu92e	0.018992733	0.985742667
D1Ertd622e	-0.092442494	0.870485449
Zcchc2	-0.548602666	0.117352514
D3Ertd254e	-0.113652207	0.855344864
D3Ertd751e	0.127403824	0.950195611
D5Ertd579e	-0.084912616	0.754457658
Smim22	1.540824485	0.852010446
D5Ertd615e	0.125792098	0.99527876
Scarna9	1.53599663	NA
D6Ertd527e	-0.384923644	0.987342614
D6Wsu163e	0.118130591	0.822597041
D7Bwg0826e	0.211542841	0.956922504
D7Ertd443e	0.251827486	0.921473132
D8Ertd738e	0.103891013	0.868415875
Daam1	0.038303723	0.953505913
Triobp	-0.548402643	0.032618947
Dab1	-0.101271927	0.625886453
Dab2	-0.596610652	0.724085902
Col19a1	-0.546769536	0.161844023
Dach1	-0.059212811	0.973100313
Dach2	0.374060784	0.946239011

Dact1	-0.312892821	0.689698377
Dact2	0.038216078	0.969755848
Dact3	-0.073289895	0.881771584
Dad1	0.002134723	0.99765039
Dag1	-0.113727326	0.627261794
Pycard	-0.546726963	0.460296955
Daglb	-0.107843614	0.84870301
Dalir	-0.211287542	0.873797321
Dalrd3	-0.069086911	0.921311891
Dancr	0.521545979	0.744046401
Dand5	-0.048934703	0.974962198
Dao	-0.351761254	0.969755848
Dap	-0.099314492	0.959276014
Dap3	0.060103917	0.91462326
Fktn	-0.546515851	0.05361418
Dapk2	0.921211866	0.587242457
Dapk3	0.061131964	0.939052665
Dapl1	-0.747739383	0.974962198
Notch4	0.546302365	0.122206254
Dars	-0.120350628	0.774225415
Dars2	0.068978758	0.944391691
Daw1	0.12099164	0.985293518
Daxx	0.07125344	0.965520236
Dazap1	0.010458588	0.992040075
Adamts2	0.544795133	0.298952496
Dazl	-0.243461485	0.911631946
Dbf4	-0.049448515	0.989840188
Prkn	0.544580837	0.394955649
Dbhos	-0.216902991	0.987269122
Dbi	0.199766386	0.620124996
Dbil5	-0.488712138	0.883426561
Dbn1	0.057713965	0.932673884
Dbndd1	0.042771225	0.975831674
Dbndd2	0.074717848	0.962648047
Dbnl	0.042511424	0.968005385
Dbp	-0.055679127	0.964176371
Dbpht2	-0.146488645	0.768330892
Dbr1	-0.02640688	0.98459388
Dbt	-0.100780097	0.90358675
Dbx2	0.057164795	0.965752549
Dcaf1	-0.09129906	0.911631946
Dcaf10	0.352105692	0.774804043
Dcaf11	0.091198981	0.852946549
Dcaf12	-0.034831484	0.962648047

Dcaf12l1	-0.054565602	0.944753667
Dcaf12l2	-0.226279658	0.949607377
Dcaf13	-0.037670476	0.962648047
Dcaf15	0.037850079	0.971138085
Fgd5	-0.543651161	0.164373636
Dcaf4	0.108498288	0.872352604
Dcaf5	-0.106877951	0.69189201
Dcaf6	0.051506281	0.91462326
Dcaf7	-0.010568163	0.985673938
Dcaf8	0.19730626	0.760547704
Dcakd	-0.123989091	0.759486652
Dcbld1	-0.036825163	0.974197921
Dcbld2	0.19148654	0.973100313
Sult1a1	0.54348771	0.020989202
Dcdc2a	-0.159112701	0.707777756
Dcdc2b	-0.309430373	0.790879485
Mir1963	1.535996395	NA
Dcdc5	0.453591359	0.961373288
Dchs1	-0.149047388	0.882096017
Dchs2	-0.319359488	0.710772024
Dck	0.05900508	0.932673884
Dclk1	-0.144998642	0.660781615
Dclk2	-0.019553977	0.974823941
Dclk3	0.138687559	0.821998633
Dclre1a	0.008196087	0.99527876
Dclre1b	-0.066374784	0.98526072
Dclre1c	0.032612674	0.985673938
Kif1bp	0.543251035	0.082828853
Dcp1a	0.051120425	0.974809035
Dcp1b	-0.011717008	0.993062852
Dcp2	0.007115447	0.994120754
Tnfrsf25	-0.542594334	0.377457124
Dcst1	-0.566778581	0.596982103
Dcst2	0.179422184	0.965520236
Dct	0.409880422	0.923011738
Dctd	0.23275772	0.767515266
Dctn1	0.029540614	0.974809035
Dctn2	0.080402472	0.815987743
Dctn3	0.123614297	0.630498632
Dctn4	0.073451888	0.813792356
Dctn5	-0.006517399	0.995944208
Krt9	0.541181659	0.091941163
Dctpp1	0.156431491	0.911631946
Dcun1d1	-0.047713726	0.968005385

Dcun1d2	-0.003506124	0.999227272
Epb41l2	-0.540383548	0.000767316
Dcun1d4	-0.086750042	0.720841863
Dcun1d5	-0.024669021	0.984232465
Dcx	0.062611777	0.965520236
Dcxr	-0.068165993	0.971138085
Dda1	0.019201666	0.985704372
Ddah1	0.025166449	0.968601153
Ddah2	0.056397633	0.968005385
Ddb1	-0.007996038	0.991438757
Ddb2	-0.121862994	0.944494911
Pla2g12a	0.538938092	0.194420918
Ddhd1	-0.111797071	0.780228245
Ddhd2	-0.020796088	0.974962198
Ddi2	-0.043461913	0.936031628
Ddias	0.422679263	0.910328122
Ddit3	0.167701228	0.724085902
Fam110b	0.537942858	0.357105886
Ddit4l	-0.140255392	0.942511266
Ddn	-0.005360975	0.99527876
Ddo	0.007413865	0.99527876
Ddost	0.040883372	0.957499962
Fam241b	0.535418187	0.464952787
Ddr2	-0.149029583	0.953505913
Ddrgk1	0.057507453	0.949607377
Ddt	0.110384588	0.89703631
Ddx1	0.084594257	0.690802386
Ddx10	-0.001423189	0.998010523
Ddx11	-0.003690136	0.998010523
Ddx17	-0.036876267	0.962648047
Ddx18	0.004651473	0.99527876
Ddx19a	0.067156961	0.911631946
Ddx19b	-0.078536361	0.894953269
Ddx20	-0.080527933	0.921311891
Ddx21	0.100313336	0.837755777
Ddx23	-0.008061898	0.993103295
Ddx24	-0.071105329	0.89703631
Ddx25	0.050416823	0.932673884
Ddx27	0.061375019	0.939592821
Ddx28	0.100858332	0.89703631
Ddx31	-0.157896535	0.874252042
Ddx39	0.063254869	0.957499962
Ddx39b	0.029193385	0.962648047
Ddx3x	0.012281823	0.985742667

Ddx3y	-0.271404879	0.661684145
Rpl31-ps9	0.535116093	0.089315573
Ddx41	0.009561763	0.992385525
Ddx42	0.065648247	0.875723186
Ddx43	0.795304692	0.951390592
Ddx46	-0.085452087	0.780228245
Ddx47	0.098851633	0.778639508
Ddx49	0.136023086	0.80931022
Ddx5	-0.020450424	0.974809035
Ddx50	0.027052199	0.974962198
Ddx51	-0.060606409	0.950460219
Ddx52	-0.057969554	0.961373288
Ddx54	-0.062435298	0.910328122
Ddx55	0.177938902	0.518646722
Ddx56	0.073349082	0.91907636
Ddx58	0.145718373	0.939052665
Ddx59	0.167820377	0.837755777
Ddx6	0.178839004	0.774804043
Ddx60	-0.22370962	0.970673196
Deaf1	0.129397322	0.578812716
Decr1	-0.056790249	0.968005385
Decr2	-0.083079443	0.882096017
Dedd	-0.158998615	0.861366392
Dedd2	-0.02561063	0.984328758
Def6	0.12646027	0.975831674
Def8	-0.003722572	0.995514978
Defb36	-0.041810499	0.99765039
Defb42	-0.067847352	0.994960308
Degs1	0.082829074	0.812033693
Degs2	-0.199389758	0.944391691
Dek	0.055416097	0.955993235
Dele1	-0.037809868	0.964011524
Dennd1a	-0.063720745	0.890692547
Dennd1b	-0.006404037	0.995411429
Dennd1c	-0.403532453	0.871869598
Dennd2a	-0.080766261	0.902293377
Dennd2c	0.767726891	0.772658285
Dennd2d	-0.804999968	0.818088737
Dennd3	0.090694416	0.962648047
Pgf	0.533167678	0.490781464
Dennd4b	0.001887442	0.997375151
Dennd4c	-0.217104881	0.510556831
Dennd5a	-0.057867829	0.8643613
Maml2	0.526404376	0.000135022

Dennd6a	-0.025175694	0.97469529
Dennd6b	0.122463505	0.648436128
Denr	0.026794541	0.97487264
Depdc1b	0.002953699	0.999486844
Depdc5	-0.023171433	0.985446046
Depdc7	-0.332652863	0.873753967
Depp1	-0.747545696	0.638898437
Deptor	0.062175935	0.969755848
Dera	-0.108428132	0.966213117
Derl1	0.02444919	0.968859834
Derl2	-0.085620123	0.858106541
Derl3	0.006911311	0.998121731
Mir365-1	1.535996395	NA
Desi1	-0.014747886	0.990835405
Paqr3	-0.525259753	0.065698296
Det1	-0.247507423	0.903441375
Deup1	-0.172149854	0.975800973
Dexi	0.017070374	0.989556168
Dffa	0.041067626	0.948104382
Dffb	0.130274992	0.897878088
Dgat1	0.079987266	0.955423359
Haus2	0.524383413	0.237504632
Dgat2l6	-0.608282171	0.757357446
Dgcr2	-0.208103824	0.813744313
Dgcr6	0.069745356	0.895520088
Dgcr8	-0.08814922	0.90358675
Dgka	0.110043535	0.891972665
Wnt3	0.521367926	0.410106728
Dgkd	-0.073515326	0.798580668
Dgke	0.037268184	0.99527876
Dgkeos	0.245973967	0.981392364
Dgkg	-0.095520549	0.667733898
Dgkh	0.234544939	0.911631946
Mt2	0.521136436	0.001387335
Dgkk	0.455098205	0.922598585
Dgkq	0.020980111	0.984232465
Cdh12	-0.519342653	0.219979549
Dglucy	-0.110142598	0.953911274
Dguok	-0.050767573	0.971138085
Dhcr24	0.014563284	0.98691726
Dhcr7	-0.29175152	0.820673991
Dhdds	0.57267266	0.648241111
Dhdh	0.090544968	0.932673884
Dhfr	0.251411151	0.710375942

Dhh	-0.917930137	0.944753667
Dhodh	-0.184526906	0.814814675
Dhps	0.100207705	0.860521845
Dhrs1	0.15062221	0.670916216
Dhrs11	0.081253032	0.965520236
Dhrs13	-0.127908359	0.962648047
Dhrs13os	0.974168059	0.928957615
Dhrs2	0.506427143	0.985673938
Dhrs3	0.21386036	0.578812716
Dhrs4	0.251307024	0.542211613
Dhrs7	0.034778107	0.97469529
Dhrs7b	0.002452139	0.99734533
Mir199b	1.533778148	0.943152911
Dhrs9	-0.332242549	0.990697821
Dhtkd1	0.060595162	0.96607835
Dhx15	-0.014833837	0.985229869
Dhx16	-0.017043945	0.988034453
Dhx29	0.068046747	0.911631946
Dhx30	0.062149719	0.950046478
Dhx32	-0.074842677	0.89383516
Dhx33	0.202290981	0.664771806
Dhx34	-0.098030188	0.936031628
Dhx35	0.091911295	0.948407374
Dhx36	-0.092632004	0.780228245
Dhx37	0.10562696	0.899249451
Dhx38	-0.017208851	0.984328758
Dhx40	0.07457642	0.894430387
Dhx57	-0.030789862	0.974823941
Dhx58	-0.412657972	0.939592821
Catsper3	1.528088479	0.588224175
Dhx8	-0.048633814	0.954174097
Dhx9	-0.057815955	0.894953269
Diablo	-0.002586847	0.99765039
Diaph1	-0.006246469	0.99527876
Diaph2	-0.019922305	0.997375151
Diaph3	-0.420516119	0.84277074
Dicer1	0.028505354	0.980132658
Dido1	-0.040159353	0.942102196
Diexf	-0.046504946	0.960176242
Dimt1	-0.054384512	0.965520236
Pla2g4b	-0.518490216	0.503479638
Dio3	0.645795665	0.919785445
Dpt	1.527077419	0.513797506
Dip2a	0.055581969	0.980226283

Dip2b	-0.087181074	0.875723186
Dip2c	-0.090073821	0.865547585
Diras1	0.135661536	0.6883862
Diras2	-0.054351402	0.91462326
Dirc2	0.060390198	0.873706405
Dis3	-0.001753934	0.998010523
Dis3l	-0.083892943	0.89703631
Dis3l2	-0.05177751	0.958389182
Disc1	-0.04333483	0.991438757
Disp1	-0.191985838	0.893784925
Disp2	-0.074750902	0.782546806
Disp3	-0.06960138	0.936031628
Dixdc1	-0.107960162	0.619769322
Dkc1	0.031194601	0.980226283
Dkk2	-0.022707687	0.996920836
Dkk3	-0.111272655	0.688142448
Dkkl1	-0.156309196	0.968005385
Dlat	0.060759788	0.890704638
Dlc1	-0.352069144	0.871869598
Dld	0.031867373	0.965520236
Dlec1	-0.292242592	0.891972665
Dleu2	-0.00831128	0.997241386
Dleu7	0.052058779	0.985293518
Dlg1	0.125497436	0.891972665
Grem1	-0.516267034	0.414554646
Dlg3	-0.08414232	0.734581634
Dlg4	-0.016431653	0.975831674
Spen	-0.515343188	0.049354177
Dlgap1	-0.067366327	0.899097089
Dlgap2	-0.127006402	0.828102037
Dlgap3	-0.077242892	0.889908105
Dlgap4	0.129421184	0.89703631
AC154232.2	1.525515496	0.905559486
Dlk1	-0.063847414	0.994960308
Dlk2	0.055265988	0.965520236
Dll1	-0.331766618	0.742422553
Dll3	0.483453768	0.8549794
Dll4	0.467956017	0.852010446
Dlst	-0.234500652	0.544661714
Cep250	-0.514606789	0.084448813
Dlx1as	-0.12073364	0.965520236
Dlx2	-0.296455167	0.76758211
Dlx3	0.740854943	0.976546786
Duox1	1.520319098	0.889908105

Dlx4os	-0.060163471	0.997115148
Dlx5	0.002653063	0.998010523
Dlx6	-0.121184858	0.962648047
Dlx6os1	-0.180364958	0.806077586
Nos3	-0.513285531	0.067353494
Dmac1	0.015896327	0.991438757
Fos	-0.512934994	0.238771637
Dmap1	0.047244078	0.965520236
Dmbx1	-0.726086797	0.886647768
Dmc1	0.176864251	0.975831674
Dmd	-0.256765811	0.568915531
Dmgdh	-0.240422536	0.975831674
Dmkn	-0.512459646	0.938579664
Dmp1	0.153358831	0.891972665
Dmpk	0.09787668	0.951817826
Dmrt2	0.13052177	0.97607383
Dmrt3	0.533234296	0.857889912
Dmrta1	-0.706167152	0.959276014
Dmrta2	-0.009671092	0.997241386
Dmrta2os	0.367567786	0.985673938
Dmrtb1	0.599357796	0.965520236
Dmrtc1a	-0.003134695	0.997884966
Dmtf1	-0.099268071	0.789386933
Dmtn	-0.050170896	0.897862979
Dmwd	-0.017628246	0.980226283
Dmxl1	-0.194029101	0.611941335
Rmst	0.512256028	0.480840274
Dna2	0.412447447	0.795419877
Dnaaf1	0.264292872	0.966010627
Dnaaf2	0.137169361	0.869133292
Dnaaf3	-0.027693572	0.99527876
Dnaaf5	-0.040065897	0.985742667
Dnah1	-0.343084971	0.549579107
Dnah10	-0.263875679	0.962648047
Dnah11	-0.351272231	0.948104382
Rptoros	1.506270732	0.860142574
Dnah14	-0.130835835	0.976978183
Dnah17	-0.320406748	0.868415875
Dnah2	0.175695577	0.909728442
Dnah2os	0.858424699	0.899271839
Dnah3	-0.736241392	0.874252042
Dnah5	-0.186597243	0.898330978
Dnah6	-0.223805738	0.963666931
Dnah7a	-0.042476501	0.991438757

Zbtb16	0.511576289	2.02566E-06
Dnah7c	-0.245785403	0.903496663
Dnah8	-0.71762487	0.757217583
Flrt1	-0.511252967	0.264217957
Dnaic1	0.432594843	0.623223056
Dnaic2	-0.622505852	0.52053053
Dnaja1	0.022434429	0.971661054
Dnaja2	0.032075547	0.951189675
Dnaja3	-0.087580234	0.92517502
Dnaja4	-0.148752065	0.5542082
Dnajb1	-0.036337507	0.973100313
Dnajb11	-0.099215986	0.891972665
Dusp6	-0.509606674	0.000134553
Dnajb13	0.053559118	0.993103295
Dnajb14	-0.124347875	0.704858662
Dnajb2	-0.10097002	0.898812578
Dnajb3	-0.402007707	0.882824434
Dnajb4	0.091802968	0.91462326
Dnajb5	-0.085521416	0.89703631
Dnajb6	0.069841693	0.8549794
Dnajb7	0.642536394	0.974962198
Dnajb9	-0.078508461	0.89703631
Dnajc1	-0.018463264	0.985033402
Dnajc10	-0.039925216	0.957773729
Dnajc11	0.071485641	0.897878088
Dnajc12	-0.020836689	0.991996237
Dnajc13	-0.168491505	0.535163601
Kif26b	-0.509287644	0.455841215
Dnajc15	-0.066751385	0.894953269
Dnajc16	0.006955493	0.993277997
Dnajc17	0.122765196	0.873797321
Dnajc18	-0.063614184	0.87806697
Dnajc19	0.022924145	0.985673938
Dnajc19-ps	0.27681071	0.973100313
Dnajc2	0.024387704	0.980419928
Dnajc21	-0.106362372	0.911631946
Dnajc22	0.688906201	0.984232465
Dnajc24	-0.078069825	0.972218452
Dnajc25	0.145571574	0.954610535
Dnajc27	0.035042197	0.962648047
Dnajc28	-0.070136351	0.962648047
Dnajc3	-0.107055104	0.699575734
Dnajc30	-0.052781585	0.959276014
Cntnap5c	-0.509227663	0.115616253

Dnajc5	-0.014954651	0.980144634
Dnajc6	-0.059067594	0.828907376
Dnajc7	0.056324103	0.873753967
Dnajc8	0.085600708	0.813667053
Dnajc9	0.097731672	0.890704638
Dnal1	-0.127379435	0.992385525
Dnal4	-0.02107418	0.988090086
Dnali1	-0.355298257	0.921107945
Atad2	-0.508954848	0.056924302
Dnase1l1	-0.214508462	0.930110675
Dnase1l2	-0.497876048	0.604176395
Dnase1l3	-0.123254422	0.99527876
Acap1	1.502592379	0.607527044
Dnd1	-0.014423113	0.995944208
Dner	-0.13827106	0.548869952
Dnhd1	-0.1685078	0.945526153
Dnlz	-0.082568456	0.900330211
Dnm1	0.013562496	0.98340069
Dnm1l	-0.123263302	0.706984274
Dnm2	0.063905312	0.91462326
Dnm3	-0.064334455	0.898478018
Dnm3os	-0.694938471	0.974823941
Dnmbp	-0.197715837	0.874422885
Dnmt1	0.020192253	0.985742667
Dnmt3a	0.298179178	0.975800973
Dnmt3b	0.345817814	0.936031628
Dnmt3l	0.181888742	0.982908579
Dnpep	0.129178139	0.848380246
Dnph1	0.081303838	0.975831674
Lncenc1	1.489129166	0.571020232
Dnttip1	0.14382337	0.713014362
Dnttip2	-0.054941452	0.93912725
Doc2a	0.236756417	0.755384283
Doc2b	-0.147775177	0.813499809
Doc2g	0.135527908	0.962648047
Dock1	-0.041617488	0.965520236
Dock10	0.20084197	0.727420355
Dock11	0.093706111	0.965520236
Dock2	0.11932145	0.938615214
Dock3	-0.006633739	0.994960308
Cdc42ep1	-0.508702564	0.146214787
Dock5	-0.14592231	0.91462326
Dock6	-0.18081225	0.625886453
Dock7	-0.093450206	0.91462326

Dock8	-0.291098264	0.745879732
Dock9	-0.102789798	0.833916587
Dohh	0.036495318	0.965290366
Dok1	0.169865463	0.939904081
Dok2	0.480360249	0.935897306
Dok3	-0.242242291	0.849424799
Dok4	-0.027451056	0.976978183
Dok5	0.27576721	0.770166577
Dok6	-0.807065756	0.609927197
Dok7	-0.147164619	0.959276014
Dolk	-0.048852347	0.968005385
Dolpp1	0.057571455	0.960201673
Rps3a3	0.507569751	0.070905587
Dop1a	-0.041799831	0.962648047
Dop1b	-0.089097709	0.823072541
Pou3f4	-0.504095538	0.261889255
Dpagt1	0.059792888	0.96905593
Dpep1	-0.019862726	0.998010523
Dpep2	-0.875516159	NA
Gsta2	1.488530515	0.955993235
Dpf1	0.075732836	0.920642026
Dpf2	-0.013955163	0.985742667
Dpf3	-0.019163586	0.993103295
Dph1	0.01299971	0.99527876
Dph2	-0.197724604	0.770166577
Dph3	-0.094422655	0.915297582
Dph5	0.04883823	0.981045362
Dph6	-0.320102469	0.731137993
Dph7	0.049598929	0.963500155
Dpm1	0.144948522	0.910328122
Dpm2	0.160515915	0.596787845
Dpm3	0.11137186	0.950195611
Dpp10	-0.11142498	0.835714527
Dpp3	-0.092327991	0.896196194
Dpp4	-0.00677219	0.998010523
Dpp6	-0.022558768	0.968005385
Dpp7	0.033756555	0.982408617
Strip2	-0.503940248	0.251423178
Dpp9	-0.099646604	0.707777756
Dppa1	0.201115557	0.991438757
Zp3r	1.486042464	0.841017002
Cd200r2	1.484753309	0.948104382
Dpy19l1	-0.064005524	0.963943597
Dpy19l2	0.163332915	0.985742667

Alox5ap	0.502381139	0.333416246
Dpy19l4	-0.114225433	0.759486652
Tnip2	0.502048405	0.123600847
Dpyd	0.1347559	0.887525326
Dpys	0.22785725	0.991438757
Rpl17-ps9	0.500377887	0.088465115
Dpysl3	-0.049428412	0.962648047
Dpysl4	0.13648247	0.759365171
Dpysl5	0.038777549	0.984328758
DQ267100	0.08251524	NA
DQ267101	0.040719528	NA
DQ267102	0.263166161	0.980339705
Dqx1	-0.909779583	0.909547396
Phospho2	-0.499752149	0.477528928
Dram1	-0.484180579	0.707520358
Dram2	0.077624437	0.91462326
Drap1	0.061594769	0.945647427
Draxin	-0.124563141	0.984328758
Drc1	-0.0933776	0.911631946
Drc3	-0.051294938	0.983977081
Drc7	0.328675267	0.903795193
Drd1	0.143177249	0.965520236
Drd2	-0.110338323	0.969476616
Olfr558	1.481473673	0.819775497
Drd5	0.106748289	0.912042382
Drg1	0.091853604	0.709960234
Drg2	0.028137494	0.968630572
Drosha	0.025888933	0.971138085
Drp2	-0.743225178	0.66964967
Dsc1	0.49320474	0.985742667
Tsga10ip	1.476335659	0.89703631
Dsc3	0.324272522	0.964973812
Dscam	-0.030456774	0.97487264
Dscaml1	-0.042037243	0.942812446
Dse	0.082764715	0.962648047
Dsel	-0.068732026	0.98302504
Dsg1c	0.072605964	0.99527876
Dsg2	-0.26969022	0.882824434
Dsn1	0.077328795	0.974809035
Fcgbp	0.499351776	0.444359209
Dspp	-0.02475907	0.998010523
Dst	-0.136541092	0.629280869
Dstn	0.020333682	0.975831674
Kcnh5	0.499040388	0.438549066

Dtd1	0.175760856	0.688142448
Dtd2	0.067578359	0.968005385
Morn3	1.473289094	0.837755777
Dtl	0.263536289	0.950520575
Dtna	-0.111039654	0.874252042
Dtnb	0.045453521	0.956103364
Dtnbos	0.228167702	0.968005385
Dtnbp1	0.040511173	0.97469529
Dtwd1	0.112669203	0.919707895
Dtwd2	0.300141787	0.779231312
Dtx1	0.20029519	0.841017002
Dtx2	-0.086659213	0.953505913
Dtx3	0.040229005	0.962648047
Dtx3l	-0.24071354	0.758046356
Bcl9l	-0.499031989	0.062680012
Dtymk	0.021459322	0.98526072
Zic5	-0.49896099	0.41351939
AC120150.1	1.472892609	0.95070766
Prss43	1.472192001	0.513882458
Cndp1	1.472087813	0.510569404
Dus1l	-0.116052734	0.859910936
Dus2	0.153695658	0.836557013
Dus3l	0.083102367	0.852261195
Dus4l	0.016403244	0.994630727
Dusp1	-0.069569733	0.964011524
Dusp10	0.064823878	0.975831674
Dusp11	-0.099497012	0.695239733
Dusp12	-0.023562646	0.991996237
Dusp13	0.12582763	0.984769634
Dusp14	0.117000529	0.868797028
Dusp15	-0.017027833	0.994960308
Dusp16	-0.177311812	0.862285215
Dusp18	-0.022481663	0.980731359
Rara	-0.498797467	0.144634493
Dusp2	0.327400107	0.962648047
Dusp22	-0.017788377	0.985673938
Dusp23	0.368033043	0.550601446
Dusp26	-0.010382338	0.99527876
Dusp27	0.111881047	0.985742667
Dusp28	0.176139359	0.585734325
Dusp3	0.091793598	0.850260513
Dusp4	-0.116272251	0.896069965
Dusp5	-0.211101609	0.801926713
Efna1	0.498631445	0.404443644

Dusp7	-0.104391291	0.786324313
Dusp8	-0.056401106	0.89703631
Dusp9	-0.185942065	0.974823941
Osbp2	0.498522106	0.193510645
Duxbl1	0.803264962	0.893784925
Dvl1	-0.023918716	0.973100313
Dvl2	-0.06062069	0.959276014
Dvl3	0.001864224	0.99765039
DXBay18	0.52572981	0.740833211
Dxo	0.167022126	0.865884071
Dydc2	-0.101366251	0.991996237
Dym	0.061481254	0.942812446
Dync1h1	-0.115918519	0.5141747
Dync1i1	-0.002409008	0.997115148
Dync1i2	0.111266287	0.610726714
Dync1li1	-0.008594407	0.991438757
Dync1li2	0.027419059	0.962648047
Dync2h1	-0.073580599	0.91462326
Dync2li1	0.088609032	0.90458554
Dynll1	-0.008709284	0.993489613
Cgn	-0.496727488	0.337479705
Naa30	-0.495558098	0.31865776
Dynlrb2	-0.389196248	0.911631946
Dynlt1-ps1	0.212137539	0.938394547
Dynlt1a	0.152909593	0.891972665
Dynlt1b	0.170957247	0.941398449
Dynlt1c	0.17630758	0.890704638
Dynlt1f	0.01055235	0.99527876
Dynlt3	-0.022324493	0.970892908
Dyrk1a	-0.316158818	0.956164302
Dyrk1b	0.061731631	0.962648047
Nav2	-0.49505842	0.318279733
Dyrk3	-0.137114508	0.852275035
AC124484.1	1.470766526	0.910328122
Dysf	-0.057781157	0.98459388
Rpl7a-ps10	1.470525732	0.750453415
Obscn	0.494872637	0.465695122
Dzank1	0.023021171	0.971138085
Slc8b1	-0.492545365	0.329158589
Dzip1l	0.077850273	0.904480183
Gpr3	-0.491358417	0.389090965
E2f1	0.174656525	0.879277236
E2f2	0.143996563	0.974962198
E2f3	-0.087744688	0.855344864

E2f4	-0.037043696	0.965520236
E2f5	-0.085144982	0.965520236
E2f6	-0.031452786	0.983479691
E2f7	-0.114353455	0.991996237
E2f8	0.535185798	0.965520236
E4f1	0.075735519	0.910328122
Eaf1	-0.060696458	0.962648047
Eaf2	0.391202427	0.857344087
Eapp	0.164396475	0.852946549
Pcdhgb1	1.464605368	0.596787845
Ears2	0.100397402	0.962648047
Ebag9	0.063038361	0.950460219
Pygm	0.491131987	0.185359794
Kansl2	0.490614657	3.03564E-07
Ebf3	0.192159519	0.962648047
Ebf4	-0.202990021	0.615496563
Ebi3	0.321507348	0.936489142
Ebna1bp2	-0.060249674	0.912042382
Ebp	0.160204792	0.852035098
Ebpl	-0.057959144	0.962812066
Ecd	0.070833258	0.911631946
Ece1	0.001877556	0.99765039
Ece2	0.099079361	0.833916587
Ecel1	-0.439011356	0.852261195
Ech1	-0.032906247	0.965520236
Echdc1	-0.127689541	0.894148925
Echdc2	0.160208165	0.813552442
Echdc3	0.017177738	0.99765039
Echs1	0.072238904	0.89703631
Eci1	-0.027595556	0.98526072
Eci2	0.100645368	0.888526258
Ecm1	0.029620481	0.993489613
Ecm2	0.033734597	0.985742667
Ecpas	-0.059180945	0.89703631
Ecscr	0.090030143	0.968630572
Ecsit	0.046930254	0.956922504
Ect2	0.012801826	0.99765039
Ect2l	-0.680654413	0.878577494
Eda	-0.07477527	0.962648047
Eda2r	-0.664066769	0.814301191
Edar	-0.97856194	0.968005385
Edaradd	0.635835135	0.749626226
Edc3	0.002421103	0.997258205
Edc4	0.032669398	0.974823941

Edem1	0.0070534	0.99527876
Edem2	0.082611874	0.923340746
Fgfrl1	-0.490258994	0.131091021
Edf1	0.086973359	0.795137348
Edil3	-0.015675083	0.985673938
Edn1	0.267850497	0.8989475
Edn3	0.079060487	0.971661054
Ednra	0.185209472	0.882096017
Ednrb	-0.260755454	0.962648047
Edrf1	-0.186345225	0.624713015
Eea1	-0.047280995	0.962648047
Eed	-0.071880532	0.957485565
Hebp2	0.489917765	0.422629014
Eef1a2	0.0762939	0.783229859
Eef1akmt1	0.068025089	0.950195611
Eef1akmt2	-0.050648034	0.968005385
Eef1akmt3	0.453230167	0.974823941
Eef1akmt4	-0.226918466	0.938939871
Eef1b2	0.03140485	0.971661054
Eef1d	-0.001062265	0.998010523
Eef1e1	-0.035774878	0.974197921
Eef1g	0.047232793	0.92306576
Eef2	0.047305565	0.868415875
Eef2k	0.0104352	0.989200732
Eef2kmt	-0.179817231	0.936031628
Eefsec	0.020704012	0.985742667
Eepd1	-0.035726704	0.984232465
Efcab1	-0.124386174	0.899141332
Efcab10	0.209943979	0.928672524
Efcab11	0.496138026	0.918235054
Efcab12	-0.125848204	0.948104382
Efcab14	-0.067800059	0.909243078
Efcab2	-0.194023754	0.6883862
Efcab5	-0.288992803	0.874252042
Efcab6	-0.243345187	0.703372057
Efcab7	-0.083417663	0.975831674
Efcab8	0.096567112	0.99527876
Efcab9	-0.041275979	0.99527876
Efcc1	-0.015887153	0.99527876
Efemp1	-0.136348695	0.962648047
Efemp2	-0.22121159	0.80931022
Efhb	0.468218452	0.950770028
Efhc1	0.013044165	0.995944208
Efhc2	0.146858002	0.962648047

Efhd1	0.028252535	0.98345216
Efhd2	0.026792473	0.974823941
Efl1	-0.039232162	0.970461999
Ddc	0.489307548	0.03797934
Efna2	-0.346319718	0.638329646
Efna3	-0.070215373	0.945647427
Efna4	0.240453772	0.965520236
Efna5	-0.076504595	0.980226283
Efnb1	-0.094327864	0.956164302
Efnb2	-0.275900195	0.56399886
Ccnb2-ps	0.488162316	0.442302609
Efr3a	-0.054617258	0.910328122
Efr3b	0.017221521	0.984232465
Efs	-0.241051932	0.821998633
Eftud2	0.049679802	0.936031628
Egf	-0.265545886	0.884629276
Egfem1	0.138232751	0.875056988
Egfl6	-0.007629565	0.995886687
Egfl7	0.175360783	0.771211584
Egfl8	-0.128188868	0.975800973
Egflam	-0.166825782	0.962648047
Egfr	-0.020475752	0.985742667
Egln1	0.048428508	0.932673884
Egln2	0.027870226	0.973100313
Egln3	-0.388438902	0.630888733
Med18	0.487975624	0.442302609
Cytl1	1.464000485	0.836720624
Egr3	-0.051874795	0.965520236
Trpc5	-0.487861216	0.084448813
Ehbp1	0.016744396	0.985673938
Ehbp1l1	-0.055205107	0.962648047
Ehd1	0.170589719	0.522639613
Ehd2	-0.24648921	0.659663162
Rsl24d1	0.487561225	0.07437226
Ehd4	-0.043722591	0.981392364
Ehhadh	0.302495458	0.710375942
Ehmt1	-0.263750692	0.544361994
Ehmt2	0.092596929	0.676362507
Ei24	-0.109849704	0.873797321
Eid1	0.071954603	0.867827234
Eid2	0.071096967	0.921311891
Eid2b	0.086648608	0.881771584
Setd6	0.486482262	0.438549066
Zbtb20	-0.484137692	0.055853534

Eif1-ps1	0.023571577	0.997241386
Eif1-ps2	-0.756887493	0.980626239
Eif1a	0.049778357	0.959324877
Eif1ad	0.107774686	0.815987743
Eif1ax	-0.022464582	0.968005385
Eif1b	0.079868749	0.869437456
Eif2a	0.085178102	0.858549868
Eif2ak1	0.03632964	0.962648047
Eif2ak2	-0.09713415	0.948104382
Eif2ak3	-0.075334492	0.917018212
Cdc42ep2	-0.479955379	0.146214787
Eif2b1	0.049071163	0.948104382
Eif2b2	0.106486458	0.84870301
Eif2b3	0.082307272	0.911631946
Eif2b4	0.032746715	0.975831674
Eif2b5	0.078292879	0.807017299
Eif2d	-0.023588709	0.98526072
Eif2s1	-0.037048284	0.962648047
Eif2s2	-0.038591244	0.962648047
Eif2s3x	0.014374496	0.98175286
Eif2s3y	0.027896832	0.987326705
Eif3a	-0.044089907	0.947602075
Eif3b	0.078652916	0.786324313
Eif3c	0.044327473	0.89703631
Eif3d	0.076626718	0.858549868
Eif3e	0.031294723	0.966213117
Eif3f	0.090874272	0.80931022
Eif3g	0.10211064	0.74632237
Spata6	-0.479765409	0.434635199
Eif3i	0.07312442	0.881371608
Eif3j1	-0.109663443	0.813499809
Eif3k	0.04244387	0.959276014
Eif3l	0.062802013	0.91462326
Eif3m	0.042633208	0.954827342
AC125371.1	1.455374369	0.950195611
Smim3	0.479411984	0.065899401
Eif4a1	0.188346843	0.668010503
Eif4a2	0.027763299	0.965290366
Eif4a3	0.154971054	0.758595939
Eif4b	0.030328075	0.955993235
Eif4e	-0.099047664	0.815987743
Eif4e2	0.050679935	0.935510251
Eif4e3	-0.133577406	0.779231312
Eif4ebp1	0.241676527	0.936834221

Eif4ebp2	-0.134078465	0.666177474
Eif4ebp3	0.19242803	0.975800973
Eif4enif1	-0.069034141	0.911631946
Eif4g1	0.023865656	0.969755848
Eif4g2	-0.041447525	0.939393695
Eif4g3	-0.07475242	0.783229859
Eif4h	0.018024322	0.975800973
Eif5	0.127792959	0.975831674
Eif5a	0.089390874	0.749519672
Eif5a2	-0.128307805	0.770793864
Eif5al3-ps	0.632688873	0.947447204
Eif5b	0.001267005	0.998010523
Eif6	0.133340603	0.720661866
Eipr1	-0.067029686	0.898812578
Elac1	0.025897457	0.980226283
Elac2	0.104617818	0.889908105
Elavl1	-0.058017019	0.935897306
Elavl2	0.326966493	0.957849305
Elavl3	-0.051754533	0.89703631
Elavl4	-0.138310035	0.821998633
Cst7	1.450559885	0.864150749
Elf1	-0.384302152	0.610479949
Elf2	0.064602364	0.911834483
Elf3	0.352504251	0.991438757
Elf4	-0.515637125	0.69189201
Elf5	0.07149174	0.99527876
Elfn1	0.008878352	0.99527876
Elfn2	-0.097259477	0.779231312
Elk1	0.088503477	0.904480183
Elk3	-0.07449823	0.965290366
Elk4	-0.07392077	0.910328122
Ell	0.171192352	0.573810203
Ell2	-0.034739737	0.975800973
Ell3	0.023568064	0.993380259
Elmo1	0.042617382	0.965520236
Elmo2	0.011344137	0.985742667
Elmo3	0.211345511	0.84277074
Elmod1	0.115545182	0.774804043
Elmod2	-0.045895932	0.968005385
Elmod3	0.139907911	0.729712198
Elmsan1	0.074805213	0.922887207
Eln	0.215012253	0.834224299
Eloa	-0.038377552	0.962648047
Srbd1	-0.477092227	0.065899401

Eloc	0.009383504	0.991996237
Elof1	-0.013783806	0.991438757
Elovl1	-0.274143108	0.780228245
Nadk	-0.476945003	0.285037596
Elovl4	-0.049227387	0.950460219
Elovl5	-0.011519382	0.990915306
Lin28b	-0.476471202	0.442068044
Elovl7	-0.022670183	0.994960308
Elp1	-0.108185078	0.891972665
Elp2	0.035313748	0.945182261
Elp3	0.075892821	0.98526072
Pdk4	0.476367593	0.11393407
Elp5	0.160997364	0.874971335
Elp6	-0.030712699	0.985673938
Emb	-0.024067907	0.984232465
Emc1	-0.050509454	0.921154826
Thrsp	0.471817979	0.007601713
Emc2	-0.043841833	0.962648047
Emc3	0.057650068	0.911631946
Emc4	0.05050511	0.936031628
Emc6	0.029402981	0.974823941
Emc7	-0.051902785	0.89703631
Emc8	0.066431936	0.891972665
Emc9	0.156145283	0.800711882
Emcn	0.122646709	0.951901417
Emd	0.035248531	0.97487264
Mir339	1.448657868	0.938414123
Eme2	0.09526754	0.837755777
Emg1	-0.00866284	0.99527876
Emid1	0.013964592	0.99527876
Emilin1	0.019202924	0.99640101
Cd46	0.471729905	0.226389202
AC154457.3	1.447272642	0.707777756
Eml1	-0.234342295	0.776275984
Eml2	0.094075835	0.660403254
Naa60	0.47149433	0.327922951
Spsb4	-0.471362452	0.442302609
Eml5	-0.109818747	0.84870301
Eml6	-0.147403445	0.86783605
Emp1	-0.355357768	0.857344087
Emp2	-0.02034731	0.985293518
Emp3	-0.92824976	0.522639613
Emsy	-0.119541134	0.779964055
Emx1	0.070090875	0.962648047

Emx2	0.078008241	0.946985353
Emx2os	-0.067686305	0.964605855
En2	0.016044647	0.99765039
Enah	0.0117738	0.985673938
Enc1	0.003066077	0.99527876
Endod1	0.054620508	0.947745582
Endog	0.211648768	0.857614057
Endou	0.464814787	0.814814675
Endov	0.283861048	0.953505913
Eng	-0.219139131	0.60123302
Engase	0.231012595	0.74632237
Enho	-0.212780337	0.550601446
Enkd1	-0.089869027	0.962648047
Enkur	-0.017424742	0.99527876
Eno1	0.040652279	0.937924083
Eno1b	0.071601367	0.858549868
Eno2	-0.057178685	0.895520088
Snord50a	1.446553232	0.873797321
Eno4	-0.023978016	0.99527876
Enoph1	-0.023649108	0.98459388
Enox1	0.038881567	0.962648047
Enox2	0.099458599	0.874971335
Enpep	0.096738803	0.975831674
Enpp1	-0.380530449	0.774896252
Enpp2	-0.011602676	0.99527876
Enpp3	-0.257973027	0.980132658
Enpp4	-0.118045149	0.831837418
Enpp5	0.048748007	0.929503395
Enpp6	-0.162367498	0.897469371
Ensa	0.10662054	0.752665311
Entpd1	0.011347965	0.99527876
Entpd2	0.154403903	0.903596982
Entpd3	0.203038604	0.732587258
Entpd4	-0.099158824	0.84870301
Entpd4b	-0.040169893	0.962648047
Egr1	-0.471343428	0.012905834
Entpd6	0.031804389	0.965520236
Entpd7	-0.009214255	0.99527876
Entr1	0.022773002	0.978063275
Eny2	0.017316334	0.983977391
Eogt	0.136624738	0.707069842
Eomes	0.787954171	0.822597041
Ep300	-0.153580025	0.572708219
Ep400	-0.152485439	0.521202205

Epas1	0.015135567	0.981641687
Epb41	0.145276403	0.849526037
Epb41l1	0.08978188	0.981045362
Epor	0.470597516	0.32696666
Arc	-0.469256384	0.161734551
Epb41l4a	0.254325107	0.729405774
Epb41l4aos	0.016726054	0.99527876
Epb41l4b	-0.013208892	0.98771048
Epb41l5	-0.152132427	0.936031628
Epc1	0.027042979	0.974823941
Epc2	-0.069546847	0.894953269
Epcam	0.065876756	0.99527876
Epdr1	0.028435343	0.980339705
Zfp688	0.469108067	0.426109731
Epgn	-0.489288331	0.980226283
Epha1	0.825583561	0.872265039
Epha10	-0.021524845	0.984328758
Epha2	-0.11582233	0.97670849
Epha3	-0.0877275	0.952034929
Epha4	-0.137886981	0.572363201
Epha5	-0.236535205	0.5141747
Rpl17-ps8	0.468137033	0.205552132
Epha7	-0.065337351	0.956164302
Epha8	-0.059958636	0.985742667
Ephb1	-0.042581519	0.974823941
Ephb2	-0.044994754	0.97487264
Ddit4	0.468129444	0.034210436
Ephb4	-0.492873197	0.573810203
Ephb6	-0.092041836	0.859472511
Ephx1	-0.072125267	0.932673884
Ephx2	-0.11759648	0.832639343
Socs1	1.440614545	0.80931022
Ephx4	-0.039889378	0.97487264
Epm2a	0.101580698	0.860826773
Rps19-ps6	0.466454566	0.24802413
Epn1	-0.044967115	0.939592821
Epn2	-0.159466009	0.858549868
Epn3	-0.067296363	0.985673938
Mir1843b	1.416809713	NA
Epop	0.100074756	0.873797321
Ap5b1	0.466334057	0.480408134
Qrich2	1.410436002	0.932673884
Eprs	-0.058104194	0.920642026
Gpr63	-0.466333792	0.365076065

Eps15l1	-0.057973561	0.911631946
Eps8	-0.094172021	0.837755777
Eps8l1	0.750512904	0.868625126
Eps8l2	0.094542718	0.971661054
Eps8l3	0.861928039	0.936031628
Epsti1	0.761736044	0.69189201
Platr6	1.409764104	0.862312446
Epyc	-0.205761102	0.965520236
Eqtn	0.460275278	0.911631946
Eral1	-0.067160957	0.962648047
Erap1	-0.164821856	0.862312446
Erbb2	-0.405141495	0.745688677
Kmt5b	-0.463799858	0.233991947
Pitpnm2os2	1.406238012	0.74463793
Erbin	-0.156700156	0.853090538
Erc1	-0.151369357	0.871869598
Erc2	0.02334046	0.965520236
Ercc1	0.180874485	0.544661714
Aoc2	-0.463458828	0.492284803
Ercc3	0.096792357	0.801673014
Ercc4	-0.028987111	0.975831674
Ercc5	-0.053064672	0.968005385
Ercc6	-0.139906529	0.742849809
Ercc6l	0.588014406	0.912042382
Ercc6l2	0.037683001	0.980419928
Ercc8	0.09764983	0.927471971
Erf	0.021826644	0.980226283
Erg	-0.208618837	0.948104382
Erg28	-0.07432397	0.912397394
Ergic1	0.009378391	0.988034453
Ergic2	0.128718834	0.915417975
Ergic3	0.034761506	0.968005385
Erh	0.047571031	0.959276014
Eri1	-0.079478911	0.940439828
Eri2	0.227374591	0.891972665
Eri3	-0.022713842	0.97487264
Erich1	-0.143863268	0.935897306
Erich2	-0.065961582	0.993103295
Erich3	-0.16356433	0.810946393
Erich5	-0.121485662	0.982309415
Erich6	-0.223117226	0.89703631
Erlec1	0.119065883	0.738901675
Erlin1	-0.041807062	0.971138085
Erlin2	0.064460502	0.899249451

Ermap	-0.365613506	0.911631946
Ermard	0.177811366	0.6883862
Ermn	-0.212638608	0.573810203
Ermp1	0.105132853	0.688142448
Ern1	-0.201000257	0.640856315
AY074887	1.402496684	0.895520088
Slc25a35	-0.46213477	0.347698938
Ero1lb	-0.100746784	0.826209871
Erp27	0.712109891	0.899097089
Zmym1	-0.461035456	0.000657163
Erp44	-0.115612001	0.800386557
Errfi1	0.010310953	0.993380259
Erv3	0.252490686	0.971661054
Esam	0.039732334	0.981502501
Esco1	-0.208026825	0.858106541
Esco2	0.983748835	0.965520236
Esd	-0.017085493	0.99100709
Esd-ps	0.8523615	0.804491173
Esf1	-0.017982078	0.991438757
Mov10l1	1.400294082	0.89703631
Espl1	0.089766958	0.981641687
Espn	-0.14338604	0.881371608
Mir7669	1.398608723	NA
Esr1	-0.111961963	0.965520236
Cxcr2	1.398513358	0.948432875
Irs3	1.398513358	0.948432875
Esrra	0.06396371	0.959659722
Esrrb	0.169920355	0.965290366
Esrrg	-0.070874769	0.968630572
Ess2	0.034774516	0.968859834
Esyt1	-0.057671997	0.971138085
Esyt2	-0.333593625	0.596787845
Esyt3	0.047439513	0.985673938
Etaa1	0.008391437	0.99527876
Etaa1os	-0.172386398	0.965011162
Etf1	0.02218701	0.974809035
Etfa	0.033855719	0.966213117
Etfb	0.149004437	0.624713015
Etfbkmt	-0.4736549	0.770793864
Etfdh	-0.051620796	0.948345831
Etfrf1	-0.109245435	0.962648047
Ethe1	-0.040706531	0.984748462
Ephb3	-0.460316077	0.020326008
Pik3ca	-0.459843174	0.19936941

Etnk2	-0.102512864	0.983076603
Etnppl	0.295194655	0.792517766
Etohd2	-0.251188583	0.629280869
Ets1	-0.246582517	0.737087313
Ets2	0.09467526	0.883333293
Etv1	-0.122546386	0.896196194
Etv3	-0.046702754	0.965520236
Etv4	0.237636017	0.65012378
Etv5	-0.101423129	0.860521845
Etv6	-0.072572423	0.953505913
mt-Tp	0.458691036	0.076369582
Eva1b	-0.632325742	0.719622087
Eva1c	0.300687883	0.889547181
Adcy7	-0.456261351	0.276340437
Evc2	-0.007602734	0.99527876
Evi2	-0.675381134	0.984836179
Ralgapa2	0.455077671	0.278616594
Evi2b	0.125724173	0.99527876
Hdac3	0.453141002	0.04370223
Evi5l	0.177673959	0.874971335
Evl	0.079796256	0.795137348
Evpl	0.43526835	0.831837418
Ewsr1	0.111862254	0.595417602
Exd1	0.15879282	0.962648047
Exd2	-0.111191433	0.586145911
Exo1	-0.078567847	0.993103295
Exo5	0.439882134	0.56399886
Dcn	-0.451952032	0.369497288
Exoc2	-0.065092058	0.889908105
Exoc3	-0.032005604	0.958375046
Exoc3l	-0.029413308	0.992385525
Exoc3l2	-0.041767829	0.99527876
Exoc3l4	-0.169583781	0.962648047
Exoc4	-0.098331999	0.794475043
Exoc5	-0.128493613	0.710375942
Exoc6	-0.062018996	0.910328122
Cbfa2t2	-0.451855714	0.226253856
Exoc7	0.06319921	0.912231446
Exoc8	-0.001669963	0.99765039
Exog	0.077060369	0.950460219
Exosc1	0.159417635	0.688916428
Exosc10	0.101204056	0.809849028
Nr3c1	-0.450781471	0.000356917
Exosc3	0.132898686	0.897878088

Exosc4	0.054649775	0.965241989
Exosc5	0.049225359	0.974823941
Exosc6	-0.447982811	0.89703631
Exosc7	0.046029969	0.968977192
Exosc8	0.183872262	0.664907827
Exosc9	0.045467486	0.962648047
Exph5	-0.038105463	0.991996237
Ice1	-0.450587637	0.003487984
Ext2	0.014693536	0.985742667
Extl1	-0.132908059	0.63237627
Extl2	0.109705087	0.713014362
Extl3	-0.048659216	0.911631946
Nup210	-0.448942582	0.266643009
Eya2	-0.526504458	0.90358675
Eya3	-0.127951198	0.956164302
Eya4	-0.340031003	0.831704864
Ezh1	0.0914399	0.819775497
Ezh2	0.121453945	0.950460219
Ezr	0.14146937	0.685353017
F11r	-0.03112982	0.987342614
Glp1r	1.398284152	0.6883862
F13a1	-0.595346619	0.932673884
F2r	-0.085697064	0.961887657
Rps13-ps5	0.448357967	0.495252404
F2rl2	0.332759999	0.970461999
F2rl3	-0.370804253	0.939393695
Htr1b	-0.448054543	0.122067616
F5	0.013762314	0.997241386
F7	0.556631402	0.97607383
F8	-0.114158882	0.975831674
F8a	0.086886253	0.961607556
F9	-0.163519605	0.985742667
Fa2h	-0.137585493	0.798734777
Faah	0.011240298	0.985742667
Faap100	-0.156230515	0.822597041
Faap20	0.002086686	0.997916818
Faap24	-0.015147188	0.99527876
Fabp3	0.053108296	0.923602336
Fabp3-ps1	0.275504698	0.663678416
Mir1224	1.397040982	NA
Tmem164	0.447275839	0.509250303
Fabp7	-0.027790219	0.985742667
Fadd	0.007412686	0.997258205
Dyx1c1	0.446095483	0.475240163

Fads2	0.040419071	0.956844001
Fads3	0.097061578	0.89703631
Fads6	-0.106058828	0.874624896
Faf1	0.057595864	0.939393695
Faf2	0.035583959	0.962648047
Fah	0.059524553	0.962838652
Fahd1	0.064613101	0.962648047
Fahd2a	0.084422145	0.951901417
Zfp945	-0.445821625	0.268043605
Faim2	0.55812459	0.736130784
Fam102a	-0.104939434	0.737087313
Fam102b	0.877156063	0.82814939
Fam104a	0.088284065	0.910328122
Mob3a	-0.44575433	0.20982796
Fam107b	0.046678675	0.976978183
Fam110a	-0.101829336	0.964941885
Nubp1	-0.445646859	0.423850642
Fam111a	-0.593194537	0.862285215
Fam114a1	-0.077075878	0.972025261
Synj2	-0.4440758	0.065698296
Fam117a	0.086085905	0.970461999
Fam117b	-0.026631505	0.978956158
Fam118a	-0.243073779	0.962648047
Fam118b	0.254992573	0.900386082
Klhl8	0.443436391	0.128640258
Fam120aos	0.108125094	0.928099251
Fam120b	-0.104341511	0.688142448
BC030500	-0.440521634	0.144051788
Fam122a	0.190282072	0.789386933
Fam122b	0.359137509	0.779415976
Fam124a	0.044170937	0.968005385
Fam126a	-0.108783073	0.866518414
Fam126b	-0.076383712	0.918235054
Fam129a	0.208631566	0.932931799
Fam129b	0.059375873	0.965241989
Fam129c	-0.167872979	0.962648047
Fam131a	-0.084972164	0.837755777
Tomm40l	0.440297488	0.161478968
Fam131c	0.100288423	0.975831674
Fam133b	0.321548193	0.568220567
Fam135a	-0.232765797	0.573101161
Fam135b	-0.059373988	0.939904268
Fam136a	0.047992407	0.962648047
Fam136b-ps	-0.051667236	0.997115148

Fam13a	0.002366223	0.99765039
Zfp68	0.440200279	0.422728382
Fam13c	-0.094430533	0.751142243
Fam149a	-0.005534724	0.99527876
Fam149b	-0.040948191	0.962648047
Fam151b	-0.00818124	0.995411429
Fam155a	-0.03952286	0.962648047
Fam160a1	0.329527424	0.610307639
Prox1os	0.439712209	0.502326577
Fam160b1	-0.074302867	0.875723186
Fam160b2	-0.038697793	0.968977192
Fam161a	0.097283165	0.97487264
Fam161b	-0.203776774	0.531183586
Dnah7b	-0.439004346	0.444079162
Fam163a	-0.212504714	0.58168712
Gatd1	0.437980112	0.436138179
Fam166a	0.172326012	0.99100709
Fam166b	-0.223626764	0.965290366
Fam167a	0.144908231	0.962648047
Asb15	1.395586098	0.869437456
Fam168a	-0.624852166	0.911631946
Fam168b	-0.015872572	0.975350978
Fam169a	-0.060935387	0.930250446
Fam169b	-0.005566007	0.998121731
Fam171a1	-0.035886289	0.964717692
Fam171a2	-0.065508604	0.947745582
Fam171b	-0.101182632	0.707777756
Fam172a	-0.049518972	0.955993235
Fam173a	0.038063616	0.962648047
Fam173b	0.324470736	0.759486652
Fam174a	-0.00635469	0.99527876
Fam174b	-0.124552499	0.695239733
Fam177a	0.133103344	0.882096017
Fam178b	-0.978745289	0.968005385
Gatad2b	1.392855121	0.574956844
Fam181a	-0.46596551	0.904275238
Fam181b	-0.187936904	0.816545416
Fam183b	0.153660758	0.924353966
Fam184a	-0.031551316	0.980339705
Fam184b	-0.184582321	0.837382879
Fam185a	-0.105121003	0.889908105
Olfr1348	1.391889752	NA
Fam186b	0.187727965	0.957499962
Mrps36-ps1	1.38878415	0.791185365

Alpk2	1.386291754	0.95126064
Ube2nl	1.381073417	0.837755777
Fam189a2	0.139993535	0.868797028
Fam189b	-0.08432983	0.964941885
Fam192a	0.090884658	0.940059526
Fam193a	-0.036070373	0.965520236
Fam193b	-0.152377169	0.83977902
Fam196a	-0.618340533	0.792913409
Fam196b	-0.395272417	0.858106541
Fam198a	-0.137408154	0.975831674
Fam198b	-0.146472836	0.949607377
Fam199x	-0.235242827	0.825910982
Fam19a1	-0.112300937	0.837755777
Fam19a2	0.122642633	0.869316117
Pzp	1.379670803	0.939393695
Fam19a4	0.423455479	0.936507368
Fam19a5	0.014092184	0.985742667
Fam204a	-0.094909978	0.916287981
Slc12a1	1.374595593	0.89703631
Fam205a3	0.080917533	0.997115148
Fam205a4	0.080560379	0.997115148
Fam205c	-0.094569475	0.970461999
Fam206a	-0.137811141	0.780434674
Fam207a	-0.123986136	0.874422885
Fam208a	-0.13201472	0.777427974
Zfp428	0.437714112	0.477881972
Fam20a	-0.045156557	0.98340069
Fam20b	-0.067360708	0.867740588
Fam20c	-0.125121512	0.7037205
Fam210a	-0.078813821	0.894264807
Fam210b	-0.077005975	0.926934696
Fam213a	0.076305254	0.842727003
Fam213b	0.186834095	0.612165149
Tep1	-0.43762197	0.45256507
Fam214b	0.006627437	0.99527876
Fam216a	0.11384027	0.665532517
Fam216b	-0.138135495	0.981392364
Fam217a	0.571844506	0.932673884
Fam217b	-0.006740704	0.994960308
Fam219a	-0.004671324	0.99527876
Fam219aos	0.764360929	0.970461999
Fam219b	0.010882131	0.991996237
Fam220-ps	0.603101016	0.894953269
Fam220a	0.025841962	0.975800973

Fam221a	-0.057606385	0.989840188
Fam221b	0.180563375	0.774693469
Fam222a	-0.226548526	0.928489442
Fam222b	-0.07547597	0.959276014
Atp8a1	-0.433220238	0.324926604
Fam228a	-0.053280012	0.984748462
Fam228b	-0.107349435	0.968005385
Mfsd4b5	1.373611057	0.948104382
Fam229b	0.196038961	0.89703631
Fam234a	-0.10255451	0.933201883
Fam234b	-0.09863153	0.676362507
Fam241a	-0.211345674	0.924988225
Tppp3	0.43185495	0.303490982
Fam24a	-0.1076817	0.991996237
Fam24b	0.373422837	0.969755848
Nr4a1	-0.430822245	0.096389386
Fam3a	0.040185872	0.97487264
Fam3c	0.065628597	0.947479954
Fam43a	0.232079519	0.874971335
Fam43b	-0.267568083	0.660403254
Fam45a	0.023431028	0.980109544
Gys2	1.373568912	0.911631946
Stac	-0.430276293	0.456330081
Fam49b	0.026681604	0.974962198
Fam50a	0.08148133	0.903441375
Fam53a	0.123224215	0.861378744
Fam53b	-0.199701233	0.889908105
Fam53c	-0.14858631	0.604176395
Fam57a	-0.028939099	0.98459388
Fam57b	0.032621809	0.965520236
Fam69a	0.007127668	0.99527876
Fam69b	-0.076552099	0.956141882
Fam69c	-0.035199339	0.985742667
Olfr1564	1.369803668	0.572124072
Fam71e1	0.123695171	0.942274311
Fam71f2	0.975079892	0.740833211
Fam72a	0.323303967	0.89703631
Fam76a	-0.013288382	0.985742667
Fam76b	-0.086239196	0.924361537
Fam78a	-0.348912537	0.801926713
Fam78b	-0.191183889	0.852010446
Fam81a	0.039055064	0.962648047
Tfap2c	1.367416318	0.925337971
Fam83c	-0.437681508	0.935897306

Fam83d	0.376444254	0.707726545
Fam83e	0.040788608	0.99765039
Kbtbd13	1.362564192	0.528975995
Fam83h	0.027545452	0.99527876
Fam84a	0.060908241	0.937726496
Fam84b	0.032282513	0.991377399
Fam89a	0.437300977	0.813552442
Fam89b	-0.041398063	0.985742667
Fam8a1	-0.021645426	0.973100313
Ip6k3	1.360844953	0.579325713
Cldn12	-0.429196601	0.422728382
Fam92a	0.038955628	0.965520236
Fam92b	-0.176043207	0.985742667
Fam98a	-0.039773809	0.954827342
Ptch1	-0.429086882	0.010025367
Fam98c	0.059431062	0.975800973
Fan1	0.171008381	0.8989475
Fanca	-0.495645621	0.778631013
Fancb	-0.227730215	0.911631946
Fancc	-0.03938805	0.981392364
Fancd2	-0.042724042	0.98043358
Fancd2os	-0.081542245	0.993242999
Fance	-0.104176652	0.961887657
Fancf	0.06377504	0.985742667
Fancg	0.089494963	0.91462326
Fanci	0.260564862	0.89703631
Zfp804a	-0.428178071	0.128640258
Fancm	-0.264363649	0.831215716
Fank1	0.039809895	0.987342614
Fap	-0.100435472	0.993103295
Far1	-0.427213683	0.640342243
Far1os	-0.252574234	0.988034453
Far2	-0.099922324	0.911631946
Far2os1	-0.585435578	0.968005385
Far2os2	-0.805839523	0.965520236
Farp1	-0.072572311	0.920790661
Farp2	-0.182472934	0.860521845
Fars2	0.019607074	0.987549838
Farsa	0.045933494	0.959276014
Farsb	-0.105217991	0.770793864
Gar1	0.427052066	0.065333411
Eya1	0.425572477	0.243398308
Fasn	-0.170184315	0.562965533
Fastk	0.086811298	0.809593157

Fastkd1	-0.055641946	0.962648047
Fastkd2	0.008135466	0.99527876
Fastkd3	0.169614622	0.852010446
Fastkd5	-0.127565009	0.884140163
Fat1	-0.073401758	0.963666931
Fat2	-0.347507238	0.985293518
Psmg1	0.425567354	0.160287523
Fat4	-0.141478098	0.911631946
Fate1	-0.96066577	0.971661054
Fau	0.095985447	0.870812051
Faxc	-0.153871589	0.77448401
Fbf1	0.142415692	0.532283878
Fbh1	0.045447687	0.955993235
Nsd2	0.424420143	0.508471705
Fblim1	0.019721609	0.995852544
Fbll1	0.105318092	0.881539811
Fbln1	0.003329674	0.998010523
Fbln2	0.067477886	0.97487264
Fbln5	0.098584128	0.975831674
Fbln7	-0.272394217	0.961373288
Fbn1	-0.032395674	0.984748462
Fbn2	-0.092570783	0.968005385
Fbp1	-0.293217253	0.985229869
Fbrs	-0.201844337	0.965520236
AC117239.2	0.424159737	0.426109731
Fbxl12	0.394320818	0.589856572
Fbxl12os	0.184569606	0.936031628
Fbxl13	0.046464588	0.99527876
Fbxl14	-0.038088072	0.97487264
Fbxl15	-0.188709333	0.776477378
Fbxl16	-0.080221749	0.788592804
Fbxl17	-0.275023162	0.624713015
Fbxl18	0.120986044	0.956922504
Zbed6	-0.424089823	0.144051788
Fbxl2	0.034298571	0.974809035
Fbxl20	-0.131164938	0.596982103
Fbxl21	-0.182134237	0.89383516
Fbxl22	-0.19986364	0.970640978
Ercc2	-0.423691703	0.144051788
Fbxl4	-0.131668211	0.864150749
Fbxl5	-0.085771888	0.875723186
Fbxl6	0.128695814	0.891972665
Fbxl7	0.122576182	0.96188696
Fbxl8	0.558572115	0.810908327

Fbxo10	0.075706611	0.855344864
Abhd4	-0.422755646	0.456330081
Ltb4r1	1.356641637	0.947033215
Fbxo16	-0.186901978	0.698018236
Fbxo17	-0.14227208	0.969755848
Fbxo2	0.137920419	0.621784586
Fbxo21	-0.02925643	0.981694523
Fbxo22	-0.031401068	0.974962198
Fbxo24	-0.987788593	0.868797028
Fbxo25	0.018463808	0.984748462
Fbxo27	-0.018848848	0.989840188
Fbxo28	-0.114750079	0.890704638
Fbxo3	0.037021483	0.953505913
Fbxo30	-0.094990791	0.955993235
Fbxo31	0.0444805	0.924448385
Fbxo32	-0.010317183	0.99527876
Fer1l6	1.352875978	0.951497773
Fbxo34	-0.173430817	0.841017002
Fbxo36	-0.339593138	0.733715112
Fbxo38	0.062940663	0.891352425
Fbxo4	-0.174184358	0.876118979
n-R5s200	1.350470577	NA
Fbxo41	-0.160258507	0.573810203
Fbxo42	-0.147141805	0.742849809
Atp8b5	1.34664433	0.665532517
Fbxo44	-0.167473528	0.8047234
Fbxo45	-0.022661806	0.97487264
Fbxo46	-0.196966906	0.6883862
Fbxo47	-0.33952	0.952034929
Fbxo48	-0.226675953	0.976978183
Fbxo5	0.315503159	0.936031628
Fbxo6	-0.056609952	0.962648047
Fbxo7	-0.032289227	0.975831674
Fbxo8	0.035600156	0.975900915
Fbxo9	0.045198137	0.97487264
Fbxw10	-0.367099124	0.868415875
Fbxw11	0.071542246	0.849381566
Fbxw15	-0.280912663	0.968005385
Fbxw17	0.162968989	0.873797321
Fbxw2	0.151370542	0.825910982
Fbxw23	-0.065785622	0.99527876
Fbxw4	0.188968447	0.655744783
Fbxw5	-0.131388151	0.574956844
Fbxw7	0.054795194	0.937550602

Fbxw8	-0.101925586	0.835960581
Fbxw9	-0.061524693	0.967662387
Fcer1g	-0.038481296	0.984675558
Fcer2a	0.228904944	0.962648047
Dapp1	-0.422494856	0.290428635
Tomm6	-0.422332418	0.221279819
Fcgr1	-0.028825034	0.994980068
Fcgr2b	0.094623276	0.973100313
Fcgr3	0.205952696	0.898478018
Fcgr4	0.690756175	0.975831674
Fcgrt	-0.016082408	0.99527876
Fcho1	0.070813688	0.899097089
Fcho2	-0.109410921	0.874971335
Fchsd1	-0.304451718	0.883921639
Fchsd2	-0.039738279	0.965520236
AC091309.4	1.346296996	0.910328122
Fcnaos	0.319228801	0.965520236
Fcor	0.068891576	0.976978183
Fcrl1	-0.143392125	0.98347579
Fcrlb	0.128350275	0.985742667
Fcrls	-0.085156692	0.922306337
Fdft1	0.038298031	0.99165967
Fdps	0.01399598	0.985742667
Fdx1	-0.123186238	0.897878088
Fdx1l	-0.488283977	0.615948828
Fdxacb1	0.066741455	0.982914099
Fdxr	0.024282027	0.987066554
Fech	-0.013893094	0.985673938
Fem1a	-0.020583397	0.978063275
Fem1b	-0.028655069	0.963666931
Fem1c	-0.16251591	0.606319546
Fen1	-0.125036644	0.985293518
Fendrr	-0.234707916	0.950460219
Fer	-0.175269976	0.585818114
Fer1l5	-0.087197837	0.947745582
AC164431.3	1.344076117	0.872352604
AC132353.1	1.343414096	0.91892799
Fermt1	0.030027814	0.993103295
Fermt2	-0.085422718	0.854341109
Fermt3	-0.091244375	0.975831674
Fes	0.164046392	0.942812446
Olfr78	1.343210252	0.853090538
Fez1	0.261657992	0.642397758
Fez2	0.003759514	0.996638606

Fezf2	-0.057003156	0.962648047
Fgb	0.199694612	0.993242999
Fgd1	-0.174617845	0.787744512
Fgd2	0.044214671	0.985293518
Fgd3	-0.036282834	0.981045362
Fgd4	-0.098635892	0.874137058
Pld1	0.422233329	0.220337275
Cirbp	0.421810154	0.085461442
Fgf1	-0.154359045	0.837755777
Fgf10	-0.200723394	0.673443553
Spr	-0.421687333	0.278515147
Fgf12	-0.036329195	0.968005385
Fgf13	0.082062726	0.795890296
Fgf14	-0.008916288	0.993872582
Fgf16	0.128359734	0.968005385
Vmn2r-ps24	1.341240092	0.921311891
Fgf18	0.321239314	0.911631946
Fgf2	-0.649044671	0.957485565
Fgf22	0.061970664	0.991996237
Fgf23	0.076737158	0.991438757
Fgf2os	-0.542418431	0.932673884
Fgf5	0.252200343	0.812431087
Fgf7	-0.088780338	0.988034453
Fgf9	-0.155246994	0.889908105
Fgfbp1	-0.613060274	0.945647427
Fgfbp3	-0.044566338	0.985742667
Trim36	-0.421417505	0.18626066
Fgfr1op	-0.388131293	0.942511266
Fgfr1op2	-0.069339451	0.905251009
Slc2a1	0.420089344	0.073934148
Kcns1	0.419424038	0.126698562
Gga3	0.419391907	0.340941022
Hist1h2bq	1.340048762	0.80931022
Fggy	-0.047011871	0.983479691
Fgl1	-0.88474961	0.961607556
Fgl2	-0.316796018	0.922887518
Fbxl3	-0.416070696	0.403453477
Nbdy	0.415934503	0.369820939
Fhad1	0.306070163	0.757721895
Myh13	1.332529661	0.858106541
Fhdc1	-0.005548446	0.99765039
Fhit	0.302222158	0.823690933
Fhl1	0.015015327	0.988208387
Fhl2	0.104077046	0.875723186

Fhl3	0.415221656	0.871869598
Fhl4	0.163055675	0.962493928
Fhod1	0.099542664	0.93912725
Fhod3	-0.146554743	0.89011487
St8sia4	-0.415909322	0.423074171
Fibin	0.012481298	0.997115148
Fibp	0.097153936	0.849424799
Ficd	0.021675558	0.985673938
Fig4	-0.020375851	0.977042935
Fign	-0.751396227	0.708626104
Fignl1	0.03361422	0.99527876
Fignl2	0.36772029	0.84870301
Filip1	-0.128897849	0.920474335
Filip1l	-0.149208696	0.957499962
Fip1l1	-0.034765906	0.99527876
Firre	0.092948053	0.803910915
Ints6	-0.41566434	0.507235129
Atp6v0d2	1.33245582	0.930571344
Fitm2	-0.152483379	0.619798471
Fiz1	0.020169021	0.987326705
Fjx1	0.008689987	0.99527876
Fkbp10	-0.067115205	0.971661054
Fkbp11	0.178750871	0.973100313
Fkbp14	-0.091256431	0.895520088
Fkbp15	-0.041907543	0.965520236
Tbc1d5	0.415465651	0.442302609
Fkbp1b	0.036967007	0.975800973
Fkbp2	0.141647972	0.630888733
Fkbp3	0.099214793	0.80819125
Fkbp4	0.042053691	0.948104382
Ncf1	0.414389638	0.389731767
Fkbp7	-0.035316592	0.99100709
Fkbp8	0.027146334	0.968005385
Fkbp9	-0.061262269	0.90358675
Fkbpl	0.165178493	0.895520088
Fkrp	0.032700584	0.971661054
AC154187.2	0.414372668	0.39534064
Flad1	-0.081656346	0.919785445
Flcn	-0.025429373	0.974823941
Fli1	-0.143178297	0.964011524
Flicr	-0.080183899	0.987597026
Flii	-0.065246445	0.91462326
Mul1	0.413244725	0.164373636
Flnb	-0.180652671	0.578812716

Flnc	0.09762314	0.98526072
Flot1	-0.033430955	0.970461999
Flot2	0.012765807	0.985742667
Rin2	0.412021281	0.010822967
Inhbb	-0.411989202	0.028541628
Flrt3	0.083333937	0.908413609
Flt1	-0.146441946	0.740041931
Flt3	0.106409117	0.882824434
Flt3l	-0.059790695	0.985673938
Flt4	-0.175224915	0.923340746
Flvcr1	-0.004863055	0.99765039
Flvcr2	-0.194516922	0.938615214
Flywch1	0.017650118	0.980339705
Flywch2	0.257081352	0.78686705
Fmc1	-0.113299368	0.911631946
Fmn1	-0.423719906	0.968005385
Fmn2	-0.060552118	0.91462326
Fmnl1	0.019991968	0.981081415
Fmnl2	-0.108803075	0.835345968
Hinfp	-0.411788116	0.208908041
Fmo1	-0.018810157	0.99527876
Repin1	0.411615201	0.393039158
Fmo5	0.093197906	0.965520236
Rps18-ps1	1.328473044	0.909604637
Fmr1	-0.129359603	0.596787845
Ostn	1.322562812	0.920642026
Fn1	-0.118456118	0.932673884
Fn3k	-0.193401981	0.589856572
Fn3krp	0.054541913	0.923996098
Fnbp1	0.119198216	0.911631946
Fnbp1l	-0.051892633	0.936031628
Fnbp4	0.043073596	0.960201673
Fndc1	-0.063171281	0.968005385
Fndc10	0.122028186	0.818840999
Fndc3a	-0.119507282	0.679604282
Fndc3b	-0.118210683	0.911631946
Fndc4	0.024071748	0.984232465
Fndc5	-0.021013953	0.984563037
Fndc7	0.525821207	0.98459388
Fndc8	-0.132394642	0.965520236
Fndc9	-0.013234617	0.996094752
Fosb	-0.411450272	0.459780215
Fnip2	0.145218977	0.56545234
Fnta	0.137336991	0.527794911

Fntb	0.088621766	0.897878088
Focad	-0.096969172	0.721943953
Folh1	0.391566981	0.527794911
Folr1	0.438124589	0.911834483
AC154442.1	1.321550155	0.895198797
Fopnl	0.027459115	0.984328758
Prr18	-0.411153799	0.037510114
Uba6	-0.40974995	0.038848214
Fosl1	-0.029096202	0.985742667
Fosl2	-0.031199904	0.975831674
Foxc1	-0.187276415	0.962648047
Scarna17	1.321524954	0.814301191
Cldn15	1.319425598	0.964941885
Foxd2	0.502386054	0.984748462
CT485613.4	1.315608211	0.803433197
Foxf1	-0.41164798	0.89703631
Foxf2	-0.412015192	0.6883862
Foxg1	-0.115818913	0.993103295
Foxj1	-0.040353567	0.98526072
Foxj2	-0.021037105	0.98459388
Foxj3	-0.068725673	0.901479608
Dsp	0.409184104	0.350732723
Foxk2	-0.00681341	0.993103295
Foxl2	-0.850522602	0.555272056
Foxl2os	-0.584349334	0.962648047
Foxm1	-0.393869226	0.802538464
Agt	0.408222201	0.045554492
Foxn3	0.061948082	0.939060335
Foxo1	0.07289631	0.936905058
Foxo3	0.026991909	0.972025261
Foxo4	0.147210075	0.639505683
Foxo6	-0.278937295	0.771684575
Il7	1.31321173	0.956922504
Foxp1	-0.207835258	0.984836179
Foxp2	-0.237413507	0.971138085
Zfp580	0.40815066	0.128640258
Foxp4	0.229222313	0.736130784
Foxq1	0.025646761	0.989464948
Foxr2	-0.452080187	0.876359276
Foxred1	0.040101433	0.965752549
Foxred2	0.446321147	0.918540666
Foxs1	-0.281370848	0.97487264
Fpgs	0.037853271	0.98459388
Fpgt	-0.089918108	0.891972665

Fpr1	0.170139854	0.990915306
Trim17	-0.407768422	0.238827832
Fra10ac1	-0.004775555	0.997115148
Fras1	-0.169189595	0.760750768
Tdo2	0.403779957	0.383515294
Frat2	0.059096693	0.968977192
Frem1	-0.180952757	0.910328122
Frem2	-0.097324797	0.965520236
Frem3	0.066020021	0.975800973
Frg1	0.030764573	0.975831674
Frg2f1	0.013701395	0.994960308
Pcmtd1	-0.403624019	0.200754354
Frmd3	0.122692673	0.968005385
Frmd4a	-0.144068186	0.623438925
Frmd4b	0.183061562	0.74463793
Frmd5	-0.376390737	0.623223056
Paqr6	-0.403332911	0.395640848
Frmd7	-0.140063415	0.946985353
Hmgb3	0.402972795	0.414106926
Mir1945	1.301400618	0.958456122
Frmpd1	0.175818143	0.670918119
Frmpd2	-0.407797173	0.895520088
Frmpd3	-0.190135665	0.642397758
Frmpd4	-0.055859843	0.965520236
Frrs1	0.943395021	0.6883862
Pik3c2b	-0.402932697	0.082752362
Frs2	-0.043283527	0.961300588
Frs3	0.122324384	0.914969279
Frs3os	0.42721433	0.90918338
Kif6	-0.402548676	0.442566158
Fryl	-0.153471279	0.774225415
Frzb	0.173153271	0.571020232
Fsbp	0.041798382	0.993489613
Fscn1	0.272620683	0.898474424
Fscn2	-0.005387055	0.998205084
Fsd1	0.111646164	0.832639343
Il5	1.299705809	0.852010446
Kncn	1.299098546	0.959276014
Fam19a3	1.294869864	0.950460219
Fst	0.164082732	0.969476616
Fstl1	0.044392525	0.959276014
Fstl3	0.170627376	0.979613636
Fstl4	-0.210104811	0.786324313
Fstl5	-0.454482259	0.860521845

Fth1	0.11863588	0.549579107
Ftl1	-0.505079883	0.783229859
Ftl1-ps1	-0.061218888	0.947602075
Npm3-ps1	1.28906671	0.810946393
Ftl2-ps	0.114832207	0.936031628
Fto	-0.025252495	0.965290366
Ftsj1	-0.007498069	0.99527876
Ftsj3	0.084469032	0.882096017
Ftx	-0.067368428	0.929997774
Fubp1	-0.072300376	0.875995577
Eml4	-0.401358995	0.184761973
Ugt8a	-0.401070785	0.064044233
Fuca2	-0.010280721	0.994960308
Fuk	0.022396384	0.984328758
Fundc1	-0.058937799	0.956164302
Fundc2	-0.045135774	0.947745582
Rprm	0.400293351	0.068273848
Furin	-0.272264574	0.932673884
Pcp4	0.39965028	0.020140149
Fut10	-0.011559709	0.99527876
Fut11	0.032535761	0.980419928
Fut2	-0.322578126	0.951497773
Fut4	-0.524685542	0.968005385
Fut7	0.551153283	0.959276014
Fut8	0.049819634	0.956922504
Fut9	-0.048809992	0.962648047
Fuz	0.013165236	0.994824604
Fv1	-0.067633987	0.97487264
Fxn	0.083014487	0.97442139
Fxr1	0.052503331	0.950460219
Fxr2	-0.030792779	0.964941885
Fxyd1	0.117508907	0.8616433
Fxyd2	0.077841159	0.973100313
Fxyd3	-0.93621934	0.928489442
Ralgapb	-0.399489692	0.151118156
Fxyd5	-0.015544288	0.99527876
Fxyd6	0.125628545	0.714072377
Fxyd7	0.035177894	0.975800973
Fyb	-0.187455846	0.911631946
Fyb2	-0.127387421	0.928957615
Fyco1	-0.213300766	0.968005385
Fyn	0.115860333	0.573810203
Fyttd1	-0.246954454	0.860142574
Fzd1	-0.076590462	0.962648047

Fzd10	0.930503359	0.618152081
Fzd2	0.150734118	0.886647768
Gys1	-0.399444664	0.241783095
Fzd4	0.041108469	0.97607383
Fzd5	0.502911191	0.710375942
Fzd6	-0.243205314	0.623438925
Fzd7	0.098262109	0.948104382
Fzd8	-0.263847654	0.624540263
Fzd9	-0.251163916	0.876359276
Fzr1	-0.0330008	0.968005385
G0s2	0.133930255	0.951468402
Pex3	0.3991876	0.160993185
G3bp1	-0.057328609	0.911631946
G3bp2	-0.079504886	0.752462699
G6pc3	0.017643348	0.985673938
G6pdx	-0.029182606	0.973100313
Mapre2	-0.398722781	0.396800203
Nr4a2	-0.397760269	0.435075615
Gab2	0.012277085	0.993489613
Gab3	-0.23737928	0.953505913
Gabarap	0.044407253	0.936031628
Dennd4a	-0.397527789	0.423074171
Gabarapl2	0.078852389	0.795890296
Gabbr1	-0.036852084	0.950195611
Gabbr2	-0.097670865	0.563984287
Sh3bp2	0.397064804	0.41351939
Gabpb1	0.11659321	0.902093754
Gabpb2	0.038403706	0.965241989
Gabra1	-0.139516919	0.549579107
Gabra2	-0.11918358	0.77527846
Gabra3	-0.070439307	0.948407374
Gabra4	-0.009479002	0.994960308
Gabra5	-0.034465394	0.98526072
Wfs1	-0.395325717	0.350732723
Adssl1	0.39452764	0.489851241
Gabrb3	-0.1097853	0.627261794
Gabrd	-0.031469713	0.981045362
Gabre	0.249584343	0.985673938
Gabrg1	-0.139143992	0.855344864
Gabrg2	-0.091595917	0.899097089
Gabrg3	-0.130602082	0.871869598
Gabrq	0.677433905	0.969755848
Gabrr1	0.412094582	0.980226283
Gabrr2	0.614211899	0.777427974

Gabrr3	0.913373883	0.905585111
Gad1	0.014652133	0.980226283
Gad1-ps	0.253540282	0.985673938
Gad1os	-0.713032842	0.941515087
Gad2	-0.115465688	0.702951568
Gadd45a	0.256763775	0.836624642
Gadd45b	-0.168821159	0.881371608
Gadd45g	0.121576884	0.869316117
Gadd45gip1	-0.016095321	0.991996237
Gadl1	0.207021009	0.910328122
Gak	-0.096710225	0.911631946
Gal	0.791881436	0.788485041
Gal3st1	-0.130640684	0.959276014
Gal3st3	-0.09189516	0.851373921
Gal3st4	0.186793988	0.930250446
Galc	0.049548179	0.953505913
Gale	-0.071284793	0.945647427
Galk1	0.158010364	0.911276003
Galk2	-0.128556152	0.747570216
Galm	0.137593685	0.949607377
Galns	0.225049901	0.539206847
Galnt1	0.067908427	0.874252042
Galnt10	-0.055438962	0.962648047
Galnt11	-0.059197376	0.948104382
Galnt12	-0.320113994	0.921311891
Galnt13	-0.015023721	0.99765039
Galnt14	0.162973185	0.795137348
Plekho2	-0.394479529	0.310553098
Galnt16	0.002227079	0.997143136
Galnt17	-0.053954886	0.895520088
Galnt18	0.047709192	0.935822376
Galnt2	0.107060606	0.800386557
Galnt3	0.023394638	0.99472062
Galnt4	0.07084462	0.963666931
Pcsk9	1.285842301	0.871869598
Galnt6	-0.269427581	0.736771155
Galnt6os	0.277748926	0.992385525
Galnt7	-0.003352569	0.997115148
Galnt9	-0.162392612	0.596982103
Ndufa3	0.394321299	0.128640258
Galr2	-0.550586307	0.947033215
Galt	0.034911612	0.968005385
Gamt	0.086891473	0.959015607
Gan	-0.083235606	0.99527876

Ganab	-0.043462767	0.948432875
Ganc	-0.224878383	0.701191891
Gap43	0.055163398	0.945663104
Snapc5	0.394147697	0.135479179
Gapdh-ps15	0.08035054	0.873797321
Gapdhs	-0.38780376	0.895520088
Gapvd1	-0.133460434	0.571020232
Micu1	-0.394146569	0.00067146
Garem1	-0.042033937	0.956164302
Garem2	-0.129994685	0.894953269
Garnl3	-0.119497966	0.911631946
Gars	0.009393873	0.990550129
Gart	0.010899324	0.991996237
Gas1	0.082190378	0.99527876
Gas2	0.011620497	0.99527876
Slirp	0.394129015	0.492371645
Gas2l2	0.612596842	0.861366392
Spint2	0.394074423	0.439971449
Gjc2	-0.393660245	0.284116109
Gas6	0.03094549	0.962648047
Gas7	-0.440165799	0.945526153
Gas8	0.006803534	0.994960308
Gata2	-0.067195587	0.985673938
Shisal2b	1.284062062	0.74428322
Gatad1	0.099228266	0.943070488
Gatad2a	-0.019874964	0.985742667
Tmtc3	-0.393619308	0.148468014
Gatb	0.001979608	0.99765039
Gatc	0.008113462	0.993380259
Rfx5	-0.393144255	0.388363872
Gatm	-0.043789511	0.966010627
Gba	-0.014448123	0.989556168
Gba2	0.0506724	0.950460219
Gbe1	0.142023979	0.8549794
Cited2	-0.39284157	0.422728382
Gbgt1	-0.971917953	0.815987743
Mir6947	1.277867301	0.929095022
Gbp11	-0.528867434	0.893455801
Gbp2	-0.358184423	0.896263151
Gbp3	-0.379305018	0.736130784
Gbp4	-0.582995059	0.752462699
Pfdn4	0.392296061	0.294902721
Phf24	1.277633498	0.663678416
Gbp7	0.084023633	0.981045362

Gbp9	-0.617561944	0.509834443
Gbx1	-0.06476425	0.99527876
Hsf2bp	1.275980798	0.596982103
Gca	-0.123122458	0.895520088
Gcat	0.049486725	0.984748462
Gcc1	-0.061451664	0.946985353
Gcc2	-0.032349182	0.97487264
Gcdh	0.06977532	0.911631946
Gcfc2	0.120957996	0.891265024
Gch1	0.127974498	0.973100313
Fpr2	1.272244004	0.936031628
Gck	0.384670727	0.74463793
Gckr	-0.973635561	0.936031628
Gclc	-0.011944415	0.985742667
Mir6910	1.267134634	0.946239011
Gcn1l1	-0.112924788	0.702848528
Gcnt1	-0.150959872	0.948104382
Gcnt2	0.083752918	0.911206484
Gcnt4	-0.296652069	0.754350047
Gcnt7	-0.938075246	0.963666931
Gcsh	0.083431956	0.885469597
Gda	-0.011965782	0.984232465
Gdap1	-0.606884594	0.882096017
Gdap10	-0.664726146	0.565530155
Gdap1l1	-0.045840105	0.968005385
Gdap2	-0.039310275	0.963666931
Gde1	-0.071387599	0.8549794
Gdf1	0.576074668	0.981746088
Gdf10	0.074316655	0.962648047
Gdf11	0.055904689	0.962648047
Sbspon	1.265031231	0.936005368
Gdf5	0.804629366	0.945663104
Gdf6	0.592432228	0.962648047
Xiap	1.264929841	0.544361994
Gdf9	0.281400241	0.942274311
Gdi1	0.069535964	0.792913409
Gdi2	-0.139987509	0.764783842
Gdnf	-0.013175077	0.99827037
Gdpd1	-0.078704821	0.90358675
Gdpd2	-0.341125769	0.670918119
Gdpd3	0.316915954	0.932673884
Gdpd5	0.094588728	0.950520575
Gdpgp1	-0.019892323	0.988034453
Gem	-0.192174467	0.952034929

Gemin2	0.082494949	0.957773729
Gemin4	-0.192869226	0.8549794
Gemin5	-0.094686195	0.858549868
Gemin6	0.16302187	0.918407269
Lmbr1	-0.392269635	0.010117553
Gemin8	-0.160002926	0.887625873
Gen1	0.31163345	0.852010446
Get4	0.040905372	0.965520236
Gfap	0.020227822	0.984232465
Gfer	-0.149862515	0.803910915
Gfm1	0.054091764	0.911631946
Gfm2	0.004071906	0.99527876
Gfod1	-0.072014434	0.871869598
Gfod2	-0.030475383	0.975800973
Atp5k	0.391865242	0.084047203
Gfpt2	0.137015192	0.910328122
Gfra1	0.337314976	0.573101161
Kirrel2	0.391823356	0.389153312
Gfra4	0.131436042	0.865075531
Trim55	1.263990815	0.938394547
Rnaseh2c	0.390801631	0.091941163
Gga1	-0.048924977	0.965520236
Gga2	0.205237676	0.623618199
Mettl4	-0.390374319	0.338645928
Ggact	0.284927133	0.661684145
Ggct	0.164451746	0.698427195
Dlx1	0.389038831	0.050986442
Ggh	0.007179093	0.99527876
Ggn	-0.324972044	0.950460219
Ggnbp1	-0.556372755	0.961373288
Ggnbp2	-0.028182859	0.984836179
Ggps1	-0.051376937	0.931937569
Ggt1	-0.021436262	0.99527876
Ggt5	0.454766793	0.669849621
Ggt6	0.264817488	0.989840188
Ggt7	-0.038095806	0.949607377
Ggta1	0.199192002	0.897878088
Ghdc	-0.040799987	0.985735386
Ghitm	-0.070404965	0.74463793
Ghr	-0.103022818	0.939052665
Gtsf1l	1.261991923	0.91462326
Ghsr	-0.358618974	0.851875536
Gid4	0.013172614	0.985742667
Gid8	0.049729011	0.948104382

Gigyf1	0.022573452	0.981392364
Gigyf2	-0.080852249	0.971138085
Gimap1	0.123092466	0.974962198
Gimap3	0.909853583	0.962648047
Gimap4	0.825405314	0.945117894
Gimap5	-0.674122934	0.904299868
Gimap6	0.325116588	0.611658468
Gimap8	-0.642419602	0.582101678
Gimap9	-0.057066162	0.99527876
Gin1	-0.066698806	0.962648047
Ginm1	-0.155966827	0.896196194
Gins1	0.12258348	0.97487264
Gins2	0.032545816	0.995812671
Gins3	0.026161024	0.992385525
Gins4	0.116804609	0.837382879
Gipc1	-0.03417373	0.965520236
Gipc2	-0.087369228	0.991258057
Gipc3	-0.056115125	0.985673938
Gipr	0.417984354	0.778738711
Ppl	0.388866733	0.508473202
Git2	-0.068680594	0.910328122
Gja1	-0.275072121	0.65012378
Gja3	-0.017982684	0.995411429
Gja4	0.162704508	0.963666931
Gja5	-0.055236275	0.99527876
Gja6	0.493375844	0.965290366
Gjb1	0.087226875	0.964011524
Gjb2	-0.704349109	0.677705721
Gjb3	-0.777604949	0.90358675
Cd55	-0.388744878	0.323832374
Gjc1	0.370600004	0.74632237
Pgr	-0.388522747	0.031985521
Gjc3	-0.10265448	0.758595939
Gjd2	0.079668675	0.968005385
Gjd3	-0.0939187	0.991996237
Gk	-0.028617457	0.981502501
Gk5	-0.373255316	0.544721388
Gkap1	0.062281321	0.970348278
Gkn3	-0.177572157	0.955993235
Gla	-0.067632474	0.961727797
Glb1	-0.065543733	0.958375046
Il10ra	-0.387327347	0.465412834
Glb1l2	-0.48015033	0.824306801
Glcci1	0.399221425	0.963666931

Glce	2.1498E-05	0.99991697
Gldc	-0.087841796	0.868797028
Gldn	-0.131583255	0.965520236
Gle1	0.112556568	0.585734325
Chrac1	0.387211773	0.106842205
Gli1	-0.079269659	0.980626239
Gli2	-0.009566719	0.99527876
Gli3	-0.09078095	0.962648047
Glipr1	0.023927823	0.99527876
Cep295	-0.386890328	0.037188261
Glipr2	-0.232821765	0.868415875
Glis1	0.447561433	0.960201673
Glis2	-0.026234876	0.99527876
Glis3	0.173290264	0.854454414
Glmn	-0.104206097	0.924361537
Glmp	-0.119696581	0.9069528
Glns-ps1	0.756395233	0.607818467
Glo1	0.009960879	0.989840188
Glod4	0.021970872	0.975831674
Glod5	0.811264016	0.944753667
Lsmem1	1.260358693	0.912928483
Glp2r	0.385206306	0.674357582
Glra1	0.073053065	0.997115148
Glra2	0.198579843	0.823072541
Glra3	0.053855111	0.988034453
Jaml	1.258337909	0.896263151
Glrb	0.021306411	0.974823941
Glrp1	0.025444993	0.997258205
Glrx	0.082330413	0.953505913
Glrx2	0.407773002	0.844084751
Plekhd1	0.386283575	0.508473202
Glrx5	0.004334621	0.996487647
Cep85l	-0.386111163	0.028541628
Gls2	-0.075748277	0.956164302
Glt1d1	0.155100232	0.66978628
Glt28d2	0.524811513	0.968005385
Glt8d1	0.003213899	0.996638606
Glt8d2	-0.023777948	0.985742667
Gltp	-0.056127635	0.962648047
Gltpd2	0.523878279	0.98526072
Glud-ps	-0.273481467	0.989200732
Glud1	0.061028135	0.84870301
Erbb3	-0.385587887	0.196638564
Glyat	0.806253894	0.965256427

Glycam1	-0.086929816	0.99527876
Glyctk	-0.011978708	0.99527876
Glyr1	0.024981038	0.962648047
Gmcl1	-0.089539514	0.825249193
Zbtb7b	0.383642263	0.076364505
Gmeb1	-0.063268811	0.968005385
Gmeb2	0.062663882	0.950770028
Gmfb	-0.027384696	0.965290366
Gmfg	0.199494106	0.963943597
Asic3	1.252135415	0.684925922
Gmip	0.002125419	0.998010523
Gmnc	-0.098963739	0.982437522
Gmnn	0.037950453	0.987326705
Gmppa	0.026790586	0.980339705
Gmppb	0.012599089	0.994960308
Gmpr	-0.014379871	0.985742667
Gmpr2	-0.08191017	0.899097089
Gmps	-0.066458203	0.872352604
Gna11	-0.043256429	0.958389182
Gna12	-0.06091611	0.923011738
Gna13	-0.03120755	0.965520236
Gna14	0.08096491	0.985446046
Gna15	-0.406476836	0.837755777
Gnai1	-0.069963347	0.897878088
Gnai2	-0.053418106	0.896196194
Gnai3	0.004388948	0.99527876
Gnal	0.037785823	0.962648047
Mpv17l	-0.383088372	0.413935733
Gnaq	-0.063705325	0.883131585
Gnas	0.055434729	0.887135194
Gnat1	-0.34102806	0.98187819
Gnaz	-0.037729948	0.97089157
Gnb1	-0.089174579	0.810908327
Gnb1l	-0.232709837	0.911631946
Gnb2	-0.081510442	0.886895881
Gnb3	0.766032216	0.968005385
Gnb4	0.006911518	0.99527876
Gnb5	-0.138894276	0.889972515
Gne	0.072124918	0.932673884
Gng10	0.040263959	0.962648047
Gng11	-0.047968089	0.980109544
Gng12	-0.002581246	0.996487647
Gng13	0.300932614	0.791185365
Gng2	0.018216117	0.981694523

Gng3	0.02298825	0.982309415
Gng4	-0.031271449	0.976257618
Gng5	0.071839545	0.965520236
Gng7	-0.029966869	0.985673938
Gng8	0.115077058	0.98459388
Gngt2	-0.504490087	0.528197276
Gnl1	0.053840292	0.890704638
Gnl2	0.081385898	0.911631946
Gnl3	0.040116426	0.965520236
Gnl3l	0.007950016	0.991996237
Gnmt	0.546939733	0.825148733
Gnpat	0.031213388	0.969755848
Gnpda1	-0.066205378	0.932673884
Gnpda2	0.190309122	0.875995577
Gnpnat1	-0.343619095	0.629280869
Gnptab	-0.141098817	0.732729469
Gnptg	0.097835073	0.700314498
Gnrh1	-0.041822833	0.99527876
Gns	-0.040757229	0.927471971
Golga1	-0.172344609	0.91507125
Golga2	-0.004303915	0.99527876
Golga3	-0.051927079	0.921473132
Golga4	-0.102126092	0.852010446
Golga5	0.048237638	0.962648047
Golga7	0.125146224	0.727420355
Golga7b	-0.021748821	0.983694404
Plekha6	-0.382403084	0.247730004
Golim4	-0.260334124	0.861139061
Golm1	0.070163235	0.99527876
Golph3	-0.062025279	0.864121718
Golph3l	0.877561784	0.774804043
Golt1b	0.065118973	0.932673884
Trim59	-0.381883272	0.30307072
Gon7	-0.005987255	0.997241386
Gopc	-0.811103967	0.707520358
Gorab	0.06866483	0.962648047
Gorasp1	-0.06543069	0.952034929
Gorasp2	0.025709943	0.969755848
Gosr1	0.04374393	0.948407374
Gosr2	-0.010727701	0.991996237
Got1	0.040889414	0.948104382
Got1l1	-0.022997374	0.99640101
Got2	0.091149427	0.696573735
Got2-ps1	0.088799954	0.993103295

Gp1ba	0.131079303	0.981746088
Gp1bb	-0.103589856	0.985742667
Gp5	0.645156	0.56488584
Gp9	0.417837598	0.958720519
Gpaa1	-0.041184297	0.974823941
Gpalpp1	-0.096587919	0.803093104
Gpam	-0.131210217	0.623042338
Gpank1	-0.067827415	0.964011524
Rpl36-ps2	1.249256362	0.705332654
Gpat3	-0.223562171	0.91462326
Gpat4	-0.006586122	0.99527876
Gpatch1	-0.041878808	0.961859633
Gpatch11	0.068502255	0.909547396
Gpatch2	-0.104949409	0.825249193
Gpatch2l	-0.062119252	0.928957615
Gpatch3	-0.082309987	0.965520236
Gpatch4	-0.060166035	0.99165967
Gpatch8	0.012087869	0.988034453
Gpbp1	0.017612349	0.988034453
Gpbp1l1	-0.078989686	0.889972515
Gpc1	-0.056081708	0.955993235
Gpc2	0.896490341	0.629280869
Gpc3	0.076044141	0.984328758
Gpc4	0.170378979	0.702951568
Hmox2	0.381742701	0.377143991
Rps26	0.381491917	0.000548523
Hmgb2	0.380758817	0.333307116
Mt1	0.380638763	0.000552397
Gpd1l	-0.04276385	0.920642026
Gpd2	-0.115151189	0.641911698
Gper1	-0.305760851	0.864150749
Hbq1a	1.245064194	0.718702264
Gphn	-0.089347174	0.837755777
Gpi1	-0.048122137	0.936787526
Gpkow	0.040227498	0.956922504
Gpld1	0.12547119	0.798580668
Gpm6a	-0.003810707	0.99527876
Gpm6b	0.010918868	0.989840188
Gpn1	0.136238211	0.620124996
Gpn2	-0.006706586	0.996094752
Gpn3	0.135820801	0.8871895
Gpnmb	-0.011494415	0.99527876
Gpr1	-0.354665752	0.934674534
Gpr101	-0.175358182	0.920642026

Gpr107	-0.026915779	0.974823941
Gpr108	0.147716485	0.84870301
Gpr12	-0.421772349	0.813552442
Gpr132	-0.274673154	0.993103295
Gpr135	-0.017892338	0.987342614
Gpr137	0.022075233	0.985742667
Gpr137b	-0.034684361	0.984836179
Gpr137b-ps	-0.107509205	0.914969279
Gpr137c	-0.085450614	0.938394547
Gpr139	-0.415914735	0.948104382
Esrp1	1.240954655	0.884426973
Wwp1	-0.380589844	0.054826708
Gpr149	-0.259220804	0.911206484
Gpr15	-0.129494167	0.985742667
Gpr150	0.058551155	0.98771048
Gpr151	0.119422894	0.97336875
Gpr153	-0.088843921	0.980626239
Gpr155	-0.148683096	0.768977675
Gpr156	0.406977634	0.869316117
Gpr157	-0.101904509	0.971661054
Gpr158	-0.076771583	0.852010446
Snord100	1.238664513	0.904346827
Gpr161	-0.053720412	0.962648047
Gpr162	0.010461799	0.991438757
Gpr165	-0.110347824	0.965520236
Gpr17	-0.061707649	0.962648047
Gpr173	0.038498274	0.965520236
Gpr176	-0.272433039	0.554782791
Gpr179	-0.662539204	0.91462326
Gpr180	0.017518968	0.991438757
Gpr182	-0.651521727	0.665532517
Gpr183	0.437486083	0.849517436
Gpr19	0.296133015	0.758595939
Ccdc28b	0.379962548	0.055853534
Gpr21	0.779382485	0.875723186
Gpr22	-0.043320171	0.97469529
Gpr25	-0.048954307	0.994960308
Pggt1b	-0.379000325	0.407983695
Gpr27	0.155601348	0.851875536
Fcf1	0.377126197	0.034538038
Gpr34	0.15069058	0.871869598
Gpr35	0.044452305	0.99527876
Gpr37	-0.086751904	0.890704638
Smad4	-0.376959272	0.020683402

Gpr39	-0.052097173	0.991996237
Gpr4	-0.157482963	0.961300588
Gpr45	-0.111282466	0.882824434
Gpr55	-0.620762757	0.695239733
Gpr6	-0.644008139	0.898478018
Gpr61	0.093094735	0.903795193
Gpr62	-0.141484409	0.900182217
Pet100	0.376080158	0.386030517
Gpr65	0.381583836	0.975831674
Gpr68	0.127986451	0.891972665
Gpr75	0.117371743	0.891574277
Olfr77	1.234355766	0.920642026
Gpr83	0.009504742	0.99527876
Gpr84	-0.362335636	0.940504226
Gpr85	-0.149564627	0.629280869
Gpr88	0.371651628	0.962648047
Gpr89	-0.132147757	0.685061817
Gprasp1	-0.031356806	0.952633594
Gprasp2	-0.027315391	0.984748462
Gprc5b	0.061106544	0.928016592
Gprc5c	-0.057134556	0.984843578
Gprc5d	-0.604561778	0.895520088
Gprin1	-0.08933065	0.910328122
Gprin2	0.015666395	0.99527876
Gprin3	-0.243273829	0.861366392
Gps1	0.064457739	0.959276014
Gps2	0.159004407	0.74463793
Gpsm1	-0.121665999	0.865884071
Gpsm2	-1.13354E-05	0.99991697
Gpsm3	0.207171148	0.919785445
Gpt	0.180225092	0.777561139
Gpt2	0.045305154	0.958375046
Gpx1	-0.076795739	0.907572296
Gpx2	0.848246171	0.954827342
Gpx3	-0.014714961	0.99527876
Gpx4	0.10933348	0.600671501
Gpx4-ps2	0.139696913	0.754350047
Gpx7	0.079882796	0.973100313
Gpx8	0.110363587	0.965520236
Gramd1a	-0.240587952	0.65469339
Gramd1b	0.644035471	0.806077586
Gramd1c	0.042905656	0.985673938
Gramd2	-0.032444607	0.994960308
Ankrd63	-0.375601182	0.104879534

Gramd4	0.02854424	0.975350978
Grap	0.040733076	0.985673938
Grap2	-0.700457268	0.922889447
Grasp	-0.039897875	0.968005385
Grb10	0.141949147	0.632633706
Lefty1	-0.375353705	0.509250303
Grb2	0.681982185	0.664771806
Guca1a	1.231591181	0.936031628
Grcc10	0.031142108	0.975831674
Greb1	-0.027699284	0.997529192
Greb1l	-0.097924475	0.899097089
Prox2	-0.375031802	0.399337171
Grem2	0.119333758	0.896196194
Grhl1	0.018399015	0.985742667
Grhl2	0.1498896	0.984748462
Grhl3	0.54146833	0.852010446
Grhpr	0.040028521	0.97487264
Gria1	-0.116569762	0.663393433
Gria2	-0.155881474	0.549579107
U2af1l4	0.374846855	0.323832374
Gria4	0.020763502	0.977133618
Grid1	-0.093717475	0.736130784
Grid2	-0.039650869	0.981392364
Grid2ip	0.235032103	0.942812446
Grik1	-0.125497355	0.922887518
Grik2	0.02720252	0.980132658
Grik3	0.044703139	0.980132658
Grik4	0.06348754	0.903441375
Grik5	-0.033097718	0.95520652
Grin1	0.018614567	0.987607832
Grin1os	-0.088889908	0.911631946
Ccdc141	0.374499508	0.090653603
Ccdc51	0.374299448	0.436138179
Grin2c	-0.073666152	0.891972665
Grin2d	0.014803483	0.99527876
Grin3a	-0.067197172	0.955993235
Grin3b	0.439797599	0.97607383
Grina	0.022982875	0.970673196
Grip1	-0.080471615	0.956164302
AC147227.1	1.230796853	0.895198797
Grip2	-0.097889865	0.921117568
Gripap1	0.047116423	0.926806424
Grk2	-0.003467234	0.99527876
Grk3	0.040451365	0.962648047

Grk4	-0.215739821	0.911631946
Grk5	-0.070271577	0.965520236
Grk6	-0.006402001	0.99527876
Grm1	-0.042476326	0.944753667
Grm2	0.118552572	0.922718296
Grm3	-0.075696432	0.911631946
Grm4	0.109959199	0.962648047
Dubr	0.374277915	0.298968315
Grm7	-0.052618791	0.935897306
Grm8	0.40005766	0.613198201
Grn	-0.117903866	0.855344864
Grp	-0.059427707	0.976978183
Grpel1	0.010677541	0.991996237
Grpel2	-0.01310164	0.990915306
Grpr	-0.347961988	0.965520236
Grrp1	0.171431455	0.975831674
Grsf1	-0.027246664	0.965520236
Grtp1	0.050923102	0.985653607
Grwd1	0.067353169	0.948104382
Grxcr2	-0.873047384	0.973590542
Gsap	-0.012624602	0.994960308
Gsdmc4	0.999760379	0.962648047
Gsdmd	-0.205941745	0.932673884
Gsdme	-0.039493796	0.965520236
Tbca	0.3742291	0.034538038
Gsg1	-0.770561651	0.911631946
Hddc3	0.373722789	0.47047681
Gsk3a	0.042174204	0.941064107
Dyrk4	1.228520688	0.868415875
Gskip	0.004667852	0.99527876
Gsn	-0.118270155	0.858861871
Gspt1	-0.026594886	0.980132658
Gspt2	0.005581816	0.99527876
Gsr	0.104952577	0.875723186
Gss	0.193105029	0.783229859
AC140186.1	1.224747394	0.833916587
Gssos2	-0.108195384	0.98623785
Mir8102	1.224121075	0.911631946
Gsta3	0.663554273	0.665532517
Gsta4	0.085630725	0.825910982
Gstcd	0.219753521	0.803910915
Gstk1	-0.046506149	0.975831674
Gstm1	0.198606631	0.534461098
Gstm2	-0.018924047	0.995944208

Gstm2-ps1	0.362205574	0.871869598
Gstm4	0.089015768	0.938394547
Gstm5	0.091579967	0.754112547
Gstm6	-0.106807538	0.894953269
Gstm7	0.059662987	0.958389182
Gsto1	0.118337085	0.786324313
Gsto2	-0.023465251	0.99527876
Gstp-ps	0.130706439	0.850740887
Gstp1	0.090195181	0.813346233
Gstp2	-0.013473942	0.99527876
Gstt1	-0.165423272	0.911631946
Gstt2	0.205691072	0.937607785
Gstt3	0.076813209	0.980132658
Gstz1	0.086619468	0.968005385
Gsx1	-0.587551927	0.911631946
Gsx2	0.983157443	0.806077586
Gt(ROSA)26Sor	-0.149785594	0.908554604
Gtdc1	0.168768771	0.801926713
Gtf2a1	0.020032566	0.984328758
Gtf2a2	0.231415469	0.544661714
Gtf2b	0.096349072	0.862285215
Gtf2e1	0.028128246	0.981502501
Gtf2e2	-0.02982928	0.985673938
Gtf2f1	0.122265176	0.609167039
Dcps	0.373696626	0.468667381
Gtf2h1	0.016138435	0.985735386
Gtf2h2	0.089933804	0.881407916
Gtf2h3	-0.002162672	0.99765039
Gtf2h4	0.040232987	0.981392364
Gtf2h5	0.072501443	0.852946549
Gtf2i	-0.04253035	0.929095022
Gtf2ird1	0.012993	0.99527876
Gtf2ird2	0.097256253	0.940060336
Gtf3a	0.044321933	0.971661054
Gtf3c1	-0.076772206	0.868625126
Gtf3c2	-0.115914851	0.707777756
Gtf3c3	-0.05036998	0.947033215
Gtf3c4	-0.134791874	0.690084266
Gtf3c5	0.109526702	0.897878088
Gtf3c6	0.074809715	0.932673884
Gtpbp1	0.029243144	0.968005385
Gtpbp10	0.205823159	0.758046356
Gtpbp2	-0.021835052	0.993380259
Gtpbp3	0.051314895	0.962648047

Gtpbp4	-0.031038217	0.97469529
Rad1	0.372978421	0.370298654
Gtpbp8	-0.049030711	0.964011524
Gtse1	-0.002695432	0.99900438
Ppp1r2-ps1	1.223968386	0.909243078
Ferd3l	1.22142776	0.896196194
Guca1b	-0.429779411	0.917685272
Gucd1	-0.075319502	0.936905058
Gucy1a1	0.013443964	0.991996237
Gucy1a2	-0.254167905	0.962094124
Gucy1b1	0.049439704	0.945647427
Gucy1b2	0.487608091	0.91462326
Nipsnap2	0.372549038	0.40129356
Gucy2e	0.006150953	0.998010523
Gucy2f	0.211320335	0.971661054
Gucy2g	0.236667408	0.813630269
Guf1	-0.052181932	0.962648047
Guk1	0.052777955	0.950460219
Gulp1	-0.446171762	0.758595939
Gusb	-0.042680562	0.98043358
Gvin1	-0.739182235	0.985673938
Tmem30c	1.218727874	0.724910334
Gxylt2	-0.049213959	0.99100709
Gyg	0.032716659	0.980339705
Gypa	0.97407056	0.968005385
Gypc	0.415299833	0.841017002
Brcc3	0.372022722	0.052514536
Rps4x-ps	1.218454778	0.779231312
Gzf1	-0.061052607	0.911631946
Gzma	-0.900368024	0.964011524
Gzmk	-0.247667477	0.974962198
Gzmm	-0.094229498	0.981694523
H13	-0.041849647	0.948407374
Fam90a1b	1.218077488	0.596982103
Cgnl1	-0.371611446	0.403453477
H1fx	0.323549498	0.553668477
H2-Aa	0.063040965	0.99527876
H2-Ab1	-0.695786272	0.91462326
H2-D1	0.001451493	0.998010523
H2-DMa	-0.123894094	0.858549868
H2-DMb1	-0.068982353	0.985742667
H2-DMb2	0.406968482	0.964941885
H2-Eb1	-0.069659637	0.991996237
H2-K1	0.064174719	0.972218452

H2-K2	0.269401764	0.952385926
H2-Ke6	0.117330422	0.889972515
H2-M10.2	-0.296164384	0.962648047
H2-M3	-0.110908183	0.974962198
H2-M5	-0.117544258	0.980339705
Snord14e	1.208145084	0.812033693
H2-Oa	-0.997118252	0.792913409
H2-Ob	0.333426761	0.962648047
Dcdc2c	1.203806108	0.965520236
H2-Q4	-0.001258918	0.999242708
H2-Q5	0.243097773	0.993290512
H2-Q6	0.150061766	0.984328758
H2-Q7	-0.712497299	0.874971335
H2-T-ps	0.292099915	0.983479691
H2-T10	0.350836683	0.89011487
H2-T22	0.023270094	0.984563037
H2-T23	-0.002731615	0.998010523
H2-T24	-0.306172875	0.786324313
H2afj	0.079971063	0.951390592
H2afv	0.021312067	0.982200476
H2afx	0.155904401	0.779231312
H2afy	0.053000054	0.922887518
H2afy2	0.119253927	0.88456107
Cutc	0.371373491	0.508471705
H2al1k	0.668825376	NA
H2al1m	0.075060051	NA
H3f3a	-0.153128882	0.815912409
H3f3a-ps1	-0.407834101	0.936972904
H3f3a-ps2	0.28131005	0.953505913
H3f3aos	-0.324459823	0.882824434
Ndst3	-0.371027154	0.121443772
H3f3c	0.241793771	0.724910334
Tgif2-ps2	1.203656675	0.80931022
H6pd	-0.06480107	0.973100313
Haao	-0.026583384	0.997241386
Habp4	0.093085478	0.898478018
Hacd1	0.250574721	0.721943953
Hacd2	0.035985623	0.962648047
Hacd3	-0.062048361	0.894953269
Hacd4	-0.232142377	0.868415875
Hace1	-0.10841026	0.856578046
Hacl1	-0.200169613	0.764783842
Hadh	0.057385945	0.960176242
Hadha	-0.017532088	0.981502501

Hadhb	-0.125456327	0.749519672
Hagh	0.054759167	0.932673884
Haghl	0.070942564	0.8549794
BB365896	1.201511216	0.962648047
Gas5	0.370955052	0.003660124
Hamp2	-0.62577983	0.976956375
Ptpn7	1.188978906	0.87564489
Hap1	0.122541129	0.837755777
Hapln1	-0.145417708	0.805999925
Hapln2	0.122907492	0.938939871
Hapln3	-0.337816549	0.891972665
Hapln4	-0.137462464	0.578812716
Harbi1	0.041797408	0.98526072
Hars	0.090523555	0.731132047
Hars2	-0.01481139	0.985742667
Has1	-0.349903808	0.968005385
Has2	-0.974336134	0.955993235
Has2os	0.662616354	0.965241989
Has3	-0.216570976	0.910328122
Haspin	-0.419179995	0.972218452
Hat1	0.101115712	0.89703631
Haus1	-0.094586119	0.965520236
Zfhx2	-0.370887474	0.00014965
Haus3	-0.108842016	0.963943597
Haus4	-0.166963642	0.924361537
Haus5	-0.257286351	0.814682509
Haus6	-0.174952325	0.736948517
Haus7	0.102968371	0.95363833
Haus8	0.09727557	0.965520236
Havcr2	0.025224624	0.991996237
Hax1	-0.02253012	0.988553438
Hba-a1	0.18320535	0.968005385
Hba-a2	0.25159139	0.951817826
Hba-ps4	-0.581855671	0.968005385
Hbb-bs	-0.182635368	0.962648047
Hbb-bt	-0.155797226	0.97487264
Hbegf	0.130701449	0.886647768
Hbp1	-8.10714E-05	0.999724849
Abca12	1.188479203	0.948104382
Hbq1b	-0.399585183	0.965520236
Hbs1l	-0.0716316	0.908554604
Dhx58os	1.187315822	0.932673884
Hcar1	-0.453174508	0.948104382
Hcar2	-0.517742299	0.981392364

Hccs	-0.104745818	0.8549794
Prom1	0.370598499	0.151118156
Hcfc1r1	-0.091058896	0.911631946
Hcfc2	-0.153262985	0.969755848
Hck	0.570882451	0.667414999
Hcls1	-0.088535949	0.961583757
Kif21b	-0.370287349	0.040114812
Hcn2	0.032325022	0.969653237
Hcn3	-0.366165306	0.534461098
Hcn4	-0.353849034	0.821998633
Hcrtr1	-0.060362385	0.989556168
Hcrtr2	-0.237911797	0.910328122
Aplnr	1.183287249	0.936031628
Hdac1	0.102111544	0.873797321
Hdac10	0.086465811	0.948104382
Hdac11	-0.017223139	0.985673938
Hdac2	0.034501317	0.966608583
Htra1	0.370278902	0.000767316
Hdac4	0.021351356	0.984328758
Hdac5	-0.104088578	0.760547704
Hdac6	0.020014104	0.984947802
Hdac7	-0.056848384	0.965520236
Hdac8	0.065510906	0.956164302
Hdac9	-0.097064245	0.889566486
Hdc	-0.198914014	0.91507125
Hddc2	0.040879472	0.972025261
Map2	0.369075994	0.128871401
Hdgf	0.045683389	0.912917153
Hdgfl2	0.081386673	0.860142574
Hdgfl3	-0.023465084	0.97487264
Hdhd2	0.029449075	0.96607835
Hdhd3	-0.030743603	0.985742667
Hdhd5	0.001719496	0.998010523
Hdlbp	-0.103144638	0.729405774
Hdx	-0.086888416	0.949948601
Heatr1	-0.060237447	0.932931799
Heatr3	-0.06577251	0.915417975
Heatr4	0.114145289	0.99527876
Heatr5a	-0.055142033	0.968005385
Heatr5b	-0.141705212	0.571020232
Heatr6	-0.00276035	0.997375151
Hebp1	0.076035003	0.911631946
Ankrd54	-0.368732989	0.509250303
Heca	-0.159276147	0.809593157

Hectd1	-0.04953062	0.941064107
Hectd2	0.212521547	0.968630572
Hectd2os	0.050090233	0.993380259
Hectd3	-0.091258233	0.810908327
Nes	-0.368384638	0.329158589
Hecw1	-0.053248594	0.962648047
Hecw2	-0.11717282	0.875119971
Heg1	-0.035157382	0.993114821
Helb	-0.216633298	0.803910915
Hells	-0.038109866	0.991438757
Helq	-0.088251436	0.932673884
Helz	-0.737223601	0.825148733
Helz2	0.267850703	0.89703631
Hemk1	0.007086848	0.99527876
Henmt1	0.270641693	0.894953269
Eva1a	0.368301435	0.209137754
Hepacam2	-0.363804769	0.951390592
Heph	0.299220449	0.553031868
Herc1	-0.103283277	0.689605643
Herc2	-0.12536031	0.583892778
Lgals3bp	0.367842451	0.323832374
Herc4	-0.083875663	0.889908105
Herc6	-0.374987648	0.539353804
Herpud1	0.062903141	0.950195611
Herpud2	-0.115177448	0.708626104
Hes1	0.232746403	0.819778986
Hes3	0.447831858	0.985742667
Hes5	-0.162933167	0.939052665
Hes6	0.045045518	0.984748462
Hes7	0.432926404	0.861366392
Hexa	-0.152533743	0.565635635
Hexb	0.101746598	0.688916428
Hexdc	-0.064886279	0.962648047
Hexim1	-0.004537538	0.99527876
Hexim2	0.032460858	0.991559279
Hey1	-0.092890224	0.822693227
Hey2	-0.006979849	0.997115148
Heyl	-0.211224163	0.868415875
Hfe	0.086406022	0.969755848
Vmn2r85	1.181585519	0.70326723
Hfm1	-0.2951253	0.911631946
Hgd	0.077489443	0.997115148
Hgf	-0.376992583	0.861909929
AC087802.4	1.181378293	0.949450803

Hgh1	-0.016756768	0.994960308
Hgs	-0.071070662	0.898478018
Hgsnat	-0.080369147	0.871869598
Hhat	-0.629055534	0.751630609
Hhatl	0.023563372	0.991996237
Hhex	-0.373873466	0.867827234
Hhip	-0.017998192	0.99527876
Hhipl1	-0.078404735	0.959597516
Hhipl2	-0.177143777	0.975831674
Hibadh	-0.035746736	0.965520236
Hibch	0.012451292	0.993380259
Hic1	-0.39754243	0.852010446
Hic2	-0.05041839	0.97469529
Hid1	-0.038740309	0.951817826
Hif1a	-0.111175935	0.814301191
Hif1an	-0.106036033	0.623438925
Ak4	-0.367795569	0.426109731
Higd1a	0.057301299	0.948407374
Higd1b	0.086041744	0.978063275
Higd2a	0.041107482	0.965520236
Hikeshi	0.061948027	0.932673884
Hilpda	0.03762512	0.985742667
Mrgbp	0.367772282	0.04370223
Hint1	0.131171541	0.740240396
Hint2	0.264544502	0.688803555
Hint3	0.021279589	0.994960308
Hip1	0.040976784	0.965520236
Hip1r	-0.031303354	0.968005385
Hipk1	-0.001983254	0.997258205
Hipk2	-0.232074291	0.596982103
Hipk3	-0.172944978	0.513773444
Hipk4	-0.332275016	0.911631946
Hira	-0.085591544	0.798109023
Hirip3	0.125336405	0.837755777
Hist1h1b	-0.156017489	0.99527876
Igsf9b	-0.367649016	0.014874927
Rpl3l	1.178285256	0.630931694
Hist1h1e	-0.484310498	0.873753967
Hist1h2ab	0.304931249	0.991996237
Rpl38	0.367605636	0.082752362
Qprt	1.171476096	0.703479789
Hist1h2ae	-0.259239338	0.985742667
Hist1h2af	0.988112711	NA
Gclm	1.170575771	0.5542082

Snord73a	1.170267872	0.939904081
Hist1h2aj	-0.390374597	NA
Hist1h2ap	-0.348898636	NA
Hist1h2bb	-0.718532016	0.968005385
Hist1h2bc	0.015520593	0.991996237
Hist1h2be	-0.467084087	0.730891136
Hist1h2bf	-0.022413528	0.997375151
Hist1h2bg	-0.092891287	0.968005385
Hist1h2bh	0.598807827	0.942812446
Hist1h2bj	-0.256638362	0.987342614
Hist1h2bk	0.681546465	0.962648047
Hist1h2bl	0.488302301	NA
Hist1h2bn	0.603251815	0.951901417
Hist1h2bp	0.300299406	0.985446046
Polr2k	0.367597403	0.442302609
Hist1h2br	0.062173145	0.994960308
Hist1h3b	-0.950875937	0.975350978
Mogat1	1.167974726	0.918451137
Olfr91	1.158142319	0.935897306
Hist1h3f	0.135993562	0.99527876
Hist1h3g	0.438351321	NA
Pi16	1.158048996	0.56399886
Hist1h4a	0.088972331	0.997115148
Hist1h4b	0.301885714	0.979400804
BC053393	1.156635633	0.962648047
Hc	1.155943017	0.767515266
Hist1h4h	0.325841833	0.895520088
Hist1h4i	0.098465287	0.980896205
Hist1h4j	0.33221122	0.969661076
Hist1h4k	-0.802488862	0.740833211
Hist1h4m	-0.609450199	0.975831674
Hist1h4n	-0.920449689	0.890692547
Hist2h2ab	0.136051672	0.991438757
Hist2h2ac	0.672985423	0.950252558
Hist2h2be	0.101599757	0.939060335
Hist2h3c1	-0.414511142	0.760750768
Hist2h3c2	-0.25904923	0.948104382
Hist2h4	-0.178036922	0.962648047
Hist3h2a	0.005867357	0.99527876
Hist3h2ba	0.216497409	0.610726714
Hist3h2bb-ps	0.060182791	0.99527876
Hist4h4	-0.663732605	0.91462326
Hivep1	-0.160431713	0.881371608
Hivep2	-0.082488833	0.874273224

Hivep3	-0.1189545	0.736130784
Hjurp	-0.204604928	0.521202205
Hk1	-0.086990731	0.895198797
Hk1os	-0.759538878	0.883489696
Hk2	0.101818077	0.950195611
Hk3	-0.253082907	0.956164302
Hkdc1	0.007901087	0.996094752
Hlcs	0.168059466	0.8616433
Hlf	-0.100239098	0.910328122
Hltf	-0.078289365	0.971138085
Hlx	0.626035071	0.89703631
Hmbox1	-0.02094798	0.985673938
Hmbs	0.026974972	0.988034453
Hmces	0.097373295	0.936507368
Hmcn1	-0.254912931	0.779723523
Hmcn2	0.11012874	0.985673938
Hmg20a	-0.083578057	0.793949146
Hmg20b	0.010765523	0.99527876
Hmga1	-0.068500989	0.975800973
Hmga1b	0.160288503	0.764044481
Hmga2	-0.540474546	0.93912725
Hmgb1	0.110263134	0.731867452
Hmgb1-ps1	0.277717432	0.930336691
Hmgb1-ps2	0.103328623	0.890704638
Hmgb1-ps3	0.115923278	0.96007778
Hmgb1-ps4	0.467323836	0.91462326
Hmgb1-ps5	0.618002884	0.939260148
Hmgb1-ps6	0.642238114	0.934135315
Hmgb1-ps7	0.568429094	0.825594767
Hmgb1-ps9	0.356843993	0.935897306
Hmgb1-rs16	-0.556945424	0.97487264
Rpl21-ps15	0.367340692	0.027390747
Etl4	-0.366857142	0.459813984
Hmgcl	0.161592971	0.660403254
Hmgcll1	-0.172065745	0.841017002
Hmgcr	-0.105599114	0.618152081
Hmgcs1	-0.122385545	0.659056223
Hmgcs2	-0.251871263	0.873797321
Hmgn1	0.088878112	0.852010446
Mettl16	-0.366082016	0.141036549
Hmgn2-ps1	0.28413206	0.962648047
Hmgn3	0.109639085	0.710375942
Hmgn5	-0.09964064	0.936031628
Hmgxb3	-0.064701506	0.89703631

Hmgxb4	-0.065592169	0.962648047
Hmmr	-0.313168852	0.970348278
Hmox1	-0.001452707	0.998658264
Rps28	0.365227603	0.266643009
Hnf1b	-0.063312333	0.99527876
Trp53inp1	0.364882173	0.238827832
Hnrnpa0	0.02321367	0.971661054
Hnrnpa1	0.09364858	0.748784855
Hnrnpa1l2-ps2	-0.104809273	0.991996237
Hnrnpa2b1	0.076626901	0.833916587
Hnrnpa3	-0.009634754	0.991438757
Hnrnpab	0.089213631	0.740833211
Hnrnpc	-0.086605562	0.803910915
Hnrnpd	0.070292933	0.852010446
Hnrnpdl	0.044573063	0.956739982
Hnrnpf	0.103148532	0.91462326
Hnrnph1	-0.045340679	0.968005385
Hnrnph2	0.070756791	0.873753967
Hnrnph3	0.093850607	0.860521845
Hnrnpk	0.054287699	0.884426973
Hnrnpl	0.071708295	0.780228245
Hnrnpll	-0.027611464	0.969755848
Hnrnpm	0.017568184	0.98553345
Hnrnpr	0.019351615	0.982437522
Hnrnpu	0.008976949	0.99527876
Hnrnpul1	0.013419812	0.985742667
Hnrnpul2	-0.019620263	0.970348278
Hoga1	-0.483256981	0.674357582
Homer1	-0.602961333	0.665688648
Slco2a1	0.364774761	0.333416246
Homer3	0.08904106	0.860826773
Homez	-0.02238447	0.985742667
Hook1	0.32279084	0.911276003
Hook2	0.010835687	0.994960308
Snx22	0.364640371	0.160748539
Hopx	0.280054312	0.955993235
Hopxos	-0.086170403	0.994532146
Olfr221	1.151135946	0.911276003
Hoxa7	-0.905808462	0.962620517
Hoxc10	-0.978745289	0.968005385
AC110166.1	1.149958353	0.962094124
Cox6c2	1.148965324	0.544661714
Hp1bp3	0.037132052	0.955993235
Hpca	-0.02122605	0.98526072

Per2	-0.364071028	0.0047925
Hpcal4	-0.077659567	0.76998058
Hpdl	0.106437616	0.972218452
Hpf1	0.072307175	0.932673884
Hpgd	-0.044339644	0.981392364
Hpgds	-0.161171264	0.951817826
Hpn	0.018109871	0.99527876
Hprt	0.004622722	0.99527876
Glb1l	0.363752923	0.240832274
Hps3	-0.031922649	0.985673938
Hps4	0.037265164	0.980226283
Abi1	0.363091227	0.140151036
Hps6	-0.182971334	0.871869598
Hpse	0.422254413	0.87847909
Fam229a	1.147062903	0.962648047
Hpx	-0.92904671	0.905281292
Hr	-0.031832613	0.985742667
Hras	0.100582748	0.852157293
Hrasls	-0.22205044	0.615775357
Cuzd1	1.146787226	0.914969279
Hrct1	0.226371857	0.967881817
Hrh1	-0.369470026	0.639505683
Hrh2	-0.057506241	0.97469529
Hrh3	0.044204252	0.968005385
Hrk	0.080062774	0.91462326
Hs1bp3	-0.038490493	0.975831674
Hs2st1	-0.029649447	0.966213117
Hs3st1	-0.190890132	0.883426561
Hs3st2	-0.089493002	0.968005385
Hs3st3a1	0.417439733	0.962648047
Hs3st3b1	-0.556018426	0.891972665
Hs3st4	0.008778226	0.991438757
Hs3st5	-0.008625467	0.99765039
Hs3st6	-0.686551668	0.965007958
Hs6st1	-0.160002157	0.615948828
Hs6st2	-0.051035836	0.97469529
Hs6st3	-0.190803299	0.86783605
Crls1	0.362726879	0.431061843
Hsbp1l1	-0.287312931	0.962648047
Hscb	0.35405799	0.638858785
Hsd11b1	0.19776246	0.851875536
Hsd17b1	0.736215096	0.969755848
Hsd17b10	0.11742238	0.938615214
Hsd17b11	0.077069871	0.858549868

Hsd17b12	0.065464001	0.905767935
Mir5117	1.14672431	0.54807923
Hsd17b4	0.016037428	0.985673938
Hsd17b7	-0.12189642	0.793949146
Ttc29	1.1463422	0.884426973
Hsd3b3	0.060110398	0.99527876
Hsd3b4	-0.501728011	0.982200476
Gfy	1.145458203	0.591099262
Hsd3b7	0.086257781	0.968005385
Hsdl1	-0.068319261	0.918781935
Hsdl2	-0.10608594	0.837755777
Hsf1	0.226779002	0.690084266
Hsf2	-0.068714455	0.90358675
Tbc1d16	0.36255016	0.377143991
Col24a1	1.138308708	0.813552442
Hsf4	0.023997256	0.991996237
Hsf5	0.023504352	0.99527876
Kbtbd12	1.137316904	0.544361994
Hsp25-ps1	0.107956401	0.984328758
Hsp90aa1	-0.029647027	0.971138085
Hsp90ab1	0.057765713	0.877275726
Hsp90b1	-0.004188923	0.99527876
Hspa12a	-0.058204677	0.875056988
Hspa12b	-0.186431094	0.874252042
Hspa13	-0.127193114	0.795563654
Hspa14	-0.00325643	0.995411429
Hspa1a	-0.034742796	0.980132658
Hspa1b	-0.049715849	0.968005385
Hspa1l	0.288883635	0.627261794
Dnajc14	-0.362149084	0.31865776
Hspa4	-0.026721383	0.964941885
Hspa4l	-0.087150717	0.847678217
Hspa5	-0.366347211	0.889908105
Hspa8	-0.039020721	0.968005385
Hspa9	0.024291346	0.966010627
Hspb1	-0.599231103	0.539833449
Epha6	-0.362069754	0.09657124
Hspb2	0.640817521	0.868415875
Hspb3	0.10884263	0.99100709
Hspb6	0.232992567	0.898474424
Olfr527	1.134840046	0.968005385
Hspb8	0.223859125	0.764110268
Hspb9	0.35094695	0.981045362
Hspbap1	0.098974525	0.957499962

Hspbp1	-0.105338765	0.835714527
Hspd1	-0.021558221	0.975631569
Hspd1-ps3	0.098590389	0.911206484
Hspd1-ps4	-0.712919358	0.940431936
Pwwp2a	0.36172739	0.412816247
Hspg2	-0.283869049	0.795137348
Hsph1	-0.138673085	0.770166577
Htatip2	-0.070839012	0.968005385
Htatsf1	0.02489444	0.974962198
Htr1a	-0.033855758	0.975350978
Ndufa12	0.361305938	0.000134553
Htr1d	-0.359445391	0.980339705
Htr1f	0.306022175	0.926988689
Htr2a	-0.046662849	0.981392364
Htr2b	0.584438129	0.984328758
Htr2c	-0.161476674	0.8549794
Htr3a	-0.00846521	0.99527876
Htr4	-0.074852921	0.939553162
Htr5a	-0.079980467	0.962648047
Htr5b	-0.156355749	0.918407269
Htr6	-0.316152753	0.744309911
Htr7	-0.437615581	0.807481321
Pcdhb14	-0.361163436	0.399438388
Htra2	0.109935193	0.910328122
Htra3	-0.239464309	0.865547585
Htra4	0.344143816	0.816844103
Uqcrh	0.360685277	0.02035027
Hunk	-0.455558126	0.825148733
Hus1	-0.031816618	0.980339705
Snhg1	0.360667064	0.159596426
Hvcn1	0.017638683	0.99527876
Tmem45a2	1.134840046	0.968005385
Hyal2	0.039999407	0.989840188
Hyal3	0.62989786	0.890704638
Hydin	-0.660692952	0.621568634
Hyi	-0.121379402	0.965520236
Hykk	-0.235274071	0.76034043
Hyls1	0.051430033	0.975831674
Hyou1	0.255707368	0.707777756
Hypk	0.197530917	0.618152081
Iah1	0.118426745	0.935890893
Iars	-0.118815498	0.736130784
Iars2	-0.056149791	0.920642026
Iba57	0.094912796	0.953505913

Ibsp	-0.448514734	0.974809035
Ibtk	0.104917184	0.932673884
Ica1	0.040523707	0.972025261
Ica1l	-0.174394676	0.909243078
Icam1	-0.626485534	0.724910334
Icam2	-0.369903251	0.89383516
Icam4	0.725906147	0.648241111
Icam5	-0.042207017	0.942448508
Man1a	-0.360666583	0.213895263
Ice2	0.036158371	0.990563704
Ick	-0.109891414	0.905251009
Icmt	-0.032602626	0.99527876
Icos	-0.310917641	0.965520236
Icosl	0.047945457	0.965520236
Ict1os	-0.23480923	0.904696184
Id1	0.245552728	0.813552442
Ccdc88c	-0.360267114	0.004094616
Id3	-0.092482186	0.949607377
Id4	0.097287177	0.778269738
Ide	-0.095235896	0.757721895
Idh1	-0.012058027	0.987260085
Prdm5	0.359867586	0.509250303
Idh3a	-0.005269811	0.99527876
Idh3b	0.054265249	0.911631946
Idh3g	0.084209441	0.869316117
Idi1	0.010578223	0.993277997
Idnk	0.034577321	0.974823941
Ido1	-0.582231404	0.975831674
Ido2	-0.258178465	0.865075531
Ids	-0.031916736	0.944753667
Idua	0.352457646	0.76034043
Ier2	-0.059724022	0.9813031
Ier3	-0.441501014	0.5141747
Ier3ip1	0.206548799	0.890553353
Ier5	0.032351202	0.974823941
Ier5l	-0.241599224	0.873797321
Iffo1	0.004502543	0.99527876
Pde6d	0.359582642	0.445106721
Ifi203	-0.25725375	0.928672524
Ifi203-ps	-0.155834245	0.987342614
Ifi204	0.180786728	0.993103295
Pnpla2	0.359295208	0.193510645
Ifi207	-0.712860181	0.882096017
Arhgef4	-0.358516757	0.314194702

Ifi211	-0.736153044	0.936031628
Ifi27	0.015317571	0.99165967
Ifi27l2a	0.973204747	0.609167039
Ifi30	-0.269685906	0.862094492
Ifi35	0.049558517	0.985673938
Tcp11l1	-0.357569179	0.327338387
Ifi47	0.377392151	0.939260148
Ifih1	-0.235726323	0.890136672
Ifit1	0.047913003	0.994960308
Ifit1bl1	0.094497409	0.97487264
Ifit1bl2	0.159196834	0.980226283
Ifit2	-0.856297181	0.894430149
Ifit3	-0.180177023	0.911631946
Ifit3b	0.014269038	0.99527876
Ifitm1	-0.24760106	0.899930239
Ifitm10	-0.045482363	0.971661054
Ifitm2	-0.130347483	0.959276014
Ifitm3	-0.268923986	0.84870301
Ifitm6	-0.033468632	0.995886687
Ifitm7	0.020629845	0.99527876
Ifnar1	-0.012652887	0.99527876
Ifnar2	-0.049834143	0.964941885
Ifngr1	0.304379318	0.899271839
Ifngr2	-0.119609125	0.624713015
Ifnlr1	0.509669534	0.932673884
Ifrd1	0.19175929	0.51889485
Ifrd2	-0.024992571	0.991438757
Ift122	-0.108752368	0.708626104
Ift140	0.043659392	0.965520236
Ift172	-0.008264415	0.994960308
Ift20	0.08362293	0.864907053
Ift22	0.236953234	0.803910915
Ift27	0.189120726	0.642397758
Ift43	0.165872521	0.91907636
Ift46	0.014969261	0.986246396
Ift52	-0.011786257	0.993103295
Ift57	0.06919257	0.91462326
Ift74	-0.023929744	0.985742667
Ift80	-0.071448958	0.929133542
Ift81	0.03996645	0.965520236
Ift88	0.031904625	0.976978183
Ift88os	-0.143246623	0.991377399
Igbp1	0.124971769	0.839488666
Igbp1b	0.061343064	0.991438757

Igdcc3	0.070804341	0.980626239
Igdcc4	-0.11185913	0.84277074
Igf1	-0.054424031	0.980226283
Igf1r	-0.102599077	0.787308413
Igf2	0.049771743	0.982309415
Cdk19os	1.126298935	0.90458554
Igf2bp2	0.276845765	0.97487264
Igf2bp3	0.023238499	0.99527876
Igf2os	0.620310928	0.865931221
Igf2r	-0.056278438	0.950195611
Igfals	0.351973786	0.989378233
Igfbp1	-0.952289644	0.962648047
Igfbp2	-0.087501157	0.951901417
Igfbp3	0.176452236	0.760547704
Igfbp4	-0.040368642	0.970461999
Igfbp5	0.106626984	0.909547396
Igfbp6	-0.071261049	0.973100313
Igfbp7	0.081995741	0.945183336
Trim6	1.124020943	0.962648047
Igflr1	0.529089055	0.972218452
Mir5621	1.123880259	NA
Igha	0.116761509	0.99527876
Ighd	-0.278763219	0.968977192
Large2	1.123842055	0.820614009
Ighj1	0.09269461	0.99527876
Ighj4	0.655783733	NA
Ighm	-0.138534552	0.920642026
Ighmbp2	0.045742515	0.975831674
Igip	-0.133461047	0.811913793
Igkc	-0.607451012	0.919785445
Iglc3	0.099776521	0.99527876
Iglon5	0.044274848	0.962648047
Igsf1	0.220321921	0.795137348
Igsf10	0.360975115	0.730301028
Igsf11	-0.068409716	0.911631946
Igsf21	0.07174561	0.928672524
Igsf3	-0.044986706	0.974823941
Igsf5	-0.174931246	0.984836179
Igsf6	-0.118275426	0.980419928
Igsf8	-0.129722516	0.971484753
Igsf9	0.358066456	0.922353876
Adipor2	0.357539605	0.103621073
Igtp	-0.241339878	0.882824434
Gpr82	1.12344163	0.936031628

Iigp1	0.109882734	0.980132658
Ptgfrn	0.357516931	0.403453477
Ikbip	-0.030533586	0.98606288
Ikbkb	-0.085367402	0.8226891
Ikbke	-0.008929087	0.997375151
Ikbkg	-0.257003808	0.841017002
Ikzf1	0.388280823	0.729048067
Ikzf2	-0.024590231	0.993103295
Ikzf4	-0.004177285	0.99765039
Rps21	0.356050027	0.159596426
Mertk	0.356034942	0.005424982
Il10rb	-0.144293818	0.89703631
Il11	0.196920473	0.985446046
Il11ra1	0.084571842	0.924361537
Il11ra2	0.001937934	0.999486844
Il12a	0.447954645	0.932673884
Il12rb1	0.496728796	0.935897306
Il12rb2	0.034536316	0.99527876
Il13ra1	-0.154198538	0.962648047
Il15	-0.377863877	0.908554604
Il15ra	0.336286126	0.737087313
A2ml1	0.355387058	0.442068044
Il17b	0.564643259	0.966010627
Il17d	0.065423076	0.976257618
Il17ra	0.018286621	0.990697821
Il17rb	-0.048446602	0.992774037
Il17rc	0.338837486	0.770793864
Il17rd	-0.013978379	0.99527876
Il17re	0.387102521	0.968005385
Il18	0.097664096	0.920675565
Il18bp	0.055829671	0.976546786
Il18rap	-0.548830734	0.968005385
Il1a	-0.362850116	0.962648047
Il1b	-0.924801965	0.91462326
Il1bos	0.998250124	0.90358675
Il1r1	0.196338579	0.854278008
Il1r2	-0.177451652	0.984232465
Il1rap	0.071193856	0.91462326
Il1rapl1	-0.324761653	0.609167039
Il1rapl2	0.090333549	0.981746088
Il1rl1	-0.809438738	0.873797321
Il1rl2	-0.056573434	0.99527876
Il1rn	-0.599165925	0.829694131
Il20rb	0.476278081	0.638329646

Il21	0.34859701	0.98175286
Il21r	-0.105029462	0.974823941
Il23a	0.49578127	0.98526072
Il27ra	-0.354867874	0.849526037
Osbpl8	-0.355208006	0.037253581
Il2rb	0.493440095	0.939592821
Il2rg	-0.488745126	0.911631946
Tcfl5	1.122205161	0.890792346
Il33	0.033131477	0.97500491
Il34	-0.214684899	0.732729469
Il3ra	0.047885405	0.991438757
Il4	-0.074226181	0.971661054
Il4i1	-0.716361824	0.953505913
Il4ra	-0.391096726	0.710937984
AC126254.1	1.120888753	0.911631946
Il6	0.001157444	0.999724849
Il6ra	0.179531542	0.890692547
Il6st	-0.187114453	0.889972515
Rps15a-ps4	1.118839621	0.70724751
Il7r	0.297824045	0.962648047
Ildr1	0.68728782	0.964941885
Ildr2	-0.059777762	0.920642026
Ilf2	0.08701797	0.718702264
Ilf3	0.048634912	0.956164302
Tceal3	0.354738896	0.151118156
Ilkap	0.095193762	0.854278008
Ilvbl	0.097759496	0.936031628
Immp1l	-0.071750709	0.942812446
Immp2l	-0.125415731	0.965520236
Immt	0.092390406	0.861378744
Naa25	-0.354036233	0.41351939
Imp4	-0.106783333	0.895198797
Impa1	0.04304675	0.962648047
Impa2	-0.119464812	0.970461999
Impact	-0.023227774	0.968005385
Impad1	-0.06116321	0.921117568
Impdh1	0.005678325	0.997115148
Impdh2	0.027550082	0.97487264
Impdh2-ps	0.199910157	0.600999414
Il2ra	1.116235086	0.798321484
Ina	-0.077224003	0.860521845
Inafm1	0.083374082	0.94999152
Inafm2	0.10685192	0.775455132
Inava	0.041688435	0.988034453

Inca1	0.119056483	0.962094124
Incenp	-0.042197773	0.972025261
Inf2	-0.056477772	0.958389182
Ing1	0.128585186	0.958085726
Ing2	-0.078938656	0.908554604
Ing3	-0.119582823	0.828102037
Ing4	-0.002083073	0.997625755
Ing5	0.093314598	0.887135194
Inha	0.025100143	0.989200732
Inhba	-0.419394798	0.679604282
Thbs4	-0.353803905	0.228067551
Inip	-0.097009417	0.864150749
Inka1	-0.173519979	0.871869598
Inka2	-0.117773855	0.640856315
Inmt	0.148557114	0.963666931
Ino80	-0.095106643	0.857611652
Ino80b	0.271886819	0.801926713
Ino80c	0.138101932	0.771684575
Ino80d	-0.08428624	0.883131585
Ino80dos	0.055286946	0.98250278
Ino80e	0.123464958	0.842129981
Inpp1	-0.034591103	0.968005385
H2-M6-ps	1.115833675	0.780228245
Inpp4b	0.123257651	0.949607377
Inpp5a	0.093042739	0.889650776
Inpp5b	-0.012096979	0.991438757
Inpp5d	0.17379555	0.727640208
Inpp5e	-0.120823036	0.881407916
Inpp5f	-0.054873918	0.903441375
Inpp5j	0.028732051	0.97487264
Inpp5k	0.011094046	0.991996237
Inppl1	-0.168185564	0.700063392
Epb41l3	-0.352063695	0.423074171
Insig1	0.070319351	0.889908105
Insig2	0.139274424	0.779346076
Insl5	-0.687379396	0.867827234
Insl6	-0.605284952	0.959659722
Insm1	-0.184236853	0.897878088
Insm2	-0.048914068	0.99527876
Insr	-0.086476342	0.936031628
Rps12-ps10	1.11577789	0.946985353
Insyn1	0.032542827	0.962648047
Ints1	-0.012564407	0.989840188
Ints10	-0.091338994	0.873797321

Ints11	-0.121812773	0.87491981
Ints12	-0.032567023	0.984328758
Ints13	0.030272783	0.98043358
Ints14	0.016478335	0.987342614
Ints2	-0.064150255	0.942812446
Ints3	-0.090922807	0.710375942
Ints4	-0.136671937	0.858393367
Ints5	-0.194700886	0.533008784
Snrnp25	0.351854842	0.082752362
Ints6l	-0.026973533	0.981392364
Ints7	-0.223622418	0.83886846
Klhl26	0.351478181	0.057309283
Ints9	-0.070648454	0.947033215
Intu	-0.164691021	0.858106541
Invs	-0.106447406	0.873753967
Ip6k1	0.056430566	0.885128793
Slc40a1	-0.350602037	0.420373478
Fabp4	1.112806316	0.559262988
Ipcef1	0.024512864	0.986864921
Ipmk	-0.110172834	0.631994664
Ipo11	-0.071939352	0.969755848
Ipo13	-0.03506962	0.968005385
Ipo4	0.248643241	0.938414123
Ipo5	0.010450765	0.985673938
Ipo7	-0.109470537	0.810908327
Ipo8	-0.044019783	0.962648047
Ipo9	0.05169261	0.910328122
Ipp	0.15502386	0.837382879
Ippk	0.162514953	0.69189201
Iqca	-0.221392566	0.951817826
Iqcb1	0.044098471	0.968005385
Iqcc	-0.117986183	0.8643613
Iqcd	-0.105220273	0.969755848
Iqce	-0.116054859	0.854278008
Iqcg	0.322984666	0.781249046
Iqch	0.372072853	0.966010627
Iqcj	0.531261891	0.956164302
Iqck	0.146685858	0.909243078
Iqcm	0.202815971	0.987342614
Iqgap1	-0.229283668	0.571438091
Iqgap2	-0.164362195	0.604176395
Iqgap3	0.494937733	0.931745318
Iqschfp	0.031085458	0.994960308
Iqsec1	-0.076809652	0.875119971

Prkd3	-0.350561118	0.437951149
Iqsec3	0.017574404	0.98459388
Iqub	0.254652789	0.881185267
Irak1	0.004249625	0.997529192
Irak1bp1	0.091639984	0.921618725
Irak2	0.114999013	0.968005385
Irak3	-0.566370395	0.894953269
Irak4	-0.064783131	0.984836179
Ireb2	-0.10493141	0.786324313
Irf1	0.012329988	0.99527876
Irf2	-0.108742847	0.889972515
Irf2bp1	0.013513609	0.990608029
Irf2bp2	0.116993632	0.861366392
Irf2bpl	-0.01086829	0.993380259
Irf3	0.069159632	0.962648047
Irf4	0.053161004	0.99527876
Irf5	0.016773455	0.99527876
Irf6	-0.578405112	0.888622599
Irf7	-0.75420693	0.66923652
Irf8	-0.052722753	0.981683975
Irf9	-0.092248537	0.932673884
Irgm1	-0.072851977	0.968005385
Irgm2	-0.055876089	0.985477412
Irgq	-0.069915408	0.89703631
Irs1	-0.050793068	0.966010627
Irs2	0.140397096	0.65012378
Fibcd1	-0.349950014	0.124108735
Paqr8	1.111052459	0.604176395
Irx1	-0.155563472	0.985673938
Irx2	-0.450508511	0.985742667
Rspo3	-0.349907812	0.323832374
Rpl21-ps11	1.106297384	0.527794911
Isca1	0.055549068	0.895520088
Isca2	-0.043828529	0.975831674
Iscu	-0.036227813	0.981392364
Isg15	-0.225655225	0.962648047
Isg20	0.361683462	0.907829139
Isg20l2	-0.028256822	0.985048364
Islr	-0.237506469	0.928672524
Islr2	0.135670294	0.754747571
Ism1	0.087726524	0.950046478
Fgr	1.103361019	0.903279667
Isoc1	0.009117607	0.99527876
Isoc2a	0.099002232	0.91462326

Isoc2b	0.073278005	0.969755848
Ispd	0.11646847	0.948432875
Ist1	0.069965555	0.928716457
Isy1	-0.011765089	0.989200732
Isyna1	-0.200809428	0.77156461
Itch	-0.101361478	0.717156625
Itfg1	-0.0431025	0.930250446
Itfg2	0.126393927	0.935897306
Itga1	0.123414189	0.939052665
Itga10	-0.450192699	0.861366392
Itga11	0.280813817	0.786324313
Itga2	0.395151224	0.962648047
Itga2b	0.205700887	0.946985353
Itga3	0.0957824	0.923011738
Itga4	-0.107161592	0.713014362
Itga5	-0.407845165	0.858549868
Itga6	-0.129887776	0.854278008
Itga7	-0.033881493	0.978384252
Itga8	-0.006035025	0.99527876
Itga9	-0.423064207	0.860521845
Itgad	0.744940731	0.723108545
Itgae	-0.470196766	0.95126064
Itgal	0.54045313	0.968005385
Itgam	-0.186755059	0.517996718
Itgav	0.413500123	0.950460219
Itgax	0.822063983	0.911631946
Itgb1	-0.043810817	0.965520236
Gria3	-0.349767804	0.151118156
AF357425	1.102732294	NA
Itgb2	-0.108951464	0.962648047
Itgb3	0.167270137	0.938394547
Itgb3bp	0.032682061	0.985742667
Itgb4	-0.182618137	0.890704638
Itgb5	0.046670223	0.950195611
Itgb7	-0.12409158	0.98459388
Itgb8	-0.1124676	0.89734717
Itgbl1	-0.204447438	0.832383921
Itih2	-0.968653653	0.891703152
Itih3	0.010110517	0.99527876
Itih4	0.004001227	0.999242708
Itih5	-0.0381071	0.981502501
Itk	0.282098497	0.944040391
Itm2a	0.043593823	0.971661054
Itm2b	-0.06238549	0.873797321

Itm2c	0.031692021	0.961887657
Itpa	0.025677714	0.975831674
Itpa-ps1	-0.068983576	0.975831674
Itpa-ps2	-0.973537991	NA
Itpk1	0.12006015	0.752941962
Itpka	-0.097448124	0.852010446
Itpkb	0.091900314	0.84870301
Itpkc	-0.053475423	0.97469529
Lgals9	0.349229735	0.34409789
Itpr2	-0.101758831	0.851875536
Itpr3	-0.051374006	0.98526072
Itprid1	-0.441547901	0.707777756
Itprid2	0.028366351	0.975831674
Itpripl1	-0.003440372	0.998840551
Itpripl2	-0.135400183	0.948104382
Itsn1	-0.017828645	0.98340069
Itsn2	-0.042267162	0.959659722
Ivd	-0.266553308	0.911631946
Ivns1abp	-0.004273725	0.99527876
Iws1	-0.025534258	0.980896205
Iyd	0.206284864	0.795137348
Izumo1	0.417326754	0.972218452
Pcdhac2	-0.349151045	0.004849187
Jade1	0.068051888	0.975831674
Jade2	-0.06981153	0.867827234
Jade3	0.127315644	0.965290366
Jag1	0.046931474	0.974962198
Jag2	-0.0373954	0.975831674
Jagn1	0.068119119	0.921311891
Jak1	-0.032498683	0.962648047
Jak2	-0.108396248	0.7851601
Jak3	-0.231986968	0.872352604
Otulin	0.348415746	0.34587585
Jakmip2	-0.069377457	0.929997774
Jakmip3	-0.129129078	0.810246066
Jam2	-0.563695937	0.89703631
Jam3	-0.119593162	0.795137348
Exosc2	0.347432428	0.302984782
Jarid2	-0.051140509	0.976257618
Jazf1	0.022671017	0.982392834
Jcad	-0.129623615	0.611658468
Jchain	0.301670722	0.934798735
Jdp2	0.091096255	0.916961045
Jhy	0.707787653	0.962648047

Jkamp	0.020591834	0.985742667
Jmjd1c	-0.238734273	0.624713015
Jmjd4	-0.806231661	0.544661714
Jmjd6	0.038833618	0.971661054
Jmjd7	0.306630988	0.811777777
Jmjd8	0.170442439	0.837755777
Jmy	-0.026381319	0.969755848
Josd1	-0.042010999	0.942274311
Josd2	0.032416247	0.975831674
Jph1	0.008676877	0.99527876
Mir1943	1.100570912	0.942812446
Jph3	-0.050480455	0.934798735
Jph4	-0.090984148	0.782639526
Jpt1	0.020686624	0.985742667
Jpt2	0.021612847	0.991996237
Jpx	0.473577848	0.510932065
Jrk	0.006520816	0.996617271
Jrkl	-0.025786715	0.985742667
Jsrp1	0.328450819	0.881771584
Jtb	0.092062254	0.955706294
Jun	0.217434272	0.621993647
Tmem200a	-0.346962754	0.055691244
Jund	-0.020065836	0.980896205
Junos	0.13140009	0.976147937
Jup	-0.169819642	0.680804075
Kalrn	-0.133783409	0.544361994
Flrt2	-0.346659912	0.082752362
Kank2	-0.105948425	0.956737477
Kank3	0.045053208	0.970348278
Kank4	-0.40972588	0.519524068
Kansl1	-0.013137478	0.987326705
Kansl1l	-0.000951645	0.998658264
Rpl17-ps10	0.346525341	0.000657163
Kansl2-ps	0.034729462	0.978384252
Kansl3	-0.068366086	0.859989558
Kantr	-0.092751071	0.883351387
Onecut2	-0.345805438	0.431269992
Kat14	0.302060591	0.760547704
Kat2a	-0.009165077	0.991541583
Kat2b	0.026899289	0.985742667
Kat2b-ps	-0.582342373	0.981392364
Kat5	0.080479588	0.874891526
Snrnp35	0.345796472	0.378223206
Rpl36	0.345053513	0.034088092

Kat7	0.041869443	0.950195611
Kat8	-0.059965768	0.962648047
Katna1	-0.118572699	0.859910936
Katnal1	-0.099832336	0.751630609
Katnal2	-0.076416794	0.962648047
Katnb1	-0.126699315	0.657504489
Katnbl1	0.038743438	0.975831674
Kazald1	0.176988316	0.948104382
Kazn	0.006841631	0.99527876
Cyb5r1	0.34483022	0.19334431
Slamf1	1.097333334	0.962648047
Slc25a48	1.097267525	0.875723186
Kbtbd2	-0.015838715	0.987150769
Kbtbd3	0.155802081	0.908413609
Kbtbd4	0.053306016	0.970348278
Kbtbd6	0.128201215	0.962648047
Kbtbd7	-0.074070108	0.889972515
Kbtbd8	0.048048121	0.976147937
Kcmf1	0.096647417	0.875995577
Kcna1	-0.082860288	0.929618757
Kcna10	-0.089098797	0.99527876
Kcna2	-0.3079879	0.901479608
Kcna3	-0.591443606	0.917685272
Kcna4	-0.042774428	0.976978183
Ndufs6	0.34394393	0.212635473
Kcna6	-0.121771599	0.844084751
Snord17	1.096798522	0.971661054
Chmp1b	-0.34379884	0.067353494
Kcnab2	0.034808361	0.951817826
Kcnab3	-0.01999165	0.988034453
Kcnab3os	0.004363628	0.999227272
Kcnb1	-0.119973582	0.770166577
Kcnb2	-0.189555334	0.526260058
Kcnc1	-0.003492701	0.99527876
Kcnc2	-0.127945121	0.54807923
Kcnc3	0.038880176	0.99527876
Kcnc4	0.203460551	0.596982103
Kcnd1	-0.017537723	0.990835405
Atp11a	-0.343584567	0.445365777
Kcnd3	0.008586712	0.99527876
Kcnd3os	0.190162353	0.962648047
Kcne1l	-0.048747015	0.985742667
Kcne2	-0.053575521	0.99527876
Kcne3	-0.959604492	0.89703631

Kcne4	-0.1017016	0.98340069
Kcnf1	-0.178337281	0.703372057
Kcng1	0.004232446	0.997258205
Kcng2	0.143831979	0.813346233
Tsix	1.092960861	0.951817826
Kcng4	0.135302507	0.92380088
Kcnh1	0.164841846	0.961887657
Kcnh2	-0.072875024	0.932673884
Kcnh3	-0.033986271	0.965520236
Kcnh4	-0.236697635	0.936031628
Ctnnd2	-0.342854978	1.9702E-05
Kcnh6	0.13322795	0.968005385
Kcnh7	-0.092179654	0.956164302
Kcnh8	0.483682114	0.962648047
Kcnip1	-0.115874873	0.84870301
Kcnip2	0.022879934	0.97469529
Kcnip3	0.025390739	0.972374093
Kcnip4	-0.278582034	0.948104382
Kcnj10	-0.025400825	0.974823941
Kcnj11	0.104793697	0.832980941
Kcnj12	-0.403921116	0.615540363
Slc28a2	1.091476406	0.95494593
Kcnj14	-0.00945514	0.99618421
Kcnj16	-0.302957905	0.962648047
Kcnj2	0.285871981	0.857344087
AI413582	0.342195335	0.367929038
Kcnj4	0.061773664	0.936031628
Kcnj5	0.237275852	0.968005385
Kcnj6	-0.177162922	0.52001079
Kcnj8	0.274485964	0.889972515
Kcnj9	0.016101419	0.989840188
Dyrk2	-0.341938201	0.179582907
Kcnk10	-0.135090409	0.907829139
Kcnk12	0.053530607	0.981502501
Kcnk13	-0.184166165	0.866104092
Kcnk2	-0.045297954	0.971661054
Kcnk3	-0.104390449	0.944494911
Kcnk4	-0.147955909	0.875056988
Kcnk5	-0.955725466	0.872612157
Kcnk6	0.164197921	0.922100633
Mir6418	1.091274136	0.92517502
Kcnk9	0.013217389	0.991996237
Kcnma1	-0.111892366	0.629280869
Kcnmb1	-0.15669972	0.962648047

Kcnmb2	0.248611399	0.860826773
Kcnmb3	-0.849436628	0.975831674
Kcnmb4	0.092129619	0.803910915
Kcnmb4os1	0.098606417	0.992385525
Kcnmb4os2	0.134674433	0.975831674
Kcnn1	0.09370678	0.959276014
Kcnn2	0.025427274	0.975831674
Kcnn3	-0.067835343	0.993380259
Kcnn4	-0.061433304	0.99527876
Kcnq1	0.29746824	0.965752549
Kcnq1ot1	0.750563325	0.962648047
Sez6	-0.341138748	0.091758274
Phldb1	-0.341043993	0.409844299
Kcnq4	0.010756595	0.99527876
Kcnq5	-0.003312441	0.99527876
Kcnrg	-0.424437114	0.889972515
Sgf29	0.340927193	0.360222394
Kcns2	-0.088382153	0.89703631
Kcns3	-0.148563847	0.815987743
Kcnt1	-0.054103433	0.97487264
Kcnt2	-0.083857612	0.949607377
Kcnu1	0.396981823	0.566580926
Kcnv1	-0.07674183	0.928672524
Mpig6b	1.091211119	0.96905593
Kcp	-0.155773417	0.959276014
Kctd1	-0.191424479	0.668637933
Kctd10	-0.126599192	0.599763688
Kctd11	0.178565613	0.962648047
Kctd12	-0.12309879	0.799736155
Kctd12b	-0.26050075	0.879401677
Kctd13	0.057094534	0.89703631
Kctd14	0.30214345	0.852010446
Kctd15	-0.11989271	0.869316117
Kctd16	-0.340802627	0.655744783
Kctd17	0.007180915	0.993489613
Kctd18	0.10919152	0.829222584
Kctd2	0.042139954	0.942568828
Kctd20	-0.190174015	0.962636653
Pus10	-0.340534057	0.389731767
Tuba8	0.339884786	0.224950217
Kctd4	0.209569354	0.510932065
Kctd5	0.162944565	0.701308571
Kctd6	0.044493891	0.97487264
Kctd7	-0.206014309	0.603499717

Kctd8	-0.081825261	0.965520236
Kctd9	0.020033724	0.99527876
Kdelc1	-0.099836506	0.932673884
Kdelc2	0.02254276	0.991996237
Kdelr1	0.116805261	0.886647768
Kdelr2	0.02359261	0.985735386
Kdelr3	-0.877937736	0.742849809
Kdf1	-0.149814815	0.994120754
Kdm1a	0.003397309	0.995773227
Kdm1b	0.115287123	0.902647137
Zfp329	-0.339824186	0.080124665
Kdm2b	0.11376769	0.853090538
Kdm3a	-0.161565965	0.694560824
Kdm3b	-0.14354313	0.519524068
Klhl34	-0.339815735	0.064044233
Kdm4b	0.037269457	0.965520236
Kdm4c	-0.109788139	0.7851601
Kdm4d	-0.14546163	0.970892908
Kdm4dl	-0.038147842	0.99527876
Kdm5a	-0.107238233	0.744046401
Kif16b	0.339560081	0.31865776
Kdm5c	-0.05966743	0.970348278
Kdm5d	-0.18717781	0.740833211
Kdm6a	-0.163343647	0.629280869
Kdm6b	-0.107630529	0.84277074
Kdm6bos	-0.080139517	0.993103295
Kdm7a	-0.168632828	0.580757434
Kdm8	0.14192133	0.944391691
Kdr	-0.341373316	0.550301004
Kdsr	0.016489136	0.98553345
Keap1	-0.031282403	0.981502501
Adam23	-0.339309181	0.037253581
Khdc1c	-0.538224597	0.985742667
Khdc3	-0.881051086	0.538077796
Khdc4	0.033889204	0.97487264
Khdrbs1	0.078507029	0.798580668
Khdrbs2	-0.011887521	0.99527876
Khdrbs3	-0.109577807	0.869316117
Khk	0.181233543	0.791185365
Khnyn	-0.054703644	0.958456122
Khsrp	-0.066806769	0.87847909
Kidins220	-0.03681041	0.981746088
Kif11	0.088069084	0.980339705
Kif13a	-0.097313958	0.89703631

Kif13b	-0.07652633	0.932931799
Kif14	-0.93988493	0.65012378
Kif15	-0.42732483	0.882096017
Szt2	-0.339083177	0.173273246
Kif17	-0.071539807	0.938615214
Kif18a	0.352616193	0.938673121
Kif18b	0.170120699	0.985742667
Kif19a	-0.077258075	0.974823941
Kif1a	-0.041118434	0.965290366
Kif1b	-0.000553176	0.998840551
Cbfa2t3	-0.338253687	0.212230812
Kif1c	0.046074125	0.911631946
Kif20a	-0.290401622	0.895520088
Kif20b	-0.114137886	0.988034453
Kif21a	-0.124198179	0.510932065
Rpl37rt	0.337749419	0.225616139
Kif22	0.060909424	0.984232465
Kif23	0.41106602	0.936031628
Kif24	0.382446683	0.945647427
Kif26a	-0.246272133	0.782136004
Ccdc58	0.335130146	0.31865776
Kif27	-0.272019582	0.86783605
Kif28	-0.063592499	0.985742667
Kif2a	-0.051225723	0.944753667
Kif2c	0.059892766	0.994960308
Kif3a	-0.109740314	0.721943953
Kif3b	-0.022059858	0.971138085
Btg2	0.334817459	0.310553098
Kif4	0.230415556	0.950252558
Kif5a	-0.040000938	0.965290366
Kif5b	-0.076530667	0.852035098
Kif5c	-0.063891619	0.849517436
Map3k13	-0.33444697	0.065698296
Kif7	0.07792974	0.975831674
Kif9	0.136255336	0.952034929
Kifap3	-0.082226693	0.803910915
Kifc1	-0.154127213	0.962648047
Kifc2	-0.008635518	0.988034453
Kifc3	-0.145770762	0.56399886
Kifc5b	0.220069827	0.91221238
Kin	-0.149503251	0.895520088
Kirrel	0.057565857	0.974823941
Snx21	-0.334342941	0.105803602
Kirrel3	0.06679981	0.973100313

Kirrel3os	0.882883436	0.948104382
Kiss1r	0.099663806	0.974823941
Kit	-0.135484589	0.74463793
Kitl	0.015795234	0.994960308
Kiz	-0.073009882	0.958389182
Kl	0.028064703	0.975831674
Klb	0.031772501	0.99527876
Klc1	0.036426515	0.940743591
Klc2	0.044373664	0.971661054
Klc3	0.222901944	0.934798735
Klc4	-0.08309222	0.910328122
Klf1	0.614870867	0.975800973
Klf10	-0.254981262	0.707069842
Klf11	0.050547231	0.984328758
Klf12	-0.140949113	0.872265039
Ptbp1	0.334333169	0.134709123
Klf14	0.651602921	0.948407374
Dcun1d3	-0.33390872	0.323832374
Klf16	-0.066878925	0.936031628
Klf2	0.209150664	0.837755777
Cacna2d1	-0.333775162	0.09162748
Klf4	0.035852885	0.985742667
Klf5	0.386554033	0.529257466
Klf6	-0.035106055	0.972387414
Klf7	-0.147851609	0.529257466
Klf8	-0.187635785	0.929997774
Klf9	0.062214504	0.895164489
Klhdc1	-0.145787343	0.813346233
Klhdc10	-0.007590437	0.993489613
Klhdc2	0.118510427	0.56399886
Klhdc3	0.043072149	0.932673884
Klhdc4	0.219116047	0.736367995
Tprn	0.333761625	0.314194702
Hist1h1d	1.089680714	0.911416083
Klhdc8a	0.07715826	0.979485839
Klhdc8b	0.010150497	0.99527876
Klhdc9	0.309113123	0.710375942
Klhl1	0.149133313	0.962648047
Klhl10	0.036523789	0.99527876
Klhl11	-0.283202501	0.558118096
Klhl12	0.099122141	0.69189201
Rpl31-ps8	0.33297273	0.018059921
Klhl14	-0.228433207	0.951390592
Klhl15	-0.12560708	0.881454827

Klhl18	-0.048651385	0.965520236
Klhl2	0.08077044	0.788592804
Klhl20	0.003759028	0.99765039
Klhl21	-0.061541839	0.960201673
Klhl22	-0.004639657	0.99527876
Klhl23	0.038184228	0.950770028
Klhl24	-0.012743791	0.985742667
Bex1	0.33267838	0.153854743
Zc2hc1a	-0.331963731	0.481098488
Klhl28	-0.182847826	0.629280869
Klhl29	0.072485127	0.850171696
Klhl3	-0.069664712	0.911631946
Klhl30	0.850494192	0.971661054
Mir1668	1.086376524	0.957849305
Klhl32	-0.203571892	0.56399886
Klhl33	0.195675392	0.894953269
Slc25a20	0.331816284	0.157002576
Klhl35	-0.600509803	0.815987743
Klhl36	0.332864111	0.663393433
Klhl38	-0.726297125	0.965520236
Klhl4	-0.187785871	0.775426591
Nlk	-0.331655094	0.146214787
Pkd1l2	1.085690908	0.965520236
Klhl42	-0.044756982	0.962648047
Klhl5	0.02867245	0.970461999
Klhl6	0.080610032	0.980226283
Klhl7	-0.087337388	0.74428322
Atat1	0.331580973	0.031985521
Klhl9	-0.077131061	0.804540244
Klk10	-0.30966915	0.911631946
Klk11	-0.362036685	0.976147937
Eldr	1.085147449	0.901260561
Klk13	-0.34192466	0.889908105
Klk14	-0.50822923	0.965520236
Mir194-1	1.084913433	NA
Klk2-ps	-0.382898021	0.962648047
Washc3	0.33132911	0.318279733
Klk6	0.471404783	0.887625873
Klk7	-0.332857487	0.935897306
Klk8	-0.121480698	0.921311891
Klk9	0.067091698	0.993103295
Klkb1	0.062656401	0.99527876
Avil	1.082055559	0.737369978
Klrb1a	0.282376565	0.993242999

Cx3cl1	1.079891564	0.852010446
Mir6931	1.079164802	0.940439828
Klrg1	-0.69677176	0.80931022
Klrg2	0.189114272	0.984748462
Kmo	0.405665345	0.98526072
Kmt2a	-0.595266482	0.873797321
Kmt2b	0.040185932	0.970461999
Kmt2c	-0.17302529	0.626162306
Kmt2d	-0.308332348	0.929095022
Kmt2e	-0.180422006	0.658829956
Kmt5a	-0.395548678	0.87031025
Junb	-0.330759558	0.244654485
Kmt5c	0.120880909	0.91462326
Mir5100	1.078693682	0.962648047
Kndc1	0.027602827	0.981045362
CT033750.3	1.078524265	0.969755848
Knop1	-0.178639303	0.925337971
Knstrn	-0.803328648	0.66923652
Kntc1	-0.193062083	0.947745582
Kpna1	0.42426473	0.961583757
Kpna2	-0.037627347	0.97469529
Kpna3	-0.028193562	0.962648047
Kpna4	-0.067231483	0.890704638
Kpna6	-0.081666974	0.815987743
Kpnb1	0.017531844	0.975831674
Kptn	-0.011748566	0.99527876
Kras	-0.077584423	0.889972515
Krba1	-0.081280271	0.924361537
Krcc1	0.732399748	0.841627966
Kremen1	-0.105867999	0.884426973
Kremen2	0.064064795	0.99527876
Kri1	-0.005662072	0.99527876
Krit1	-0.199016979	0.567296227
Krr1	0.279618952	0.813499809
Krt1	0.115382547	0.962648047
Krt10	0.273043387	0.751863834
Krt12	-0.017017416	0.99527876
Krt15	0.485583897	0.928957615
Krt18	0.005271035	0.99900438
Krt2	0.380386386	0.665532517
Krt20	0.212737259	0.962648047
Krt222	0.013152045	0.987342614
Tmem64	-0.330596997	0.001557531
Krt26	-0.379294181	0.972218452

Krt28	-0.205908181	0.99527876
Krt5	-0.342775777	0.89703631
Krt73	-0.00598657	0.997241386
Krt75	0.535907245	0.980132658
Krt77	-0.03200183	0.985673938
Krt78	-0.660780437	0.981502501
Krt79	0.426058868	0.936905058
Krt8	0.897666383	0.667414999
Krt8-ps	0.89009751	0.975831674
Krt80	0.080364395	0.993103295
Krt87	0.118841625	0.981045362
Thsd7b	-0.32954618	0.178907688
Krt90	0.841096555	0.87564489
Krtcap2	0.024531334	0.984836179
Krtcap3	-0.342876617	0.959276014
Ksr1	0.033984541	0.969755848
Ksr2	0.087082883	0.776477378
Kti12	0.167696795	0.742969797
Ktn1	-0.112956929	0.813552442
Kxd1	0.602261018	0.69189201
Ky	0.083246019	0.962648047
Kyat1	0.148557942	0.868415875
Kyat3	0.028752641	0.987342614
L1cam	-0.149477752	0.689605643
L2hgdh	-0.042242712	0.965520236
L3hypdh	-0.016473637	0.99527876
L3mbtl1	-0.063094907	0.97041548
L3mbtl2	0.068007752	0.951755452
L3mbtl3	-0.162570795	0.887135194
AC133487.1	1.077993124	0.969755848
Lacc1	-0.044482758	0.984232465
Lactb	-0.052065124	0.973100313
Lactb2	0.014538421	0.992098779
Lactbl1	-0.687806169	0.975831674
Lad1	0.491785001	0.777427974
Lag3	0.162613985	0.948104382
Lage3	0.149877158	0.876684592
Lair1	0.116960912	0.944098708
Lama1	-0.07113913	0.98187819
Lama2	-0.234207374	0.707069842
Lama3	-0.095617182	0.968630572
Lama4	-0.25257925	0.852010446
Lama5	-0.043372243	0.975831674
Lamb1	-0.100278722	0.780007405

Lamb2	-0.196897549	0.777427974
Lamb3	0.25063424	0.928489442
Lamc1	-0.034953263	0.974823941
Lamc2	0.412926467	0.882824434
Lamc3	-0.182450604	0.956164302
Lamp1	0.080520047	0.751142243
Lamp2	-0.00446049	0.99527876
Nin	-0.329146435	0.000679868
Lamtor1	0.056833189	0.951390592
Sgk3	0.328742644	0.184761973
Lamtor3	0.139558796	0.6883862
Lamtor4	0.049273622	0.968977192
Lamtor5	0.096465495	0.874252042
Lancl1	-0.000346109	0.999223454
Lancl2	-0.018862575	0.982914099
Lancl3	-0.285300698	0.867814377
Ccin	1.077993124	0.969755848
Lap3	-0.049177478	0.962648047
Laptm4a	-0.033567263	0.956922504
Laptm4b	0.288447607	0.91462326
Laptm5	0.009263873	0.99527876
Large1	-0.097377107	0.7851601
Ern2	1.077993124	0.969755848
Larp1	-0.119138016	0.982908926
Larp1b	0.043542511	0.972218452
Larp4	0.692876913	0.754350047
Larp4b	0.007472352	0.99527876
Larp6	0.01606703	0.985673938
Larp7	0.121445688	0.87564489
Lars	0.065459011	0.938939871
Lars2	-0.081615165	0.935890893
Las1l	0.095552134	0.708953765
Lasp1	-0.147417712	0.742849809
Lat	0.097934757	0.987342614
Lat2	-0.029923862	0.993380259
Lats1	-0.047267556	0.962648047
Cox4i1	0.328710258	5.25016E-06
Layn	-0.305971108	0.710772024
Lbh	-0.02080081	0.980132658
Lbhd1	0.244541493	0.799267833
Lbhd2	0.962742047	0.911631946
Lbp	0.10601997	0.969755848
Lbr	-0.091111983	0.920642026
Lbx2	0.836254691	0.97487264

Lrrc28	0.328676887	0.306736562
Lca5l	0.239838213	0.895520088
Lcat	-0.048223507	0.966968327
Lck	-0.212988292	0.95126064
Lclat1	-0.135564401	0.698427195
Lcmt1	0.044200558	0.939592821
Lcmt2	0.051442625	0.975831674
Hal	1.077993124	0.969755848
Lrtm1	1.077563623	0.965290366
Lcorl	0.078876068	0.936787526
Cdh11	-0.328298992	0.000872245
Lcp2	0.065987528	0.981509462
Lct	-0.045543632	0.962812066
Lctl	0.129489485	0.99527876
Ldah	-0.264691588	0.973100313
Ldb1	0.034750509	0.949607377
Ldb2	-0.046616558	0.980226283
Ldb3	0.165039917	0.889908105
Ldha	0.004951928	0.99527876
Ldha-ps2	0.220874603	0.985673938
Ldhb	0.031412197	0.962648047
Ldhd	0.177982112	0.91334561
Ldlr	-0.036520759	0.975800973
Ldlrad3	-0.066403143	0.962648047
Ldlrad4	-0.20394931	0.513557515
Ldlrap1	-0.224916805	0.864907053
Ldoc1	0.083429241	0.976978183
Leap2	0.294172543	0.993380259
Lef1	-0.334311494	0.810908327
Ntng1	0.328263279	0.09657124
Lefty2	0.011510842	0.996487647
Lekr1	-0.129202128	0.93912725
Lemd1	0.495749618	0.911631946
Lemd2	0.032444332	0.972387414
Lemd3	-0.088906451	0.897878088
Lenep	-0.084503492	0.975831674
Leng1	0.152326271	0.86128643
Leng8	-0.00519181	0.99527876
Leng9	0.308069256	0.591119873
Leo1	-0.042294437	0.965520236
Trim14	1.075503554	0.819240687
Leprot	0.111034903	0.842256259
Leprotl1	-0.046596526	0.925924974
Letm1	-0.006243619	0.99527876

Letm2	-0.095017691	0.956922504
Letmd1	-0.046912993	0.971138085
Lfng	0.255246378	0.731134488
Lgals1	0.160447432	0.852010446
Zcwpw2	1.074710994	0.562965533
Lgals12	-0.971309588	0.779415976
Lgals2	-0.769886925	0.969755848
Lgals3	-0.153055594	0.970673196
Snrpf	0.327954106	0.347698938
Lgals4	0.401989764	0.666177474
Lgals8	-0.16411705	0.544661714
Sdc4	0.327583409	0.000363092
Lgalsl	0.036781289	0.962648047
Lgi1	-0.115619042	0.895520088
Lgi2	0.065815838	0.936031628
Lgi3	0.128893443	0.623042338
Lgi4	0.006089096	0.996094752
Lgmn	-0.012243085	0.985742667
Lgr4	-0.172591422	0.544661714
Lgr5	-0.475547707	0.807021791
Lgr6	0.41551743	0.821254064
Lhb	-0.018049813	0.998010523
Lhcgr	-0.24758337	0.976978183
Lhfp	-0.003286548	0.997258205
Lhfpl1	-0.046953674	0.99100709
Lhfpl2	0.118807894	0.874252042
Lhfpl3	0.02549519	0.984836179
Lhfpl4	0.050926183	0.925788724
Lhfpl5	-0.441437298	0.62543868
Lhpp	-0.227800755	0.753987708
Lhx1	-0.488970512	0.91462326
Lhx1os	-0.183683806	0.99527876
Lhx2	0.049237487	0.959276014
Ace3	1.074566675	NA
Lhx5	-0.005457411	0.99930339
Lhx6	-0.076810826	0.948432875
Lhx9	-0.122986226	0.821998633
Lias	-0.059535913	0.955993235
Lif	-0.379416609	0.895520088
Lifr	-0.061731369	0.970348278
Lig1	0.130100368	0.89703631
Lig3	-0.075891753	0.950195611
Lig4	-0.133464004	0.852010446
Lilr4b	-0.978106725	0.759486652

Selenok-ps5	1.07402121	0.961373288
Lilra6	-0.952275183	0.974809035
Lilrb4a	-0.122548035	0.985742667
Lima1	-0.183519826	0.754134958
Limch1	0.04529335	0.962648047
Limd1	0.071788345	0.977133618
Frat1	0.327383489	0.226389202
Lime1	-0.064516325	0.975831674
Limk1	0.192474089	0.911631946
Limk2	-0.012161484	0.985673938
Lims1	-0.178532746	0.890635602
Lims2	0.323039603	0.838183693
Thrb	-0.326828247	0.209137754
Lin37	0.167518463	0.7851601
Lin52	0.040825924	0.968005385
Lin54	-0.002589887	0.997867015
Lin7a	-0.198732537	0.861366392
Lin7b	-0.001530515	0.99765039
Lin7c	0.003570146	0.995411429
Lin9	-0.015404041	0.99527876
Lingo1	-0.204060573	0.519411876
Lingo2	-0.388503398	0.549579107
Lingo3	-0.0684265	0.962648047
Lingo4	-0.357261257	0.962648047
Lins1	-0.236485987	0.550739492
Lipa	-0.120114409	0.839488666
Lipc	-0.552858525	0.89703631
Lipe	-0.14107336	0.852946549
Lipg	0.174906837	0.962648047
Liph	-0.013030605	0.997258205
Eme1	1.073758594	0.946907532
Lipo2	0.82043078	0.889972515
Lipo3	-0.194111382	0.836515405
Lipt1	-0.2838739	0.844269777
Lipt2	-0.044528555	0.98526072
Litaf	0.035329568	0.98187819
Lix1	-0.164883991	0.555615011
Lix1l	0.009620504	0.993103295
Llgl1	-0.121685119	0.789413986
Llgl2	-4.15862E-05	0.99991697
Llph	0.158273291	0.708626104
Llph-ps1	0.271812766	0.965520236
Llph-ps2	0.283365121	0.673443553
Lman1	-0.086372322	0.810908327

Zfp366	-0.326553709	0.425470982
Lman2l	-0.118417992	0.663732405
Snrpd1	0.326268887	0.057804391
Lmbr1l	0.020991611	0.99165967
Lmbrd1	-0.074468115	0.745988863
Egr4	-0.32612887	0.318279733
Lmcd1	-0.116735514	0.95126064
Lmf1	0.038668881	0.972218452
Lmf2	-0.020335583	0.991942027
Lmln	-0.120047345	0.873797321
Lmna	-0.03928974	0.965520236
Lmnb1	0.159019864	0.89703631
Lmnb2	0.068099365	0.934135315
Lmntd1	-0.228277041	0.962648047
Lmo1	-0.175711314	0.771450635
Lmo2	-0.171540865	0.980174892
Lmo3	-0.092459275	0.861366392
Lmo4	0.001761159	0.997529192
Gjb6	0.325336988	0.146637503
Lmod1	-0.065479646	0.988553438
Vnn1	1.072098892	0.958389182
Cldn14	1.071118009	0.661553809
Ankrd26	-0.324962723	0.485432358
Lmtk3	-0.037101458	0.962648047
Lmx1a	0.121456522	0.991438757
Pcdhb1	1.069656094	0.958951979
Clcnka	1.069272177	0.965520236
Lncpint	-0.450900886	0.921311891
Lncppara	0.099908038	0.965520236
Lnp1	0.105627673	0.97607383
Lnpep	-0.008582835	0.991438757
Lnpk	0.079027923	0.932673884
Lnx1	-0.058846126	0.965241989
Lnx2	-0.243943588	0.780499133
D6Ertd474e	1.069127492	0.911631946
Lonp1	0.02476381	0.971138085
Lonp2	-0.09475708	0.869437456
Lonrf1	0.026226578	0.98043358
Lonrf2	-0.056825066	0.911631946
Lonrf3	0.264724112	0.737369978
Lor	-0.184498227	0.920642026
Mir6988	1.068517653	0.959276014
Loxhd1	0.34660261	0.963330868
Loxl1	0.016152735	0.99527876

Loxl2	-0.294073455	0.971661054
Loxl3	0.025687938	0.993242999
Vrk1	0.324683573	0.435075615
Mir8114	0.323765511	0.31865776
Lpar2	-0.268164071	0.936834221
Lpar3	0.175895215	0.985673938
Lpar4	0.071087703	0.985293518
Lpar5	0.627864401	0.904346827
Lpar6	0.032144904	0.990550129
Lpcat1	-0.076207012	0.987342614
Lpcat2	-0.066816746	0.962648047
Lpcat3	-0.052838023	0.97487264
Lpcat4	0.022062	0.980896205
Trabd	-0.323740062	0.492371645
Lpin1	0.09758179	0.962648047
Lpin2	0.373847088	0.889972515
Lpin3	0.039701732	0.994960308
Lpl	-0.174863337	0.707069842
Mir344g	1.062696264	0.961887657
Lpp	0.041633884	0.984328758
Lppos	0.316924855	0.71596464
Lpxn	-0.016026016	0.99527876
Lrat	-0.181458799	0.985673938
Lrba	0.033355038	0.974852055
Lrch1	0.147104103	0.792517766
Lrch2	-0.223464111	0.825148733
Lrch3	-0.161140892	0.674542577
Lrch4	-0.104703494	0.993785185
Lrfn1	-0.098299258	0.898478018
Lrfn2	0.174593332	0.613183359
Anxa7	-0.323560831	0.482385306
Lrfn4	-0.118702317	0.839488666
Lrfn5	-0.059781207	0.904346827
Lrg1	-0.479792627	0.89703631
Lrguk	-0.182245196	0.92306576
Lrif1	-0.232219111	0.891972665
Lrig1	-0.176834857	0.920642026
Lrig2	-0.011130574	0.991996237
Lrig3	0.279849568	0.897878088
Lrit3	-0.929028241	0.962648047
Naa38	0.322563659	0.128871401
Lrmp	-0.281903278	0.855344864
Homer2	-0.322537283	0.023105145
Lrp10	-0.0714045	0.975831674

Lrp11	0.002306124	0.996645986
Lrp12	-0.035859714	0.965520236
Lrp1b	-0.11799846	0.788435146
Gpr146	0.321791188	0.323832374
Lrp2bp	-0.520929481	0.89383516
Lrp3	0.149177232	0.864150749
Lrp4	-0.172380045	0.661553809
Lrp5	0.144367824	0.901479608
BC037034	0.32177562	0.065333411
Lrp8	-0.001889114	0.999290575
Dupd1	1.061089237	0.762817527
Lrp8os3	-0.232551426	0.948104382
Lrpap1	0.073343811	0.796563869
Lrpprc	0.044832762	0.95520652
Lrrc1	0.28689572	0.510569404
Lrrc10	0.777443767	0.925472767
Lrrc10b	-0.049558445	0.968005385
Lrrc14	-0.110033189	0.811913793
Lrrc15	-0.382135144	0.963280717
Fancl	0.321711007	0.471012596
Lrrc18	-0.107141565	0.991996237
Lrrc19	-0.171360939	0.99527876
Lrrc2	0.019821111	0.99527876
Lrrc20	0.090268846	0.873753967
Lrrc23	-0.092330334	0.980339705
Lrrc24	-0.099311353	0.950460219
Lrrc25	-0.465616033	0.968005385
Cox6b1	0.321025696	0.065698296
Lrrc27	-0.025154715	0.989200732
Tmem56	-0.320400536	0.055853534
Lrrc29	-0.237063273	0.968977192
Lrrc3	0.189514383	0.767515266
Cryaa	1.055502098	0.871869598
Lrrc32	-0.539741883	0.788592804
Lrrc34	-0.360725514	0.974823941
Lrrc36	-0.020410362	0.99527876
Lrrc38	0.529828611	0.918407269
Lrrc39	0.218681935	0.947033215
Lrrc3b	0.213616419	0.837755777
Lrrc4	0.011053758	0.985742667
Lrrc40	-0.281351588	0.749626226
Lrrc41	-0.044933253	0.962648047
Lrrc42	0.088559744	0.941857776
Lrrc43	0.844118981	0.813599082

Lrrc45	0.020030299	0.985742667
Lrrc46	0.042408732	0.992385525
Chm	-0.320322442	0.146214787
Lrrc49	-0.0196757	0.981045362
Lrrc4b	-0.241783251	0.932673884
Lrrc4c	-0.073212898	0.991996237
Lrrc51	0.029195549	0.993489613
Lrrc55	-0.11836472	0.911631946
Lrrc56	0.170610059	0.945663104
Lrrc57	0.410597569	0.624220551
Alg13	-0.319467481	0.426109731
Lrrc59	0.037297528	0.954827342
Lrrc6	0.286108812	0.621993647
Lrrc61	-0.082826552	0.987326705
Lrrc7	-0.180184619	0.599763688
Lrrc71	-0.145567045	0.980226283
Lrrc73	-0.006101477	0.99527876
Lrrc74b	-0.454202826	0.878577494
Lrrc75a	0.078309612	0.971661054
Chml	1.052671196	0.774225415
Lrrc8a	0.182289853	0.968005385
Lrrc8b	-0.101089038	0.744924545
Aff1	0.319061288	0.116621969
Lrrc8d	-0.938618824	0.881407916
Lrrc8dos	0.755995749	0.975831674
Lrrc9	-0.067277162	0.988034453
Lrrcc1	0.016254699	0.99527876
Lrrd1	0.107229758	0.993380259
Nrep	0.31896195	0.243398308
Lrrfip2	0.043779461	0.959276014
Lrriq1	-0.136060551	0.98526072
Lrriq3	0.219344467	0.97487264
Lrrk1	-0.207019303	0.939060335
Lrrk2	0.034420333	0.976978183
Lrrn1	0.041371827	0.956164302
Lrrn2	-0.113220804	0.786324313
Arhgef2	0.318772457	0.000101973
Lrrn4	0.284009582	0.882824434
Lrrn4cl	-0.630018852	0.868415875
Rps23-ps1	0.318416285	0.000750039
Rbm14	-0.317753634	0.104879534
Lrrtm3	0.257698094	0.544361994
Lrrtm4	0.919242215	0.579542933
Lrsam1	0.030293358	0.980339705

Gucy2c	1.05066377	0.940504226
Lrtm2	0.145886003	0.852010446
Lrwd1	0.227257658	0.659463099
Lsamp	-0.044201746	0.911206484
Lsg1	0.107917265	0.86783605
Lsm1	0.34092795	0.710772024
Lsm10	0.347351658	0.839488666
Lsm11	-0.080380244	0.909943341
Lsm12	0.055679408	0.956057861
Lsm14a	0.015655716	0.98698961
Shank2	-0.317647573	0.059829752
Lsm2	0.299855562	0.683281689
Lsm3	0.150934847	0.813346233
Lsm4	0.020608592	0.987342614
Lsm5	0.118237332	0.956922504
Lsm6	0.135144145	0.778643537
Lsm7	0.159265479	0.749519672
Lsm8	-0.016738263	0.985673938
Atf7ip2	1.047291547	0.968497942
Lsmem2	0.080162326	0.99527876
Lsp1	-0.016150235	0.99527876
Lsr	0.215528054	0.871869598
Lss	-0.127626246	0.865454573
Lst1	0.095015495	0.975831674
Vill	1.046842324	0.561142625
Lta4h	0.088584865	0.847233342
Ltb	-0.726002865	0.822693227
Aox4	1.046554072	0.630347713
Ltb4r2	0.277550349	0.98340069
Ltbp1	0.091576575	0.958951979
Ltbp2	-0.177443874	0.986483021
Ltbp3	0.014206504	0.993103295
Ltbp4	-0.068232476	0.895520088
Arhgap33	-0.31747792	0.005045332
Ltc4s	0.193735976	0.89703631
Ltf	-0.627753884	0.978063275
Ltk	0.09279339	0.89011487
Ltn1	-0.093505844	0.85252367
LTO1	0.078101665	0.964011524
Ltv1	-0.001077963	0.998658264
Luc7l	-0.050650443	0.99527876
Luc7l2	-0.065898344	0.92380088
Luc7l3	0.248556101	0.689698377
Lum	0.140628987	0.973100313

Lurap1	0.14386087	0.871869598
Lurap1l	0.123788535	0.752941962
Luzp1	0.221097524	0.97487264
Luzp2	-0.139911609	0.5141747
Lvrn	0.660608915	0.965520236
Lxn	0.08471504	0.89011487
Ly6a	-0.120432758	0.946985353
Ypel3	0.317413032	0.41351939
Ly6c2	0.058028908	0.985742667
Ly6e	0.003276853	0.996630127
Ly6g5b	-0.141329822	0.985742667
Ly6g6d	0.055500714	0.99527876
Ly6g6e	-0.425312126	0.910328122
Ly6g6f	-0.19780947	0.974962198
Ly6h	0.042571767	0.97487264
Ly6k	0.060832785	0.995411429
Ly75	-0.015166071	0.99527876
Ly86	0.21787865	0.794684894
Ly9	0.717154689	0.831704864
Ly96	-0.177059311	0.966538721
Lyar	-0.316028928	0.8549794
Lyg2	0.259893959	0.970673196
Lrrfip1	-0.317201197	0.064044233
Lyn	0.052995488	0.97487264
Lynx1	-0.011451102	0.987075145
Lypd1	-0.174723115	0.903441375
Lypd6	-0.044656113	0.968005385
Lypd6b	0.015901943	0.993103295
Ndst4	1.046068384	0.544361994
Lypla1	-0.107869996	0.790879485
Lypla2	0.051097407	0.956164302
Lyplal1	0.090128674	0.968601153
Lyrm1	0.306764506	0.69189201
Lyrm2	0.023862044	0.99100709
Lyrm4	0.096313985	0.911631946
Lyrm7	0.025450654	0.985742667
Lyrm9	-0.079047444	0.860521845
Rpl23a	0.316930917	0.012427547
Lysmd2	-0.195322062	0.813346233
Lysmd3	-0.177864571	0.833916587
Lysmd4	-0.069355211	0.963943597
Lyst	0.080444748	0.91462326
Hgfac	1.042753322	0.967541135
Lyz1	-0.633206498	0.90358675

Sgip1	0.315688668	0.233530207
Lyzl4	-0.54901271	0.911631946
Lzic	-0.031569071	0.985293518
Ryr3	-0.315425785	0.00021266
Lztr1	-0.040911214	0.962648047
Lzts1	-0.058664015	0.951390592
Lzts2	0.152238408	0.817175245
Lzts3	-0.004531345	0.99527876
M1ap	0.912473879	0.962648047
M6pr	0.061950759	0.869437456
M6pr-ps	-0.943282904	0.852275035
Maats1	-0.256205656	0.939904081
Mab21l1	0.100037956	0.989840188
Cfap221	1.041188488	0.847678217
Macc1	-0.952564488	0.974809035
Ly6c1	0.315312238	0.328933138
Maco1	0.027375452	0.978063275
Macrod1	0.170079839	0.837755777
Macrod2	0.324934962	0.909728442
Mad1l1	-0.05034113	0.971364074
Mad2l1	-0.016031252	0.992385525
Mad2l1bp	0.267795919	0.56488584
Mad2l2	-0.093644577	0.971661054
Mir320	1.040672069	0.965520236
Madd	0.160610898	0.710375942
Maea	0.074354338	0.868415875
Mael	0.899004648	0.962648047
Maf	-0.1328365	0.878577494
Maf1	-0.038722535	0.97487264
Mafa	0.276505461	0.873797321
Mafb	-0.121355192	0.911631946
Atraid	0.314929733	0.239650538
Mafg	-0.067592027	0.89703631
Mafk	-0.002202808	0.99765039
Mag	-0.004366445	0.995944208
Plet1	1.038359273	0.642771234
Maged1	0.016630855	0.975831674
Maged2	-0.049165377	0.968630572
Magee1	-0.010498011	0.986246396
Magee2	0.120601602	0.912397394
Mageh1	0.045624458	0.968005385
Magel2	-0.015987511	0.995944208
Tipin	0.314323964	0.467859532
Zfas1	0.314171283	0.41351939

Magi3	-0.116921933	0.703372057
Magix	0.602009231	0.837755777
Magoh	0.268569334	0.625886453
Magohb	-0.025732053	0.993242999
Magt1	-0.103986713	0.929997774
Maip1	0.155745117	0.716433689
Mak	-0.171251763	0.970461999
Mak16	0.047441825	0.956922504
Mal	0.002796614	0.997375151
Rps13-ps4	0.313547572	0.38218128
Malat1	-0.074077742	0.911276003
Mall	-0.435286591	0.948104382
Malsu1	0.123111906	0.91462326
Malt1	-0.218219888	0.825594767
Mamdc2	-0.672472564	0.754350047
Mamdc4	0.172731304	0.965290366
Maml1	-0.080670662	0.955423359
Ankrd16	0.313311621	0.442132509
Maml3	0.106921208	0.962648047
Mamld1	0.08846266	0.859910936
Mamstr	0.623569985	0.792641681
Jakmip1	0.312871472	0.089553702
Rpl14	0.311681894	0.132404127
Man1b1	-0.056248122	0.890704638
Man1c1	0.009586089	0.992669879
Man2a1	-0.040423509	0.973100313
Man2a2	-0.067790225	0.824368034
Man2b1	0.10831224	0.758595939
Man2b2	-0.124157478	0.852010446
Man2c1	0.157774034	0.783611405
Man2c1os	0.016512036	0.99527876
Manba	-0.171864245	0.800386557
Manbal	0.124237241	0.79212401
Manea	-0.140772719	0.859910936
Maneal	0.03651999	0.969755848
Manf	-0.085678189	0.901479608
Mansc1	0.084657897	0.961373288
Mansc4	0.148645279	0.983797678
Maoa	0.047884558	0.962648047
Maob	0.071532625	0.911631946
Map10	0.154923978	0.916961045
Map1a	-0.036695981	0.950460219
Map1b	-0.054390187	0.860521845
Map1lc3a	0.094317037	0.881371608

Tefm	0.311551569	0.46220464
Map1s	-0.125222681	0.852946549
Gabrb2	-0.311133222	0.399438388
Map2k1	-0.004360971	0.99527876
Map2k2	-0.046470774	0.950604431
Map2k3	-0.06143493	0.981502501
Map2k3os	-0.279694224	0.957499962
Map2k4	-0.062721017	0.858549868
Map2k5	0.060729318	0.91462326
Map2k6	-0.124858755	0.911631946
Map2k7	0.036293748	0.994960308
Map3k1	-0.205249099	0.836569459
Map3k10	-0.019640164	0.976978183
Snrpd2	0.311087821	0.000588458
Map3k12	0.021598693	0.974823941
Tsc22d1	0.311017505	0.274566821
Map3k14	-0.246539887	0.849835417
Map3k15	-0.361307584	0.807017299
Map3k19	-0.20216992	0.869316117
Map3k2	-0.092370343	0.86783605
Map3k20	0.138956835	0.911631946
Map3k21	-0.349935282	0.631708623
Map3k3	-0.154185347	0.627261794
Map3k4	0.116224061	0.801926713
Map3k5	0.005062044	0.99527876
Cdc42bpg	0.310905916	0.497690258
Map3k7	-0.121048344	0.643053315
Map3k7cl	-0.246715369	0.966608583
Map3k8	0.317899161	0.84277074
Map3k9	-0.078456554	0.951817826
Map4	-0.131986428	0.56399886
Map4k1	-0.249161731	0.899097089
Map4k2	0.174092176	0.737279585
Map4k3	-0.021525462	0.975831674
Map4k4	-0.027738764	0.973100313
Map4k5	-0.239659924	0.936031628
Map6	-0.063119514	0.908413609
Hspb11	0.310843249	0.463081676
Map7	0.074516056	0.84870301
Map7d1	-0.01010787	0.989200732
Map7d2	-0.008106869	0.99165967
Map9	-0.178013028	0.566572868
Mapk1	0.032206565	0.948345831
Mapk10	0.04557823	0.962648047

Mapk11	0.05955584	0.962648047
Mapk12	0.139275871	0.950520575
Mapk13	-0.407558743	0.980339705
Mapk14	0.130600076	0.911631946
Mapk15	-0.305354674	0.918407269
Mapk1ip1	-0.110252859	0.92380088
Mapk1ip1l	-0.013587375	0.987053625
Cnppd1	0.310565635	0.477528928
Mapk4	-0.030126084	0.975831674
Mapk6	-0.086637594	0.909547396
Mapk7	-0.048443063	0.981045362
Mapk8	-0.029615032	0.968005385
Mapk8ip1	0.046809973	0.944074069
Mapk8ip2	0.015244691	0.984748462
Mapk8ip3	-0.117463111	0.613183359
Mapk9	-0.056831959	0.939260148
Mapkap1	-0.066866396	0.879133004
Mapkapk2	0.050326855	0.956714002
Mapkapk3	-0.335497675	0.890704638
Mapkapk5	0.278411205	0.836557013
Mapkbp1	-0.03772719	0.965520236
Mapre1	-0.039862392	0.959659722
Anapc13	0.30964264	0.18316876
Mapre3	0.048139881	0.918235054
Mapt	-0.039085625	0.948104382
Marc1	-0.32736618	0.965520236
Marc2	-0.016197024	0.987899733
Marcks	0.059380129	0.949111839
Dmxl2	-0.309534525	0.128640258
Marf1	-0.063815877	0.928672524
Mark1	-0.022994829	0.97469529
Mark2	-0.014376016	0.991438757
Mark3	0.00033906	0.999242708
Mark4	-0.084219483	0.909547396
Mars	-0.048961797	0.935897306
Mars2	-0.181411016	0.534461098
Marveld1	0.067826448	0.974809035
Marveld2	0.13028272	0.974823941
Marveld3	-0.115897528	0.99527876
Mas1	0.048035042	0.946239011
Masp1	-0.126141703	0.86783605
Masp2	-0.239540079	0.968005385
Hps5	-0.309518325	0.178586844
Mast2	0.055343407	0.959276014

Mast3	-0.044335854	0.894148925
Mast4	-0.064331056	0.974809035
Mastl	-0.07031843	0.985742667
Mat2a	-0.125894124	0.895198797
Mat2b	0.04248862	0.942274311
Matk	0.113385515	0.56399886
Mir1969	1.038267056	0.962094124
Matn2	-0.099536888	0.869602864
Matn4	-0.046318741	0.97932602
Matr3	-0.141724979	0.607527044
Mau2	-0.044005235	0.962648047
Mavs	0.002004246	0.998010523
Max	0.171012061	0.563984287
Maz	-0.02879548	0.98187819
BC021767	1.037794733	0.932673884
Mb21d2	0.060895964	0.942812446
Mbd1	-0.027498645	0.968005385
Mbd2	-0.009413777	0.994960308
Mbd3	0.111900239	0.63519309
Mbd4	-0.149751695	0.907230149
Mbd5	-0.094114045	0.932956012
Mbd6	0.21536543	0.813744313
Mbip	0.215860958	0.80931022
Mblac1	-0.048047332	0.985653607
Mblac2	-0.033531135	0.973100313
Mbnl1	0.227215886	0.974823941
Mbnl2	-0.128286917	0.745988863
Mboat1	-0.079964729	0.973100313
Cdkl5	-0.308499083	0.022383499
Mir493	1.03714075	NA
Mboat7	-0.14441755	0.855904169
Mbp	0.108905421	0.89703631
Mbtd1	-0.159493158	0.710772024
Mbtps1	0.010088659	0.99100709
Mbtps2	-0.045762653	0.962648047
Mc3r	-0.730468127	0.962648047
Mc4r	0.206864723	0.78347729
Mc5r	0.321399824	0.985742667
Mcam	-0.173008546	0.74632237
Hist1h1c	-0.307748027	0.22999796
Mcc	0.207262311	0.84870301
Mccc1	-0.086637682	0.911631946
Mccc1os	-0.20237091	0.966213117
Mccc2	0.015437556	0.987607832

Mcee	0.124206929	0.822515132
Mcemp1	-0.019284207	0.998010523
Mcf2	-0.091532133	0.965520236
Mcf2l	-0.021626862	0.991438757
Mcfd2	0.057910604	0.962648047
Mchr1	-0.158459151	0.875723186
Mcidas	0.373355575	0.982408617
Mcl1	-0.093906272	0.732729469
Mcm10	-0.339564397	0.932673884
Mcm2	-0.227025499	0.749626226
Mcm3	0.035755243	0.991438757
Mcm3ap	-0.046096204	0.956072972
Mcm4	-0.105949897	0.911631946
Mcm5	0.11308033	0.957849305
Mcm6	0.232023409	0.707520358
Mcm7	0.231862372	0.529257466
Mcm8	-0.113493777	0.936507368
Mcm9	-0.076318503	0.971750542
Mcmbp	-0.082901054	0.907829139
Mcmdc2	0.086814776	0.980226283
Mcoln1	-0.09059564	0.857611652
Mcph1	0.042838149	0.976978183
Mcrip1	0.006004108	0.99527876
Mcrip2	0.105322197	0.962648047
Mcrs1	-0.023503278	0.985742667
Mctp1	-0.035240189	0.971661054
Mctp2	0.577080389	0.522113371
Mcts1	0.093035919	0.848380246
Mcts2	0.285562857	0.5840348
Mcu	0.104597931	0.807017299
Mcub	0.329018897	0.756001016
Mcur1	0.00569718	0.99527876
Mdc1	-0.093358585	0.89703631
Mdfi	-0.031997201	0.99527876
Mdfic	0.065859739	0.987453225
Nxn	0.307164755	0.465695122
Mdga2	-0.070223598	0.938615214
Mdh1	0.095923395	0.571020232
Mdh1b	-0.256356618	0.953505913
Mdh2	0.075059478	0.764831511
Mdk	0.060731223	0.969755848
Mdm1	-0.101481646	0.939592821
Mdm2	-0.002712783	0.997115148
Mdm4	-0.066669731	0.865884071

Mdm4-ps	-0.057535447	0.99527876
Rps13-ps1	0.306687458	0.160287523
Mdp1	0.136248512	0.563984287
Mdrl	0.876158489	0.904985162
Me1	-0.085278536	0.843408683
Me2	0.03075048	0.975800973
Me3	-0.028556905	0.968005385
Dpy30	0.306660838	0.365076065
Rorb	-0.305769286	0.383515294
Mecom	-0.049795732	0.990661552
Mecp2	-0.077321987	0.84870301
Mecr	-0.056546977	0.964941885
Med1	-0.044336039	0.954827342
Med10	0.120947242	0.8549794
Med11	-0.054275123	0.981045362
Med12	-0.080405231	0.924707867
Med12l	-0.089448206	0.895198797
Med13	-0.074073803	0.898478018
Med13l	-0.10600434	0.623042338
Bcor	-0.305375623	0.214650211
Med15	-0.06830282	0.912042382
Med16	-0.012382151	0.991438757
Med17	-0.029864896	0.972376117
Chrna7	-0.305333011	0.411803902
Med19	0.005014944	0.99527876
Med20	-0.063569361	0.950195611
Ankhd1	-0.305259448	0.27948574
Med22	0.208112354	0.701145989
Med23	-0.127167268	0.749519672
Med24	-0.022469688	0.974823941
Med25	-0.099600424	0.758595939
Med26	-0.192976357	0.732729469
Med27	0.14310108	0.69189201
Med28	-0.003349624	0.995411429
Med29	0.151339789	0.875056988
Med30	0.188853226	0.791616882
Med31	0.025536474	0.985742667
Klhdc7a	0.305168027	0.365352735
Med6	0.056598925	0.97487264
Med7	0.117482229	0.912358021
Med8	-0.018002737	0.985742667
Med9	-0.076667016	0.873797321
Med9os	-0.680460454	0.89703631
Medag	0.043470709	0.976978183

Mef2a	0.025120696	0.984328758
Mef2b	-0.40271713	0.968005385
Wdr93	1.035583121	0.833916587
Mef2d	-0.056302029	0.890704638
Meg3	-0.029375154	0.968005385
Megf10	0.059419415	0.936031628
Megf11	0.272064166	0.883426561
Megf6	-0.319916367	0.789413986
Megf8	-0.084138558	0.801926713
Megf9	-0.112451013	0.745988863
Mei1	0.134815738	0.984748462
Mei4	-0.760244235	0.661684145
Meig1	0.381162355	0.74463793
Acyp2	0.30464766	0.350732723
Meis1	-0.273870726	0.911631946
Nob1	0.304192353	0.341690144
Meis3	0.137214619	0.788592804
Melk	0.470658173	0.932931799
Meltf	-0.456850072	0.949607377
Memo1	-0.146786281	0.875723186
Amigo1	0.303701106	0.507697207
Meox1	-0.336948188	0.951390592
Mepce	-0.001038374	0.998010523
Sipa1l1	-0.303685463	0.065333411
Mesd	0.046329686	0.928957615
Mesp2	0.36745544	0.948104382
Mest	-0.090179151	0.928957615
Met	-0.254520207	0.862838439
Metap1	0.025363631	0.97487264
Metap1d	0.210393337	0.710772024
Metap2	-0.086417687	0.979709328
Metrn	0.016015693	0.993449239
Metrnl	-0.033864281	0.98459388
Mettl1	-0.152054745	0.865547585
Mettl11b	0.109207301	0.983797678
Mettl13	0.177042395	0.573810203
Mettl14	0.275246479	0.829694131
Mettl15	-0.12541564	0.932673884
Fam208b	-0.303472866	0.260708948
Mettl17	-0.073314748	0.950460219
Mettl18	0.142222014	0.942812446
Mettl2	-0.026357101	0.985653607
Mettl21a	0.195359302	0.703372057
Mettl21c	0.816199468	0.774031506

Mettl22	0.086245726	0.935897306
Mettl23	-0.073675616	0.962648047
Mettl24	0.205088413	0.961373288
Mettl25	0.068220072	0.981045362
Mettl26	0.035460755	0.985742667
Mettl27	0.280984007	0.91241376
Mettl3	0.028922581	0.982914099
Arpc1b	0.303194342	0.378321779
Mettl4-ps1	-0.952275183	0.974809035
Mettl5	0.048230033	0.97487264
Mettl5os	-0.026720701	0.996094752
Mettl6	0.010034028	0.994532146
Mettl7a1	0.113125206	0.850260513
Mettl7a3	-0.475362554	0.949607377
Mettl8	-0.050941181	0.97469529
Mettl9	0.066888741	0.894953269
Mex3a	-0.196920116	0.873753967
Mex3b	-0.042051932	0.976257618
Mex3c	-0.13817563	0.736130784
Mex3d	-0.080456532	0.962648047
Mfap1a	0.023486352	0.974991393
Mfap1b	-0.002614673	0.996216706
Mfap2	0.28023456	0.783229859
Igf2bp1	1.035431392	0.971138085
Mfap3l	-0.2552096	0.609167039
Mfap4	-0.25413724	0.88797692
Mfap5	0.190944688	0.993380259
Mff	0.048316646	0.904346827
Tmem258	0.303037079	0.399005965
Sbk1	-0.302847513	0.067960585
Mfn1	0.002169584	0.997115148
Mfn2	0.04421863	0.932673884
Mfng	-0.384985732	0.631468136
Mfrp	0.234677895	0.980896205
Mfsd1	0.068887079	0.917791707
Mfsd10	0.13047752	0.89703631
Mfsd11	0.030793046	0.985673938
Mfsd12	0.048135555	0.957169848
Mfsd13a	0.071336153	0.91462326
Tbc1d7	0.302749764	0.404892636
Mfsd14a	-0.04347011	0.952385409
Mfsd14b	-0.117478851	0.815662897
Adgrb3	-0.30250195	0.41745316
Mfsd2b	0.540477408	0.70685392

Mfsd3	0.405171271	0.603627131
Mfsd4a	0.089592697	0.91462326
Mfsd4b1	0.203042202	0.971661054
Mfsd4b3	-0.300855453	0.98526072
Mfsd4b4	0.078214302	0.944494911
AC126942.2	1.034899753	0.971138085
Mfsd5	-0.024144913	0.985742667
Mfsd6	-0.117012959	0.821998633
Mfsd6l	-0.737469964	0.972218452
Mfsd7a	-0.235537921	0.962648047
Mfsd8	-0.172484391	0.571020232
Mfsd9	0.06996936	0.965290366
Mga	-0.158953385	0.97487264
Mgat1	0.373601446	0.917685272
Mgat2	-0.003424502	0.995500778
Mgat3	-0.035785545	0.942812446
Mgat4a	-0.139623255	0.639556956
Mgat4b	0.037614847	0.962648047
Mgat4c	0.297589728	0.789833208
Mgat5	-0.017000175	0.985742667
Mgat5b	0.112559385	0.820931824
Mgea5	-0.05706937	0.89011487
Mgl2	-0.157371774	0.991438757
Mgll	0.241683824	0.69189201
Mgme1	-0.037086487	0.98526072
Mgmt	0.041915457	0.993103295
Mgp	-0.229004438	0.91907636
Mgrn1	-0.051770717	0.908413609
Mgst1	0.149028008	0.869316117
Cysrt1	1.034899753	0.971138085
Mgst3	0.08319005	0.895198797
Mhrt	-0.505648062	0.937550602
Mia	0.475264087	0.872352604
Mia2	0.077774093	0.87564489
Mia3	0.022779233	0.975831674
Miat	0.08023522	0.824368034
Mib1	0.003951636	0.99527876
Mib2	0.002663371	0.995944208
Mical1	0.047936879	0.976546786
Mical2	-0.106549946	0.720841863
Mical3	-0.126278595	0.635276515
Micall1	-0.052449885	0.915230696
Micall2	-0.241853011	0.932673884
Ssbp3	0.302369512	0.421157413

Micu2	-0.037806245	0.969755848
Micu3	-0.008318891	0.991996237
Mid1	0.008087692	0.99527876
Mid1-ps1	0.0880179	0.98526072
Mid1ip1	0.147829673	0.533008784
Mid2	0.147847744	0.788577401
Rab34	0.301855437	0.130213261
Yeats2	-0.301844876	0.417721745
Mief2	-0.066233172	0.948104382
Mien1	0.027563835	0.975831674
Mier1	-0.076169648	0.875723186
Nr2e1	-0.301297533	0.360222394
Mier3	-0.024606831	0.981392364
Mif	-0.031798249	0.980419928
Mif4gd	0.060540226	0.979037366
Miga1	-0.061358251	0.91462326
Miga2	0.052651385	0.95363833
Miip	-0.000236811	0.999486844
Mill2	0.406731686	0.890704638
Tmed2	1.031778372	0.620124996
Minar1	0.218305185	0.911631946
Minar2	0.071055671	0.927471971
Mindy1	0.143177959	0.810767226
Mindy2	-0.041806151	0.954827342
Mindy3	-0.213945818	0.779231312
Mindy4	-0.085871057	0.962648047
Mindy4b-ps	0.783239933	0.721943953
Mink1	-0.02580333	0.962648047
Dnajb12	0.300949105	0.38218128
Minpp1	0.010921587	0.988034453
Mios	0.020168097	0.985742667
Mipep	-0.082329816	0.864280235
Mipepos	0.34527409	0.985742667
Mipol1	-0.053360838	0.985742667
Mir101a	0.08251524	NA
Mir103-2	-0.563060721	0.971138085
Mir106b	-0.1377232	0.993277997
Mir1188	-0.32247959	0.971661054
Mir1191	0.101885033	NA
Mir1198	0.51108413	0.962648047
Hist1h3h	1.031771455	0.974809035
n-R5s171	1.030601373	NA
Mir124-2hg	-0.103818574	0.99100709
Mir1249	0.08251524	NA

Mir124a-1	0.08251524	NA
Mir124a-1hg	-0.055723058	0.891972665
Mir124a-2	-0.086949046	0.99527876
Mir124a-3	-0.904043206	NA
Lhx3	1.029973472	0.968005385
Mir125a	0.841698986	NA
Mir126a	-0.527730335	NA
Mir128-1	-0.470934264	0.985742667
Mir129-1	0.673749514	NA
Mir1291	-0.104629748	0.99100709
Mir1306	0.08251524	NA
Adra2b	1.028026081	0.875007504
Mir132	0.445869032	0.976956375
Mir134	0.08251524	NA
Mir137	0.088716843	NA
Mir138-1	-0.326146586	NA
Mir138-2	0.08251524	NA
Mir140	0.077663335	NA
Mir142b	0.375502933	0.988034453
Loxl4	1.026690721	0.929618757
Mir147	-0.527730335	NA
Mir149	0.822919702	NA
Mir154	0.07551355	NA
Fbxo15	1.019323431	0.965290366
Mir16-1	0.988112711	NA
Cox7b2	1.018758062	0.903795193
Mir17hg	0.8421127	0.911631946
Mir181b-1	0.097809398	NA
Mir181c	0.674153254	0.97487264
Mir181d	0.348146212	0.985673938
Mir1839	0.08251524	NA
Zbtb9	1.016689695	0.953688946
Mir186	0.160169067	0.978063275
Mir1892	0.08251524	NA
Mir1893	0.08251524	NA
Mir1894	0.08251524	NA
Mir1896	-0.527730335	NA
Scnn1g	1.015194367	0.91462326
Mir1898	-0.328450107	0.969111286
Rnf2	-0.300917247	0.089315573
Mir1901	0.08251524	NA
Mir1902	-0.326146586	NA
Mir1903	0.08251524	NA
Mir1906-1	0.08251524	NA

Mir1906-2	0.08251524	NA
Tmem243	0.300848496	0.394767148
Mir191	0.08251524	NA
Mir1929	0.618124384	NA
Mir1930	-0.484971009	NA
Ccer2	1.012881658	0.513557515
Sh2d4a	1.010989549	0.951390592
Znrd1	0.300549431	0.395640848
Mir1946b	-0.386733605	0.987066554
Mir1949	-0.107162822	0.99527876
Shisa3	1.008746116	0.770793864
Mir1957a	-0.355996586	NA
Ms4a7	1.008027766	0.957499962
Mir1966	0.207987573	0.983008673
Tex38	1.007859786	0.872352604
AL805899.1	1.004311383	0.955908516
Mir1981	0.343898266	0.985673938
Mir1982	0.08251524	NA
Mir199a-1	-0.203292666	0.989840188
Fbxo11	0.299739341	0.41351939
Mir207	0.08251524	NA
Rgn	1.001883569	0.900330211
Mir212	-0.080098368	0.99527876
Mir2139	-0.326146586	NA
Mir215	-0.78365969	0.980419928
Mir7648	1.000088469	NA
Mir21a	0.08251524	NA
Mir22	0.114703301	0.968005385
Mir221	0.127366621	0.993489613
Mir222	-0.279098024	NA
Mir22hg	0.071162611	0.91462326
Mir23b	-0.820993687	0.871869598
Mir24-1	0.08251524	NA
Mir24-2	-0.880450057	NA
Mir25	-0.527730335	NA
Mir26a-1	-0.417059313	0.985673938
Olfr559	-1.000855117	0.957485565
Mef2c	-1.003437068	0.549579107
Myrfl	-1.004040923	0.803910915
Mir2861	-0.306087198	0.975800973
Mir29b-2	0.08251524	NA
Mir29c	-0.231875485	0.991996237
Mir3057	0.08251524	NA
Mir3060	-0.090932649	0.988034453

Mir3061	0.493591789	NA
Mir3062	-0.335715145	NA
Mir3064	0.08251524	NA
F3	0.299286945	0.222955004
Tnfrsf14	-1.00598466	0.703633347
Mir3070b	-0.058069949	0.99527876
Snord23	-1.007487862	0.949607377
Mir3076	0.08251524	NA
Mir3077	-0.335715145	NA
Mir3078	0.08251524	NA
Mir3082	0.068950499	0.99527876
Angpt2	-1.007648523	0.522639613
Mir3091	0.03693313	0.994960308
Clec4e	-1.010902581	0.909547396
Mir3094	0.071360757	NA
Mir3097	-0.401799676	0.984328758
Mir3098	0.408932853	0.985293518
Mir31	-0.828528075	NA
Mir3100	0.14397826	0.989840188
Comp	-1.012678091	0.752941962
Mir3102	0.08251524	NA
Mir3103	0.08251524	NA
Mir3104	-0.651950395	NA
Mir3112	0.08251524	NA
Mir3113	0.08251524	NA
Calhm6	-1.014033606	0.936031628
Mir324	0.08251524	NA
Mir330	0.08251524	NA
Mir331	-0.326146586	NA
Mir337	-0.670010319	0.979037366
Mir338	0.352374804	NA
Alox12	-1.017719965	0.751142243
Mir341	0.68043795	0.950460219
Mir343	-0.328256357	0.965290366
Mir344-2	0.129739255	NA
Rpl22-ps1	-1.017747	0.932673884
Mir345	-0.706236515	0.962648047
Mir3470b	-0.619777806	0.980339705
Mir3473e	-0.776594032	0.968859834
Mir3474	-0.248456593	0.99161214
Mir3535	-0.629377213	0.968005385
Mir3572	0.08251524	NA
Rho	-1.017801344	0.629280869
Cd79b	-1.018243009	0.861909929

Mir370	-0.74826329	0.971661054
Mir374b	-0.131981309	0.97607383
Mir377	0.08251524	NA
Mir378b	-0.266036491	NA
Mir382	-0.499627983	0.980896205
Mir3960	0.08251524	NA
Mir409	-0.066814603	0.99527876
Mir410	0.07551355	NA
Mir412	-0.235634634	0.984232465
Mir421	-0.216306494	0.966608583
Mir423	0.071627449	NA
Mir425	0.808600305	NA
Mir453	-0.283905218	0.985673938
RF01953	-1.018255955	0.928672524
Mir467f	-0.951711466	NA
Mir485	0.402885789	0.976978183
Mir491	-0.45856176	0.969755848
Kcng3	-1.018541299	0.858549868
Mir496a	-0.267770996	0.985742667
Mir5046	0.08251524	NA
Acot12	-1.019558345	0.638414898
Mir5107	0.08251524	NA
Mir5113	0.418179897	0.98526072
Mir5114	0.08251524	NA
Mir5116	0.08251524	NA
Ankdd1b	-1.02199559	0.971138085
Mir5119	0.098046726	0.99527876
Mir5121	0.08251524	NA
Mir5122	0.08251524	NA
Mir5125	0.063951989	0.980226283
Mir5129	-0.335832243	NA
Mir5130	-0.859852938	0.962648047
AC126937.1	-1.022678741	0.92938762
Mir5132	0.08251524	NA
Sema6d	-0.299227504	0.236002915
Mir5134	0.08251524	NA
Mir5136	0.743600307	0.91462326
Mir541	-0.009540196	0.998010523
Mir546	0.082554751	0.99527876
Mir5617	0.08251524	NA
Mir5620	0.599604406	0.962648047
Dlgap5	-1.024410722	0.825910982
Mir5625	0.08251524	NA
Mir568	0.08251524	NA

Mir5710	0.467893109	NA
Mir6359	0.097809398	NA
Mir6362	0.442783712	0.981392364
Madcam1	-1.024512354	0.953505913
Mir6369	0.08251524	NA
Mir7016	-1.025650987	0.923011738
Mir6390	0.074463198	0.995632078
Prpf39	-0.298387037	0.338081307
Mir6395	0.996175054	0.920642026
Mir6397	-0.519049768	NA
Apol10b	-1.029252688	0.785866838
Mir6409	-0.806625944	NA
Nr4a3	-1.031842497	0.810503622
Mir6516	0.08251524	NA
Mir6537	0.08251524	NA
Mir665	0.647493205	0.980731359
Mir668	0.07020994	NA
Mir670hg	-0.057509873	0.979807057
Mir671	0.08251524	NA
AC122821.1	-1.03466383	0.8226891
Mir6769b	-0.600417794	0.962648047
Mir677	-0.269645001	0.982914099
Mir678	0.08251524	NA
Mir682	0.08251524	NA
Mir7049	-1.03509265	0.936031628
Mir686	0.082554751	0.99527876
Mir688	0.08251524	NA
Mir208a	-1.035863366	0.945526153
Mir6898	0.65555324	NA
Mir6899	0.988112711	NA
Fxyd4	-1.036503023	0.948104382
Mir6902	-0.231875485	0.991996237
Mir6903	0.08251524	NA
Mir6904	-0.039555696	0.99527876
Mir6905	0.068933251	0.99527876
Mir6906	0.466420081	0.980226283
Mir6907	0.959614892	0.947232837
Vmn1r-ps136	-1.039529057	0.938414123
Mir6912	0.08251524	NA
Mir6913	-0.335715145	NA
Mir6914	0.08251524	NA
Mir6915	-0.517646587	0.971138085
Mir6916	-0.26352717	0.985742667
Mir6918	-0.586933396	0.974197921

Mir6919	0.238730015	0.988090086
Mir692-2	0.061115388	NA
Mir692-3	0.08251524	NA
Mir6920	0.040719528	NA
Slc22a6	-1.04044796	0.71585388
Mir6922	0.673528852	NA
Mir6925	-0.659950818	NA
Mir6926	-0.335832243	NA
Mir6928	-0.241350699	0.98340069
Scarna3a	-1.040967769	0.950460219
Ifi209	-1.041071874	0.949607377
Mir6933	0.08251524	NA
Mir6934	0.08251524	NA
Mir6935	0.08251524	NA
Mir6936	0.08251524	NA
Mir6937	-0.667084855	NA
Mir6939	0.112373867	0.99527876
Dpep2nb	-1.042440343	0.954170013
Mir6941	0.063254221	0.996431931
Mir6942	0.478475897	0.973100313
Mir6943	0.113270228	NA
Mir6944	-0.080600028	0.99527876
Mir6945	0.08251524	NA
Rpl13-ps5	-1.042949851	0.970461999
Osgin1	-1.0443423	0.891972665
Mir6948	-0.399405161	0.971661054
Mir6951	-0.248650487	0.991996237
Mir6953	-0.161855204	0.985742667
Mir6954	0.08251524	NA
Mir6955	-0.06128835	0.993380259
Mir6958	0.08251524	NA
Mir6959	-0.335832243	NA
Mir6962	0.947776825	NA
Mir6966	0.08251524	NA
Ulbp1	-1.045309741	0.834224299
Mir697	0.469065439	0.970348278
Mir6970	0.08251524	NA
Mir6971	0.08251524	NA
Mir6972	-0.048222086	0.99527876
Mir99b	-1.045969258	0.962648047
Mir6973b	0.08251524	NA
Mir6975	-0.253781972	0.991559279
Mir6976	-0.733527068	0.962648047
Amd-ps5	-1.047015994	0.949111839

Mir6979	0.101885033	NA
Mir698	0.08251524	NA
Mir6981	0.988112711	NA
Mir6982	-0.994675425	0.552172819
Mir6984	0.282475484	0.987597026
Mir6985	0.681882546	NA
Mir6986	0.312684536	0.985742667
Vil1	-1.048960477	0.724910334
Mir6989	-0.22546087	0.985293518
Mir6990	0.079697477	NA
Mir6991	0.17293715	0.972218452
Mir6992	0.08251524	NA
Mir6993	0.388495453	0.97487264
Mir6994	-0.806354819	NA
Mir6995	0.673528852	NA
Mir6997	0.08251524	NA
Mir6998	-0.077042122	0.99527876
Mir6999	-0.120285144	0.989200732
Mir7-1	-0.085624387	0.991606359
Mir700	0.399835657	0.985446046
Mir7000	0.539128695	0.965520236
Mboat4	-1.049459645	0.950460219
Mir7004	-0.101108878	0.99527876
Mir7005	0.686679186	0.97487264
Mir7006	-0.145789285	0.994960308
Mir7008	0.08251524	NA
Mir7010	0.08251524	NA
Mir7011	0.08251524	NA
Mir7013	0.435012218	0.962363869
Atp4a	-1.050831528	0.89703631
Mir7015	-0.335832243	NA
Rdh1	-1.052679678	0.951390592
Fam205a1	-1.052700607	0.869316117
Mir7019	0.08251524	NA
Mir702	0.493591789	NA
Mir7020	0.08251524	NA
Mir7021	0.08251524	NA
Mir7022	-0.828528075	NA
Saa3	-1.05475181	0.968005385
Mir7025	0.08251524	NA
Mir7026	-0.477661223	0.932673884
Mir7027	-0.574460388	0.97487264
Mir7028	0.688518437	0.962648047
Mir7029	0.08251524	NA

Mir703	0.347547149	0.932673884
Mir7030	-0.516032826	0.968005385
Mir7031	-0.643839218	NA
Ripply3	-1.06036546	0.795833907
Mir7035	-0.828528075	NA
Mir7036	0.08251524	NA
Mir7037	0.08251524	NA
Mir704	-0.643839218	NA
Aqp10-ps	-1.061078214	0.944391691
Mir7042	0.101885033	NA
Mir7044	0.80408995	NA
Mir7045	0.08251524	NA
Mir7046	-0.082480485	0.99527876
Mir7047	0.070324605	NA
Mir7048	0.699714487	NA
Phf11d	-1.063682028	0.571020232
Mir705	0.08251524	NA
Mir7050	0.08251524	NA
Mir7051	0.08251524	NA
Mir7052	0.188833598	0.938939871
Rps7-ps2	-1.064753391	0.803910915
Mir7056	0.077222069	0.99527876
Mir7058	0.097809398	NA
Mir7059	-0.303473532	NA
Mir7060	0.959668927	0.948104382
Mir7061	-0.438040474	0.969755848
Mir7063	0.661479179	0.959659722
Mir7065	0.988112711	NA
Mir7067	-0.527730335	NA
Mir7068	0.08251524	NA
Mir7069	-0.285430843	0.974197921
Mir707	0.08251524	NA
Mir7070	0.493591789	NA
Mir7075	-0.77280014	0.962648047
Mir7077	0.08251524	NA
Mir7078	0.6210625	0.962121011
Mir7079	0.08251524	NA
Mir7080	0.08251524	NA
Mir7081	-0.904169594	NA
AC151267.2	-1.065060767	0.89703631
Mir7084	0.08251524	NA
Mir7085	0.517393361	NA
Mir7087	-0.036139309	0.997241386
Mir709	0.078866702	0.995411429

Mir7090	-0.335832243	NA
Mir7091	0.847720003	NA
Mir7092	0.08251524	NA
Mir7093	0.974166378	0.824368034
Mir7115	0.08251524	NA
Mir7116	-0.494905839	NA
Mir7117	0.08251524	NA
Mir7118	-0.448116796	NA
Mir713	0.723186268	NA
Mir719	0.100038069	0.99527876
Mir7211	-0.355996586	NA
Mir7219	0.08251524	NA
Mir7220	-0.344197783	0.960201673
Mir7225	0.08251524	NA
Mir7226	0.822919702	NA
Mir7231	-0.335832243	NA
Mir7235	-0.494905839	NA
Elovl2	0.298103224	0.395640848
Mir7238	0.08251524	NA
Mir7240	-0.757908635	0.930250446
Mir744	-0.478077082	0.985673938
Mir7578	0.922334605	0.955993235
Mir760	0.069451846	NA
Mir761	0.08251524	NA
Mir7646	0.08251524	NA
Wisp2	-1.0687469	0.889972515
Cacna1f	-1.069421832	0.891972665
Mir7650	-0.120584705	0.99527876
Mir7651	-0.01070275	0.998658264
Mir7652	0.08251524	NA
Mir7653	0.08251524	NA
Mir7654	0.08251524	NA
Trim30b	-1.071297224	0.944901371
Mir7657	0.08251524	NA
Mir7658	0.08251524	NA
Mir7662	0.812887549	NA
Mir7664	-0.620065749	0.924707867
Mir7665	0.947776825	NA
Adamts20	0.297717522	0.126698562
Mir7670	0.08251524	NA
Mir7672	0.08251524	NA
Mir7673	0.08251524	NA
Mir7676-1	0.08251524	NA
Mir7676-2	-0.806354819	NA

Mir7677	-0.806354819	NA
Mir7682	-0.15014944	0.99527876
Mir770	-0.177407397	0.993870257
Mir7b	0.08251524	NA
Mir8091	-0.379466967	0.976978183
Mir8092	0.040719528	NA
Mir8093	0.08251524	NA
Mir8094	0.145972101	0.985673938
Mir8097	0.082554751	0.99527876
Fam167b	-1.074321991	0.549579107
Mir8101	0.08251524	NA
Dytn	-1.074520829	0.949607377
Mir8103	0.043341216	0.997529192
Mir8104	-0.224046112	0.992385525
Mir8107	0.08251524	NA
Mir8108	0.101885033	NA
Mir8112	-0.494905839	NA
Nkd1	-0.296920173	0.353015192
Mir8115	-0.240722446	0.991996237
Mir8116	0.173256133	0.989556168
Mir877	-0.262826469	0.991996237
Mir9-2	0.08251524	NA
Mir9-3	0.088716843	NA
Eml3	0.296318816	0.237800215
Mir92b	-0.806625944	NA
Mir93	0.08251524	NA
Mir99a	-0.331901885	NA
Mir99ahg	-0.029067573	0.99527876
Mir142hg	-1.075356769	0.910328122
Mirg	-0.183285486	0.754350047
Mirlet7a-1	0.097809398	NA
Mirlet7b	0.08251524	NA
Mirlet7c-2	0.08251524	NA
Mirlet7d	0.408932853	0.985293518
Mirlet7f-1	0.08251524	NA
Pth2r	-1.07612766	0.921311891
Hpcal1	0.295897868	0.220337275
Mis12	0.490600751	0.922306337
Mis18a	-0.097092559	0.91462326
Mis18bp1	0.43955342	0.927313116
Misp	-0.758188928	0.935510251
Mitd1	0.028901143	0.988481007
Mitf	-0.611807008	0.624713015
Mki67	-0.167500929	0.962094124

Mkks	-0.083054538	0.956164302
Mkl1	0.099302369	0.707777756
Mkl2	0.061450365	0.904480183
Mkln1	0.310171271	0.959276014
Mkln1os	0.212871856	0.90358675
Mknk1	0.061227932	0.962648047
Mknk2	-0.05885835	0.953505913
Mkrn1	0.164747743	0.757721895
Mkrn2	0.112032869	0.79212401
Mkrn2os	0.197985563	0.936905058
Mkrn3	0.01835783	0.997115148
Mks1	0.243741959	0.825148733
Mkx	-0.070656482	0.984328758
Mlc1	0.082460473	0.780007405
Mlec	-0.121926009	0.714072377
AC174800.1	-1.078020642	0.975831674
Mlf2	-0.001805674	0.997241386
Mlh1	0.143371934	0.809627943
Mlh3	-0.167965572	0.624713015
Mlip	-0.11671976	0.869316117
Mlkl	0.409982634	0.961373288
Mllt1	-0.036736296	0.970892908
Mllt10	-0.00275071	0.997115148
Mllt11	0.062965182	0.895198797
Mllt3	-0.141614621	0.711190474
Mllt6	-0.141556858	0.962648047
Zfp933	-0.295705742	0.31338078
Casc4	-0.295281379	0.128640258
Mlx	0.101574479	0.91462326
Mlxip	-0.092058204	0.98526072
Mlxipl	0.198434707	0.928672524
Synm	0.294770147	0.443439702
Mmaa	0.027726508	0.980339705
Mmab	-0.048082131	0.957499962
Mmachc	-0.007591121	0.99527876
Mmadhc	-0.021469888	0.97607383
Mmd	-0.065379958	0.89703631
Mmd2	0.053358032	0.912042382
Mme	-0.149115339	0.980339705
Mmel1	0.646078918	0.894953269
Mmgt1	-0.034069806	0.968005385
Mmgt2	-0.235947265	0.721943953
Mmp11	0.24660541	0.960201673
Mmp14	-0.004794913	0.997115148

Mmp15	-0.020388199	0.987326705
Mmp16	0.082407287	0.965290366
Mmp17	-0.095725566	0.810908327
Mmp19	-0.145005244	0.974823941
Mmp2	-0.20093877	0.965290366
Mmp21	-0.419563425	0.974823941
Mmp23	0.149731204	0.976140679
Mmp24	0.001445268	0.99765039
Mmp25	-0.152375249	0.980132658
Mmp28	0.075788106	0.979037366
Mmp9	0.264990512	0.906200009
Col11a1	-0.294768643	0.452387635
Mms19	-0.039659046	0.962648047
Mms22l	-0.486157304	0.818088737
Sos2	-0.294587225	0.116655757
Mnat1	0.060395643	0.963439167
Mnd1	-0.042942912	0.99360769
Mndal	-0.248539855	0.854558027
Mns1	0.10819077	0.970461999
Mnt	-0.101201029	0.77156461
Moap1	0.002594672	0.998010523
Mob1a	0.370379393	0.911631946
Mob1b	0.308297613	0.948104382
Mob2	0.070730918	0.944972801
Gramd3	-0.294240831	0.177374543
Mob3b	-0.071889869	0.97607383
Mob3c	0.029689794	0.985742667
Mob4	0.165468464	0.871869598
Mobp	0.100348614	0.860826773
Mocos	0.266961081	0.903795193
Mocs1	-0.261219933	0.891972665
Mocs2	-0.044073149	0.969755848
Mocs3	0.054140651	0.97420657
Mog	0.099462345	0.875723186
Foxd1	-1.078926576	0.74428322
Mogs	-0.034208629	0.975831674
Mok	0.192500349	0.8226891
Mon1a	-0.181240201	0.685671097
Mon1b	-0.023347065	0.976978183
Mon2	-0.06850679	0.909728442
Morc1	-0.023540129	0.998010523
Morc2a	-0.036474925	0.962648047
Morc2b	-0.245616154	0.913640344
Morc3	-0.055073095	0.953505913

Morc4	-0.058980198	0.975831674
Morf4l1	0.084976542	0.871869598
Morf4l2	0.042924462	0.947360766
Morn1	0.183850492	0.873797321
Morn2	0.215444229	0.655744783
Zglp1	-1.082123228	0.861366392
Morn4	0.022808201	0.97607383
Morn5	-0.464856027	0.891352425
Mos	-0.279971613	0.991438757
Mosmo	-0.010071967	0.989200732
Mospd1	-0.012276957	0.994980068
Mospd2	0.025337969	0.984836179
Mospd3	0.126764587	0.936905058
Mov10	0.006970253	0.99527876
Prr29	-1.082606434	0.87847909
Moxd1	-0.015070915	0.99527876
Mpc1	-0.029446009	0.973100313
Mpc1-ps	0.21349911	0.869158427
Mpc2	0.087638667	0.91082801
Mpdu1	-0.031018751	0.975831674
Mpdz	-0.040014279	0.962648047
Mpeg1	0.041988691	0.964941885
Mpg	0.005900054	0.99527876
Mphosph10	-0.139701795	0.811219747
G2e3	-0.293299829	0.394810291
Mphosph8	-0.072483214	0.925759393
Mphosph9	-0.010155193	0.993743828
Mpi	0.103079379	0.825592611
Muc15	-1.082694449	0.948104382
Pla2g2c	-1.084999152	0.90358675
Mplkip	-0.216114136	0.869316117
Mpnd	0.126222973	0.599763688
Mpo	0.613251093	0.949607377
Mpp1	-0.075508206	0.921311891
Mpp2	-0.097664874	0.756543929
Mpp3	0.051946699	0.947033215
Mpp4	-0.351180247	0.911631946
Mpp5	0.185855011	0.860521845
Mpp6	-0.175019697	0.812431087
Mpp7	-0.267787183	0.910524966
Mppe1	-0.021184709	0.988034453
Mpped1	0.228833386	0.951390592
Mpped2	0.010516186	0.991996237
Mprip	-0.062883504	0.90458554

Mpst	0.191093349	0.682386822
Mpv17	-0.190552574	0.873753967
Kcnj3	-0.293086696	0.215724925
Mpv17l2	-0.077468855	0.962648047
Smad6	-1.088844056	0.590331212
Mpzl1	-0.038130908	0.982309415
Foxp3	-1.089098456	0.779964055
Mpzl3	0.067165975	0.968005385
Mr1	0.047990434	0.98526072
Sphk1	-1.089352538	0.702848528
Mrap2	-0.188583907	0.955908516
Mras	0.052036549	0.962648047
Ccdc175	-1.089407802	0.968005385
Mrc2	-0.197826847	0.950460219
Mre11a	-0.142419332	0.872352604
Mreg	0.270061744	0.874252042
Mrfap1	0.066165072	0.910328122
Elovl6	-0.292953472	0.139374532
Mrgpre	-0.083384557	0.962648047
Mrgprf	-0.304004416	0.97487264
Mrgprh	0.661153949	0.98459388
Mri1	0.095206731	0.905585111
Mrip-ps	-0.095255378	0.936905058
Ngp	-1.092372956	0.898478018
Mrm1	-0.096645205	0.947360766
Mrm2	0.083501289	0.956737477
Mrm3	0.073373444	0.962648047
Mrnip	0.058142175	0.971138085
Mro	-0.16088667	0.786324313
Mroh1	-0.041184143	0.968005385
Mroh2a	-0.240720326	0.914969279
Mroh3	-0.453572933	0.945526153
Mroh5	-0.426748053	0.89703631
Mroh6	0.386751495	0.973218231
Mroh7	0.235176728	0.8616433
Mroh8	0.279265062	0.948104382
Mrpl1	0.043903719	0.965520236
Mrpl10	0.028085917	0.969755848
Mrpl11	0.069734966	0.911631946
Mrpl12	0.043379117	0.957499962
Mrpl13	0.082935773	0.874422885
Mrpl14	0.351854487	0.688803555
Mrpl15	0.031365412	0.984748462
B3galt5	0.292083891	0.000179759

Mrpl17	-0.078972365	0.910328122
Mrpl18	0.134822972	0.613183359
Mrpl19	0.096926197	0.861366392
Mrpl2	0.080775797	0.921311891
Mrpl20	0.032675281	0.975800973
Mrpl21	-0.008589186	0.99527876
Mrpl22	0.172922291	0.690084266
Mrpl23	0.107100423	0.905970457
Mrpl23-ps1	0.039686805	0.980132658
Mrpl24	0.07001661	0.922889447
Mrpl27	0.13776267	0.758595939
Mrpl28	0.060410652	0.954469162
Mrpl3	0.105726967	0.89703631
Rplp1	0.291901023	0.341452788
Mrpl32	0.233863424	0.56651873
Mrpl33	0.001892027	0.99765039
Mrpl34	0.114589535	0.921154826
Mrpl35	-0.066490641	0.911631946
Mrpl36	-0.053219592	0.959659722
Mrpl37	0.074585516	0.874971335
Mrpl38	0.086781547	0.858549868
Mrpl39	0.071655829	0.89703631
Mrpl4	0.050167117	0.962648047
Mrpl40	0.015111402	0.991438757
Gab1	-0.291764886	0.24802413
Ccdc189	0.290942887	0.442068044
Mrpl43	-0.021143648	0.985673938
Mrpl44	0.142906603	0.852035098
Mrpl45	-0.088719057	0.895520088
Mrpl46	-0.032943696	0.974823941
Mrpl47	0.119321204	0.871869598
Nrip2	0.290879178	0.275969466
Mrpl48-ps	-0.034678735	0.99527876
Mrpl49	0.031678027	0.968005385
Mrpl50	0.065776522	0.928672524
Mrpl51	0.061051443	0.936031628
Mrpl52	0.162018452	0.708127282
Mrpl53	0.116906535	0.963039534
Mrpl54	-0.005645318	0.99527876
Mrpl55	0.131225218	0.915297582
Mrpl57	0.066751785	0.959276014
Mrpl58	0.088226271	0.896196194
Mrpl9	0.055085238	0.961607556
Mrps10	0.126156259	0.811913793

Mrps11	0.116214616	0.804891561
Mrps12	0.007006399	0.99527876
Mrps14	0.055510396	0.965520236
Mrps15	-0.005670777	0.995292452
Mrps16	0.125705025	0.889908105
Mrps17	0.040313704	0.961373288
Mrps18a	0.043541113	0.962648047
Mrps18b	0.090225159	0.931948968
Pcdh10	-0.290592527	0.098798554
Mrps2	0.034922957	0.97469529
Pcgf1	0.290487788	0.146214787
Mrps22	0.03862009	0.969755848
Mrps23	0.32889256	0.615496563
Mrps24	-0.122346816	0.962648047
Mrps25	-0.0041548	0.99527876
Mrps26	0.062835291	0.89703631
Mrps27	-0.068818496	0.962648047
Mrps28	-0.08515219	0.962648047
Mrps30	-0.114937911	0.80931022
Mrps31	0.016119707	0.987607832
Mrps33	0.105644271	0.749744784
Mrps34	0.053624278	0.962648047
Mrps35	-0.013694361	0.991996237
Mrps36	0.090114301	0.90918338
Srrm3os	-1.094074601	0.895520088
Mrps36-ps2	0.272268094	0.975800973
Mrps5	0.028401405	0.970461999
Mrps6	-0.212738024	0.676872366
Mrps7	0.057765144	0.936031628
Mrps9	0.018604789	0.985673938
Mrrf	-0.069790051	0.957499962
Mrs2	-0.093706956	0.815864767
Mrto4	0.178510387	0.774896252
Mrto4-ps1	0.703986596	0.774896252
Mrvi1	0.258716909	0.936031628
Mir6946	-1.095693698	0.944269511
Ms4a4b	-0.08625908	0.99527876
Ms4a6b	0.201901469	0.962648047
Ms4a6c	0.458366475	0.950460219
Ms4a6d	0.120463083	0.981392364
Fam214a	0.290438148	0.022383499
Msantd1	0.055685646	0.985742667
Msantd2	-0.406467051	0.74463793
Msantd3	0.248264952	0.548869952

Msantd4	-0.007169912	0.994960308
Msc	0.334959374	0.86128643
Msh2	-0.018747118	0.985673938
Msh3	-0.001097513	0.998010523
Msh5	0.408408067	0.968005385
Msh6	-0.114638173	0.869316117
Msi1	0.029458975	0.976546786
Msi2	0.018364892	0.98347579
Msl1	-0.161347288	0.597777294
Msl2	0.082858463	0.803910915
Msl3	-0.021303738	0.987814724
Msl3l2	0.137344476	0.835345968
Msln	0.96169931	0.7851601
AC154218.2	-1.100686134	0.911631946
Msmo1	0.007321465	0.99527876
Pif1	-1.101720987	0.811219747
Msn	-0.104558949	0.916962532
Slfn9	-1.102463736	0.879133004
Msra	-0.087535071	0.86783605
Msrb1	0.10824975	0.860521845
Msrb2	0.142352414	0.770793864
Msrb3	-0.145260503	0.89703631
Mss51	-0.079903868	0.991438757
Mst1	-0.139726698	0.992103386
Mst1r	-0.389415457	0.871869598
Mstn	0.751858628	0.852010446
Msto1	0.092484332	0.922999286
Msx1	-0.243640246	0.890704638
Mir7001	-1.104581863	0.958393136
Hist1h2ad	-1.106275483	0.927659499
mt-Atp6	0.10042652	0.84870301
mt-Atp8	0.102897535	0.895520088
mt-Co1	0.115339559	0.610808738
Crlf1	0.290128862	0.307478675
Ikzf5	-0.28982279	0.15875926
Rcbtb1	0.289726199	0.136070877
mt-Nd1	0.129682378	0.782546806
mt-Nd2	0.054383127	0.955993235
mt-Nd3	0.184208962	0.665532517
mt-Nd4	0.008006636	0.99527876
mt-Nd4l	-0.01987121	0.984328758
mt-Nd5	0.013172845	0.98771048
mt-Nd6	0.04482072	0.971138085
mt-Rnr1	0.170406516	0.605064513

mt-Rnr2	0.189464568	0.550561335
mt-Ta	0.272961863	0.758223648
mt-Tc	0.049074589	0.975831674
mt-Td	-0.048490326	0.985673938
mt-Te	0.096524223	0.91462326
mt-Tf	0.136636611	0.968005385
mt-Tg	0.069162667	0.988941187
mt-Th	0.221998316	0.891703152
mt-Ti	0.117679616	0.968005385
mt-Tk	0.088135366	0.965520236
mt-Tl1	0.139903965	0.879401677
mt-Tl2	-0.043225097	0.988034453
mt-Tm	-0.065956401	0.962648047
mt-Tn	0.245264245	0.76998058
Cnbd2	0.289369679	0.310553098
mt-Tq	0.0461481	0.989640643
mt-Tr	-0.026477149	0.993103295
mt-Ts1	-0.783211032	0.889547181
Sec61g	0.289367908	0.426667884
mt-Tt	0.282075325	0.695835819
mt-Tv	-0.041144436	0.992252966
mt-Tw	0.103815922	0.961373288
mt-Ty	0.031344364	0.985742667
Gtpbp6	0.289173954	0.496022017
Tmem184b	-0.288817709	0.237504632
Rasgrp3	-0.288664459	0.333307116
Mta1	0.00675183	0.99527876
Mta2	0.018009793	0.981392364
Mta3	0.000492054	0.999044516
Mtag2	0.390042812	0.909547396
Mtap	0.200695801	0.762953497
Mtbp	-0.049546654	0.985673938
Mtch1	0.01561297	0.985293518
Mtch2	0.100019088	0.884140163
Mtcl1	-0.221351636	0.571020232
Mtcp1	-0.143233351	0.962648047
Mtdh	-0.096619645	0.795137348
Mterf1a	0.213959926	0.860521845
Mterf1b	0.280646576	0.89703631
Mterf2	0.156878062	0.798580668
Mterf3	-0.079732272	0.944391691
Mterf4	0.112143063	0.899097089
Mtf1	-0.049980957	0.962648047
Mtf2	0.113396245	0.962648047

Mtfmt	-0.068911422	0.968977192
Mtfp1	0.062973899	0.933201883
Mtfr1	-0.036457724	0.99100709
Mtfr1l	-0.035197103	0.980339705
Mtfr2	0.262281077	0.968005385
Slc5a3	-0.288597254	0.128640258
Mtg2	0.034989841	0.974823941
Mthfd1	-0.05496451	0.945526153
Mthfd1l	-0.000500027	0.999235749
Mthfd2	0.163001377	0.938414123
Mthfd2l	-0.16840296	0.886647768
Mthfr	-0.072392723	0.932673884
Snhg8	0.288340685	0.278515147
Mthfsd	0.021249984	0.982146631
Mthfsl	0.090404798	0.962648047
Mtif2	0.052375407	0.966608583
Mtif3	0.016389408	0.987342614
Mtln	0.064933018	0.966010627
Mtm1	-0.182465371	0.860521845
Mtmr1	-0.118870633	0.881890358
Mtmr10	0.00170402	0.99765039
Mtmr11	-0.220586458	0.873797321
Mtmr12	-0.001243494	0.99765039
Mtmr14	-0.106771268	0.898474424
Cblb	0.288267007	0.052514536
Mtmr3	0.118141218	0.889908105
Mtmr4	-0.043570913	0.960951064
Mtmr6	0.008071203	0.991996237
Mtmr7	-0.033533973	0.974823941
Mtmr9	-0.114993903	0.638329646
Mto1	0.053909912	0.95363833
Mtor	-0.015219745	0.985293518
Mtpap	0.067986577	0.939052665
Rpl37	0.288175419	0.082752362
Mtr	-0.057410273	0.965520236
Mtrex	0.054552054	0.940963923
Mtrf1	0.226142235	0.651020894
Mtrf1l	-0.068891683	0.969755848
Mtrr	0.095383492	0.920642026
Mtss1	-0.103981461	0.821998633
Mtss1l	-0.104735803	0.682013498
Mttp	0.037304253	0.974809035
Mturn	-0.108584296	0.71596464
Mtus1	-0.18689996	0.615496563

Mtus2	0.014065844	0.985742667
Mtx1	0.036526906	0.974823941
Mtx2	0.02498295	0.975800973
Mtx3	-0.085199284	0.841017002
Muc1	-0.596458897	0.874971335
Zfp750	-1.11094971	0.944753667
Atf1-ps	-1.112264849	0.882096017
Muc3a	-0.040901456	0.984748462
Muc5b	-0.952275183	0.974809035
Muc6	0.158843029	0.98459388
Nck1	-0.287638413	0.36779665
Mum1	0.011527893	0.993380259
Mum1l1	0.177524415	0.946985353
Mus81	0.048688804	0.967485535
Musk	0.300156684	0.964011524
Dgki	-0.287381038	0.412816247
Mut	-0.103379618	0.891972665
Mutyh	-0.021607752	0.99527876
Mvb12a	0.102257711	0.890704638
Mvb12b	0.057765245	0.904480183
Mvd	0.056037441	0.962838652
Mvk	-0.140012949	0.851099074
Mvp	-0.087581648	0.971661054
Mx1	-0.464381054	0.962648047
Mx2	-0.242291666	0.97487264
Mxd1	-0.0634717	0.962121011
Mxd3	-0.768811578	0.707777756
Srl	0.286811901	0.357627019
Rps14	0.286736861	4.99859E-06
Mxra7	-0.066599922	0.962648047
Mxra8	-0.02319087	0.993242999
Mxra8os	0.066319115	0.99527876
Myadm	0.30222698	0.89703631
Myadml2	0.262379788	0.911631946
Myadml2os	0.745619944	0.961373288
Myb	0.337257531	0.962094124
Mybbp1a	0.058775256	0.910328122
Mybl1	-0.122947666	0.969755848
Mybl2	-0.299264621	0.932673884
Usp27x	-1.11415416	0.873797321
Mir1970	-1.114559209	0.959659722
Mybpc3	0.542052664	0.974962198
Mybph	0.628239477	0.849381566
Myc	0.141447067	0.920642026

Mycbp	-0.108526335	0.962648047
Mycbp2	-0.08366603	0.851137446
Mycbpap	0.092669213	0.984328758
Mycl	-0.140627338	0.973602213
Mycn	0.243788211	0.758271454
Myct1	-0.262874332	0.969755848
Myd88	0.119440294	0.945647427
Mydgf	0.044361118	0.964941885
Myef2	-0.025312026	0.981045362
Myg1	0.086292037	0.936031628
Olfr61	-1.119353698	0.965520236
Myh10	-0.095494206	0.815987743
Myh11	0.216313772	0.869316117
Mir6977	-1.120358914	0.962094124
Myh14	-0.101853186	0.850260513
Myh15	-0.419719982	0.702848528
Slc6a12	-1.121394935	0.590745439
Myh3	-0.305798679	0.874624896
AC133598.1	-1.121447046	0.968005385
Myh6	-0.215491506	0.928672524
Myh7	-0.07507385	0.975800973
Myh7b	0.111418379	0.942511266
Myh8	-0.280748626	0.911276003
Myh9	-0.137238153	0.59164618
Txndc2	-1.122835069	0.962648047
Casp12	-1.128409702	0.708953765
Myl12a	-0.202711163	0.852010446
Myl12b	0.1346127	0.780499133
Myl2	0.552896688	0.911631946
Snora73a	-1.130154831	0.953505913
Myl4	0.217970325	0.911631946
Scrg1	0.286495226	0.186938452
Myl6b	-0.081108197	0.962648047
Myl7	-0.216181824	0.99527876
Myl9	0.096655187	0.941515087
Olfr551	-1.131121646	0.779231312
Mylip	-0.045057312	0.97469529
Mylk	-0.065879591	0.922505072
Igfn1	-1.132171767	0.629280869
Mylk3	0.193817773	0.954174097
Mir6377	-1.133150632	0.971138085
Mir6929	-1.134524696	0.965290366
Mymk	0.151721411	0.981502501
Mynn	-0.111290055	0.905970457

Myo10	-0.003791566	0.997529192
Myo15	0.288406846	0.964011524
Mir7024	-1.136161821	0.951497773
Myo16	-0.027605891	0.984328758
Myo18a	-0.033758393	0.94925672
Myo18b	0.070168811	0.981392364
Myo19	-0.157681143	0.861397728
Myo1a	-0.934344669	0.696211886
Myo1b	-0.043862894	0.97487264
Myo1c	-0.116876322	0.87414784
Myo1d	-0.105904786	0.935897306
Myo1e	-0.205107158	0.861139061
Myo1f	-0.114077536	0.965520236
Myo1g	-0.420330111	0.950460219
Myo1h	0.39352349	0.740514972
Myo3b	-0.058178104	0.988481007
Myo5a	-0.079534757	0.852010446
Myo5b	0.057288039	0.959276014
Myo5c	0.319784447	0.951390592
Myo6	0.309556366	0.894953269
Myo7a	-0.031604321	0.980339705
Sis	-1.139634359	0.936031628
Atp5o	0.286050372	0.091203947
Myo9b	-0.134104115	0.732729469
Myoc	-0.053259764	0.994960308
Arl13a	-1.139757959	0.969755848
Myod1	0.048250313	0.99527876
Myof	-0.203151607	0.923011738
Rasa2	-0.285988221	0.19682892
Car7	-0.285642767	0.238827832
Crabp2	-1.144136255	0.764783842
Myom3	-0.231029473	0.955908516
Pkd1l1	-1.146183607	0.84277074
Six2	-1.147298512	0.905867377
Myoz2	0.613511855	0.962648047
Alx4	-1.147510664	0.759486652
Mypop	-0.078158364	0.895520088
Mypopos	0.440339015	0.849526037
Myrf	-0.092380452	0.921311891
Mir5131	-1.14785117	0.954770762
Myrip	0.032563855	0.962648047
Mysm1	0.511981428	0.92517502
Myt1	0.145030909	0.852010446
Myt1l	-0.150609751	0.578812716

Myzap	-0.52905954	0.91462326
Mzf1	-0.138790286	0.911631946
Mzt1	0.137603534	0.801926713
Mzt2	0.037247987	0.980132658
n-R5s161	-0.701179677	NA
Ptgds	-1.149725323	0.849526037
n-R5s185	-0.651950395	NA
Bcas1os1	-1.15243949	0.935897306
Mir3093	-1.15476408	0.959276014
Fam186a	-1.155192095	0.961373288
n-R5s207	-0.698426933	0.957499962
Ism2	-1.15624406	0.894430387
n-R5s39	0.6540263	0.974823941
n-R5s67	-0.410617874	0.97500491
n-R5s85	-0.813826816	0.962648047
Usp48	-0.285531089	0.482234498
N4bp2	-0.090913688	0.962648047
N4bp2l1	0.12947281	0.821998633
N4bp2l2	0.052723526	0.975831674
N4bp2os	-0.020213933	0.99527876
N4bp3	-0.286457095	0.615775357
N6amt1	0.199454553	0.854278008
Naa10	0.110995015	0.871981999
Naa15	-0.096251055	0.86783605
Naa16	-0.002193696	0.998340722
Naa20	-0.055276234	0.962648047
Donson	-0.28522206	0.499218448
Zfp658	-0.285184618	0.438549066
Naa35	0.039111643	0.962648047
Ndufa1	0.284685457	0.322813856
Naa40	0.049080955	0.962648047
Naa50	0.061560371	0.959276014
Bex3	0.284674199	0.128640258
Naa80	-0.069261643	0.968005385
Lrrtm1	-0.284349154	0.400767795
Naalad2	-0.003688992	0.998047032
Naaladl1	-0.056167166	0.99527876
Naaladl2	-0.581929435	0.813346233
Nab1	-0.097820865	0.885532627
Nab2	-0.0719975	0.948104382
Nabp1	0.047112894	0.98340069
Bcorl1	-0.284048983	0.232491866
Rps4l	0.283705676	0.482385306
Nacad	-0.101275175	0.852010446

Nacc1	0.009163393	0.987814724
Nacc2	-0.143103505	0.6883862
Oaf	-0.28312424	0.441831227
Nadk2	-0.028717911	0.97469529
Nadsyn1	0.077752039	0.971661054
Nae1	-0.039335808	0.968005385
Naf1	-0.089366526	0.936031628
Naga	-0.023620992	0.985742667
Nagk	0.203313737	0.703372057
Naglu	-0.15984499	0.871869598
Aox3	-1.156647332	0.544661714
BB031773	-1.159324901	0.962648047
Naif1	-0.071688196	0.971661054
Fbxo33	-1.15989367	0.623438925
Naip2	-0.005045651	0.998010523
Naip5	0.26045923	0.915297582
Naip6	0.145208469	0.976929237
Nalcn	-0.089996831	0.707777756
Nampt	-0.056389564	0.940431936
Nanos1	-0.102603756	0.84870301
Nanos2	0.390292489	0.886647768
Nanos3	0.330388506	0.974823941
Nanp	0.213885016	0.638329646
Nans	-0.024601694	0.985293518
Nap1l1	-0.026203037	0.970461999
Nap1l2	-0.029784595	0.965290366
Nap1l3	-0.113414161	0.777427974
Nap1l4	0.082247286	0.920609434
Nap1l5	0.017015819	0.982934928
Napa	0.068415513	0.774896252
Napb	-0.074736116	0.760547704
Napepld	0.236270328	0.965520236
Napg	-0.058870076	0.882096017
Naprt	0.210006253	0.837755777
Napsa	-0.93817574	0.655964276
Narf	0.014848169	0.984328758
Narfl	0.122384859	0.683281689
Nars	0.031063739	0.957499962
Nars2	-0.051613388	0.962648047
Nasp	0.200555937	0.711294758
Nat1	0.316891636	0.89383516
Selenbp1	-0.282782955	0.404892636
Nat14	0.196791814	0.965290366
Nat2	0.294273145	0.960176242

Nat8	-0.448343566	0.871869598
Nat8b-ps	0.178130491	0.984836179
Nat8f1	-0.256862183	0.89703631
Nat8f2	-0.291796219	0.970461999
Nat8f3	-0.081281087	0.975831674
Nat8f4	-0.138030562	0.911276003
Nat8f5	0.049297992	0.992385525
Nat8f6	-0.142278343	0.976978183
Nat8f7	0.083741997	0.987326705
Nat8l	-0.047980277	0.938615214
Nat9	0.058801503	0.968005385
Natd1	0.030947977	0.981392364
Nav1	-0.066807122	0.958389182
Elp4	-0.282327234	0.410106728
Nav3	-0.194881064	0.544661714
Naxd	0.153001305	0.571020232
Naxe	0.101842412	0.779964055
Nbas	-0.119719801	0.853090538
Cntrl	-0.282045293	0.386343119
Nbea	-0.098343293	0.619769322
Nbeal1	-0.161969971	0.69189201
Nbeal2	-0.033638412	0.980339705
Nbl1	-0.109458292	0.875723186
Nbn	-0.039957333	0.973100313
Nbr1	-0.045142858	0.91462326
Rasa1	-0.281940182	0.509250303
Ncam1	0.041182248	0.928672524
Ncam2	-0.100500589	0.909728442
Ncan	-0.021217039	0.97607383
Ncapd2	0.043915378	0.962648047
Ncapd3	-0.05858946	0.945526153
Ncapg	0.578053223	0.946985353
Ncapg2	-0.153083948	0.964011524
Ncaph	-0.097471653	0.982408617
Ncaph2	-0.132372054	0.643053315
Ncbp1	0.048598487	0.916426816
Ncbp2	-0.075022586	0.992385525
Ncbp3	-0.000960354	0.998658264
Ncdn	-0.047298255	0.91334561
Nceh1	-0.053781575	0.889972515
Plxnd1	-0.281716006	0.243596082
Ncf2	-0.032625157	0.989402966
Ncf4	0.388335959	0.899097089
Uqcc3	0.281574199	0.32150293

Nck2	-0.063903056	0.932673884
Nckap1	-0.064499197	0.890704638
Nckap1l	0.122889905	0.871869598
Nckap5	0.062819395	0.97487264
Nckap5l	-0.1200291	0.874273224
Nckap5los	-0.252821598	0.97487264
Nckipsd	-0.013241365	0.988481007
Ncl	0.071945002	0.852010446
Rps27rt	0.281278068	0.291313919
Ncmap	-0.288530383	0.970348278
Ncoa1	-0.007839716	0.988941187
Ncoa2	-0.094632905	0.767515266
Ncoa3	-0.142499201	0.810908327
F12	-1.159948245	0.858549868
Ncoa5	0.19880536	0.581475897
Ncoa6	-0.043332703	0.962648047
Ncoa7	-0.155810572	0.759365171
Ncor1	-0.079863924	0.815987743
Rpl17-ps5	0.281247975	0.404443644
Ncs1	0.012985239	0.98526072
Rbm15	-0.280767886	0.491912953
Naip1	-1.160232966	0.674357582
Ndc1	0.053588836	0.953505913
Ndc80	-0.79417501	0.875007504
Nde1	0.122201841	0.861366392
Ndel1	-0.040143719	0.956164302
Ndfip1	-0.046350076	0.932673884
Ndfip2	-0.115666132	0.701313526
Ndn	0.10441552	0.703372057
Ndnf	-0.103240894	0.936031628
Ndor1	0.266707938	0.777427974
Ndp	-0.165523391	0.837755777
Ndrg1	0.125888805	0.771450635
Ndrg2	0.040174635	0.95126064
Ndrg3	-0.052610954	0.944098708
Ndrg4	-0.020251078	0.99527876
Ndst1	-0.058452951	0.911631946
Ndst2	0.232332851	0.859472511
Mrpl16	0.280745441	0.095099902
Cryba2	-1.160557411	0.90358675
Trappc1	0.280670452	0.103621073
Ndufa10	0.041343064	0.948104382
Ndufa11	0.116249449	0.829694131
Hps1	-0.280392734	0.187264287

Rps15	0.280117382	0.002475905
Ndufa2	0.185979548	0.670918119
Rab4b	0.279580369	0.357211168
Ndufa4	0.145847542	0.632633706
Ndufa4l2	-0.209573944	0.939592821
Ndufa5	0.127614467	0.805149212
U2af1	0.279533986	0.142152696
Wdr54	0.279277798	0.169102369
Grm5	-0.279241563	0.074297594
Ndufa9	0.099847695	0.625886453
Ndufab1	0.02989176	0.980325883
Ndufab1-ps	-0.064725075	0.984328758
Ndufaf1	0.031358788	0.975831674
Ndufaf2	-0.020225904	0.991438757
Ndufaf3	0.110650561	0.855904169
Ndufaf4	0.96779344	0.6883862
Ndufaf5	0.069023082	0.928333994
Ndufaf6	0.104028846	0.953505913
Ndufaf7	0.052584718	0.938939871
Ndufaf8	-0.024864428	0.985673938
Atpif1	0.278767242	0.132404127
Taf15	0.277594683	0.015604115
Ndufb11	0.057671048	0.962648047
Ndufb2	0.013973727	0.990550129
Naaa	0.277423629	0.37706439
Ndufb4	0.112130344	0.822693227
Ndufb4c	0.229049794	0.522890184
Ndufb5	0.07552901	0.909547396
Ndufb6	0.051715059	0.948345831
Zfp462	-0.277244744	0.034088092
Stxbp5l	-0.277036879	0.41351939
Dcaf17	-0.275960258	0.318279733
Ndufc1	0.080676109	0.965007958
Ndufc2	0.174244294	0.758595939
Ndufs1	0.048503786	0.949607377
Ndufs2	-0.056984244	0.922889447
Ndufs3	0.116415102	0.729405774
Ndufs4	0.126410663	0.587203888
Smurf2	-0.275815684	0.029750576
AA414768	0.275266824	0.468229908
Ndufs7	-0.023548869	0.97487264
Ndufs8	0.130227682	0.583646785
Ndufv1	0.051888761	0.89703631
Klf15	0.275060358	0.481689886

Fmnl3	-0.274429223	0.369497288
Neat1	-0.009217753	0.99527876
Apoe	0.274287269	0.001264615
Nebl	-0.157520158	0.66923652
Necab1	-0.139129065	0.771684575
Necab2	0.052795061	0.945117894
Necab3	0.071835616	0.947360766
Necap1	-0.022203434	0.974823941
Necap2	0.041709708	0.973218231
Nectin1	0.072751372	0.948104382
Nectin2	-0.208102849	0.888622599
Nectin3	0.044967839	0.974962198
Nectin4	0.129418459	0.875723186
Nedd1	0.085104921	0.977902698
Nedd4	-0.06359588	0.99165967
Nedd4l	0.366399661	0.889972515
Hmgn2	0.274020507	0.493073764
Nedd9	-0.071666125	0.959276014
Nefh	0.002122531	0.99765039
Nefl	-0.01110691	0.985742667
Pclo	-0.273993316	0.243596082
Negr1	0.025646215	0.974823941
Neil1	0.011760389	0.99527876
Neil2	0.25341215	0.874252042
Neil3	-0.932159163	0.962648047
Iffo2	0.273980771	0.220337275
Nek10	0.132697957	0.948104382
Nek11	-0.134433643	0.968005385
Nek2	0.361263263	0.873797321
Nek3	0.11527316	0.970348278
Nek4	-0.158815864	0.721390644
Kctd21	-0.273640543	0.332957946
Nek6	0.265242687	0.571020232
Ano10	-0.273491353	0.468667381
Nek8	-0.093072852	0.972218452
Nek9	0.024137162	0.97487264
Nelfa	-0.065608815	0.936905058
Nelfb	-0.061904759	0.946239011
Nelfcd	0.018335201	0.982907766
Nelfe	-0.023271492	0.985742667
Nell1	-0.093912973	0.89703631
Nell2	-0.038457466	0.955993235
Nemf	0.072537311	0.911631946
Nemp1	-0.650945616	0.597841348

Nemp2	0.193812318	0.932673884
Nenf	0.06270675	0.948407374
Neo1	-0.052375377	0.948104382
Nepro	-0.04760998	0.98043358
Rpl32	0.273397911	0.010843046
Nespas	-0.732252519	0.972387414
Net1	0.270075023	0.578473551
Neto1	-0.07654807	0.873797321
Neto2	-0.126215475	0.861366392
Neu1	-0.019297742	0.98175286
Neu2	0.35796959	0.953505913
Neu3	-0.084034594	0.97487264
Neu4	0.194643106	0.841017002
Neurl1a	0.049961606	0.921117568
Neurl1b	-0.105472002	0.896196194
Neurl2	0.389454536	0.670918119
Neurl3	-0.583573774	0.80931022
Neurl4	-0.075128357	0.934798735
Neurod1	-0.008425829	0.99527876
Neurod2	0.013238763	0.993114821
Neurod4	-0.751442764	0.946239011
Tma16	0.273351809	0.414106926
Neurog1	0.993946752	0.911631946
Neurog2	0.02090856	0.99527876
Nexmif	0.474708651	NA
Nexn	0.572104326	0.895520088
Nf1	-0.131750835	0.688803555
Nf2	-0.081123676	0.866126508
Nfam1	0.188039777	0.890704638
Nfasc	-0.114763296	0.663732405
Nfat5	-0.067135357	0.980132658
Nfatc1	-0.223546391	0.8549794
Slc8a1	-0.273117807	0.15929297
Nfatc2ip	-0.031663698	0.982908926
Nfatc3	-0.048707069	0.971891838
Nfatc4	-0.023957518	0.99527876
Zfp456	-1.166947711	0.607400517
Nfe2l1	-0.047213927	0.935822376
Nfe2l2	-0.004611616	0.996094752
Nfe2l3	-0.118058096	0.963666931
Nfia	-0.041608398	0.962636653
Nfib	0.033568687	0.972218452
Nfic	-0.000538284	0.999242708
Nfil3	-0.221344773	0.758271454

Rps17	0.272939981	0.056924302
Nfkb1	-0.21497296	0.736771155
Nfkb2	-0.272815949	0.884259444
Nfkbia	0.202136173	0.850102526
Nfkbib	0.099940835	0.928489442
Nfkbid	-0.203646833	0.91082801
Nfkbie	-0.24882734	0.962648047
Nfkbil1	0.184702467	0.813744313
Nfkbiz	-0.220559493	0.742422553
Nfrkb	0.004510497	0.99527876
Nfs1	0.07511547	0.871869598
Glul	0.27276932	0.000324248
Nfx1	0.103629037	0.642397758
Nfxl1	0.040353024	0.975831674
Nfya	-0.068770189	0.944040391
Nfyb	-0.015893474	0.98459388
Pik3ip1	0.272530564	0.103621073
Ngb	0.321989935	0.939060335
Ngdn	0.020410184	0.985742667
Ngef	-0.044779605	0.919785445
Ngf	0.167183337	0.895520088
Ngfr	0.234061931	0.948520404
Ngly1	0.028534068	0.980852848
Dppa2	-1.168195646	0.882096017
Ngrn	0.061261355	0.951390592
Nhej1	0.066008043	0.98526072
Nhlh1	0.238748115	0.934798735
Nhlh2	0.206374522	0.915230696
Nhlrc1	0.169138793	0.839488666
Nhlrc2	-0.182747514	0.837755777
Zbtb34	-0.272201006	0.224597392
Cfap97d1	-1.170765028	NA
Rps10-ps2	0.272155696	0.18316876
Nhs	0.172033248	0.956164302
Nhsl1	-0.207694967	0.975831674
Nhsl2	0.014214215	0.99100709
Nicn1	0.094466136	0.69189201
Nid1	-0.084751849	0.97469529
Nid2	-0.232609574	0.915106125
Nif3l1	0.235046709	0.629280869
Nifk	-0.042838461	0.968005385
Nim1k	0.009851057	0.994960308
Rps3	0.272116651	2.42541E-05
Ninj1	-0.37976587	0.744046401

Ninj2	0.185175629	0.965520236
Ninl	-0.176038759	0.76998058
Nip7	0.157800829	0.673443553
Nipa1	-0.02749667	0.972374093
Nipa2	-0.132097903	0.816844103
Nipal2	-0.172147743	0.909547396
Nipal3	-0.039659904	0.962648047
Nipal4	-0.316055796	0.852275035
Nipbl	-0.024316394	0.985293518
Nipsnap1	0.117640169	0.722843611
Mt3	0.272019827	0.119226239
Nipsnap3b	0.208042807	0.568915531
Nisch	-0.040384021	0.93912725
Nit1	0.07443994	0.935897306
Nit2	0.017317931	0.991996237
Nkain1	-0.10593859	0.944074069
Nkain2	0.121219196	0.562965533
Nkain3	0.127104286	0.959659722
Rpl6l	0.271963878	0.346053851
Nkap	-0.152040936	0.721943953
Nkapd1	-0.209410613	0.902293377
Nkapl	-0.549390145	0.91462326
Dgat2	0.271900679	0.220442078
Nkd2	0.093279878	0.936031628
Nkiras1	0.079958031	0.862094492
Nkiras2	0.050717384	0.99100709
Nkpd1	0.311475087	0.936507368
Nkrf	-0.089019165	0.871869598
Nktr	-0.120105044	0.974962198
Nkx2-1	0.004153537	0.998840551
Nkx2-2	-0.475239767	0.702951568
Nkx2-2os	-0.435900655	0.961373288
Nkx3-1	0.370371802	0.860521845
Carmn	-1.171582906	0.950195611
Nkx6-2	0.3100809	0.7037205
Nle1	0.07035749	0.962648047
Nlgn1	-0.068622249	0.936031628
Nlgn2	0.015941561	0.985446046
Nlgn3	0.00435706	0.99527876
Armh4	-0.271828204	0.105964011
Nln	-0.045271589	0.966010627
Nlrc3	-0.467793607	0.852010446
Nlrc4	-0.766143226	0.87806697
Nlrc5	-0.329329818	0.947033215

Lgals1-ps2	-1.171611398	0.911631946
Papolb	-1.174493781	0.731132047
Nlrp1a	0.114412893	0.99527876
Nlrp1b	0.399407921	0.958389182
Nlrp1c-ps	0.656403768	0.962648047
Nlrp3	0.221435583	0.962648047
Nlrp4f	-0.92781723	0.946985353
Nlrp5-ps	0.092364977	0.99527876
Nlrp6	-0.136005783	0.974962198
Nlrx1	-0.165061917	0.872685246
Nmb	0.192935287	0.955993235
Nmbr	0.193514213	0.932673884
Nmd3	-0.005302593	0.99527876
Nme1	0.011293493	0.991996237
Nme2	0.020278008	0.98691726
Nme3	-0.03667608	0.985673938
Nme4	0.015632833	0.99527876
Nme5	0.011367451	0.99527876
Nme6	0.013541955	0.99527876
Nme7	-0.010012388	0.994960308
Nek5	-1.175738949	0.736130784
Nmi	-0.050133291	0.981683975
Nmnat1	0.127064899	0.898478018
Nmnat2	0.116536779	0.670918119
Nmnat3	0.333936084	0.810908327
Nmral1	0.094331403	0.962648047
Nmrk1	0.083505323	0.961373288
Nmrk2	0.049488881	0.99640101
Nmt1	-0.000263809	0.999235749
Nmt2	-0.13524772	0.624713015
Nmur1	-0.782854104	0.956737477
Nnat	-0.024395315	0.985673938
Nnmt	0.312458182	0.961373288
Nnt	-0.06896108	0.94370905
Noa1	0.016648206	0.99100709
Caskin2	-0.271597914	0.314095916
Noc2l	0.018168321	0.981502501
Noc3l	0.020442157	0.985673938
Noc4l	0.076414538	0.929997774
Vwa1	-0.271423315	0.462575025
Nod1	-0.142024517	0.89703631
Nod2	-0.203261972	0.98526072
Nodal	-0.172474747	0.981641687
Nog	-0.291340686	0.684977158

Nol10	-0.062368086	0.924448385
Nol11	-0.070032958	0.948104382
Nol12	0.149843627	0.610726714
Nol3	-0.065580592	0.971661054
Nol4	-0.089100392	0.891265024
Nol4l	-0.099038019	0.854558027
Nol6	-0.072627786	0.860521845
Nol7	-0.163626913	0.6883862
Nol8	0.029926305	0.980132658
Nol9	0.008274637	0.99527876
Nolc1	-0.139563759	0.895198797
Nom1	0.007240972	0.994960308
Nomo1	-0.016120774	0.98526072
Nono	0.046536822	0.921311891
Rpl31	0.271339906	0.058966988
Nop14	0.039407338	0.965520236
Nop16	-0.027850344	0.985673938
Nop2	-0.021206411	0.985446046
Commd5	0.270887021	0.438549066
Nop56	0.116841491	0.648241111
Nop58	-0.006746543	0.99527876
Nop9	-0.167600054	0.786324313
Nos1	-0.207328299	0.962648047
Nos1ap	-0.108035453	0.6883862
Nos2	0.481324394	0.924361537
Tmem136	-0.270844072	0.404892636
Nosip	0.125755085	0.591756211
Kcnq2	-0.270573325	0.19334431
Notch1	-0.048295629	0.970461999
Notch2	-0.037863641	0.980339705
Notch3	-0.073616133	0.965520236
Mphosph6	0.270542675	0.41351939
Notum	-0.275876551	0.92517502
Notumos	0.602201733	0.928489442
Ptbp3	-0.270528572	0.349136927
Nova1	0.034433607	0.972218452
Alcam	-0.270353744	0.082752362
Nox1	0.421763587	0.987260085
H60b	-1.176738906	0.965520236
Noxo1	-0.532846063	0.834315076
Noxred1	-0.218660869	0.962648047
Npas1	-0.15121486	0.871869598
Npas2	-0.062155292	0.956072972
Npas3	-0.312178701	0.875995577

Npas4	-0.115502659	0.932673884
Npat	-0.074972868	0.951390592
Npb	0.784700004	0.861139061
Npbwr1	-0.414983335	0.720841863
Npc1	0.084488712	0.868415875
Npc1l1	-0.333437984	0.948104382
Npc2	0.019265371	0.981502501
Npcd	-0.103491836	0.975800973
Npdc1	0.015508864	0.987342614
Npepl1	0.079047865	0.956922504
Npepps	-0.217392927	0.620124996
Npff	0.145524003	0.974823941
Rpl36a-ps2	0.270300866	0.186938452
Nphp1	0.04214469	0.965520236
Nphp3	-0.154358623	0.865884071
Nphp4	0.042569897	0.973100313
Nphs1	0.657231289	0.540221783
Nphs1os	0.321494737	0.971661054
Nphs2	-0.618694232	0.963323228
Npl	-0.045605953	0.985293518
Nploc4	-0.038860655	0.959659722
Npm1	0.059949193	0.906200009
Npm2	0.397086825	0.951468402
Npm3	0.230547735	0.825148733
Ovca2	0.270247891	0.420373478
Npnt	0.210395498	0.86783605
Nppa	-0.501259463	0.942511266
Nppc	-0.00697988	0.99765039
Npr1	0.049751724	0.978063275
Npr2	-0.015450852	0.990550129
Npr3	0.060573086	0.963666931
Nprl2	0.170110518	0.581475897
Nprl3	0.076722562	0.958389182
Npsr1	0.637502699	0.962636653
Nptn	-0.100472597	0.710375942
Nptx1	0.053412089	0.928672524
Nptx2	-0.253903484	0.708626104
Nptxr	0.020448782	0.980325883
Npw	0.186115066	0.98340069
Npy	0.133815274	0.596982103
Npy1r	0.144158878	0.804540244
Npy2r	-0.007765036	0.99527876
Ihh	-1.178699381	0.69189201
Npy5r	0.24193595	0.614267464

Nqo1	0.082000021	0.969755848
Nqo2	-0.152106902	0.891972665
Nr1d1	-0.117450532	0.665532517
Nr1d2	-0.08379848	0.80931022
Nr1h2	0.130712496	0.817988462
Nr1h3	-0.116650781	0.962812066
Nr1h4	0.213238891	0.992052613
Nr1i3	0.489108561	0.872352604
Nr2c1	-0.025281438	0.989378233
Nr2c2	-0.042442286	0.959659722
Nr2c2ap	0.094117093	0.941398449
Zdhhc23	-0.270177529	0.104879534
Nr2f1	-0.033401578	0.978063275
Nr2f2	0.038919121	0.980132658
Nr2f6	-0.073175446	0.962648047
Pdap1	0.269972535	0.485743931
Nr3c2	0.061841348	0.992385525
Sdhaf3	-0.269861357	0.386030517
Fam131b	0.269132416	0.431061843
Nup210l	-1.181493809	0.813499809
Nr6a1	-0.177647929	0.689698377
AC162182.1	-1.181603347	0.942781802
Nradd	0.223718021	0.957499962
Spata48	-1.181609167	0.961887657
Nrarp	0.046830993	0.968005385
Nras	0.111498125	0.956164302
Nrbf2	-0.041536664	0.965520236
Nrbp1	0.070963858	0.889972515
Nrbp2	0.029295361	0.966608583
Nrcam	-0.090195719	0.713014362
Nrd1	-0.002914833	0.99527876
Nrde2	0.021831568	0.98771048
Fam162a	0.269111963	0.419287643
Nrf1	-0.049549273	0.993872582
Nrg1	-0.072824821	0.985446046
Nrg2	0.125648581	0.962648047
Nrg3	-0.031224308	0.971138085
Zbbx	-1.184207528	0.874422885
Nrgn	0.054695267	0.956922504
Ilk	0.268790404	0.1403916
Pxn	-0.26877061	0.121839657
Nrip3	-0.021179782	0.975387279
Nrl	0.425954633	0.897162816
Nrm	-0.388396267	0.837755777

Nrn1	-0.053445587	0.965520236
Nrn1l	-0.90715187	0.932673884
Nrp1	-0.090342453	0.873797321
Nrp2	0.000605005	0.998840551
Nrros	0.031855785	0.988034453
Nrsn1	0.032794968	0.957499962
Nrsn2	-0.014980039	0.989402966
Nrtn	0.000951757	0.999486844
Nrxn1	-0.123853175	0.690084266
Nrxn2	0.172946465	0.833916587
Nrxn3	-0.123526357	0.823072541
Nsa2	0.101620764	0.873797321
Nsd1	-0.083453599	0.873797321
Rpl17	0.268737447	0.013696201
Nsd3	-0.034287593	0.971138085
Nsdhl	0.095547337	0.873797321
Nsf	0.005881546	0.992385525
Nsfl1c	0.119433162	0.529312432
Nsg1	-0.005496784	0.99527876
Nsg2	0.062568304	0.8549794
Nsl1	0.187959973	0.961887657
Nsmaf	-0.039720871	0.962648047
Nsmce1	0.132443566	0.84870301
Nsmce2	0.010938339	0.99527876
Nsmce3	0.066245407	0.900330211
Nsmce4a	0.062575095	0.950460219
Nsmf	0.02886074	0.965011162
Nsrp1	0.14574154	0.824368034
Nsun2	0.050131135	0.90358675
Nsun3	-0.016208273	0.991438757
Nsun4	-0.007630927	0.99527876
Nsun5	0.168311044	0.7851601
Nsun6	-0.043107256	0.981392364
Nsun7	-0.038575507	0.980896205
Nt5c	0.127912601	0.767515266
Nt5c1a	0.04437106	0.98691726
Nt5c2	-0.016602244	0.98526072
Nt5c3	0.056297335	0.936031628
Nt5c3b	0.059774037	0.955401619
Nt5dc1	-0.194875683	0.924361537
Nt5dc2	0.128315124	0.935890893
Nt5dc3	-0.061770381	0.833916587
Nt5e	-0.130088495	0.961373288
Nt5m	-0.02173678	0.985293518

Ntan1	0.124328649	0.813552442
Ntf3	0.31630072	0.6883862
Ntf5	0.758812249	0.951390592
Nthl1	0.335323753	0.80931022
Ntm	0.029738238	0.97487264
Ntmt1	-0.531720105	0.544661714
Ntn1	-0.08653713	0.962648047
Ntn3	-0.170526781	0.89011487
Ntn4	-0.296567055	0.756543929
Ntn5	0.30387596	0.85787868
Fam163b	0.268658058	0.481098488
Ntng2	-0.003216141	0.997241386
Ntpcr	-0.155281477	0.878577494
Btbd18	-1.184392233	0.936972904
Ntrk2	-0.052785067	0.87847909
Ntrk3	-0.073337743	0.819545118
Nts	0.53151554	0.842256259
Ntsr1	-0.34261852	0.706406235
Ntsr2	-0.019008621	0.980896205
Nuak1	-0.101022516	0.885128793
Nuak2	-0.248345452	0.938673121
Nub1	-0.015140566	0.98187819
Klhl13	-0.268558814	0.456330081
Nubp2	0.170201501	0.638785294
Nubpl	0.025285038	0.987342614
Nucb1	-0.028839176	0.969653237
Nucb2	-0.031839032	0.976978183
Nucks1	0.078837984	0.871869598
Nudc	0.051726775	0.905585111
Nudc-ps1	0.095666908	0.91221238
Nudcd1	-0.107055907	0.875119971
Nudcd2	0.009794786	0.99527876
Nudcd3	0.317955523	0.89703631
Nudt1	0.593012366	0.55918966
Nudt10	-0.116921076	0.898474424
Nudt11	-0.028368193	0.980896205
Nudt12	-0.008332674	0.996094752
Nudt13	0.046020811	0.979037366
Nudt14	-0.041143518	0.984769634
Nudt15	0.092470573	0.956164302
Nudt16	-0.19926649	0.544661714
Nudt16l1	-0.041099554	0.97469529
Nudt17	-0.139190411	0.874971335
Nudt18	0.094445136	0.876612181

Nudt19	0.159733226	0.5840348
Nudt2	0.137347806	0.754350047
Nudt21	-0.041470245	0.961887657
Nudt22	0.239150358	0.762817527
Nudt3	0.050689084	0.948407374
Nudt4	-0.03920314	0.950460219
Nudt5	-0.100195786	0.932673884
Nudt6	-0.11835379	0.973100313
Nudt7	-0.166128762	0.938394547
Nudt8	0.320127233	0.810946393
Nudt9	0.147155364	0.654893207
Nuf2	-0.218184419	0.860142574
Nufip1	-0.052711045	0.962648047
Cox7c	0.268359169	0.09162748
Numa1	-0.068013385	0.903441375
Numb	0.145210925	0.959276014
Numbl	-0.149217817	0.544661714
Nup107	0.006572595	0.99527876
Nup133	-0.069276968	0.89703631
Nup153	-0.15644638	0.660781615
Nup155	0.013334818	0.991996237
Nup160	-0.100612496	0.89703631
Nup188	-0.126000166	0.881771584
Nup205	-0.133642556	0.728564969
Rpl30	0.268068578	0.02460799
Sfta3-ps	-1.187042547	0.936031628
Slit3	-0.26797534	0.431061843
Nup35	0.178514645	0.781552764
Nup37	0.116995455	0.956675687
Nup43	-0.100493995	0.964941885
Nup50	-0.116441592	0.740240396
Nup54	-0.018503204	0.985742667
Nup62	0.477822174	0.87847909
Hp	-1.187434727	0.870812051
Nup85	-0.076499751	0.899097089
Nup88	0.111147285	0.74428322
Nup93	0.086920605	0.947033215
Nup98	-0.066684117	0.891972665
Nupl2	0.012001998	0.994960308
Nupr1	0.177617191	0.910328122
Nupr1l	0.449595885	0.895520088
Nus1	-0.044367413	0.959659722
Nusap1	0.337083943	0.865547585
Nutf2	-0.179591538	0.935510251

Fgfr3	0.267954154	0.353015192
Nutf2-ps2	0.134159564	0.963943597
Nutm1	-0.077806778	0.99527876
Nvl	-0.014364938	0.985742667
Nwd1	-0.268634027	0.736130784
Nwd2	-0.06604143	0.948104382
Rrp15	0.267947387	0.337479705
Mrap	-1.191592425	0.779964055
Ppfia1	-0.267923827	0.279860152
Bex2	0.267779275	0.002739963
Fam189a1	-1.192343287	0.527794911
Nxnl2	0.492674321	0.981045362
Nxpe2	0.012519784	0.998121731
Nxpe3	-0.049232418	0.957485565
Nxpe4	-0.177459763	0.889972515
Nxph1	-0.045006382	0.968379096
Nxph2	0.324050574	0.910328122
Nxph3	0.504811059	0.89011487
Nxph4	-0.605107756	0.810908327
Nxt1	0.274680156	0.779231312
Nxt2	-0.044344882	0.962648047
Nyap1	0.045658058	0.951390592
Nyap2	-0.160288452	0.849424799
Nynrin	0.111509328	0.959276014
Nyx	0.384967534	0.895520088
Oacyl	0.001696869	0.999242708
Agtrap	-0.26756466	0.497690258
Oard1	0.064970136	0.962648047
Oas1a	0.411657484	0.911631946
Oas1b	-0.306992524	0.957849305
Oas1c	-0.031601296	0.99527876
Oas1g	-0.118684085	0.994960308
Hist1h4c	-1.19247299	0.884629276
Nfatc2	-0.267446543	0.357080756
Oasl1	0.572887303	0.968005385
Oasl2	0.263609162	0.882824434
Oat	0.005755559	0.99527876
Oaz1	0.013145447	0.991996237
Oaz1-ps	0.024909598	0.984836179
Mcat	0.267394883	0.41351939
Oaz2-ps	0.361168937	0.966010627
Oaz3	0.422242947	0.891972665
Clec4d	-1.203096094	0.821998633
Arntl	-0.266752745	0.19334431

Obsl1	0.027014813	0.98459388
Oca2	0.489935203	0.899249451
Ocel1	-0.017421183	0.987814724
Ociad1	0.002018826	0.996873492
Ociad2	0.020411163	0.985742667
Ocln	0.150502121	0.89703631
Ocrl	-0.039802886	0.932673884
St3gal4	0.266620679	0.186938452
Odc1	0.107293382	0.737672778
Odf2	0.011692094	0.992669879
Odf2l	-0.120382339	0.948407374
Odf3	-0.733167303	0.962648047
Odf3b	0.068564322	0.985742667
Odf3l2	0.553614644	0.942812446
Odr4	0.044413532	0.965520236
Ofd1	0.089469789	0.939592821
Ogdh	-0.020669922	0.974962198
Ogdhl	0.022466691	0.975831674
Ogfod1	-0.077198578	0.736130784
Ogfod2	-0.187451954	0.841017002
Ogfod3	-0.13274687	0.949607377
Ogfr	0.069745203	0.911631946
Ogfrl1	-0.107640891	0.709960234
Ogg1	-0.133644218	0.928099251
Ogn	-0.225658128	0.959276014
Ogt	-0.114766413	0.558458137
Oip5	0.569016479	0.633028652
Mir369	-1.20942783	0.89703631
Ola1	0.037246921	0.958375046
Olfm1	0.07282751	0.871869598
Olfm2	-0.09084572	0.944753667
Olfm3	-0.279311742	0.654893207
Olfm4	0.296949288	0.795563654
Olfml1	-0.216017977	0.767515266
Olfml2a	-0.810441948	0.562965533
Olfml2b	0.085944238	0.912042382
Olfml3	-0.083240193	0.994960308
Olfr1029	-0.679287873	0.97487264
Olfr1033	0.916524277	0.961373288
Olfr111	-0.060913726	0.99527876
Olfr1152	0.399740545	0.980132658
Olfr1279	-0.425810724	0.989640643
Olfr1344	0.132972413	0.98340069
Dsc2	-1.211129602	0.715274321

Olfr1347	-0.457461793	0.98459388
Cd300ld2	-1.21347856	0.84870301
Olfr1349	0.717952754	0.935897306
Olfr1366	0.086780168	0.992385525
Olfr1372-ps1	0.32050269	0.962648047
Olfr1388	-0.652664816	0.974962198
Olfr1392	-0.530453586	0.985742667
Matn1	-1.213495511	0.962648047
Olfr1402	-0.940727289	0.969755848
Olfr1417	0.115105533	0.985673938
Olfr1418	-0.455572574	0.982204947
BC147527	-1.214086696	0.962648047
Olfr1420	0.516050543	0.968005385
Olfr1428	-0.530262535	0.985742667
Olfr1511	-0.922279056	0.97487264
Olfr1535	0.348189823	0.980419928
Knl1	-1.215712621	0.928489442
Mfap3	-1.220340457	0.544661714
Tbx4	-1.223414023	0.613183359
Olfr286	-0.054428675	0.995411429
Olfr287	0.325074671	0.690084266
Olfr288	0.125936485	0.98526072
Olfr31	0.187873756	0.98526072
Olfr316	-0.750837187	0.777427974
Olfr317	0.916373036	0.936031628
Olfr374	-0.201975878	0.990915306
Olfr45	-0.633982125	0.98459388
Olfr46	-0.340474642	0.974823941
Olfr460	-0.576817402	0.962648047
Nek7	-1.229075309	0.634318752
Cpa5	-1.235338323	0.965290366
Olfr5	-0.586304619	0.981392364
Olfr523	0.366409639	0.985742667
Esm1	-1.236563208	0.896069965
Mir1258	-1.237971847	NA
Olfr53	0.294703523	0.98459388
Eif3s6-ps1	-1.241097017	0.911631946
Pllp	-0.26624985	0.431890949
Olfr543	-0.79379535	0.868415875
Snord60	-1.244992683	NA
Olfr545	0.620646199	0.965520236
Olfr55	-0.546163591	0.965520236
Olfr550	0.213624458	0.945182261
Med4	0.265968983	0.213423291

Trib3	-1.252763575	0.889908105
Pjvk	-1.255267273	0.958893576
Olfr56	0.070158051	0.996369585
AC151292.1	-1.258022834	0.882096017
Gfra2	0.265783957	0.432230256
Olfr624	0.189892907	0.985673938
Tnfrsf17	-1.261020702	0.915106125
AC130718.2	-1.263122572	0.774896252
Cd40	-1.268090897	0.654327196
Olfr688	0.878575171	NA
Olfr690	0.389394109	0.985742667
Kcnk7	-1.26902431	0.936031628
Olfr692	-0.079003158	0.99527876
Olfr750	-0.496320967	0.98302504
Aipl1	-1.269125542	0.962648047
Ttc6	-1.273447056	0.962648047
Olfr784	-0.580527834	0.971138085
Olfr856-ps1	0.991899454	0.874252042
Olfr691	-1.275176829	0.895198797
Olfr920	0.253399054	0.953505913
Cep112it	-1.27633151	0.928957615
Olig1	0.062660463	0.932673884
Olig2	0.138711072	0.815662897
Oma1	0.005259269	0.997258205
Phactr1	-0.265481847	0.39534064
Omg	-0.022234282	0.976978183
Omp	0.453697915	0.91462326
Onecut1	-0.106364433	0.985673938
Cadps	-0.265058753	0.323819286
Cyp24a1	-1.276525898	0.939060335
Opa1	-0.099445601	0.620124996
Opa3	-0.046543691	0.956922504
Opalin	-0.071166951	0.962121011
Opcml	-0.106187248	0.661553809
Ophn1	-0.104456474	0.816025904
Oplah	-0.01239542	0.993103295
Opn1mw	-0.871730664	0.962648047
Opn1sw	0.801018748	0.976530608
Opn3	-0.323062835	0.962648047
Opn4	-0.810168045	0.829694131
Oprd1	-0.372974173	0.561625181
Oprk1	-0.070094983	0.992385525
Oprl1	-0.034349881	0.966213117
Pcare	-1.278320807	0.932673884

Optc	-0.089107706	0.991996237
Optn	0.095013081	0.923011738
Orai1	-0.142885651	0.932673884
Ufd1	0.264814947	0.236655597
Orai3	-0.125128349	0.872157363
Orc1	-0.203441427	0.974823941
Orc2	-0.036241647	0.973100313
Orc3	-0.004766483	0.99527876
Orc4	-0.026913742	0.974823941
Orc5	-0.06458209	0.950252558
Orc6	0.163338429	0.609167039
Ormdl1	0.109528979	0.932673884
Ormdl2	0.095716079	0.970461999
Ormdl3	0.137765619	0.716433689
Os9	0.009254985	0.989840188
Osbp	-0.094356501	0.76998058
Rps18	0.264296341	0.04538814
Osbpl10	0.040464729	0.979037366
Osbpl11	-0.100818237	0.861366392
Osbpl1a	-0.015049659	0.988034453
Osbpl2	-0.047741782	0.932673884
Osbpl3	-0.060409153	0.965520236
Osbpl5	0.161659175	0.744936214
Osbpl6	0.053633612	0.994960308
Osbpl7	0.109915936	0.956164302
Pou3f1	-0.264294866	0.459482348
Osbpl9	0.048892025	0.944391691
Oscar	0.188845532	0.946239011
Oscp1	0.050122944	0.956164302
Oser1	0.255019462	0.574956844
Osgep	-0.101958921	0.908554604
Osgepl1	0.145522965	0.886647768
Myo15b	-1.27865192	0.683281689
Osgin2	-0.117100164	0.894953269
AC124466.1	-1.280572798	0.949111839
Osmr	-0.219021927	0.892605308
Osr1	-0.14061183	0.974823941
Rpl41	0.264259182	0.278279958
Ostc	-0.025515678	0.976978183
Ostf1	0.092505474	0.911834483
Ostm1	-0.02796553	0.965290366
Ifi44	-1.283681882	0.814276256
Otoa	-0.840404673	0.903441375
Otof	0.171785616	0.958389182

Otogl	0.038692586	0.988941187
Otop1	0.348766918	0.983166509
Otop2	0.794353853	0.953505913
Pcdh12	-1.286717827	0.618152081
Otos	0.125777822	0.984748462
Otub1	0.036765093	0.968005385
Otub2	-0.095133863	0.86783605
Otud1	-0.193613114	0.737087313
Otud3	-0.064945834	0.969476616
Otud4	-0.144644558	0.521202205
Otud5	0.067674521	0.932467947
Otud6b	-0.009874346	0.988208387
Otud7a	0.0164917	0.985673938
Otud7b	0.064920265	0.932673884
Zc3h3	-0.264133272	0.370298654
Otulinl	0.123363434	0.777427974
Otx1	-0.15281924	0.936031628
Otx2	0.951934328	0.740833211
Tmem145	0.263789107	0.355665731
Ovgp1	0.220921893	0.86783605
Ovol2	0.463571285	0.955993235
Oxa1l	0.045074554	0.947033215
Oxct1	-0.028668834	0.957499962
Oxct2b	0.181776635	0.994120754
Oxgr1	-0.120210834	0.98526072
Oxld1	-0.210536126	0.868415875
Oxnad1	-0.296220325	0.837755777
Oxr1	0.319998699	0.873753967
Oxsm	0.05613171	0.968005385
Oxsr1	-0.104163894	0.798109023
Oxtr	0.058813126	0.973168955
P2rx1	-0.505654385	0.911631946
P2rx3	-0.20846771	0.956922504
P2rx4	-0.118565687	0.962648047
P2rx5	0.155422472	0.970461999
P2rx6	-0.265640939	0.898474424
P2rx7	0.066280605	0.973100313
P2ry1	-0.163360083	0.968630572
P2ry10b	0.510775382	0.922306337
P2ry12	-0.245823339	0.819726438
P2ry13	-0.113855412	0.911631946
P2ry14	0.095038453	0.976978183
P2ry2	0.139017861	0.980132658
P2ry6	0.09782307	0.968630572

P3h1	0.047067017	0.981045362
P3h2	-0.029126883	0.992654978
P3h3	-0.2364018	0.723108545
P3h4	0.02152434	0.988481007
Gsg1l	0.263761375	0.44386328
P4ha2	0.077986843	0.924448385
P4ha3	-0.556342877	0.661553809
P4hb	0.013025198	0.985742667
P4htm	0.088257931	0.837755777
Pa2g4	0.060697106	0.879401677
Pabpc1	0.070712	0.889512303
Pabpc1l	0.449865457	0.953588521
Pabpc1l2a-ps	-0.216554196	0.804540244
Pabpc1l2b-ps	-0.097056966	0.901566742
Pabpc4	0.058996141	0.949607377
Pabpc4l	-0.878352041	0.911631946
Pabpc5	-0.550626144	0.941398449
Midn	-0.263758841	0.262204978
Pabpn1l	0.063555011	NA
Pacrg	0.040791412	0.974823941
Ext1	-0.263692053	0.282435203
Pacs2	-0.544568455	0.813552442
Pacsin1	0.468400142	0.871869598
Pacsin2	0.139836146	0.932673884
Pacsin3	0.029824225	0.984232465
Padi2	-0.203424524	0.619684138
Padi6	0.566600639	0.975831674
Paf1	0.080609431	0.858549868
Pafah1b1	-0.474635092	0.901129754
Pafah1b2	-0.024684741	0.971138085
Pafah1b3	0.050788138	0.981641687
Pafah2	-0.159520403	0.962648047
Pag1	0.028204586	0.980226283
Wdr72	-1.286741396	0.89703631
Pagr1b	0.605049966	0.891352425
Paics	0.024254597	0.971661054
Paip1	-0.036985332	0.969755848
Phactr4	0.263509419	0.452669584
Paip2b	0.040098235	0.940431936
Pak1	0.046100274	0.933285475
Pak1ip1	0.080264517	0.894953269
Pak2	-0.081925715	0.872352604
Pak3	-0.056195064	0.898478018
Pak4	0.333999651	0.534461098

Pak6	-0.073764574	0.959659722
Pak7	-0.134116103	0.785603619
Pakap	0.214673919	0.976546786
Palb2	-0.055049672	0.98623785
Pald1	-0.093689113	0.911631946
Palld	0.080489362	0.965520236
Palm	-0.025594572	0.965520236
Palm2	0.090776494	0.982392834
Palm3	-0.163827577	0.955993235
Palmd	-0.11852122	0.849517436
Pam	-0.040892252	0.925788724
Pam16	0.140381788	0.911631946
Pamr1	0.203285655	0.902409791
Pan2	-0.014625776	0.991996237
Ndufv3	0.263498371	0.184761973
Panct2	0.155375254	0.936031628
Pank1	-0.082885777	0.927659499
Pank2	0.239063584	0.882824434
mt-Co3	0.263435133	0.105302431
Pank4	-0.045709952	0.962648047
Pantr1	-0.297141447	0.603499717
Pantr2	-0.235681938	0.898474424
Panx1	-0.184318382	0.630498632
Panx2	0.195311129	0.736130784
Paox	-0.057051605	0.966213117
Papln	-0.794260033	0.873797321
Frmd8	-0.263220726	0.445106721
Hpse2	-1.290794985	0.911631946
Papolg	-0.103370546	0.889908105
Pappa	0.165122293	0.965520236
Pappa2	0.017982742	0.997115148
Papss1	-0.054254738	0.932673884
Dnajc4	0.263087797	0.442068044
Dstyk	-0.262559535	0.115563764
Paqr4	0.005855519	0.99527876
Paqr5	0.28677725	0.8549794
Usp31	-0.261631592	0.035000584
Paqr7	0.446511483	0.8643613
Slc4a8	-1.295920237	0.578812716
Paqr9	-0.041913745	0.962648047
Pard3	-0.037978882	0.98459388
Pard3b	-0.001147334	0.99900438
Pard6a	-0.250870506	0.833916587
Pard6b	0.213644322	0.831837418

Pard6g	-0.100533485	0.965520236
Parg	-0.019279518	0.98459388
Park7	0.163720835	0.520320584
Dpy19l3	-0.261501134	0.19334431
Parm1	-0.124391411	0.66923652
Parn	0.084082439	0.86467336
Parp1	-0.06024906	0.901479608
Parp10	-0.084053996	0.980419928
Parp11	-0.018534809	0.985673938
Parp12	-0.081144756	0.957849305
Klhl25	-0.261492366	0.455841215
Parp16	0.107248079	0.948104382
Parp2	0.068466354	0.944391691
Parp3	0.054415208	0.985742667
Parp4	-0.36632786	0.544661714
Parp6	0.008102574	0.989840188
Parp8	-0.01911568	0.985673938
Parp9	-0.085372714	0.965520236
Parpbp	0.144251866	0.98459388
Pars2	0.244393436	0.861366392
Particl	0.240879433	0.571020232
Parva	-0.416358378	0.962648047
Parvb	0.229649743	0.56488584
Parvg	0.216581675	0.653736313
Pask	-0.147786343	0.921311891
Pate2	-0.140421667	0.985742667
Lockd	-1.296141562	0.860142574
Patj	0.031110599	0.989556168
Patl1	-0.09593356	0.886647768
Patz1	-0.05360034	0.968005385
Spem1	-1.300284671	0.956164302
Pawr	0.256948048	0.87806697
Pax2	-0.19565801	0.980226283
Pax5	0.097627471	0.990661552
Pax6	-0.195813661	0.882824434
Pax6os1	0.056542319	0.99527876
Paxbp1	-0.074615768	0.907572296
Paxip1	0.079244616	0.874252042
Paxx	0.134816129	0.911631946
Pbdc1	-0.006054273	0.995944208
Pbk	0.959836286	0.661553809
Pbld1	0.565520015	0.968005385
Btg1	0.261432296	0.417001009
AF357399	-1.305275011	0.801926713

Foxc2	-1.307434191	0.740833211
Pbx1	-0.072519176	0.909547396
Pbx2	0.108278996	0.783246276
Pbx3	-0.128676852	0.953505913
Pbx4	0.278406885	0.605310774
Pbxip1	-0.074346456	0.889972515
Galnt5	-1.309317583	0.688142448
Pcbd1	0.066153847	0.974809035
Pcbd2	0.274557231	0.724910334
Pcbp1	0.054548958	0.911834483
Pcbp2	0.063879727	0.848380246
Rps27	0.261352129	0.349775121
Pcbp4	-0.138030783	0.911631946
Pcca	-0.07282865	0.904851193
Pccb	0.04693871	0.934135315
Kat6b	-0.261298949	0.400756364
Tspan18	0.260967941	0.392219282
Pcdh11x	0.446376955	0.871869598
Fam187a	-1.311780667	0.873797321
Pcdh15	-0.406968734	0.510932065
Pcdh17	-0.096309338	0.89260868
Pcdh18	-0.256801063	0.911631946
Pcdh19	0.430223729	0.956072972
Pcdh20	-0.039683097	0.962648047
Pcdh7	-0.738087657	0.921473132
Pcdh8	-0.330875483	0.953505913
Pcdh9	0.269637416	0.721943953
Pcdha1	-0.180803029	0.969661076
Pcdha11	-0.428917614	0.596982103
Pcdha12	0.076513175	0.965520236
Pcdha2	0.470005492	0.54080899
Pcdha3	-0.159437335	0.956922504
Boc	-0.26061799	0.449949801
Pcdha5	0.096326277	0.965520236
Pcdha6	-0.213493269	0.93178771
Pcdha7	-0.197063114	0.910196388
Pcdha8	-0.059725789	0.994960308
Pcdha9	-0.37111298	0.550739492
Pcdhac1	-0.199320785	0.868415875
Unc13b	-0.260281544	0.200754354
Dnah12	-1.315261638	0.777427974
Pcdhb10	-0.07088242	0.975831674
Pcdhb11	-0.112798756	0.962648047
Pcdhb12	-0.14629053	0.923011738

Pcdhb13	-0.052091131	0.981045362
Papss2	-0.260107162	0.144051788
Pcdhb15	-0.236128103	0.90358675
Pcdhb16	0.014876944	0.99527876
Pcdhb17	-0.210710377	0.771684575
Pcdhb18	-0.057076054	0.97487264
Rpl39l	-1.315307319	0.956922504
Pcdhb2	-0.045709276	0.991996237
Pcdhb20	-0.096033326	0.936031628
Pcdhb21	-0.113949306	0.99527876
Pcdhb22	0.09994775	0.994960308
Pcdhb3	-0.067290434	0.981392364
Pcdhb4	-0.378725887	0.744046401
Pcdhb5	0.070167765	0.971661054
Pcdhb6	-0.368233897	0.852010446
Pcdhb7	-0.286762669	0.721943953
Pcdhb8	-0.04792337	0.985293518
Pcdhb9	0.191108632	0.631007755
Pcdhga1	0.065306661	0.965520236
Pcdhga10	-0.100273385	0.957499962
Pcdhga11	0.140287586	0.881185267
Pcdhga12	-0.079984839	0.962648047
Pcdhga2	-0.259748787	0.654893207
Pcdhga3	0.015235857	0.99527876
Pcdhga4	0.560840735	0.965520236
Pcdhga5	-0.329233613	0.6883862
Pcdhga6	-0.246193002	0.736486324
Pcdhga7	-0.095589803	0.914675447
Pcdhga8	-0.049999257	0.991996237
Olfr544	-1.320347875	0.905585111
Adam33	-1.321196291	0.947447204
Pcdhgb2	-0.333434001	0.665532517
Fam83g	-1.32181993	0.916287981
Pcdhgb5	-0.161324472	0.953505913
Pcdhgb6	-0.107586419	0.920642026
Pcdhgb7	0.017603191	0.994960308
Pcdhgb8	-0.65147035	0.966312931
Pcdhgc3	-0.288778891	NA
Pcdhgc4	-0.178676276	0.922887207
Pcdhgc5	-0.113576921	0.742969797
Pced1a	0.029140117	0.980731359
Pced1b	0.083609053	0.98526072
Pcf11	0.128823204	0.740794505
Atox1	0.259870143	0.421157413

Pcgf2		0.317690884	0.674542577
Pcgf3		-0.050424215	0.959276014
Pcgf5		-0.156729576	0.985742667
Pcgf6		-0.048663404	0.968005385
Pcid2		0.071395328	0.948104382
Pcif1		0.074868949	0.860521845
Pck2		0.026092219	0.98526072
Pclaf		-0.413914521	0.949551444
Dusp19		-0.25986103	0.48945043
Pcm1		-0.14641732	0.723108545
Pcmt1		-0.072957484	0.869316117
	44621	-0.259575273	0.353015192
Pcmtd2		-0.437611846	0.74463793
Pcna		0.050585116	0.958389182
Pcna-ps2		0.13368658	0.954543296
Pcnp		-0.051249968	0.932673884
Pcnt		-0.123301195	0.815987743
Pcnx		-0.271708511	0.911631946
Pcnx2		-0.125892439	0.625275976
Pcnx3		-0.133219299	0.776927656
Pcnx4		0.010327517	0.991438757
Pcolce		-0.640379403	0.731867452
Pcolce2		0.306544899	0.642397758
Pcp2		0.015863271	0.998010523
Psma6		0.259483915	0.020326008
Pcp4l1		0.067346648	0.909547396
Pcsk1		-0.154269306	0.786324313
Pcsk1n		-0.042075611	0.974962198
Pcsk2		-0.026863462	0.961373288
Pcsk2os1		0.114999939	0.968497942
Pcsk2os2		-0.806658582	0.813346233
Pcsk4		-0.121493521	0.97469529
Pcsk5		0.118022533	0.932673884
Pcsk6		0.070426757	0.956164302
Pcsk7		0.014051434	0.99527876
Mir7055		-1.325620917	0.932673884
Pctp		-0.170377553	0.956072972
Pcx		-0.089098933	0.758595939
Pcyox1		-0.060838833	0.890704638
Pcyox1l		-0.104609921	0.934135315
Pcyt1a		-0.078173715	0.993380259
Pcyt1b		0.029991217	0.965520236
Pcyt2		-0.012986264	0.985673938
Borcs8		0.259320534	0.480408134

Pdcd10	0.028483425	0.980339705
Pdcd11	-0.111252388	0.758595939
Pdcd2	-0.094037945	0.962648047
Pdcd2l	0.041799519	0.976978183
Pdcd4	-0.118100882	0.852010446
Pdcd5	0.13184757	0.854278008
Pdcd5-ps	0.278324322	0.69189201
Pdcd6	-0.040230346	0.951390592
Pdcd6ip	-0.093664717	0.66923652
Pdcd7	0.106961026	0.798580668
Pdcl	-0.04642267	0.965520236
Pdcl3	0.125945713	0.827471691
Pde10a	-0.575313589	0.833916587
Pde11a	-0.353368029	0.644545791
Pde12	-0.150613717	0.708001635
Pde1a	-0.211930544	0.933201883
Pde1b	0.105194205	0.813552442
Pde1c	0.013066272	0.99527876
Pde2a	-0.021530644	0.984328758
Pde3a	0.231121652	0.873797321
Pde3b	-0.185420033	0.634318752
Pde4a	-0.030403367	0.969755848
Pde4b	0.011769686	0.989200732
Pde4c	-0.025540071	0.99527876
Pde4d	-0.076012282	0.968005385
Pde4dip	-0.087873527	0.775426591
Pde5a	-0.186984386	0.723500005
Pde6a	-0.947737119	0.969755848
Pde6b	0.190925634	0.989200732
Lox	-1.328314083	0.874252042
Akap10	-0.259110581	0.106206739
Pde6g	0.278403531	0.97469529
Pde6h	-0.23884037	0.965520236
Pde7a	-0.179155694	0.52033017
Pde7b	0.133795879	0.938544177
Pde8a	-0.091259411	0.946907532
Pde8b	0.002662989	0.995717436
Pde9a	0.052480422	0.970357584
Pdf	-0.105572583	0.988034453
Pdgfa	-0.105976163	0.911631946
Faim	0.258965004	0.121443772
Pdgfc	0.027722038	0.985742667
Pdgfd	0.323129325	0.84870301
Pdgfra	0.068006974	0.922887518

Pdgfrb	-0.149460283	0.872954356
Pdgfrl	-0.192670198	0.967507473
Pdha1	-0.003073142	0.99527876
Pdhb	0.074545623	0.832619865
Pdhx	-0.012541223	0.988034453
Pdia2	-0.357819649	0.974823941
Pdia3	-0.051747957	0.911631946
Pdia4	-0.041341695	0.971138085
Pdia5	-0.091061427	0.975631569
Pdia6	-0.085532186	0.877016623
Pdik1l	-0.094294198	0.934798735
Pdk1	0.009254094	0.993103295
Pdk2	0.115397124	0.665532517
Pdk3	0.058270177	0.943422244
Pfkfb2	-0.2588426	0.49943116
Pdlim1	0.021657959	0.993839396
Pdlim2	0.281666361	0.659923463
Fmod	-1.33067524	NA
Pdlim4	0.08356693	0.968005385
Pdlim5	0.010942558	0.989840188
Pdlim7	-0.170120312	0.702951568
Pdp1	0.082813419	0.843305383
Pdp2	0.25797648	0.684925922
Tnrc18	-0.258622648	0.010934674
Pdpn	-0.049760755	0.980731359
Pdpr	-0.182980564	0.677111529
Pdrg1	0.096813452	0.932673884
Pds5a	0.22200403	0.965290366
Pds5b	-0.111078669	0.759365171
Pdss1	0.220656756	0.522113371
Pdss2	0.024438082	0.985742667
Pdxdc1	-0.03862216	0.962648047
Prkca	-0.25860591	0.021002465
Pdxk-ps	0.740854943	0.976546786
Pdxp	0.024226796	0.984328758
Pdyn	0.302635675	0.707520358
Pdzd11	0.084145422	0.889972515
Pdzd2	-0.010174445	0.99527876
Pdzd3	-0.002629721	0.998658264
Pdzd4	0.059633933	0.930250446
Pdzd7	0.21963122	0.911631946
Pdzd8	-0.033753541	0.964941885
Pdzd9	0.085445311	0.989840188
Pdzk1	-0.121463078	0.986877982

Pdzph1	0.066130491	0.985742667
Pdzrn3	0.2749164	0.7851601
Pdzrn4	-0.213610162	0.924361537
Pea15a	0.100758176	0.786324313
Peak1	-0.09304492	0.80818949
Peak1os	0.102400042	0.989428475
Pear1	-0.065501687	0.985742667
Pebp1	0.037037108	0.946985353
Pecam1	0.038967348	0.984328758
Pecr	0.052150078	0.975290119
Pef1	-0.009377159	0.993103295
Peg10	0.182461535	0.736486324
Kcnv2	-1.332117362	0.875007504
Peg13	-0.064001029	0.897878088
Peg3	-0.085852178	0.826209871
Peli1	-0.013894784	0.988090086
Peli2	-0.041000519	0.973100313
Peli3	-0.039767637	0.974809035
Pelo	-0.107768868	0.960201673
Pelp1	0.023733868	0.984232465
Pemt	-0.071290943	0.984836179
Penk	-0.149918961	0.883333293
Pepd	0.000707545	0.998658264
Per1	-0.166088542	0.65012378
Kank1	0.258558044	0.240817904
Per3	0.017037308	0.985742667
Perm1	0.218250493	0.868564766
Perp	-0.167568331	0.962648047
Pes1	0.013887083	0.985742667
Rpl27	0.258323968	0.10579256
Pet117	-0.836157097	0.959276014
Pex1	0.078355061	0.916962532
Pex10	0.046573459	0.968005385
Zfp90	0.258284256	0.323832374
Pex11b	-0.125652167	0.951497773
Pex11g	0.02587746	0.993380259
Pex12	-0.201272494	0.852010446
Pex13	-0.124791529	0.683629533
Cdh10	-0.258198753	0.362846678
Pex16	0.104384177	0.881407916
Pex19	0.286957791	0.514712196
Pex2	0.193131422	0.529257466
Pex26	0.450495224	0.702951568
Ank1	-0.258189606	0.417721745

Pex5	-0.054958643	0.947745582
Pex5l	-0.097692014	0.948345831
Mrps18c	0.25791835	0.260775968
Pex7	-0.09850158	0.844084751
Pf4	-0.796773596	0.971138085
Pfas	0.034133227	0.97487264
Pfdn1	0.182332963	0.623042338
Ubl5	0.257130989	0.02035027
Pex11a	0.257110578	0.431061843
mt-Co2	0.256681697	0.168279006
Pfdn6	0.105049291	0.861366392
Pfkfb1	0.226883726	0.965290366
Cic	0.256404727	0.010934674
Pfkfb3	0.347184237	0.56399886
Pfkfb4	-0.015338432	0.99527876
Pfkl	-0.103467745	0.780007405
Pfkm	0.088132382	0.655744783
Pfkp	0.081850134	0.740833211
Pfn1	-0.012860494	0.991438757
Pfn2	-0.022381226	0.972218452
Pfn4	0.064854626	0.980339705
Pgam1	0.014580415	0.982113252
Pgam1-ps1	-0.184784092	0.985742667
Pgam1-ps2	-0.087480009	0.859989558
Pgam2	0.178572281	0.883426561
Pgam5	-0.02368902	0.978063275
Pgap1	-0.116070473	0.886647768
Pgap2	-0.004897131	0.99765039
Pgap3	0.031986614	0.984328758
Pgbd1	-0.042676681	0.987342614
Dctn6	0.256341995	0.089553702
Pgd	0.123999714	0.826681377
Lpar1	-0.25613546	0.479970543
Pgghg	0.015477606	0.99527876
Arpp19	0.256051455	0.434676934
Pgk1	0.017640554	0.983219084
Pgk1-rs7	0.058226057	0.894953269
Pgls	0.052995646	0.962648047
Pglyrp1	0.247265763	0.858549868
Tceal5	0.256004599	0.090130915
Pgm2	0.102324443	0.956072972
Pgm2l1	-0.105778271	0.715274321
Pgm3	0.067881002	0.959276014
Pgm5	-0.129759659	0.909547396

Pgp	-0.061410929	0.956922504
Pgpep1	0.199265478	0.641233173
Pgpep1l	0.113689641	0.980226283
Uqcrb	0.255954631	0.155421558
Pgrmc1	0.063420734	0.873797321
Pgrmc2	0.01712043	0.984748462
Pgs1	0.07755474	0.870485449
Chmp2a	0.255924942	0.038340894
Phactr2	-0.466402339	0.932673884
Phactr3	0.008201971	0.991996237
Polr1c	0.255905513	0.19783713
Phax	0.10108632	0.866564418
Phb	0.079052688	0.83886846
Phb2	0.093027811	0.815987743
Phc1	-0.034927888	0.965520236
Phc2	-0.066030021	0.882096017
Phc3	0.61674488	0.962648047
Pheta1	-0.042046412	0.99527876
Pheta2	0.429209858	0.968005385
Phex	0.17276595	0.98340069
Phf1	-0.088867699	0.895520088
Phf10	0.036803107	0.959276014
Phf11b	-0.229740472	0.97487264
Phf11c	0.611518024	0.826209871
AC132460.3	-1.333107425	0.517425857
Phf12	-0.086549945	0.858549868
Phf13	-0.110709203	0.89703631
Pdgfb	-0.255465839	0.410106728
Phf19	0.089761714	0.985673938
Phf2	0.022955995	0.975831674
Phf20	-0.092526607	0.779231312
Phf20-ps	0.124903533	0.985673938
Phf20l1	0.069638916	0.91507125
Phf21a	0.093534151	0.962648047
Phf21b	-0.390991753	0.596729594
Phf23	-0.053363688	0.961373288
Mir219a-2	-1.333940045	0.891111468
Phf2os1	0.391060066	0.879096011
Phf3	-0.120106941	0.528197276
Phf5a	0.164884585	0.732729469
Phf6	-0.138268931	0.822597041
Phf7	-0.013829405	0.99527876
Phf8	-0.01606184	0.985293518
Phgdh	0.000152282	0.999486844

Phip	-0.155985608	0.782546806
Phka1	0.09318425	0.815987743
Phka2	0.01633526	0.991996237
Phkb	-0.063564765	0.910328122
Fnip1	-0.254641643	0.084102849
Phkg2	0.083991329	0.84870301
Phlda1	-0.09385627	0.949607377
Phlda3	0.119146863	0.911631946
Xk	-0.25447892	0.213895263
Sertad4	-1.334098438	NA
Phlpp1	0.029600217	0.965151504
Phlpp2	0.386910113	0.860521845
Phospho1	0.107631046	0.965520236
Rpl36a	0.253824898	0.209384449
Lmbrd2	-0.25380551	0.220337275
Phrf1	-0.05857881	0.969755848
Phtf1	-0.134739003	0.610307639
Phtf1os	-0.517880774	0.948104382
Phtf2	-0.021549396	0.988034453
Phyh	-0.027564989	0.974823941
Phyhd1	0.169931734	0.871869598
Phyhip	-0.007718143	0.991438757
Phyhipl	0.01405854	0.98340069
Phykpl	0.09291212	0.953505913
Pi15	0.01901691	0.99527876
Gpr143	-1.334534482	0.87564489
Pi4k2a	-0.074806968	0.825910982
Pi4k2b	0.193762959	0.948407374
Pi4ka	-0.081581193	0.848380246
Pi4kb	0.036314392	0.974962198
Pianp	-0.123712392	0.800711882
Pias1	-0.068279663	0.90358675
Pias2	-0.096280658	0.779231312
Pias3	-0.019712459	0.985446046
Pias4	0.150411593	0.810908327
Pibf1	0.090240117	0.957499962
Picalm	0.054036995	0.897878088
Pick1	0.173686631	0.596982103
Pid1	-0.018583917	0.985229869
Pidd1	0.182506214	0.968005385
Piezo1	-0.061147012	0.980339705
Piezo2	-0.144656398	0.981250052
Cyb5r2	-1.336416822	0.689698377
Olfr1346	-1.33981425	0.947602075

Piga	-0.322990801	0.555447881
Pigb	0.11743228	0.98459388
Pigc	-0.224128358	0.932673884
Pigf	0.031780525	0.985742667
Pigg	-0.013031552	0.991866617
Pigh	0.022035992	0.985742667
Pigk	-0.070522224	0.992385525
Pigl	-0.177084123	0.84870301
Pigm	0.13865545	0.868797028
Pign	0.051805104	0.971138085
Pigo	-0.197709451	0.73481128
Pigp	0.208939723	0.759365171
Pigq	-0.201839965	0.807017299
Pigs	-0.038807237	0.968005385
Pigt	-0.045284244	0.962648047
Pigu	-0.113431486	0.871869598
Pigv	0.123540081	0.962648047
Pigw	-0.22966273	0.855344864
Pigx	0.199513589	0.852010446
Pigyl	0.172752933	0.65012378
Pigz	-0.220332335	0.90358675
Pih1d1	0.043940651	0.968005385
Pih1d2	0.060180171	0.985673938
Pih1h3b	-0.697818887	0.962648047
Pik3ap1	-0.503932685	0.571020232
Pik3c2a	-0.012819762	0.991559279
Pxmp4	0.25356686	0.480408134
Pik3c2g	0.298335987	0.984836179
Pik3c3	-0.008395464	0.994960308
Wdr46	-0.253366426	0.480840274
Pik3cb	0.021312141	0.991438757
Pik3cd	-0.033982248	0.97487264
Pik3cg	0.119881383	0.962648047
Tmsb10	0.253246193	0.15929297
Pik3r1	0.077056841	0.87847909
Pik3r2	-0.006766029	0.991996237
Pik3r3	-0.055281655	0.927313116
Pik3r4	-0.109309799	0.740833211
Pik3r5	-0.480561767	0.727420355
Pik3r6	0.219529276	0.918407269
Pikfyve	-0.180605166	0.510932065
Pilra	-0.280049515	0.962094124
Pim1	0.803361694	0.803093104
Pim2	0.010421859	0.992669879

Pim3	0.022600594	0.985742667
Pimreg	0.278999716	0.965520236
Pin1	0.023360477	0.981392364
Pin4	0.176962084	0.703633347
Pink1	0.070657079	0.89703631
Pinx1	-0.032475076	0.989840188
Pip4k2a	-0.019283268	0.976978183
Pip4k2b	-0.088794965	0.825249193
Pip4k2c	-0.123239421	0.695239733
Pip4p1	-0.071704905	0.974823941
Pip4p2	-0.122108208	0.621993647
Pip5k1a	0.208961029	0.950195611
Pip5k1b	-0.048106427	0.968005385
Pip5k1c	-0.589973126	0.864150749
Pip5kl1	0.08252787	0.981205299
Pipox	-0.203550309	0.959276014
Pir	-0.017223157	0.99527876
Pirb	-0.573150365	0.962648047
Pirt	0.152744623	0.962648047
Pisd	-0.180536135	0.968859834
Pisd-ps1	0.095660807	0.858549868
Pisd-ps2	0.073267827	0.916287981
Pithd1	0.026580511	0.975800973
Pitpna	0.023098042	0.980419928
Pitpnb	-0.012770656	0.985446046
Pitpnc1	0.003877609	0.99527876
Pitpnm1	-0.029244672	0.968005385
Pitpnm2	0.135512579	0.841017002
Mir3069	-1.341725213	0.936031628
Pitpnm3	-0.070397599	0.922157348
Pitrm1	-0.014185935	0.985742667
Piwil1	-0.35477983	0.985673938
Piwil2	-0.093807543	0.985673938
Pja1	-0.11709134	0.901840668
Pja2	-0.098377129	0.549850404
Tmc3	-1.344544582	0.708626104
Pkd1	-0.115250421	0.874422885
Tmem174	-1.345453219	0.882096017
Slfn4	-1.353448694	0.894264807
Pkd1l3	0.299342268	0.874809001
Pkd2	-0.081767307	0.783611405
Pkd2l1	0.075473176	0.991438757
Pkd2l2	-0.238962792	0.568220567
Pkdcc	0.098740036	0.944753667

Pkdrej	0.233323226	0.941515087
Pkhd1	0.092180409	0.99527876
Pkhd1l1	-0.8494733	0.774896252
Pkia	0.116400613	0.750303621
Pkib	-0.075492565	0.985673938
Pkig	0.144648299	0.95070766
Pklr	0.524018979	0.984232465
Pkm	0.00151893	0.997625755
Pkmyt1	-0.301597588	0.868415875
Pkn1	0.227647304	0.810908327
Pkn2	-0.476331596	0.558118096
Pkn3	-0.032295144	0.99527876
Pknox1	-0.070292808	0.962648047
Pknox2	0.122487032	0.849381566
Pkp2	0.023983035	0.985477412
Bmp15	-1.353734966	0.952385926
Pkp4	-0.05002795	0.909458076
Pla1a	-0.137220461	0.968005385
Stmn4	0.253227067	0.207659271
Pla2g15	0.005722918	0.998121731
Pla2g16	0.028199931	0.984836179
Ccdc150	-1.356155995	0.813947253
Pla2g2d	-0.048288797	0.99527876
Pla2g2f	0.246601668	0.860826773
Plch2	-0.252958685	0.049860765
Pla2g4a	-0.335978544	0.889972515
Rhoq	-0.252788448	0.193510645
AC122413.1	-1.358315731	0.894430387
Pla2g4d	-0.229822907	0.992385525
Pla2g4e	0.338453941	0.544361994
Pla2g4f	-0.280240568	NA
Pla2g5	-0.099449622	0.985742667
Pla2g6	-0.072252799	0.959659722
Pla2g7	-0.003870867	0.99527876
Pla2r1	-0.542550325	0.889972515
Plaa	0.013166518	0.985742667
Vpreb3	-1.358461802	0.947981239
Plac9a	-0.249717112	0.968005385
Plac9b	0.076114621	0.991438757
Plag1	0.065843372	0.981392364
Plagl1	0.118388736	0.873753967
Plagl2	0.046543134	0.97487264
Plat	0.193335867	0.543640901
Platr14	-0.290748511	0.98526072

Platr17	0.279007792	0.959276014
Nlrp12	-1.361248015	0.87847909
Platr23	-0.307238335	0.985673938
Platr25	0.258362713	0.865486573
Platr31	-0.869263407	0.974278441
Fam13b	-0.252742657	0.056924302
Platr8	-0.441495784	0.985033402
Plau	-0.208784407	0.953401321
Plaur	-0.147231919	0.975800973
Plb1	0.077800536	0.980132658
Plbd1	0.757309375	0.971661054
Plbd2	-0.076936558	0.871869598
Pfdn2	0.252515635	0.115563764
Plcb2	-0.071035272	0.98340069
Plcb3	-0.145375565	0.859910936
Plcb4	-0.173338229	0.928957615
Plcd1	0.141123446	0.90358675
Plcd3	-0.120617023	0.910328122
Plcd4	0.146130162	0.721943953
Plce1	0.126572793	0.932673884
Plcg1	-0.137729598	0.861909929
Plcg2	0.272926714	0.6883862
Plch1	-0.063192836	0.974823941
Rpl7a	0.252046013	0.000772356
Plcl1	-0.081513928	0.932673884
Plcl2	-0.123897696	0.74463793
Plcxd1	0.286729217	0.799858326
Plcxd2	0.032668701	0.984232465
Plcxd3	-0.450368386	0.936031628
Plcz1	-0.288817764	0.925928378
Arhgap20	0.252027567	0.460614424
Pld2	-0.078222917	0.962648047
Pld3	-0.03697339	0.961373288
Pld4	-0.045515038	0.983078916
Pld5	-0.016575625	0.99527876
Pld6	0.245699658	0.968005385
Plec	-0.094083897	0.895198797
Plek	-0.172219881	0.851373921
Fam47e	-1.364623015	0.831358057
Plekha1	0.040660874	0.975831674
Plekha2	0.24977784	0.710772024
Plekha3	-0.041619409	0.957499962
Plekha4	-0.222815397	0.959659722
Plekha5	0.036368433	0.965520236

Rpl35a	0.251954449	0.045503629
Plekha7	-0.015140371	0.99527876
Plekha8	-0.098978638	0.86783605
Plekhb1	-0.20012715	0.959276014
Plekhb2	-0.007253533	0.994960308
Med21	0.251608198	0.294902721
Plekhf1	0.427134309	0.5649195
Plekhf2	0.245585447	0.6883862
Plekhg1	-0.205053223	0.980896205
Plekhg2	-0.183746608	0.903538125
Plekhg3	0.015627406	0.994960308
Plekhg4	0.12648196	0.972374093
Plekhg5	0.027477623	0.968977192
Plekhg6	0.627843855	0.980896205
Plekhh1	-0.12127361	0.911631946
Plekhh2	-0.125163595	0.872265039
Plekhh3	0.03248835	0.988553438
Plekhj1	0.1299388	0.89703631
Plekhm1	-0.123917595	0.752097504
Plekhm2	-0.05134821	0.949607377
Plekhm3	-0.174283193	0.544661714
Plekhn1	-0.051788481	0.962648047
Plekho1	0.234676288	0.59684147
Dot1l	-0.251210025	0.442302609
Plekhs1	-0.405762957	0.90358675
Nags	-1.365813983	0.962648047
Papola	-0.251095888	0.064081282
Plgrkt	-0.10662789	0.891853883
Plin1	-0.093382465	0.985742667
Plin2	-0.14728218	0.935131589
Plin3	0.114077915	0.89703631
Srms	-1.374775641	0.670918119
Plin5	0.019447937	0.99527876
Plk-ps1	-0.287512949	0.918235054
Plk1	0.032254475	0.994960308
Plk2	-0.104718833	0.821998633
Plk3	-0.180241841	0.889908105
Plk4	-0.116003338	0.962648047
Plk5	-0.036950785	0.985673938
Romo1	0.25106071	0.322635312
Pln	-0.231091462	0.975831674
Adi1	0.250926553	0.146214787
Plod2	0.003920478	0.99765039
Plod3	-0.077090056	0.904480183

Plp1	-0.037816431	0.972025261
Plp2	0.160577871	0.942511266
Plpbp	-0.021715245	0.979459279
Plpp1	-0.013638561	0.994960308
Plpp2	0.243500236	0.946985353
Plpp3	-0.10047567	0.685353017
Sirt3	0.250650305	0.160993185
Plpp5	0.253125514	0.871869598
Plpp6	-0.132493716	0.825148733
Plpp7	-0.373392123	0.740833211
Plppr1	0.20040853	0.882096017
Plppr2	-0.104959253	0.850260513
Plppr3	0.231578096	0.655744783
Plppr4	-0.078439498	0.873797321
Rab8b	-0.250439905	0.161844023
Plrg1	0.004414832	0.99527876
Pls1	0.069317763	0.965520236
Pls3	0.08359895	0.962648047
Plscr1	-0.635703939	0.740514972
Plscr2	0.298974133	0.911631946
Plscr3	0.102670011	0.916938833
Plscr4	0.2731775	0.759365171
Pltp	-0.143756346	0.710375942
Plvap	0.057965846	0.971455475
Plxdc1	0.089367569	0.965520236
Plxdc2	0.096562701	0.89703631
Fam120c	-0.250287255	0.235649093
Plxna2	-0.125372093	0.6883862
Plxna3	-0.042004934	0.983797678
Plxna4	-0.054339947	0.911631946
Plxna4os1	0.352980651	0.985673938
Plxnb1	0.020341472	0.980339705
Plxnb2	-0.123525167	0.655744783
Plxnb3	-0.069044073	0.962648047
Plxnc1	-0.118392068	0.774896252
Ranbp2	-0.250228686	0.128015512
Pm20d1	-0.26750668	0.822693227
Pm20d2	-0.06326067	0.980339705
Pmaip1	0.357182368	0.89703631
Eppk1	-1.374951604	0.771684575
Pmel	-0.820289351	0.861397728
Pmepa1	-0.097064239	0.871869598
Pmf1	0.133739559	0.965290366
Pmfbp1	-0.797551067	0.828102037

Pml	0.345376976	0.529257466
Pmm1	0.015225858	0.984748462
Pmm2	-0.017698435	0.985742667
Pmp22	-0.118901732	0.889972515
Pmpca	0.040753527	0.948104382
Pmpcb	0.110677893	0.74463793
Pms1	-0.18304631	0.953219396
Pms2	0.229325641	0.873797321
Pmvk	0.025901542	0.980339705
Pnck	0.112854231	0.777427974
Pnisr	0.201593016	0.731137993
Pnkd	-0.045269735	0.942812446
Pnkp	-0.000749883	0.99900438
Pnldc1	0.053196312	0.991438757
Sun3	-1.37880479	0.877275726
Pnliprp2	0.515243563	0.980339705
Pnma1	0.16436035	0.936031628
Pnma2	0.077013384	0.991996237
Pnma3	0.069560866	0.895520088
Pnma5	0.166343379	0.966213117
Pnmal1	-0.079571678	0.826789198
Pnmal2	0.102223589	0.660781615
Pnmt	0.175643259	0.994960308
Pnn	0.042274491	0.965520236
Pno1	-0.080660447	0.91462326
Pnoc	0.048427616	0.980339705
Pnp	-0.174016766	0.749626226
Pnp2	-0.271190115	0.89011487
Pnpla1	-0.852720249	0.922073518
Supt4a	0.250148871	0.090763381
Pnpla3	-0.350678816	0.73432031
Pnpla6	-0.016508098	0.985673938
Pnpla7	-0.028262792	0.984328758
Pnpla8	-0.082561541	0.961669173
Pnpo	0.02131133	0.98340069
Pnpt1	-0.11562929	0.854278008
Pnrc1	0.060945632	0.935897306
Pnrc2	-0.016455021	0.985742667
Poc1a	0.122060365	0.883333293
Poc1b	-0.333774318	0.779964055
Poc5	-0.105911181	0.730883419
Podn	-0.224511013	0.868415875
Podnl1	-0.116813376	0.99527876
Podxl	-0.060832264	0.965520236

Podxl2	0.020591315	0.991438757
Pof1b	-0.193811006	0.979459279
Pofut1	-0.198215053	0.7851601
Pofut2	-0.235583022	0.962648047
Pogk	0.22581462	0.710375942
Poglut1	-0.03788141	0.956164302
Pogz	-0.069527785	0.911631946
Pola1	-0.06662189	0.971661054
Pola2	0.092988258	0.962648047
Polb	0.086328754	0.874971335
Pold1	-0.165409651	0.948104382
Pold2	0.052250287	0.965520236
Pold3	0.032717325	0.974823941
Pold4	0.06715659	0.968005385
Poldip2	0.024723205	0.968630572
Poldip3	0.024372434	0.975831674
Pole	-0.095796227	0.971661054
Pole2	-0.271771731	0.974823941
Pole3	0.053939713	0.939060335
Pole4	-0.095711504	0.9354258
Polg	0.066188708	0.944074069
Polg2	0.14873043	0.950252558
Polh	-0.115652422	0.945440711
Poli	-0.039878873	0.97487264
Cep350	-0.249909999	0.186859869
Poll	-0.170836765	0.813346233
Polm	0.093462587	0.950460219
Poln	-0.783105593	0.842993113
Polq	0.119032715	0.974085322
Polr1a	-0.009582898	0.993103295
Polr1b	-0.09487356	0.898533163
Fat3	-0.249855001	0.359948476
Polr1d	0.127187687	0.79403092
Polr1e	0.087169126	0.961607556
Polr2a	-0.049619075	0.924361537
Polr2b	0.020187859	0.974987859
Polr2c	-0.083050418	0.935890893
Polr2d	0.035395975	0.980419928
Top3b	0.249709346	0.311609026
Polr2f	0.256959318	0.609167039
Polr2g	0.0587269	0.942812446
Polr2h	-0.012856327	0.993103295
Polr2i	0.279770015	0.573810203
Timm8b	0.249636575	0.151118156

Smarca2	0.249605616	0.235649093
Polr2l	0.103816391	0.91462326
Polr2m	0.07914455	0.745988863
Polr3a	-0.038997184	0.965520236
Polr3b	-0.040015363	0.968005385
Polr3c	0.190603239	0.615775357
Polr3d	0.051784354	0.962648047
Polr3e	0.004835536	0.995411429
Polr3f	-0.090291518	0.852010446
Polr3g	0.293847818	0.62543868
Polr3gl	0.081652889	0.920642026
Polr3h	0.026324158	0.988481007
Polr3k	-0.008148213	0.993103295
Polrmt	-0.100282451	0.919670491
Cacna1e	-0.249593276	0.350732723
Pomc	-0.132363953	0.975831674
Pomgnt1	0.001841759	0.99765039
Pomgnt2	-0.046023779	0.955706294
Pomk	0.016819617	0.98526072
Pomp	0.043704762	0.965520236
Pomt1	-0.034684277	0.980339705
Pomt2	-0.086046605	0.89383516
Pon2	0.101946528	0.778738711
Pon3	-0.044288187	0.991996237
Pop1	-0.066948376	0.964941885
Pop4	0.035953797	0.968005385
Pop5	0.141361507	0.813499809
Pop7	0.015426436	0.993290512
Popdc2	0.079204811	0.993380259
Popdc3	0.167454093	0.871869598
Por	-0.091761812	0.805804328
Porcn	0.006937671	0.994824604
Postn	-0.060004634	0.985742667
Fuom	0.249492992	0.482385306
Pot1b	0.203737964	0.948104382
Pou2f1	-0.077684388	0.975350978
Pou2f2	-0.437882626	0.958852441
Pou2f3	0.157745039	0.984748462
Polr2j	0.248881665	0.419040911
Pou3f2	-0.13445025	0.884426973
Elob	0.248786592	0.235039388
Cenpb	0.248682473	0.09657124
Pou6f1	0.049813033	0.964011524
Pou6f2	0.446606893	0.873797321

Pp2d1	-0.773345366	0.858549868
Ppa1	0.12479479	0.644845851
Ppa2	0.019219217	0.985673938
Ppan	-0.039043791	0.975800973
Ppara	-0.217965884	0.926954786
Ppard	-0.186873522	0.861366392
Pparg	-0.204167667	0.948104382
Ppargc1a	-0.212775297	0.684977158
Ppargc1b	-0.22629586	0.875995577
Ppat	0.120445118	0.850960585
Ppbp	-0.217639395	0.992040075
Ppcdc	-0.126380624	0.90358675
Ppcs	-0.042542314	0.987326705
Ppdpf	-0.022876885	0.985293518
Ppef1	0.54517377	0.965520236
Ppef2	-0.131768727	0.980339705
Men1	-0.248404011	0.507235129
Ppfia2	-0.056668278	0.939904081
Ppfia3	-0.063160699	0.894953269
Ppfia4	0.029284766	0.965520236
Ppfibp1	-0.072562355	0.936031628
Ppfibp2	0.03918718	0.985742667
Pphln1	0.054501181	0.965520236
Ppia	0.080788094	0.660403254
Ppib	0.064111801	0.882096017
Ppic	-0.00442277	0.998010523
Ppid	0.022217201	0.975831674
Ppie	0.030844249	0.980896205
Ppif	0.155754883	0.682355533
Ppig	-0.440004005	0.894148925
Ppih	-0.093243041	0.946907532
Ppil1	0.171861827	0.87235248
Ppil2	0.048364143	0.921311891
Ppil3	-0.113609945	0.859803076
Ppil4	-0.02192591	0.981392364
Ppil6	0.377960981	0.809571745
Ppip5k1	-0.001237954	0.998205084
Ppip5k2	-0.083087734	0.928957615
Mea1	0.248362641	0.130062489
Ppm1a	-0.002657829	0.995411429
Ppm1b	0.041905571	0.927471971
Ppm1d	-0.129087762	0.83886846
Zadh2	0.24829479	0.429521854
Ppm1f	-0.058715304	0.942759993

Ppm1g	0.156145234	0.635909956
Ppm1h	0.054879517	0.948104382
Ppm1j	0.378169765	0.985673938
Ppm1k	-0.010705336	0.993103295
Ppm1l	-0.08265534	0.868415875
Ppm1m	0.212628113	0.788592804
Ppme1	0.053234062	0.911631946
Ppox	-0.045193359	0.975800973
Ppp1ca	0.093860334	0.69189201
Ppp1cb	0.331244278	0.911631946
Ppp1cc	0.040515136	0.962648047
Ppp1ccb	0.027455177	0.971661054
Ppp1r10	-0.029238082	0.974987859
Ppp1r11	0.054694029	0.964204868
Ppp1r12a	0.162319306	0.962648047
Ppp1r12b	-0.094635321	0.727005393
Ppp1r12c	-0.035515233	0.960027376
Ppp1r13b	0.039264892	0.950195611
Ppp1r13l	-0.393835448	0.643053315
Ppp1r14a	-0.092350364	0.962648047
Ppp1r14b	-0.001870032	0.998010523
Ppp1r14c	-0.035774739	0.974197921
Ppp1r15a	0.079203908	0.949607377
Ppp1r15b	-0.076337861	0.882096017
Ppp1r16a	0.047468764	0.968005385
Fbl	0.247952222	0.477528928
Lpo	-1.379150802	0.623042338
Ppp1r18	-0.175240301	0.881939002
Ppp1r1a	0.124745863	0.705578481
Ppp1r1b	0.117622543	0.876612181
Ppp1r1c	-0.917670161	0.962648047
Ppp1r2	-0.034112779	0.96188696
Mir3087	-1.383487349	NA
Ppp1r2-ps2	0.108450962	0.974962198
Ppp1r2-ps3	0.851359705	0.976978183
Ppp1r2-ps4	-0.091850431	0.976140679
Ppp1r2-ps6	0.044858402	0.99527876
Ppp1r21	-0.001279006	0.998010523
Ppp1r26	-0.127472263	0.993380259
Ppp1r32	0.376584985	0.936031628
Ppp1r35	0.088645702	0.961373288
Ppp1r36	0.445501602	0.777249347
Ppp1r37	-0.063585904	0.962648047
Mir6968	-1.383487349	NA

Ppp1r3b	0.029107574	0.99527876
Ppp1r3c	-0.016681698	0.985742667
Ppp1r3d	-0.113865994	0.938615214
Ppp1r3e	-0.023352266	0.98771048
Ppp1r3f	0.478396386	0.891788042
Ppp1r3g	0.528409893	0.772658285
Ppp1r42	-0.654186234	0.927392429
Ppp1r7	0.01607288	0.980419928
Rps11	0.247674072	0.023105145
Ppp1r9a	-0.393948656	0.926885804
Ppp1r9b	-0.019586455	0.976978183
Ppp2ca	0.012549286	0.98526072
Ppp2cb	0.073285667	0.874971335
Ppp2r1a	0.001731624	0.997115148
Ppp2r1b	-0.038686213	0.98340069
Ppp2r2a	-0.301877032	0.873753967
Ppp2r2b	-0.000545045	0.999582233
Ppp2r2c	-0.014443664	0.98149586
Ppp2r2d	-0.004144075	0.99527876
Ppp2r3a	0.117873594	0.760002272
Ppp2r3c	0.014844179	0.985742667
Ppp2r3d	0.021799122	0.985742667
Ppp2r5a	-0.070054735	0.853090538
Ppp2r5b	-0.034383677	0.964973812
Ppp2r5c	-0.056522885	0.905559486
Ppp2r5d	-0.025509899	0.969755848
Ppp2r5e	-0.127728894	0.615540363
Ppp3ca	-0.092129656	0.883333293
Ppp3cb	-0.393611879	0.89703631
Ppp3cc	0.147471097	0.80931022
Ppp3r1	-0.047824477	0.94789347
Ppp3r2	0.121691093	0.989840188
Ppp4c	0.131716485	0.828102037
Ppp4r1	0.070264725	0.936713201
Ppp4r1l-ps	-0.062453374	0.97487264
Ppp4r2	-0.053642639	0.910328122
Ppp4r3a	-0.002816472	0.998010523
Ppp4r3b	-0.208698188	0.89703631
Ppp4r4	-0.170910186	0.84870301
Ppp5c	0.008371811	0.991438757
Ppp6c	0.098930013	0.911631946
Ppp6r1	-0.001814437	0.997115148
Ppp6r2	0.024784474	0.98187819
Ppp6r3	-0.066680626	0.903795193

Fus	0.247476085	0.146214787
Ppt1	-0.053626107	0.910644485
Ppt2	-0.049285763	0.962648047
Pptc7	-0.088818892	0.786324313
Ppwd1	0.090744432	0.962648047
Shank3	-0.247403701	0.02035027
Pqlc1	0.146950312	0.789833208
Pqlc2	-0.236722895	0.727640208
Pqlc3	-0.646743963	0.571020232
Pradc1	0.232329143	0.807302961
Praf2	0.396618731	0.920642026
Prag1	-0.010611161	0.99527876
Pram1	0.551372401	0.957169848
Pramef8	0.084640013	0.911631946
Prc1	0.038315756	0.990095302
Prcc	0.093834357	0.852010446
Mir6940	-1.383674901	NA
Prcp	-0.064483261	0.968630572
Prdm1	0.18701732	0.976257618
Prdm10	-0.080877744	0.924361537
Prdm11	-0.106874878	0.909243078
Plac8	-1.384845639	0.911276003
Prdm15	-0.105023003	0.8616433
Prdm16	0.171697079	0.688142448
Prdm2	-0.067808684	0.889972515
Prdm4	0.050882356	0.939592821
Lrrc58	0.247223558	0.056924302
Plod1	-0.247175674	0.500117204
Prdm9	0.318162276	0.80818949
Prdx1	0.076693186	0.815987743
Prdx2	0.096788598	0.668010503
Prdx3	0.056905739	0.895198797
Prdx4	0.115285367	0.942781802
Prdx5	0.115153948	0.619769322
Prdx6	-0.102862485	0.854278008
Prdx6b	-0.783366355	0.980226283
Preb	-0.0047507	0.99527876
Prelid1	0.05841162	0.935897306
Myocos	-1.385625024	0.948407374
Cadps2	-0.24686838	0.225577018
Prelid3b	-0.009497784	0.993380259
Ndufa13	0.246796269	0.056924302
Prep	0.018482656	0.984512582
Psma2	0.246628728	0.012905834

Prex1	-0.013230243	0.994630727
Prex2	-0.168817237	0.701408817
Myo7b	-1.389135355	0.962648047
Cinp	0.246276551	0.146214787
Prickle2	-0.072679615	0.823690933
Prickle3	0.599454137	0.837755777
Uts2r	-1.389135355	0.962648047
Prim1	0.295612097	0.641911698
Prim2	0.188346655	0.867827234
Ccdc12	0.246272034	0.442302609
Primpol	0.187730426	0.858549868
Prkaa1	-0.045863566	0.962648047
Prkaa2	-0.209468639	0.574956844
Prkab1	0.153910781	0.771684575
Prkab2	-0.668464493	0.59983323
Prkaca	0.065433415	0.869437456
Prkacb	0.218597816	0.703372057
Prkag1	0.085197797	0.851443045
Prkag2	0.03680042	0.965520236
Prkag2os1	-0.947447249	0.965320168
Prkag2os2	0.218714724	0.869316117
Tpte	-1.393434202	0.959778108
Prkar1a	-0.038148555	0.91462326
Prkar1b	-0.023763301	0.973100313
Izumo4	0.246258731	0.456330081
Prkar2b	-0.078198764	0.99293935
Nrip1	-0.246175192	0.252498589
Arhgap17	-0.245895991	0.404892636
Prkcd	0.048517365	0.97607383
Prkce	-0.053635806	0.865547585
Gbf1	-0.245572896	0.106596954
Prkch	-0.064572093	0.974823941
Prkci	-0.02335621	0.974823941
Prkcq	0.079065477	0.965256427
Prkcsh	0.132081297	0.639430281
Prkcz	-0.013588589	0.98526072
Prkcz2	-0.002206294	0.998010523
Prkd1	0.184080834	0.84870301
Prkd2	-0.078681443	0.968005385
Ggcx	-0.245521535	0.392219282
Prkdc	-0.072104655	0.897878088
Prkg1	-0.051024902	0.981392364
Prkg2	-0.231639493	0.948432875
Ccdc190	-0.245506233	0.394767148

Prkra	0.052912714	0.964941885
Prkrip1	0.055626047	0.965011162
Ar	-0.245325117	0.360222394
Mir6901	-1.398814709	NA
Prlr	-0.346159053	0.962648047
Prmt1	0.037149993	0.963943597
Prmt2	-0.102344607	0.839649856
Dab2ip	-0.244717986	0.422629014
Prmt5	-0.035430733	0.965520236
Prmt6	0.130236194	0.882096017
Prmt7	-0.004112246	0.99527876
Prmt8	-0.001344181	0.998010523
Prmt9	-0.109271568	0.821998633
Prn	0.732144738	0.869210848
Prnp	-0.056937384	0.89703631
Prob1	0.139387378	0.956164302
Proca1	0.248399714	0.90872336
Procr	-0.967356698	0.659056223
Prodh	0.123807247	0.779964055
Prok1	-0.27622085	0.985742667
Prok2	-0.85498106	0.962648047
Prokr1	0.552405771	0.984748462
Erp29	0.244202904	0.200754354
Galntl6	-0.243811095	0.421691269
Prom2	-0.848212953	0.936031628
Prorsd1	0.220236844	0.868415875
Pros1	-0.234683429	0.629280869
Proscos	-0.017497421	0.997115148
Proser1	-0.067234553	0.91462326
Proser2	0.364372514	0.792034341
Proser3	0.257270565	0.89703631
Prox1	0.085977516	0.957209681
Dgkb	-0.243740303	0.296105357
Ankrd34a	-0.243637911	0.130179939
Prox2os	-0.074298275	0.974962198
Proz	0.600463955	0.936031628
Prpf18	-0.044195499	0.962094124
Prpf19	0.112757499	0.549579107
Prpf3	0.075048212	0.949607377
Prpf31	0.114349448	0.7851601
Prpf38a	0.156565728	0.727640208
Prpf38b	0.006787859	0.996094752
Sap18b	0.243450506	0.113604388
Prpf4	-0.06707486	0.92488271

Prpf40a	-0.150945729	0.84870301
Prpf40b	0.027499138	0.968630572
Prpf4b	-0.060213076	0.908413609
Prpf6	0.07080921	0.852010446
Prpf8	-0.04732186	0.909547396
Prph	0.395354144	0.916426816
Prps1	0.078668469	0.824368034
Prps1l3	0.250553599	0.708953765
Prps2	-0.107797734	0.873797321
Prpsap1	0.039013759	0.970892908
Prpsap2	0.116257996	0.632633706
Prr11	-0.192825388	0.965520236
Prr12	-0.087536389	0.813499809
Prr13	0.128894187	0.825249193
Prr14	-0.193300696	0.779415976
Prr14l	-0.105437217	0.810908327
Prr15	0.276890869	0.975800973
Prr15l	0.269934612	0.975312818
Prr16	-0.134551212	0.962648047
Tpd52l2	-0.24306365	0.146214787
Prr19	-0.803102192	0.965520236
Mir6897	-1.398814848	NA
Mir7014	-1.398814848	NA
Prr3	0.096962792	0.864150749
Prr32	0.742241126	0.886647768
Prr33	0.75378348	0.912042382
Prr36	-0.125072187	0.764516583
Prr5	0.292074319	0.578812716
Prr5l	-0.024639525	0.993380259
Prr7	-0.091840179	0.916426816
Prrc1	0.02818137	0.981392364
Ost4	0.24304406	0.423023136
Prrc2b	-0.171951391	0.630888733
Reps2	-0.242986955	0.197976816
Prrg1	0.110348412	0.95363833
Prrg2	0.280170454	0.891972665
Prrg3	-0.200117252	0.615948828
Prrg4	-0.127831821	0.980339705
Prrt1	-0.110764503	0.80931022
Prrt2	-0.102298804	0.84870301
Prrt3	-0.276637871	0.513797506
Prrt4	-0.089133742	0.985293518
Zscan18	0.242935612	0.436138179
Prrx2	-0.149304267	0.985742667

Prss12	0.325790451	0.580757434
Prss16	0.581748776	0.815987743
Prss22	0.29241656	0.991996237
Prss23	-0.034723557	0.984836179
Prss23os	0.670306795	0.873797321
Prss30	-0.078918262	0.997115148
Prss35	0.079426493	0.954174097
Prss36	-0.127810039	0.915417975
Prss38	0.907096667	0.971661054
Mir1956	-1.39901195	NA
Fam71b	-1.399521052	0.873797321
Cyp2b19	-1.405486784	0.950460219
Prss50	0.997096805	0.643053315
Prss52	0.112005731	0.991996237
Taz	0.242427634	0.483420706
Abr	0.242235942	0.273065025
Prss57	-0.264003378	0.962812066
Prtg	0.098991643	0.946985353
Prtn3	0.425354629	0.889972515
Prune1	-0.106171962	0.736130784
Prune2	-0.008849718	0.991438757
Prx	-0.311999565	0.814301191
Sipa1l2	0.242207704	0.428254781
Psat1	-0.218041026	0.819726438
Mir130a	-1.413862753	NA
Psd	0.031033127	0.973100313
Psd2	0.060114407	0.932673884
Atxn7l3	0.242045116	0.378457533
Psd4	-0.124774398	0.985293518
Psen1	0.057773512	0.8871895
Psen2	0.063037779	0.962648047
Psenen	0.274728568	0.664771806
Psg16	-0.041734292	0.987453225
Plppr5	-0.241808879	0.146214787
Psip1	-0.061516581	0.962648047
Pskh1	0.026934231	0.985673938
Hcn1	-0.241599715	0.41351939
Macf1	-0.24148323	0.005658911
Psma3	0.059445303	0.90125879
Psma4	0.020918384	0.98340069
Psma5	0.09764746	0.727452809
Ankrd17	-0.240891506	0.161844023
Nov	-0.240576239	0.232043388
Psma8	-0.159059499	0.985742667

Trp53bp2	0.24029647	0.485827814
Psmb10	0.213387791	0.595105754
Psmb11	0.063224862	0.994806961
Psmb2	0.180915552	0.534461098
Pfdn5	0.24003975	0.011372038
Psmb4	0.091656914	0.813552442
Psmb5	0.074687608	0.87847909
Psmb6	0.138984312	0.52053053
Frmd6	-0.239833648	0.283970805
Psmb7-ps2	-0.464256787	0.968005385
Psmb8	-0.046670536	0.991438757
Psmb9	-0.44076198	0.908865777
Ndufb9	0.239628473	0.056924302
Psmc2	0.099192319	0.6883862
Rpl9-ps6	0.239498824	0.315413695
Psmc3ip	0.279432827	0.861366392
Psmc4	0.006371605	0.994960308
Plxna1	-0.239360336	0.220337275
Psmc6	0.072960589	0.881771584
Psmd1	-0.033122594	0.957485565
Psmd10	0.032523695	0.975800973
Psmd11	-0.061405978	0.957499962
Psmd12	0.066283804	0.882096017
Psmd13	0.023392354	0.972218452
Psmd14	0.099994396	0.67239949
Psmd2	0.003719806	0.99527876
Psmd3	0.037548676	0.962648047
Zfp516	-0.239337244	0.399438388
Psmd5	-0.023685557	0.971976293
Psmd6	0.092121266	0.786271601
Psmd7	0.087612278	0.821998633
Psmd8	0.187624883	0.615948828
Shisa4	0.239161683	0.37167168
Psme1	0.13795543	0.610726714
Psme2	-0.059205382	0.968005385
Psme2b	-0.093134666	0.968005385
Psme3	0.066359062	0.860521845
Psme4	-0.111331741	0.83886846
Psmf1	0.033549078	0.965520236
Senp6	-0.239030363	0.310553098
Psmg2	0.110201352	0.86783605
Psmg3	0.01277415	0.99527876
Psmg4	0.124870919	0.957499962
Pspc1	-0.058269346	0.964941885

Psph	0.029288722	0.981392364
Pspn	0.917873458	0.91462326
Psrc1	0.040918071	0.97932602
Pstk	0.041788673	0.968005385
Pstpip1	0.004458474	0.99765039
Pstpip2	-0.005958889	0.996094752
Ptafr	-0.431242017	0.784009698
Ptar1	-0.569092335	0.770793864
Slc25a39	-0.238967027	0.34587585
Ptbp2	-0.018032656	0.97607383
Rps3a2	0.238946369	0.079050693
Ptcd1	0.008788382	0.99527876
Ptcd2	-0.008676169	0.994960308
Ptcd3	-0.027990324	0.969755848
Mapk3	0.238487222	0.509250303
Ptch2	-0.51269875	0.69189201
Ptchd1	0.10852044	0.981045362
Ptchd4	0.284472767	0.925924974
Lepr	-1.415883208	0.54807923
Ptdss1	-0.264813849	0.745988863
Ptdss2	0.003262988	0.995632078
Pten	-0.022042378	0.971661054
Pter	0.989674838	0.962648047
Ptgdr	-0.803858338	0.910328122
Ptgdr2	0.891067409	0.948104382
AC154378.2	-1.416456449	0.871869598
Ptger1	-0.499819608	0.965520236
Ptger2	0.087805719	0.99527876
Ptger3	-0.223144365	0.970348278
Ptger4	0.100844443	0.984232465
Ptges	0.017793796	0.99527876
Ptges2	0.054363504	0.948104382
Ptges3	0.085665245	0.860521845
Ptges3-ps	0.014456331	0.993158976
Ptges3l	0.470389704	0.655641038
Tmem74	-0.238233918	0.442068044
Dynlrb1	0.238193346	0.186859869
Ptgis	0.071647705	0.98459388
Ptgr1	0.138705899	0.964941885
Ptgr2	0.027679035	0.981683975
Ptgs1	0.080661264	0.962648047
Ptgs2	0.009976305	0.99527876
Ptgs2os	0.218463027	0.965520236
Ptgs2os2	0.435463108	0.97442139

Pth1r	0.101139561	0.971138085
R3hcc1	0.238016658	0.436138179
Pthlh	-0.076468382	0.985293518
Ptk2	-0.010621484	0.989640643
Ptk2b	0.056050229	0.934135315
Ptk7	-0.00738051	0.99527876
Rhou	0.237882319	0.269754422
Ptms	0.091097035	0.887695241
Ptn	-0.014841573	0.985742667
Ptov1	-0.043429832	0.948104382
Ptp4a1	0.238215605	0.7851601
Ptp4a2	0.109347277	0.609927197
Ptp4a3	-0.009013215	0.993103295
Ptpa	0.166815965	0.615496563
Ptpdc1	0.122346041	0.852275035
Ptpmt1	0.034251527	0.963666931
Trpv2	-0.237653647	0.434762828
Ptpn11	0.11230312	0.56651873
Ptpn12	-0.076394088	0.886647768
Ptpn13	-0.095674406	0.961373288
Ptpn14	-0.144898123	0.872265039
Ptpn18	-0.131492784	0.97487264
Ptpn2	0.008339654	0.99527876
Ptpn20	0.026699499	0.99527876
Ptpn21	-0.095811317	0.944074069
Ptpn22	0.100124111	0.980132658
Snrnp48	-0.237648896	0.44386328
Ptpn3	-0.041337289	0.955726049
Rb1	-0.237540826	0.41394684
Nfu1	0.237255675	0.11526116
Ptpn6	-0.278274952	0.786324313
Mir7082	-1.424354665	0.868797028
Setd2	-0.237145531	0.422728382
Ptpra	-0.002655384	0.99527876
Ptprb	-0.106520773	0.91462326
Ptprc	-0.121127754	0.961373288
Ptprcap	0.233765103	0.968497942
Ptprd	-0.07229805	0.875119971
Ptpre	-0.0546329	0.936031628
Ptprf	-0.071669317	0.932673884
Ptprg	-0.393442778	0.860521845
Ptprh	-0.128024166	0.985653607
Ptprj	-0.367991665	0.956922504
Ptprk	0.056007826	0.951390592

Ptprm	0.18695083	0.74632237
Ptprn	-0.05652107	0.920200633
Ptprn2	-0.139105707	0.590745439
Ptpro	-0.058474683	0.962648047
Ptprq	0.733984142	0.948254447
Ptprr	-0.131885163	0.708626104
Ptprs	-0.150205684	0.542557253
Ptprt	-0.267750587	0.783229859
Fgf11	-0.237110903	0.389731767
Ptprv	-0.437194244	0.975831674
Ptprz1	-0.062380224	0.909243078
Ptrh1	-0.01310191	0.99640101
Ptrh2	0.050137103	0.957499962
Ptrhd1	0.073491122	0.958389182
Pts	-0.060847976	0.97487264
Pttg1	-0.338333774	0.723536516
Pttg1ip	0.058511405	0.99527876
Ptx3	0.175028713	0.971740377
Ptx4	0.430527953	0.969755848
Puf60	0.081754145	0.768977675
Pum1	-0.036536811	0.985673938
Pum2	0.003870729	0.998775547
Pum3	-0.017765178	0.984836179
Pura	-0.100653434	0.911276003
Purb	-0.047640527	0.911631946
Purg	0.02215446	0.98526072
Pus1	0.059867843	0.965520236
Atp5g3	0.236810356	0.008848823
Pus3	-0.153450229	0.884259444
Pus7	0.020269963	0.985742667
Pus7l	-0.183636771	0.91462326
Pusl1	0.136476675	0.83977902
Parl	0.236416741	0.413463762
Pvr	0.029342506	0.985742667
Pvt1	0.159648931	0.909547396
Pwp1	-0.038573841	0.971661054
Pwp2	0.042744967	0.962648047
Mdn1	-0.235584495	0.240817904
Pwwp2b	0.042186033	0.965520236
Pxdc1	-0.107876737	0.985742667
Pxdn	-0.064808566	0.881771584
Pxk	-0.070677282	0.895198797
Pxmp2	0.001668008	0.998631534
Prkcb	-0.235431182	0.053012025

Rasl10b	-0.235199668	0.400756364
Pxylp1	-0.055170576	0.962648047
Ssu72	0.235159442	0.456330081
Pycr1	0.484700132	0.814814675
Pycr2	-0.0097694	0.99527876
Pycrl	-0.04265027	0.974823941
Pygb	0.033159903	0.948104382
Pygl	-0.056832469	0.97487264
Nhp2	0.235077705	0.111255411
Pygo1	-0.171982576	0.56399886
Pygo2	-0.390603953	0.899286919
Pym1	0.197638672	0.56399886
Pyroxd1	-0.03182745	0.98526072
Pyroxd2	0.033724543	0.992040075
Pyurf	-0.385458268	0.710772024
Osm	-1.425003082	0.91462326
Qars	0.008101185	0.99527876
Mdga1	-0.234968022	0.442068044
Qk	-0.073935691	0.891972665
Qpct	-0.14377621	0.857668455
Qpctl	-0.037436355	0.968005385
Nox4	-1.427168155	0.957499962
Qrfp	0.084931735	0.99527876
Qrfpr	-0.312520571	0.929618757
Qrich1	0.013000509	0.993449239
Clec4a1	-1.434669948	0.910328122
Qrsl1	0.065459804	0.948104382
Qser1	-0.175539189	0.751142243
Qsox1	0.093169104	0.962648047
Qsox2	-0.097379195	0.825148733
Qtrt1	0.158622241	0.811913793
Qtrt2	-0.130032393	0.833916587
Rgma	-0.234802891	0.323832374
R3hcc1l	-0.025707488	0.985446046
Phpt1	0.234495861	0.185197923
R3hdm2	-0.092573911	0.855842288
R3hdm4	0.081470713	0.882824434
R74862	0.162991837	0.873797321
Rab10	-0.019140606	0.981045362
Rab10os	0.010815398	0.99527876
Rab11a	-0.055207167	0.949607377
Rab11b	0.122764031	0.623042338
Rab11b-ps2	0.042179472	0.968005385
Rab11fip1	-0.048673378	0.987326705

Rab11fip2	-0.039680843	0.951497773
Rab11fip3	-0.071466535	0.882824434
Rab11fip4	-0.04060061	0.948104382
Rab11fip5	-0.010531761	0.991996237
Rab12	-0.018563427	0.980419928
Rab13	0.175243126	0.911631946
Rab14	-0.032549405	0.969755848
Rab15	-0.080447962	0.89703631
Rab18	-0.027900019	0.965520236
Rab1a	0.002581967	0.99527876
Rab1b	-0.016139825	0.984328758
Rab20	0.587945414	0.912397394
Rab21	-0.024451561	0.971795434
Rab22a	0.012217721	0.988034453
Rab23	-0.002874664	0.997115148
Rab24	0.098291609	0.792913409
Rgs18	-1.443508322	0.956164302
Rab26	-0.039084045	0.970461999
Ndufb1-ps	0.234488384	0.459780215
Rab27a	0.184620413	0.780228245
Rab27b	-0.054473843	0.958389182
Rab28	0.112703953	0.904346827
Rab29	0.046478508	0.983977391
Rab2a	0.00319553	0.99527876
Rab2b	0.606551884	0.795563654
Rab30	-0.125513224	0.810908327
Rbbp7	0.234369857	0.200558701
Rab32	0.249991412	0.962648047
Rab33a	0.081563472	0.921076877
Rab33b	-0.256621564	0.959276014
Trio	-0.234218236	0.259538512
Rab35	0.032502727	0.968005385
Rab36	-0.094732068	0.847678217
Rab37	0.054318783	0.983166509
Rab38	0.024837598	0.99527876
Rab39	-0.578932614	0.573443942
Rab39b	-0.021550561	0.984328758
Rab3a	0.055787321	0.912917153
Rab3b	0.045682583	0.963943597
Rps5	0.234044836	0.09894647
Rab3d	0.070375124	0.956922504
Rab3gap1	-0.075113865	0.867827234
Rab3gap2	-0.064361122	0.951390592
Rab3il1	-0.085089488	0.959276014

Rab3ip	0.094364117	0.852010446
Rab40b	-0.004686394	0.99527876
Rab40c	0.171977853	0.869316117
Rab42	-0.036210582	0.99527876
Rab43	-0.094373	0.889908105
Rab4a	0.083607908	0.868415875
Imp3	0.233696039	0.310553098
Rab5a	0.02916409	0.97041548
Rab5b	0.021750623	0.969755848
Rab5c	0.132801812	0.815987743
Rab5if	-0.014950817	0.985742667
Rab6a	0.007238375	0.993380259
Rab6b	-0.036849817	0.955993235
Rab7	0.018396855	0.973100313
Rab7-ps1	0.221471189	0.74632237
Rab7b	-0.708019212	0.746275361
Rab8a	0.035240529	0.975800973
Tceal6	0.233446473	0.145093191
Rab9	0.035733688	0.966010627
Rab9b	-0.187224885	0.549579107
Spaca1	-1.443508322	0.956164302
Rabac1	0.00045183	0.999227272
Rabep1	-0.397805445	0.517996718
Rabep2	0.157664621	0.865884071
Rabepk	0.065529727	0.958389182
Rabgap1	-0.089096826	0.962648047
Fbrsl1	-0.232504415	0.383515294
Rabgef1	-0.008231691	0.996369585
Rabggta	0.060592551	0.921659319
Rabggtb	0.102035879	0.792913409
Rabif	-0.06754735	0.948407374
Rabl2	-0.025430418	0.990915306
Rabl3	-0.036482822	0.980419928
Rabl6	0.025374016	0.972374093
Rac1	-0.015415789	0.97607383
Dzip3	-0.232168084	0.193510645
Rac3	0.004049699	0.996873492
Racgap1	-0.219937467	0.836720624
Rack1	0.085011445	0.780007405
Uqcrq	0.232119897	0.313219565
Rad17	-0.08549238	0.902293377
Rad18	-0.055425855	0.968005385
Rad21	0.025064714	0.97041548
Rad21l	0.10493595	0.985673938

Rad23a	0.097399028	0.839488666
Rad23b	0.087198383	0.745988863
Rad50	-0.110988666	0.89011487
Rad51	0.064335031	0.985742667
Rad51ap1	0.338192389	0.837755777
Rad51ap2	-0.187376528	0.971138085
Rad51b	0.106560508	0.993380259
Rad51c	0.045810129	0.984748462
Rad51d	-0.043478483	0.969755848
Rad52	0.024759182	0.984328758
Rad54b	0.232957852	0.936031628
Rad54l	0.028799486	0.992385525
Rad54l2	-0.064435543	0.936031628
Rad9a	-0.164640161	0.874422885
Rad9b	-0.048206599	0.985742667
Radil	-0.074133588	0.962648047
Rae1	0.03666492	0.970892908
AC183097.1	-1.444511552	0.948104382
Raet1e	0.322108008	0.99100709
Raf1	0.010788115	0.991438757
Rag1	-0.553411055	0.974987859
Rai1	-0.093347975	0.955993235
Rai14	0.161180994	0.895198797
Rai2	-0.136143001	0.948104382
Rala	-0.031387577	0.969755848
Ralb	-0.097709278	0.903596982
Ralbp1	-0.015073632	0.985742667
Ralgapa1	-0.017612889	0.98966731
Syf2	0.23189335	0.089057569
Lztfl1	-0.231653714	0.450404435
Ralgds	-0.038858475	0.962648047
Ralgps1	-0.101998305	0.751142243
Ralgps2	-0.039973609	0.959659722
Raly	0.066301019	0.974823941
Ralyl	-0.06951994	0.941398449
Ramac	-0.02117177	0.981502501
Ramp1	0.106651393	0.837683265
Ramp2	0.211706981	0.516846854
Ramp3	0.418355311	0.5141747
Ran	0.053574463	0.897862979
Ranbp1	0.139043454	0.799082182
Ranbp10	-0.048719212	0.955908516
Ranbp17	0.020103533	0.993103295
Lrfn3	-0.231345337	0.497690258

Prkx	-0.23106096	0.455841215
Ranbp3l	-0.276027089	0.932673884
Ranbp6	-0.056926739	0.941398449
Ranbp9	0.106705361	0.911631946
Rangap1	0.049019649	0.967541135
Rangrf	-0.038727708	0.987326705
Rap1a	0.04898355	0.939592821
Rap1b	-0.040796581	0.962648047
Rap1gap	0.05951738	0.911834483
Rap1gap2	-0.066555119	0.947033215
Rap1gds1	-0.015924875	0.984328758
Rap2a	-0.044247882	0.948104382
Rap2b	-0.054511165	0.911631946
Rap2c	-0.094880426	0.806422207
Rapgef1	0.011770758	0.991438757
Rapgef2	-0.027766706	0.962648047
Bloc1s2	0.230406214	0.394955649
Mir6364	-1.452454936	0.932673884
Rapgef3os2	-0.267885198	0.978754261
Rapgef4	0.017140981	0.980731359
Rapgef4os2	0.028928953	0.994002549
Rapgef4os3	-0.331055768	0.920452533
Prelp	-0.230147089	0.323832374
Rapgef6	-0.508312295	0.904346827
Rapgefl1	-0.064128756	0.882096017
Raph1	-0.20143019	0.57472851
Rapsn	0.092851004	0.991438757
Bloc1s1	0.230016641	0.386321341
Rarb	0.104696997	0.965520236
Rarg	-0.15823455	0.815987743
Rarres1	-0.02871561	0.99527876
Rarres2	0.02890284	0.985742667
Rars	0.072652469	0.891972665
Rars2	0.064991581	0.962648047
Gmds	-0.229367196	0.497972882
Mlycd	0.22917745	0.310553098
Rasa3	0.176637193	0.960711044
Rasa4	0.040651438	0.98623785
Rasal1	-0.09024936	0.871869598
Rasal2	0.098184022	0.752097504
Rasal3	-0.000957818	0.999242708
Rasd1	-0.052745859	0.969755848
Rasd2	-0.01723451	0.985293518
Rasgef1a	0.071755978	0.854278008

Rasgef1b	0.016642522	0.99527876
Rasgef1c	-0.22049736	0.932673884
Rasgrf1	-0.082140762	0.6883862
Rasgrf2	0.122175074	0.824368034
Ssna1	0.229072256	0.128640258
Rasgrp2	0.215737257	0.670918119
Marcksl1	0.228963433	0.300118082
Rasgrp4	-0.877493389	0.621993647
Rasip1	-0.04987091	0.983977391
Rasl10a	-0.037440984	0.985742667
Nhlrc3	0.228829097	0.39534064
Rasl11a	0.121999428	0.889908105
Rasl11b	-0.064216637	0.963666931
Rasl12	-0.136909972	0.936483158
Rasl2-9	0.279384195	0.97487264
Rassf1	0.040083894	0.981250052
Rassf10	-0.079307386	0.987342614
Rassf2	-0.000530529	0.99900438
Rassf3	0.070879684	0.952034929
Rassf4	-0.276516095	0.681047846
Rassf5	-0.103369802	0.83140737
Rassf6	-0.804644961	0.944972801
Rassf7	-0.27522521	0.965520236
Rassf8	0.064618154	0.962648047
Rassf9	-0.759674666	0.544661714
Syne2	-0.228794107	0.450679274
Kdm4a	0.228629988	0.184761973
Mfhas1	-0.228531947	0.089553702
Rb1cc1	-0.158495005	0.8549794
Rbak	0.1074418	0.873797321
Rbbp4	0.074956545	0.789833208
Rbbp5	-0.187102059	0.821998633
Rbbp6	-0.01517785	0.985742667
Lsm14b	0.228268628	0.145093191
Rbbp8	0.148832407	0.948104382
Rbbp9	0.007606	0.993380259
Rbck1	0.039177417	0.957499962
Rbfa	0.136388071	0.618152081
Rbfox1	-0.035141443	0.955401619
Rbfox2	0.088621205	0.944494911
Rbfox3	0.091108332	0.759486652
Rbks	-0.266983475	0.929503395
Rbl1	-0.231766288	0.927934062
Rbl2	-0.026882701	0.971661054

Rbm10	-0.100142242	0.790508337
Rbm11	0.067991693	0.962648047
Rbm12	-0.162503302	0.910328122
Rbm12b1	-0.748635342	0.868415875
Rbm12b2	-0.08332059	0.927471971
Sf3a3	0.228168585	0.106206739
Csgalnact1	0.227726998	0.496022017
Rbm15b	0.078822027	0.871869598
Rbm17	0.101429866	0.809627943
Rbm18	-0.022713638	0.981502501
Atp2b1	-0.227550582	0.338645928
Rbm20	0.390291593	0.740833211
Rbm22	0.086415538	0.825910982
Rbm24	-0.13211172	0.874971335
Rbm25	-0.024760959	0.965520236
Rbm26	-0.143810402	0.786324313
Rbm27	-0.066307523	0.920790661
Twf1	0.227313661	0.110827573
Rps13	0.227155731	0.220337275
Ndufs5	0.226930077	0.219354159
Glra4	-1.459294282	0.955993235
Rbm34	0.028954667	0.981392364
Rbm38	0.385565508	0.868415875
Rbm39	-0.019161727	0.983977391
Rbm3os	0.187447844	0.981392364
Rbm4	0.050878127	0.968005385
Rbm41	-0.144834473	0.936031628
Rbm42	-0.154920888	0.874252042
Rbm43	0.380362051	0.698275031
Rbm45	0.045096595	0.962094124
Rbm46	-0.02792179	0.99527876
Rbm47	0.092350351	0.989378233
Rbm48	0.28735144	0.660781615
Rbm4b	0.050975826	0.965520236
Rbm5	-0.07318539	0.861139061
Rbm6	-0.127083205	0.642397758
Rbm6-ps1	0.064435877	0.99527876
Rbm7	0.110597197	0.871869598
Rbm8a	0.102911881	0.890704638
Rbm8a2	0.352326659	0.984232465
Rbms1	0.031847072	0.985673938
Rbms2	0.130504506	0.887709902
Rbms3	-0.443969189	0.962648047
Rbmx	0.045224893	0.945647427

Rbmx2	0.252757185	0.661008103
Rbmxl1	0.140485168	0.650703267
Rbp1	0.085350375	0.974962198
Rbp2	0.811301899	0.968005385
Rbp3	-0.80732634	0.898478018
Rbp4	0.183006848	0.911631946
Rbpj	-0.152249369	0.670918119
Rbpj-ps3	-0.100512329	0.968005385
Rbpjl	0.190509173	0.968005385
Rbpms	-0.212263967	0.901573402
Rbpms2	0.165538232	0.968005385
Rbsn	-0.042106273	0.952385926
Rpl27-ps3	0.226760985	0.135809942
Rbx1-ps	-0.18832132	0.98459388
Rc3h1	-0.090738016	0.871869598
Rc3h2	0.504768482	0.961373288
Rcan1	0.277379145	0.754350047
Rcan2	0.461454805	0.691803511
Rcan3	-0.414587151	0.942511266
Eif2ak4	-0.226494623	0.400459245
Rcbtb2	0.059547357	0.945914524
Rcc1	0.063947372	0.95363833
Rcc1l	0.019415575	0.991996237
Rcc2	-0.055096878	0.895520088
Rccd1	0.005424208	0.997115148
Rce1	-0.067206389	0.976978183
Rchy1	0.326861225	0.905251009
Rcl1	0.120222	0.875723186
Rcn1	0.162206759	0.707777756
Rcn2	0.055947955	0.89703631
Rcn3	-0.077114908	0.968005385
Rcor1	-0.16992153	0.522113371
Rcor2	0.1585265	0.795137348
Rcor3	0.115756676	0.957499962
Rcsd1	0.095896336	0.965520236
Rcvrn	0.768155052	0.899097089
Rd3	0.133190196	0.980419928
Aldh1a2	-1.464224519	0.631312676
Rdh10	0.144357193	0.886647768
Rdh11	-0.014299248	0.988941187
Rdh12	-0.080003699	0.968005385
Rdh13	0.007688481	0.99527876
Rdh14	0.011274574	0.993103295
Rdh16	0.402049699	0.953505913

Rdh16f1	-0.802499165	0.920642026
Rdh5	0.088861222	0.975831674
Rdm1	0.232271258	0.814052898
Rdx	0.015487462	0.985673938
Rec114	-0.401189876	0.968630572
Rec8	0.404414273	0.635909956
Reck	0.014798683	0.99527876
Recql	0.081426764	0.946450282
Recql4	-0.115236994	0.963563887
Recql5	-0.003983933	0.997115148
Reep1	-0.076832584	0.871869598
Reep2	0.02709773	0.968597938
Reep3	-0.125103518	0.860521845
Reep4	-0.292391289	0.86783605
Reep5	-0.000829493	0.998010523
Reep6	-0.35175832	0.798580668
Rel	0.064833329	0.980896205
Rela	-0.00776201	0.99527876
Relb	-0.179705309	0.868415875
Relch	-0.316358017	0.691803511
Rell1	-0.256234273	0.705332654
Rell2	-0.127513259	0.898611672
Reln	-0.074886226	0.849526037
Relt	-0.039037567	0.975312818
Rem1	0.876406051	0.860826773
Rem2	-0.110279591	0.932673884
Renbp	-0.005926709	0.997258205
C1rb	-1.467547746	0.935897306
Ints8	-0.226138296	0.447061098
Reps1	0.070816042	0.896196194
Car4	0.226017648	0.31865776
Rer1	-0.001433954	0.99765039
Rpl35	0.225674677	0.146214787
Rerg	-0.050959093	0.974823941
Resf1	0.293873464	0.956164302
Resp18	0.114742509	0.900513955
Rest	-0.021988011	0.991438757
Ret	-0.009380839	0.997258205
Retn	0.623062017	0.89703631
Retreg1	0.063218859	0.938414123
Retreg2	-0.040242872	0.970461999
Retreg3	0.021152596	0.976978183
Retsat	-0.153521228	0.740833211
Rev1	-0.133442774	0.768330892

Rev3l	-0.157456851	0.810455125
Rex1bd	-0.024025634	0.989556168
Rexo1	-0.038002379	0.974823941
Rexo2	-0.033944701	0.975831674
Rexo4	-0.095071189	0.807017299
Rexo5	0.610170573	0.779964055
Prss40	-1.467826958	0.944753667
Rfc1	-0.089658734	0.836720624
Rfc2	-0.017958537	0.985742667
Rfc3	-0.137357025	0.948407374
Rfc4	0.05321116	0.98526072
Rfc5	0.127819875	0.873797321
Rfesd	-0.159313247	0.882824434
Rffl	-0.614752614	0.861909929
Rfk	-0.025740273	0.973590542
Rflna	0.941515354	0.866560751
Rflnb	0.282469125	0.642397758
Rfng	-0.063711403	0.903441375
Rft1	0.070060163	0.957499962
Rftn1	-0.13229391	0.868415875
Rftn2	-0.033907412	0.968005385
Rfwd3	-0.094959274	0.779231312
Rfx1	-0.108269903	0.813716057
Dzip1	-0.225645718	0.265123922
Rfx3	0.067568654	0.962648047
Rfx4	-0.06232336	0.973100313
Scnm1	0.225347502	0.445106721
Mxd4	0.225133759	0.232043388
Rfx8	0.381723998	0.956922504
Rfxank	-0.323529035	0.849517436
Rfxap	0.064916834	0.959276014
Rgcc	0.256975125	0.620124996
Rgl1	-0.074883581	0.813552442
Rgl2	-0.01685202	0.985742667
Rgl3	-0.212255782	0.921473132
Lamp5	0.225069739	0.307478675
Rgmb	0.043623657	0.948104382
Tmem170b	-1.470361929	0.757498268
Rgp1	-0.041499365	0.948104382
Rgs1	0.598321621	0.89703631
Rgs10	-0.041249428	0.975800973
Rgs11	-0.09802701	0.860826773
Rgs12	-0.078174461	0.948407374
Rgs13	0.182404159	0.981502501

Snhg14	-0.224269905	0.30307072
Rgs16	-0.030305073	0.985742667
Rgs17	0.050895385	0.940994409
Klra2	-1.47089572	0.815987743
Rgs19	-0.010910175	0.99527876
Rgs2	-0.088592758	0.935897306
Rgs20	-0.010062131	0.99527876
Rgs22	-0.269948671	0.957499962
Rgs3	0.156349153	0.874971335
Rgs4	-0.122310592	0.719639497
Rgs5	-0.031803332	0.974823941
Rgs6	0.715686693	0.861397728
Rgs7	-0.082154778	0.900894985
Shank1	-0.22425259	0.215151964
Pex6	-0.223963042	0.082752362
Rgs9	0.168940621	0.911631946
Rgs9bp	0.370450763	0.910328122
Rgsl1	-0.552545612	0.661553809
Rhbdd1	0.214158283	0.578812716
Rhbdd2	-0.079780344	0.927659499
Rhbdd3	-0.154801463	0.838040823
Rhbdf1	0.007307398	0.99527876
Rhbdf2	0.653627321	0.789386933
Rhbdl1	-0.137843179	0.874252042
Rhbdl2	0.521544009	0.895520088
Rhbdl3	0.067018343	0.932673884
Rhcg	0.413837045	0.955993235
Rheb	0.034905751	0.961607556
Rhebl1	0.010841697	0.99527876
Rhno1	0.107824981	0.97041548
Rpl39	0.223684843	0.291313919
Rhoa	-0.018060556	0.984748462
Abcb8	-0.223339994	0.436138179
Rhobtb1	0.119565214	0.976978183
Rhobtb2	-0.128483873	0.689698377
Rhobtb3	-0.207240773	0.534501962
Rhoc	-0.099660409	0.965520236
Rhod	0.383484521	0.922306337
Rhof	0.086942106	0.968005385
Rhog	0.248448602	0.665532517
Rhoh	0.070366706	0.987814724
Rhoj	0.275179703	0.790508337
Atp5j2	0.22308238	0.050086835
Rhot1	-0.04550619	0.950460219

Rhot2	0.084664333	0.942511266
Sept6	-0.222884017	0.260861196
Rhov	0.232094837	0.701843345
Rhpn1	0.031732987	0.98526072
Rhpn2	0.171417726	0.861533056
Sh3glb1	-0.222698568	0.19334431
Ribc1	0.627856916	0.860521845
Ribc2	0.443175101	0.932673884
Ric1	-0.11739474	0.911631946
Ric3	-0.060305377	0.894953269
Ric8a	0.027300922	0.969755848
Ric8b	-0.060745246	0.932673884
Rictor	-0.116007156	0.700214481
Bcl9	-0.222627788	0.199328636
Rif1	-0.16591814	0.824579267
Riiad1	0.336201599	0.84870301
Rilp	-0.153972321	0.971661054
Entpd5	-0.222579068	0.420814913
Rilpl2	0.154768627	0.813744313
Rimbp2	-0.026772338	0.968005385
Rimbp3	-0.015399481	0.99527876
Rimkla	-0.162050295	0.707777756
Rimklb	0.160138611	0.559262988
Fbxl19	0.221829934	0.314194702
Rims2	0.020657833	0.985293518
Rims3	-0.005079829	0.99765039
Rims4	-0.104772687	0.911631946
Rin1	-0.118240525	0.655744783
Tomm5	0.221817182	0.436138179
Cyhr1	-0.221689776	0.239528315
Ring1	0.099034737	0.82627259
Rinl	0.374430737	0.871869598
Rint1	0.070916108	0.95520652
Riok1	-0.103234331	0.904346827
Riok2	0.124121145	0.740514972
Snx9	0.221649682	0.497690258
Riox1	-0.110711498	0.890704638
Riox2	-0.084661051	0.932673884
Ripk1	-0.266870635	0.575395756
Ripk2	-0.147718896	0.891972665
Foxd2os	-1.475785904	0.909547396
Ripk4	-0.330455063	0.948104382
Ripor1	-0.122492098	0.58966519
Rbm19	-0.221631216	0.498066773

Ripor3	-0.333495175	0.985742667
Ripply2	-0.091944537	0.974823941
Nek1	-0.221603568	0.399438388
Rit1	0.060885388	0.964011524
Rit2	0.107007481	0.782459189
Rita1	0.006590214	0.996070372
Rlbp1	-0.012835634	0.99165967
Rlf	-0.031879352	0.980226283
Rlim	0.047570768	0.932673884
Rln1	-0.326449354	0.958389182
Rpl36al	0.22152203	0.243596082
Rmc1	-0.071362869	0.911631946
Rmdn1	-0.149485022	0.909547396
Rmdn2	0.228846462	0.894953269
Rmdn3	0.045128508	0.958389182
Rmi1	-0.072220556	0.953505913
Rmi2	-0.147907455	0.99527876
Rmnd1	-0.114885508	0.828907376
Rmnd5a	-0.246094921	0.559262988
Rmnd5b	-0.132757733	0.713014362
Rmrp	0.804193472	0.872954356
Lrrtm2	-0.221393009	0.445369233
Rn7s1	-0.836766134	0.90918338
Rn7s2	-0.953745473	0.902293377
Rn7sk	-0.48779063	0.928672524
Rnase1	0.588275109	0.891972665
Rnase10	0.094344833	0.99527876
Brip1	-1.484055259	0.837755777
Rnase2b	-0.710360009	0.911631946
Rnase4	-0.025997984	0.984748462
Rnase6	-0.071253328	0.99527876
Rnaseh1	0.169447204	0.819778986
Rnaseh2a	0.016813807	0.991438757
Rnaseh2b	0.105450954	0.948104382
Fgfr2	-0.221179304	0.482385306
Rnasek	0.824468967	0.73783619
Rnasel	0.252511806	0.890704638
Rnaset2a	0.146548876	0.80931022
Rnaset2b	0.161967962	0.701191891
Rnd1	-0.2394434	0.754134958
Rnd2	-0.426426336	0.837755777
Rnd3	-0.051280761	0.965520236
Rnf10	-0.003839898	0.99527876
Rnf103	-0.032660115	0.976978183

Rnf11	-0.014880346	0.981641687
Dnah9	-0.221053977	0.395640848
Rnf112	0.102487761	0.774225415
Rnf113a1	0.032138489	0.985742667
Rnf113a2	-0.047520544	0.965520236
Nkain4	0.221049925	0.467019925
Rnf115	-0.003916165	0.99527876
Rnf121	0.008353977	0.99527876
Rnf122	-0.138348927	0.962363869
Rnf123	-0.080069446	0.904750766
Rnf125	0.192717401	0.966213117
Rnf126	0.10385923	0.873797321
Rnf128	-0.325119672	0.909547396
Rnf13	-0.622386068	0.56399886
Rnf130	-0.067930822	0.944494911
Rnf135	-0.177637369	0.911631946
Rnf138	-0.156953317	0.69189201
Rnf138rt1	-0.091077395	0.987342614
Rnf139	0.06478802	0.912042382
Rnf14	-0.048688647	0.909547396
Rnf141	-0.062235678	0.869316117
Rnf144a	0.373929334	0.953505913
Rnf144b	0.14673599	0.871869598
Rnf145	0.012463079	0.986246396
Rnf146	0.013713778	0.985742667
Rnf149	-0.187364843	0.912917153
Rnf150	-0.070276069	0.891972665
Rnf152	-0.249180976	0.86783605
Rnf157	-0.016780986	0.981045362
Rnf165	-0.02968308	0.971661054
Rnf166	0.058854213	0.962648047
Rnf167	0.173883647	0.732729469
Rnf168	-0.088458355	0.86467336
Rnf169	0.053039372	0.947745582
Rps12-ps19	-1.4842754	0.948104382
H2afz	0.220957171	0.128640258
Rnf170-ps	-0.104170387	0.982437522
Rnf180	0.000162853	0.999653079
Rnf181	0.044546126	0.968005385
Rnf182	0.076320897	0.871869598
Smlr1	-1.485337686	0.84277074
Rnf185	0.050936359	0.954469162
Rnf187	0.036974414	0.959659722
Rnf19a	0.045251874	0.962636653

Rnf19b	0.179582909	0.764044481
Tmem88b	-0.220786293	0.343659999
Rnf20	-0.077358432	0.871869598
Rnf207	0.182346438	0.920642026
Rnf208	0.071903824	0.99527876
Rnf213	-0.268170043	0.607960928
Rnf214	-0.069720739	0.90358675
Rnf215	-0.075155399	0.910328122
Rnf216	-0.082235012	0.87806697
Rnf217	-0.130243443	0.938939871
Rnf219	-0.057054338	0.965520236
Rnf220	0.054493631	0.926297546
Rnf223	-0.17252575	0.968005385
Rnf225	0.456891079	0.968005385
Rnf227	-0.279124408	0.936507368
Rnf24	-0.190428877	0.59983323
Rnf25	0.163650491	0.632633706
Rnf26	-0.084090564	0.991996237
Rnf31	0.024935396	0.984232465
Rnf32	-0.027806651	0.98526072
Rnf34	0.012473582	0.985742667
Rnf38	-0.121868842	0.578812716
Rnf39	0.336517999	0.835345968
Rnf4	-0.057288843	0.881371608
Rnf40	-0.001940329	0.997115148
Rnf41	-0.041655914	0.950417507
Rnf43	-0.040981534	0.99100709
Rnf44	-0.03109994	0.965752549
Rnf5	0.017634714	0.98526072
Rnf6	-0.114836673	0.898611672
Rnf7	0.050826537	0.937980401
Rnf8	0.03922249	0.980339705
Rnft1	-0.189183377	0.543640901
Rnft2	-0.052656191	0.939060335
Rngtt	-0.099617837	0.8549794
Rnh1	0.074835782	0.909728442
Rnls	0.582183595	0.663732405
Rnmt	0.06700704	0.985735386
Rnpc3	-0.111058808	0.91462326
Rnpep	0.071468487	0.916961045
Rnpepl1	-0.095734677	0.895520088
Rnps1	0.184179749	0.52053053
THSD8	-1.488248354	0.911631946
Rnu2-10	0.33445141	0.985742667

Syce1l	-1.492007531	0.890580085
Rnu7	0.08251524	NA
Rnu73b	0.337385115	0.974197921
Hnmt	0.220522268	0.421157413
Robo2	-0.086041144	0.828907376
Robo3	-0.011805905	0.994806961
Robo4	0.113874806	0.962648047
Rock1	-0.087266057	0.911631946
Rock2	-0.056396792	0.922100633
Rogdi	0.047396938	0.946239011
Rom1	0.089579562	0.971661054
Zdhhc2	-0.220314422	0.235039388
Ropn1l	0.65191762	0.86783605
Ror1	0.081474398	0.962648047
Ror2	0.313910016	0.981502501
Rora	0.621153225	0.928672524
Raver2	-0.219847167	0.200754354
Rorc	-0.041134139	0.988034453
Rp1	-0.033216802	0.996431931
Rp2	-0.053428929	0.973100313
Rp9	0.052888018	0.961373288
Rpa1	-0.079371911	0.882096017
Rpa2	0.117336396	0.91462326
Rpa3	0.115401321	0.950252558
Rpain	0.012502179	0.993380259
Rpap1	-0.111389002	0.901479608
Rpap2	0.017705088	0.985742667
Rpap3	0.071441021	0.935897306
Rpe	-0.045211668	0.974823941
Rpe65	-0.487808878	0.970348278
Rpf1	0.220814073	0.764783842
Rpf2	0.13212873	0.911631946
Rpgr	0.135445802	0.889972515
Rpgrip1	-0.046358209	0.99527876
Rpgrip1l	-0.170752605	0.708001635
Rph3a	0.051358577	0.936031628
Rph3al	-0.071510334	0.983694404
Rpia	0.146892165	0.894953269
Rpl10	0.128700638	0.810908327
Rpl10-ps1	0.114293128	0.627261794
Rpl10-ps2	0.525383159	0.875723186
Rpl10-ps3	0.121664826	0.539353804
Rpl10-ps5	0.030327354	0.971661054
Phkg1	0.219645399	0.431890949

Rpl10a	0.109909744	0.751863834
Rpl10a-ps1	0.115529912	0.739376483
Spred1	-0.21959846	0.016957576
Rpl12	0.092517073	0.822693227
Rpl12-ps1	0.041124001	0.998010523
Rpl13	0.150302502	0.544361994
Dthd1	-1.501230971	0.90358675
Rpl13-ps3	0.618039987	0.813744313
Gbx2	-1.504399625	0.952385926
Rpl13-ps6	0.034418012	0.98340069
Tns3	0.219185656	0.126698562
Scara3	-0.218936692	0.463966612
Pcdh1	-0.21880343	0.109320915
Rpl15	0.090892417	0.78686705
Rpl15-ps2	0.375924619	0.710375942
Rpl15-ps3	0.052305689	0.944753667
Rpl15-ps5	0.440180379	0.956164302
Cacna1g	-0.218357952	0.399438388
Zfp81	-0.218322977	0.237871883
Txnl4b	0.218244321	0.404461151
Cmtm6	-0.218207769	0.504583609
Cox5b	0.217881977	0.459780215
Tpm1	0.217841306	0.319241271
Dpysl2	0.21779203	0.488852978
Rpl18	0.093687176	0.771684575
Rpl18-ps1	0.661310832	0.839488666
Rpl18-ps2	-0.450386058	0.89703631
Nedd8	0.217761701	0.114293014
Rpl18a-ps1	-0.927468326	NA
Rpl19	0.102164901	0.604176395
Rpl19-ps1	0.295559298	0.667733898
Ube2m	0.217517825	0.144051788
Rpl19-ps3	-0.868204984	0.968005385
Rpl19-ps6	0.396775714	0.80931022
Rpl21	0.089136917	0.740240396
Rpl21-ps10	0.284927243	0.959276014
Klhdc7b	-1.50447666	0.639505683
Rpl21-ps13	0.689175273	0.984232465
Rpl21-ps14	0.636353201	0.981392364
Il16	0.217394958	0.31865776
Rpl21-ps6	-0.329804917	0.965520236
Rpl21-ps7	-0.246576974	0.984232465
Rpl21-ps8	0.530743107	0.663393433
Id2	0.21722563	0.318279733

Duox2	-1.504597043	0.952385926
Rpl22l1	0.04767806	0.962648047
Cwc27	0.217113635	0.456330081
Actr8	0.217016178	0.244113683
Rpl23a-ps3	0.119618716	0.89703631
Rpl24	0.067263829	0.87564489
Sh3bp4	-0.216832466	0.497972882
Rpl26-ps6	-0.952476156	0.89703631
Tcaim	-0.216733389	0.445106721
Rpl27-ps1	0.454048293	0.708953765
Rpl27-ps2	-0.637356182	0.984328758
Pcbp3	0.216721093	0.240817904
Prmt3	-0.216593346	0.255356792
Serpina3m	-1.504597043	0.952385926
Cul2	-0.216253583	0.323832374
Rpl28-ps1	0.17634574	0.628383657
Rpl28-ps3	0.500869017	0.83265523
Nup214	-0.216243442	0.467859532
Rpl29-ps2	0.29430227	0.923011738
Rpl3	0.062678884	0.858549868
Rpl3-ps1	0.062861315	0.898352299
Rpl3-ps2	0.320315391	0.774896252
Itgb1bp1	-0.216203934	0.459780215
Rpl30-ps1	0.084722793	0.984328758
Rpl30-ps10	0.284384048	0.728733751
Rpl30-ps2	0.574948731	NA
Rpl30-ps3	0.284252982	0.980731359
Rpl30-ps8	0.137448053	0.965520236
Rpl30-ps9	0.301318988	0.777427974
Cox7b	0.216069445	0.306736562
Rpl31-ps10	-0.670010319	0.979037366
Trappc3	0.215752845	0.207659271
Rpl31-ps12	0.200491439	0.837755777
Rpl31-ps13	0.175718645	0.945647427
Rpl31-ps14	0.469150533	0.874252042
Rpl31-ps15	-0.088232362	0.99527876
Rpl31-ps16	-0.45407483	0.972218452
Rpl31-ps17	-0.322036276	NA
Rpl31-ps4	0.937152122	0.901479608
P4ha1	-0.215576719	0.40161727
Ddr1	-0.215572595	0.404892636
Tob1	0.214933957	0.42493601
Rpl27a-ps2	-1.507736444	0.89703631
Rpl34	0.04141856	0.957849305

Rpl34-ps1	0.160900336	0.962648047
Chit1	-1.518832238	0.922900813
Soga3	-0.214907013	0.323832374
Rbm3	0.214361487	0.216777095
Rpl35a-ps2	0.33951425	0.74463793
Rpl35a-ps3	0.521542978	0.589856572
Rpl35a-ps4	0.515384561	0.603499717
Rpl35a-ps5	0.65348033	0.788592804
Rpl35a-ps6	0.535148242	0.566580926
Tlcd2	-1.520188616	0.852035098
Fgd6	-0.21409446	0.485827814
Slc28a1	-1.520703804	0.951390592
Lrmda	-1.529444519	0.89703631
Rpl36-ps3	-0.369867539	0.981502501
Slc24a2	-0.21396732	0.204010697
Rpl36a-ps1	0.391030577	0.573810203
Smg1	-0.213782631	0.010278451
Rpl36a-ps3	0.442278729	0.715121059
Exoc6b	-0.213735299	0.160287523
Prrc2c	-0.213666353	0.216245761
Epg5	-0.2134794	0.295213318
Ttc39c	0.213350989	0.227955785
Nat10	-0.213134747	0.178171285
Rpl38-ps1	-0.812818542	0.973069492
Rpl38-ps2	0.313547915	0.620124996
Mrpl48	-0.213111333	0.508471705
Rpl39-ps	0.315480521	0.963943597
Msr1	-1.531128087	0.740833211
AC140278.2	-1.533163415	0.92517502
Rpl4	0.074777913	0.783120889
Rps7	0.213094679	0.161844023
Rpl5	0.104143568	0.745879732
Rpl5-ps1	0.100841002	0.869316117
Ndufb7	0.212420132	0.41351939
Rpl6	0.102222607	0.84870301
Smc3	-0.212292355	0.209137754
Rpl7	0.085617023	0.890704638
Rpl7-ps7	0.236255418	0.936031628
Rasgrp1	-0.21223134	0.276254053
Hist1h3d	-1.534091946	0.861139061
Pou3f3	-0.211977743	0.237504632
Rpl7a-ps3	0.225156654	0.953505913
Rpl7a-ps5	0.378305587	0.6883862
Rpl7a-ps7	0.441471221	0.965520236

Rpl7l1	0.128874633	0.59983323
Rpl8	0.11263237	0.573101161
Rpl9	0.062576187	0.842727003
Ooep	-1.53545183	0.905281292
Rpl9-ps4	0.142635343	0.772658285
Nfix	-0.211516321	0.477881972
Rpl9-ps7	0.005402559	0.997258205
Rpl9-ps8	0.620847759	0.974962198
Rplp0	0.064796982	0.850260513
Slc9a2	-0.211125772	0.353015192
Srrm2	-0.21103252	0.434762828
Rpn1	-0.03454872	0.958893576
Rpn2	0.000408083	0.99900438
Rpp14	-0.065623598	0.90358675
Rpp21	0.124266373	0.918655498
Rpp25	0.279225563	0.629280869
Rpp25l	0.144120979	0.889972515
Rpp30	0.155879565	0.764358191
Rpp38	-0.270722355	0.777545346
Rpp40	-0.057672894	0.975831674
Rpph1	0.623237925	0.962648047
Rprd1a	-0.068148054	0.829694131
Rprd1b	0.29221619	0.5958847
Rprd2	-0.077651345	0.897044791
Pgbd5	0.210883675	0.237504632
Rprml	-0.017507403	0.989402966
Asph	-0.210589191	0.421691269
Tmem26	-1.536262822	0.950460219
Rps10-ps1	0.865892126	0.593692285
Gabpa	-0.210540935	0.342676783
Fabp5	0.210520991	0.122206254
Rps11-ps1	0.670383137	0.894148925
Rps11-ps2	-0.163430685	0.989840188
Rps12	0.099134003	0.889972515
Rps12-ps1	0.863412513	0.860521845
Rab9b-ps1	-1.537435622	NA
Rps12-ps12	0.047462582	NA
Hormad2	-1.538267657	0.883131585
Rps12-ps24	0.308257572	0.958299088
Rps12-ps26	-0.149689433	0.988034453
Rps12-ps3	0.091761757	0.894953269
Rps12-ps4	0.156285214	0.895520088
Rps12-ps9	0.215236482	0.771450635
Mrpl30	0.210488958	0.131091021

Ufc1	0.210320014	0.161844023
Fam160a2	-0.210313209	0.128640258
Ip6k2	0.210193003	0.065333411
Rapgef3	0.210049612	0.420814913
Dr1	-0.209810966	0.310002108
Tnfrsf19	-0.209476527	0.275950342
Robo1	-0.209472445	0.157397825
Rps15a-ps3	0.349939388	0.953505913
Cyp3a57	-1.544535417	0.950195611
Rps15a-ps5	0.463191104	0.962648047
Rps15a-ps6	0.683205198	0.949550088
Rps15a-ps7	0.220926914	0.872265039
Rps16	0.110252965	0.810908327
Rps16-ps2	0.107222854	0.873797321
Rplp2	0.209312242	0.160287523
Ndufa8	0.209030928	0.065333411
Mir466i	-1.545600575	0.928099251
Rps18-ps3	0.377094735	0.668637933
Rps19	0.102789703	0.731137993
Rps19-ps1	0.594311203	0.953505913
Rps19-ps2	0.624895932	0.938615214
Rps19-ps3	0.374696996	0.864121718
Rps19-ps4	0.50781167	0.7851601
Zeb2	-0.208971309	0.241783095
Rps19-ps7	-0.413283671	0.962648047
Rps19bp1	0.17201057	0.869316117
Ppp1r8	0.208876676	0.123600847
Rps2-ps10	0.050465463	0.966010627
Emc10	-0.208663928	0.404892636
Rps2-ps13	0.008594931	0.99527876
Ptcra	-1.561050836	0.89383516
Rps2-ps6	0.093355093	0.965290366
AC165953.1	-1.568794074	0.82972991
Rps20	0.059613914	0.948104382
Tmem126a	-0.20843051	0.465255523
Prdm8	0.208354062	0.310553098
Akap9	-0.208282222	0.322635312
Rps23-ps2	0.116863305	0.911631946
Ctsl	0.208106839	0.154257956
Dlg5	0.208092514	0.337479705
Rps25	-0.235803142	0.605064513
Rps25-ps1	0.170530855	0.911631946
Ndufa6	0.208014763	0.360222394
Rps26-ps1	0.303866245	0.86783605

Vxn	-0.207978121	0.411812468
Togaram1	-0.207947917	0.498066773
Rps27a-ps1	-0.22741355	0.989464948
Rps27a-ps2	0.141531195	0.621993647
Rps27l	0.240666013	0.605700484
Mtg1	0.207777076	0.417721745
Psmd4	0.207631137	0.254652818
Rps29	0.183544	0.63519309
Cplane1	-0.207578841	0.417721745
Rps3a1	0.118498571	0.540997524
Lamtor2	0.207518339	0.220337275
Meis2	-0.20735575	0.370298654
Gpr26	-0.207194688	0.452387635
Rps4x	0.08554744	0.673602191
Sfrp5	-1.574773836	0.899271839
Atp2c1	-0.207115151	0.346782424
Ptpn23	-0.207079637	0.380767313
Rps6-ps1	-0.024086829	0.99527876
Rps6-ps3	0.401426254	0.778941315
Tenm2	-0.206965059	0.080124665
Rps6ka1	-0.047710706	0.98043358
Rps6ka2	0.121331671	0.78752614
Rps6ka3	0.689113624	0.783611405
Rps6ka4	0.040695033	0.985742667
Rps6ka5	0.109633572	0.962648047
Rps6ka6	-0.092674833	0.970461999
Rps6kb1	-0.095645563	0.858549868
Rps6kb2	0.112987053	0.837096894
Rps6kc1	0.048411197	0.956164302
Rps6kl1	0.050555059	0.97487264
Ssbp1	0.206908998	0.477637546
Rps7-ps1	0.746788196	0.965520236
Avl9	-0.206719551	0.146214787
Rps8	0.114323394	0.724910334
Rps8-ps1	0.223487888	0.940060336
Rps8-ps2	0.233188407	0.89260868
Rps8-ps4	0.235335276	0.981392364
Rps9	-0.0174364	0.984748462
Trpm2	-0.206463331	0.480408134
Rpsa-ps1	0.171427685	0.962648047
Rpsa-ps10	0.093326566	0.895326715
Rpsa-ps12	0.590445079	0.871869598
Rpsa-ps2	0.278964291	0.563984287
Rpsa-ps4	0.74559841	0.971661054

Rpsa-ps5	0.277148131	0.962648047
Rpsa-ps9	0.234678683	0.878577494
Rptor	-0.068975634	0.894953269
Pmch	-1.576205841	0.791185365
Rpusd1	-0.070709681	0.920642026
Rpusd2	0.164806035	0.911631946
Rpusd3	0.094279303	0.948104382
Rpusd4	0.043425988	0.981600842
Rrad	-0.54794986	0.862742768
Rraga	0.077301513	0.866574046
Rragb	0.008256987	0.992165685
Rragc	0.060456381	0.91082801
Rragd	0.087427026	0.870812051
Rras	-0.24927395	0.65012378
Rras2	0.067738203	0.952034929
Rrbp1	0.04850286	0.962648047
Rreb1	0.019020177	0.992669879
Rrh	-0.647147232	0.963666931
Rrm1	0.116612594	0.795563654
Rrm2	0.296892947	0.938414123
Rrm2b	0.003245213	0.997115148
Rrn3	0.063611404	0.90918338
Rrnad1	0.06999303	0.951755452
Rrp1	0.035056657	0.955993235
Rrp12	-0.125834953	0.740833211
Gapdh	0.206340897	0.091861512
Rrp1b	-0.005463593	0.99527876
Rrp36	0.22877091	0.613419844
Rrp7a	0.06869419	0.921473132
Rrp8	-0.099702163	0.971138085
Rrp9	0.057639858	0.964011524
Rrs1	0.000755519	0.998829745
Hcst	-1.580427334	0.884426973
Rsad1	-0.027320031	0.985742667
Rsad2	0.034074813	0.99527876
Rsbn1	-0.088351675	0.973100313
Rsbn1l	-0.153704135	0.852010446
Rsc1a1	-0.033479077	0.975831674
Rsf1	0.051202071	0.966213117
Rsf1os2	0.294108003	0.978063275
Rsg1	-0.685631973	0.770166577
Rsl1	0.218292478	0.802538464
Rps24	0.206086291	0.055853534
Neurod6	-0.205810325	0.252638478

Ms4a4a	-1.58257303	0.835960581
Rsph1	0.045107107	0.985673938
Rsph10b	0.008868164	0.996094752
Rsph14	-0.084553673	0.99527876
Rsph3a	-0.033567889	0.974809035
Rsph3b	-0.015072941	0.99527876
Rsph4a	-0.477485411	0.513882458
Rsph6a	-0.070989788	0.99527876
Rsph9	-0.00971265	0.99527876
Rspo1	0.178860942	0.968005385
Rspo2	0.043692049	0.972387414
Mrpl41	0.205770323	0.234691956
Rspry1	-0.143322892	0.689722735
Atxn2	-0.205632227	0.200754354
Rsrc2	-0.029736301	0.974962198
Rsrp1	0.090262919	0.904480183
Rsu1	0.025441641	0.980626239
Rtbdn	-0.488296515	0.65469339
Cops9	0.20549703	0.471017922
Rtcb	0.114747353	0.531294496
Rtel1	-0.175012476	0.762758921
Rtf1	0.0265642	0.968005385
Rtf2	0.02662617	0.966917555
Rtkn	0.134849303	0.804491173
Rtkn2	0.346626515	0.852946549
Rtl1	-0.081048135	0.968005385
Rtl10	0.218062942	0.953505913
Rtl3	0.276242261	0.818346815
Rtl4	0.030993339	0.991438757
Rtl5	-0.697131174	0.878621231
Rtl6	-0.100837395	0.803910915
Rtl8a	0.021698896	0.981502501
Rtl8b	0.032270063	0.974085322
Rtl8c	0.148241899	0.549850404
Rtl9	0.35537085	0.887625873
Rtn1	0.01214958	0.985742667
Rtn2	0.027736541	0.974809035
Rtn3	-0.063068362	0.825910982
Rtn4	-0.032710771	0.981045362
Rtn4ip1	-0.075854551	0.954543296
Usp46	-0.205309728	0.225616139
Rtn4rl1	0.000173621	0.999486844
Rtn4rl2	-0.207721433	0.707520358
Rtp1	0.439011436	0.6883862

Rtp2	0.063473886	0.99527876
Rtp3	-0.073096589	0.99527876
Rtp4	0.622267548	0.663097563
Rtraf	0.08130964	0.874273224
Rttn	-0.087265221	0.965520236
Rubcn	0.151803244	0.965520236
Rubcnl	0.066968313	0.988034453
Rufy1	0.007171964	0.994960308
Rufy2	0.034433339	0.957499962
Rufy3	0.014422049	0.98526072
Actb	0.204495638	0.043661658
Rundc1	-0.048499652	0.960176242
Rundc3a	0.045730906	0.921102947
Rundc3b	0.014388488	0.985742667
Runx1	0.031776762	0.99527876
Runx1t1	0.155238632	0.984328758
Runx2	0.166883876	0.874252042
Runx2os1	-0.369959987	0.954543296
Rusc1	-0.045325571	0.959276014
Rusc2	0.054895047	0.971661054
Ruvbl1	0.033510602	0.969755848
Ruvbl2	0.129254898	0.832383921
Rwdd1	0.018874249	0.985742667
Rwdd2a	0.077615102	0.951497773
Rwdd2b	0.15647047	0.705443353
Rwdd3	0.083541273	0.980419928
Rwdd4a	-0.213851234	0.8549794
Cyp2f2	-1.592002949	0.883333293
Rxfp2	0.634124611	0.932673884
Rxfp3	-0.015531198	0.99527876
Rxra	0.058533928	0.97487264
Rxrb	-0.035442388	0.962648047
Rxrg	-0.276196179	0.944753667
Rxylt1	0.077316468	0.89703631
Rybp	-0.150472583	0.593692285
Rybp-ps	0.037877883	0.99527876
Ryk	-0.105165822	0.76034043
Ryr1	0.208515921	0.664907827
Ryr2	-0.194053512	0.800386557
Nxf1	0.204430314	0.344888915
S100a1	0.198363927	0.544661714
S100a10	-0.051659235	0.965520236
S100a11	0.021095101	0.99527876
S100a13	0.081260273	0.952185901

S100a16	0.172149364	0.738901675
S100a3	0.771186921	0.861378744
S100a4	0.211495232	0.87564489
S100a6	0.010114019	0.99527876
Omd	-1.592754463	NA
S100a8	-0.674258802	0.955903963
S100a9	-0.655855202	0.922887518
S100b	0.108267806	0.727420355
S100pbp	-0.136962922	0.698427195
S1pr1	-0.053568008	0.911276003
S1pr2	0.235056501	0.90358675
S1pr3	0.041587141	0.976978183
S1pr4	-0.624917399	0.887135194
S1pr5	-0.044726265	0.974809035
BC060293	-1.594712808	0.724985969
Saa2	-0.21834798	0.993103295
Grip1os3	-1.597293938	0.911631946
Saal1	-0.245987631	0.527794911
Sac3d1	0.274201624	0.571020232
Sacm1l	-0.103258895	0.70685392
Sacs	-0.044615241	0.962648047
Sae1	0.075869406	0.869316117
Safb	-0.011519231	0.985742667
Safb2	-0.042566105	0.962923888
Sag	-0.03157984	0.994960308
Sall1	-0.09423778	0.921473132
Psmc1	0.204249917	0.146214787
Sall3	0.06147211	0.975831674
Sall4	0.375697534	0.955993235
Samd1	-0.022883967	0.98771048
Samd10	0.15444192	0.767515266
Samd11	-0.293065654	0.956922504
Samd12	-0.093366657	0.852332064
Ephx3	-1.599627995	0.882339809
Samd14	0.078062053	0.869316117
Samd15	0.074808464	0.984305885
Samd4	0.86830002	0.630888733
Samd4b	-0.129142792	0.74463793
Samd5	-0.246535462	0.628806842
Samd8	-0.018692009	0.975831674
Samd9l	-0.029889463	0.993489613
Samhd1	0.043849161	0.968630572
Samm50	0.046065122	0.918407269
Samsn1	-0.162740038	0.969755848

Sap130	-0.023584975	0.976978183
Dmac2	0.204062728	0.408765725
Klf3	-0.203831207	0.349775121
Sap25	-0.577277522	0.691826732
Sap30	0.146695599	0.891972665
Sap30bp	0.029343266	0.971661054
Sap30bpos	-0.190589997	0.966213117
Sap30l	0.053505192	0.968005385
Sapcd2	-0.130884864	0.975831674
Sar1a	0.036496865	0.99527876
Sar1b	0.130432324	0.54807923
Saraf	-0.000642437	0.998658264
Sardh	-0.036828444	0.98526072
Sarm1	-0.388631721	0.670918119
Sarnp	-0.055091404	0.956164302
Sars	0.07127709	0.837755777
Sars2	0.092153518	0.953505913
Sart1	0.064626015	0.90872336
Sart3	0.059378125	0.948432875
Sash1	-0.137905823	0.573810203
Sash3	-0.613348509	0.5141747
Sass6	0.311367398	0.785603619
Sat1	0.136073984	0.890704638
Sat2	0.156710749	0.757498268
Satb1	-0.075388224	0.910328122
Satb2	-0.217845392	0.74463793
Satl1	-0.095969952	0.99527876
Sav1	0.098860771	0.923011738
Saxo2	0.001095556	0.999242708
Saysd1	-0.006280498	0.99527876
Chordc1	-0.203706624	0.436138179
Zfp397	-0.203371478	0.435080924
Sbf2	0.041753056	0.97487264
Rian	-0.202948019	0.167322542
Sbk2	0.796199784	0.962648047
Sbk3	0.040525663	0.993103295
Sbno1	-0.07721255	0.798109023
Sbno2	-0.036280505	0.979037366
Sbsn	0.262389721	0.815987743
Ttc39d	-1.60155631	0.895204216
Sc5d	-0.079255478	0.838040823
Scaf1	0.083063289	0.799736155
Scaf11	-0.018532475	0.985673938
Scaf4	-0.07334183	0.932673884

Scaf8	-0.079791914	0.889972515
Scai	-0.611253738	0.852010446
Scamp1	0.027010453	0.962648047
Scamp2	-0.014172236	0.991438757
Scamp3	0.008309804	0.994960308
Lmo7	-0.20273366	0.421157413
Scamp5	-0.009116836	0.989840188
Scand1	0.246698923	0.698018236
Scap	-0.032786654	0.962648047
Scaper	-0.05041909	0.953505913
Camk2n1	0.202535501	0.205552132
Scara5	-0.167223646	0.99527876
Scarb1	-0.031405043	0.97487264
Scarb2	0.01760714	0.980132658
Scarf1	-0.07856594	0.976978183
Scarf2	-0.18555469	0.962648047
Scarna13	0.340761714	0.968005385
Olfr482	-1.61202746	0.942511266
Scarna2	-0.121911259	0.99527876
Trcg1	-1.612101208	0.833916587
Scarna6	0.078122755	0.99527876
Cmah	-1.619859811	0.911631946
Sccpdh	0.149889436	0.873753967
Scd1	0.014861857	0.98526072
Scd2	-0.049179991	0.895520088
Scd3	0.169568131	0.958389182
Scd4	0.173359843	0.981694523
Scel	0.288346626	0.923011738
Scfd1	-0.052580945	0.965520236
Scfd2	0.004074793	0.996094752
Scg2	-0.043447774	0.962648047
Scg3	0.041119541	0.945914524
Scg5	0.024295842	0.974962198
Scgb1c1	-0.168373804	0.99527876
Scgn	0.227498962	0.911631946
Schip1	0.075456045	0.851875536
Scin	-0.336418799	0.871869598
Sclt1	0.236554165	0.861366392
Scly	-0.054109659	0.968005385
Scmh1	-0.06703068	0.962648047
Scml2	0.35619816	0.916938833
Scml4	0.049550926	0.985446046
Scn1a	0.035801252	0.993103295
Scn1b	0.086578592	0.7851601

Foxn2	-0.20244618	0.442302609
Scn2b	0.025210119	0.970348278
Scn3a	0.266581602	0.936031628
Scn3b	-0.237859193	0.694560824
Mir6403	-1.621474947	0.890704638
Scn4b	0.066810944	0.91507125
Scn5a	-0.20535263	0.957849305
Scn7a	0.365102955	0.760547704
Scn8a	0.219954457	0.965520236
Scn9a	0.255462952	0.897878088
Cdkl2	-0.202121846	0.483420706
Scnn1a	0.363859251	0.731137993
Raet1d	-1.6255819	0.84870301
Sco1	-0.096493504	0.92543611
Sco2	0.249090264	0.909547396
Scoc	-0.125986076	0.640856315
Scp2	0.006775492	0.994023148
Capns2	-1.625914575	0.89383516
Scpep1	-0.099982364	0.92517502
Scpep1os	-0.929150012	0.940431936
Slc16a1	-0.202067959	0.148467541
Scrib	-0.139842216	0.666177474
Scrn1	-0.076428668	0.770166577
Scrn2	-0.079211822	0.979400804
Scrn3	-0.15900547	0.563984287
Scrt1	-0.014340923	0.987814724
Scrt2	0.056234154	0.962648047
Sctr	-0.801952502	0.886647768
Scube1	-0.127636022	0.886895881
Scube2	-0.264160141	0.894953269
Scube3	-0.043414921	0.989640643
Scx	-0.13399434	0.97487264
Scyl1	0.016227843	0.984328758
Scyl2	-0.021322309	0.993103295
Scyl3	-0.107982517	0.882096017
Sdad1	0.097806544	0.874326117
Sdc1	-0.446477704	0.911276003
Sdc2	-0.018758097	0.98526072
Sdc3	0.022024809	0.974823941
Naca	0.201531461	0.1403916
Sdcbp	0.067131463	0.861366392
Sdcbp2	0.015420448	0.99527876
Sdccag8	0.009002348	0.992385525
Sde2	-0.093705436	0.886647768

Sdf2	0.196019597	0.702951568
Sdf2l1	0.067699042	0.965520236
Sdf4	0.062491967	0.873797321
Sdha	0.056782482	0.873797321
Sdhaf1	0.150537572	0.80931022
Sdhaf2	-0.000439442	0.999223454
Wscd2	-0.201379535	0.399005965
Sdhaf4	0.067695853	0.940431936
Fam32a	0.201364349	0.067353494
Sdhc	0.047920633	0.917685272
Sdhd	0.031628423	0.97469529
Sdk1	-0.228883952	0.685353017
Sdk2	0.022800632	0.985742667
Sdr39u1	0.088059121	0.872612157
Sdr42e1	0.017474504	0.99527876
Sdr9c7	-0.37250988	0.985673938
Sds	-0.387631789	0.937550602
Sdsl	0.180774891	0.912397394
Sebox	-0.111538843	0.921311891
Sec1	0.185358817	0.963666931
Sec11a	0.098974706	0.91462326
Sec11c	0.08584157	0.871869598
Sec13	0.126598109	0.513797506
Sec14l1	-0.029053368	0.964941885
Sec14l2	0.10952446	0.727908091
Sec14l3	0.173415251	0.976978183
Sec14l4	0.710883115	0.873753967
Sec14l5	0.095643843	0.911631946
Rida	0.201335153	0.418613599
Sec16b	-0.101275925	0.974823941
Sec22a	0.101110866	0.89703631
Sec22b	0.112307162	0.778639508
Sec22c	-0.031118401	0.969661076
Sec23a	-0.044291701	0.922117688
Sec23b	-0.019364766	0.983219084
Sec23ip	-0.021513606	0.979926967
Sec24a	-0.173609907	0.635646343
Sec24b	-0.027728387	0.969755848
Sec24c	-0.004283576	0.99527876
Sec24d	0.085725723	0.89703631
Sec31a	0.081232833	0.921117568
Cstf3	-0.200841854	0.234691956
Sec61a1	-0.05154094	0.949111839
Sec61a2	0.030688353	0.974823941

Sec61b	-0.052702967	0.971484753
Pot1a	-0.200787884	0.500548155
Sec62	-0.045507592	0.950195611
Sec63	-0.027715735	0.970461999
Secisbp2	-0.070292136	0.93178771
Secisbp2l	-0.122014355	0.624713015
Seh1l	-0.017481858	0.984748462
Scamp4	-0.200725993	0.243596082
Sel1l3	-0.090517257	0.873753967
Dock4	-0.200072736	0.177297397
Selenbp2	0.526462639	0.916287981
Selenof	0.181485219	0.861366392
Selenoh	-0.018494241	0.99527876
Selenoi	0.143122391	0.98302504
Selenok	0.097927845	0.737087313
Selenok-ps1	-0.436123222	0.971661054
Bglap	-1.635091077	0.946985353
Selenok-ps6	0.912033078	0.972387414
Selenom	0.094600908	0.858549868
Selenon	-0.202276995	0.644545791
Selenoo	0.016934687	0.989840188
Selenop	0.111151294	0.813792356
Atp6v0a1	-0.200016972	0.31865776
Selenot	-0.020347907	0.975831674
Selenov	0.747891645	0.808174635
Selenow	0.13577078	0.695239733
Sell	0.194328695	0.993103295
Selplg	-0.086162387	0.872612157
Sem1	0.014003225	0.989378233
Polr2e	0.199832735	0.337479705
Sema3b	0.009369474	0.99765039
Sema3c	0.04667964	0.971484753
Sema3d	0.010181405	0.995500778
Sema3e	-0.219915823	0.571020232
Nfyc	0.199812379	0.328249806
Sema3g	0.087863327	0.962936077
Sema4a	-0.10675649	0.869602864
Sema4b	0.072254285	0.955993235
Sema4c	0.0017965	0.998010523
Sema4d	-0.039134317	0.955993235
Sema4f	0.087340259	0.813346233
Sema4g	-0.179347375	0.703372057
Sema5a	0.068994352	0.962648047
Sema5b	0.076992647	0.964941885

Sema6a	-0.291562703	0.533008784
Sema6b	-0.025041719	0.974823941
Sema6c	-0.2772721	0.525749648
Ctnnd1	-0.199645748	0.146214787
Sema7a	-0.035378084	0.961300588
Senp1	-0.070777195	0.964011524
Abca8b	-0.199125413	0.456330081
Senp3	-0.133268729	0.821998633
Senp5	-0.105847257	0.770793864
Agtpbp1	-0.198935853	0.235039388
Senp7	0.056574818	0.965520236
Senp8	0.541522255	0.757721895
Sephs1	-0.105737276	0.882209226
Sephs2	0.03972483	0.962812066
Sepsecs	0.074126681	0.980896205
Sept1	0.031560353	0.993766047
Sept10	0.002671098	0.998010523
Sept11	-0.001648225	0.997529192
Sept2	0.06130971	0.942812446
Sept3	0.054477772	0.882096017
Sept4	0.04622808	0.966010627
Ptpn1	-0.198268432	0.423074171
Ptma	0.198220138	0.356806958
Sept7	-0.069827721	0.887135194
Sept8	0.103246725	0.639505683
Sept9	-0.221433842	0.664771806
Serac1	-0.119332887	0.844084751
Serbp1	-0.062841022	0.932673884
Serf1	-0.20427985	0.909243078
Serf2	-0.140646003	0.875007504
Sergef	0.2018457	0.770793864
Serhl	-0.098760958	0.958051038
Serinc1	-0.035614503	0.955993235
Serinc2	-0.27924162	0.596000466
Serinc3	0.04722073	0.91462326
Serinc4	0.382437509	0.957209681
Serinc5	0.028597162	0.971891838
Serp1	0.085535755	0.815987743
Serp2	0.06961152	0.911631946
Serpina10	0.594441037	0.911631946
Serpina12	-0.294194438	0.983479691
Serpina3c	0.36720081	0.939052665
Serpina3e-ps	-0.554574527	0.976993105
Serpina3g	0.197806606	0.933201883

Serpina3h	0.605433161	0.90358675
Serpina3i	0.532998957	0.968005385
Stpg2	-1.636937693	0.942812446
Serpina3n	-0.077195456	0.915417975
Serpinb1a	-0.095575464	0.951497773
Serpinb1b	0.055887853	0.991438757
Serpinb6a	0.085663418	0.874252042
Serpinb6b	-0.157842288	0.971661054
Serpinb6c	-0.297757877	0.984748462
Arhgap27os3	-1.637588228	0.891853883
Serpinb8	-0.261973901	0.852010446
Serpinb9	0.058188213	0.965520236
Serpinc1	-0.153066691	NA
Serpind1	-0.252308198	0.967485535
Serpine1	0.537578871	0.787947063
Serpine2	0.079568421	0.767515266
Tceal1	0.198016869	0.41351939
Serpinf1	0.111698155	0.968630572
Serpinf2	-0.0285549	0.99527876
Serping1	-0.387691087	0.868797028
Serpinh1	-0.183788468	0.868415875
Serpini1	-0.105249403	0.813499809
Sertad1	0.056021499	0.980226283
Sertad2	-0.074471154	0.910328122
Sertad3	0.039644243	0.989840188
Olfr648	-1.644997218	0.91402915
Sertm1	0.265303653	0.911631946
Sesn1	0.164934529	0.514625489
Sesn2	0.091744612	0.890704638
Sesn3	-0.1171304	0.631708623
Sestd1	-0.003728701	0.99527876
Set	0.119761236	0.544721388
Setbp1	0.040146881	0.965520236
Setd1a	-0.044449851	0.962648047
Setd1b	-0.129115096	0.673443553
Mast1	-0.198012725	0.422728382
Cacna1a	-0.197808098	0.260708948
Setd4	0.064924195	0.968005385
Setd5	-0.110120999	0.737369978
B3gat3	0.197297674	0.151118156
Hspa2	0.197097285	0.31865776
Setdb1	-0.129013766	0.538077796
Setdb2	-0.055705921	0.97487264
Setmar	0.140471276	0.905585111

Setx	0.022542424	0.970673196
Dio2	0.196943701	0.402477518
Sez6l	0.187231073	0.79699812
Sez6l2	-0.006676135	0.993290512
Sf1	0.094347288	0.886647768
Sf3a1	-0.040138659	0.957849305
Sf3a2	-0.084159158	0.886647768
Tada3	0.196889197	0.492950206
Sf3b1	-0.012027532	0.985947768
Sf3b2	-0.012913369	0.984748462
Sf3b3	0.030591985	0.962648047
Sf3b4	-0.076913937	0.869700647
Sf3b5	-0.091564491	0.920452533
Sf3b6	0.141363316	0.76998058
Sfi1	-0.114191736	0.884259444
Sfmbt1	-0.066514015	0.993489613
Sfmbt2	0.092487996	0.975831674
Sfn	0.043863172	0.991438757
Sfpq	0.020227786	0.97607383
Sfr1	0.010517787	0.989640643
Sfrp1	-0.119777395	0.966010627
Sfrp2	0.406049994	0.929133542
Sfrp4	0.23570336	0.922100633
Slc13a4	-1.648273043	0.778848805
Sfswap	0.032618968	0.965520236
Sft2d1	-0.037175486	0.980339705
Sft2d2	-0.06642036	0.933199302
Sft2d3	0.188768333	0.858549868
Ch25h	-1.648351664	0.520182905
Sftpc	0.307780973	0.953505913
Sfxn1	0.060515886	0.895520088
Sfxn2	0.034144413	0.980226283
Sfxn3	-0.070890867	0.89703631
Sfxn4	-0.058317198	0.968630572
Sfxn5	0.0587551	0.911631946
Sgca	-0.021527545	0.997115148
Sgcb	0.030830576	0.962648047
Sgcd	-0.892698007	0.52302406
Sgce	-0.034158156	0.972387414
Olfr658	-1.650373822	0.932673884
Sgcz	-0.301628157	0.962648047
Rpl14-ps1	0.196862951	0.435075615
Mrpl42	0.196506507	0.309076191
Sgk1	0.223319438	0.796563869

Sgk2	-0.085506216	0.981045362
Rps13-ps2	0.196318783	0.442302609
Sgms1	-0.145810597	0.732729469
Sgms2	-0.451384281	0.595666235
Sgo1	0.843113274	0.940431936
Sgo2a	0.865125134	0.874252042
Sgpl1	-0.123172881	0.69189201
Sgpp1	-0.02775838	0.974823941
Sgpp2	0.296680487	0.824368034
Sgsh	-0.007138876	0.995632078
Sgsm1	-0.338674435	0.659732239
Sgsm2	0.225476935	0.770793864
Sgsm3	0.030798389	0.975800973
Sgta	0.080385944	0.868415875
Fis1	0.195965604	0.065346116
Sh2b1	-0.05069015	0.957499962
Selenos	0.195960137	0.459813984
Sh2b3	-0.083085414	0.966010627
Sh2d1a	0.420035984	0.982437522
Sh2d1b1	0.491268122	0.985673938
Sh2d3c	-0.007813733	0.99527876
n-R5s197	-1.654220774	0.901129754
Sh2d4b	-0.221400617	0.972218452
Art4	-1.661064812	0.939904081
Sh2d6	-0.311483661	0.962648047
Sh2d7	-0.701280448	0.968005385
Ago2	-0.195822836	0.386030517
Sh3bgrl	-0.085793556	0.887625873
Sh3bgrl2	-0.115970614	0.924361537
Sh3bgrl3	0.045416159	0.939052665
Sh3bp1	-0.050332399	0.985742667
Sidt2	-0.195765306	0.216747565
Gnao1	-0.195457366	0.360222394
Sh3bp5	0.100368045	0.81656551
Sh3bp5l	0.259854461	0.758595939
Sh3d19	-0.059987806	0.949607377
Sh3d21	-0.083943462	0.985742667
Sh3gl1	0.094764938	0.890553353
Sh3gl2	-0.043729803	0.953505913
Sh3gl3	0.045262584	0.965520236
Cyp4x1	-0.195196287	0.475189371
Sh3glb2	-0.004745835	0.99527876
Sh3kbp1	0.220822645	0.865884071
Sh3pxd2a	-0.024477772	0.97487264

Sh3pxd2b	-0.001311402	0.998010523
Sh3rf1	-0.095651828	0.895520088
Sh3rf2	-0.256396258	0.99100709
Sh3rf3	0.104633717	0.900436267
Sh3tc1	0.473423828	0.770166577
Sh3tc2	-0.304615025	0.814301191
Sh3yl1	-0.049612334	0.975800973
Tmem121b	-0.195118214	0.477528928
Gtf2f2	0.195093344	0.490781464
Usmg5	0.19480103	0.431168668
Sharpin	-0.046087111	0.962648047
Shb	-0.007793884	0.995411429
Shc1	-0.037208122	0.985742667
Shc2	-0.026610704	0.973100313
Shc3	-0.310192955	0.522639613
Shc4	-0.02988034	0.985742667
Shcbp1	0.592228455	0.84293526
Shcbp1l	-0.060803745	0.983479691
Shd	0.003307902	0.997529192
She	-0.169851781	0.8989475
Shf	0.077185055	0.945663104
Shh	0.271081175	0.916370249
Shisa2	0.035675781	0.985742667
Rpl13-ps1	-1.666114935	0.86783605
Nop10	0.194777857	0.509250303
Shisa5	0.115988117	0.819778986
Nutf2-ps1	0.194589239	0.425470982
Cbs	0.19458702	0.410106728
Shisa8	-0.126400302	0.985293518
Shisa9	0.198876097	0.627261794
Shisal1	-0.062182462	0.962648047
Frk	-1.667302995	0.869316117
Shkbp1	-0.228468801	0.707777756
Shld1	-0.040935983	0.98187819
Shld2	-0.136780374	0.936031628
Shmt1	0.160862006	0.91916847
Shmt2	0.011667472	0.992385525
Shoc2	-0.106239092	0.860521845
Shpk	0.060160715	0.972218452
Shprh	-0.052776937	0.964941885
Shq1	-0.007670076	0.99640101
Shroom1	-0.042935487	0.98459388
Shroom2	0.641533735	0.921513641
Shroom3	-0.327260874	0.871869598

Shroom4	-0.166624923	0.921473132
Shtn1	0.060339816	0.936031628
Siae	0.033288612	0.975350978
Siah1a	-0.025504106	0.976978183
Siah1b	-0.073303697	0.974962198
Siah2	-0.153321654	0.855904169
Siah3	-0.077665144	0.936246519
Sidt1	-0.024366463	0.984328758
Rps23	0.194582023	0.079793019
Sigirr	-0.418026755	0.904696184
Siglec1	-0.510321154	0.98526072
Siglece	0.309255145	0.947033215
Siglecf	0.201868679	0.965547219
Siglecg	0.923519967	0.948432875
Siglech	0.426734259	0.647235427
Sigmar1	-0.090663494	0.911631946
Sik1	-0.283917171	0.665532517
Sik2	-0.312266927	0.630888733
Sik3	-0.089714215	0.819726438
Sike1	-0.156189963	0.544361994
Sil1	-0.004665924	0.99527876
Sim2	-0.242242835	0.977894594
Simc1	0.139264663	0.809072044
Cops5	0.194494835	0.105302431
Sin3b	-0.000308813	0.999242708
Sinhcaf	-0.064727757	0.98771048
Sipa1	-0.045106618	0.968005385
Ndufa7	0.194384435	0.488233881
Mal2	-0.194319682	0.409844299
Sipa1l3	-0.105325516	0.731646766
Sirpa	0.023230581	0.99527876
Sirt1	-0.034626885	0.975831674
Sirt2	0.087869297	0.88821228
Gse1	-0.194096148	0.444823369
Sirt4	-0.030677252	0.987185029
Sirt5	-0.095844871	0.936834221
Sirt6	0.183125219	0.595417602
Sirt7	0.270404763	0.573810203
Usp46os1	-1.668507822	0.948104382
Siva1	0.127785131	0.932673884
Rln3	-1.675634292	0.939052665
Irx6	-1.676954394	0.793949146
Six3	-0.359643249	0.985673938
Six3os1	-0.685903693	0.962648047

Six4	0.2186811	0.965520236
Spred2	-0.193957867	0.132398025
Ska1	0.630139732	0.975800973
Ska2	-0.19098048	0.729712198
Rtn4r	-0.193929582	0.399438388
Skap1	0.069404994	0.992669879
Skap2	0.040288822	0.974962198
Ski	-0.486693836	0.905559486
Skida1	0.079877184	0.955026616
Skil	-0.12182066	0.922889447
Skiv2l	0.059116624	0.90358675
Skor1	0.152170708	0.987665265
Skp1a	0.054273365	0.874252042
Skp2	-0.090843193	0.950604431
Sla	0.202506619	0.911276003
Sla2	0.200777416	0.97041548
Slain1	-0.040121489	0.972218452
Slain1os	-0.073013902	0.989640643
Slain2	-0.152993697	0.721943953
Gpat2	-1.678181659	0.852010446
Slamf6	-0.368093738	0.962648047
Slamf8	-0.043564293	0.997258205
Slamf9	0.648379971	0.865547585
Slbp	0.122949015	0.920642026
Slc10a1	0.796810242	0.956164302
Slc10a3	0.663605182	0.648241111
Slc10a4	0.864794862	0.770793864
Slc10a5	0.448123708	0.969755848
Slc10a7	-0.068795236	0.984328758
Rpl29	0.193720855	0.183613297
Slc11a2	0.001223251	0.998010523
Hsd3b6	-1.680180693	0.821998633
Slc12a2	-0.070099253	0.911631946
Slc12a3	0.092170667	0.992669879
Slc12a4	0.113632194	0.870485449
Slc12a5	-0.129391287	0.56399886
Slc12a6	-0.025557555	0.969841793
Slc12a7	-0.301873769	0.909109599
Slc12a8	0.14706747	0.973100313
Slc12a9	-0.004219115	0.995411429
Slc13a1	0.909839645	0.936031628
Psmc5	0.19345944	0.146214787
Cxcl10	-1.680997706	0.740925526
Slc13a5	-0.025744797	0.991438757

Slc14a1	-0.187943493	0.566310402
Slc14a2	0.283807657	0.959659722
Slc15a1	0.338957608	0.987066554
Slc15a2	0.123551903	0.754350047
Slc15a3	-0.523006158	0.655744783
Slc15a4	-0.045896733	0.972218452
Insrr	-1.681710743	0.926051604
Ripor2	-0.19337247	0.234819833
Slc16a10	0.303332611	0.52302406
Slc16a11	0.260251143	0.656577995
Slc16a12	-0.339680054	0.891972665
Slc16a13	0.123372304	0.928957615
Slc16a14	-0.077227639	0.962648047
Slc16a2	-0.13831644	0.5141747
Tarsl2	-0.193113207	0.194013941
Slc16a4	0.011570684	0.995411429
Slc16a5	0.128423642	0.980226283
Slc16a6	-0.213603158	0.617743758
Slc16a7	-0.546527605	0.659732239
Slc16a8	0.347283255	0.948104382
Slc16a9	0.226987265	0.928957615
Slc17a2	0.095120407	0.99527876
Slc17a5	-0.133708871	0.975831674
Slc17a6	0.376991797	0.89703631
Oaz2	0.192897876	0.120903042
Slc17a8	-0.110505252	0.933285475
Slc17a9	0.353958494	0.953505913
Muc2	-1.682019722	0.936905058
Slc18a2	0.017083189	0.994960308
Slc18a3	-0.533495316	0.981045362
Slc18b1	0.069955928	0.974823941
Slc19a1	-0.098225087	0.948104382
Slc19a2	0.397111264	0.933201883
Slc19a3	0.432147289	0.749780051
Slc1a1	-0.068791612	0.815987743
Slc1a2	-0.04966288	0.962648047
Slc1a3	0.008085438	0.988034453
Slc1a4	-0.045923549	0.962648047
Slc1a5	-0.010322131	0.997115148
Slc1a6	0.030385796	0.984328758
Slc20a1	-0.137354318	0.626162306
Slc20a2	0.088386266	0.8549794
Slc22a12	0.365949819	0.962648047
Slc22a13b-ps	0.40372837	0.942781802

Slc22a15	0.014914994	0.993839396
Slc22a17	-0.031351968	0.968005385
Slc36a2	-1.68315152	0.932673884
Pcdhgb4	-1.686630512	NA
Slc22a21	-0.05431223	0.985293518
Slc22a23	-0.027168071	0.974374227
Slc22a3	-0.265486194	0.966213117
Slc22a4	-0.011270343	0.99527876
Slc22a5	-0.007476842	0.99527876
Pla2g4c	-1.688715996	0.654893207
Slc22a7	-0.545265994	0.971661054
Slc22a8	-0.426510527	0.662600961
Slc23a1	-0.255763127	0.969755848
Slc23a2	-0.071469501	0.855344864
Slc23a3	-0.304739075	0.984328758
Slc23a4	0.016460428	0.996920836
Slc24a1	-0.181622022	0.994960308
Slc43a2	-0.192557534	0.465255523
Slc24a3	-0.108760107	0.624713015
Slc24a4	0.012684148	0.99100709
Slc24a5	0.568436832	0.696211886
Slc25a1	0.107437509	0.895520088
Slc25a10	-0.144564012	0.891972665
Slc25a11	0.007077398	0.99527876
Slc25a12	-0.005669917	0.994960308
Slc25a13	0.416297153	0.858549868
Slc25a14	0.051956466	0.955993235
Slc25a15	0.042297346	0.97487264
Slc25a16	0.00521126	0.99527876
Slc25a17	0.050511318	0.938414123
Slc25a18	0.016333096	0.985293518
Slc25a19	0.121757924	0.809102338
Slc25a2	-0.969359091	0.930571344
Rpl23	0.192508007	0.09657124
Slc25a21	0.671054496	0.959276014
Slc25a22	-0.005909812	0.99527876
Slc25a23	-0.021025603	0.969755848
Slc25a24	-0.17338585	0.962648047
Slc25a25	-0.086573539	0.968005385
Slc25a26	-0.303545232	0.868415875
Slc25a27	0.053324549	0.923340746
Slc25a28	0.097999863	0.837755777
Slc25a29	0.011968314	0.995072436
Slc25a3	0.06300486	0.876359276

Slc25a30	0.043965675	0.975800973
Slc25a32	-0.166838165	0.639505683
Slc25a33	0.135171949	0.777427974
Slc25a34	0.094281646	0.95520652
Commd9	0.192445053	0.504583609
Slc25a36	-0.089721953	0.863674591
Slc25a37	-0.001261643	0.998010523
Slc25a38	-0.127161588	0.862838439
Tpd52l1	0.192258707	0.422728382
Slc25a4	0.069297265	0.822597041
Slc25a40	0.079782488	0.961373288
AC140462.1	-1.692215963	NA
Slc25a42	-0.306270897	0.539496054
Slc25a43	0.638214227	0.975800973
Slc25a44	-0.056869563	0.942018841
Slc25a45	0.191074855	0.90358675
Slc25a46	-0.028489316	0.965658786
Slc25a47	-0.34042401	0.891972665
Serpine3	-1.694717747	0.911416083
Slc25a5	0.009456076	0.985742667
Slc25a5-ps	-0.364528945	0.980896205
Slc25a51	0.293523288	0.895520088
Slc25a53	0.204127993	0.69189201
Slc26a1	0.206268414	0.938615214
Slc26a10	0.211000839	0.670918119
Slc26a11	-0.230138332	0.815987743
Slc26a2	0.058252527	0.980896205
Slc26a3	0.014575671	0.99765039
Slc26a4	-0.0564844	0.959659722
Slc26a6	0.101696702	0.962648047
Slc26a7	-0.383066241	0.968005385
Slc26a8	0.178597652	0.814301191
Slc26a9	0.094712518	0.99527876
Slc27a1	0.017312911	0.985673938
Slc27a2	0.176718952	0.820614009
Ncald	0.19218092	0.202632501
Slc27a4	-0.082569494	0.841867328
Slc27a5	-0.296871478	0.974962198
Slc27a6	0.823978872	0.948104382
Haglr	-1.701470331	0.895520088
Tmem156	-1.70365185	0.757721895
Olfr461	-1.714256911	0.873797321
Slc29a1	0.227440868	0.658829956
Slc29a2	-0.151722372	0.89703631

Slc29a3	-0.132722202	0.932673884
Slc29a4	0.155153317	0.80818949
Gaa	-0.192159032	0.266785634
Slc2a10	-0.392728569	0.785482415
Slc2a12	-0.3282991	0.849517436
Slc2a13	-0.072285421	0.882096017
Slc2a2	-0.73212431	0.981392364
Slc2a3	0.091975207	0.74463793
Psmd9	0.191808984	0.436138179
Slc2a4rg-ps	0.001901797	0.998010523
Slc2a5	0.027877547	0.989840188
Slc2a6	-0.155835267	0.695239733
Slc2a8	-0.223640797	0.690084266
Slc2a9	-0.10624549	0.940504226
Slc30a1	0.095003646	0.874252042
Slc30a10	-0.171927399	0.590745439
Slc30a2	-0.181594556	0.89703631
Slc30a3	0.084055145	0.890704638
Slc30a4	-0.085526934	0.858549868
Slc30a5	-0.024912783	0.989200732
Slc30a6	0.220607443	0.654893207
Slc30a7	0.059199023	0.968005385
Slc30a9	-0.041819098	0.936031628
Slc31a1	0.095583431	0.853090538
Slc31a2	-0.195731278	0.795230309
Slc32a1	0.121228631	0.798580668
Slc33a1	-0.21941496	0.873797321
Slc34a1	-0.799410757	0.966213117
Ccdc154	-1.736790162	NA
Slc35a1	-0.105962996	0.894953269
Slc35a2	0.163016655	0.91462326
Slc35a3	0.041612776	0.971661054
Slc35a4	0.051082252	0.962648047
Myl6	0.191779175	0.041498062
Slc35b1	0.00060526	0.998658264
Slc35b2	0.042313057	0.970461999
Slc35b3	0.096856788	0.911631946
Slc35b4	-0.012499689	0.985446046
Slc35c1	0.024317818	0.985742667
Slc35c2	-0.058269912	0.948104382
Slc35d1	-0.178652849	0.770166577
Slc35d2	-0.014971806	0.997258205
Slc35d3	0.169210105	0.973100313
Slc35e1	-0.060077401	0.903795193

Slc35e2	-0.187509952	0.98459388
Slc35e3	-0.022309591	0.979711552
Slc35e4	0.056122824	0.961859633
Slc35f1	-0.104849991	0.881371608
Slc35f2	0.113254214	0.962648047
Slc35f3	0.075654793	0.905281292
Slc35f4	-0.096523935	0.909547396
Slc35f5	0.082172865	0.875723186
Slc35f6	0.154226719	0.959276014
Slc35g1	0.099866873	0.962648047
Slc35g2	-0.112855116	0.902647137
Slc35g3	-0.582472672	0.936031628
Slc36a1	-0.165309942	0.985923036
Ube4bos1	-1.739485504	0.724910334
Slc36a4	0.062041767	0.878119351
Slc37a1	-0.026062877	0.991120895
Slc37a2	-0.159491657	0.950460219
Slc37a3	0.070348943	0.949607377
Slc37a4	0.036132406	0.980226283
Slc38a1	-0.078218788	0.837755777
Slc38a10	-0.036195135	0.962648047
Chchd1	0.191656076	0.434676934
Slc38a2	-0.077964571	0.889547181
Slc38a3	0.030382592	0.975831674
Slc38a4	0.212773515	0.962648047
Slc38a5	-0.292274222	0.89703631
Slc38a6	-0.154805951	0.858549868
Slc38a7	-0.025097586	0.976548735
Slc38a9	-0.082298976	0.921473132
Slc39a1	-0.071436298	0.89703631
Slc39a1-ps	0.273247753	0.96765937
Psmc3	0.191536272	0.197055082
Slc39a11	0.153850749	0.80819125
Slc39a12	-0.040891524	0.950195611
Slc39a13	-0.017970066	0.991996237
Slc39a14	-0.09423936	0.874971335
Slc39a2	-0.108626731	0.928672524
Slc39a3	-0.619278296	0.853090538
Slc39a4	-0.765509296	0.695239733
Nr6a1os	-1.752313628	0.935897306
Slc39a6	0.118763966	0.849517436
Slc39a7	-0.176225795	0.56399886
Slc39a8	-0.274799884	0.873797321
Slc39a9	0.391617613	0.95363833

Slc3a1	-0.953449572	0.817988462
Slc3a2	-0.040065514	0.958389182
Rims1	-0.191506039	0.388066232
Slc41a1	-0.035155575	0.951497773
Slc41a2	-0.125179942	0.874971335
Slc41a3	0.1850431	0.90918338
Slc43a1	0.391224766	0.975831674
Tmem63c	-0.191476835	0.436138179
Slc43a3	-0.021423849	0.99527876
Slc44a1	0.0639518	0.953505913
Slc44a2	0.035994272	0.978063275
Slc44a3	-0.405010732	0.969755848
Slc44a4	-0.043791756	0.99527876
Slc44a5	-0.220875096	0.804540244
Slc45a1	-0.077966319	0.875723186
Slc45a2	-0.171210878	0.975831674
Slc45a3	-0.15271126	0.98187819
Slc45a4	-0.074378265	0.939052665
Slc46a1	-0.09250756	0.920642026
Slc46a3	-0.10651748	0.969755848
Slc47a1	-0.567117094	0.915297582
Slc48a1	-0.086197992	0.985742667
Slc4a1	-0.018079343	0.99765039
Slc4a10	0.143043346	0.852946549
Slc4a11	-0.114344471	0.985673938
Slc4a1ap	-0.07030141	0.932673884
Slc4a2	0.022857775	0.984748462
Slc4a3	0.053359808	0.949607377
Slc4a4	-0.070009452	0.873797321
AC139750.1	-1.75882253	0.932673884
Slc4a7	-0.10581178	0.852010446
Nkx6-1	-1.761501482	0.930250446
Slc50a1	0.138293303	0.852010446
Slc51a	-0.903912258	0.940317783
Slc52a2	-0.055529988	0.971750542
Lrrn3	-0.191401344	0.449795449
Slc5a1	-0.047755298	0.993103295
Slc5a10	-0.874186235	0.924707867
Slc5a11	-0.890718991	0.837755777
Slc5a2	-0.194594159	0.992385525
Golgb1	-0.191315945	0.064044233
Slc5a5	0.087640032	0.89703631
Slc5a6	0.102197045	0.905361361
Slc5a7	-0.060183083	0.993380259

Slc5a8	-0.466301325	0.981392364
Slc6a1	0.048924693	0.942274311
Slc6a11	-0.086589794	0.795563654
Gchfr	-1.763813808	0.665532517
Slc6a13	-0.797101567	0.89383516
Slc6a15	0.118769573	0.571020232
Slc6a16	0.069337146	0.991438757
Slc6a17	0.664727783	0.544361994
Slc6a19	0.265655469	0.968005385
Slc6a20a	-0.966400882	0.786324313
Pde6c	-1.77329787	0.911631946
Slc6a3	0.847520448	0.921311891
Veph1	-1.775235962	0.871869598
Eef1a1	0.191308135	0.01931564
Slc6a7	0.139651628	0.745879732
Slc6a8	-0.239523988	0.945663104
Slc6a9	-0.023048107	0.984836179
Slc7a1	-0.159248898	0.711101122
Slc7a10	-0.067314052	0.948432875
Ndufv2	0.19104997	0.37706439
Slc7a14	-0.046625018	0.928957615
Slc7a2	-0.583188798	0.920642026
Slc7a3	0.107757365	0.97469529
Slc7a4	0.009877201	0.997529192
Slc7a5	0.138431761	0.6883862
Slc7a6	0.066947337	0.932673884
Slc7a6os	0.158418534	0.623438925
Slc7a7	0.332432939	0.869316117
Slc7a8	-0.075044116	0.841017002
Rnf111	-0.190613642	0.431061843
Slc8a2	0.080470118	0.819726438
Slc8a3	-0.191663978	0.654936641
Sall2	-0.190521449	0.157852526
Slc9a1	-0.105016844	0.849517436
Ndufb10	0.190513849	0.215455321
Slc9a3	-0.117940904	0.973168955
Slc9a3r1	0.058380631	0.91462326
Slc9a3r2	-0.30755443	0.810455125
Slc9a4	-0.070935123	0.910328122
Slc9a5	0.119453539	0.89703631
Slc9a6	-0.068245782	0.935897306
Slc9a7	-0.243246774	0.774896252
Slc9a8	0.017934269	0.98526072
Slc9a9	0.025313259	0.98526072

Slc9b1	-0.413283117	0.971138085
Slc9b2	0.004044918	0.998010523
Slco1a4	-0.181967529	0.670918119
Peg12	-1.77766262	0.770019887
Slco1b2	-0.469317472	0.97487264
Slco1c1	-0.166144913	0.6883862
Cnksr2	-0.190490837	0.403453477
Slco2b1	-0.060513214	0.965520236
Slco3a1	-0.068083611	0.899930239
Slco4a1	-0.209522484	0.962648047
Slco4c1	-0.240472683	0.90125879
Slco5a1	-0.473137817	0.623438925
Slf1	-0.182248593	0.776477378
Slf2	-0.088112877	0.852035098
Esr2	-1.777755448	0.895520088
Slfn2	0.021281006	0.99527876
Slfn3	-0.365298828	0.981502501
D10Jhu81e	0.190464246	0.327922951
Slfn5	-0.224071636	0.8549794
Slfn8	-0.112008665	0.985673938
Tlr5	-1.780734975	0.615496563
Desi2	-0.190360135	0.31865776
Slit1	-0.025680216	0.962648047
Slit2	-0.140981236	0.739332889
Rapgef5	-0.190332357	0.442302609
Slitrk1	-0.066798699	0.888921078
Slitrk2	-0.076276606	0.940963923
Slitrk3	-0.037980266	0.934798735
Slitrk4	-0.165148656	0.797361951
Slitrk5	-0.023268636	0.980339705
Slitrk6	0.172848104	0.968005385
Slk	-0.028282916	0.971138085
Slmap	-0.043479795	0.928716457
Sln	0.75482848	0.781249046
Slpi	0.759226691	0.760547704
Sltm	0.000133909	0.999635996
Slu7	0.060743803	0.89703631
Slx1b	0.137031239	0.889972515
Slx4	-0.233332319	0.786687146
Slx4ip	-0.387602142	0.740833211
Smad1	-0.006544395	0.99527876
Smad2	-0.086504707	0.942812446
Smad3	0.097630116	0.850171696
Fzd3	-0.190278841	0.189900407

Smad5	0.575757019	0.876359276
Aqp5	-1.782065457	0.928489442
Tubg2	0.189921341	0.4648833
Smad9	0.042219806	0.968005385
Smagp	-0.458653034	0.868415875
Smap1	0.150721157	0.517996718
Smap2	0.006587919	0.992040075
Smarca1	-0.118524038	0.9089345
H3f3b	0.18958013	0.299535449
Smarca4	-0.090976847	0.69189201
Smarca5	-0.107401978	0.728087796
Smarca5-ps	0.872900482	0.650504271
Smarcad1	-0.016349209	0.988034453
Smarcal1	-0.123866772	0.736130784
Smarcb1	0.092629906	0.792641681
Smarcc1	-0.081777789	0.825148733
Smarcc2	0.003095197	0.99527876
Smarcd1	0.025178824	0.969755848
Smarcd2	0.003010722	0.997258205
Smarcd3	-0.043787728	0.955706294
Smarce1	0.05480427	0.91462326
Smarce1-ps1	0.0395085	0.985742667
Smc1a	0.015607879	0.985673938
Smc1b	-0.079568072	0.99527876
Smc2	-0.142118418	0.899097089
Smc2os	0.026457776	0.995411429
Utp20	-0.189395047	0.31865776
Smc4	0.15679435	0.911631946
Smc5	-0.12490558	0.853776382
Smc6	0.016464452	0.991438757
Smchd1	-0.029631901	0.980626239
Smco1	0.687995228	0.962648047
Smco3	0.759060409	0.623438925
Smco4	0.32789276	0.920642026
Smcr8	-0.017722817	0.981392364
Smdt1	-0.041647988	0.97607383
Map3k11	-0.188784762	0.442302609
Smg5	0.006514288	0.994960308
Smg6	0.014085326	0.99100709
Smg7	-0.03328185	0.960201673
Smg8	-0.011558023	0.991996237
Smg9	0.138346193	0.6883862
Smim1	-0.293330933	0.895198797
Smim10l1	0.175974916	0.886647768

Smim10l2a	0.082841231	0.869316117
Smim11	0.07850438	0.965520236
Smim12	0.036619248	0.973100313
Smim13	-0.041573429	0.949450803
Smim14	0.116038824	0.894953269
Rpl37a	0.188753732	0.422728382
Smim17	-0.149441658	0.777427974
Smim18	0.059857998	0.973100313
Smim19	0.170812744	0.593166543
Smim20	-0.265988178	0.871869598
Zmym2	-0.18848977	0.482234498
Smim24	0.657022361	0.6883862
Smim26	0.234680237	0.594746202
Smim27	0.158560395	0.968005385
Magi1	-0.188411315	0.422728382
Smim4	0.179857161	0.95227747
Smim5	0.476743369	0.903795193
Smim6	-0.831770511	0.950460219
Smim7	0.030724562	0.962094124
Smim8	-0.296029945	0.673443553
Smkr-ps	0.690335119	0.911631946
Mir7017	-1.794585974	NA
Smn1	0.117102943	0.898478018
Smndc1	0.027551673	0.974823941
Smo	0.016909966	0.991996237
Smoc1	-0.139476665	0.806077586
Smoc2	0.130644076	0.860521845
Il31ra	-1.794624408	0.936031628
Smox	0.189917261	0.793949146
Smpd1	-0.004737437	0.99527876
Smpd2	0.108055042	0.939592821
Smpd3	-0.13716513	0.638329646
Smpd4	-0.104616635	0.825910982
Smpd5	0.271836342	0.975350978
Smpdl3a	0.02787644	0.981392364
Smpdl3b	0.008204989	0.997258205
Olfr564	-1.797092185	0.894953269
Sms	0.030316315	0.965520236
Sms-ps	0.01291632	0.988034453
Smt3h2-ps4	0.155354544	0.889972515
Smtn	0.220574935	0.861366392
Smtnl2	0.125984524	0.962648047
Smu1	0.113694472	0.771450635
Smug1	-0.238646927	0.53452841

Smurf1	-0.053171481	0.939592821
Mn1	-0.188291825	0.508471705
Smyd1	0.594253095	0.665532517
Smyd2	0.028849747	0.97607383
Smyd3	0.281998735	0.615948828
Smyd4	0.149688385	0.873753967
Smyd5	0.188587957	0.898478018
Snai1	-0.107917192	0.993290512
Snai2	-0.040800506	0.99527876
Snap23	0.071847213	0.962648047
Snap25	0.009740991	0.985742667
Snap29	-0.057211468	0.919785445
Snap47	0.012532224	0.985673938
Snap91	0.006253682	0.99527876
Snapc1	-0.100565124	0.835714527
Snapc2	0.072894546	0.963666931
Snapc3	-0.106267398	0.87847909
Snapc4	0.036879376	0.975831674
D2hgdh	-0.188271516	0.504583609
Snapin	0.310571938	0.595105754
Limd2	0.188215662	0.468229908
Sncaip	0.034445852	0.989840188
Sncb	0.116102452	0.660403254
Sncg	0.148224882	0.960176242
Snd1	-0.063709474	0.938544177
Rilpl1	0.188050686	0.509250303
Snf8	0.084575752	0.839488666
Kcnab1	-0.18800104	0.428038709
Snhg10	0.106772096	0.97487264
Snhg11	-0.079118871	0.738804116
Snhg12	0.258957293	0.69189201
Cpd	-0.187852275	0.128640258
Snhg15	0.188404085	0.951497773
Snhg17	0.124815897	0.87847909
Snhg18	0.125506785	0.970461999
Snhg20	0.288408998	0.84870301
Snhg3	0.235003818	0.871869598
Snhg5	0.239125887	0.740833211
Snhg6	0.12169619	0.936031628
Hsbp1	0.187760271	0.435075615
Snhg9	-0.147808838	0.991438757
Snip1	0.043705567	0.968005385
Snn	0.014243982	0.982914099
Snora15	-0.46300884	0.975831674

Abhd12b	-1.801084756	0.857344087
Snora17	0.671040663	0.909243078
Mab21l2	-1.802384917	0.642397758
Snora20	0.132318872	0.991996237
Snora21	0.585823108	0.974823941
Snora23	0.356746511	0.968005385
Snora24	-0.025669862	0.996094752
Snora26	0.08251524	NA
Snora28	0.08251524	NA
Snora2b	-0.538230841	0.968005385
Snora3	0.08251524	NA
Snora30	-0.43145113	0.97469529
Snora31	0.41473962	0.921311891
Snora33	-0.686157344	0.957485565
Snora34	-0.014782901	0.997375151
Snora41	-0.205032729	0.980226283
Snora43	-0.377677582	0.970348278
Snora44	-0.230628215	0.970348278
Snora47	-0.585713883	0.974823941
Snora52	-0.46277017	0.875119971
Snora57	0.659692204	0.814276256
Snora5c	-0.659950818	NA
Snora61	0.878067035	0.948104382
Snora62	0.426918682	0.973100313
Snora64	0.08251524	NA
Snora65	0.510932692	NA
Snora68	0.559800039	0.920642026
Snora69	-0.193129814	0.991438757
Snora70	0.637284521	0.942812446
Mgst2	-1.81180506	0.86783605
Espnl	-1.81224523	0.861378744
Mir1231	-1.817592926	NA
Snora75	0.08251524	NA
Snora78	0.47650247	0.965520236
Snora7a	0.273679959	0.91462326
Snora81	0.08251524	NA
Snora9	0.827245301	NA
Snorc	-0.977583402	0.970398378
Mir6973a	-1.817599387	NA
Snord104	0.245069768	0.956164302
Snord107	0.08251524	NA
Snord110	-0.512975781	0.980896205
Snord12	-0.213962361	0.97469529
Snord14a	0.08251524	NA

Snord14c	-0.214295629	0.990079957
Bicd1	-0.187547297	0.434676934
Hist1h2ah	-1.82933422	NA
Snord15b	-0.496562034	0.962648047
Snord16a	-0.903821583	NA
Klk1b24	-1.839010845	0.920642026
Snord19	0.08251524	NA
Snord1a	0.08251524	NA
Cd300ld3	-1.842021837	0.806485043
Snord1c	0.08251524	NA
Snord2	0.08251524	NA
Snord21	0.08251524	NA
Snord22	0.08251524	NA
Bcl3	-1.852144149	0.771684575
Snord32a	0.332701696	0.965520236
Snord33	0.08251524	NA
Snord34	0.410939147	0.965520236
Snord35a	0.181055502	0.985742667
Snord35b	-0.793079247	0.89703631
Snord37	0.12959792	0.988159112
Snord38a	0.988112711	NA
Snord42b	0.627204556	0.89703631
Snord43	-0.919884566	NA
Snord45b	0.08251524	NA
Snord45c	0.08251524	NA
Snord47	0.08251524	NA
Snord49a	0.574171804	0.911631946
Snord49b	0.719000665	0.948104382
Wfdc17	-1.854819757	0.869316117
Snord50b	0.062907007	0.99640101
Snord52	0.08251524	NA
Snord53	-0.459307394	0.965434039
Snord55	-0.067215391	0.99527876
Vmn1r84	-1.855992163	0.942812446
Snord58b	-0.279070929	0.975631569
Snord59a	-0.191352023	0.985742667
Hepacam	0.187508353	0.365013954
Snord64	0.08251524	NA
Snord65	0.185470167	0.985742667
Snord66	0.8734892	0.972376117
Snord68	0.621156184	0.963317548
Snord69	0.08251524	NA
Snord7	0.167050389	0.985742667
Snord71	0.815233242	0.935188133

Snord72	0.591307722	0.962648047
Abcb11	-1.863029705	0.916962532
Snord78	0.08251524	NA
Snord8	0.819536319	NA
Snord80	-0.441016211	0.927934062
Snord83b	0.397330571	0.968005385
Snord87	0.013654606	0.998010523
Snord88a	0.888452878	0.969755848
Snord88c	-0.185486086	0.98526072
Snord89	-0.304772065	0.968005385
Snord91a	-0.09923345	0.971661054
Snord93	-0.544125141	NA
Snord95	0.08251524	NA
Snord96a	0.08251524	NA
Snord98	0.134804359	NA
Snord99	0.08251524	NA
Snph	-0.070614823	0.964941885
Snrk	-0.132861816	0.914578983
Snrnp200	-0.060693784	0.89011487
Kat6a	-0.187484148	0.410106728
Snrnp27	0.141987176	0.782639526
Ufsp2	0.187335013	0.456330081
Snrnp40	0.113035804	0.889972515
Pank3	-0.18728626	0.264861182
Snrnp70	0.041079933	0.961373288
Snrpa	0.107898689	0.803910915
Snrpa1	0.151072805	0.890704638
Snrpb	0.151139475	0.813744313
Snrpb2	0.221224738	0.534684113
Snrpc	0.20535767	0.558852325
Prepl	-0.187149666	0.396190711
Cers5	0.187133165	0.349775121
Snrpd3	0.127442404	0.740605207
Snrpe	0.194094448	0.65012378
Snrpert	0.419977661	0.932673884
Mier2	-0.186713322	0.434762828
Snrpg	0.165595053	0.668010503
Snrpn	0.10662048	0.557507639
Snta1	0.168493832	0.724910334
Sntb1	0.10161976	0.965520236
Sntb2	-0.051913407	0.969653237
Sntg1	-0.284467847	0.54807923
Sntg2	0.160094393	0.958389182
Sntn	-0.89406804	0.519110875

Snu13	0.199490983	0.563984287
Snupn	-0.005254481	0.99527876
Snurf	0.082597848	0.99527876
Snw1	0.003554665	0.99527876
Snx1	0.115331649	0.52053053
Snx10	-0.348169634	0.889908105
Snx11	0.026437233	0.975831674
Snx12	0.039098369	0.962648047
Ncor2	-0.186522569	0.252498589
Snx14	-0.121888619	0.702951568
Snx15	-0.055511607	0.951390592
Snx16	-0.043070128	0.970348278
Snx17	0.038312469	0.962648047
Snx18	-0.09544693	0.868415875
Snx19	-0.124637349	0.557791921
Snx2	0.004720188	0.99527876
Snx20	0.170464911	0.966010627
Spred3	-0.185982814	0.421691269
Ccdc93	-0.185930972	0.369497288
Snx24	0.171680867	0.738005162
Snx25	-0.004388437	0.99527876
Snx27	-0.081292805	0.74632237
Snx29	0.046233291	0.981392364
Snx3	-0.029945334	0.99527876
Fuca1	0.185860345	0.507697207
Snx31	0.425932545	0.956164302
Snx32	0.046960191	0.952185901
Snx33	-0.071857001	0.965290366
Snx4	0.048043798	0.945183336
Snx5	-0.000730235	0.998840551
Snx6	-0.008945296	0.994960308
Snx7	-0.124919297	0.887625873
Snx8	-0.15485719	0.952034929
Rsl1d1	0.185409438	0.266785634
Soat1	-0.01127657	0.99165967
Sobp	-0.038698145	0.947447204
Fgf17	-1.86338466	0.849424799
Socs2	-0.141412993	0.770793864
Socs3	0.618123808	0.812431087
Socs4	-0.11189922	0.91462326
Socs5	-0.067501614	0.871869598
Socs6	0.080292625	0.965007958
Socs7	-0.029905274	0.962648047
Kctd3	0.185137123	0.421157413

Sod2	0.04926467	0.891265024
Sod3	-0.065244057	0.965520236
Soga1	-0.219334364	0.673349785
Chmp4b	0.184635441	0.064044233
Son	-0.140641432	0.610808738
Sorbs1	-0.112799971	0.80931022
Sorbs2	0.267763509	0.710375942
Sorbs2os	-0.381931645	0.898478018
Sorbs3	0.015261305	0.992669879
Sorcs1	0.062951347	0.981392364
Sorcs2	-0.032679838	0.974823941
Sorcs3	-0.222187063	0.522113371
Sord	0.161508318	0.686130003
Dtx4	-0.184559138	0.456330081
Arhgef17	-0.184514878	0.128640258
Lrrc8c	0.184144232	0.425531968
Alg10b	-0.184117269	0.41351939
Sost	0.250802941	0.99100709
Sostdc1	0.012730763	0.99527876
Sowaha	0.019801498	0.985673938
Sowahb	0.483894119	0.629280869
Sowahc	-0.172612073	0.8549794
Sox1	-0.072233357	0.95126064
Sox10	-0.291076762	0.588224175
Sox11	-0.251762847	0.729405774
Sox12	-0.469638691	0.932673884
Sox13	-0.391825156	0.572363201
Sox17	0.430461818	0.514712196
Sox18	-0.05027688	0.984748462
Sox1ot	-0.150968154	0.655744783
Sox2	-0.224123516	0.56399886
Slc6a6	-0.184052757	0.422770804
Sox2ot	0.581541462	0.637629898
Sox3	0.01740431	0.995632078
Sox4	-0.141828486	0.84277074
Sox5	0.006748585	0.99527876
Sox6	-0.196966002	0.54905206
Sox7	-0.125123654	0.969476616
Sox8	0.029456071	0.984512582
Sox9	-0.330135881	0.563984287
Sp1	-0.041592076	0.971661054
Arl2	0.184047674	0.417721745
Sp110	-0.062697774	0.985742667
Sp140	0.17969893	0.965520236

Sp2	0.084207531	0.921311891
Sp3	-0.081076148	0.938394547
Sp3os	-0.040695239	0.985293518
Sp4	-0.160428798	0.850260513
H2-Q1	-1.869280359	0.782241142
Sp7	-0.380529455	0.914675447
Sp8	0.386768625	0.797072888
Sp9	-0.408753333	0.543640901
Spa17	0.231622626	0.89703631
Spaar	0.151413235	0.97487264
Msx2	-1.897785903	0.605451839
Spaca6	-0.040876725	0.965520236
Spaca9	-0.541654832	0.925217889
Spag1	-0.038447061	0.969755848
Spag16	0.010438749	0.99765039
Spag17	-0.210330134	0.973100313
Spag17os	0.447604174	0.875995577
Spag4	0.136749858	0.968005385
Spag5	0.290583003	0.524885942
Spag6	-0.104753631	0.970461999
Spag6l	-0.285062863	0.562965533
Spag7	0.117942411	0.710375942
Spag8	0.016633109	0.995411429
Spag9	-0.120569984	0.529138606
Sparc	0.037729454	0.948407374
Rpl22	0.183899992	0.124108735
Spast	-0.092002922	0.815987743
Spata1	-0.47426881	0.852010446
Spata13	-0.019553554	0.992152669
Spata17	0.660426999	0.895520088
Spata18	-0.583365269	0.914207631
Spata19	-0.092862404	0.996070372
Spata2	-0.0148957	0.985742667
Spata21	-0.240033978	0.972376117
Spata24	0.284740388	0.737087313
Spata25	0.99149423	0.965290366
Spata2l	-0.04576191	0.968005385
Spata3	-0.052244042	0.994960308
Spata31d1a	-0.059390784	0.99527876
Spata31d1b	0.546037537	0.890704638
Spata31d1d	0.536373109	0.98526072
Spata32	-0.095116428	0.985742667
Spata33	0.334052198	0.821998633
Spata4	0.113056691	0.988034453

Spata45	-0.976100413	0.899097089
Spata46	-0.298813762	0.965520236
AC132100.1	-1.898549448	0.866574046
Spata5	-0.089360757	0.938615214
Spata5l1	0.127241232	0.968005385
Bnip3l	0.183868216	0.146214787
Spata7	-0.064878779	0.962648047
Spata9	-0.209586846	0.951817826
Spatc1	-0.664045862	0.97487264
Spats1	0.342437284	0.664771806
Spats2	-0.089718681	0.816025904
Spats2l	0.023891379	0.991438757
Spc24	0.130800134	0.970348278
Spc25	0.517196321	0.918451137
Spcs1	0.156053712	0.550104301
Spcs2	-0.107564194	0.909604637
Spcs3	0.0031718	0.995500778
Spdef	-0.173762351	0.987342614
Spdl1	0.623130358	0.880133341
Spdya	0.30705156	0.962648047
Rpl17-ps3	0.18376212	0.509250303
Specc1l	-0.069904104	0.966213117
Speer4a	-0.101435132	0.99527876
Speer4b	0.132248441	0.987342614
Spef1	-0.030839524	0.991438757
Spef2	-0.53708484	0.513797506
Speg	-0.114659124	0.84870301
AC115894.1	-1.902399279	0.917018212
Ago3	-0.183744874	0.237504632
Spg11	-0.189085877	0.603499717
Spg20	-0.076875593	0.852275035
Spg21	0.133981143	0.647235427
Spg7	-0.007774563	0.99527876
AC123705.1	-1.905237332	0.648241111
Sphk2	-0.197777036	0.777427974
Sphkap	-0.124862479	0.644868623
Spi1	-0.074995902	0.975350978
Spice1	-0.027664131	0.985673938
Spidr	0.115773159	0.923011738
Spin1	-0.025054984	0.962648047
Spin2c	0.00662806	0.995411429
Spin4	0.183652406	0.98526072
Spindoc	0.051805014	0.949607377
Spink10	-0.273276817	0.938615214

Spink4	0.053901532	0.997241386
Spink8	-0.103493047	0.939904081
Spint1	0.563020838	0.730301028
Ahcyl2	0.183656775	0.243596082
Spire1	0.046170284	0.929095022
Spire2	-0.031227637	0.983219084
Spn	0.316614762	0.971891838
Spns1	-0.146611668	0.736130784
Spns2	0.085530905	0.930250446
Spns3	0.864131424	0.813552442
Pbp2	-1.906237203	0.692380838
Spocd1	-0.194895771	0.962648047
Spock1	-0.048054995	0.931198698
Spock2	-0.012210771	0.985742667
Spock3	-0.064215429	0.939052665
Spon1	-0.077644864	0.821998633
Spop	0.025851366	0.97487264
Spopl	-0.164447693	0.810908327
Spout1	0.11638203	0.911950951
Spp1	-0.633980622	0.80931022
Sppl2a	-0.03400886	0.971138085
Sppl2b	-0.121723967	0.935897306
Sppl3	-0.133877636	0.626946895
Shisa6	-0.183600563	0.272297898
Slc25a41	-1.914226656	0.940046864
Huwe1	-0.183540737	0.214654626
Rpl28	0.183377176	0.421691269
Grin2a	-0.183030263	0.313975709
Sprn	0.000367518	0.99900438
Sprr2g	0.988306481	0.971661054
Sprtn	0.030171782	0.97487264
Spry1	0.663642669	0.962648047
Spry2	0.083376951	0.936031628
Spry4	-0.208279261	0.702848528
Spryd3	-0.016697532	0.980419928
Spryd4	-0.040361656	0.981045362
Spryd7	-0.081508551	0.821998633
Kcnk1	0.182993824	0.357105886
Spsb2	0.194776782	0.962648047
Spsb3	0.142730547	0.813599082
Hspe1	0.182989709	0.49943116
Sptan1	-0.049723321	0.909243078
Sptb	-0.08444512	0.758595939
Sec16a	-0.182936284	0.459918184

Sod1	0.182831527	0.234605889
Sptbn4	-0.007972839	0.991438757
Sptbn5	-0.213957323	0.963666931
Sptlc1	0.067436725	0.89383516
Sptlc2	0.000215699	0.999486844
Sptlc3	0.578165448	0.968005385
Sptssa	0.153418977	0.817764263
Sptssb	-0.13241353	0.975831674
Spty2d1	-0.102928139	0.868415875
Spx	-0.286557258	0.965520236
Sqle	-0.115685118	0.910328122
Sqor	0.068097663	0.980339705
Lmtk2	-0.182724949	0.39534064
Sra1	0.128702248	0.815987743
Srarp	0.34603095	0.873797321
Evc	0.182461209	0.467829613
Srsf5	0.182430235	0.404892636
Srcap	-0.023571205	0.99527876
Srcin1	0.063479727	0.891972665
Srd5a1	0.031942893	0.984328758
Srd5a3	0.022453855	0.985673938
Srebf1	0.074130765	0.925099239
Srebf2	-0.129494246	0.544661714
Srek1	-0.063705203	0.911631946
Srek1ip1	0.046365902	0.965520236
Vps13b	-0.182393987	0.404892636
Srfbp1	-0.018348846	0.989556168
Srgap1	-0.097975011	0.874422885
Srgap2	-0.109788146	0.757721895
Srgap3	-0.060208936	0.869437456
Srgn	-0.170538652	0.884629276
Sri	0.010822811	0.992152669
R3hdm1	-0.182286494	0.319241271
Srm	-0.055241527	0.950928922
Snora74a	-1.917544906	0.806077586
Psmb3	0.181876868	0.24126198
Srp19	0.071764132	0.895520088
Srp54a	0.178509273	0.771684575
Srp54b	0.172067356	0.771684575
Srp54c	0.119088249	0.865547585
Srp68	-0.006724443	0.994960308
Srp72	-0.187375384	0.748784855
Srp9	0.137321343	0.512730366
Srpk1	0.058549504	0.945117894

Man1a2	-0.181529317	0.457412669
Srpk3	0.026336143	0.993290512
Srpr	-0.037304916	0.962648047
Srprb	-0.140974087	0.685353017
Srpx	-0.303408328	0.975831674
Srpx2	0.384166195	0.895520088
Srr	-0.019471398	0.99165967
Srrd	-0.02176141	0.985742667
Srrm1	-0.080026374	0.74632237
Cndp2	0.181339275	0.161387122
Srrm3	0.006727655	0.99527876
Fbxo43	-1.918267968	0.869316117
Srrm4	-0.036910322	0.972376117
Srrm4os	-0.264881818	0.971795434
Srrt	0.03351175	0.962648047
Srsf1	0.149212811	0.607253593
Srsf10	-0.13044212	0.740833211
Srsf11	-0.026216991	0.975800973
Srsf12	-0.019788536	0.98526072
Foxk1	0.181190088	0.252498589
Srsf3	-0.015437535	0.985742667
Srsf4	0.111473193	0.841017002
Srsf2	0.18118441	0.122206254
Srsf6	0.075919145	0.861366392
Srsf7	0.095052214	0.795890296
Srsf9	-0.024445647	0.974962198
Srxn1	0.042440493	0.948104382
Ss18	0.236875007	0.760547704
Ss18l1	-0.038096773	0.962648047
Ss18l2	0.137663779	0.86849507
Ssb	0.106085944	0.90358675
Phf14	0.18117607	0.491413988
Ssbp2	0.141543843	0.520182905
Prelid3a	0.181094239	0.419052801
Ssbp4	0.108513742	0.837555331
Ssc4d	0.448827967	0.902876216
Ssc5d	-0.380722886	0.655744783
Ssh1	-0.02075717	0.985735386
Ssh2	0.014108258	0.985673938
Ssh3	0.036237409	0.970461999
Zfp664	-1.923159931	NA
Sos1	-0.180968397	0.18316876
Sspn	-0.148108607	0.94417444
Sspo	-0.190926854	0.962648047

Ssr1	-0.72299369	0.604176395
Ssr2	0.049279508	0.957485565
Ssr3	0.06688997	0.865075531
Ssr4	0.099380377	0.875723186
Ssrp1	0.222320028	0.589856572
Sssca1	0.154146749	0.724910334
Sst	-0.064447568	0.938939871
Sstr1	0.276953496	0.87806697
Sstr2	0.002828629	0.998010523
Sstr3	-0.063334452	0.959276014
Fam107a	0.18061792	0.082752362
Sstr5	0.437465185	0.963604673
Ssu2	-0.041794011	0.995295917
Ctss	0.180445951	0.278515147
Ssx2ip	7.9509E-05	0.999790901
St13	-0.055936835	0.889908105
St14	0.92120566	0.522639613
St18	0.281693341	0.510932065
St3gal1	-0.017011135	0.99527876
St3gal2	-0.189788678	0.590745439
St3gal3	0.232757544	0.74463793
Prkcg	-0.180292931	0.435105731
St3gal5	0.082894747	0.889972515
St3gal6	0.119407159	0.91462326
St5	-0.048467157	0.964717692
St6gal1	0.015932817	0.988034453
St6gal2	-0.169642345	0.810908327
St6galnac2	0.320455057	0.745988863
St6galnac3	-0.018116907	0.991996237
St6galnac4	-0.284343291	0.813499809
St6galnac5	-0.111293722	0.654893207
St6galnac6	-0.052888869	0.962648047
St7	-0.052546312	0.963943597
St7l	-0.040852737	0.975831674
Ero1l	-0.180142909	0.268560405
St8sia2	0.183729965	0.962648047
St8sia3	-0.062208571	0.890704638
St8sia3os	-0.36508194	0.777427974
Mfge8	0.180074177	0.473456274
St8sia5	-0.114551137	0.875056988
St8sia6	-0.10953399	0.947745582
Stab1	0.048805632	0.975831674
Stab2	-0.286811177	0.910328122
Ptpn9	-0.179553213	0.403784647

Stac2	0.036958939	0.965520236
Stac3	0.330768392	0.895520088
Stag1	0.240683354	0.710375942
Stag2	-0.041412637	0.970348278
Stag3	-0.265768425	0.927659499
Stam	-0.042668287	0.957169848
Stam2	-0.117503865	0.873797321
Stambp	0.009455003	0.99527876
Stambpl1	-0.047427016	0.962648047
Stamos	0.406212901	0.945663104
Stap1	-0.056663285	0.99100709
Stap2	0.029636675	0.99527876
Star	0.070319891	0.956739982
Stard10	0.059267024	0.970673196
Stard13	0.058593429	0.958389182
Stard3	-0.075400925	0.916426816
Stard3nl	0.090199571	0.832619865
Stard4	-0.083742245	0.87847909
Stard5	0.015192059	0.989840188
Stard6	0.212972202	0.948104382
Stard7	0.238409116	0.615775357
Stard8	-0.04590793	0.973100313
Stard9	-0.212169416	0.722060735
Stat1	-0.037389042	0.974823941
Stat2	-0.075533469	0.951390592
Stat3	0.02308652	0.985293518
Stat4	0.755097823	0.971138085
Tmem151a	-0.179178128	0.451614556
Stat5b	0.255428659	0.69189201
Stat6	-0.211895495	0.821998633
Stau1	0.016098108	0.994960308
Stau2	-0.035503222	0.949607377
Stbd1	0.180499206	0.959276014
Stc1	0.043558162	0.985742667
Stc2	0.215540359	0.865486573
Tnip3	-1.925561193	0.874252042
Steap2	-0.115188995	0.945183336
Steap3	-0.013985534	0.99527876
Steap4	0.404564662	0.962648047
Stil	-0.01860995	0.998010523
Stim1	0.191253299	0.8549794
Stim2	0.017149262	0.98526072
Stip1	0.030904646	0.965520236
Stk10	-0.087784346	0.936031628

Stk11	0.040288552	0.980419928
Stk11ip	-0.042962761	0.962648047
Stk16	0.107128553	0.866560751
Stk17b	0.152322376	0.932673884
Stk19	0.071799331	0.946985353
Stk24	0.037504285	0.962648047
Stk25	-0.023292456	0.968005385
Stk26	0.078927523	0.969755848
Stk3	-0.257007314	0.598116371
Stk31	0.044770721	0.99765039
Stk32a	0.007324044	0.99527876
Stk32b	0.058324829	0.97487264
Stk32c	-0.065428128	0.962923888
Stk33	0.12330443	0.962648047
Stk35	-0.136591325	0.809593157
Stk36	-0.365097772	0.787744512
Stk38	-0.088517149	0.985673938
Rps15a	0.179063105	0.134709123
Stk39	0.023950269	0.97487264
Stk4	-0.119469052	0.710870539
Stk40	-0.202450769	0.663678416
Stkld1	-0.491574296	0.911631946
Stmn1	0.017605149	0.991996237
Stmn2	0.02837207	0.968005385
Stmn3	0.077809795	0.874252042
Fam49a	-0.17895124	0.425271237
Stmnd1	0.004701811	0.99900438
Stn1	-0.025142121	0.985742667
Stom	-0.111764457	0.844996232
Stoml1	-0.075704807	0.941398449
Stoml2	0.009820546	0.993380259
Stoml3	0.128859837	0.97607383
Ston1	0.255425799	0.90872336
Ston2	-0.100991036	0.903441375
Stox1	-0.180776064	0.975290119
Stox2	-0.07795065	0.865075531
Stpg1	-0.047287039	0.98340069
Cd300e	-1.932835546	0.825249193
Stra6	-0.029942454	0.986246396
Strada	0.106751061	0.869316117
Stradb	0.031835251	0.97487264
Strap	-0.026083758	0.971138085
Strbp	-0.147945573	0.90872336
Strc	-0.591787694	0.810908327

Strip1	-0.056198265	0.895520088
Myo9a	-0.178943702	0.299535449
Strn	0.052501692	0.891972665
Strn3	-0.096168325	0.848460959
Strn4	0.011994929	0.991438757
Ndufb8	0.178582949	0.29472142
Stt3b	-0.061543999	0.899097089
Stub1	0.105420808	0.748784855
Stum	-0.071203562	0.882824434
Stx11	-0.461301538	0.974197921
Stx12	-0.013071318	0.985742667
Stx16	-0.658468064	0.833916587
Stx17	-0.15561066	0.815987743
Stx18	-0.064497955	0.950460219
Stx1a	0.023392895	0.97607383
Stx1b	-0.015215125	0.981045362
Stx2	0.046650662	0.968630572
Stx3	0.153533648	0.544661714
Stx4a	0.153136679	0.544661714
Stx5a	-0.050836425	0.953505913
Stx6	0.420989493	0.810908327
Stx7	-0.026878643	0.968005385
Stx8	0.158057189	0.689605643
Stxbp1	-0.264394636	0.939904081
Stxbp2	0.070970117	0.942812446
Stxbp3	0.157580278	0.733575265
Stxbp4	-0.138826567	0.777427974
Stxbp5	-0.114466626	0.642397758
Ppm1e	-0.178578855	0.404892636
Stxbp6	0.258819582	0.52033017
Styk1	-0.838371572	0.775485217
CT485613.2	-1.937173975	0.873797321
Styxl1	-0.06760468	0.99527876
Sub1	-0.021042266	0.975831674
Sucla2	0.028610797	0.97469529
Bptf	-0.178472759	0.121443772
Suclg2	0.246018701	0.661553809
Suco	-0.000657532	0.99900438
Suds3	0.054310124	0.89703631
Sufu	0.109700289	0.87299221
Sugct	0.151002363	0.90872336
Sugp1	0.073186671	0.909109599
Sugp2	-0.152768883	0.690084266
Sugt1	-0.084853824	0.781249046

Sulf1	0.043662577	0.991438757
Sulf2	0.078653981	0.911631946
Ncln	-0.178445817	0.314996191
Sult1c2	-0.136469925	0.99527876
Sult2b1	0.025034909	0.985742667
Sult4a1	0.014430998	0.98526072
Sult5a1	-0.004239402	0.998010523
Sec31b	-1.937790495	0.873797321
Sult6b2	-0.581384386	0.965290366
Sumf1	0.021463061	0.981250052
Sumf2	-0.1876882	0.866574046
Sumo1	0.075014848	0.84277074
Sumo2	0.431900668	0.930250446
Sumo3	0.748880646	0.911631946
Sun1	0.097860473	0.928672524
Sun2	-0.150962386	0.874252042
Odaph	-1.938415466	0.911631946
Suox	0.088738329	0.9089345
Supt16	-0.03422897	0.969755848
Supt20	-0.130310286	0.698018236
Supt3	0.050866597	0.968497942
N4bp1	-0.178210899	0.349775121
Supt4b	0.742161892	0.882824434
Supt5	0.044958814	0.911631946
Supt6	0.019450607	0.971661054
Supt7l	0.064148262	0.950460219
Supv3l1	0.073612919	0.911631946
Surf1	0.116664808	0.746276035
Surf2	0.127995177	0.749519672
Surf4	0.020485242	0.975831674
Surf6	0.107828953	0.872612157
Susd1	0.059447864	0.966213117
Susd2	-0.209378975	0.912600198
Susd3	-0.203550492	0.959276014
Susd4	0.021353794	0.975831674
Susd5	0.271186969	0.815987743
Susd6	-0.072972606	0.894148925
Suv39h1	0.042722805	0.968630572
Suv39h2	0.073960945	0.970461999
Suz12	0.013276248	0.985742667
Sv2a	-0.024420232	0.971138085
Sv2b	-0.109176536	0.930336691
Sv2c	0.176419507	0.894953269
Svbp	0.121059441	0.988034453

Svep1	-0.222482761	0.97607383
Svil	-0.127930822	0.911631946
Svip	-0.030571667	0.965520236
Svop	-0.032965042	0.963943597
Svopl	0.183323763	0.993380259
Swap70	-0.125004724	0.871869598
Swi5	0.188284587	0.6883862
Swsap1	0.081011538	0.952056681
Swt1	-0.338367523	0.624713015
Syap1	-0.089962537	0.91462326
Sybu	0.229334754	0.944753667
Slfn10-ps	-1.943649342	0.889908105
Clhc1	-1.94431992	0.911631946
Syce2	0.045404798	0.976978183
Sycp2	-0.336248009	0.927659499
Sycp3	0.882224397	0.816025904
Syde1	0.07459433	0.959276014
Syde2	0.403945539	0.780228245
Flna	-0.178206653	0.394767148
Syk	-0.047635186	0.984328758
Sympk	-0.001438582	0.99765039
Syn1	-0.057904426	0.908413609
Syn2	0.016101885	0.981392364
Syn3	-0.073076396	0.973100313
Syna	-0.045676639	0.974962198
Sync	-0.429447765	0.948104382
Syncrip	-0.055714175	0.928672524
Syndig1	-0.048782232	0.962648047
Syndig1l	-0.504780743	0.66923652
Syne1	-0.177369726	0.549579107
Stt3a	-0.178084828	0.31865776
Syne3	0.17963472	0.968005385
Syne4	-0.094972288	0.978063275
Syngap1	-0.041101634	0.949450803
Syngr1	0.036269631	0.962648047
Syngr2	0.424686363	0.798109023
Syngr3	-0.062084084	0.911950951
Syngr4	-0.405840265	0.975831674
Synj1	-0.012098023	0.988034453
Nabp2	0.178041253	0.486814463
Synj2bp	-0.076248035	0.874742989
Slc35a5	-0.177756941	0.393039158
Synpo	-0.696792734	0.572363201
Synpo2	0.001999987	0.99900438

Synpo2l	0.540168928	0.874809001
Synpr	-0.640142541	0.810908327
Synrg	-0.035868221	0.953505913
Syp	0.073580619	0.852010446
Sypl	-0.291983904	0.951817826
Sypl2	0.188021662	0.852010446
Sys1	0.087901343	0.932673884
Syt1	-0.045096991	0.911631946
Syt10	-0.055576886	0.985742667
Syt11	-0.05579103	0.891972665
Syt12	-0.116026791	0.852010446
Rgs14	-0.177075914	0.419052801
Syt14	-0.10599929	0.991996237
Syt15	-0.520911253	0.736130784
Syt16	-0.029111245	0.980174892
Syt17	0.18145171	0.965520236
Syt2	0.166817513	0.916961045
Syt3	-0.042700509	0.962648047
Syt4	-0.109466747	0.510920155
Syt5	0.026031165	0.962648047
Syt6	-0.476234491	0.787744512
Syt7	0.171933375	0.980419928
Syt9	0.00456401	0.997652613
Sytl1	-0.171073832	0.98526072
Sytl2	-0.057322534	0.959276014
Sytl4	0.213738638	0.922100633
Sytl5	-0.87129765	0.912358021
Syvn1	0.277631954	0.681327288
Szrd1	0.105930598	0.79212401
Rfx7	-0.177037586	0.450822812
T2	0.071947833	0.991996237
Tab1	-0.023604153	0.985673938
Tab2	0.087018094	0.95126064
Tab3	-0.025081755	0.984328758
Tac1	-0.215402984	0.804540244
Tac2	0.326879208	0.852010446
Tacc1	0.054451995	0.948104382
Tacc2	0.001338782	0.99765039
Tacc3	0.127801797	0.956164302
Taco1	0.17551063	0.756543929
Taco1os	-0.071763829	0.992385525
Tacr1	-0.24436686	0.951817826
Tacr2	-0.41353459	0.984305885
Tacr3	-0.083695688	0.991438757

Tada1	-0.06146177	0.949607377
Tada2a	0.104488019	0.865075531
Tada2b	0.022282936	0.980896205
Kcnd2	-0.176973571	0.496022017
Taf1	-0.114419926	0.771684575
Taf10	-0.114042136	0.809593157
Taf11	0.072518767	0.932673884
Taf12	0.013720419	0.989200732
Taf13	0.068983697	0.962648047
Dut	0.176940845	0.499392203
Taf1a	-0.123955918	0.894953269
Taf1b	0.051338492	0.962648047
Taf1c	0.126611643	0.895986783
Taf1d	0.079190218	0.918235054
Taf2	-0.027312244	0.981502501
Taf3	-0.066134057	0.962363869
Taf4	0.004146604	0.998658264
Taf4b	-0.265124822	0.544661714
Taf5	-0.212520935	0.871869598
Taf5l	0.095201386	0.89703631
Taf6	-0.124632086	0.867041127
Taf6l	-0.047766119	0.968005385
Taf7	0.020952964	0.989840188
Taf7l	0.217986078	0.976978183
Taf8	0.050922822	0.964941885
Taf9	0.028756562	0.985742667
Taf9b	-0.056216623	0.925924974
Tagap	0.170995918	0.868415875
Tagap1	0.058281094	0.920642026
Tagln	0.018613326	0.99527876
Tagln2	-0.313456837	0.623042338
Tagln3	0.063897298	0.90358675
Tal1	0.460687809	0.841017002
Tal2	0.490984463	0.932956012
Taldo1	0.155686603	0.528197276
Tamm41	0.064683366	0.962648047
Tanc1	0.095688845	0.786324313
Tanc2	-0.550921023	0.903795193
Atp13a3	-0.176830341	0.468043447
Tango6	-0.004573973	0.99765039
Tank	-0.069209317	0.936031628
Taok1	-0.096673378	0.749802375
Taok2	0.012799126	0.985742667
Taok3	-0.189834014	0.74463793

Tap1	-0.17870982	0.935890893
Tap2	-0.176875368	0.812945428
Tapbp	-0.042103063	0.986209991
Tapbpl	-0.192238572	0.83886846
Tapt1	-0.016653189	0.984748462
Tarbp1	-0.138128262	0.80931022
Tarbp2	0.118564419	0.874252042
Tardbp	0.676820667	0.806485043
Adad2	-1.946429268	0.873753967
Tars	0.037161449	0.965520236
Tars2	-0.000336418	0.99930339
Gpr37l1	-0.176734623	0.185730446
Tas1r1	-0.0441436	0.985742667
Tas1r3	-0.179862118	0.968005385
Tasp1	0.119027012	0.881371608
Tat	0.090534367	0.99527876
Tatdn1	0.179175641	0.661553809
Tatdn2	-0.054796496	0.955026616
Tatdn3	-0.10367158	0.872352604
Tax1bp1	-0.072079819	0.869316117
Tax1bp3	-0.025637935	0.99100709
Fam114a2	0.176628791	0.434762828
Tbata	0.04788807	0.969755848
Tbc1d1	-0.106842249	0.871869598
Tbc1d10a	0.076816867	0.911631946
Tbc1d10b	-0.072036314	0.874252042
Tbc1d10c	-0.43129433	0.89703631
Tbc1d12	-0.145693449	0.897289612
Tbc1d13	0.001562739	0.998010523
Tbc1d14	0.011684222	0.988034453
Tbc1d15	-0.00967295	0.991438757
Grb14	0.176628177	0.403047809
Tbc1d17	0.074320333	0.935161126
Tbc1d19	-0.029739306	0.980731359
Tbc1d2	-0.241064179	0.807481321
Tbc1d20	-0.001138001	0.998010523
Tbc1d22a	0.154781792	0.611941335
Tbc1d22b	0.039227482	0.961887657
Tbc1d23	-0.091349057	0.868415875
Tbc1d24	-0.191986264	0.69189201
Tbc1d25	0.531836571	0.534501962
Tbc1d2b	-0.081055485	0.932673884
Ppp1r16b	0.176475525	0.29771766
Tbc1d31	-0.103748909	0.936905058

Tbc1d32	-0.192479989	0.704844245
Tbc1d4	0.073723454	0.962648047
Bag1	0.176277062	0.185359794
Meaf6	0.176223306	0.342676783
Tbc1d8	0.011769899	0.99527876
Tbc1d8b	-0.147509995	0.910328122
Tbc1d9	-0.23143934	0.657504489
Tbc1d9b	-0.001318457	0.99765039
Cox7a2	0.176222614	0.064452764
Tbcb	0.021208823	0.984328758
Tbcc	0.133923655	0.795563654
Tbccd1	0.023050517	0.98691726
Tbcd	0.070912457	0.91507125
Tbce	0.003308156	0.99527876
Tbcel	-0.053190157	0.975800973
Tbck	-0.054653776	0.959659722
Tbk1	0.141412301	0.865547585
Tbkbp1	0.074377102	0.894148925
Tbl1x	0.000522313	0.99900438
Tbl1xr1	0.204847815	0.98043358
Tbl2	0.004486905	0.99527876
Tbl3	0.071084072	0.936489142
Tbp	0.116119022	0.732729469
Tbpl1	0.115139731	0.872352604
Tbr1	0.035585629	0.97487264
Tbrg1	0.074799188	0.89703631
Tbrg4	0.155831075	0.740514972
Tbx1	0.046020389	0.993380259
Tbx15	0.186568913	0.975831674
Tbx18	-0.56445751	0.851373921
Tbx2	0.106338692	0.97487264
Ntrk1	-1.946429268	0.873753967
Tbx3	0.174180994	0.945663104
Tbx3os1	0.21812032	0.962648047
Vmn2r115	-1.950597548	0.911631946
Tbx6	-0.427188821	0.962648047
Tbxa2r	-0.984003644	0.5141747
Tbxas1	-0.077615344	0.97469529
Tc2n	0.772121342	0.957499962
Tcaf1	-0.088757082	0.711294758
Tcaf2	-0.049181364	0.99527876
Map6d1	-0.176189626	0.298968315
Tcam1	-0.352526827	0.959659722
Dio3os	-1.951137188	0.911631946

Tcea1	-0.026808665	0.971661054
Tcea1-ps1	0.065901626	0.980339705
Tcea2	0.119624213	0.803910915
Tcea3	0.729422391	0.89703631
Lysmd1	0.175880653	0.442302609
Idh2	0.175875772	0.311609026
Akap13	-0.175767288	0.220337275
Lats2	-0.175665892	0.481156726
Ceacam16	-1.955274806	0.631896124
Tceal8	0.066837775	0.957837415
Tceal9	0.140794625	0.776477378
Tceanc	0.539716507	0.833916587
Tceanc2	0.011812459	0.993103295
Tcerg1	0.023465653	0.974962198
Tcerg1l	0.140379242	0.920642026
Tcf12	-0.203369033	0.795137348
Tcf15	-0.011113721	0.997529192
Tcf19	0.247663496	0.89703631
Tcf20	-0.021403764	0.97469529
Tcf23	0.484305911	0.911631946
Cldn34c2	-1.961044116	0.813071094
Tcf25	-0.066035051	0.862312446
Tcf3	0.069249636	0.968005385
Tcf4	0.106036751	0.92306576
Tcf7	-0.20885263	0.910328122
Grin2b	-0.175627539	0.31865776
Tcf7l2	0.103773496	0.968005385
Gdf15	-1.96227901	0.864907053
Tchh	-0.086181558	0.968005385
Tchp	-0.136110875	0.89703631
Tcim	0.658373491	0.69189201
Tcirg1	-0.017621022	0.991996237
Tcn2	-0.171684258	0.871869598
Tcof1	-0.167727371	0.665532517
Tcp1	0.0174072	0.980339705
Tcp10b	0.325468317	0.98187819
Tcp10c	0.970708578	0.959276014
Tcp11	0.617099719	0.90358675
Hook3	-0.175393318	0.323832374
Tcp11l2	-0.071976476	0.929997774
Tcta	-0.034797378	0.976978183
Tcte1	0.250311608	0.911631946
Srp14	0.175067567	0.455841215
Tcte3	-0.248717235	0.976978183

Lmx1b	-1.96773544	0.828907376
Tctex1d2	-0.036730523	0.984563037
Tctex1d4	-0.020712922	0.997274688
Tctn1	0.095603114	0.810908327
Tctn2	-0.015172273	0.991996237
Tctn3	-0.007009083	0.99527876
Tdg	0.155122426	0.707777756
Tdg-ps	0.287568622	0.854278008
Tdgf1	0.103254255	0.984232465
Rslcan18	-1.970822516	0.597624681
Snx13	-0.175040326	0.398534324
Tdp1	-0.079962532	0.930025175
Tdp2	0.048350314	0.97487264
Tdpx-ps1	0.767742199	0.857478301
Tdrd1	0.023451912	0.99527876
Tdrd12	-0.242959417	0.975831674
Tdrd3	-0.118327257	0.874624896
Tdrd5	0.115020859	0.968005385
Tdrd6	-0.11619755	0.984232465
Tdrd7	-0.012169108	0.988034453
Tdrd9	0.703828219	0.949607377
Tdrkh	-0.092984397	0.80931022
Tdrp	-0.035785277	0.969755848
Tead1	-0.430143551	0.937726496
Tead2	0.03722637	0.994960308
Tead3	0.305889373	0.858549868
Tead4	0.810908941	0.792517766
Tec	-0.397261367	0.812431087
Tecpr1	-0.065094716	0.810908327
Tecpr2	-0.080349626	0.792913409
Tecr	0.120769275	0.638329646
Tecrl	0.735027845	0.852010446
Tecta	0.73174542	0.89703631
Tectb	-0.642973391	0.894953269
Tedc1	0.513475275	0.832980941
Tedc2	0.177459316	0.871869598
Teddm2	0.201026064	0.89703631
Teddm3	-0.287490623	0.971661054
Tef	0.043156489	0.939503568
Fgfr1	-0.174628151	0.307478675
Tek	-0.008351611	0.99527876
Tekt1	0.003963558	0.998010523
Tekt2	-0.297804821	0.948104382
Tekt3	-0.563732336	0.98459388

Tekt4	-0.060028933	0.991996237
Med14	-0.174618133	0.212635473
Telo2	0.047083519	0.975800973
Ten1	-0.00868714	0.99527876
Tenm1	-0.072800324	0.915417975
Txn1	0.174437257	0.389731767
Tenm3	-0.207178013	0.582779449
Tenm4	-0.240984169	0.793949146
Tent2	-0.128092922	0.727640208
Tent4a	-0.041983299	0.962648047
Tent4b	-0.118870523	0.852010446
Tent5a	-0.190025138	0.86783605
Tent5b	0.833722412	0.959659722
Tent5c	-0.491484189	0.692234894
Rps27a	0.174251174	0.186859869
Tepsin	0.037169675	0.970673196
Terb1	0.638656797	0.899249451
Terf1	-0.098823755	0.962648047
Terf2	0.076460274	0.875723186
Terf2ip	-0.05876667	0.911631946
Tert	0.010259754	0.997115148
Tes	0.032890852	0.994960308
Tes3-ps	-0.069825436	0.973100313
Tesc	-0.018420556	0.991438757
Tesk1	-0.030415057	0.974197921
Tesk2	0.01952231	0.99527876
Tesmin	0.269764185	0.993103295
Tespa1	0.543422653	0.839154326
Tet1	0.580011472	0.754457658
Tet2	0.093799779	0.87564489
Tet3	-0.02538021	0.974809035
Tex10	0.021170106	0.98526072
Tex11	0.212854881	0.97487264
Tex13c2	0.596935231	0.585818114
Tex14	0.950264688	0.889972515
Tex15	0.004238618	0.999077673
Tex2	-0.010613342	0.99165967
Tex26	-0.067024828	0.99527876
Tex261	-0.154610159	0.576885155
Tex264	0.084021093	0.939592821
Tex30	-0.083314973	0.968005385
Wnt6	-1.990088526	NA
Tex50	0.038223247	0.99527876
Tex52	-0.062398314	0.988941187

Tex9	-0.072396711	0.97607383
Tfam	-0.09766694	0.91082801
Tfap2b	-0.016582521	0.998350011
AI838599	-2.011561637	0.850777141
Tfap2e	-0.471873453	0.974197921
Tfap4	0.137471363	0.936837873
Tfb1m	0.145665095	0.918407269
Tfb2m	-0.079828955	0.939592821
Tfcp2	-0.128813013	0.981502501
Tfcp2l1	0.11150549	0.965520236
Tfdp1	0.166955315	0.609167039
Rnu1a1	-2.014990262	0.803831758
Tfe3	0.053052669	0.951390592
Tmem8b	-0.173984702	0.503067237
Tfg	0.075755367	0.87847909
Tfip11	0.062989653	0.918540666
Tfpi	0.28214475	0.895198797
Tfpt	0.138393222	0.847678217
Tfr2	-0.144626845	0.948104382
Tfrc	-0.014501105	0.985673938
Tgds	-0.17006101	0.854278008
Tgfa	0.078685965	0.883426561
Tgfb1	-0.221017549	0.823072541
Tgfb1i1	0.001522448	0.998768596
Tgfb2	0.006852322	0.994824604
Tgfb3	0.060810628	0.965520236
Tgfbi	0.009112279	0.99765039
Tgfbr1	0.030522934	0.971661054
Tgfbr2	-0.03603641	0.985742667
Tgfbr3	0.10200294	0.895520088
Tgfbr3l	0.2134135	0.874971335
Tgfbrap1	-0.04876805	0.99527876
Tgif1	-0.094349084	0.984328758
Mpzl2	-2.019980515	0.779231312
Adgre4	-2.036586027	0.889972515
Tgm1	-0.734282337	0.939553162
Tgm2	-0.286722987	0.7851601
Tgm3	-0.573810401	0.815864767
Tgm4	0.116362175	0.985477412
Tgm6	-0.58738123	0.97487264
Tgm7	-0.083970868	0.985742667
Tgoln1	-0.065068052	0.841017002
Tgs1	-0.101700501	0.864150749
Tgtp1	-0.368070989	0.91462326

Tgtp2	-0.799706016	0.539381969
Th	-0.607038648	0.911631946
Tha1	-0.066605867	0.974823941
Thada	-0.006975099	0.99527876
Thap1	-0.007517818	0.99527876
Thap11	0.013149839	0.991438757
Thap12	-0.006489033	0.992385525
Thap2	-0.191198417	0.655744783
Thap3	-0.0069942	0.99527876
Thap4	0.246542122	0.771684575
Thap6	-0.427766509	0.789833208
Thap7	0.010525564	0.99527876
Thap8	0.605337086	0.974962198
Daam2	0.17394303	0.394955649
Thbs1	0.117045511	0.975831674
Thbs2	0.061702233	0.984328758
Thbs3	0.188435181	0.826209871
Rpl10-ps6	0.173347062	0.488671938
Theg	-0.781714964	0.928672524
Thegl	-0.728902762	0.705443353
Them4	0.017699627	0.985742667
Them6	0.053555495	0.929997774
Themis2	0.632408765	0.708001635
Thg1l	0.294103552	0.596982103
Thnsl1	-0.113341198	0.939904081
Thnsl2	0.148649	0.928957615
Thoc1	-0.149339715	0.775485217
Thoc2	-0.417917701	0.595105754
Thoc3	0.100768335	0.809593157
Thoc5	0.085653438	0.859803076
Thoc6	-0.301010001	0.629782439
Thoc7	-0.019582264	0.987326705
Thop1	-0.020165337	0.980339705
Thpo	-0.119643735	0.945663104
Thra	0.084517628	0.725853134
Thrap3	0.034138485	0.948407374
Ctdspl	0.173337339	0.442068044
Tmed8	-0.17328064	0.207659271
Thsd1	-0.297313317	0.723108545
Thsd4	-0.146450504	0.650504271
Thsd7a	0.171413373	0.980339705
Ap4s1	0.173258896	0.41351939
Lman2	-0.173140065	0.161844023
Thtpa	-0.039359515	0.97487264

Thumpd1	0.005696838	0.99527876
Thumpd2	0.343655265	0.621993647
Thumpd3	-0.010326167	0.994806961
Thy1	0.060039938	0.912567488
Thyn1	0.097558831	0.793949146
Tia1	0.320749675	0.928489442
Tial1	0.054728772	0.949111839
Tiam1	-0.050170333	0.986246396
Tiam2	0.10529908	0.910328122
Ticam1	0.09212519	0.951949091
Ticam2	0.058866584	0.989840188
Ticrr	0.328824093	0.975800973
Tie1	0.040926	0.985742667
Tifa	0.06280357	0.968005385
Tifab	-0.013559946	0.99527876
Tigar	0.061307745	0.911631946
Tigd2	0.177311421	0.731867452
Tigd3	0.103476277	0.974823941
Tigd5	0.082965418	0.963943597
Timeless	0.063179898	0.984748462
Timm10	0.103463327	0.90358675
Timm10b	0.072925462	0.960201673
Timm13	-0.03624327	0.97487264
Timm17a	0.00140108	0.997884966
Timm17b	0.102593557	0.912042382
Timm21	-0.17952643	0.792641681
Timm22	-0.02273768	0.996094752
Timm23	-0.043844147	0.984748462
Timm29	0.08102321	0.837755777
Timm44	0.047172664	0.965520236
Timm50	-0.015566277	0.993380259
Timm8a1	-0.065144089	0.970348278
Cacnb2	-0.172811308	0.260861196
Timm9	0.173718959	0.821998633
Timmdc1	0.153343239	0.780007405
Timp1	-0.309481361	0.984335668
Timp2	0.001115435	0.998010523
Timp3	-0.012801111	0.994960308
Timp4	0.119436946	0.736771155
Tinagl1	0.235351889	0.837755777
Tinf2	0.032065005	0.989200732
Tiparp	-0.144740225	0.886647768
Tbc1d30	-0.172790709	0.396025513
Tiprl	0.07534214	0.896196194

Tirap	0.044568027	0.991438757
Tjap1	0.339167088	0.534501962
Tjp1	-0.164975559	0.591428304
Tjp2	-0.128626364	0.861366392
Tjp3	-0.664743152	0.69466447
Tk1	0.311009095	0.959276014
Tk2	0.077897068	0.97487264
Tkfc	-0.075024214	0.962648047
Tkt	-0.029620016	0.969755848
Tktl1	-0.817472537	0.97487264
Tktl2	-0.199901869	0.980132658
Tlcd1	0.129056263	0.803910915
Mir3072	-2.056056507	0.538031574
Tldc1	0.149057744	0.837755777
Tle1	-0.142705308	0.6883862
Tle2	-0.095407639	0.90358675
Tle3	0.075444004	0.965520236
Tle4	-0.307095408	0.596982103
Tle6	0.04710269	0.991438757
Tlk1	-0.067881914	0.891972665
Tlk2	0.022069699	0.98526072
Tll1	-0.131397081	0.952041545
Tll2	-0.211148211	0.975831674
Tln1	-0.11515177	0.770793864
Tln2	-0.168213627	0.548494586
Tlnrd1	-0.119714132	0.896196194
Tlr1	-0.438957961	0.959276014
Tlr12	-0.347854816	0.93987194
Tlr13	0.209043515	0.948104382
Tlr2	-0.689657895	0.660781615
Ehd3	0.172577817	0.218541263
Tlr4	0.041871411	0.994960308
AC154912.5	-2.062067513	0.903795193
Tlr6	0.045784564	0.994980068
Tlr7	0.261493454	0.889972515
Tlr9	-0.196695646	0.969979757
Tm2d1	-0.089061994	0.911631946
Tm2d2	0.166983998	0.873753967
Tm2d3	-0.059443831	0.958725703
Tm4sf1	-0.090095186	0.939052665
Tm4sf19	0.198824999	0.993103295
Tm4sf5	0.623474233	0.97469529
Tm6sf1	-0.052873165	0.974962198
Tm6sf2	0.091773093	0.98459388

Tm7sf2	0.146145039	0.871869598
Tm7sf3	0.088364654	0.814814675
Tm9sf1	-0.047808496	0.981392364
Tm9sf2	-0.047231148	0.909728442
Tm9sf3	-0.025176496	0.971661054
Tm9sf4	0.000757141	0.998010523
Tmem131l	-0.172464437	0.497690258
Tma7	0.185238157	0.568220567
Tma7-ps	0.424274382	0.911631946
Tmbim1	-0.121729837	0.864150749
Tmbim4	-0.101015052	0.942812446
Tmbim6	-0.009687987	0.987260085
Folr2	-2.078070308	0.904346827
Tmc4	-0.00412965	0.99765039
Tmc5	-0.095286575	0.995944208
Tmc6	0.251469905	0.869316117
Tmc7	0.152028356	0.638414898
Tmc8	0.773997312	0.874252042
Tmcc1	0.128753052	0.703372057
Tmcc2	0.103461239	0.873797321
Tmcc3	0.309710159	0.54807923
Tmcc3os	-0.750892042	0.959276014
Tmco1	0.016222704	0.989378233
Tmco3	-0.098712616	0.815987743
Tmco4	-0.291535187	0.795137348
Tmco5	0.068572175	0.989347727
Tmco5b	0.689042603	0.951390592
Tmco6	-0.091232701	0.936031628
Tmed1	0.019283176	0.993103295
Tmed10	-0.043179157	0.939904081
Tmed10-ps	0.130132057	0.965520236
Syce1	-2.092481662	0.80931022
Tmed3	0.008784626	0.99527876
Tmed4	0.059500739	0.879969865
Tmed5	-0.125924626	0.890704638
Tmed7	-0.126926096	0.529257466
Chd8	-0.172316051	0.380767313
Tmed9	0.165462817	0.581125035
Tmeff1	-0.038346655	0.961373288
Tmeff2	-0.030418956	0.962648047
Tmem100	-0.002221237	0.99765039
Tmem101	0.064379357	0.964941885
Tmem102	0.287467858	0.964011524
Tmem104	-0.091812594	0.91462326

Tmem106a	-0.563564153	0.849381566
Tmem106b	-0.069172556	0.89703631
Tmem106c	-0.031263264	0.975800973
Tmem107	0.090493672	0.971138085
Tmem108	0.103890667	0.904346827
Tmem109	0.031417328	0.978652522
Tmem11	0.105712341	0.852010446
Tmem110	0.131241362	0.889972515
Tmem114	0.140042693	0.952385409
Tmem115	-0.065370289	0.957499962
Tmem116	-0.16120501	0.936031628
Tmem117	0.086185642	0.944753667
Tmem119	0.066748674	0.956164302
Tmem120a	-0.118460672	0.911631946
Tmem120b	-0.015735972	0.992385525
Tmem121	-0.286920148	0.610726714
Snca	0.17224665	0.324787134
Tmem123	-0.124713717	0.87806697
Tmem125	0.007308126	0.997115148
Tsc22d4	0.172211342	0.475741945
Tmem126b	-0.010489835	0.989640643
Tmem127	-0.115895749	0.811913793
Tmem128	0.01769069	0.985673938
Tmem129	-0.37100449	0.770793864
Tmem130	-0.061752275	0.895520088
Tmem131	-0.211323989	0.837755777
Frrs1l	-0.171963437	0.309245307
Tmem132a	-0.047627371	0.936031628
Tmem132b	-0.042500469	0.911631946
Tmem132c	0.018561277	0.99527876
Tmem132d	-0.182116239	0.852010446
Tmem132e	-0.185314805	0.861397728
Tmem134	-0.418022104	0.548869952
Tmem135	0.0456186	0.956737477
Itpr1	-0.17180994	0.126707005
Tmem138	0.052826352	0.97469529
Tmem140	0.209151408	0.961546604
Tmem141	0.02744041	0.988034453
Tmem143	-0.059991642	0.94867599
Tmem144	-0.001139044	0.998010523
Srf	-0.17180427	0.366338083
Tmem147	0.076002309	0.932673884
Tmem147os	0.187520544	0.815987743
Tmem14a	0.107096989	0.850777141

Tmem14c	0.049203277	0.956164302
Tmem150a	-0.10848873	0.920642026
Tmem150b	-0.131542169	0.957499962
Tmem150c	0.004841376	0.99527876
Tmem150cos	0.929075604	0.848733914
Rpl7a-ps11	0.171726501	0.38218128
Tmem151b	-0.023654597	0.980626239
Tmem154	-0.217764798	0.979037366
Mir674	-2.123469304	NA
Tmem158	-0.206882297	0.78686705
Tmem159	-0.053860485	0.980896205
Tmem160	0.121321885	0.911631946
Tmem161a	0.25213602	0.84384699
Tmem161b	-0.007299855	0.99527876
Tmem163	0.346288456	0.605310774
Kcnq3	-0.170732894	0.482385306
Tmem165	-0.058442646	0.936031628
Tmem167	-0.119846774	0.788592804
Tmem167b	0.032539938	0.962648047
Tmem168	0.347617956	0.74032489
Tmem169	-0.116030113	0.939052665
Tmem17	-0.069553664	0.97469529
Tmem170	0.087436937	0.965256427
Asb17	-2.125152839	0.802611737
Tmem171	0.116645286	0.985293518
Tmem173	-0.352696789	0.719622087
Tnr	-0.170395819	0.224597392
Tmem175	-0.083857055	0.857611652
Tmem176a	-0.008389071	0.99527876
Tmem176b	-0.088967051	0.902496496
Tmem177	0.020186312	0.989998473
Tmem178	0.037764912	0.961373288
Tmem178b	-0.13289681	0.571020232
Tmem179	-0.07298161	0.875723186
Tmem18	0.098204634	0.777427974
Tmem181a	-0.289481018	0.56399886
Tmem181b-ps	-0.282563408	0.903441375
Tmem181c-ps	-0.272186202	0.890704638
Hist1h2ag	-2.132755451	NA
Tmem183a	0.022891691	0.976978183
Tmem184a	-0.413396107	0.98501783
Glg1	-0.170388007	0.232043388
Tmem184c	-0.008752911	0.99527876
Tmem185a	0.029656119	0.974962198

Tmem185b	0.065962506	0.969755848
Tmem186	-0.005432791	0.99527876
Tmem189	-0.060182006	0.988034453
Tmem19	0.04601172	0.957499962
Tmem190	0.52806034	0.948104382
Tmem191c	-0.051671877	0.947033215
Tmem192	0.027352068	0.98691726
Tmem196	-0.14897315	0.968005385
Tmem198	-0.182999728	0.673443553
Tmem198b	0.236416373	0.764358191
Tmem199	0.0770671	0.891972665
Cacna1d	-0.169959421	0.430539896
Tmem200b	-0.613770064	0.854454414
Tmem200c	-0.067107842	0.971138085
Tmem201	-0.008549285	0.991120895
Tmem202	0.190045642	0.95347365
Tmem203	-0.093035397	0.962648047
Tmem204	-0.062356585	0.965520236
Tmem205	-0.133018353	0.852010446
Tmem206	0.027370302	0.985742667
Tmem208	0.13064473	0.84870301
Tmem209	-0.043141182	0.971891838
Tmem210	-0.088411149	0.988034453
Tmem212	0.211438731	0.962648047
Tmem214	0.007615555	0.99527876
Tmem215	0.172741294	0.868415875
Tmem216	0.00062424	0.999242708
Tmem218	-0.177355802	0.818088737
Tmem219	0.121066741	0.87806697
Tmem220	0.061986693	0.985742667
Tmem222	-0.011211606	0.987342614
Tmem229a	-0.025281303	0.980339705
Tmem229b	-0.068974908	0.89703631
Tmem230	0.035319031	0.971138085
Tmem231	0.213670761	0.631708623
Tmem232	0.153276555	0.974962198
Mir7647	-2.132756284	NA
Tmem234	-0.011777115	0.991438757
Tmem236	-0.335280177	0.974197921
Tmem237	-0.034317969	0.971661054
Tmem238	-0.263712405	0.968005385
Tmem240	0.140856707	0.764997798
Tmem241	-0.003318398	0.99765039
Tmem242	0.109619312	0.731137993

Aff4	-0.169954512	0.220337275
Tmem245	-0.654872509	0.942511266
Tmem246	0.005731007	0.99527876
Tmem248	0.014560525	0.985446046
Tmem249	0.065100166	0.99527876
Tmem25	-0.050303671	0.953505913
Tmem250-ps	0.115137139	0.760547704
Tmem251	0.085579499	0.948104382
Tmem252	0.237050065	0.91462326
Tmem253	-0.621924645	0.869316117
Tmem254a	-0.062667495	0.968005385
Tmem254b	-0.045312931	0.980132658
Tmem254c	-0.030480845	0.985742667
Tmem255a	-0.104348253	0.881185267
Tmem255b	-0.359323909	0.890068126
Tmem256	0.069681011	0.959276014
Kdm5b	-0.169840679	0.128640258
Tmem259	-0.056297815	0.932673884
AC154912.4	-2.141976678	0.902293377
Exoc1	-0.169309597	0.307478675
Tmem263	-0.139747075	0.767515266
Tmem265	0.866697443	0.67239949
Tmem266	0.072262251	0.971138085
Tmem267	0.23506379	0.973100313
Tmem268	0.150043967	0.779231312
Tmem269	-0.022727837	0.99527876
Tmem273	-0.17599034	0.98340069
Tmem28	-0.110583256	0.864280235
Tmem29	0.141733664	0.873797321
Tmem30a	-0.144777616	0.581125035
Mir684-1	-2.153456779	0.858549868
Slc34a3	-2.169416559	0.889908105
Tmem33	-0.103567426	0.874252042
Tmem35a	0.09356146	0.818088737
Tmem35b	-0.170104439	0.962648047
Tmem37	-0.291567054	0.968005385
Tmem38a	0.078934461	0.883426561
Tmem38b	-0.012345893	0.993290512
Tmem39a	0.046295169	0.98043358
Tmem39b	0.12938932	0.920642026
Tmem40	0.335728683	0.965520236
Tmem41a	-0.002507645	0.99765039
Tmem41b	-0.031037795	0.973100313
Tmem42	-0.198817486	0.695239733

Tmem43	-0.119080905	0.737860635
Tmem44	-0.089548721	0.91462326
Tmem45a	-0.394554087	0.91462326
Gata6	-2.174294376	0.897878088
Tmem45b	0.111876942	0.99527876
Tmem47	-0.35756001	0.547004721
Tmem50a	0.026903896	0.974823941
Tmem50b	-0.075505977	0.891265024
Tmem51	-0.09414605	0.97607383
Xcr1	-2.175743334	0.836720624
Tmem52	0.612206703	0.722843611
Tmem53	0.351041328	0.867827234
Kbtbd11	0.169258844	0.491413988
Tmem59	0.200202262	0.8549794
Tmem59l	-0.026406832	0.965520236
Tmem60	0.093741249	0.882824434
Tmem62	-0.05039222	0.97487264
Tmem63a	-0.184570631	0.648241111
Tmem63b	-0.045863371	0.914675447
Slc39a10	-0.169174588	0.360222394
Rhob	0.169008415	0.360222394
Tmem65	-0.037101166	0.968005385
Tmem67	-0.209482975	0.605064513
Tmem68	-0.046160177	0.965520236
Tmem69	0.089858513	0.932832967
Tmem70	-0.277717791	0.796563869
Tmem71	0.583223161	0.948104382
Tmem72	-0.102638719	0.98501783
Klf13	0.168498204	0.186938452
Tmem74b	-0.439632591	0.727005393
Tmem74bos	0.213994039	0.97487264
Tmem79	0.010455337	0.99727603
Tmem8	-0.031114989	0.981392364
Tmem80	0.051744384	0.971138085
Tmem81	0.080094708	0.964011524
Tmem82	-0.125010669	0.98526072
Tmem86a	0.085323273	0.945182261
Tmem86b	0.069826861	0.970348278
Tmem87a	-0.183713755	0.882096017
Tmem87b	-0.09764147	0.984305885
Tmem88	0.02651738	0.99527876
Eps15	-0.16824784	0.413412065
Tmem89	0.198775802	0.993380259
Nufip2	-0.168169369	0.445106721

Tmem9	0.164123526	0.896196194
Tmem91	-0.188149416	0.89703631
Tmem94	-0.08453733	0.852010446
Tmem97	0.116077551	0.89703631
Tmem98	-0.140496155	0.90358675
Tmem9b	-0.03393292	0.965520236
Tmf1	-0.126650596	0.897878088
Tmie	0.164156173	0.948407374
Tmigd3	0.009898643	0.997676331
Tmod1	0.118659823	0.80818949
Tmod2	-0.110151696	0.55300141
Tmod3	-0.097697774	0.889972515
Bhlhe23	-2.17924946	0.888622599
Tmpo	-0.17883297	0.581475897
Tmppe	-0.025330531	0.989556168
Tmprss11a	-0.477323585	0.984305885
Tmprss2	0.333810563	0.990550129
Tmprss3	0.135254218	0.994980068
Tmprss5	-0.77260858	0.918451137
Tmprss6	0.214897206	0.972218452
Tmprss7	0.113402789	0.984748462
Tmprss9	0.237898001	0.966608583
Sbds	0.168069567	0.421157413
Tmsb15b1	0.147885747	0.955993235
Tmsb15b2	0.272085221	0.896196194
Tmsb15l	0.121573641	0.962648047
Tmsb4x	0.075024641	0.889908105
Tmtc1	-0.053645057	0.957499962
Tmtc2	-0.028142536	0.985742667
Zfp831	-0.167527539	0.465255523
Tmtc4	-0.218198683	0.789833208
Tmub1	-0.016159426	0.990079957
Tmub2	-0.108611426	0.76998058
Tmx1	-0.021370884	0.984748462
Tmx2	0.282189622	0.891972665
Tmx3	-0.163739328	0.567985794
Tmx4	-0.045846858	0.940158904
Tnc	-0.302864361	0.636632633
Tnf	-0.148093261	0.985742667
Tnfaip1	0.020565707	0.982204947
Rpl18a	0.167426627	0.311609026
Vps13c	-0.167157197	0.41351939
Tnfaip6	0.15869802	0.89703631
Tnfaip8	-0.185473451	0.874252042

Tnfaip8l1	-0.124745363	0.939052665
Tnfaip8l2	0.223753028	0.91462326
Tnfaip8l3	-0.012593959	0.985742667
Tnfrsf10b	-0.064712569	0.990690958
Tnfrsf11a	0.26554012	0.861397728
Tnfrsf11b	-0.795021274	0.779415976
Adam10	-0.16687376	0.298968315
Tnfrsf13b	0.5267891	0.874252042
Tnfrsf13c	0.656103233	0.911631946
Uhmk1	-0.166848839	0.224597392
Lrrc17	-2.185216059	0.723708381
Tnfrsf18	-0.049531761	0.987549838
Kars	0.166495232	0.146214787
Tnfrsf1a	-0.003134943	0.99765039
Tnfrsf1b	-0.25900915	0.860521845
Tnfrsf21	-0.143314856	0.613183359
Tnfrsf22	-0.344198871	0.911631946
Tnfrsf23	-0.439640963	0.848460959
Rtca	0.166359973	0.421157413
Tnfrsf26	-0.480521066	0.972218452
Tnfrsf4	0.551798813	0.91462326
Tnfrsf8	0.308332835	0.858549868
Tnfrsf9	0.339003841	0.911631946
Tnfsf10	-0.850470428	0.573316774
Aagab	0.166099649	0.298968315
Tnfsf12	-0.012708579	0.994960308
Tnfsf13	-0.858153431	0.816545416
Tnfsf13b	0.40965042	0.902293377
Tnfsf13os	-0.666127211	0.957849305
AC154237.1	-2.195381863	0.905559486
Tnfsf9	-0.105974297	0.986442322
Tnfsfm13	-0.060795274	0.987342614
Tnik	-0.13023722	0.882096017
Tnip1	0.007443324	0.99527876
Mtmr2	0.165008393	0.399438388
Pkp3	-2.211126565	0.810908327
Bnipl	-2.221942499	0.883333293
Tnk2	0.119441511	0.899438847
Tnk2os	-0.519469989	0.89703631
Tnks	-0.119555378	0.566572868
Tnks1bp1	-0.053305107	0.910524966
Tnks2	-0.071447335	0.869437456
Tnmd	-0.011857543	0.998326395
Tnn	-0.990675455	0.910328122

Tnnc1	0.610301934	0.639249849
Spo11	-2.234197753	0.886647768
Tnni1	0.098554904	0.971661054
Ighg2c	-2.235711724	0.760750768
Tnni3	-0.754875683	0.786324313
Tnni3k	0.404442834	0.965520236
Tnnt1	0.254897058	0.91462326
Tnnt2	0.237593284	0.911276003
AC122141.1	-2.266231721	0.810908327
Tnpo1	-0.596168115	0.953505913
Tnpo2	-0.07158341	0.903441375
Tnpo3	-0.109833402	0.932673884
Ccser2	-0.164825466	0.334979132
Sptbn2	-0.164820998	0.316193532
Tnrc6a	-0.032172057	0.975800973
Tnrc6b	-0.092904029	0.866560751
Tnrc6c	-0.066305403	0.867970668
Tns1	0.684069765	0.578812716
Tns2	0.105769727	0.911631946
Pqbp1	0.164694946	0.442302609
Gbp10	-2.297393358	0.860142574
Tnxb	-0.229058198	0.581319682
Nefm	0.164199641	0.318121654
Tob2	0.089640605	0.874422885
Toe1	0.00145659	0.998010523
Rab31	0.16332873	0.41394684
Togaram2	0.121717839	0.974823941
Tollip	0.032240968	0.950460219
Tom1	0.209359101	0.84277074
Tom1l1	-0.177643509	0.615775357
Tom1l2	-0.054853784	0.902293377
Tomm20	0.02013822	0.968005385
Tomm22	0.062182676	0.921311891
Tomm34	-0.04975074	0.95126064
Tomm40	-0.067125464	0.911631946
Tspyl2	0.163178047	0.208908041
Ptpru	-0.163060255	0.442132509
Plcb1	-0.162954573	0.442068044
Tomm6os	0.128440102	0.928672524
Tomm7	0.182601308	0.55734258
Tomm70a	-0.046519451	0.89690885
Tomt	-0.870128672	0.780410511
Tonsl	-0.098513943	0.962648047
Top1	-0.141596462	0.7851601

Top1mt	-0.050633019	0.968005385
Top2a	0.147104872	0.968977192
Top2b	-0.067856797	0.91462326
Top3a	0.242532021	0.744046401
Rps10	0.162499903	0.320642688
Topbp1	-0.070452484	0.936031628
Topors	-0.055507747	0.957499962
Tor1a	0.003035677	0.996487647
Tor1aip1	-0.063933209	0.935822376
Tor1aip2	-0.265888334	0.629280869
Tor1b	-0.087780327	0.944494911
Tor2a	-0.006632045	0.996742429
Tor3a	0.090767936	0.962648047
Tor4a	-0.017083147	0.99527876
Tox	0.11030458	0.962648047
Tox2	-0.150456626	0.792517766
Tox3	-0.042049774	0.980896205
Tox4	-0.030433961	0.971138085
Tpbg	-0.113744424	0.968005385
Tpbgl	0.241223728	0.895198797
Tpcn1	-0.111919128	0.673833998
Tpcn2	-0.128295252	0.955993235
Tpd52	-0.238163891	0.961300588
Tpd52-ps	-0.86831095	0.965520236
Specc1	-0.161848068	0.23873271
Gabrb1	-0.161826273	0.350732723
Tpgs1	0.11803797	0.848380246
Tpgs2	-0.01879571	0.984748462
Tph1	0.12091039	0.988034453
Tph2	-0.309694468	0.9069528
Tpi-rs11	-0.047273797	0.99527876
Tpi1	0.074294728	0.87564489
Tpk1	0.318301109	0.903441375
Acadl	0.161708657	0.465315287
Atp5h	0.1616365	0.161387122
Tpm3	0.031403844	0.969755848
Tpm3-rs7	0.120939702	0.91507125
Tpm4	-0.126042088	0.792641681
Tpmt	-0.03262566	0.985735386
Tpo	0.31197232	0.985742667
Tpp1	-0.179480383	0.861366392
Tpp2	-0.117983942	0.778639508
Dgkz	-0.161562081	0.414106926
Rpl26	0.161198855	0.169063144

Tpr	-0.03101428	0.973100313
Tpra1	-0.112066823	0.916426816
Tprgl	0.004805727	0.99527876
Tprkb	-0.029490353	0.968005385
Git1	-0.161170064	0.492371645
Tpst1	0.018400366	0.981045362
Tpst2	0.274479508	0.707655454
Fam91a1	-0.160811474	0.431061843
Tpt1-ps3	0.113709226	0.630703567
Tpt1-ps5	0.660648647	0.950195611
Tpt1-ps6	0.326821227	0.947447204
Cnr1	-0.160735685	0.333667896
Tpx2	0.041766846	0.989840188
Tra2a	0.003524058	0.995411429
Tra2b	0.059912224	0.89703631
mt-Cytb	0.160627718	0.508471705
Trabd2b	0.420587123	0.689605643
Trac	-0.357111808	0.928489442
Tradd	0.072789735	0.973100313
Kif3c	-0.16038566	0.509250303
Traf2	-0.014845333	0.994960308
Traf3	-0.269030421	0.91462326
Traf3ip1	-0.003045631	0.997375151
Traf3ip2	-0.036812336	0.989556168
Traf3ip3	-0.40463385	0.864150749
Traf4	-0.010160461	0.99527876
Traf5	0.531909134	0.573810203
Traf6	-0.048822422	0.962094124
Traf7	0.060779816	0.925924974
Trafd1	0.000424117	0.999242708
Traip	-0.049391419	0.980144634
Trak1	0.003389883	0.99527876
Trak2	-0.100651556	0.56399886
Tram1	-0.070268045	0.91334561
Tram1l1	-0.015157256	0.987342614
Tram2	-0.121335282	0.968630572
Trank1	-0.055321863	0.970348278
Trap1	0.038452361	0.962648047
Rbx1	0.160380524	0.327922951
Trappc10	-0.015885131	0.985653607
Trappc11	-0.012325536	0.991438757
Trappc12	-0.06444708	0.911631946
Trappc13	-0.100085884	0.814052898
Trappc2	0.033587946	0.968005385

Trappc2l	0.103427528	0.757217583
Rgs7bp	-0.159668564	0.425470982
Trappc3l	0.740854943	0.976546786
Trappc4	0.088463639	0.936031628
Trappc5	0.107033334	0.779346076
Trappc6a	-0.071939537	0.971661054
Trappc6b	0.177820939	0.783229859
Trappc8	-0.053409323	0.946985353
Trappc9	-0.044052271	0.951949091
Trarg1	-0.233609298	0.97487264
Trat1	-0.372597699	0.911834483
Trav12-4	0.109468081	NA
Trav3-3	-0.45553246	0.974809035
Trav3d-3	0.641174791	0.959659722
Trav3n-3	-0.043938664	0.996487647
Trav9d-4	0.136297555	0.994960308
Trav9n-4	0.135331011	0.991438757
Trbc2	-0.284960571	0.974823941
AU021063	-2.307943309	0.696211886
Clec10a	-2.317073292	0.936031628
Trdc	-0.383790881	0.963666931
Trdmt1	-0.022490562	0.991996237
Barhl1	-2.327270779	0.873797321
Trdv4	-0.73212431	0.981392364
Trem2	0.061649113	0.963943597
Treml2	0.312272916	0.959381512
Treml4	0.531794061	0.981392364
Trerf1	0.271267782	0.744924545
Trex1	0.083916952	0.985673938
Trf	-0.062511881	0.873797321
Trh	-0.988942492	0.893225143
Trhde	-0.129410885	0.811913793
Trhr	0.59687601	0.630888733
Trhr2	-0.33225092	0.985446046
Triap1	0.050047676	0.962648047
Trib1	-0.000283506	0.999486844
Trib2	-0.054938933	0.963943597
AC129574.2	-2.3606099	NA
Tril	-0.043002384	0.962648047
Trim10	0.05869532	0.997241386
Trim11	0.091144214	0.89383516
Trim12a	-0.096124452	0.980339705
Trim12c	-0.282898102	0.918451137
Trim13	-0.175717857	0.833916587

Kdm2a	-0.159484493	0.235649093
Trim15	-0.196205582	0.984232465
Trim16	0.785081405	0.565635635
Rbm28	-0.159464051	0.445106721
Trim2	-0.42433535	0.798580668
Trim21	-0.119129411	0.965520236
Trim23	-0.128908355	0.882096017
Trim24	-0.085994153	0.86783605
Trim25	-0.10925546	0.928672524
Card11	-2.391011474	0.77156461
Trim27	0.15299746	0.740833211
Trim28	0.013478631	0.984748462
Trim29	0.514979741	0.968630572
Trim3	0.000239685	0.999242708
Trim30a	-0.07127227	0.98526072
Mir155hg	-2.410498151	0.852275035
Trim30d	-0.253834287	0.895520088
Smok4a	-2.411028402	0.858549868
Trim32	0.026540832	0.968005385
Trim33	-0.133690277	0.695239733
Trim34a	0.096654626	0.98187819
Trim35	0.017093777	0.980339705
Fam120a	-0.159154251	0.130213261
Lrp6	-0.159143772	0.491845399
Trim39	-0.01900562	0.985673938
Trim40	0.855979961	0.968005385
Trim41	0.162154025	0.608872006
Trim43a	-0.322579366	0.961373288
Trim43c	0.96657286	0.932673884
Trim44	-0.05302069	0.873797321
Trim45	0.109380563	0.92306576
Trim46	-0.14314183	0.630498632
Trim47	-0.140793038	0.967662387
Trim5	0.542706156	0.891972665
Obp2b	-2.42920712	0.894430387
Nxnl1	-2.479096317	0.630703567
Trim56	-0.076390282	0.970348278
Htt	-0.159089751	0.39534345
Rnf114	0.159006493	0.377143991
Trim62	-0.009955539	0.99527876
Snora16a	-2.502594463	0.6883862
Trim65	0.0409818	0.991996237
Trim66	0.185635409	0.962648047
Trim67	-0.040150877	0.987814724

Trim68	-0.297358087	0.631946271
Trim7	0.323274784	0.858549868
Trim71	-0.365630225	0.792517766
Trim72	0.612542457	0.76034043
Cxcr6	-2.554425632	0.879133004
Trim8	-0.115022286	0.804491173
Trim9	-0.055729641	0.889972515
Stk38l	-0.158825763	0.490781464
Lrp1	-0.157825539	0.144051788
Trip10	-0.109173215	0.947745582
Trip11	-0.047599416	0.962648047
Ndufb3	0.157660597	0.386030517
Trip13	0.222029095	0.962648047
Trip4	-0.124070026	0.882096017
Trip6	-0.118069827	0.955706294
Triqk	0.1504038	0.940504226
Trir	0.109052877	0.861366392
Trit1	0.044500702	0.968005385
Trmo	0.381609922	0.690084266
Trmt1	0.097458526	0.81426867
Trmt10a	0.02750867	0.987941353
Trmt10b	0.081750424	0.962648047
Trmt10c	0.097530427	0.940431936
Trmt11	0.071368384	0.962648047
Trmt112	0.196769836	0.610726714
Trmt112-ps1	0.850779405	0.89260868
Trmt112-ps2	0.337046619	0.517996718
Trmt12	0.137778107	0.852010446
Trmt13	-0.008547858	0.99527876
Trmt1l	-0.015232178	0.989200732
Trmt2a	0.093058919	0.935897306
Trmt2b	-0.16829787	0.90458554
Trmt44	-0.099115591	0.959276014
Trmt5	0.022743119	0.985742667
Trmt6	-0.229240942	0.851875536
Trmt61a	0.091439329	0.962648047
Trmt61b	0.068033659	0.980339705
Trmt9b	0.05737051	0.984328758
Trmu	0.12005951	0.887135194
Trnau1ap	-0.194887385	0.770448741
Trnp1	0.031482081	0.969991625
Trnt1	-0.037794167	0.974823941
Tro	-0.354747043	0.922999286
Troap	0.241299898	0.976978183

Trove2	-0.148916101	0.721943953
Trp53	-0.004506821	0.99527876
Trp53bp1	0.017858455	0.985293518
Arhgef28	-0.157646339	0.383515294
Trp53cor1	-0.045269756	0.99527876
Trp53i11	0.434209662	0.868415875
Trp53i13	0.018592541	0.994960308
Ptpn4	-0.157622203	0.486807247
Trp53inp2	-0.01012021	0.99100709
Trp53rka	-0.01900997	0.991438757
Trp53rkb	-0.27866985	0.629280869
Trp53tg5	-0.545974277	0.950195611
Trp63	-0.136219283	0.965520236
Trp73	0.109834085	0.959659722
Trpa1	0.533730082	0.889972515
Trpc1	-0.065761088	0.920642026
Trpc3	0.148720997	0.928957615
Trpc4	-0.051383281	0.962648047
Trpc4ap	-0.02800934	0.970673196
Btf3	0.157507623	0.370298654
Trpc6	0.126979376	0.932673884
Trpc7	0.547294802	0.526260058
Trpm1	0.353583068	0.990915306
St8sia1	-0.157005169	0.509250303
Trpm3	0.007502566	0.99527876
Trpm4	-0.244218017	0.786324313
Trpm5	-0.97451538	0.858106541
Trpm6	-0.037082549	0.99527876
Trpm7	-0.057346777	0.980339705
Trps1	-0.397085576	0.911631946
Trpt1	0.09821641	0.936031628
Sgtb	-0.156840876	0.232043388
Trpv3	0.137467405	0.97469529
Trpv4	-0.281586502	0.939060335
Trpv6	-0.161861702	0.962648047
Trrap	-0.07344884	0.855344864
Trub1	0.025486598	0.979459279
Trub2	-0.016423495	0.98612132
Tsacc	-0.087688768	0.984328758
Tsc1	-0.033017358	0.968977192
Tsc2	-0.080301536	0.789833208
Rpsa	0.156590509	0.146214787
Tsc22d2	-0.037715842	0.970461999
Tsc22d3	-0.008793096	0.99765039

Minos1	0.156270296	0.44386328
Tsen15	0.002042939	0.99765039
Tsen2	-0.167152247	0.630703567
Tsen34	0.023272398	0.985673938
Tsen54	0.104505	0.965290366
Tsfm	0.05887949	0.969476616
Tsg101	0.035669357	0.965290366
Tsg101-ps	0.049166728	0.985293518
Tsga10	-0.040862014	0.98340069
Acox2	-2.560077404	0.623223056
Tshb	-0.016735795	0.997115148
Tshr	-0.579205197	0.959659722
Tshz1	-0.138140079	0.903441375
Tshz2	0.639864271	0.857611652
Tshz3	0.150571579	0.948407374
Lrp8os2	-2.581360706	0.838183693
Tsks	-0.08390285	0.994960308
Tsku	-0.538480573	0.781249046
Agfg1	-0.155240323	0.48945043
Tsn	0.071337919	0.861366392
Tsnax	0.068856216	0.857894889
Tsnaxip1	0.112170531	0.98623785
Tspan11	-0.204425095	0.983166509
Tspan12	-0.133473124	0.879401677
Tspan13	-0.160472986	0.632140484
Tspan14	0.369980114	0.944753667
Tspan15	0.021848065	0.985673938
Tspan17	0.045044074	0.971661054
Slc17a7	-0.15471993	0.420373478
Tspan2	-0.008597507	0.99527876
Tspan3	-0.038433576	0.971138085
Tspan31	-0.117670935	0.675634247
Tspan32	0.295576341	0.98459388
Tspan32os	0.238117562	0.976978183
Tspan33	0.122757404	0.853090538
Tspan4	0.199904531	0.779231312
Tspan5	-0.045392217	0.957485565
Tspan6	-0.019431834	0.989200732
Tspan7	0.086893823	0.664858171
Tmem51os1	-2.624106464	0.655744783
Tspan9	0.109962014	0.915972649
Tspo	0.234711722	0.928957615
Snora19	-2.636952649	NA
Tspoap1	-0.014017297	0.989200732

Tspyl1	0.007159839	0.994960308
Snx30	-0.154505753	0.338465684
Tspyl3	-0.015819934	0.985742667
Tspyl4	0.003102652	0.99527876
Tspyl5	-0.108048473	0.703372057
Tsr1	0.050729026	0.948104382
Tsr2	0.048550058	0.939553162
Tsr3	0.096800697	0.899097089
Tssc4	0.027352673	0.985742667
Tssk1	0.123265647	0.987342614
Tssk2	0.660686562	0.98459388
Tssk4	0.55415482	0.861366392
Tssk6	0.499514595	0.911631946
Tst	-0.032335365	0.980419928
Tsta3	0.168678211	0.675809528
Tstd1	0.484533314	0.921107945
Tstd2	-0.107118218	0.874971335
Tstd3	-0.060063549	0.962648047
Tsx	-0.888046725	0.947033215
Ttbk1	-0.090776643	0.874252042
Ttbk2	-0.362490642	0.980339705
Ttc1	0.156500106	0.513797506
Ttc12	0.012074201	0.99527876
Ttc13	0.000406134	0.999242708
Ttc14	-0.086083066	0.980339705
Ttc16	-0.355475779	0.922999286
Ttc17	0.02042949	0.983219084
Ttc19	-0.045912668	0.949607377
Ttc21a	-0.521621534	0.83886846
Ttc21b	0.048985396	0.97487264
Ttc22	-0.031050147	0.99527876
Ttc23	-0.233444145	0.868797028
Ttc25	-0.038879228	0.993380259
Ttc26	-0.027373447	0.984328758
Ttc27	-0.074675281	0.92517502
Ttc28	0.068165116	0.969755848
Rapgef3os1	-2.649117172	0.871869598
Ttc3	-0.164922098	0.638785294
Ttc30a1	-0.075459317	0.968005385
Ttc30a2	0.268893166	0.92355149
Ttc30b	-0.017719431	0.985742667
Ttc32	-0.11776924	0.962648047
Ttc33	0.053859041	0.965520236
Ttc34	-0.243176555	0.858549868

Ttc37	-0.103351005	0.860521845
Ttc38	0.13681974	0.874422885
Ttc39a	0.322793895	0.911631946
Ttc39aos1	0.332316593	0.947981239
Ttc39b	-0.169061725	0.56488584
Gpc5	0.154088423	0.483668464
Oprm1	-2.681619233	0.66262844
Ttc4	0.230839478	0.795137348
Ttc41	-0.174227797	0.965520236
Ttc5	0.091113835	0.837755777
AC098838.1	-2.722092499	0.792641681
Ttc7	-0.095653986	0.962648047
Ttc7b	-0.073280367	0.891352425
Ttc8	-0.074646521	0.920642026
Ttc9	-0.156331078	0.648241111
Ttc9b	0.016156406	0.988208387
Ttc9c	0.001745626	0.997676331
Ttf1	-0.057334279	0.963943597
Ttf2	-0.050495592	0.985742667
Tti1	0.044647265	0.980226283
Tti2	-0.007096864	0.99527876
Ttk	0.991669056	0.70724751
Ttl	-0.052420524	0.937924083
Ttll1	0.02329359	0.98526072
Ttll10	-0.211278116	0.989840188
Ttll11	-0.15761844	0.705578481
Ttll12	-0.167984886	0.813599082
Ttll13	0.062694785	0.989378233
Ttll3	-0.237995895	0.610726714
Ttll4	-0.579743707	0.835806351
Ttll5	-0.12661541	0.858549868
Ttll6	-0.231934046	0.969755848
Ttll7	0.373089424	0.964206509
Ttll8	0.285693756	0.889972515
Ttll9	0.203203353	0.971138085
AU022793	-2.754712266	0.822597041
Ttpa	0.12943041	0.962648047
Ttpal	0.187725297	0.852010446
Ttr	0.885113052	0.873797321
Nop53	0.15387503	0.309076191
Ttyh2	0.082442629	0.937607785
Ttyh3	0.038040738	0.965520236
Tub	-0.079542479	0.742849809
Tuba1a	0.020455923	0.980226283

Tuba1b	0.032738297	0.965520236
Tuba1c	0.02956836	0.969111286
Tuba3a	0.495565749	0.985742667
Tuba4a	0.196101343	0.815987743
Dpp8	-0.153845987	0.389731767
Tubb2a	-0.02120287	0.97487264
Tubb2a-ps2	0.334352715	0.965520236
Tubb2b	0.049588515	0.921311891
Tubb3	0.032173976	0.973100313
Tubb4a	-0.033818358	0.968005385
Tubb4b	0.038166389	0.962648047
Tubb4b-ps1	0.207331605	0.886647768
Tubb4b-ps2	0.332637623	0.987342614
Tubb5	0.008101696	0.99527876
Tubb6	0.0212419	0.993380259
Tubd1	0.078155288	0.966848493
Tube1	0.0178181	0.99527876
Tubg1	0.019179665	0.985673938
Pex14	0.153805984	0.41394684
Tubgcp2	0.130004483	0.74428322
Tubgcp3	-0.060638468	0.920193202
Tubgcp4	-0.098987809	0.860521845
Tubgcp5	-0.035043464	0.980226283
Tubgcp6	-0.073540737	0.883333293
Tufm	-0.064850019	0.961591202
Tuft1	0.242442729	0.869316117
Tug1	-0.045445643	0.965520236
Oit3	-2.78064214	0.627261794
Tulp3	0.094060811	0.869133292
Tulp4	-0.109083199	0.754350047
Tunar	0.049099801	0.962648047
Tusc1	-0.014254295	0.99527876
Tusc2	0.026729995	0.975831674
Tusc3	0.045001865	0.931745318
Tut1	0.123089658	0.736367995
Tut4	-0.009691562	0.99527876
Tut7	-0.063086637	0.911631946
Tvp23a	-0.032799767	0.971138085
Tvp23b	0.138763838	0.852010446
Tvp23bos	-0.79835105	0.921311891
Rabgap1l	-0.153764312	0.49943116
Twf2	0.013616879	0.99100709
Twist1	-0.398249202	0.873797321
Twist2	-0.128483524	0.97487264

Twistnb	0.031431199	0.98340069
Twnk	-0.136035316	0.833044598
Twsg1	-0.04563849	0.965255389
Txlna	-0.083271074	0.872352604
Cfl1	0.153425783	0.456330081
Txlng	-0.036112365	0.980339705
Eif4a-ps4	0.153408828	0.147036924
Txn2	0.031844943	0.984328758
Txndc11	-0.040858321	0.962648047
Txndc12	0.018081327	0.985673938
Txndc15	0.026533192	0.975800973
Txndc16	-0.098763525	0.800597905
Txndc17	0.122133926	0.839628543
Fam98b	0.153385251	0.389453327
Txndc5	-0.00964875	0.991438757
Txndc9	0.070925849	0.947745582
Txnip	0.256415781	0.962648047
Txnl1	0.0192646	0.981694523
Txnl4a	-0.029059582	0.994960308
Magi2	-0.153306463	0.241783095
Txnrd1	-0.005588602	0.99527876
Txnrd2	0.145038371	0.852010446
Txnrd3	0.068403932	0.969476616
Tyk2	0.122735212	0.852010446
Tymp	0.093188065	0.966010627
Tyms	0.269451647	0.862094492
Tyro3	-0.178915432	0.556319214
Tyrobp	0.035024478	0.975800973
Tyrp1	-0.77223041	0.871869598
Tysnd1	-0.152749422	0.861366392
Tyw1	-0.008837615	0.99527876
Tyw3	-0.019524903	0.987326705
Tyw5	0.018647771	0.993103295
Cacna2d3	-0.152971852	0.443312385
Shisa7	-0.152459473	0.497972882
U2af2	0.147772488	0.852010446
U2surp	0.069133506	0.89011487
Sult6b1	-2.828146174	0.810908327
Uap1	0.043312267	0.969755848
Uap1l1	-0.049405102	0.97469529
Uba1	0.059380342	0.880939938
Uba1y	0.363670505	0.962648047
Uba2	0.025846554	0.97487264
Uba3	0.050302367	0.961607556

Uba5	0.004421171	0.99527876
Uba52	0.149239073	0.836720624
Uba52-ps	0.237570581	0.732729469
Tppp	-0.152136768	0.259438027
Uba7	-0.59235092	0.580757434
Ubac1	-0.034981877	0.97500491
Ubac2	0.011156764	0.989840188
Ubald1	-0.027134837	0.97487264
Ubald2	0.246330151	0.518646722
Ubap1	-0.004373789	0.997529192
Ubap1l	-0.217814384	0.945182261
Ubap2	-0.112367186	0.76998058
Ubap2l	-0.034640044	0.962648047
Ubash3a	0.228858914	0.962648047
Ubash3b	-0.018867109	0.98526072
Zyg11b	-0.151906141	0.41745316
Ubc	0.052549396	0.89703631
Ube2a	0.326275213	0.591119873
Ube2b	0.0344336	0.959276014
Ube2c	0.46662608	0.91142247
Ube2cbp	-0.45714388	0.813552442
Ube2d-ps	0.089522136	0.91462326
Ube2d1	0.090445308	0.852010446
Ube2d2a	0.062255907	0.871869598
Ube2d3	0.052603928	0.968005385
Ube2e1	0.104924719	0.875995577
Ube2e2	-0.030986701	0.97469529
Ube2e3	-0.044974423	0.952385926
Ube2f	-0.076272353	0.873797321
Ube2g1	-0.010458828	0.991438757
Ube2g2	0.295784419	0.727452809
Ube2h	-0.05306286	0.938615214
Ube2i	0.047883015	0.947421926
Ube2j1	-0.03218522	0.957499962
Ube2j2	-0.044027403	0.962648047
Ube2k	0.079892735	0.837755777
Ube2l3	-0.394306401	0.945647427
Ube2l6	0.189902421	0.898474424
Pdxk	0.151835368	0.332533586
Ube2n	-0.053033973	0.89383516
Ube2n-ps1	0.117871013	0.849517436
Dennd5b	-0.151049446	0.459780215
Ube2o	-0.061176403	0.869437456
Ube2q1	0.026788562	0.971138085

Ube2q2	-0.032181777	0.968593828
Ube2ql1	-0.031407321	0.966312931
Ube2r2	-0.028115099	0.97442139
Ube2s	0.024169765	0.991996237
Ube2t	0.446775792	0.86128643
Ube2v1	-0.374263572	0.968630572
Ube2v2	-0.045218202	0.948407374
Ube2w	0.025966275	0.975831674
Ube2z	0.067620148	0.839488666
Ube3a	-0.149981655	0.991996237
Ube3b	-0.06854903	0.852010446
Ube3c	-0.05188044	0.930336691
Ube4a	-0.136354796	0.620124996
Ube4b	-0.061320611	0.873797321
Cyp19a1	-2.847879222	0.84614651
Ube4bos3	-0.503276097	0.810908327
Ubfd1	-0.13416187	0.638858785
Ubiad1	-0.026196015	0.985742667
Ubl3	-0.205128563	0.942812446
Ubl4a	0.008163014	0.994960308
Pabpn1	0.150933103	0.445106721
Ubl7	0.057714263	0.932673884
Sin3a	-0.150882982	0.403784647
Ubn1	0.023828099	0.97932602
Ubn2	-0.039977716	0.962648047
Ubox5	0.085735586	0.89703631
Ubp1	0.035733359	0.968005385
Ubqln1	0.024596503	0.970929501
Ubqln2	-0.056626949	0.872265039
Ubqln4	-0.044825385	0.948104382
Ubr1	-0.05612885	0.903441375
Ubr2	-0.081915655	0.858393367
Ubr3	-0.128157574	0.527779763
Ubr4	-0.147379677	0.544661714
Ubr5	-0.084786591	0.813552442
Ubr7	-0.027339086	0.975831674
Ubtd1	-0.09396401	0.936507368
Ubtd2	-0.151975067	0.861366392
Ubtf	-0.002048441	0.99765039
Ubxn1	0.052795265	0.911631946
Ubxn10	0.426937153	0.736486324
Ubxn11	0.055904691	0.975831674
Ubxn2a	-0.111403816	0.87847909
Ubxn2b	-0.137101162	0.813552442

Ubxn4	-0.059558354	0.921661632
Ubxn6	0.03892497	0.962648047
Ubxn7	0.020295111	0.980132658
Ubxn8	-0.072750031	0.911834483
Uchl1	0.066202777	0.855344864
Uchl1os	-0.061943448	0.991996237
Uchl3	0.027397489	0.976978183
Uchl4	0.974793722	0.953505913
Uchl5	-0.074800607	0.939592821
Uck1	0.020560574	0.984748462
Uck2	-0.093473744	0.8549794
Uckl1	0.189688276	0.544661714
Uckl1os	-0.823523495	0.854434649
Ucma	0.219015547	0.920642026
Ucn2	-0.849436628	0.975831674
Ucp2	0.191502625	0.8549794
Ucp3	0.171385728	0.934798735
Uevld	-0.024473789	0.984328758
Usp9x	-0.150824466	0.306980292
Zmym4	-0.150579707	0.404892636
Ufl1	-0.020546827	0.984328758
Ufm1	0.014869241	0.985742667
Ufsp1	0.026963477	0.989200732
Rab3c	-0.150496473	0.461007377
Ugcg	-0.098922939	0.729712198
Ugdh	-0.098367341	0.915297582
Uggt1	-0.09536953	0.792517766
Uggt2	0.033030062	0.975831674
Ugp2	-0.038563662	0.968005385
Ugt1a6a	0.489923854	0.663393433
Ugt1a6b	0.935093559	0.87847909
Ugt1a7c	-0.699897675	0.807017299
Sept5	0.150328201	0.396025513
Unc13a	-0.150125855	0.370478684
Uhrf1	0.205581566	0.965520236
Uhrf1bp1	0.049385931	0.962648047
Orai2	0.14977248	0.394955649
Uhrf2	-0.048683413	0.944172695
Uimc1	0.152259052	0.520182905
Snord57	-2.869753773	0.578812716
Ulk1	0.003746409	0.99527876
Ulk2	-0.100693866	0.759486652
Ulk3	0.017269428	0.985742667
Ulk4	0.108591771	0.934798735

Umad1	-0.103402359	0.855344864
Umodl1	-0.225762345	0.889972515
Umps	0.018464926	0.985673938
Unc119	0.048997766	0.97469529
Unc119b	-0.052513402	0.950604431
Suclg1	0.14953823	0.404892636
Agrn	-0.149450798	0.442302609
Unc13c	-0.02183281	0.99527876
Unc13d	0.198834944	0.952390496
Unc45a	-0.011129358	0.991996237
Unc45b	0.045710197	0.99527876
Unc50	-0.031563864	0.980144634
Unc5a	-0.103115171	0.689698377
Unc5b	-0.165169243	0.8549794
Unc5c	0.034993062	0.974962198
Unc5cl	-0.984317982	0.921311891
Unc5d	-0.116689797	0.986246396
Unc79	-0.121462704	0.736130784
Unc80	-0.137304749	0.566580926
Unc93a	-0.615186023	NA
Unc93b1	-0.145140287	0.851875536
Ung	-0.00108271	0.999242708
Unk	0.090042917	0.922304268
Unkl	-0.165928688	0.974823941
Uox	0.059708276	0.991996237
Upb1	-0.053731607	0.99527876
Upf1	-0.040283457	0.965332265
Upf2	0.012742529	0.988208387
Upf3a	0.107764902	0.871869598
Upf3b	0.062045464	0.962648047
Upk1a	-0.699820346	0.8549794
Upk1b	-0.174553473	0.962648047
Upp1	0.23288953	0.890553353
Upp2	-0.056495296	0.939904081
Uprt	-0.279072156	0.903795193
Uqcc1	0.332092076	0.703633347
Uqcc2	0.164082803	0.673443553
Arhgef11	-0.149057553	0.463966612
Uqcr10	0.174136595	0.6883862
Uqcr11	0.166336622	0.779231312
Herc3	-0.14867483	0.235039388
Uqcrc1	0.131130497	0.544661714
Uqcrc2	0.027796273	0.960201673
Uqcrfs1	-0.006495691	0.994960308

Arl6ip5	0.148621667	0.319241271
Uqcrh-ps2	0.54725562	0.971138085
Src	-0.148439381	0.407983695
Urah	-0.28755489	0.965520236
Urb1	-0.119223273	0.859870227
Urb2	-0.245627557	0.544661714
Urgcp	0.049799924	0.969755848
Uri1	0.240182424	0.533113207
Urm1	0.034609189	0.97487264
Urod	0.197462856	0.668010503
Uros	-0.141922898	0.874178441
Usb1	0.009713962	0.99527876
Use1	0.167152019	0.655744783
Usf1	0.009056039	0.994960308
Map1lc3b	0.148433146	0.414106926
Usf2-ps1	0.239102538	0.837755777
Usf3	-0.587664118	0.962648047
Ush1g	0.189816714	0.971661054
Ush2a	-0.686042514	0.980896205
Ushbp1	-0.06429254	0.978384252
Pom121	-0.148377542	0.481689886
Uso1	-0.034641866	0.96675665
Usp1	0.163179307	0.859910936
Usp10	-0.101072544	0.8549794
Usp11	-0.007229792	0.991438757
Usp12	-0.004641849	0.99527876
Usp13	-0.40973072	0.928672524
Usp14	-0.022020821	0.975800973
Usp15	-0.109620779	0.798109023
Usp16	0.014238347	0.988298347
Usp18	0.240100632	0.962648047
Appbp2	-0.148006973	0.458609802
Usp2	0.14779668	0.86467336
Usp20	-0.122614961	0.511951611
Usp21	-0.139036316	0.911631946
Usp22	-0.000427253	0.998658264
Usp24	-0.065383977	0.905585111
Usp25	-0.148162639	0.595941204
Slc22a2	-2.874031319	NA
Usp28	-0.134950679	0.659663162
Usp29	-0.471030486	0.713014362
Usp3	0.053825677	0.962812066
Usp30	0.136585933	0.66923652
Dagla	-0.147697127	0.341690144

Usp32	-0.101650658	0.852035098
Usp33	-0.133573804	0.690084266
Usp34	-0.138377128	0.638329646
Usp35	-0.027958983	0.984748462
Usp36	-0.009698517	0.994806961
Usp37	-0.143352615	0.891972665
Usp38	-0.099597164	0.833916587
Usp39	0.073146873	0.932673884
Usp4	0.018300011	0.976978183
Usp40	0.008376785	0.99527876
Usp42	-0.02315842	0.987453225
Setd7	-0.147674786	0.234819833
Usp45	0.150227836	0.938939871
Tango2	0.147204862	0.436138179
Slc18a1	-2.882981842	0.56488584
Usp47	-0.039870739	0.968630572
Gpd1	0.147199444	0.31865776
Usp49	-0.227461487	0.974823941
Usp5	0.02476324	0.965520236
Arfgef3	-0.146931791	0.440528706
Usp51	-0.177079472	0.975800973
Usp53	-0.139965905	0.800386557
Usp54	0.180418814	0.542810942
Usp6nl	0.03565069	0.974823941
Usp7	0.05850975	0.901534588
Usp8	-0.073277398	0.855344864
Sorl1	-0.146659073	0.278279958
Uspl1	0.071041462	0.946239011
Ust	0.872390156	0.774804043
Utp11	0.089860098	0.911631946
Utp14a	-0.019257134	0.989402966
Utp14b	-0.10699685	0.962648047
Utp15	-0.002618802	0.997115148
Utp18	-0.023712733	0.984232465
Rere	-0.146495033	0.355109377
Utp23	-0.003696199	0.997115148
Utp3	0.038294712	0.966213117
Utp4	0.083394675	0.860142574
Utp6	0.042182568	0.961373288
Utrn	-0.070794786	0.932673884
Uts2	0.323462049	0.984328758
Eid3	-2.929256955	0.544661714
Uty	0.108155348	0.945647427
Uvrag	0.001822825	0.997529192

Uvssa	-0.106533216	0.944074069
Uxs1	-0.099259734	0.959276014
Uxt	0.132937103	0.928957615
Vac14	0.034572574	0.980626239
Vamp1	-0.033249217	0.98526072
Vamp2	0.030410179	0.964011524
Vamp3	0.116366316	0.849381566
Vamp4	-0.072838779	0.962648047
Vamp5	-0.396174051	0.80931022
Vamp7-ps	0.279347721	0.911631946
Vamp8	0.163425315	0.841052157
Vamp9	-0.428311343	0.962648047
Vangl1	-0.259814742	0.956164302
Vangl2	0.043547867	0.981392364
Gon4l	-0.146047342	0.459482348
Vapb	0.028936827	0.962648047
Vars	0.070688801	0.912042382
Vars2	-0.12118875	0.858549868
Vash1	-0.286490816	0.548869952
Vash2	-0.018000885	0.99527876
Vasn	-0.059361426	0.975800973
Vasp	0.033741812	0.976546786
Vat1	0.029268639	0.975800973
Vat1l	0.368545296	0.59684147
Vav1	0.279881783	0.810246066
Vav2	0.067812008	0.948104382
Vav3	0.086923414	0.954770762
Vax1	-0.052797237	0.99527876
Vax2os	0.458376759	0.984101725
Vbp1	-0.112647994	0.736130784
Vcam1	-0.066476297	0.968005385
Vcan	-0.336238385	0.549579107
Vcl	-0.136444922	0.837755777
Vcp	0.005656496	0.992654978
Vcp-rs	0.031372654	0.971138085
Vcpip1	-0.121929386	0.744309911
Vcpkmt	0.122060513	0.905251009
Vdac1	0.077053828	0.723108545
Vdac2	0.031317391	0.964011524
Vdac3	0.056379367	0.879401677
Vdac3-ps1	0.164063064	0.788592804
Vdr	-0.874208778	0.867041127
Vegfa	-0.160666865	0.814814675
Vegfb	0.07322181	0.895520088

Vegfc	-0.516685136	0.534684113
Vegfd	-0.313376655	0.861909929
Hsd17b2	-2.935222714	0.544661714
Vezf1	-0.13687379	0.709244129
Vezt	0.009402137	0.991438757
Vgf	-0.656509305	0.56488584
Vgll2	-0.783323524	0.968005385
Tnk1	-2.949381838	0.715121059
Vgll4	-0.12995259	0.89703631
Vhl	0.316793736	0.627170228
Nxf2	-2.967115528	0.544661714
Rab25	-2.972255303	0.819778986
Vim	-0.055838298	0.968630572
Vip	-0.14097952	0.79199083
Vipas39	0.075858495	0.815987743
Vipr1	-0.097460007	0.883333293
Vipr2	-0.032655392	0.99527876
Virma	-0.090846254	0.874971335
Vis1	-0.987900865	0.936031628
Vit	-0.217050442	0.623117533
Vkorc1	-0.138720292	0.891972665
Vkorc1l1	-0.109720814	0.980132658
Vldlr	-0.159158425	0.621993647
Vma21	-0.098423719	0.994960308
Vmac	0.180262257	0.808459207
Psmb1	0.145477787	0.403453477
Vmn1r4	0.227273671	0.985742667
Vmn1r51	-0.50610238	0.985673938
F2rl1	-3.019844869	0.5542082
Vmn1r90	-0.269870461	0.99527876
Mir27b	-3.100095059	0.544661714
Vmn2r-ps88	0.572990195	0.973100313
Mir1900	-3.101441708	0.604176395
Vmn2r1	0.235112828	0.932673884
Arid3c	-3.10338423	0.787744512
Vmn2r29	0.376234132	0.965520236
Vmn2r30	-0.088567147	0.99527876
Vmn2r57	0.021983981	0.998010523
Vmn2r84	0.144287596	0.97041548
Mir7237	-3.103698995	NA
Vmn2r86	-0.934162791	0.974823941
Vmn2r87	-0.600869631	0.8549794
Vmp1	-0.049341767	0.962648047
Mir5133	-3.1039278	NA

Vopp1	-0.013729126	0.985446046
Vpreb1	0.624964792	0.980144634
Rps2-ps5	-3.193574815	0.647235427
Vps11	0.06596154	0.932673884
Vps13a	0.083886421	0.919785445
Ubb	0.145341203	0.39089542
Ptpn5	-0.145314863	0.484867133
Vps13d	-0.09992546	0.833916587
Vps16	0.059539751	0.911631946
Vps18	-0.110013551	0.852010446
Vps25	0.148194752	0.951390592
Vps26a	0.042943792	0.944098708
Vps26b	-0.016974637	0.981695356
Vps26c	0.033864092	0.969755848
Vps28	0.052951511	0.920642026
Vps29	0.018899945	0.984328758
Vps33a	-0.018748595	0.981045362
Vps33b	0.003109829	0.997001768
Vps35	-0.056051144	0.911631946
Vps35l	0.061616767	0.89703631
Vps36	-0.075693973	0.912042382
Vps37a	-0.00286174	0.997115148
Vps37b	0.099247136	0.84870301
Vps37c	-0.077633206	0.89703631
Vps37d	0.050352643	0.97487264
Vps39	0.148652461	0.866574046
Vps41	-0.067441872	0.846622565
Vps45	0.093824509	0.855344864
Vps4a	0.056190519	0.910524966
Vps4b	0.187770681	0.745988863
Vps50	-0.015450711	0.985293518
Vps51	-0.441161926	0.615775357
Vps52	-0.073091362	0.873753967
Vps53	-0.002853345	0.996920836
Vps54	-0.156965353	0.742969797
Vps72	0.079065041	0.91462326
Vps8	0.022886216	0.976978183
Vps9d1	0.073559697	0.90358675
Chrm1	-0.14461119	0.425470982
Vrk2	0.118766403	0.971795434
Vrk3	0.025822408	0.981144704
Vsig1	0.094798195	0.99527876
Vsig10	-0.012275381	0.996094752
Vsig10l	0.170489169	0.966213117

Vsig2	0.23628801	0.80993221
Vsig8	-0.614805406	0.968005385
Vsir	0.149633571	0.783229859
Vsnl1	-0.026639058	0.971661054
Vstm2a	-0.115111078	0.852010446
Vstm2b	-0.013991854	0.99527876
Vstm2l	-0.184353517	0.80931022
Vstm4	-0.15599526	0.965520236
Vstm5	-0.041087504	0.980144634
Vsx1	-0.712458465	0.954827342
Vsx2	0.17569582	0.975831674
Vta1	0.083831039	0.878119351
Vti1a	-0.024954042	0.974962198
Vti1b	-0.000727426	0.998840551
Vtn	-0.16625418	0.859910936
Rpl27a	0.144598218	0.482385306
Vwa3a	0.109215178	0.935897306
Vwa3b	0.086932046	0.955993235
Vwa5a	0.05088068	0.978384252
Vwa5b1	0.207277425	0.908413609
Vwa5b2	0.087522017	0.951949091
Hectd4	-0.144240795	0.387432606
Vwa8	0.077248391	0.874252042
Vwc2	-0.056786693	0.980896205
Vwc2l	0.691512817	0.6883862
Vwce	-0.008786473	0.998010523
Vwf	-0.288152138	0.871869598
Rpl19-ps11	0.144198562	0.456330081
Wac	0.070752142	0.962648047
Wapl	-0.09594503	0.860521845
Wars	0.183811766	0.962648047
Wars2	-0.974975651	0.770793864
Was	0.261763659	0.91507125
Wasf1	-0.079235474	0.882096017
Wasf2	0.145500091	0.724910334
Wasf3	0.018386458	0.975831674
Washc1	0.11936503	0.80818949
Washc2	-0.059451289	0.89703631
Tpt1	0.143903522	0.309077406
Washc4	-0.088020087	0.928672524
Washc5	-0.105376471	0.714817503
Wasl	-0.133796573	0.654893207
Wbp1	0.119007937	0.895520088
Wbp11	-0.003784286	0.99527876

Wbp1l	0.008954643	0.993489613
Wbp2	0.087965035	0.732729469
Wbp4	-0.258801765	0.745988863
Wbscr25	-0.409620547	0.956851175
Wdcp	-0.072383731	0.962648047
Wdfy1	0.040717935	0.962648047
Wdfy2	0.014445364	0.985742667
Wdfy3	-0.11852769	0.775455132
Wdfy4	-0.050086073	0.986246396
Wdhd1	0.281972529	0.683281689
Wdpcp	-0.035076636	0.984328758
Wdr1	0.031375823	0.956164302
Wdr11	-0.031353979	0.965520236
Wdr12	0.090678021	0.872352604
Wdr13	-0.06503923	0.968005385
Wdr17	-0.138395404	0.874971335
Wdr18	0.005531881	0.99527876
Wdr19	-0.081129139	0.938673121
Wdr20	-0.080700455	0.911631946
Wdr24	0.068397089	0.928333994
Wdr25	0.277025116	0.6883862
Wdr26	0.030715794	0.974197921
Wdr27	0.393544631	0.807017299
Wdr3	-0.072649435	0.898474424
Wdr31	0.050988373	0.977133618
Wdr33	-0.071321513	0.861397728
Wdr34	-0.056076018	0.962648047
Wdr35	-0.074457111	0.882824434
Wdr36	-0.01184377	0.993380259
Wdr37	-0.048069023	0.933241478
Wdr38	0.178597533	0.97487264
Wdr4	0.089128537	0.975800973
Wdr41	-0.021192462	0.978063275
Wdr43	0.074497834	0.912042382
Wdr44	-0.14405194	0.687703291
Wdr45	-0.056261908	0.974823941
Wdr45b	0.041272221	0.953505913
Rpl11	0.143630696	0.498239628
Wdr47	-0.015171398	0.980132658
Wdr48	-0.09199941	0.688916428
Wdr49	0.011871012	0.998350011
Wdr5	-0.018984636	0.985742667
Wdr53	-0.098083265	0.948104382
Akap6	-0.143462233	0.465869831

Wdr55	-0.001864893	0.998010523
Wdr59	-0.005891109	0.99527876
Wdr5b	0.194878103	0.80931022
Wdr6	0.097941052	0.822849224
Wdr60	-0.101765522	0.854278008
Wdr61	0.137072089	0.758223648
Wdr62	0.258549579	0.90358675
Wdr63	-0.256588688	0.965520236
Wdr66	-0.068227752	0.976116764
Rpl13a	0.142879229	0.310553098
Wdr70	0.005743427	0.99527876
Efnb3	0.142818666	0.503067237
Wdr73	0.21192109	0.688142448
Wdr74	-0.299821918	0.965520236
Wdr75	0.010098542	0.994960308
Wdr76	0.147247069	0.962648047
Wdr77	-0.003314077	0.997529192
Wdr78	-0.236576231	0.819778986
Wdr81	-0.155751438	0.981045362
Wdr82	0.013512258	0.983219084
Wdr83	0.10136382	0.882096017
Wdr83os	0.173647351	0.625886453
Wdr86	0.077151512	0.994960308
Wdr89	0.263222982	0.707520358
Wdr90	-0.125622035	0.911631946
Wdr91	-0.125694748	0.74632237
Wdr92	-0.173620051	0.815662897
Oas3	-3.435566093	0.644239557
Wdr95	-0.26003846	0.976530608
Wdsub1	-0.005534494	0.99527876
Wdtc1	-0.06740571	0.874422885
Wdyhv1	0.072276214	0.965290366
Wee1	-0.006104276	0.99527876
Wee2	0.069123557	0.997258205
Wfdc1	0.172749983	0.915297582
Usf2	0.142659152	0.491912953
Wfdc18	0.6894605	0.869316117
Wfdc2	-0.14901287	0.992385525
Wfdc3	-0.085486527	0.985742667
Wfikkn1	-0.365771091	0.966010627
AC166164.1	-3.508435789	0.653967958
Psmb7	0.141875646	0.285037596
Whamm	0.103936074	0.861366392
Whrn	0.155151001	0.84870301

Wif1	0.17558029	0.972218452
Wipf1	-0.07296276	0.96460487
Wipf2	-0.02748424	0.97487264
Wipf3	-0.059457969	0.957499962
Wipi1	-0.005670384	0.995411429
Wipi2	0.032800615	0.956922504
Wisp1	0.5741919	0.91142247
Abhd16b	-3.82980408	0.695239733
Wisp3	-0.985505209	0.921311891
Wiz	-0.190652368	0.619798471
Wls	-0.140719819	0.87847909
Wnk1	-0.133039924	0.779231312
Wnk2	-0.044659146	0.962648047
Wnk3	-0.120216042	0.902164359
Wnk4	-0.016022328	0.99527876
Wnt1	-0.233503902	0.982119785
Wnt10a	-0.176775579	0.964941885
Wnt10b	0.195020001	0.969755848
Wnt11	-0.077756223	0.98771048
Wnt16	0.286829221	0.965290366
Wnt2	-0.127769454	0.938579664
Wnt2b	-0.205615186	0.91507125
Psma7	0.141661467	0.374179097
Wnt3a	-0.42481504	0.962648047
Wnt4	-0.222346068	0.74463793
Wnt5a	-0.189370378	0.932673884
Wnt5b	-0.282432031	0.875042526
CT009594.1	-3.94564646	NA
Wnt7a	-0.285929255	0.66401455
Wnt7b	-0.09159894	0.948104382
Wnt8b	0.022912098	0.99640101
Wnt9a	-0.023261972	0.989840188
Wnt9b	-0.632494943	0.650504271
Wrap53	0.068909797	0.957485565
Wrap73	0.013093017	0.989840188
Wrb	0.013767212	0.99765039
Wrn	-0.146708318	0.911631946
Wrnip1	0.034157795	0.969755848
Wsb1	-0.061357374	0.89383516
Wsb2	-0.05238802	0.916287981
Wscd1	0.023713673	0.975831674
Arpc1a	0.141021166	0.508471705
Gfral	-3.94564646	NA
Wtap	-0.222603002	0.56399886

Wtip	-0.00925497	0.99527876
Wwc1	-0.033911114	0.965290366
Wwc2	-0.074780212	0.891265024
Wwox	-0.028336236	0.984512582
Lpgat1	-0.140802248	0.323882465
Wwp2	-0.066529854	0.910328122
Wwtr1	-0.147702674	0.837755777
Xab2	0.005432582	0.99527876
Xaf1	0.227012758	0.904480183
Xbp1	-0.138621392	0.510932065
Epm2aip1	-0.140778117	0.353015192
Hcfc1	-0.140649884	0.426899054
Atxn2l	-0.140280964	0.394328457
Vmn2r-ps89	-4.229371488	0.642397758
Klrb1b	-4.360623786	0.589856572
Xist	-0.375098287	0.971138085
Gfpt1	-0.140131397	0.431890949
Xkr4	-0.141012597	0.962648047
Xkr5	0.131901307	0.99527876
Xkr6	-0.38770215	0.89703631
Xkr7	0.517219678	0.864907053
Xkr8	-0.120376774	0.967662387
Xkrx	-0.092296427	0.98459388
Xlr	-0.419729279	0.909547396
Xlr3a	-0.377946137	0.959659722
Xlr3b	0.175592102	0.993380259
Xlr3c	0.676769307	0.966515082
Glrx3	-0.139714793	0.414106926
Xlr4b	0.626466813	0.965520236
Xlr4c	0.068620557	0.996070372
Xndc1	0.068030272	0.972218452
Xntrpc	0.015869	0.99527876
Xpa	-0.029769942	0.985673938
Xpc	-0.063548066	0.948432875
Xpnpep1	0.080248604	0.795890296
Xpnpep2	-0.16080679	0.974962198
Xpnpep3	0.045195383	0.99527876
Xpo1	-0.128658996	0.740240396
Xpo4	-0.149471305	0.758595939
Xpo5	-0.039426036	0.961373288
Xpo6	0.073278662	0.916922462
Xpo7	0.035495681	0.966608583
Xpot	-0.111047613	0.66923652
Xpr1	0.017796068	0.98526072

Xrcc1	0.100247638	0.896986942
Xrcc2	-0.036651807	0.98526072
Xrcc3	-0.011046423	0.99527876
Xrcc4	0.009544157	0.99527876
Xrcc5	-0.028731214	0.984748462
Xrcc6	0.023560921	0.98501783
Xrn1	-0.074684625	0.921311891
Xrn2	-0.031286696	0.971795434
Xrra1	-0.38477307	0.889972515
Xxylt1	0.000321836	0.99930339
Xylb	-0.071597102	0.968005385
Xylt1	0.008292131	0.99527876
Xylt2	0.091006571	0.903441375
Yae1d1	-0.085039608	0.964011524
Yaf2	0.023017052	0.97487264
Yap1	-0.543669968	0.886647768
Yars	-0.018030295	0.980419928
Yars2	-0.160473695	0.813552442
Ybey	0.122096133	0.932030893
Ybx1	0.11653058	0.803910915
Ybx1-ps2	-0.570710871	0.911631946
Ybx2	0.167610285	0.928099251
Ybx3	-0.233313552	0.655744783
Ydjc	0.260406874	0.849424799
Fry	-0.139565286	0.431269992
Edem3	-0.139120516	0.504583609
Yes1	-0.073381592	0.971661054
Yif1a	-0.011880659	0.994960308
Yif1b	0.030542101	0.98340069
Yipf1	0.085328644	0.895520088
Yipf2	0.036090764	0.980339705
Yipf3	-0.088893876	0.839781404
Yipf4	-0.008810759	0.993380259
Yipf5	-0.04409478	0.951817826
Yipf6	-0.108381804	0.890635602
Cxcl14	0.139083137	0.404461151
Yjefn3	0.129616305	0.89703631
Yju2	0.035811736	0.981502501
Ykt6	0.059519175	0.869316117
Ylpm1	-0.099062521	0.824306801
Yme1l1	-0.092976905	0.89703631
Yod1	-0.010699627	0.99527876
Ypel1	-0.007094004	0.998093116
Ypel2	-0.063407633	0.962648047

Dazap2	0.139070356	0.261771263
Ypel4	0.122890283	0.778941315
Ypel5	-0.459247084	0.822597041
Yrdc	-0.101653525	0.89703631
Ythdc1	0.012252785	0.990333976
Ythdc2	-0.120273388	0.852010446
Ythdf1	-0.075389003	0.89703631
Ythdf2	-0.07842034	0.894953269
Ythdf3	-0.122272036	0.942812446
Ywhab	-0.061271521	0.861397728
Ywhae	0.042487236	0.95363833
Ywhag	-0.009496063	0.99100709
Ywhah	0.118123259	0.670364941
Ywhaq	-0.510338668	0.74463793
Mir1897	-4.688182383	NA
Ywhaq-ps3	-0.07598708	0.920642026
Ywhaz	-0.01205341	0.985742667
Yy1	-0.07705334	0.837755777
Nova2	-0.138926598	0.488671938
Cdc42bpa	-0.13892158	0.318279733
Zan	0.234313766	0.985673938
Zap70	-0.019468202	0.993103295
Psd3	-0.138760093	0.319241271
Zbed3	0.114192099	0.891972665
Zbed4	-0.104749178	0.900513955
Zbed5	-0.020246203	0.987342614
Arhgap35	-0.138746184	0.491797671
Zbp1	-0.872308695	0.932956012
Zbtb1	0.035143861	0.975312818
Zbtb10	-0.008200256	0.99527876
Zbtb11	-0.19474108	0.513797506
Zbtb11os1	-0.215072293	0.889972515
Zbtb12	-0.195840765	0.860142574
Zbtb14	-0.005077802	0.99527876
H1f0	0.138580936	0.482385306
Zbtb17	-0.051942644	0.971138085
Zbtb18	-0.324691886	0.873797321
Zbtb2	0.237270421	0.962648047
Usp19	-0.13839608	0.483438211
Zbtb21	-0.001871818	0.998010523
Zbtb22	0.07059129	0.903795193
Zbtb24	-0.032377953	0.985293518
Zbtb25	0.292262536	0.894148925
Zbtb26	-0.104994637	0.962648047

Zbtb3	-0.272973655	0.815987743
Zbtb32	0.302002315	0.897878088
Zbtb33	-0.104210828	0.791185365
Atp1a1	0.138353319	0.260708948
Zbtb37	-0.378388722	0.512082024
Zbtb38	0.015298915	0.99100709
Zbtb39	0.021824951	0.987342614
Zbtb4	-0.052613621	0.947745582
Zbtb40	-0.03506279	0.984328758
Zbtb41	-0.146412667	0.821998633
Zbtb42	-0.122723721	0.985653607
Zbtb43	-0.063980853	0.971540209
Zbtb44	0.708185879	0.956922504
Eif1	0.138234326	0.442068044
Zbtb46	-0.147844779	0.864150749
Zbtb48	0.170518569	0.758595939
Zbtb49	0.213986834	0.813552442
Zbtb5	-0.001223596	0.99900438
Zbtb6	0.066451616	0.949607377
Zbtb7a	0.380600927	0.959659722
Dapk1	0.138190937	0.485743931
Zbtb7c	-0.138045094	0.955993235
Zbtb8a	0.004935251	0.997258205
Zbtb8b	-0.060152168	0.981392364
Zbtb8os	0.136351702	0.945647427
Zer1	-0.137600774	0.306736562
Trip12	-0.137521807	0.465255523
Zc2hc1c	0.16471835	0.960201673
Zc3h10	-0.024717797	0.985742667
Zc3h11a	-0.309870277	0.962648047
Zc3h12a	0.391779296	0.89703631
Zc3h12b	-0.077298428	0.869437456
Zc3h12c	-0.155893922	0.849424799
Zc3h12d	0.946929699	0.969755848
Zc3h13	-0.057563789	0.895520088
Zc3h14	0.013440842	0.987326705
Zc3h15	-0.024999218	0.980419928
Zc3h18	-0.035746078	0.964011524
Syt13	0.137446813	0.404443644
Zc3h4	-0.059582139	0.928672524
Zc3h6	-0.03868179	0.98459388
Zc3h7a	-0.026090724	0.980339705
Zc3h7b	-0.045849047	0.949450803
Zc3h8	-0.005010943	0.997258205

Zc3hav1	-0.098782764	0.962648047
Zc3hav1l	0.136508524	0.859803076
Zc3hc1	0.089048706	0.925788724
Zc4h2	0.012096792	0.991996237
Zcchc10	0.169523878	0.883333293
Zcchc12	0.01740933	0.984748462
Zcchc14	-0.100698295	0.7851601
Zcchc17	0.010168267	0.993103295
Zcchc18	-0.020427714	0.97487264
Camta2	-0.137242108	0.298968315
Zcchc24	0.108975982	0.562289782
Zcchc3	0.192967308	0.774225415
Zcchc4	0.114778639	0.891265024
Zcchc7	-0.144503152	0.795137348
Zcchc8	0.077645914	0.962176581
Zcchc9	0.029858345	0.985742667
Zcrb1	-0.007710612	0.99527876
Zcwpw1	0.074741153	0.968005385
Prkar2a	-0.13681098	0.481098488
Zdbf2	-0.197321328	0.707655454
Zdhhc1	-0.129415599	0.865193014
Zdhhc12	0.084232421	0.965520236
Zdhhc13	-0.027116521	0.975831674
Zdhhc14	0.001412569	0.998010523
Zdhhc15	-0.175877386	0.852010446
Zdhhc16	-0.051952312	0.968005385
Zdhhc17	0.020546036	0.980339705
Zdhhc18	-0.040384263	0.968005385
Pdpk1	-0.135574157	0.350732723
Zdhhc20	-0.031862503	0.971138085
Zdhhc21	0.10785899	0.928672524
Zdhhc22	0.218021492	0.828102037
Acot13	0.133036199	0.435075615
Zdhhc24	0.304696735	0.91507125
Zdhhc3	-0.073582754	0.962648047
Zdhhc4	0.070405062	0.951755452
Zdhhc5	-0.102871448	0.861139061
Zdhhc6	0.040099528	0.97487264
Zdhhc7	0.036471577	0.965520236
Zdhhc8	-0.080261458	0.849381566
Zdhhc9	-0.066491939	0.89703631
Zeb1	-0.01223621	0.99165967
Trim37	-0.132711225	0.41351939
Zeb2os	0.116814286	0.962648047

Evi5	-0.132384378	0.481098488
Pacs1	-0.132373981	0.485432358
Zfand1	-0.003571302	0.997241386
Zfand2a	0.076174272	0.853055342
Zfand2b	0.041560593	0.968497942
Zfand3	0.109762385	0.991967441
Zfand4	-0.05616465	0.991996237
Zfand5	-0.014771512	0.98459388
Zfand6	-0.049836617	0.971138085
Vapa	0.132194576	0.442068044
Zfat	-0.054049866	0.975800973
Zfc3h1	-0.042892604	0.956922504
Iqsec2	-0.131863444	0.202604353
Zfhx2os	-0.315788876	0.825148733
Zfhx3	-0.013183816	0.99527876
Zfhx4	-0.414316654	0.681884782
Zfp1	0.197117289	0.573810203
Zfp101	-0.186304107	0.873797321
Zfp105	0.261330698	0.734581634
Zfp106	-0.138905203	0.5141747
Zfp108	0.032712172	0.985742667
Paip2	0.130892697	0.322635312
Zfp11	0.083919601	0.962648047
Zfp110	0.060049285	0.962648047
Zfp111	0.014870942	0.99527876
Zfp112	0.087250364	0.911631946
Zfp113	0.083012041	0.950252558
Zfp114	0.017490739	0.99527876
Zfp119a	0.182347459	0.909547396
Brk1	0.130693835	0.381457246
Zfp12	-0.101702726	0.784009698
Zfp120	-0.033963877	0.985673938
Zfp125	-0.04557786	0.984328758
Zfp128	-0.057776039	0.965241989
Zfp13	0.130263991	0.861366392
Zfp131	0.166519551	0.855904169
Zfp133-ps	0.160306553	0.942511266
Zfp14	-0.122892676	0.962648047
Zfp141	0.006974835	0.99527876
Zfp142	-0.096483143	0.873797321
Zfp143	-0.070300256	0.962648047
Zfp146	-0.005710414	0.99527876
Zfp148	-0.144793627	0.76998058
Zfp157	0.048132627	0.962648047

Zfp160	0.11993232	0.962648047
Zfp169	-0.109140348	0.945663104
Zfp174	0.102906806	0.930250446
Zfp180	0.695037357	0.87847909
Zfp182	-0.259652093	0.861366392
Aip	0.130685405	0.41351939
Zfp185	-0.043826388	0.987665265
Zfp189	0.033542706	0.975831674
Zfp2	0.083175505	0.911631946
Zfp202	-0.192479215	0.881026445
Zfp207	-0.004656485	0.99527876
Zfp212	0.196446868	0.571020232
Zfp213	-0.109886258	0.918235054
Zfp217	-0.160701111	0.909943341
Zfp219	0.020716306	0.987814724
Zfp229	-0.212663007	0.936031628
Zfp235	-0.0829201	0.948104382
Zfp236	-0.287321316	0.759486652
Zfp239	-0.195382241	0.962648047
Zfp24	0.091599333	0.78576919
Zfp248	0.042808498	0.976978183
Zfp251	0.076995527	0.911631946
Zfp26	0.067364517	0.956164302
Zfp260	-0.424762073	0.928957615
Zfp263	0.127834793	0.975831674
Zfp266	0.023849937	0.981392364
Zfp27	-0.109198186	0.965520236
Zfp273	-0.158774237	0.935897306
Zfp275	0.121443595	0.872352604
Zfp276	-0.025508464	0.988034453
Zfp277	-0.07169627	0.902496496
Zfp28	-0.014009551	0.99765039
Zfp280b	0.056235587	0.985673938
Zfp280c	-0.036424562	0.973602213
Zfp280d	-0.140818088	0.926051604
Zfp281	-0.049877218	0.958389182
Zfp282	-0.117117452	0.855904169
Zfp286	0.002077945	0.998010523
Zfp287	-0.153264659	0.927415966
Zfp292	0.468666844	0.971661054
Zfp296	0.120782222	0.98526072
Zfp3	0.009208429	0.99527876
Zfp30	-0.042843828	0.975900915
Zfp300	0.249397589	0.911631946

Zfp316	0.026072899	0.975800973
Zfp317	-0.080725032	0.950252558
Zfp318	-0.03265422	0.968005385
Zfp319	-0.096567152	0.890704638
Zfp322a	-0.036868228	0.968005385
Zfp324	-0.442106636	0.56651873
Zfp326	-0.127653455	0.837555331
Wdr7	-0.130651882	0.238827832
Zfp330	0.154601793	0.736130784
Zfp334	0.005537615	0.99527876
Zfp335	-0.065170616	0.923340746
Zfp335os	0.335346538	0.84870301
Zfp341	0.106113966	0.912397394
Zfp346	0.077771422	0.868415875
Zfp35	-0.010209259	0.994960308
Zfp354a	0.167727902	0.889972515
Zfp354b	-0.25553818	0.862285215
Zfp354c	0.002426802	0.997115148
Zfp358	-0.114326923	0.929095022
Zfp36	0.118754295	0.959276014
Zfp362	0.101934728	0.873797321
Zfp365	-0.010796893	0.985293518
Atrn	-0.129630555	0.374915032
Zfp367	0.037675121	0.968379096
Zfp369	-0.069414556	0.912397394
Zfp36l1	0.382585905	0.786062749
Zfp36l1-ps	-0.657195936	0.964941885
Zfp36l2	-0.090590696	0.928957615
Zfp37	-0.14185716	0.871869598
Zfp382	-0.29648272	0.723108545
Zfp383	0.087618698	0.925678705
Zfp384	-0.071561671	0.920642026
Zfp385a	-0.20453016	0.594430425
Zfp385b	-0.196679674	0.789546531
Zfp385c	-0.854833314	0.940963923
Zfp386	0.141528947	0.873797321
Zfp389	0.29718292	0.957849305
Zfp39	0.033561332	0.980419928
Zfp395	0.104071359	0.948104382
Mtpn	-0.129089528	0.251012111
Zfp398	-0.068584969	0.915417975
Zfp40	-0.065212124	0.973100313
Zfp407	-0.099881528	0.882096017
Zfp408	-0.053011415	0.968005385

Zfp41	-0.097320162	0.951390592
Zfp410	0.049144729	0.962648047
Zfp414	0.213145795	0.70114989
Zfp418	0.071827036	0.975831674
Zfp420	-0.081953663	0.962648047
Zfp422	0.046376713	0.962648047
Zfp423	0.186357721	0.89703631
Zfp426	-0.025425246	0.992152669
Cltc	-0.129070918	0.491912953
Zfp429	-0.497430012	0.938414123
Zfp433	0.232820351	0.91462326
Zfp438	-0.242266372	0.6883862
Zfp442	0.018574249	0.99527876
Zfp444	-0.068528078	0.962648047
Zfp445	-0.023793114	0.97487264
Zfp446	-0.185291574	0.898478018
Zfp449	-0.022401703	0.989840188
Zfp451	0.225630166	0.962648047
Zfp454	0.048891377	0.98526072
Zfp455	-0.116866791	0.966917555
Atp5c1	0.127864193	0.349775121
Zfp457	-0.517145483	0.904346827
Zfp458	-0.117169353	0.974823941
Zfp459	0.418534433	0.873797321
Zfp46	0.043276388	0.965520236
Rps6-ps4	0.127461005	0.452669584
Zfp467	0.144873307	0.956922504
Zfp469	0.349039674	0.911631946
Zfp472	0.214998647	0.87208087
Zfp473	0.485715406	0.915417975
Zfp474	-0.509992116	0.89703631
Zfp488	-0.302952807	0.694292239
Zfp493	-0.208865991	0.894148925
Zfp503	0.017441454	0.99527876
Zfp507	0.051844063	0.965320168
Zfp51	0.382850513	0.774896252
Zfp511	-0.03600967	0.980226283
Zfp512	0.063002738	0.946239011
Zfp512b	0.021457012	0.980419928
Zfp513	0.059290922	0.968005385
Prrc2a	-0.126661095	0.493073764
Zfp518a	0.078001537	0.962812066
Zfp518b	-0.153867979	0.910328122
Zfp52	-0.063801851	0.981045362

Zfp521	0.20694985	0.935897306
Zfp523	0.003764061	0.995411429
Zfp524	-0.611488134	0.705466472
Zfp526	-0.057128348	0.971661054
Zfp53	0.100507367	0.962648047
Zfp532	0.097485258	0.861909929
Zfp536	0.080756146	0.950460219
Zfp54	0.095346669	0.990337756
Zfp541	-0.065346792	0.989840188
Zfp551	-0.205453214	0.871869598
Zfp553	-0.044601894	0.965520236
Zfp558	-0.349034488	0.86849507
Zfp560	0.097053641	0.937924083
Zfp563	-0.020024345	0.985742667
Zfp566	0.25532322	0.756001016
Zfp568	-0.438709464	0.89703631
Arhgap21	-0.126475577	0.421691269
Zfp574	-0.041100051	0.965520236
Zfp575	0.104509991	0.962648047
Zfp579	0.391395648	0.852010446
Zfp58	-0.050014531	0.987814724
Rps6	0.126191284	0.499392203
Zfp583	-0.094608349	0.98340069
Zfp59	-0.163785683	0.861366392
Zfp592	-0.075314716	0.916922462
Zfp593	-0.172582232	0.84870301
Zfp595	-0.109261766	0.975831674
Zfp597	-0.05491701	0.949111839
Zfp598	-0.093424215	0.852010446
Zfp599	0.184054402	0.911631946
Zfp60	0.205838832	0.961373288
Mxi1	0.125799641	0.493073764
Zfp606	-0.021540099	0.991438757
Zfp607a	-0.546470086	0.724910334
Zfp607b	0.026490692	0.985742667
Zfp608	-0.196538939	0.529257466
Zfp609	-0.128384694	0.621784586
Zfp61	0.128049999	0.832980941
Zfp612	-0.033793074	0.978384252
Zfp617	0.048653648	0.962648047
Zfp618	-0.133262227	0.948345831
Zfp619	-0.342778135	0.760547704
Zfp62	0.684754767	0.894953269
Zfp622	-0.257321723	0.736367995

Zfp623	0.026710297	0.98187819
Zfp626	0.235519002	0.621993647
Zfp628	0.096726427	0.944494911
Zfp629	0.224768717	0.86783605
Zfp637	0.055870573	0.97487264
Zfp638	-0.157888177	0.744309911
Zfp639	-0.004918934	0.99527876
Zfp64	0.206955467	0.642397758
Zfp641	-0.02527261	0.984748462
Zfp644	0.41716233	0.871869598
Zfp646	-0.097626508	0.958725703
Zfp647	0.061370512	0.981045362
Zfp648	-0.563230737	0.901129754
Zfp65	-0.006648542	0.99527876
Zfp651	0.054229128	0.938394547
Zfp652	-0.022039573	0.98501783
Zfp652os	-0.057392554	0.985673938
Zfp653	0.038331897	0.974823941
Zfp654	-0.198084564	0.689605643
Zfp655	-0.161775015	0.647397128
Fh1	0.125626002	0.456330081
Zfp661	0.257007871	0.823072541
Fads1	0.125564696	0.485432358
Ncstn	0.125432015	0.489134742
Zfp667	-0.167041581	0.648436128
Zfp668	-0.113610573	0.939904081
Zfp672	0.065204154	0.911631946
Zfp677	0.665314121	0.757721895
Sort1	-0.125425022	0.355274289
Zfp687	0.034385104	0.981045362
Camk1d	-0.125236607	0.243596082
Zfp689	-0.06883509	0.969755848
Zfp69	0.020493068	0.997115148
Zfp691	0.307248743	0.573810203
Zfp692	-0.072029997	0.911631946
Zfp697	-0.028717523	0.975831674
Zfp7	0.189125026	0.911631946
Zfp703	-0.243923432	0.825148733
Zfp704	-0.117625638	0.585734325
Zfp706	-0.020219834	0.975831674
Zfp707	0.024498285	0.989840188
Zfp708	0.38771779	0.984837276
Zfp709	0.172380197	0.852010446
Zfp710	-0.219843178	0.586145911

Zfp711	0.003686022	0.99765039
Zfp712	0.086212858	0.965520236
Zfp715	0.114852723	0.79212401
Zfp719	-0.155372677	0.745988863
Zfp72	0.157051957	0.911631946
Zfp729a	-0.048531241	0.968630572
Zfp729b	-0.266230503	0.809980757
Zfp738	-0.064143077	0.953505913
Zfp74	-0.112174008	0.876612181
Etnk1	-0.124933183	0.442132509
Zfp746	0.023196804	0.98340069
Zfp747	0.083144032	0.968005385
Zfp748	-0.089246678	0.948104382
Gabarapl1	0.124728188	0.323832374
Zfp758	-0.004846789	0.99765039
Zfp759	0.077891842	0.971661054
Zfp760	0.006595218	0.99527876
Zfp763	0.128026141	0.874252042
Zfp764	0.022369886	0.991996237
Zfp768	-0.14712552	0.911631946
Zfp770	-0.042410721	0.965290366
Zfp771	0.053015146	0.964011524
Zfp772	-0.017608381	0.985742667
Zfp773	0.186916064	0.965520236
Zfp775	0.321901784	0.736771155
Zfp777	0.067789399	0.91462326
Zfp78	0.098757736	0.97487264
Zfp780b	-0.069272484	0.957499962
Zfp781	0.112026824	0.963280717
Zfp783	-0.025851267	0.991438757
Zfp784	0.43900278	0.955993235
Zfp786	0.354391983	0.571020232
Zfp787	0.118954523	0.874252042
Zfp788	0.378333687	0.779231312
Zfp790	-0.107102912	0.913701113
Zfp791	0.038389622	0.982934928
Zfp799	0.062814132	0.962648047
Zfp800	-0.064267638	0.971661054
Sdhb	0.124264681	0.442302609
Pgm1	0.123461694	0.486814463
Zfp808	-0.759630409	0.744046401
Zfp809	0.058033991	0.969755848
Ranbp3	0.122819437	0.476512188
Zfp810	0.002561627	0.99765039

Zfp811	0.246134638	0.544661714
Zfp819	0.136627687	0.99527876
Zfp82	-0.146741408	0.945663104
Zfp820	0.758111598	0.94417444
Zfp821	0.02006046	0.982309415
Zfp825	0.126300464	0.968005385
Zfp827	-0.475483859	0.84277074
Zfp830	0.082863884	0.959276014
Prickle1	-0.121999773	0.424763021
Zfp839	0.045160536	0.978652522
Zfp84	-0.075512209	0.961887657
Zfp846	0.487555118	0.932673884
Zfp85	0.143208912	0.917685683
Zfp850	-0.050840212	0.980419928
Zfp853	0.053417786	0.985653607
Sparcl1	0.121799949	0.497690258
Zfp862-ps	0.051306656	0.949712083
Zfp865	-0.00815747	0.995411429
Zfp866	0.078470909	0.89703631
Zfp867	0.188921618	0.852010446
Zfp868	0.078170574	0.948104382
Zfp869	0.149065844	0.695239733
Zfp87	0.041299573	0.970461999
Zfp870	-0.099743228	0.936031628
Zfp871	-0.113362889	0.511540781
Zfp873	0.431702686	0.674083347
Zfp874a	-0.04315766	0.984748462
Zfp874b	0.152714129	0.710375942
Zfp879	0.324335222	0.829694131
Zfp882	-0.06228615	0.97487264
Zfp9	-0.020054924	0.985446046
B3gat1	-0.120948714	0.459730077
Zfp91	-0.048092589	0.899097089
Zfp92	-0.316147891	0.841017002
Zfp93	0.117672546	0.90358675
Zfp930	0.406870082	0.680282967
Zfp931	0.049468702	0.97487264
Zfp932	-0.058059814	0.947033215
Psap	0.120634077	0.452669584
Zfp934	0.204259426	0.898478018
Zfp937	0.101775868	0.975831674
Zfp938	0.072524223	0.928957615
Zfp939	0.219463182	0.814301191
Zfp94	0.006092786	0.99527876

Zfp940	-0.055125722	0.962648047
Zfp941	0.128804628	0.826209871
Zfp942	-0.303427068	0.520182905
Zfp943	-0.059261218	0.971661054
Zfp944	-0.202138722	0.822693227
Atp2a2	-0.120531205	0.484146817
Zfp946	-0.152527672	0.907572296
Zfp947	0.279698716	0.974962198
Zfp948	0.01811982	0.993103295
Zfp949	0.086192358	0.911631946
Zfp950	-0.032978968	0.975800973
Zfp951	0.207823605	0.958389182
Zfp952	-0.864996725	0.799736155
Zfp953	0.029256989	0.99527876
Zfp954	0.115726684	0.90358675
Zfp955a	0.122824019	0.914675447
Zfp955b	-0.135680742	0.889908105
Zfp956	0.160284924	0.582268679
Zfp958	0.023940636	0.991996237
Zfp959	0.763720791	0.664907827
Zfp960	-0.244448813	0.948104382
Eif3h	0.120293231	0.481164867
Zfp963	-0.068859942	0.965752549
Zfp964	-0.071866657	0.97487264
Zfp965	0.017132711	0.99527876
Zfp966	-0.281883901	0.905559486
Zfp968	0.246841273	0.882096017
Zfp969	0.238573947	0.908413609
Zfp97	-0.416842282	0.873797321
Zfp970	0.082002735	0.959659722
Zfp971	0.240316211	0.736317959
Zfp972	0.227367092	0.971876411
Zfp973	-0.011719013	0.99527876
Zfp974	-0.092099867	0.942812446
Zfp975	0.050949226	0.985742667
Zfp976	-0.561662494	0.824306801
Zfp977	-0.415356393	0.934635614
Zfp979	0.649163202	0.973100313
Zfp981	-0.849373153	0.97487264
Zfp982	-0.07111465	0.993856593
Zfp983	0.131130076	0.925924974
Zfp984	0.110711082	0.970348278
Arpc3	0.118620522	0.41351939
Zfp988	-0.663210746	0.9637275

Zfp991	-0.264761501	0.911631946
Zfp992	0.154168939	0.980144634
Zfp993	0.056960778	0.99527876
Zfp994	-0.03200225	0.993103295
Zfp995	-0.048694127	0.985673938
Zfpl1	-0.043965478	0.975831674
Zfpm1	-0.373584489	0.58075932
Zfpm2	0.17613569	0.721943953
Zfr	-0.166836886	0.56399886
Zfr2	-0.059442861	0.966213117
Zfx	-0.304021481	0.968005385
Zfyve1	-0.067433761	0.897878088
Zfyve16	-0.121738861	0.90358675
Zfyve19	-0.0894469	0.91462326
Zfyve21	0.206621264	0.814814675
Zfyve26	-0.091340369	0.951901417
Zfyve27	0.001869649	0.997222376
Zfyve28	-0.037657982	0.971661054
Zfyve9	-0.051745353	0.944074069
Rgs8	-0.117649148	0.410106728
Zgpat	0.014908371	0.993380259
Zgrf1	-0.096705803	0.971934842
Zhx1	0.040143738	0.981045362
Zhx2	0.030022129	0.974823941
Zhx3	0.096183174	0.756001016
Zic1	-0.085385207	0.97487264
Zic2	-0.088120131	0.974197921
Zic3	-0.100727846	0.97487264
Zic4	-0.173059429	0.970461999
Rps2	0.117138181	0.504583609
Zik1	0.116402111	0.822849224
Zim1	-0.008038569	0.99527876
Zkscan1	0.043420324	0.942511266
Zkscan14	0.220844592	0.70724751
Zkscan16	-0.066641749	0.968005385
Zkscan17	0.091966204	0.936031628
Zkscan2	-0.030479473	0.981045362
Zkscan3	-0.069024021	0.91462326
Zkscan4	0.345394146	0.642771234
Zkscan5	0.004914398	0.997115148
Zkscan6	-0.090886119	0.918407269
Zkscan7	0.310128608	0.814191877
Zkscan8	-0.07801119	0.939393695
Zmat1	-0.041639953	0.984328758

Zmat2	0.120941895	0.621784586
Zmat3	-0.15631719	0.70036487
Zmat4	0.004124063	0.99527876
Zmat5	0.122802996	0.858549868
Zmiz1	-0.481603115	0.660403254
Zmiz1os1	0.443428172	0.814276256
Zmiz2	-0.000407171	0.99900438
Zmpste24	-0.063289169	0.909547396
Sbf1	-0.115829388	0.442068044
Srpk2	-0.113870071	0.465918106
Zmym3	-0.088453584	0.680836096
Psma1	0.112284873	0.481098488
Zmym5	-0.013555086	0.99527876
Zmym6	0.007715043	0.99527876
Zmynd10	-0.535783833	0.550301004
Zmynd11	-0.021814497	0.974962198
Zmynd12	-0.432667521	0.928957615
Zmynd15	0.141979523	0.985742667
Zmynd19	-0.046472897	0.962648047
Zmynd8	-0.188037661	0.758595939
Znf41-ps	0.225051569	0.962648047
Znfx1	-0.033267495	0.965290366
Znhit1	0.037149863	0.975350978
Znhit2	-0.031649476	0.980109544
Znhit3	0.22225654	0.631708623
Znhit6	-0.09988112	0.939393695
Gls	-0.10879032	0.399438388
Znrd1as	0.215315343	0.948520404
Znrf1	-0.114054289	0.956164302
Znrf2	-0.095309509	0.821998633
Znrf3	-0.03794522	0.985673938
Zp2	0.805226402	0.962648047
Ik	0.108505843	0.507235129
Sptbn1	-0.108260212	0.442302609
Zpbp	0.000761861	0.999486844
Zpld1	-0.021816172	0.995852544
Zpr1	0.024363405	0.976978183
Zranb1	0.132565813	0.736130784
Zranb2	-0.041324548	0.961876667
Zranb3	0.052589476	0.97487264
Zrsr1	0.043362105	0.938394547
Zrsr2	0.003549024	0.99527876
Zscan10	0.677960164	0.923011738
Zscan12	0.046346531	0.981502501

Sqstm1	0.105217441	0.442302609
Zscan2	0.436988557	0.65012378
Zscan20	-0.092613639	0.965520236
Zscan21	-0.182376342	0.540394513
Zscan22	-0.130464785	0.8549794
Zscan25	0.103040401	0.911631946
Zscan26	-0.445968393	0.911631946
Zscan29	-0.052447708	0.97469529
Zscan30	-0.77459325	0.787744512
Zswim1	0.070953143	0.904346827
Zswim2	-0.699303399	0.976978183
Zswim3	-0.014117517	0.99527876
Zswim4	0.156534181	0.644169569
Zswim5	-0.129682926	0.803910915
Ttyh1	0.104921618	0.445106721
Zswim7	0.213693052	0.873797321
Zswim8	-0.10254315	0.67239949
Zswim9	-0.006891735	0.99527876
Zup1	-0.060096345	0.974809035
Zw10	-0.046836913	0.962648047
Zwilch	0.073455344	0.97469529
Zwint	0.015997234	0.98526072
Zxda	-0.323800241	0.974823941
Zxdb	-0.188552576	0.529257466
Zxdc	0.099796705	0.895870154
Clu	0.098399183	0.421691269
Zyx	-0.006517763	0.995717436
Zzef1	-0.086400017	0.831186237
Zzz3	-0.359793224	0.8549794
